# Ultrastructural and proteomic profiling of mitochondria-associated endoplasmic reticulum membranes reveal aging signatures in striated muscle

**DOI:** 10.1038/s41419-022-04746-4

**Published:** 2022-04-02

**Authors:** Xue Lu, Yingchao Gong, Wanyu Hu, Yankai Mao, Ting Wang, Zeyu Sun, Xiaoling Su, Guosheng Fu, Yanpeng Wang, Dongwu Lai

**Affiliations:** 1 Key Laboratory of Cardiovascular Intervention and Regenerative Medicine of Zhejiang Province, Department of Cardiology, Sir Run Run Shaw Hospital, Zhejiang University School of Medicine, Hangzhou, Zhejiang Province China; 2 Department of Diagnostic Ultrasound and Echocardiography, Sir Run Run Shaw Hospital, Zhejiang University School of Medicine, Hangzhou, Zhejiang Province China; 3 Hangzhou Medical College, Hangzhou, Zhejiang Province China; 4 State Key Laboratory for Diagnosis and Treatment of Infectious Diseases, National Clinical Research Center for Infectious Diseases, Collaborative Innovation Center for Diagnosis and Treatment of Infectious Disease, The First Affiliated Hospital, School of Medicine, Zhejiang University, Hangzhou, Zhejiang Province China; 5 Department of Gynecology, Zhejiang Provincial People’s Hospital (People’s Hospital of Hangzhou Medical College), Hangzhou, Zhejiang Province China

**Keywords:** Organelles, Cardiovascular diseases, Cell signalling

## Abstract

Aging is a major risk for developing cardiac and skeletal muscle dysfunction, yet the underlying mechanism remains elusive. Here we demonstrated that the mitochondria-associated endoplasmic reticulum membranes (MAMs) in the rat heart and skeletal muscle were disrupted during aging. Using quantitative morphological analysis, we showed that the mitochondria-endoplasmic reticulum contacts (MERCs) were reduced by half over the lifespan with an early onset of accelerated thickening in the clefts. The ultrastructural changes were further validated by proteomic profiling of the MAM fractions. A combination of subcellular fractionation and quantitative mass spectrometry identified 1306 MAM-enriched proteins in both heart and skeletal muscle, with a catalog of proteins dysregulated with aging. Functional mapping of the MAM proteome suggested several aging signatures to be closely associated with the ER-mitochondria crosstalk, including local metabolic rewiring, calcium homeostasis imbalance, and impaired organelle dynamics and autophagy. Moreover, we identified a subset of highly interconnected proteins in an ER-mitochondria organization network, which were consistently down-regulated with aging. These decreased proteins, including VDAC1, SAMM50, MTX1 and MIC60, were considered as potential contributors to the age-related MAM dysfunction. This study highlights the perturbation in MAM integrity during the striated muscle aging process, and provides a framework for understanding aging biology from the perspective of organelle interactions.

## Introduction

Aging is an evolutionarily conserved yet poorly understood process that leads to loss of many physiological functions over the lifespan. Studies have suggested that aging may independently contribute to the development of heart failure and sarcopenia, two highly prevalent geriatric syndromes with shared pathophysiological and clinical characteristics [1–3]. The aged striated muscles rewire their metabolism and substrate selection toward redox-related pathways, thus lead to a dramatic increase in the energetic cost of physical activity and decrease in working capacity [4, 5]. Given that aging has a complex and widespread implication on striated muscle, from physical performance to biological mechanisms deeply rooted in cellular energetics and metabolism, it is not surprising that the coexisting cardiac and skeletal muscle dysfunction would inevitably result in adverse health outcomes and death among older adults [3, 6].

Currently, no treatment is available to prevent or delay age-associated muscular deterioration [3]. A detailed understanding of the underlying mechanisms is urgently needed for identifying therapeutic targets. Compelling evidence demonstrated that mitochondrial dysfunction is a major determinant of aging [5]. Far from being standalone organelles, mitochondria are physically interacting with other subcellular compartments [7]. Of interest in this regard are the mitochondria-endoplasmic reticulum (ER) contacts (MERCs), which appear as sites of parallel juxtaposition between the ER and outer mitochondrial membrane (OMM) [8]. MERCs, also known as mitochondria-associated endoplasmic reticulum membranes (MAMs) when biochemically isolated or purified, control a diverse array of cellular processes including lipid and calcium (Ca^2+^) exchanges, autophagy initiation, apoptosis induction, inflammasome formation and metabolic modulation [8]. To achieve this functional diversity, the ultrastructural signatures (i.e., length and thickness) and protein composition of MAMs (or MERCs) are highly dynamic according to the changing intracellular environment [8, 9].

To date, a direct link between the spatial organization of MAMs and aging remains highly underappreciated. There have been apparently contradictory observations regarding the role of MAMs in replicative senescence [9–11]. Furthermore, it raises the question regarding the characteristics and effects of MAMs in post-mitotic striated myocytes, during the so-called “premature aging”. Defective sarcoplasmic reticulum (SR)/ER–mitochondria Ca^2+^ communication accompanied by cristae disassembly have been reported in the aged mouse myocardium [12, 13]. This may suggest an altered structure or (and) composition of MAMs underlying these functional defects. To shed light on this issue, in this study we used a rat model of normal aging to describe the ultrastructural and proteomic signatures in MAMs that derived from cardiac and skeletal muscle. Our findings support a model where intermyofibrillar mitochondria respond to aging-evoked stress by loosening their contacts with the ER. Thus, local metabolic remodeling is coupled with the machineries that organize MERC assembly, which ultimately lead to the age-related striated muscle dysfunction.

## Results

### Development of age-related cardiac and skeletal muscle dysfunction in older rats

**Fig. 1:**
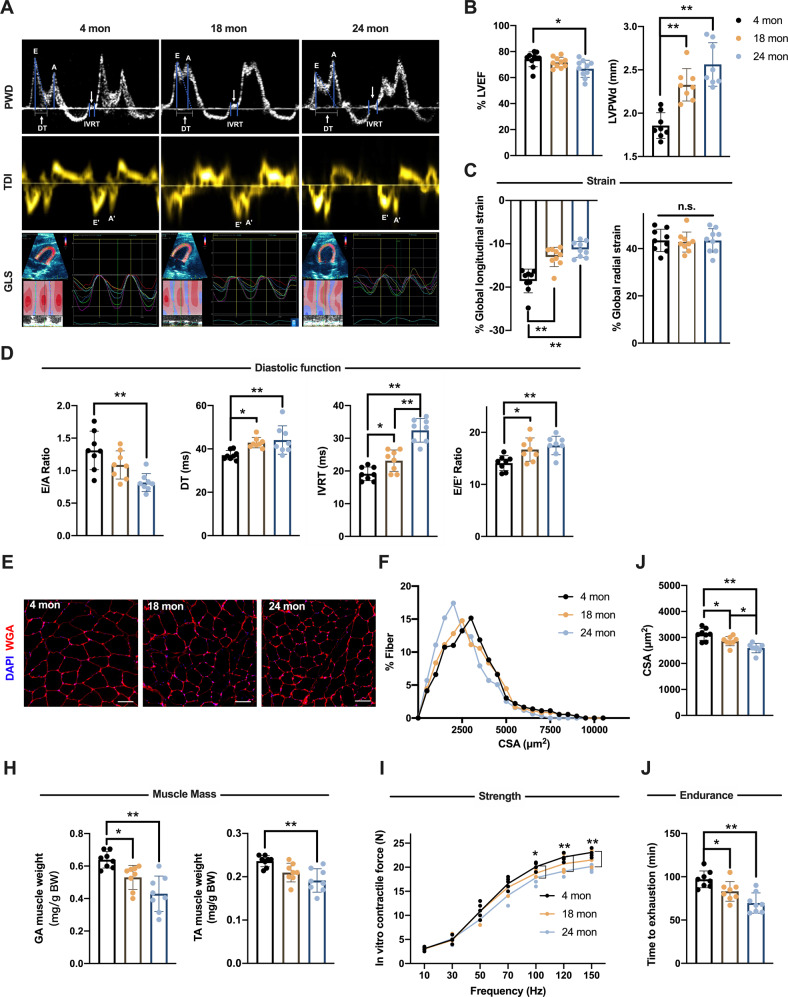
Development of age-related cardiac dysfunction and sarcopenia in older rats. **A** to **D** Echocardiographic assessment was performed in 4-, 18- and 24-month-old male F344 rats. **A** Representative pulse wave Doppler (PWD) measurements of mitral inflow (upper), tissue Doppler Imaging (TDI) of lateral wall motion at the septal annulus (middle), and left ventricular global longitudinal strain (GLS) curves (lower) were shown for the indicated age groups. **B** Left ventricular ejection fraction (LVEF) and left ventricular posterior wall at end-diastole (LVPWd) measured by M-mode echocardiography. **C** Global longitudinal and radial strain measured by strain echocardiography. **D** Ratios of the early (**E**) to late (**A**) diastolic mitral flow velocity, deceleration times (DT), isovolumic relaxation times (IVRT), and ratio of the early-diastolic mitral flow velocity (E) to early-diastolic mitral annular velocity (E’) evaluated for LV diastolic function. **E** Representative gastrocnemius (GA) muscles cross-section of the indicated age groups. DAPI, blue; WGA, red. Bar = 50 μm. **F** Frequency distributions of GA myofiber cross-sectional areas (CSA). **J** Mean CSA. **H** GA and tibialis anterior (TA) muscle weight relative to body weight (BW). **I** Quantification of the strength/stimulation frequency relationship in GA muscles. **J** Treadmill running time to exhaustion in rats of the indicated age groups. Data represent mean ± SD, *n* = 8 rats per group; The variance was similar among the groups. ^*^P < 0.05, ^**^P < 0.01.

To tackle the age-related functional alterations of the heart, we first performed conventional echocardiographic evaluation of the left ventricle (LV). Global LV systolic function, reflected by the ejection fraction (EF), fractional shortening (FS) and cardiac output (CO), was mildly declined only in the 24-month-old rat group when compared with that of the four-month-old rat group. Nevertheless, the EF was not below 50% in the aged hearts (Fig. 1B and Supplementary Table 1). The left ventricular posterior wall at end-diastole (LVPWd) significantly increased during aging, indicating LV remodeling and hypertrophy (Fig. 1B and Supplementary Table 1). In the speckle-tracking-based echocardiographic strain analysis, a significant impairment of global longitudinal strain (GLS), an earlier marker of age-related LV dysfunction in comparison to EF and FS, was already observed in the 18-month-old rat group (Fig. 1A, C and Supplementary Table 1). In contrast to systolic function, LV diastolic function was remarkably compromised during aging, especially in the early active relaxation phase. The 18- and 24-month-old rats both had prolonged deceleration times (DT) and isovolumic relaxation times (IVRT), reduced ratios of the early- to late-diastolic mitral flow velocity (E/A), as well as the increased ratios of the early-diastolic mitral flow velocity to early-diastolic mitral annular velocity (E/E’) (Fig. 1A, D and Supplementary Table 1).

We then accessed the age-related alterations of skeletal muscle. Like humans, aged rats exhibit sarcopenia, a general loss of muscle mass and strength [14]. The cross-sectional area (CSA) of the gastrocnemius (GA) muscle was significantly reduced during aging (Fig. 1E–J). Moreover, 18- and 24-month-old rats showed reduction of the GA and tibialis anterior (TA) muscle weight (Fig. 1H). To define whether the declined muscle fiber size and muscle mass would lead to functional loss, we next assessed the GA strength in vitro and exercise capacity in vivo. There was a significant decrease in force generation at high-frequency stimulation in aged muscle (24-month-old) compared with that of young muscle (Fig. 1I). Similar results were found in the running activity using a treadmill exhaustion test, which showed a significant reduction in running time during aging (Fig. 1J).

All the above findings suggested an impairment of functional reserve in the aged cardiac and skeletal muscle, especially when coping with high-energy demand such as exercise. We thus hypothesized that the cardiac and skeletal muscle abnormalities during aging may be associated with defective mitochondrial energy metabolism, an established characteristic of the aging etiology [5, 7].

### Cardiac and skeletal muscle aging was accompanied by defective mitochondrial ultrastructure and the loss of MERCs

**Fig. 2:**
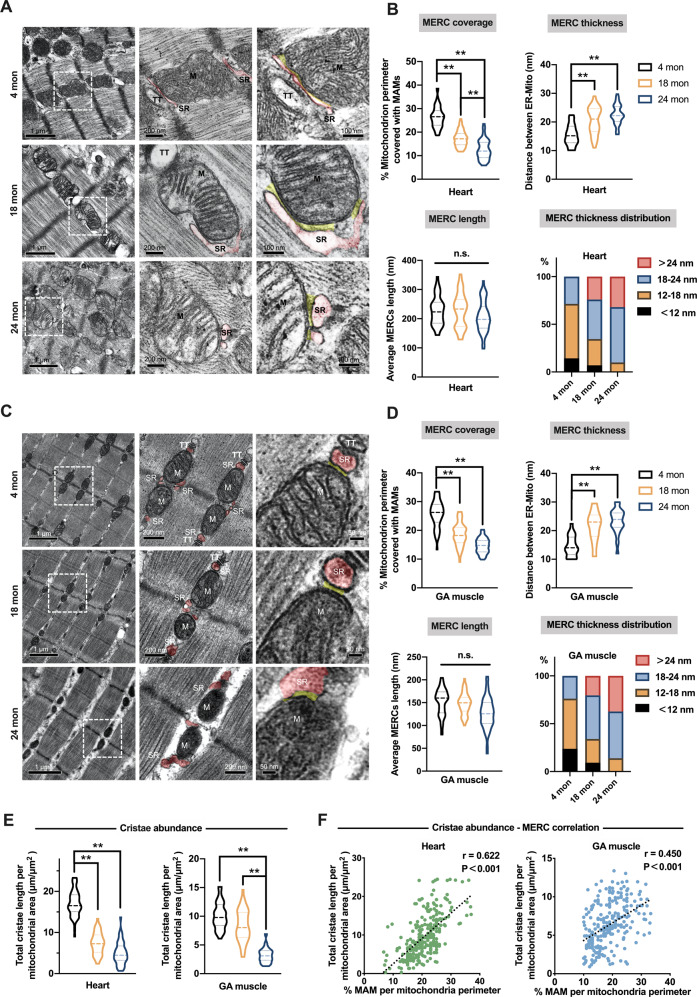
Structural signatures of MERCs in cardiac and skeletal muscle during aging. **A** and **C** Representative TEM images from heart (**A**) and GA muscle (**C**) used to analyze MERCs. The MERCs were shown in yellow, and the junctional sarco-endoplasmic reticulum (jSR/ER) apposition to mitochondria were shown in red. **B** and **D** Effect of aging on MERC coverage, thickness and length in heart (**B**) and GA muscle (**D**). (*n* = 4 rats per group; total MERCs quantified in hearts/GA muscles: *n* = 85/70 for 4-month-old group, *n* = 77/72 for 18-month-old group, *n* = 81/85 for 24-month-old group). **E** Effect of aging on mitochondria cristae abundance in heart (left) and GA muscle (right). (*n* = 4 rats per group; total mitochondria quantified in hearts/GA muscles: *n* = 122/80 for 4-month-old group, *n* = 126/79 for 18-month-old group, *n* = 110/80 for 2-month-old group). **F** The correlation between cristae abundance and MERC coverage for individual mitochondrion from all age groups were calculated, using simple linear regression analyses to determine the correlation constant (*r*) and *P*-value. Total mitochondria quantified in hearts/GA muscles: *n* = 235/210. Data represent as the violin plots with mean ± SD. The variance was similar between the groups being compared. *P < 0.05; **P < 0.01.

To assess the ultrastructure of aged mitochondria and the surrounding MERCs in vivo, we performed transmission electron microscopy (TEM) analysis of heart and GA muscle samples from 4-, 18- and 24-month-old rats (Fig. 2A, C). There was an increase in the area of individual mitochondrion from the hearts and GA muscles in 24-month-old rats compared with that of 4-month-old rats (Supplementary Fig. 1A and Supplementary Table 2). However, the enlarged size was attributed to distinct mitochondrial morphological adaptions between the heart and GA muscle. In the heart, the aged mitochondria became more circular and swollen, while in the GA muscle they tended to be longer and less circular (Supplementary Fig. 1C, E and Supplementary Table 2). Moreover, strong reductions in the mitochondrial cristae abundance were both observed in the aged hearts and GA muscles (starting from 18 months and exacerbated at 24-months of age), indicating a compromised efficiency of mitochondrial bioenergetics during aging (Fig. 2E and Supplementary Table 2).

The age-related structural alterations in MERCs were characterized by several parameters, including the thickness, coverage and length (Fig. 2A, C). These parameters are recognized as key structural elements that define the biological activity of MERCs. Of which, the thickness of a MERC determines the mitochondrial Ca^2+^ response and is central to the metabolic state of striated myocytes [8]. Data showed that the MERCs thickness has been fast increased by 40% by middle-age, from ≈15 mm to ≈21 mm (at 4 and 18 months, respectively), and slowly peaked at 24 months (to a maximum of ≈23 mm). Following this, the MERCs coverage, as a parameter reflecting the degree of organelle interconnections, was reduced by nearly one half throughout the aging process (from ≈26% at 4 months to 13% and 15% at 24 months, in the heart and GA muscle, respectively). Aging did not impact the MERCs length in our observations (Fig. 2B, D and Supplementary Table 2). These results suggested that the aged striated muscle tends to widen the cleft and loosen the ER-mitochondria communication prior to reshaping the mitochondrial morphology. Thus, the increased thickness of MERCs may be considered as an early hallmark of aging. We then proceeded to assess whether the mitochondrial organization and MERCs dynamics are interdependent across ages. Simple linear regression analyses showed that the MERCs coverage was correlated with the cristae abundance (Fig. 2F) and mitochondrial area (Supplementary Fig. 1B) in both tissues (All *P* < 0.05). Significant correlations between the MERCs coverage and the mitochondrial circularity were also observed in the heart and skeletal muscle aging processes (Supplementary Fig. 1D). All together, these findings suggested, from a morphological perspective, that mitochondria adapt to aging through an extensive and parallel remodeling of their shape, cristae and MERCs. The highly structural plasticity of MERCs over the lifespan shed lights on a critical role of the mitochondria-ER communication in orchestrating cellular metabolism and mitochondrial fitness [7].

### Subcellular fractions showed a reduced quantity of MAM proteins in aged striated muscles

**Fig. 3:**
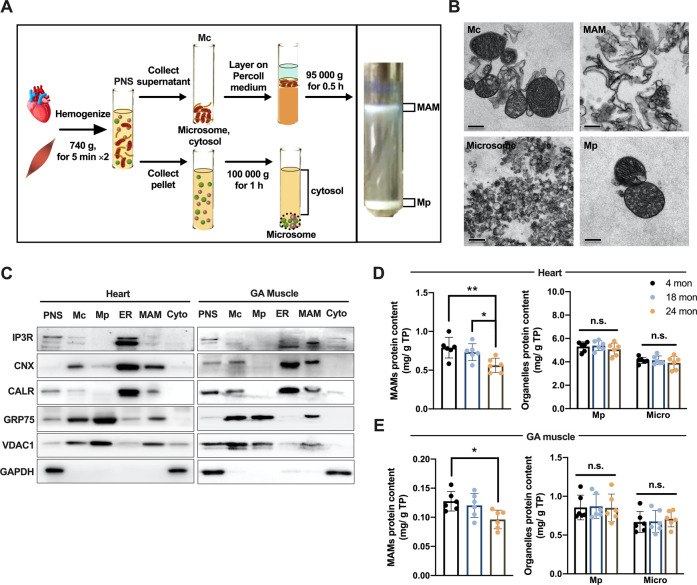
Mitochondria-associated membranes (MAMs) purified and identified from rat heart and GA muscle. **A** Scheme for mitochondria-associated membranes (MAMs) using Percoll gradient centrifugation. **B** Subcellular fractions prepared from rat heart were validated by transmission electronic microscopy (TEM). Bar = 0.5 μm. **C** Protein components of subcellular fractions prepared from rat heart and GA muscle were revealed by immunoblot analysis. CALR (calreticulin) and GAPDH were used as markers for ER and cytosolic fractions, respectively; IP3R and CANX (calnexin) was considered as markers of both ER and MAMs, whereas VDAC1 and GRP75 was adopted as markers of both mitochondria and MAMs. **D** and **E** The protein content (in mg) of MAMs, pure mitochondria and microsome was quantified and normalized by total tissue mass (in g) to obtain the relative protein abundance of subcellular fractions in heart (**D**) and GA muscle (**E**) (*n* = 6 rats per group, data represent mean ± SD. Variance was similar between groups being compared. *P < 0.05; **P < 0.01). PNS post-nuclear supernatant, Mc crude mitochondria, Mp pure mitochondria, Microsome microsome/ER fraction, MAM mitochondria-associated membrane, Cyto cytosol.

To elucidate the protein composition of MAMs in aged hearts and skeletal muscles, we first isolated subcellular fractions according to a well-established method [15] (Fig. 3A). The isolated subcellular fractions were fixed and identified using TEM. In the TEM images, MAMs appeared as ribosome-free membrane fragments, visually similar to the membranes surrounding mitochondria in the crude mitochondrial fractions (Mc, containing both MAMs and pure mitochondria) (Fig. 3B). Furthermore, we observed that the membrane structures in MAMs looked distinct from those of the microsome, which were described as small vesicles derived from ER pieces. This was consistent with recent biochemical observations suggesting that MAMs are special subcellular fractions with unique molecular composition apart from the ER membranes [16, 17]. Several organelle marker proteins were investigated using Western blot to further confirm the purity of MAMs (Fig. 3C). CANX (Calnexin), IP3R and CALR (Calreticulin), commonly acknowledged MAM and ER markers, were consistently presented in our isolated MAM and ER fractions. GRP75 was reported to be enriched in the MAM fractions and functioned as an ER-mitochondria tethering protein by bridging the gap between IP3R and VDAC1 [18]. VDAC1 was an OMM protein enriched in the Mc, pure mitochondrial fractions (Mp) and MAM fractions. GAPDH as a cytosolic marker was absent in Mc, Mp, MAM and ER.

We next assessed the overall protein abundance of MAMs by performing total protein quantification upon subcellular fractionation. After normalization to tissue mass, only MAM fractions from the 24-month-old hearts and GA muscles revealed a ≈ 30% reduction in protein abundance relative to those of the young tissues (4-month-old), whereas no changes were found for the Mp and microsomal fractions (Fig. 3D, E). Thus, the protein level analysis suggested that aged striated muscles have a reduced quantity of MAM proteins but not mitochondrial or ER proteins, probably due to the loss of the ER-mitochondria association.

### Proteomic profiling of MAMs identified deregulated proteins in aged striated muscle mainly from mitochondria

**Fig. 4:**
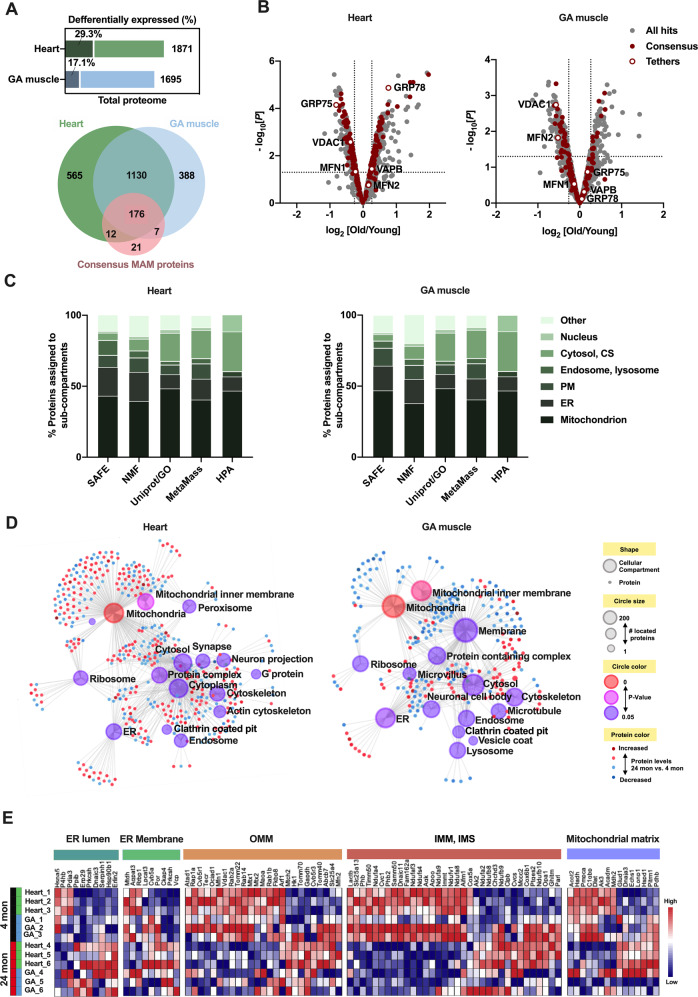
Subcellular mapping of the MAM proteome. **A** Bar plots (upper) represented the percentage of deregulated MAM proteins with aging in rat heart and GA muscle. Venn diagrams (lower) showed the intersections between the hits obtained in the MAMs proteome of this study, with a consensus set of 216 MAM proteins (related to Supplementary Tables 5). **B** The volcano plots showed all of the detected proteins (gray), consensus MAM proteins that are commonly recognized in previous literatures (red dots) and the known ER-mitochondria tethers (red circles). The dashed lines in the volcano plots indicated the cutoff values of >1.2 or <0.83 for fold-change and *P* < 0.05. **C** Subcellular mapping of the MAM proteome according to different subcellular location methods. HPA, the immunofluorescence-based location data from Human Protein Atlas; Uniprot/GO, proteins assigned with a single location in either Uniprot or Gene Ontology Consortium; SAFE, the BioID-based location predictions using spatial analysis of functional enrichment; NMF, the BioID-based location predictions using non-negative matrix factorization. **D** Subcellular localization network with age-associated MAM proteins in rat heart (left) and GA muscle (right). The circles represented the top 15 PANTHER: CC (Cellular Component) terms obtained from enrichment analysis, which were colored by term significance and sized by number of associated proteins. All proteins located in a cellular component were shown as small dots surrounding the CC term and colored by relative protein levels: red represented up-regulation in the 24-month-old group compared with that in the 4-month-old group, whereas blue represented down-regulation at 24 months. **E** Heatmap showing altered expression profile of age-associated MAM proteins with different subcellular origins. (*n* = 3 rats per group). CS cytoskeleton, PM plasma membrane, ER mem ER membrane, OMM outer mitochondrial membrane, IMM inner mitochondrial membrane, IMS intermembrane space.

To identify age-related MAM proteins in striated muscle, we carried out high-throughput, quantitative proteomics on MAM fractions from 4- and 24-month-old rats (*n* = 3 per group). Since the total protein quantification of MAMs showed no significant changes at 18 months compared with those at 4 months, the 18-month-old groups were not included in the proteomic analysis. Mass spectrometry identified 1871 and 1695 proteins with an FDR of <1%, in the heart and GA muscle respectively (Supplementary Table 3). The two protein sets displayed a great overlap with 1306 proteins in common (Fig. 4A). In addition, we analyzed the intersection between our datasets and a set of proteins ubiquitous to MAMs from diverse tissue types [19–21] (Supplementary Fig. 2A), which was previously identified as a consensus of MAM proteins. Of the 216 consensus MAM proteins, 176 co-existed in the heart and GA muscle MAM proteomes (Fig. 4A, B and Supplementary Table 5), highlighting that the core protein components of MAMs are relatively conserved across tissues. Notably, there was tissue-specific alterations in the up- and downregulated proteins derived from the heart and GA muscle (Supplementary Fig. 2C and Supplementary Tables 4). Principal component analysis (PCA) also showed a clear separation among the experimental groups, indicating both the age factor and tissue origin of MAMs are major determinants of their proteomic characteristics (Supplementary Fig. 2B). Moreover, we identified tissue-specific alterations in consensus MAM proteins, especially in those acting as ER-mitochondrial tethers (Fig. 4B). The chaperone proteins GRP75 and GRP78 selectively changed in the heart, and MFN2 was only altered in the GA muscle, in addition to a significant reduction of VDAC1 in both tissues (Fig. 4B and Supplementary Tables 4).

It is well-established that the MAMs could function as a spatially defined platform that dynamically recruits components required for numerous cellular physiological functions [22, 23]. Given this perspective, multiple proteome-wide subcellular localization methods were used to achieve a complete picture of the subcellular components assembled at MAMs (see “Methods”). All the localization results suggested the mitochondrion to be the greatest subcellular source for MAMs in heart and GA muscle (Fig. 4C and Supplementary Fig. 2D). However, when we performed localization analyses on the recently published MAM proteomes derived from the mouse liver [19], brain [24, 25] and testis [24], the ER, and not the mitochondria, was identified as the main MAM source (Supplementary Tables 6). Enrichment analysis also revealed the vast majority of age-related MAM proteins derived from mitochondria in the heart and GA muscle (Fig. 4D and Supplementary Tables 6). By further mapping the deregulated MAM proteins to sub-organelle compartments, it showed that the missing mitochondrial components in aged MAM fractions mainly derived from the OMM and inner mitochondrial membranes (IMM). Moreover, as a compensatory response, the ER-resident proteins tended to be increased during aging (Fig. 4E and Supplementary Fig. 2E). Collectively, these findings indicated a striated muscle-specific spatial organization of MAMs, with the mitochondrial proteins highly enriched and aging-sensitive.

### Proteomic changes revealed the metabolic implications of the ER–mitochondria association in the aged striated muscle

**Fig. 5:**
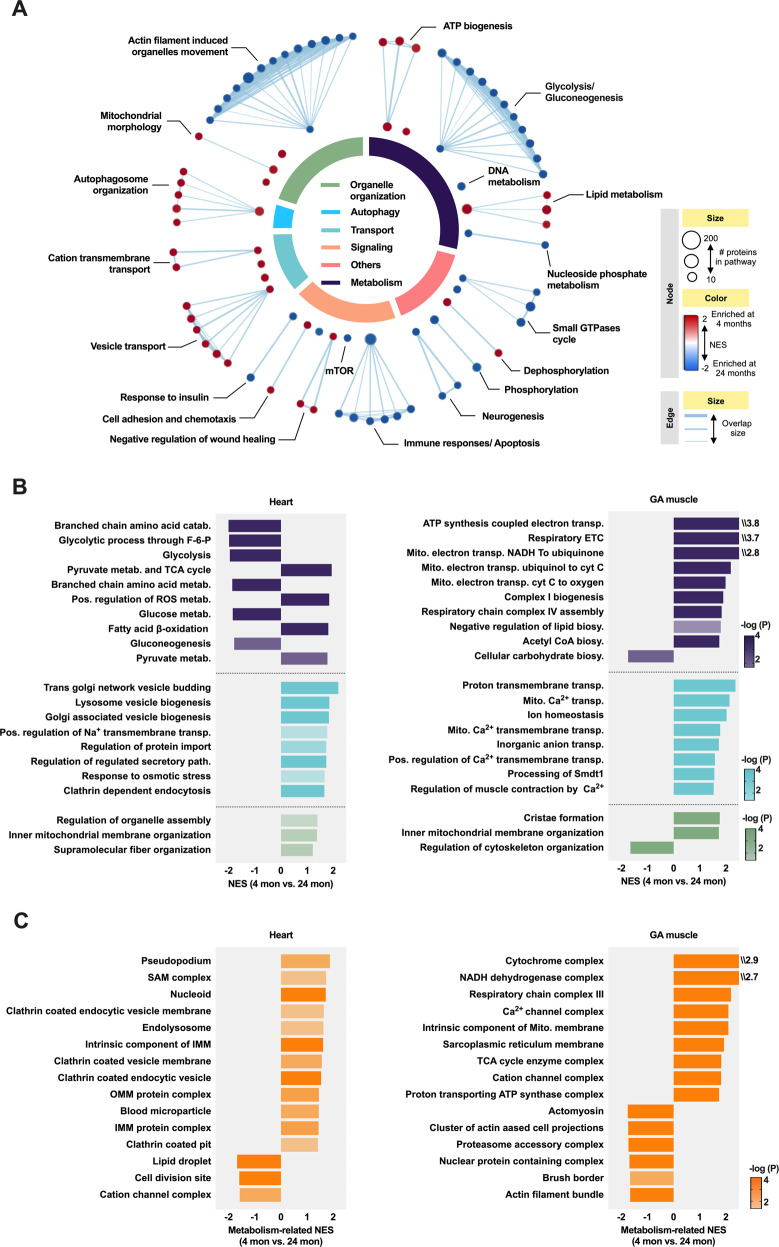
Functional overview of MAM proteome in aged striated muscle. **A** Enrichment map for the striated muscle derived-MAM proteome between 4-month-old vs. 24-month-old rats. Meta-analysis of the MAM proteome derived from the heart and GA muscle were combined using the Fisher’s method (–2*∑ log *P* < 0.05), and plotted with Cytoscape after Gene-Set Enrichment Analysis (GSEA). The enrichment map was created with parameters *q* < 0.1, and Jaccard Overlap combined coefficient >0.375 with combined constant = 0.5. Gene-set nodes were colored according to the Normalized Enrichment Score (NES): red represented enrichment in the 4-month-old group, whereas blue represented enrichment in the 24-month-old group. The number of proteins in each gene-set was proportional to their node size. Edge thickness was proportional to the overlap between gene-sets. All gene-sets have *P* < 0.05. **B** and **C** The GSEA enrichment plots showed the MAM-associated biological processes (**B**) and cellular components (**C**), which were significantly changed during aging in rat heart and GA muscle. The most enriched biological processes involved in metabolism, material transport and organelle organization were highlighted by purple, lime green and green in colors respectively, corresponding to the colors shown in **A**. (*n* = 3 per group, P and NES were calculated by GSEA, and only pathway with –log *P* > 1.5 were shown). Mito. mitochondrial, TCA tricarboxylic acid, Metab. metabolism, ETC electronic transmission chain, IMM inner mitochondrial membrane, OMM outer mitochondrial membrane.

To gain functional insight into the age-related proteomic alterations in the MAM fractions, we performed Gene-Set Enrichment Analysis (GSEA) using a combined dataset derived from heart and GA muscle samples. In the enrichment map, the overall functional “landscape” fitted well with the known role of MAMs as hubs for metabolism, organelle organization, autophagy, transportation, signaling and others (Fig. 5A and Supplementary Table 7). Among these functional categories, the clusters of “Metabolism” occupied the largest part, with glucose metabolism (mainly referred to glycolysis/gluconeogenesis) enriched in the 24-month-old rat group and energetic metabolism (such as ATP biosynthesis and fatty acid beta-oxidation) enriched in the 4-month-old rat group. This was not surprising, since it is well-known that the aged straited muscles undergo a moderate metabolic shift in fuel preference (i.e., from fatty acids to carbohydrates) [26]. Apart from metabolism, many gene-sets mapping to the functional category of “Signaling” (such as immune responses and apoptosis) were strongly induced in the aged MAMs, whereas those related to autophagy/mitophagy (such as autophagosome organization) were repressed during aging. In addition, gene-sets mapping to the categories of “Transport” and “Organelle organization” showed significant down-regulation in the aged MAMs, except for a cluster related to actin filament-induced organelle movement. Most importantly, gene-sets in these groups (such as cation transmembrane transport and mitochondrial membrane organization) are responsible for the fundamental activity and structural integrity of MAMs. The down-regulation of these functions may have a role in supporting the loss of MAM integrity or attenuated ER-mitochondria interaction during aging. Similar enrichment patterns on metabolism (purple), material transportation (lime green) and organelle organization (green) were observed in the GSEA results for single datasets, from either the heart or GA muscle (Fig. 5B). Enrichment analysis of cellular components revealed that several transmembrane protein complexes were affected by aging, such as the OMM import sorting and assembly machinery (SAM) and Ca^2+^ channel complexes (Fig. 5C).

Similarly, pathway enrichment of age-related MAM proteins demonstrated the impaired metabolic activity and defective MAM integrity in striated muscle (Supplementary Fig. 3A). To explore the functional implications of canonical MAM components, we also performed GSEA based on the expression profiles of 176 consensus MAM proteins identified in our datasets. The results further suggested impaired mitochondrial transmembrane transport and reduced metabolic activity in the aged MAMs (Supplementary Fig. 3B).

### Identifying potential protein candidates linking metabolism and membrane architecture at MERCs

**Fig. 6:**
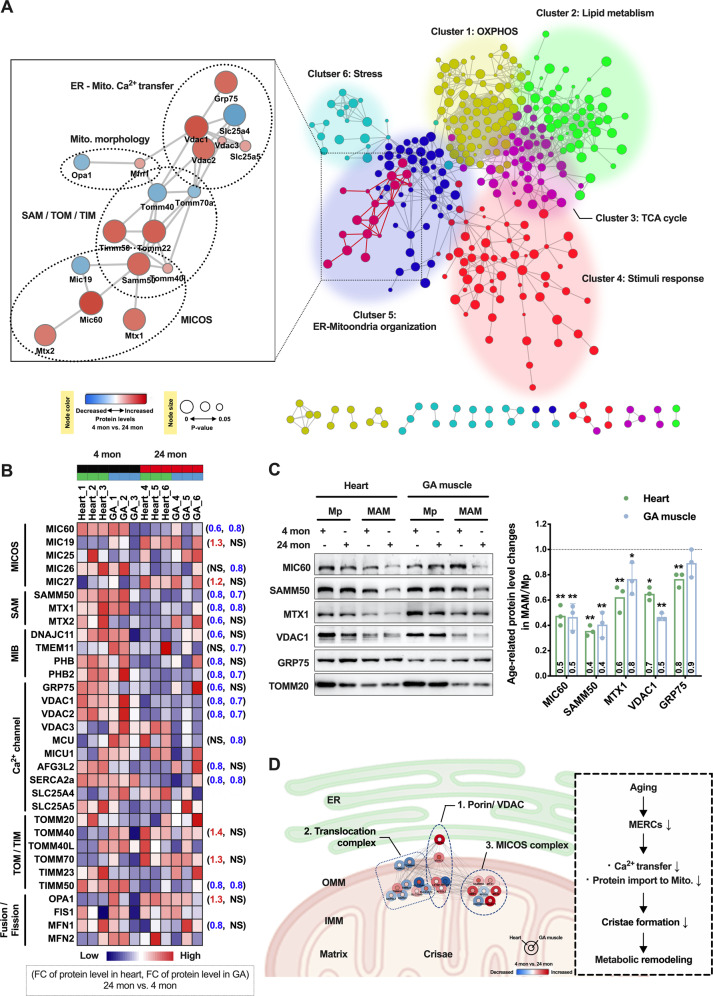
ER-mitochondria membrane organization was perturbed in aged straited muscle. **A** STRING-based Protein–Protein Interaction (PPI) network of age-associated MAM proteins in straited muscle (the right panel). The age-associated proteins were obtained from meta-analysis of combined heart and GA muscle MAM proteome, and visualized as a PPI network using the stringApp in Cytoscape with K-means clustering. In the right panel, node color represented different K-means clusters. The zoomed panel to the left showed a subnetwork of proteins involving ER-mitochondria membrane organization, in which upregulated proteins were colored in red, and downregulated proteins in blue (*n* = 3 per group, 4-month-old vs. 24-month-old). In the entire figure, node size showed significance of the age-associated MAM proteins. **B** Heatmap showing altered expression profile of MAM proteins involving ER-mitochondria membrane organization. Fold changes of protein levels in the 24-month-old group relative to their 4-month-old control were denoted beside the heatmap. NS, not significant. **C** Immunoblot analysis showing significantly altered abundance of MIC60, MTX1, SAMM50, VDAC1 and GRP75 in MAM and pure mitochondrial fractions during aging. Right panel: Age-related abundance changes of the indicated proteins in the MAM fractions relative to the pure mitochondria fractions. The values within the bars showed the mean fold changes of protein levels in the 24-month-old group compared to the 4-month-old control and normalized to TOMM20. (*n* = 3 independent experiments, the variance was similar between groups. *P < 0.05; **P < 0.01). **D** A putative responsive network of MAM proteome during aging. The circles represented deregulated MAM proteins in aging straited muscle, with inner circles indicated GA muscle and outer circles indicated heart. The upregulated proteins were colored in red, and downregulated proteins in blue (4-month-old vs. 24-month-old). Mito. mitochondrial, OXPHOS oxidative phosphorylation, TCA tricarboxylic acid, MICOS the mitochondrial contact site and cristae organizing system, SAM the sorting and assembly machinery complex, MIB mitochondrial intermembrane space bridging complex, TOM/TIM the outer/inner membrane translocase complex, OMM outer mitochondrial membrane, IMM inner mitochondrial membrane, IMS intermembrane space, FC fold changes.

The ER and OMMs, as well as the IMMs, are highly connected as an inter-organelle membrane network via protein–protein interactions. To identify proteins involved in age-related MAM dysfunction, we performed Protein–Protein Interaction (PPI) analysis with differentially expressed proteins from the combined dataset of heart and GA muscle samples. The global interactome of 513 age-related MAM proteins was shown in Fig. 6A. This network was further subdivided into six communities representing complexes or clusters of functionally related proteins, including the oxidative phosphorylation (OXPHOS) complex, TCA cycle, lipid metabolism, cellular response to stimuli or stress, and ER-mitochondria organization (Fig. 6A and Supplementary Table 8). Among these, the ER-mitochondria organization community contained a subset of proteins that were crucial for controlling the ER-mitochondrial membrane architecture and cristae formation (Fig. 6A, enlarged in the left panel). These proteins actually resided in a highly interconnected supramolecular complex (also known as the ER–mitochondria interaction network, ERMIONE) [27] composed of the Ca^2+^ channels, protein translocases SAM/TOM/TIM, fission and fusion components, and the mitochondrial contact site and cristae organizing system (MICOS).

Of note, the supramolecular complex showed an overall down-regulation in the aged group, with several core subunits significantly decreased in both tissue types, such as MIC60 in the MICOS complex, and SAMM50 and MTX1 in the SAM complex, as well as VDAC1 in the Ca^2+^ channel complex (Fig. 6A, B and Supplementary Fig. 4A). Thereby, these proteins may be recognized as potential candidates in shaping the ER-mitochondria membrane architecture and modulating metabolic activity. To validate these results, Western blot analysis showed that the protein levels of MIC60, MTX1, SAMM50 and VDAC1 in the MAM fractions were significantly decreased with aging, while their protein levels in the Mp fractions and mRNA levels were not largely affected (Fig. 6C and Supplementary Fig. 4B). This suggested that the altered subcellular distribution of these subunits, rather than changes in their expression levels, may play a causal role in age-related MAM dysfunction. According to our study, a putative responsive network of the MAM proteome to the aging process is summarized in Fig. 6D. In striated muscle, aging-evoked stress locally impacts the mitochondrial components of MAMs, especially the molecular machines for mitochondrial Ca^2+^ transfer (Porin/VDAC), protein import (SAM/TOM/TIM translocation complexes) and cristae assembly (MICOS complex). Reduced abundance of the core subunits in these complexes (shown in Fig. 6D as expression-view) was considered as readout for the age-related proteomic response. The resulting perturbations in MAM integrity and cristae architecture, however, may amplify the cellular damage, thus leading to metabolic remodeling in the aged striated muscle.

## Discussion

The heart and skeletal muscle are both sensitive to age-related changes, due to their limited regenerative capacity and high oxidative metabolic activity [4]. With aging, a loss of cardiac and skeletal muscle function diminishes the quality of life and increases morbidity and mortality [6]. Our study revealed a combination of LV hypertrophy, diastolic dysfunction with preserved EF, and peripheric skeletal muscle weakness in older rats. The compound phenotype was consistent with the clinical manifestation in older humans [28], of whom the main symptom was exercise intolerance or early fatigue, which reflected a declined functional reverse of the aged striated muscle. Collectively, these findings illustrated that the normal aging rat models accurately recapitulate key aspects of human age-related myopathy, and pointed to the aging hallmarks as potential initiating factors.

Mitochondrial dysfunction has been described as both a major driver and consequence of aging [5]. Dysfunctional mitochondria can contribute to aging through a number of mechanisms, such as production of reactive oxygen species, triggering inflammatory reactions and apoptotic signaling [5]. In tissues with high-energy demand, such as striated muscle, another mitochondrial contributor to aging is the defective bioenergetics [26, 29]. Within the striated myocytes, electrochemical coupling and metabolic adaptations rely on the interconnection between the SR/ER and mitochondria. This privileged inter-organelle communication facilitates mitochondrial ATP transport for SR Ca^2+^ cycling and ensures energy replenishment by reciprocal Ca^2+^ and ADP exchange [12]. An increasing number of evidence support that the controlling mechanism that matches the ATP supply and demand is impaired during aging [26, 30]. Thus, the defective ER–mitochondria communication may play a causal role in the limited ATP production. Our TEM imaging revealed an early onset of accelerated thickening of MERCs over the lifespan. On the basis of the diffusion law, a wider MERCs cleft within the range of 12-24 nm would theoretically slow down the rate of Ca^2+^ transfer, a key aspect in controlling ATP production and energy homeostasis [8]. Thus, the widening of the MERCs cleft during aging would blunt the Ca^2+^-dependent control mechanism thus leading to an ATP supply-demand mismatch. We also noted that the ultrastructural modification in MERCs occurred prior to the changes in mitochondrial morphology. Therefore, the remodeling of MERCs could serve as an early marker for striated muscle aging, possibly indicating an increased risk of age-related mitochondrial dysfunction.

Our study provides two in-depth proteomic profiles of MAMs derived from the rat heart and skeletal muscle. In both tissue types, the proteomic maps of MAMs have not been previously studied. Previously, several proteomic studies have generated lists of over 1,500 MAM-resident proteins [19–21]. A similar amount of identified MAM hits was obtained in the present study. The quality of our datasets was characterized in terms of sensitivity and specificity. The sensitivity of the MAM proteome can be estimated by comparing it to a true positive list of 216 consensus MAM proteins generated from the literature [19–21]. We found the majority of these proteins (81%) were present in our datasets. To characterize its specificity, we employed several bioinformatic tools for spatial mapping of the total MAM proteome. In the entire human proteome, only 8% of proteins have mitochondrial annotation, 4% have an ER annotation, with the remaining proteins mainly having cytosolic (36%) and nucleoplasmic (31%) annotations [31]. In our MAM proteome, there was a strong enrichment of the mitochondrial and ER components over the others with clear absence of the nuclei, indicating a good yield of MAM-resident proteins. However, we observed that there seem to be poor separation for the internal sub-organelle components since some ER lumen or mitochondrial matrix proteins were included in our MAM proteome. It is worth noting that, the consensus MAM sets also contain proteins targeted to the internal compartments of mitochondria or ER (see GO cellular components terms of the consensus MAM proteins in Supplementary Table 5) [19]. These proteins may either have a previously unrecognized MAM distribution, or result from the inherent deficiency in the methodologies currently available. Further research is needed to derive accurate conclusions. Nevertheless, the MAM proteome obtained in this study feature high coverage with moderate specificity, and should be valuable resources for ER-mitochondria interaction studies.

We have shown that the subcellular composition of MAMs could largely influence their biological activity. In striated muscle, the mitochondrial components represented a substantial proportion of the MAM proteome, whereas in the liver [19], brain and testis [24], the ER components were predominant. Thus, the MAM fractions derived from striated muscle would display a higher metabolic potential than those from other tissues. It is reasonable to infer that the mitochondria-predominant MAM composition may facilitate a rapid metabolic adaptation to changing energy demands during myofibril contraction, since the abundant mitochondrial enzymes could generate a metabolically active microdomain, which reacts instantaneously to the ER-driven regulatory signals [32]. However, in tissues like the liver, the ER-predominant MAM composition may serve as a platform for lipid and protein biosynthesis/transfer, as previously demonstrated [25]. That is, different tissue types are characterized by distinct MAM compositional signatures. Moreover, the major components will be responsible for the tissue-specific MAM bioactivities. In light of the subcellular composition discussed above, we propose a proteomic classification of MAMs into the “mitochondria-predominant” subtype featured with high metabolic activity, and the “ER-predominant” subtype with preference for controlling lipid and protein homeostasis. Thus, such classification would provide a mean to speculate on the functions of MAMs in various tissues and physiological conditions. We can clearly see that in striated muscle, the aging process led to a shift of MAMs from the “mitochondria-predominant” subtype to the “ER-predominant” subtype. The loss of mitochondrial components could largely explain the age-related metabolic remodeling of MAMs.

Another aging hallmark of our proteome was the perturbations in MAM integrity. Among the canonical bridging molecules that tether the ER and OMM in mammalian cells [33], a significant reduction of VDAC1 was observed in the MAM proteome from both tissues. We noted that the decreased abundance of VDAC1 was restricted to MAMs rather than the whole mitochondria, suggesting a MAM-specific alteration induced by VDAC1. It is likely that different bridging molecules form tethers of different width, and conceivably, different functions of the MERCs may be associated with them [8]. VDAC1 is well-known to interact with IP3R and GRP75 to form a tethering machinery that coordinates Ca^2+^ transfer [18]. In this work, the average MERCs thickness in the young striated muscle was ≈ 15 nm, which is an ideal width for assembly of the IP3R-GRP75-VDAC1 complex and Ca^2+^ diffusion [34]. Thereby, as a result of the VDAC1 loss in MAMs and the disassembly of the tethering complex with aging, there was a profound thickening of MERCs, along with an impairment in muscle functional reverse due to aberrant local Ca^2+^ dynamics. Furthermore, the VDAC1-dependent tethering complex functionally interacted with other OMM and IMM complexes, to form a large ER-mitochondria organization network (ERMIONE) expanding from the ER to the OMM and IMM [35]. This interaction network not only promotes the transport of lipids and Ca^2+^ from the ER directly to the mitochondrial matrix [36], but also potentially provides a structural basis for ER-oriented mitochondrial protein import and cristae assembly [37, 38]. Hence, it is conceivable that the down-regulation of ERMIONE in aged MAM fractions would destabilize the interaction network, leading to aberrant ER-mitochondria membrane architecture and exchanges. Collectively, these findings suggested an age-related perturbation in MAM integrity, from the VDAC1-dependent tethering complex to a large multi-membrane-spanning connection linking the ER lumen to the mitochondrial matrix.

In conclusion, our comparative quantitative description of the ultrastructural and proteomic changes occurred in the ER-mitochondria junctions, indicates a breakdown of inter-organelle communication during the striated muscle aging process. These findings provide a subcellular clue for the maladaptive responses to aging, whereby the structural and functional uncoupling between ER and mitochondria contributes to local metabolic rewiring. We hope this will suggest research frontiers in integrating subcellular, high-resolution omics data to obtain a better characterization of organelle remodeling and interaction in the context of aging.

## Materials and methods

### Experimental animals

All animal procedures were performed in accordance with the guidelines of the Animal Welfare Ethics Committee of Zhejiang University. Sample size was estimated with the PASS software and based on sample availability. No randomization or blinding was performed, because there were no animal treatment groups. Forty-two F344 and Brown Norway inbred male rats at 4-, 18-, and 24-months of age were studied. The median survival of male and female F344 rats was 28 months [39]. We therefore adopted 18- and 24-month-old male F344 rats as middle-aged and older adult models, respectively. Four-month-old rats were used as young controls. During the study, five rats reached humane endpoints and had to be euthanized, which included four rats in the 24-month-old group and one rat in the 18-month-old group.

### Echocardiography

The conventional transthoracic echocardiography was performed using a VisualSonics Vevo 2100 system (VisualSonics, Toronto, Ontario, Canada) with a MS400 (30 MHz centerline frequency) probe. Rats were anesthetized with isoflurane (Zoetis IsoFlo, Kalamazoo, MI, USA) (induction 3.0% and maintenance 1-3%) throughout the procedure. B- and M-mode images were acquired from a parasternal short-axis view to evaluate left ventricular structure and global systolic function. Diastolic function was evaluated using tissue Doppler imaging (TDI) and pulsed wave Doppler (PWD) techniques. From an apical long-axis view, transmitral inflow velocities were recorded by setting the sample volume in the mitral orifice close to the tip of the mitral leaflets. From the PW spectral waveforms, we measured: the peak early- and late-diastolic transmitral velocities (E and A waves) to obtain the E/A ratio; E-wave deceleration time (DT) and isovolumetric relaxation time (IVRT). From the TDI spectral waveforms, we measured E’ (early-diastolic myocardial relaxation velocity) and calculated E/E’ ratio.

Advanced measures of cardiac contractility were performed using speckle-tracking-based echocardiographic strain analysis. This technique has emerged as a sensitive tool for the early detection of subtle cardiac alterations [40, 41]. The strain echocardiography was performed using a Vivid E95 system (GE Vingmed Ultrasound AS, Horten, Norway) equipped with a 12S (9.0–12.0 MHz) probe. Standard 2D images of the left ventricle (LV) were obtained in the parasternal short-axis view at the midpapillary level and in the apical 4-, 2- and 3 chamber views. All strain analysis was performed with the vendor-dependent software (EchoPAC version 203, GE Vingmed Ultrasound AS, Horten, Norway). The LV endocardial and epicardial borders were manually traced to ensure it truly represented LV wall motion, and poorly tracked segments would be rejected. The strain curves of the global and regional LV wall were generated by the software automatically. Global circumferential, radial, and longitudinal strain (GLS) were assessed as previously described [42]. GLS was averaged from 3 apical views. The reference frame of zero strain was set at LV end-diastole (R-R gating) [43]. All echocardiographic analysis was performed by one investigator experienced with strain imaging and blinded to the animals’ information.

### Muscle function and histological analysis

In vitro muscle function was assessed by platinum electrodes stimulating the isolated GA muscle with a supramaximal current (600 mA; 500 ms train duration; 0.25 ms pulse width) from a base stimulator (Grass S88, Grass Technologies, Warwick, USA) amplified via a high power bi-polar stimulator (701 C, Aurora Scientific Inc., Aurora, Canada). The muscle was set at an optimal length (L_0_) equivalent to the maximal twitch force produced, after which bath temperate was increased to 25 °C and a 15 min thermo-equilibration period followed. A force-frequency protocol was then performed at 10, 30, 50, 70, 100, 120, and 150 Hz respectively, separated with 1 min rest intervals.

Treadmill exhaustion test was performed to evaluate exercise activity in vivo. The running protocol was started with an adaptation period of 10 m/min for 20 min before an increase of 2 m/min every 20 min until fatigue response initiated. Rats were considered to be exhausted when the animal’s hindlimbs remained on the electric grid despite receiving three shocks. Upon fatigue initiation, the rats were quickly removed from treadmill running lane and the running distance were calculated.

For quantification of cross-sectional area (CSA), transverse sections from frozen gastrocnemius (GA) muscles were stained with wheat germ agglutinin-Alexa 594 conjugate (WGA, Thermo, Waltham, USA) and DAPI, with the area of single myofibers (over 100 fibers were analyzed for each rat) evaluated using Image J software.

### Transmission electron microscopy and statistical analysis

Freshly dissected tissue samples from LV and GA muscle (pre-perfused with PBS) were fixed overnight using 2.5% glutaraldehyde, sequentially incubated with 1% OsO_4_ for 1 h and immersed in 2% uranyl acetate for 30 min. After dehydration in a gradient of ethanol and acetone, the samples were embeded in epon resin. Ultrathin sections were contrasted with 1% uranyl acetate and lead citrate. Three representative areas containing transverse fibers were randomly selected for each sample, and imaged using a Tecnai G2 Spirit transmission electron microscopy (TEM) from FEI. TEM analysis was performed blinded using the ImageJ software. For morphometric analysis of mitochondria, the aspect ratio (a measure of the length to width ratio), circularity (4*π*·(surface area/perimeter^2^)) and area of individual intermyofibrillar mitochondrion were calculated. Cristae and MERCs were identified manually from high-resolution, high-magnification TEM images, and they were outlined using an optical pen in ImageJ to calculate their structural parameters. Cristae abundance was estimated by the total cristae length per mitochondrial area (μm/μm^2^). MERCs were manually identified according to two well-established criteria: being ribosome-free and having a gap of 10–30 nm between the OMM and the ER [8]. The MERCs coverage was calculated as the percentage of mitochondrial perimeter covered by MERCs. As the thickness of individual MERC is rather uniform, it was quantified as an average distance measured from three randomly selected positions between the two organelle membranes [8]. A schematic representation of the ultrastructural analysis approach for MERCs was shown in the Supplementary Fig. 5.

### Subcellular fractionation of MAMs

Heart or GA muscle subcellular fractionation was performed by strictly following a previously reported protocol [15]. In brief, the fresh tissues dissected from LV and bilateral GA muscles were washed and cut into small pieces using scissors. The GA muscles need to be softened in ice-cold PBS supplemented with 0.5 mM EDTA and 0.25% trypsin for 30 min before mincing [15]. Samples were then homogenized by a motor-driven homogenizer (WIGGENS WB6000-D, Berlin, Germany). The homogenate was centrifuged two times at 740 × *g* to remove unbroken cells and nuclei. The obtained supernatant was centrifuged twice at 9000 × *g* to pellet crude mitochondria. To subfraction for cytosolic and ER fractions (microsome), the obtained supernatant was centrifuged at 20,000 × *g* for 30 min, and at 100,000 × *g* for 1 h to give a supernatant (cytosol) and a pellet (ER). Crude mitochondria were layered on the top of the 30% Percoll medium (Sigma-Aldrich, St. Louis, MO, USA) and centrifuged at 95,000 × *g* for 30 min to obtain MAMs (interphase) and the pure mitochondrial fractions (pellet). The collected MAMs was sequentially centrifugated at 100,000 × *g* for 1 h to obtain highly purified MAM fractions. The isolated subcellular fractions were validated by immunoblot and TEM analysis. The protein content (in mg) of the MAM fractions or pure mitochondria was quantified as total volume multiplied by protein concentration and normalized by total tissue extract (in g). The protein concentration was determined using a BCA kit (Meilunbio, Dalian, China).

### Nano-liquid chromatography with tandem mass spectrometry (LC-MS/MS) analysis

According to the protein concentration, 40 μg of MAM lysates were adjusted to 100 μl volume with 100 mM triethylammonium bicarbonate (TEAB). Samples were reduced by 10 mM tris (2-carboxyethyl) phosphine and incubated at 55 °C for 45 min followed by alkylation by 30 mM iodoacetamide in the dark for 30 min. Proteins were then precipitated by acetone and digested by 1 μg trypsin (Promega, Wisconsin, USA) in 40 μl 100 mM TEAB at 37 °C for 16 h. After digestion, a set of 6-plex TMT isobaric isotope labels (Thermo Fisher) was used to label all 6 samples from hearts or GA muscles according to manufacturer’s instruction. After labeling, peptides from each sample were combined, completely dried using a freeze dryer (Labconco Corporation, Kansas City, MO, USA), and resuspended in 100 μl of 2% acetonitrile (ACN) and 0.1% formic acid (FA). The peptides were desalted by 100 mg Sep-pak C18 column (WAT043395, Waters, USA) and dried again before nano-LC-MS/MS analysis.Labeled peptides were separated by the U3000 UPLC nano-liquid phase (Thermo Fisher) system equipped with Acclaim PepMap 100 C18 column (Thermo Fisher) with a flow rate of 400 nl/min. Mobile phase A: 0.1% FA, B: 0.1% FA dissolved in 98% ACN. The 120-minute concentration gradient is set as follows: 6 min 2–5% B; 64 min 5–18% B; 20 min 18–32% B; 10 min 32–80% B. It was then held at 80% B for 10 min, and then returned to 3% ACN within 4 min and kept under rebalanced conditions for 5 min until the next injection. The peptides were sprayed into the Q Exactive HF-X mass spectrometry via nanospray source with voltage at 2.0 kV. The mass spectrometer was operated using the dependent acquisition mode with a full MS1 scan at 70,000 FWHM resolution (*m*/*z* 200 Th) using automatic gain control (AGC = 10e6) followed by 20 MS2 scans with a resolution of 45,000 FWHM. The precursor was fragmented by high-energy collision dissociation, with normalized collision energy set to 28%. MS2 AGC was set to 5e4 and dynamic exclusion was set to 40 s.

The collected RAW files were loaded into the MaxQuant software with 6-plex TMT quantitative module, and the UniProt database for Rattus norvegicus and common contamination sequences were searched. Other search parameters were set as follows: oxidation and carbamoyl were set as variable and fixed modification respectively; protease was set to trypsin and a maximum of two missed cut sites were allowed, and the mass error was set to 7 ppm. The results were filtered by peptide-spectrum-match and protein level 1% FDR. For quantitative analysis, relative protein intensity in each sample was normalized again to the median to correct for systematic errors between different labels. The resulting datasets were further statistically meta-analyzed by NetworkAnalyst 3.0. The differential expression analysis was performed on each individual dataset or a combined proteomic profile of the heart and GA muscle MAM fractions (using Fisher’s method for *P*-value combination). An optimized cutoff of protein expression ratio >1.2 or <0.83 and *P* < 0.05 was used to determine age-related MAM proteins. The volcano plots and heat maps were created using Prism 8.0 with log_2_-transformed data. Subcellular location enrichment networks were constructed by NetworkAnalyst 3.0 in Bipartite view showing enriched PANTHER: CC (Cellular Component) terms with differential proteins in the heart or GA muscle datasets. The Venn diagrams were generated with a web-based tool (http://bioinfogp.cnb.csic.es/tools/venny/).

### Subcellular mapping of MAM proteomes

Different proteome-wide subcellular localization methods were used for spatial mapping of the total MAM proteomes, including the proximity biotinylation-dependent humancellmap (with the NMF- and SAFE-based location prediction) [44], the protein annotation databases Uniprot and Gene Ontology Consortium (GO), the clustering algorithm-based subcellular location assignment tool (“MetaMass”) [45], as well as the immunofluorescence-based Human Protein Atlas (HPA) [31]. Full-text annotations from Uniprot and GO were retrieved from the Uniprot website (http://www.uniprot.org), while those from the HPA [46] were retrieved from the Human Protein Atlas website (http://www.proteinatlas.org). NMF- and SAFE-based location predictions [44], as well as the fractionation-based localizations from Christoforou et al. [47] were retrieved as Excel files from journal webpages. To better recognize the spatial organization of MAMs, proteins only listed with a single location in Uniprot, GO or HPA were incorporated into the location analysis. The location predictions by MetaMass for individual proteins were obtained as an output table following the user guide [45], using the marker sets derived from Uniprot, GO and Christoforou et al. [47]. To generate a heatmap by MetaMass, the high-resolution proteomic dataset from Christoforou et al. [47] were K-means clustered and classified into eight subcellular compartments, the data from our MAM proteomes were afterwards aligned to the reference dataset for subcellular mapping.

### Western blot analysis

Western blots were performed on isolated subcellular fractionations resolved in lysing buffer containing: 50 mm Tris-HCl, pH 7.5, 150 mm NaCl, 2 mm EDTA, 2 mm EGTA, and 1% SDS supplemented with a protease inhibitor mixture (NCM Biotech, Suzhou, China). Protein samples were separated by sodium dodecyl-sulfate polyacrylamide gel electrophoresis (SDS-PAGE) and blotted to PVDF membranes (Millipore, Sigma-Aldrich, Ireland). After blocking in 5% non-fat milk, the following primary antibodies were used: anti-Calnexin rabbit pAb (1:1000, Cat.A15631, ABclonal, Wuhan, China), anti-Calreticulin rabbit pAb (1:1000, Cat.A18013, ABclonal), anti-GRP75 mouse mAb (1:1000, Cat. sc-133137, Santa Cruz, CA, USA), anti-IP3R rabbit pAb (1:1000, Cat. ab5804, Abcam, MA, USA), anti-VDAC1 mouse mAb (1:1000, Cat. Ab186321, Abcam), anti-TOMM20 rabbit mAb (1:1000, Cat. Ab187735, Abcam,), anti-SAMM50 rabbit pAb (1:1000, Cat. ab246987, Abcam), anti-MIC60 (1:1000, Cat. 10179-1-AP, Proteintech, Chicago, USA), anti-MTX1 rabbit pAb (1:1000, ab233205, Abcam), anti-GAPDH Rabbit mAb (1:5000, Cat. A19056, ABclonal). After incubation of the blots with horseradish peroxidase (HRP)-conjugated secondary antibodies for 1 h, signals were detected using the ECL (YEASON, Shanghai, China). Quantification was performed with Image Lab software for the ChemiDoc XRS system (Bio-Rad, Hercules, CA).

### Gene expression analysis

Total RNA was isolated using Trizol reagent (Thermo Fisher) following manufacturer’s instructions. cDNA was generated using 2 μg of RNA and the Multiscribe Reverse Transcriptase (Thermo Fisher). For gene expression analysis, qPCR reactions were conducted using SYBR Green qPCR master mix (Applied Biosystems, CA, USA) and analyzed by a CFX 384 Real-Time system (Bio-Rad, CA, USA). The oligos used are described in Supplementary Table 9.

### Functional enrichment analysis

Gene-set enrichment analysis (GSEA) was performed with the GSEA 4.1.0 software (http://www.broad.mit.edu/gsea), using gene matrix from websites, including c2.cp.reactome.v7.4.symbols.gmt, c5.go.bp.v7.4.symbols.gmt, c2.cp.biocarta.v7.4.symbols.gmt, c2.cp.kegg.v7.4.symbols.gmt [48]. The GSEA enrichment map was created using the EnrichmentMap plugin [49] for Cytoscape [50], broadly following a published protocol [51]. The parameters used to create the map include *q* < 0.1, and Jaccard Overlap combined coefficient >0.375 with combined constant = 0.5. Groupings were facilitated by the Cytoscape AutoAnnotate plugin [52]. In the enrichment map for the combined striated muscle MAM proteome, only gene sets enriched with *P* < 0.05 were plotted. Functionally coherent gene-sets were automatically grouped into clusters, which could be manually mapped to six major functional themes, including metabolism, organelle organization, autophagy, transportation, signaling and others. In the bar plots showing GSEA results for MAM proteome in rat heart or GA muscle, only gene sets enriched with –log *P* > 1.5 were shown. In the bar plots showing GSEA results for consensus MAM proteins in rat heart or GA muscle, only the top 20 gene-sets with the highest NES were shown.

### Protein–protein interaction analysis

The STRING Protein–protein interaction (PPI) analysis was performed on a total of 525 age-associated MAM proteins from the combined striated muscle dataset using the STRING website (https://string-db.org/, version 11.5) [53]. A minimum required interaction score of 0.8 was selected. Disconnected nodes were hidden. The interaction network was organized by *K*-means clustering into complexes or functional related clusters. Visualization and functional characterization of the clusters was performed using Cytoscape stringApp [54]. Statistically significant terms that span the categories of GO Biological Process, KEGG Pathway, Reactome Pathways and STRING Clusters, were further filtered to eliminate redundant terms. Then the clusters were manually named according to the representative pathways with high significance and covering the most proteins for each cluster. The protein expression data derived from the single or combined dataset of heart and GA muscle, were automatically integrated into the network using the Cytoscape stringApp, shown as node colors ranging from red to bule.

### Statistical analysis

GraphPad Prism 8 software was used for statistical analyses. The correlation analyses between MERC coverage and the morphological parameters of mitochondria were calculated using simple linear regression to determine the correlation constant (*r*) and *P*-value. For experiments with two groups, statistical analyses were performed using Student’s *t*-test (two-tailed). For more than two groups, statistical analyses were performed using analysis of variance, followed by the Bonferroni post hoc test to determine the differences within and between groups. Data were presented as mean ± SD. No samples/results were excluded from the analyses. The significance between samples was denoted as *P < 0.05; **P < 0.01. The number of biological replicates was presented by individual data points in each bar graph, or listed in the figure legends for the Violin plots.

## Supplementary information

The reproducibility checklist

Supplementary Figure 1–5

Supplementary Table 1.Age-related echocardiographic parameters in rats.

Supplementary Table 2. Ultrastructural analysis of mitochondria and MERCs.

Supplementary Table 3. Total MAM proteome.

Supplementary Table 4. Differential expression analysis.

Supplementary Table 5. Consensus MAM proteins.

Supplementary Table 6. Subcellular mapping of the MAM proteome.

Supplementary Table 7. GSEA summary.

Supplementary Table 8. STRING interaction network.

Supplementary Table 9. List of oligos used for qPCR assays.

Uncropped Western blots

## Data Availability

The datasets used and/or analyzed during the current study are available from the corresponding author on reasonable request.

## References

[1] Laddu DR, Ozemek C, Sabbahi A, Severin R, Phillips SA, Arena R (2021). Prioritizing movement to address the frailty phenotype in heart failure. Prog Cardiovasc Dis.

[2] Bekfani T, Pellicori P, Morris DA, Ebner N, Valentova M, Steinbeck L (2016). Sarcopenia in patients with heart failure with preserved ejection fraction: Impact on muscle strength, exercise capacity and quality of life. Int J Cardiol.

[3] Cohen S, Nathan JA, Goldberg AL (2015). Muscle wasting in disease: molecular mechanisms and promising therapies. Nat Rev Drug Discov.

[4] Petr MA, Alfaras I, Krawcyzk M, Bair WN, Mitchell SJ, Morrell CH, et al. A cross-sectional study of functional and metabolic changes during aging through the lifespan in male mice. Elife. 2021;10:e62952. 10.7554/eLife.62952 PMC8099423 33876723

[5] Lopez-Otin C, Blasco MA, Partridge L, Serrano M, Kroemer G (2013). The hallmarks of aging. Cell.

[6] Upadhya B, Taffet GE, Cheng CP, Kitzman DW (2015). Heart failure with preserved ejection fraction in the elderly: scope of the problem. J Mol Cell Cardiol.

[7] Mottis A, Herzig S, Auwerx J (2019). Mitocellular communication: Shaping health and disease. Science..

[8] Giacomello M, Pellegrini L (2016). The coming of age of the mitochondria-ER contact: a matter of thickness. Cell Death Differ.

[9] Janikiewicz J, Szymanski J, Malinska D, Patalas-Krawczyk P, Michalska B, Duszynski J (2018). Mitochondria-associated membranes in aging and senescence: structure, function, and dynamics. Cell Death Dis.

[10] Pinton P, Rimessi A, Marchi S, Orsini F, Migliaccio E, Giorgio M (2007). Protein kinase C beta and prolyl isomerase 1 regulate mitochondrial effects of the life-span determinant p66Shc. Science..

[11] Ziegler DV, Vindrieux D, Goehrig D, Jaber S, Collin G, Griveau A (2021). Calcium channel ITPR2 and mitochondria-ER contacts promote cellular senescence and aging. Nat Commun.

[12] Fernandez-Sanz C, Ruiz-Meana M, Miro-Casas E, Nunez E, Castellano J, Loureiro M (2014). Defective sarcoplasmic reticulum-mitochondria calcium exchange in aged mouse myocardium. Cell Death Dis.

[13] Cury DP, Dias FJ, Sosthenes MC, Dos Santos Haemmerle CA, Ogawa K, Da Silva MC (2013). Morphometric, quantitative, and three-dimensional analysis of the heart muscle fibers of old rats: transmission electron microscopy and high-resolution scanning electron microscopy methods. Microsc Res Tech.

[14] Christian CJ, Benian GM (2020). Animal models of sarcopenia. Aging Cell.

[15] Wieckowski MR, Giorgi C, Lebiedzinska M, Duszynski J, Pinton P (2009). Isolation of mitochondria-associated membranes and mitochondria from animal tissues and cells. Nat Protoc.

[16] Manganelli V, Matarrese P, Antonioli M, Gambardella L, Vescovo T, Gretzmeier C (2021). Raft-like lipid microdomains drive autophagy initiation via AMBRA1-ERLIN1 molecular association within MAMs. Autophagy..

[17] Garofalo T, Matarrese P, Manganelli V, Marconi M, Tinari A, Gambardella L (2016). Evidence for the involvement of lipid rafts localized at the ER-mitochondria associated membranes in autophagosome formation. Autophagy..

[18] Szabadkai G, Bianchi K, Varnai P, De Stefani D, Wieckowski MR, Cavagna D (2006). Chaperone-mediated coupling of endoplasmic reticulum and mitochondrial Ca2+ channels. J Cell Biol.

[19] Carreras-Sureda A, Jana F, Urra H, Durand S, Mortenson DE, Sagredo A (2019). Non-canonical function of IRE1alpha determines mitochondria-associated endoplasmic reticulum composition to control calcium transfer and bioenergetics. Nat Cell Biol.

[20] Sala-Vila A, Navarro-Lerida I, Sanchez-Alvarez M, Bosch M, Calvo C, Lopez JA (2016). Interplay between hepatic mitochondria-associated membranes, lipid metabolism and caveolin-1 in mice. Sci Rep.

[21] Poston CN, Krishnan SC, Bazemore-Walker CR (2013). In-depth proteomic analysis of mammalian mitochondria-associated membranes (MAM). J Proteom.

[22] Lewis SC, Uchiyama LF, Nunnari J (2016). ER-mitochondria contacts couple mtDNA synthesis with mitochondrial division in human cells. Science..

[23] Hamasaki M, Furuta N, Matsuda A, Nezu A, Yamamoto A, Fujita N (2013). Autophagosomes form at ER-mitochondria contact sites. Nature..

[24] Wang X, Wen Y, Dong J, Cao C, Yuan S (2018). Systematic in-depth proteomic analysis of mitochondria-associated endoplasmic reticulum membranes in mouse and human testes. Proteomics..

[25] Ma JH, Shen S, Wang JJ, He Z, Poon A, Li J (2017). Comparative proteomic analysis of the mitochondria-associated ER membrane (MAM) in a long-term type 2 diabetic rodent model. Sci Rep.

[26] Yaniv Y, Juhaszova M, Sollott SJ (2013). Age-related changes of myocardial ATP supply and demand mechanisms. Trends Endocrinol Metab.

[27] Pfanner N, Warscheid B, Wiedemann N (2019). Mitochondrial proteins: from biogenesis to functional networks. Nat Rev Mol Cell Biol.

[28] Sanders NA, Supiano MA, Lewis EF, Liu J, Claggett B, Pfeffer MA (2018). The frailty syndrome and outcomes in the TOPCAT trial. Eur J Heart Fail.

[29] Ubaida-Mohien C, Lyashkov A, Gonzalez-Freire M, Tharakan R, Shardell M, Moaddel R, et al. Discovery proteomics in aging human skeletal muscle finds change in spliceosome, immunity, proteostasis and mitochondria. Elife. 2019;8:e49874. 10.7554/eLife.49874 PMC6810669 31642809

[30] Picca A, Mankowski RT, Burman JL, Donisi L, Kim JS, Marzetti E (2018). Mitochondrial quality control mechanisms as molecular targets in cardiac ageing. Nat Rev Cardiol.

[31] Thul PJ, Akesson L, Wiking M, Mahdessian D, Geladaki A, Ait Blal H (2017). A subcellular map of the human proteome. Science.

[32] Gbel J, Engelhardt E, Pelzer P, Sakthivelu V, Jahn HM, Jevtic M (2020). Mitochondria-Endoplasmic Reticulum Contacts in Reactive Astrocytes Promote Vascular Remodeling. Cell Metab.

[33] Gordaliza-Alaguero I, Canto C, Zorzano A (2019). Metabolic implications of organelle-mitochondria communication. EMBO Rep.

[34] Filadi R, Pozzan T (2015). Generation and functions of second messengers microdomains. Cell Calcium.

[35] Wiedemann N, Meisinger C, Pfanner N (2009). Cell biology. Connecting organelles. Science..

[36] van der Laan M, Bohnert M, Wiedemann N, Pfanner N (2012). Role of MINOS in mitochondrial membrane architecture and biogenesis. Trends Cell Biol.

[37] Tirrell PS, Nguyen KN, Luby-Phelps K, Friedman JR. MICOS subcomplexes assemble independently on the mitochondrial inner membrane in proximity to ER contact sites. J Cell Biol. 2020;219:e202003024. 10.1083/jcb.202003024 PMC7545361 33053165

[38] Latorre-Muro P, O’Malley KE, Bennett CF, Perry EA, Balsa E, Tavares CDJ (2021). A cold-stress-inducible PERK/OGT axis controls TOM70-assisted mitochondrial protein import and cristae formation. Cell Metab.

[39] Solleveld HA, Haseman JK, McConnell EE (1984). Natural history of body weight gain, survival, and neoplasia in the F344 rat. J Natl Cancer Inst.

[40] Yoshida Y, Nakanishi K, Daimon M, Ishiwata J, Sawada N, Hirokawa M (2019). Alteration of cardiac performance and serum B-type natriuretic peptide level in healthy aging. J Am Coll Cardiol.

[41] de Lucia C, Wallner M, Eaton DM, Zhao H, Houser SR, Koch WJ (2019). Echocardiographic strain analysis for the early detection of left ventricular systolic/diastolic dysfunction and dyssynchrony in a mouse model of physiological aging. J Gerontol A Biol Sci Med Sci.

[42] Voigt JU, Pedrizzetti G, Lysyansky P, Marwick TH, Houle H, Baumann R (2015). Definitions for a common standard for 2D speckle tracking echocardiography: consensus document of the EACVI/ASE/Industry Task Force to standardize deformation imaging. J Am Soc Echocardiogr.

[43] Aiello A, Farzaneh F, Candore G, Caruso C, Davinelli S, Gambino CM (2019). Immunosenescence and Its Hallmarks: how to oppose aging strategically? A review of potential options for therapeutic intervention. Front Immunol.

[44] Go CD, Knight JDR, Rajasekharan A, Rathod B, Hesketh GG, Abe KT (2021). A proximity-dependent biotinylation map of a human cell. Nature..

[45] Lund-Johansen F, de la Rosa Carrillo D, Mehta A, Sikorski K, Inngjerdingen M, Kalina T (2016). MetaMass, a tool for meta-analysis of subcellular proteomics data. Nat Methods.

[46] Uhlen M, Fagerberg L, Hallstrom BM, Lindskog C, Oksvold P, Mardinoglu A (2015). Proteomics. Tissue-based map of the human proteome. Science.

[47] Christoforou A, Mulvey CM, Breckels LM, Geladaki A, Hurrell T, Hayward PC (2016). A draft map of the mouse pluripotent stem cell spatial proteome. Nat Commun.

[48] Subramanian A, Tamayo P, Mootha VK, Mukherjee S, Ebert BL, Gillette MA (2005). Gene set enrichment analysis: a knowledge-based approach for interpreting genome-wide expression profiles. Proc Natl Acad Sci USA.

[49] Merico D, Isserlin R, Stueker O, Emili A, Bader GD (2010). Enrichment map: a network-based method for gene-set enrichment visualization and interpretation. PLoS ONE.

[50] Shannon P, Markiel A, Ozier O, Baliga NS, Wang JT, Ramage D (2003). Cytoscape: a software environment for integrated models of biomolecular interaction networks. Genome Res.

[51] Reimand J, Isserlin R, Voisin V, Kucera M, Tannus-Lopes C, Rostamianfar A (2019). Pathway enrichment analysis and visualization of omics data using g:Profiler, GSEA, Cytoscape and EnrichmentMap. Nat Protoc.

[52] Kucera M, Isserlin R, Arkhangorodsky A, Bader GD (2016). AutoAnnotate: A Cytoscape app for summarizing networks with semantic annotations. F1000Res.

[53] Szklarczyk D, Gable AL, Lyon D, Junge A, Wyder S, Huerta-Cepas J (2019). STRING v11: protein-protein association networks with increased coverage, supporting functional discovery in genome-wide experimental datasets. Nucleic Acids Res.

[54] Doncheva NT, Morris JH, Gorodkin J, Jensen LJ (2019). Cytoscape StringApp: network analysis and visualization of proteomics data. J Proteome Res.

