# Supplementary material for: Ultrastructural and proteomic profiling of mitochondria-associated endoplasmic reticulum membranes reveal aging signatures in striated muscle
Source: Cell Death Dis. 2022 Apr 2;13(4):296. doi: 10.1038/s41419-022-04746-4 (PMC8976840; doi:10.1038/s41419-022-04746-4)
Supplement: Supplementary file 2 — Supplementary Figure 1–5 [file 41419_2022_4746_MOESM2_ESM.pdf]

Supplementary Figures:

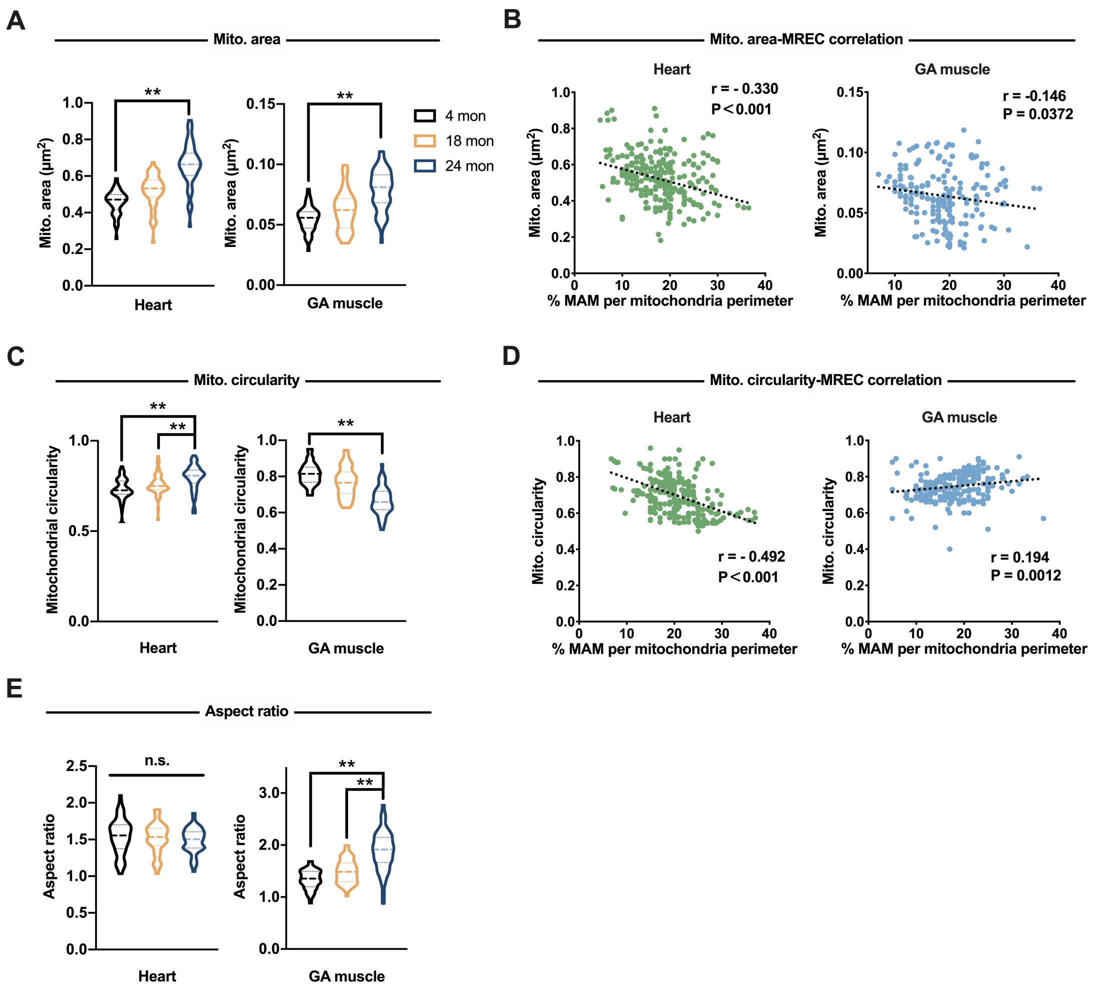

Supplementary Figure 1. Effects of aging on mitochondrial morphology in heart and GA muscle. (A) Effect of aging on mitochondrial area in heart (left) and GA muscle (right). **(B)** The correlation between mitochondrial area and MERC coverage from samples described in A were calculated, using simple linear regression analyses to determine the correlation constant (r) and P value. Total mitochondria quantified in hearts/GA muscles: n = 235/210. **(C and E)** TEM-derived morphological parameters of intermyofibrillar mitochondria in heart (left) and GA muscle (right). The mitochondrial circularity **(C)** was calculated as 4π*(area/perimeter^2), and the aspect ratio **(E)** was measured as length to width ratio. (n = 4 rats per group; total mitochondria

quantified in hearts/GA muscles: n = 122/80 for 4-month-old group, n = 126/79 for 18-month-old group, n = 110/80 for 24-month-old group). **(D)** The correlation between mitochondrial circularity and MERC coverage from samples described in C and E were calculated, using simple linear regression analyses. Total mitochondria quantified in hearts/GA muscles: n = 235/210. Data represent mean ± SD. The variance was similar between the groups that were being compared. **P < 0.01.

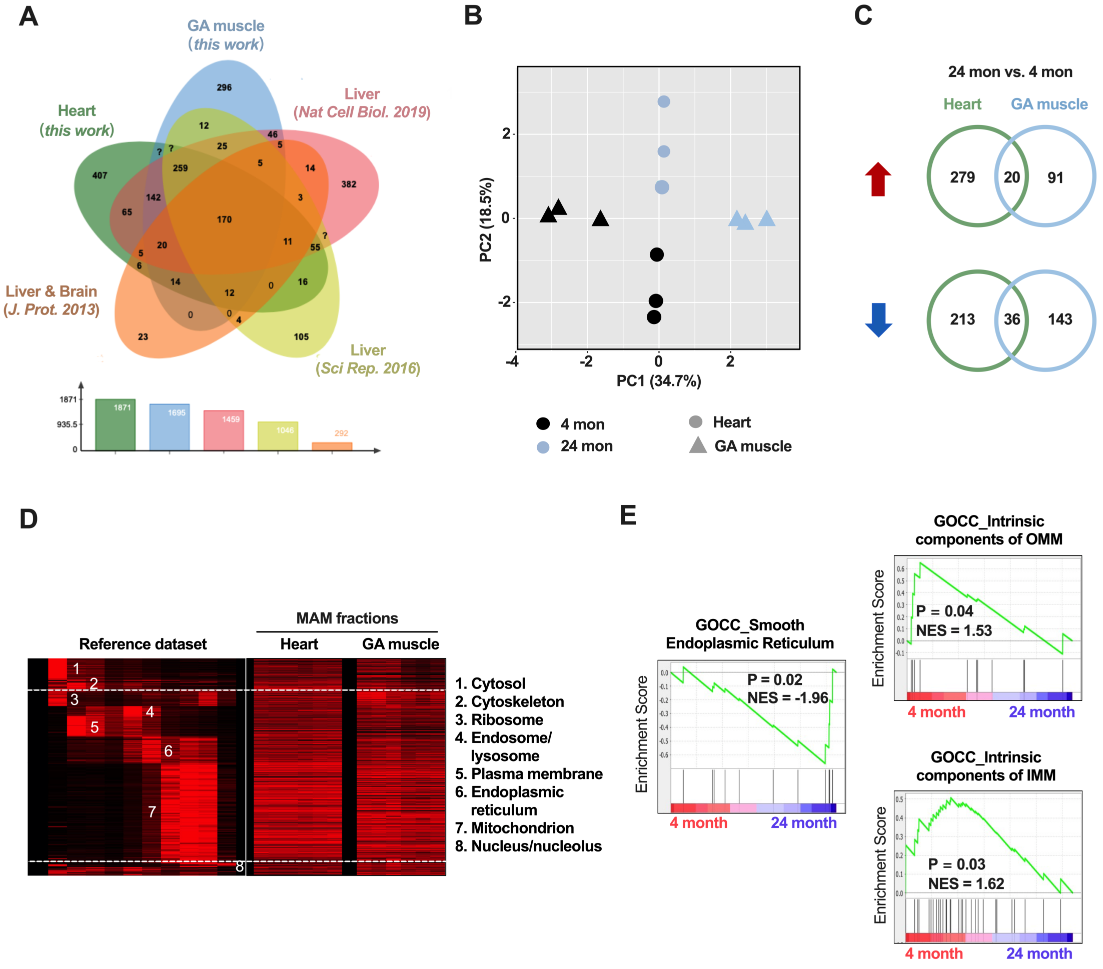

Supplementary Figure 2. Proteomic identification of MAM proteins. (A) Venn diagrams representing the intersections among MAM proteomic datasets from different tissue types. The heart and GA muscle MAM proteome were from this work, datasets on liver and brain were obtained from three other studies (related to Supplementary

Tables 5). **(B)** Principal component analysis (PCA) of the samples. Results from three biological replicates. **(C)** Venn diagrams showing overlaps of age-related MAM proteins in the heart and GA muscle. The red and blue arrows indicated up- and down-regulated proteins in the 24-month-old group vs. the 4-month-old group, respectively. Statistical significance of the differential expressed proteins was evaluated with $P < 0.05$. **(D)** Subcellular mapping of the MAM proteome using the meta-analysis tool "MetaMass". The left heatmap showed a subcellular proteomics dataset with high resolution and well coverage of cytoplasmic organelles from Christoforou et al. The "Christoforou" dataset was clustered and classified into eight fractions and served as a visual reference. The right heatmaps showed the MAM proteome of rat heart and GA muscle that were aligned to the clustered reference dataset by "MetaMass", thus help visualizing the subcellular mapping of MAM proteins as a heatmap view. The white dotted lines on the heatmap classified the eight subcellular fractions into cytosol (fraction 1), membrane (fraction 2-7) and nuclei (fraction 8). **(E)** The GSEA results showing the increase of ER-derived components, as well as the decrease of IMM and OMM components in the aged MAM fractions. The GSEA was performed using the combined dataset derived from the heart and GA muscle samples. OMM, outer mitochondrial membranes; IMM, inner mitochondrial membranes; NES, normalized enrichment score.

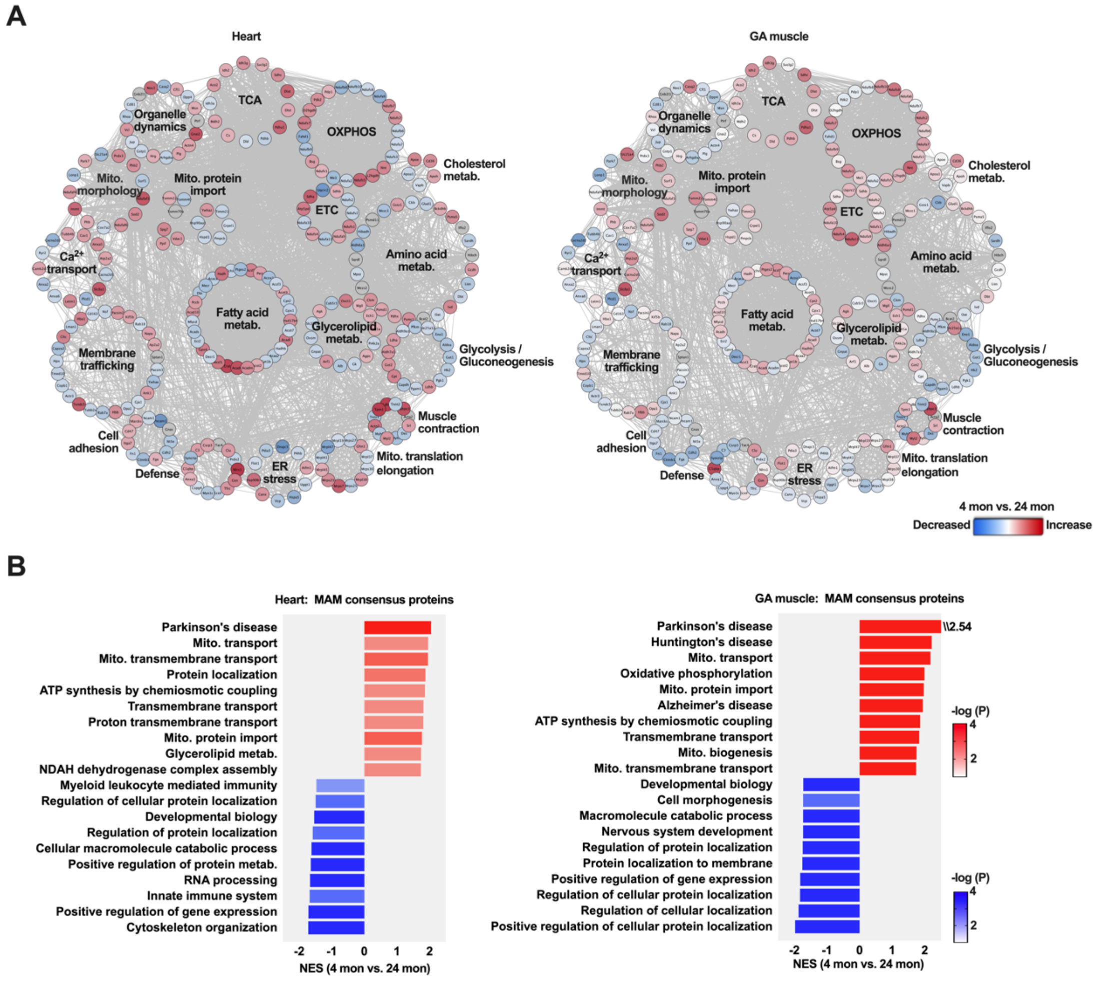

Supplementary Figure 3. Functional overview of the age-related MAM proteins in the heart and GA muscle. (A) MAM proteomic landscapes showing the main categories altered in the aged heart (left) and GA muscle (right). Each dot represented an age-associated deregulated MAM protein, color-coded according to mean relative abundance in the MAM proteome: red represented up-regulated, whereas blue represented down-regulated, in the 4-month-old group vs. 24-month-old group (n =3 per group, P ＜ 0.05). **(B)** Bar plots showing the GSEA results on 176 consensus MAM proteins between the 4-month-old vs. 24-month-old groups, in rat heart (left) and GA muscle (right). The gene-set bars were colored according to the Normalized Enrichment Score (NES): red represented enrichment at 4 months, whereas blue

represented enrichment at 24 months. The color gradient showed the -log P of each gene-set. (n = 3 per group, only the top 20 gene-sets with the highest NES were shown). Mito., mitochondrial; TCA, tricarboxylic acid; Metab., metabolism; OXPHOS, oxidative phosphorylation; ETC, electronic transmission chain.

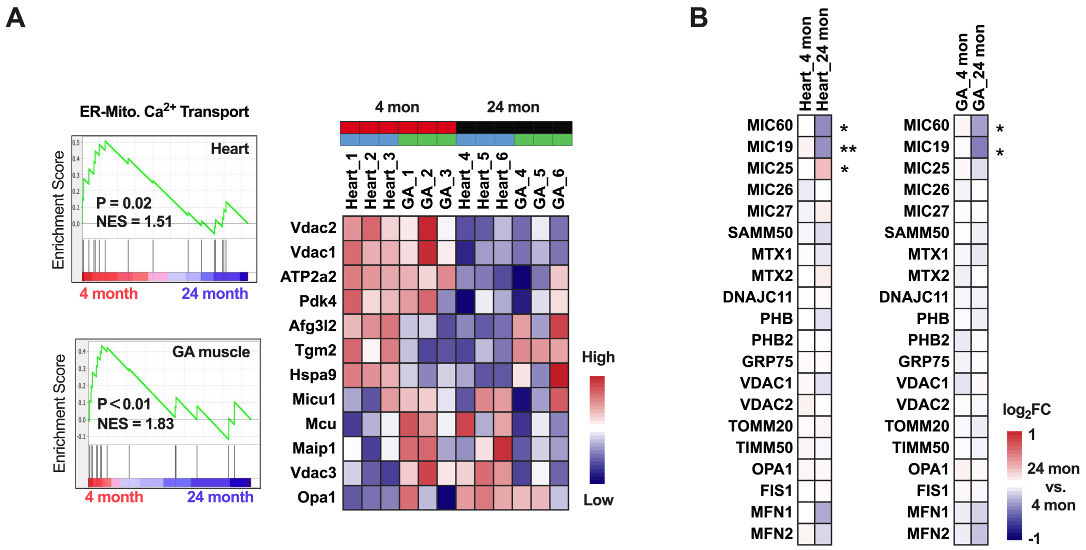

Supplementary Figure 4. (A) GSEA results showing the ER-mitochondria Ca^{2+} signaling pathway were consistently down-regulated in the aged heart and GA muscle. NES, normalized enrichment score. **(B)** Heatmap showing relative mRNA levels of genes involved in mitochondrial membrane organization in the aged heart and GA muscle. n = 3 independent experiments, 24-moth-old vs. 4-month-old. *P < 0.05; **P < 0.01.

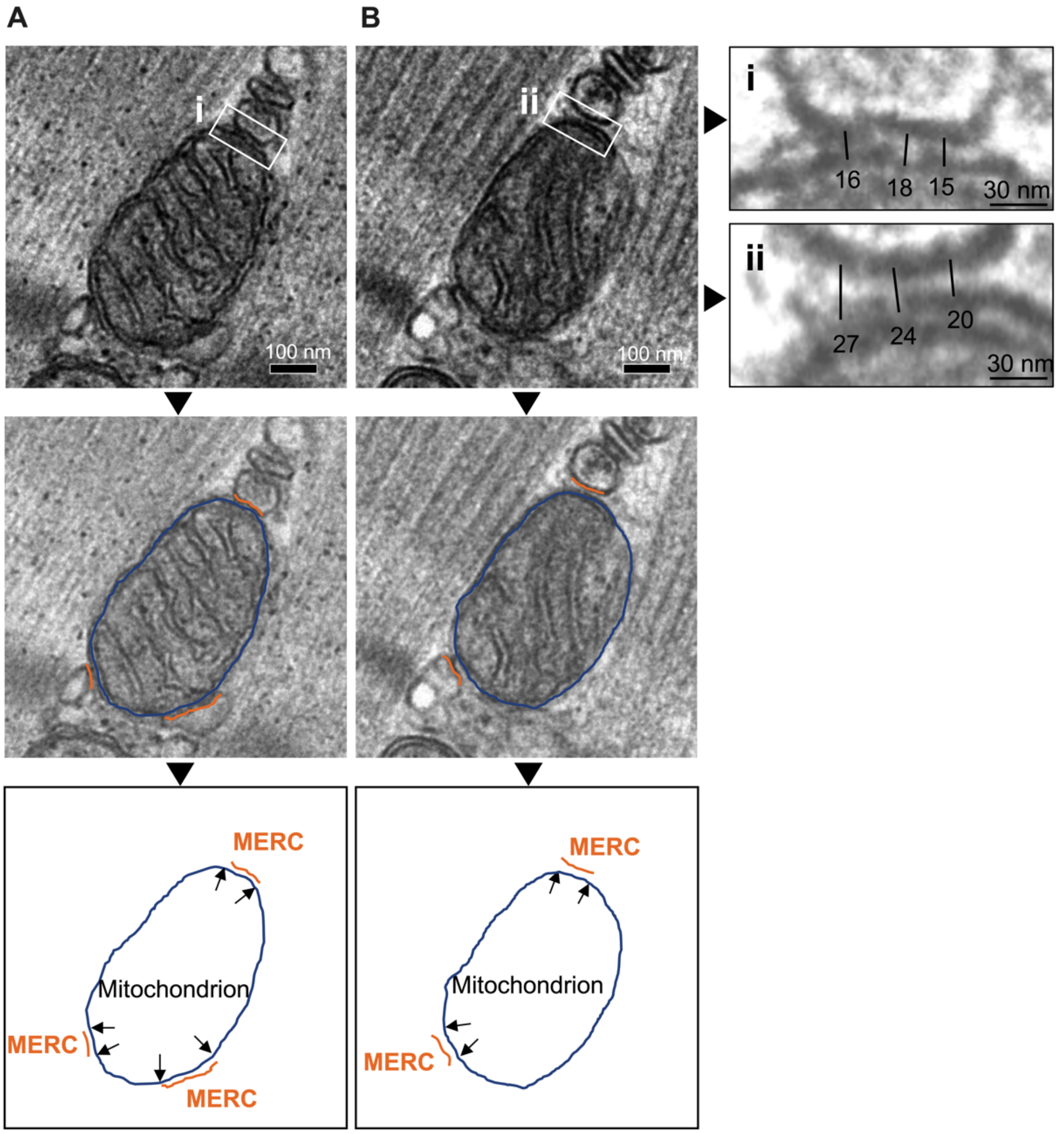

Supplementary Figure 5. Schematic defining the ultrastructural parameters of MERCs used in this study. The top left panels showed the original TEM images obtained from the GA muscles in the 4-month-old group **(A)** and the 18-month-old group **(B)**, which had been adopted in Figure 2C. Arrows in the bottom panels indicated the two extremities of the MERCs, which were defined as the structures where the cytosolic side of the OMM (indicated by the bule outlines) interfaces the ER membrane (indicated by the orange outlines) with a gap of 10-30 nm between these two membranes. The MERC length was defined by the distance between each pair of arrows

along the interface. The MERC coverage was calculated as the percentage of mitochondrial perimeter covered by all surrounding MERCs. The small white boxes in the top left panels were magnified in the right panels, showing that the width of each MERC cleft is rather uniform, but could vary from MERC to MERC. The MERC width was then quantified as an average distance measured from three randomly selected positions between the two organelle membranes. The left panels clearly documented two MERCs with their average constant width of 16 nm **(i)** and 24 nm **(ii)**, respectively. OMM, outer mitochondrial membrane.
