# Supplementary material for: Ultrastructural and proteomic profiling of mitochondria-associated endoplasmic reticulum membranes reveal aging signatures in striated muscle
Source: Cell Death Dis. 2022 Apr 2;13(4):296. doi: 10.1038/s41419-022-04746-4 (PMC8976840; doi:10.1038/s41419-022-04746-4)
Supplement: Supplementary file 3 — Supplementary Table 1.Age-related echocardiographic parameters in rats. [file 41419_2022_4746_MOESM3_ESM.pdf]

Supplementary Table 1. Echocardiographic parameters				
**Parameter**	**Units**	**4 mon**	**18 mon**	**24 mon**
**LVEF**	%	77±2.4	70.6±4.9	61.8±6.8*
**LVFS**	%	30±3.6	29±4.3	26±4.1
**CO**	mL/min	108.2±13.4	100.2±8.4	94.6±6*
**LVPWd**	mm	1.8±0.1	2.3±0.1**	2.5±0.2**
**E**	mm/s	923.3±39.5	865.3±40.1	794.8±54.6
**A**	mm/s	734.6±92.8	899.3±113.1	862±48.9
**E/A**		1.3±0.3	1.1±0.2	0.8±0.1**
**IVRT**	ms	18.8±1.8	23.1±2.7*	32±3.4**
**DT**	ms	34±3.3	42±2.8*	44.6±5.9**‡
**E'**	mm/s	64.1±6	52.9±5.9	46.9±3.7
**E/E'**		14.5±1.5	16.5±1.9*	17±1.8**
**LV mass**	mg	1312.1±190.4	1577.6±136.4	1675.6±275.1
**Global longitudinal strain**	%	-19.9±3	-13±2**	-11.1±2.6**
**Global radial strain**	%	43.5±4.7	42.8±4.2	43.4±5.0
**Global circumferential strain**	%	-16±2.5	-16±4.7	-18.4±4.7

Values are mean ± SD. *P < 0.05, **P < 0.01 compared with 4 mon. ‡P < 0.01 compared with 18 mon. LVEF = left ventricular ejection fraction; LVFS = left ventricular fractional shortening; CO = cardiac output; LVPWd = left ventricular posterior wall at end diastole; E = early diastolic transmitral flow velocity; A = late diastolic transmitral flow velocity; IVRT = isovolumic relaxation times; DT = deceleration times; E' = early diastolic mitral annular velocity.
