# Supplementary material for: Ultrastructural and proteomic profiling of mitochondria-associated endoplasmic reticulum membranes reveal aging signatures in striated muscle
Source: Cell Death Dis. 2022 Apr 2;13(4):296. doi: 10.1038/s41419-022-04746-4 (PMC8976840; doi:10.1038/s41419-022-04746-4)
Supplement: Supplementary file 4 — Supplementary Table 2. Ultrastructural analysis of mitochondria and MERCs. [file 41419_2022_4746_MOESM4_ESM.pdf]

Supplementary Table 2. Ultrastructural analysis of mitochondria and MERCs.

		Heart									GA muscle								
		4 months			18 months			24 months			4 months			18 months			24 months		
**Parameter**	**Units**	**Mean**	**SD**	**N**	**Mean**	**SD**	**N**	**Mean**	**SD**	**N**	**Mean**	**SD**	**N**	**Mean**	**SD**	**N**	**Mean**	**SD**	**N**
**MAM thickness**	µm	15.46	3.71	85	20.44	4.74	77	22.61	3.37	81	14.64	3.59	70	21.43	5.39	72	23.26	4.20	85
**MAM coverage**	%	26.38	4.43	85	17.24	3.36	77	12.65	4.17	81	25.72	4.59	70	18.54	3.99	72	14.70	2.61	85
**MAM length**	µm	227.17	49.07	85	227.40	57.47	77	206.71	52.81	81	152.00	28.77	70	147.84	23.21	72	127.12	33.65	85
**cristae abundance**	µm/µm2	17.06	3.11	122	7.35	2.62	126	4.97	2.75	110	10.10	2.29	80	8.36	2.73	79	3.20	1.26	80
**Mitochondrial area**	µm2	0.46	0.06	122	0.52	0.09	126	0.66	0.12	110	0.06	0.01	80	0.06	0.02	79	0.08	0.02	80
**Mitochondrial circularity**		0.73	0.06	122	0.76	0.06	126	0.80	0.07	110	0.81	0.06	80	0.77	0.08	79	0.67	0.08	80
**Mitochondrial aspect ratio**		1.54	0.24	122	1.52	0.21	126	1.49	0.17	110	1.34	0.18	80	1.49	0.22	79	1.88	0.38	80
