# Supplementary material for: Ultrastructural and proteomic profiling of mitochondria-associated endoplasmic reticulum membranes reveal aging signatures in striated muscle
Source: Cell Death Dis. 2022 Apr 2;13(4):296. doi: 10.1038/s41419-022-04746-4 (PMC8976840; doi:10.1038/s41419-022-04746-4)
Supplement: Supplementary file 5 — Supplementary Table 3. Total MAM proteome. [file 41419_2022_4746_MOESM5_ESM.pdf]

Supplementary Table 3a. Total MAM proteome in heart

Fasta headers	Protein names	Gene names	Unique pepti	Unique sequ	Mol. weight [	Sequence le	Q-value	Score	Heart_4 mon_	Heart_4 mon_	Heart_4 mon_	Heart_24 mon	Heart_24 mon	Heart_24 mon
tr|Q5RJR9|Q5RJR9_RAT S	Myosin-binding protein C, c	Mybpc3	13	37.6	46.561	417	0	234.39	49.65461255	52.59440003	51.63894088	28.60134846	31.44486275	31.17721018
sp|P04785|PDIA1_RAT Pr	Aconitate hydratase, mitoc	Aco2	20	43.6	56.951	509	0	149.65	126.2018188	126.8296501	124.0297838	91.85539903	95.6750198	95.69456169
tr|D4A4D5|D4A4D5_RAT S	NADH-ubiquinone oxidored	Ndufs1	6	70.4	11.706	115	0	83.036	25.81888345	25.99280124	26.67959594	18.08635107	18.7123984	19.32882324
tr|F1M1A6|F1M1A6_RAT S	Fibrillin 1 (Fibrillin 1, isoform	Fbn1	4	15.1	33.279	292	0	6.511	8.191775088	8.282024667	8.626383493	5.5925758	5.942514495	5.456088773
tr|D4A771|D4A771_RAT U	Nicotinamide nucleotide tra	Nnt	2	14.5	12.584	110	0	3.9609	11.54447813	12.03849574	12.33125763	8.304877404	7.95558233	8.571140773
sp|P19945|RLA0_RAT 60	RCG55135, isoform CRA_	Tln1	9	43.5	34.215	317	0	80.509	75.93718891	76.03786157	73.88807903	55.06202297	54.19294797	54.82778953
tr|F1LWG8|F1LWG8_RAT	Sarcoplasmic/endoplasmic	Atp2a2	25	37.1	98.363	904	0	323.31	383.2224502	373.0026431	366.3831018	299.7160549	295.2154953	281.7713127
sp|P62912|RL32_RAT 60S	Talin 2	Tln2	6	39.3	15.86	135	0	20.726	37.7217966	36.41512977	36.72887726	30.35703097	31.29897878	31.50519144
sp|P10860|DHE3_RAT Glu	Dynamin-like 120 kDa prot	Opa1	27	52	61.415	558	0	323.31	250.8726137	256.5273074	260.351562	320.6106299	330.5809077	315.8577525
sp|P24049|RL17_RAT 60S	Filamin A (Filamin alpha) (F	Flna	4	27.2	21.397	184	0	19.401	25.16181904	23.92907271	26.22047844	14.3510226	15.6213593	15.62504745
sp|P29147|BDH_RAT D-be	Very long-chain specific ac	Acadvl	12	39.7	38.201	343	0	323.31	330.9244292	301.9683364	327.4968434	187.5997759	202.0198486	181.0312804
tr|Q6P3V9|Q6P3V9_RAT (	Clathrin heavy chain 1	Cltc	14	34.7	47.3	421	0	203.46	95.68101751	91.14840678	89.00702279	60.49617501	64.82154316	67.6852706
tr|A0A0G2K757|A0A0G2K	Trifunctional enzyme subu	Hadha	10	22.2	69.122	630	0	160.44	43.9850398	45.17896061	45.59607496	36.01644857	38.09206874	37.34007546
tr|D3ZRM9|D3ZRM9_RAT (	MICOS complex subunit M	Immt	5	24.2	24.202	211	0	14.194	28.14771428	26.47127806	26.47019229	15.83846981	17.56560654	18.4403482
tr|F1LX28|F1LX28_RAT A	Myomesin 2	Myom2	11	17	67.13	594	0	18.726	45.48815438	45.33163562	48.05028577	32.34101731	31.38271487	32.88846136
tr|F1LTF8|F1LTF8_RAT La	Long-chain-fatty-acid--CoA	Acsl1	9	7.8	196.95	1773	0	109.64	17.10640077	18.131703	18.79659542	11.34531151	12.68994211	12.32727037
sp|P29117|PPIF_RAT Pep	Myosin-7 (Myosin heavy ch	Myh7	5	35	21.81	206	0	67.43	30.38883021	31.13095736	27.19682297	43.28658009	42.86764237	46.36186966
sp|Q497B0|NIT2_RAT Om	Transitional endoplasmic re	Vcp	6	23.2	30.701	276	0	22.32	10.30251476	11.12434818	9.895369632	15.31067996	14.48699701	14.93520895
tr|F1LQJ7|F1LQJ7_RAT P	ATP synthase subunit alph	Atp5f1a	2	4.7	71.206	641	0	2.7495	5.466632349	5.755514874	6.153849858	8.623895116	7.897359583	8.354026422
tr|Q32Q54|Q32Q54_RAT	Succinate dehydrogenase	Sdha	4	16.7	34.546	299	0	27.861	32.47405282	31.15521414	30.04628318	19.73395819	19.19518927	18.72778091
tr|G3V886|G3V886_RAT A	IQ motif containing GTPas	Iqgap1	9	18.7	79.795	715	0	40.477	18.29414038	18.49888402	18.30763789	19.86764181	19.67732595	20.08590047
tr|B0K031|B0K031_RAT 6	Glycogen phosphorylase,	Pygm	6	23.1	30.313	260	0	20.724	50.61947655	47.6146264	47.21057712	25.60127986	31.22309296	29.669215
sp|Q5RKI9|RRFM_RAT Rib	60 kDa heat shock protein	Hspd1	3	18.3	29.152	262	0	8.4867	3.033500515	3.322274766	3.165136218	4.206563204	4.062704789	3.957743115
tr|G3V679|G3V679_RAT T	Stress-70 protein, mitocho	Hspa9	11	14.8	85.875	761	0	28.574	28.73061972	27.47484276	25.68964017	17.42531165	13.99504751	14.12167172
sp|Q5M9G9|FAKD4_RAT	Annexin A6 (Annexin VI) (A	Anxa6	6	15.6	71.18	629	0	111.71	11.95195375	11.98760407	11.16802033	15.39575135	14.62110559	14.47429007
tr|D3ZIL6|D3ZIL6_RAT En	Endoplasmic reticulum cha	Hspa5	6	24.3	31.779	296	0	62.519	47.86841804	46.15398792	49.89670248	82.89252047	83.21273517	75.92329739
sp|A0JPQ9|CHID1_RAT C	Sodium/potassium-transpo	Atp1a1	4	10.2	44.874	393	0	32.947	23.15713467	24.40136642	23.45530319	14.26638525	16.51955966	16.08750615
tr|Q6IRE3|Q6IRE3_RAT G	Electron transfer flavoprote	Etfdh	6	21.9	35.875	310	0	10.81	12.46070023	11.61376433	11.76115618	7.12863523	7.272609881	7.367003025
sp|Q9QY17|PACN2_RAT	Mitochondrial proton/calciu	Letm1	14	33.6	55.977	488	0	150.56	70.23571983	71.54797971	69.95652544	47.93078351	51.01228509	52.57965512
tr|Q6PDV8|Q6PDV8_RAT	Aspartate aminotransferas	Got2	2	20.3	14.759	128	0	11.554	2.615020658	2.627865071	2.476041146	1.916797303	1.653198923	1.799944695
sp|Q5XIE6|HIBCH_RAT 3-	Albumin	Alb	12	36.1	43.024	385	0	189.97	27.95952593	28.01657744	29.5431909	41.90113175	39.21138469	38.66534556
sp|F1LR10|LIMA1_RAT LI	Glutamate dehydrogenase	Glud1	5	12.1	83.796	755	0	10.599	1.474168656	1.299449813	1.368766979	1.85125761	1.907481865	2.044929754
tr|G3V6S5|G3V6S5_RAT (	Ankyrin 1 (Ankyrin 1, eryth	Ank1	8	11.8	100.96	935	0	44.127	16.97163877	18.14073984	16.8753169	12.24463763	13.59926367	13.47956758
sp|Q5XIK2|TMX2_RAT Th	AFG3-like matrix AAA pept	Afg3l2	7	29.5	33.865	295	0	15.875	23.32140077	24.37045092	25.11273311	18.75303298	17.87045823	18.2673753
tr|M0RAM5|M0RAM5_RAT	3-ketoacyl-CoA thiolase, m	Acaa2	11	60.5	22.155	200	0	121.1	76.96983261	69.98317978	71.73645649	98.66545059	94.79186577	104.4766575
sp|P12001|RL18_RAT 60S	ATP synthase subunit beta	Atp5f1b	4	23.4	21.658	188	0	25.857	30.3593261	31.26555869	32.08430423	21.26697936	24.05122805	23.2717841
tr|D3ZBM3|D3ZBM3_RAT	NADH dehydrogenase [ub	Ndufs2	11	32.5	47.551	422	0	103.21	22.98011003	20.60636988	21.78321502	29.13564887	27.65973	27.97130892
tr|F1LQS3|F1LQS3_RAT 6	Pyruvate dehydrogenase E	Pdha1	10	28.9	33.545	298	0	100.93	35.93400727	35.40205261	38.43865809	20.32945792	24.59093329	24.62579747
sp|Q5U1X1|ORN_RAT Olig	Methylcrotonoyl-CoA carbo	Mccc1	2	11.8	26.751	237	0	25.16	1.670091867	1.9524803	1.97012192	3.161834453	2.740328823	3.037804459
sp|Q9QX69|LANC1_RAT C	Carnitine O-palmitoyltransf	Cpt2	4	14	45.239	399	0	8.1698	16.21649315	15.07725159	14.86085376	19.27865264	21.12373599	20.74802225
tr|B1WC34|B1WC34_RAT	Isocitrate dehydrogenase	Idh2	8	14.1	59.218	525	0	34.931	13.96301739	14.1787996	14.79750915	11.14912647	11.49735708	11.3171497
tr|A0A0A0MXZ3|A0A0A0M	Methylmalonate-semialdeh	Aldh6a1	5	14	57.74	527	0	95.543	2.645362043	2.522799933	2.026032696	4.69889217	3.990613253	4.165515903
tr|A0A140TAF1|A0A140TA	Unconventional myosin-Ic	Myo1c	3	10.8	31.188	277	0	5.3421	5.098628435	5.515800842	4.768173534	7.858252608	7.035793764	7.175039036
tr|A1L1J4|A1L1J4_RAT Pr	Carnitine O-palmitoyltransf	Cpt1b	9	20.1	61.18	538	0	67.084	15.88158167	17.45441574	16.56801704	22.43540885	20.52580801	21.96755817
tr|F1M9A7|F1M9A7_RAT A	Endoplasmin (94 kDa gluc	Hsp90b1	2	3.6	78.239	699	0	2.2734	3.306254011	3.875567094	3.644042373	2.301398111	1.941237974	2.386512734
sp|P62836|RAP1A_RAT R	Sarcalumenin	Srl	4	29.3	20.987	184	0	239.96	88.24917249	89.17932723	95.84928716	69.53718036	73.157863	67.92650877
tr|G3V9N9|G3V9N9_RAT	Acetyl-CoA acetyltransfera	Acat1	2	4.6	73.124	655	0.0030554	1.5427	1.366319189	1.528129389	1.460642832	1.956641964	1.8137367	1.820834894
sp|P15178|SYDC_RAT As	Elongation factor Tu, mitoc	Tufm	7	16.2	57.126	501	0	46.094	11.36585868	11.17952046	11.94386087	9.331810609	8.987891261	9.070555108
tr|D4A533|D4A533_RAT T	NADH dehydrogenase [ub	Ndufa9	6	14.1	69.564	608	0	7.851	5.028057804	5.098679402	5.472240967	3.706942404	3.95430581	4.218280336
sp|Q5I0P2|GCSH_RAT Gl	ATP-binding cassette, sub	Abca8a	3	24.1	18.485	170	0	86.438	14.74288268	13.34360547	8.051046956	30.81537478	26.10079959	29.69539191
tr|F1LWG4|F1LWG4_RAT	Actinin alpha 2 (RCG3055	Actn2	9	30.2	37.781	328	0	13.571	23.40433123	25.81919882	24.05524466	16.35020026	16.80216895	15.89759526
sp|P08461|ODP2_RAT Dih	Sodium/potassium-transpo	Atp1a2	22	42.4	67.165	632	0	323.31	268.3916732	295.4570564	274.5281893	407.9129734	373.1554738	368.2115676
tr|A0A0G2JXD0|A0A0G2J	Long-chain specific acyl-Co	Acadl	5	25.5	21.424	188	0	117.54	27.90171384	25.86771237	29.33378725	16.77512317	20.44795962	19.2805756
sp|P16303|CES1D_RAT C	Methylcrotonoyl-CoA carbo	Mccc2	8	16.8	62.146	565	0	44.267	21.7995471	14.76334036	14.26614738	38.36285639	46.69398898	50.11697272
sp|Q5BJQ0|COQ8A_RAT	Trifunctional enzyme subu	Hadhb	17	32.4	72.225	649	0	323.31	85.43432132	89.94983662	84.14362295	105.3105678	99.65902575	107.0378883
sp|P06761|BIP_RAT Endo	Apoptosis-inducing factor 1	Aifm1	31	48.8	72.346	654	0	323.31	330.1150599	320.5176364	324.1882657	239.5757908	258.9734707	262.1642956
sp|P35565|CALX_RAT Ca	Succinate--CoA ligase [GD	Suclg2	20	31	67.254	591	0	108.79	70.84972419	65.3363423	69.52724795	53.87275966	54.53443419	57.26583484
sp|B5DEH2|ERLN2_RAT E	NADH dehydrogenase [ub	Ndufv1	8	27.4	37.71	339	0	83.095	25.70086703	24.17544546	25.14047909	20.77391253	19.28612143	21.26232149
tr|F1M7K3|F1M7K3_RAT M	Tripartite motif-containing p	Trim72	4	27	16.731	148	0	31.739	4.142137223	2.465106854	2.754495654	9.074426251	8.562668951	6.882473599
sp|Q9Z1E1|FLOT1_RAT F	Dihydrolipoyllysine-residue	Dlat	15	48.8	47.499	428	0	135.8	64.84523997	57.45051166	67.2028674	43.95065778	39.66342872	37.34366837
tr|G3V7K1|G3V7K1_RAT	Desmin	Des	38	32.7	164.71	1464	0	323.31	125.3286567	111.1531135	123.8884363	164.8258176	169.1468931	169.5852992
tr|Q642E2|Q642E2_RAT 6	Peroxisomal multifunctiona	Hsd17b4	3	23.4	15.733	137	0	4.3647	3.478573937	3.909146083	3.833552679	2.731572878	2.494550328	2.599058958
sp|P17209|MYL4_RAT My	Cytochrome b-c1 complex	Uqcrc2	7	37.3	21.282	193	0	36.172	25.98354826	16.2068564	22.27845466	46.37258816	43.70369486	39.45886515
tr|A0A0G2K6E2|A0A0G2K	NADH dehydrogenase [ub	Ndufa10	5	11.6	56.367	524	0	42.751	7.748814798	8.823759354	7.828555575	10.36395227	10.53570047	11.36385752
sp|P61354|RL27_RAT 60S	NLR family member X1	Nlrx1	3	28.7	15.798	136	0	16.65	49.77023675	47.77633824	54.27742691	27.41251919	27.4660903	26.31497792

sp|Q91ZW1|TFAM_RAT Tr	Fructose-bisphosphate ald	Aldoa	8	29.1	28.186	244	0	68.672	18.67450412	19.18235438	20.14567846	29.72724226	26.87143325	26.83954262
tr|G3V8C4|G3V8C4_RAT (	Medium-chain specific acyl	Acadm	7	32.8	28.669	253	0	142.46	18.24111273	19.94097319	18.85575195	12.73206197	15.02670225	12.93909143
sp|P05065|ALDOA_RAT F	Heat shock protein HSP 90	Hsp90ab1	20	68.7	39.351	364	0	323.31	228.2541283	227.0909951	258.1318833	145.1118252	178.9466319	151.2152694
sp|O35077|GPDA_RAT G	EH domain-containing prot	Ehd2	4	16	37.452	349	0	15.359	9.708046901	9.526730264	9.037861672	12.62268447	12.46097625	11.48652969
tr|G3V6H0|G3V6H0_RAT	Sorting and assembly mac	Samm50	4	31.8	22.177	201	0	323.31	111.8165737	112.2185092	118.4858221	94.34243507	100.5225541	91.88607828
tr|A0A0G2K2T1|A0A0G2K	Dihydrolipoyl dehydrogena	Dld	4	7.7	76.956	685	0	5.4546	4.112234414	4.039514369	3.748848901	4.79524854	4.680258421	5.081964304
tr|A0A0G2K513|A0A0G2K	Malonyl-CoA decarboxylas	Mlycd	8	19.6	50.989	453	0	120.33	33.0808805	37.4462806	35.35152473	46.32484401	44.51227143	42.48152807
sp|A0JPM9|EIF3J_RAT Eu	Succinate--CoA ligase [AD	Sucla2	5	20.1	29.187	259	0	12.727	21.53281404	20.86938944	22.06433943	18.91840135	18.933514	17.68224442
tr|D4A9G1|D4A9G1_RAT	Malate dehydrogenase, m	Mdh2	8	26.5	46.394	407	0	198.52	26.80288525	25.70980551	26.33460343	19.3967109	21.55026668	21.22587913
sp|Q9Z1N4|BPNT1_RAT 3	Short-chain specific acyl-C	Acads	8	28.9	33.174	308	0	45.312	21.97856526	21.59899524	19.51537346	30.53976084	27.11937057	30.64545967
sp|P40307|PSB2_RAT Pro	Hydroxysteroid dehydroge	Hsdl2	2	13.4	22.912	201	0	23.119	7.106901146	6.350043722	7.409224759	4.269715689	5.163114709	4.712664598
tr|A0A0G2JZH8|A0A0G2J	EH domain-containing prot	Ehd1	11	32.9	54.169	502	0	149.69	43.39495769	48.29001117	48.93344568	67.26716317	59.26879397	65.78102889
tr|F1LMV6|F1LMV6_RAT D	Solute carrier family 25 me	Slc25a12	16	6.3	332.39	2877	0	40.941	29.06752472	29.33833398	31.10324812	35.8107147	35.07887803	38.14129414
tr|G3V836|G3V836_RAT (	Caveolae-associated prote	Cavin1	9	24.6	51.42	447	0	126.99	16.97203747	14.80234145	15.69061573	22.2748149	22.03698268	25.24172471
sp|Q63704|CPT1B_RAT C	Citrate synthase, mitochon	Cs	25	38.5	88.216	772	0	323.31	278.2356783	284.3988198	293.2279356	243.1913248	232.5377267	217.2888637
sp|Q6DGG0|PPID_RAT Pe	Succinyl-CoA:3-ketoacid co	Oxct1	3	7.8	40.765	370	0	3.5929	11.48865955	10.09509984	9.865006102	6.305265708	7.586620202	7.232525578
sp|P45479|PPT1_RAT Pa	2-oxoisovalerate dehydrog	Bckdha	3	15	34.455	306	0	8.772	3.690445313	4.03047753	4.779167226	6.690691188	5.845236714	6.514970348
tr|D3ZJ32|D3ZJ32_RAT Ex	Electron transfer flavoprote	Etfa	7	11.5	93.95	845	0	19.123	13.0280562	12.15216966	13.08406376	14.33235898	15.00576823	15.03478385
tr|D4A4L5|D4A4L5_RAT In	Cytochrome b-c1 complex	Uqcrfs1	5	45.5	16.68	154	0	47.949	13.53201823	13.81542356	14.20751436	21.34467211	19.02444616	20.16853737
sp|Q6PDV7|RL10_RAT 60	Cytochrome b-c1 complex	Uqcrc1	4	24.3	24.604	214	0	135.72	23.09892387	18.84561324	21.0136566	11.10311847	13.74711019	12.59211908
sp|P34058|HS90B_RAT H	Voltage-dependent anion-	Vdac1	20	33.1	83.28	724	0	323.31	214.4190949	198.6962973	198.1220314	140.1464338	161.5517685	162.7433742
tr|F1LMZ4|F1LMZ4_RAT R	ATP-dependent 6-phospho	Pfkm	5	8.7	85.913	779	0	13.767	7.364464015	7.339339726	7.915458091	11.29322699	9.943660177	11.08207081
sp|P11507|AT2A2_RAT S	Amine oxidase [flavin-cont	Maoa	42	43.5	114.77	1043	0	323.31	1959.830159	1716.09563	1904.840331	1268.041149	1459.8209	1393.484046
sp|B4F7E8|NIBL1_RAT Ni	Cytochrome c oxidase sub	Cox4i1	6	9.6	84.73	747	0	16.593	9.42616308	9.880118211	9.505878837	8.816607856	8.770700789	8.436150054
sp|P19836|PCY1A_RAT C	Succinate dehydrogenase	Sdhb	5	21	41.68	367	0	39.593	5.589034517	5.781674143	5.191692423	4.312598614	4.203289827	4.617709149
sp|P18418|CALR_RAT Ca	Heat shock cognate 71 kD	Hspa8	17	48.3	47.995	416	0	213.77	123.2075508	125.8879164	132.5525125	79.76310863	97.68991936	91.85528192
sp|P11598|PDIA3_RAT Pr	Voltage-dependent anion-	Vdac2	21	45.7	56.623	505	0	270.61	123.2673564	128.2945692	115.9886835	95.76607873	96.70209522	104.9796648
sp|Q5RJR8|LRC59_RAT L	Atypical kinase COQ8A, m	Coq8a	8	31.3	34.869	307	0	108.45	21.07031724	20.05750085	20.90633723	16.17311288	15.92686518	17.77412024
tr|G3V7Q7|G3V7Q7_RAT	Fumarate hydratase, mitoc	Fh	35	28.2	188.83	1657	0	323.31	103.4677092	109.2696461	105.6860238	93.13581025	96.83947474	94.88359083
tr|G3V6I9|G3V6I9_RAT 60	ATP synthase subunit d, m	Atp5pd	3	12.4	17.258	145	0	3.1862	17.89902458	15.14764381	16.03665527	9.800571328	11.85454382	11.68465296
sp|Q68FX9|SIR5_RAT NA	Electron transfer flavoprote	Etfb	7	29	34.098	310	0	13.502	16.66344048	17.57855336	17.03708122	21.29996622	19.60471107	20.5966068
tr|Q9JKB7|Q9JKB7_RAT (	NADH:ubiquinone oxidored	Ndufb10	10	29.7	50.9	454	0	77.127	27.67445248	30.88315774	28.42654592	37.40927556	42.9271735	39.21711371
sp|Q5FVQ4|MLEC_RAT M	3-hydroxyacyl-CoA dehydr	Hsd17b10	6	27.1	32.418	291	0	33.138	12.54283328	13.53718406	13.33116007	10.40041143	11.43913433	11.22168098
tr|D3ZB55|D3ZB55_RAT C	L-lactate dehydrogenase E	Ldhb	3	58.1	8.3585	74	0	28.411	10.77418175	12.3138815	11.55646411	7.906864831	8.471082608	9.368253272
tr|Q6P2A5|Q6P2A5_RAT	Pyruvate dehydrogenase E	Pdhb	10	48	25.494	227	0	113.3	26.11193099	26.59113508	26.1037359	36.1088986	33.12416378	34.67208398
sp|Q6AXR4|HEXB_RAT B	Prohibitin	Phb	6	13.6	61.527	537	0	20.615	12.31078747	12.90460537	12.10667221	9.316185252	9.731049022	10.63911647
tr|A0A0G2JXA8|A0A0G2J	Sodium/calcium exchange	Slc8a1	15	49.3	41.658	379	0	155.14	54.79789585	47.46813449	56.98396913	34.5120739	31.82232932	30.04801025
sp|Q4V8H8|EHD2_RAT EI	Prohibitin-2 (B-cell recepto	Phb2	20	52.1	61.237	543	0	323.31	256.7016681	293.3643149	288.9351607	215.8729918	202.2684401	192.6261106
tr|F1M853|F1M853_RAT R	Delta(3,5)-Delta(2,4)-dieno	Ech1	17	16.4	157.5	1453	0	247.6	46.98329487	43.26315085	47.14775603	34.74428407	39.37296917	37.03827111
sp|Q5I0K3|CLYBL_RAT C	Isocitrate dehydrogenase	Idh3B	13	45.6	37.321	338	0	96.505	34.99984349	31.73262056	33.18367341	52.01073792	45.1056201	46.89772636
tr|A0A0G2QC06|A0A0G2C	Dihydrolipoyllysine-residue	Dlst	8	9.6	107.41	979	0	210.67	98.40017968	94.56813671	91.20576115	72.7403786	70.43644022	80.93797165
tr|A0A0H2UI06|A0A0H2UI	Phosphate carrier protein,	Slc25a3	4	17.9	38.124	329	0	55.804	19.04370414	18.83419829	20.46606605	15.58976621	15.49902611	16.20812524
tr|A0A0H2UHL9|A0A0H2U	Myosin regulatory light cha	Myl2	3	6.4	77.343	706	0	2.6806	4.082331604	4.293306843	4.777439646	2.921941815	3.411460467	3.045657532
tr|F1LPD0|F1LPD0_RAT C	Creatine kinase M-type (EC	Ckm	7	8.2	134.49	1312	0	36.397	20.75255005	21.31790201	18.43589763	14.73123963	13.46580928	16.04439125
tr|D3ZZC1|D3ZZC1_RAT R	Perilipin 4	Plin4	7	19.7	46.353	417	0	98.52	20.52768092	21.34406127	21.55287101	15.71693925	17.5786903	17.61449243
tr|Q6P7A7|Q6P7A7_RAT	L-lactate dehydrogenase A	Ldha	17	33.2	68.4	606	0	303.6	68.95986661	78.44927058	77.50029205	52.32758544	59.0182399	58.84158202
tr|A0A0G2K261|A0A0G2K	Myosin light chain 3 (Myos	Myl3	25	30.9	112.68	1011	0	199.18	69.45824677	69.56463147	73.484977	86.03061306	79.5558233	85.64468228
tr|A0A0G2K8S1|A0A0G2K	Junctophilin-2 (JP-2) (Junc	Jph2	11	14.1	119.98	1088	0	115.92	47.42186942	46.34281028	49.48784185	36.77254225	41.65281494	38.29886886
tr|B2RYD7|B2RYD7_RAT	Cadherin 13 (T-cadherin)	Cdh13	2	2.2	93.435	824	0	2.5163	5.121753275	5.774064174	5.563331556	4.092975537	3.891765421	4.086163944
sp|Q64361|LXN_RAT Late	Acyl-coenzyme A thioester	Acot2	2	12.1	25.58	223	0	3.5091	4.075553633	4.221249947	3.718328319	3.072249071	3.324060927	2.852205052
sp|Q64057|AL7A1_RAT A	Glyceraldehyde-3-phospha	Gapdh	17	43.8	58.748	539	0	232.83	43.97307868	43.24935778	41.89800644	59.75831091	65.59544776	58.25645115
tr|G3V9X8|G3V9X8_RAT	D-beta-hydroxybutyrate de	Bdh1	3	10.1	37.94	335	0	15.976	11.0684254	10.85134054	11.73236318	9.143872284	8.994433143	9.271244732
sp|P02650|APOE_RAT Ap	ATP synthase subunit O, m	Atp5po	6	23.1	35.753	312	0	19.593	14.66712889	16.61541663	13.99235211	20.50046878	18.47361972	20.4010499
tr|F1LQ95|F1LQ95_RAT T	Voltage-dependent anion-	Vdac3	15	39.9	35.371	296	0	53.783	38.64958111	35.12381311	34.13227196	42.02049211	46.22231931	44.5977514
tr|Q68G41|Q68G41_RAT	MICOS complex subunit	Chchd3	10	39.8	32.264	289	0	67.666	56.02191753	57.52185512	55.99453687	77.3324975	70.06355296	73.90100297
sp|Q5BJS4|FUND1_RAT F	NADH dehydrogenase [ub	Ndufa12	2	26.5	17.158	155	0	19.162	3.280338243	3.501204168	2.866264854	5.070428443	4.508991959	4.269043006
tr|D3ZIN7|D3ZIN7_RAT Mi	Ras-related protein Rab-11	Rab11b	6	40.7	20.321	177	0	57.551	30.81823456	31.34689024	31.35819707	26.73845864	24.47383361	26.11480157
sp|P0CD94|UQCC3_RAT U	Guanine nucleotide-bindin	Gnai2	2	20.2	9.6781	89	0.00046468	1.9821	5.622525664	6.955511901	5.846026488	3.369564898	3.192372849	3.85010983
tr|D3ZT90|D3ZT90_RAT G	NADH dehydrogenase [ub	Ndufv2	9	31.8	49.713	447	0	114.24	34.41016008	33.15806292	33.24858855	44.63643735	40.43667914	42.68067788
sp|P17764|THIL_RAT Ace	ATP synthase-coupling fac	Atp5pf	24	66.7	44.695	424	0	323.31	259.8075733	284.9885925	281.8049663	468.2398737	399.3360843	411.4342615
sp|P19944|RLA1_RAT 60S	[Pyruvate dehydrogenase	Pdk2	2	28.9	11.498	114	0	47.252	10.64061586	10.05276939	9.088642058	6.612130363	6.714587372	7.998328443
sp|P61314|RL15_RAT 60S	Hydroxyacyl-coenzyme A d	Hadh	7	28.4	24.146	204	0	20.749	16.82332083	16.69960191	18.48720152	9.657772924	12.98694354	11.77652877
tr|D4A9N5|D4A9N5_RAT T	Cytochrome c-1 (Cytochron	Cyc1	3	4.5	71.919	643	0	7.5407	8.05103253	7.739338735	7.29405275	6.165505567	6.596833502	6.31941345
tr|D3ZI60|D3ZI60_RAT 60S	Isocitrate dehydrogenase	Idh3g	4	27	16.549	148	0	11.916	32.21728736	30.91978072	31.49640348	21.37071437	25.27783087	22.96330721
tr|Q4V7D1|Q4V7D1_RAT	Platelet glycoprotein 4 (Ad	Cd36	3	11.8	32.207	287	0	15.082	11.08636708	11.10912825	10.77381796	7.152073266	8.053056367	8.498769323
sp|Q6AYT0|QOR_RAT Qu	Mitochondrial carrier 2	Mtch2	4	17.6	34.975	329	0	12.866	4.516520403	4.209739869	3.848106233	7.892975624	6.685803093	6.512917257
tr|D4A071|D4A071_RAT S	Malic enzyme	Me3	2	11	35.478	310	0	20.869	4.091501799	4.956943725	4.11792284	7.649046435	7.131959424	6.139254733

sp|A0A0G2K309|ORNT1_	Histidine rich calcium bindi	Hrc	4	14.3	32.797	301	0	7.4991	19.93321306	21.66510685	20.21635219	18.37238192	16.85057887	16.75065448
tr|B2RYC9|B2RYC9_RAT	Glioblastoma amplified sec	Nipsnap2	3	7.2	57.491	515	0	7.4452	2.531651625	2.56189615	2.706280463	3.2187802	2.945809327	3.031439878
tr|D3ZUB0|D3ZUB0_RAT	Superoxide dismutase [Mn	Sod2	3	9.2	38.09	325	0	49.622	7.303063579	7.34314471	6.933878467	4.799154879	5.715314943	4.735607888
tr|A0A0G2KAQ5|A0A0G2	ADP/ATP translocase 1 (A	Slc25a4	2	8	29.789	264	0.00047281	2.1255	1.373974308	1.078903391	1.449544438	2.011938367	2.229669538	2.595414721
tr|F1LQ55|F1LQ55_RAT N	NADH dehydrogenase [ub	Ndufb8	11	27.6	58.787	547	0	145.51	47.84449579	62.61102206	50.87723509	97.31125295	90.14058788	76.63674644
tr|D4ACG2|D4ACG2_RAT	Cytochrome c oxidase sub	Cox6b1	2	4	72.269	672	0	6.2913	0.719142642	0.627870026	0.823270463	0.956662501	1.088438277	1.081465573
sp|Q2PS20|JPH2_RAT Ju	NADH dehydrogenase (Ub	Ndufb7	13	23.8	74.258	692	0	323.31	79.47369456	85.2649493	85.3215185	55.34848785	67.83931318	58.15379661
tr|G3V6P2|G3V6P2_RAT	Ubiquinone biosynthesis p	Coq9	15	48.5	48.899	454	0	323.31	198.8138152	210.7580986	221.0464963	313.600921	269.6890729	284.039978
tr|F1LU48|F1LU48_RAT E	NADH-ubiquinone oxidore	Mtnd5	5	16.6	35.011	313	0	7.8452	11.64056583	11.62803303	12.93643419	9.058800894	9.833102377	9.481686538
sp|P62329|TYB4_RAT Th	DnaJ (Hsp40) homolog, su	Dnajc11	3	61.4	5.0526	44	0	15.365	14.42272326	17.56618716	19.1510111	8.78752733	10.92494243	8.066593712
tr|A0A0G2K5L2|A0A0G2K	NDUFA4, mitochondrial co	Ndufa4	10	40.2	34.662	323	0	107.49	46.66831861	49.31735702	46.06828019	41.06517513	42.2468178	40.67583472
tr|D4A2F1|D4A2F1_RAT A	Hemoglobin subunit alpha	Hba1	4	2.6	206.59	1940	0	13.226	2.462915032	2.463632422	2.499494355	1.711671085	1.386486407	1.703911373
tr|A0A0G2K7X7|A0A0G2K	Hexokinase-1 (EC 2.7.1.1)	Hk1	4	6.5	93.664	846	0	21.176	9.165809282	9.469179871	8.384522274	12.00765305	11.51174922	13.44055885
sp|B5DFN3|UQCC2_RAT	Guanine nucleotide-bindin	Gnb1	5	46.3	16.33	136	0	107.13	37.56550458	40.89359667	34.86152018	24.08475212	25.26016779	24.2793384
tr|D3ZPF2|D3ZPF2_RAT M	Cytochrome b-c1 complex	Uqcrh	7	28.4	41.832	380	0	28.285	7.288311526	6.133635221	6.548575745	9.074860289	9.072935724	8.752839308
tr|M0R6J0|M0R6J0_RAT M	Ryanodine receptor 2 (RY	Ryr2	6	20.8	38.374	336	0	36.477	9.139494809	10.04135444	10.54242692	13.04760738	12.15089106	11.67900696
tr|A0A0H2UHR5|A0A0H2U	Guanine nucleotide-bindin	Gnb2	4	18.8	46.213	409	0	28.095	7.229702019	6.98167117	7.345880154	10.59876666	9.325452357	10.52978939
sp|P07483|FABPH_RAT F	Propionyl-CoA carboxylase	Pcca	6	46.6	14.775	133	0	56.547	79.58134467	69.0129087	71.95109524	39.78650006	56.15682085	44.47456595
tr|A0A0G2K3N1|A0A0G2K	Ras-related protein Rab-1	Rab1b	2	1.8	205.33	1850	0	2.7236	2.752852677	3.022917601	2.977301141	2.450229639	2.424159681	2.333491664
tr|D4A568|D4A568_RAT C	Propionyl coenzyme A car	Pccb	2	5.4	36.318	331	0	4.9065	7.41988389	8.647303192	7.802380118	5.979303393	6.481303871	5.43453132
sp|P35213|1433B_RAT 14	Hemoglobin subunit beta-1	Hbb	6	38.6	28.054	246	0	50.079	15.43264082	17.35596176	18.35265968	10.93471185	13.09357621	13.03609997
sp|Q8R431|MGLL_RAT M	Heat shock protein beta-1	Hspb1	8	38.3	33.499	303	0	73.589	21.30635009	20.81041218	21.08380682	17.11757892	18.40427577	16.65107957
tr|D3ZXA6|D3ZXA6_RAT	Myosin-6	Myh6	18	23.2	98.818	878	0	75.941	39.49403646	41.17183617	42.39481661	62.28441034	53.4667991	58.10246934
tr|A0A0G2K5P5|A0A0G2K	Actin, alpha cardiac muscle	Actc1	24	20	187.6	1688	0	88.568	40.38474149	35.96138535	41.92627593	46.8934334	52.29841903	53.89363322
tr|G3V7K9|G3V7K9_RAT	EH-domain-containing 4 (F	Ehd4	6	27.7	33.282	307	0	60.318	12.76770241	12.21447628	10.95285808	14.82455774	14.95735831	15.64095891
sp|O35179|SH3G2_RAT E	Isovaleryl-CoA dehydroger	Ivd	2	5.4	39.899	352	0.00045914	1.8786	1.937383116	2.217259668	2.345111512	1.345039437	1.678058074	1.219381947
sp|Q6AXT0|RM37_RAT 39	Phosphoglycerate kinase	Pgk1	11	29.3	48.365	423	0	86.131	18.20443195	19.00494697	18.60551459	25.78835011	22.87499772	24.51287748
sp|Q5U2X6|CCD47_RAT	Alpha-crystallin B chain (A	Cryab	5	14.7	55.732	483	0	37.452	16.1160197	16.02279027	15.0870097	10.48895512	10.81765557	12.84516252
sp|P04636|MDHM_RAT M	NADH dehydrogenase (Ub	Ndufb9	19	65.7	35.683	338	0	323.31	593.192007	593.1495413	503.8408949	848.3700938	709.3362322	830.1672607
tr|Q5XIJ3|Q5XIJ3_RAT Is	Mitochondrial 2-oxoglutara	Slc25a11	10	37.9	42.85	393	0	323.31	63.7288684	69.15559563	64.53820592	84.39429092	75.26434891	80.19885896
sp|Q568Z6|IST1_RAT IST	Annexin A5 (Anchorin CII)	Anxa5	4	7.9	39.941	366	0	5.726	11.62461766	11.57286075	11.96323071	7.606076703	9.261996105	8.386875875
sp|P16975|SPRC_RAT SP	Medium-chain acyl-CoA lig	Acsf2	6	24.3	34.296	301	0	20.409	8.362819161	9.542901449	9.543571495	6.126876212	6.274514991	6.346616903
sp|Q3BCU4|POPD1_RAT	Fibrinogen alpha chain [Cl	Fga	8	30.9	40.86	356	0	36.935	30.83697365	27.79255897	30.9304901	22.26222781	25.66511027	24.39328494
sp|Q5XIH4|LIAS_RAT Lip	NADH dehydrogenase [ub	Ndufa2	3	11	41.874	373	0	31.895	6.886816466	6.012351336	6.624484569	8.736310881	7.633067562	8.589105318
sp|Q6AYD9|NUD19_RAT	Dolichyl-diphosphooligosa	Rpn1	6	26.9	39.938	357	0	60.015	8.367204906	9.481070448	7.703436892	12.71036009	10.68616375	11.61074169
sp|Q6IRK9|CBPQ_RAT Ca	Annexin A2 (Annexin II) (A	Anxa2	2	6.6	52.041	472	0	14.801	3.580004268	4.625767375	3.736075279	5.629903042	5.494068504	6.621217796
sp|Q5XI73|GDIR1_RAT R	Protein NDRG2 (Antidepre	Ndrg2	4	23.5	23.407	204	0	53.071	12.40727387	11.99759216	11.15283857	8.737612994	10.16085065	9.81120761
sp|P00406|COX2_RAT Cy	OCIA domain-containing p	Ociad1	7	32.2	25.928	227	0	159.97	342.8217605	382.4104676	342.9770085	304.5599156	272.7964667	284.7328961
tr|B0BNG3|B0BNG3_RAT	NADH dehydrogenase [ub	Ndufs6	8	27.1	40.392	358	0	41.524	26.96077208	25.15998523	27.13452538	22.16717355	23.00648955	22.82421031
tr|G3V648|G3V648_RAT 1	L-2-hydroxyglutarate dehy	L2hgdh	5	16.8	43.245	376	0	40.868	21.46064859	22.46177551	18.74686205	14.39963483	13.32777558	14.68575842
sp|P62832|RL23_RAT 60	Carnitine O-acetyltransfera	Crat	3	30.7	14.865	140	0	78.991	40.21728576	32.44129894	33.32711492	18.12801869	20.43945517	20.51550971
sp|P11661|NU5M_RAT NA	NADH dehydrogenase (Ub	Ndufb11	7	15.9	68.617	609	0	323.31	51.00223252	53.20319757	51.77243571	64.83221166	59.32963347	64.49784715
sp|B2RYW9|FAHD2_RAT	Triosephosphate isomeras	Tpi1	7	35.5	34.581	313	0	145.76	26.12867656	23.96854942	24.38557892	38.9396925	32.69828728	37.25795183
tr|A0A140TAE6|A0A140T	Lon protease homolog, mi	Lonp1	8	27.3	40.334	373	0	130.24	21.11297859	21.26891283	18.86622213	32.29891565	26.83741547	31.70998724
tr|F1LPG5|F1LPG5_RAT	Complement component 1	C1qbp	9	48.1	15.064	129	0	128.73	101.713411	103.847543	103.523931	84.32918526	82.12024092	73.44419026
tr|D4A830|D4A830_RAT F	Popeye domain-containing	Popdc2	12	48	35.046	304	0	124.94	32.08292406	34.39515855	36.25457799	47.71810504	40.96591737	46.10831295
tr|D4A4A9|D4A4A9_RAT	ATP synthase subunit f, m	Atp5mf	5	19.9	33.633	292	0	102.72	10.58240506	11.13623876	11.59101571	14.36881815	13.0889969	12.92882597
sp|Q4FZU1|COQ4_RAT U	Methylmalonyl-CoA mutase	Mmut	2	10.2	30.013	265	0	3.0125	0.648691622	0.574647804	0.501453693	0.873631088	0.836837506	0.841253951
tr|A0A0G2K930|A0A0G2K	Tubulin alpha-4A chain (A	Tuba4a	12	72.3	21.203	188	0	83.417	87.24443808	83.72393052	84.10697731	81.08258325	78.48949658	78.31001544
sp|Q5PPN5|TPPP3_RAT	Guanine nucleotide-bindin	Gnb3	2	8.5	18.979	176	0	8.6121	2.714776432	2.418971415	2.22517557	3.537928123	3.401516806	2.990070098
sp|Q8CHM7|HACL1_RAT	Peroxisomal acyl-coenzym	Acox1	2	5.2	63.615	581	0	19.878	0.99971074	1.490507604	1.021785126	2.032164524	2.145998871	1.774229733
tr|A0A140TA99|A0A140T	Vinculin (Metavinculin)	Vcl	3	25.6	13.039	117	0	7.4557	15.33774924	13.86488836	15.72254979	10.86005736	11.07540571	11.18472535
tr|D4AB01|D4AB01_RAT	Testicular haploid express	Theg	4	39.9	17.38	163	0	142.51	16.05980242	16.80947084	14.36561412	27.92251349	22.23520169	26.85288771
tr|M0RDK9|M0RDK9_RAT	Enoyl-CoA hydratase, mito	Echs1	13	30.7	45.155	411	0	88.739	40.96684952	37.10668572	36.03313362	53.30851065	45.65186722	48.86458733
tr|F1M953|F1M953_RAT S	Protein disulfide-isomerase	Pdia3	32	47	73.744	679	0	323.31	352.801325	361.9016005	366.2679298	548.0594072	485.4861244	482.9536791
sp|Q505J8|SYFA_RAT Ph	Solute carrier family 25 me	Slc25a13	2	5.9	57.719	508	0	11.29	2.588586575	2.741729236	2.643982876	2.442590576	2.375815175	2.455496584
sp|B0LPN4|RYR2_RAT Ry	Dihydropyrimidinase-relate	Dpysl2	4	0.9	562.95	4953	0	323.31	982.4867211	1069.438492	986.5006102	781.0942498	899.8358272	801.116026
tr|A0A0H2UHD9|A0A0H2U	Moesin (Membrane-organiz	Msn	5	10.9	62.863	562	0	12.784	8.254371637	7.209494625	7.713383566	5.809594651	6.217208107	6.261413635
tr|G3V6T7|G3V6T7_RAT	NADH dehydrogenase [ub	Ndufa5	9	17.7	72.747	643	0	48.987	24.75314731	26.4061177	23.80867186	19.92450074	21.89698641	21.6041611
tr|D4ACY1|D4ACY1_RAT	ATP synthase subunit gan	Atp5f1c	6	53.7	15.658	136	0	76.891	18.14024058	22.00042112	21.14191634	13.36488894	15.61220067	14.16478663
sp|Q641Z6|EHD1_RAT EH	ATP synthase subunit delt	Atp5f1d	19	40.8	60.602	534	0	323.31	152.6917213	164.251674	162.7589894	132.780814	142.7046073	144.7326352
sp|P04166|CYB5B_RAT C	Tubulin beta-5 chain	Tubb5	4	47.3	16.265	146	0	167.19	20.46827401	21.98092058	19.74257642	23.53092002	23.7941321	24.8490711
tr|D4ACB8|D4ACB8_RAT	Sarcoglycan, beta	Sgcb	13	29.2	59.588	548	0	58.977	36.73061813	34.08790604	36.74615306	25.8768938	30.98169752	28.3362458
sp|Q3MIE4|VAT1_RAT Sy	NADH dehydrogenase (Ub	Ndufs3	11	42.1	43.118	404	0	110.15	50.8268027	46.27289369	46.82998598	57.63152617	67.36175582	63.62015084
tr|D3ZHF8|D3ZHF8_RAT T	Pre-B-cell leukemia transc	Pbxip1	2	3.8	73.206	659	0	2.6694	2.869234413	2.937638383	2.749731721	4.165633448	3.684649446	4.33664102
sp|Q5RKI8|ABCB8_RAT A	Acetyl-coenzyme A synthe	Acss1	15	26.2	77.77	714	0	75.443	30.34776368	32.34855244	32.28533174	36.90058337	34.3115153	37.05623566
sp|P47853|PGS1_RAT Bi	NADH-cytochrome b5 redu	Cyb5r3	4	15.4	41.706	369	0	113.56	17.29419041	21.75119463	17.46792924	24.33736207	25.76912619	28.60725379

tr|A0A0G2JUS7|A0A0G2J	Ribosome-binding protein	Rrbp1		3	19.9	19.834	176	0	7.1824	16.47046768	19.08818101	18.5374584	14.13096549	14.08597967	13.02326815
tr|M0R776|M0R776_RAT	2,4-dienoyl-CoA reductase	Decr1		5	62.1	11.413	103	0	68.586	22.09777779	25.48626267	25.44830246	30.96772201	29.02960002	30.4832655
tr|D4A6W6|D4A6W6_RAT	Ras-related protein Rap-1	Rap1a		4	12.8	28.156	257	0	53.712	10.49189922	9.937668604	10.98112757	5.026156598	7.633067562	6.073042555
tr|D3ZAP9|D3ZAP9_RAT	CD81 antigen (26 kDa cell	Cd81		5	15.1	38.066	351	0	60.327	9.177770406	10.55455174	10.00897111	13.22252458	12.55060003	11.49063588
sp|P69897|TBB5_RAT Tu	Serpin H1 (47 kDa heat sh	Serpinh1		3	12.2	49.67	444	0	262.99	26.46318933	29.63940339	28.19672541	16.96609976	22.55640809	20.16905064
sp|Q7M0E7|RM14_RAT 39	Cytochrome c, somatic	Cycs		2	24.8	15.912	145	0	8.5934	2.350639949	2.240850572	2.377150271	3.686151998	3.140364889	3.70854922
sp|P84100|RL19_RAT 60S	Alpha-aminoadipic semiald	Aldh7a1		3	13.8	23.466	196	0	85.493	7.422276114	6.802836893	6.688876192	4.688475265	5.846479671	4.75228925
sp|P56522|ADRO_RAT NA	Septin-2 (Vascular endoth	Septin2		5	13.4	54.362	494	0	17.463	11.79645914	12.69675808	9.802185006	16.60020598	14.69699142	15.68869327
tr|G3V7G0|G3V7G0_RAT	Caveolin-1	Cav1		3	8.8	56.67	523	0	5.9838	11.28571915	12.23587932	13.02176617	7.977178939	9.580585743	9.94055233
tr|A0A0G2K9B4|A0A0G2K	Isocitrate dehydrogenase	Idh3a		5	23.1	33.652	295	0	24.439	4.081534195	4.446172101	4.612534269	5.698915037	5.04529542	5.262071693
tr|D3ZQL1|D3ZQL1_RAT	Protein FAM210A	Fam210a		2	12	26.44	241	0	6.1977	6.024020725	6.045169329	6.069041379	4.469286225	4.636820327	5.100288139
tr|M0R3V4|M0R3V4_RAT	Collagen type VI alpha 1 c	Col6a1		2	14.5	17.952	166	0	11.421	10.61749103	10.66489629	9.602204517	7.991502183	8.963686299	8.733334945
tr|G3V9W6|G3V9W6_RAT	NADH dehydrogenase [ub	Ndufa8		9	23.1	54.107	484	0	52.392	31.07061427	31.05818703	31.91102271	28.85439244	26.49134993	27.30867386
tr|D4A4P4|D4A4P4_RAT	Junction plakoglobin	Jup		4	10.2	54.604	490	0	12.141	4.927185659	4.701819506	4.981922313	6.126008136	5.519451005	6.076122191
tr|F1LMC7|F1LMC7_RAT	Phosphatidylethanolamine	Pebp1		8	20.2	50.579	436	0	182.15	23.79864962	28.29148758	26.18226227	17.93226769	21.53325779	19.65885758
tr|D4A520|D4A520_RAT	Calreticulin (CALBP) (CRP	Calr		3	13	32.847	307	0	12.353	5.795961961	5.598559258	6.052812596	6.515773993	6.47083686	6.641235431
tr|F1LMQ3|F1LMQ3_RAT	Glycogen phosphorylase,	Pygb		3	9.9	39.877	353	0	2.5387	4.16685688	3.721179842	3.786908015	3.009313604	3.415908946	3.179005778
tr|Q5RKJ9|Q5RKJ9_RAT	Signal recognition particle	Srprb		7	32.5	22.541	200	0	116.77	32.66862043	32.92786135	35.97293007	27.90037757	29.42276711	27.82348638
sp|Q6P6R2|DLDH_RAT Di	Mitochondrial import recep	Tomm40		20	47	54.037	509	0	323.31	217.4492463	244.8460046	235.1184218	357.3345599	299.9256501	313.1117436
sp|Q63569|PRS6A_RAT 2	Catalase (EC 1.11.1.6)	Cat		4	12.8	49.16	439	0	5.5562	6.676699388	6.277749014	6.544387672	7.074380517	7.23008765	7.097534859
sp|P07895|SODM_RAT Su	Voltage-dependent calciu	Cacna2d1		9	33.3	24.674	222	0	323.31	87.38398453	97.84993596	92.35224615	128.4013736	120.7631361	109.67611
sp|P0DP31|CALM3_RAT	Annexin	Anxa11		10	55.7	16.837	149	0	166.57	56.08969724	49.13186402	52.81160134	38.1384589	45.64074603	38.81522119
tr|G3V8S6|G3V8S6_RAT	Tropomyosin alpha-1 chain	Tpm1		10	23.1	86.502	754	0	323.31	24.32693259	23.17901511	29.56360776	12.97729327	14.83175418	13.88402646
tr|F1LSM0|F1LSM0_RAT	60S ribosomal protein L4 (	Rpl4		2	1.5	294.03	2617	0	5.2406	1.008801194	0.953671835	1.022203933	1.365699632	1.356262913	1.19264044
tr|F1M957|F1M957_RAT v	3-hydroxyisobutyrate dehy	Hibadh		4	2.2	308.98	2812	0	8.6782	2.05543941	2.221254902	1.978236312	3.515922411	2.828251713	3.256253319
tr|G3V8Y8|G3V8Y8_RAT	Isoleucyl-tRNA synthetase	Iars2		2	3.4	115	1030	0	9.1647	1.479750514	1.235335822	1.085339135	2.368326725	1.785999122	2.070439407
sp|Q9R066|CXAR_RAT C	Ribosomal protein L3-like	Rpl3l		4	12.1	39.948	365	0	5.3934	7.983252828	7.227092679	8.471948299	5.900308531	5.872385523	6.425660898
sp|P14841|CYTC_RAT Cy	Glypican-1 (HSPG M12) [C	Gpc1		2	20	15.437	140	0	2.6224	7.719709397	7.986662736	8.126955781	8.639954511	8.574444338	8.338628241
sp|I6L9G5|RCN3_RAT Re	Synaptosomal-associated	Snap23		3	13.7	37.941	328	0	7.7516	4.896884145	5.683220166	6.106734036	3.495349024	4.168748692	3.815258614
sp|P08644|RASK_RAT GT	Fibronectin (FN) [Cleaved	Fn1		2	7.9	21.656	189	0	2.9899	3.140871538	3.169932694	2.679790901	4.082645439	3.5965303	4.314518967
sp|P63031|MPC1_RAT Mi	Paraplegin (EC 3.4.24.-)	Spg7		3	15.6	12.454	109	0	4.9285	110.9194894	136.8415159	122.8518882	90.08886558	96.48621313	77.99178636
tr|F1MA10|F1MA10_RAT	Tubulin beta-4B chain (Tu	Tubb4b		2	9.5	34.215	306	0	11.082	3.170535125	2.633049363	2.940236695	2.005644821	2.383730852	2.151639147
sp|P05712|RAB2A_RAT R	ATP-binding cassette sub-	Abcb6		11	60.4	23.535	212	0	92.595	80.45051968	82.21620536	76.84067054	72.31068128	70.87474629	70.48773953
sp|P02767|TTHY_RAT Tra	Calpain-2 catalytic subunit	Capn2		2	19	15.72	147	0	6.1309	1.029055364	1.336167915	1.343690892	0.815904074	0.834286173	0.588929093
tr|A0A0G2K0X8|A0A0G2K	Mitochondrial import inner	Timm50		7	18	57.507	523	0	30.068	25.41539487	22.39043205	24.20287424	16.83198211	17.71345307	20.74237625
tr|D4AEL2|D4AEL2_RAT	Proline dehydrogenase (E	Prodh1		2	6	44.866	397	0	3.1659	5.553151145	6.097012244	5.377485814	4.925459851	4.573494912	4.465267158
tr|D3ZVP9|D3ZVP9_RAT	3-hydroxyisobutyryl-CoA h	Hibch		2	3.5	97.02	862	0.0017913	1.7109	4.294043498	7.039697186	9.151986663	21.39502048	14.25999372	15.64814472
tr|D3ZKU5|D3ZKU5_RAT	2-methoxy-6-polyprenyl-1,4	Coq5		5	38.7	14.275	124	0	14.884	14.63563126	16.03563209	18.44270325	7.890805436	10.33551889	12.32983674
sp|Q5RK28|NMES1_RAT	DnaJ heat shock protein fa	Dnaja3		2	37.3	9.5981	83	0	7.706	5.930325254	6.996891109	5.415178472	8.440297167	8.138100829	10.01138396
tr|Q5XIG9|Q5XIG9_RAT	Peroxiredoxin-5, mitochon	Prdx5		6	42.2	18.366	166	0	81.77	31.56301388	36.7271385	33.40302374	27.92815598	24.7067246	27.85582256
tr|A0A0G2K585|A0A0G2K	Mitochondrial import recep	Tomm22		2	20.5	16.239	151	0	20.27	5.841414232	5.814016513	5.845502979	4.524843051	4.971306738	4.327145475
tr|D4A9W3|D4A9W3_RAT	Dihydrolipoamide acetyltra	Dbt		11	26.5	66.055	618	0	85.462	19.04689378	17.87581779	17.25224348	23.16849853	20.90196621	24.22339168
tr|D3ZUZ4|D3ZUZ4_RAT	ATPase family AAA domai	Atad1		2	5.9	46.568	409	0	3.51	3.521155358	3.917374362	3.417781726	2.664080016	3.11930003	2.890803159
tr|D3ZYM7|D3ZYM7_RAT	Gap junction alpha-1 prote	Gja1		4	48.4	10.816	91	0	9.7406	12.27649892	11.25371766	10.91673595	14.0298347	13.68299975	13.82807974
sp|P16290|PGAM2_RAT	Septin-7 (CDC10 protein h	Septin7		8	32.8	28.755	253	0	61.813	73.22998786	65.68830338	62.93103288	49.08098343	57.99051029	48.90410933
sp|P20037|HA2B_RAT Ra	NADH dehydrogenase (Ub	Ndufs5		3	10.9	28.601	256	0	2.3185	3.84558109	3.038089977	3.722306988	1.534366683	2.18204464	2.584892631
sp|Q6TUG0|DJB11_RAT	Elongation factor G, mitoc	Gfm1		5	20.9	40.495	358	0	14.758	3.73940618	4.0038902	4.559345741	2.706745922	2.969687196	3.281608991
sp|Q9JIX3|FHIT_RAT Bis(	Aspartate aminotransferas	Got1		2	19.3	17.348	150	0	12.443	3.199082341	3.665722191	2.934425743	5.334757404	4.190009807	4.924184277
tr|D4A0W1|D4A0W1_RAT	Synaptojanin-2-binding pr	Synj2bp		3	21.3	20.158	183	0	16.27	9.417391589	9.390702064	8.675593352	7.713718053	7.621292175	7.643657009
tr|D4A772|D4A772_RAT	Malate dehydrogenase, cy	Mdh1		8	16.8	70.926	626	0	69.193	24.93774732	20.13740552	25.68283456	29.26542614	33.33415818	32.12933104
sp|Q642A4|CH082_RAT	Alpha-enolase (EC 4.2.1.1	Eno1		4	31.2	24.397	218	0	117.55	16.80298692	17.41731714	16.00786227	27.02752775	22.34248855	23.72705698
tr|F1LMP9|F1LMP9_RAT	Cytochrome b-c1 complex	Uqcrb		3	4.8	82.218	766	0.0017762	1.659	6.747668724	7.504380934	7.247983946	5.50186192	5.985232982	6.389218537
tr|F1LTJ5|F1LTJ5_RAT Ur	40S ribosomal protein SA	Rpsa		26	16.3	263.11	2449	0	216.47	56.00995641	63.88569192	59.86850445	45.97761385	51.20069128	51.23180101
sp|P18422|PSA3_RAT Pr	Alpha-actinin-4 (Non-musc	Actn4		4	13.7	28.419	255	0	4.652	5.090654353	4.73487531	4.762048477	3.58107147	4.150169748	3.329651315
tr|A0A0G2K398|A0A0G2K	NADH:ubiquinone oxidore	Ndufa13		7	36.2	22.074	196	0	38.251	33.76226587	35.92381113	32.22722223	37.65320475	38.95886806	39.98394312
sp|Q498C8|RER1_RAT Pr	Ubiquinol-cytochrome c re	Uqcr10		3	23	22.988	196	0	20.628	3.17388424	2.840421025	3.328575769	2.263246197	2.594248605	2.527919362
tr|F1LSP2|F1LSP2_RAT A	Myosin heavy chain 14	Myh14		16	21.2	119.19	1069	0	98.707	20.74178504	20.49887906	20.85136877	39.83163998	31.40168633	35.96655772
sp|Q91ZN1|COR1A_RAT	NADH dehydrogenase (Ub	Ndufb6		6	19.5	51.065	461	0	10.144	16.18818515	13.32600742	13.83425235	17.54597413	18.73202405	19.25183233
sp|P14604|ECHM_RAT En	Enoyl-CoA delta isomerase	Eci2		15	47.9	31.516	290	0	271.32	134.7619965	134.2779075	148.1949654	189.7091991	162.4349225	187.3291364
tr|M0RAK2|M0RAK2_RAT	Calsequestrin-2 (Calseque	Casq2		2	12.9	22.929	209	0	20.448	8.672612271	8.761452731	5.92926444	12.96123388	10.6946682	12.30622619
tr|B2GUZ6|B2GUZ6_RAT	F-actin-capping protein su	Capza2		10	30.3	43.605	396	0	122.05	40.69174367	38.29479217	40.10917573	65.01016712	53.2783929	58.54901659
tr|D3ZUX7|D3ZUX7_RAT	Complex I assembly factor	Acad9		9	22.6	65.265	583	0	47.307	29.22740507	27.52953941	27.63657064	32.0059402	29.86630669	31.75823488
sp|P68511|1433F_RAT 14	Integrin alpha-7 (H36-alph	Itga7		5	18.7	28.211	246	0	19.806	11.82197621	11.74408506	12.13180065	8.493683805	10.14253338	9.115723105
sp|Q4V7C7|ARP3_RAT A	Peroxisomal trans-2-enoyl-	Pecr		9	29.2	47.357	418	0	97.68	40.61598989	38.38991679	42.64767152	27.6221594	34.80150224	31.04427255
tr|F1LYX9|F1LYX9_RAT	Cytochrome b5 type B (Cy	Cyb5b		8	9	122.29	1128	0	18.784	20.670417	20.39519323	22.10203209	17.66967488	19.4588271	18.36027766
tr|A0A0G2K588|A0A0G2K	Calmodulin-3	Calm3		5	5.4	154.01	1438	0	18.541	2.682242175	2.788625672	2.934896901	2.253480349	2.517250658	2.483469946
sp|P07340|AT1B1_RAT S	Filamin B (Filamin, beta (P	Flnb		10	30.3	35.201	304	0	157.53	139.2394439	125.9259663	116.8158279	93.43529627	104.4673088	94.64235267

tr|F7EPH4|F7EPH4_RAT F	Troponin I, cardiac muscle	Tnni3	2	10.7	32.771	289	0	3.429	10.75065821	12.96310701	9.247265325	15.11406088	14.84614632	17.45948407
sp|O88656|ARC1B_RAT A	Alpha-soluble NSF attachn	Napa	7	30.4	41.056	372	0	51.824	14.25925456	15.11625268	13.96408261	9.726784918	12.12472353	11.48345006
tr|F1LP67|F1LP67_RAT U	Acyl-CoA thioesterase 9 (S	Acot9	7	17.7	56.724	497	0	75.61	16.20094368	17.66892175	18.83062351	12.46946916	15.11632603	14.06418518
tr|A0A0G2K466|A0A0G2K	Basal cell adhesion molecu	Bcam	5	19.4	34.847	310	0	18.713	17.97996152	18.67296207	17.50614541	24.72278755	21.68830039	22.05430126
sp|P52873|PYC_RAT Pyr	Transforming protein RhoA	Rhoa	17	21	129.78	1178	0	87.074	35.99261678	35.73261065	44.24385087	54.82764261	46.57427254	53.051866
sp|Q8VHI8|SEC20_RAT V	Dolichyl-diphosphooligosac	Rpn2	4	18.4	26.17	228	0	4.9124	5.184748528	4.410975993	4.986529194	3.920836184	3.943119192	3.568477101
tr|D4A994|D4A994_RAT E	Cytochrome c oxidase sub	Mtco2	7	8.6	111.18	993	0	13.342	19.65252535	19.4753382	17.36584496	15.15703062	15.12810142	16.42575286
tr|Q66HL0|Q66HL0_RAT 5	Tissue-type transglutamina	Tgm2	12	27.6	63.95	576	0	150.59	56.09767132	45.14756949	54.5234762	33.5623994	40.35359724	36.33970697
tr|D4A5G8|D4A5G8_RAT	WD repeat-containing prot	Wdr1	26	66.2	43.274	390	0	323.31	298.8127985	325.2928921	315.7440634	479.8286804	395.7249656	433.7872874
sp|Q4QQW3|HOT_RAT Hy	ATP synthase subunit e, m	Atp5me	5	15	50.225	467	0	18.303	11.44320728	12.69009936	12.59615325	16.46478622	14.15924874	15.0188724
sp|P62907|RL10A_RAT 60	Sodium/potassium-transpo	Atp1b1	8	35.5	24.831	217	0	60.154	39.79067234	35.36923462	36.18442776	24.24013762	29.84013917	30.69114094
sp|Q6PCT3|TPD54_RAT T	Cadherin-2 (Neural cadher	Cdh2	5	34.5	23.992	220	0	30.505	14.9091423	14.66155702	13.83111129	20.55776175	22.9096697	18.74831182
tr|D3ZF54|D3ZF54_RAT A	Ras-related protein Rab-14	Rab14	4	7.3	79.039	688	0	8.9159	8.675801904	7.966686566	8.754119722	7.218915072	7.659889277	6.683323792
sp|O35796|C1QBP_RAT C	Regulator of microtubule d	Rmdn1	8	43	30.997	279	0	286.92	50.51581348	48.04268717	48.16336374	67.23678053	58.02649064	62.08033275
sp|Q4TU93|MRC2_RAT C-	Flotillin-2 (Reggie-1) (REG-	Flot2	2	2.3	167.02	1480	0	6.0847	2.075853061	2.229530743	2.202088817	1.583933789	1.848146998	1.861486091
sp|P56571|ES1_RAT ES1	Prostaglandin E synthase	Ptges2	9	43.6	28.172	266	0	323.31	107.2553984	117.0603521	122.2812633	199.7571719	156.7173179	173.9840462
tr|B4F7C9|B4F7C9_RAT S	BCS1 homolog, ubiquinol-	Bcs1l	5	7	80.597	705	0	13.464	11.54088979	11.22327779	11.13975084	7.600000175	9.360778518	8.743087127
tr|A0A0G2K4S6|A0A0G2K	Plasminogen (EC 3.4.21.7	Plg	2	3.9	70.798	614	0.00046882	2.0628	5.465436236	5.768356697	5.92926444	3.998181703	4.962867711	4.218793609
tr|G3V811|G3V811_RAT C	CD163 molecule	Cd163	2	4.2	83.053	732	0	4.0472	0.964465294	1.228772224	1.026758463	1.509713341	1.370589634	1.391122992
tr|G3V7Q3|G3V7Q3_RAT	Glypican 4	Gpc4	2	11.9	29.588	252	0	43.258	5.246946372	5.659439012	5.380626869	4.80653352	5.095929584	4.695315981
tr|F1LQS6|F1LQS6_RAT F	MICOS complex subunit	Apool	4	3.5	146.26	1331	0	18.56	5.704260011	6.291542083	6.459055683	7.531856256	6.971683323	6.949712322
tr|D3ZY50|D3ZY50_RAT A	Ornithine aminotransferase	Oat	2	11.2	38.649	348	0	53.497	1.883797281	2.111671344	1.710566092	2.483086294	2.216912869	2.514728253
tr|A0A0G2K542|A0A0G2K	Basigin (Glycoprotein CE9	Bsg	7	16.2	56.885	507	0	18.382	16.92020594	14.91221038	13.97193525	10.15648224	12.61078534	11.72058204
sp|P09895|RL5_RAT 60S	NADH dehydrogenase (Ub	Ndufs7	9	35	34.458	297	0	28.204	41.16620159	34.80181628	32.30051351	22.61032605	27.57991904	23.98574642
tr|F1M8H2|F1M8H2_RAT T	Mitochondrial potassium ch	Ccdc51	2	8.6	40.141	360	0	18.31	2.912015366	3.636994557	3.276591313	5.168955002	4.189159363	4.34059322
sp|P51868|CASQ2_RAT C	NADH:ubiquinone oxidored	Ndufaf2	13	36.3	47.838	413	0	170.02	275.412853	298.0587147	260.8803062	225.0138259	248.1074052	211.4118913
tr|A0A0G2JSU4|A0A0G2J	LIM domain-binding 3	Ldb3	9	45.1	39.255	357	0	301.86	65.10041061	45.39441787	59.26646895	25.92507198	40.56555421	28.55489997
tr|Q5U2U3|Q5U2U3_RAT	5'-nucleotidase (5'-NT) (EC	Nt5e	2	4.6	58.868	526	0	2.3573	2.401036151	2.647746116	2.728581952	4.27179907	3.351340574	3.954150206
sp|Q64232|TECR_RAT Ve	Protein kinase C and case	Pacsin2	6	17.5	36.122	308	0	51.862	16.65267547	17.45061075	16.72664031	11.01370671	14.00158939	12.94884361
tr|G3V8I4|G3V8I4_RAT Sy	Sarcolemmal membrane-as	Slmap	6	28.2	34.195	298	0	21.867	14.93904511	12.31911336	14.39859519	9.65082832	11.67464207	10.18692322
tr|F1LQZ0|F1LQZ0_RAT T	Polyribonucleotide nucleot	Pnpt1	8	31.7	27.733	259	0	30.565	43.49463372	50.97728156	52.17292019	33.98037771	34.79626874	35.93576136
tr|A0A0A0MXU4|A0A0A0M	Dihydrolipoamide acetyltra	Pdhx	5	22.3	34.513	296	0	30.969	6.429502827	6.821861816	6.580509802	5.704991565	4.602802542	4.974279692
tr|F1LNQ5|F1LNQ5_RAT A	Protein disulfide-isomerase	P4hb	2	5.9	44.043	390	0	2.8077	3.494641714	3.388481499	3.294861781	4.637258816	3.974847318	4.473890139
sp|P70473|AMACR_RAT A	von Willebrand factor A do	Vwa8	4	16	41.828	382	0	10.394	7.416295552	8.184997559	7.253219038	10.66951481	9.053310078	9.378518726
tr|G3V7L1|G3V7L1_RAT U	Glycerol-3-phosphate dehy	Gpd2	17	6.3	391.31	3419	0	27.406	32.59286665	29.68363633	34.35423983	36.67748799	37.39470415	39.63081151
tr|B2GV73|B2GV73_RAT A	Alpha-1-inhibitor 3 (Alpha-1	A1i3	4	27	20.535	178	0	35.199	17.83882026	19.04632618	18.99971696	12.8140951	15.9641539	14.82793495
tr|Q5XIU4|Q5XIU4_RAT B	Erythrocyte membrane pro	Epb42	3	10.4	28.234	241	0	17.026	3.913001959	4.36536374	4.781523017	3.091824172	3.705910562	2.951471992
sp|Q58FK9|KAT3_RAT Ky	NADH dehydrogenase [ub	Ndufa11	12	28.9	51.044	454	0	75.927	42.35035286	40.46125529	40.25785233	62.64466164	51.83721637	56.18796218
tr|A0A0G2K2I6|A0A0G2K	NAD(P)(+)--arginine ADP-ri	Art3	5	3	239.23	2095	0	5.8519	12.07953908	10.49795259	12.09253746	8.840479929	9.665630205	9.233262553
sp|P24473|GSTK1_RAT G	Fumarylacetoacetate hydro	Fahd2	10	46.9	25.493	226	0	109.54	49.28381771	50.90118187	47.61106161	60.54391915	56.25102395	54.32991501
tr|F1LT35|F1LT35_RAT Si	Non-specific lipid-transfer p	Scp2	4	28.8	17.734	156	0	24.525	19.49065147	16.26012619	18.21497677	13.18215907	12.96797209	13.60993884
tr|A0A0G2K7X0|A0A0G2K	Laminin subunit alpha 2	Lama2	7	27.1	26.316	229	0	59.037	24.29344144	24.42276946	21.4324639	16.68180507	19.3044387	20.24860791
tr|G3V7V5|G3V7V5_RAT	Kinesin-1 heavy chain (Co	Kif5b	2	10.4	22.185	201	0	2.4581	7.575777205	9.068229616	8.964570393	5.87209608	6.404960112	7.243304305
tr|M0RAK4|M0RAK4_RAT	Chloride intracellular chann	Clic1	3	16.5	22.883	206	0	44.516	10.65496921	11.34551292	11.97788896	14.29416366	13.09815553	12.66910999
tr|D3ZRJ0|D3ZRJ0_RAT A	NADH dehydrogenase (Ub	Ndufb5	2	5.3	51.71	455	0	4.4058	7.829751737	7.750278065	7.636427723	9.466362297	8.699394279	8.742573854
sp|P05708|HXK1_RAT He	ATP synthase F(0) comple	Atp5pb	6	5.3	102.41	918	0	323.31	234.4300553	255.3097124	226.9935601	207.4917238	210.6420487	193.8579651
sp|Q5XIT9|MCCB_RAT Me	Leucyl-cystinyl aminopepti	Lnpep	23	50.6	61.516	563	0	323.31	98.0732423	97.71676149	92.72393764	153.6710487	124.3153779	137.6700029
sp|P49432|ODPB_RAT Py	Histidine triad nucleotide b	Hint2	15	59.1	38.982	359	0	323.31	389.864861	422.4389059	412.3681441	597.4528978	496.5811557	536.0106778
sp|Q2PQA9|KINH_RAT Ki	Chloride intracellular chann	Clic4	15	20	109.53	963	0	144.26	35.26378563	31.93666286	35.15939688	28.50716228	30.84824314	29.58606482
tr|D3ZH41|D3ZH41_RAT C	RAS-related 2 (RCG40097	Rras2	13	46.3	36.315	326	0	136.28	55.08097578	62.37796676	70.47479948	38.77606029	46.48988227	48.17680192
tr|F1LW74|F1LW74_RAT I	Protein FAM162A (E2-indu	Fam162a	4	3	162.97	1425	0	2.4353	3.555922539	4.421344576	4.251260616	2.706572307	2.992322106	3.079636184
sp|P19804|NDKB_RAT Nu	Thioredoxin-dependent pe	Prdx3	2	16.4	17.283	152	0	45.397	56.21728256	51.14850587	50.56470014	32.3640213	44.38208799	37.60338435
tr|F1LMW7|F1LMW7_RAT	Syntrophin, basic 1 (Predic	Sntb1	6	33.3	29.959	309	0	64.588	4.930773996	5.670378343	5.369633177	1.095337547	3.416301459	2.213077889
tr|E9PT51|E9PT51_RAT D	Delta-1-pyrroline-5-carboxy	Aldh4a1	9	28.5	41.797	368	0	18.068	27.47749264	28.42466205	28.22761245	37.71700829	32.72968831	35.03650759
sp|B4F795|CTL2_RAT Ch	Cytochrome c heme lyase	Hccs	4	7.1	79.896	705	0	13.72	9.91258212	9.40259264	10.35605767	11.20772156	11.03811698	12.32059783
sp|Q6AYQ8|FAHD1_RAT A	Cytoskeleton-associated p	Ckap4	4	24.4	24.48	221	0	94.043	18.19765398	20.87129193	19.29497611	27.35826448	23.05228272	28.33727235
sp|P97519|HMGCL_RAT H	Amylo-1, 6-glucosidase, 4-	Agl	7	30.8	34.191	325	0	98.869	26.32882604	29.19136645	23.74375673	46.75454134	36.48930772	37.28823492
tr|A0A0G2JYY6|A0A0G2J	Flotillin-1 (Reggie-2) (REG-	Flot1	5	23.5	23.331	204	0	18.459	30.83019568	29.10052244	26.71205351	34.54723095	34.81720276	35.14686122
tr|Q5RKK3|Q5RKK3_RAT	60S ribosomal protein L10	Rpl10	4	9.9	62.328	554	0	25.873	9.65581666	11.98332346	10.35605767	14.4200346	13.04385791	12.83489707
sp|P18297|SPRE_RAT Se	NADH dehydrogenase [ub	Ndufa6	5	27.1	28.128	262	0	11.132	12.09389242	11.26988885	12.69823753	21.14110842	16.96964112	22.94893557
sp|P56574|IDHP_RAT Iso	ATP synthase subunit g, m	Atp5mg	25	56.6	50.967	452	0	323.31	889.4690472	890.1285916	928.1816928	1272.945775	1089.942909	1129.66188
sp|P03889|NU1M_RAT NA	Acylglycerol kinase	Agk	4	14.2	36.145	318	0	35.619	51.41688481	59.80960213	59.42352169	45.94289083	42.6726943	49.42764748
sp|P06685|AT1A1_RAT S	Parkinson disease protein	Park7	29	28.6	113.05	1023	0	323.31	513.0126062	455.5945907	511.3218404	360.455291	442.4143753	385.5139903
tr|A0A0G2K1W9|A0A0G2I	Erythrocyte membrane pro	Epb41	8	24.9	51.842	482	0	30.267	21.4132028	20.67010337	21.73662271	39.51783072	30.08742229	34.62434962
tr|F1LP30|F1LP30_RAT M	Sarcoglycan, gamma (Sarc	Sgcg	26	54.3	79.295	715	0	323.31	121.49711	129.2363029	123.9460223	172.3563718	151.3726006	149.3623549
sp|Q641Y0|OST48_RAT D	Peptidyl-prolyl cis-trans iso	Ppia	4	10.9	48.895	441	0	17.247	20.53406019	24.00041617	22.71401426	17.67575141	17.03440575	19.99043174
tr|A0A0H2UHU1|A0A0H2U	Elongation factor 1-alpha 2	Eef1a2	3	41.3	10.276	92	0	24.978	8.234037726	9.032082262	6.599356131	4.797418728	5.178749807	4.406138143

sp|Q5XI78|ODO1_RAT 2-(	ATP synthase membrane s	Atp5md	51	49.5	116.29	1023	0	323.31	411.1835709	467.3947993	462.6721366	575.5339938	501.6707397	526.1045148
tr|G3V7I0|G3V7I0_RAT Pe	Enoyl-[acyl-carrier-protein]	Mecr	8	37	28.299	257	0	141.13	80.2591417	77.58839281	78.30649611	131.4960624	104.068254	116.9235205
sp|Q9Z1W6|LYRIC_RAT P	NADH:ubiquinone oxidored	Ndufb4	5	10	63.968	581	0	67.281	18.87266007	19.68033175	19.37297898	13.07104542	14.52690249	16.5227614
sp|Q9ES40|PRAF3_RAT P	Myoglobin	Mb	4	25	21.548	188	0	26.657	9.060551391	9.398787656	9.7273232	6.696767716	8.282022227	7.514312289
tr|A0A096MJ01|A0A096M	Hemopexin	Hpx	15	29.1	76.815	726	0	151.19	31.06941816	28.89552889	34.19404604	35.45133148	41.50562261	41.98160046
tr|G3V965|G3V965_RAT 1	Clusterin (Dimeric acid glyc	Clu	3	10.9	41.531	359	0	6.1814	7.677845463	7.709850104	7.884571052	6.54094818	5.113919759	5.591079492
tr|Q642E6|Q642E6_RAT T	Erythrocyte membrane pro	Epb41l2	2	6.2	61.312	563	0	35.475	4.751755841	5.114850587	5.207921206	6.560913915	5.616925043	6.196228002
tr|M0RB74|M0RB74_RAT	ATPase family AAA domai	Atad3	4	9.2	92.662	818	0	26.4	3.909812326	3.544580993	4.302669213	2.951326167	3.300640991	2.91615883
tr|F1LMK0|F1LMK0_RAT A	Dihydroorotate dehydroge	Dhodh	2	5.1	57.651	544	0	3.6477	0.913311554	0.793957605	0.841226826	0.947417498	0.974740373	1.036451558
sp|Q68FT1|COQ9_RAT Ub	3-mercaptopyruvate sulfur	Mpst	8	35.9	35.145	312	0	323.31	32.06458367	36.9083509	38.68418387	46.68943568	40.44322102	46.03286186
sp|Q4G064|COQ5_RAT 2-	Mitochondrial import recep	Tomm70	13	47.1	37.3	327	0	189.51	38.95419107	38.89693099	41.98019737	55.41793389	46.5049286	50.22886616
tr|F1LRA1|F1LRA1_RAT P	Inorganic pyrophosphatas	Ppa2	5	11	57.985	517	0	37.291	20.87136388	21.20137435	23.04120747	17.95830995	18.84912373	20.04175901
sp|P19357|GLUT4_RAT S	Elongation factor 2 (EF-2)	Eef2	4	14.3	54.895	509	0	64.83	12.50575379	13.46679185	12.17315787	11.37699626	10.7941048	11.32741516
sp|P14882|PCCA_RAT Pr	14-3-3 protein theta (14-3-	Ywhaq	2	3.7	81.622	737	0	247.29	8.049437713	7.071563932	8.364105418	12.02024014	9.568810356	10.38145358
tr|A0A0A0MY09|A0A0A0M	Reticulon 4-interacting pro	Rtn4ip1	25	35.2	92.899	804	0	323.31	124.4714429	127.795165	126.9928455	98.08818044	108.7326157	112.7711443
sp|Q63081|PDIA6_RAT P	Mitochondrial Rho GTPase	Rhot1	8	28	48.173	440	0	88.74	42.8168367	43.98847605	42.03150127	37.60459253	37.44049732	40.81236526
tr|A0A0G2JYL4|A0A0G2J	Cytochrome c oxidase sub	Cox5a	2	5.8	60.635	533	0	6.8692	0.555115762	0.771317947	0.908707153	0.439289559	0.402580858	0.165941063
tr|A0A0G2K3Z9|A0A0G2K	Sarcoglycan, delta	Sgcd	9	46.7	22.164	199	0	30.8	60.01693293	58.08309036	59.13035657	41.6311603	51.15751486	42.90805768
tr|M0R6N2|M0R6N2_RAT	Glutathione peroxidase 1 (	Gpx1	3	9.2	38.175	338	0	40.878	7.124444128	6.571684077	5.716719732	9.623049907	8.036047475	8.002434624
sp|P63029|TCTP_RAT Tra	Ubiquitin-40S ribosomal pr	Rps27a	3	23.3	19.462	172	0	24.301	16.10126765	15.02826241	14.88074711	12.49334124	13.94794596	13.91687592
sp|P35435|ATPG_RAT AT	Transmembrane protein 14	Tmem143	13	50.9	30.19	273	0	264.61	586.6133889	656.5500977	622.2429555	561.1673459	535.2698441	529.2868055
sp|P21670|PSA4_RAT Pro	Plastin-3 (T-plastin)	Pls3	3	13.4	29.497	261	0	11.522	11.83991789	9.678929649	10.85600889	8.569206365	8.530613731	7.732453186
tr|D4A264|D4A264_RAT Z	Sorbin and SH3 domain-co	Sorbs1	9	30	40.535	377	0	80.01	26.15818067	27.35641261	26.02992111	30.92648843	28.30018021	29.24679157
sp|P62959|HINT1_RAT Hi	14-3-3 protein zeta/delta (	Ywhaz	2	16.7	13.777	126	0	2.5894	5.515672957	5.481555981	6.357494911	3.6101954	4.985633459	4.136105377
tr|F1LRY5|F1LRY5_RAT R	NADPH--cytochrome P450	Por	6	11	101.41	919	0	20.6	9.571291384	10.21543248	8.679257916	14.6053687	11.45810579	14.27462699
sp|B0BNC9|QORL2_RAT (	14-3-3 protein epsilon (14-	Ywhae	5	18.9	37.548	350	0	41.713	5.108197334	5.140534233	4.360045814	8.716345146	6.502041636	7.278720121
sp|F1LU71|AUHM_RAT Me	Dystroglycan 1	Dag1	11	33	33.341	315	0	52.509	73.32966389	74.41123064	75.00315348	102.4372382	86.90889833	96.86482344
tr|A0A0G2JVY2|A0A0G2J	60S ribosomal protein L9	Rpl9	13	16.6	119.61	1111	0	153.33	19.29289423	19.63800129	18.81596526	27.29619709	33.01295179	37.01055439
tr|Q52KS1|Q52KS1_RAT	Complement C3 [Cleaved	C3	18	31.2	85.342	780	0	323.31	107.4348153	93.31724802	114.5647387	75.04945918	83.42207538	81.95938431
tr|D3ZLG3|D3ZLG3_RAT U	Ras-related protein Rab-10	Rab10	3	14.1	24.112	206	0	17.436	13.49454004	13.95525675	13.04741812	12.5953401	11.57193453	11.00097372
sp|P85108|TBB2A_RAT T	Cysteine and glycine-rich p	Csrp3	2	6.1	49.906	445	0	24.154	2.918992688	3.102679591	3.65247087	2.193192512	2.688386283	2.293610375
sp|P00388|NCPR_RAT NA	Peptidylglycine alpha-amid	Pam	16	28.8	76.962	678	0	119.42	27.92643349	26.36045788	28.01663827	22.48054878	24.81531984	25.25558307
tr|F1LM47|F1LM47_RAT S	Solute carrier family 12 me	Slc12a7	20	51.6	50.306	465	0	323.31	206.1539583	224.0707886	208.6550351	283.9778478	241.8010312	257.5499741
sp|B2GUZ5|CAZA1_RAT F	MICOS complex subunit	Apoo	5	29.4	32.909	286	0	33.586	5.961025472	5.98048459	6.688876192	5.568703726	5.058771696	5.295947691
sp|P04897|GNAI2_RAT G	60S ribosomal protein L12	Rpl12	11	43.7	40.499	355	0	323.31	157.6117303	148.7225804	156.5711115	124.0523158	143.2017903	128.1077325
tr|A0A0G2JZF6|A0A0G2J	Glutaryl-CoA dehydrogena	Gcdh	8	53	19.554	166	0	151.29	23.69618265	27.14047973	29.72589559	21.02869266	22.00623584	17.66274006
sp|Q641X9|RM09_RAT 39	Protein kinase C and case	Pacsin3	2	10.3	30.076	262	0	24.736	6.241713181	6.017107567	6.004126246	7.852610118	6.879442791	7.912611902
tr|G3V9Z6|G3V9Z6_RAT S	Biglycan (Bone/cartilage p	Bgn	5	18.4	49.856	429	0	57.505	8.327733197	7.904379943	8.456243025	7.196779149	7.825398884	7.108826858
sp|Q5XFX0|TAGL2_RAT T	GTP:AMP phosphotransfe	Ak3	6	33.7	22.393	199	0	33.702	27.18683733	34.17875005	28.51501897	20.90629403	22.29865795	19.96733447
sp|Q5XIM4|ATP5S_RAT A	ATP-binding cassette sub-	Abcb7	4	20.5	23.324	200	0	17.858	15.77273545	15.76595381	13.78923056	23.24315302	18.15110495	22.12051344
tr|Q5XFW4|Q5XFW4_RAT	FAST kinase domain-conta	Tbrg4	8	50	20.54	178	0	24.394	16.72165128	15.90531137	17.17109956	20.36114267	18.17138479	20.84041133
tr|Q4G079|Q4G079_RAT	Acyl carrier protein	Ndufab1	2	10.2	34.574	315	0	22.405	6.50126957	5.603791112	5.695779367	4.524409013	5.317699374	4.602978223
tr|D3ZQB6|D3ZQB6_RAT	Na(+)/H(+) exchange regul	Slc9a3r2	2	6.4	46.476	419	0	10.608	2.762341835	3.325556565	3.627604186	4.206693415	4.056817096	4.430467269
tr|Q7TP77|Q7TP77_RAT A	Fibrinogen beta chain (Live	Fgb	3	20.5	19.406	166	0	6.5211	1.293914518	1.500162752	1.328195021	1.650905806	1.672301218	1.670445994
sp|P62632|EF1A2_RAT E	Synaptic vesicle membrane	Vat1	5	13.2	50.454	463	0	131.32	57.65261743	57.34111835	64.18221971	41.49313631	52.71186595	48.52531408
sp|P00507|AATM_RAT As	Cysteinyl-tRNA synthetase	Cars2	27	59.1	47.314	430	0	323.31	558.7838405	552.3410812	500.7469559	791.641366	633.0906011	736.7003025
sp|Q794F9|4F2_RAT 4F2	Laminin subunit alpha 4	Lama4	3	9.1	58.071	527	0	6.0406	4.236630103	3.592904298	3.938830366	5.472781394	5.339156746	6.894792144
sp|Q5FVJ3|PLPP7_RAT In	Glutathione S-transferase	Gstk1	2	10.7	29.768	271	0	3.3165	6.896784069	7.674178373	6.648565989	5.695008698	5.624317369	5.688601305
tr|F1LP46|F1LP46_RAT A	Cytochrome b5	Cyb5a	11	19	78.922	695	0	33.261	18.62386869	18.35144086	18.59766195	26.91120565	23.07125418	23.0649352
tr|P97949|P97949_RAT A	Sushi domain containing 2	Susd2	2	4.7	79.123	725	0	4.4992	0.948038684	1.103445542	0.995504967	1.215479183	1.230789622	1.199107676
tr|Q4V8I6|Q4V8I6_RAT 60	Calnexin	Canx	2	13.8	19.024	167	0	39.192	26.27221005	20.81659528	24.76878761	14.96388384	19.2874298	18.92128472
sp|Q920P6|ADA_RAT Ade	Filamin-C (FLN-C) (ABP-28	Flnc	2	7.7	39.899	352	0	2.4436	2.581888345	2.866104672	2.158375804	3.346343881	3.466608529	3.090979511
sp|Q07984|SSRD_RAT Tr	Leucine-rich repeat-contai	Lrrc59	5	36.4	18.979	173	0	100.08	18.18649026	17.14050451	18.20712414	15.33368396	15.80060686	16.98060065
sp|P35280|RAB8A_RAT R	Mitochondrial glutamate ca	Slc25a18	3	16.4	23.668	207	0	15.016	17.43573038	19.13146271	20.68332234	14.75684786	15.03193576	14.86694368
tr|Q52KJ9|Q52KJ9_RAT T	Ras-related protein Rab-1A	Rab1A	2	11.5	31.434	278	0	19.513	1.346543464	1.175026816	1.457397075	0.671369519	0.993842667	1.039633848
tr|D3ZM09|D3ZM09_RAT S	Ubiquinol-cytochrome-c red	Uqcc2	5	13.3	58.286	518	0	9.4183	8.271914619	9.099145116	9.076601348	10.24980035	9.497503846	9.756287431
tr|D3ZXK4|D3ZXK4_RAT A	Succinate-semialdehyde d	Aldh5a1	3	12.7	33.215	307	0	13.629	9.258707345	10.12839346	8.962999866	12.03977184	10.34991103	11.9207584
tr|D4AD70|D4AD70_RAT U	Succinate dehydrogenase	Sdhc	2	31.4	8.2458	70	0	5.9099	25.83164198	21.50339501	20.90162564	15.93786444	17.15608475	15.9155598
sp|Q3KRD5|TOM34_RAT	Pyridine nucleotide-disulfid	Pyroxd2	3	11.3	34.461	309	0	4.92	2.571641649	2.419970224	2.271139672	3.397126292	2.76872059	3.154266034
sp|P09456|KAP0_RAT cA	Thioredoxin-like protein 1 (	Txnl1	6	20.2	43.094	381	0	37.153	15.55823262	13.79021554	14.68233714	11.06188489	13.35459729	12.26875728
sp|P24368|PPIB_RAT Pep	Sarcoglycan, alpha (Sarco	Sgca	12	54.2	23.802	216	0	60.527	88.77147491	89.46945731	79.29592837	59.9883509	75.06809246	72.01215944
sp|P07896|ECHP_RAT Pe	NADH dehydrogenase (Ub	Ndufv3	10	16.1	78.657	722	0	36.155	10.68566943	11.43017382	10.32045905	17.3636783	16.16498967	13.37126704
sp|Q99NA5|IDH3A_RAT Is	Cytochrome c oxidase sub	Cox5b	13	39.3	39.613	366	0	223.24	185.7841642	197.0363728	187.3168029	253.6603142	214.3512956	239.6264915
tr|G3V8B0|G3V8B0_RAT	ADP-ribose glycohydrolase	Macrod1	37	19.7	222.9	1935	0	323.31	392.0258374	201.5119859	376.9789267	482.3460991	751.7276256	596.7821652
tr|F1LX07|F1LX07_RAT S	Dehydrogenase/reductase	Dhrs4	19	40.2	71.916	655	0	323.31	380.7425105	413.4638984	384.5855144	370.8070902	341.3226775	351.2838341
tr|G3V8V6|G3V8V6_RAT	Heat shock protein family A	Hspa12b	8	33.7	27.078	243	0	103.45	27.02735567	21.75024338	26.14038154	36.86108594	55.35151521	46.23355149
tr|Q99PV2|Q99PV2_RAT	Ferrochelatase (EC 4.99.1	Fech	14	25.3	68.02	593	0	51.409	37.24095941	36.34711567	39.45583634	29.37870999	34.86692106	31.6242707

tr|A0A0G2K401|A0A0G2K	Creatine kinase S-type, mi	Ckmt2	4	5.5	79.811	723	0	323.31	163.1297955	175.8854145	182.7203927	255.8652258	203.7992405	234.1704027
sp|P41562|IDHC_RAT Iso	Mitochondrial ribosomal pr	Mrpl19	7	16.7	46.734	414	0	14.089	19.94876252	20.86130385	19.05677946	23.1945408	26.57443182	22.98691775
tr|D3ZDR2|D3ZDR2_RAT (	Metaxin 1	Mtx1	3	19.5	23.288	200	0	14.399	2.102645979	2.121231368	2.539752208	1.660150809	1.667394807	1.892282453
tr|G3V644|G3V644_RAT M	Mitochondrial ribosomal pr	Mrps21	8	33	49.299	458	0	104.59	9.320905189	9.59712248	9.607963118	12.08143946	10.94980158	10.70481538
sp|P13086|SUCA_RAT Su	Adenylosuccinate syntheta	Adssl1	8	27.5	36.148	346	0	56.712	52.52926934	55.06764004	57.86346447	66.11262288	58.75460207	64.42085624
tr|G3V6S3|G3V6S3_RAT (	ADP/ATP translocase 2 (A	Slc25a5	5	23.5	37.063	315	0	22.125	11.42686041	10.87274358	12.46318193	9.583986514	9.604136517	9.534553626
tr|A0A0G2K6H2|A0A0G2K	60S ribosomal protein L6 (	Rpl6	6	41.4	23.862	215	0	90.443	6.488511038	6.958841263	5.233259048	22.82647682	14.11149301	15.41203928
sp|P62260|1433E_RAT 14	Translocon-associated pro	Ssr4	15	54.1	29.174	255	0	119.33	86.35134083	87.58123369	88.14846781	56.46396475	71.80369349	70.75464133
tr|D4A565|D4A565_RAT N	Heat shock 70 kDa protein	Hspa4	7	30.2	21.664	189	0	143.91	194.9703074	224.4845806	214.66492	188.6675086	177.5205017	174.1790898
tr|M0RD20|M0RD20_RAT (	2-oxoisovalerate dehydrog	Bckdhb	3	15.6	25.446	243	0	6.6899	6.167554212	7.658482811	6.807712765	4.854277667	5.855572887	5.332390053
sp|Q498U3|S2540_RAT S	NADH dehydrogenase [ub	Ndufs4	3	8.6	38.061	337	0	3.4001	3.947330385	4.225054932	4.94333969	3.036701383	3.377704357	3.317178788
tr|A0A0G2K913|A0A0G2K	Hydroxymethylglutaryl-CoA	Hmgcl	5	15.2	37.296	329	0	15.315	12.28566911	10.58166225	11.320885	8.905585585	10.2079522	9.748075068
sp|Q71TY3|RS27_RAT 40	Acyl-CoA dehydrogenase	Acad10	3	29.8	9.461	84	0	19.285	11.07002021	8.903664031	11.26277549	6.964568978	8.138100829	7.921850811
tr|A0A0G2JSK5|A0A0G2J	Aminopeptidase N (AP-N) (	Anpep	11	16.4	88.344	798	0	89.84	41.60078909	36.3190539	40.52798304	30.25893845	35.68465627	33.70148531
sp|O35567|PUR9_RAT Bif	Neural cell adhesion molec	Ncam1	12	28.2	64.208	592	0	62.813	25.03702465	23.78686141	24.05315062	32.22295905	27.33983198	30.01464752
sp|Q5XII0|EPDR1_RAT Ma	RCG43947 (Thioredoxin d	Txndc5	2	10.7	25.639	224	0	9.4441	9.23797473	8.58832593	12.1689698	4.883792231	7.39101794	6.075608919
tr|A0A0H2UHP9|A0A0H2U	Collagen type VI alpha 2 c	Col6a2	6	32.7	23.545	208	0	44.575	45.54397296	41.94234556	44.82861057	39.69708829	39.22250589	39.59436915
tr|D3ZFJ6|D3ZFJ6_RAT La	Plasma membrane calcium	Atp2b1	8	18.4	60.42	550	0	65.005	25.15583848	26.66628352	25.99275196	30.63090876	27.56748946	29.12822557
tr|D3ZXF9|D3ZXF9_RAT M	Actin-related protein 3 (Act	Actr3	8	33.7	29.441	276	0	67.798	34.16216611	37.46245178	38.17114492	51.57235984	41.81897874	47.26574288
sp|Q6AY30|SCPDL_RAT S	Vimentin	Vim	6	15.6	47.088	429	0	15.878	22.9956595	23.92098711	23.88458068	22.24313015	21.04850436	19.80821994
tr|A0A0G2JVK4|A0A0G2J	Receptor of activated prote	Rack1	9	15.7	77.801	681	0	95.409	13.96820055	13.36595975	13.78085442	20.53692794	16.48881281	18.59176364
sp|D3ZF77|AK1CA_RAT A	Citramalyl-CoA lyase, mitoc	Clybl	4	11.1	37.194	324	0	26.97	18.56725271	21.15381204	19.83785509	16.68310718	16.66151849	17.101733
tr|A0A0G2K9J1|A0A0G2K	Adenylate kinase 2, mitoch	Ak2	2	5.3	59.692	533	0	35.343	0.850914358	0.776930299	0.566803338	0.972721896	1.135866919	0.982968543
tr|A0A0G2JVG3|A0A0G2J	Hexokinase-2 (EC 2.7.1.1)	Hk2	2	6.7	53.193	489	0	28.272	4.089508278	4.371261466	3.488821915	6.455442753	4.915242812	5.391929686
sp|M0RC99|RAB5A_RAT R	Myosin-11 (Myosin heavy c	Myh11	2	12.1	23.624	215	0	62.876	33.0705142	35.77827046	37.52461114	31.48509496	29.62621963	31.15257309
sp|P14668|ANXA5_RAT A	D-2-hydroxyglutarate dehy	D2hgdh	15	56.4	35.744	319	0	310.55	91.31520727	103.5716816	82.14381806	64.88429618	80.13805077	59.10848383
tr|Q5M7T6|Q5M7T6_RAT V	Neurolysin, mitochondrial (	Nln	5	15.4	40.301	351	0	6.2123	15.6523268	14.25537491	14.88807623	15.9799661	18.15895521	17.91732332
sp|P0C089|PTPM1_RAT P	Alpha-actinin-1 (Alpha-acti	Actn1	7	39.4	21.886	193	0	27.99	14.38524507	13.55478212	14.12794097	15.87796724	14.87689316	14.86745695
tr|A0A1W2Q6E9|A0A1W2	Mitochondrial ribosomal pr	Mrpl50	16	31.1	67.651	576	0	265.26	106.6852515	105.5312487	115.9310975	81.57304585	99.58706505	92.12218372
tr|A0A0G2JSL0|A0A0G2J	Stress-induced-phosphopr	Stip1	2	8.8	25.303	238	0	2.2144	3.47594249	3.029100701	3.722621094	2.439161678	2.964715366	2.667324226
sp|D4A7N1|MIC25_RAT M	Programmed cell death 6-i	Pdcd6ip	5	20.3	29.211	261	0	12.075	11.91487427	12.1426572	14.54151319	10.47549996	9.791888522	9.211705099
tr|G3V778|G3V778_RAT F	Acylpyruvase FAHD1, mito	Fahd1	10	33	46.609	412	0	87.768	27.62541187	22.649171	25.30957254	45.70851048	34.13095937	49.92962818
sp|Q9JLJ3|AL9A1_RAT 4-	Gup1, glycerol uptake/tran	Hhatl	10	23.5	53.652	494	0	57.323	29.95583752	30.40658341	30.96294767	46.69377606	50.34893828	38.4348612
tr|D4AA77|D4AA77_RAT F	Ras-related protein Rab-2A	Rab2a	3	2.2	190.13	1716	0	5.2332	0.759172537	0.941543446	0.833112434	0.613295274	0.703252282	0.454405452
tr|B0BNJ4|B0BNJ4_RAT E	Reticulocalbin-2 (Calcium-b	Rcn2	3	17.3	27.676	254	0	6.2028	4.659655186	4.532783063	3.866533754	5.144648891	4.949914785	5.710158758
tr|D4ACA5|D4ACA5_RAT		Complement factor H	Cfh	3	34.9	18.377	172	0	80.786	14.75085676	15.53575224	13.29084987	19.93361554	16.34423723	18.05128749
tr|D4A6U0|D4A6U0_RAT N	Solute carrier family 25 me	Slc25a46	3	5.1	101.82	904	0	3.5835	1.297183892	1.442279423	1.443262329	1.879166234	1.621405378	1.605670979
tr|D4A478|D4A478_RAT N	Mitochondrial ribosomal pr	Mrpl44	4	29.2	20.813	192	0	4.6916	4.335110023	4.577824569	4.615047113	5.094300517	4.796180565	5.371398778
tr|A0A0G2K266|A0A0G2K	Methyltransferase like 7A,	Mettl7a	3	4.9	83.633	739	0	5.6751	3.776405924	4.052784253	4.249794791	3.152676258	3.563428378	3.662508659
sp|P61589|RHOA_RAT Tr	40S ribosomal protein S4,	Rps4x	2	18.7	21.782	193	0	161.15	55.53948554	55.6336315	54.12560926	42.43630023	49.98193872	41.90050338
tr|Q6IMZ3|Q6IMZ3_RAT A	Maleylacetoacetate isomer	Gstz1	32	53.9	75.755	673	0	323.31	214.1998077	213.4548814	214.8115025	142.807085	174.1972258	180.7695113
tr|A0A0G2JZM2|A0A0G2J	Endoplasmic reticulum resi	Erp29	3	5.6	84.712	751	0	6.9136	3.152433958	3.183297703	2.989498904	3.818620305	3.341527751	3.655528151
tr|Q6AYG2|Q6AYG2_RAT	Integrin beta-1 (Beta oligo	Itgb1	5	17.2	44.507	383	0	6.4571	2.441903325	2.475855936	2.477349919	2.708525476	2.796654425	2.591103231
tr|D3ZPE6|D3ZPE6_RAT M	Mitochondrial ribosomal pr	Mrps9	3	22.7	15.047	128	0	15.138	5.158832759	5.426859327	5.558096465	6.749286278	6.010092132	5.992972014
sp|P04906|GSTP1_RAT G	Laminin subunit gamma 1	Lamc1	2	12.4	23.439	210	0	25.561	5.520457406	5.308904803	4.757232193	6.242330241	6.075772625	5.645999671
tr|F8WG67|F8WG67_RAT	Protein disulfide-isomerase	Pdia6	4	15.9	40.968	372	0	26.274	19.68322557	18.41374749	19.81953227	17.43269029	14.05850376	16.11624942
tr|G3V8A5|G3V8A5_RAT V	Acyl-CoA dehydrogenase f	Acad8	9	14.2	91.726	796	0	31.502	14.21778933	13.84681469	14.78023335	15.88317569	16.53656855	18.32537511
tr|G3V950|G3V950_RAT L	Myomesin 1	Myom1	3	25.4	14.224	122	0	5.5159	4.983801645	5.981911459	5.757553445	7.02837252	6.577862045	6.245502181
tr|F1LSM5|F1LSM5_RAT N	Cofilin 2 (Cofilin 2, muscle (	Cfl2	6	7.5	124.62	1093	0	32.942	5.053574868	5.186669672	5.134472875	5.52443188	5.802976158	5.389876595
tr|D4A5W8|D4A5W8_RAT	NADH dehydrogenase (Ub	Ndufs8	6	15.3	57.755	510	0	10.501	6.20303888	5.846834505	5.930311459	8.104786023	6.824490985	7.258702486
sp|P63018|HSP7C_RAT H	[Pyruvate dehydrogenase	Pdk4	17	35	70.87	646	0	323.31	187.3510715	173.7260857	177.0769642	147.7507744	167.7011373	162.0607215
sp|Q5PQN9|RM38_RAT 39	Creatine kinase B-type (EC	Ckb	5	20.3	44.837	380	0	44.747	10.2391208	11.67179035	9.66711965	14.37315853	11.95201786	12.76406544
tr|Q6IMX8|Q6IMX8_RAT A	Caveolae-associated prote	Cavin4	12	30.2	49.628	453	0	323.31	134.3234219	141.6120154	135.7145076	243.2217075	177.5074179	220.9536308
sp|P27881|HXK2_RAT He	Ras homolog family memb	Rhog	16	23.2	102.54	917	0	95.995	48.2312388	53.17466019	45.87405831	42.20843044	44.24470847	37.94779033
sp|P20760|IGG2A_RAT Ig	Pyruvate carboxylase, mito	Pc	3	11.8	35.185	322	0	9.5921	8.06259495	6.862289778	7.884047543	16.56114259	29.76948684	22.15592925
tr|G3V6R7|G3V6R7_RAT S	Peroxiredoxin-2 (EC 1.11.1	Prdx2	4	9.9	48.004	456	0	31.379	7.213355149	8.428516576	8.53791045	10.89781864	9.09190718	11.48755624
sp|Q99M64|P4K2A_RAT P	Glycine cleavage system H	Gcsh	12	32.4	54.304	478	0	62.124	48.25914809	45.36159987	52.75401533	39.60941268	41.53767783	41.47807995
tr|D3Z946|D3Z946_RAT S	Carbonic anhydrase 14 (M	Ca14	2	7.9	28.399	253	0	2.2982	4.7381999	5.635657858	5.692638312	4.058122309	4.785582717	3.723177492
tr|D4A3E8|D4A3E8_RAT M	39S ribosomal protein L37	Mrpl37	10	29.6	47.648	415	0	42.514	27.36306455	29.59326795	26.56599446	34.99342171	30.13321547	35.23925031
tr|A0A0G2K459|A0A0G2K	Cardiac troponin C (Tropon	Tnnc1	10	40.9	33.757	303	0	323.31	100.6887414	110.3302855	102.6549059	99.15157281	91.86110277	88.56520393
sp|Q9ESN0|NIBAN_RAT P	Carnitine O-palmitoyltransf	Cpt1a	6	10.8	103.46	937	0	31.965	7.241663143	8.462761437	8.299713795	6.339554686	6.826453549	6.893252325
sp|P08050|CXA1_RAT Ga	40S ribosomal protein S5 [	Rps5	11	37.7	43.031	382	0	182.37	91.45076667	102.8772719	120.1034653	73.66921929	73.07281854	70.12331591
sp|B2RZD7|LYRM9_RAT L	60S ribosomal protein L19	Rpl19	3	48.7	9.3787	78	0	4.6733	3.482002793	3.414735893	3.826485306	4.548715125	3.901316568	4.66036211
tr|A0A0G2KA79|A0A0G2K	D-glutamate cyclase	Dglucy	2	14.4	17.043	146	0	4.5176	9.232392872	6.953609409	8.88813806	5.369914458	6.414511259	5.943184563
tr|D3ZD31|D3ZD31_RAT M	T-complex protein 1 subun	Cct5	15	11.5	164.99	1456	0	28.697	28.16485856	29.41015306	27.51511652	34.27205105	42.52811871	44.7086183
sp|P17178|CP27A_RAT S	Annexin A3 (35-alpha calc	Anxa3	8	18	60.732	533	0	12.168	9.527035225	10.55264924	9.694865634	10.80797284	10.71363965	11.5014146
tr|A0A0H2UI21|A0A0H2UI	CDGSH iron-sulfur domain-	Cisd1	21	34.2	71.013	626	0	290.64	175.4258309	178.5108539	175.5535527	225.6562017	194.6406061	217.4993055

sp|P19643|AOFB_RAT An	Guanine nucleotide-bindin	Gnas	5	11.5	58.458	520	0	14.874	9.762669367	9.587610018	8.147372637	34.93482662	52.33374519	27.26504568
tr|Q5VLR5|Q5VLR5_RAT	Ubiquitin-like modifier-activ	Uba1	7	26.1	46.878	406	0	26.205	16.6076219	16.28248047	16.36175444	14.03504316	15.30996573	13.34765649
tr|G3V912|G3V912_RAT	Cytosol aminopeptidase (E	Lap3	2	7.1	37.54	336	0	26.513	8.416245514	6.466571376	8.392374911	5.092564366	5.59219673	5.386796959
sp|P97852|DHB4_RAT Pe	Heat shock protein 75 kDa	Trap1	22	40.5	79.427	735	0	323.31	94.41313837	114.7821176	99.95359876	165.4334704	154.6435414	121.8663365
tr|Q6P725|Q6P725_RAT	Ras-related protein Rab-7a	Rab7a	22	46.9	53.422	469	0	323.31	162.6314153	125.6786423	174.6321766	180.5987477	230.2480682	240.0935696
sp|P28480|TCPA_RAT T-c	Lysosome-associated mem	Lamp1	13	29.1	60.359	556	0	22.223	29.44031308	30.41181527	30.69072292	25.02053741	28.45980213	26.73534826
tr|D4ACC5|D4ACC5_RAT	Erlin-2 (Endoplasmic reticu	Erlin2	6	8.5	104.56	939	0	37.423	7.420681298	6.707236654	7.810232755	6.072187461	6.285701608	6.027361285
sp|Q920F5|DCMC_RAT M	60S acidic ribosomal prote	Rplp2	20	49.8	54.761	492	0	323.31	95.68899159	108.3183999	103.9427384	128.0237608	110.0540758	124.7971236
sp|Q5U2U0|CLPX_RAT AT	Alpha-1-macroglobulin (Alp	A1m	8	16.7	69.207	633	0	30.742	16.10246376	18.50839648	17.46740573	21.51004047	18.84323604	22.47569814
tr|D3ZSA9|D3ZSA9_RAT N	Nidogen-1 (NID-1) (Entacti	Nid1	6	6	133.44	1214	0	11.863	12.54402939	11.98284784	11.53552375	10.18209047	11.45352647	10.67350574
tr|D3ZF16|D3ZF16_RAT L	NADH dehydrogenase [ub	Ndufaf3	2	4	65.986	575	0.0038961	1.4864	6.397207792	6.28631023	9.119005588	3.716361022	3.615436338	5.313912236
sp|Q6AYQ4|TM109_RAT	Cytochrome c oxidase sub	Cox6a2	3	9.1	26.242	243	0	3.5083	5.312732554	5.853968851	6.695158302	4.366853327	4.418975667	4.717078743
sp|P24329|THTR_RAT Th	Aspartate-beta-hydroxylas	Asph	11	43.1	33.406	297	0	68.266	24.74078748	24.99589527	24.01598148	35.1014971	28.38914981	32.44499375
sp|P80067|CATC_RAT Dip	Mitochondrial fission proce	Mtfp1	3	10	52.235	462	0	9.6793	6.557486853	6.215442391	6.131338966	8.602193231	7.080932747	7.937248992
tr|D3ZJH9|D3ZJH9_RAT M	V-type proton ATPase 116	Atp6v0a1	5	12.8	65.351	584	0	18.39	3.93628628	3.930453997	2.894324944	6.715431337	5.604037536	9.030519837
sp|Q9ESS6|BCAM_RAT B	Proteasome subunit alpha	Psma5	11	25.5	67.511	624	0	162.49	37.24574386	32.61537699	43.7067305	24.9098578	30.29414576	26.29239392
sp|Q64244|CD38_RAT AD	Mitochondrial import inner	LOC1009111	4	16.8	34.436	303	0	28.924	4.676002056	4.165982545	3.522274149	2.060333571	3.359125413	2.957117991
tr|A0A0G2JSH4|A0A0G2J	Aquaporin-1 (AQP-1) (Aqu	Aqp1	2	6.7	46.71	420	0	13.838	2.200049398	2.044418241	2.167694267	1.910069718	1.82538125	1.54079331
tr|D4A1Y9|D4A1Y9_RAT S	60S acidic ribosomal prote	Rplp0	4	11.4	42.057	361	0	6.1897	12.22466738	15.76642943	13.57563884	10.63435775	10.93083013	9.500164355
tr|D3ZA85|D3ZA85_RAT H	Zinc-binding alcohol dehyd	Zadh2	3	15.4	28.367	253	0	36.884	9.716818392	10.09938045	10.81308114	14.4695149	11.53660837	13.6258503
tr|B2RYS8|B2RYS8_RAT	Neutral cholesterol ester h	Nceh1	9	50	21.959	186	0	323.31	195.4208431	221.1837565	200.9123351	185.6856696	175.9373663	176.1859861
tr|D3ZM57|D3ZM57_RAT (	Retinol dehydrogenase 14	Rdh14	4	8.5	79.785	681	0	27.396	6.034785736	6.406167245	6.73232745	4.961919018	5.254831891	5.981166742
tr|A0A0G2JXM0|A0A0G2J	Deoxyguanosine kinase (D	Dguok	5	7.4	85.653	755	0	4.9345	6.744479091	7.43113498	8.554662742	4.889868759	6.076099719	6.353289448
tr|A0A0G2JSI0|A0A0G2JS	60S ribosomal protein L23	Rpl23	4	9.6	59.976	531	0	9.4492	5.643656983	6.297725183	5.712531659	6.613432477	7.560452676	8.253424973
tr|M0R959|M0R959_RAT M	Microsomal glutathione S-t	Mgst3	5	25.3	26.233	237	0	26.246	7.695787149	8.111275982	8.198153023	13.20906941	9.96066907	11.33665407
tr|Q5U2U8|Q5U2U8_RAT	Hypoxia up-regulated prote	Hyou1	5	13.2	61.49	574	0	28.925	28.06837216	28.73429267	26.10740046	26.01448375	21.95913429	23.18042156
sp|Q5XIG0|NUDT9_RAT A	Mitochondrial ribosomal pr	Mrpl21	3	8.9	38.562	350	0	3.6407	5.392872084	5.473470388	5.292153825	6.108646628	5.781649624	6.499572167
sp|P15429|ENOB_RAT Be	Unconventional myosin-Ib	Myo1b	6	17.7	47.013	434	0	23.878	25.16819831	21.88912532	23.45373267	19.84376973	21.83876366	18.81657709
sp|P35286|RAB13_RAT R	Cathepsin D (EC 3.4.23.5)	Ctsd	2	12.3	22.901	203	0.0047414	1.4443	1.271307994	1.389485262	1.237313836	1.128194201	1.215743294	1.092398282
sp|Q99P39|NFS1_RAT Cy	Guanine deaminase (Guar	Gda	8	22.6	50.012	451	0	27.54	19.37303376	20.9550016	22.11511981	24.06131409	23.47096315	23.22404974
sp|Q5QD51|AKA12_RAT A	RGD1565784 (RGD15657	RGD1565784	3	2.6	181.11	1687	0	3.7418	1.527315917	1.77483508	1.945202885	1.150113105	1.407747522	1.470885569
tr|G3V9Z3|G3V9Z3_RAT A	Branched-chain-amino-acid	Bcat2	18	35.4	59.52	526	0	323.31	95.00720752	86.70608723	85.91308382	305.254376	481.6983749	257.4729832
sp|P29266|3HIDH_RAT 3-	Evolutionarily conserved si	Ecsit	8	26.9	35.302	335	0	199.23	53.47419813	56.18059804	54.94228351	80.84820289	65.1728422	69.88721047
sp|Q08851|STX5_RAT Sy	Metaxin 2	Mtx2	2	7.9	39.75	355	0	4.4462	5.84699609	4.851355402	7.230708145	3.340484372	3.327331868	3.761518963
tr|D4A6G6|D4A6G6_RAT	Gamma-adducin (Adducin-	Add3	5	33.8	16.117	145	0	18.845	45.57985633	33.85057013	39.1673828	26.0426962	29.57388458	27.53708021
tr|D4A249|D4A249_RAT M	Unc-45 myosin chaperone	Unc45b	2	7.9	31.189	279	0.0055202	1.3349	2.467061555	2.308769548	2.381390695	2.499579726	2.740917593	2.786506147
sp|P04762|CATA_RAT Ca	Adenylyl cyclase-associate	Cap1	21	46.9	59.756	527	0	209.33	54.5905697	72.35178271	61.2243931	109.1300996	98.58615715	75.91816467
tr|D4ABM5|D4ABM5_RAT	Pyruvate dehydrogenase p	Pdpr	6	34.4	25.799	218	0	9.6734	20.82750643	18.62587538	19.92894567	24.29569445	21.18719225	22.66612232
sp|P14925|AMD_RAT Pep	Kynurenine--oxoglutarate t	Kyat3	7	11.2	108.67	976	0	116.24	7.179864002	7.360267141	8.260974119	14.01377531	9.803009721	14.020557
sp|Q9EPF2|MUC18_RAT (	Prostacyclin synthase (EC	Ptgis	7	14.8	71.326	648	0	48.673	40.60801581	31.67031393	30.44938521	20.32989196	26.23883329	25.15138872
sp|P15978|HA11_RAT Cla	Mitochondrial potassium ch	Abcb8	2	6.3	36.57	319	0	17.484	0.598574513	0.157640513	0.506516027	1.37741865	3.313855592	2.634012828
sp|P61983|1433G_RAT 14	Gelsolin (Actin-depolymeriz	Gsn	3	17.8	28.302	247	0	22.134	14.49369259	15.04871421	19.12431214	8.97069124	13.60711393	10.89215991
tr|A0A0G2JSS8|A0A0G2J	T-complex protein 1 subun	Cct2	10	44.1	22.206	213	0	189.17	79.90828206	81.72631359	76.31192632	122.3942918	94.70027942	107.6332846
sp|Q09073|ADT2_RAT AD	Glucose-6-phosphate isom	Gpi	9	25.2	32.901	298	0	101.4	159.6371473	205.8116186	163.8845341	127.346662	125.5910448	117.3033423
sp|Q6P9T8|TBB4B_RAT T	Vitronectin (Vitronectin, iso	Vtn	5	16.2	49.8	445	0	192.03	130.7988774	139.6857419	134.5941981	95.93101306	122.3462715	108.7316882
tr|A0A0G2JVP4|A0A0G2J	Glycerol kinase (GK) (Glyce	Gk	4	10.2	51.708	470	0	5.6691	9.416992885	8.61305833	7.097213316	10.04363244	11.08456434	10.70019592
tr|D3ZE10|D3ZE10_RAT M	Cofilin-1 (Cofilin, non-musc	Cfl1	3	15.4	21.479	188	0	5.5631	7.132418211	8.130776528	8.993886905	6.700240017	6.501125773	5.817946025
tr|F7FLB2|F7FLB2_RAT P	Pitrilysin metallepetidase 1	Pitrm1	2	4.5	69.057	620	0.001773	1.6347	2.801295229	2.434999913	2.672514124	5.125985269	3.897391439	3.679959931
sp|O35987|NSF1C_RAT N	Mitochondrial import inner	Timm23	5	20	40.679	370	0	31.946	14.63323904	15.09627651	16.25234103	13.68434069	13.4095491	13.98770755
sp|Q6IUR5|NENF_RAT Ne	Monoglyceride lipase (MGL	Mgll	6	49.1	18.991	171	0	22.37	22.39640719	18.53788511	20.10693878	15.84541441	16.6222672	17.0416801
sp|Q9ER34|ACON_RAT A	Surfeit locus protein 1	Surf1	46	60.3	85.432	780	0	323.31	567.3958497	606.704799	602.7160648	929.8823743	709.9250016	820.7743703
sp|Q4FZT9|PSMD2_RAT 2	Myosin light chain kinase 3	Mylk3	8	10.2	100.19	908	0	19.626	20.25496729	18.32718409	21.16442723	16.82156521	17.84036557	16.80506138
tr|D3ZAQ0|D3ZAQ0_RAT	Phosphatidylserine lipase	Abhd16a	4	21.2	16.344	151	0	15.463	49.19211576	47.23650605	40.57509886	37.87890436	39.29119565	34.70082725
sp|D3ZAF6|ATPK_RAT AT	Paralemmin-1 (Paralemmin	Palm	2	23.9	10.452	88	0	285.06	215.0370864	239.4714638	218.4603612	212.3138827	188.0463893	189.7261199
sp|P13803|ETFA_RAT Ele	Collagen type XIV alpha 1	Col14a1	18	65.5	34.951	333	0	323.31	214.7540064	222.2824458	223.2504698	326.3789909	256.2716735	303.3236332
tr|D3ZUL3|D3ZUL3_RAT C	Mitochondrial E3 ubiquitin	Mul1	14	19	108.8	1025	0	218.94	41.27783875	44.0184403	46.95772221	49.49331924	57.77070307	62.64493271
tr|Q5U1W6|Q5U1W6_RAT	Tropomodulin-1 (Erythrocy	Tmod1	13	63.8	28.237	254	0	152.76	81.08844629	88.19954369	85.51521687	79.50268601	78.70537868	71.55534674
sp|Q05096|MYO1B_RAT U	Myosin regulatory light cha	Rlc-a	13	12.9	131.92	1136	0	77.699	28.61778646	24.94928421	27.44391928	18.45181082	22.22669725	24.20953332
tr|B1WC61|B1WC61_RAT	Leukocyte surface antigen	Cd47	22	39.8	68.842	625	0	169.01	128.8651624	150.6393414	140.5307917	125.1460908	97.9254271	117.6934295
sp|Q4FZT0|STML2_RAT S	Vesicle-associated membra	Vapb	6	19.8	38.413	353	0	56.656	15.1702935	18.62968036	17.27527788	20.35115981	19.05846394	21.26540112
sp|P50503|F10A1_RAT H	NAD(P)H-hydrate epimeras	Naxe	5	15.2	41.279	368	0	14.727	15.3142257	14.68296006	14.10228902	10.84747027	13.53253648	12.18458056
sp|Q4FZT8|SPRY4_RAT S	Retinol dehydrogenase 13	Rdh13	5	27.1	23.249	207	0	7.8957	11.319609	12.32291834	12.80450988	13.7121191	13.17469555	15.08251821
tr|D3Z9I1|D3Z9I1_RAT Cy	Membrane primary amine o	Aoc3	4	38	11.928	108	0	7.3634	15.36366501	15.39972404	15.0697339	14.6756828	13.6417859	13.35586886
tr|D3ZQ57|D3ZQ57_RAT F	Podocalyxin (Podocalyxin-	Podxl	14	9.8	205.95	1841	0	36.514	27.00383213	24.08365021	27.62348291	21.54910387	23.42516998	23.34928828
sp|O35509|RB11B_RAT F	Coiled-coil domain-contain	Ccdc141	11	53.7	24.488	218	0	323.31	71.01717992	58.71566905	72.85153095	49.93169732	54.41668032	53.05699873
sp|D4ACN8|PLRKT_RAT F	AP-2 complex subunit beta	Ap2b1	4	23.1	17.312	147	0	24.118	81.18812232	89.545557	82.61497628	79.2596249	76.64468594	72.51003396
sp|P47875|CSRP1_RAT C	NADH-cytochrome b5 redu	Cyb5r1	4	30.1	20.613	193	0	39.972	10.04654671	12.76001595	11.30256218	8.441165243	9.541988641	8.552149683

sp|P01946|HBA_RAT Hen	Dystrobrevin beta (DTN-B)	Dtnb	6	59.9	15.328	142	0	323.31	68.35383633	77.70254235	74.82516038	62.80959596	68.0486534	63.861389
tr|B2RZD6|B2RZD6_RAT I	Fatty acid-binding protein,	Fabp4	7	56.1	9.3267	82	0	323.31	373.7372789	415.2046789	376.2146033	367.7297629	320.4737005	325.5586065
tr|F1LZW6|F1LZW6_RAT S	Transcription factor A, mito	Tfam	18	36.8	54.099	484	0	266.99	206.9234573	244.4227001	212.5813536	194.6398674	183.087643	174.7796189
sp|B0BNM1|NNRE_RAT N	Chloride intracellular chann	Clic5	5	24.8	30.89	282	0	70.321	13.08865923	9.905326235	10.02729393	13.16566564	14.33915049	15.31349093
sp|Q68FU3|ETFB_RAT Ele	Staphylococcal nuclease d	Snd1	16	74.9	27.687	255	0	323.31	154.2267322	162.6630929	156.5711115	229.7665387	181.3932957	210.251895
tr|Q4PP99|Q4PP99_RAT	Mitochondrial ribosomal pro	Mrps36	9	60.2	18.42	161	0	86.095	55.6232134	42.24246372	45.913845	61.290464	58.2168594	63.33785085
tr|D4AE99|D4AE99_RAT	Thiosulfate sulfurtransferas	Tst	4	8.3	72.407	636	0	13.163	4.608621057	4.994517948	5.093743864	4.133254236	4.646240637	4.359789619
sp|P06907|MYP0_RAT My	Septin-11	Septin11	4	18.1	27.57	248	0	23.688	11.92205094	10.81043695	9.086548021	18.27342132	34.25132999	32.17501231
tr|G3V6Q6|G3V6Q6_RAT	Mitochondrial ribosomal pro	Mrpl12	4	14.2	42.044	359	0	19.368	30.01045999	26.239174	29.37043289	21.54823579	26.49592924	23.67880935
sp|O88767|PARK7_RAT P	Transmembrane protein 12	Tmem126a	5	38.1	19.974	189	0	132.24	19.52733225	16.97403643	16.60675672	20.86592852	21.21663071	20.62381025
tr|A0A0G2K747|A0A0G2K	Sqrdl protein (Sulfide quino	Sqor	3	14.2	17.17	148	0	2.3873	12.35504363	10.95407512	10.3979384	18.13322714	14.37643921	13.93176082
tr|Q6TXE9|Q6TXE9_RAT	Enoyl-CoA delta isomerase	Eci1	6	5.7	166.78	1486	0	28.51	15.77552638	15.0506167	15.27913756	13.377042	14.81147435	14.01799064
tr|A0A0G2KA25|A0A0G2K	Thioredoxin, mitochondrial	Txn2	5	9.6	74.728	643	0	4.5121	7.977272266	8.787136377	8.774013069	6.1555227	7.325599123	7.703196642
tr|A0A0G2JSP8|A0A0G2J	Peptidyl-prolyl cis-trans iso	Ppif	14	42.5	43.018	381	0	323.31	121.8120863	97.40760649	103.3825836	71.0215893	95.6030591	79.80877171
tr|F1LSW7|F1LSW7_RAT	Protein LYRIC (Lysine-rich	Mtdh	4	20.1	23.322	214	0	16.191	38.45222257	32.54022854	28.57888708	22.20233061	23.48666366	23.89541043
sp|P42123|LDHB_RAT L-l	Beta-2-glycoprotein 1 (Apo	Apoh	15	39.8	36.612	334	0	323.31	246.7779223	211.3573837	225.2869203	161.0323281	210.2429939	166.6801757
sp|P11980|KPYM_RAT Py	[Pyruvate dehydrogenase	Pdp1	7	15.1	57.817	531	0	323.31	197.05553	171.5952943	167.5438629	131.9387809	164.4759896	140.5032682
sp|P23693|TNNI3_RAT Tr	Galectin-1 (Gal-1) (14 kDa	Lgals1	8	44.5	24.159	211	0	164.04	26.87824033	25.88388356	30.96556522	32.34882998	40.74872689	44.29338069
sp|Q6NYB7|RAB1A_RAT	Phosphatidate cytidylyltran	Cds2	4	32.2	22.677	205	0	107.46	26.48152972	24.97972409	26.28225251	22.29825294	24.59878355	21.04520714
tr|B0BNK1|B0BNK1_RAT	Mitochondrial ribosomal pro	Mrps30	2	15.3	23.425	216	0	43.667	6.715373689	6.591184623	6.541246617	7.66901217	8.919201504	7.659568463
sp|P52287|GBB3_RAT Gu	Catenin (Cadherin associa	Ctnnd1	9	34.7	37.18	340	0	281.62	89.28181619	70.8821074	80.13877808	59.70622639	71.64014645	58.23592024
sp|Q1JU68|EIF3A_RAT Eu	Cytochrome c oxidase ass	Cox20	2	2	163.19	1354	0	3.3239	2.410844273	2.634951855	2.967145064	2.939433534	3.35709743	3.239828593
sp|Q1HCL7|NAKD2_RAT	Ion channel TACAN (Trans	Tmem120a	8	24.5	48.116	425	0	11.742	13.21624456	15.38926033	13.40549837	21.65066869	16.25984695	22.16311507
tr|B2GV57|B2GV57_RAT	Ras-related protein Rab-18	Rab18	11	27.6	60.917	550	0	109.97	18.53974212	19.00922758	20.87387966	23.65679095	21.09756847	25.56252015
tr|G3V7J0|G3V7J0_RAT A	Hepatitis B virus x interacti	Lamtor5	25	53.8	57.747	535	0	323.31	212.4255743	245.6307827	233.5531295	345.59384	264.0172615	303.7958441
tr|A0A0G2JVE2|A0A0G2J	Polyadenylate-binding prot	Pabpc4	2	18.2	14.832	137	0	2.1898	2.037298372	2.306486557	2.112516404	1.996182799	1.801895894	1.916868216
sp|P29314|RS9_RAT 40S	NADH dehydrogenase [ub	Ndufaf4	4	19.6	22.591	194	0	5.3552	34.99824867	26.71194334	29.76411176	20.77955502	26.32911126	22.07380562
sp|Q4QQV3|F162A_RAT	Alpha glucosidase 2 alpha	Ganab	7	32.3	17.827	155	0	141.75	61.64364579	74.58245495	71.02971916	83.74757474	77.61288444	81.60009343
sp|P04692|TPM1_RAT Tro	Myosin light polypeptide 6	Myl6	9	29.9	32.68	284	0	206.81	123.6341642	87.99502577	110.1044409	125.1677927	141.0429694	152.6575656
tr|F1M5Z5|F1M5Z5_RAT A	Lactamase, beta (Lactama	Lactb	4	7.5	92.226	799	0	37.954	6.762420776	5.934824775	7.098260334	4.543072635	6.057520775	5.043366197
sp|Q63610|TPM3_RAT Tro	Solute carrier family 2, faci	Slc2a4	4	14.1	29.006	248	0	6.1569	6.199450543	6.301054545	5.425648654	4.710611188	5.567402998	4.916587841
sp|P82995|HS90A_RAT H	Myristoylated alanine-rich C	Marcks	11	15.8	84.814	733	0	61.28	35.52932258	33.05295022	33.2056608	23.78526611	31.10664746	27.03048006
sp|P52555|ERP29_RAT E	Caveolae-associated prote	Cavin2	3	14.2	28.574	260	0	90.218	20.56515911	20.83276646	19.17247498	17.88756181	16.39788066	19.12608052
tr|M0RCH6|M0RCH6_RAT	Mitochondrial ribosomal pro	Mrps22	2	13.5	24.812	222	0	19.361	3.999799848	5.003079164	4.6773447	3.401813899	3.97916496	3.625604352
tr|A0A0G2K2G8|A0A0G2K	Integrin alpha 5 (Mapped)	Itga5	4	18.3	23.754	208	0	32.78	9.036629143	11.47012616	9.698006689	7.540971047	7.50877181	8.316044243
sp|Q4V8F9|HSDL2_RAT H	39S ribosomal protein L41	Mrpl41	19	35.7	58.343	524	0	323.31	105.377502	109.4979452	108.968426	158.0244468	125.2246994	137.7469938
tr|A0A0G2K6J5|A0A0G2K	NADH dehydrogenase [ub	Ndufc2	7	50.3	16.961	151	0	65.386	35.05167503	27.04202576	33.95899044	18.99739621	27.27768411	24.66788583
tr|A0A0G2K943|A0A0G2K	Iron-sulfur cluster assembly	Iba57	13	19.1	101.58	915	0	47.165	48.28307034	45.91427389	43.7182477	48.16950425	52.56925293	54.76619681
sp|Q5RJY4|DRS7B_RAT D	NADH:ubiquinone oxidored	Ndufb3	2	8.6	35.342	325	0	4.6352	2.058110727	2.136831805	2.087754422	1.983639109	1.874249106	1.732398008
sp|P11951|CX6C2_RAT C	Ras-related protein Rab-5A	Rab5a	6	63.2	8.4548	76	0	27.843	490.2864707	531.2234165	470.8598195	450.3575204	439.666785	432.1140184
tr|G3V9N1|G3V9N1_RAT	Bifunctional purine biosynt	Atic	3	11.5	32	288	0.0013599	1.7906	2.508965359	2.411694382	2.40290692	1.911979484	2.261135989	1.79778895
tr|D3ZXF8|D3ZXF8_RAT M	GrpE protein homolog 1, m	Grpel1	2	13.5	17.575	156	0	4.0922	1.592783135	1.842373557	1.871702203	2.109727062	2.01391828	2.492760182
sp|Q3B7U9|FKBP8_RAT F	Enoyl CoA hydratase dom	Echdc2	5	18.9	43.555	403	0	49.635	12.07794426	13.13100195	12.06322095	14.37966909	13.10665998	13.47084194
sp|Q4V7F2|CREL1_RAT C	Phosphatidylinositol 4-kina	Pi4k2a	3	9	45.696	420	0	5.9097	9.605181235	7.405926957	8.373528582	11.8908969	9.502083163	10.67093938
tr|D3ZAN3|D3ZAN3_RAT A	Phosphoglycerate mutase	Pgam2	11	15.3	90.571	797	0	65.676	25.12952401	28.3062319	25.99798705	21.49094281	22.7605148	25.08722963
sp|P15650|ACADL_RAT L	Heat shock protein HSP 90	Hsp90aa1	23	50.5	47.872	430	0	323.31	421.1910446	445.1118581	445.0141736	617.3318246	488.436513	595.1396926
sp|Q66HA6|ARL8B_RAT A	CDGSH iron sulfur domain	Cisd2	3	13.4	21.539	186	0	21.826	38.69463468	33.96709778	40.35627204	30.52370144	32.80819089	31.89168578
sp|P11960|ODBA_RAT 2-	Long-chain fatty acid trans	Slc27a1	18	58	50.164	441	0	323.31	81.66656728	74.85356011	86.36330167	115.6016017	90.0620853	103.8555976
sp|P35738|ODBB_RAT 2-	Peptidyl-prolyl cis-trans iso	Ppib	8	25.1	42.823	390	0	99.944	36.65765527	35.19040034	37.13616737	52.79200578	41.34272975	47.42947687
tr|D3ZIF5|D3ZIF5_RAT Ad	Glycerol-3-phosphate dehy	Gpd1l	5	8.1	87.764	794	0	11.811	6.186293306	6.341482507	6.856399115	5.311319368	6.145509084	5.671663306
tr|Q9WVJ6|Q9WVJ6_RAT	GTP-binding protein RAD (	Rrad	15	27.4	76.934	686	0	159.64	47.2464396	58.30187697	54.81664132	42.40808778	44.22900796	42.01906937
sp|Q5FVN0|MBOA5_RAT	Endoglin	Eng	6	14.6	56.017	487	0	21.391	23.8839723	22.83133464	22.67789213	19.79689366	20.87121936	22.2960527
tr|F1M4A0|F1M4A0_RAT	60S ribosomal protein L10	Rpl10a	7	6.2	194.03	1733	0	11.062	4.184001157	4.413116297	4.991136074	3.379200535	4.184056695	3.647418442
sp|Q6P6V0|G6PI_RAT Glu	Dihydropyrimidinase-relate	Dpysl3	11	28.5	62.826	558	0	74.913	49.83004237	45.06909168	46.47609381	35.81462104	44.4645157	38.42872685
sp|Q5U2V4|PLBL1_RAT P	Nucleoside diphosphate-lin	Nudt19	7	16.2	63.027	550	0	53.959	20.96466065	19.95809562	20.37549897	21.34380403	22.57995886	23.80866734
tr|D3ZH23|D3ZH23_RAT S	RCG43475 (Translocase o	Tomm6	3	29.9	15.194	134	0	63.823	10.46717957	11.61899619	10.27857832	13.79805857	12.90974934	11.52040569
tr|A0A0G2JYK4|A0A0G2J	Glutaredoxin 5 (Glutaredox	Glrx5	6	21	38.434	338	0	40.28	11.38938222	11.66370476	11.45123878	14.95346693	13.02488646	12.9621887
sp|Q06000|LIPL_RAT Lipo	Mitochondrial import inner	Timm44	9	22.4	53.082	474	0	41.906	21.54716739	23.21706496	25.48861267	18.99088565	21.22971448	19.9837592
tr|D3ZCV0|D3ZCV0_RAT A	Probable proline--tRNA liga	Pars2	24	33.9	103.83	894	0	323.31	133.2389467	107.0675112	137.2326841	139.7948633	161.8069019	176.6787279
sp|Q64560|TPP2_RAT Tri	Mitofusin-1 (EC 3.6.5.-) (Mi	Mfn1	8	6.8	138.29	1249	0	9.4477	11.39934983	10.8237544	12.73854773	8.633443945	10.561868	10.08272887
sp|P50115|S10A8_RAT P	Guanine nucleotide-bindin	Gnao1	2	32.6	10.238	89	0	4.6359	2.06995224	1.567891479	1.477342773	2.564858997	2.299536835	3.518279031
sp|Q8VID1|DHRS4_RAT D	Acidic leucine-rich nuclear	Anp32a	8	34.1	29.821	279	0	103.42	53.0077143	55.88571173	56.64892328	62.54917334	57.84462633	58.43096387
tr|A0A0U1RVI9|A0A0U1RV	Chaperonin subunit 8 (The	Cct8	4	17.2	28.589	267	0	41.919	46.26164039	31.43583176	38.92499807	23.83257622	27.71664437	24.931708
tr|Q6P503|Q6P503_RAT A	Sulfite oxidase, mitochond	Suox	2	10.9	28.309	247	0	14.114	2.803009657	3.024439594	3.059806181	2.633133127	2.25779963	2.731021368
sp|Q6RUV5|RAC1_RAT R	Glutathione S-transferase	Gstm2	7	35.4	21.45	192	0	54.023	57.80013796	58.02125936	59.71145171	48.35180008	55.83299771	49.52722239
tr|A0A0U1RRQ7|A0A0U1R	Mitochondrial ribosomal pro	Mrps23	2	5.1	44.749	410	0.0038978	1.4874	2.046388826	2.365083321	2.917673451	1.613578564	1.721103655	1.552136637
sp|P01161|ANF_RAT Natr	Septin-8	Septin8	2	11.2	16.556	152	0	2.5599	5.734561525	6.295822691	6.339695601	24.34777897	11.92061683	25.86689086

tr|D3ZDX7|D3ZDX7_RAT M	Adenylyl cyclase-associate	Cap2	4	19.4	24.019	211	0	4.3872	6.659555111	6.126025252	5.906753548	8.170325716	6.698232668	7.570772286
tr|A0A0H2UHY3|A0A0H2U	4-trimethylaminobutyraldeh	Aldh9a1	5	26.5	31.672	287	0	36.795	4.583502697	4.183533037	3.806330204	4.994037808	5.759276388	4.671346146
tr|G3V9W2|G3V9W2_RAT	Inter-alpha-trypsin inhibitor	Itih4	6	6.5	133.26	1153	0	8.0161	8.381159551	9.656099741	9.390706828	7.454597544	8.65621786	7.340312845
sp|Q498R3|DJC10_RAT D	Dynactin subunit 1 (150 kD	Dctn1	2	3.3	90.746	793	0	26.124	0.823443643	0.859926526	0.885096891	0.955403791	0.990767983	1.141980424
sp|P52481|CAP2_RAT Ad	Succinate--CoA ligase [AD	Suclg1	9	26.4	52.912	477	0	57.421	41.92373944	35.18421724	37.66700563	28.09916684	35.07887803	32.11701249
tr|Q6IRH7|Q6IRH7_RAT C	Stomatin-like protein 2, mit	Stoml2	9	18	75.678	677	0	22.09	8.886317686	8.796648838	8.997551469	7.794883103	8.607807935	7.918771175
sp|P12075|COX5B_RAT C	Fatty acid-binding protein,	Fabp3	8	42.6	13.915	129	0	103.68	603.6779257	697.5012447	661.192035	586.8189741	557.0150588	571.0158758
sp|Q920J4|TXNL1_RAT T	E3 ubiquitin-protein ligase	Nedd4	3	19	32.249	289	0	105.46	3.259725239	2.567698752	3.07640142	2.503920103	2.518035683	2.074750897
sp|Q6AXX6|PXL2A_RAT I	Integrin subunit alpha 6 (In	Itga6	3	15.3	25.763	229	0	6.0901	2.711068484	2.93140772	3.082317073	4.67805836	4.961297659	7.420896658
tr|Q641Z9|Q641Z9_RAT S	Acid ceramidase (AC) (ACD	Asah1	4	29	18.202	169	0	106.27	48.39869454	53.39820303	53.58115976	47.51410732	44.48152459	48.53763263
sp|Q6P502|TCPG_RAT T-	RCG25684, isoform CRA_	Tmod3	11	25.5	60.646	545	0	55.225	34.48073071	34.3033633	32.53033402	29.75936105	31.6574739	32.58255083
sp|P31430|DPEP1_RAT D	NADH dehydrogenase (ub	Ndufaf6	2	5.1	45.522	410	0	10.671	2.971502023	2.660730626	3.687912438	2.068580287	2.338853544	2.132750712
tr|B0BMW2|B0BMW2_RAT	Coiled-coil domain-contain	Ccdc90b	15	57.9	27.249	261	0	323.31	110.2257442	120.4420322	132.295993	167.5255321	134.6908022	144.6248479
tr|F1LPK7|F1LPK7_RAT P	Calcium/calmodulin-depen	Camk2d	10	22.5	70.748	630	0	120.16	43.62620608	44.08312504	39.26109094	27.81704233	38.93335472	32.32488793
sp|P97576|GRPE1_RAT C	Transmembrane emp24 do	Tmed10	7	39.2	24.297	217	0	62.586	27.69199546	30.07317164	28.51292493	42.40070914	32.36334294	38.38047922
tr|M0R402|M0R402_RAT T	T-complex protein 1 subun	Cct3	5	12.6	52.1	459	0	8.7122	7.551057549	8.433272806	9.687536506	6.069583235	7.588582767	6.629943432
sp|Q641Y2|NDUS2_RAT M	Alpha-centractin (Centracti	Actr1a	26	64.8	52.561	463	0	323.31	921.4052482	1006.418434	901.1162706	899.4997351	821.5295033	820.8256976
sp|Q5FVL2|EMC8_RAT EI	Laminin subunit beta 1	Lamb1	5	36.7	23.405	207	0	40.26	12.8729603	14.3828419	12.97255632	11.22985748	10.34729428	12.70606562
tr|D4AAU8|D4AAU8_RAT	Endonuclease (EC 3.1.30.	Endog	8	31.9	31.092	273	0	17.683	14.62845459	15.89722578	14.45251663	11.77370672	14.3162539	12.22564238
tr|F2Z3T8|F2Z3T8_RAT G	Pentatricopeptide repeat d	Ptcd3	3	68.7	7.1872	67	0	38.698	7.19700828	6.791897562	7.726994803	6.576105234	6.363222906	5.244107149
sp|P54313|GBB2_RAT Gu	Ubiquinone biosynthesis C	Coq3	4	18.8	37.331	340	0	323.31	32.61798501	32.17209628	32.6727285	26.26969792	30.57544667	28.84027959
sp|P10252|CD48_RAT CD	Ras-related C3 botulinum t	Rac1	7	30.4	27.679	240	0	46.043	16.60682449	14.16976276	14.28656424	16.00166799	18.31530618	18.07592458
sp|Q5I0K5|ABHDA_RAT M	Phospholipase B-like 1 (EC	Plbd1	5	18.9	33.152	297	0	6.7618	6.541139983	8.11555659	7.387237376	8.203312582	8.445569269	9.488359083
tr|B5DF65|B5DF65_RAT B	Troponin T, cardiac muscle	Tnnt2	2	16.5	22.094	206	0	45.075	1.718335066	1.667486951	1.830920842	2.011721348	2.812027847	2.740927531
tr|F1M9C9|F1M9C9_RAT H	Cytochrome c oxidase sub	Cox7a2l	8	18.1	57.327	507	0	23.262	23.79426387	25.76212405	26.17126858	31.96514066	26.75171682	29.82422335
tr|G3V7L8|G3V7L8_RAT A	60S ribosomal protein L8	Rpl8	3	13.7	26.143	226	0	3.8281	5.738548566	5.777869159	6.329748927	3.941539782	5.397510331	4.879632206
sp|P08082|CLCB_RAT Cla	ATP synthase mitochondri	Atpaf1	3	12.2	25.117	229	0	2.4634	4.124195538	4.153188284	4.955118646	3.280500361	3.927549514	3.707676657
tr|A0A0G2JYK0|A0A0G2J	LIM domain 7	Lmo7	2	5.4	67.982	609	0	10.121	6.349762	5.522459565	5.310476645	5.176333643	4.64604438	4.69608589
sp|Q99MA2|XPP2_RAT X	Rho GDP-dissociation inhi	Arhgdia	6	11.4	76.079	674	0	29.16	14.29473923	15.81208925	15.13988413	18.87673373	22.70948812	17.49233353
sp|P45592|COF1_RAT Co	Armadillo repeat-containing	Armc10	7	42.8	18.532	166	0	73.915	30.155987	34.20966555	29.50078666	24.6116739	27.11152031	28.99374813
sp|P11505|AT2B1_RAT P	Annexin A1 (Annexin I) (An	Anxa1	9	10.8	134.69	1220	0	97.686	48.2990185	47.95707501	48.59839983	36.50864733	45.18085174	37.37138509
tr|A0A0H2UHG7|A0A0H2U	Mitogen-activated protein k	Map3k20	3	22.9	13.241	118	0	7.6037	7.619634659	6.18595376	6.91346161	5.482330223	5.903066948	5.69527385
tr|A0A0G2K6J0|A0A0G2K	Actin, alpha skeletal muscl	Acta1	5	16.4	43.785	391	0	6.1085	15.71173371	12.31007652	12.52129144	15.2177959	16.60525831	18.39466693
tr|B1H282|B1H282_RAT C	Methylglutaconyl-CoA hydr	Auh	5	10.5	71.114	617	0	11.855	6.628057484	7.789279158	7.144329138	6.229743147	6.024222597	6.382545992
sp|Q5M7V7|FAKD2_RAT I	Monocarboxylate transport	Slc16a1	4	7.7	77.767	679	0	5.8708	2.80627903	2.389958408	2.274280726	2.865343298	2.794103091	3.198869432
sp|P63039|CH60_RAT 60	Aldehyde dehydrogenase	Aldh3a2	33	67.2	60.955	573	0	323.31	315.1556808	343.6757241	315.1786735	389.6313054	338.4507914	393.5569734
tr|D4A4P3|D4A4P3_RAT N	Very-long-chain enoyl-CoA	Tecr	4	43.4	11.267	99	0	62.945	212.8163043	246.9102088	219.6330217	205.1218779	197.2377331	189.5002799
sp|B1WBW4|ARM10_RAT	Actin-related protein 2/3 co	Arpc1b	5	21.9	33.421	306	0	52.774	10.07405729	9.170012955	9.92049807	9.010622709	8.807335327	8.862166393
tr|A0A0G2JTY6|A0A0G2J	Syntaxin binding protein 3	Stxbp3	4	15	43.549	381	0	12.782	5.582256547	5.380248265	6.184213388	6.978892222	6.041689421	6.544226891
sp|Q5XIF3|NDUS4_RAT N	26S proteasome non-ATP	Psmd1	8	37.7	19.74	175	0	99.478	138.3583078	178.3015797	175.7681914	137.1125103	124.4396736	121.2863384
tr|A0A0G2JSH5|A0A0G2J	28S ribosomal protein S7,	Mrps7	27	42.9	68.758	608	0	323.31	228.8402234	197.3597965	311.8334502	150.3419795	184.3306005	120.8500566
sp|Q62636|RAP1B_RAT R	Histone H4 [Cleaved into:	H4c2	2	15.2	20.798	184	0	9.2281	17.27903966	16.01470468	14.93100398	13.04934353	14.80100734	14.52869697
sp|Q9JHU5|ARFP1_RAT A	Mitochondrial ribosomal pro	Mrpl47	2	7.1	40.779	366	0	8.9084	16.90226425	24.58067632	9.005927615	25.84564308	26.96367378	34.88765851
sp|Q6P7Q4|LGUL_RAT La	40S ribosomal protein S3 (	Rps3	2	14.1	20.819	184	0	5.6466	1.193441077	0.889748093	0.878553027	1.35658484	1.131810952	1.57821089
sp|P06686|AT1A2_RAT S	Nitric oxide synthase, endo	Nos3	23	27.2	112.22	1020	0	323.31	145.6825027	101.2696659	101.8801124	64.56744866	82.62396581	77.47851367
tr|G3V945|G3V945_RAT A	NADH:ubiquinone oxidored	Ndufa7	12	27.5	56.145	523	0	106.8	46.90355404	47.94280632	47.27758629	61.7635651	50.89845634	59.33945654
sp|P01835|KACB_RAT Ig	14-3-3 protein beta/alpha	Ywhab	2	28.3	11.601	106	0	12.055	2.957268285	2.700159779	2.278416449	2.997420971	4.439582594	4.286186314
tr|D4AE56|D4AE56_RAT F	Peptide deformylase (EC 3	Pdf	12	40.6	43.451	384	0	154.48	52.93594756	59.08189882	56.25105634	52.55762542	51.50488878	49.62063802
tr|F1LQ09|F1LQ09_RAT A	Ceruloplasmin (EC 1.16.3.	Cp	4	9.1	66.194	583	0	9.7438	4.414850849	4.247551903	5.170071496	4.927196001	5.724669834	5.979113651
tr|Q68FZ8|Q68FZ8_RAT F	Guanine nucleotide-bindin	Gng12	4	10	58.678	541	0	323.31	179.8195504	197.6023642	195.117089	278.3223365	209.8243135	262.4927902
sp|P36860|RALB_RAT Ra	Alkyldihydroxyacetonepho	Agps	5	19.9	23.317	206	0	18.698	27.31880839	22.20541467	26.86753572	18.07115975	23.20209181	20.63150934
sp|Q9WVK7|HCDH_RAT H	UDP-glucose:glycoprotein	Uggt1	11	41.7	34.447	314	0	323.31	97.96160514	116.6846099	88.67721204	132.9457483	114.1754612	123.8578346
tr|G3V7Y3|G3V7Y3_RAT	Peptidyl-prolyl cis-trans iso	Fkbp8	4	37.5	17.563	168	0	263.2	66.13305431	97.09845149	94.48816341	60.04911617	57.63921124	53.16991872
tr|G3V6V1|G3V6V1_RAT	Reticulocalbin 1 (Reticuloc	Rcn1	3	6.8	72.688	650	0	3.7386	5.340243139	6.132208352	6.165367059	4.812610048	5.581598881	4.577930515
sp|Q5FVQ8|NLRX1_RAT I	Multidrug resistance-assoc	Abcc1	21	26.3	107.59	973	0	323.31	71.53948234	81.35532759	75.94546992	86.38652398	86.42479908	79.88576262
sp|P16970|ABCD3_RAT A	Antisecretory factor (Protea	Psmd4	6	12	75.315	659	0	38.242	21.11537081	22.25868445	20.61997773	22.27177664	23.84057946	22.43155669
sp|Q6MG60|DDAH2_RAT	Prolow-density lipoprotein	Lrp1	3	16.1	29.688	285	0	4.3368	3.146014821	3.2273404	2.803705513	3.457761359	3.235353011	3.575303628
sp|Q68FR6|EF1G_RAT El	Adenylate kinase 4, mitoch	Ak4	7	21.7	50.06	437	0	16.615	11.70236497	11.72553576	12.69509648	8.911662113	11.38745347	9.944145239
tr|A0A0G2K4C6|A0A0G2K	Snta1 protein (Syntrophin,	Snta1	10	20.8	63.436	571	0	323.31	23.43503145	25.55950862	26.85235396	40.00742525	29.50781158	32.59846228
tr|D3ZJ54|D3ZJ54_RAT 40	Protein disulfide-isomerase	Pdia4	2	25	12.093	108	0	33.332	10.3356072	8.22066929	9.450386869	7.290965331	7.581386697	7.969071899
tr|D3ZCI0|D3ZCI0_RAT Gl	Cathepsin B (EC 3.4.22.1)	Ctsb	10	24.6	57.462	524	0	74.195	19.99341738	19.10577907	18.8238179	21.41194795	19.95928105	20.27632464
tr|F1LMM8|F1LMM8_RAT	40S ribosomal protein S6	Rps6	11	36.1	46.239	407	0	323.31	71.18463566	70.64905209	73.29127862	87.71901972	75.55873358	82.13389703
tr|D3ZZZ9|D3ZZZ9_RAT C	Cell surface glycoprotein M	Mcam	16	23.7	104.07	932	0	66.785	20.79640751	20.08318449	20.47077763	19.02040021	19.94161797	19.91344084
tr|F1LM19|F1LM19_RAT U	Calcium-independent phos	Pnpla8	2	6.8	38.346	352	0	14.065	12.26972095	11.54432336	11.47846125	7.458503884	10.84251472	8.345814059
sp|B4F7A1|LYRM7_RAT C	Coatomer subunit beta (Be	Copb1	3	34.6	12.03	104	0	15.869	3.84940865	3.774116691	3.783871662	4.420239965	4.801152395	5.777910754
sp|P14562|LAMP1_RAT L	Iron-sulfur cluster assembly	Isca2	6	13.8	43.969	407	0	83.106	32.00398065	25.86390739	37.93294827	18.92795018	24.89840173	23.93133951
tr|G3V741|G3V741_RAT F	Serine hydroxymethyltrans	Shmt2	15	38.5	39.531	356	0	323.31	1225.656371	1508.866654	1276.891126	1148.810992	1093.410107	1046.56303

tr|Q5XFV4|Q5XFV4_RAT	40S ribosomal protein S8	Rps8	7	50.8	14.736	132	0	69.038	142.915496	158.3777289	138.6670991	136.027416	131.0665998	113.0893734
sp|P0DN35|NDUB1_RAT N	Dehydrogenase/reductase	Dhrs7	2	31.6	6.9981	57	0	10.853	46.49687583	51.0914311	45.49137313	45.60434143	42.13626	40.01371294
sp|Q5M818|RM16_RAT 39	MAGUK p55 subfamily me	Mpp7	2	10.4	28.895	251	0	9.3895	12.81275597	13.2865307	15.0524581	16.09628821	15.64687264	14.87926222
tr|F7F469|F7F469_RAT In	Acyl-CoA synthetase family	Acsf3	3	5.9	157.13	1388	0	15.168	13.95225238	13.5771364	12.47208158	16.23691642	15.11370928	19.50128286
tr|A0A0G2K013|A0A0G2K	60S acidic ribosomal prote	Rplp1	22	32.7	102.97	892	0	172.4	91.72985956	98.90581919	98.69717684	80.99143533	91.9919404	89.35051116
tr|F1M6Q3|F1M6Q3_RAT	AP-2 complex subunit alph	Ap2a2	3	2.2	160.76	1645	0	14.216	12.17004491	15.91101885	15.18542942	10.10135946	11.19446796	12.73070271
tr|A0A0G2K673|A0A0G2K	Cytochrome b-c1 complex	Uqcrq	3	9.8	42.88	379	0	2.7928	1.411093663	1.39975872	1.374577931	1.540703633	1.61852695	1.857636546
sp|P10888|COX41_RAT C	3-ketoacyl-CoA thiolase A,	Acaa1a	17	63.3	19.514	169	0	323.31	654.751925	763.7079773	684.4881914	629.7018991	617.5732577	589.8016565
sp|P0C5H9|MANF_RAT M	Caveolae-associated prote	Cavin3	7	34.1	20.388	179	0	31.777	16.87674719	16.84466695	17.28836561	12.34359823	13.93159126	15.87655107
tr|Q4KLL7|Q4KLL7_RAT V	Serine protease inhibitor A	Serpina3n	5	12.4	49.451	444	0	7.1267	14.56346582	15.49104367	14.50277351	12.91869818	14.5046601	13.24910814
tr|F1M8Y4|F1M8Y4_RAT D	Fatty acid-binding protein 5	Fabp5	2	6.6	46.226	409	0	2.5653	12.26214557	13.14384378	12.19148069	11.82231895	11.49016101	11.76780313
sp|Q920A6|RISC_RAT Re	Reticulon-4 (Foocen) (Glut	Rtn4	3	7.3	51.174	452	0	4.6363	7.202988842	7.918648635	8.57403258	8.389514756	9.461523496	9.143439831
sp|Q4KLZ1|TM186_RAT T	Ezrin (Cytovillin) (Villin-2) (p	Ezr	2	14.4	24.64	216	0	11.384	7.00204196	8.070372397	6.60563824	6.596505006	5.147348774	6.038140012
sp|Q6AYE2|SHLB1_RAT E	Diablo homolog (Drosophil	Diablo	6	16.7	40.787	365	0	19.416	9.315722035	8.599265261	8.969805485	8.487173239	8.550239376	7.787886637
tr|D3ZFC6|D3ZFC6_RAT In	Aspartate--tRNA ligase, cy	Dars1	10	13.4	103.75	932	0	57.32	22.87724437	18.45274858	18.54112296	23.29219928	23.60703429	29.64714427
tr|B5DFN1|B5DFN1_RAT E	CD48 antigen (BCM1 surfa	Cd48	2	26.5	12.823	113	0	8.463	2.062855307	2.184917298	1.719256344	2.304089145	2.688386283	2.237099051
tr|D4A3V2|D4A3V2_RAT N	Fibrinogen gamma chain	Fgg	9	58.5	15.224	130	0	135.02	80.71366441	95.60975126	89.60905829	80.46190933	78.26053072	72.97197938
tr|D3ZD23|D3ZD23_RAT A	Proteasome (Prosome, ma	Psmd12	4	9.7	67.3	599	0	7.5965	8.194566017	7.243263864	6.927072848	6.140331381	6.984767086	6.155679459
tr|M0R3V7|M0R3V7_RAT	Annexin A4 (36 kDa zymog	Anxa4	15	21.1	107.45	965	0	323.31	67.97108036	53.85480119	72.55313074	48.38218272	54.22107806	46.76684182
sp|Q7TQ16|QCR8_RAT C	Radixin	Rdx	4	42.7	9.8492	82	0	46.781	108.945904	125.4360745	117.5016249	102.8452337	110.6428451	100.652776
sp|Q9Z0V5|PRDX4_RAT F	Ras-related protein Rab-21	Rab21	2	11	31.007	273	0	2.6578	27.41768702	28.32430558	25.20225317	25.32566592	25.0050344	22.64251177
sp|P36876|2ABA_RAT Se	Nucleoside diphosphate ki	Nme2	3	9.6	51.677	447	0	9.1146	0.827709777	0.74520624	0.740660721	0.411511146	0.606661399	0.670847415
sp|P97532|THTM_RAT 3-m	NAD-dependent protein de	Sirt3	7	31.3	32.94	297	0	125.65	37.09662852	37.53760023	35.5787277	54.54985848	40.45303384	52.64638057
tr|D3Z865|D3Z865_RAT M	3'(2'),5'-bisphosphate nucl	Bpnt1	2	8.1	26.547	247	0	11.431	2.644724116	2.483323218	2.675498126	2.704792752	2.975444052	2.774803529
tr|D3ZEY0|D3ZEY0_RAT (	Aminopeptidase (EC 3.4.1	Enpep	19	15.1	173.5	1530	0	69.979	37.75169941	30.38280226	39.21449863	39.38154288	43.32426572	47.48439705
tr|D3ZW08|D3ZW08_RAT	LRP chaperone MESD (LD	Mesd	3	8.3	54.852	484	0	3.8883	1.281993265	1.25754742	1.533567654	1.149158222	1.008103969	1.214916475
tr|M0RCT5|M0RCT5_RAT	Biliverdin reductase B (Biliv	Blvrb	3	4.7	85.75	780	0	2.9785	2.990001894	2.895878676	3.567714741	2.572932098	2.662938363	2.488807982
tr|M0RD75|M0RD75_RAT	39S ribosomal protein L38	Mrpl38	4	19	28.421	247	0	48.729	26.35713403	19.22848982	21.97272533	16.07371825	17.50084191	16.71934484
tr|F1LQC1|F1LQC1_RAT A	Ras-related protein Rab-6A	Rab6a	15	33.9	74.508	660	0	281.45	54.57860858	60.90353521	54.55488675	70.27070407	68.91872366	58.88777657
tr|A0A0G2K7W8|A0A0G2K	40S ribosomal protein S16	Rps16	2	26.2	14.317	130	0	14.559	1.074906339	0.98468246	0.736996157	0.691031427	0.677804362	0.603711347
sp|Q68FY0|QCR1_RAT Cy	Frataxin, mitochondrial (Fx	Fxn	18	55	52.848	480	0	323.31	632.942809	736.2169633	676.5308526	620.0662621	607.7473514	580.7167298
tr|Q4QQV0|Q4QQV0_RAT	Carboxylesterase 1D (Carb	Ces1d	2	6.5	50.059	447	0.0013605	1.7906	0.472623878	0.485611163	0.560468878	0.301517311	0.448426365	0.418486628
tr|A0A0G2JUM8|A0A0G2J	Sorbin and SH3 domain-co	Sorbs2	2	21.5	9.3683	79	0	2.9424	1.94547681	1.970744226	1.969389007	1.629941785	1.754990602	1.883094872
tr|B2GV62|B2GV62_RAT	C-1-tetrahydrofolate syntha	Mthfd1	2	21.5	17.525	149	0	36.788	2.966199258	2.930884535	3.446731781	3.681507795	3.524438763	3.446061563
tr|M0RB00|M0RB00_RAT (	ATP-binding cassette subf	Abcb10	8	7.2	190.6	1734	0	24.751	8.509143577	6.715322246	7.595594011	9.041439386	8.299031119	9.613597621
sp|P07154|CATL1_RAT C	Lysine--tRNA ligase (EC 2.	Kars1	2	11.4	37.66	334	0	3.4691	1.630779639	1.73649986	1.678841439	1.547083988	1.55441651	1.654226576
tr|D3ZEG8|D3ZEG8_RAT T	AFG1-like ATPase (EC 3.6	Afg1l	3	20.6	29.873	267	0	4.6699	1.629743008	2.195428568	1.739987305	1.310490036	1.649862564	0.995338415
tr|D4A833|D4A833_RAT M	Carbonyl reductase [NADP	Cbr1	9	33.2	50.001	440	0	66.908	9.385096554	10.73576413	11.70147614	14.85884672	11.76295748	12.57158818
tr|A0A0G2JSZ0|A0A0G2J	Glycogenin-1 (GN-1) (GN1	Gyg1	4	9.3	68.181	604	0	31.064	17.65422024	20.13502741	18.70236378	13.33797861	17.89597157	14.89927986
tr|C6ZII9|C6ZII9_RAT NAD	RAB5C, member RAS onc	Rab5c	4	20	35	320	0	45.376	9.587239549	9.628989226	9.416882284	12.15956624	10.29953854	10.82235482
tr|F1LPM9|F1LPM9_RAT S	Thrombomodulin	Thbd	2	3.4	92.773	835	0	3.0394	1.643179338	1.435763387	1.746688222	1.247467761	1.470745843	1.386041592
sp|P62828|RAN_RAT GTP	Voltage-dependent L-type	Cacna1c	5	25.9	24.423	216	0	10.6	13.55554177	13.31887307	12.99349668	11.02542572	12.76844469	12.04753675
tr|Q6P722|Q6P722_RAT P	Phosphatidylinositol-3-pho	Sacm1l	5	19.6	41.342	368	0	285.21	30.77158617	27.72834986	30.06094143	22.87205078	28.08626068	25.89563413
sp|Q566R0|THEM4_RAT A	Carbonyl reductase family	Cbr4	3	15.7	26.089	230	0	3.0126	3.047016585	3.620109938	3.313551057	3.56114914	3.904129577	3.79672947
tr|A0A0G2JVH7|A0A0G2J	26S proteasome regulator	Psmc1	2	6	45.338	419	0	5.7729	4.786044396	5.330307842	5.903088984	2.991040617	4.959335095	3.574431064
tr|G3V928|G3V928_RAT L	Coiled-coil domain-contain	Ccdc127	14	4.3	504.88	4545	0	49.318	17.80174077	16.4189843	17.35956285	18.34764177	20.93271305	22.9822983
tr|D3ZPP2|D3ZPP2_RAT A	Proteasome subunit alpha	Psma7	3	18.3	21.39	186	0	36.103	25.12593567	25.47151835	25.0436299	21.40456931	22.46874687	24.43024058
sp|Q7TS56|CBR4_RAT Ca	Mitochondrial-processing p	Pmpcb	3	20.3	25.286	236	0	43.534	8.472462797	9.269893802	9.48598549	12.46122245	9.642079431	12.1199082
sp|P11348|DHPR_RAT Dih	Thioredoxin reductase 2, m	Txnrd2	3	20.3	25.552	241	0	19.961	2.398803408	2.250458159	2.339300561	2.508824729	2.599809205	2.954602955
tr|Q6P9U0|Q6P9U0_RAT S	Superoxide dismutase [Cu	Sod1	2	5	43.018	379	0.00046773	2.0391	6.026412949	5.498202788	5.088822878	4.66677338	4.942326202	4.850375663
tr|A0A0G2K9A2|A0A0G2K	ADP-ribosylation factor 1	Arf1	11	33.7	34.347	300	0	19.32	49.20806393	49.69309925	54.24078127	28.87392414	43.61014595	42.94552659
sp|Q03344|ATIF1_RAT AT	Mitochondrial ribosomal pro	Mrps27	2	19.6	12.248	107	0	3.3806	8.11761612	11.06109032	10.46023599	14.89877818	12.81881718	10.97531009
sp|Q66H47|RM24_RAT 39	Syntaxin-binding protein 1	Stxbp1	5	31.9	25	216	0	24.776	10.97592604	11.87440578	12.27890671	14.80415796	12.29415827	13.5580983
tr|A0A0G2K2U7|A0A0G2K	40S ribosomal protein S2	Rps2	7	11.4	93.726	828	0	28.366	13.23179402	12.99449813	12.73697721	17.1518679	14.52559412	19.77075103
tr|B2GV15|B2GV15_RAT	Lipoprotein lipase (LPL) (E	Lpl	14	37.8	53.273	482	0	186.54	55.9900212	60.61340513	59.7795079	87.05060166	67.34867206	70.64685406
tr|Q6IRL2|Q6IRL2_RAT M	Caveolin-3	Cav3	7	11.8	86.18	757	0	13.047	9.610763093	9.38547021	9.953479146	9.827915703	10.29037991	10.63141738
sp|P83565|RM40_RAT 39	Lectin, mannose-binding 2	Lman2	3	17	24.397	206	0	7.1384	11.17527811	11.57714136	11.75330354	12.71383239	11.70604311	12.29904037
tr|A0A0G2JSR0|A0A0G2J	Mitochondrial-processing p	Pmpca	12	53	30.783	283	0	323.31	340.9797474	363.9182423	354.0963425	343.2326749	291.0941098	289.6141195
sp|Q07009|CAN2_RAT Ca	Desmoplakin	Dsp	10	22	79.918	700	0	190.99	20.11382603	19.97046182	19.34994457	16.46869255	19.25537458	16.67417685
sp|Q4G069|RMD1_RAT R	Mitochondrial amidoxime re	Mtarc2	9	32.9	35.4	310	0	155.85	51.38100144	53.60272096	40.99181213	40.56125736	37.22069009	37.75633962
tr|F1M3J4|F1M3J4_RAT A	1-acylglycerol-3-phosphate	Agpat3	2	1.8	148.9	1325	0.00046253	1.9499	3.034178312	3.045414572	3.15550365	2.299358134	2.791028407	2.816019327
tr|Q499Q2|Q499Q2_RAT	Four and a half LIM domai	Fhl1	2	16	18.348	163	0.00047125	2.1033	1.617143958	1.335834979	1.776789997	1.911328428	1.980554683	1.825403021
sp|Q6AXM8|PON2_RAT S	10 kDa heat shock protein	Hspe1	3	11	39.617	354	0	6.207	3.061609156	2.851312794	3.539288195	2.642985783	2.885296922	2.32604921
sp|Q5RKI0|WDR1_RAT W	Proteasome subunit beta t	Psmb4	11	28.7	66.181	606	0	159.44	49.61872918	47.52092865	43.27012388	40.03607174	42.90296854	43.4829231
tr|D4AAE9|D4AAE9_RAT (	ATP-dependent zinc meta	Yme1l1	3	31.1	15.293	135	0	61.125	22.50286119	20.8356202	19.53579032	22.9332501	23.71105021	22.8149714
sp|A0JPJ7|OLA1_RAT Ob	Dynamin-2 (EC 3.6.5.5)	Dnm2	2	6.8	44.535	396	0	9.2717	3.356012287	3.252738672	3.881924923	3.091520345	2.918333424	2.965176373
tr|F1M9V7|F1M9V7_RAT A	Transcription elongation fa	Tefm	8	10.2	103.34	920	0	16.685	13.64883854	14.34193831	14.04627355	14.46213626	14.47522163	15.36584474

tr|A0A0G2JUV4|A0A0G2J	ER membrane protein com	Emc8	5	16.8	33.443	298	0	12.858	6.236928731	6.816629962	7.277823967	7.516664936	7.214387134	7.859231542
tr|A0A0G2JY30|A0A0G2J	Chaperonin containing Tcp	Cct6a	3	3.9	126.45	1133	0	5.0808	3.600019216	3.96926484	3.399930064	3.829124017	4.259419172	4.182556556
sp|P63095|GNAS2_RAT G	Rho GTPase-activating pro	Arhgap1	11	34	45.663	394	0	84.496	52.42959331	45.40202784	49.17216584	34.50946967	47.03482101	37.1193682
tr|F1M6F4|F1M6F4_RAT U	Mitochondrial ribosomal pro	Mrpl45	4	24.2	13.644	124	0	6.8227	30.06069671	22.89411689	25.50327092	20.3936955	20.04236295	21.94702727
tr|F1M983|F1M983_RAT C	Cysteine and glycine-rich p	Csrp1	9	11	140.01	1234	0	91.82	23.66747596	20.57783249	22.44754811	25.27227928	30.87571904	37.30568619
tr|F1LYS7|F1LYS7_RAT S	Integrin subunit alpha V	Itgav	6	24.9	32.087	289	0	121.44	33.93211383	28.82228294	38.12350559	43.64249101	39.82762995	37.53255272
sp|Q5BK85|TMED1_RAT T	Choline-phosphate cytidyly	Pcyt1a	3	13.2	25.209	227	0	8.7413	6.018438867	4.50034557	4.943496743	3.654510649	4.626876667	3.807610851
tr|G3V936|G3V936_RAT G	Peroxiredoxin-6 (EC 1.11.1	Prdx6	19	39.7	51.83	466	0	323.31	281.0545165	292.0468389	299.7979752	374.361859	306.1927728	342.4966055
tr|D3Z8B0|D3Z8B0_RAT C	T-complex protein 1 subun	Cct7	2	16	12.252	106	0.00047393	2.1375	6.702216453	7.081552017	6.787295909	9.091787759	7.398868198	7.927496811
sp|Q3KRE0|ATAD3_RAT A	Alpha-1-antiproteinase (Al	Serpina1	16	32.5	66.758	591	0	126.23	55.47569287	69.1984017	62.83680123	52.42307374	55.29329247	50.22527325
sp|Q9WVJ4|SYJ2B_RAT S	60S ribosomal protein L11	Rpl11	6	54.5	15.825	145	0	176.72	14.48093406	15.02207931	16.20522521	14.31152517	14.34176724	13.19008178
sp|Q9EQS0|TALDO_RAT	Guanine nucleotide-bindin	Gng5	2	7.1	37.46	337	0	3.3112	0.560817232	0.680854437	0.680352469	0.509256437	0.50188008	0.601863565
tr|F1LPX0|F1LPX0_RAT M	Apolipoprotein A-I (Apo-AI	Apoa1	4	5.5	80.654	710	0	7.5057	13.25810849	11.66703412	12.09306097	16.46782448	13.24796462	18.2109153
sp|Q6PCU8|NDUV3_RAT	AP-2 complex subunit mu	Ap2m1	2	19.4	11.941	108	0	101.97	68.33390112	74.25427503	80.65181703	66.85916773	66.23001029	63.35324903
tr|A0A0H2UHQ8|A0A0H2U	Phospholipid hydroperoxid	Gpx4	3	32.1	15.379	134	0	23.459	6.293544718	3.864865574	5.629817216	3.24664542	3.716050478	3.257844465
sp|P68101|IF2A_RAT Euk	ATP-binding cassette sub-	Abcd3	5	20.3	36.108	315	0	8.1309	6.323846232	5.367882065	4.577930315	6.452404489	6.746642593	6.897358507
tr|F1M5V2|F1M5V2_RAT C	60S ribosomal protein L30	Rpl30	4	24.7	18.985	170	0	16.252	14.45342348	14.17594586	17.93804044	9.372610153	14.37316827	11.57789223
sp|P18420|PSA1_RAT Pro	NADH:ubiquinone oxidored	Ndufa3	7	31.6	29.517	263	0	15.77	11.06124872	9.598073726	11.65174277	7.744100692	9.371245529	9.914888695
tr|D3Z9E1|D3Z9E1_RAT E	CD9 antigen (CD antigen C	Cd9	3	5	94.504	900	0	3.7863	5.207873367	5.220914534	5.226767535	5.589103498	5.313577988	5.392442958
tr|D4ACC2|D4ACC2_RAT	Beta-2-microglobulin	B2m	2	2.5	90.221	840	0	3.2042	4.349463372	4.199751784	4.354182512	4.622067496	4.771125158	5.446336592
tr|G3V9L3|G3V9L3_RAT D	Anoctamin	Ano6	2	15.1	21.095	192	0	17.035	1.433381224	1.196382292	0.870438635	1.302416935	1.714627192	1.878167454
sp|P54921|SNAA_RAT Alp	Golgi apparatus protein 1	Glg1	12	49.2	33.192	295	0	163.34	20.78125675	22.04798343	23.09565242	18.81640249	20.34656045	14.88388168
sp|Q5XIN6|LETM1_RAT M	All-trans-retinol 13,14-redu	Retsat	28	37.5	83.059	739	0	323.31	204.1165802	202.8579992	181.6419639	186.4669375	164.508699	179.9328768
sp|O08776|NDUF3_RAT N	Coiled-coil domain-contain	Ccdc47	6	53.5	20.696	185	0	82.49	13.3362545	14.75953537	14.15306941	15.6323019	14.97894652	14.67908587
sp|P13221|AATC_RAT As	Similar to 106 kDa O-GlcN/	Trak1	14	43.1	46.428	413	0	177.38	93.79913401	76.94630165	73.46403663	58.90759702	75.0157574	63.02988724
tr|D3ZU87|D3ZU87_RAT U	39S ribosomal protein L46	Mrpl46	2	19.8	11.458	106	0	87.424	9.82765814	9.654197249	9.058802037	9.328338307	8.679768634	8.378150239
tr|F1LPM3|F1LPM3_RAT S	Protein ERGIC-53 (ER-Gol	Lman1	6	5.9	134.14	1196	0	44.258	5.877696308	5.117228702	6.03239574	6.187207452	7.916985228	9.065935653
tr|A0A0G2K5E4|A0A0G2K	Rab GDP dissociation inhi	Gdi2	13	27.1	52.403	480	0	189.36	58.72911859	56.85122658	59.44969714	72.89663217	63.33391926	63.05555087
sp|Q63942|RAB3D_RAT G	Striated muscle-specific se	Speg	2	8.7	24.29	219	0.0047069	1.4016	5.299974022	5.291306749	7.570989081	11.05146799	13.56590007	7.127304675
sp|P38718|MPC2_RAT Mit	cAMP-dependent protein k	Prkar1a	6	45.7	14.258	127	0	30.462	29.79595716	39.55233959	34.9253883	28.3370195	26.49069574	28.32238744
sp|D3ZAW2|PISD_RAT Ph	Inosine-5'-monophosphate	Impdh2	8	22.2	45.823	406	0	20.514	36.00019216	40.42320545	37.27280325	36.26558621	34.34226215	33.63168022
sp|P50399|GDIB_RAT Ra	Blood vessel epicardial sul	Bves	8	20.4	50.537	445	0	37.244	31.57218407	30.82418048	27.68996857	23.90332437	29.15585834	25.38441452
tr|B2RYM3|B2RYM3_RAT	Histone cell cycle regulatio	Nfu1	2	3.5	100.59	904	0	26.93	1.323139531	1.395097614	1.473992315	1.261400372	1.288031087	1.010633941
sp|Q07014|LYN_RAT Tyro	60S ribosomal protein L7a	Rpl7a	8	19.3	58.659	512	0	13.448	15.42426804	13.54479403	15.84033935	11.10094829	13.93289964	13.26655941
tr|D4A6E3|D4A6E3_RAT M	Limbic system-associated r	Lsamp	2	2	165.32	1487	0	12.805	16.60323615	13.51863476	12.05013322	11.04669357	11.24484045	10.01497687
sp|P18886|CPT2_RAT Ca	Mitochondrial ribosomal pro	Mrpl20	25	41.8	74.109	658	0	323.31	261.8528255	254.2966352	243.9290805	329.9207386	278.5271551	277.8037147
sp|Q792I0|LIN7C_RAT Pro	Decorin (Bone proteoglyca	Dcn	3	21.8	21.834	197	0	22.891	7.219335711	5.905811767	6.447538482	4.792210276	5.987784316	5.73530912
tr|E9PTK4|E9PTK4_RAT	Acetyl-CoA carboxylase 1	Acaca	3	5.1	82.709	745	0	2.7468	2.228835837	2.523180432	2.32893508	3.907771649	2.579725628	3.528698467
sp|Q9R0T3|DNJC3_RAT D	Plexin B2	Plxnb2	4	11.9	57.56	504	0	19.947	2.925770659	3.432381573	3.785180435	2.695981787	2.843036366	2.734614277
sp|O35878|HSPB2_RAT H	Mitochondrial ribosomal pro	Mrpl39	3	25.3	20.346	182	0	14.992	10.1522033	11.23136338	12.15692909	9.323563892	10.42448848	9.669544345
sp|P22002|CAC1C_RAT V	Collagen alpha-1(XV) chai	LOC1083480	9	5.8	243.48	2169	0	43.621	6.496086417	6.474656969	7.979849714	5.668532398	6.312850417	5.42888532
tr|F1M8Z8|F1M8Z8_RAT S	Major vault protein (MVP)	Mvp	21	28	104.28	938	0	119.99	55.43183542	47.48525692	66.00926658	61.21667759	72.45133978	75.53321014
sp|Q01129|PGS2_RAT De	Fermitin family homolog 2 (	Fermt2	9	26.8	39.805	354	0	36.76	24.83687518	30.62346754	23.988759	30.35876712	32.84744218	42.52464298
sp|Q05030|PGFRB_RAT P	Dynamin-1-like protein (EC	Dnm1l	4	4.6	122.83	1097	0	9.2214	2.94423066	2.59671176	3.249368838	2.272664815	2.665358859	2.511186672
tr|B0BNI6|B0BNI6_RAT Si	Myosin light chain 4 (Myos	Myl4	3	11	32.491	300	0.00090909	1.8195	10.05133116	10.66442067	10.50578128	9.495008785	8.894996541	10.18948959
sp|E9PU28|IMDH2_RAT In	Peroxisomal bifunctional e	Ehhadh	3	10.5	55.814	514	0	37.132	4.851033169	4.141250145	5.157088469	3.606375868	4.370238648	3.978222695
tr|D3ZS58|D3ZS58_RAT N	ADP-ribosylation factor-like	Arl8a	5	42.3	10.844	97	0	304.12	153.8160669	177.3027712	162.3454172	149.2048007	153.008071	142.7308717
sp|P97615|THIOM_RAT T	Vesicle-fusing ATPase (EC	Nsf	3	22.3	18.232	166	0	67.638	18.48193002	17.75215578	15.92514783	24.86992633	18.21259864	24.45744403
sp|Q499N5|ACSF2_RAT A	Dipeptidyl peptidase 4 (EC	Dpp4	18	37.7	67.886	615	0	307.28	101.2588891	101.7072591	109.4919352	144.5909799	109.2167149	143.5161789
sp|P04182|OAT_RAT Orn	Heterogeneous nuclear rib	Hnrnpu	11	31.4	48.332	439	0	152.72	25.33605275	29.99088884	31.66654395	40.10942411	30.15545786	38.90093773
sp|Q8CFN2|CDC42_RAT C	Parvin, beta	Parvb	2	14.1	21.258	191	0	14.462	18.01863582	18.63586346	20.14986653	16.01295297	18.57567307	16.00692234
sp|B1H267|SNX5_RAT So	NADH-ubiquinone oxidored	Mtnd1	3	8.9	46.793	404	0	3.2258	1.071796447	1.175407314	1.22893769	1.242259309	1.264611151	1.458618351
tr|A0A0G2K0X5|A0A0G2K	Heterogeneous nuclear rib	Hnrnpk	9	25.1	62.896	558	0	72.716	14.79870125	15.44728635	15.4367138	14.16351832	13.51029408	14.99988131
tr|Q99JC6|Q99JC6_RAT T	Catenin beta-1 (Beta-cater	Ctnnb1	2	5.2	50.044	464	0	3.1869	3.674337666	3.280752871	3.989348997	4.24658148	6.153686436	7.594896103
sp|O88989|MDHC_RAT M	Ras-related protein Rab-12	Rab12	9	33.5	36.483	334	0	176.62	99.07398967	82.66329106	79.69379532	68.69948759	82.03519646	65.90421434
sp|P63245|RACK1_RAT R	Tripeptidyl-peptidase 1 (TP	Tpp1	8	37.9	35.076	317	0	96.82	52.55319159	35.77398986	42.77750178	30.40390704	33.05154889	31.99998632
tr|Q7TQ70|Q7TQ70_RAT	Heterogeneous nuclear rib	Syncrip	13	26	86.656	782	0	305.98	33.10440405	29.18137836	33.69095376	38.28993806	47.22780652	62.99395815
sp|Q68FS4|AMPL_RAT Cy	Actin-related protein 2/3 co	Arpc3	12	32.9	56.149	519	0	83.565	38.61888089	38.57208043	35.34628964	39.95447265	38.78747076	42.32241353
tr|G3V9U2|G3V9U2_RAT	Protein kinase C substrate	Prkcsh	27	79.3	41.885	397	0	323.31	296.2092605	301.5925941	286.4432572	391.8709399	309.6468863	354.9486011
sp|P20761|IGG2B_RAT Ig	Motile sperm domain-conta	Mospd1	3	12.9	36.497	333	0	26.339	11.79007988	8.260146006	9.709000381	14.98081131	31.38729419	18.92641744
tr|A0A0G2K3W1|A0A0G2I	ATP-dependent 6-phosph	Pfkl	11	28.1	53.76	481	0	149.64	44.54322559	47.65743247	45.70862942	55.44397615	47.37565305	51.21434974
tr|A0A0H2UHM3|A0A0H2U	Transgelin-2	Tagln2	5	18.2	38.432	346	0	24.235	6.888411282	5.758844235	6.948536722	10.08747025	10.63971639	18.55070183
tr|G3V6A8|G3V6A8_RAT	Translational activator of c	Taco1	3	1.3	364.25	3187	0	3.9165	4.897282849	5.013542871	3.362394459	4.949331924	6.398679905	5.763025846
sp|Q5M9I5|QCR6_RAT Cy	Vanin 1	Vnn1	6	57.3	10.424	89	0	323.31	82.81882222	97.06991411	94.72897762	78.63895098	81.66885109	83.6891133
sp|O08590|AOC3_RAT Me	ATP-dependent Clp protea	Clpp	10	12.6	84.98	763	0	70.116	21.77562485	18.41993059	22.54282678	22.24399823	24.86503814	24.35530276
tr|A0A0G2JSV3|A0A0G2J	26S proteasome regulator	Psmc5	3	13.5	34.806	318	0	17.887	3.898728351	3.415449327	3.810518277	3.914369022	4.007295051	4.466960958
sp|P70470|LYPA1_RAT A	F-actin-capping protein sul	Capza1	3	23.9	24.708	230	0	22.174	5.258110088	5.164315387	5.021185498	6.657270285	5.312138774	6.432333443

sp|P67779|PHB_RAT Prol	Phytanoyl-CoA dioxygenas	Phyh	15	58.8	29.82	272	0	323.31	143.9042822	170.6868542	157.4191963	148.3844694	136.9085001	134.0565631
tr|Q6P9Y4|Q6P9Y4_RAT	40S ribosomal protein S26	Rps26	9	26.5	32.904	298	0	323.31	2158.663909	2688.982637	2262.763526	2254.218213	1826.100856	1826.788857
sp|O35142|COPB2_RAT	Mannosyl-oligosaccharide	Mogs	6	8.8	102.55	905	0	8.402	4.433988648	5.024957825	4.373395297	5.215831074	4.803703729	5.261558421
sp|Q63524|TMED2_RAT T	ATP-dependent RNA helic	Supv3l1	2	10.4	22.733	201	0	4.2198	5.171989995	5.586668681	6.474760957	4.460171433	4.732266381	5.365752778
sp|P15791|KCC2D_RAT C	Tensin 1	Tns1	2	6	60.08	533	0	55.656	8.348465812	7.302241125	6.995129035	4.934140605	7.214387134	5.985786197
tr|Q6PDW1|Q6PDW1_RAT	Secretory carrier-associate	Scamp1	4	39.4	14.515	132	0	18.405	20.99416475	13.43207136	14.57920584	9.6885896	11.81005902	11.13853081
tr|X1WI37|X1WI37_RAT 4	Malectin	Mlec	7	32.8	29.466	262	0	91.031	32.25237332	21.20708183	24.0861317	15.97041727	19.71984818	19.19383252
tr|A0A0G2JT68|A0A0G2J	Mitochondrial ribosomal pr	Mrps35	8	7.2	159.16	1393	0	30.009	6.085819865	6.002838875	6.985182362	6.881233739	6.948786737	7.370595934
tr|D3ZTW9|D3ZTW9_RAT	ATP-binding cassette sub-	Abcd1	4	17.4	41.326	368	0	16.877	4.071167888	4.315994064	4.187497204	4.420239965	4.312342995	4.766096286
tr|F1LPB8|F1LPB8_RAT E	Chitinase domain-containi	Chid1	2	6.8	47.39	427	0	7.5386	2.769279287	2.810361647	2.512686786	3.337489511	2.762440384	3.128910363
tr|F1MAA7|F1MAA7_RAT	RCG64242 (Solute carrier	Slc30a9	19	15.2	177.38	1607	0	89.01	37.96381	42.00607905	44.9511117	33.45606016	38.02403317	38.82599992
sp|Q5U2Y3|MPP7_RAT M	Nck-associated protein 1 (	Nckap1	7	15.8	65.622	576	0	47.531	21.04081314	14.63064152	15.69323328	11.96728754	14.27569424	11.70569714
sp|Q5EB62|S2546_RAT S	Laminin subunit beta-2 (La	Lamb2	9	34.4	46.21	418	0	91.441	19.99142386	21.62230078	18.73115678	18.52559723	18.19689812	17.81877496
sp|Q62651|ECH1_RAT De	Tropomyosin alpha-4 chair	Tpm4	15	52.9	36.171	327	0	323.31	259.6600528	250.8388554	201.1217387	304.9331881	247.4401333	304.5708859
tr|D4A742|D4A742_RAT R	Sodium/potassium-transpo	Atp1b3	2	31.6	8.1828	79	0	28.367	10.06289358	12.62398775	11.43919807	10.11438059	10.01627506	9.035139292
tr|D3ZKG1|D3ZKG1_RAT	39S ribosomal protein L10	Mrpl10	19	30	92.035	833	0	284.58	58.52976652	61.06524706	67.0824603	73.66487891	63.36597448	75.77444831
sp|Q63707|PYRD_RAT Di	Estradiol 17-beta-dehydro	Hsd17b8	8	26.3	42.662	395	0	125.67	16.51591995	17.0496605	16.02984965	14.89226762	15.81303643	16.19067397
tr|D4A197|D4A197_RAT M	Protein Niban 1 (Protein F	Niban1	3	24.7	18.816	178	0	27.841	5.39725783	4.757182032	4.975378449	6.466293695	5.24933671	7.125251585
sp|P85834|EFTU_RAT Elo	NSFL1 cofactor p47 (XY b	Nsfl1c	24	63.5	49.522	452	0	323.31	173.3645305	208.6177947	213.5707859	255.8044605	216.0783524	220.9125689
tr|A0A0G2K8V0|A0A0G2K	Cytochrome c oxidase sub	Cox7a2	6	6.1	162.54	1445	0	6.5738	5.396859126	5.319368511	5.525638899	7.038789425	5.709492669	7.822275908
tr|A0A0G2JWS2|A0A0G2J	Lipoyl synthase, mitochon	Lias	6	8.3	108.95	952	0	7.202	5.068326921	3.220729239	4.322510209	5.80221601	7.778951524	4.847142045
tr|G3V7I3|G3V7I3_RAT C	Mesencephalic astrocyte-d	Manf	2	1.8	131.91	1197	0	2.2148	2.411761292	2.319138131	2.682513148	2.038024033	2.333096688	2.26645825
tr|D3ZF11|D3ZF11_RAT H	Myosin light chain 7 (Myos	Myl7	2	25.6	16.39	156	0	65.761	7.262395758	5.260866872	6.447538482	4.638994967	5.070089152	5.359593506
sp|Q561S0|NDUAA_RAT	Vacuolar protein sorting-as	Vps35	21	62.5	40.493	355	0	323.31	319.999936	363.3094448	319.5342695	318.1973803	292.3501511	295.4346319
tr|D4A6V3|D4A6V3_RAT U	3-oxoacyl-[acyl-carrier-prot	Oxsm	3	26	19.015	177	0	19.687	2.173535574	1.941873905	2.126179993	2.466549457	2.623948748	3.602763717
sp|Q5I0E7|TMED9_RAT T	1-phosphatidylinositol 4,5-	Plcb4	2	14.9	27.028	235	0	21.028	3.174601907	2.933643149	3.497564518	2.862044612	2.708862372	2.946031301
tr|G3V9S0|G3V9S0_RAT	F-actin-capping protein su	Capzb	6	31.1	34.232	305	0	69.323	13.19830287	13.19568669	13.59814973	13.16002315	12.88489019	12.33650928
tr|A0A0G2K890|A0A0G2K	Regulator of MON1-CCZ1	Rmc1	7	13.5	69.287	585	0	46.2	13.67276079	11.95431046	13.20708841	14.28504887	13.43113731	15.64147218
tr|F7EMB2|F7EMB2_RAT	Voltage-dependent L-type	Cacnb2	3	13.5	28.17	244	0	11.851	6.871665709	6.594038361	6.390475986	4.52701324	6.268954391	5.632654581
sp|Q9JJW3|USMG5_RAT	Adenylate cyclase type 5 (	Adcy5	5	46.6	6.4075	58	0	130.42	86.45899094	99.89511519	83.09660468	84.25105848	76.05591659	75.39462651
tr|F1LQI1|F1LQI1_RAT Hy	Trimeric intracellular cation	Tmem38a	4	15.5	34.158	309	0	10.563	8.326537085	8.925542693	7.415506869	6.573501008	7.277843386	7.769922093
sp|P35284|RAB12_RAT R	ER membrane protein com	Emc2	7	26.3	27.271	243	0	35.502	43.52254301	46.48264347	45.59345741	44.81873318	40.67414944	41.71161902
sp|P47860|PFKAP_RAT A	Syntaxin-12 (Syntaxin-13)	Stx12	3	5.1	85.719	788	0	3.4364	4.552005071	4.876563425	4.722942345	4.311253098	4.674566984	4.380012563
sp|P53042|PPP5_RAT Se	Peroxiredoxin-1 (EC 1.11.	Prdx1	2	5.2	56.916	499	0	6.2151	3.998205032	3.702630542	3.529760329	3.150940107	3.684322352	3.236184357
sp|P40329|SYRC_RAT Ar	ATP-dependent Clp protea	Clpx	3	5.9	75.81	660	0	3.6943	6.543133504	6.047547444	5.749700808	9.273649557	6.477247904	7.956240082
sp|Q924S5|LONM_RAT Lo	ER membrane protein com	Emc3	21	29.4	105.79	950	0	287.6	82.28455868	85.64544776	87.96000453	109.7030294	89.63032111	95.95633077
sp|P97541|HSPB6_RAT H	Nascent polypeptide-asso	Naca	6	50	17.505	162	0	27.201	38.73211287	36.14212212	44.87886744	26.48150832	37.80488013	32.8669039
sp|Q5RJQ7|SDHF2_RAT S	Transmembrane protein 65	Tmem65	2	12.8	19.524	164	0	9.6032	2.506772487	2.299685147	2.936624482	4.183559205	2.715011741	4.583268551
tr|D3ZWF3|D3ZWF3_RAT	Tumor protein D54 (Tumor	Tpd52l2	2	19.9	18.487	166	0	12.193	2.810903998	3.210217969	3.1268677	2.725062313	2.68851712	2.935663193
sp|P63102|1433Z_RAT 14	Mitochondrial pyruvate car	Mpc2	7	31.4	27.771	245	0	119.61	55.74282464	58.71091282	47.70110518	48.54277667	47.96573078	44.30210633
tr|F1LPV2|F1LPV2_RAT V	AP-2 complex subunit alph	Ap2a1	3	4.1	96.472	873	0	17.718	3.310480275	3.974544256	3.154823088	2.437425527	2.226398597	3.389601566
sp|P0DMW1|HS71B_RAT	Lactate dehydrogenase D	Ldhd	5	10.5	70.184	641	0	12.559	13.3653599	10.98166126	10.6958151	12.65393519	14.64858149	18.16934021
tr|Q05BA4|Q05BA4_RAT	Alkaline phosphatase, tiss	Alpl	4	12.9	35.118	318	0	9.5356	20.91442393	15.87582274	19.90591127	12.94951486	16.75114227	16.47246068
tr|F1M790|F1M790_RAT F	Uveal autoantigen with co	Uaca	6	8.1	98.667	879	0	6.2378	7.234087764	6.783336347	7.297717314	5.208018395	6.980187769	5.655238579
tr|D4A559|D4A559_RAT D	Serine/threonine-protein p	Ppp2cb	2	9.1	35.69	320	0.00046019	1.9049	0.680109508	0.609843911	0.543611884	0.753706471	0.698607546	1.020129486
tr|Q5U2S7|Q5U2S7_RAT	NADH dehydrogenase (Ub	Ndufb2	7	16.8	60.687	530	0	19.303	17.83244099	13.99711158	17.13707147	12.95993177	14.66820714	13.79061083
tr|D3ZZV1|D3ZZV1_RAT F	Mitochondrial import inner	Timm13	3	24.8	13.771	125	0	5.244	8.716469726	9.506754095	9.3891363	8.993695238	7.205882688	8.122027163
tr|D3ZVM6|D3ZVM6_RAT	Dihydroxyacetone phosph	Gnpat	2	1.8	173.66	1609	0	3.853	0.925033456	0.824920667	1.196008965	1.016733319	1.497632977	1.634619559
tr|D3ZZT9|D3ZZT9_RAT C	Xaa-Pro aminopeptidase 2	Xpnpep2	11	8	192.56	1794	0	72.238	16.59167373	18.74668364	18.81439473	12.5028907	14.44185803	17.65196133
tr|D3ZTW8|D3ZTW8_RAT	NADH-ubiquinone oxidore	Mtnd4	4	34.5	15.823	148	0	8.4265	6.320656599	7.095820709	8.067275739	8.771901972	7.50877181	8.60296368
sp|P32089|TXTP_RAT Tri	Acyl-CoA-binding protein (	Dbi	3	10.3	33.835	311	0	5.0178	3.338070601	4.101297807	3.472383728	5.626864778	6.648514367	3.86514872
sp|P29419|ATP5I_RAT AT	BAG cochaperone 3 (Bcl2	Bag3	6	66.2	8.2545	71	0	158.28	236.3119388	244.4179439	231.2182788	274.0036614	240.0608907	260.4756285
sp|Q6P7S1|ASAH1_RAT	ADP-ribosyl cyclase/cyclic	Cd38	9	22.6	44.443	394	0	56.135	22.47136356	20.20351713	23.53801764	33.13747649	31.83672146	58.57981295
sp|P55053|FABP5_RAT F	Complex I assembly factor	Timmdc1	4	30.4	15.059	135	0	46.33	14.99845202	15.98616729	13.06469392	13.36054857	12.54798328	12.64241981
sp|P62332|ARF6_RAT AD	Mannose receptor, C type	Mrc1	2	14.9	20.082	175	0	6.5463	3.984768703	3.819586257	4.224299896	2.701971507	3.195185858	3.906107881
sp|Q5RK00|RM46_RAT 3	Tyrosine--tRNA ligase, mit	Yars2	3	12.6	31.673	277	0	37.395	9.161822241	9.551938287	10.47908232	11.01761305	9.895904441	11.47780406
tr|A0A0H2UHH9|A0A0H2U	Transferrin receptor protei	Tfrc	3	29	15.197	131	0	25.707	21.60816912	13.95097614	18.63535461	13.05368391	12.96077602	13.49291267
tr|A0A0G2JTI8|A0A0G2JT	Glutamyl-prolyl-tRNA synth	Eprs	3	2.3	107.93	944	0	45.293	69.66955996	61.31257106	65.28158888	49.80148601	64.77771255	52.81062783
sp|Q5XI32|CAPZB_RAT F	Cytochrome C oxidase ass	Coa5	8	30.5	30.628	272	0	31.295	46.6962279	36.57398787	39.86888504	30.62353012	38.85027283	33.63219349
tr|D4A8C5|D4A8C5_RAT 1	RCG37250, isoform CRA_	Romo1	12	12.6	137.55	1206	0	31.343	17.74831442	15.97427672	17.68099746	15.10234187	16.62946327	15.85448035
sp|Q68FT3|PYRD2_RAT F	Glycerol-3-phosphate acyl	Gpam	10	23.4	62.878	581	0	106.07	47.28232297	48.25196132	40.34056677	42.02049211	36.58285663	41.32563795
tr|F1LP34|F1LP34_RAT A	Malonyl-CoA-acyl carrier p	Mcat	2	8.1	30.973	271	0	18.834	0.180912	0.241759211	0.245300674	0.295688185	0.428938099	0.261348192
sp|Q66HG9|MAVS_RAT M	Pyruvate kinase (EC 2.7.1	Pkm	6	25	53.804	507	0	18.774	8.029502507	8.082262974	8.997551469	8.848292608	8.815185585	9.431899087
sp|Q5BK63|NDUA9_RAT	60S ribosomal protein L5	Rpl5	24	63.7	42.559	377	0	323.31	460.7026241	533.0783465	482.1414413	478.6567786	413.4338394	436.3433855
sp|Q9R1B1|T10B_RAT Mi	[3-methyl-2-oxobutanoate	Bckdk	3	43	11.351	100	0	11.471	7.666283043	9.446825587	8.277202902	8.077441648	6.865704839	7.090349041
tr|Q6S399|Q6S399_RAT F	Chondroitin sulfate proteo	Cspg4	75	21.2	513.98	4513	0	323.31	175.3819734	140.8557747	176.0404162	167.7295298	205.8533913	209.8053478
tr|A0A0G2K477|A0A0G2K	Phosphatidylglycerophosp	Ptpmt1	2	4.7	51.136	466	0.0018042	1.7546	2.269144824	1.937260361	1.924576626	2.63868881	2.563305505	2.063664207

tr|B1H248|B1H248_RAT V	RCG37273, isoform CRA_	Uqcc1		5	17.4	43.717	386	0	7.4819	6.503661795	7.098674448	7.200868124	8.23760156	7.493071294	10.2474894
sp|P84817|FIS1_RAT Mito	Cytochrome c oxidase sub	Cox6c2		3	25	16.994	152	0	7.4435	7.180262706	8.180241329	7.530678878	7.505813993	6.687765657	6.600686888
sp|P35171|CX7A2_RAT C	Methylmalonyl CoA epimer	Mcee		3	30.1	9.3529	83	0	31.904	155.0959072	177.3646022	152.3149822	158.7666513	132.4142274	134.4261194
tr|F1MAR6|F1MAR6_RAT	Guanine nucleotide-bindin	Gnaq		14	32.7	68.094	599	0	190.01	43.67405058	46.19822087	38.75538112	40.4822625	35.45765298	38.84396446
tr|A0A0G2JV81|A0A0G2J	FAD-dependent oxidoredu	Foxred1		3	20.4	20.568	181	0	42.543	69.84498978	71.89518456	63.56971402	53.69046383	63.71531096	65.05731439
sp|Q5U2X7|TIM21_RAT M	PNKD metallo-beta-lactam	Pnkd		6	28.2	27.876	245	0	23.457	35.91407206	39.80679794	36.78960432	36.9331362	33.50032197	33.92629875
tr|D3ZUD8|D3ZUD8_RAT T	Cysteine desulfurase, mito	Nfs1		4	7.2	69.968	609	0	11.738	9.471216647	12.46132466	12.36162116	8.057909951	10.12944962	9.908729422
tr|A1L1L6|A1L1L6_RAT M	Peptidylprolyl isomerase (E	Fkbp3		9	20.4	72.156	631	0	121.57	28.95628626	32.08315477	29.19662786	33.8905319	30.347135	35.22590522
sp|P36365|FMO1_RAT Di	Similar to RIKEN cDNA 57	Trabd		3	6.4	59.825	532	0	5.2854	4.21031563	4.761938263	5.542391191	5.117738553	5.856815844	6.000157832
tr|Q5U2X8|Q5U2X8_RAT	Utrophin (Utrophin, isoform	Utrn		15	38.5	50.443	439	0	163.2	51.1258308	54.56347957	50.51444326	63.23495291	52.78709759	58.77485657
sp|P11530|DMD_RAT Dys	Nucleobindin-1 (Bone 63 k	Nucb1		129	41.8	425.82	3677	0	323.31	486.2196885	425.9157106	535.8639485	492.8932152	607.60343	590.4175838
sp|P18163|ACSL1_RAT L	Golgi integral membrane p	Golim4		37	55.4	78.178	699	0	323.31	653.595683	676.3360177	665.7465644	655.2667198	633.7905824	591.6494382
sp|P53987|MOT1_RAT Mo	Heat shock protein beta-6	Hspb6		5	12.8	53.238	494	0	52.398	20.91522134	19.00589822	22.33970523	18.17446073	19.72115656	18.45471983
tr|B1WC24|B1WC24_RAT	Aldo-keto reductase family	Akr1c15		3	12.6	29.294	261	0	17.002	3.921374746	3.594236043	4.247281947	3.305457529	3.700349962	3.586236336
tr|G3V6L9|G3V6L9_RAT F	Histidine-rich glycoprotein (	Hrg		4	21.9	25.178	224	0	27.533	23.17308283	16.86939935	18.01237874	15.11492896	14.8526882	15.87090507
tr|D4AC23|D4AC23_RAT T	Inter-alpha trypsin inhibitor	Itih1		11	26.5	59.658	544	0	39.342	16.15150437	16.25727245	16.55859388	15.15659658	15.03782345	16.29692141
sp|Q6IRE4|TS101_RAT Tu	Microtubule-associated pro	Mapre2		3	10	44.077	391	0	12.82	12.29164967	11.36644033	11.034002	10.19380949	11.36390269	10.55134684
sp|Q03346|MPPB_RAT M	Mitochondrial import inner	Timm9		9	23.3	54.265	489	0	42.987	20.94592155	23.2551148	22.42556073	24.88858995	22.38370241	24.6760982
sp|P07687|HYEP_RAT Ep	Short/branched chain spec	Acadsb		3	6.4	52.581	455	0	5.1778	6.4462484	6.740054647	6.618202459	7.035317124	6.621038464	6.93020796
tr|A0A0G2K059|A0A0G2K	ATP synthase mitochondri	Atpaf2		7	24.2	36.057	310	0	25.378	19.33715038	24.20873908	22.49885201	19.09158239	18.80790987	19.10144343
tr|D3ZC56|D3ZC56_RAT D	CD99 molecule (Xg blood	Cd99		2	0.4	847.2	7512	0.0059272	1.321	2.261330223	2.5278891	2.557394466	2.271970355	2.294761261	1.813597749
sp|P29975|AQP1_RAT Aq	PRA1 family protein 3 (ADI	Arl6ip5		3	14.5	28.856	269	0	80.704	18.84036504	16.40328873	26.52620777	11.38697913	17.30523965	14.38754698
sp|Q99P75|RAB9A_RAT F	Phosphoinositide phospho	Plcd3		2	11.4	22.895	201	0.00046361	1.9564	4.310789071	3.811310416	4.271834525	4.29528051	4.426629668	4.68648769
sp|Q9WUS0|KAD4_RAT A	Thioredoxin domain contai	Tmx4		8	47.5	25.203	223	0	49.28	18.2291516	17.50958801	20.35979369	21.38633973	20.45188474	19.53156595
sp|P04797|G3P_RAT Glyc	Importin 5	Ipo5		12	44.7	35.828	333	0	323.31	210.5157815	201.6927227	214.319404	131.6783583	204.1525021	160.3258597
tr|F1M964|F1M964_RAT M	Ig gamma-2B chain C regi	Igh-1a		10	21	58.381	523	0	41.118	25.47360567	27.29315474	27.93601786	33.99773922	27.33590685	30.99756474
sp|P37397|CNN3_RAT Ca	Cytosolic acyl coenzyme A	Acot7		2	7	36.434	330	0.0017937	1.7207	1.248661599	1.538688222	1.48917408	2.134250192	1.432083322	2.223497325
sp|Q63159|COQ3_RAT Ub	Corrinoid adenosyltransfer	Mmab		4	17.1	38.708	345	0	54.315	20.40448134	15.94906869	20.60008438	21.58035458	21.88259427	22.79752013
tr|D3ZTN1|D3ZTN1_RAT T	Sodium channel protein	Scn7a		2	13.1	24.599	214	0	2.9816	5.609767132	5.036372779	5.242943967	6.38426057	5.31737228	6.121803461
tr|A0A0G2JUL7|A0A0G2J	BWK4 (Endoplasmic reticu	Erp44		6	22.6	49.341	429	0	68.197	14.02242431	16.36238515	13.45470823	12.26069703	14.00224358	12.04035093
sp|P56741|MYPC_RAT My	Mitochondrial translational	Mtif2		46	44.8	140.76	1274	0	323.31	241.4711703	206.7295711	210.770012	221.9582004	310.7851737	284.5686489
sp|Q7M0E3|DEST_RAT D	Coronin	Coro1c		5	29.1	18.533	165	0	23.949	18.65058187	20.70244574	16.09528829	13.87054287	15.96284553	17.23569718
tr|A0A0H2UHR7|A0A0H2U	DnaJ homolog subfamily C	Dnajc10		26	13.5	287.3	2693	0	108.72	39.64793626	32.64676811	41.73467159	38.6944612	48.0749802	47.90425412
sp|P21531|RL3_RAT 60S	Profilin-1 (Profilin I)	Pfn1		5	12.4	46.135	403	0	7.3309	10.22117912	10.23921364	12.98669106	7.608680929	9.962631634	9.883065788
tr|Q5XIW0|Q5XIW0_RAT M	Bifunctional epoxide hydro	Ephx2		2	17.7	21.873	209	0	73.805	10.75105691	12.91934968	11.44338614	11.2185725	9.952818812	10.03396796
sp|Q5RJS6|MSPD1_RAT M	60S ribosomal protein L18	Rpl18		3	21.1	24.088	213	0	34.346	10.39262189	10.72101981	10.31941203	10.87568272	12.53162858	11.14776971
tr|Q5XI19|Q5XI19_RAT Fe	COX15 homolog, cytochro	Cox15		14	26.9	77.875	680	0	36.356	23.12563704	23.08864673	23.61078541	23.45496342	29.75967402	28.65293506
sp|P54311|GBB1_RAT Gu	40S ribosomal protein S24	Rps24		6	26.8	37.377	340	0	323.31	49.37551966	44.56302872	43.54234863	35.81331893	45.77943392	36.70053768
sp|P51886|LUM_RAT Lum	Glutathione S-transferase	Gstp1		3	10.7	38.279	338	0	7.5905	5.693893704	5.963837782	6.348595256	6.152918474	8.870137391	7.782240637
tr|D4A8H8|D4A8H8_RAT (	Acidic leucine-rich nuclear	Anp32e		7	6.7	145.26	1253	0	12.83	15.14557385	16.39044691	16.60413917	14.11316994	14.95081643	15.78929472
tr|D4A1T7|D4A1T7_RAT F	Hsp90 co-chaperone Cdc3	Cdc37		2	12.6	23.915	222	0	3.4071	9.387887483	9.307468025	8.859868567	10.15474609	9.186110277	10.29368395
sp|B2GV71|NDUF5_RAT A	Calcium uniporter protein	Mcu		2	7	38.229	343	0	4.4857	3.766757284	4.232617339	4.471553259	5.520959579	4.211663436	5.018985744
tr|G3V6G7|G3V6G7_RAT	Protein S100-A10 (Calpac	S100a10		17	25.1	85.897	784	0	150.17	62.26961128	63.99032899	60.23496084	72.59280578	62.83346531	67.84951786
tr|D3ZVS2|D3ZVS2_RAT L	Oligoribonuclease, mitocho	Rexo2		15	41.9	50.732	463	0	291.65	59.42685082	63.42909376	48.0173047	65.30097238	63.77876721	86.95352766
sp|P38062|MAP2_RAT Me	28S ribosomal protein S25	Mrps25		3	9	53.051	478	0	3.5312	0.947201405	0.602899814	0.867559335	0.845288426	1.605312349	1.27178709
tr|G3V8U9|G3V8U9_RAT	Putative 60S ribosomal pro	Rpl37a-ps1		4	22.4	25.76	232	0	40.574	6.900372406	5.833992682	7.672549853	5.49621943	5.686596083	6.054564738
tr|A0A0H2UHS3|A0A0H2U	Myosin-10 (Cellular myosin	Myh10		12	33.5	42.308	379	0	152.28	40.33290996	55.23886435	48.45600535	40.26046923	40.7703151	37.22356256
tr|F1M8K0|F1M8K0_RAT D	Aldo-keto reductase family	Akr1a1		10	12.1	96.705	893	0	118.48	27.09912242	24.26914321	29.52539159	28.03753348	30.77693662	31.28448418
tr|F1LNY3|F1LNY3_RAT N	39S ribosomal protein L24	Mrpl24		11	16.9	92.72	842	0	98.641	15.54108835	15.46821376	20.778601	17.65752182	43.01352634	36.80575858
sp|Q04462|SYVC_RAT Va	Complement C4 [Cleaved	C4		7	7	140.37	1264	0	10.283	12.10425873	10.83897434	12.3008941	11.75460907	14.55830353	15.34736692
tr|B4F7A5|B4F7A5_RAT C	39S ribosomal protein L9,	Mrpl9		3	24.8	16.588	165	0	26.674	10.13505903	6.331970045	8.565132925	5.602992705	6.90495613	6.006317104
sp|Q641Y8|DDX1_RAT AT	60S ribosomal protein L23	Rpl23a		6	12.8	82.496	740	0	11.505	11.63259174	12.54218058	11.86638152	8.115202928	11.75183628	9.92720724
tr|D3ZUY0|D3ZUY0_RAT F	Ras-related protein Rab-35	Rab35		9	42.5	36.195	334	0	79.655	20.30321049	19.95904687	19.03531558	22.29608275	20.64683282	20.17418337
tr|G3V827|G3V827_RAT (	Mitochondrial ribosomal pro	Mrpl15		2	6.1	51.581	457	0	3.9574	2.350121633	1.5440152	2.157852295	2.241457504	2.433841666	2.959119755
tr|D3ZX69|D3ZX69_RAT 3	Mitochondrial ribosomal pro	Mrpl13		6	34.4	29.465	262	0	32.629	12.27689762	13.27035952	13.66097083	16.07805862	13.57505871	14.06521173
tr|F1M978|F1M978_RAT In	Translationally-controlled tu	Tpt1		2	10.5	30.495	277	0	3.3644	3.390779287	2.884273473	2.955575512	3.137875572	3.629566802	4.089859507
tr|F1M6W2|F1M6W2_RAT	Haptoglobin (Liver regener	Hp		2	2.3	99.979	898	0	2.3752	3.325989866	3.016449127	2.918563416	2.139805874	3.072002225	2.440098403
sp|Q62930|CO9_RAT Com	Tubulin beta-2A chain	Tubb2a		3	8.8	62.28	554	0	6.1299	9.926138061	9.755029342	8.671928788	9.991113879	12.01285736	15.03119094
sp|D3ZHR2|ABCD1_RAT A	Plasminogen receptor (KT)	Plgrkt		4	7.5	81.937	737	0	33.067	2.103244035	2.685034966	2.484626696	3.050503782	2.974789863	2.465248765
sp|Q9Z1H9|CAVN3_RAT (	40S ribosomal protein S11	Rps11		4	23.2	27.91	263	0	46.533	9.075303444	6.925072024	7.857348577	9.400822604	11.20362659	8.349920241
sp|Q7TSA0|MIRO2_RAT M	Signal transducer and acti	Stat1		4	9.5	69.152	622	0	20.287	6.755642806	5.850163867	6.025590121	8.006693502	6.35249422	7.078030497
sp|B0K020|CISD1_RAT C	Destrin (Actin-depolymerizi	Dstn		8	58.3	12.097	108	0	84.861	122.3343887	135.8807573	132.4739861	136.0881813	139.23741	139.2252192
tr|B5DEI0|B5DEI0_RAT Po	Beta-enolase (EC 4.2.1.11	Eno3		2	4.9	54.633	494	0	7.0279	1.515115571	1.371031086	1.647483242	1.152370101	1.397476768	1.419660954
sp|P63322|RALA_RAT Ra	LIM and cysteine-rich dom	Lmcd1		2	14.1	23.553	206	0	13.295	12.40328683	10.69057994	12.16739927	10.52411218	11.46791861	9.754234341
sp|Q6MG12|CF136_RAT U	Phosphoglucomutase-1 (P	Pgm1		5	23.8	20.445	172	0	4.9288	7.991625614	8.986422447	8.54366905	10.86613389	8.455382091	10.72483301
sp|P31399|ATP5H_RAT A	Ubiquitin carboxyl-terminal	Usp5		16	82.6	18.763	161	0	323.31	537.7322623	623.1613576	547.6952549	558.0422744	480.0498207	497.6178801
tr|Q3KR94|Q3KR94_RAT	Phosphoglycerate mutase	Pgam1		7	20.9	54.724	478	0	74.699	16.42062966	14.95358959	15.04460546	15.39488328	21.62615251	22.15746907

tr|B0BNE6|B0BNE6_RAT	Myelin protein P0 (Myelin p	Mpz		6	30.7	23.97	212	0	87.825	110.8636708	132.4372462	119.857416	113.2274155	103.8523719	109.9840736
tr|D4A7L4|D4A7L4_RAT N	39S ribosomal protein L3,	Mrpl3		6	49.7	17.634	151	0	289.37	92.45948812	98.59666419	95.81787661	95.85288627	83.18002576	85.32132048
sp|Q6Q0N1|CNDP2_RAT	Purine nucleoside phosph	Pnp		4	11.2	52.693	475	0	14.29	9.477595913	9.817811588	8.189776877	9.848749513	10.29692179	10.20899395
sp|Q60587|ECHB_RAT Tr	Guanine nucleotide-bindin	Gna13		23	49.9	51.414	475	0	323.31	695.2602647	762.8042934	686.8439825	708.3495309	618.4694955	647.0828896
tr|B1PLB1|B1PLB1_RAT (	40S ribosomal protein S17	Rps17		4	7.5	41.238	386	0	8.2661	24.62316976	15.22564599	23.11711629	12.77763593	18.21063608	15.23085402
tr|Q63318|Q63318_RAT L	Mitochondrial import inner	Timm21		2	15.7	14.158	134	0	3.7814	3.627848764	3.043084019	4.108447325	3.733158281	5.851909433	4.68931069
tr|D3ZJX5|D3ZJX5_RAT R	Aldo-keto reductase family	Akr1b1		8	31.7	39.854	353	0	190.81	40.56415835	47.85719417	42.44297945	42.71842474	38.00244496	36.43260933
sp|Q4KMA2|RD23B_RAT	Solute carrier family 25 me	Slc25a34		2	5.5	43.496	415	0	2.7501	4.279291445	3.661346459	3.725762149	3.696525499	3.483290328	3.374203385
sp|Q6AY56|TBA8_RAT Tu	Rho-associated protein kin	Rock2		3	7.8	50.037	449	0	5.9888	6.967753404	5.932922282	6.349118765	5.021382183	5.178095618	6.435413079
tr|A0A0G2JTB2|A0A0G2J	Histidyl-tRNA synthetase 2	Hars2		3	5.5	75.706	655	0	5.7322	3.317497468	3.256353408	3.197593784	3.279849304	3.819739303	4.166593775
tr|F1LM84|F1LM84_RAT N	T-complex protein 1 subun	Cct4		14	15.2	137.04	1246	0	82.604	40.08172635	44.54257693	43.75960492	34.25468954	41.80720335	39.46348461
tr|D4AEH9|D4AEH9_RAT	Growth hormone-inducible	Ghitm		27	22.5	174.33	1532	0	136.02	90.71316403	81.99266252	93.91753846	90.57064743	97.31049022	106.3449702
sp|P05197|EF2_RAT Elon	Thioredoxin (Trx)	Txn		21	28.2	95.283	858	0	124.15	69.01169814	77.86901043	76.49515451	55.15317089	69.53366054	70.47234135
sp|Q6MGB5|DHB8_RAT E	Proteasome subunit beta t	Psmb2		4	26.6	26.79	259	0	32.294	9.567304342	9.643257918	10.94657597	13.33754457	10.28972572	11.53067115
tr|A0A0G2JU25|A0A0G2J	Glycogen [starch] synthase	Gys1		3	6.5	61.854	542	0	4.8377	10.80328715	11.66560725	11.83549448	11.4902801	12.3863988	12.87287925
tr|Q6AYI1|Q6AYI1_RAT D	Transmembrane emp24 pr	Tmed4		2	4.2	69.238	615	0.0043253	1.466	0.273319656	0.437649332	0.388396661	0.262974763	0.318445717	0.244507715
sp|P02680|FIBG_RAT Fib	Carboxylic ester hydrolase	Bche		6	16.4	50.632	445	0	45.953	21.8964322	18.38045387	20.52208152	21.76395253	25.93659836	34.73418998
sp|P62718|RL18A_RAT 6	Protein lin-7 homolog C (Li	Lin7c		6	30.7	20.732	176	0	16.603	16.45013377	18.07129887	11.93234367	10.92689917	12.08416387	13.16801105
sp|O70377|SNP23_RAT S	Syntrophin, beta 2	Sntb2		9	62.9	23.235	210	0	195.01	18.21400084	14.65252018	18.05216543	13.30889808	16.058357	14.83409423
sp|Q68FQ0|TCPE_RAT T-	Integral membrane protein	Itm2b		11	26.4	59.536	541	0	85.426	22.21818644	21.55666479	21.74656938	21.39588856	21.42793349	21.60262129
sp|Q63514|C4BPA_RAT (	Ubiquinone biosynthesis m	Coq6		4	8.8	62.265	558	0	3.7973	7.817790613	7.390231395	6.923931793	9.158195528	8.251275383	13.08229451
tr|A0A0A0MXZ0|A0A0A0M	Proteasome activator com	Psme1		4	39.5	14.122	129	0	8.2322	3.454890912	4.02120288	4.251365318	4.573455274	4.11189974	4.366872782
tr|F8WFS9|F8WFS9_RAT	Mitochondrial coenzyme A	Slc25a42		3	5.1	80.576	725	0	10.656	5.134910511	3.938587151	4.473123787	4.571719123	5.528217127	6.197767821
tr|Q3ZB99|Q3ZB99_RAT	Ectonucleotide pyrophosp	Enpp1		2	1.9	131.36	1164	0	2.4861	2.946224181	2.959612169	3.046561399	1.925868691	2.840485032	2.624363302
sp|Q5HZA9|T126A_RAT T	40S ribosomal protein S3a	Rps3a		5	25	21.657	196	0	67.794	29.68511742	38.06601747	37.33195978	30.74158837	30.33928474	28.97013758
tr|F2Z3T7|F2Z3T7_RAT Is	Eukaryotic translation initia	Eif4a1		2	9.8	32.032	297	0	10.347	1.293316462	1.895786029	1.526186175	2.027693936	1.666609781	2.67045519
sp|Q9QWE9|GGT5_RAT (	Intercellular adhesion mole	Icam2		2	4.7	61.608	572	0	19.035	0.08361623	0.149982982	0.286846359	0.186510341	0.387161643	0.583899021
sp|P08503|ACADM_RAT M	PPARGC1 and ESRR-indu	Perm1		20	49.4	46.555	421	0	323.31	175.5574032	173.4264431	217.015476	241.1990918	195.5041345	218.61824
tr|Q6WN19|Q6WN19_RAT	Aminoacyl tRNA synthetas	Aimp1		3	7.7	50.966	469	0	3.4491	2.429583367	2.315238022	2.52954378	2.024655672	2.39655294	2.249982196
tr|D3ZEH6|D3ZEH6_RAT N	Neudesin (Neuron-derived	Nenf		6	38.1	23.092	210	0	18.909	7.399151275	7.293204287	7.276776949	9.947710109	7.797922981	8.052735349
tr|D3ZPC4|D3ZPC4_RAT N	Nucleosome assembly pro	Nap1l4		2	3.1	140.39	1255	0.0095278	1.1856	0.865227836	1.293694774	0.887138577	1.313441492	1.079802993	1.526626984
sp|Q9ES21|SAC1_RAT Pl	Omega-amidase NIT2 (EC	Nit2		9	18.6	67.038	587	0	43.547	18.12748205	18.37522202	20.52469907	14.69651661	18.11577879	17.90141187
tr|A0A0G2JTU0|A0A0G2J	T-complex protein 1 subun	Tcp1		2	3.4	92.988	850	0.00046168	1.9399	2.827529961	3.273333151	3.114355832	2.845855006	2.586725441	2.980933844
sp|P0CC10|LRC4B_RAT L	Acyl-protein thioesterase 1	Lypla1		3	5.2	76.118	709	0	6.9071	3.761494389	3.555758135	4.465742308	3.501208533	3.370573706	3.372766222
sp|P14480|FIBB_RAT Fibr	14-3-3 protein gamma [Cle	Ywhag		11	33.4	54.235	479	0	110.39	23.97487684	22.70339203	24.62325207	26.40424961	30.1423741	43.10464112
tr|B2RYT0|B2RYT0_RAT N	Calumenin (CBP-50) (Croc	Calu		2	29.9	10.6	87	0	102	0.469075411	0.455765815	0.629100925	0.489681336	0.279384142	0.405762598
tr|G3V7K3|G3V7K3_RAT	Caseinolytic peptidase B p	Clpb		14	17.2	120.66	1059	0	49.842	25.87589814	21.23561921	23.08518224	22.94583719	32.77482729	35.99786736
sp|P01041|CYTB_RAT Cy	Vesicle-associated membra	Vapa		2	32.7	11.196	98	0	14.669	4.858209844	4.305625481	4.358004129	4.705402735	5.51035779	4.769227249
sp|Q505J9|ATAD1_RAT A	Elongation factor 1-alpha	Eef1a1		3	8	40.717	361	0	183.69	30.3302207	30.89504832	33.7904205	31.54238793	28.23083627	28.23769744
tr|Q6PDW4|Q6PDW4_RAT	Syntaxin-4	Stx4		4	19.6	26.407	240	0	5.1007	10.06847544	10.53124621	11.35281906	10.22375809	9.562268474	10.10582614
tr|A0A1B0GWQ1|A0A1B0	Transmembrane 9 superfa	Tm9sf2		3	6.5	62.203	540	0	10.977	5.791576216	6.281078376	5.611494397	4.557829916	5.126087659	5.889804202
sp|P48303|S1PR1_RAT S	Syntaxin-7	Stx7		2	6.5	42.745	383	0	10.515	2.038653966	1.858164243	2.329092133	1.515876677	2.014245374	1.790243841
tr|A0A0G2K719|A0A0G2K	ADP-ribosylation factor-like	Arl8b		2	4.4	73.145	662	0	19.479	20.47664679	20.21160272	17.42761904	21.63938371	20.26217018	21.50920566
sp|P41499|PTN11_RAT T	Ras-related protein Rab-3A	Rab3a		7	14.9	68.458	597	0	7.7148	6.06309373	5.583339319	5.759647481	5.742752845	7.292889714	6.906597416
tr|M0R9L0|M0R9L0_RAT N	Ectonucleoside triphospha	Entpd1		4	2.9	220.19	2163	0	30.579	8.617989805	6.555512892	7.424930033	6.042672897	6.401427496	6.915836324
sp|P11608|ATP8_RAT AT	Similar to RIKEN cDNA 11	Vta1		2	31.3	7.6298	67	0	18.009	24.64390238	33.8748269	32.69995097	25.53574017	25.09662075	24.53597475
tr|D3ZF99|D3ZF99_RAT M	Mitochondrial ribosomal pr	Mrps14		2	24.4	15.062	127	0	8.8498	1.58257631	1.500353001	2.046554254	2.397450655	1.756102722	2.117147222
tr|Q5XIC6|Q5XIC6_RAT P	Short chain dehydrogenas	Sdr39u1		6	20.8	52.936	456	0	45.86	12.33789935	9.62661111	10.42934895	9.432941394	9.691797732	8.903741481
tr|A0A0G2JVM0|A0A0G2J	Lysophospholipid acyltrans	Lpcat3		12	26.3	61.844	562	0	139.99	35.53370832	38.85222242	42.63824836	49.40217133	40.75853972	42.26903317
tr|B2GVB9|B2GVB9_RAT	5'-nucleotidase (EC 3.1.3.	Nt5c3a		3	6.2	75.622	665	0	3.9653	1.753660253	1.633622588	2.217689389	2.107296451	2.268724572	2.197217763
sp|Q9JLA3|UGGG1_RAT	Thrombospondin 1	Thbs1		15	12.5	176.43	1551	0	49.68	26.14143509	25.71123238	27.3020483	24.2974306	24.71392067	26.43303064
sp|Q3ZU82|GOGA5_RAT	Complement C7	C7		5	9.6	82.333	728	0	9.4643	4.510141137	5.386431365	5.108088014	6.209777413	5.400257921	5.210744424
sp|O88775|EMB_RAT Em	Kinectin 1	Ktn1		4	17.4	37.005	328	0	6.5658	9.92215102	9.172866694	10.92040051	9.220696957	8.289218296	9.597172895
tr|M0RAD5|M0RAD5_RAT	26S proteasome non-ATP	Psmd11		7	34.6	29.722	272	0	33.639	20.09787787	20.83990081	21.07228962	112.185725	23.74899312	66.26863796
tr|A0A096MJZ2|A0A096M	Transmembrane emp24 do	Tmed9		2	5.7	49.613	442	0.0018026	1.746	1.859954774	1.72104211	1.701142928	1.920139393	2.628593484	1.967784867
tr|Q6P6Q5|Q6P6Q5_RAT	Fascin	Fscn1		2	3.1	82.797	733	0	3.5699	2.516580608	2.509767861	2.907779128	2.21884414	2.634742853	2.230477834
tr|Q5U302|Q5U302_RAT (	40S ribosomal protein S14	Rps14		25	37.6	100.24	908	0	274.7	70.45899414	54.75848504	67.33374469	49.93169732	60.78193121	55.3923895
sp|P32551|QCR2_RAT Cy	Disks large homolog 1 (Syn	Dlg1		21	48.9	48.396	452	0	323.31	803.468566	893.6957647	818.0353712	821.0257185	761.0825165	779.9178636
sp|Q5XI74|EEPD1_RAT E	Serine hydrolase-like 2 (Sir	Serhl2		2	4.2	62.868	569	0	3.7744	4.753749361	5.375967657	5.199283305	5.530942446	5.193076528	5.649079307
tr|G3V9M6|G3V9M6_RAT	EH domain-binding protein	Ehbp1l1		45	22.7	311.95	2872	0	323.31	137.0824545	106.6584754	145.3941915	126.6999458	273.555325	216.436831
sp|P19511|AT5F1_RAT A	60S ribosomal protein L15	Rpl15		20	49.6	28.868	256	0	143.11	288.4624392	333.3737282	348.0026962	303.8654553	290.3745028	280.9706073
sp|P07943|ALDR_RAT Ald	60S ribosomal protein L32	Rpl32		9	31.3	35.797	316	0	23.45	29.87091354	26.4308501	22.29730099	19.60027458	25.06391134	21.7227271
sp|Q09325|MGAT1_RAT A	Solute carrier family 25 me	Slc25a51		2	6.5	51.585	447	0.00046664	2.0264	0.803109732	0.779498663	1.028747797	0.922199907	1.000907899	1.153631715
tr|D3ZJZ0|D3ZJZ0_RAT R	60S ribosomal protein L7	Rpl7		3	20.6	21.185	189	0	13.129	3.896575349	4.262105969	4.474380209	4.349925857	4.725004892	4.476405175
sp|O35814|STIP1_RAT St	Proliferation-associated pr	Pa2g4		6	15.8	62.569	543	0	95.005	19.22790545	15.73598955	15.03884686	13.79805857	14.15990293	15.21391602
sp|P01048|KNT1_RAT T-k	1-phosphatidylinositol 4,5-	Plcd1		2	5.8	47.775	430	0	10.487	4.182805045	2.762894463	3.138123147	4.191285076	4.56145785	8.375583875
sp|P26453|BASI_RAT Bas	Protein RER1	Rer1		9	23.5	42.435	388	0	152.48	109.1013986	96.31367341	120.4908621	83.86042455	106.0831536	94.17014178

tr|D3ZLT1|D3ZLT1_RAT N	Beta-hexosaminidase subu	Hexb	9	70.8	16.568	137	0	323.31	210.946382	241.7639671	210.8171279	209.8094851	192.6191646	206.8540298
tr|F1LS79|F1LS79_RAT C	Sarcosine dehydrogenase	Sardh	8	5.4	252.01	2326	0	28.032	5.427559344	5.080130102	5.490040278	6.728452468	5.780144991	9.608978167
tr|A0A0H2UH99|A0A0H2U	60S ribosomal protein L24	Rpl24	3	19.9	17.676	156	0	20.578	28.24021364	21.87105165	25.0431064	18.89843561	19.35154024	25.04360145
tr|G3V852|G3V852_RAT F	Serine/threonine-protein p	Ppp1cc	44	26.7	269.67	2541	0	323.31	124.0368553	125.6643736	129.432398	134.0265022	146.9044954	192.8108888
tr|A0A096MJB5|A0A096M	UBX domain-containing pr	Ubxn4	9	13.8	103.4	929	0	76.138	16.8683744	14.64728833	16.17381466	14.4834041	15.34921702	14.44041407
sp|P85973|PNPH_RAT Pu	Phosphatidylserine decarb	Pisd	6	34.3	32.302	289	0	23.506	12.15848249	14.1336154	12.30037059	12.10834979	11.71781849	11.4526537
sp|Q4QR80|RT25_RAT 28	RCG22622 (Similar to isoc	LOC684270	5	40.4	19.775	171	0	25.041	7.793070957	7.633750411	7.943727584	9.457247505	7.788110159	8.656857314
sp|Q5U2R4|TM10C_RAT t	SPARC (Basement-membr	Sparc	4	10.4	48.292	414	0	6.7613	3.619515848	4.304531548	4.003745498	4.677624322	4.191383602	4.186765392
sp|P14408|FUMH_RAT Fu	Ras GTPase-activating pro	Rasa3	16	44.6	54.463	507	0	323.31	194.448005	221.3692495	234.0452281	280.7355861	214.5213846	257.4319214
sp|Q5RK30|SBDS_RAT R	cAMP-dependent protein k	Prkar2a	3	18.4	28.752	250	0	19.186	6.490105855	4.939821294	5.246085022	4.438903586	4.802918703	4.9863416
tr|D3ZDI7|D3ZDI7_RAT Se	Mitochondrial Rho GTPase	Rhot2	2	6	56.336	486	0	2.6021	1.156002759	1.261923152	1.168001227	1.529548864	1.225686954	1.300838324
tr|D4A5K6|D4A5K6_RAT A	Actin-related protein 2/3 co	Arpc5	2	5.9	54.847	475	0	4.6152	3.444564475	2.96603308	3.627028326	2.342154252	3.40962874	2.582326268
sp|Q8R4Z9|MFN1_RAT Mi	2',3'-cyclic-nucleotide 3'-ph	Cnp	13	22.3	83.846	741	0	59.051	33.50829133	30.13738075	32.5062526	33.49685971	32.76828541	36.66050241
sp|Q5RJQ4|SIR2_RAT NA	Importin subunit beta-1 (Ka	Kpnb1	3	13.4	39.319	350	0	5.7529	11.12583879	12.67202568	10.31470045	12.09446059	13.14460289	12.42684528
tr|Q499Q4|Q499Q4_RAT	Endoplasmic reticulum ami	Erap1	10	20.8	61.389	562	0	23.769	37.15164969	34.10027224	31.26187139	28.58702522	33.1339766	31.40151035
sp|P0C5I0|CDNF_RAT Ce	39S ribosomal protein L11	Mrpl11	5	25.7	21.361	187	0	8.0653	7.301468762	9.243734532	9.791191314	6.836093818	8.342207538	7.20994158
tr|D3ZWW5|D3ZWW5_RAT	Mitochondrial import inner	Timm10	5	10.2	62.684	567	0	32.943	10.11671864	10.50413569	10.15869473	12.86140521	10.38065787	11.2853268
tr|F1LVY9|F1LVY9_RAT P	Dihydropteridine reductase	Qdpr	8	42	25.879	231	0	49.922	21.46423693	25.45344467	20.39329828	25.96283326	22.95677125	26.78205608
tr|F1LN42|F1LN42_RAT T	Cullin-associated NEDD8-d	Cand1	11	8	203.91	1909	0	33.229	18.82960002	17.90578204	18.74424451	18.02471772	22.84032575	23.52380099
tr|Q3MID7|Q3MID7_RAT L	DnaJ homolog subfamily C	Dnajc3	2	5.8	53.497	481	0	2.2777	2.84969791	2.226153819	1.811812758	4.081516941	3.460066648	9.92926033
sp|Q920L2|SDHA_RAT Su	Coiled-coil-helix-coiled-coil-	LOC1009115	35	69.8	71.614	656	0	323.31	732.8979348	857.0252253	747.3616383	717.3775151	714.1118059	704.3127953
sp|Q5XJW2|G45IP_RAT G	2-hydroxyacyl-CoA lyase 1	Hacl1	2	11.4	26.467	228	0	14.396	5.983751607	6.492255023	6.392046514	8.701587864	6.334504046	7.328507573
sp|Q68FX0|IDH3B_RAT Is	Syntenin-1 (Syndecan-bind	Sdcbp	15	41.3	42.353	385	0	323.31	48.13953685	54.44457381	58.46026488	82.76230916	58.06508774	60.16069286
tr|E2RUH2|E2RUH2_RAT	14-3-3 protein eta	Ywhah	3	7.2	49.919	456	0	3.661	3.996610215	4.239371186	5.35602194	3.766231954	3.934025976	3.754333145
sp|O08700|VPS45_RAT V	Complement component re	Cr1l	3	5.6	64.893	570	0	2.6	1.649438993	1.99823524	2.127855222	2.046531172	2.154045386	2.299974957
sp|B0BNE5|ESTD_RAT S-	Rho GDP dissociation inhib	Arhgdib	3	10.6	31.363	282	0	3.7649	9.577271946	9.36549404	10.99212127	8.554883121	9.627033103	9.119316014
sp|Q9JI92|SDCB1_RAT S	Insulin-like growth factor 2	Igf2r	4	21	32.423	300	0	19.844	15.10530473	16.31720096	16.44080432	15.76251321	18.73529499	20.98412769
tr|A0A0G2K428|A0A0G2K	Deoxyuridine 5'-triphospha	Dut	10	13	139.84	1263	0	56.937	9.42137863	7.986662736	8.275108865	8.759748917	9.79254271	9.439084904
tr|Q6P9X2|Q6P9X2_RAT	26S proteasome non-ATP	Psmd2	2	10.6	20.626	179	0	2.2682	0.884804209	0.975693183	1.82280645	1.743312433	2.185184743	1.377777902
sp|Q91Y81|SEPT2_RAT S	Apolipoprotein E (Apo-E)	Apoe	10	42.7	41.592	361	0	231.78	25.70325925	25.98328878	43.74913474	20.23961212	23.76861876	21.18533058
tr|E9PTU4|E9PTU4_RAT M	Mitochondrial ribosomal pro	Mrps11	14	10	227.29	1972	0	95.649	21.33346197	17.46820881	19.86507756	19.23524887	25.7298749	23.45091627
tr|A0A0G2K0J2|A0A0G2K	Thioredoxin domain contai	Tmx1	6	6.2	122.35	1076	0	16.006	13.62451759	13.13385569	14.82054356	10.60831549	12.3752776	13.8475841
tr|B5DF05|B5DF05_RAT R	Ectonucleotide pyrophosp	Enpp3	4	8.1	67.815	619	0	9.4889	9.226013606	7.997602066	8.591831891	8.446807733	7.676243982	7.849479361
sp|P21571|ATP5J_RAT A	DEAD-box helicase 3, X-lin	Ddx3x	11	57.4	12.494	108	0	323.31	246.6184406	296.3179342	253.0852553	236.91948	233.7479749	247.2537238
tr|Q3KRE2|Q3KRE2_RAT	Endophilin-B1 (SH3 domai	Sh3glb1	4	24.6	28.142	244	0	91.267	10.04295837	8.8618092	8.735796902	7.745402805	9.024525799	8.514680776
tr|Q5BKA0|Q5BKA0_RAT	60S ribosomal protein L17	Rpl17	3	10.1	34.214	306	0	2.5879	3.090794299	3.680466507	3.666762669	3.346300477	4.96954043	6.041219648
tr|F1LN59|F1LN59_RAT E	Four and a half LIM domai	Fhl2	3	3.4	102.11	906	0	2.2467	9.057760462	8.631132007	9.727846709	9.176425111	10.28318384	11.91049294
tr|M0R671|M0R671_RAT T	Guanine nucleotide-bindin	Gna11	2	2	151.85	1400	0.0026327	1.5801	2.510440565	2.10891273	2.790617784	2.58964255	2.757599391	3.165917325
tr|D3ZCZ9|D3ZCZ9_RAT N	Uncharacterized protein	Chmp4b	9	65.5	13.04	116	0	292.43	79.33016107	95.70487587	82.67256228	78.06168084	78.75771373	75.82577558
sp|Q5U316|RAB35_RAT R	Ubiquitin-conjugating enzy	Ube2m	3	17.9	23.025	201	0	24.45	3.441016008	2.665439295	4.010184661	2.593809312	2.688713377	2.891059795
sp|Q4G061|EIF3B_RAT E	Actin-related protein 2/3 co	Arpc2	3	5.3	90.91	797	0	2.6905	5.858957214	5.685598281	6.576321729	5.558286821	5.781649624	5.429911865
sp|Q66H61|SYQ_RAT Glu	Proteasome (Prosome, ma	Psmd3	4	6.6	87.699	775	0	7.8368	2.542974822	2.846271189	2.591527261	2.893859575	2.814382924	3.741090709
tr|D3ZZN3|D3ZZN3_RAT A	Guanine nucleotide-bindin	Gnai3	18	33.1	74.891	682	0	257.1	99.0580415	98.93911281	101.5084209	128.3102257	96.19182845	131.9572778
sp|P51146|RAB4B_RAT R	40S ribosomal protein S27	Rps27	2	8.9	23.629	213	0	3.4844	5.214252633	4.884173394	5.883719146	5.787892766	6.026054324	5.619822763
tr|G3V6Y6|G3V6Y6_RAT	RCG29001 (Slc25a24 prot	Slc25a24	23	31.9	96.774	846	0	211.13	99.14575641	82.18766798	91.08535405	89.55933958	106.0177348	113.171497
tr|A0A0G2K4U2|A0A0G2K	Protein SET (Liver regener	Set	2	11.5	25.661	218	0	9.5844	4.341090585	4.031571463	3.517039057	3.482024066	3.670126469	3.546303721
tr|M0R9K1|M0R9K1_RAT	Ribosome maturation prote	Sbds	3	10.2	50.969	463	0	35.594	3.777522295	4.472283808	4.806127946	3.761761366	3.776497465	4.026521656
tr|D4A4H1|D4A4H1_RAT (	FUN14 domain-containing	Fundc1	2	5.9	40.294	372	0	3.6789	8.432991088	10.18451698	9.565035369	10.32445483	11.60987745	9.545332353
sp|P30835|PFKAL_RAT A	Ragulator complex protein	Lamtor1	4	6.8	85.338	780	0	34.054	10.30889403	11.58760506	9.587022753	11.93082837	11.61053163	17.75872206
tr|A0A096MKH2|A0A096M	Extended synaptotagmin 2	Esyt2	2	11	33.975	309	0	21.763	2.38070224	2.564131579	2.254858538	2.639600289	2.646321983	2.408634787
sp|Q5U2R7|MESD_RAT L	Glutathione hydrolase 5 pr	Ggt5	2	15.2	25.215	224	0	45.188	2.725621185	2.504012822	2.036136422	1.968491193	2.207623397	2.094871187
sp|Q91ZW6|TMLH_RAT Tr	Actin-related protein 2/3 co	Arpc4	6	17.3	49.567	421	0	11.755	11.97428119	12.36620004	13.63374835	14.81848121	12.37266085	15.15027021
tr|D3ZYL4|D3ZYL4_RAT M	Nudix hydrolase 8 (RCG47	Nudt8	8	65.4	18.319	159	0	95.175	11.54088979	10.95645324	12.73540668	13.09144519	12.21369313	12.505376
tr|M0R6K0|M0R6K0_RAT	40S ribosomal protein S19	Rps19	15	10.5	196.53	1801	0	32.885	24.60323455	25.66652381	24.36359154	24.94024044	25.79529371	27.18754151
tr|G3V7W7|G3V7W7_RAT	Acidic leucine-rich nuclear	Anp32b	10	13	109.19	964	0	98.676	24.52987299	37.66601846	25.33103642	18.7278588	22.5171568	25.45370633
sp|Q6P791|LTOR1_RAT F	Desmoglein 2	Dsg2	3	30.4	17.721	161	0	19.127	8.549412694	5.186194049	7.362108937	4.966259395	5.8437975	5.520761133
sp|Q5XIA8|GHITM_RAT G	Mitochondrial antiviral-sign	Mavs	5	12.1	37.178	346	0	23.132	25.36196852	29.07769253	29.54109687	26.18723076	25.97519546	25.38800743
tr|Q5FVL8|Q5FVL8_RAT A	Acyl-CoA thioesterase 11	Acot11	10	17.8	77.393	715	0	44.121	26.42770466	29.28221046	27.91245995	29.34962946	28.28840483	30.1090897
tr|G3V8G5|G3V8G5_RAT	Oxidoreductase NAD-bindi	Oxnad1	11	10.9	133.55	1171	0	37.893	30.81823456	30.96020868	30.42216274	27.40557459	29.54837124	30.64238003
sp|Q62991|SCFD1_RAT S	Ras-related protein Ral-B	Ralb	2	4.7	72.262	637	0	10.451	2.830719594	2.681562917	2.811924606	2.796808745	2.604192265	2.502769
tr|G3V7U0|G3V7U0_RAT	Xanthine dehydrogenase/	Xdh	6	48.5	20.862	194	0	116.27	27.67883823	19.524803	30.20176539	30.18992645	33.89545163	28.84746541
tr|A0A0G2K7K2|A0A0G2K	Rho-related GTP-binding p	Rhob	23	45.6	66.133	608	0	323.31	261.0036857	298.5153128	257.9224796	306.3915547	291.1660705	283.1212199
sp|Q08163|CAP1_RAT Ad	Latent-transforming growth	Ltbp4	9	31.2	51.588	474	0	76.102	17.32608674	19.4196903	14.52161984	13.72948061	15.39370182	15.3591722
tr|Q5M9H7|Q5M9H7_RAT	RCG45489, isoform CRA_	Stom	2	6.8	45.745	412	0	2.7214	1.793610407	1.931267511	1.737526812	1.765187933	2.230323726	2.155334711
tr|D4AEG6|D4AEG6_RAT	Hebp1 protein (Heme bind	Hebp1	5	29.4	21.601	187	0	8.5091	8.122799274	7.93481982	9.45562196	10.05969184	8.859016192	14.00464555
tr|A0A0H2UHI5|A0A0H2UI	Sideroflexin-3	Sfxn3	5	14.2	45.481	408	0	46.518	8.870768225	7.199982164	8.348923653	11.19687062	10.56906407	24.67455838
tr|A0A0G2JVQ1|A0A0G2J	Coenzyme Q10A	Coq10a	4	7.9	47.273	417	0	103.12	85.40641204	81.04141636	70.62661713	67.66213748	67.85239695	78.60258087

sp|P32198|CPT1A_RAT C	Similar to SCO cytochrome	Sco1	10	15.9	88.124	773	0	85.91	29.99052478	29.77590721	31.43201185	27.95767055	30.79656227	27.8137342
tr|F1LNH3|F1LNH3_RAT C	Presenilins-associated rho	Parl	12	12.1	109.66	1027	0	97.995	33.90380583	35.39349139	37.87955034	34.03376435	49.99371411	45.50111135
tr|Q5PPI8|Q5PPI8_RAT R	40S ribosomal protein S12	Rps12	3	7	74.877	657	0	31.237	1.265845747	1.186584456	1.246527597	0.919725892	1.276517375	0.987690652
tr|B2RZ79|B2RZ79_RAT In	Malic enzyme	Me2	2	15	17.967	167	0	2.4326	2.852169876	2.875569571	2.649322669	4.026003519	3.09444088	2.895627922
tr|M0R979|M0R979_RAT T	UTP--glucose-1-phosphate	Ugp2	2	2.5	127.04	1144	0	21.282	9.983551456	7.18095724	11.15493261	11.88265019	10.5782227	11.37309643
sp|Q5HZE2|T120A_RAT T	Acyl-CoA thioesterase 13 (	Acot13	3	13.7	40.657	343	0	66.172	2.848222705	3.37449818	2.240514387	3.48402064	2.899100292	3.731287201
sp|B0BN93|PSD13_RAT 2	Tryptophanyl tRNA synthet	Wars2	7	20.7	42.817	376	0	17.827	17.01868586	14.04229577	14.40958889	13.67348975	14.30186176	13.12078996
sp|O35795|ENTP2_RAT E	Hydroxyacid-oxoacid trans	Adhfe1	3	4.8	54.389	495	0	3.6419	3.099486049	2.451789408	4.560392759	3.404635144	5.075911426	4.887587933
sp|Q75Q39|TOM70_RAT I	Proteasome (Prosome, ma	Psmd6	15	33.6	67.444	610	0	125.19	50.1609668	42.71333057	44.61711288	43.46019517	42.2304631	41.46935431
tr|G3V7V4|G3V7V4_RAT	Unconventional myosin-Id	Myo1d	5	5.8	99.459	872	0	22.66	12.04564922	10.8794023	13.98188192	10.50154222	11.26446609	10.95734554
tr|G3V913|G3V913_RAT H	Coatomer subunit alpha	Copa	3	19.9	22.807	206	0	323.31	17.31292951	15.4834337	14.22740771	11.5857684	15.2510888	14.51227224
sp|Q68FR9|EF1D_RAT El	DNA polymerase delta-inte	Poldip2	4	20.6	31.33	281	0	11.036	5.471018094	6.158843245	7.929592837	4.33694813	5.915954455	5.747114392
tr|D3ZWT2|D3ZWT2_RAT	ATP synthase protein 8 (A	Mt-atp8	3	11.5	30.112	262	0	11.79	7.418289073	6.656344985	7.045385912	8.06572263	7.210462005	10.7155941
tr|A0A0H2UHN6|A0A0H2U	Ribose-phosphate pyropho	Prps1	6	23.2	31.919	302	0	23.292	20.322747	28.15022753	20.05039979	19.33768177	19.36004469	17.57702352
sp|P62282|RS11_RAT 40	ATP synthase subunit s, m	Dmac2l	4	29.1	18.431	158	0	24.079	11.23867206	7.790706027	8.367246473	7.631250889	7.374009047	7.440914294
sp|Q66X93|SND1_RAT St	26S proteasome non-ATP	Psmd13	11	17.3	101.95	909	0	68.605	20.92837857	20.68532331	27.63552362	18.20484337	19.99591559	20.58736789
sp|Q68FW7|SYTM_RAT T	Threonine--tRNA ligase, m	Tars2	9	15.4	81.671	723	0	17.812	9.550160065	10.46656147	12.24330809	12.75159367	10.92886756	12.44224346
tr|D3ZYY0|D3ZYY0_RAT S	Retinal dehydrogenase 1 (	Aldh1a1	5	18.8	31.943	298	0	21.422	13.06274346	14.89366108	13.91277872	15.99776165	13.66337411	15.92017925
tr|G3V879|G3V879_RAT 5	Integrin-linked protein kina	Ilk	5	40.2	20.126	179	0	8.8976	9.009915966	9.887728181	10.38170962	10.37263302	10.506262	10.23876377
sp|Q5FWT1|FA98A_RAT F	Very low-density lipoprotei	Vldlr	2	5.8	55.07	515	0	4.2975	1.601514756	1.273908854	1.378975407	1.357583127	1.143128407	1.292164016
tr|Q6MGA3|Q6MGA3_RAT	Leucine-rich repeat-contai	LOC1009111	2	4.4	77.749	703	0	4.2585	2.290794459	1.860922857	2.316266159	2.068623691	2.670396108	2.832700689
sp|P97633|KC1A_RAT Ca	Epidermal growth factor re	Eps15	2	6.2	37.495	325	0	2.9821	2.023543079	2.505677503	2.530486096	2.080299305	2.108513889	2.110936622
tr|A0A0G2JUP3|A0A0G2J	Caspase recruitment doma	Card19	13	5.1	815.45	7496	0	14.707	9.49952464	9.426849417	12.47260509	10.37523725	15.69462838	12.6727029
sp|Q63638|SPEG_RAT St	Class I histocompatibility a	RT1-Aw2	11	5.4	354.16	3259	0	37.185	20.07036728	18.87224814	20.8817323	19.59810439	22.82070011	26.503349
sp|P20070|NB5R3_RAT N	NADPH:adrenodoxin oxido	Fdxr	14	53.5	34.174	301	0	255.9	52.54920455	52.46122557	52.97912426	60.79132065	57.63790287	51.73788789
sp|P11240|COX5A_RAT C	High mobility group protein	Hmgb1	8	41.1	16.129	146	0	121.47	264.8192842	292.0563514	303.0489669	266.8897834	271.3049177	269.7915279
sp|Q8CG09|MRP1_RAT M	Dolichyl-diphosphooligosac	Ddost	12	11.3	171.49	1532	0	49.566	34.9719342	35.3050255	34.42281953	30.08792759	34.50646338	33.84006894
tr|G3V9E4|G3V9E4_RAT	Myoferlin	Myof	2	4.1	81.367	732	0	3.0463	1.229164967	1.094075768	2.009542158	0.851278146	1.119970146	1.067042611
sp|Q5XI37|RT15_RAT 28S	Prelamin-A/C [Cleaved into	Lmna	4	17.1	29.712	257	0	4.7522	8.049437713	8.478932622	8.31437205	8.238903673	7.500921552	8.069673348
sp|P81795|IF2G_RAT Euk	Nucleolin (Protein C23)	Ncl	4	11.2	51.079	472	0	6.5197	3.18740031	2.917471964	3.367367796	1.973526031	3.212848938	2.669633953
sp|Q6PCT8|DHSD_RAT S	Dual specificity protein pho	Dusp3	2	10.7	16.976	159	0	4.5057	14.04714397	17.16285879	15.60633076	14.75901804	14.45232504	13.54988594
tr|M0R7S5|M0R7S5_RAT	B-cell receptor-associated	Bcap29	14	53	141.26	1395	0	323.31	57.06652236	53.94516957	60.45483468	55.84763121	64.74958246	72.34065397
sp|Q09326|MGAT2_RAT A	CTD small phosphatase 1	Ctdsp1	2	6.3	51.109	442	0	2.7974	2.069593406	2.533929513	2.277421781	2.360427239	2.489185985	2.663526008
sp|P12007|IVD_RAT Isova	Actin-related protein 2 (Act	Actr2	14	33	46.435	424	0	319.84	121.4492655	126.2018277	137.4944387	159.4263886	127.6059444	140.2671627
sp|P07151|B2MG_RAT Be	Endonuclease G-like 1 (Pr	Exog	2	11.8	13.72	119	0	38.039	18.07166347	13.31506808	16.50100787	15.48342697	19.57331003	22.3016987
tr|G3V8L3|G3V8L3_RAT L	Aminopeptidase (EC 3.4.1	Npepps	7	12.5	74.311	665	0	17.119	17.7853939	14.72243677	17.01352331	15.82675079	19.9344219	22.11897362
tr|Q5XIP0|Q5XIP0_RAT D	60S ribosomal protein L27	Rpl27	2	8	37.822	337	0	9.9084	4.746173983	4.055067243	4.794977202	4.984054941	4.633418548	5.07169885
sp|Q7TP52|CMBL_RAT Ca	Elongation factor 1-gamma	Eef1g	4	20.4	27.903	245	0	10.22	9.70405986	10.72862978	9.955573182	10.11958904	9.533484195	8.40124751
sp|P40241|CD9_RAT CD9	60S ribosomal protein L18	Rpl18a	5	15.5	25.215	226	0	38.07	11.44799173	10.69866553	14.49282684	8.502798597	11.5680094	10.85674409
tr|B0BN81|B0BN81_RAT 4	Protein Niban 2 (Meg-3) (N	Niban2	4	27	22.906	204	0	85.809	20.29124937	13.10341581	15.00115421	11.79584265	13.77131516	13.27477177
sp|P63086|MK01_RAT Mit	Serine/threonine-protein p	Ppp2r1b	3	10.3	41.275	358	0	2.2657	10.50386035	11.30032872	10.12414312	12.18864677	10.33878983	12.22666892
sp|Q06647|ATPO_RAT AT	ER membrane protein com	Emc4	12	55.4	23.397	213	0	323.31	469.9525599	535.4564619	469.5039308	492.936619	430.7502002	440.1005416
tr|G3V818|G3V818_RAT A	GLI pathogenesis-related	Glipr2	2	8.1	42.321	372	0	7.9578	3.203069382	2.74900627	2.953690879	3.245820749	2.951631602	3.450270399
tr|D3ZDP2|D3ZDP2_RAT N	Platelet endothelial cell ad	Pecam1	3	18.4	23.47	206	0	5.2917	4.0241208	4.702247566	4.983963999	8.795774046	21.35662698	5.309292781
tr|D3ZL85|D3ZL85_RAT C	60S ribosomal protein L14	Rpl14	10	47.4	31.129	274	0	138.56	37.41559182	37.89431754	37.84709277	38.53256514	34.07862432	32.80069172
tr|B5DFL0|B5DFL0_RAT S	Ras-related protein R-Ras	Rras	6	18	53.362	499	0	49.184	14.18031115	12.50983821	18.6751413	15.36189641	18.50698332	20.59455371
tr|A0A0G2JSH9|A0A0G2J	Prosaposin (Sulfated glyco	Psap	6	36.4	21.797	198	0	86.592	36.47305526	29.37638383	27.62138888	26.08479786	28.34401082	27.06846224
tr|Q4VBH2|Q4VBH2_RAT	26S proteasome regulator	Psmc2	3	10.6	49.821	434	0	13.19	3.267061395	3.045129198	3.453170943	3.376075463	3.493691919	3.416445728
tr|A0A0G2KAY3|A0A0G2K	Voltage-dependent calciur	Cacna2d2	3	9.8	44.267	398	0	20.317	11.0979295	7.504856557	8.585549781	10.51543142	12.26864493	25.91359867
sp|Q8R4A1|ERO1A_RAT	Magnesium transporter pro	Magt1	5	11.6	54.018	464	0	5.1983	9.767055112	10.43659721	8.968234957	10.8409597	9.874970419	10.42148885
tr|D4AE17|D4AE17_RAT N	Adiponectin, C1Q and coll	Adipoq	3	8.2	51.317	453	0	52.66	1.164096453	1.153528652	0.85180171	1.069078266	1.220257193	1.547517183
sp|P23358|RL12_RAT 60S	Saccharopine dehydrogen	Sccpdh	4	40	17.845	165	0	115.2	14.87604986	15.29461134	11.57112237	12.12788149	12.35826871	12.04445712
sp|Q5XIJ4|F210A_RAT Pr	Thioredoxin-related transm	Tmx2	5	20.9	31.474	273	0	220.96	45.14925587	58.65859428	58.25609632	49.8231879	49.2754155	43.31816257
tr|B0BN83|B0BN83_RAT A	Complex III assembly facto	Lyrm7	2	9.6	31.204	282	0	6.7507	0.267610213	0.157583438	0.231626616	0.342390642	0.238360001	0.249127169
sp|P50398|GDIA_RAT Ra	Trafficking protein particle	Trappc3	4	11.2	50.536	447	0	13.858	8.22566494	15.0877153	7.308187497	6.366030986	7.264759623	6.242422545
tr|A0A096MJS3|A0A096M	Proteasome subunit alpha	Psma1	2	2.7	82.267	774	0	2.5563	3.400547539	2.710433238	4.096773071	2.601925817	3.07560026	2.790355692
sp|Q6AYS7|ACY1A_RAT	Inactive hydroxysteroid de	Hsdl1	2	5.1	45.804	408	0	2.7742	1.112583879	0.968844211	1.216164067	0.993599109	1.530931154	1.654021267
tr|Q5U3Z7|Q5U3Z7_RAT S	FUN14 domain-containing	Fundc2	9	22.6	55.764	504	0	47.671	20.44235824	19.84299484	20.82990489	23.4881836	19.70676442	23.18093483
sp|Q9JKW1|TIM22_RAT M	Thymosin beta-4 (T beta 4	Tmsb4x	2	16.1	19.918	192	0	7.643	10.04176226	9.112938186	11.54494691	7.937681508	10.32636026	8.725122582
tr|A0A0G2K7J6|A0A0G2K	Glycerol-3-phosphate dehy	Gpd1	3	18	21.029	183	0	17.508	17.86314121	20.30197111	18.73691538	21.34163384	19.12977045	19.99351138
tr|E9PSY8|E9PSY8_RAT	5'-AMP-activated protein k	Prkag1	2	3.9	98.793	897	0	17.661	0.71599288	0.709629633	0.796990304	0.946506018	0.82669759	0.725767594
tr|B2RYP6|B2RYP6_RAT	Interferon gamma-induced	Igtp	2	6.4	46.582	392	0	5.6727	2.335489192	2.437282904	2.693192735	2.497452942	2.673405374	2.816173309
tr|B5DEL8|B5DEL8_RAT N	RCG35015, isoform CRA_	Slc25a19	8	65.1	12.7	106	0	180.94	96.2591385	130.2921861	102.7072568	100.1845825	86.60142989	97.48588341
tr|F1M748|F1M748_RAT C	Mitochondrial ribosomal pro	Mrpl51	2	2.4	134.82	1352	0.0091552	1.2138	8.856414876	5.913421736	3.856430028	14.55849263	6.552348706	8.303725698
sp|Q6AYD3|PA2G4_RAT	Translocon-associated pro	Ssr1	3	10.2	43.656	394	0	20.683	1.268875899	1.435287764	0.953048377	1.351550003	1.32545065	1.615371833
sp|Q9EPB1|DPP2_RAT D	Non-muscle caldesmon (C	Cald1	3	6.6	55.114	500	0	3.9612	8.042261039	11.1143601	11.0224848	8.418595282	9.225361567	8.233407338
tr|A0A0G2KAA3|A0A0G2K	Ras-related protein Rab-8A	Rab8a	2	38.5	10.17	91	0	38.098	34.70799207	42.70144	37.09428664	39.07077189	31.01767787	31.4908198

sp|Q63584|TMEDA_RAT T	Heat shock protein beta-2	Hspb2	4	24.2	24.857	219	0	55.474	27.30365764	28.30195129	25.24518092	23.80089147	25.27979344	27.02688715
sp|Q6P799|SYSC_RAT Se	60S ribosomal protein L31	Rpl31	2	4.1	58.587	512	0.00091158	1.8427	4.401693613	4.446790411	4.215819048	3.855166279	4.016322848	4.44417165
tr|D3ZJS3|D3ZJS3_RAT R	Amine oxidase [flavin-cont	Maob	2	56.8	7.8938	74	0	59.987	5.930723958	5.537203881	6.437591808	7.324386234	5.976008929	11.03844263
sp|P10817|CX6A2_RAT C	Carboxypeptidase Q (EC 3	Cpq	2	43.6	10.487	94	0	82.489	5.7804125	6.4794132	5.973762717	7.611285155	6.057717031	6.530881801
tr|Q3V5X8|Q3V5X8_RAT	DnaJ homolog subfamily B	Dnajb11	7	30.3	32.267	294	0	55.1	24.62954903	22.2410864	25.28863218	23.73665389	28.18700566	26.09529721
sp|Q9QYU2|EFTS_RAT E	Hsc70-interacting protein (	St13	2	6.5	35.178	324	0	5.0859	1.42823794	1.324229775	1.264012802	1.58211083	1.702655549	1.273737526
tr|G3V6P7|G3V6P7_RAT	Obscurin, cytoskeletal calm	Obscn	69	37.7	226.41	1960	0	323.31	352.3986338	279.1479411	356.1275579	245.1184522	320.7092082	299.4278935
tr|Q4V797|Q4V797_RAT I	Cystatin-B (Cystatin-beta) (	Cstb	2	7.4	48.271	417	0	11.592	3.735459009	2.640278834	3.464216986	3.08006175	4.548570343	4.25805897
tr|D3ZTN2|D3ZTN2_RAT C	Ankyrin-3 (ANK-3) (Ankyrin	Ank3	3	27.4	12.572	106	0	10.148	9.826860732	9.268942555	7.055332585	10.98636233	10.95830603	8.493636596
tr|A0A140TAH5|A0A140TA	Histidine triad nucleotide-b	Hint3	8	22.4	49.526	434	0	38.321	28.52847673	27.36164446	28.9699484	23.61902967	28.48858641	26.76049862
tr|A0A0G2K2B9|A0A0G2K	Cell division control protein	Cdc42	6	4.7	202.06	1848	0	14.61	8.909043821	9.124828763	10.73560179	9.561850591	10.91316704	10.98300918
tr|G3V7E8|G3V7E8_RAT	Charged multivesicular bod	Chmp6	2	2.1	134.01	1221	0	2.3982	3.874925714	3.483320741	3.552899433	3.939022364	3.83871076	5.775344391
tr|A0A0G2JYN3|A0A0G2J	Growth arrest and DNA da	Gadd45gip1	14	18.9	115.48	1011	0	142.9	47.90031437	45.26171903	46.23894417	49.76242262	46.34726925	47.84882067
tr|F1LXA0|F1LXA0_RAT N	Early endosome antigen 1	Eea1	12	80	17.177	145	0	323.31	218.7530089	238.7104669	219.6225515	227.5442657	206.2982393	209.9080023
tr|Q66HF3|Q66HF3_RAT E	Cytosolic non-specific dipe	Cndp2	29	54.5	68.163	616	0	323.31	429.6834427	507.2520133	461.6984097	455.7829916	435.8855774	409.3452416
sp|P38983|RSSA_RAT 40	Collagen type IV alpha 2 c	Col4a2	9	40	32.824	295	0	172.55	34.31168016	18.81897835	24.35992698	18.81206211	19.8461065	19.88675066
tr|Q6IRJ7|Q6IRJ7_RAT An	60S ribosomal protein L13	Rpl13	2	5.2	50.019	463	0	7.2114	2.252518862	2.695879172	1.868875254	2.118103989	3.021564318	4.241274953
sp|Q4FZY0|EFHD2_RAT E	EFR3 homolog A (Similar t	Efr3a	2	11.7	26.759	239	0.008761	1.2333	2.081594401	2.103490627	2.244283653	3.15224222	2.318835386	2.201734563
sp|P20059|HEMO_RAT He	Alpha-2-macroglobulin rece	Lrpap1	18	40.7	51.35	460	0	127.42	32.97681873	27.2222869	33.74539871	30.97987507	38.31122177	57.83043481
sp|P05508|NU4M_RAT NA	Protein-L-isoaspartate(D-a	Pcmt1	5	13.5	51.782	459	0	29.14	52.36181361	55.85717435	52.60219768	53.88144042	49.17924983	51.42992427
sp|Q9ER30|KLH41_RAT K	ATPase H+-transporting V	Atp6v1d	2	3.3	68.212	606	0	11.089	5.41081377	4.288550613	4.810525423	4.677190285	6.007540799	5.668070397
tr|F1LWK7|F1LWK7_RAT	Isocitrate dehydrogenase	Idh1	7	13.8	67.072	600	0	25.33	19.20876765	13.35692292	21.85441227	18.70181654	21.0844847	27.8250262
sp|P26376|IFM3_RAT Inte	Alpha-2-HS-glycoprotein (5	Ahsg	2	20.4	14.971	137	0	10.998	8.077745707	7.644214119	10.27648428	6.413775133	8.655563671	7.193003581
tr|D3ZWC6|D3ZWC6_RAT	Eukaryotic translation initia	Eif5a	17	41.9	58.292	539	0	140.9	32.90505198	28.79136744	37.61779577	33.09537483	41.33357112	37.24358019
tr|Q5XI86|Q5XI86_RAT Pe	Methanethiol oxidase (MTO	Selenbp1	2	17.1	19.529	181	0	7.3468	7.023970687	7.628042934	6.55119329	9.913855168	6.659635566	8.477725142
tr|D3ZEJ2|D3ZEJ2_RAT M	Rab GDP dissociation inhib	Gdi1	2	6.8	37.551	336	0	3.9037	2.743921704	2.437330466	3.315540392	2.800671681	3.936184797	3.245987865
tr|A0A0G2K0D7|A0A0G2K	Glycogen synthase kinase	Gsk3b	5	27.8	25.318	227	0	7.4515	6.472164169	6.314371991	6.634431242	5.115568364	6.651785308	5.850282204
tr|E9PT65|E9PT65_RAT R	Ribosome-releasing factor	Gfm2	6	11.5	68.485	583	0	45.5	28.71905731	23.8505949	31.60267583	21.48573436	27.92663877	24.4240813
tr|G3V615|G3V615_RAT C	Choline transporter-like pro	Slc44a2	2	2.4	85.352	763	0.00091075	1.838	3.558274893	2.952858321	3.369461833	2.950588303	4.509907822	4.582036697
tr|F1LRV4|F1LRV4_RAT H	Mitochondrial ribosomal pro	Mrps2	16	28.8	94.025	840	0	99.957	23.01280377	21.2456073	21.93293864	19.74741336	21.94277958	21.51074547
sp|Q80W89|NDUAB_RAT	Mitochondrial import recept	Tomm20	4	36.2	14.854	141	0	147.2	50.53574868	53.8262638	49.17635392	48.13044086	45.97176524	51.7584188
tr|Q6IRG9|Q6IRG9_RAT A	Abhydrolase domain-conta	Abhd11	2	7.9	46.977	418	0	3.208	3.809538237	3.305295022	3.897682548	3.55989043	4.194196611	5.209717878
sp|Q9WTT7|BZW2_RAT B	Nucleosome assembly prot	Nap1l1	4	10.3	48.049	419	0	8.5442	15.44579806	14.80804893	16.15967992	12.76374672	14.6649362	15.58295909
tr|G3V624|G3V624_RAT C	NADH dehydrogenase (Ub	Ndufaf1	7	15.4	53.177	474	0	26.136	12.11303022	9.321736717	12.00458793	7.407287435	11.09503136	9.960056692
tr|C0JPT7|C0JPT7_RAT F	NAD-dependent protein de	Sirt5	39	24.3	280.49	2639	0	323.31	96.08769572	81.52179567	103.2045905	93.55248645	110.5120075	105.1541775
sp|Q3T1J1|IF5A1_RAT Eu	Serine/threonine-protein ki	Stt3a	4	29.9	16.832	154	0	13.934	8.781059795	9.712698888	10.04666377	7.956779167	8.664068118	9.701880525
sp|Q6AXS5|PAIRB_RAT F	Tyrosine-protein kinase Ly	Lyn	2	6.4	44.754	407	0	11.332	5.758085069	3.576400177	4.874079432	4.00920626	3.883195556	3.773067598
tr|Q5XI34|Q5XI34_RAT Pr	Aspartyl aminopeptidase (A	Dnpep	6	12.6	65.322	589	0	16.512	13.36815083	11.32220739	12.0600799	12.07883523	14.8912853	13.32455922
tr|Q5XI04|Q5XI04_RAT R	ER membrane protein com	Emc1	3	17.3	31.378	284	0	18.541	15.02038075	12.35335822	13.16573119	13.23945205	15.67042341	15.44232237
sp|B3DMA2|ACD11_RAT A	Ras-related protein Ral-A	Rala	2	2.4	87.37	779	0.0022017	1.6064	2.935020595	2.252646025	1.842961552	2.717162827	4.321894142	2.409558678
tr|D3ZTW7|D3ZTW7_RAT	Unconventional myosin-XV	Myo18a	6	26.5	34.192	298	0	26.703	10.43807417	12.30389342	12.11557186	14.35840124	11.16045017	13.25270105
sp|O35303|DNM1L_RAT D	ARP1 actin-related protein	Actr1b	11	19.5	83.907	755	0	36.175	18.99227131	17.33027811	18.3081614	16.47910946	23.39507732	26.4263581
tr|A0A0G2K2P6|A0A0G2K	Sodium-coupled neutral ar	Slc38a2	3	7.7	74.799	679	0	6.7431	3.113560305	3.696637691	3.466520426	4.16910575	3.292659895	3.898973391
sp|P16391|HA12_RAT RT	Agrin [Cleaved into: Agrin	Agrn	3	12.4	41.83	371	0	151.03	49.62271622	39.56851078	42.44350296	43.49925857	48.30983376	56.27521854
sp|P62193|PRS4_RAT 26	5'-AMP-activated protein k	Prkaa2	2	7	49.184	440	0	43.521	2.915484092	2.289316564	2.763604713	2.399620843	2.274285171	2.545319306
tr|A0A096MJA9|A0A096M	Proteasome subunit beta t	Psmb5	9	16.3	74.889	657	0	82.018	22.61848539	22.84465209	21.9936657	20.76045736	20.37599892	23.04081138
tr|B5DEP4|B5DEP4_RAT	tRNA nucleotidyl transferas	Trnt1	2	18.4	16.564	141	0	5.6815	5.26608417	6.591660246	5.650757582	6.475842525	5.780537504	7.174012491
tr|G3V8F5|G3V8F5_RAT M	Cell cycle progression 1 (C	Ccpg1	9	37.7	37.92	361	0	209.67	55.52752441	54.72519142	51.93315301	57.87458728	54.00650434	55.61309676
tr|B5DFG4|B5DFG4_RAT	RGD1563250 (Predicted),	Tmem205	3	29	18.618	169	0	3.5207	2.071188223	1.804846896	2.153716573	1.597171939	1.954910507	1.942737159
sp|P61805|DAD1_RAT Do	Diacylglycerol O-acyltransfe	Dgat1	2	19.5	12.497	113	0	2.5001	4.256166605	4.018824765	4.345125804	4.09657805	4.058387147	4.08472678
sp|Q5XII9|MFR1L_RAT Mi	Mitofusin-2 (EC 3.6.5.-) (Mi	Mfn2	3	14.9	31.73	289	0	8.8679	3.40166391	3.428576524	4.045940334	3.812717392	4.723827354	3.686735131
tr|Q6P9V6|Q6P9V6_RAT	Family with sequence simil	Fam126a	2	15.8	26.411	241	0	81.35	4.656864257	3.560324117	3.630535837	3.503812759	3.623940784	3.345819405
tr|Q6AYS2|Q6AYS2_RAT	Inositol polyphosphate-5-p	Inpp5a	2	7.5	35.588	322	0	4.817	2.301399989	2.596854447	2.584826344	3.249379858	8.649675978	2.847174979
sp|Q32Q90|APOP1_RAT	Quinone oxidoreductase (I	Cryz	2	13	22.769	193	0.00047214	2.115	2.546204325	2.366938251	1.986193651	2.124918381	2.148092274	1.986365339
tr|F1LNV5|F1LNV5_RAT C	Mitochondrial ribosomal pro	Mrpl1	2	7.1	54.226	477	0	6.109	1.140812132	1.382969226	1.487812956	1.054538003	0.976964612	1.409600809
tr|Q66WT9|Q66WT9_RAT	Integrin subunit alpha 2b	Itga2b	2	3.7	64.713	597	0	11.573	1.942327048	1.732647313	2.240776142	1.212614534	1.931163477	1.844137474
sp|Q68FV8|UBTD1_RAT U	Cytoplasmic FMR1-interact	Cyfip1	3	17.6	25.958	227	0	6.7195	4.880935979	3.715900426	4.358579989	4.576927575	4.757256369	4.920694022
sp|P68136|ACTS_RAT Ac	Tumor susceptibility gene 1	Tsg101	2	5.8	42.051	377	0	52.618	4.010963564	2.621681971	3.893494475	2.890213659	3.302276461	2.60747663
sp|Q63135|CR1L_RAT Co	Murinoglobulin-1 (Alpha-1	Mug1	5	13.1	61.68	559	0	19.765	9.588834366	7.516747134	8.015448335	9.410371433	9.327414921	8.776449852
tr|G3V837|G3V837_RAT C	Nicalin (Nicastrin-like protei	Ncln	3	10.4	38.593	336	0	6.5518	5.228605982	3.865436322	5.790011011	5.06348384	6.250637122	5.862087476
sp|Q9QWJ9|NRP1_RAT N	G-rich RNA sequence-bind	Grsf1	4	5.9	103.08	922	0	8.5251	3.911247661	2.597710568	3.056769828	2.700929817	2.791682595	2.607271321
sp|Q63570|PRS6B_RAT 2	Eukaryotic translation initia	Eif3j	3	8.9	47.408	418	0	4.5862	4.376973957	4.29221291	4.082062465	3.988589469	3.901447406	4.331251657
sp|P11730|KCC2G_RAT C	SEC63 homolog, protein tr	Sec63	2	4.7	59.037	527	0	3.6487	1.982636035	1.775738764	2.058071455	1.809372971	2.284228832	2.509236236
tr|D4A414|D4A414_RAT C	Integrin alpha-1 (CD49 ant	Itga1	6	17.9	45.914	414	0	25.843	29.58265045	37.18088292	29.68558539	30.25025769	29.33706846	27.35384186
tr|A0A0G2K3S4|A0A0G2K	Heat shock 70 kDa protein	Hspa1b	3	2.6	183.23	1623	0	4.7341	6.185894602	3.889788224	4.414700168	3.540922983	4.406938604	3.919606953
tr|G3V9E3|G3V9E3_RAT	GTP-binding protein SAR1	Sar1b	4	11.9	60.611	531	0	15.06	5.199899285	4.045554782	5.282730661	3.951826476	4.702697076	4.326426893
tr|B0BN68|B0BN68_RAT N	Bis(5'-adenosyl)-triphospha	Fhit	9	33.1	45.01	390	0	89.017	17.94168592	17.91814824	16.17486168	19.79038309	16.80609408	18.9967358

tr|A0A0G2K0I6|A0A0G2K	Cadherin 5	Cdh5	5	8.4	82.043	730	0	12.441	5.265286762	4.777158201	7.736417967	5.042215993	4.56531756	4.742845033
tr|M0R3X6|M0R3X6_RAT	Microtubule-associated pro	Mapre1	7	40.1	30.611	277	0	43.822	11.80004748	11.8087698	11.48840792	11.53107965	12.14042405	12.3832171
sp|P16617|PGK1_RAT Ph	Protein phosphatase (EC 3	Pptc7	18	55.9	44.538	417	0	317.09	91.86143193	86.56815653	72.9981135	72.50599824	82.44079313	74.87108836
sp|P09605|KCRS_RAT Cr	Nicotinamide-nucleotide ad	Nmnat3	10	34.1	47.385	419	0	323.31	1064.260939	1086.656048	792.7498801	815.253017	898.9199638	877.029058
sp|Q62667|MVP_RAT Maj	Mitochondrial inner membr	Immp1l	10	15.2	95.797	861	0	36.383	19.2422588	20.30910546	18.42542744	18.7682243	21.01448657	25.03230945
tr|A0A0G2KB58|A0A0G2K	Sterol 26-hydroxylase, mito	Cyp27a1	3	2.4	194.14	1702	0	3.2548	2.388556712	2.823964467	2.674712862	2.371364989	2.689367565	2.203890308
tr|D4A3D2|D4A3D2_RAT F	FAD synthase (EC 2.7.7.2	Flad1	3	7.6	56.553	490	0	7.4346	5.497731271	5.375492034	6.09835789	5.192827076	7.255600989	6.895818689
tr|A0A0U1RS13|A0A0U1R	RNA-splicing ligase RtcB h	Rtcb	5	4.7	174.93	1646	0	9.9774	4.431596423	4.677229792	4.898160852	4.396367891	5.3549881	5.776370936
sp|Q66HF1|NDUS1_RAT N	MICOS complex subunit M	Chchd6	45	74.7	79.411	727	0	323.31	1219.715679	1379.639864	1226.581899	1281.973759	1135.278149	1189.150185
sp|P13832|MRLCA_RAT M	Platelet-activating factor ac	Pafah1b1	3	29.1	19.895	172	0	71.877	23.27435368	17.38925538	22.40304984	16.55853836	20.71879352	18.57687874
sp|Q66H98|CAVN2_RAT C	60S ribosomal protein L27	Rpl27a	7	21.8	46.386	417	0	64.441	8.703711193	8.712463553	9.855582938	9.485893993	11.4574516	9.091599288
tr|Q6PDW2|Q6PDW2_RAT	Proteasome (Prosome, ma	Psmd7	3	21.9	18.579	160	0	7.2027	7.48646748	6.154087014	6.683117592	5.747961297	6.309906571	6.716686518
sp|Q3SWT0|PECA1_RAT	Nodal modulator 1 (Similar	Nomo1	5	9.9	76.188	678	0	16.211	9.613155318	8.8247106	10.30684781	8.469811731	9.54591377	9.019741111
tr|A0A0G2K2V2|A0A0G2K	Collagen beta(1-O)galacto	Colgalt1	5	6.6	103.82	942	0	6.6831	9.106402366	12.87749485	35.3326784	7.127767154	8.896304918	11.49987478
tr|D3ZMX6|D3ZMX6_RAT	RNA transcription, translati	Rtraf	9	23.2	56.525	522	0	22.875	19.21833655	16.18117276	19.90015267	17.30334706	22.90116525	21.75198364
tr|Q4G067|Q4G067_RAT	Adaptor-related protein co	Ap3b1	6	24.4	37.44	332	0	91.338	22.29314282	20.93930603	22.08685032	25.00751628	22.12922321	21.87568236
sp|P13233|CN37_RAT 2,3	Fat storage-inducing trans	Fitm2	6	12.9	47.268	420	0	20.183	19.10032013	17.81255992	20.12840265	16.34542585	19.43265958	17.68019133
sp|P62076|TIM13_RAT Mi	Trimethyllysine dioxygenas	Tmlhe	4	53.7	10.458	95	0	29.341	17.18414807	19.40827534	15.27233194	18.96484338	17.56560654	19.8220783
tr|A0A0G2JXB1|A0A0G2J	NAD kinase 2, mitochondri	Nadk2	2	8.7	26.335	252	0	4.6739	1.430909258	1.155050646	1.203861602	1.15397604	1.532632044	2.114067586
tr|F1MAC0|F1MAC0_RAT	Transmembrane 9 superfa	Tm9sf3	2	4.8	47.432	420	0.00090868	1.81	1.269115121	1.269675808	1.22438316	2.1297362	0.987758717	1.909374434
tr|A0A0G2K7Q7|A0A0G2K	Prolactin regulatory elemen	Preb	3	4.1	113.16	978	0	5.6121	1.447694702	1.495216272	1.697897171	7.79184484	1.545127038	2.339137664
tr|D4A104|D4A104_RAT M	Mitochondrial uncoupling p	Ucp3	6	28.1	35.439	306	0	39.996	9.987139793	9.832555904	11.96898931	11.61788719	11.52417879	11.11030081
sp|P59215|GNAO_RAT G	Eukaryotic translation initia	Eif4g1	6	20.1	40.068	354	0	59.044	18.64340519	16.39520314	18.21445326	17.32157664	21.48223111	18.96131999
tr|D3ZD09|D3ZD09_RAT C	Death-associated protein 3	Dap3	9	70.9	10.071	86	0	323.31	382.7719145	424.5126226	176.5586902	412.9608319	386.9588442	457.7776533
sp|P62909|RS3_RAT 40S	Interferon-gamma-inducible	RGD1309362	9	46.1	26.674	243	0	50.555	27.62900021	20.30815421	23.16161457	20.56817866	22.68004965	20.20908591
sp|B2RYG6|OTUB1_RAT	Phosphatidylinositol-bindin	Picalm	4	20.3	31.27	271	0	5.9649	4.336306136	4.515280134	4.25780448	4.264290218	4.607316441	5.013083108
sp|Q00238|ICAM1_RAT In	Transmembrane emp24 do	Tmed7	2	5	60.141	545	0	4.0545	2.582247179	2.355142798	2.64016126	2.341459791	2.922847323	3.379490094
sp|Q9QZ76|MYG_RAT My	60S ribosomal protein L22	Rpl22	7	59.7	17.157	154	0	128.51	116.3538267	104.3802408	104.7384722	86.33877983	114.5090972	94.3446545
tr|D3ZMR9|D3ZMR9_RAT	Proteasome subunit alpha	Psma4	3	20.1	23.39	209	0	77.855	3.673420646	4.063057711	4.599655944	3.900740238	3.695836064	3.789851615
tr|F7EZZ0|F7EZZ0_RAT D	ATP-dependent RNA helic	Ddx1	6	17.9	45.111	396	0	11.611	14.76680492	15.61993752	15.29798388	21.562125	14.99268447	68.26013602
sp|Q68FP1|GELS_RAT G	Mitochondrial import inner	Timm10b	7	15.3	86.067	780	0	75.309	17.37273513	16.60067231	18.24848136	13.97818422	17.92213909	16.24148796
sp|Q5M7W7|SYPM_RAT F	m-AAA protease-interactin	Maip1	5	17.7	53.332	475	0	59.512	10.43448583	7.621384211	10.30946536	8.801416536	11.10091905	14.65547533
tr|F8WFF9|F8WFF9_RAT L	Mannose-P-dolichol utilizat	Mpdu1	3	18.9	20.898	190	0	5.128	3.579884657	3.095164746	3.281617	3.420998365	3.978445353	3.329394679
tr|G3V9Y1|G3V9Y1_RAT	Myeloid-derived growth fac	Mydgf	12	8.5	229	1976	0	24.906	23.91546993	20.43134059	25.76607251	21.8603089	26.91460967	31.57602307
sp|Q6AY41|CC50A_RAT C	Transmembrane protein 18	Tmem186	2	7.3	37.172	328	0	2.9506	2.372130102	2.270719702	2.665760856	2.248488915	2.84172799	3.249426792
tr|G3V804|G3V804_RAT S	Plasminogen activator inhi	Serbp1	5	20.6	35.451	321	0	18.476	16.67101586	19.50244871	19.32010455	22.51570583	19.01070821	60.52511647
sp|Q6AYS8|DHB11_RAT I	ATP-binding cassette sub-	Abcc9	2	5.4	32.937	298	0	2.6789	1.713151913	1.631006661	2.012212054	1.406368964	1.719271928	1.744049298
tr|A0A0G2JT93|A0A0G2J	Alpha-adducin (Erythrocyte	Add1	5	8.6	85.47	781	0	35.558	10.2769977	9.244210155	10.72722564	10.11611674	10.40420865	13.07972815
tr|G3V6W6|G3V6W6_RAT	Phenylalanine--tRNA ligase	Farsa	3	9.2	45.796	403	0	6.635	9.866731145	10.0199514	8.52586974	10.19424352	9.834410753	10.03396796
tr|M0RDA4|M0RDA4_RAT	Protein kinase, cAMP-depe	Prkaca	2	2.3	108.73	987	0.0047434	1.4449	1.674198519	1.608367003	1.780297509	1.640185075	1.798101603	2.151844456
tr|D3ZML4|D3ZML4_RAT S	Cytochrome P450 4B1 (EC	Cyp4b1	6	24.2	42.114	376	0	27.419	5.21584745	5.492019688	5.816186468	6.122535835	5.274392117	6.201874002
tr|Q5RK17|Q5RK17_RAT	Sepiapterin reductase (SP	Spr	4	19.4	26.906	237	0	46.174	3.063443195	2.547151835	2.501954848	2.768552891	3.273099669	2.806729091
tr|D3ZDX5|D3ZDX5_RAT F	Kelch-like protein 41 (Kel-li	Klhl41	3	1.7	231.81	2121	0	6.3123	1.041654414	0.905919278	1.32866618	1.046595113	1.486969709	1.281847234
tr|Q6P9V7|Q6P9V7_RAT	NAD(P)(+)--arginine ADP-ri	Art4	10	42.2	28.635	249	0	22.733	34.83438128	31.32596282	31.34772688	28.00671681	31.62934381	32.43472829
tr|D4A133|D4A133_RAT A	Tight junction protein ZO-1	Tjp1	6	11.8	68.264	617	0	7.9287	10.6924474	9.398787656	10.0367171	8.031867689	9.254800035	10.39890485
sp|Q7TPB1|TCPD_RAT T-	Elongation factor 1-delta (E	Eef1d	6	15.2	58.099	539	0	23.161	14.47575091	16.96357272	12.61447607	15.39184501	16.18854044	16.82507902
tr|F1M9Q8|F1M9Q8_RAT	RCG40784 (Transmembra	Tmem242	6	15.1	71.575	644	0	49.825	11.25661375	12.10746109	10.41259666	10.94078837	10.79083386	10.35322358
sp|P63012|RAB3A_RAT R	Interferon-induced transme	ifitm3	4	26.8	24.97	220	0	21.804	12.351854	9.263235078	9.894846123	10.01151365	9.001629213	9.259952733
tr|R9PXU6|R9PXU6_RAT	Stromal interaction molecu	Stim1	38	44	116.57	1066	0	280.12	157.874875	130.0068123	168.3867126	142.0996035	181.1643298	188.0015236
sp|Q91XU8|CDS2_RAT Ph	DnaJ heat shock protein fa	Dnajc19	3	12.6	51.322	443	0	66.915	9.449287919	8.006163282	9.676019305	9.24283288	10.24589511	9.494518356
tr|A0A0G2JSS9|A0A0G2J	NADH dehydrogenase [ub	Ndufb1	5	11.2	60.249	536	0	7.9631	7.405929245	6.873229109	8.946771083	6.071753424	7.720074589	7.109853404
sp|O08730|GLYG_RAT G	GTPase IMAP family mem	Gimap4	7	27	37.378	333	0	43.694	9.318911668	7.781193566	9.639897175	8.50410071	9.85338221	11.40440606
tr|O55158|O55158_RAT T	Wolfram syndrome 1 homo	Wfs1	2	12.8	25.545	235	0	9.4125	3.239790033	3.036187484	6.000461682	2.355305594	3.371685826	3.355366277
tr|A0A0G2JXT0|A0A0G2J	Protein tyrosine kinase 9-li	Twf2	2	8	34.579	299	0	2.8753	0.543353991	0.565135342	0.655485785	0.657089678	0.560279458	0.711806577
tr|A0A0G2K7L0|A0A0G2K	Dipeptidase 1 (EC 3.4.13.1	Dpep1	3	25.9	23.065	201	0	4.6678	7.833738778	7.827329004	8.805947126	8.494985918	8.045860297	9.599739259
tr|D3ZY44|D3ZY44_RAT M	Beta-adducin (Adducin-63	Add2	2	8.6	32.175	291	0	13.689	4.274108291	4.692069233	4.412867886	5.462364489	4.23907392	4.850580972
sp|P97675|ENPP3_RAT E	COP9 signalosome comple	Cops4	10	15.7	99.071	875	0	19.481	16.11362748	15.62088877	17.55221421	16.97608263	18.17530991	53.02106964
tr|B5DF63|B5DF63_RAT S	NSF attachment protein ga	Napg	21	24.3	86.02	749	0	150.23	97.85794207	95.9569561	110.057325	79.77178939	107.8560035	87.67724216
tr|D3ZKG5|D3ZKG5_RAT	Cat eye syndrome chromos	Hdhd5	4	18.4	41.701	365	0	35.731	7.698976782	7.907709304	8.911172462	7.368658079	9.99272429	10.20026831
tr|Q4KLZ0|Q4KLZ0_RAT V	GTP-binding nuclear prote	Ran	6	18.9	57.013	512	0	33.655	30.0311926	22.82324905	26.00688671	25.9758544	19.64069141	24.92965491
tr|A0A140TAB9|A0A140TA	LIM domain and actin-bind	Lima1	3	18.4	21.192	196	0	14.141	4.848640945	5.80593092	7.300858369	6.626453608	6.792435764	6.874261236
sp|Q6AXT5|RAB21_RAT F	Guanine nucleotide-bindin	Gng10	10	44.8	24.163	223	0	45.462	37.2784376	36.10787726	27.46590666	37.06117732	37.95926854	35.95885863
sp|Q5XIG4|OCAD1_RAT O	Hydroxyacylglutathione hy	Hagh	12	61.5	27.659	247	0	299.21	61.80312744	66.60625592	58.16709977	64.50668338	56.9451176	52.82089328
sp|Q0H8B9|CL2DB_RAT O	Sphingosine 1-phosphate	S1pr1	3	15	23.537	207	0	3.9973	7.443008729	6.233040445	8.657794041	6.76968605	9.832448188	9.110077105
sp|P17220|PSA2_RAT Pro	Spectrin alpha chain, non-	Sptan1	2	15	25.926	234	0.00046211	1.9442	1.205721164	1.909531536	2.029540207	2.03741638	1.883603997	2.062021734
sp|A0JPQ4|TRI72_RAT Tr	CDP-diacylglycerol--glycero	Pgs1	22	64.2	52.831	477	0	323.31	282.1748751	230.3680381	319.4819186	186.2455782	272.8030086	264.5869428
tr|A0A0G2JU96|A0A0G2J	T-kininogen 1 (Alpha-1-MA	Map1	82	46.5	571.85	5364	0	323.31	227.7318259	198.3490925	262.0948474	181.9572857	234.847011	202.3166991

tr|Q6IRK8|Q6IRK8_RAT S	Sec1 family domain-contai	Scfd1	2	1.4	282.38	2452	0	10.508	4.296435722	1.897545834	5.463341312	2.294236489	3.25092269	2.708488697
tr|D4A8F2|D4A8F2_RAT R	Membrane-associated prog	Pgrmc1	4	15.5	31.337	277	0	4.6799	11.13979344	10.32006956	11.62347328	10.15604821	12.45639694	15.15078348
tr|Q4V7D9|Q4V7D9_RAT	Alpha-methylacyl-CoA race	Amacr	2	5.3	51.645	456	0	6.7467	5.147669043	4.658775617	5.018096794	3.546218243	5.235075408	4.45977514
sp|P61765|STXB1_RAT S	Microtubule-associated tur	Mtus1	6	16.8	67.568	594	0	42.482	10.45362363	8.592130915	10.19429335	8.846990495	9.14555061	9.366200181
tr|B0BMT9|B0BMT9_RAT	Isochorismatase domain-co	Isoc1	11	31.6	50.201	450	0	67.671	24.1152207	28.75950069	21.8392305	24.44326727	55.38357043	26.89908225
sp|B5DFC9|NID2_RAT Nid	Valine--tRNA ligase (EC 6.	Vars1	2	1.9	152.97	1396	0	4.3004	1.83443771	1.889365117	2.08759737	1.834807581	2.109364334	2.995716098
tr|D4A8D5|D4A8D5_RAT R	Carboxymethylenebutenoli	Cmbl	28	15.5	275.31	2578	0	165.48	57.45725241	46.75374862	51.36881017	50.92998404	57.07857198	58.74406021
tr|Q5I0M1|Q5I0M1_RAT A	Multiple inositol polyphosp	Minpp1	14	46.1	38.457	345	0	67.228	59.18762834	53.37442188	56.0311825	63.67767137	55.34889846	58.11273479
sp|Q62920|PDLI5_RAT PI	COX assembly mitochondr	Cmc1	4	8.5	63.201	591	0	3.8116	3.452857521	4.284888315	3.112104743	3.794835039	4.19812174	3.977504114
sp|Q64303|PAK2_RAT Se	Coronin-1A (Coronin-like p	Coro1a	2	5.5	57.96	524	0	5.0094	0.91482663	0.869106051	0.932893275	0.832397506	1.293330011	1.017306486
sp|P51635|AK1A1_RAT A	Serine protease inhibitor A	Serpina3l	6	24	36.505	325	0	24.843	4.799201632	5.100581895	5.418843036	4.498366751	5.14041438	4.943688639
tr|D3ZZX1|D3ZZX1_RAT In	GPI transamidase compon	Pigs	7	18.2	47.622	412	0	12.949	25.51546961	21.25226602	23.75893849	17.68920657	25.03970638	20.63304916
sp|P63088|PP1G_RAT Se	SH3 and multiple ankyrin r	Shank3	2	7.4	36.983	323	0	20.546	15.3724365	14.91553974	13.89288537	14.42524305	16.11003786	15.49364964
tr|Q3MHS9|Q3MHS9_RAT	DnaJ (Hsp40) homolog, su	Dnajb4	5	14.3	58.017	531	0	40.178	7.147967672	6.250638499	6.380529313	5.654643191	6.840191501	6.020175467
tr|A0A0G2JU89|A0A0G2J	Acyl-coenzyme A thioester	Acot8	2	0.5	573.87	5193	0	2.3424	0.346027342	0.267029065	0.365938119	0.352369169	0.40512565	0.328119837
tr|D3ZCT5|D3ZCT5_RAT R	Tropomodulin-2 (Neuronal	Tmod2	4	5.6	96.821	857	0	4.0737	1.921833655	1.798140611	2.207323908	1.119296428	1.694478197	2.156207274
sp|Q9Z2L0|VDAC1_RAT V	Prenylcysteine oxidase (EC	Pcyox1	18	70	30.755	283	0	323.31	642.1528744	646.6571376	683.493524	677.2724313	620.9096174	593.6512018
tr|D4A617|D4A617_RAT E	Atlastin-1 (EC 3.6.5.-) (Spa	Atl1	8	23.2	52.016	465	0	21.775	24.82690757	17.21707982	25.11639767	19.77475774	32.18017025	27.73776984
sp|Q5I0G4|GARS_RAT GI	LYR motif-containing 4 (RO	Lyrm4	2	3.3	72.029	637	0	2.2995	0.199886329	0.176204082	0.250917927	0.193784813	0.25257551	1.408933554
tr|A0A0G2K1E2|A0A0G2K	Dipeptidyl peptidase 1 (EC	Ctsc	7	8.1	119.46	1099	0	64.185	13.20667566	11.63897236	13.73635614	10.80233035	12.55125422	12.7892158
tr|B2RYJ3|B2RYJ3_RAT C	Mitochondrial ribosomal pro	Mrps34	2	2.5	87.773	759	0	2.4576	5.706652236	4.819013032	4.942711479	10.31924638	4.844394233	5.587486583
tr|D4A7K0|D4A7K0_RAT R	Mitochondrial ribosomal pro	Mrps6	3	29.3	14.756	140	0	11.009	8.588086995	9.043497216	70.56379604	7.789674651	8.143334335	8.931458207
sp|P70584|ACDSB_RAT S	Cullin-5 (CUL-5) (Vasopres	Cul5	8	25.7	47.823	432	0	26.757	18.71517194	17.93527067	18.92694919	21.59511186	18.44614382	18.67491382
tr|M0RDY2|M0RDY2_RAT	Dedicator of cyto-kinesis 1	Dock1	2	15.4	18.35	175	0	5.0921	2.783194061	3.438326797	3.757800908	3.633459821	3.205914544	16.37083268
sp|Q9ET09|MRS2_RAT M	Succinate dehydrogenase	Sdhaf2	2	5.1	49.365	434	0	2.9467	1.985546575	1.990387459	1.930911086	1.846570003	2.053365827	1.652481449
tr|A0A0G2JSU3|A0A0G2J	Ig gamma-2A chain C regio	Igg-2a	3	12.8	33.381	289	0	19.273	2.517976073	3.139207443	2.568178754	2.414161106	2.449411344	2.729532877
sp|Q6AYU3|DNJB6_RAT D	Glutathione S-transferase	Gstm7	2	6.4	38.922	357	0.0055155	1.3309	2.058708784	2.210030197	3.603575117	1.605852693	2.565922257	2.115710058
tr|A0A096MJM1|A0A096M	Vesicle-associated membra	Vamp7	5	41.6	20.622	185	0	87.439	15.84888794	16.00328973	16.64759043	17.69311291	15.78359797	16.74911466
sp|P81155|VDAC2_RAT V	Myeloid-associated differen	Myadm	17	67.1	31.745	295	0	323.31	300.8661248	312.8410799	298.4892024	313.6182825	289.4651813	272.6709877
tr|A0A0G2JU01|A0A0G2J	Ras interacting protein 1 (R	Rasip1	2	3	96.684	862	0	2.1789	1.461330383	1.00365982	1.486975341	1.223725899	1.75237385	1.568920654
tr|G3V7D4|G3V7D4_RAT	Golgin subfamily A membe	Golga5	4	13.2	35.792	340	0	5.9846	21.08068355	13.24705398	18.42123937	14.21343265	15.78098121	15.80982562
tr|Q5M7T5|Q5M7T5_RAT	Dimethylaniline monooxyge	Fmo3	6	10.5	52.233	465	0	8.7846	15.57418079	14.17309212	13.29346741	11.29149084	14.12196002	14.37882134
tr|D3ZFR9|D3ZFR9_RAT R	Tripeptidyl-peptidase 2 (TP	Tpp2	7	28.4	36.407	334	0	70.303	15.01081185	17.59615142	17.40196709	18.2881786	18.18839368	16.34003632
sp|Q6AY55|DCAKD_RAT	Mammalian ependymin-rela	Epdr1	3	10.4	27.352	240	0	4.3428	9.463242564	9.810201619	10.1230961	7.717624392	7.774372207	10.83826628
tr|A0A0G2K350|A0A0G2K	Seryl-tRNA synthetase 2 (R	Sars2	3	13.5	34.659	312	0	10.643	3.014482328	3.202084814	3.00190607	2.633523761	3.20002685	2.889673959
sp|Q9Z270|VAPA_RAT Ve	Tetraspanin	Tspan8	6	37.3	27.841	249	0	22.062	25.38868169	15.18949864	22.8825842	17.22739046	18.33362345	18.79040018
tr|A9UMV9|A9UMV9_RAT	39S ribosomal protein L16	Mrpl16	9	61.6	12.5	112	0	50.173	136.0179145	158.6535903	148.0117372	126.8431782	138.6028475	151.5745603
tr|D3ZE15|D3ZE15_RAT N	Lysosome-associated mem	Lamp2	13	63.2	16.776	144	0	172.33	473.142193	548.1555981	446.2444201	471.7555791	457.5719152	448.1127284
sp|Q4V8B7|HSDL1_RAT I	LIM and senescent cell an	Lims1	5	17	36.907	330	0	15.596	6.002490702	6.631136962	6.598309112	6.651193757	6.800286023	6.413342353
tr|D4A6C5|D4A6C5_RAT R	Long-chain-fatty-acid--CoA	Acsl6	8	18.9	50.622	439	0	40.15	27.2422572	25.88293231	27.70567385	27.68075449	28.16476326	26.78770207
tr|Q6AY58|Q6AY58_RAT	Obg-like ATPase 1	Ola1	3	16.3	27.911	245	0	6.8664	4.652079808	4.574828144	6.305143998	3.936721964	4.851328628	4.985879655
tr|A0A0G2K4U8|A0A0G2K	Adenine phosphoribosyltra	Aprt	4	20.6	31.106	272	0	30.843	18.26025052	17.60756637	17.60456512	19.76911525	14.01990666	14.11294609
tr|A0JN30|A0JN30_RAT C	Ras-related protein Rap-1b	Rap1b	4	27.5	20.709	182	0	4.4505	17.46124744	12.70436805	17.33286388	11.94254739	15.92097748	14.5112457
tr|O89035|O89035_RAT M	Platelet-derived growth fac	Pdgfrb	5	22.4	31.454	286	0	7.2641	10.75783488	12.55026617	10.94709948	11.45468901	9.950202059	10.97479681
sp|P85968|6PGD_RAT 6-p	Metabolism of cobalamin-a	Mmaa	3	8.5	53.236	483	0	3.7936	3.451222834	3.220681677	10.33302327	3.181626572	3.621193194	3.501289705
tr|F1M7X5|F1M7X5_RAT D	Netrin-1	Ntn1	14	19.7	88.053	767	0	36.019	37.74492144	37.23367708	38.64387367	32.07799046	39.35268934	36.13388462
sp|O35244|PRDX6_RAT R	Huntingtin interacting prote	Hip1	3	18.8	24.818	224	0	39.391	14.75245157	13.35026419	12.4500942	13.77158227	14.31952484	14.39883898
tr|G3V715|G3V715_RAT N	Serine/threonine-protein p	Ppp2r2a	3	6.9	62.213	552	0	13.219	6.511237174	5.726977489	6.386287913	5.307847067	8.193052635	7.690364824
tr|D4A9P9|D4A9P9_RAT C	Biphenyl hydrolase-like (Bi	Bphl	14	28	56.377	503	0	93.846	77.39245899	91.71439824	90.72413275	72.31502165	89.07426117	81.2715989
sp|Q4V888|PP4P2_RAT T	Guanine nucleotide-bindin	Gng2	2	14.4	28.024	257	0	4.0202	2.642331892	2.028389743	2.346263232	1.748694501	2.101186982	2.467250529
tr|D4A131|D4A131_RAT M	Ubiquitin specific peptidase	Usp9x	3	10.2	33.082	294	0	4.5847	3.867828781	5.032567794	28.16845592	5.038309653	4.968624567	4.517056373
sp|P61023|CHP1_RAT Ca	Uncharacterized protein	Mia3	3	18.5	22.432	195	0	6.0125	3.5608266	2.780444955	3.705816451	3.230282199	3.116225346	2.870888178
tr|D3ZJJ6|D3ZJJ6_RAT 60	Transgelin (Smooth muscle	Tagln	2	18.1	12.227	105	0.00046447	1.9803	29.9542427	26.65724669	9.601157499	14.85450634	17.29084751	16.16860324
tr|D3ZXD2|D3ZXD2_RAT A	Mitochondrial carrier 1 (Mtc	Mtch1	24	20.1	183.96	1615	0	323.31	155.4387928	101.169785	124.6527597	103.6221612	121.0509789	111.7445989
sp|Q642G4|PEX14_RAT R	Laminin subunit alpha 5 (L	Lama5	2	7.2	40.936	376	0	4.9668	1.90879603	2.560279032	2.130577469	1.951216493	2.269444179	6.775712878
tr|D4ADG2|D4ADG2_RAT	Anoctamin	Ano10	5	26.5	38.673	358	0	63.161	17.1287282	10.34004573	8.493412174	15.99125108	13.06152099	14.38241425
tr|D3ZUV3|D3ZUV3_RAT E	Arfaptin-1 (ADP-ribosylatio	Arfip1	2	3.2	65.348	589	0.0004649	1.9833	0.486897486	0.311437992	0.33673678	0.34778139	0.400376244	0.764263046
tr|Q66H18|Q66H18_RAT S	5-demethoxyubiquinone h	Coq7	2	10.5	28.628	258	0	2.5973	4.339097064	3.617731823	4.810211318	3.953085185	3.969875488	3.874387629
sp|Q63798|PSME2_RAT R	Mitochondrial fission regula	Mtfr1l	3	21	26.857	238	0	8.3898	16.5019653	13.28462821	16.78684386	11.06969757	15.82154088	15.0132264
tr|B2RYJ7|B2RYJ7_RAT A	Mitochondrial ribosomal pro	Mrpl55	2	10.6	42.281	376	0	13.254	2.999849886	2.286367701	2.132723857	1.970314152	2.495989542	2.16005682
sp|Q68FS2|CSN4_RAT CO	Hypoxanthine-guanine pho	Hprt1	3	10.3	46.289	406	0	10.652	3.838563898	3.014927133	3.698120866	2.905361575	3.576773817	3.263593119
sp|Q62952|DPYL3_RAT D	Serine (Or cysteine) peptid	Serpinc1	9	25.3	61.967	570	0	60.11	14.69344336	17.11672335	31.74507032	11.27326125	15.97331254	20.78959733
tr|Q6GMM8|Q6GMM8_RAT	Eukaryotic peptide chain re	Etf1	11	21.2	71.346	646	0	60.928	31.5945115	28.29243883	31.31526932	28.84223938	27.1285292	31.25317454
tr|G3V9T7|G3V9T7_RAT A	Palmitoyl-protein thioestera	Ppt1	2	7.2	38.822	348	0	3.9601	1.504669522	1.25198263	1.692400325	1.390092551	0.959694045	1.553419819
sp|P62250|RS16_RAT 40	Transmembrane emp24 do	Tmed1	3	20.5	16.445	146	0	44.548	8.11761612	6.507474961	9.410600175	4.679794511	9.541988641	5.399115503
tr|F1LS29|F1LS29_RAT C	Thioredoxin-related transm	Tmx3	2	4.2	82.099	713	0	5.808	1.041175969	1.218736577	0.86609351	0.97875502	0.984487776	0.878671531
sp|Q4KM74|SC22B_RAT V	Membrane palmitoylated p	Mpp6	3	16.7	24.74	215	0	7.8457	8.414650698	6.176916922	7.499791839	7.479771731	6.542535884	6.147467096

tr|Q9JKC1|Q9JKC1_RAT (	von Willebrand factor (vWF	Vwf	3	7.5	67.776	597	0	22.898	12.75215295	14.61494596	11.22037125	12.70341548	11.86435664	11.24375171
sp|Q5I0L3|SYYM_RAT Ty	Collectin-12 (Collectin plac	Colec12	5	14	52.627	471	0	28.599	9.078891781	9.812104111	9.633615065	10.50154222	9.08144017	10.17665777
tr|D3ZJY1|D3ZJY1_RAT M	Tubulin polymerization-pro	Tppp3	5	17.5	30.236	257	0	7.2678	10.5955623	11.37214781	11.65750137	13.28372389	11.0426963	11.30277807
tr|D3ZF13|D3ZF13_RAT A	39S ribosomal protein L14	Mrpl14	6	23.7	17.514	156	0	111.69	182.2556326	197.8877381	180.5425947	190.3342134	179.6662389	171.5921955
tr|A0A0G2K845|A0A0G2K	Basic leucine zipper and W	Bzw2	3	16.5	23.466	218	0	15.907	8.320955227	7.762644266	6.817659439	7.596961911	8.567902457	8.041956622
tr|A0A0G2K3C1|A0A0G2K	Nicotinamide phosphoribos	Nampt	5	14	42.996	364	0	25.418	10.77099212	9.465374887	10.03567008	9.91342113	9.691143544	9.629509075
tr|D4A4Z9|D4A4Z9_RAT K	Neuropilin-1 (Vascular end	Nrp1	9	8.4	151.79	1325	0	21.163	13.09663332	12.00187276	11.41145208	12.59924644	15.13595168	11.69645823
sp|P62243|RS8_RAT 40S	Eukaryotic translation initia	Eif4e	6	38	24.205	208	0	47.629	38.47973315	27.95569769	27.61510677	24.76792747	31.4141159	27.61869057
sp|Q9EPT8|CLIC5_RAT C	Mitochondrial ribosomal pro	Mrpl32	6	43	28.299	251	0	68.625	30.99406308	24.30576618	31.76234612	29.41907549	33.3164951	31.02630801
tr|A9UMV2|A9UMV2_RAT	A-kinase anchor protein 2	Akap2	3	17.4	18.742	167	0.0017881	1.6955	3.270131417	3.775828934	3.481283384	4.608612328	3.158485901	3.914320245
sp|Q6JE36|NDRG1_RAT F	Ribosome-recycling factor,	Mrrf	2	7.9	42.954	394	0	3.3654	3.124604409	2.491551497	3.183459038	2.652751632	2.912184056	7.561533377
tr|M9MMM9|M9MMM9_RAT	Eukaryotic translation initia	Eif1b	2	0.7	611.47	5358	0.00091033	1.8377	0.486538652	0.404840852	0.595596341	0.417118913	0.707439087	0.615054673
sp|Q07936|ANXA2_RAT A	Mitochondrial ribosomal pro	Mrpl27	19	51	38.678	339	0	303.29	114.5158007	109.1222029	115.847336	88.76505059	119.7491444	106.6221374
tr|Q9ET45|Q9ET45_RAT E	Coatomer subunit beta' (B	Copb2	4	22.5	20.978	187	0	5.0738	5.010116118	6.224003606	5.263884332	5.801781972	6.403913411	5.429398593
tr|Q5XIH3|Q5XIH3_RAT N	Proteasome activator comp	Psme2	23	63.6	50.73	464	0	323.31	521.584745	605.0876806	479.1155585	519.3695151	481.5740791	504.3212215
sp|Q5XIM7|SYK_RAT Lys	Adipocyte plasma membra	Apmap	4	8.5	71.622	626	0	44.102	5.582655251	4.600844726	4.225032809	5.611673459	4.889467798	5.049474142
tr|A0A0G2KAL9|A0A0G2K	[Protein ADP-ribosylarginin	Adprhl1	3	14.1	32.801	291	0	4.4701	5.080686749	5.189047787	4.806127946	5.117738553	4.747770641	4.824968664
tr|Q5BJZ3|Q5BJZ3_RAT N	CD34 antigen (Predicted) (	Cd34	45	42.1	113.87	1086	0	323.31	1153.371312	1409.604118	925.7211999	1334.405513	1269.844656	1257.774745
tr|B1H271|B1H271_RAT M	Iron-sulfur cluster assembly	Isca1	7	30.2	35.185	318	0	22.66	20.55798243	24.44274563	23.28620974	20.70924091	18.67772643	100.5911832
sp|Q9JHW5|VAMP7_RAT	Glutathione S-transferase	Lancl1	3	12.7	24.775	220	0	9.5378	6.660751223	5.355040242	6.041295395	4.961919018	5.416808882	6.347130175
tr|Q5XID6|Q5XID6_RAT S	Lymphocyte cytosolic prote	Lcp1	4	21.9	32.18	292	0	131.97	11.63019952	10.68011623	12.7081842	10.62046855	13.38861508	13.87530083
tr|G3V9G1|G3V9G1_RAT	Glutamyl-tRNA(Gln) amidot	Qrsl1	4	4	139.1	1262	0	30.998	10.52060592	10.53267308	13.16625469	9.781907707	18.74249106	12.75636635
tr|D4A5I9|D4A5I9_RAT My	Eukaryotic translation initia	Eif2s1	7	6.5	148.13	1285	0	7.7603	3.972687968	4.180060988	4.120016877	3.355762499	3.230184925	4.564790734
sp|Q920Q0|PALM_RAT P	Cerebral dopamine neurot	Cdnf	8	28.2	41.926	383	0	72.401	17.23119516	11.00211305	17.05016895	11.93256452	14.15859455	13.72337211
tr|D3ZVQ0|D3ZVQ0_RAT	60S ribosomal protein L13	Rpl13a	2	3.1	95.778	858	0	23.768	2.056715263	2.150244376	1.460066972	2.127652819	1.780242266	2.445333785
tr|D3ZUF9|D3ZUF9_RAT F	Lysosomal alpha-glucosida	Gaa	17	23.6	108.54	954	0	73.859	40.54821019	40.13545349	40.66671296	46.5158206	38.12543233	43.10772076
tr|Q6PDV6|Q6PDV6_RAT	Tyrosine-protein kinase (EC	Jak1	4	30.5	16.273	151	0	20.925	9.328879272	7.619481719	8.674546334	7.096950477	8.28071385	8.633760042
tr|G3V8U8|G3V8U8_RAT	Atlastin-3 (EC 3.6.5.-)	Atl3	8	26.2	44.229	393	0	76.803	33.22401529	35.51953151	34.0998144	39.16843037	31.85961805	37.74145471
tr|A0A0G2JVL6|A0A0G2J	Alpha-parvin (Actopaxin)	Parva	10	47.7	19.965	172	0	218.92	228.5292342	334.914747	231.6213808	268.7474648	215.5615437	214.7738275
tr|A0A0G2K0B4|A0A0G2K	DnaJ (Hsp40) homolog, su	Dnajc30	6	8.7	102.43	886	0	56.536	6.852129206	7.01401354	7.822273465	6.254917334	6.775426872	7.531250288
tr|M0R785|M0R785_RAT (	ATPase H+-transporting V	Atp6v1a	2	24.7	15.849	154	0	19.94	5.926338212	5.917226721	6.968953578	5.990588373	7.164668833	6.816774694
sp|P70490|MFGM_RAT La	SPRY domain-containing p	Spryd4	3	7.5	47.412	427	0	6.1146	11.25143059	12.43897037	11.44338614	10.52888659	15.74696343	12.81539271
tr|Q5U1Z9|Q5U1Z9_RAT M	Transmembrane anterior p	Tapt1	9	25.5	29.718	263	0	76.449	65.04459203	71.21504356	64.98318868	69.30714037	62.61234971	61.97767821
sp|P19139|CSK21_RAT C	Vesicle-trafficking protein S	Sec22b	2	6.9	45.073	391	0	2.6964	3.486149316	3.232619816	3.646502866	3.349121722	3.572259919	2.862316524
tr|A0A0G2K1F2|A0A0G2K	Endoplasmic reticulum-golg	Ergic1	13	6.9	276.39	2455	0	36.675	34.5182089	23.82158189	26.77225706	22.89809305	26.54957267	27.14801951
sp|P36972|APT_RAT Ade	Glutamine--tRNA ligase (EC	Qars1	5	32.8	19.546	180	0	9.2613	8.731221778	9.414007594	7.524396769	8.501062446	8.586219725	10.70070919
tr|D3ZG43|D3ZG43_RAT N	Thioredoxin domain-contai	Txndc12	14	38.3	30.226	264	0	258.76	252.152454	273.540345	239.201793	256.2992635	235.9787565	245.087713
sp|Q9Z269|VAPB_RAT Ve	DnaJ (Hsp40) homolog, su	Dnaja4	4	22.2	26.916	243	0	71.162	21.00253754	17.06012421	18.3542302	15.20000035	19.94488891	17.08633482
tr|Q4FZU0|Q4FZU0_RAT A	G-protein-signaling modula	Gpsm1	2	7.5	47.28	413	0	5.0311	3.090993651	2.931550407	2.946204699	3.050633994	2.901847883	3.402330729
tr|D3ZFF4|D3ZFF4_RAT L	Myosin VI	Myo6	3	10.1	44.566	376	0	4.4631	2.631925714	2.62886388	2.815536819	2.539163965	2.518035683	2.777780511
sp|Q99068|AMRP_RAT Al	CD166 antigen (Activated	Alcam	5	17.2	42.031	360	0	14.143	5.500920904	5.788332866	5.937640586	5.438058378	5.980522827	6.945606141
sp|Q6QBQ4|PLS3_RAT P	Mitochondrial import recep	Tomm40l	2	8.4	31.646	296	0	2.7977	1.289767995	1.289937351	1.649734331	1.772002325	1.474736391	1.372645175
tr|Q5XIM0|Q5XIM0_RAT E	Reticulocalbin-3	Rcn3	16	44.7	47.393	418	0	154.29	53.55393896	60.1758319	52.63884332	56.3033708	50.15006508	53.05699873
sp|P97878|EXOC5_RAT E	Oxidative stress-responsive	Oxsr1	2	4	81.736	708	0.00047259	2.1235	2.022067874	1.766464114	2.565718261	1.837194788	2.031188848	1.885301945
sp|P15999|ATPA_RAT AT	Prostaglandin E synthase	Ptges3	35	61.7	59.753	553	0	323.31	2411.681551	2767.936068	2576.764304	2608.827016	2458.766235	2410.482569
sp|Q6AXV4|SAM50_RAT	Mitochondrial ribosomal pro	Mrpl33	20	53.9	51.96	469	0	323.31	157.001713	170.3634305	156.0318971	181.6664805	161.0284179	160.1667452
tr|D4A2Z6|D4A2Z6_RAT S	Tyrosine-protein phosphat	Ptpn11	5	8	83.591	727	0	12.679	8.501169494	8.129825282	8.326936269	7.692450206	7.946423696	8.612715862
tr|D3ZF12|D3ZF12_RAT S	Normal mucosa of esopha	Nmes1	2	11.7	20.327	180	0	3.6442	3.970853928	3.869241307	4.417841222	3.353462099	4.605877227	3.126857272
sp|Q63362|NDUA5_RAT N	Carbonic anhydrase 4 (EC	Ca4	10	68.1	13.412	116	0	265.23	136.7036856	132.556152	120.0668197	139.7601403	137.0916728	126.8912762
tr|Q6AY18|Q6AY18_RAT	Mitochondrial import inner	Timm22	2	10.1	22.399	198	0	12.455	0.912235053	0.791817301	0.887662086	0.876105103	0.684215407	0.864864495
sp|Q5U300|UBA1_RAT Ub	RAB5B, member RAS onc	Rab5b	16	20.8	117.79	1058	0	84.25	21.33346197	18.6449003	21.00632747	17.3936269	19.19715184	53.96035867
tr|D4ACN7|D4ACN7_RAT	Solute carrier family 43 me	Slc43a1	10	7.3	233.38	2048	0	17.217	7.712532722	5.959081552	8.83369311	7.176813415	9.120037272	8.517760413
tr|D4AE68|D4AE68_RAT (	40S ribosomal protein S20	Rps20	3	11.7	42.158	359	0	27.836	17.19770401	14.96880953	16.09528829	14.22558571	16.33704116	15.68664018
tr|F1LZX9|F1LZX9_RAT In	ATP-binding cassette subf	Abce1	12	14	115.58	1047	0	39.941	22.39042662	19.87153222	22.13972474	19.7144265	24.98213782	23.86872025
sp|P15651|ACADS_RAT S	Lumican (Keratan sulfate p	Lum	19	59	44.765	412	0	323.31	172.1405088	155.8807078	142.7609406	181.8314148	148.4810888	169.4723792
sp|P10760|SAHH_RAT Ad	Sorting nexin 2	Snx2	2	6.2	47.538	432	0	3.687	0.859247274	0.603660811	0.824579235	1.461665368	0.703317701	0.74162772
tr|B2RYS2|B2RYS2_RAT	Tripartite motif-containing 2	Trim25	12	72.1	13.558	111	0	174.8	355.0101458	355.2619023	324.7641257	371.0284494	334.5322043	363.1199025
tr|Q2I6B2|Q2I6B2_RAT V-	Ectonucleoside triphospha	Entpd5	8	13.7	95.584	832	0	81.443	29.41041027	27.21943316	29.10920183	24.57868703	29.10025235	28.42247562
tr|D4A0U0|D4A0U0_RAT F	Mitochondrial intermediate	Mipep	3	4.6	77.271	672	0.0022114	1.6253	1.025706249	1.339735088	1.100102092	1.228630525	1.093606363	1.467703278
tr|A0A0G2JZF0|A0A0G2J	Mitochondrial ornithine tran	Slc25a15	2	2.2	114.7	1056	0	3.7988	0.916700539	1.013219844	1.303694794	0.797500875	1.066457554	1.044150648
tr|Q80ZA3|Q80ZA3_RAT A	Vacuolar protein-sorting-as	Vps36	2	5.7	46.465	418	0	3.8564	3.917387704	3.941678701	3.519342497	3.560411276	4.310053336	4.103769197
tr|F1LQQ7|F1LQQ7_RAT	Elongin-B (EloB) (Elongin	Elob	7	5.5	195.07	1704	0	26.242	13.06553439	8.682974923	12.12499503	10.33183348	13.04778304	14.53023679
tr|A0A0G2JU49|A0A0G2J	Proteasome subunit alpha	Psma6	8	22.2	51.925	463	0	102.61	33.73116694	31.31502349	27.70933841	34.57283917	32.09185485	30.77634421
tr|G3V7Q6|G3V7Q6_RAT	Glucosylceramidase (EC 3	Gba	6	29.7	28.559	263	0	13.203	7.510389727	6.535536723	8.619577875	5.333889329	6.736175582	8.268823154
sp|P11232|THIO_RAT Thi	Mitochondrial fission 1 prot	Fis1	4	31.4	11.673	105	0	23.121	28.68157912	26.86889896	22.80981643	27.86174821	26.46583659	28.30288308
tr|Q6IN10|Q6IN10_RAT Er	RCG56113, isoform CRA_	Smyd1	2	2.9	85.592	754	0.00047461	2.1536	2.644524764	1.995904687	1.840658112	2.134814441	2.173867287	2.988684262
tr|D3ZXL1|D3ZXL1_RAT R	Dynactin subunit 2	Dctn2	4	9.2	64.016	555	0	3.7552	1.921036247	2.146772327	2.113615774	2.062286741	2.098897323	1.672499084

tr|F1LSK3|F1LSK3_RAT V	Plasma membrane calcium	Atp2b4	2	6.2	24.744	211	0	3.2416	2.22046305	2.888078458	3.173093557	3.49335245	2.129317073	4.169981375
tr|Q68FT7|Q68FT7_RAT F	Cytochrome C oxidase ass	Coa3	4	8.3	65.651	589	0	7.2023	6.431496347	6.437558369	6.577892256	6.371239438	7.242517225	6.492899622
tr|D3ZN51|D3ZN51_RAT Ir	Growth factor receptor-bou	Grb10	2	1.9	114.6	1036	0.0058923	1.2905	3.197208431	3.083321732	9.688583524	3.613320472	4.105619533	2.99191788
tr|D3ZJ77|D3ZJ77_RAT Ca	RCG20695, isoform CRA_	Slirp	4	22.4	23.671	196	0	3.9002	2.822267066	2.770266621	2.402278709	2.60092753	2.397468804	2.654184445
sp|P62944|AP2B1_RAT A	Peptidyl-tRNA hydrolase 2	Ptrh2	12	15.7	104.55	937	0	69.455	56.83128692	50.8441071	53.87432488	45.97327347	52.53065583	55.64902585
tr|F1LM33|F1LM33_RAT L	60S ribosomal protein L3 (	Rpl3	48	39.4	156.68	1392	0	323.31	179.7916411	194.1112908	205.6553278	214.5535172	186.4567121	202.5836009
tr|B5DEY8|B5DEY8_RAT	Phosphatidylinositol 4-kina	Pi4ka	2	4.7	46.636	406	0	2.8746	2.932030314	2.726461736	2.947775226	2.296884119	2.859914421	2.951010046
tr|F1LRI7|F1LRI7_RAT AP	Complement C1q subcomp	C1qb	3	4	103.86	963	0	2.3886	2.089807706	1.762040819	2.140681196	1.680507177	1.814914239	2.118276422
sp|Q9WVK3|PECR_RAT P	Mitochondrial ribosomal pr	Mrpl28	5	27.4	32.433	303	0	168.3	18.02581249	20.30672734	20.06296401	20.37807014	17.528972	18.02767695
tr|A2IBE0|A2IBE0_RAT Ca	Mitochondrial dicarboxylate	Slc25a10	3	12.8	37.525	337	0	86.347	6.433091164	4.881795279	5.479046586	4.865562648	5.398229938	5.431964956
tr|F1LNC4|F1LNC4_RAT V	Annexin	Anxa7	3	19.9	23.096	201	0	5.3651	1.988257764	2.035999712	4.015524454	3.901868736	4.075723134	2.051858935
tr|F1M779|F1M779_RAT C	60S ribosomal protein L21	Rpl21	38	29.6	191.56	1675	0	323.31	99.25340653	99.8190155	95.39383421	93.49606155	109.6746466	102.4286995
sp|D4A6D7|TTC19_RAT T	Phenylalanyl-tRNA synthet	Farsb	2	5.5	41.333	365	0	2.2356	0.426334328	0.442172507	0.343594749	0.343280419	0.602925985	0.447558394
tr|B2GUX5|B2GUX5_RAT	Nebulette	Nebl	7	28.6	33.81	297	0	21.371	11.01858738	10.6011628	11.44443316	10.69295284	11.57978479	11.61536114
sp|Q52KK3|S2551_RAT S	Transmembrane protein 11	Tmem11	3	12.1	33.774	298	0	20.725	7.661897297	8.122690936	8.353635235	7.829172082	7.456436757	13.06330342
sp|Q5XIM9|TCPB_RAT T-c	Adenylate kinase isoenzym	Ak1	9	25.8	57.458	535	0	75.18	17.26787594	17.53241792	15.40739729	17.93791018	16.60525831	17.33373226
sp|Q5M7U6|ARP2_RAT A	39S ribosomal protein L40	Mrpl40	6	20.3	44.733	394	0	16.936	15.7392443	13.34075173	12.57783043	12.40175928	13.83280885	13.13772796
tr|D4ADS4|D4ADS4_RAT	Vacuolar protein sorting-as	Vps4a	5	34.2	16.732	152	0	78.495	22.39321755	26.6039769	23.56314608	29.98115432	23.15695283	24.63247002
sp|Q4AEF8|COPG1_RAT	Mitochondrial ribosomal pr	Mrps5	2	3.5	97.613	874	0	4.3019	4.957487173	4.94077254	7.154275812	4.612518667	5.157750366	5.541805313
tr|Q5RK10|Q5RK10_RAT	Methylenetetrahydrofolate	Mthfd1l	6	26.6	23.446	203	0	8.0516	18.29134945	18.35144086	15.6544936	12.98076558	18.69211857	16.54072594
tr|D3ZVB7|D3ZVB7_RAT C	Myocardial zonula adheren	Myzap	2	5.7	34.069	298	0	2.6122	4.345875035	5.658963389	5.562284538	4.87641359	6.315598008	5.620336036
sp|P62994|GRB2_RAT Gr	Serine/threonine-protein ki	Mtor	3	16.6	25.206	217	0	3.6222	4.176027075	4.29839601	4.605833352	4.63074825	3.271725874	9.625916166
sp|P17046|LAMP2_RAT L	Pcyox1l protein (Prenylcys	Pcyox1l	3	6.6	45.155	411	0	9.376	15.22411856	12.57499857	15.18909398	12.93519162	12.71938058	14.96754513
sp|P10960|SAP_RAT Pros	Integrin beta	Itgb3	6	12.8	61.123	554	0	16.126	15.39436523	15.16333937	19.78969225	15.36363256	19.40780043	19.90574175
tr|Q6P6T6|Q6P6T6_RAT C	Ragulator complex protein	Lamtor3	10	31	44.622	407	0	77.405	45.55194704	48.82270902	51.86300279	49.81884752	46.81566798	57.20937485
tr|D4A1H7|D4A1H7_RAT M	Protein transport protein S	Sec23a	10	40.1	39.78	352	0	72.18	18.21200732	23.78210518	18.84580528	19.34115407	18.17138479	19.29546051
tr|A0A0G2K6R8|A0A0G2K	Leucine-rich repeat-contai	Lrrc4b	7	7.1	182.51	1699	0	20.819	3.406567971	3.844604031	5.073588762	3.336447821	3.97255766	3.850058503
tr|A0A0G2JX56|A0A0G2J	Prolyl 4-hydroxylase subun	P4ha2	3	18	18.753	167	0	6.7007	2.986413557	2.355237923	3.296746414	2.132817867	6.028343982	3.149441271
sp|F1LMZ8|PSD11_RAT 2	B-cell receptor-associated	Bcap31	8	23.2	47.463	422	0	21.118	9.661797222	8.180241329	8.947818101	8.475454221	9.44582298	10.2531354
sp|Q07969|CD36_RAT Pla	40S ribosomal protein S25	Rps25	10	29.4	52.73	472	0	323.31	216.7834104	187.9852656	266.1520432	183.8930939	223.6080582	208.9481824
sp|Q63083|NUCB1_RAT N	COP9 (Constitutive photon	Cops7a	13	32.9	53.506	459	0	27.406	28.88611434	29.28886918	31.06450844	25.1251405	31.13739431	28.91008468
sp|P54319|PLAP_RAT Ph	Vacuolar protein sorting-as	Vps29	3	5	87.083	795	0	2.9496	3.163797025	2.067771334	2.032053051	2.181516898	2.670526946	3.522744504
tr|D3ZYX8|D3ZYX8_RAT C	Mycophenolic acid acyl-glu	Abhd10	6	43.3	13.273	120	0	53.729	75.39096425	87.34817838	82.85055539	80.36642104	80.45860297	76.86258643
tr|D4A040|D4A040_RAT M	tRNA methyltransferase 10	Trmt10c	2	15.7	20.168	191	0	19.548	0.937353413	1.280615139	1.170199965	1.206885236	1.022103596	1.521904875
tr|A0A0G2K0Q7|A0A0G2K	Armadillo repeat-containing	Armc1	2	1.1	214	1952	0	2.9409	1.910390847	1.933931	2.318203143	1.830597415	2.380459911	2.426548004
tr|D3ZKG9|D3ZKG9_RAT	Acid sphingomyelinase-like	Smpdl3b	2	1.9	145.35	1298	0	3.6353	1.088223057	0.904825345	1.004509324	0.829619665	1.164978292	1.343799249
tr|D4A0T0|D4A0T0_RAT N	HBS1-like protein	Hbs1l	16	73.3	20.858	176	0	323.31	263.5713403	282.753164	249.0437647	275.9264484	235.6254949	253.1871562
sp|D3ZTX0|TMED7_RAT T	Ubiquitin domain-containin	Ubtd1	5	28.8	25.479	226	0	11.569	15.96570825	16.57689116	14.98283139	13.99207342	13.63851496	36.59788314
tr|Q642B0|Q642B0_RAT C	DnaJ homolog subfamily C	Dnajc5	12	32.5	62.561	557	0	152.99	25.56969337	22.35571156	25.18445386	24.34430667	24.32794965	21.77097473
tr|D3ZFQ8|D3ZFQ8_RAT C	Muscarinic acetylcholine re	Chrm2	11	36.5	35.434	326	0	323.31	546.6632349	574.4575543	526.2837314	541.7658606	535.9174904	539.1416413
tr|D3ZPN5|D3ZPN5_RAT M	Lysosome membrane prote	Scarb2	2	7.7	37.284	336	0	4.4247	3.796101908	4.126458267	4.199747318	4.878583779	3.927026163	4.004194294
tr|B2RYU0|B2RYU0_RAT	Calpain small subunit 1 (C	Capns1	2	31.4	11.842	105	0	29.62	31.00203716	36.35567688	33.49934942	43.02962977	30.25881959	35.88186773
sp|Q8K1Q0|NMT1_RAT G	AP-1 complex subunit beta	Ap1b1	2	5.2	56.86	496	0	6.1303	3.463901625	3.252881359	2.775121914	3.329112584	3.472365385	3.151956307
sp|Q6AXN3|TMED5_RAT	Casein kinase I isoform ga	Csnk1g2	3	14.4	26.136	229	0	4.8996	5.961025472	6.058962398	6.01407292	6.016196598	5.778117008	7.170419582
tr|Q6AYJ9|Q6AYJ9_RAT M	Cytochrome c oxidase sub	Cox7c	7	25.1	41.712	374	0	146.5	75.9850334	46.09453504	64.1874548	60.03175467	53.62249588	53.80124414
sp|P10980|ACM2_RAT Mu	Proteasome 26S subunit,	Psmc6	3	8.2	51.538	466	0	6.6972	2.855518991	2.454880958	3.440502022	2.561169676	2.679620161	2.88495185
sp|Q5HZE0|MCATL_RAT	Protein tyrosine phosphata	Ptprm	2	7.2	32.634	306	0.00091617	1.8679	2.482730628	2.086796257	2.665813207	2.178608845	2.436981769	2.237971615
tr|G3V8T4|G3V8T4_RAT D	Embigin	Emb	2	1.7	126.94	1140	0.0017817	1.6813	2.754886068	2.588388356	2.429501184	2.566898974	2.317854104	2.627494265
tr|G3V803|G3V803_RAT C	Antigen-presenting glycop	Cd1d	11	15.5	99.667	906	0	156.85	38.98010684	28.38518533	35.98706482	32.15524917	33.42509034	31.01398946
tr|C0KUC5|C0KUC5_RAT	ADP-ribosylation factor 6	Arf6	2	6.9	41.609	362	0	9.3661	10.33799943	10.35479004	12.69457297	8.937704375	11.79697526	10.44817903
tr|A0A140TAA4|A0A140TA	Aa2-277 (Mitochondrial rib	Mrpl49	18	26.7	95.881	868	0	94.073	46.2137959	50.29238433	46.47714083	46.29446137	47.08977282	46.79969127
tr|A0A0G2JVV5|A0A0G2J	Eukaryotic translation initia	Eif2s3	2	5	52.293	458	0.00090827	1.8081	1.227849244	1.276810154	1.629736282	1.052020584	1.244592993	1.492186386
tr|A0A0G2JW28|A0A0G2J	Similar to potassium chann	LOC681355	8	16.7	74.963	670	0	76.347	11.80124359	9.870130127	13.60443184	11.69818416	13.29506617	12.63164108
tr|Q5RK08|Q5RK08_RAT	DnaJ (Hsp40) homolog, su	Dnajc28	10	36.7	32.941	281	0	323.31	161.2439249	175.9757828	177.8465227	187.2568861	167.0011559	175.9088188
sp|Q5FWT5|GATA_RAT G	Activator of Hsp90 ATPase	Ahsa1	3	7.6	56.837	525	0	8.1626	3.138718535	2.731931401	3.081322406	3.434236515	2.81516795	3.125676745
tr|D4A5E5|D4A5E5_RAT S	Copine 3 (Copine 3 protein	Cpne3	7	31.3	32.846	297	0	260.78	23.4402146	22.62206049	26.11787065	22.16109703	27.74608284	26.08349193
sp|Q5U367|PLOD3_RAT M	Metalloreductase STEAP3	Steap3	2	4.6	85.059	741	0	2.3591	3.492568452	3.222346357	3.055984564	3.491052051	3.485645405	3.126600636
sp|Q6IMY8|HNRPU_RAT H	Cytochrome c oxidase ass	Coa7	5	8.3	87.731	798	0	35.842	9.957236983	7.035892201	8.627954021	6.913786567	8.870137391	7.903886267
tr|F7FF30|F7FF30_RAT Lo	Plexin B1	Plxnb1	2	3.1	89.774	806	0.0021988	1.603	1.321704196	1.659306234	1.169781158	1.415483756	1.468325347	1.568458709
sp|Q7TT47|SPG7_RAT Pa	RCG41110 (Required for m	Rmnd1	17	28.3	86.102	781	0	193.5	46.72413719	60.72755467	48.14242338	49.51936151	50.50332669	46.34031221
sp|P52296|IMB1_RAT Imp	IlvB (Bacterial acetolactate	Ilvbl	6	9.4	97.123	875	0	20.163	11.04250963	11.48772422	10.30004219	12.0232784	10.43364712	11.4675386
tr|A0A0G2K6G2|A0A0G2K	Dishevelled associated act	Daam1	4	12.3	46.446	415	0	8.355	7.516370289	8.505091891	9.427352467	7.178549566	9.13835454	7.618506647
tr|D3ZEV8|D3ZEV8_RAT S	Synapsin-2 (Synapsin II)	Syn2	10	15	89.67	820	0	109.46	32.2790865	26.25439394	29.46099997	26.1946094	31.15374901	26.1686952
tr|D4A9Z6|D4A9Z6_RAT M	39S ribosomal protein L22	Mrpl22	5	26.9	36.206	320	0	33.133	12.29284579	13.79211803	13.66987048	13.63095405	12.31770904	12.64755254
sp|P28494|MA2A1_RAT A	Prostaglandin F2 receptor	Ptgfrn	2	2	131.24	1148	0.0022075	1.621	2.18860659	2.691455877	1.950019169	2.475013192	2.212464389	2.61517572
tr|A0A0G2K8R3|A0A0G2K	Serine/threonine-protein p	Ppp5c	11	9.8	195.68	1729	0	53.235	17.00233899	13.57238017	18.73691538	17.40187362	17.39159249	17.58472261
sp|P47942|DPYL2_RAT D	V-type proton ATPase sub	Atp6v0d1	15	42.3	62.277	572	0	266.58	52.14651338	57.5646612	54.99986951	53.4126797	57.02034923	58.23078751

tr|G3V8V3|G3V8V3_RAT	Serum paraoxonase/aryles	Pon2	34	42.5	97.287	842	0	323.31	287.0789359	246.9577711	299.557161	238.2563162	307.174055	365.3013114
sp|Q498T4|RM02_RAT 39	ETHE1, persulfide dioxyge	Ethe1	2	6.2	32.999	304	0.0017849	1.6886	3.758583849	3.681465315	4.484012777	4.590382744	3.906222979	4.039764092
tr|A0A0G2JWD2|A0A0G2J	ER membrane protein com	Emc7	2	5.5	63.389	563	0	4.1041	1.547251124	1.120235037	2.026242099	1.683588845	1.989844155	1.573540108
tr|Q6PCT9|Q6PCT9_RAT	Steroid 5 alpha-reductase	Tecrl	7	20.1	45.598	389	0	18.219	6.066682067	5.465384796	6.672647409	4.551753389	6.339541295	6.083308009
tr|Q4KLG6|Q4KLG6_RAT	1-acylglycerol-3-phosphate	Agpat5	4	16.4	30.359	262	0	6.0291	10.47236272	10.60306529	11.92239699	10.69208477	10.45392695	14.60466133
sp|D3Z8L7|RRAS_RAT Ra	Tropomyosin alpha-3 chain	Tpm3	5	23.4	23.909	218	0	16.129	18.34796543	18.06559139	21.29582802	17.65665375	20.81234243	16.01410816
tr|Q8R3Z7|Q8R3Z7_RAT	Ufm1-specific protease 2 (	Ufsp2	16	35.3	61.467	541	0	321.89	158.3373718	169.8545138	146.9856593	157.1346695	157.1229146	146.9910351
tr|G3V6H9|G3V6H9_RAT	Transthyretin (Prealbumin)	Ttr	2	9	45.373	391	0	13.621	5.135707919	4.964553694	5.166563984	4.810873897	5.384884499	5.499716952
tr|D3ZZ21|D3ZZ21_RAT N	Glycylpeptide N-tetradecar	Nmt1	8	55.5	15.638	128	0	171.6	87.15273613	98.23519065	84.81894973	93.74346304	77.4100861	87.5694549
tr|Q6TXF3|Q6TXF3_RAT	Complement component C	C9	3	7	66.449	600	0	29.032	21.94228318	11.57095826	19.95093306	11.42821271	19.72900682	15.03170421
sp|P62890|RL30_RAT 60S	Lactadherin (MFGM) (Milk	Mfge8	3	34.8	12.784	115	0	38.194	20.79959714	19.05155804	10.70523826	14.97039441	15.26482675	14.2048219
tr|D3ZDQ9|D3ZDQ9_RAT	Calcium uptake protein 1,	Micu1	13	44.4	43.156	387	0	105.29	29.91158136	27.96235641	35.21331832	27.99456375	35.85801614	35.22744504
sp|P62268|RS23_RAT 40	Metalloreductase STEAP4	Steap4	2	15.4	15.807	143	0	2.2508	2.338957918	0.941923945	2.05608212	1.081752167	1.50463279	1.860716182
tr|D3ZC84|D3ZC84_RAT	Peroxiredoxin-like 2A (Pero	Prxl2a	4	2.3	289.44	2547	0	9.0791	3.203188993	2.916901216	3.3016674	2.502748202	3.207550014	3.22201803
tr|G3V9X2|G3V9X2_RAT	C-type mannose receptor	Mrc2	2	5.5	74.439	673	0	7.7696	1.50347341	1.345252315	1.102929041	1.850346131	1.121540198	1.423561826
tr|A0A140UHX9|A0A140U	Mitochondrial ribosomal pr	Mrps17	2	5.4	55.596	480	0.00047348	2.1328	2.807355532	3.070194535	3.935898715	2.874588301	3.790431673	4.027240238
tr|A0A0G2K5A4|A0A0G2K	Collagen type VI alpha 6 c	Col6a6	2	2.1	162.36	1446	0	2.717	0.850515654	0.678476322	0.608788771	0.690293562	0.789474283	0.806659371
sp|P18614|ITA1_RAT Inte	4F2 cell-surface antigen he	Slc3a2	7	8.1	130.81	1180	0	12.652	9.209268032	7.16240794	8.225375498	7.26839537	8.52014672	7.533303379
tr|A0A0G2JW85|A0A0G2J	Tafazzin family protein	Taz	3	5.5	95.991	834	0	20.379	4.48821241	4.065863887	4.980351786	4.138766514	4.68863203	4.124248778
sp|P62815|VATB2_RAT V	Dynactin subunit 4 (Dynac	Dctn4	2	5.1	56.55	511	0	3.6972	2.593091931	2.082848586	2.46996844	1.801950927	2.232940479	5.176355153
tr|Q3KRE3|Q3KRE3_RAT	Calcineurin B homologous	Chp1	2	48.5	7.2293	68	0	10.596	3.015120255	2.68945826	3.026353947	3.032143988	2.602556795	2.799389291
tr|D4A978|D4A978_RAT	Tubulin alpha-8 chain (Alp	Tuba8	5	10.4	89.47	788	0	26.523	4.826712217	4.253164256	4.256391006	4.295193703	4.288792221	4.381141763
sp|Q6PDU7|ATP5L_RAT	Ephrin B3 (Ephrin B3 (Pred	Efnb3	8	65	11.433	103	0	134.46	177.5788332	191.0197408	178.2705651	194.106001	170.1085497	164.0265559
tr|A0A0G2K2L1|A0A0G2K	Cytoplasmic dynein 1 light	Dync1li1	4	11.6	49.749	466	0	69.988	16.58210483	7.043026548	15.05193459	12.08360964	18.99108256	14.037495
tr|F1LQN3|F1LQN3_RAT	Ataxin-10 (Neuronal beta-	Atxn10	7	8.3	126.4	1163	0	46.315	14.60572845	15.05394606	15.43776082	12.50592833	14.63549773	15.98741798
sp|Q9WUH4|FHL1_RAT F	Ubiquitin thioesterase OTU	Otub1	7	31.1	31.904	280	0	40.666	19.83672666	21.63133761	19.23163151	19.5520964	22.73761821	20.51243008
sp|P62744|AP2S1_RAT A	Protein transport protein S	Sec61a1	2	14.1	17.018	142	0	2.7344	5.006129077	4.446505037	4.471134452	4.303614034	4.686276953	4.550727062
tr|M0RBJ0|M0RBJ0_RAT	60S ribosomal protein L38	Rpl38	2	68.2	4.8827	44	0	9.0938	4.789632733	4.061297906	4.199119107	3.734113164	4.149253884	4.583627842
tr|G3V996|G3V996_RAT	Protein disulfide isomerase	Creld1	2	5.3	41.967	360	0.0017873	1.6932	1.872354473	1.316334432	1.828565051	1.41092636	2.193754608	1.923951379
tr|G3V8Z9|G3V8Z9_RAT	FAST kinase domain-conta	Fastkd2	2	9.5	30.219	275	0	6.8004	4.559181745	3.752095342	4.202626618	4.122186274	4.136628053	3.78600207
tr|D3ZEA3|D3ZEA3_RAT	S-phase kinase-associated	Skp1	2	6.9	50.162	434	0	7.3511	1.180124359	1.234194327	1.494461522	1.073548854	1.449942659	1.116008826
sp|Q6AY94|TIDC1_RAT C	Dedicator of cytokinesis pr	Dock9	3	16.1	32.05	285	0	28.75	8.247992371	9.36692091	9.761874803	8.990222937	8.48089543	9.017174747
sp|P07150|ANXA1_RAT A	Calpain-1 catalytic subunit	Capn1	13	40.8	38.829	346	0	52.714	39.84409869	44.15494412	37.05711749	39.578596	42.45746639	43.19857003
tr|M0R8T0|M0R8T0_RAT	Wiskott-Aldrich syndrome p	Wasf2	3	8.3	54.619	481	0	10.18	2.725501573	3.391097426	3.287846759	2.765992068	2.981135489	3.225918903
tr|D3ZTR1|D3ZTR1_RAT	OX-2 membrane glycoprot	Cd200	2	14.2	13.999	127	0	6.073	11.84470234	13.24705398	12.92125242	14.04328987	10.17262604	11.62306023
sp|P08753|GNAI3_RAT G	NAD-dependent protein de	Sirt2	4	16.4	40.522	354	0	19.292	14.83976778	13.84205846	13.79551267	13.54675074	14.86053846	12.89392343
sp|Q920J3|CORO6_RAT	Glucan (1,4-alpha-), branc	Gbe1	2	5.7	52.948	472	0	2.9195	1.230002246	0.615836762	0.996342582	1.098549426	1.034271496	1.043894012
sp|P70566|TMOD2_RAT	IST1 homolog	Ist1	4	14.5	39.491	351	0	9.8027	5.283228448	3.564890099	5.378532832	4.279915575	5.946766718	5.315452054
sp|P62982|RS27A_RAT U	BCL2-like 13	Bcl2l13	7	50.6	17.951	156	0	121.04	33.91656436	31.6408253	33.34857879	27.58483216	36.87854968	42.61035952
sp|Q5U204|LTOR3_RAT	28S ribosomal protein L42	Mrpl42	2	31.5	13.58	124	0	6.9495	2.327236016	1.695120652	1.928031786	1.734284449	2.087318193	1.750875825
tr|F1LQ81|F1LQ81_RAT	E3 UFM1-protein ligase 1	Ufl1	12	19.1	82.629	744	0	36.097	23.23209105	21.03395503	24.20601529	20.27954358	23.45526263	22.48545033
tr|G3V7V6|G3V7V6_RAT	LUC7-like 2 (S. cerevisiae)	Luc7l2	7	14.3	67.477	609	0	37.624	11.61106172	16.2953223	25.87182135	13.99554572	13.86813501	18.02408404
tr|A0A140TAJ5|A0A140TA	Immunoglobulin heavy con	Ighm	5	2.7	231.7	2044	0	7.3225	8.015547862	7.645640988	7.405560195	6.897293134	8.372300194	6.855783419
tr|D4A9D8|D4A9D8_RAT	Lactoylglutathione lyase (E	Glo1	3	6.2	77.806	682	0	4.216	2.386403709	1.941255595	1.99074818	1.898828142	2.217370801	1.902239944
tr|D4A899|D4A899_RAT	Insulin-degrading enzyme	Ide	2	1.1	266.49	2335	0	2.4709	0.588128465	0.524136633	0.624127588	0.638903498	0.50219409	0.715707449
sp|A6ZIQ8|TM38A_RAT	Mitochondrial ribosomal pr	Mrpl35	4	17.2	33.276	297	0	30.978	14.14881352	17.13337016	19.01961031	15.3237011	29.28407922	14.60158169
sp|Q9WV97|TIM9_RAT Mi	26S proteasome regulator	Psmc3	3	38.2	10.376	89	0	26.814	24.24200861	31.93856535	27.4276905	28.69596868	24.4411242	26.0634743
sp|P56499|UCP3_RAT Mi	LYR motif-containing prote	Lyrm1	3	13	34.014	308	0	11.649	2.940881545	2.677757932	2.921338015	2.920292472	2.369142456	4.309540222
tr|Q5XI38|Q5XI38_RAT L	Transforming growth factor	Tgfbi	5	11.8	70.121	627	0	8.1697	6.85252791	7.148139248	4.791836147	7.09651644	6.311084109	6.76288106
sp|Q9QUR2|DCTN4_RAT	Taxilin beta	Txlnb	2	6.6	53.088	467	0	6.0264	0.230116077	0.166701133	0.307975188	0.147616223	0.249886797	0.221081949
sp|Q99J82|ILK_RAT Integ	Leucyl-tRNA synthetase 2	Lars2	8	19.5	51.373	452	0	17.728	22.84375322	22.40898135	25.53834603	20.11026888	26.48938736	29.27758793
tr|M0RC65|M0RC65_RAT	Coxsackievirus and adeno	Cxadr	5	32.5	18.709	166	0	88.458	14.22297249	10.49462323	10.66073998	9.756299482	12.03967907	11.19961026
tr|D3ZT71|D3ZT71_RAT B	Vesicle transport through i	Vti1b	2	5.8	46.734	434	0	5.7086	3.641803409	2.86648517	2.936676832	2.994252496	3.106347104	2.888493432
sp|Q5I0K8|RT07_RAT 28S	40S ribosomal protein S9	Rps9	7	36	28.197	242	0	51.261	32.14910895	36.15686643	29.14375343	33.373593	30.34059312	29.84013481
sp|Q6MG61|CLIC1_RAT C	Mitochondrial 2-oxodicarbo	Slc25a21	7	48.5	26.98	241	0	143.94	12.38574385	13.24087088	11.40988155	13.17130813	10.59065228	11.75959077
sp|Q6AYH5|DCTN2_RAT	DnaJ heat shock protein fa	Dnajc13	4	15.9	44.147	402	0	7.3861	5.951855277	5.660390258	9.676542814	6.435911056	6.109921247	6.441572352
tr|D3ZZ20|D3ZZ20_RAT A	Mitochondrial ribosomal pr	Mrpl58	5	6.7	87.202	789	0	4.6124	8.635931491	7.839219581	7.472569364	8.09784142	7.529705832	7.674453371
tr|Q0D2L2|Q0D2L2_RAT	Dimethylaniline monooxyge	Fmo1	8	24.5	41.235	359	0	64.273	17.5055036	18.34668463	20.54406891	19.38585995	18.2093277	20.69515516
tr|Q4V8H5|Q4V8H5_RAT	Very-long-chain (3R)-3-hyd	Hacd3	8	18.5	52.555	475	0	13.357	24.69214558	23.28507906	24.50075093	22.12811016	22.2737988	36.53115769
tr|A0A0A0MY48|A0A0A0M	Mitochondrial import inner	Magmas-ps1	9	12.9	97.253	860	0	40.361	20.25895434	20.85987698	22.23186235	18.51301013	20.75215711	30.77993711
tr|D3ZYU4|D3ZYU4_RAT	Ubiquitin-specific peptidas	Usp24	4	35.9	13.078	117	0	66.264	21.42516392	23.77401958	19.13687636	18.74999472	21.99118951	20.89943769
tr|G3V6N2|G3V6N2_RAT	Plexin D1 (Plexin D1 (Pred	Plxnd1	2	8.4	26.117	227	0	23.023	10.37507891	9.937668604	10.0539929	11.2046833	9.516475302	10.5220903
sp|P62804|H4_RAT Histor	ERO1-like protein alpha (E	Ero1a	6	51.5	11.367	103	0	51.151	68.04683414	55.72875612	67.70543617	42.32084621	60.8323037	72.19180488
sp|P62278|RS13_RAT 40	Mitochondrial ribosomal pr	Mrpl53	4	27.2	17.222	151	0	4.4431	29.97497532	20.8356202	25.62996013	17.79033736	24.33710828	27.88661892
tr|B1WC67|B1WC67_RAT	Epoxide hydrolase 1 (EC 3	Ephx1	6	18.9	52.89	475	0	19.284	8.568151789	10.21448124	8.114915071	9.297955668	8.846586617	9.93490633
tr|D3ZRH1|D3ZRH1_RAT	Leucine-rich repeat-contai	Lrrc57	3	6.7	48.023	433	0	4.6764	24.82730628	29.65319646	19.55934823	22.95495198	19.75713691	25.75910359
sp|P27615|SCRB2_RAT L	Proteasome subunit beta	Psmb1	4	8.6	54.09	478	0	6.6928	22.01444863	18.9312254	23.78930202	18.97526029	20.7698202	22.22111489

tr|D3ZFD0|D3ZFD0_RAT M	Prostaglandin reductase 2	Ptgr2	6	3.9	232.66	2044	0	13.272	7.576175909	6.137915829	7.253219038	6.703712319	7.405410079	7.715001914
tr|D3ZUX5|D3ZUX5_RAT M	Elongation factor Ts, mitoc	Tsfm	12	47.1	26.434	227	0	323.31	117.5858225	123.1625962	114.92596	125.9751028	111.8858026	107.9207173
sp|B1PRL5|CAVN4_RAT (	Carboxypeptidase D (EC 3	Cpd	4	15.2	41.093	362	0	87.56	13.74851457	10.20163941	12.80293936	11.70903511	14.78007331	12.49870345
sp|Q6AYT3|RTCB_RAT tR	BCL2-interacting protein 3	Bnip3	6	16	55.249	505	0	12.123	8.248789779	7.043502171	7.090931206	6.135556966	6.96906657	8.150770434
tr|F1M6D0|F1M6D0_RAT M	Acid phosphatase 6, lysop	Acp6	3	25.6	14.341	125	0	9.6575	5.521254815	5.65610965	6.103069472	6.291810539	5.103321911	6.745943061
sp|Q704E8|ABCB7_RAT A	Tricarboxylate transport pr	Slc25a1	11	17.4	82.557	752	0	112.64	46.30549785	58.2590709	53.47645794	61.03438176	47.46920196	58.0562748
tr|F7FDM3|F7FDM3_RAT M	Serine/threonine-protein ki	Pak2	5	9.5	79.21	727	0	26.17	13.24255903	12.79949267	13.17724839	14.13356971	12.14565756	14.06161882
tr|D3ZGM1|D3ZGM1_RAT	Ankyrin 2	Ank2	9	19.1	77.89	687	0	55.061	16.60841931	16.32243281	16.63921428	18.09676798	15.19286605	17.86650932
tr|Q6P136|Q6P136_RAT H	Peroxisomal membrane pr	Pex14	11	14.9	111.22	998	0	78.46	21.66119677	20.89174373	20.34147087	21.43582003	19.72377331	23.75836662
tr|A0A0G2KAK2|A0A0G2K	MIA SH3 domain ER expo	Mia2	9	19.7	72.372	638	0	132.04	37.91038365	25.08531241	32.14607831	29.26586018	37.71394797	35.20126813
sp|D3ZUI5|COBL_RAT Pro	Mitochondrial pyruvate car	Mpc1	2	2.1	122.27	1133	0.0013569	1.7804	0.49216038	0.550153215	0.640042266	0.480696756	0.680224859	0.636252836
tr|D3ZIQ3|D3ZIQ3_RAT Fa	Immunity-related GTPase f	Irgm	2	7.9	32.262	292	0	2.2106	1.149264659	0.957571944	1.392534294	1.223725899	1.242237915	1.241350019
tr|A0A0G2K1I6|A0A0G2K	Mitochondrial import recep	Tomm34	4	15.8	33.955	304	0	33.142	2.638703684	2.827959701	2.936833885	2.506697945	2.966416255	3.338530933
sp|P0C2C0|RM22_RAT 39	Vesicle transport protein S	Bnip1	6	31.1	24.039	206	0	6.2378	9.410214915	12.4560928	12.19095718	12.68258167	11.8996828	11.15803517
sp|Q9NQR8|NDUF4_RAT	Cytochrome c oxidase ass	Cox18	9	58.6	20.158	174	0	65.717	30.70460388	29.88720301	30.92368449	31.45775058	29.99452757	30.87335275
tr|F1LN92|F1LN92_RAT A	Transmembrane emp24 do	Tmed5	27	37.2	89.35	802	0	323.31	134.2755774	143.0626658	134.1020995	143.1803574	136.7842044	137.0078811
sp|Q4V7C6|GUAA_RAT G	Coiled-coil domain contain	Ccdc58	2	3.2	76.757	693	0.0026362	1.5957	3.486627761	3.437946299	3.596507744	3.14659973	4.35630444	3.596399136
sp|Q5EB94|MYZAP_RAT	Isoleucyl-tRNA synthetase	Iars	5	12.4	53.955	466	0	7.062	3.915673277	4.094924457	4.270316349	3.77078935	3.822552312	4.358044491
tr|Q6AXM6|Q6AXM6_RAT	Polypeptide N-acetylgalact	Galnt2	5	23.1	31.091	277	0	22.463	17.25750963	12.10841233	19.23529607	13.5198404	16.80609408	14.64572315
sp|P70580|PGRC1_RAT M	Sideroflexin-1 (Tricarboxyla	Sfxn1	3	21.5	21.598	195	0	10.444	6.328630681	5.639462843	6.026637139	5.375990986	6.321354864	5.784583299
tr|M0R6W5|M0R6W5_RAT	Guanine nucleotide excha	Mcf2l	2	4.1	49.66	434	0.0075251	1.2431	1.023951951	1.107631025	1.330184356	1.002062844	1.277171563	1.410678681
tr|A0A0G2K6B2|A0A0G2K	28S ribosomal protein S15	Mrps15	2	3.8	66.962	580	0	2.6224	2.956431006	2.840706399	3.738954579	2.744116568	3.22050294	3.084358293
tr|Q6P9V1|Q6P9V1_RAT	ATP-binding cassette subf	Abca9	2	15.3	25.904	236	0	236.25	21.70744645	16.69769942	20.21425815	16.21000608	21.4554094	17.73562478
sp|P97584|PTGR1_RAT P	GPRIN family member 3 (R	Gprin3	2	7.6	35.718	329	0	3.6875	3.38424054	3.246936071	3.796540583	3.518353022	3.687462455	3.496670251
tr|B0BMW0|B0BMW0_RAT	Nucleoside-triphosphatase	Ntpcr	8	57.2	23.897	215	0	156.6	23.72768028	25.22038936	26.33983852	24.87860708	26.25453381	25.4619187
sp|P12368|KAP2_RAT cA	Serine/threonine-protein ki	Cdc42bpa	3	12	45.48	401	0	20.289	6.29952528	5.410212519	5.543438209	5.563495274	6.547115201	5.752247119
sp|P61149|FGF1_RAT Fib	Ras suppressor protein 1	Rsu1	2	15.5	17.417	155	0.00046041	1.9122	1.382386965	0.866204751	1.072199055	1.006142799	0.871444061	2.073519043
tr|G3V8V4|G3V8V4_RAT	OXA1L, mitochondrial inne	Oxa1l	2	6.6	44.951	380	0.00091366	1.8543	2.129957212	1.975928518	2.292341792	1.994446648	2.246874687	1.968708758
tr|D3Z9L0|D3Z9L0_RAT A	Galectin-3 (Gal-3) (35 kDa	Lgals3	11	32.4	54.258	490	0	133.41	69.58981913	79.33868574	73.86190357	79.32907093	66.9757848	69.5125214
sp|P26772|CH10_RAT 10	LYR motif-containing prote	Lyrm9	9	72.5	10.902	102	0	40.608	136.7993746	162.0210017	177.5638277	162.7250751	136.80383	155.8193255
tr|D3ZEH2|D3ZEH2_RAT F	Translocase of inner mitoc	Timm29	4	11.7	54.229	487	0	27.827	8.032293436	8.649205684	7.557377844	16.31591128	5.556412637	7.578471376
sp|A1L1I3|NUMBL_RAT N	28S ribosomal protein S10	Mrps10	2	2	65.512	614	0	2.3391	2.388756064	2.047509791	2.449551584	1.879687079	2.1985956	2.489680546
tr|G3V8C3|G3V8C3_RAT	Proteasome subunit alpha	Psma3	14	31.3	53.7	466	0	97.314	85.23496926	55.17227712	87.30038302	66.66819114	99.58706505	81.96964977
tr|F1LST1|F1LST1_RAT F	Retinoid-inducible serine c	Scpep1	17	10.5	262.75	2387	0	194.25	34.3443739	33.44676613	37.29636116	30.83143418	34.95785322	36.33662734
tr|A0A0G2JT25|A0A0G2J	Protein S100-A8 (Calgranu	S100a8	2	12	22.865	217	0.00047192	2.112	1.192843021	1.092030588	1.39462833	1.136267302	1.27017175	1.46108206
tr|D4A3B0|D4A3B0_RAT	Dehydrogenase/reductase	Dhrs7b	41	24.1	253.64	2375	0	323.31	91.79763927	84.93201314	115.7426342	80.40548443	94.13113572	101.5304723
sp|P20411|FCERG_RAT H	CAAX prenyl protease (EC	Zmpste24	2	15.1	9.7644	86	0	2.2655	2.814532206	1.787629341	2.18104375	2.211291884	2.209258867	2.915542903
sp|P97710|SHPS1_RAT T	AFG3(ATPase family gene	Afg3l1	3	8.8	55.69	509	0	4.2228	3.052239609	3.05402335	4.004111955	2.356607707	3.692892217	3.321695588
tr|F1LNG8|F1LNG8_RAT F	Transmembrane protein 43	Tmem43	2	11.2	18.75	169	0	2.7016	3.19804571	4.018539391	3.978250603	3.077544331	3.589203392	3.987204968
tr|A0A140UHX6|A0A140U	26S proteasome regulator	Psmc4	94	50.5	268.02	2329	0	323.31	364.2162242	237.6022651	343.8303284	271.4775619	387.3775246	362.8222043
tr|D3ZM69|D3ZM69_RAT E	Mitochondrial ribosomal pr	Mrpl4	15	21.7	110.71	996	0	126.61	32.00437935	24.60731122	30.95143047	26.34435241	29.12053218	28.41118362
tr|Q6P3V8|Q6P3V8_RAT	40S ribosomal protein S18	Rps18	2	3	46.153	406	0	22.482	18.48073391	18.29769546	17.912912	15.50816712	18.73987431	18.76730291
sp|P02651|APOA4_RAT A	Transmembrane 9 superfa	Tm9sf4	2	7.4	44.456	391	0	2.5747	6.795114515	6.435180253	5.664892328	6.650325681	6.681877964	6.118210552
sp|P23928|CRYAB_RAT A	Succinate dehydrogenase	Sdhd	10	57.7	20.089	175	0	314.78	87.39594565	68.61814155	80.6675223	61.67241718	89.42752278	71.61180673
tr|D4AC65|D4AC65_RAT (	Antigen peptide transporte	Tap1	2	10	25.711	231	0	6.3504	7.337352134	5.907714259	6.661130208	6.232781411	6.864396463	6.129502552
sp|Q32PX9|AFG1L_RAT A	Arginine-hydroxylase NDUI	Ndufaf5	8	19	54.502	480	0	43.983	45.48416734	47.63840755	44.31347758	51.32929873	42.90754785	47.00910653
tr|Q6AXW2|Q6AXW2_RAT	Mitochondrial fission factor	Mff	6	27.3	39.471	352	0	55.805	14.13007442	11.43825942	13.48925983	11.51892659	12.8397512	13.32712559
tr|D3ZNY3|D3ZNY3_RAT M	Lysocardiolipin acyltransfe	Lclat1	3	8.7	46.329	415	0	9.2043	3.388546544	3.772689822	3.719322986	3.729642576	3.575923372	3.764444617
sp|P25113|PGAM1_RAT F	Canopy 2 homolog (Zebra	Cnpy2	5	26.4	28.832	254	0	23.724	6.716968505	6.337677522	6.033966267	5.447607207	6.982150334	5.955503107
tr|G3V9N0|G3V9N0_RAT	Syntaxin-5	Stx5	2	4.2	70.833	644	0	65.746	5.593818967	6.043266836	6.070611906	5.349948724	6.430277194	6.503678348
tr|Q6P7Q2|Q6P7Q2_RAT	40S ribosomal protein S13	Rps13	17	42.8	61.628	558	0	196.8	72.22525344	78.00694112	79.83514278	78.58252608	77.69138702	68.20880875
sp|Q5XIE8|ITM2B_RAT In	Mitochondrial poly(A) polyn	Mtpap	4	26.7	30.314	266	0	22.842	5.620930848	5.59570552	5.148084112	5.605596931	5.548431541	5.44223041
sp|E9PT87|MYLK3_RAT M	AarF domain containing ki	Adck1	8	15.8	85.54	786	0	72.864	17.23877054	16.43039925	17.42971307	16.78944642	16.98599582	17.96711077
tr|A0A0G2K876|A0A0G2K	Mitochondrial ribosomal pr	Mrpl48	2	2.5	103.27	916	0	2.6356	1.235504363	0.892031084	1.199673529	1.055883519	1.003197558	1.107077881
sp|Q5FVM7|DJC16_RAT D	60S ribosomal protein L28	Rpl28	3	5.7	88.868	771	0	3.8462	1.035514371	1.108867645	1.306783498	1.124418073	1.010393628	1.583600253
sp|O35115|FHL2_RAT Fo	Dephospho-CoA kinase do	Dcakd	5	26.2	32.086	279	0	19.395	9.004732813	7.746473081	9.162980355	8.343940797	9.019946482	9.262005824
sp|P19234|NDUV2_RAT N	N(G),N(G)-dimethylarginine	Ddah2	11	52.8	27.378	248	0	323.31	308.6288942	339.8422021	288.1865426	335.9061185	298.3490566	327.5911664
tr|A0A0G2K0W9|A0A0G2I	Sodium/nucleoside cotrans	Slc28a2	6	28.6	27.855	248	0	43.256	33.35279672	27.72359363	31.73093557	27.27145694	33.63377636	36.09128299
tr|Q6AYS3|Q6AYS3_RAT	Coatomer subunit gamma-	Copg1	2	4.4	51.215	456	0	2.4235	0.728352708	1.06877262	0.964879683	1.015648224	0.496411067	0.986048179
sp|Q68FU7|COQ6_RAT U	Nidogen-2 (NID-2)	Nid2	8	21.8	51.495	476	0	22.786	31.61604153	27.2489218	22.51089272	26.76189668	29.88069883	28.54412125
tr|E9PU34|E9PU34_RAT F	Protein FAM98A	Fam98a	4	10.9	51.698	450	0	6.2984	8.325739677	9.787847334	8.54419256	9.107413117	9.038917939	9.153192012
tr|D4A0T8|D4A0T8_RAT D	Antigen peptide transporte	Tap2	2	9.1	38.493	340	0	47.572	2.781639115	2.240993259	2.502164252	2.327440373	2.528829788	2.441432912
sp|P00173|CYB5_RAT Cy	Tyrosine-protein phosphat	Sirpa	5	47.8	15.355	134	0	109.5	17.19531179	15.55287467	15.69480381	14.33235898	16.67329388	16.26253214
sp|Q6Q7Y5|GNA13_RAT	Transmembrane emp24 do	Tmed2	10	25.5	44.011	377	0	23.488	18.6146985	14.17309212	18.5662514	13.93478045	18.08306938	16.83072502
sp|Q6AY04|MAIP1_RAT m	Oxysterol-binding protein	Osbp	5	17.2	33.09	291	0	11.435	17.14627118	20.78710665	17.19779853	18.0555344	17.61728741	21.79099237
tr|F1LRT9|F1LRT9_RAT C	Raftlin lipid raft linker 1 (Sir	Rftn1	68	18.2	531.8	4643	0	323.31	196.8202946	203.3003287	229.0195404	186.818508	221.5997006	202.4706809
tr|F7FL53|F7FL53_RAT So	Late endosomal/lysosoma	Lamtor2	2	5.3	67.146	606	0	7.6154	2.838773417	0.758713935	2.155758259	1.435579702	1.757803612	1.705348537

tr|E9PT53|E9PT53_RAT W	CD59 glycoprotein (MAC-in	Cd59	4	6.1	100.56	890	0	10.796	4.479839623	4.060061286	4.805970894	3.790841892	3.857943892	5.07565105
tr|F1MAN8|F1MAN8_RAT	Oxygen-dependent coprop	Cpox	6	2.1	403.77	3713	0	8.983	7.866831221	8.957885062	10.64712875	9.188144129	9.317602099	10.12019777
tr|F1LQC7|F1LQC7_RAT	Fragile X mental retardatio	Fxr1	5	6.2	133.19	1202	0	50.438	5.862944256	5.456823581	6.772637654	4.95584249	6.481107615	5.901609474
tr|A0A0G2JYU2|A0A0G2J	Transmembrane protein 18	Tmem182	4	26.6	20.75	192	0	20.054	10.4994746	11.1514587	11.94647841	12.78154227	10.77251659	11.01842499
sp|P10111|PPIA_RAT Pe	Mitochondrial ribosomal pr	Mrpl43	5	42.1	17.874	164	0	131.72	38.4067703	35.86435824	28.68672996	28.85960089	37.31554738	31.92453523
tr|A0A0G2JVL8|A0A0G2J	Phosphatase domain-cont	Pald1	2	2.2	143.58	1254	0	4.8819	2.473241469	1.967842925	2.427878306	2.37010628	2.050487399	2.785376947
tr|Q7TP91|Q7TP91_RAT	Intercellular adhesion mole	Icam1	7	14.9	83.262	733	0	73.348	19.8602502	20.17926035	19.34523299	19.78864694	19.50004096	20.61970407
tr|M0R7G4|M0R7G4_RAT	Coagulation factor XIII A c	F13a1	5	31.1	22.369	196	0	115.86	60.41962411	68.07593124	58.77960545	69.91479316	61.93788171	60.54564738
sp|P63004|LIS1_RAT Plat	Type 2 phosphatidylinosito	Pip4p2	4	15.4	46.67	410	0	12.019	5.725790034	5.733636212	5.431407255	5.523563805	6.228460143	5.479186044
sp|P39069|KAD1_RAT Ad	C-type lectin domain family	Clec2d11	4	25.8	21.584	194	0	7.144	26.03179146	30.97780673	24.25051357	21.53304447	27.73038232	28.32392726
sp|Q8VIF7|SBP1_RAT Me	Fermitin family member 3 (	Fermt3	6	16.9	52.531	472	0	13.888	12.44674558	11.33742732	9.9566202	10.14519726	12.32425093	9.976481418
sp|P20788|UCRI_RAT Cyt	Dipeptidyl peptidase 2 (EC	Dpp7	18	53.6	29.445	274	0	323.31	237.009671	249.0600251	220.5282223	236.5418672	231.1377641	227.6672376
sp|Q63355|MYO1C_RAT	60S ribosomal protein L35	Rpl35a	25	29.5	119.81	1044	0	323.31	111.1507378	84.57529583	112.3660003	84.57224638	109.8643612	99.20534694
sp|P62749|HPCL1_RAT H	ATPase Get3 (EC 3.6.-.-)	Get3	3	14.5	22.338	193	0	2.8925	6.748466132	6.69724857	7.504503421	5.066956142	8.149876216	6.531908347
tr|G3V8L9|G3V8L9_RAT	Kynurenine--oxoglutarate t	Kyat1	19	45.2	43.908	392	0	323.31	165.1871088	146.2873903	149.9277806	154.5868682	166.7591063	149.5420004
tr|B5DF57|B5DF57_RAT	COP9 signalosome comple	Cops8	12	20.6	76.5	689	0	149	55.65909677	38.45745526	52.57602223	44.04614607	57.08707643	53.65752778
tr|A0A0G2JVH4|A0A0G2J	Golgi autoantigen, golgin s	Golgb1	38	46.1	86.229	776	0	323.31	436.8999874	506.586141	466.8392693	503.5271389	479.6573078	456.899957
sp|Q5XHZ0|TRAP1_RAT	Mitochondrial calcium unip	Mcur1	13	24.1	80.46	706	0	83.562	37.5874333	31.06056515	43.53240195	34.93829892	35.19270677	37.31030564
tr|G3V6R5|G3V6R5_RAT	Calcium binding protein 39	Cab39	7	22.3	60.805	546	0	58.578	2.563348603	2.311100101	3.444637744	2.73170309	3.095487581	2.949572883
sp|P97829|CD47_RAT Le	Adenylosuccinate lyase (A	Adsl	3	10.9	32.995	303	0	71.852	24.77746826	16.94549904	22.77997641	18.52993761	26.84264897	23.18914719
sp|P14669|ANXA3_RAT A	Alpha-2 antiplasmin (Serin	Serpinf1	16	48.5	36.363	324	0	85.283	26.89538461	29.05153326	25.56609202	27.96895553	26.35266203	25.72214796
tr|A0A0G2JZ56|A0A0G2J	Sialic acid binding Ig-like le	Siglec1	3	2.3	202.65	1848	0	4.971	1.529867623	1.433195023	1.822701748	1.328025159	1.650843846	1.620761196
tr|D3ZLA3|D3ZLA3_RAT	DnaJ homolog subfamily C	Dnajc16	3	6.9	59.685	533	0	6.4178	10.07246248	8.884163485	9.35301417	8.533615273	9.144242234	9.983153964
tr|M0RBJ7|M0RBJ7_RAT	V-type proton ATPase sub	Atp6v1e1	20	16.3	186.23	1662	0	117.47	35.43164007	31.49338215	30.83521144	30.45165119	32.1716658	38.60426611
sp|B2GV06|SCOT1_RAT	FYVE and coiled-coil doma	Fyco1	18	46.2	56.203	520	0	323.31	255.1905794	258.0873511	270.7641587	272.4888698	255.4997315	264.0788028
sp|Q63258|ITA7_RAT Inte	PDZ and LIM domain prote	Pdlim5	13	13.7	124.19	1135	0	168.7	37.13131578	31.82346456	39.01661217	27.87910972	37.14349589	38.30656795
sp|Q64428|ECHA_RAT Tr	alpha-1,2-Mannosidase (E	Man1c1	38	60.2	82.664	763	0	323.31	1292.519054	1401.470963	1235.429203	1384.753887	1162.754053	1288.673761
tr|G3V6T1|G3V6T1_RAT	SEC24 homolog C, COPII	Sec24c	6	6.5	138.36	1224	0	18.14	10.287364	9.80877475	9.835166082	9.721576466	10.00711643	10.52722302
sp|Q66HG5|TM9S2_RAT	C4b-binding protein alpha	C4bpa	8	16	75.585	663	0	21.833	18.14103799	17.04728238	17.93961097	14.43739611	19.88208685	21.37934766
sp|Q5EB77|RAB18_RAT	6-phosphogluconate dehy	Pgd	8	45.6	22.976	206	0	65.794	64.27110602	50.86313202	56.9996744	54.14186304	60.88529294	62.38829636
tr|D4A305|D4A305_RAT	Elastin microfibril interfacer	Emilin1	3	27.1	16.69	144	0	4.8912	10.34836574	9.020191685	9.23836567	8.87433487	10.56840988	8.246752428
sp|P04642|LDHA_RAT L-	Ly6-C antigen (RCG59507	LOC1009111	13	39.5	36.45	332	0	323.31	86.3393797	78.06877212	85.53092215	127.4291291	83.33703092	62.27537637
sp|Q9EQP5|PRELP_RAT	Golgin subfamily A membe	Golga4	3	8.5	43.179	377	0	3.0467	5.317517003	6.377629861	6.539676089	4.435431284	7.903247276	7.203269035
tr|A0A0G2JSM7|A0A0G2J	Endonuclease/exonucleas	Eepd1	5	9.7	80.338	735	0	11.317	13.7026636	11.22232654	15.43776082	11.57708765	14.95539574	12.03778457
sp|Q9JJK4|PEX3_RAT Pe	39S ribosomal protein L23	Mrpl23	2	5.6	42.209	372	0	3.1709	2.873540417	3.41097847	4.59196036	3.954821336	4.129170307	3.421219164
tr|B5DFE0|B5DFE0_RAT	S-formylglutathione hydrola	Esd	3	7	60.933	540	0	8.7055	5.960626768	4.417744467	5.692838312	4.962353055	5.253981446	5.28619551
tr|G3V940|G3V940_RAT	Coronin-1B (Coronin-2)	Coro1b	2	4.3	53.938	484	0	3.7591	5.314726075	5.506764004	6.019308011	4.451056642	5.664419104	6.116157462
tr|Q5RJN0|Q5RJN0_RAT	Peptidyl-prolyl cis-trans iso	Fkbp4	7	34.4	23.945	218	0	152.37	189.7273481	222.0208531	215.2093695	182.4824714	241.3954346	177.6128842
tr|F1M7L9|F1M7L9_RAT	RBR-type E3 ubiquitin tran	Arih1	5	9	88.596	792	0	11.284	8.010364708	7.871086327	10.29637763	9.495008785	10.1104781 6	7.727320459
tr|D3ZAC0|D3ZAC0_RAT	A-kinase anchor protein 12	Akap12	4	6.3	112.64	1034	0	12.851	2.785466675	3.967267223	4.370463646	3.188918406	4.558056071	4.049259637
tr|D4ABM3|D4ABM3_RAT	Aminopeptidase B (AP-B)	Rnpep	4	4.9	112.64	968	0	6.2583	6.087015978	4.693448539	6.082652616	4.799154879	5.884030072	5.564389312
tr|A0A0H2UHV7|A0A0H2U	Peptidyl-prolyl cis-trans iso	Fkbp1a	2	3	99.264	907	0	2.8185	0.354432025	0.438091661	0.368466668	0.422487959	0.215993308	0.437472585
tr|M0R589|M0R589_RAT	V-type proton ATPase sub	Atp6v1b2	3	10.1	42.13	377	0	18.421	3.712693003	4.428241111	3.546146165	3.706899	3.859056012	3.807970142
tr|D4A8Y1|D4A8Y1_RAT	Arginine--tRNA ligase, cyto	Rars1	3	6.9	70.994	655	0	7.7408	2.9123742	2.892026129	3.901033006	2.923374139	3.570820705	3.642491024
sp|Q01177|PLMN_RAT Pl	Prostaglandin reductase 1	Ptgr1	15	26.6	90.535	812	0	153.94	49.28381771	47.7382884	43.7182477	39.00219393	55.16637996	40.591658
tr|A0A0H2UHA6|A0A0H2U	Adenosylhomocysteinase	Ahcy	2	8.1	27.884	246	0	2.3736	3.08126527	2.757519923	2.769572717	2.981448384	2.860568609	2.889263341
tr|D4A678|D4A678_RAT S	Cyclin Y	Ccny	92	48.8	280.07	2416	0	323.31	376.4564411	222.8198999	323.3715915	246.6896687	377.4207807	362.4629134
sp|Q5XIC0|ECI2_RAT En	Cd300 molecule-like family	Cd300lg	11	29.2	43.021	391	0	170.38	123.1118618	132.4515149	120.4437462	137.5725903	121.0640627	123.9502237
sp|Q5XIB4|UFSP2_RAT	Ribonuclease inhibitor (Rib	Rnh1	3	9.1	52.306	461	0	6.1371	5.724593922	4.431713159	6.46690832	4.37206178	5.927729842	5.544371677
sp|P31044|PEBP1_RAT P	Calcium/calmodulin-depen	Camk2g	8	74.9	20.801	187	0	214.98	26.17971069	22.23633017	21.74552237	20.79214211	24.5353273	23.01155484
sp|P62716|PP2AB_RAT S	A-kinase anchor protein 1,	Akap1	4	19.1	35.575	309	0	29.738	6.595363745	5.144339218	5.464911839	5.431547812	5.622289386	5.667043851
tr|D4AE31|D4AE31_RAT	Signal peptidase complex	Spcs3	3	8.4	57.154	521	0	12.961	7.157137867	2.926033179	2.570220439	3.781900715	3.689359601	3.57997441
sp|P05943|S10AA_RAT P	Ectonucleoside triphospha	Entpd2	2	17.9	11.075	95	0	25.375	13.93032365	12.88700731	14.09548341	11.56016017	15.45519551	12.59725181
sp|P46462|TERA_RAT Tra	Adaptor protein, phosphot	Appl1	36	58.8	89.348	806	0	323.31	149.3665288	250.7294621	173.5066319	189.8654527	200.1684961	148.1561641
tr|A0A0G2JWK0|A0A0G2J	ADP-ribose pyrophosphata	Nudt9	5	11.1	62.389	567	0	6.9634	7.40832147	5.767881074	9.09335364	6.185905339	8.603228618	8.755918944
tr|F1LNF0|F1LNF0_RAT M	Clustered mitochondria pro	Cluh	22	15.8	228.91	2000	0	171.69	61.64364579	48.24720509	68.99850373	54.41964717	64.40744205	67.37730698
tr|Q6IMZ5|Q6IMZ5_RAT T	Growth factor receptor-bou	Grb2	8	24	40.454	359	0	72.103	16.60483097	10.05229377	16.58215179	13.87922362	15.73060873	16.0151347
tr|D3ZA93|D3ZA93_RAT A	Peptidyl-prolyl cis-trans iso	Ppid	2	15.7	15.27	140	0	18.348	21.13570472	23.90671842	25.52368778	25.3473678	22.72060932	24.00576406
sp|P61203|CSN2_RAT CO	Non-lysosomal glucosylcer	Gba2	3	7	51.596	443	0	3.4129	0.180234203	0.093174561	0.251179682	0.132707028	0.146963372	0.1949769
sp|Q9ES71|GNPAT_RAT	Amyloid-beta A4 protein (A	App	7	14.5	77.075	678	0	29.176	11.64176194	13.46298686	10.45238335	13.17608254	10.98905287	12.45353546
tr|G3V6D9|G3V6D9_RAT	Methionine aminopeptidas	Metap2	12	39.5	37.35	337	0	110.53	29.97856366	19.74406524	30.73836225	22.81866415	27.5544057	26.26159756
sp|Q4V885|COL12_RAT C	EH domain-containing prot	Ehd3	3	5	81.545	742	0	8.6606	5.156839238	4.626385685	5.552861374	4.702364471	5.213487198	5.827698206
tr|D4A465|D4A465_RAT L	Heat shock protein beta-7	Hspb7	3	29.6	13.48	125	0	4.1809	12.36899828	8.707707323	11.23241196	9.842238947	10.38131206	10.950673
tr|D3ZH76|D3ZH76_RAT F	Adhesion G protein-couple	Adgrl2	7	16	83.366	788	0	22.442	3.773854217	3.975257691	4.11446768	4.28929079	4.149188465	3.640745897
sp|P49242|RS3A_RAT 40	FAM20B, glycosaminoglyc	Fam20b	10	41.7	29.945	264	0	22.518	19.45596421	13.59378321	15.52623387	12.19342118	14.11607232	19.58956577
tr|B2RYV0|B2RYV0_RAT	Latexin (Endogenous carb	Lxn	3	16.4	24.923	219	0	7.9444	1.637158905	1.716428566	2.034461193	1.862716205	1.420896704	1.915636361
tr|D4ADS9|D4ADS9_RAT	Transmembrane protein 10	Tmem109	6	10.6	92.523	819	0	14.184	11.93042373	8.649205684	11.60043888	9.272347444	11.53268324	10.19410904

sp|Q63377|AT1B3_RAT S	Ras-related protein Rab-4E	Rab4b	5	25.8	31.829	279	0	32.657	18.59436459	17.92480696	22.02559975	19.51259896	20.53365827	19.79076866
sp|Q63357|MYO1D_RAT	Cathepsin L1 (EC 3.4.22.1	Ctsl	11	12.9	116.09	1006	0	18.199	28.48142964	26.83465409	29.63637553	23.88900112	31.19169192	27.67874348
tr|D4A601|D4A601_RAT T	Reticulon	Rtn2	3	13.6	32.812	295	0	33.687	5.527235377	6.183100022	11.88051626	7.148600964	7.194107301	7.234578669
sp|Q5XI31|PIGS_RAT GP	ATP-dependent 6-phosph	Pfkp	3	7.9	61.724	555	0	10.076	4.046448232	3.824532737	4.497624014	3.895618593	3.774338644	5.166089699
sp|A2VD12|PBIP1_RAT P	Inorganic pyrophosphatas	Ppa1	17	33.5	80.275	722	0	258.31	65.94566337	63.20555091	65.97785603	62.60125787	65.48423577	65.77076344
sp|Q6IMY1|MTUS1_RAT M	COP9 signalosome comple	Cops2	6	16.6	50.744	440	0	10.378	10.25945471	7.072039555	13.80074776	8.227184655	9.412459384	11.44290151
tr|M0R757|M0R757_RAT E	Family with sequence simil	Fam173a	3	10.2	50.107	462	0	22	13.95105627	12.9093616	12.59510623	11.11527153	12.85021821	14.48250243
tr|A0A0G2JW01|A0A0G2J	Solute carrier family 25 me	Slc25a40	2	5.4	48.639	427	0.0017802	1.6717	2.019556038	1.791101389	2.959187725	1.894704784	2.402571472	2.103186205
tr|G3V667|G3V667_RAT I	ATPase inhibitor, mitochon	Atp5if1	7	8.4	119.48	1073	0	56.203	8.673010975	7.709850104	9.592781353	7.015351389	13.78701567	7.351091572
tr|G3V6H5|G3V6H5_RAT	Protein NDRG1 (N-myc dov	Ndrg1	15	54.1	34.136	314	0	312.57	291.8992688	358.7957818	328.0569982	342.6988086	309.7057632	305.5717676
sp|P21913|SDHB_RAT Su	Inositol monophosphatase	Impa1	17	53.5	31.83	282	0	323.31	377.8319703	414.8241804	380.2456237	399.9223393	387.2532288	372.7078365
tr|Q6AYF2|Q6AYF2_RAT	Eukaryotic translation initia	Eif3a	5	21.6	41.085	365	0	23.778	4.891302287	5.140534233	5.570137175	4.902021815	5.196543725	5.803061116
sp|Q5U2V8|EMC3_RAT E	Inactive phospholipid phos	Plpp7	4	20.3	29.98	261	0	30.653	3.186363679	3.112287177	3.52295471	2.916255921	3.465234734	3.256715265
tr|Q6MGC4|Q6MGC4_RAT	Transaldolase (EC 2.2.1.2	Taldo1	2	17.3	14.497	127	0	2.7168	0.451731781	0.484422106	0.397688948	0.355012458	0.449832869	0.599399856
tr|B2RZ72|B2RZ72_RAT A	Rho-associated protein kin	Rock1	2	16.1	19.667	168	0	19.019	3.270171288	3.500680983	3.325015907	2.992820172	3.072983508	3.785129507
tr|Q5XI77|Q5XI77_RAT A	Serine/threonine-protein ki	Cdc42bpb	15	31.4	54.16	503	0	207.5	112.5581634	109.7642941	97.91191314	91.80765488	116.6090412	121.0040384
tr|A0A0G2K791|A0A0G2K	Very-long-chain (3R)-3-hyd	Hacd1	2	20.3	7.579	64	0	7.7241	3.746582854	3.582012529	4.566674869	4.408086909	3.439721396	3.676674986
tr|D4A8G5|D4A8G5_RAT	Major prion protein (PrP) (	Prnp	3	5	74.752	685	0	5.4985	1.735598955	1.688176555	1.824167574	1.467264454	1.907023933	2.037795263
sp|O35112|CD166_RAT C	Ubiquitin-like protein 3 (Me	Ubl3	5	9.1	65.021	583	0	7.7599	11.82995029	9.177622924	10.77905305	10.15431206	10.52588765	11.94026276
sp|P62074|TIM10_RAT Mi	Sorting nexin-5	Snx5	5	24.4	10.333	90	0	19.967	12.60064538	16.07891379	13.57511533	14.35883528	14.47849257	14.38241425
tr|F1LPC8|F1LPC8_RAT N	AP-3 complex subunit mu-	Ap3m1	3	7.3	65.018	575	0	9.1928	1.737193772	1.906630235	2.113929879	2.046357557	1.852006708	1.735580299
tr|F1LXN6|F1LXN6_RAT S	KN motif and ankyrin repea	Kank2	2	3.3	72.504	659	0	4.3262	5.133315695	4.869904702	5.656516182	4.680228549	5.5385533	5.146072064
tr|D4A5L9|D4A5L9_RAT U	TAP-binding protein (Tap-b	Tapbp	12	64.8	11.635	105	0	232.84	382.524718	340.3463626	215.2878959	402.57865	278.6776183	311.3050237
sp|Q9ER24|ATX10_RAT A	60S ribosomal protein L26	Rpl26	3	8.8	53.726	475	0	5.9768	2.733475656	3.103773524	3.376110399	3.348210243	2.967920888	3.08723262
sp|Q8VHK0|ACOT8_RAT	Peroxisomal biogenesis fa	Pex3	2	7.8	36.003	320	0	9.8418	3.982097385	5.63756035	5.139603264	5.167218851	4.974904773	5.068927177
tr|G3V8Z7|G3V8Z7_RAT S	Golgi membrane protein 1	Golm1	2	21.6	11.034	97	0	2.5592	2.051930813	2.494262549	2.649636775	2.43768595	2.739347541	2.220161052
sp|Q498E0|TXD12_RAT T	Platelet factor 4 (PF-4) (C-	Pf4	4	31.2	19.018	170	0	7.8286	3.302785286	3.025771339	3.571588709	3.239179972	3.201662321	3.640745897
sp|P22734|COMT_RAT Ca	Cytoplasmic dynein 1 light	Dync1li2	3	15.2	29.597	264	0	2.3342	5.776824163	5.281318665	4.476369544	4.510953845	5.548039028	5.06379445
tr|G3V885|G3V885_RAT M	Perilipin-5 (Lipid storage dr	Plin5	3	2.8	223.38	1938	0	323.31	3587.340424	1564.229181	3108.597232	2337.466644	3481.131507	3042.988514
sp|Q6P4Z9|CSN8_RAT CO	Prolargin (Proline-arginine-	Prelp	2	12.9	23.235	209	0	3.9512	3.17874843	2.497306536	2.839775292	2.639296463	3.067030395	3.009112516
sp|P21775|THIKA_RAT 3-	Acylamino-acid-releasing e	Apeh	10	33.5	43.833	424	0	46.636	12.23702721	15.87249338	12.86523694	14.12054858	12.64741988	13.17160396
tr|F1MA89|F1MA89_RAT C	Serine/threonine-protein ki	Tnni3k	2	8.5	39.367	341	0	3.6796	1.288492142	1.536072295	1.657063459	1.276765306	1.675310483	1.397846864
sp|Q5BJT2|UBL3_RAT Ub	Sialidase-3 (EC 3.2.1.18) (	Neu3	2	14.5	13.18	117	0	3.2274	1.516391424	2.173835281	1.339293415	1.636495754	1.517978229	2.136394948
tr|G3V6W2|G3V6W2_RAT	Acyl-coenzyme A thioester	Them4	4	13.7	45.371	417	0	11.697	9.46962183	9.101523232	9.56451186	9.650394283	7.906518217	10.00471142
sp|P57093|PAHX_RAT Ph	Ubiquinone biosynthesis p	Coq4	10	30.2	38.588	338	0	33.542	36.48940213	51.01533141	43.34655621	52.78332503	47.58237651	35.85004482
sp|P27605|HPRT_RAT Hy	GTPase KRas (K-Ras 2) (K	Kras	5	26.1	24.477	218	0	8.8055	10.1613735	9.135768093	8.508593939	8.542730065	9.933193166	9.867154334
sp|Q63750|RM23_RAT 39	Casein kinase I isoform alp	Csnk1a1	2	19.2	17.05	146	0	3.7725	0.775240314	1.248463019	1.443209978	1.299639093	1.108979785	1.233702255
tr|A0A0G2K0I3|A0A0G2K	Tescalcin	Tesc	5	13.4	54.091	478	0	8.5319	8.541039908	8.256341021	9.633091556	8.233695221	9.137046164	8.655317495
sp|P61621|S61A1_RAT P	Echinoderm microtubule-as	Eml1	3	6.7	52.264	476	0	5.9529	8.980013157	9.855385811	10.54399745	8.09129893	9.330031674	11.13750426
tr|D4AD15|D4AD15_RAT E	Cell cycle control protein 5	Tmem30a	5	4.1	175.09	1592	0	11.628	5.025665579	4.555327597	5.698920422	4.481005243	5.076565615	5.381150959
tr|A0A0G2K0Z7|A0A0G2K	Phospholipase A-2-activat	Plaa	18	35	69.061	614	0	149.16	50.65137288	49.85956733	47.61891425	52.48383901	47.39854964	49.64938129
sp|A4F267|TM40L_RAT M	Magnesium transporter MR	Mrs2	5	16.9	34.048	308	0	7.7545	6.339794397	5.157181041	5.02642059	5.119040666	5.462340378	5.578247675
sp|Q6P0K8|PLAK_RAT Ju	Cytochrome c oxidase ass	Coa6	16	29.5	81.8	745	0	218.57	41.66458175	33.7387987	48.92506953	33.17263354	45.59887798	51.0064743
tr|A0A0G2K7E5|A0A0G2K	Myosin light chain kinase	Mylk	19	23.7	122.73	1084	0	208.65	48.64190406	43.41392337	43.05862619	40.71968112	47.36387766	44.93086538
tr|D3ZN27|D3ZN27_RAT D	Coronin-6	Coro6	2	1.6	254.51	2243	0	5.3393	2.81206024	2.50810318	3.019757732	3.076242218	2.347161734	3.154625325
sp|P13383|NUCL_RAT Nu	Hippocalcin-like protein 1 (	Hpcal1	3	4.3	77.146	713	0	17.109	26.85033104	21.59281214	23.10507558	21.29128547	23.55731599	25.19039744
sp|P97536|CAND1_RAT C	Vacuolar protein sorting-as	Vps26a	10	10.9	136.36	1230	0	19.949	13.75050809	12.81328574	12.44904718	12.22336978	13.41739936	13.84809737
sp|B2RZ78|VPS29_RAT V	Sorting nexin	Snx6	3	19.8	20.468	182	0	6.762	1.912025534	1.964656251	2.416361105	1.714709348	2.003778363	2.382714516
tr|F1M9D6|F1M9D6_RAT S	Receptor-type tyrosine-pro	Ptprc	7	12.8	87.249	749	0	23.998	7.854870097	6.773823885	6.453820592	6.352575817	6.971683323	8.30218588
tr|M0R8A4|M0R8A4_RAT	Alanine--tRNA ligase, mito	Aars2	2	15.8	28.137	247	0	26.87	3.64004911	3.254308228	3.534524262	3.19634045	3.849570284	3.552103702
sp|O54922|EXOC7_RAT E	Asparaginyl-tRNA syntheta	Nars2	2	3.4	75.045	653	0	2.5415	1.300333655	1.491363725	1.281236252	1.134965189	1.581434481	1.241709309
tr|Q2IBC6|Q2IBC6_RAT C	Phospholipid scramblase 3	Plscr3	3	24.2	20.551	178	0	228.14	45.5001155	39.84960402	54.92657823	38.10330185	53.22605785	44.34111505
sp|P11517|HBB2_RAT He	Alpha-1,6-mannosyl-glycop	Mgat2	2	17.7	15.982	147	0	45.174	11.05128112	11.18380107	10.32621765	9.008886558	10.26486657	12.47303982
sp|P04157|PTPRC_RAT R	Tryptophan--tRNA ligase, c	Wars1	3	2.4	143.27	1273	0	2.8733	3.292299367	2.599232562	3.462803511	2.632178244	3.307771642	3.718404056
tr|F1LUA1|F1LUA1_RAT E	Aminoacylase-1A (ACY-1A	Acy1a	5	5.1	161.1	1411	0	14.362	2.196500932	2.058924745	2.37364276	1.687842415	2.224697708	3.023176187
tr|G3V6S0|G3V6S0_RAT	UV excision repair protein	Rad23b	91	49.2	273.31	2356	0	323.31	458.4300105	282.7484078	451.0816444	354.365742	473.8546587	413.4052286
tr|G3V9D1|G3V9D1_RAT	Phosphoenolpyruvate carb	Pck2	7	12	85.909	756	0	20.675	7.698578078	8.456578337	7.983514278	8.142113266	8.210715716	7.570772286
tr|A1L1M0|A1L1M0_RAT F	Threonine synthase-like 1	Thnsl1	2	4.8	40.605	351	0	11.243	10.53894631	8.490823199	10.27543726	8.104786023	11.07736828	9.34874891
tr|D3ZUC9|D3ZUC9_RAT C	AP-2 complex subunit sign	Ap2s1	2	5.3	58.204	527	0	7.7489	0.94373268	0.88432359	1.255217848	0.846113098	1.166548344	0.964952671
tr|Q8R490|Q8R490_RAT C	Golgi brefeldin A-resistant	Gbf1	13	21.8	78.085	714	0	323.31	41.59281501	31.66698457	37.38274017	36.37756794	41.81832455	34.95541051
tr|F1LPQ9|F1LPQ9_RAT A	DnaJ homolog subfamily A	Dnaja2	5	8.5	95.941	870	0	8.5066	9.659404997	8.803307561	10.89841313	9.272347444	9.924034532	9.705473434
tr|B0BMY7|B0BMY7_RAT	H2-K region expressed ge	Pfdn6	5	24.1	39.472	349	0	10.692	6.356141266	4.865148471	6.405134242	5.793101218	5.75751008	5.709645485
tr|F1M9C0|F1M9C0_RAT I	Protein Mpv17	Mpv17	2	5.1	42.504	370	0.0030688	1.5722	1.73603753	1.517094944	1.646697978	1.510755032	1.782335668	1.534377401
sp|Q9JJ31|CUL5_RAT Cu	Casein kinase II subunit al	Csnk2a1	4	6.2	90.889	780	0	9.6395	1.344629684	1.374312886	1.850709487	1.324249031	1.615452266	1.504556258
sp|P08010|GSTM2_RAT C	Guanine nucleotide-bindin	Gna12	5	25.2	25.702	218	0	58.432	39.22969562	35.22226708	30.6493657	33.39225662	37.94225965	35.65140829
tr|G3V9N7|G3V9N7_RAT	Eukaryotic translation initia	Eif3b	8	25.7	48.598	424	0	114.21	15.77592508	13.53337908	16.49420225	14.66787013	14.91156514	15.586552
tr|B2RYT4|B2RYT4_RAT M	Drebrin (Developmentally-r	Dbn1	2	21.9	14.924	128	0	21.443	2.880358258	3.61335609	4.182052709	3.568223954	3.544391503	3.302653171

tr|Q5M860|Q5M860_RAT	Estradiol 17-beta-dehydrog	Hsd17b11	2	16.5	22.884	200	0	19.759	0.711766616	0.696597561	0.521121931	0.561297557	0.558918746	0.753176356
sp|Q5U2Z3|NP1L4_RAT N	Translation factor GUF1, m	Guf1	4	16.6	43.916	386	0	22.368	4.989383503	4.236469886	4.798694117	4.510519807	4.951942768	4.38175769
tr|A0A0G2K9I3|A0A0G2K	Peroxiredoxin-4 (EC 1.11.1	Prdx4	2	1.2	254.96	2213	0	3.7793	0.486498781	0.483946482	0.592874093	0.444237589	0.560671971	0.594985711
sp|Q9QVC8|FKBP4_RAT	cGMP-dependent 3',5'-cyc	Pde2a	2	5	51.45	458	0	3.7575	5.063941176	3.859300784	4.690589481	4.096144012	4.954428683	4.844524354
tr|M0R6L8|M0R6L8_RAT	Cystatin-C (Cystatin-3)	Cst3	2	12.4	19.364	178	0	10.96	8.275104252	9.709369526	9.759257257	8.176836282	10.90924192	7.977797535
tr|B0BN63|B0BN63_RAT	RCG60109 (TRIO and F-a	Triobp	3	10.4	38.104	338	0	6.422	2.791487107	2.513430159	2.195283198	2.224920668	2.718348101	2.704536497
tr|M0RBX6|M0RBX6_RAT	Osteoglycin (Osteoglycin (	Ogn	3	19.9	15.37	136	0	2.3461	10.51103702	9.412105102	12.01610513	6.752758579	11.53006649	12.431978
sp|P06765|PLF4_RAT Pla	Serine/threonine-protein p	Ppp2r5a	2	22.9	11.286	105	0	3.1474	3.05040557	3.666055127	6.737562542	4.433695133	3.607455242	4.651995765
tr|G3V743|G3V743_RAT	Vacuolar protein sorting-as	Vps45	10	18.1	81.025	730	0	33.288	16.84245863	17.75310703	17.11560759	15.82197638	16.52021385	20.28248391
sp|P23514|COPB_RAT Co	Synaptophysin-like 1 (Syna	Sypl1	5	8.5	107.01	953	0	48.059	7.058259242	7.39260951	6.479472539	6.824808838	7.077007618	7.207888489
tr|G3V985|G3V985_RAT	Histidine triad nucleotide-b	Hint1	7	20.4	31.768	284	0	18.432	20.5934671	26.34951855	27.52506319	22.29738486	27.68131821	22.8375554
tr|D3ZRC4|D3ZRC4_RAT	C-type lectin domain family	Clec10a	7	11.6	87.96	776	0	48.518	8.278293885	10.74527659	9.722088109	10.15300994	9.986182408	9.088006379
sp|P31211|CBG_RAT Cor	Apolipoprotein A-IV (Apo-A	Apoa4	2	5.3	44.67	396	0	2.4521	2.257263441	1.758568771	1.55969076	2.234209075	2.38719805	1.20542093
tr|G3V7N9|G3V7N9_RAT	DEP domain-containing M	Deptor	2	10.3	26.647	253	0	7.2948	1.750470619	1.563753558	2.004935278	1.441048577	1.491418189	2.220366361
sp|Q62760|TOM20_RAT M	Natriuretic peptides A (Atria	Nppa	2	17.2	16.284	145	0	13.664	1.734602195	1.733931495	1.499225455	1.64404801	1.575743044	1.807233167
tr|A0A0G2JY31|A0A0G2J	Similar to RIKEN cDNA 18	Smim8	6	17.5	46.121	411	0	39.308	19.18604152	15.97332547	14.58391743	13.01505455	16.35470424	22.09998253
tr|B2RYP4|B2RYP4_RAT	Muscular LMNA-interacting	Mlip	5	11.9	58.53	519	0	7.5467	5.992523098	6.399508522	6.347548238	5.496653467	6.529844633	6.501625257
sp|Q5XIF6|TBA4A_RAT T	Exocyst complex compone	Exoc7	4	12.1	49.924	448	0	284.44	77.35258858	71.9475031	66.18725968	61.68977869	74.57090945	76.09781011
tr|D3ZVM5|D3ZVM5_RAT	Proteasome 26S subunit,	Psmd8	18	27.6	76.194	685	0	103.33	40.24918209	23.54857424	39.46002441	30.14261634	41.83337088	34.84505688
tr|Q5BJU0|Q5BJU0_RAT	RCG25591, isoform CRA_	Stt3b	9	35.3	23.399	204	0	142.4	39.86921705	34.313827	43.84336638	33.36057187	41.98841347	40.60243672
sp|P80432|COX7C_RAT C	Dolichyl-diphosphooligosac	Dad1	2	28.6	7.3746	63	0	6.6364	19.26458623	24.79375546	21.49790255	27.49628847	18.3087643	21.46147129
tr|M0R5J4|M0R5J4_RAT U	Tight junction protein 2	Tjp2	11	37.3	47.074	434	0	175.67	60.66682067	50.85837579	50.6772546	55.85631197	53.66501811	50.68157268
sp|P16409|MYL3_RAT My	Vacuolar protein sorting 13	Vps13a	13	64.5	22.156	200	0	323.31	456.9149348	167.9567777	392.9773658	271.0044608	423.8943082	375.9927817
tr|F1LSS1|F1LSS1_RAT S	Clathrin light chain B (Lcb)	Cltb	2	1.9	142.95	1232	0.0017921	1.7124	10.23792469	3.781964472	9.478132853	6.404226304	10.15888808	8.167708433
sp|Q5HZY0|UBXN4_RAT	Peptidylprolyl isomerase (E	Fkbp11	2	6.3	56.393	506	0	20.528	2.453904319	1.926606404	2.631156902	2.483086294	2.223127657	2.427882513
sp|P62747|RHOB_RAT Rh	Cullin 4A (RGD1563853 pr	Cul4a	2	13.3	22.123	196	0	18.559	8.276699068	6.076084829	6.577368747	5.789194879	7.869229492	7.764276093
tr|G3V6D3|G3V6D3_RAT	Corticosteroid-binding glob	Serpina6	26	71.6	56.344	529	0	323.31	2690.654832	2998.99376	2742.664348	2920.943528	2787.103278	2780.449529
sp|P48500|TPIS_RAT Trio	Adenosine deaminase (EC	Ada	12	72.3	26.849	249	0	288.77	95.48963952	73.04619241	66.15584914	72.86624954	91.12841202	76.12860647
tr|A1L1J8|A1L1J8_RAT R	IQ motif-containing GTPas	Iqgap2	2	17.7	23.674	215	0	7.6245	1.973705063	2.168508302	2.121049603	2.108251333	2.256949185	1.825197712
tr|F1LQT3|F1LQT3_RAT R	Iron-sulfur cluster assembly	Iscu	14	11.7	159.44	1378	0	23.275	22.62845299	23.79684949	24.46305828	20.82643109	22.99340578	28.25617526
sp|P63255|CRIP1_RAT Cy	Carboxypeptidase (EC 3.4	Ctsa	2	23.4	8.5497	77	0.00047059	2.0986	1.170435849	2.641134955	2.137330737	2.407042888	1.901790428	1.877192236
sp|Q00715|H2B1_RAT His	Cullin-associated NEDD8-d	Cand2	5	36	13.99	125	0	35.481	29.49214462	23.61421023	25.13000891	20.76740196	25.23269189	30.57616785
tr|F1M1E4|F1M1E4_RAT	AP2-associated protein kin	Aak1	2	8.6	42.928	385	0	27.615	7.671864901	8.323879498	8.685016516	6.604751723	8.694160773	9.89487106
tr|D3ZZ00|D3ZZ00_RAT A	Single-stranded DNA-bindi	Ssbp1	5	5.4	143.44	1278	0	11.318	9.630299595	8.706280453	8.554139233	7.876916229	10.12094517	8.521353321
tr|B2RYW3|B2RYW3_RAT	Endoplasmic reticulum met	Ermp1	12	69.8	21.892	179	0	312.59	124.7306005	132.5228584	122.9827655	125.2025157	123.3733469	133.6716086
tr|D3ZZT3|D3ZZT3_RAT T	Transmembrane protein 33	Tmem33	2	9.6	25.804	229	0	4.1039	12.37019439	10.58213788	13.68086417	12.89873245	11.53791675	11.74059968
sp|P51638|CAV3_RAT Ca	Multifunctional procollagen	Plod3	2	21.9	17.402	151	0	41.72	4.573535094	4.247742153	7.168934067	4.917213134	6.657018813	4.910223259
sp|Q5XIP9|TMM43_RAT T	Poly [ADP-ribose] polymera	Parp3	5	13	44.774	400	0	4.6073	10.14343181	9.210440917	9.633091556	8.550976782	10.27075426	10.46819666
tr|A0A0G2JVM2|A0A0G2J	E3 ubiquitin-protein ligase	Ubr4	6	6.3	153.47	1377	0	9.0436	7.546671803	7.214726479	7.496127275	7.498001315	6.446173966	8.090204256
tr|A0A0G2K8Q8|A0A0G2K	Numb-like protein	Numbl	2	38.7	7.099	62	0	172.16	44.65884978	50.18774725	45.87301129	50.29194862	43.15156004	46.12679077
sp|P83868|TEBP_RAT Pro	Catechol O-methyltransfera	Comt	3	23.1	18.721	160	0	7.7447	11.33436105	10.39046177	8.582932235	8.909491924	11.35539825	9.575102169
tr|A0A0H2UHQ2|A0A0H2U	Rano class II histocompati	RT1-Ba	4	10.4	47.507	414	0	6.6775	6.649188803	5.484885342	6.036583813	5.442832792	6.221067817	6.719252881
tr|G3V7L6|G3V7L6_RAT 2	Glycine--tRNA ligase (EC 6	Gars1	6	17.1	48.633	433	0	16.028	10.12230049	8.352416883	8.601778564	8.422501622	9.057235207	9.884605606
tr|Q4KMA8|Q4KMA8_RAT	Similar to RIKEN cDNA 22	Tmem70	9	10.9	106.43	930	0	20.061	18.97432962	17.75786326	18.2223059	17.30638532	18.2976431	19.67476903
sp|Q5XIB3|ARHL1_RAT [	Lipopolysaccharide-binding	Lbp	6	20.4	39.877	353	0	8.2824	5.375329103	4.814256801	4.370620699	4.378572345	4.686734884	5.692194214
tr|B2RYW4|B2RYW4_RAT	Peroxisomal acyl-coenzym	Acox3	3	33.6	12.735	119	0	5.18	10.14103959	11.14051937	10.28486043	12.49768161	9.286855255	10.19667541
sp|Q8R491|EHD3_RAT EH	Nucleobindin-2 (DNA-bindi	Nucb2	2	4.7	60.79	535	0	3.5299	3.411870736	3.805507814	3.20021133	3.540619156	3.389676001	3.411313001
tr|D3ZYT2|D3ZYT2_RAT M	Signal peptidase complex	Sec11a	4	11.1	48.136	432	0	7.1014	7.139196181	8.906042146	8.295002212	8.569206365	7.985020798	8.012186806
sp|Q6PEC4|SKP1_RAT S	Mitogen-activated protein	Mapk1	2	15.3	18.672	163	0	5.8607	4.848242241	4.71675407	5.644475472	4.487515809	5.724015646	4.811572247
sp|Q4KM93|TM177_RAT T	High affinity immunoglobul	Fcer1g	2	6.8	34.001	311	0.00046533	1.9899	3.792154737	5.40260255	4.598923032	4.202222827	4.496693221	5.311345872
sp|M0R7Z9|PLIN5_RAT P	40S ribosomal protein S23	Rps23	2	5.1	51.804	475	0	3.0509	1.37995487	1.648224217	2.279515818	2.006990337	1.604265648	1.808670331
sp|P62963|PROF1_RAT P	Eukaryotic translation initia	Eif4g2	5	42.1	14.957	140	0	26.074	18.58120735	18.53027514	16.68999467	15.89098837	15.72406684	23.17734192
tr|F1LNM0|F1LNM0_RAT	Tetratricopeptide repeat pr	Ttc19	11	17.1	100.61	911	0	20.906	15.13241661	14.25537491	15.42100853	12.96123388	14.49746403	17.94093386
sp|P08733|MLRV_RAT My	S-adenosylhomocysteine h	Ahcyl1	14	74.7	18.88	166	0	323.31	334.8277427	133.1506808	298.0965705	209.2278746	317.8831153	265.3260554
tr|D3ZL10|D3ZL10_RAT C	Cation-transporting ATPas	Atp13a1	4	2.2	246.79	2264	0	6.0724	3.336396044	3.436566992	4.837224389	3.383584315	3.505663563	4.980952237
sp|Q5RKL5|STEA3_RAT M	Proteasome subunit beta t	Psmb6	5	12.3	54.673	488	0	6.411	6.484922701	5.136729249	6.808759784	4.673283945	7.414568714	6.701801609
tr|O88321|O88321_RAT A	Fat storage-inducing trans	Fitm1	3	13.2	41.073	380	0	49.473	6.633639342	5.973350244	5.97795079	5.902044682	6.418501807	6.36509472
tr|F1M614|F1M614_RAT L	DnaJ heat shock protein fa	Dnajc15	23	10.2	343.86	3121	0	145.09	53.99650055	62.24954852	61.72696187	50.88658027	52.1060877	72.18153943
tr|D3ZZW1|D3ZZW1_RAT	Rho guanine nucleotide ex	Arhgef1	6	3.9	214.96	1864	0	9.6219	7.47012061	7.266569395	8.190300386	8.962444524	6.31213081	7.342365936
sp|Q2TA68|OPA1_RAT Dy	ATP-dependent (S)-NAD(P	Naxd	39	46.7	111.31	960	0	323.31	190.6961991	208.9792683	191.0703634	205.5385541	195.5695533	192.2668197
tr|A0A0G2JSG6|A0A0G2J	Endothelin-converting enz	Ece1	11	54.3	25.529	232	0	96.381	75.65410898	75.07234672	64.3654479	70.10142937	73.6223366	69.92313956
tr|B1WBY5|B1WBY5_RAT	Cytochrome c oxidase ass	Cox16	7	15.7	63.204	559	0	323.31	21.08108226	23.31123833	20.68646339	23.15287318	19.67601758	21.78996582
sp|Q5U2Q7|ERF1_RAT Eu	CCZ1 homolog B, vacuola	Ccz1b	6	17.2	49.03	437	0	8.783	8.555393256	7.961454712	8.439490733	6.698937904	7.714841084	11.03433645
sp|D4AAT7|NNRD_RAT AT	Myozenin 2 (Myozenin 2 (F	Myoz2	2	7.3	36.518	343	0	2.1739	0.554637318	0.732459541	0.502474536	0.558389505	0.616179837	0.641642199
tr|M0RA19|M0RA19_RAT	Exocyst complex compone	Exoc5	8	16.2	69.764	650	0	60.163	22.25725944	18.12409303	21.04349662	19.74177087	21.07728863	20.14492683
sp|Q6AYC2|IRGM_RAT In	Cytochrome c oxidase ass	Coa8	5	14.4	46.337	411	0	4.9212	6.568650569	6.224003606	12.08102026	7.548783726	7.496342235	9.130608013
sp|O70595|ABCB6_RAT A	L-xylulose reductase (XR)	Dcxr	5	9.7	93.304	836	0	191.13	13.64445279	11.84634402	12.97046228	11.96989177	13.63197308	13.1012856

sp|A2RRU1|GYS1_RAT G	TPD52-like 1 (Tumor prote	Tpd52l1	9	14.6	84.071	738	0	23.033	12.85980306	11.08820083	13.30917269	11.84011449	12.40798701	13.27631159
tr|G3V7I2|G3V7I2_RAT Ra	Cysteine-rich protein 1 (CR	Crip1	3	7.4	61.283	554	0	4.1873	2.503702465	3.15542619	3.339988269	2.508737922	2.831588073	3.81120376
sp|P07632|SODC_RAT Su	Engulfment and cell motilit	Elmo1	6	37.7	15.911	154	0	42.697	10.99705736	11.9262487	12.02081671	10.13738459	12.29023314	12.26105819
sp|P33124|ACSL6_RAT L	Serine (Or cysteine) peptid	Serpinb6a	4	8.3	78.179	697	0	9.3263	4.770096231	4.514328888	4.800474048	3.983294209	5.407323153	4.833745627
tr|A0A096MIY2|A0A096MI	Phosphatidate cytidylyltran	Tamm41	2	6.4	51.545	470	0	3.1288	0.360807304	0.420993012	0.234809551	0.333688186	0.331065007	0.333806899
tr|A0A0G2JX74|A0A0G2J	Alpha-1,3-mannosyl-glycop	Mgat1	2	1.4	288.26	2545	0	7.0287	3.114876028	2.300874205	3.122679627	2.586777901	3.021760574	2.836806871
tr|A0A0G2K7B6|A0A0G2K	Transmembrane protein 17	Tmem177	56	31.1	242.46	2134	0	323.31	232.6478478	222.7485564	217.4499886	207.3181087	238.4908391	223.9511433
tr|A0A0G2K478|A0A0G2K	Eukaryotic translation initia	Eif2a	5	26	32.145	277	0	79.374	10.54213594	9.446825587	8.683969498	10.68904651	7.610825165	10.04628651
sp|Q9Z1P2|ACTN1_RAT A	Ubiquinol-cytochrome-c red	Uqcc3	9	14.1	102.96	892	0	95.228	11.21195889	12.1840364	13.7913246	10.81535148	13.10927673	13.60121321
tr|O35370|O35370_RAT T	60S ribosomal protein L36	Rpl36	2	5.9	61.844	577	0	43.666	9.325689639	8.247304183	9.90845736	7.930736904	11.51763691	8.405866965
tr|A0A0G2K1Y8|A0A0G2K	Ras-related protein Rab-9A	Rab9a	4	2.4	287.7	2498	0	323.31	669.5438482	385.8682474	694.6966195	493.0668303	696.5141441	593.9591654
sp|Q75Q41|TOM22_RAT I	ATP-binding cassette subf	Abcc4	6	66.2	15.49	142	0	188.8	22.71895883	23.59994154	22.53811519	25.08477499	21.04981273	22.36329142
tr|Q5M9H2|Q5M9H2_RAT	Proteasome subunit alpha	Psma2	39	64.1	70.821	655	0	323.31	596.6208626	603.9937475	577.3782228	625.8389636	537.2716599	606.7396555
tr|M0R544|M0R544_RAT (	Rho guanine nucleotide ex	Arhgef15	5	6.2	106.2	953	0	8.0232	6.114526563	6.850399201	6.549622763	6.399451889	6.596179314	6.583235616
sp|O70257|STX7_RAT Sy	Fibroblast growth factor 1 (	Fgf1	4	21.8	29.85	261	0	21.832	10.42850527	8.798551331	12.05222726	8.975031617	10.00711643	11.94642203
tr|A0A0G2K9Y5|A0A0G2K	Dematin actin-binding prot	Dmtn	5	12.8	57.926	515	0	26.944	2.682919972	2.311052539	3.632943979	2.829014343	3.1508319	2.759815966
tr|Q5U362|Q5U362_RAT A	Endophilin-A1 (Endophilin-	Sh3gl2	5	22.3	35.893	319	0	45.58	17.7750276	18.87890686	15.3676106	14.49338697	19.29658844	17.78592551
sp|Q9JHE5|S38A2_RAT S	Mitochondrial basic amino	Slc25a29	2	5.8	55.553	504	0	13.247	2.279710484	2.152670054	2.600793373	2.270451223	2.253089475	2.551119288
tr|D3ZKT0|D3ZKT0_RAT F	Ceramide synthase 2 (Lon	Cers2	2	5	37.785	337	0.00046751	2.0387	3.786134304	4.369882159	3.727856185	4.097793355	3.739012483	3.990797876
tr|Q4QR73|Q4QR73_RAT	Serine--tRNA ligase, cytop	Sars1	4	8.5	61.875	555	0	7.8231	8.337700801	7.006879194	9.591210826	7.208932205	9.33853612	8.617848589
sp|Q9WVR7|PPM1F_RAT	Sodium/hydrogen exchang	Slc9a1	2	6.9	49.165	450	0.0034767	1.5115	2.193191687	2.05079159	2.054668645	2.312335861	1.879220936	2.074956206
sp|Q62703|RCN2_RAT Re	Complement factor B (Com	Cfb	3	14.7	37.432	320	0	92.296	5.581857843	4.525363343	5.008516577	4.538732258	5.067210724	5.417593321
sp|Q5BK81|PTGR2_RAT I	Microtubule-actin cross-link	Macf1	2	7.4	38.136	351	0	5.0915	2.970465392	2.998137638	3.032531355	3.235186825	2.756421852	3.043091168
tr|D3ZNG0|D3ZNG0_RAT	Similar to solute carrier fam	Slc25a35	2	2.6	159.4	1420	0	3.5199	2.163687581	2.816354498	0.183432365	1.96753631	1.513202655	1.870263055
sp|Q63041|A1M_RAT Alph	Interferon gamma-inducible	Ifi47	14	13.6	167.12	1500	0	83.023	20.99575957	16.27772424	15.18176486	19.25695076	15.95107014	17.70277533
tr|G3V640|G3V640_RAT M	Ubiquitin carboxyl-terminal	Usp14	13	30.2	51.06	453	0	59.565	38.22775214	40.19300388	39.42337877	38.59246234	40.57275028	38.85884937
sp|Q62658|FKB1A_RAT F	Serine/threonine-protein p	Pgam5	2	25	11.922	108	0	3.7091	3.200996121	3.117186095	3.077867246	3.125809324	2.85441924	3.381029912
tr|A0A140TAI8|A0A140TA	Tubulin beta chain	Tubb6	3	8.2	52.895	475	0	2.2317	0.595783584	0.40925939	0.664018984	0.453048554	0.550054497	0.646415635
sp|Q9ERM3|DGAT1_RAT	Protein cordon-bleu	Cobl	4	9.6	56.87	498	0	13.058	2.814412595	2.883655163	3.076296718	2.961395842	2.97884583	2.852564343
sp|Q99ML5|PCYOX_RAT	RCG21429, isoform CRA_	Tbl2	5	13.1	56.287	504	0	9.7673	22.71178215	22.46700736	20.63463599	23.15374125	21.09298915	21.38909984
tr|D3ZWA8|D3ZWA8_RAT	V-type proton ATPase sub	Atp6v1h	3	5.4	75.401	671	0	3.6416	0.686727996	0.838380801	0.701659291	0.650405498	0.73766258	0.853367187
sp|P11762|LEG1_RAT Ga	Calponin-3 (Calponin, acid	Cnn3	7	64.4	14.857	135	0	66.933	25.76665321	25.67270691	23.69454687	21.98053734	26.15444302	27.33536404
tr|G3V824|G3V824_RAT I	Volume-regulated anion ch	Lrrc8a	9	3.7	273.59	2481	0	19.729	11.83274122	11.87583265	11.66954208	12.40175928	11.43717177	11.47780406
tr|Q6IN22|Q6IN22_RAT Ca	Structural maintenance of	Smc1a	5	20.9	37.544	339	0	48.845	9.487563516	10.85276741	12.21451509	9.391273774	10.84774823	12.53155291
sp|P13852|PRIO_RAT Ma	Solute carrier family 4 mem	Slc4a11	2	8.3	27.804	254	0	3.2364	3.413066848	3.396234155	5.539773645	3.717055482	4.576504178	3.916527317
sp|Q64542|AT2B4_RAT P	Mitochondrial ribosomal pro	Mrps24	2	3	133.09	1203	0	7.3736	4.463094049	2.867674228	3.648335148	3.412838457	3.812935746	3.669694477
tr|A0A0G2K9E5|A0A0G2K	LETM1 domain-containing	Letmd1	4	5.8	101.58	902	0	5.423	3.017313127	3.238137044	3.212618496	2.946812175	2.932594726	3.629351243
tr|A0A0G2JTW5|A0A0G2J	39S ribosomal protein L2,	Mrpl2	5	8	105.62	952	0	51.308	20.17363165	17.67367798	22.55486749	17.50734478	17.61663322	24.8603631
tr|D3Z979|D3Z979_RAT a	DNA damage-binding prote	Ddb1	2	5.1	70.667	625	0	3.8099	1.922551323	1.603230273	1.787469584	1.337660796	1.935284862	2.006434301
sp|P85515|ACTZ_RAT Alp	Protein-glutamine gamma-	Tgm1	3	15.2	42.613	376	0	55.192	6.14801771	4.033141019	5.570137175	4.416767663	5.702427436	5.526407132
tr|D4A4W6|D4A4W6_RAT	Disabled homolog 2 (Adap	Dab2	4	32.4	12.576	111	0	7.3485	3.649897103	3.284652981	3.918361159	3.883291923	3.166663254	3.763161435
tr|F1LPV8|F1LPV8_RAT S	Pgm2 protein (Phosphoglu	Pgm2	23	54.8	46.638	431	0	323.31	213.0953972	246.5772726	425.0265949	322.1297618	288.6343623	283.3213962
sp|P62198|PRS8_RAT 26	Protein kinase C delta type	Prkcd	7	23.9	45.626	406	0	33.586	11.29409194	9.87060575	10.86019696	8.966350863	11.81332997	11.37720261
tr|F7F588|F7F588_RAT Ni	Alpha-mannosidase 2 (EC	Man2a1	5	23.3	27.577	245	0	12.216	22.67948712	19.66606305	20.84246911	21.84858988	20.06591372	21.39987857
tr|M0R629|M0R629_RAT A	Acyl-CoA dehydrogenase	Acad11	9	22.8	50.25	457	0	101.95	29.66159387	32.21442674	31.57388283	23.16155393	40.03304504	29.63328591
tr|D3Z9Z0|D3Z9Z0_RAT A	Lon protease homolog 2,	Lonp2	27	21	187.12	1707	0	323.31	120.6717925	69.85000532	96.23144883	75.18401087	109.7138979	99.76994691
sp|P04218|OX2G_RAT O	GMP synthase [glutamine-	Gmps	3	12.6	31.088	278	0	5.7632	4.787240508	4.159418947	5.419890054	4.206085762	5.429761807	4.788167012
sp|Q5U1Z2|TPPC3_RAT T	Tensin 2	Tns2	2	15	20.302	180	0	15.799	4.651681104	4.472141121	4.404177634	4.209644871	5.298466242	4.066402945
tr|D3ZN37|D3ZN37_RAT R	MAP kinase-activated prot	Mapkapk3	3	2.7	158.03	1354	0	3.2567	6.460203045	6.336250653	7.423359506	6.70544847	6.942899043	6.532934892
tr|D4ABI7|D4ABI7_RAT Ve	alpha-1,2-Mannosidase (E	Man1a1	2	6.9	43.104	362	0	5.2776	1.484694445	1.75504916	1.66345027	1.315568277	2.057356374	1.505428821
tr|Q6AYL0|Q6AYL0_RAT	Protein phosphatase 1F (E	Ppm1f	4	15.4	35.638	318	0	15.149	14.60453234	18.75952547	14.0091044	16.91488331	14.39998999	15.89502889
sp|P31232|TAGL_RAT Tra	Sec61 translocon alpha 2	Sec61a2	5	29.4	22.603	201	0	9.0358	4.234636582	7.02637974	14.30855162	5.583895046	10.5559803	9.758340522
sp|P27274|CD59_RAT CD	Lipase maturation factor	Lmf1	3	23	13.79	126	0	4.1724	12.419235	10.45229277	10.66545157	9.253249785	13.91392818	10.51182484
sp|P04639|APOA1_RAT A	DEAD (Asp-Glu-Ala-Asp) b	Ddx5	8	29.3	30.062	259	0	38.371	19.37383116	17.80162059	15.85028602	17.13494043	19.11995763	16.66134503
sp|P68035|ACTC_RAT Ac	EPH receptor B4 (RCG216	Ephb4	2	5.8	42.019	377	0	323.31	754.4678283	421.0548428	671.6098667	487.8583779	768.3440051	601.6069286
sp|P15129|CP4B1_RAT C	Ras-related protein Rab-13	Rab13	3	7	58.936	511	0	11.212	14.66114833	13.60757628	14.20123225	14.65571707	14.24494739	13.53500103
sp|P48284|CAH4_RAT Ca	GTP-binding protein Rab-3	Rab3d	4	15.2	35.076	309	0	7.6799	17.9010181	16.55691499	19.22744344	18.78862408	16.4783458	18.35103875
sp|P02091|HBB1_RAT He	Metallo-beta-lactamase do	Mblac2	3	27.9	15.979	147	0	323.31	164.5093117	171.8283496	165.5702335	149.0789298	162.4545481	191.2094779
sp|Q4V897|CC90B_RAT C	DnaJ homolog subfamily B	Dnajb6	5	23	29.8	256	0	55.681	10.51103702	10.95692886	10.78899972	10.24068556	11.76295748	10.21977268
tr|F1M013|F1M013_RAT 6	Dystonin	Dst	6	18.1	29.864	265	0	36.839	43.61025792	36.21917306	36.59590594	31.33622002	42.070187	42.87674805
sp|Q5XIE0|AN32E_RAT A	Integrin subunit alpha 9	Itga9	2	5.4	29.418	258	0	25.545	3.930704422	3.379492223	3.085248724	3.190177115	3.290762749	3.924996317
tr|D3ZNQ6|D3ZNQ6_RAT	EF-hand domain-containin	Efhd2	3	19.7	20.9	183	0	19.334	5.863741664	5.856346967	5.408372853	4.692381604	6.562161529	5.884158202
tr|D3ZUY8|D3ZUY8_RAT A	Collagen type XXIV alpha	Col24a1	10	12.7	107.67	977	0	30.445	22.61130871	19.78306633	20.45245481	20.03431228	21.02299102	21.77559419
sp|P07335|KCRB_RAT Cr	Neural cell adhesion molec	L1cam	7	22.8	42.725	381	0	87.695	24.31058572	20.5450145	21.11469386	20.02736768	23.79478629	22.1626018

Supplementary Table 3b. Total MAM proteome in GA muscle													
Fasta headers	Protein names	Gene names	Unique pepti	Unique sequ	Mol. weight [	Sequence le	Q-value	Score	GA_4 mon_1	GA_4 mon_2	GA_4 mon_3	GA_24 mon_1	GA_24 mon_2	GA_24 mon_3
tr|D3ZVM5|D3ZVM5_RAT	Heat shock protein family A	Hspa12b	5	7.7	76.194	685	0	13.266	0.318009624	0.300419805	0.302481274	0.597429949	0.585565707	0.575169741
sp|O35795|ENTP2_RAT E	Ectonucleoside triphospha	Entpd2	2	8.1	54.389	495	0	9.6418	0.407833809	0.381949983	0.381218613	0.748056594	0.785050654	0.789542693
sp|D3ZVR7|PXL2B_RAT F	Prostamide/prostaglandin	Prxl2b	2	10	21.607	201	0.00054407	3.7968	0.756754833	0.783317555	0.758238511	0.925499799	0.90854904	0.929210227
tr|Q6GMN8|Q6GMN8_RAT	Alpha-actinin-1 (Alpha-acti	Actn1	4	6.1	102.61	887	0	11.038	1.524748352	1.401876824	1.43373764	2.638415018	2.601393721	2.612302055
sp|Q5RJQ4|SIR2_RAT NA	NAD-dependent protein de	Sirt2	3	13.7	39.319	350	0	24.218	1.081121339	1.028924874	1.037924148	1.299916309	1.355839699	1.364163802
tr|A0A0G2K9E5|A0A0G2K	Leucyl-tRNA synthetase (E	Lars2	5	9.3	101.58	902	0	11.391	0.81038945	0.783959214	0.83443741	1.053789265	1.023580505	1.02959976
sp|P62494|RB11A_RAT R	Ras-related protein Rab-11	Rab11a	10	45.4	24.393	216	0	43.489	36.00543467	33.72362232	35.29756233	47.9365416	46.66998595	44.80191495
tr|A0A096MJB5|A0A096M	Protein unc-45 homolog B	Unc45b	19	22.6	103.4	929	0	109.42	8.15356547	7.764073811	7.21877091	11.71507393	11.52094327	12.03028494
tr|F1LMC7|F1LMC7_RAT S	Septin-7 (CDC10 protein h	Septin7	7	21.6	50.579	436	0	82.609	6.039789621	5.077496967	5.408475322	9.10150454	9.207370899	8.453203001
sp|P47942|DPYL2_RAT D	Dihydropyrimidinase-relate	Dpysl2	9	22.9	62.277	572	0	94.756	8.012580184	6.75765636	7.523840934	13.02365037	11.84991277	11.77324059
tr|A0A0H2UHH3|A0A0H2U	Mitochondrial potassium ch	Abcb8	5	8.6	77.506	710	0	21.511	3.285107703	3.19862072	3.193859042	3.861166527	3.705275142	3.78708615
tr|D4A4K4|D4A4K4_RAT V	Vacuolar protein sorting 13	Vps13c	6	2.3	418.62	3739	0	11.756	2.69301967	2.496744499	2.598217823	3.398058277	3.395180942	3.304188217
tr|A0A0H2UHU1|A0A0H2U	Putative 60S ribosomal pro	Rpl37a-ps1	2	29.3	10.276	92	0	9.6783	1.496501122	1.40839213	1.413521605	1.741941607	1.786412236	1.84889051
sp|P35565|CALX_RAT Ca	Calnexin	Canx	16	25.4	67.254	591	0	134.22	37.08013391	40.45857382	39.22322353	48.85093253	52.17618035	52.05097187
sp|Q1JU68|EIF3A_RAT Eu	Eukaryotic translation initia	Eif3a	8	6.8	163.19	1354	0	14.541	2.042831652	1.817573134	1.969142394	2.787488811	2.656347806	2.641054446
tr|A0A0G2JV84|A0A0G2J	Glutamic--pyruvic transamin	Gpt2	6	16	60.288	544	0	36.239	3.281294577	3.157653261	3.345845203	4.093317902	4.038882571	4.302190826
sp|P47853|PGS1_RAT Big	Biglycan (Bone/cartilage p	Bgn	5	15.7	41.706	369	0	26.206	6.26907886	6.372167381	6.366221396	8.945622088	9.079558159	8.642887504
tr|D4ACN7|D4ACN7_RAT	Myoferlin	Myof	5	3.1	233.38	2048	0	9.3586	1.226170614	1.184798651	1.285593441	1.593405339	1.571179897	1.622935323
tr|F1LST1|F1LST1_RAT F	Fibronectin (FN) [Cleaved i	Fn1	11	7.1	262.75	2387	0	93.012	8.492231195	7.237419853	6.980935208	12.11523194	13.69861349	12.32898469
tr|A0A0G2JVE6|A0A0G2J	Aminopeptidase N (AP-N)	Anpep	12	16.4	109.43	965	0	105.93	7.520385997	7.619453746	7.985791046	11.72403269	11.35807641	12.41635945
tr|E2RUH2|E2RUH2_RAT	Ribonuclease inhibitor (Rib	Rnh1	5	12.9	49.919	456	0	27.387	3.670735074	3.275965308	3.675887976	5.873244658	5.198527018	5.592187683
tr|G3V928|G3V928_RAT L	Prolow-density lipoprotein	Lrp1	19	5.1	504.88	4545	0	109.87	7.692980085	7.546896921	8.246952941	11.22353654	11.05445374	12.15626715
sp|F1M775|DIAP1_RAT P	Protein diaphanous homol	Diaph1	4	3.1	140.3	1265	0	9.3366	1.447382048	1.403555009	1.365268401	1.822451011	1.799732804	1.931083741
sp|Q63083|NUCB1_RAT N	Nucleobindin-1 (Bone 63 k	Nucb1	11	31.4	53.506	459	0	36.946	10.13839748	9.592308356	9.970804399	13.42022488	12.95978036	14.01958465
tr|D4AC23|D4AC23_RAT T	T-complex protein 1 subun	Cct7	10	21.9	59.658	544	0	52.538	5.521003904	4.869599453	5.478453903	8.590257878	8.479323648	8.335551874
tr|F7F9U6|F7F9U6_RAT P	Plectin (PCN) (PLTN) (Plec	Plec	130	36.5	517.41	4544	0	323.31	113.7615799	103.3515204	109.3861275	137.5289357	143.8082073	138.4686711
sp|D3ZTX0|TMED7_RAT T	Transmembrane emp24 do	Tmed7	2	9.7	25.479	226	0	7.67	1.696890885	1.649014253	1.557000566	2.078850768	2.058917625	1.985032574
sp|Q5U2Q7|ERF1_RAT Eu	Eukaryotic peptide chain re	Etf1	3	8	49.03	437	0	10.173	0.79423384	0.83998098	0.885983725	1.469774429	1.512073246	1.336732193
tr|G3V8C4|G3V8C4_RAT	Chloride intracellular chann	Clic4	4	29.2	28.669	253	0	61.306	1.076254587	0.897187347	1.314957002	2.141860725	2.41177786	2.294095379
tr|D4AAI5|D4AAI5_RAT Cu	Cullin-associated NEDD8-d	Cand2	17	17.8	135.64	1235	0	118.3	9.628141122	8.708299698	9.911345474	12.80386209	13.71479232	12.48849442
sp|P11505|AT2B1_RAT P	Plasma membrane calcium	Atp2b1	7	6.9	134.69	1220	0	172.66	15.95441686	16.31097159	17.12828688	19.44051268	20.20951309	20.82567187
tr|Q5XI38|Q5XI38_RAT Ly	Lymphocyte cytosolic prote	Lcp1	4	7.5	70.121	627	0	15.308	0.408054569	0.447488046	0.395132001	0.822653216	0.851653495	0.945943509
sp|Q5PPN5|TPPP3_RAT T	Tubulin polymerization-pro	Tppp3	3	16.5	18.979	176	0	7.2783	2.450936393	2.132232833	2.670163123	4.766120899	4.024645203	4.363149959
sp|Q6PEC4|SKP1_RAT S	S-phase kinase-associated	Skp1	3	16	18.672	163	0.0015576	3.0507	1.216788319	1.204393929	1.167956244	1.635929594	1.565840884	1.523470337
tr|F1LS29|F1LS29_RAT C	Calpain-1 catalytic subunit	Capn1	5	7.9	82.099	713	0	9.5842	0.776924259	0.711353031	0.771959801	1.015923566	1.133812252	1.074709518
tr|Q6IMZ3|Q6IMZ3_RAT A	Annexin A6 (Annexin VI) (A	Anxa6	40	56.5	75.755	673	0	323.31	76.23240032	69.04744345	74.69413149	101.3056762	98.4427742	99.11751861
sp|Q793F9|VPS4A_RAT V	Vacuolar protein sorting-as	Vps4a	2	6.4	48.906	437	0.0010499	3.1791	2.806058763	2.468116636	2.697422753	3.602079141	3.856385393	4.067193367
sp|P62193|PRS4_RAT 26	26S proteasome regulator	Psmc1	6	13.4	49.184	440	0	19.119	3.156515073	2.774484126	3.131198482	4.154536107	3.925361129	4.212682498
sp|Q5XIM9|TCPB_RAT T-c	T-complex protein 1 subun	Cct2	10	28.2	57.458	535	0	50.673	7.300127844	5.65499006	6.435285225	11.22353654	10.79721038	10.16798342
tr|Q66WT9|Q66WT9_RAT	Phosphatidylinositol-bindin	Picalm	3	6.7	64.713	597	0	11.853	1.25135731	1.210958594	1.347979575	1.764816311	1.632767301	1.81515979
tr|C0KUC5|C0KUC5_RAT	LIM and senescent cell an	Lims1	2	7.2	41.609	362	0	4.5557	0.495641051	0.592695476	0.552830793	0.81459033	0.829488501	0.74268544
sp|Q9JHW0|PSB7_RAT P	Proteasome subunit beta t	Psmb7	2	8.3	29.927	277	0.0015275	2.8481	0.701012964	0.55315941	0.686796325	1.104973656	0.960267359	1.049716274
tr|A0A0G2JSH9|A0A0G2J	Peroxiredoxin-2 (EC 1.11.1	Prdx2	3	19.7	21.797	198	0	13.373	2.210207779	1.88978445	2.105532021	2.800688053	2.967197018	2.753016054
sp|Q6P502|TCPG_RAT T-	T-complex protein 1 subun	Cct3	7	16.3	60.646	545	0	43.737	5.595761227	5.015305403	6.357988622	10.36289817	8.82015762	10.45449134
tr|A0A0G2JXB1|A0A0G2J	Galectin-3 (Gal-3) (35 kDa	Lgals3	4	17.1	26.335	252	0	12.726	4.769567376	4.36022093	4.415236843	5.460723549	5.889632612	5.509689656
tr|M0RBJ0|M0RBJ0_RAT G	Guanine nucleotide-bindin	Gng2	2	68.2	4.8827	44	0	57.802	0.839339098	0.813574244	0.958615089	1.192470895	1.314853335	1.186772725
tr|Q68FS8|Q68FS8_RAT F	RNA 3'-terminal phosphate	Rtca	4	16.1	39.313	366	0	11.384	2.511444669	2.250890388	2.541457419	3.350815741	3.822679502	3.495904691
tr|F1LQ55|F1LQ55_RAT N	Non-specific lipid-transfer p	Scp2	16	33.5	58.787	547	0	122.51	29.72581955	24.81245966	28.83895091	39.56965551	37.64759299	42.43670058
sp|P07150|ANXA1_RAT A	Annexin A1 (Annexin I) (An	Anxa1	10	33.2	38.829	346	0	208.02	7.890660523	7.528634319	7.806956894	10.32288237	11.00537796	11.33738279
sp|P13286|PHKG1_RAT F	Phosphorylase b kinase ga	Phkg1	7	26.5	45.014	388	0	88.951	6.847570091	5.843045504	6.449006515	9.536303103	9.323858459	8.39915257
tr|Q4V8H5|Q4V8H5_RAT	Aspartyl aminopeptidase (I	Dnpep	2	5.7	52.555	475	0	8.9571	1.637486409	1.603801973	1.753489445	2.46246494	2.284612273	2.590559964
tr|G3V7L6|G3V7L6_RAT 2	26S proteasome regulator	Psmc2	4	12.9	48.633	433	0	16.17	2.328816041	2.033219914	2.763788062	4.756863512	4.212049958	5.26534513
tr|A0A0H2UHQ8|A0A0H2U	40S ribosomal protein S17	Rps17	4	32.8	15.379	134	0	111.85	9.623625579	8.021724576	9.15027123	13.20103385	12.59252097	12.04654071
sp|P68101|IF2A_RAT Euk	Eukaryotic translation initia	Eif2s1	4	13.3	36.108	315	0	54.459	2.093957633	1.57181774	1.908311339	3.646275698	3.320057252	2.982831986
sp|P04797|G3P_RAT Glyc	Glyceraldehyde-3-phospha	Gapdh	24	75.7	35.828	333	0	323.31	162.9508949	167.2360768	204.3420313	301.6892833	279.2627466	279.7414623
tr|Q5XI34|Q5XI34_RAT Pr	Protein phosphatase 2 (Fo	Ppp2r1a	6	14.9	65.322	589	0	71.176	2.495690441	2.171966332	2.372868496	3.433893323	3.490905674	3.935420041
tr|G3V7C6|G3V7C6_RAT	Tubulin beta-4B chain (Tub	Tubb4b	3	8	61.186	548	0	323.31	62.30947608	52.84308596	62.28093739	85.57409085	89.37184475	84.14798747
tr|Q4KLM9|Q4KLM9_RAT	Asparagine--tRNA ligase (E	Nars	2	7.2	46.338	404	0	17.084	0.529673193	0.444378467	0.555346362	0.838599811	0.80705386	0.852249322
sp|B2GUZ5|CAZA1_RAT F	F-actin-capping protein sub	Capza1	3	15.4	32.909	286	0	27.106	0.948615238	0.76115564	0.882873566	1.400373889	1.329576068	1.320273227
sp|Q5RJR8|LRC59_RAT L	Leucine-rich repeat-contain	Lrrc59	7	28.7	34.869	307	0	62.445	8.676866731	7.391911595	7.440141062	11.70850417	10.96762736	10.38235637
tr|D3ZWH1|D3ZWH1_RAT	Stabilin 1	Stab1	3	1.9	242.16	2252	0	21.462	0.665691383	0.746594917	0.800957462	0.982955323	1.047093735	1.124696008
sp|P52944|PDLI1_RAT PD	PDZ and LIM domain prote	Pdlim1	3	11.6	35.584	327	0	5.1352	2.986630311	3.530457136	2.921491427	4.950970013	5.888014729	5.08957963
sp|P62268|RS23_RAT 40	40S ribosomal protein S23	Rps23	4	28.7	15.807	143	0	14.677	4.945372517	4.355581242	5.861735283	8.670886732	7.65420343	8.406061271
tr|D4A644|D4A644_RAT M	MAP7 domain-containing 1	Map7d1	2	2.8	90.418	817	0.0015106	2.7681	0.362693431	0.301150309	0.308116151	0.510147721	0.496792479	0.482257863
tr|A0A140TAB9|A0A140TA	Protein-L-isoaspartate(D-a	Pcmt1	7	40.3	21.192	196	0	132.3	5.27415422	5.489639474	5.680156873	6.699959179	6.393333451	6.97433443

tr|R9PXS4|R9PXS4_RAT	Phosphatidylinositol transf	Pitpna	2	12.9	31.851	271	0	23.756	0.749279101	0.793287948	0.789888954	1.013056762	0.964365995	1.064651261
sp|Q5XI32|CAPZB_RAT F	F-actin-capping protein sul	Capzb	11	46.3	30.628	272	0	47.504	11.16242229	9.887471493	12.03174223	16.69972888	14.63321045	15.61671394
tr|F1M779|F1M779_RAT C	Clathrin heavy chain 1	Cltc	23	20.2	191.56	1675	0	323.31	17.19869982	15.42252068	17.8646628	22.8860524	26.20970103	23.43573876
sp|Q3MIE4|VAT1_RAT Sy	Synaptic vesicle membrane	Vat1	13	47	43.118	404	0	309.02	16.45514041	17.28876118	18.52465687	21.64197903	22.989575	24.36434955
sp|P97675|ENPP3_RAT E	Ectonucleotide pyrophosp	Enpp3	5	6.4	99.071	875	0	13.061	1.025831023	0.928924788	0.898195673	1.353131353	1.562335471	1.387328273
tr|D4A9D8|D4A9D8_RAT C	Oxysterol-binding protein	Osbp	10	17.7	77.806	682	0	161.76	11.47600166	11.46496545	11.34064656	14.30953129	13.68567043	13.63351014
tr|A0A0H2UHD9|A0A0H2U	Nicalin (Nicastrin-like protei	Ncln	4	8.5	62.863	562	0	10.197	3.385854484	3.251286116	3.404435113	3.854298143	3.804613145	3.823458433
tr|A0A0H2UHR7|A0A0H2U	Filamin-C (FLN-C) (ABP-28	Flnc	65	30.8	287.3	2693	0	323.31	102.4777397	94.93098004	102.2464827	130.8576445	132.9737857	146.0174437
tr|G3V7Z3|G3V7Z3_RAT N	Nucleolar protein 3 (Apopt	Nol3	2	12.2	24.491	221	0	50.634	0.082965577	0.112517372	0.056710093	0.175185597	0.213555134	0.20116514
tr|F1LNM0|F1LNM0_RAT D	Disks large homolog 1 (Syn	Dlg1	4	5.4	100.61	911	0	8.7613	1.017602701	1.006071942	1.07172426	1.211164844	1.32563922	1.284002542
tr|Q7TQ70|Q7TQ70_RAT	Fibrinogen alpha chain [Cl	Fga	11	16.8	86.656	782	0	127.37	19.16597138	22.87316875	18.11256078	28.25414235	31.19655505	30.89307272
tr|Q6P725|Q6P725_RAT D	Desmin	Des	28	55.4	53.422	469	0	323.31	76.4531602	73.154061	76.41844032	89.13370546	90.8225463	96.38858142
tr|F1M7V6|F1M7V6_RAT C	Cell adhesion molecule 4 (	Cadm4	2	6.5	42.057	382	0	4.5274	1.275941933	1.324877745	1.604613444	2.014825485	2.041013056	2.247055248
sp|P20059|HEMO_RAT He	Hemopexin	Hpx	5	12.8	51.35	460	0	14.811	3.12821767	2.899410196	3.565019949	5.018459351	4.58955594	5.531736542
sp|B2GV71|NDUF5_RAT A	Arginine-hydroxylase NDUI	Ndufaf5	3	9.6	38.229	343	0	25.116	5.020782085	5.71915596	5.444150677	6.606788058	7.272383094	6.719626851
sp|Q63355|MYO1C_RAT U	Unconventional myosin-Ic	Myo1c	14	16.6	119.81	1044	0	66.583	9.566930428	9.637224485	8.236433285	11.75747873	12.76940949	12.34625644
sp|O88989|MDHC_RAT Ma	Malate dehydrogenase, cy	Mdh1	13	37.1	36.483	334	0	117.82	22.7397728	19.67770697	26.95410297	39.09304939	34.44364579	41.85352487
tr|Q6IMX4|Q6IMX4_RAT P	Phospholipid phosphatase	Plpp3	3	9.9	35.233	312	0	4.8824	3.316014086	3.005925589	3.106088521	4.505480661	4.018605107	4.532006758
tr|F1M9W9|F1M9W9_RAT	Similar to TRS85 homolog	Trappc8	4	3.6	160.35	1433	0	46.648	1.272530189	1.185193518	1.325522396	1.592748363	1.5173044	1.55842024
sp|P08082|CLCB_RAT Cla	Clathrin light chain B (Lcb)	Cltb	4	13.1	25.117	229	0	7.8765	1.629258086	1.287316014	1.509204737	2.051616133	2.297501406	2.069460974
sp|P29975|AQP1_RAT Aq	Aquaporin-1 (AQP-1) (Aqu	Aqp1	3	13	28.856	269	0	179.79	4.437725139	5.846500591	7.622176849	13.04156789	12.29914489	10.85783761
tr|G3V9T7|G3V9T7_RAT A	ATPase Get3 (EC 3.6.-.-) (	Get3	2	7.2	38.822	348	0	6.5802	2.018046338	1.924532752	2.402140583	2.860114505	3.210634447	3.356511473
sp|P29147|BDH_RAT D-be	D-beta-hydroxybutyrate de	Bdh1	8	35	38.201	343	0	178.24	8.974390842	9.843048947	9.967602765	7.073240913	7.642338957	7.521036256
tr|O70509|O70509_RAT C	CD44 antigen (Extracellula	Cd44	2	5.5	39.725	364	0.0010549	3.2479	1.265355493	1.092794623	1.256641518	1.576085067	1.763869736	1.563398569
tr|A0A0G2JSP8|A0A0G2J	Creatine kinase M-type (E	Ckm	28	61.4	43.018	381	0	323.31	419.8150496	346.3675242	446.847545	612.8987439	632.9696728	567.5701695
sp|P06765|PLF4_RAT Pla	Platelet factor 4 (PF-4) (C-	Pf4	3	23.8	11.286	105	0	8.2379	5.020280358	4.040674752	4.257259053	6.655762622	7.217914374	6.070818477
tr|A0A0G2JUA5|A0A0G2J	AHNAK nucleoprotein	Ahnak	112	54	581.12	5450	0	323.31	146.8053201	116.5598239	137.3912813	204.1164244	214.773939	208.1551202
sp|P50503|F10A1_RAT H	Hsc70-interacting protein (	St13	2	5.4	41.279	368	0	4.2303	3.176333289	2.394523285	2.938460089	4.578823056	4.512329002	4.146541839
sp|P62078|TIM8B_RAT M	Mitochondrial import inner	Timm8b	3	31.3	9.2864	83	0	4.7404	4.322177411	3.940625307	4.213305185	5.699146058	5.237464064	5.108172165
sp|Q6P752|TOIP2_RAT T	Torsin-1A-interacting prote	Tor1aip2	2	3.8	61.697	578	0.00055218	3.9446	0.284413984	0.291416835	0.238485176	0.143166983	0.187701367	0.16178554
tr|A0A0H2UHA6|A0A0H2U	Transmembrane protein 33	Tmem33	4	14.2	27.884	246	0	15.535	3.392928835	3.545412726	3.818772347	4.524294061	4.68253027	4.582196444
tr|M0RCS9|M0RCS9_RAT	60S ribosomal protein L21	Rpl21	2	16.7	16.622	144	0	14.189	10.00092428	9.813433917	9.217505553	12.44610887	11.9885114	13.4394569
sp|P85973|PNPH_RAT Pu	Purine nucleoside phosph	Pnp	3	13.8	32.302	289	0	10.047	1.802002692	1.525963801	1.468909881	2.243811432	2.131290915	2.457973849
tr|G3V9J8|G3V9J8_RAT G	Glycerol-3-phosphate acylt	Gpam	2	3.5	87.52	772	0	5.2962	0.800455255	0.70222173	0.802512541	1.398522412	1.215892838	1.17305692
tr|Q3MHS9|Q3MHS9_RAT	Chaperonin containing Tcp	Cct6a	9	20	58.017	531	0	57.946	7.811889384	6.469403397	8.519091869	12.11224569	11.49289997	10.65667247
tr|A0A0G2K2V2|A0A0G2K	AP-1 complex subunit beta	Ap1b1	2	2.2	103.82	942	0.0024913	2.6328	0.959603059	0.92640751	1.050227572	1.323507714	1.268096523	1.462612803
sp|Q01177|PLMN_RAT Pl	Plasminogen (EC 3.4.21.7	Plg	10	14.7	90.535	812	0	58.048	10.25680505	9.081449081	9.36066435	12.32904772	11.99714011	12.4763026
tr|G3V7S2|G3V7S2_RAT	Neurofilament medium poly	Nefm	2	3	95.609	844	0	18.28	0.370540441	0.400696298	0.449847934	0.66366506	0.580064906	0.709493191
tr|G3V6E7|G3V6E7_RAT	Fibromodulin (FM) (Collage	Fmod	4	17.3	43.133	376	0	36.3	2.928329633	2.433417692	2.73286942	3.972195446	3.457739077	4.011720556
sp|Q6PCT3|TPD54_RAT T	Tumor protein D54 (Tumor	Tpd52l2	5	33.6	23.992	220	0	56.673	5.79745548	5.584407571	5.690219153	6.517200442	6.989253608	6.637535219
sp|O08590|AOC3_RAT Me	Membrane primary amine c	Aoc3	13	23.6	84.98	763	0	59.004	14.1582342	14.52271734	16.1764867	18.04891838	18.78523695	20.06063475
sp|B1H267|SNX5_RAT So	Sorting nexin-5	Snx5	3	8.7	46.793	404	0	58.058	0.793581595	0.70434414	0.76464178	1.052773939	1.093796618	1.231984082
sp|P97829|CD47_RAT Le	Leukocyte surface antigen	Cd47	2	7.6	32.995	303	0	8.8749	4.618045822	5.178188071	5.394296655	6.723251959	6.303271309	7.243164207
sp|P62198|PRS8_RAT 26	26S proteasome regulator	Psmc5	6	19.2	45.626	406	0	9.382	1.266358947	1.069448107	1.261992821	1.896749007	1.709508874	1.597230888
tr|A0A0G2K613|A0A0G2K	MARCKS-related protein (I	Marcksl1	3	18.6	19.718	199	0	8.4939	1.920510609	1.580504816	1.591852644	2.341163308	2.519474923	2.48672624
tr|D3ZAN3|D3ZAN3_RAT A	Alpha glucosidase 2 alpha	Ganab	5	9.9	90.571	797	0	93.521	3.475262235	2.93287518	4.524778479	6.247243093	6.303810604	7.740387537
sp|P05943|S10AA_RAT P	Protein S100-A10 (Calpac	S100a10	3	38.9	11.075	95	0	57.462	9.151500473	7.73051011	11.04197981	18.7936901	15.2668812	20.05352285
tr|F1LRV4|F1LRV4_RAT H	Heat shock 70 kDa protein	Hspa4	7	9.9	94.025	840	0	38.792	1.487520208	1.150445215	1.434057803	2.111162035	2.342424618	1.907919271
sp|P13233|CN37_RAT 2,3	2',3'-cyclic-nucleotide 3'-ph	Cnp	14	30	47.268	420	0	102.08	10.86640336	10.76259564	9.85920457	12.49090268	14.1020056	13.31550666
sp|P07335|KCRB_RAT Cr	Creatine kinase B-type (E	Ckb	4	18.9	42.725	381	0	102.74	5.367977169	3.765353686	4.214677315	7.978673086	8.993271078	6.960618625
sp|P14668|ANXA5_RAT A	Annexin A5 (Anchorin CII)	Anxa5	9	33.9	35.744	319	0	323.31	15.95491859	12.11205386	14.60311206	22.16218446	26.59259995	21.65268411
sp|Q9Z0V5|PRDX4_RAT P	Peroxiredoxin-4 (EC 1.11.1	Prdx4	4	14.7	31.007	273	0	8.907	6.318749833	5.053311359	6.441231117	9.31770932	8.418383397	8.027301857
tr|D3ZVM6|D3ZVM6_RAT	Sialic acid binding Ig-like le	Siglec1	4	3.4	173.66	1609	0	39.385	1.908770197	1.817573134	1.877209747	2.558443139	2.332393745	2.335750787
sp|Q6AYC4|CAPG_RAT M	Macrophage-capping prote	Capg	2	7.4	38.798	349	0	8.6611	0.550595209	0.546347953	0.597333511	0.69083997	0.700165738	0.703173761
tr|A0A0G2JVV5|A0A0G2J	Ubiquitin carboxyl-terminal	Usp14	2	5.5	52.293	458	0	16.045	0.152625353	0.125478884	0.131852454	0.19869936	0.23550441	0.196684643
tr|D3ZIS5|D3ZIS5_RAT Cy	Cytochrome c oxidase ass	Cox19	2	20.7	10.864	92	0.0015182	2.8079	1.375584915	1.191165882	1.29894883	1.643693854	1.55117208	1.766392483
tr|B1WC61|B1WC61_RAT	Complex I assembly factor	Acad9	24	41	68.842	625	0	105.02	59.87610013	58.71673366	56.84730637	52.32812654	53.29198017	54.63563912
tr|D3ZNQ6|D3ZNQ6_RAT	Ubiquitin-conjugating enzy	Ube2m	2	14.2	20.9	183	0	4.7435	0.864124411	0.671520815	0.623403963	1.129281763	1.238866774	1.09360685
tr|F1LSW7|F1LSW7_RAT	60S ribosomal protein L14	Rpl14	7	31.8	23.322	214	0	32.758	12.49250056	10.9136323	12.88474912	18.01188883	16.36056996	15.3098863
sp|P69897|TBB5_RAT Tu	Tubulin beta-5 chain	Tubb5	2	6.1	49.67	444	0	86.456	9.679819003	7.194478059	7.797351991	14.97367415	16.31634783	12.50170223
sp|P54311|GBB1_RAT Gu	Guanine nucleotide-bindin	Gnb1	6	21.8	37.377	340	0	115.4	12.42025188	13.23989294	13.46607445	16.11382587	18.35002649	18.07743095
tr|A0A0G2K7Q7|A0A0G2K	Insulin-degrading enzyme	Ide	3	3.1	113.16	978	0	4.5872	0.831512156	0.714561326	0.852595251	1.060777099	1.016461821	1.184029564
sp|Q9ESS6|BCAM_RAT B	Basal cell adhesion molec	Bcam	2	4.5	67.511	624	0.001056	3.2864	0.636240008	0.536278843	0.595549743	0.75635838	0.768170744	0.798798322
sp|O35077|GPDA_RAT G	Glycerol-3-phosphate deh	Gpd1	17	54.7	37.452	349	0	111.55	35.08627081	32.08591113	41.30703017	55.83219682	48.30296897	50.50565784
tr|D4AEH9|D4AEH9_RAT	4-alpha-glucanotransferas	Agl	35	27.1	174.33	1532	0	323.31	35.02405666	30.81049049	35.91456302	44.96163549	42.95209133	44.65967697
tr|Q4G079|Q4G079_RAT	Aminoacyl tRNA synthetas	Aimp1	2	5.7	34.574	315	0	4.544	0.613461602	0.470321234	0.482440572	0.838301186	0.753178363	0.764315772
sp|P34058|HS90B_RAT H	Heat shock protein HSP 9	Hsp90ab1	17	24.2	83.28	724	0	323.31	66.11758401	55.77003814	67.37610991	98.05663202	109.6277371	88.11439507

tr|D4AD15|D4AD15_RAT E	Eukaryotic translation initia	Eif4g1	11	9.2	175.09	1592	0	71.797	2.028432087	2.223792635	2.422676781	2.772079741	3.040379249	2.762667917
sp|Q4KM49|SYYC_RAT T	Tyrosine--tRNA ligase, cyto	Yars1	3	5.3	59.115	528	0.00055648	4.0892	1.075251133	0.893288035	1.038610212	1.572382112	1.515578659	1.300156713
sp|P28480|TCPA_RAT T-c	T-complex protein 1 subun	Tcp1	6	12.2	60.359	556	0	12.389	5.196386535	4.757703997	4.785071359	6.000578523	6.595029504	5.87300609
tr|F1LPK7|F1LPK7_RAT P	Plastin-3 (T-plastin)	Pls3	7	14.3	70.748	630	0	33.114	2.452140538	2.135391769	2.345425916	2.797821249	2.944708447	2.760026354
sp|Q9ES53|UFD1_RAT Ub	Ubiquitin recognition factor	Ufd1	3	10.7	34.485	307	0	14.643	2.363184341	2.326754224	2.368203258	3.420753806	3.077806271	3.135534615
tr|G3V8C3|G3V8C3_RAT	Vimentin	Vim	18	40.8	53.7	466	0	297.86	23.73820953	16.80603618	22.10682843	43.32815463	49.86638303	36.45254567
sp|P62832|RL23_RAT 60S	60S ribosomal protein L23	Rpl23	7	52.9	14.865	140	0	71.545	9.419924417	8.432879915	8.491649288	10.79769674	10.89536193	10.01294402
tr|G3V624|G3V624_RAT C	Coronin	Coro1c	5	11.6	53.177	474	0	32.767	1.774508052	1.420830443	1.512360634	2.272240568	2.282239378	2.153584577
sp|Q5RKL5|STEA3_RAT M	Metalloreductase STEAP3	Steap3	2	5.7	54.673	488	0.00055402	4.0029	1.009575069	0.825667048	0.899201901	1.305829092	1.130145051	1.330433082
tr|Q9WVJ6|Q9WVJ6_RAT	Tissue-type transglutamina	Tgm2	11	21.1	76.934	686	0	91.219	8.965359756	7.482237438	7.639099774	11.58547051	11.39960207	11.20632065
tr|Q8R3Z7|Q8R3Z7_RAT E	EH-domain-containing 4 (P	Ehd4	9	21.1	61.467	541	0	51.999	9.074736242	7.144132507	8.25335621	11.66669661	11.33272958	12.05263662
tr|A0A0G2K7P7|A0A0G2K	Mitochondrial carrier 2	Mtch2	8	36.3	26.998	245	0	160.84	31.04937537	30.83714402	29.45823848	36.77810539	34.12815865	37.34661296
sp|Q6MG60|DDAH2_RAT	N(G),N(G)-dimethylarginine	Ddah2	4	15.4	29.688	285	0	10.976	1.487068654	1.261699013	1.603104102	1.975765284	1.981906405	2.122089025
tr|F8WFS9|F8WFS9_RAT	Beta-adducin (Adducin-63	Add2	4	5.4	80.576	725	0	8.7322	2.026174315	2.315747304	2.440331508	3.695429437	3.928327248	3.131470673
sp|Q6AXM8|PON2_RAT S	Serum paraoxonase/aryles	Pon2	2	7.9	39.617	354	0	4.6564	0.695092585	0.655084473	0.767020137	0.918511965	0.868587335	1.015660438
tr|A0A0G2K1L8|A0A0G2K	Brain acid soluble protein 1	Basp1	3	21.9	21.719	219	0	21.836	1.246189522	1.095410617	1.063354273	1.385741245	1.498860536	1.626389674
tr|D3ZDT1|D3ZDT1_RAT E	Erythrocyte membrane pro	Epb41l2	19	25.6	102.59	926	0	156.78	14.83707083	13.70484891	14.97587378	21.52551513	25.41855635	21.59477294
tr|A0A0G2JW88|A0A0G2J	Microtubule-associated pro	Map4	14	8.4	233.39	2201	0	54.402	10.5437929	9.539988469	11.41885792	14.15962134	14.11279149	12.47935055
tr|M0R5J4|M0R5J4_RAT U	Alpha-enolase (EC 4.2.1.1	Eno1	10	38.2	47.074	434	0	303.74	17.46762549	15.21027963	17.93784301	20.07658476	22.26961717	21.36008027
tr|Q4V8F6|Q4V8F6_RAT P	Pcbp2 protein (Poly(rC)-bin	Pcbp2	7	26.3	35.346	335	0	17.598	5.042858073	4.383221937	4.233795646	6.636650597	7.501043861	6.072139258
sp|Q9ESR9|ABCA2_RAT	ATP-binding cassette sub-	Abca2	2	0.7	270.92	2434	0.0024704	2.5631	0.998486902	0.995459889	0.907983527	1.172164368	1.147024961	1.140748579
tr|A0A0G2JUL7|A0A0G2J	Septin-11	Septin11	5	16.1	49.341	429	0	77.96	3.167854103	2.422312056	2.342590182	4.142232741	4.557899367	3.623715673
tr|B1WC34|B1WC34_RAT	Glucosidase 2 subunit beta	Prkcsh	7	13	59.218	525	0	20.274	6.516931998	6.357853449	6.471417956	10.27569956	8.779171256	9.563167218
sp|O35244|PRDX6_RAT P	Peroxiredoxin-6 (EC 1.11.1	Prdx6	2	8.9	24.818	224	0.0015666	3.1098	2.202280492	2.078530911	2.651410693	3.082948769	3.390866588	3.231850045
tr|D3ZTW7|D3ZTW7_RAT	ATP synthase mitochondri	Atpaf2	2	11.4	34.192	298	0	10.092	0.621940789	0.666683693	0.538286225	0.275278854	0.448525643	0.335346352
tr|F1LZX9|F1LZX9_RAT In	Integrin subunit alpha V	Itgav	5	6.7	115.58	1047	0	14.49	2.395395214	3.022460647	2.998879505	3.953919572	3.535235662	4.006031037
tr|M0R9K1|M0R9K1_RAT	Heterogeneous nuclear rib	Hnrnpk	7	17.7	50.969	463	0	15.379	4.755067466	3.997831674	3.845894765	6.075832121	6.178694335	5.196258113
tr|A0A0G2JSS9|A0A0G2J	Atlastin-3 (EC 3.6.5.-)	Atl3	4	9	60.249	536	0	25.106	1.894119769	1.702321307	2.022289525	2.461927414	2.289196274	2.26524139
sp|P16290|PGAM2_RAT P	Phosphoglycerate mutase	Pgam2	13	41.1	28.755	253	0	116.99	59.28907954	58.76609205	66.51166861	74.53689655	85.92575443	76.59108693
sp|Q6AYH6|EMC10_RAT	ER membrane protein com	Emc10	3	22.5	26.968	258	0	51.244	1.8367222	1.97379242	2.271285209	2.856650451	2.750185008	2.862234501
tr|D3ZPR0|D3ZPR0_RAT C	Chromosome segregation	Cse1l	3	3.3	110.21	971	0	21.316	0.236584349	0.168623047	0.277595427	0.778755284	0.63793118	0.51689281
tr|D3ZM27|D3ZM27_RAT A	Ankyrin repeat domain 2	Ankrd2	3	12.3	37.073	332	0	23.453	1.379699076	1.082034495	1.283032133	1.896092031	1.78037214	2.263920609
tr|D3ZM60|D3ZM60_RAT P	Protein phosphatase 1, re	Ppp1r3a	12	14.1	122.13	1096	0	241.67	5.95299085	5.522216007	7.143761188	8.385400862	8.273313241	9.225555219
sp|Q6XFR6|GLPC_RAT G	Glycophorin-C (CD antigen	Gypc	4	42.1	10.443	95	0	127.1	2.808216189	3.407505401	3.946334611	6.382819018	5.194751958	4.995804163
tr|A0A0G2JXM0|A0A0G2J	MIA SH3 domain ER expo	Mia2	5	9.4	85.653	755	0	27.371	1.30654728	1.345114682	1.574197917	2.323006884	1.843739216	2.155108556
tr|Q3KRF2|Q3KRF2_RAT	Vigilin (High density lipopro	Hdlbp	3	3.5	141.69	1268	0	16.738	3.561258243	2.637465252	3.707081043	4.75931224	5.38722608	4.671399976
sp|O88377|PI42B_RAT Pl	Phosphatidylinositol 5-pho	Pip4k2b	3	10.1	47.263	416	0	15.267	3.497037187	3.551039582	4.163359688	4.961063552	5.104474099	4.444632
tr|A0A0G2JY31|A0A0G2J	Alpha-1-antiproteinase (Al	Serpina1	5	14.1	46.121	411	0	17.326	4.866651551	3.860911517	4.72606981	6.321899439	6.277385185	5.521983081
tr|G3V852|G3V852_RAT R	RCG55135, isoform CRA_	Tln1	22	12.3	269.67	2541	0	236.13	21.49850021	20.4190699	19.41928501	27.99732452	33.06574896	28.19563108
sp|P36860|RALB_RAT Ra	Ras-related protein Ral-B (	Ralb	3	19.4	23.317	206	0	21.564	4.988671557	4.474436231	4.780497595	7.026655352	6.614983392	5.885807508
tr|D4A8G5|D4A8G5_RAT	Transforming growth factor	Tgfbi	3	5.5	74.752	685	0	5.4022	0.20750927	0.186243991	0.214047557	0.240244124	0.237375761	0.264521999
sp|Q9QZR6|SEPT9_RAT S	Septin-9 (Eighth septin) (E	Septin9	4	5.1	63.791	564	0	7.0359	2.033098148	1.794275977	1.883750229	2.659557695	3.124401295	2.598687848
tr|E9PTW1|E9PTW1_RAT	Secretory carrier-associate	Scamp3	5	15.7	38.497	350	0	10.995	1.661368614	1.554246155	1.634480119	1.851835749	1.777352092	1.949981073
sp|A1L1L2|TM214_RAT T	Transmembrane protein 21	Tmem214	2	3.1	76.565	685	0	5.1856	0.455859117	0.547927421	0.549263257	0.690660795	0.700705032	0.657647449
tr|G3V8V3|G3V8V3_RAT	Glycogen phosphorylase,	Pygm	56	55.6	97.287	842	0	323.31	382.5718545	298.114768	404.1011515	553.6634122	522.3496342	492.0214838
tr|A0A0G2K0B4|A0A0G2K	E3 ubiquitin-protein ligase	Nedd4	4	5.8	102.43	886	0	41.787	2.65975517	2.338649595	2.428165297	3.285775131	3.872510292	3.283157316
sp|P11030|ACBP_RAT Ac	Acyl-CoA-binding protein (	Dbi	3	41.4	10.027	87	0	29.972	5.316801015	5.180162406	6.099113608	6.566175006	7.721075919	7.511790787
sp|P19944|RLA1_RAT 60	60S acidic ribosomal prote	Rplp1	3	66.7	11.498	114	0	107.1	9.53983717	8.189543081	9.617709858	11.71387942	11.08681139	11.74276103
tr|A0A0G2JVH7|A0A0G2J	Wiskott-Aldrich syndrome p	Wasf2	4	11.2	45.338	419	0	6.64	1.160344032	1.037217082	1.09454734	1.489842055	1.706434897	1.429694871
sp|Q9QX69|LANC1_RAT G	Glutathione S-transferase	Lancl1	2	5.8	45.239	399	0	12.518	6.753747142	7.432879054	8.060800767	8.937260577	9.610223711	9.358039735
tr|D4A4J3|D4A4J3_RAT A	Alpha-mannosidase (EC 3	Man2a2	4	4.6	131.96	1164	0	7.7297	0.986395281	0.879714479	1.100493232	1.774432049	1.368243467	1.697102268
tr|D3ZZZ9|D3ZZZ9_RAT C	Catenin (Cadherin associa	Ctnnd1	5	6.9	104.07	932	0	9.5782	3.064799377	2.773990542	2.490551431	3.823898078	3.393724848	3.505962948
sp|P50137|TKT_RAT Tran	Transketolase (TK) (EC 2.2	Tkt	4	7.5	67.643	623	0	9.0138	2.031793658	1.505035846	2.012272983	2.863160484	2.6992217	3.502203801
tr|A0A0G2JZM2|A0A0G2J	Protein transport protein S	Sec23a	5	7.3	84.712	751	0	13.279	0.992165142	0.863623646	1.029005309	1.404494919	1.499831266	1.212070765
sp|Q5QD51|AKA12_RAT	A-kinase anchor protein 12	Akap12	4	3.6	181.11	1687	0	9.9924	1.380351321	0.956614841	1.188446704	2.050361906	2.286931238	1.683488062
sp|Q91Y81|SEPT2_RAT S	Septin-2 (Vascular endoth	Septin2	7	28.8	41.592	361	0	52.574	3.858431145	3.218265357	3.352705848	4.968170836	5.821681535	4.66550726
sp|P62076|TIM13_RAT Mi	Mitochondrial import inner	Timm13	3	43.2	10.458	95	0	36.297	4.577054727	4.12290582	4.29019015	4.847048379	4.997154541	5.090697214
tr|D4A781|D4A781_RAT I	Importin 5	Ipo5	8	10.3	123.7	1097	0	56.772	2.047598058	1.744917593	1.969005181	2.726091431	2.435506807	3.008130026
tr|D4A8D5|D4A8D5_RAT F	Filamin B (Filamin, beta (Pr	Flnb	11	5.9	275.31	2578	0	73.201	3.202021711	2.653013143	2.744441042	3.965207612	3.978427684	3.918859476
tr|A0A0U1RVI9|A0A0U1R	40S ribosomal protein S2	Rps2	6	26.6	28.589	267	0	56.978	16.06680371	11.78135269	14.47413193	19.13352579	21.15166017	20.63568257
tr|A0A0G2K6J5|A0A0G2K	Myosin light polypeptide 6	Myl6	4	28.5	16.961	151	0	13.908	2.878156933	2.382035614	2.392352729	4.313225637	5.374336947	4.079994785
tr|E9PTU4|E9PTU4_RAT M	Myosin-11 (Myosin heavy c	Myh11	8	5.1	227.29	1972	0	28.685	3.217625421	3.207505229	3.218786053	5.013084094	5.447411319	4.490960941
sp|P11980|KPYM_RAT Py	Pyruvate kinase PKM (EC	Pkm	37	76.6	57.817	531	0	323.31	161.6464047	143.405849	161.2983431	217.8293022	226.4011232	188.262123
sp|A2RRU1|GYS1_RAT G	Glycogen [starch] synthase	Gys1	8	15	84.071	738	0	38.246	6.883192707	5.835148162	6.15399877	7.745745284	8.123389436	7.735409208
sp|P05545|SPA3K_RAT S	Serine protease inhibitor A	Serpina3k	3	13.2	46.561	416	0	15.687	3.249234222	1.453900561	2.105806446	5.334584185	4.227689491	4.537696277
tr|M0RB00|M0RB00_RAT	Complement C4 [Cleaved	C4	4	4	190.6	1734	0	41.909	2.306689881	2.337070126	2.137777053	3.49678383	3.300966235	4.105394424
tr|C0JPT7|C0JPT7_RAT F	Filamin A (Filamin alpha) (F	Flna	35	17.8	280.49	2639	0	323.31	26.27644642	26.39933171	24.6987802	36.13247731	41.98783319	35.22625111

sp|P63018|HSP7C_RAT H	Heat shock cognate 71 kD	Hspa8	27	47.8	70.87	646	0	323.31	111.6944647	93.97836323	108.8967348	136.274709	157.1449544	131.0519765
tr|G3V8L9|G3V8L9_RAT (	Caveolae-associated prote	Cavin1	14	43.9	43.908	392	0	162.04	14.91333334	11.69003968	14.15899962	20.69892006	23.82008816	18.25827638
tr|Q499Q4|Q499Q4_RAT	Phosphoglucomutase-1 (P	Pgm1	24	45	61.389	562	0	323.31	40.66898713	40.91958113	53.21573816	60.3641357	67.66525079	64.93366869
tr|M0RC65|M0RC65_RAT	Cofilin 2 (Cofilin 2, muscle	Cfl2	7	41.6	18.709	166	0	46.164	10.97026085	8.545910614	10.16747623	13.41604413	13.34375787	14.0866397
tr|Q5XI86|Q5XI86_RAT P	Aminoacyl-tRNA hydrolase	Ptrh2	3	27.6	19.529	181	0	19.158	4.369189231	4.402570424	4.204294871	5.173087576	4.878186228	4.740588592
tr|A0A0G2K3Z3|A0A0G2K	RAB22A, member RAS on	Rab22a	4	29.9	20.841	187	0	24.108	1.615912148	1.648816819	1.589428549	2.668815082	2.477356042	2.18853448
tr|B4F7A5|B4F7A5_RAT C	CD99 molecule (Xg blood	Cd99	2	24.8	16.588	165	0	60.549	1.894671669	2.15399988	1.94851472	2.26166923	2.437502196	2.359626448
sp|P51886|LUM_RAT Lum	Lumican (Keratan sulfate p	Lum	6	25.1	38.279	338	0	63.394	3.278986633	2.943141724	3.636965249	4.882106999	5.572527587	6.569464187
tr|Q5VLR5|Q5VLR5_RAT	BWK4 (Endoplasmic reticu	Erp44	3	9.1	46.878	406	0	26.925	1.921564236	1.646003391	1.602097874	2.028801153	2.203556346	2.267679755
sp|P09456|KAP0_RAT cA	cAMP-dependent protein k	Prkar1a	3	9.7	43.094	381	0.00054675	3.833	0.997433275	0.873495323	1.026398264	1.815881252	1.411063431	1.495429136
tr|F7EMB2|F7EMB2_RAT	RNA transcription, translati	Rtraf	9	40.6	28.17	244	0	36.628	4.676497018	3.740032835	4.944238328	9.083587017	8.334792786	6.525776808
tr|D3ZFR7|D3ZFR7_RAT F	PDZ and LIM domain prote	Pdlim3	8	32.4	39.404	364	0	39.115	7.277550129	7.619453746	7.993566444	9.104490794	9.916003556	10.77757475
sp|Q80U96|XPO1_RAT E	Exportin-1 (Exp1) (Chromo	Xpo1	2	3	123.04	1071	0	4.8812	0.210017905	0.169822456	0.217871224	0.26667247	0.243874257	0.28762551
tr|Q4V8E2|Q4V8E2_RAT	26S proteasome regulator	Psmd14	2	12.6	34.577	310	0	11.656	0.884946082	0.8356868	0.763589814	0.928963853	1.007725254	1.044433149
sp|Q4AEF8|COPG1_RAT	Coatomer subunit gamma-	Copg1	2	3.1	97.613	874	0	4.8399	0.740498879	0.736871315	0.687711078	0.948912029	0.950937568	1.116974517
sp|Q9Z1X1|ESYT1_RAT E	Extended synaptotagmin-1	Esyt1	12	16	121.16	1088	0	70.194	4.030372988	4.611455105	4.501315072	5.344140198	5.485701211	6.317702966
tr|Q5U3Y8|Q5U3Y8_RAT	Transcription factor BTF3	Btf3	6	57.4	17.699	162	0	142.39	0.252273353	0.207448352	0.473338783	0.928844403	0.681344368	0.612456411
tr|G3V8V6|G3V8V6_RAT	ADP-ribose glycohydrolase	Macrod1	18	79.4	27.078	243	0	323.31	108.5285673	117.9566662	138.964656	191.2934503	154.2273724	165.5548459
sp|B1PRL5|CAVN4_RAT (	Caveolae-associated prote	Cavin4	5	17.1	41.093	362	0	19.607	3.912918697	3.082875309	3.06433006	5.112884698	5.571448999	4.287763831
sp|A0JPQ4|TRI72_RAT Tr	Tripartite motif-containing p	Trim72	38	77.4	52.831	477	0	323.31	524.0538511	468.337027	602.4103887	769.617347	726.6990152	642.1943084
tr|Q80ZA3|Q80ZA3_RAT A	Alpha-2 antiplasmin (Serin	Serpinf1	2	6.5	46.465	418	0	4.3588	0.33075349	0.185108748	0.285059809	0.653153446	0.600288441	0.428806863
tr|G3V9L3|G3V9L3_RAT D	Dual specificity protein pho	Dusp3	2	15.1	21.095	192	0	10.125	2.393137443	2.171225956	2.364955886	2.563758671	2.782111228	2.916386531
tr|G3V6F1|G3V6F1_RAT M	Myosin-binding protein H (	Mybph	5	14.3	52.624	484	0	28.028	2.386564819	1.94476969	2.028875745	2.684343602	2.864946826	2.714713399
sp|P06302|PTMA_RAT Pr	Prothymosin alpha [Cleave	Ptma	2	25	12.382	112	0	25.087	1.575924506	0.943090644	1.331285338	1.940348314	2.365506412	1.944596349
tr|A0A0U1RRW6|A0A0U1F	Diacylglycerol lipase-beta (	Daglb	2	5.8	39.569	359	0.0010712	3.571	0.721082044	0.902073827	0.6826342	1.122174479	1.028326295	1.098889975
tr|A0A1W2Q6E9|A0A1W2	Moesin (Membrane-organiz	Msn	25	38.7	67.651	576	0	313.95	41.78282107	36.92155202	39.13998103	56.7800338	65.04428068	51.77157584
tr|Q6IRH7|Q6IRH7_RAT C	Caseinolytic peptidase B p	Clpb	2	3.5	75.678	677	0	5.4159	2.182111067	1.693732949	2.493067001	4.328813883	3.143923747	3.455366867
sp|Q63041|A1M_RAT Alp	Alpha-1-macroglobulin (Alp	A1m	4	2.5	167.12	1500	0	5.7358	1.366252793	1.245854972	1.070397869	1.493186659	1.596311009	1.775536353
tr|D3ZZV1|D3ZZV1_RAT F	Presequence translocase-	Pam16	8	47.2	13.771	125	0	66.043	22.66601893	23.37761144	21.35581647	25.93262859	24.06924211	25.48802958
tr|D3ZAP9|D3ZAP9_RAT (	Glycerol-3-phosphate dehy	Gpd1l	4	15.1	38.066	351	0	47.783	3.47701828	3.616834308	3.213160324	4.18009844	4.19872939	4.862202063
sp|P04642|LDHA_RAT L-l	L-lactate dehydrogenase A	Ldha	20	56.9	36.45	332	0	323.31	126.7362401	99.70393578	120.4363401	168.9025188	182.7883964	143.182844
sp|Q6P4Z9|CSN8_RAT CO	COP9 signalosome comple	Cops8	2	16.3	23.235	209	0.00055556	4.0739	0.921572153	0.891708566	0.821036283	1.130715165	0.991654285	1.035695673
tr|A0A140TAA4|A0A140T/	Programmed cell death 6-i	Pdcd6ip	7	10.4	95.881	868	0	30.264	3.828076662	3.641464142	3.268045486	4.262817672	5.22274133	4.823391415
sp|Q04462|SYVC_RAT Va	Valine--tRNA ligase (EC 6.	Vars1	2	2.4	140.37	1264	0	14.675	0.701916072	0.592152533	0.729789702	0.983373399	0.80705386	0.986908047
tr|D4A5E5|D4A5E5_RAT S	Beta-sarcoglycan	Sgcb	6	31	32.846	297	0	56.308	6.738193605	7.314418932	7.380682137	9.331446088	8.460987643	10.11921611
tr|Q6P9V1|Q6P9V1_RAT	CD81 antigen (26 kDa cell	Cd81	2	18.2	25.904	236	0	61.471	1.293100996	1.157898331	1.063948862	1.417753887	1.403081876	1.607492343
tr|M0R9X8|M0R9X8_RAT	Cytoplasmic dynein 1 heav	Dync1h1	51	14.3	532.04	4644	0	304.99	29.7825147	27.81789167	26.24699914	35.00188159	39.1872781	32.97787508
sp|P19139|CSK21_RAT C	Casein kinase II subunit al	Csnk2a1	2	10.2	45.073	391	0	13.467	0.801358364	0.58889488	0.969775072	1.254107175	1.109975446	1.460784029
sp|P38983|RSSA_RAT 40	40S ribosomal protein SA	Rpsa	8	37.3	32.824	295	0	323.31	17.51127574	11.92251767	20.30888201	29.69053046	28.27142299	23.20511004
sp|Q3B7U9|FKBP8_RAT F	Peptidyl-prolyl cis-trans iso	Fkbp8	3	9.4	43.555	403	0	16.262	1.50036442	1.198964507	1.349626129	1.785839538	1.775087056	1.677696944
sp|Q8VIF7|SBP1_RAT Me	Methanethiol oxidase (MTC	Selenbp1	3	6.6	52.531	472	0	5.8657	1.114686875	1.29570694	1.360328736	1.490797656	1.58870696	1.626389674
tr|G3V9N0|G3V9N0_RAT	Polyadenylate-binding pro	Pabpc1	5	8.2	70.833	644	0	52.303	3.350783767	2.575668555	3.121730792	5.099327106	5.241077335	3.938874392
sp|P06761|BIP_RAT Endo	Endoplasmic reticulum cha	Hspa5	40	51.8	72.346	654	0	323.31	155.0486947	141.4710004	165.0991405	181.4985376	208.2053349	222.0639624
sp|O08557|DDAH1_RAT N	N(G),N(G)-dimethylarginine	Ddah1	3	10.5	31.426	285	0	5.6346	0.190786709	0.201717844	0.259826356	0.369859487	0.285863708	0.388705913
sp|Q6P7Q4|LGUL_RAT La	Lactoylglutathione lyase (E	Glo1	3	25	20.819	184	0	31.672	2.880615395	2.757949067	3.547228009	4.633770128	3.798573049	4.921942013
tr|B2RYJ3|B2RYJ3_RAT C	Cullin 4A (RGD1563853 pr	Cul4a	2	4	87.773	759	0	10.992	0.327893646	0.329442534	0.34684678	0.458682622	0.427239699	0.519270216
tr|D4A133|D4A133_RAT A	H(+)-transporting two-secto	Atp6v1a	5	9.6	68.264	617	0	13.089	4.006290092	3.808986497	4.894841683	6.04417783	5.727844334	7.442805368
tr|E9PT65|E9PT65_RAT R	Radixin	Rdx	9	17.7	68.485	583	0	43.35	4.624367583	4.117377681	4.431336491	5.892177508	5.801727648	5.008199187
tr|Q6P3V8|Q6P3V8_RAT	RNA helicase (EC 3.6.4.13	Eif4a1	2	4.7	46.153	406	0	81.603	6.712605528	6.602671034	6.467758945	8.819004924	10.32478861	8.706894594
sp|Q07936|ANXA2_RAT A	Annexin A2 (Annexin II) (A	Anxa2	30	62.8	38.678	339	0	323.31	133.5396582	117.4680182	143.7442388	152.048102	179.7198121	181.1095847
sp|Q62667|MVP_RAT Maj	Major vault protein (MVP)	Mvp	22	33.1	95.797	861	0	209.19	17.24536043	15.44719987	17.42786839	28.16395748	26.93666969	21.82743363
tr|D4AA52|D4AA52_RAT M	Alpha-1-inhibitor 3 (Alpha-1	A1i3	7	6.6	163.6	1477	0	194.95	17.37781636	12.71965557	14.28615025	20.76163139	19.842793	21.77866632
sp|A1L134|AUP1_RAT An	Lipid droplet-regulating VL	Aup1	4	19.3	46.199	410	0	44.823	5.110591218	4.613478799	5.776205906	6.904816194	6.218062816	7.714479905
tr|F1M614|F1M614_RAT L	Laminin subunit alpha 2	Lama2	3	1.1	343.86	3121	0	5.3733	0.155254403	0.121648673	0.131898191	0.176099391	0.168383847	0.187225818
tr|D3ZWC6|D3ZWC6_RAT	Syntrophin, basic 1 (Predic	Sntb1	5	8.7	58.292	539	0	9.3404	4.647798233	5.016292571	5.018790673	5.499425399	6.176537158	6.375512544
tr|G3V7Q7|G3V7Q7_RAT	IQ motif containing GTPas	Iqgap1	23	19.5	188.83	1657	0	123.18	14.76883596	13.4457174	13.70711179	16.89861339	19.22907613	16.51078123
sp|P63029|TCTP_RAT Tra	Translationally-controlled tu	Tpt1	4	29.1	19.462	172	0	23.157	3.367742139	2.881493102	3.195780023	4.146652397	5.017809512	4.080197982
sp|Q5BJK8|GOLI4_RAT G	Golgi integral membrane p	Golim4	2	4.6	76.596	653	0.0010627	3.3737	0.260140432	0.219042637	0.272678631	0.494965606	0.364649207	0.396925236
sp|P26453|BASI_RAT Bas	Basigin (Glycoprotein CE9	Bsg	10	23.5	42.435	388	0	152	31.95097879	33.07258523	31.67971541	35.4689317	38.29906045	35.30143404
sp|Q6AYT3|RTCB_RAT tR	RNA-splicing ligase RtcB h	Rtcb	7	19.2	55.249	505	0	25.196	5.740760329	5.289244435	5.680156873	7.336031253	7.726468861	6.476298311
tr|M0RAK4|M0RAK4_RAT	Frataxin, mitochondrial (Fx	Fxn	2	9.7	22.883	206	0	4.3032	0.664236375	0.600148592	0.67083389	0.453683633	0.521777982	0.566290028
tr|Q8R490|Q8R490_RAT (	Cadherin-13	Cdh13	13	23.1	78.085	714	0	323.31	34.0863289	31.7571843	41.04861253	60.3701082	45.24301334	53.95188084
tr|D4A4Z9|D4A4Z9_RAT K	Kinectin 1	Ktn1	26	22.2	151.79	1325	0	123.43	15.04378236	14.38253953	14.78880685	16.5175674	17.62305782	16.05155576
sp|P17046|LAMP2_RAT L	Lysosome-associated men	Lamp2	2	7.3	45.155	411	0	7.1681	3.570941574	3.757258911	3.104624917	2.086137227	2.792843184	2.73919865
sp|B2GV54|NCEH1_RAT N	Neutral cholesterol ester h	Nceh1	4	16.4	45.821	408	0	21.65	0.995928094	0.842251465	0.882050288	1.1191285	1.06634654	1.269473949
tr|Q68FT7|Q68FT7_RAT P	Phenylalanyl-tRNA synthet	Farsb	9	18.2	65.651	589	0	41.708	11.13131521	12.01284351	13.69247575	17.07002436	14.39214591	15.4439964
sp|P10111|PPIA_RAT Per	Peptidyl-prolyl cis-trans iso	Ppia	6	44.5	17.874	164	0	67.361	17.56847262	16.00198811	15.02206879	21.65810481	27.23112436	22.21757208

tr|D3ZH41|D3ZH41_RAT C	Cytoskeleton-associated p	Ckap4	11	31.9	36.315	326	0	43.76	13.42872314	10.13623775	10.37375296	24.47951746	18.13916243	17.86204201
sp|Q66HG5|TM9S2_RAT T	Transmembrane 9 superfa	Tm9sf2	2	4.4	75.585	663	0	4.7669	0.864375275	0.771817051	0.936295123	1.206506288	0.989982473	1.151822822
sp|Q63081|PDIA6_RAT P	Protein disulfide-isomerase	Pdia6	5	15	48.173	440	0	142.84	8.608631859	7.482237438	7.635440763	8.920537556	10.18565069	9.356210961
tr|G3V7I8|G3V7I8_RAT R	STE20-like serine/threonin	Slk	2	1.7	137.64	1203	0.0015699	3.1494	0.544725003	0.526111016	0.510843644	0.636370699	0.652330337	0.743650626
tr|D3ZW08|D3ZW08_RAT	Adenylosuccinate lyase (A	Adsl	2	4.5	54.852	484	0	7.4717	1.330078276	1.273051441	1.331559764	1.620699699	1.774655621	1.966440039
sp|P02625|PRVA_RAT Pa	Parvalbumin alpha	Pvalb	15	84.5	11.925	110	0	315.75	40.43066681	32.33023514	48.79748263	60.80610127	59.07429323	64.37995656
sp|Q08013|SSRG_RAT Tr	Translocon-associated pro	Ssr3	2	12.4	21.064	185	0	8.3546	3.112212579	3.16930184	2.860523159	4.207094175	4.440656795	3.617822957
sp|P97541|HSPB6_RAT H	Heat shock protein beta-6	Hspb6	6	61.7	17.505	162	0	77.457	5.041854619	3.980112015	4.712348519	6.96334677	5.770987875	7.696192165
sp|Q5XI73|GDIR1_RAT Rh	Rho GDP-dissociation inhi	Arhgdia	8	61.8	23.407	204	0	32.157	8.550431527	6.697439132	6.976361445	10.6513703	12.73866971	9.622500774
sp|Q3B7D0|HEM6_RAT O	Oxygen-dependent coprop	Cpox	5	13.1	49.278	443	0	12.196	2.887037501	2.966044014	3.520242804	3.758917194	3.779320244	4.1774278
sp|P18422|PSA3_RAT Pro	Proteasome subunit alpha	Psma3	4	18	28.419	255	0	33.481	2.219188692	1.936033256	2.142808193	2.399096633	2.821857215	2.885094176
tr|D4A4W6|D4A4W6_RAT	RCG20695, isoform CRA_	Slirp	2	24.3	12.576	111	0	22.96	2.87810676	2.272953585	2.792739984	4.223876922	3.273785805	4.269476091
tr|F1LQS3|F1LQS3_RAT 6	60S ribosomal protein L6 (	Rpl6	11	29.9	33.545	298	0	73.052	41.11151035	38.40822655	40.11053364	54.11868436	53.66355392	46.12168018
tr|A0A0G2K428|A0A0G2K	Dynactin subunit 1 (150 kD	Dctn1	2	1.9	139.84	1263	0.001548	2.9782	0.737889898	0.563475312	0.70719531	0.909493478	1.128527168	0.882373293
sp|P04785|PDIA1_RAT Pr	Protein disulfide-isomerase	P4hb	23	48.5	56.951	509	0	250.32	82.488936	72.48772281	88.61209377	93.50558113	109.7302031	114.0951776
sp|P06686|AT1A2_RAT S	Sodium/potassium-transpo	Atp1a2	30	29.9	112.22	1020	0	323.31	180.6417889	181.5993666	179.401299	226.3162357	208.3347655	204.6296503
tr|D4ACB8|D4ACB8_RAT	CCT-theta (T-complex prot	Cct8	13	27	59.588	548	0	39.497	10.43843023	7.972366192	10.33807761	12.84566964	13.48397438	12.18674671
sp|Q07009|CAN2_RAT Ca	Calpain-2 catalytic subunit	Capn2	8	16.3	79.918	700	0	81.629	6.15368165	5.573055143	6.259652707	8.912176045	10.55021361	8.087956195
sp|Q99J82|ILK_RAT Integ	Integrin-linked protein kina	Ilk	3	7.5	51.373	452	0	7.7682	0.840041515	0.911106411	0.847426899	0.934816911	0.970999315	0.962311037
tr|M0R3X6|M0R3X6_RAT	Carbonyl reductase [NADP	Cbr1	4	17.3	30.611	277	0	9.6914	0.671260553	0.655084473	0.713095465	0.973279861	0.844103376	1.062111297
sp|P62944|AP2B1_RAT A	AP-2 complex subunit beta	Ap2b1	13	19.5	104.55	937	0	60.474	16.35981228	14.77148359	18.43226685	18.91552925	22.1542082	22.44413686
tr|F1LU48|F1LU48_RAT E	Endoplasmic reticulum-gol	Ergic1	3	15	35.011	313	0	69.756	1.488724353	1.472261879	1.53294257	1.828423519	1.869355693	1.655650058
tr|A0A0G2K013|A0A0G2K	Alpha-actinin-4 (Non-musc	Actn4	12	21.1	102.97	892	0	248.98	9.370755171	7.213727829	8.139012123	11.09393312	12.47441553	9.919981344
sp|Q9R0T3|DNJC3_RAT D	DnaJ homolog subfamily C	Dnajc3	5	11.5	57.56	504	0	10.799	2.396950568	2.44812649	2.525586459	3.013966304	2.834908136	3.302867435
tr|F1LRA1|F1LRA1_RAT F	Protein ERGIC-53 (ER-Gol	Lman1	5	7.9	57.985	517	0	44.89	4.062433343	3.688996265	3.123971936	4.421148852	5.192055487	4.532413152
sp|P85108|TBB2A_RAT T	Tubulin beta-2A chain	Tubb2a	2	6.1	49.906	445	0	7.335	1.688863253	1.559724936	1.604384756	2.056453864	2.18813253	1.844216976
tr|A0A0G2JUE5|A0A0G2J	Myosin-binding protein C, s	Mybpc1	35	30.3	129.63	1158	0	221.62	43.56294847	43.44722397	52.92759106	67.95519303	59.55965806	79.71930644
sp|Q7M6Z3|SMYD2_RAT	N-lysine methyltransferase	Smyd2	2	5.3	49.648	433	0	20.258	1.362891222	1.725569106	1.669149247	2.058006716	2.045381339	2.5528669
tr|D3ZJ32|D3ZJ32_RAT Ex	Extended synaptotagmin 2	Esyt2	6	10.3	93.95	845	0	33.726	2.074540798	2.450693126	2.549141341	2.888842267	2.732226509	3.122022007
sp|Q5RJY4|DRS7B_RAT D	Dehydrogenase/reductase	Dhrs7b	2	8.6	35.342	325	0	6.9676	0.999139147	1.100247739	1.018897292	1.330734449	1.144921714	1.263378036
sp|Q6NYB7|RAB1A_RAT	Ras-related protein Rab-1A	Rab1A	6	36.6	22.677	205	0	138.12	7.764225319	6.742848845	7.060976069	8.672081234	8.099121195	8.245535554
tr|D4A4T0|D4A4T0_RAT S	STIP1 homology and U-Bo	Stub1	3	10.5	34.886	304	0	6.9538	1.416626183	1.276012944	1.243148915	1.484645973	1.60844513	1.765782892
sp|Q4FZT9|PSMD2_RAT 2	26S proteasome non-ATP	Psmd2	5	8	100.19	908	0	32.98	0.547785538	0.50246835	0.503205459	0.973100686	0.718286025	0.937774985
sp|Q4V885|COL12_RAT C	Collectin-12 (Collectin plac	Colec12	2	2.8	81.545	742	0	71.094	0.976812296	0.934847794	0.919189248	1.567843006	1.679200537	1.26937235
sp|P14480|FIBB_RAT Fibr	Fibrinogen beta chain (Live	Fgb	10	26.9	54.235	479	0	97.643	14.76733078	15.42597577	12.60209053	17.31967519	16.92359317	18.39848239
sp|Q9WTT7|BZW2_RAT B	Basic leucine zipper and W	Bzw2	3	9.1	48.049	419	0	8.4414	0.635838627	0.861402518	0.767706201	1.390519251	0.986369201	1.315498094
sp|P05197|EF2_RAT Elon	Elongation factor 2 (EF-2)	Eef2	26	43.6	95.283	858	0	323.31	40.84559504	39.80753674	39.27765131	61.63627984	70.44800917	53.93562507
tr|B1PLB1|B1PLB1_RAT C	CD34 antigen (Predicted) (	Cd34	4	11.4	41.238	386	0	35.74	7.05829543	5.885493714	6.878482906	10.49130709	12.34714208	8.940063278
tr|Q5RKG9|Q5RKG9_RAT	Eukaryotic translation initia	Eif4b	2	4.9	69.063	611	0	20.2	0.232605654	0.1965747	0.227425816	0.410365034	0.369389603	0.294717089
sp|P62083|RS7_RAT 40S	40S ribosomal protein S7 (	Rps7	9	46.9	22.127	194	0	163.48	7.75569596	5.863282441	7.585129365	9.899431574	10.27193777	8.37730888
sp|Q5XIB4|UFSP2_RAT U	Ufm1-specific protease 2 (	Ufsp2	2	3.5	52.306	461	0.0097357	2.037	1.830450613	1.435144375	1.342902697	2.250321465	2.305698679	1.856612
tr|D4AA43|D4AA43_RAT K	Kelch-like family member 4	Klhl40	3	7.9	62.711	560	0	15.122	1.007016261	0.784650231	1.03344186	1.407839524	1.160992683	1.561163401
sp|P31720|C1QA_RAT Co	Complement C1q subcomp	C1qa	2	9.8	25.917	245	0.0010661	3.4088	0.467704892	0.424136594	0.518710517	0.671190419	0.63793118	0.540199519
sp|P61227|RAP2B_RAT R	Ras-related protein Rap-2b	Rap2b	2	19.7	20.504	183	0.00054526	3.8171	2.590466672	2.352025717	3.496230546	4.503868084	3.843226614	5.510502444
sp|P63245|RACK1_RAT R	Receptor of activated prote	Rack1	14	54.6	35.076	317	0	89.524	13.10811959	10.27690914	13.0919406	16.19923273	19.04679467	14.94108355
sp|P19804|NDKB_RAT Nu	Nucleoside diphosphate ki	Nme2	8	51.3	17.283	152	0	23.922	10.59296214	10.46693892	9.694091708	11.11065614	12.72464806	12.18573073
sp|P18420|PSA1_RAT Pro	Proteasome subunit alpha	Psma1	2	7.6	29.517	263	0.0039235	2.5033	1.854232472	1.634552246	2.089661061	2.641102646	3.176766767	2.378726976
tr|F2Z3T8|F2Z3T8_RAT G	Guanine nucleotide-bindin	Gng5	4	49.3	7.1872	67	0	6.8191	2.609431952	2.648620246	2.947013027	2.967619644	3.312507132	3.201472077
sp|P48500|TPIS_RAT Trio	Triosephosphate isomeras	Tpi1	23	91.2	26.849	249	0	323.31	95.28799176	92.59632848	120.1207504	126.5216039	132.5045997	145.8142466
tr|Q6P9V7|Q6P9V7_RAT	Proteasome activator comp	Psme1	5	22.9	28.635	249	0	11.812	4.128259925	3.517377164	3.956625579	5.290327903	6.559975377	7.434067892
tr|D3ZN27|D3ZN27_RAT D	DnaJ heat shock protein fa	Dnajc13	6	4.4	254.51	2243	0	43.53	2.980208205	3.129568341	3.002721466	3.179643669	3.370211618	3.450083742
sp|Q63569|PRS6A_RAT 2	26S proteasome regulator	Psmc3	5	14.8	49.16	439	0	11.293	0.729260194	0.767374797	0.947363631	1.418291412	1.22888983	1.815464585
sp|P63012|RAB3A_RAT R	Ras-related protein Rab-3A	Rab3a	2	14.5	24.97	220	0	7.7406	1.419987754	0.961748113	1.145819228	1.744211159	1.737983611	1.571018461
sp|Q5HZY2|SAR1B_RAT C	GTP-binding protein SAR1	Sar1b	6	42.4	22.41	198	0	63.884	7.38441798	6.476313571	5.869510681	7.961352813	8.684255467	7.940130297
tr|Q5BJU0|Q5BJU0_RAT F	RAS-related 2 (RCG40097	Rras2	4	23	23.399	204	0	14.847	4.61558736	5.39783288	4.742535358	2.951374423	3.711638814	4.178646982
tr|A0A0G2K8Q8|A0A0G2K	Ubiquinol-cytochrome c re	Uqcr10	4	53.2	7.099	62	0	223.68	65.46533891	67.66047285	59.65102338	45.39583681	56.53421726	41.49589796
sp|Q641Y8|DDX1_RAT AT	ATP-dependent RNA helic	Ddx1	11	18.6	82.496	740	0	125.45	11.65461647	8.941764854	11.62330515	15.65991529	14.35978826	12.71709117
sp|Q99068|AMRP_RAT Al	Alpha-2-macroglobulin rec	Lrpap1	5	13.9	42.031	360	0	11.658	1.904806554	1.986428166	1.995395796	2.339849356	2.239958708	2.596046286
tr|A0A0G2JVQ1|A0A0G2J	Creatine kinase (EC 2.7.3.	Ckmt2	6	7.9	47.273	417	0	323.31	214.1922734	159.5805915	163.7910442	254.8887127	259.0014612	216.2118856
tr|G3V8R5|G3V8R5_RAT	Hormone-sensitive lipase (	Lipe	3	3.2	116.84	1068	0	9.0441	0.329785157	0.247976521	0.242400317	0.399130747	0.377775629	0.392627617
tr|A2VD14|A2VD14_RAT A	ATP-binding cassette subf	Abcf2	5	9.3	71.426	624	0	16.632	3.017737384	3.082480442	3.135086181	3.724814175	3.901524323	3.403348407
sp|P02680|FIBG_RAT Fib	Fibrinogen gamma chain	Fgg	5	16.2	50.632	445	0	57.751	8.116437672	9.897836753	7.25627577	10.33243838	11.22055637	12.08616414
tr|G3V811|G3V811_RAT C	Coagulation factor XIII A c	F13a1	4	7.1	83.053	732	0	16.45	1.915292648	1.868264195	2.120305277	2.615420863	2.930039643	3.521710724
tr|A0A0G2K0W9|A0A0G2I	Proteasome subunit alpha	Psma7	3	20.6	27.855	248	0	16.743	4.496075989	4.210023367	4.070283601	4.785471824	4.946730528	4.522558092
tr|A0A0G2K0X9|A0A0G2K	Protein transport protein S	Sec31a	2	2.2	131.97	1216	0.0010616	3.3513	1.723131207	1.349902445	1.801331011	2.55939874	2.230790706	3.202894457
tr|G3V8G2|G3V8G2_RAT	26S proteasome non-ATP	Psmd5	2	4.6	55.852	504	0.0015682	3.1354	0.130047638	0.104496635	0.129766818	0.26452834	0.21591185	0.176944044
tr|B0BNJ1|B0BNJ1_RAT L	LOC683667 protein (Sorci	Sri	2	11.6	21.624	198	0	5.5702	0.474829415	0.507256113	0.460715196	0.59214428	0.615226892	0.73067649

sp|P62859|RS28_RAT 40	40S ribosomal protein S28	Rps28	2	36.2	7.8409	69	0.0010532	3.2193	0.95794736	1.088352368	1.067379185	1.426354298	1.176416498	1.281970571
tr|A0A0G2KAY8|A0A0G2K	Selenoprotein F (15 kDa s	Selenof	2	18	17.672	161	0	10.388	1.287331136	1.328283473	1.290853269	1.508595729	1.598899622	1.776958733
tr|G3V6P8|G3V6P8_RAT	Guanine nucleotide-bindin	Gng12	4	45.8	7.9851	72	0	40.368	2.693722088	2.645313235	3.042238783	4.719654789	3.750683719	5.336565718
sp|P63036|DNJA1_RAT D	DnaJ homolog subfamily A	Dnaja1	2	5	44.868	397	0	6.0916	0.9859939	1.050839996	0.97425736	1.323149364	1.43560132	1.164014648
tr|B5DFL0|B5DFL0_RAT S	Snta1 protein (Syntrophin,	Snta1	4	8.6	53.362	499	0	14.578	3.913871978	4.029914624	4.150827576	5.500321275	6.286013893	4.974366868
sp|Q5U2Z3|NP1L4_RAT N	Nucleosome assembly pro	Nap1l4	5	15.8	43.916	386	0	20.489	1.355766698	1.264808591	1.410045545	2.139411996	2.250852452	3.007926829
tr|M0R6L8|M0R6L8_RAT D	DnaJ heat shock protein fa	Dnajc19	4	21.3	19.364	178	0	41.425	12.95609631	13.24236086	18.61658952	22.12097416	22.67516645	20.12870578
sp|B0BNE5|ESTD_RAT S-	S-formylglutathione hydrola	Esd	2	9.6	31.363	282	0	8.3888	0.479174371	0.422591677	0.512490199	0.543133881	0.573863022	0.655402121
tr|G3V7V5|G3V7V5_RAT	Peptidylprolyl isomerase (E	Fkbp11	3	23.4	22.185	201	0	16.928	3.659546562	2.45395078	3.128591437	7.52655425	5.615131834	4.806526054
sp|Q63610|TPM3_RAT Tr	Tropomyosin alpha-3 chain	Tpm3	6	21	29.006	248	0	19.687	2.585198538	2.745214604	2.476692928	3.265110255	3.937441321	3.216915058
sp|P0C595|DUPD1_RAT D	Dual specificity phosphata	Dusp29	2	11.6	24.073	215	0	4.8451	0.740950433	0.632132825	0.668867172	0.839256787	0.905421133	0.754958545
tr|R9PXU3|R9PXU3_RAT	Protein kinase C and case	Pacsin2	7	15.3	55.342	484	0	12.416	4.63119107	4.692106704	4.428043381	4.896799368	5.52345181	5.327015453
tr|G3V667|G3V667_RAT I	Integrin subunit alpha 6 (In	Itga6	2	1.7	119.48	1073	0.0071702	2.2607	1.023422734	1.09195553	1.195536038	1.352534102	1.385231236	1.19835496
tr|Q5XI77|Q5XI77_RAT An	Annexin	Anxa11	12	30.8	54.16	503	0	218.08	19.23972525	23.13081952	23.08927283	25.44467471	29.21357006	32.81938133
tr|Q5XFV4|Q5XFV4_RAT	Fatty acid-binding protein,	Fabp4	7	59.8	14.736	132	0	119.68	30.74482708	27.62588755	30.9566034	46.94032731	41.72573618	35.93744099
tr|F1M949|F1M949_RAT C	Cytoskeleton-associated p	Ckap5	2	1.6	187.92	1698	0	5.3005	0.070838835	0.067176761	0.072109955	0.102105992	0.087678461	0.083892975
tr|D3ZWP4|D3ZWP4_RAT	Smoothelin-like 1	Smtnl1	4	10	50.601	470	0	16.451	1.716508411	0.822804262	1.255177913	2.183728004	2.380013428	1.899892985
sp|M0RC99|RAB5A_RAT I	Ras-related protein Rab-5A	Rab5a	4	32.1	23.624	215	0	46.941	3.750610013	3.747140442	4.103306174	4.929648161	4.38376125	5.38492663
sp|D4A7N1|MIC25_RAT M	MICOS complex subunit M	Chchd6	2	11.1	29.211	261	0	7.25	3.413148433	3.846795019	3.387557926	4.0723544	4.858501987	5.180611935
tr|A0A0G2JSR0|A0A0G2J	Voltage-dependent anion-	Vdac3	20	68.2	30.783	283	0	323.31	319.1635963	380.0200705	306.917825	231.7273277	292.2435594	243.0948634
sp|P20411|FCERG_RAT H	High affinity immunoglobul	Fcer1g	2	15.1	9.7644	86	0.00056085	4.1946	1.163153703	1.600939187	1.589657237	2.065651526	2.044680257	2.80533931
sp|Q63750|RM23_RAT 39	39S ribosomal protein L23	Mrpl23	3	27.4	17.05	146	0	8.234	1.227475105	1.016535919	1.365451351	1.692250342	1.385878389	1.736725705
sp|P97570|PLPL9_RAT 8	85/88 kDa calcium-indepen	Pla2g6	2	3.7	89.555	807	0	9.0535	0.805522698	0.912537804	0.779826674	0.919109216	1.048603759	0.974259027
sp|O35796|C1QBP_RAT C	Complement component 1	C1qbp	5	31.9	30.997	279	0	323.31	52.65123134	49.2986604	38.49873939	28.10662141	34.13840524	33.77034381
tr|B5DFK6|B5DFK6_RAT A	AP-3 complex subunit delt	Ap3d1	2	2.6	135.67	1204	0.00055835	4.1459	0.394272128	0.409802919	0.453474928	0.65978293	0.580873847	0.8227959
tr|Q3KR94|Q3KR94_RAT	Vitronectin (Vitronectin, iso	Vtn	3	9.8	54.724	478	0	28.71	1.370868681	0.959477628	1.282986395	1.429758627	1.793746638	1.745767976
tr|G3V6T1|G3V6T1_RAT C	Coatomer subunit alpha	Copa	6	7	138.36	1224	0	9.8728	3.341853026	3.533764148	3.062820718	4.009762519	3.635544394	4.334194371
sp|Q5FVQ4|MLEC_RAT M	Malectin	Mlec	7	35.1	32.418	291	0	167.78	8.523338269	7.036037646	7.073325231	10.33721639	10.75676331	8.517718084
tr|F7F588|F7F588_RAT Ni	Nicotinamide-nucleotide ad	Nmnat3	6	28.2	27.577	245	0	23.161	2.46518544	1.6952137	2.179352563	4.66261734	3.076565894	3.47903933
tr|M0RBJ7|M0RBJ7_RAT C	Complement C3 [Cleaved	C3	5	5.8	186.23	1662	0	47.221	3.723466582	2.224286219	2.339662974	4.741753067	4.501543117	4.048905627
tr|A0A0G2K3Z9|A0A0G2K	Peroxiredoxin-1 (EC 1.11.1	Prdx1	7	29.6	22.164	199	0	63.575	11.02946463	10.28628724	12.43194653	16.18012071	19.00472972	13.88750653
tr|D4A871|D4A871_RAT L	Leiomodin 3	Lmod3	2	4.3	66.09	578	0	8.2828	0.413538445	0.465607508	0.566186182	1.01538604	0.653462855	0.977611779
sp|P13221|AATC_RAT As	Aspartate aminotransferas	Got1	12	25.9	46.428	413	0	82.534	10.89098798	9.752723104	15.52564015	19.62267417	15.89515902	18.27656412
sp|P29314|RS9_RAT 40S	40S ribosomal protein S9	Rps9	9	29.9	22.591	194	0	26.518	13.4482905	10.02320705	12.22475504	16.05828155	18.43901004	14.08968765
tr|A0A0G2JTW5|A0A0G2J	26S proteasome non-ATP	Psmd1	3	5.3	105.62	952	0.00055897	4.1633	0.589679743	0.515350888	0.537097046	0.997169892	0.712623435	0.836684423
tr|A0A0G2JVM2|A0A0G2J	Uncharacterized protein	Mia3	10	8.6	153.47	1377	0	54.617	1.802404073	1.785539543	1.708849514	1.952591954	2.209111077	2.314923083
sp|P60192|SNAPN_RAT S	SNARE-associated protein	Snapin	2	25.7	14.904	136	0	101.3	0.808432714	0.759428097	0.639503611	0.518091156	0.585943213	0.597369026
tr|D3ZEA3|D3ZEA3_RAT C	Growth factor receptor-bou	Grb10	3	6.7	50.162	434	0	5.4987	3.109954807	2.552667548	2.623968112	3.99082967	4.067465167	3.150164807
sp|Q5FVI3|LRC57_RAT Le	Leucine-rich repeat-contai	Lrrc57	2	16.7	26.724	239	0	128.43	1.008370924	1.022952509	1.057316905	0.869537401	0.921114596	0.978546486
tr|A0A0G2JUP3|A0A0G2J	Non-specific serine/threoni	Obscn	2	0.4	815.45	7496	0	57.366	0.779081685	0.775173421	0.848981978	0.989286182	0.863841545	0.912669982
tr|D3ZLS5|D3ZLS5_RAT H	HECT-type E3 ubiquitin tra	Hectd1	2	0.9	289.03	2610	0.0010582	3.312	0.313980756	0.229857059	0.419359226	1.384248118	0.689271994	1.077960672
sp|Q9NQR8|NDUF4_RAT	NADH dehydrogenase [ub	Ndufaf4	10	63.2	20.158	174	0	128.3	19.75048334	17.46941287	17.75900886	23.97543781	20.51745012	21.09998797
sp|Q66H98|CAVN2_RAT C	Caveolae-associated prote	Cavin2	2	4.8	46.386	417	0	11.579	0.293264449	0.313963745	0.276625789	0.444647228	0.599101993	0.434821497
sp|P16617|PGK1_RAT Ph	Phosphoglycerate kinase 1	Pgk1	14	44.6	44.538	417	0	323.31	32.97400014	31.58689787	41.07422561	46.70919126	44.81643158	46.82982211
sp|P05964|S10A6_RAT P	Protein S100-A6 (Calcyclin	S100a6	2	16.9	10.035	89	0.001569	3.1457	10.00744673	10.43288164	11.50027091	12.63543737	13.00562037	15.57912248
sp|P50398|GDIA_RAT Ra	Rab GDP dissociation inhi	Gdi1	3	9.6	50.536	447	0	13.203	0.315420713	0.253613249	0.282283535	0.35869687	0.423232742	0.506925992
sp|Q9Z1N1|F16P2_RAT F	Fructose-1,6-bisphosphata	Fbp2	2	10.9	36.887	339	0	5.9589	0.425128339	0.249644835	0.460486508	0.64096953	0.605627454	0.625461026
tr|F1LMW7|F1LMW7_RAT	Myristoylated alanine-rich C	Marcks	2	11	29.959	309	0	25.876	1.496751985	1.179961529	1.212733388	2.179129173	2.155019862	1.562077788
sp|P07483|FABPH_RAT F	Fatty acid-binding protein,	Fabp3	7	57.9	14.775	133	0	24.338	12.56224062	11.72508413	13.35996314	17.82733834	15.73337074	21.25746573
sp|Q498T4|RM02_RAT 39	39S ribosomal protein L2,	Mrpl2	2	6.6	32.999	304	0.0024752	2.5701	1.570656372	1.583762469	1.777684654	1.778075278	1.953485598	1.867279849
sp|Q641X3|HEXA_RAT Be	Beta-hexosaminidase subu	Hexa	2	5.7	60.537	528	0.00055036	3.9102	1.084232046	1.057947604	1.035088414	1.549985208	1.596850303	2.119447463
tr|D3ZQ55|D3ZQ55_RAT J	Junctophilin	Jph1	17	30.3	72.025	660	0	149.86	43.73052528	48.39935065	52.44277213	36.3218058	42.35886764	40.99806503
tr|Q6IN22|Q6IN22_RAT Ca	Cathepsin B (EC 3.4.22.1)	Ctsb	4	18.9	37.544	339	0	74.223	8.743596422	9.026167691	8.590442579	6.218575056	7.883403491	6.209297308
tr|D3ZHD3|D3ZHD3_RAT P	PET100 cytochrome c oxid	Pet100	2	11.8	9.4378	76	0.0097177	2.0257	1.486416409	1.376605331	1.333343531	0.992989136	1.151986469	0.706445235
tr|D4ACA5|D4ACA5_RAT	Mitochondrial import inner	LOC1009111	2	21.5	18.377	172	0.00054496	3.8157	1.982975621	2.128580312	2.475412274	2.587708427	2.557117663	2.871581568
tr|F1LMZ4|F1LMZ4_RAT R	Ribosome-releasing factor	Gfm2	2	4	85.913	779	0	6.3822	0.611454694	0.600592817	0.664613572	0.904118221	0.998341534	1.302493479
tr|A2IBE0|A2IBE0_RAT Ca	Carbonic anhydrase 14 (M	Ca14	2	9.2	37.525	337	0	142.67	1.113583076	1.265400892	1.298857355	1.480823568	1.385716601	1.693343122
tr|A0A0G2K059|A0A0G2K	Calcium uniporter protein	Mcu	14	46.5	36.057	310	0	193.71	24.01867492	22.95954592	21.30093131	19.21475189	21.22716136	20.07993848
sp|Q6DGG0|PPID_RAT Pe	Peptidyl-prolyl cis-trans iso	Ppid	2	6.5	40.765	370	0	6.1711	3.097511977	2.892302588	3.20067395	2.567401901	2.915910134	2.587207212
sp|P62278|RS13_RAT 40	40S ribosomal protein S13	Rps13	8	46.4	17.222	151	0	125.38	14.90279707	11.00988115	14.51392367	19.09350998	19.7861671	15.45720421
tr|Q6IMZ5|Q6IMZ5_RAT T	Tropomodulin-1 (Erythrocy	Tmod1	3	11.1	40.454	359	0	31.314	2.197463913	2.161502354	2.448061168	3.675600711	3.474241481	2.705874324
tr|D3ZVQ0|D3ZVQ0_RAT	Ubiquitin carboxyl-terminal	Usp5	2	3.3	95.778	858	0	19.818	0.331696737	0.316249038	0.301136588	0.441386239	0.447500984	0.357870751
sp|Q9ESN0|NIBAN_RAT P	Protein Niban 1 (Protein F	Niban1	4	5.9	103.46	937	0	11.221	3.455795228	2.528580656	2.801292922	4.260667569	4.505479965	3.386076652
sp|Q5XI74|EEPD1_RAT E	Endonuclease/exonucleas	Eepd1	4	9.3	62.868	569	0	32.631	1.041735769	1.314117617	1.224442223	0.815187581	1.046554441	0.918146145
sp|Q6AYH5|DCTN2_RAT I	Dynactin subunit 2	Dctn2	4	14.4	44.147	402	0	54.253	2.839373436	2.446053438	2.521790236	2.953464801	3.08443959	2.998173368
sp|O08730|GLYG_RAT G	Glycogenin-1 (GN-1) (GN1	Gyg1	6	24.6	37.378	333	0	308.33	8.1987209	6.449660044	9.501993642	11.66550211	9.592966295	13.07573407
sp|Q62991|SCFD1_RAT S	Sec1 family domain-contai	Scfd1	5	11	72.262	637	0	33.394	1.648725093	1.414759362	1.554073357	1.876263306	1.930134156	2.384010101

tr|A0A140TAI6|A0A140TA	60S ribosomal protein L36	Rpl36	3	30.5	12.254	105	0	19.999	13.47638721	12.6974443	14.74764298	20.09689128	18.85480591	15.45618822
tr|A0A0G2K2B9|A0A0G2K	Ankyrin-3 (ANK-3) (Ankyrin	Ank3	5	3.4	202.06	1848	0	9.9361	0.958549433	0.911994862	0.948141171	1.040410848	1.248358353	1.157613939
tr|G3V743|G3V743_RAT M	Mannosyl-oligosaccharide	Mogs	5	8.1	81.025	730	0	14.348	2.403473019	2.581690278	2.510218614	2.606999627	2.902859213	2.861116917
tr|M0R6E0|M0R6E0_RAT	Phospholipid-transporting	Atp9a	5	6.6	90.85	808	0	10.424	1.632770175	1.746299628	1.759755501	2.012675382	2.098717542	2.474026421
sp|Q9JLA3|UGGG1_RAT	UDP-glucose:glycoprotein	Uggt1	9	6.4	176.43	1551	0	16.417	2.816795721	2.609528406	3.045165991	3.121710344	3.200981079	3.344624442
tr|Q6P7Q2|Q6P7Q2_RAT	Glypican-1 (HSPG M12) [C	Gpc1	5	12.4	61.628	558	0	21.065	4.230511888	3.695412855	3.898996159	4.222264345	4.502729564	4.581383656
tr|D3Z841|D3Z841_RAT B	Butyrophilin-like 10	Btnl10	2	5.1	50.132	453	0.0015228	2.8359	0.641708832	0.689487267	0.636164763	0.854367232	1.050868795	0.816324072
tr|G3V7L8|G3V7L8_RAT A	V-type proton ATPase sub	Atp6v1e1	4	19.5	26.143	226	0	10.377	1.177101714	1.163870696	1.321817647	1.634615642	1.444391816	1.91005284
tr|Q6PDW1|Q6PDW1_RAT	40S ribosomal protein S12	Rps12	8	67.4	14.515	132	0	193.7	7.291598485	5.076509799	7.628580118	14.43256495	11.0765648	9.132795738
sp|P14669|ANXA3_RAT A	Annexin A3 (35-alpha calc	Anxa3	7	33	36.363	324	0	250.89	7.453656306	6.005928171	6.693702861	8.086775476	8.754903015	7.483241593
tr|Q4QR73|Q4QR73_RAT	DnaJ (Hsp40) homolog, su	Dnaja4	3	5.8	61.875	555	0	8.6911	4.185707667	3.365254624	4.238872523	6.600815551	6.825847447	4.738455022
sp|Q8CFN2|CDC42_RAT C	Cell division control protein	Cdc42	3	14.7	21.258	191	0	130.88	33.54145338	37.24188793	40.46179868	41.70124352	42.42358295	45.95912249
tr|B0BNI6|B0BNI6_RAT Si	Similar to solute carrier fam	Slc25a35	2	6.7	32.491	300	0.0015322	2.8819	2.790003499	3.165353169	2.99471738	3.249462284	3.26105846	3.458414824
tr|A0A0G2KAK2|A0A0G2K	Band 4.1 (Erythrocyte mem	Epb41	15	24	72.372	638	0	279.9	28.88843718	34.25126344	32.24228832	42.10737405	47.85104038	36.51655276
sp|P61314|RL15_RAT 60S	60S ribosomal protein L15	Rpl15	5	31.4	24.146	204	0	54.628	3.255405464	3.056912799	3.392268902	4.070204298	4.63555774	3.692396297
tr|M0R649|M0R649_RAT E	Exocyst complex compone	Exoc4	3	4.6	110.2	973	0	8.2433	0.892471987	0.700839695	1.113391245	2.34683719	2.373326179	1.338662565
sp|P49242|RS3A_RAT 40	40S ribosomal protein S3a	Rps3a	14	50	29.945	264	0	48.277	37.28182816	30.39637365	42.3681433	58.26718823	56.74454202	42.3716775
sp|Q6AXV4|SAM50_RAT	Sorting and assembly mac	Samm50	20	49	51.96	469	0	323.31	135.2354955	143.435464	115.2268235	99.01820577	113.5591923	103.1123738
tr|A0A140TAH5|A0A140TA	AP-2 complex subunit mu	Ap2m1	13	38.2	49.526	434	0	71.218	12.54066635	12.1535149	13.34852873	18.54702553	15.76411051	21.8436894
tr|D3ZNY3|D3ZNY3_RAT M	Metabolism of cobalamin-a	Mmaa	5	18.1	46.329	415	0	9.7645	6.554059796	5.592798497	5.678784744	7.241665631	6.548650198	6.892344395
tr|A0A0G2K876|A0A0G2K	cGMP-dependent 3',5'-cyc	Pde2a	2	3.5	103.27	916	0.0024594	2.5374	0.795287467	0.614314448	0.681216334	0.888470251	0.787207831	0.910607532
sp|Q68FR6|EF1G_RAT El	Elongation factor 1-gamma	Eef1g	15	33.2	50.06	437	0	96.431	25.08133271	21.66043326	21.92204839	35.21271111	40.82349688	28.31958131
tr|G3V6H5|G3V6H5_RAT	Mitochondrial 2-oxoglutara	Slc25a11	19	66.9	34.136	314	0	323.31	191.5593684	248.3170943	188.7820879	135.1339601	151.3259693	146.6575146
tr|G3V7Z6|G3V7Z6_RAT A	ATP-binding cassette sub-	Abcd2	2	3.9	83.467	741	0	4.2539	0.282417111	0.282157202	0.263192646	0.369208483	0.303617275	0.366059595
sp|P62243|RS8_RAT 40S	40S ribosomal protein S8	Rps8	8	42.8	24.205	208	0	315.55	18.02203383	14.21768253	18.73322049	22.87470463	26.51871664	19.76599894
sp|P82995|HS90A_RAT H	Heat shock protein HSP 90	Hsp90aa1	4	6.8	84.814	733	0	8.6163	1.151062082	0.897927723	1.030697602	1.193366771	1.629639394	1.415775869
sp|P21670|PSA4_RAT Pr	Proteasome subunit alpha	Psma4	3	14.9	29.497	261	0	18.734	1.769591128	1.540179016	1.567291534	1.788706342	2.142292518	1.908427263
sp|Q66X93|SND1_RAT St	Staphylococcal nuclease d	Snd1	12	17.9	101.95	909	0	85.715	5.18886063	4.579569589	5.826059928	7.093547439	8.439955167	6.158091636
sp|Q794F9|4F2_RAT 4F2	4F2 cell-surface antigen h	Slc3a2	6	13.5	58.071	527	0	41.222	6.801912934	6.756669192	7.271826566	7.929101271	7.768533813	9.094086688
sp|Q6AY56|TBA8_RAT Tu	Tubulin alpha-8 chain (Alp	Tuba8	5	16.5	50.037	449	0	71.078	7.039229804	5.517280169	5.655915927	9.420436453	9.841580948	7.022288948
sp|P08753|GNAI3_RAT G	Guanine nucleotide-bindin	Gnai3	2	10.2	40.522	354	0	10.264	0.432799744	0.53667371	0.568564539	0.609136064	0.610427173	0.702960404
tr|D3ZSA9|D3ZSA9_RAT N	Nodal modulator 1 (Similar	Nomo1	16	17.8	133.44	1214	0	190.41	18.27490423	17.68412184	17.01302804	18.96390657	21.37870305	19.45104342
sp|Q78P75|DYL2_RAT Dy	Dynein light chain 2, cytop	Dynll2	2	37.1	10.35	89	0	43.933	0.237582786	0.232344721	0.248812734	0.312099364	0.29834837	0.260051662
tr|D4A8C5|D4A8C5_RAT 1	1-phosphatidylinositol 4,5-	Plcb4	2	3	137.55	1206	0.0039101	2.4772	0.137242403	0.14962007	0.134034139	0.321207439	0.199425624	0.320167527
tr|B0BNK1|B0BNK1_RAT	RAB5C, member RAS onc	Rab5c	3	20.8	23.425	216	0	30.297	2.084675683	2.011156716	2.037886059	2.651315635	2.342748194	2.950625244
sp|P17078|RL35_RAT 60S	60S ribosomal protein L35	Rpl35	4	27.6	14.552	123	0	86.413	13.16180438	11.58194482	14.08444728	17.19903053	18.19740621	14.24716541
tr|D3ZE72|D3ZE72_RAT M	Methionine aminopeptidas	Metap1	5	13.7	43.205	386	0	15.333	1.878666577	1.548816733	1.977375168	2.569193653	3.415350548	2.385534079
tr|D4A994|D4A994_RAT E	ER membrane protein com	Emc1	16	26.4	111.18	993	0	162.99	9.76059705	10.91264513	11.36854652	11.9241117	12.63512521	11.84537557
tr|D3ZBS2|D3ZBS2_RAT I	Inter-alpha-trypsin inhibitor	Itih3	2	2.9	99.066	887	0.0015504	2.9989	0.185498506	0.191456236	0.18792222	0.2527625	0.298299834	0.227712842
sp|Q63638|SPEG_RAT St	Striated muscle-specific se	Speg	30	12.4	354.16	3259	0	164.33	21.8105744	21.82183517	24.3680971	25.476329	25.54421191	29.71656145
tr|A0A0G2JWP4|A0A0G2J	Embigin	Emb	2	7.4	33.238	296	0	6.1139	3.087176401	4.351731288	4.515722427	5.312008106	5.733776571	5.136518162
sp|P97531|CIP4_RAT Cdc	Cdc42-interacting protein 4	Trip10	6	11.7	62.798	547	0	14.798	2.210709506	2.662835461	2.778652793	3.326089559	3.175903896	2.817327939
tr|D3ZUV3|D3ZUV3_RAT E	Eukaryotic translation initia	Eif2a	3	6.6	65.348	589	0	11.695	0.822932625	0.575370683	0.915255811	1.232247796	1.254074872	0.911765755
tr|D3ZML4|D3ZML4_RAT S	Similar to RIKEN cDNA 57	Trabd	6	21.5	42.114	376	0	48.976	2.003094873	1.90414774	1.989404166	2.569611729	2.165805748	2.645524782
sp|A0JPQ9|CHID1_RAT C	Chitinase domain-containin	Chid1	2	5.3	44.874	393	0	4.2551	0.590482506	0.555824763	0.69018091	0.760300235	0.677299661	0.830090677
tr|G3V715|G3V715_RAT M	5'-AMP-activated protein k	Prkaa2	5	10.5	62.213	552	0	146.55	8.191194995	7.912642547	8.313272512	10.424415	11.36562653	9.127919007
tr|A0A0G2K542|A0A0G2K	UTP--glucose-1-phosphate	Ugp2	13	31.8	56.885	507	0	140.89	12.30334948	9.114519198	10.55807563	15.635428	19.17406812	13.10926159
tr|D3ZF13|D3ZF13_RAT A	Acyl carrier protein	Ndufab1	7	28.8	17.514	156	0	120.88	65.69613333	77.80855661	45.84283142	31.55873088	43.85433062	29.3406468
sp|P51638|CAV3_RAT Ca	Caveolin-3	Cav3	2	10.6	17.402	151	0.0010718	3.5752	9.802742118	8.820836813	15.23017503	30.17251184	17.5502531	35.34512142
tr|D3ZF11|D3ZF11_RAT H	Late endosomal/lysosoma	Lamtor5	4	47.4	16.39	156	0	138.47	4.122791101	4.107604721	6.047430081	8.840505952	6.367986621	10.88425323
sp|Q63707|PYRD_RAT Di	Dihydroorotate dehydroge	Dhodh	8	28.6	42.662	395	0	37.584	7.884138072	7.186580717	7.290578996	9.950197889	8.098042606	9.66080343
sp|Q4KLZ1|TM186_RAT T	Transmembrane protein 18	Tmem186	4	19.4	24.64	216	0	35.662	1.93074584	1.987662126	1.406203584	1.018133394	1.509969998	0.831858492
sp|Q66HG9|MAVS_RAT M	Mitochondrial antiviral-sign	Mavs	6	17	53.804	507	0	42.055	3.6898007	2.828975782	3.441482597	4.159553013	3.980207356	5.167505721
sp|Q9JLZ1|GLRX3_RAT G	Glutaredoxin-3 (PKC-intera	Glrx3	2	8.6	37.849	337	0	40.252	0.451012434	0.402404098	0.41812431	0.473004695	0.534413647	0.598699967
sp|P11661|NU5M_RAT NA	NADH-ubiquinone oxidored	Mtnd5	5	9	68.617	609	0	142.09	40.30523506	38.80852304	29.57669896	25.34254483	30.42806073	22.78449202
tr|D3ZUC1|D3ZUC1_RAT S	Smoothelin-like 2	Smtnl2	2	5.5	49.579	456	0.00056054	4.1922	0.314191482	0.403332035	0.53238607	0.64425441	0.530983735	0.639156512
tr|Q5XIM0|Q5XIM0_RAT B	BCS1-like protein (Mitocho	Bcs1l	7	21.1	47.393	418	0	75.083	10.14642511	9.989149764	8.219052984	7.166412033	8.356364557	7.325052643
sp|P19633|CASQ1_RAT C	Calsequestrin-1 (Aspartact	Casq1	11	25.1	46.448	406	0	275.3	366.2406406	381.5501804	457.2116931	245.458123	360.6206786	282.6167014
tr|M0R6J0|M0R6J0_RAT M	Mitochondrial ribosomal pr	Mrpl39	6	17	38.374	336	0	68.77	3.051453439	3.305432263	3.093419196	4.317167492	3.556483856	4.621413487
sp|P63025|VAMP3_RAT V	Vesicle-associated membra	Vamp3	3	32	11.48	103	0	323.31	11.86935563	13.16388103	14.04419816	15.1283621	14.02488652	15.73964819
tr|A0A0G2JVL6|A0A0G2J	NADH dehydrogenase [ub	Ndufa8	16	81.4	19.965	172	0	323.31	243.3074912	237.9814487	201.1495443	198.2932294	196.4702918	166.0221993
tr|Q7TP42|Q7TP42_RAT A	Translocation protein SEC	Sec62	3	4.6	67.93	605	0	5.2015	4.740517383	5.501485486	5.390180268	6.603801805	5.855117779	7.545216712
sp|P70673|KCJ11_RAT A	ATP-sensitive inward rectif	Kcnj11	3	9.7	43.607	390	0	45.294	1.05382739	1.433515548	1.072410325	0.773977278	0.751722269	0.875423952
sp|P0DMW1|HS71B_RAT	Heat shock 70 kDa protein	Hspa1b	3	6.9	70.184	641	0	28.343	1.761162114	1.772607646	2.196321226	2.93710013	2.357309139	3.54589118
sp|Q6P9X4|TP4A2_RAT P	Protein tyrosine phosphata	Ptp4a2	5	40.7	19.127	167	0	15.299	3.269303302	3.046399463	2.859333981	3.41961903	3.604319256	4.177529398
sp|Q9JJW3|USMG5_RAT	ATP synthase membrane s	Atp5md	4	44.8	6.4075	58	0	66.324	55.87733595	55.7651023	32.57022716	20.40865619	33.58670721	21.61001272
sp|P36972|APT_RAT Ade	Adenine phosphoribosyltra	Aprt	4	31.1	19.546	180	0	52.553	2.334335038	2.495362464	2.401866157	2.710144836	3.11917014	2.692260118

sp|Q8VH49|HIG1A_RAT H	HIG1 domain family memb	Higd1a	2	43	10.301	93	0	178.91	3.00780319	2.614168094	2.526043836	4.390211262	3.410496899	3.35671467
sp|P50399|GDIB_RAT Ra	Rab GDP dissociation inhil	Gdi2	5	12.8	50.537	445	0	36.936	8.867021264	7.168318115	7.468955772	10.52535038	12.79529561	9.283974388
tr|Q6PDV8|Q6PDV8_RAT	60S ribosomal protein L22	Rpl22	3	39.1	14.759	128	0	107.55	4.032781277	3.208245605	4.164777555	5.281727492	5.40318919	4.198661898
tr|D3ZIQ3|D3ZIQ3_RAT Fa	Fat storage-inducing trans	Fitm1	4	15.4	32.262	292	0	17.801	15.01317701	21.05678022	17.27693419	10.49190434	15.36233629	10.87714133
tr|A0A0G2K1I6|A0A0G2K	Secretory carrier-associate	Scamp1	3	16.8	33.955	304	0	13.098	1.44201357	1.339241034	1.339472374	1.739672054	1.488560016	1.829993179
sp|Q9QWE9|GGT5_RAT G	Glutathione hydrolase 5 pr	Ggt5	3	8.6	61.608	572	0	44.035	2.950556139	2.737119829	4.121006638	5.286983299	3.834058611	5.041625111
sp|Q3SWT0|PECA1_RAT	Platelet endothelial cell ad	Pecam1	5	9	76.188	678	0	21.453	2.847651932	3.155382775	3.272207611	4.007074891	3.73644635	4.974874861
tr|G3V7U4|G3V7U4_RAT	Lamin-B1	Lmnb1	7	10.7	66.686	587	0	15.07	4.964438143	3.926755601	5.322031192	6.645609359	8.185947571	5.708924423
sp|Q08163|CAP1_RAT Ad	Adenylyl cyclase-associate	Cap1	5	12.2	51.588	474	0	18.74	4.957263447	3.811602491	3.921499075	5.384872701	6.386322625	4.916049297
sp|Q68FS4|AMPL_RAT Cy	Cytosol aminopeptidase (E	Lap3	20	49.7	56.149	519	0	276.74	29.36658301	26.92746642	28.02436363	31.73372536	32.47953609	37.97753998
tr|D4A3E8|D4A3E8_RAT M	Mitochondrial ribosomal pr	Mrps27	6	20.7	47.648	415	0	36.912	8.840429733	7.45805183	8.002256595	9.318306571	8.755981603	9.965497497
sp|P17220|PSA2_RAT Pr	Proteasome subunit alpha	Psma2	2	15	25.926	234	0	19.118	0.994974813	1.029072949	1.060152638	1.468998003	1.165145248	1.522352753
tr|M0RB60|M0RB60_RAT	Leucine-rich repeats and ir	Lrig1	3	4.8	101.2	922	0	12.076	1.45781797	1.392696164	1.555262536	1.650621963	1.628614735	1.906700088
tr|F1M9Q2|F1M9Q2_RAT	ATP-binding cassette sub-	Abcc9	11	10	174.38	1545	0	98.863	7.111478492	8.159928051	7.414527987	5.787479447	6.660823404	6.958485055
tr|M0R402|M0R402_RAT T	Thioredoxin-related transm	Tmx3	2	5.4	52.1	459	0	12.254	4.559494282	5.034555173	4.958874371	5.38815758	6.858205103	6.919979202
tr|A0A0G2JWF4|A0A0G2J	ASPSCR1 tether for SLC2	Aspscr1	4	11.8	48.177	441	0	21.582	2.347680977	1.996151768	2.043923427	2.519442664	2.445645539	2.467524113
tr|A0A0G2JSK5|A0A0G2J	Integrin beta-1 (Beta oligo	Itgb1	9	14.5	88.344	798	0	46.087	7.886646707	7.362296565	7.427791901	8.278492974	10.30375613	9.553312158
sp|O88767|PARK7_RAT P	Parkinson disease protein	Park7	9	66.7	19.974	189	0	215.92	8.317630199	9.267036605	12.0495799	14.28862751	11.98958999	17.61109358
tr|D3ZD09|D3ZD09_RAT C	Cytochrome c oxidase sub	Cox6b1	12	80.2	10.071	86	0	323.31	270.5412327	289.837367	182.8956543	149.6531264	179.6928474	126.6019598
sp|P62329|TYB4_RAT Th	Thymosin beta-4 (T beta 4	Tmsb4x	6	88.6	5.0526	44	0	36.729	10.30547257	6.178682515	7.742009452	11.95218248	15.44916266	10.31936527
sp|Q5U2V4|PLBL1_RAT P	Phospholipase B-like 1 (EC	Plbd1	5	13.8	63.027	550	0	50.018	4.494671153	3.865946072	4.57696512	4.954015992	5.111484924	4.940636147
tr|F1M853|F1M853_RAT R	Ribosome-binding protein	Rrbp1	16	12	157.5	1453	0	40.322	7.945348766	6.598722363	6.958066391	10.80964175	12.13519944	8.359529133
sp|P47820|ACE_RAT Ang	Angiotensin-converting en	Ace	3	3.2	150.91	1313	0	14.21	0.520240726	0.568065642	0.487151549	0.467486098	0.437227428	0.465788737
sp|Q6AY94|TIDC1_RAT C	Complex I assembly factor	Timmdc1	2	10.2	32.05	285	0	4.3548	1.095771767	0.923396649	0.861376877	1.302365037	1.552358527	2.075556887
sp|P29117|PPIF_RAT Pep	Peptidyl-prolyl cis-trans iso	Ppif	6	27.7	21.81	206	0	24.105	26.1755993	21.28432237	23.17571696	35.84818594	26.52518817	36.88027559
sp|Q8CG45|ARK72_RAT A	Aflatoxin B1 aldehyde redu	Akr7a2	3	12.8	40.675	367	0	16.087	1.93837209	1.95607276	2.034364261	2.800628328	2.394196867	3.339036521
tr|G3V6S0|G3V6S0_RAT	Spectrin beta chain	Sptbn1	37	19.7	273.31	2356	0	317.54	38.09211726	37.64021009	35.35976551	48.09839656	58.13592121	43.95661497
sp|P07943|ALDR_RAT Ald	Aldo-keto reductase family	Akr1b1	10	31	35.797	316	0	82.026	10.023502	9.004943586	12.60254791	13.64120767	13.16633006	17.95652867
sp|Q68FR9|EF1D_RAT Elo	Elongation factor 1-delta (I	Eef1d	4	20.3	31.33	281	0	70.204	7.282065672	5.407704556	5.569929173	10.76425069	12.64483251	7.605464655
sp|P15178|SYDC_RAT As	Aspartate--tRNA ligase, cy	Dars1	4	7.6	57.126	501	0	4.4611	0.522799534	0.438776291	0.489712856	0.520121809	0.657130056	0.633771788
tr|E9PTX9|E9PTX9_RAT S	Solute carrier family 12 me	Slc12a2	11	12	130.37	1203	0	88.028	20.58937088	26.48422813	25.13283035	17.81957408	19.28893779	18.96235437
sp|P0C5H9|MANF_RAT M	Mesencephalic astrocyte-d	Manf	2	10.6	20.388	179	0.0015472	2.9682	0.588776634	0.704739007	0.764550304	1.142122655	0.788502137	1.151314829
sp|Q9JHW5|VAMP7_RAT	Vesicle-associated membra	Vamp7	2	15.5	24.775	220	0	14.272	0.259066736	0.348159234	0.248515439	0.168938354	0.230289435	0.190212815
tr|D4A0W1|D4A0W1_RAT	ER membrane protein com	Emc4	4	26.8	20.158	183	0	18.597	2.968317275	2.884355889	3.235022914	3.663894596	3.358832509	3.218540634
sp|Q80W89|NDUAB_RAT	NADH dehydrogenase [ub	Ndufa11	8	77.3	14.854	141	0	149.2	67.55252323	73.00105001	53.60450805	46.05699341	53.20838956	45.54460039
tr|B1WC67|B1WC67_RAT	RCG29001 (Slc25a24 prot	Slc25a24	6	14.1	52.89	475	0	25.127	6.85108218	7.009384119	5.652714292	7.031433359	8.315917487	8.106040737
sp|Q75Q39|TOM70_RAT I	Mitochondrial import recep	Tomm70	16	32.3	67.444	610	0	139.24	17.99644575	15.90475209	15.12863748	19.13531754	19.13469963	18.10486256
tr|B5DEL7|B5DEL7_RAT P	Protein transport protein S	Sec61g	2	36.8	7.7412	68	0.00055525	4.0578	1.703814718	1.731492112	2.043740476	2.755894245	2.028555359	2.763277508
sp|Q7M0E3|DEST_RAT D	Destrin (Actin-depolymerizi	Dstn	2	15.2	18.533	165	0	8.9572	0.749580137	0.606960049	0.620385279	0.821160089	0.761105989	0.785773387
sp|Q62930|CO9_RAT Con	Complement component C	C9	2	4	62.28	554	0.0010471	3.1677	1.11273014	0.974482576	1.702949359	2.073833861	1.543514101	2.193309613
sp|P12368|KAP2_RAT cA	cAMP-dependent protein k	Prkar2a	4	13.2	45.48	401	0	36.041	2.815591576	2.265253678	2.218275291	3.113169658	3.499642241	2.708007894
tr|A0A0G2K2Y3|A0A0G2K	Endoplasmic reticulum ami	Erap1	5	7.9	100.74	884	0	17.179	1.710688378	1.545065496	1.610925237	1.812238023	1.948308373	2.265546185
tr|B0BN81|B0BN81_RAT 4	40S ribosomal protein S5	Rps5	6	29.4	22.906	204	0	69.284	9.987377654	7.247785114	9.546816524	14.35611685	14.2341327	10.03245095
tr|B2RYP4|B2RYP4_RAT	Sorting nexin-2	Snx2	3	7.3	58.53	519	0	7.2876	2.429612996	2.794178121	3.969020478	6.775212777	4.119560992	7.996720692
sp|P08503|ACADM_RAT M	Medium-chain specific acyl	Acadm	26	58.2	46.555	421	0	323.31	117.7703786	103.1935735	104.3229713	146.1233743	121.7510721	165.4634072
sp|P07687|HYEP_RAT Ep	Epoxide hydrolase 1 (EC 3	Ephx1	3	7.3	52.581	455	0	7.3616	0.929348921	0.905134047	0.900848456	1.496531264	1.066831905	1.49583553
tr|D3ZX69|D3ZX69_RAT 3	39S ribosomal protein L10	Mrpl10	2	14.5	29.465	262	0	55.948	0.72193498	0.756515952	0.769169805	1.013534563	0.949211827	1.314075715
tr|A0A0G2K5L2|A0A0G2K	Mitochondrial glutamate ca	LOC1009114	6	28.8	34.662	323	0	45.574	6.544025256	6.53011421	6.228551115	7.434577631	6.660284109	7.495636617
tr|A0A0G2K943|A0A0G2K	AP-2 complex subunit alph	Ap2a2	11	15.5	101.58	915	0	138.45	13.85368591	14.62636995	15.02984419	15.68738882	17.63168653	19.37586049
sp|Q1HCL7|NAKD2_RAT I	NAD kinase 2, mitochondri	Nadk2	3	9.6	48.116	425	0	8.4693	0.093662396	0.078405793	0.090212911	0.099937972	0.103851896	0.097501086
tr|D3ZYY0|D3ZYY0_RAT S	Short chain dehydrogenas	Sdr39u1	4	11.1	31.943	298	0	7.8952	8.801295027	10.58145037	9.807063666	7.084588677	7.754512163	9.101503383
tr|F1LVX2|F1LVX2_RAT E	EH domain-binding protein	Ehbp1	3	3.8	136.19	1205	0.0015306	2.8747	0.263446813	0.190735603	0.340146217	0.375419891	0.395329657	0.407400047
tr|A0A0G2JTE7|A0A0G2J	Myosin XVA	Myo15a	2	0.7	392.77	3490	0.0010576	3.3019	2.106450635	1.24284411	2.726969266	3.547430695	2.773967884	3.316176846
sp|D3ZAF6|ATPK_RAT AT	ATP synthase subunit f, m	Atp5mf	6	46.6	10.452	88	0	70.809	132.2652716	119.7878622	141.2927016	291.1657247	171.2043557	243.6333357
tr|Q5XI19|Q5XI19_RAT Fe	Fermitin family homolog 2 (	Fermt2	6	11	77.875	680	0	18.629	2.343566815	2.134799468	2.147427694	2.719820298	3.249948998	2.523809713
tr|F1M5Z5|F1M5Z5_RAT A	Anoctamin	Ano6	3	4.9	92.226	799	0	30.911	2.219138519	3.167870447	2.798274238	3.245520429	3.358293215	3.995363189
sp|Q5XIC0|ECI2_RAT Enc	Enoyl-CoA delta isomerase	Eci2	13	30.7	43.021	391	0	323.31	59.98648007	51.97931424	54.04816311	76.035996	59.73223222	77.01272093
tr|A0A1B0GWV4|A0A1B0	Translocase of outer mitoc	Tomm5	2	38.3	5.4384	47	0.0010846	3.7637	0.569610663	0.60661454	0.604788746	0.8302383	0.659664739	0.873930453
sp|P42123|LDHB_RAT L-l	L-lactate dehydrogenase E	Ldhb	8	30.2	36.612	334	0	23.21	9.817793928	7.198920313	9.115053251	11.36030697	10.89374405	10.75217511
tr|A0A0G2JSG6|A0A0G2J	Adenylate kinase 2, mitoch	Ak2	7	42.2	25.529	232	0	26.432	9.551376891	9.582930263	9.709642504	14.53111132	11.23080296	12.09733998
tr|M0R7R2|M0R7R2_RAT	Similar to Protein C6orf203	LOC683897	3	19.4	29.264	253	0	23.617	2.374272507	2.116833017	1.997088088	2.358184955	2.526216102	2.870565582
tr|Q5XI04|Q5XI04_RAT R	RCG45489, isoform CRA_	Stom	7	32.7	31.378	284	0	66.481	6.221414795	6.826758098	6.070298898	7.386200318	9.086568985	7.376969504
tr|A0A0G2K0I3|A0A0G2K	Nicotinamide phosphoribos	Nampt	4	13.8	54.091	478	0	68.852	2.709978043	1.76120586	1.92473115	3.823061927	4.446966537	2.590661562
sp|Q923V4|FBX6_RAT F-l	F-box only protein 6 (F-box	Fbxo6	2	6.7	32.786	284	0.0015244	2.8391	0.165966274	0.178672414	0.161769441	0.259947427	0.260004549	0.190182336
tr|A0A0G2K1Q1|A0A0G2K	NLR family member X1	Nlrx1	24	33.8	107.33	971	0	250.88	38.34197731	37.56567893	34.52276679	40.93019277	38.91763097	44.76838742
tr|G3V824|G3V824_RAT I	Insulin-like growth factor 2	Igf2r	11	4.5	273.59	2481	0	22.578	7.815401473	7.605633398	8.219967737	8.413471649	9.413381306	10.22487861
sp|Q641Y0|OST48_RAT D	Dolichyl-diphosphooligosa	Ddost	9	23.8	48.895	441	0	38.668	11.9988012	12.40869775	10.79819821	12.69157894	12.78019537	14.13235905

sp|P53534|PYGB_RAT Gl	Glycogen phosphorylase,	Pygb	2	5.3	96.173	838	0.001065	3.3919	0.588124389	0.539684571	0.664156196	0.948553679	0.666621635	0.92196625
tr|D3ZZC1|D3ZZC1_RAT R	RCG43947 (Thioredoxin d	Txndc5	5	18.9	46.353	417	0	59.812	2.542601916	1.790327306	1.660687784	2.605864851	2.62123975	3.01493713
tr|G3V7K5|G3V7K5_RAT	NPC intracellular cholester	Npc1	2	1.9	142.95	1278	0.0010633	3.3797	0.301603151	0.183544087	0.246749966	0.414778717	0.423335208	0.711261006
sp|Q9Z2L0|VDAC1_RAT V	Voltage-dependent anion-	Vdac1	27	88.3	30.755	283	0	323.31	1418.23171	1754.838628	1388.86902	1151.559217	1247.225835	1156.19156
tr|D3Z9Z0|D3Z9Z0_RAT A	Ankyrin 1 (Ankyrin 1, eryth	Ank1	45	31.9	187.12	1707	0	323.31	59.11347509	62.62098184	58.61277907	71.37146745	84.21079868	67.74286874
sp|Q5PQJ6|P5CR3_RAT P	Pyrroline-5-carboxylate red	Pycr3	2	10.6	28.878	274	0.0015658	3.0882	1.49740423	1.496546204	1.616962605	2.180025049	2.118293923	1.676172966
sp|P31044|PEBP1_RAT P	Phosphatidylethanolamine	Pebp1	6	50.8	20.801	187	0	64.77	7.022672813	6.599215947	7.687581667	7.484746696	8.553746256	9.029165211
tr|F1MAH8|F1MAH8_RAT	CAP-Gly domain-containin	Clip1	5	3.4	148.15	1319	0	6.975	0.691731014	0.706170401	0.664247671	0.799002085	0.825713441	1.006201612
sp|P02651|APOA4_RAT A	Apolipoprotein A-IV (Apo-A	Apoa4	5	14.1	44.456	391	0	11.638	1.96456224	1.685786249	1.943895219	2.13415619	1.989672242	2.311265535
tr|F7EZZ0|F7EZZ0_RAT D	Death-associated protein 3	Dap3	8	21.7	45.111	396	0	24.734	3.114972077	2.894523716	3.329333917	3.812848939	3.487939555	4.51006147
sp|P07895|SODM_RAT Su	Superoxide dismutase [Mn	Sod2	10	44.1	24.674	222	0	188.86	102.3673597	116.8510384	97.86939107	70.72046411	79.18996912	98.89501778
sp|P21531|RL3_RAT 60S	60S ribosomal protein L3 (	Rpl3	4	13.4	46.135	403	0	79.971	4.245965079	3.444425472	3.383533014	5.142747237	5.640478664	4.055306336
sp|P04166|CYB5B_RAT C	Cytochrome b5 type B (Cy	Cyb5b	5	52.1	16.265	146	0	17.512	2.014233213	1.904789399	2.327908401	3.046994272	2.357578786	3.452623706
tr|B0BNE6|B0BNE6_RAT	Complex I-23kD (NADH de	Ndufs8	10	46.7	23.97	212	0	323.31	120.9613623	123.6970463	100.0144861	97.656474	101.4466432	76.82577959
tr|F1M446|F1M446_RAT E	Ecm29 proteasome adapte	Ecpas	2	1.9	203.92	1839	0	6.1448	0.314392172	0.244852136	0.384415673	0.461453865	0.361623766	0.457996128
tr|Q6IRK8|Q6IRK8_RAT S	Spectrin alpha chain, non-	Sptan1	63	31.7	282.38	2452	0	323.31	46.01187795	45.50053275	45.53044337	62.57396356	73.79163358	53.16449204
sp|O08700|VPS45_RAT V	Vacuolar protein sorting-as	Vps45	2	4	64.893	570	0.0010515	3.1946	0.496478935	0.468929328	0.460760934	0.515355748	0.58810039	0.516344178
tr|Q5M9H7|Q5M9H7_RAT	DnaJ homolog subfamily A	Dnaja2	3	8.3	45.745	412	0	8.4652	1.553647827	1.377691216	1.491824436	2.168318934	2.372571167	1.662457161
tr|F1LXA0|F1LXA0_RAT N	NADH dehydrogenase [ub	Ndufa12	11	80	17.177	145	0	213.6	90.13023821	96.65852348	81.91610403	78.26971389	79.10907498	75.54868575
tr|B0BNF6|B0BNF6_RAT M	Membrane-associated ring	March5	5	24.8	31.231	278	0	15.097	0.939182771	0.942054118	0.856254262	0.959841718	1.160183741	1.17519049
tr|F1LYS7|F1LYS7_RAT S	Sarcoglycan, delta	Sgcd	7	24.2	32.087	289	0	47.794	12.42326224	13.04640807	18.61796165	23.0013218	17.57344275	28.42117987
sp|Q5XHZ0|TRAP1_RAT H	Heat shock protein 75 kDa	Trap1	15	28	80.46	706	0	130.83	13.90084825	11.35785775	11.58442816	15.47954555	13.55516122	17.96465655
tr|D3ZLT1|D3ZLT1_RAT N	Complex I-B18 (NADH deh	Ndufb7	11	59.9	16.568	137	0	287.71	61.15550398	58.77102789	52.7720831	69.72902783	61.47415268	77.05640831
sp|Q5RKI0|WDR1_RAT W	WD repeat-containing prot	Wdr1	6	12.2	66.181	606	0	19.532	5.287199122	4.489194388	4.972595662	5.451107811	5.772605758	5.358714203
sp|P62902|RL31_RAT 60S	60S ribosomal protein L31	Rpl31	8	54.4	14.463	125	0	24.317	17.14752367	15.44226403	16.49390588	18.98421309	20.2461851	17.08989299
tr|D4A9G1|D4A9G1_RAT	Ribosomal protein L3-like	Rpl3l	11	30	46.394	407	0	150.5	28.19103665	24.74878735	28.233842	34.34968375	34.68417103	28.35615679
sp|P81795|IF2G_RAT Euk	Eukaryotic translation initia	Eif2s3	6	21.6	51.079	472	0	172.42	5.099051497	4.063083458	4.796505768	7.196274572	8.143882618	5.314518831
sp|P23928|CRYAB_RAT A	Alpha-crystallin B chain (Al	Cryab	11	60.6	20.089	175	0	286.47	61.59200647	54.40774674	65.17612967	66.04996306	67.50346251	72.6825905
sp|P62260|1433E_RAT 14	14-3-3 protein epsilon (14-	Ywhae	10	34.9	29.174	255	0	159.36	48.51800432	35.81000121	45.24412578	52.40218563	63.5396497	49.77516423
tr|B2GV57|B2GV57_RAT	Cysteinyl-tRNA synthetase	Cars2	6	13.8	60.917	550	0	27.308	2.27212089	1.747237437	1.846428319	2.538494963	2.119857877	2.662390143
sp|Q5BK85|TMED1_RAT T	Transmembrane emp24 do	Tmed1	3	17.2	25.209	227	0	17.164	1.774156843	2.286675216	2.160737346	2.376401103	2.748621054	2.368363923
tr|A0A0H2UHG7|A0A0H2U	40S ribosomal protein S20	Rps20	3	22.9	13.241	118	0	23.712	7.301633025	6.19349003	5.837494337	8.061093692	9.02401085	6.987339045
sp|Q63159|COQ3_RAT Ul	Ubiquinone biosynthesis O	Coq3	3	12.5	38.708	345	0	35.85	6.600720407	5.733469891	5.301906633	4.202793969	4.757006807	5.409005487
sp|Q9EQP5|PRELP_RAT	Prolargin (Proline-arginine-	Prelp	10	29.2	43.179	377	0	41.464	7.362341992	8.01333365	9.502451018	9.284860528	10.48334112	10.06211772
tr|Q9JKB7|Q9JKB7_RAT G	Guanine deaminase (Guan	Gda	9	23.1	50.9	454	0	165.07	8.838924552	8.691517847	9.265987446	9.88868106	12.54883813	10.80805432
tr|D3ZV63|D3ZV63_RAT C	Protein odr-4 homolog	Odr4	3	7.4	50.063	447	0	8.705	0.358233078	0.314338869	0.334104275	0.480816735	0.37279255	0.529084637
tr|F1LNQ5|F1LNQ5_RAT A	Asparagine--tRNA ligase (E	Nars2	2	5.4	44.043	390	0.0015568	3.0417	0.82729765	0.764462652	0.687253701	0.646762863	0.707661928	0.588286115
sp|Q5FVN0|MBOA5_RAT	Lysophospholipid acyltrans	Lpcat3	4	9.7	56.017	487	0	11.181	2.887037501	3.603359469	2.696645214	1.969971952	2.801849398	1.932506121
tr|D4A197|D4A197_RAT M	Methylmalonyl CoA epimer	Mcee	2	17.4	18.816	178	0	27.738	1.618521128	1.378579666	1.530061099	2.123166775	1.955534916	2.93823022
sp|P62828|RAN_RAT GTP	GTP-binding nuclear prote	Ran	4	20.8	24.423	216	0	28.743	1.061403468	1.106368178	0.867551458	1.366808396	1.112186552	1.556896262
sp|P61805|DAD1_RAT Do	Dolichyl-diphosphooligosac	Dad1	3	28.3	12.497	113	0	7.1883	2.087535527	2.314562703	2.152550309	2.529058401	2.517533464	3.134518629
sp|P68255|1433T_RAT 14	14-3-3 protein theta (14-3-	Ywhaq	4	22	27.778	245	0	11.562	2.536982573	2.022064919	2.290449278	2.777514723	3.894837074	2.920348875
sp|B5DFN3|UQCC2_RAT U	Ubiquinol-cytochrome-c red	Uqcc2	5	36.8	16.33	136	0	316.18	16.81538039	17.60613559	14.72934793	13.21357612	15.49877773	13.30026688
sp|P40329|SYRC_RAT Ar	Arginine--tRNA ligase, cyto	Rars1	5	9.4	75.81	660	0	9.1312	2.447323959	2.144473712	2.161148985	2.556711112	2.954253956	2.444359643
sp|Q704E8|ABCB7_RAT A	ATP-binding cassette sub-	Abcb7	17	31	82.557	752	0	171.19	16.1074436	17.08688539	13.71488719	12.35950751	14.54746266	11.78238446
sp|P52555|ERP29_RAT E	Endoplasmic reticulum resi	Erp29	2	10.4	28.574	260	0	18.76	1.406240434	1.398717887	1.553844669	2.376102478	1.776273504	2.902264332
tr|Q5U3Z7|Q5U3Z7_RAT S	Serine hydroxymethyltrans	Shmt2	12	28.6	55.764	504	0	171.06	6.420600414	5.775424518	6.25050518	9.271721011	7.260518621	11.13621765
sp|Q5M818|RM16_RAT 39	39S ribosomal protein L16	Mrpl16	6	23.1	28.895	251	0	24.297	4.041260464	3.934603584	3.382572524	5.436833518	4.044814808	5.285664841
tr|D3ZPF2|D3ZPF2_RAT M	Malonyl-CoA-acyl carrier pr	Mcat	7	28.4	41.832	380	0	150.51	2.097218858	2.122953456	2.3569518	2.52009964	2.275012835	2.640140059
tr|A0A0G2K1T0|A0A0G2K	Glutaminase kidney isoforr	Gls	7	15.2	73.865	673	0	29.966	2.274930562	1.868955212	2.011861344	2.49352198	2.215906184	2.373647048
sp|Q68FW9|CSN3_RAT C	COP9 signalosome comple	Cops3	2	4.7	47.859	423	0	9.9149	0.595750639	0.518805975	0.556718491	0.579219773	0.686359805	0.7004814
tr|Q5U362|Q5U362_RAT A	Annexin A4 (36 kDa zymog	Anxa4	10	38.9	35.893	319	0	112.07	13.95553649	14.84107892	19.91782523	20.76043689	19.22691895	26.08339711
tr|F1LT36|F1LT36_RAT Ur	40S ribosomal protein S10	Rps10	7	37.6	18.892	165	0	20.629	6.813954382	5.281840677	6.811248583	8.114249011	8.340185729	6.801820083
sp|P27274|CD59_RAT CD	CD59 glycoprotein (MAC-in	Cd59	4	29.4	13.79	126	0	21.287	46.55223793	67.19156821	53.50388526	35.80876739	45.80711514	38.98438167
tr|F1M9C9|F1M9C9_RAT H	Histidine--tRNA ligase (EC	Hars2	5	12.8	57.327	507	0	7.0815	3.827875971	3.871178061	3.734706574	4.393854492	4.130185089	4.982088358
tr|G3V9W6|G3V9W6_RAT	Aldehyde dehydrogenase	Aldh3a2	3	7.9	54.107	484	0	17.383	0.578541403	0.688253307	0.720276274	0.732229449	0.800744117	0.783721096
tr|Q5XID6|Q5XID6_RAT S	Gamma-sarcoglycan	Sgcg	5	19.2	32.18	292	0	46.846	7.546475801	7.620934497	6.806217443	4.860367071	6.844183452	5.864268614
sp|Q7TPB1|TCPD_RAT T-	T-complex protein 1 subun	Cct4	5	11.5	58.099	539	0	16.98	3.774592563	3.142993821	3.335508497	3.921130504	4.634371293	3.772659155
tr|G3V6U9|G3V6U9_RAT	Actin-histidine N-methyltrar	Setd3	2	3.7	67.42	596	0.0010753	3.6364	0.3839215	0.431875989	0.498311532	0.339077182	0.390373543	0.244334366
tr|A0A0G2K080|A0A0G2K	Lysophospholipase D GDP	Gdpd1	2	9.8	29.329	256	0	9.3545	1.386121182	1.487069395	1.753901084	1.831111147	1.690417858	2.072813726
tr|Q4V7D9|Q4V7D9_RAT A	Acid sphingomyelinase-like	Smpdl3b	6	21.1	51.645	456	0	82.266	10.86991545	11.35884492	10.95736518	13.35393005	11.51770751	13.60201459
tr|G3V7J7|G3V7J7_RAT E	Eukaryotic translation initia	Eif5a	2	13.1	16.793	153	0.0010776	3.6845	6.343334456	5.60612526	6.385431203	7.184926807	8.427551399	6.678377838
sp|B2RZD7|LYRM9_RAT L	LYR motif-containing prote	Lyrm9	3	48.7	9.3787	78	0	5.2267	1.821921254	1.520287587	1.700662477	2.565849049	1.81699022	2.437044547
sp|D4A702|SYNP2_RAT S	Synaptopodin-2 (Myopodir	Synpo2	6	6	136.02	1262	0	101.17	2.940371081	2.329172785	3.583040577	4.594948827	3.250110787	4.812418771
tr|Q5BJZ3|Q5BJZ3_RAT N	Proton-translocating NAD(I	Nnt	53	42.2	113.87	1086	0	323.31	781.6404927	1101.481698	936.7524995	679.0741297	652.5999839	787.6834397
tr|Q5U1W6|Q5U1W6_RAT	MICOS complex subunit	Apool	19	72.8	28.237	254	0	272.84	43.77016172	38.69499876	40.69460324	47.96640414	42.22889772	48.00226944
sp|P52481|CAP2_RAT Ad	Adenylyl cyclase-associate	Cap2	3	8	52.912	477	0	66.088	0.480328343	0.465543343	0.577620591	0.675192	0.622669152	0.543034118

tr|D4AC36|D4AC36_RAT E	Eukaryotic translation initia	Eif3f	2	7.2	37.997	361	0	5.7636	0.251289968	0.23684127	0.27127906	0.462373631	0.307850735	0.352567307
tr|G3V6P7|G3V6P7_RAT	Myosin-9 (Cellular myosin h	Myh9	61	34.8	226.41	1960	0	323.31	72.22861886	73.20835522	60.73043156	89.84443388	115.5437952	80.49856736
tr|A0A096MKD4|A0A096M	LIM domain-binding 3	Ldb3	13	28.7	72.332	679	0	270.73	10.55633607	9.298625971	11.38363994	11.65654335	11.40014136	12.89387266
sp|P86252|PURA_RAT Tra	Transcriptional activator pr	Pura	2	23.9	15.322	138	0	10.834	1.070835935	1.063031517	1.132235151	1.413453681	1.478960578	2.079519231
sp|F1M3G7|AKP13_RAT A	A-kinase anchor protein 13	Akap13	2	0.9	301.4	2760	0.00055679	4.0923	0.053298459	0.039847023	0.066475079	0.078735569	0.077232331	0.062057414
tr|D3ZF97|D3ZF97_RAT E	Endoplasmic reticulum lect	Erlec1	4	9.5	54.665	482	0	8.438	0.603677926	0.675814994	0.568427326	0.722613711	0.665543046	0.836288189
tr|D4A5K6|D4A5K6_RAT Z	CAAX prenyl protease (EC	Zmpste24	2	3.8	54.847	475	0	4.488	0.908125869	0.968905079	1.007371408	1.383352242	1.314152252	2.043654941
tr|D4A6W6|D4A6W6_RAT	60S ribosomal protein L8	Rpl8	8	33.9	28.156	257	0	323.31	20.74089244	18.65450767	19.88397938	28.34671622	28.48390492	21.18126682
sp|P00406|COX2_RAT Cy	Cytochrome c oxidase sub	Mtco2	7	30.8	25.928	227	0	284.89	596.2021936	572.7053301	331.7533607	265.3883813	387.3804598	216.5573207
tr|D3Z9I1|D3Z9I1_RAT Cy	Cytochrome c oxidase ass	Coa3	3	28.7	11.928	108	0	38.898	18.40334635	17.57602698	15.88422321	19.21176564	18.19956339	20.6620982
tr|D3ZA93|D3ZA93_RAT A	Acyl-CoA thioesterase 13 (	Acot13	8	60.7	15.27	140	0	135.85	19.92658952	21.19991953	20.6359061	24.68198547	21.66075395	26.62796537
sp|Q68FW7|SYTM_RAT T	Threonine--tRNA ligase, m	Tars2	12	23.8	81.671	723	0	55.947	9.628642849	8.830214906	9.147984348	9.823580725	9.985572516	11.31604709
sp|P62250|RS16_RAT 40	40S ribosomal protein S16	Rps16	7	46.6	16.445	146	0	20.465	12.29231149	10.07108468	11.8968162	14.77240064	16.57359119	12.23957796
tr|D3ZN37|D3ZN37_RAT R	Rho-associated protein kin	Rock1	2	1.3	158.03	1354	0.0015385	2.9255	2.091198134	2.056714505	1.797534788	1.708674738	1.834517284	1.71244365
tr|D4A104|D4A104_RAT M	Mitochondrial ribosomal pr	Mrpl45	4	15.4	35.439	306	0	74.358	2.76010057	3.121967149	3.330568833	4.802553196	3.388439764	5.17888476
tr|M0R757|M0R757_RAT E	Elongation factor 1-alpha	Eef1a1	5	13.2	50.107	462	0	64.203	7.438102769	7.192503724	6.281149395	8.374053098	9.835648712	7.580979403
sp|Q9Z1N4|BPNT1_RAT 3	3'(2'),5'-bisphosphate nucl	Bpnt1	3	12	33.174	308	0	12.794	2.803148747	2.935688608	3.041003866	3.857762197	3.301128024	4.624867837
sp|Q5U300|UBA1_RAT Ub	Ubiquitin-like modifier-activ	Uba1	13	16.5	117.79	1058	0	71.286	8.037164807	6.155484075	6.153084017	7.687214708	8.665380168	8.627546122
sp|Q6AXT5|RAB21_RAT R	Ras-related protein Rab-21	Rab21	8	34.1	24.163	223	0	57.963	7.993012831	9.44373962	10.10710255	10.86399157	9.936496738	12.27107351
tr|Q6P9V6|Q6P9V6_RAT	Proteasome subunit alpha	Psma5	2	12	26.411	241	0.00054945	3.8872	1.065467456	1.245756255	1.321543222	1.40085169	1.572258485	1.307675006
sp|Q5XIU9|PGRC2_RAT M	Membrane-associated prog	Pgrmc2	4	23.5	23.403	217	0	49.132	12.71877944	10.22656359	11.04243718	11.93366771	16.00301787	15.74980805
sp|P11530|DMD_RAT Dys	Dystrophin	Dmd	64	20.5	425.82	3677	0	323.31	53.12285472	61.87567024	62.12085566	61.15847922	73.52737939	73.6599686
sp|O70257|STX7_RAT Sy	Syntaxin-7	Stx7	2	9.6	29.85	261	0	27.792	0.55506058	0.489699336	0.549491945	0.57318754	0.794650092	0.813022119
sp|P56522|ADRO_RAT NA	NADPH:adrenodoxin oxido	Fdxr	4	14.4	54.362	494	0	26.853	1.71630772	1.650396287	1.441604513	1.897585158	1.756751052	2.266257375
sp|Q63258|ITA7_RAT Inte	Integrin alpha-7 (H36-alpha	Itga7	6	6.3	124.19	1135	0	84.424	2.351393756	2.050544707	2.688046538	3.156768964	2.82137185	4.225077522
tr|F1LUE2|F1LUE2_RAT C	Calcium/calmodulin-depen	Camk2b	10	20.3	72.827	666	0	143.11	11.71030817	13.08688159	14.77782982	14.13393956	15.84176888	17.66392483
tr|A0A0G2K0D7|A0A0G2K	Proteasome subunit alpha	Psma6	3	15	25.318	227	0	15.093	1.325562733	1.093683074	1.098160613	1.346143519	1.732159233	1.362538226
tr|B0BMW0|B0BMW0_RAT	Ras-related protein Rab-14	Rab14	9	57.7	23.897	215	0	100.76	10.29694321	10.13919925	7.909866572	6.470614882	8.873547752	6.257861417
tr|D3ZEV8|D3ZEV8_RAT S	Sushi domain containing 2	Susd2	4	7.3	89.67	820	0	210.05	3.315612704	3.716143377	3.495910383	4.702035891	5.120059703	3.749189889
tr|Q3KRC3|Q3KRC3_RAT	SRP receptor subunit alph	Srpra	8	15.1	69.656	636	0	34.433	2.251048356	2.338254728	3.059802034	3.966641013	2.737403734	3.734458098
tr|D3ZAC0|D3ZAC0_RAT I	Integrin subunit alpha 2b	Itga2b	6	9.2	112.64	1034	0	31.532	2.971729019	3.151384746	3.201817391	5.26476557	3.65943513	6.218644375
tr|D3ZYL4|D3ZYL4_RAT M	39S ribosomal protein L50	Mrpl50	4	40.3	18.319	159	0	59.189	2.424645898	2.565944953	2.462880162	2.735886344	2.537810928	2.859389741
sp|Q642G4|PEX14_RAT P	Peroxisomal membrane pr	Pex14	2	5.6	40.936	376	0	4.5518	2.150050711	2.298718662	2.36660244	2.349285918	2.659044278	2.832669321
sp|Q5M7T2|SPRY7_RAT S	SPRY domain-containing p	Spryd7	2	11.2	21.681	196	0	4.3485	0.251591004	0.254037731	0.149113837	0.128522394	0.15526821	0.116157628
tr|Q4KM57|Q4KM57_RAT	LEM domain containing 2	Lemd2	3	9.6	36.243	313	0	4.7143	1.258933387	1.284255795	1.147968897	1.266410541	1.478043778	1.400434487
tr|A0A0G2QC06|A0A0G2Q	Signal recognition particle	Tf	10	11	107.41	979	0	323.31	42.90367919	33.10861685	39.65132779	47.07769498	50.72278232	41.24494952
tr|G3V7I3|G3V7I3_RAT Ca	Cation-transporting ATPas	Atp13a1	5	6.1	131.91	1197	0	11.566	3.460611807	3.571523311	3.55106997	3.984200186	3.913011291	4.754913988
sp|Q66H47|RM24_RAT 39	39S ribosomal protein L24	Mrpl24	3	21.8	25	216	0	5.6759	1.824128852	1.989685819	1.899483976	2.1808612	2.150813367	2.69490168
sp|Q52KK3|S2551_RAT S	Mitochondrial nicotinamide	Slc25a51	3	11.4	33.774	298	0	6.6984	4.060125399	4.043685613	4.420496671	5.775056631	4.632483763	6.762704639
sp|Q9Z1Z9|PDLI7_RAT PI	PDZ and LIM domain prote	Pdlim7	3	7.7	49.912	457	0	5.3548	3.229566524	3.149509128	4.67347153	5.271753404	4.191287129	5.247870179
tr|Q5XIH3|Q5XIH3_RAT N	NADH dehydrogenase [ub	Ndufv1	30	66.4	50.73	464	0	323.31	524.1541965	515.8938301	445.3290556	460.0383809	432.3090646	413.2521238
tr|Q5FVL8|Q5FVL8_RAT A	ATP-binding cassette subf	Abcb10	6	8.7	77.393	715	0	9.8944	3.415807586	3.195906009	3.160653519	3.33480942	3.499911888	3.535528127
tr|D4A3D2|D4A3D2_RAT R	RCG56113, isoform CRA_	Smyd1	4	11.6	56.553	490	0	23.012	3.991740009	3.95429758	4.15288577	5.121664285	6.483934886	4.665202464
sp|P36506|MP2K2_RAT D	Dual specificity mitogen-ac	Map2k2	3	9.2	44.281	400	0	5.5426	0.660172386	0.578134752	0.50530939	1.071527613	1.233258113	0.671779808
tr|G3V6T7|G3V6T7_RAT P	Protein disulfide-isomerase	Pdia4	8	10.9	72.747	643	0	28.878	16.13955412	15.10465269	17.4082012	23.91750448	22.68864881	37.17897534
tr|M0RBK9|M0RBK9_RAT	PNKD metallo-beta-lactam	Pnkd	2	19.7	15.598	142	0	6.0765	0.663483784	0.568608584	0.580456324	0.840929089	0.631136072	0.797782336
sp|Q75Q41|TOM22_RAT I	Mitochondrial import recep	Tomm22	3	33.8	15.49	142	0	19.553	5.063930607	5.562196298	4.796963144	3.85202859	4.969704464	4.202116249
sp|Q5FVM7|DJC16_RAT D	DnaJ homolog subfamily C	Dnajc16	4	6.2	88.868	771	0	8.1864	0.608645023	0.578430903	0.593217124	0.736290754	0.637499745	0.833463749
sp|P23358|RL12_RAT 60S	60S ribosomal protein L12	Rpl12	7	50.3	17.845	165	0	300.1	9.832845748	8.881054042	9.471349426	11.35911246	12.40754304	9.82813625
sp|Q9ES40|PRAF3_RAT P	PRA1 family protein 3 (ADP	Arl6ip5	7	28.7	21.548	188	0	127.36	18.209178	20.13229769	15.51466312	11.05332007	17.10048169	11.78136848
sp|Q6TUG0|DJB11_RAT D	DnaJ homolog subfamily B	Dnajb11	3	11.2	40.495	358	0	18.24	2.092201588	1.990870421	1.908128389	3.649739753	2.280351848	3.92353301
sp|B1WBU5|SBK2_RAT S	Serine/threonine-protein ki	Sbk2	2	7.7	39.773	362	0.0015252	2.8392	0.398321065	0.336194761	0.37934337	0.400791104	0.424737373	0.497426527
tr|Q7TP77|Q7TP77_RAT A	39S ribosomal protein L49	Mrpl49	3	22.3	19.406	166	0	20.948	3.332270041	3.664810657	3.396339552	3.533275852	3.864366949	3.943344728
sp|Q6IRK9|CBPQ_RAT Ca	Carboxypeptidase Q (EC 3	Cpq	2	7	52.041	472	0	86.49	0.861465258	0.77186641	1.045699546	1.083233728	0.992301438	1.275366665
tr|P97949|P97949_RAT A	Antigen peptide transporte	Tap1	3	5.8	79.123	725	0	35.939	0.802311645	0.773791387	0.976910143	1.567365205	0.983079506	1.811095847
sp|P24473|GSTK1_RAT G	Glutathione S-transferase	Gstk1	8	38.9	25.493	226	0	94.552	6.256535685	6.323796164	6.567924366	7.178357049	7.162367065	8.750886768
tr|B2GV65|B2GV65_RAT	HIG1 domain family, memb	Higd2a	2	34.9	11.43	106	0	90.506	3.880155924	4.107456645	4.070649502	4.65897411	4.116702733	4.399014249
tr|O88321|O88321_RAT A	26S proteasome non-ATP	Psmd4	3	7.6	41.073	380	0	10.572	0.807128224	0.735341206	0.606206612	0.960379244	0.831861396	0.801297646
sp|D4ACN8|PLRKT_RAT P	Plasminogen receptor (KT)	Plgrkt	5	29.3	17.312	147	0	32.144	11.26577805	10.91560663	9.864693086	11.64101483	11.55276163	11.93173434
tr|A0A0G2K1W9|A0A0G2K	Lactate dehydrogenase D	Ldhd	6	19.5	51.842	482	0	64.294	4.138846365	3.878729894	4.107331086	5.184435341	4.543661998	6.346861752
tr|A0A0G2K911|A0A0G2K	NSFL1 cofactor p47 (XY b	Nsfl1c	8	31.2	37.249	340	0	74.944	6.818971652	5.778386021	7.021184327	8.240268924	7.30474075	10.25535818
tr|D3ZS58|D3ZS58_RAT N	NADH dehydrogenase [ub	Ndufa2	8	66	10.844	97	0	221.97	161.5912148	151.2933188	148.8394114	224.0287652	165.3422271	197.2332755
tr|D3ZY50|D3ZY50_RAT A	ATP synthase mitochondri	Atpaf1	3	13.8	38.649	348	0	31.092	1.421342417	1.225025734	1.491229847	1.924998969	1.471626176	2.134585648
sp|Q5XI31|PIGS_RAT GP	GPI transamidase compon	Pigs	3	9.7	61.724	555	0	20.356	2.380845131	2.720239262	2.462194097	3.023641767	2.74624816	3.611930241
tr|B2RYS8|B2RYS8_RAT	NADH dehydrogenase [ub	Ndufb8	9	58.6	21.959	186	0	323.31	44.62309762	42.39342248	41.75937538	58.4397937	44.99763446	55.23710256
sp|P02650|APOE_RAT Ap	Apolipoprotein E (Apo-E)	Apoe	10	34.9	35.753	312	0	27.293	10.51469273	10.51185505	9.654757342	10.63584178	12.24791194	13.26775534
sp|A4F267|TM40L_RAT M	Mitochondrial import recep	Tomm40l	2	7.5	34.048	308	0	17.965	0.53629599	0.567423983	0.492091213	0.387000584	0.51697287	0.402035643

tr|D3ZF99|D3ZF99_RAT M	Mitochondrial ribosomal pro	Mrpl55	2	11.8	15.062	127	0.0010599	3.321	0.517029673	0.532478247	0.485184831	0.644075234	0.54026499	0.713069461
sp|P62890|RL30_RAT 60S	60S ribosomal protein L30	Rpl30	4	42.6	12.784	115	0	16.212	9.067210337	9.005930754	10.82426867	12.44192812	10.97733466	10.3732125
tr|B2RYB8|B2RYB8_RAT	Integrin beta	Itgb2	3	6.9	84.817	768	0	7.8127	0.400192507	0.650049918	1.388000005	3.787644957	1.669870746	5.934981208
tr|Q641Z9|Q641Z9_RAT S	Succinate dehydrogenase	Sdhc	2	12.4	18.202	169	0	10.821	11.18098619	11.57355389	9.55230504	7.788150089	10.41970439	7.580877805
sp|P09495|TPM4_RAT Tro	Tropomyosin alpha-4 chain	Tpm4	10	33.9	28.509	248	0	40.623	6.970493205	5.408691724	5.69433554	8.162029073	10.75406684	6.969152903
sp|O35567|PUR9_RAT Bif	Bifunctional purine biosynt	Atic	5	12.7	64.208	592	0	11.705	2.219389383	2.297879569	2.577864576	3.080559766	2.917959452	4.341204671
sp|Q99P39|NFS1_RAT Cy	Cysteine desulfurase, mito	Nfs1	12	31.3	50.012	451	0	66.407	7.071842059	7.519256226	7.636355516	9.051932726	7.896885847	10.31936527
sp|P84100|RL19_RAT 60S	60S ribosomal protein L19	Rpl19	5	17.9	23.466	196	0	36.574	8.52986072	7.72804219	8.711189935	11.7311997	11.41901666	8.68677808
sp|P70550|RAB8B_RAT R	Ras-related protein Rab-8B	Rab8b	2	10.1	23.603	207	0	4.3891	2.375777688	2.677494901	2.439371018	2.599713168	2.983969069	3.280414155
tr|M0RCE9|M0RCE9_RAT	Voltage-dependent L-type	Cacna1s	34	20.3	210.41	1850	0	323.31	97.0540708	126.6042551	125.9568726	91.15838559	91.91731365	94.82396367
tr|R9PXU6|R9PXU6_RAT	Vinculin (Metavinculin)	Vcl	17	19.5	116.57	1066	0	140.21	13.95854686	12.45213313	12.72421002	15.19047618	18.4336171	14.3111725
tr|Q5U1Z9|Q5U1Z9_RAT M	Metaxin 2	Mtx2	11	37.3	29.718	263	0	206.43	16.9754313	16.12390332	17.68491389	21.21434748	17.45857307	21.95341584
sp|P63102|1433Z_RAT 14	14-3-3 protein zeta/delta (	Ywhaz	9	37.6	27.771	245	0	232.17	14.12963576	13.62636908	13.52416125	17.12915219	20.66359886	15.21438366
tr|A1L1J8|A1L1J8_RAT RA	RAB5B, member RAS onc	Rab5b	4	27.4	23.674	215	0	143.99	6.411569328	6.592799357	6.379942686	8.199655872	6.820454505	8.952153506
tr|A0A0G2JUS7|A0A0G2J	Transmembrane protein 11	Tmem11	3	18.8	19.834	176	0	6.9716	9.701894991	9.23297932	7.425505019	6.765656764	8.155747092	6.414729587
tr|D3ZIN7|D3ZIN7_RAT Mi	Mitochondrial ribosomal pro	Mrps23	6	41.8	20.321	177	0	109.67	5.835586732	5.249264144	5.270805041	6.091360641	5.791481057	7.009385931
tr|M0RAQ6|M0RAQ6_RAT	Hexokinase-1 (EC 2.7.1.1)	Hk1	5	6.9	99.556	893	0	18.336	2.926573589	2.253358307	2.170022086	2.91404625	3.066157514	3.088799279
sp|Q63151|ACSL3_RAT L	Long-chain-fatty-acid--CoA	Acsl3	13	23.2	80.457	720	0	77.645	6.097488226	6.519748949	7.494111471	7.178357049	7.620767186	8.3407334
tr|F6Q5K7|F6Q5K7_RAT M	28S ribosomal protein S18	Mrps18b	4	19.1	29.159	257	0	67.211	1.743902705	1.548273791	1.337094017	1.081979502	1.45997742	1.169907365
tr|Q5RJR9|Q5RJR9_RAT S	Serpin H1 (47 kDa heat sh	Serpinh1	17	50.6	46.561	417	0	323.31	32.61626879	18.96151682	23.06686139	47.33331832	56.70679143	28.1590556
tr|D4A264|D4A264_RAT Z	Zinc-binding alcohol dehyd	Zadh2	5	18.8	40.535	377	0	54.023	2.675960952	2.463328873	2.817438307	3.249701185	2.757950845	3.569462045
sp|Q5M9I5|QCR6_RAT Cy	Cytochrome b-c1 complex	Uqcrh	7	71.9	10.424	89	0	323.31	135.5867044	129.0968535	111.060125	99.93797196	117.9652264	105.9672932
sp|Q5BJT2|UBL3_RAT Ub	Ubiquitin-like protein 3 (Me	Ubl3	2	16.2	13.18	117	0.0015432	2.951	1.684247365	1.998965196	2.138142954	2.261609505	2.076822195	2.485202262
sp|P35435|ATPG_RAT AT	ATP synthase subunit gam	Atp5f1c	19	59.7	30.19	273	0	323.31	745.2151125	647.9275074	526.6688654	484.0060545	567.7689968	431.6617821
tr|D3ZE15|D3ZE15_RAT N	Complex I-B16.6 (NADH de	Ndufa13	14	65.3	16.776	144	0	277.91	188.7145764	195.0100395	179.6299871	261.5898664	203.7561573	219.351281
tr|D3ZQB6|D3ZQB6_RAT	Cat eye syndrome chromo	Hdhd5	5	21.2	46.476	419	0	85.282	2.084926547	1.754838628	2.114908236	3.076737361	2.2087875	3.702657751
tr|D3ZNJ0|D3ZNJ0_RAT C	Cytochrome c oxidase cop	Cox11	2	35.3	11.679	102	0	60.603	0.811292558	0.769349132	0.876836198	1.318968609	0.916638454	1.558826635
tr|Q32Q54|Q32Q54_RAT	RCG37273, isoform CRA_	Uqcc1	7	29.8	34.546	299	0	69.828	21.81458822	22.05826183	17.32861772	15.70470909	19.01982996	15.7183125
sp|D3ZS74|OMA1_RAT M	Metalloendopeptidase OM	Oma1	3	6.2	57.144	504	0	4.6607	3.559652717	3.546646686	3.404023474	3.200666896	2.968113818	3.431084812
tr|D3ZKK3|D3ZKK3_RAT C	Consortin, connexin sorting	Cnst	3	5.5	84.316	786	0	8.4437	2.29515016	2.850742829	2.315010388	1.911799727	2.241468732	2.079316033
tr|D3ZFQ8|D3ZFQ8_RAT C	Cytochrome c-1 (Cytochrom	Cyc1	14	39.3	35.434	326	0	323.31	398.0350806	426.0714428	307.1144968	293.0112296	307.5810882	280.60505
tr|G3V6G7|G3V6G7_RAT	Polynucleotide phosphoryl	Pnpt1	15	24	85.897	784	0	160.41	17.4435426	16.50643079	16.5071698	19.34614706	17.86358306	22.33441042
tr|A0A0G2K350|A0A0G2K	NSF attachment protein ga	Napg	8	24.4	34.659	312	0	22.621	9.642189478	9.913137852	10.06136492	16.80783127	10.62032186	16.81252894
tr|A0A0A0MY09|A0A0A0M	Endoplasmin (94 kDa gluc	Hsp90b1	16	26	92.899	804	0	194.6	27.25983134	25.1777117	27.99692105	28.28340764	35.66946164	31.0068631
tr|A0A0G2JUF1|A0A0G2J	Peroxisomal trans-2-enoyl-	Pecr	7	39.6	30.247	285	0	93.652	13.16732338	12.96101807	12.34641715	11.57233099	12.02140835	12.65917999
tr|F7FDM3|F7FDM3_RAT M	Mitochondrial translational	Mtif2	5	7.2	79.21	727	0	8.537	3.579420761	3.135491347	3.054267781	3.677392464	3.480389435	4.308286739
tr|A0A0G2K6E2|A0A0G2K	Thioredoxin reductase 2, r	Txnrd2	7	18.7	56.367	524	0	91.312	10.58443278	10.49408603	11.19154187	13.38319534	11.88065254	16.50062137
tr|D3ZZT9|D3ZZT9_RAT C	Collagen type XIV alpha 1	Col14a1	25	19.3	192.56	1794	0	323.31	25.28051833	15.56072416	18.51688147	29.43251813	38.78172882	22.6666377
tr|D4A559|D4A559_RAT D	Dematin actin-binding prot	Dmtn	3	11.9	35.69	320	0	8.7082	0.319273976	0.240543149	0.285622382	0.636370699	0.779603782	0.336748412
tr|E9PT90|E9PT90_RAT S	Spartin	Spart	2	3.8	85.865	796	0	19.099	0.84234946	0.749013478	0.733814614	1.229201817	1.575386392	0.892279152
tr|B0K031|B0K031_RAT 6	60S ribosomal protein L7	Rpl7	15	47.3	30.313	260	0	118.64	26.03561746	24.4417782	26.6151871	35.1607503	34.99156876	26.53957462
tr|D3ZFR9|D3ZFR9_RAT R	Retinol dehydrogenase 13	Rdh13	8	32.6	36.407	334	0	29.275	11.34404746	10.47730418	9.189148219	11.67028012	11.9879721	11.71532942
sp|Q5PQN9|RM38_RAT 3	39S ribosomal protein L38	Mrpl38	4	12.9	44.837	380	0	6.4157	5.196386535	5.478287046	6.229923244	6.624108331	5.841635423	7.184643439
sp|Q6AXS5|PAIRB_RAT P	Plasminogen activator inhi	Serbp1	6	18.4	44.754	407	0	47.439	7.841491277	5.48914589	8.221797242	12.89165795	11.10352952	7.706860014
tr|D3ZPF0|D3ZPF0_RAT F	Four and a half LIM domai	Fhl3	2	6.2	31.767	288	0	5.4866	0.720881353	0.60325817	0.820487432	0.829043799	0.805867413	0.93109996
sp|Q5U2X6|CCD47_RAT C	Coiled-coil domain-contain	Ccdc47	14	34.6	55.732	483	0	191.33	16.13503858	16.89093261	18.23239338	17.93723249	20.29094653	18.27859609
sp|P62718|RL18A_RAT 6	60S ribosomal protein L18	Rpl18a	5	30.1	20.732	176	0	57.53	19.70733482	17.39932396	19.35936871	23.54661175	23.9290256	19.15335965
tr|Q6PDV6|Q6PDV6_RAT	40S ribosomal protein S14	Rps14	6	39.1	16.273	151	0	43.919	4.3911147	3.658245992	3.879557664	4.499627604	4.816221317	4.173363858
sp|Q568Z6|IST1_RAT IST	IST1 homolog	Ist1	2	6	39.941	366	0.0005531	3.9783	3.831689096	4.287812181	4.087160789	4.537911378	4.91270106	6.219965156
sp|P81155|VDAC2_RAT V	Voltage-dependent anion-	Vdac2	18	64.4	31.745	295	0	323.31	309.701025	377.7890715	299.2521973	262.820203	296.4716264	264.3899205
sp|Q6AYD3|PA2G4_RAT	Proliferation-associated pro	Pa2g4	11	34.8	43.656	394	0	118.89	8.333685463	7.080953776	8.725368602	10.1514714	11.79166899	8.493740825
sp|Q5XIH7|PHB2_RAT Pro	Prohibitin-2 (B-cell recepto	Phb2	22	77.9	33.312	299	0	323.31	243.6837864	284.8965927	234.3870836	216.7423058	215.0435861	225.0408001
tr|A0A0G2JTY6|A0A0G2J	G-rich RNA sequence-bind	Grsf1	3	11.8	43.549	381	0	10.527	2.464181986	1.869498154	2.278648969	2.754878919	2.40325701	3.404161195
tr|D4A9Z8|D4A9Z8_RAT C	Chromatin-modifying prote	Chmp4bl1	5	21	25.109	224	0	8.0641	1.907365362	1.896793341	1.939641619	1.952651679	2.213964725	2.30110568
sp|P11232|THIO_RAT Thi	Thioredoxin (Trx)	Txn	4	31.4	11.673	105	0	9.1992	3.295593797	3.400002927	4.515173576	4.382387277	4.637984564	6.383030837
tr|D3ZUY0|D3ZUY0_RAT R	Retinol dehydrogenase 14	Rdh14	5	25.1	36.195	334	0	40.154	2.415113085	2.094671102	2.264012925	3.038991112	2.42304911	3.599128823
tr|A0A0G2JYK4|A0A0G2J	Transcription elongation fa	Tefm	5	21.9	38.434	338	0	17.691	5.032823533	4.627002996	4.699999358	5.729426672	5.089266001	6.702456695
sp|P13383|NUCL_RAT Nu	Nucleolin (Protein C23)	Ncl	6	9.1	77.146	713	0	109.61	3.954562038	2.641561998	3.823986438	5.766635395	6.636015868	3.715154373
tr|Q6AYF2|Q6AYF2_RAT	LIM and cysteine-rich dom	Lmcd1	5	14	41.085	365	0	14.952	1.973543153	1.697385469	1.60680885	2.531805755	3.233392664	1.965424053
tr|D3ZUY8|D3ZUY8_RAT A	AP-2 complex subunit alph	Ap2a1	8	8.8	107.67	977	0	18.292	4.540779864	4.509628759	4.940121941	4.721267366	5.506194393	5.619619293
sp|P19234|NDUV2_RAT N	NADH dehydrogenase [ub	Ndufv2	14	56.9	27.378	248	0	323.31	190.751588	170.3950134	157.5021194	216.4138178	183.2575824	193.3014114
sp|P11507|AT2A2_RAT S	Sarcoplasmic/endoplasmic	Atp2a2	30	31.2	114.77	1043	0	323.31	157.4017943	152.9764397	162.4143414	123.863838	134.8505297	154.439964
tr|F1LNF0|F1LNF0_RAT M	Myosin heavy chain 14	Myh14	2	1.5	228.91	2000	0	10.222	3.931281905	4.072116042	3.192761339	4.416609746	6.873844636	4.882623372
sp|Q6P6V0|G6PI_RAT Glu	Glucose-6-phosphate isom	Gpi	16	32.6	62.826	558	0	100.32	22.33939466	20.89685905	25.3871316	26.22767048	31.62852975	24.70368873
tr|G3V6U3|G3V6U3_RAT	GDP-Man:Man(1)GlcNAc(2	Alg2	2	8	47.318	415	0	17.668	0.445583748	0.534896808	0.505721029	0.594031592	0.50681796	0.610343161
tr|D3ZFC6|D3ZFC6_RAT In	Inter-alpha-trypsin inhibitor	Itih4	3	4.1	103.75	932	0	30.437	1.609389697	1.683268971	1.334349759	1.814925651	1.686211362	2.24553127
tr|D3Z900|D3Z900_RAT M	Mitochondrial amidoxime re	Mtarc2	5	15.4	38.239	338	0	9.8313	7.925279686	9.749268017	10.62027881	10.1126501	11.3397404	12.80751389

sp|Q5XI79|NDUF7_RAT P	Protein arginine methyltran	Ndufaf7	3	9.6	48.715	436	0	14.76	0.861766295	0.839339321	0.751972455	0.940669969	0.957463029	1.27435068
sp|P02454|CO1A1_RAT C	Collagen alpha-1(I) chain (	Col1a1	10	7.6	137.95	1453	0	106.31	5.260105864	3.523152095	3.617389541	15.25617376	9.433874488	5.95875527
sp|Q5EAJ6|IKIP_RAT Inhi	Inhibitor of nuclear factor k	Ikbip	3	7.2	42.36	373	0.0015283	2.85	0.599413246	0.504689477	0.518573304	0.961991821	0.956977664	0.570983881
tr|F1LPG5|F1LPG5_RAT N	Complex I-B15 (NADH deh	Ndufb4	8	54.3	15.064	129	0	183.12	96.21618671	101.7917954	105.6173464	132.380634	103.2424931	126.7137182
sp|Q8R431|MGLL_RAT M	Monoglyceride lipase (MGL	Mgll	6	21.1	33.499	303	0	31.131	5.210434891	5.78480261	5.410304827	5.072988347	5.171346587	5.041625111
tr|Q5U302|Q5U302_RAT C	Catenin (Cadherin associa	Ctnna1	5	7	100.24	908	0	21.674	2.021508254	1.662044866	1.423995524	2.132722788	2.26891881	1.860574344
tr|A0A0G2K6H7|A0A0G2K	Calpain small subunit 1 (C	Capns1	2	20	16.189	165	0	182.74	0.72956123	0.69215262	0.716068411	0.859861939	0.728802263	0.912487105
tr|D3ZVB7|D3ZVB7_RAT C	Mimecan (Osteoglycin)	Ogn	3	10.4	34.069	298	0	93.869	3.349479277	2.233911104	2.520280894	4.492042519	6.126382792	3.116637283
sp|F1LMZ8|PSD11_RAT 2	26S proteasome non-ATP	Psmd11	3	8.5	47.463	422	0	5.0277	1.660214642	1.438402028	1.489125916	1.669614538	2.286607662	1.830297974
sp|Q3KRD5|TOM34_RAT	Mitochondrial import recep	Tomm34	2	7.4	34.461	309	0	15.684	0.567503409	0.600148592	0.531562792	0.694901275	0.618624445	0.865812729
tr|A0A0H2UHH9|A0A0H2U	40S ribosomal protein S24	Rps24	5	35.9	15.197	131	0	67.762	5.275659401	3.377544862	4.526425034	5.727037669	6.680777291	4.833754467
tr|Q6P136|Q6P136_RAT H	Hypoxia up-regulated prote	Hyou1	8	10.8	111.22	998	0	40.551	2.535176356	2.563230242	3.001578025	3.198337618	2.895524811	3.878423251
sp|P19511|AT5F1_RAT A	ATP synthase F(0) comple	Atp5pb	22	53.1	28.868	256	0	323.31	430.1807295	408.9786344	287.8589525	267.6997418	338.3586116	220.052311
sp|Q5U3Z3|ISOC2_RAT Is	Isochorismatase domain-co	Isoc2	2	21.4	23.157	210	0	31.208	0.871048244	0.710316505	0.933230702	1.227410065	0.87187703	1.373206074
sp|Q5FWT1|FA98A_RAT F	Protein FAM98A	Fam98a	3	7.2	55.07	515	0	30.275	0.847316557	0.727197072	0.709116291	0.85657706	0.898949602	0.809384891
tr|Q3B8R7|Q3B8R7_RAT	Mitochondrial ribosomal pro	Mrpl47	4	19.8	29.616	252	0	13.892	5.08851523	5.193982753	5.667350335	6.106889161	5.559584525	7.018936195
tr|G3V9N7|G3V9N7_RAT	Protein kinase C and case	Pacsin3	17	47.2	48.598	424	0	323.31	48.82405778	48.28286486	53.7234259	55.62913156	56.2861419	70.68922685
tr|D3ZWW5|D3ZWW5_RAT	Solute carrier family 30 me	Slc30a9	5	9	62.684	567	0	45.142	6.845061456	8.00691706	7.601594913	7.909391996	8.099121195	9.1072945
tr|B2RZD6|B2RZD6_RAT N	NDUFA4, mitochondrial co	Ndufa4	8	56.1	9.3267	82	0	243.54	776.3221865	785.7361157	618.7387244	630.4579167	628.2238833	572.5383389
tr|G3V6N2|G3V6N2_RAT T	Transmembrane emp24 pr	Tmed4	3	11	26.117	227	0	74.25	2.883274549	3.132974069	3.212519997	3.750675134	3.844952355	5.586396565
tr|D3ZPU3|D3ZPU3_RAT V	Very-long-chain 3-oxoacyl-	Hsd17b12	4	18.6	32.129	290	0	22.436	2.875748644	2.809232428	2.714848792	3.175044838	3.756885603	2.989639089
tr|G3V6V5|G3V6V5_RAT	Protein ATP1B4 (X,K-ATP	Atp1b4	5	15.6	41.026	352	0	13.558	8.376833985	5.344032241	5.594627496	9.91197384	10.67748705	6.820514217
tr|Q6AYF0|Q6AYF0_RAT	Deoxyribonuclease-1-like 1	Dnase1l1	3	15	34.8	307	0	32.59	1.288234244	1.426556016	1.633519629	1.785839538	1.622304993	2.351904957
sp|P14562|LAMP1_RAT L	Lysosome-associated mem	Lamp1	7	17.4	43.969	407	0	65.084	9.416914055	11.25420515	10.60427064	7.493108207	8.568846495	10.14451415
sp|Q5XIG4|OCAD1_RAT C	OCIA domain-containing p	Ociad1	11	60.7	27.659	247	0	234.81	17.36276455	18.56220749	17.04733126	19.38437111	17.94016285	20.61536286
sp|Q63425|PRAX_RAT Pe	Periaxin	Prx	5	4.7	146.4	1383	0	323.31	128.8936662	121.8954652	159.656362	178.5720088	229.0220933	149.9899472
sp|Q6AY04|MAIP1_RAT m	m-AAA protease-interactin	Maip1	3	9.6	33.09	291	0	5.6334	3.855822165	3.890477189	2.25619179	1.83690448	2.502756801	2.215254901
tr|M0R3K2|M0R3K2_RAT	Glutamyl-tRNA(Gln) amido	Gatc	2	22.2	16.965	153	0	65.816	0.646374894	0.296199663	0.472195342	0.808558097	0.563616431	0.704108468
tr|G3V784|G3V784_RAT A	ADP-dependent glucokina	Adpgk	2	5.8	53.918	496	0	5.7981	0.953732854	0.899062965	1.104563882	1.429997527	1.14244096	1.98533737
tr|A0A0A0MY48|A0A0A0M	Dynamin-2 (EC 3.6.5.5)	Dnm2	2	3.5	97.253	860	0	51.1	0.264159265	0.212650726	0.274617907	0.310743605	0.330112802	0.259370952
sp|Q9Z269|VAPB_RAT Ve	Vesicle-associated membra	Vapb	8	47.7	26.916	243	0	144.32	14.07444579	13.42548046	19.0520118	18.38517057	18.09062595	21.43323123
sp|P11348|DHPR_RAT Dih	Dihydropteridine reductase	Qdpr	2	12	25.552	241	0	4.6508	0.205833502	0.169170925	0.210050088	0.215171536	0.213409525	0.240737777
tr|D3ZZ19|D3ZZ19_RAT C	Hyaluronoglucosaminidase	Cemip2	2	1.8	152.74	1371	0	4.5557	0.343020715	0.355400109	0.388124996	0.504641069	0.389898964	0.615595807
sp|P63329|PP2BA_RAT S	Serine/threonine-protein p	Ppp3ca	2	3.6	58.643	521	0.0015496	2.9856	0.535392881	0.487058662	0.50201628	0.780965112	0.857909309	0.531492523
tr|D4A6M0|D4A6M0_RAT	Mitsugumin 29 (Predicted)	Sypl2	8	34.1	29.28	264	0	323.31	22.37903109	32.18067923	30.49328116	18.8761107	19.15357493	26.47759951
tr|A0A0G2K047|A0A0G2K	Acyl-CoA synthetase short	Acss3	3	4	74.674	683	0	5.0461	0.828953349	1.302518397	1.005907803	0.637923551	0.761699213	0.866483279
sp|P97852|DHB4_RAT Pe	Peroxisomal multifunctiona	Hsd17b4	17	31.7	79.427	735	0	195.2	24.64382677	22.31689976	22.37896736	24.64435867	25.71031455	30.63196443
sp|P05065|ALDOA_RAT F	Fructose-bisphosphate ald	Aldoa	35	92.6	39.351	364	0	323.31	566.6003006	364.0723766	449.3722625	867.1483983	1032.155284	492.5904357
sp|P03889|NU1M_RAT NA	NADH-ubiquinone oxidored	Mtnd1	3	8.8	36.145	318	0	26.465	3.275324026	3.996795148	2.673364757	2.402023162	2.834746348	2.302020067
tr|Q5XIG9|Q5XIG9_RAT M	Mitochondrial 18 kDa prote	Mtfp1	6	34.3	18.366	166	0	35.787	9.40437088	8.095762152	7.36650347	9.538692106	9.014842848	10.06293051
tr|F1LQT3|F1LQT3_RAT R	Rho-associated protein kin	Rock2	4	3.2	159.44	1378	0	7.6054	0.990358925	0.661254271	0.727045444	1.437403437	1.374229633	0.813723149
sp|Q62636|RAP1B_RAT R	Ras-related protein Rap-1b	Rap1b	4	33.2	20.798	184	0	178.6	55.31038444	60.38011121	53.0602302	58.49533802	64.00344276	60.48060401
tr|G3V6L9|G3V6L9_RAT P	Peptidylprolyl isomerase (E	Fkbp3	7	21.9	25.178	224	0	29.58	4.857319429	5.139688531	5.716289605	5.85526741	5.499183568	5.95032259
tr|D3ZXF8|D3ZXF8_RAT M	Mitochondrial ribosomal pro	Mrpl43	4	25	17.575	156	0	21.27	3.955314629	4.121721218	4.008766482	5.507129934	4.456835622	7.041592673
sp|Q09073|ADT2_RAT AD	ADP/ATP translocase 2 (A	Slc25a5	6	21.1	32.901	298	0	34.435	38.23561119	35.64810571	34.42168662	37.68771832	42.26718762	38.05577087
tr|G3V913|G3V913_RAT H	Heat shock protein beta-1	Hspb1	6	47.6	22.807	206	0	94.415	19.28738932	15.43979611	16.77564971	20.29099778	19.14278905	26.61272558
tr|G3V8G5|G3V8G5_RAT	Golgi apparatus protein 1	Glg1	6	5	133.55	1171	0	39.802	2.097770758	2.023743104	1.899438238	2.021514694	2.245082004	2.265342988
tr|D3ZQI1|D3ZQI1_RAT G	Glutathione peroxidase	Gpx7	3	17.2	21.089	186	0	6.845	2.274278316	1.74560861	1.705968043	2.310882694	2.369658978	2.213832521
tr|D4A772|D4A772_RAT D	Dystrobrevin	Dtna	9	15.8	70.926	626	0	69.565	3.265991904	3.265303897	3.45360307	4.055750829	3.895592086	5.633335098
tr|A0A0G2K0Q7|A0A0G2K	Myosin light chain kinase,	Mylk	2	1	214	1952	0.001533	2.8985	0.563790629	0.573100197	0.498768908	0.840749914	0.897817084	0.561392977
tr|F1LX07|F1LX07_RAT S	Solute carrier family 25 me	Slc25a12	29	57.7	71.916	655	0	323.31	408.7670211	416.1504076	326.2648445	317.4089237	356.1283574	295.5298778
sp|P00388|NCPR_RAT NA	NADPH--cytochrome P450	Por	9	16.2	76.962	678	0	31.581	11.10923923	12.40771058	9.00619768	6.480768145	10.13064267	8.476164275
tr|D3ZUL3|D3ZUL3_RAT C	Collagen type VI alpha 1 c	Col6a1	20	25.5	108.8	1025	0	212.91	21.03590791	22.08590253	20.52613577	25.70268705	28.85062502	21.89448867
sp|O35303|DNM1L_RAT D	Dynamin-1-like protein (EC	Dnm1l	12	20.1	83.907	755	0	46.303	3.233931549	2.917771515	2.993345251	4.481710081	4.852246174	3.13868417
tr|A0A0H2UHI2|A0A0H2UI	Dual specificity mitogen-ac	Map2k1	3	7.9	40.96	368	0	8.1717	1.080569439	0.929517088	0.872811286	1.576562867	2.170120102	1.10275072
sp|Q505J9|ATAD1_RAT A	ATPase family AAA domai	Atad1	3	10.2	40.717	361	0	207.12	14.47331876	17.01136706	16.26796197	17.27488138	16.89878563	20.03320314
sp|Q68FU7|COQ6_RAT U	Ubiquinone biosynthesis m	Coq6	7	21.2	51.495	476	0	34.388	8.160589648	6.921526195	8.59410159	10.36409268	8.318613958	12.52303793
tr|D3ZBM3|D3ZBM3_RAT	Ferrochelatase (EC 4.99.1	Fech	17	46	47.551	422	0	315.9	30.96157315	36.80210473	37.7481848	40.28157843	38.09790369	50.04236843
tr|Q3V5X8|Q3V5X8_RAT	Endonuclease (EC 3.1.30.	Endog	5	24.8	32.267	294	0	48.55	2.787545037	4.047535567	2.794478015	4.211693006	3.861185112	4.185962078
tr|F1LPD0|F1LPD0_RAT C	Collagen alpha-1(XV) chai	LOC1083480	2	1.8	134.49	1312	0	34.013	3.272012628	1.639488085	1.84061964	3.44279236	4.055223187	2.639428869
sp|A0JPM9|EIF3J_RAT Eu	Eukaryotic translation initia	Eif3j	6	32.8	29.187	259	0	82.212	6.228438973	4.594821329	4.341004662	6.109875415	7.21360002	5.691652668
tr|E9PT53|E9PT53_RAT W	Wolfram syndrome 1 homo	Wfs1	4	6.4	100.56	890	0	13.372	1.346986476	1.31431505	1.123910901	1.294959127	1.600086069	1.807438299
sp|Q5XIF3|NDUS4_RAT N	NADH dehydrogenase [ub	Ndufs4	9	38.3	19.74	175	0	113.23	77.01509444	87.38901896	63.82229568	61.67211489	60.81621369	62.04826971
sp|Q6Q0N1|CNDP2_RAT C	Cytosolic non-specific dipe	Cndp2	3	8.8	52.693	475	0.0010793	3.6938	0.413292599	0.435489022	0.474162061	0.497790602	0.603847783	0.47050291
sp|P00159|CYB_RAT Cyto	Cytochrome b (Complex III	Mt-Cyb	2	4.2	43.012	380	0	4.2339	23.16423384	27.26606493	20.69856665	20.51795308	21.4957299	16.81760886
tr|G3V7P3|G3V7P3_RAT 3	39S ribosomal protein L3,	Mrpl3	6	20.1	38.492	348	0	26.342	6.842051094	6.533075713	7.627207989	9.563776639	7.506436803	11.96830982
tr|A0A0G2K2P6|A0A0G2K	HBS1-like protein	Hbs1l	3	4.9	74.799	679	0	4.3152	0.208863933	0.192833335	0.186778779	0.311585729	0.287233516	0.200636827

sp|Q5EB77|RAB18_RAT R	Ras-related protein Rab-18	Rab18		8	50.5	22.976	206	0	261.7	8.61013704	7.887963355	7.828910959	9.176758138	9.299590217	8.148610532
sp|P04466|MLRS_RAT My	Myosin regulatory light cha	Mylpf		25	95.3	18.969	169	0	323.31	656.2589155	448.1988064	745.0203342	455.451495	506.5105618	350.4337371
sp|Q6AY30|SCPDL_RAT S	Saccharopine dehydrogen	Sccpdh		9	21.2	47.088	429	0	36.752	12.93853587	12.99112668	13.60923325	18.25915065	14.20231433	22.81497159
sp|Q4QR80|RT25_RAT 28	28S ribosomal protein S25	Mrps25		5	38	19.775	171	0	13.435	3.026417262	2.716981608	3.576865996	5.203905716	3.191867006	4.523167684
sp|Q6Q7Y5|GNA13_RAT	Guanine nucleotide-bindin	Gna13		6	16.2	44.011	377	0	17.127	2.262939286	2.66865975	2.32406644	1.971226179	2.22566741	2.258535885
tr|Q5PQZ9|Q5PQZ9_RAT	NADH dehydrogenase [ub	Ndufc2		9	50.8	14.359	120	0	123.3	97.98728302	98.32190102	78.73733841	79.85840095	81.50354149	74.14662568
tr|A0A0G2KAX9|A0A0G2K	Melanoregulin	Mreg		3	15.7	22.996	197	0	32.381	0.995225676	1.244621012	1.21076667	0.815366756	1.137587312	0.823862685
sp|Q4V897|CC90B_RAT C	Coiled-coil domain-contain	Ccdc90b		3	14.8	29.8	256	0	17.833	0.516427601	0.591264083	0.497533992	0.684807737	0.547167956	0.774455308
sp|Q9EQX9|UBE2N_RAT	Ubiquitin-conjugating enzy	Ube2n		4	23.7	17.124	152	0	7.5955	2.740232181	2.359923058	2.274578319	3.033197779	3.620552014	2.56759869
tr|F1M9V7|F1M9V7_RAT A	Aminopeptidase (EC 3.4.1	Npepps		6	7.7	103.34	920	0	40.927	0.684556318	0.674827827	0.889002409	1.167386362	0.83240069	1.591846165
tr|F1LQN3|F1LQN3_RAT R	Reticulon-4 (Foocen) (Glut	Rtn4		5	5.6	126.4	1163	0	306.96	27.07920962	38.79618345	31.8210447	22.08394461	30.18483902	23.95490738
sp|Q9ER30|KLH41_RAT K	Kelch-like protein 41 (Kel-li	Klhl41		13	21.1	68.212	606	0	105.33	13.80250976	12.54097822	15.6774891	15.02444046	15.5866827	17.09192496
sp|P11762|LEG1_RAT Ga	Galectin-1 (Gal-1) (14 kDa	Lgals1		12	76.3	14.857	135	0	184.66	50.55902975	52.92205938	65.9994071	74.03520591	67.79468141	112.4594409
tr|D3ZTW8|D3ZTW8_RAT	Mitochondrial ribosomal pro	Mrpl27		2	14.2	15.823	148	0.0010593	3.3171	2.945538869	3.371621856	2.987902472	2.764793282	2.809938812	2.905109091
sp|P97519|HMGCL_RAT H	Hydroxymethylglutaryl-CoA	Hmgcl		8	24.9	34.191	325	0	47.144	11.97973557	11.24926931	12.69539531	13.98343236	12.44043999	16.33196777
sp|Q4V7C7|ARP3_RAT Ac	Actin-related protein 3 (Act	Actr3		6	23.2	47.357	418	0	64.201	7.211322165	6.840578445	6.692788109	7.714090993	9.317926222	12.52913384
sp|P24368|PPIB_RAT Pep	Peptidyl-prolyl cis-trans iso	Ppib		10	38.9	23.802	216	0	85.13	16.37536581	16.41956004	16.36126674	17.62307858	21.53617697	17.89455355
sp|P62982|RS27A_RAT U	Ubiquitin-40S ribosomal pr	Rps27a		6	50.6	17.951	156	0	323.31	18.06769098	20.06122161	20.7324125	20.11480881	23.17886729	26.29269014
tr|D3ZH23|D3ZH23_RAT S	39S ribosomal protein L41	Mrpl41		4	35.1	15.194	134	0	31.543	5.206421075	6.36180212	5.234672309	8.209809135	5.603806654	8.062150162
tr|D4A533|D4A533_RAT T	Transmembrane anterior p	Tapt1		6	13.8	69.564	608	0	96.208	3.990786728	4.766637864	4.460288414	3.140762644	4.347736394	3.564280518
tr|B5DFG4|B5DFG4_RAT	Heat shock 27kD protein fa	Hspb7		3	29.6	18.618	169	0	45.643	3.277882834	3.047238556	3.607327261	4.3229011	3.971470789	6.514600967
tr|Q5RJN0|Q5RJN0_RAT N	Complex I-20kD (NADH de	Ndufs7		10	46.3	23.945	218	0	165.73	107.8010631	106.693083	86.3663759	89.63539611	87.89957142	81.41600232
tr|Q6AYJ9|Q6AYJ9_RAT N	NAD(P)(+)--arginine ADP-ri	Art3		9	23.8	41.712	374	0	79.905	12.39817589	11.10316849	12.72009363	13.49488123	12.61301415	15.77622367
sp|P80431|COX7B_RAT C	Cytochrome c oxidase sub	Cox7b		3	21.2	8.9952	80	0	5.552	36.30446396	36.61503645	24.42115276	23.05268536	28.07727705	22.28361114
sp|B4F795|CTL2_RAT Cho	Choline transporter-like pro	Slc44a2		4	5.4	79.896	705	0	15.923	2.698488495	3.284849817	3.196374612	2.506542047	2.983375846	2.476566385
tr|F1LS40|F1LS40_RAT C	Collagen alpha-2(I) chain (	Col1a2		5	5.8	129.63	1372	0	10.935	4.326592608	2.232134202	2.497549289	9.257984243	7.447653729	3.148640828
sp|D3ZAW2|PISD_RAT Ph	Phosphatidylserine decarb	Pisd		3	8.6	45.823	406	0	4.8548	0.233077278	0.170952763	0.192262722	0.232372359	0.237532157	0.238014936
tr|A0A0G2JVP4|A0A0G2J	Immunoglobulin heavy con	Ighm		7	16.4	51.708	470	0	69.472	4.123644037	5.471376872	7.870989583	6.886301421	7.854820895	11.3505906
sp|P35213|1433B_RAT 14	14-3-3 protein beta/alpha	Ywhab		2	10.2	28.054	246	0	8.8014	2.219439555	2.805678624	2.871591667	2.712354664	3.224116803	3.63306274
sp|Q9EPJ3|RT26_RAT 28	28S ribosomal protein S26	Mrps26		3	18	23.349	200	0	14.228	2.919248374	2.61673473	2.683061136	3.227782081	2.85329807	3.834431077
tr|D3ZGM1|D3ZGM1_RAT	Pentatricopeptide repeat d	Ptcd3		10	23.4	77.89	687	0	75.276	3.912115934	3.424089818	3.588712044	7.524762498	3.705113354	8.163037527
sp|P22734|COMT_RAT Ca	Catechol O-methyltransfera	Comt		4	25.4	29.597	264	0	52.535	5.148220743	6.118465287	6.26422647	6.275313879	6.316753666	7.228534015
tr|B0BN63|B0BN63_RAT A	Activator of Hsp90 ATPase	Ahsa1		2	13.9	38.104	338	0	13.843	0.659720832	0.609872193	0.52076871	0.765675492	1.13677837	0.69692545
tr|Q6P9Y4|Q6P9Y4_RAT A	ADP/ATP translocase 1 (A	Slc25a4		14	30.5	32.904	298	0	323.31	5732.732697	6192.009279	4535.526823	4351.927487	5207.910738	3402.942012
sp|P27881|HXK2_RAT He	Hexokinase-2 (EC 2.7.1.1)	Hk2		3	4	102.54	917	0.001087	3.7765	0.440671841	0.573643139	0.525571162	0.646942038	0.526038407	0.628651221
sp|P04157|PTPRC_RAT R	Receptor-type tyrosine-pro	Ptprc		2	1.2	143.27	1273	0.0015175	2.8078	0.548638474	0.46114551	0.43506553	0.70212801	1.282765327	0.658582155
sp|P19357|GLUT4_RAT S	Solute carrier family 2, faci	Slc2a4		5	11.8	54.895	509	0	144.74	16.2895705	11.33564648	12.3551073	14.88826729	17.51681685	16.37870311
tr|A0A0G2K9B4|A0A0G2K	39S ribosomal protein L15	Mrpl15		8	30.5	33.652	295	0	21.001	5.349914997	5.611554682	5.955954811	9.536900354	5.836781775	11.52534012
sp|P83565|RM40_RAT 39	39S ribosomal protein L40	Mrpl40		2	12.1	24.397	206	0	4.8245	4.813970216	5.401287966	5.444608053	5.857357788	5.546102169	7.195514485
sp|Q7M0E7|RM14_RAT 39	39S ribosomal protein L14	Mrpl14		2	20	15.912	145	0	5.79	1.649477684	2.025816157	2.234740839	4.505540386	1.948631949	4.380116918
tr|A0A0G2JSI1|A0A0G2JS	4-trimethylaminobutyraldeh	Aldh9a1		13	33	54.05	497	0	108.57	9.283956401	9.339099846	9.776419451	10.73976341	10.09720643	13.22203599
sp|Q5M7W6|F234A_RAT P	Protein FAM234A (Protein	Fam234a		4	6.9	60.636	552	0	6.2924	3.376572535	3.470832208	3.223862931	3.718602767	3.786708575	4.966950173
sp|P62907|RL10A_RAT 60	60S ribosomal protein L10	Rpl10a		8	35	24.831	217	0	71.781	34.82737968	30.00891033	37.34889525	44.12190091	46.78970928	34.00402049
sp|Q66H12|NAGAB_RAT A	Alpha-N-acetylgalactosami	Naga		7	20.5	46.871	415	0	26.559	2.485304693	2.246793642	2.452223293	2.5471551	2.651494158	3.241806704
sp|P63045|VAMP2_RAT V	Vesicle-associated membra	Vamp2		2	14.7	12.691	116	0	18.609	0.84310205	0.855923738	1.366274628	1.675109245	1.096115583	2.286881882
sp|Q5XIK2|TMX2_RAT Th	Thioredoxin-related transm	Tmx2		4	14.6	33.865	295	0	17.046	5.883752524	6.044921294	6.352957482	11.93247321	6.716370712	16.52297305
tr|D4A6C5|D4A6C5_RAT R	Rho GTPase-activating pro	Arhgap1		7	15.5	50.622	439	0	28.948	2.048651685	2.131048231	2.236478869	2.359916982	2.297717124	2.927968766
tr|A0A0G2K7K2|A0A0G2K	Apoptosis-inducing factor 1	Aifm1		26	48.7	66.133	608	0	323.31	121.7641255	114.052418	101.1807958	125.1180646	116.0237671	134.0186543
sp|P04897|GNAI2_RAT G	Guanine nucleotide-bindin	Gnai2		12	46.5	40.499	355	0	217.27	24.56856772	26.39686379	25.59935423	26.54660239	33.85581505	28.7422313
tr|D3ZFG5|D3ZFG5_RAT F	Myelin P2 protein	Pmp2		7	44.7	14.952	132	0	34.752	22.64193604	14.84749551	19.49383735	34.97560256	50.21908148	20.82059194
sp|P02770|ALBU_RAT Se	Albumin	Alb		2	3.3	68.73	608	0	323.31	357.3099	313.6972098	362.6445593	363.4091783	367.6207181	427.8213567
sp|Q5XIA8|GHITM_RAT G	Growth hormone-inducible	Ghitm		5	12.1	37.178	346	0	13.967	3.53035186	4.446400669	3.019598653	2.542914619	2.807350199	3.261313626
sp|B4F7E8|NIBL1_RAT Ni	Protein Niban 2 (Meg-3) (N	Niban2		2	3.6	84.73	747	0	5.7845	2.114678958	1.046644534	1.567977598	0.91833279	1.276185937	0.777371186
tr|M0RD75|M0RD75_RAT 4	40S ribosomal protein S6	Rps6		9	30.4	28.421	247	0	30.157	20.57983807	14.73791989	19.46273576	23.91571273	28.98167353	18.83637216
sp|D3Z9R8|68MP_RAT 6.8	ATP synthase subunit ATP	Atp5mpl		2	13.3	6.9142	60	0.0015512	2.9998	101.4843202	104.0820244	85.91357332	90.0893067	89.50127537	68.58512076
tr|B1WBY7|B1WBY7_RAT	ER lipid raft-associated 1 (	Erlin1		2	5.5	39.266	348	0.00054735	3.8511	0.136309191	0.16816895	0.168520316	0.171052622	0.170524845	0.203796542
tr|D3ZJS3|D3ZJS3_RAT R	RCG43475 (Translocase o	Tomm6		2	56.8	7.8938	74	0	152.27	0.37884904	0.476915514	0.443549862	0.568021321	0.432735107	0.632826922
sp|P24051|RS27L_RAT 4	40S ribosomal protein S27	Rps27l		3	38.1	9.4771	84	0	14.837	1.01108025	0.802468608	1.005084526	1.113812968	1.228404465	0.962869829
sp|B0BNN3|CAH1_RAT Ca	Carbonic anhydrase 1 (EC	Ca1		2	8.4	28.299	261	0	4.3022	0.705177298	0.721718292	0.716891689	0.726137491	0.879319291	1.001903994
tr|D3ZF54|D3ZF54_RAT A	Anoctamin	Ano10		7	13.7	79.039	688	0	59.263	2.837466874	3.08559002	3.997880925	4.864010301	3.879628976	7.734291623
sp|P09527|RAB7A_RAT R	Ras-related protein Rab-7a	Rab7a		14	74.9	23.504	207	0	251.08	32.53448729	35.69400901	35.30442297	35.29692347	36.35760112	37.73573542
sp|P08461|ODP2_RAT Dil	Dihydrolipoyllysine-residue	Dlat		25	42.6	67.165	632	0	323.31	436.0960908	422.6114201	387.9283238	387.6396421	338.6875811	404.0371349
tr|B2RYT0|B2RYT0_RAT M	Mitochondrial ribosomal pro	Mrps21		3	37.9	10.6	87	0	4.9909	0.552702463	0.565301573	0.613753322	0.746384292	0.61630548	0.98177732
tr|D3ZZN4|D3ZZN4_RAT U	60S ribosomal protein L35	Rpl35a		5	40	12.623	110	0	11.928	3.708765981	3.560417675	4.815258198	5.907228227	5.824917301	3.973925893
tr|M0R7S5|M0R7S5_RAT	Perilipin 4	Plin4		14	65.1	141.26	1395	0	323.31	51.15608488	39.68858303	44.55120061	66.04996306	75.44726696	44.80902685
tr|D3ZG95|D3ZG95_RAT M	Mitochondrial calcium upta	Micu3		4	9.4	56.882	499	0	7.6448	2.223704235	2.252716648	2.310665313	5.335599512	2.368256813	7.181900278
tr|F1LN92|F1LN92_RAT A	AFG3-like matrix AAA pept	Afg3l2		36	40.8	89.35	802	0	308.35	65.06395731	65.51338315	57.8443868	76.64519179	62.69835065	84.26990574

tr|D4A7X1|D4A7X1_RAT M	Mitochondrial ribosomal pro	Mrps16	3	25.2	15.243	135	0	7.9828	3.188776119	3.003260236	2.832348776	3.173551711	3.20653581	3.822848842
tr|Q5XIW0|Q5XIW0_RAT M	Mitochondrial import inner	Timm23	3	16.7	21.873	209	0	13.097	4.45056935	4.37670663	3.856368683	6.596037544	4.195763271	7.611865364
tr|Q5XFW4|Q5XFW4_RAT	Mitochondrial ribosomal pro	Mrpl13	5	32.6	20.54	178	0	15.645	3.663209169	3.889490021	3.6502749	4.417923698	3.753272331	4.900911112
tr|A0A0G2JW34|A0A0G2J	CDGSH iron sulfur domain	Cisd3	6	43.3	14.379	127	0	13.792	16.41500225	17.27049858	14.87067722	12.05132611	12.21986863	16.39089493
tr|M0R5Q9|M0R5Q9_RAT	Glycerophosphodiester ph	Gde1	4	15.5	32.047	283	0	7.1191	2.942478335	3.190180436	3.206436892	3.270127161	3.356621403	4.013346133
sp|P15429|ENOB_RAT Be	Beta-enolase (EC 4.2.1.11	Eno3	23	49.8	47.013	434	0	323.31	249.9052012	228.504639	284.2182368	287.9644605	339.825492	264.501679
tr|D3ZZN3|D3ZZN3_RAT A	Acetyl-coenzyme A synthe	Acss1	8	16.6	74.891	682	0	127.42	9.109355405	10.72755119	10.86451778	6.895857433	7.591106002	10.34882885
sp|Q920L2|SDHA_RAT Su	Succinate dehydrogenase	Sdha	35	68.8	71.614	656	0	323.31	564.192011	550.3953405	518.9392051	648.7935154	538.7819303	644.7749117
tr|Q6IRJ7|Q6IRJ7_RAT An	Annexin	Anxa7	2	5.2	50.019	463	0	5.5498	0.126083995	0.115982331	0.166343204	0.164482863	0.14795538	0.183832426
tr|F1LQH9|F1LQH9_RAT B	BAG cochaperone 2	Bag2	2	7.5	23.805	212	0.0024802	2.5896	0.330763524	0.316974607	0.329772921	0.371806524	0.34859981	0.473510227
tr|D4A9P9|D4A9P9_RAT G	Gup1, glycerol uptake/tran	Hhatl	19	39	56.377	503	0	296.81	47.38560648	66.03164618	61.00485737	47.00244139	47.43146945	48.4899425
tr|Q6AYG2|Q6AYG2_RAT	DnaJ (Hsp40) homolog, su	Dnajc28	2	5.5	44.507	383	0	7.9241	1.450894137	1.387118667	1.337368443	1.517793391	1.448166876	1.794433684
tr|D3ZVD3|D3ZVD3_RAT 5	5'-nucleotidase, cytosolic I	Nt5c1a	2	7.7	40.53	362	0.00054615	3.8273	1.7025604	1.39496665	1.411417674	2.170050961	2.740855217	1.532004616
tr|F1LQC1|F1LQC1_RAT A	Peroxisomal acyl-coenzyme	Acox1	3	7.3	74.508	660	0	7.5935	0.46147846	0.405321178	0.46373388	0.467659301	0.472540416	0.560732587
tr|F1M5C9|F1M5C9_RAT U	Ubiquitin-conjugating enzy	Ube2d2	2	29.7	13.603	118	0	59.768	2.463529741	2.490870851	2.377305047	2.581138669	3.464102749	2.780955657
sp|Q6AYQ8|FAHD1_RAT A	Acylpyruvase FAHD1, mito	Fahd1	6	36.7	24.48	221	0	68.016	11.87236599	13.06269634	13.35584675	19.88785351	12.58820661	22.92774598
tr|D3ZXF9|D3ZXF9_RAT M	Mitochondrial ribosomal pro	Mrpl12	7	22.5	29.441	276	0	104.07	13.61988113	12.63229123	12.90944744	15.93703964	13.47103132	19.58312154
sp|Q8VHK0|ACOT8_RAT A	Acyl-coenzyme A thioester	Acot8	3	15	36.003	320	0	11.46	0.873506706	0.795114209	1.10557011	1.137344648	0.961507736	1.241839142
tr|D3ZLT6|D3ZLT6_RAT K	Kelch-like family member 3	Klhl31	5	9.1	70.197	634	0	16.806	4.037547684	4.166785423	4.97533992	5.401476272	4.97191557	4.555374426
tr|D3ZRG1|D3ZRG1_RAT	Transmembrane 131-like	Tmem131l	2	1.1	175.95	1596	0.0097448	2.0458	1.503324609	1.272113632	1.703178047	1.676124571	1.649485423	1.829993179
tr|M0R589|M0R589_RAT P	Presenilins-associated rho	Parl	4	13	42.13	377	0	21.553	3.445108443	3.206320628	2.449661985	1.96101319	2.984939799	2.194528795
tr|G3V6W6|G3V6W6_RAT	Proteasome 26S subunit,	Psmc6	2	10.2	45.796	403	0	27.542	0.146439059	0.147783938	0.132666584	0.185667348	0.139704178	0.180388235
tr|F1LP82|F1LP82_RAT R	Ras-related protein Rab-2A	Rab2a	10	51.9	22.804	206	0	157.02	20.5191291	23.73842122	21.55889157	19.20579313	21.74488385	18.97657817
sp|D4AAT7|NNRD_RAT AT	ATP-dependent (S)-NAD(P	Naxd	4	14	36.518	343	0	26.525	2.062700041	1.965154703	2.154517028	2.619004368	2.1204511	3.266291955
tr|D3ZJX5|D3ZJX5_RAT R	Mitochondrial import inner	Timm50	10	35.7	39.854	353	0	104.78	20.73386826	20.84207125	17.41186021	16.76781547	17.37821823	18.76728514
tr|D4A1H7|D4A1H7_RAT M	RING-type E3 ubiquitin tra	Mul1	4	12.5	39.78	352	0	14.933	2.141019625	2.358244873	2.289077149	2.692824563	2.739291264	2.228767508
tr|F1LNH3|F1LNH3_RAT C	Collagen type VI alpha 2 c	Col6a2	11	10.4	109.66	1027	0	64.021	14.79843785	12.77740488	12.01207504	16.61253027	18.83215555	13.10418167
sp|P54001|P4HA1_RAT P	Prolyl 4-hydroxylase subun	P4ha1	2	4.5	60.897	534	0.0072046	2.3006	0.388522337	0.341530403	0.291060587	0.402702307	0.34997501	0.467790228
sp|B2GV06|SCOT1_RAT S	Succinyl-CoA:3-ketoacid co	Oxct1	18	46.5	56.203	520	0	323.31	66.47381018	52.9763536	63.06305094	81.34555537	60.01266524	93.70129963
sp|P05508|NU4M_RAT NA	NADH-ubiquinone oxidored	Mtnd4	6	17.9	51.782	459	0	138.18	18.94119769	19.40969094	15.71728084	14.20202615	18.1041083	12.44379106
sp|Q62760|TOM20_RAT M	Mitochondrial import recep	Tomm20	2	17.2	16.284	145	0.0010864	3.7754	1.302583636	1.246299197	1.207153397	1.329539947	1.309298604	1.577215973
tr|D3ZQ57|D3ZQ57_RAT P	Plexin B2	Plxnb2	4	3.2	205.95	1841	0	41.887	1.33685159	1.463673521	1.371442981	1.508416554	1.599277128	2.156835731
sp|Q5HZA9|T126A_RAT T	Transmembrane protein 12	Tmem126a	5	25	21.657	196	0	40.434	13.77641995	12.9141276	11.35848424	14.99457792	12.64321463	15.43790048
sp|Q64232|TECR_RAT Ve	Very-long-chain enoyl-CoA	Tecr	12	30.2	36.122	308	0	150.7	41.93083054	57.00399774	58.91464746	41.01380788	42.61934677	45.60251156
tr|A0A0G2JWM7|A0A0G2J	Interferon, lambda 1	Ifnl1	2	14.6	21.459	192	0.0015121	2.7725	1.440658907	1.300198553	1.294740968	6.19110152	1.75971717	11.80575213
tr|Q99PV2|Q99PV2_RAT	Syntaxin binding protein 3	Stxbp3	13	21.2	68.02	593	0	72.359	10.67875746	12.69645713	13.38145983	24.74051605	11.2928218	24.51065147
tr|G3V7Y3|G3V7Y3_RAT A	ATP synthase subunit delt	Atp5f1d	6	41.1	17.563	168	0	323.31	157.3315525	121.3920097	130.8873897	169.5833847	148.349065	150.0509064
tr|A0A0G2K0X5|A0A0G2K	Phosphatidylserine lipase	Abhd16a	11	25.1	62.896	558	0	214.46	5.533547079	6.748278267	6.36439189	7.506844974	6.189480221	8.461229287
tr|F1LNC4|F1LNC4_RAT V	Vesicle transport through i	Vti1b	3	16.4	23.096	201	0	5.6269	0.835024245	0.841066864	1.077258514	1.28498504	0.928988292	1.676477762
tr|A0A0G2K9C8|A0A0G2K	Phosphorylase b kinase re	Phkb	17	16.8	124.06	1085	0	79.82	15.19179182	12.70089938	14.12561115	16.7493007	19.63840047	14.07749583
tr|D4A0T0|D4A0T0_RAT N	Complex I-PDSW (NADH d	Ndufb10	13	62.5	20.858	176	0	323.31	252.4389226	253.4454304	219.6870077	230.4312935	227.6091424	184.5842553
sp|Q5M9G9|FAKD4_RAT F	FAST kinase domain-conta	Tbrg4	3	3.5	71.18	629	0	4.491	2.872387073	2.63445439	2.498235354	2.878748729	2.809507376	2.89535563
sp|P12711|ADHX_RAT Alc	Alcohol dehydrogenase cla	Adh5	2	11.8	39.575	374	0	12.394	0.180340753	0.168751379	0.173272456	0.234480654	0.170082624	0.257877453
sp|P11951|CX6C2_RAT C	Cytochrome c oxidase sub	Cox6c2	9	78.9	8.4548	76	0	92.41	664.3868929	705.5781	514.9600309	543.850582	537.309657	498.3713935
sp|P12001|RL18_RAT 60S	60S ribosomal protein L18	Rpl18	5	28.7	21.658	188	0	43.896	15.61023214	14.62143411	17.20421135	22.45722634	21.51029085	15.27534279
tr|A0A0G2K104|A0A0G2K	Calcium-activated potassiu	Kcnma1	2	3.8	117.7	1044	0	5.5959	1.351401673	1.353653683	1.235373518	1.652174815	1.394614956	2.264631798
sp|P62815|VATB2_RAT V	V-type proton ATPase sub	Atp6v1b2	5	16.2	56.55	511	0	57.864	2.161138878	2.211206247	2.345746079	2.802599255	2.340968523	3.709464854
tr|F1M5V2|F1M5V2_RAT G	GLI pathogenesis-related	Glipr2	4	35.3	18.985	170	0	33.489	4.91551976	4.693538098	3.862954902	4.851826385	5.945719215	4.827048963
sp|Q5I0K8|RT07_RAT 28S	28S ribosomal protein S7,	Mrps7	4	21.5	28.197	242	0	14.231	8.957332124	8.672268077	8.993848518	14.88767004	9.007292728	20.64889039
tr|A0A0G2JSW0|A0A0G2J	Myosin regulatory light cha	Myl12b	4	27.9	19.779	172	0	9.4102	6.27409613	6.086875921	7.334944502	8.927107314	8.656751459	6.359053578
tr|Q4V8I6|Q4V8I6_RAT 60	60S ribosomal protein L11	Rpl11	6	30.5	19.024	167	0	63.659	13.6173725	11.26407682	16.96637565	21.32364437	18.66389575	13.91595412
tr|A0A0G2K4U4|A0A0G2K	Voltage-dependent L-type	Cacnb1	17	33.8	70.366	642	0	143.9	68.37033824	84.13136561	84.01546147	62.93231402	59.79155459	79.04570802
tr|G3V7N6|G3V7N6_RAT	Membrane-bound O-acyltra	Mboat7l1	2	2.5	53.379	473	0	4.7296	0.320914623	0.412912498	0.489850069	0.475423561	0.465001083	0.713323457
sp|P61983|1433G_RAT 14	14-3-3 protein gamma [Cle	Ywhag	7	25.5	28.302	247	0	43.596	18.07672207	15.07306332	14.66622999	16.26552757	20.62584826	18.18512542
tr|D3ZT90|D3ZT90_RAT G	Glutaryl-CoA dehydrogena	Gcdh	10	37.1	49.713	447	0	183.02	15.99154466	14.41610323	14.56697933	17.0186608	15.43729819	20.83278377
tr|G3V6V1|G3V6V1_RAT A	Aminopeptidase B (AP-B)	Rnpep	2	3.4	72.688	650	0	4.3416	0.454414144	0.368820653	0.394939903	0.344028391	0.369761716	0.373141014
tr|D4A5X1|D4A5X1_RAT S	Stromal interaction molecu	Stim2	2	5	83.495	743	0	5.3323	0.259618636	0.261402002	0.246685934	0.450751131	0.238567602	0.474373815
tr|B5DEN5|B5DEN5_RAT	Elongation factor 1-beta	Eef1b2	3	20.4	24.675	225	0	54.134	3.058828826	2.095855703	1.95084734	3.920115178	5.295222479	2.328130896
tr|Q6IRL2|Q6IRL2_RAT M	Mitofusin-2 (EC 3.6.5.-) (Mi	Mfn2	4	5.5	86.18	757	0	9.2401	1.17293738	1.193831235	0.761897521	0.565530785	1.030753119	0.664739028
sp|P29266|3HIDH_RAT 3-	3-hydroxyisobutyrate dehy	Hibadh	15	60.6	35.302	335	0	323.31	20.67215584	19.52370881	26.55664293	31.79404769	22.64820174	43.50856534
sp|Q5XIJ4|F210A_RAT Pr	Protein FAM210A	Fam210a	9	30.8	31.474	273	0	83.183	25.59911497	22.49853862	22.55780151	27.76200771	23.61138129	26.21039531
tr|A0A0G2KAA3|A0A0G2K	Complex I-B9 (NADH dehy	Ndufa3	3	38.5	10.17	91	0	242.93	13.36299691	14.17819582	13.34486972	18.0244311	13.27904256	17.25448265
tr|A0A0G2K513|A0A0G2K	Transmembrane protein 14	Tmem143	8	26.5	50.989	453	0	145.64	12.82313866	14.84749551	13.54108418	12.08238315	13.21540584	12.81259381
sp|P11240|COX5A_RAT C	Cytochrome c oxidase sub	Cox5a	11	63	16.129	146	0	323.31	483.895622	458.8651531	470.4115745	616.2433483	477.4695659	529.8364661
tr|B1WBY1|B1WBY1_RAT	Cul1 protein (Cullin 1) (Cull	Cul1	3	5.7	89.69	776	0.00055432	4.0175	1.079114431	1.286378205	0.996577326	1.031870162	0.818163322	1.027161394
tr|D3ZJY1|D3ZJY1_RAT M	39S ribosomal protein L28	Mrpl28	7	26.8	30.236	257	0	14.722	5.765846679	5.665848905	5.069102071	6.292036901	5.449568496	6.784649927
tr|E9PSJ4|E9PSJ4_RAT S	Sperm-associated antigen	Spag9	2	2	146	1320	0	4.4968	0.270882407	0.313805798	0.252979432	0.291822701	0.304987083	0.345252211

tr|F1LP46|F1LP46_RAT A	ATP-dependent RNA helic	Supv3l1	7	11.9	78.922	695	0	23.262	2.789602118	2.25266729	2.363309331	3.010561975	2.455137118	3.497530267
tr|D3ZE09|D3ZE09_RAT T	Talin 2	Tln2	4	3	153.27	1452	0	7.1933	14.84008119	13.3479878	15.59561873	23.24022211	23.89343218	13.67922949
sp|Q03344|ATIF1_RAT AT	ATPase inhibitor, mitochon	Atp5if1	3	26.2	12.248	107	0	6.8691	25.15257794	35.61997143	34.31328842	14.14110657	16.40802786	33.30197447
tr|G3V7V6|G3V7V6_RAT	All-trans-retinol 13,14-redu	Retsat	8	15.6	67.477	609	0	34.385	2.747707913	2.103407536	2.591219965	2.796268397	2.80540874	2.846994718
sp|Q5HZY0|UBXN4_RAT	UBX domain-containing pr	Ubxn4	4	11.5	56.393	506	0	23.769	1.920259746	2.23524378	2.015154454	2.324081936	2.122662207	2.883062205
sp|P45592|COF1_RAT Co	Cofilin-1 (Cofilin, non-musc	Cfl1	5	26.5	18.532	166	0	20.045	7.498811736	4.897338865	5.09059876	7.74275903	9.752058101	5.97145509
sp|P29419|ATP5I_RAT AT	ATP synthase subunit e, m	Atp5me	10	78.9	8.2545	71	0	213.4	424.4008344	387.5570957	346.7644524	466.0706137	402.7503462	417.0417499
sp|P31232|TAGL_RAT Tra	Transgelin (Smooth muscle	Tagln	2	9.5	22.603	201	0.00055006	3.9083	2.490121272	2.318906241	2.054580295	3.000050361	5.386956432	2.7779077
tr|A0A0H2UHR2|A0A0H2U	Myozenin 3	Myoz3	2	15.3	27.2	248	0	8.6932	4.565816042	5.292699522	5.349473773	5.059191854	5.884239669	5.844964888
sp|P97532|THTM_RAT 3-r	3-mercaptopyruvate sulfur	Mpst	11	35.7	32.94	297	0	107.68	22.69963464	19.74286004	22.25501837	25.857375	21.75351256	31.60731056
sp|Q99ML5|PCYOX_RAT	Prenylcysteine oxidase (EC	Pcyox1	12	36.3	56.287	504	0	273.3	14.34989392	17.53900819	15.17391774	12.79669508	14.89638605	14.34165207
sp|Q5EB62|S2546_RAT S	Solute carrier family 25 me	Slc25a46	5	21.5	46.21	418	0	24.55	5.077978963	4.589984208	4.953843231	5.062894808	5.159913549	6.134114377
tr|A0A0G2K478|A0A0G2K	Deoxyguanosine kinase (D	Dguok	6	28.2	32.145	277	0	41.539	4.891737901	4.925571861	5.370055709	5.456781694	5.132193824	6.185828042
tr|A0A0H2UHX5|A0A0H2U	Neprilysin (EC 3.4.24.11) (	Mme	3	3.5	78.668	686	0	4.35	0.444078567	0.454146492	0.418229507	0.514113466	0.779010558	0.494663046
sp|P02688|MBP_RAT Mye	Myelin basic protein (MBP)	Mbp	11	52.3	21.502	195	0	240.87	85.82542055	81.29819437	102.5712199	124.0907932	173.5826434	92.1488737
tr|F1LMJ8|F1LMJ8_RAT C	Calcium uptake protein 2,	Micu2	3	12.5	49.214	431	0	18.847	1.027637241	0.74407764	0.834940524	1.145108908	0.848795236	1.274858672
tr|M0R436|M0R436_RAT F	E3 ubiquitin-protein ligase	Rnf170	3	12.5	29.753	257	0	16.368	1.689816535	2.077839893	2.179123875	2.353944474	2.03125183	2.984152767
sp|Q920F5|DCMC_RAT M	Malonyl-CoA decarboxylas	Mlycd	10	26.2	54.761	492	0	127.72	13.58526197	14.07997263	14.41878939	14.03599043	14.74214789	15.58623437
tr|D4A465|D4A465_RAT L	Late endosomal/lysosoma	Lamtor2	3	31.2	13.48	125	0	6.1512	2.917341812	3.259380891	4.08075752	3.613307456	4.133960149	4.279331151
tr|A0A096MK75|A0A096M	Ras homolog family memb	Rhog	5	39.3	19.872	178	0	130.21	5.617335487	5.86821828	5.318372181	5.556940648	6.188940926	6.195276707
tr|A0A0G2JSM7|A0A0G2J	Alpha-adducin (Erythrocyte	Add1	5	7.2	80.338	735	0	18.662	1.957788925	1.888155623	1.687032662	2.027367751	2.914022604	2.05564357
tr|A0A0G2K9I3|A0A0G2K	Golgin subfamily A membe	Golga4	2	1.1	254.96	2213	0.0015075	2.7357	0.110716097	0.129610181	0.134990056	0.153881662	0.149853696	0.26861642
tr|Q66HL0|Q66HL0_RAT 5	5'-nucleotidase (5'-NT) (EC	Nt5e	4	8.9	63.95	576	0	40.087	5.450762124	4.73988562	5.619325819	6.843896616	9.163688064	5.368670861
sp|Q5RJQ7|SDHF2_RAT S	Succinate dehydrogenase	Sdhaf2	3	21.3	19.524	164	0	5.9397	0.145385433	0.154911288	0.207054273	0.234516489	0.16571434	0.293396308
sp|Q4G069|RMD1_RAT R	Regulator of microtubule d	Rmdn1	7	28.1	35.4	310	0	31.937	6.265566771	5.77443735	5.790841949	5.840933391	7.343030642	6.853025754
sp|Q5XIM7|SYK_RAT Lys	Lysine--tRNA ligase (EC 2.	Kars1	3	4.2	71.622	626	0	4.3291	0.822430898	0.620879113	0.793319277	0.922394095	0.758894882	1.197237376
tr|G3V985|G3V985_RAT S	Similar to SCO cytochrome	Sco1	3	12.7	31.768	284	0	14.829	6.569613333	7.442257147	6.813078088	7.915961754	7.080394338	9.730296842
tr|F2Z3Q8|F2Z3Q8_RAT Ir	Importin subunit beta-1 (Ka	Kpnb1	5	7.5	97.183	876	0	20.126	2.533771521	1.820485279	1.734462589	2.396886805	2.694314122	2.266866967
sp|Q2PQA9|KINH_RAT Ki	Kinesin-1 heavy chain (Co	Kif5b	11	14.3	109.53	963	0	37.268	6.778833492	7.33465587	6.048344834	7.433383129	6.857126514	7.877139192
tr|F1LPV2|F1LPV2_RAT V	Very low-density lipoprotei	Vldlr	2	2.3	96.472	873	0.0010834	3.7373	1.063510721	0.896496329	0.941051837	0.979073193	1.073734871	1.239197579
tr|B0BNE9|B0BNE9_RAT	LOC684274 protein (Mitoc	Mtif3	2	10.5	31.526	276	0	5.8245	0.067366884	0.117349558	0.09608105	0.087742111	0.062153663	0
sp|P61765|STXB1_RAT S	Syntaxin-binding protein 1	Stxbp1	2	3.9	67.568	594	0.0010701	3.5148	0.360184796	0.332048657	0.32557878	0.344870514	0.505582976	0.401090777
sp|P11608|ATP8_RAT AT	ATP synthase protein 8 (A	Mt-atp8	3	35.8	7.6298	67	0	11.944	150.538169	87.82830858	87.07988301	69.76486287	91.66384535	59.99089897
tr|D4A6G6|D4A6G6_RAT	40S ribosomal protein S19	Rps19	9	42.8	16.117	145	0	52.969	29.74337999	23.38600236	28.08656681	35.95389932	39.83820627	25.92998329
sp|B2RZD2|T126B_RAT C	Complex I assembly factor	Tmem126b	2	17.5	25.213	229	0	33.719	0.831461984	0.917424284	0.797298451	0.962947422	0.860282203	1.166554612
sp|P15791|KCC2D_RAT C	Calcium/calmodulin-depen	Camk2d	5	14.6	60.08	533	0	48.437	3.250839748	3.681789941	3.910796469	3.808011208	4.78025039	3.855258781
tr|B2RZD0|B2RZD0_RAT M	Mitochondrial ribosomal pr	Mrpl57	3	27.5	12.039	102	0	7.2211	3.307334209	3.317919934	2.980035599	3.906677036	3.17180526	4.699847571
tr|A0A0G2JYU2|A0A0G2J	39S ribosomal protein L11	Mrpl11	7	35.9	20.75	192	0	20.679	6.818469925	7.180164128	7.599765408	9.125991822	7.044261622	10.83243797
tr|D4A0T8|D4A0T8_RAT D	Dehydrogenase/reductase	Dhrs7	4	12.9	38.493	340	0	7.3659	2.442657898	2.785244253	2.453961323	2.656750617	2.754445432	3.373986424
tr|M0R8T0|M0R8T0_RAT S	Multiple inositol polyphosp	Minpp1	2	3.7	54.619	481	0.0010707	3.5215	1.08839638	1.047730418	1.12432254	1.153530144	1.145029573	1.445747442
tr|D4A742|D4A742_RAT R	Protein MGR2 homolog (R	Romo1	3	34.2	8.1828	79	0	26.33	6.270082314	6.986679262	5.067272566	4.922899227	5.674993497	4.995702564
tr|X1WI37|X1WI37_RAT 4	40S ribosomal protein S4,	Rps4x	12	44.7	29.466	262	0	114.53	24.90321963	18.46842656	30.68858086	38.53581442	32.24548239	24.99934053
tr|A0A0G2JWD2|A0A0G2J	Fragile X mental retardatio	Fxr1	2	3.9	63.389	563	0.009298	2.0594	0.196832519	0.20166355	0.185214552	0.194667917	0.281150276	0.230506802
tr|Q4G067|Q4G067_RAT	Mitochondrial ribosomal pr	Mrpl44	5	19.6	37.44	332	0	20.843	4.544241781	4.81624304	5.222323148	5.538903675	4.921114051	6.815434289
tr|Q5U2U3|Q5U2U3_RAT	Poly [ADP-ribose] polymera	Parp3	5	11.2	58.868	526	0	6.4804	2.971829364	3.889440663	2.885358695	4.039744508	4.016070424	3.337309346
sp|P04906|GSTP1_RAT G	Glutathione S-transferase	Gstp1	3	21.9	23.439	210	0	70.885	3.594472571	4.457111438	5.125816739	5.195424755	4.505210318	6.432915728
tr|Q6P7A7|Q6P7A7_RAT	Dolichyl-diphosphooligosac	Rpn1	26	45.9	68.4	606	0	323.31	57.88926121	61.70785174	58.24687799	60.53733842	67.93489792	60.99977262
sp|P09606|GLNA_RAT Gl	Glutamine synthetase (GS	Glul	3	13.9	42.267	373	0	19.13	1.200682883	1.175272483	1.18716605	1.201847732	1.296948766	1.514021672
sp|P12075|COX5B_RAT C	Cytochrome c oxidase sub	Cox5b	9	62	13.915	129	0	191.85	664.4872383	629.6649053	627.4746126	822.4143154	675.7357076	666.770999
sp|P04904|GSTA3_RAT G	Glutathione S-transferase	Gsta3	4	19.5	25.319	221	0	18.745	1.912934531	2.330900328	2.98872575	4.330366735	2.811179188	8.815909844
tr|F1M013|F1M013_RAT 6	60S ribosomal protein L7a	Rpl7a	13	38.5	29.864	265	0	174.82	15.94638923	14.67720908	16.95997238	17.77716928	18.4050345	15.7040887
tr|A0A140UHX6|A0A140U	Spectrin beta chain	Sptb	112	53.2	268.02	2329	0	323.31	159.3234087	175.9428958	159.4368214	208.6196952	269.5123064	164.0207077
sp|P18418|CALR_RAT Ca	Calreticulin (CALBP) (CRP	Calr	17	49.5	47.995	416	0	254.09	79.63912665	71.23401986	81.80175994	74.79371439	102.2501917	94.99261727
sp|P04638|APOA2_RAT A	Apolipoprotein A-II (Apo-A	Apoa2	2	19.6	11.439	102	0	4.2342	0.415836355	0.287487908	0.477592383	0.507364532	0.506656171	1.077046285
tr|B2RZ79|B2RZ79_RAT Ir	Iron-sulfur cluster assembl	Iscu	5	36.5	17.967	167	0	5.7588	3.282950276	3.179568384	3.690295331	3.901062878	3.382938963	4.707975456
tr|A0A0G2K757|A0A0G2K	Dolichyl-diphosphooligosac	Rpn2	9	19.5	69.122	630	0	87.928	15.38947226	16.05973742	14.74581348	13.01170536	14.96002277	15.01829845
sp|Q924S5|LONM_RAT Lo	Lon protease homolog, mi	Lonp1	31	34	105.79	950	0	323.31	45.39124165	41.98967089	44.9033804	50.39124228	44.74578404	63.11302257
tr|D4ABT9|D4ABT9_RAT S	Sarcospan (Similar to Sspn	Sspn	2	12	23.743	216	0.0010817	3.7042	0.480945467	0.433134628	0.562481433	0.622096406	0.641760169	0.475684436
tr|D4AE56|D4AE56_RAT F	Microsomal prostaglandin I	Ptges2	17	47.9	43.451	384	0	230.92	42.3091327	40.86035107	34.09374778	34.39746381	37.81207774	32.89253229
tr|M0R7G4|M0R7G4_RAT	MICOS complex subunit	Apoo	8	52	22.369	196	0	210.62	45.28638071	49.32778185	41.69031155	41.40082638	39.13874162	44.70438033
tr|D3ZI60|D3ZI60_RAT 60	60S ribosomal protein L27	Rpl27a	3	18.9	16.549	148	0	5.9238	7.286079488	6.525671955	6.840978046	5.994606015	6.880316167	4.434370546
sp|P56499|UCP3_RAT Mit	Mitochondrial uncoupling p	Ucp3	5	21.8	34.014	308	0	31.222	6.432641862	7.929424397	6.669004539	8.832144441	6.890562758	11.36887834
sp|P15865|H14_RAT Histo	Histone H1.4 (H1d)	H1-4	2	8.2	21.987	219	0	4.4683	6.473783476	5.877102789	8.129864596	9.784162174	14.83436721	6.798873724
tr|D3ZC63|D3ZC63_RAT C	Cytidine/uridine monophos	Cmpk2	2	6	50.113	447	0	4.5247	2.308546271	2.289439286	2.070039616	2.457388308	2.324466119	3.283462111
tr|A0A0A0MXZ0|A0A0A0M	Iron-sulfur cluster assembl	Isca1	4	36.4	14.122	129	0	10.048	2.484752793	2.679321161	2.589802099	2.773095067	2.655862442	3.405278779
sp|Q63357|MYO1D_RAT U	Unconventional myosin-Id	Myo1d	3	3.3	116.09	1006	0	5.2755	0.455864135	0.449181038	0.434649318	0.586362892	0.482290857	0.980558137
tr|D4A601|D4A601_RAT T	Translational activator of c	Taco1	3	9.8	32.812	295	0	4.6143	2.892506325	3.062440938	2.755372337	2.915360202	3.064647491	3.234694805

tr|M0R629|M0R629_RAT A	Adenylosuccinate syntheta	Adssl1	19	48.1	50.25	457	0	323.31	53.72492712	53.14417211	54.12591709	60.72845867	77.17840153	54.4608896
sp|P09895|RL5_RAT 60S	60S ribosomal protein L5	Rpl5	12	39.1	34.458	297	0	92.033	15.69301709	13.22459184	15.90892153	19.20579313	21.35982775	14.11711926
tr|B2RYV0|B2RYV0_RAT	DnaJ (Hsp40) homolog, su	Dnajc30	3	14.2	24.923	219	0	4.2152	1.470963217	1.292153136	1.049953146	1.550283833	1.288859352	1.577215973
tr|D4ADD7|D4ADD7_RAT	Glutaredoxin 5 (Glutaredox	Glrx5	3	29.6	16.432	152	0	81.699	9.466585028	7.627351087	8.638467096	9.995588948	8.970620719	14.47779414
sp|Q06000|LIPL_RAT Lipo	Lipoprotein lipase (LPL) (E	Lpl	4	8.2	53.082	474	0	7.3621	2.987132038	3.032085532	3.929228735	3.80568193	4.053821022	3.507385327
sp|Q05096|MYO1B_RAT U	Unconventional myosin-Ib	Myo1b	6	5.2	131.92	1136	0	26.431	1.21121915	0.982478634	1.081146213	1.466011749	1.48063239	1.020151094
tr|A0A0G2K079|A0A0G2K	Breast carcinoma-amplified	Bcas1	3	14.1	37.499	347	0	17.473	2.023163953	1.821817955	2.091764993	2.593442035	3.775329466	1.980867033
tr|Q5XIP0|Q5XIP0_RAT D	DnaJ (Hsp40) homolog, su	Dnajb4	3	9.2	37.822	337	0	5.0196	3.296296215	3.426310946	2.523985642	2.501345965	2.844237927	2.619820348
tr|Q52KS1|Q52KS1_RAT	ATP-dependent 6-phospho	Pfkm	39	49	85.342	780	0	323.31	290.1738102	184.6892014	238.9699946	405.670643	452.8076394	202.9227946
tr|F1M7X5|F1M7X5_RAT D	Dipeptidyl peptidase 4 (EC	Dpp4	2	2.7	88.053	767	0	5.0601	0.307036855	0.314536302	0.289075574	0.337584055	0.499246268	0.327340385
sp|P61023|CHP1_RAT Ca	Calcineurin B homologous	Chp1	3	21.5	22.432	195	0	28.774	1.569101019	1.503604453	1.503899172	1.531589884	1.822221375	1.614299446
sp|Q4V8H8|EHD2_RAT EI	EH domain-containing prot	Ehd2	28	63.2	61.237	543	0	323.31	87.26035977	80.59236947	75.43050741	93.70267388	98.16234118	78.72973651
tr|D3ZD23|D3ZD23_RAT A	ATP-binding cassette subf	Abce1	2	4	67.3	599	0	14.977	0.818718118	0.536229484	0.528681321	1.105869533	1.341764119	0.528048331
tr|Q2IBC6|Q2IBC6_RAT C	Caveolin-1	Cav1	4	20.2	20.551	178	0	11.101	24.29763514	23.23348496	24.13392041	29.43072637	38.11462182	23.46317037
tr|F1LT35|F1LT35_RAT Si	60S ribosomal protein L23	Rpl23a	11	52.6	17.734	156	0	55.711	31.13767932	27.36379453	29.82779857	36.25133021	41.78559784	28.13162399
sp|Q5XIN6|LETM1_RAT M	Mitochondrial proton/calciu	Letm1	26	44.1	83.059	739	0	323.31	125.3715427	127.3643742	107.1815735	131.4071152	123.2718819	134.5063274
tr|Q6P6Q5|Q6P6Q5_RAT	Amyloid-beta A4 protein (A	App	4	5.7	82.797	733	0	7.0863	0.792728659	0.925222909	1.105112733	0.828386823	0.86157651	0.725718481
tr|G3V6S3|G3V6S3_RAT	Calumenin	Calu	7	29.2	37.063	315	0	64.369	14.00721437	12.4590433	11.04655357	7.777996826	13.18304818	9.190605316
tr|Q6P6T6|Q6P6T6_RAT C	Cathepsin D (EC 3.4.23.5)	Ctsd	13	33.4	44.622	407	0	84.543	53.96575608	51.13528587	65.99483334	35.8595337	42.83830024	59.5530092
tr|G3V6R7|G3V6R7_RAT	3-oxoacyl-[acyl-carrier-prote	Oxsm	4	11.8	48.004	456	0	45.421	3.549718522	3.333072958	4.08016293	4.410696964	3.676584687	6.156669256
tr|D3ZDP2|D3ZDP2_RAT M	Mitochondrial ribosomal pro	Mrpl58	3	12.6	23.47	206	0	4.2773	2.034954538	1.921077666	1.613623758	1.518390642	1.650833659	1.810079862
tr|A0A0G2JV81|A0A0G2J	ADP-ribosylation factor 1	Arf1	3	21.5	20.568	181	0	7.9688	3.299758131	3.108393594	3.432426546	3.176537965	4.017796165	3.746548327
sp|P11598|PDIA3_RAT Pr	Protein disulfide-isomerase	Pdia3	23	48.5	56.623	505	0	186.06	47.48695533	45.97141174	44.06821119	44.8899654	56.30771367	50.91408404
tr|G3V9Z3|G3V9Z3_RAT A	Amine oxidase [flavin-conta	Maoa	15	34	59.52	526	0	152.32	12.0063271	15.24186899	14.8807395	19.08037047	13.26717809	25.82635277
tr|G3V9S0|G3V9S0_RAT	NADH-cytochrome b5 redu	Cyb5r1	13	52.5	34.232	305	0	212.21	83.33183736	112.4137197	86.87863741	64.36571588	70.14061144	96.85085484
sp|P35745|ACYP2_RAT A	Acylphosphatase-2 (EC 3.	Acyp2	6	58.8	10.863	97	0	47.005	6.510911274	6.954596312	8.679630967	8.293424243	7.846731481	12.31882483
tr|D3Z9D2|D3Z9D2_RAT F	FYVE and coiled-coil doma	Fyco1	9	7.2	163.55	1451	0	36.58	2.135149419	1.963328442	2.01698396	2.056334414	2.130967338	2.419874391
tr|D4A131|D4A131_RAT M	Mitochondrial ribosomal pro	Mrpl4	3	10.5	33.082	294	0	7.1699	3.348224959	3.333517183	3.056646138	3.931642118	3.127421342	4.765175442
tr|A0A0G2K6R8|A0A0G2K	EH domain-binding protein	Ehbp1l1	5	4.6	182.51	1699	0	7.6492	0.90571758	0.753505091	0.630721985	0.87234448	0.809103178	1.306963816
tr|B0BN68|B0BN68_RAT M	Mitochondrial ribosomal pro	Mrps9	9	26.9	45.01	390	0	315.2	9.524283633	9.111064112	8.58907045	10.71647063	8.829864917	13.04220655
tr|F1LZ05|F1LZ05_RAT Po	Poly [ADP-ribose] polymera	Parp14	2	1.2	199.53	1787	0.0010588	3.3147	10.6255744	11.48273447	7.66334072	5.493811242	10.88781181	3.825896798
sp|Q5XIM4|ATP5S_RAT A	ATP synthase subunit s, m	Dmac2l	3	15	23.324	200	0	5.9453	1.729352622	2.01441437	1.801331011	2.404232989	1.724501255	2.440295701
sp|P24329|THTR_RAT Th	Thiosulfate sulfurtransferas	Tst	7	26.6	33.406	297	0	147.4	21.06149599	17.27099216	19.00307253	22.90396992	18.30904012	25.27670458
tr|D3ZFQ3|D3ZFQ3_RAT N	Nucleotide-binding protein-	Nubpl	2	8.5	34.082	319	0	4.6538	1.891560961	2.15246977	1.843226685	2.388405844	1.995874126	3.541116048
sp|D3ZBN0|H15_RAT Hist	Histone H1.5	H1-5	2	8.6	22.649	222	0.002004	2.6883	1.332285875	0.833070806	0.993101266	1.472103707	2.334928428	1.031530132
sp|Q5XIF6|TBA4A_RAT T	Tubulin alpha-4A chain (Al	Tuba4a	4	8	49.924	448	0	42.168	18.44298278	16.75470347	18.84619244	22.31388616	25.25299301	17.02182196
tr|A0A0G2JVY2|A0A0G2J	CD163 molecule	Cd163	17	20.1	119.61	1111	0	129.83	22.7638557	28.71966934	28.96427203	27.61807028	29.51557484	38.56376365
sp|P13086|SUCA_RAT Su	Succinate--CoA ligase [AD	Suclg1	14	35.8	36.148	346	0	157.19	160.1061028	180.4098295	158.2339216	191.4845705	161.9500662	225.1931979
sp|P62845|RS15_RAT 40	40S ribosomal protein S15	Rps15	2	21.4	17.04	145	0	19.482	0.742555959	0.481377512	0.704634003	0.980745495	1.211794202	0.565223243
tr|D3ZXD2|D3ZXD2_RAT A	ATP-binding cassette, sub	Abca8a	26	16.9	183.96	1615	0	258.85	37.38919774	45.54396813	39.40297243	43.65186455	52.23010977	41.75700624
sp|Q58FK9|KAT3_RAT Ky	Kynurenine--oxoglutarate t	Kyat3	9	23.6	51.044	454	0	68.917	9.546861348	11.04245768	13.20354043	13.50264549	11.19359166	18.11502242
sp|Q5XFX0|TAGL2_RAT T	Transgelin-2	Tagln2	5	30.2	22.393	199	0	57.677	5.916364779	4.412047233	4.190344893	5.442985201	7.462753968	5.083178921
tr|A9UMV9|A9UMV9_RAT	Complex I-B14.5a (NADH d	Ndufa7	15	79.5	12.5	112	0	135.28	154.255966	153.2627183	125.8928399	134.3993416	126.0923909	132.6165943
sp|O35814|STIP1_RAT St	Stress-induced-phosphopr	Stip1	4	8.8	62.569	543	0	31.005	4.289013256	3.413724558	3.509540198	3.921369405	4.842323159	3.967626783
tr|G3V6I9|G3V6I9_RAT 60	60S ribosomal protein L26	Rpl26	8	32.4	17.258	145	0	15.548	14.29871776	11.21076977	12.84084099	16.11502038	18.12945513	12.00894924
tr|A0A0G2K398|A0A0G2K	Phospholipid hydroperoxid	Gpx4	8	39.8	22.074	196	0	20.94	6.180774908	6.26802119	6.163603673	8.194280615	5.758584107	9.954829649
sp|Q6AYC2|IRGM_RAT Im	Immunity-related GTPase f	Irgm	2	7.8	46.337	411	0	27.957	0.139675779	0.154116618	0.184418718	0.185524008	0.167833767	0.291211939
tr|D3ZUB0|D3ZUB0_RAT R	Reticulocalbin 1 (Reticuloc	Rcn1	4	16	38.09	325	0	32.026	1.7707451	1.761403293	1.680583655	1.596630493	2.332933039	2.392950774
sp|Q7TQ16|QCR8_RAT C	Cytochrome b-c1 complex	Uqcrq	7	57.3	9.8492	82	0	122.63	75.72063878	87.34459641	74.30536159	100.7681505	79.28164914	86.02756074
tr|A0A0G2K7X0|A0A0G2K	Acidic leucine-rich nuclear	Anp32a	5	23.6	26.316	229	0	12.421	0.333352436	0.262349683	0.243795315	0.301145785	0.304636542	0.357738673
tr|D4A565|D4A565_RAT N	Complex I-SGDH (NADH d	Ndufb5	11	37.6	21.664	189	0	125.19	138.1655811	141.3179894	124.0999247	130.5590191	120.4298012	129.050485
tr|A0A0G2JXI4|A0A0G2JX	Signal transducer and acti	Stat5b	2	2.4	86.974	758	0.0010667	3.4111	0.194338936	0.21918084	0.163781897	0.107505139	0.212007359	0.105388181
sp|P10888|COX41_RAT C	Cytochrome c oxidase sub	Cox4i1	17	70.4	19.514	169	0	323.31	1081.221684	1131.04737	857.2604901	971.8464589	897.709225	834.7946898
sp|P54921|SNAA_RAT Al	Alpha-soluble NSF attachm	Napa	9	42.7	33.192	295	0	76.465	6.177764546	6.744329596	7.049999037	6.556021743	7.317683812	7.567263598
sp|Q5XIT9|MCCB_RAT Me	Methylcrotonoyl-CoA carbo	Mccc2	24	56.1	61.516	563	0	323.31	48.73575383	49.0390353	49.2777278	55.74858172	47.8682978	65.78912852
tr|A0A0H2UH99|A0A0H2U	60S ribosomal protein L24	Rpl24	6	30.1	17.676	156	0	71.278	18.81175212	18.63180281	21.53739488	24.27227144	24.75576371	18.55697613
tr|Q68G41|Q68G41_RAT	Enoyl-CoA delta isomerase	Eci1	14	48.1	32.264	289	0	98.324	51.52234559	61.31298467	77.24171775	86.24301172	64.46184288	152.4181527
tr|F1LT49|F1LT49_RAT Le	Leucine-rich repeat-contain	Lrrc47	2	8.8	63.53	580	0.0024963	2.6573	0.030319363	0.026168334	0.015348178	0.028220099	0.032179688	0.074466661
tr|D4A568|D4A568_RAT C	Cytochrome c oxidase ass	Cox18	2	6.6	36.318	331	0	11.876	5.027806263	4.741859956	4.100241752	3.877172847	4.780196461	3.323085548
sp|Q505J8|SYFA_RAT Ph	Phenylalanine--tRNA ligase	Farsa	9	21.7	57.719	508	0	100.98	5.456782848	5.573055143	6.265141223	6.594245792	6.292485424	5.717865096
sp|Q68FP2|PON3_RAT Se	Serum paraoxonase/lactor	Pon3	2	7.3	39.458	354	0.0015144	2.7955	0.443943101	0.341332969	0.259936126	0.416988545	0.499413449	0.369981299
tr|A0A0G2JZH0|A0A0G2J	Calcium-binding protein 39	Cab39	2	5.6	39.873	341	0.0024618	2.54	2.108708407	1.941660112	2.18831714	2.153387664	2.686386497	2.152568592
sp|Q4V8B7|HSDL1_RAT I	Inactive hydroxysteroid de	Hsdl1	3	16.7	36.907	330	0	19.423	1.53548531	1.871225698	2.066014704	2.050361906	1.983092852	2.093743028
sp|P0C5I0|CDNF_RAT Ce	Cerebral dopamine neurot	Cdnf	4	23.5	21.361	187	0	16.585	6.541516621	8.565160384	7.978015648	8.00554937	8.244730645	10.67394422
sp|Q5FVJ3|PLPP7_RAT In	Inactive phospholipid phos	Plpp7	4	12.5	29.768	271	0	56.832	2.544107097	3.350101601	2.909691117	2.50504892	2.778390097	2.539455891
tr|A0A0G2K747|A0A0G2K	Single-stranded DNA-bindi	Ssbp1	3	27.7	17.17	148	0	37.285	1.111776858	1.4435353	1.748732731	2.37753588	1.384691942	4.774827305
tr|A0A0G2JVM0|A0A0G2J	Delta-1-pyrroline-5-carboxy	Aldh4a1	14	31	61.844	562	0	254.28	39.31382251	35.77643751	41.11950587	44.8111283	38.25052397	59.46360247

sp|Q3T1K5|CAZA2_RAT F	F-actin-capping protein sul	Capza2	4	27.6	32.967	286	0	121.71	7.314677927	6.531101377	11.22127134	11.66848837	9.472703675	10.06760405
tr|A1L1J4|A1L1J4_RAT Pr	[Pyruvate dehydrogenase	Pdp1	13	36.6	61.18	538	0	118.35	21.57175235	18.97780508	20.20780183	23.91511548	19.89240807	32.39368338
sp|Q9JJ31|CUL5_RAT Cu	Cullin-5 (CUL-5) (Vasopres	Cul5	3	4.1	90.889	780	0	9.9001	0.636139663	0.588203863	0.578809769	0.677939353	0.742014972	0.580391907
tr|D4ABM5|D4ABM5_RAT	Mitochondrial ribosomal pr	Mrps34	6	24.3	25.799	218	0	12.142	8.66030974	7.108594471	8.48890503	9.278290769	7.972926338	12.026221
sp|P08733|MLRV_RAT My	Myosin regulatory light cha	Myl2	10	63.9	18.88	166	0	197.21	34.87905756	22.10219079	37.61920467	25.18845413	21.30266256	28.03510536
tr|Q99ME0|Q99ME0_RAT	CXC chemokine RTCK1 (C	Ppbp	2	27.9	12.159	111	0	9.5729	0.946708676	1.088105576	0.929434478	0.791894801	1.020075093	0.839214227
tr|D3ZJK0|D3ZJK0_RAT N	NAD(P)(+)--arginine ADP-ri	Art1	2	7.2	35.614	321	0	10.048	1.603920872	1.854986789	1.865821076	1.402344817	1.357942946	1.868702228
sp|B1WBW4|ARM10_RAT	Armadillo repeat-containing	Armc10	2	10.5	33.421	306	0	7.0092	0.189336718	0.243677406	0.222184283	0.472867328	0.149530119	0.696498736
tr|A0A0G2K3S4|A0A0G2K	ATP-binding cassette subf	Abca9	4	3.6	183.23	1623	0	57.793	1.284772328	2.073644431	1.89765447	1.912635878	2.512194451	1.943478765
tr|M0R7B4|M0R7B4_RAT	H1.3 linker histone, cluster	Hist1h1d	3	15.5	22.233	220	0	10.281	6.061865609	4.460369091	5.548889861	9.017889432	15.81588276	4.705130696
sp|P62282|RS11_RAT 40	40S ribosomal protein S11	Rps11	7	39.2	18.431	158	0	35.74	27.23474499	20.54098511	25.61261814	34.06121162	40.4556982	21.730915
sp|B0BN56|RT31_RAT 28	28S ribosomal protein S31	Mrps31	7	28.2	43.961	387	0	40.176	2.167059257	1.771768554	1.754541411	2.183250203	1.919240412	3.162356633
sp|Q6AYE2|SHLB1_RAT	Endophilin-B1 (SH3 domai	Sh3glb1	9	31	40.787	365	0	64.168	9.361222358	8.034557755	9.042330411	10.29899234	12.05322671	8.430851319
tr|B2RYS2|B2RYS2_RAT	Cytochrome b-c1 complex	Uqcrb	18	83.8	13.558	111	0	323.31	554.006953	640.1782411	487.51745	524.1890865	485.2893327	489.3189622
tr|A0A096MJS3|A0A096M	Muscular LMNA-interacting	Mlip	3	3.7	82.267	774	0.0010828	3.7338	0.560780267	0.598815915	0.585258776	0.625381285	0.790928962	0.58725997
sp|P53987|MOT1_RAT Mc	Monocarboxylate transport	Slc16a1	6	9.7	53.238	494	0	67.315	9.512242185	12.20435404	10.89333249	8.20622563	10.91261935	9.565503985
sp|Q5XIH4|LIAS_RAT Lipo	Lipoyl synthase, mitochond	Lias	3	13.7	41.874	373	0	84.099	1.969730028	1.780702421	1.568938089	2.160853299	1.691873952	2.827690992
sp|Q01129|PGS2_RAT De	Decorin (Bone proteoglyca	Dcn	9	27.1	39.805	354	0	111.5	30.29477796	32.47534878	33.7122959	36.99729643	43.75779695	30.96012777
tr|A0A0G2K466|A0A0G2K	Oxidoreductase NAD-bindi	Oxnad1	3	9.7	34.847	310	0	5.8835	1.919256292	1.838994673	1.863214031	2.042000395	1.830688295	2.261482243
tr|F1LQC7|F1LQC7_RAT N	Nitric oxide synthase, endo	Nos3	2	1.4	133.19	1202	0.0048567	2.3924	11.77151886	18.491625	23.14964651	19.29179724	28.94176575	20.98619759
tr|A0A0G2K9N9|A0A0G2K	Sarcoplasmic/endoplasmic	Atp2a3	4	4.3	107.61	985	0	323.31	729.2100212	991.5605772	921.8420306	693.7067736	880.9911029	715.4671865
tr|D3ZL85|D3ZL85_RAT C	Cytochrome c heme lyase	Hccs	8	39.8	31.129	274	0	67.429	9.226759523	9.023206188	7.842174873	7.859222931	7.96807269	8.477078662
sp|Q5XIC2|ECSIT_RAT Ev	Evolutionarily conserved si	Ecsit	7	22.1	49.619	434	0	57.814	5.229500517	4.572116473	4.093106681	5.307469	4.845774643	5.100552273
tr|A0A140TAE6|A0A140T	Enoyl-[acyl-carrier-protein]	Mecr	7	28.4	40.334	373	0	107.72	5.633892478	5.935345682	6.77786011	7.876543203	5.78177376	11.39529397
tr|A0A0G2JVK4|A0A0G2J	Neurolysin, mitochondrial (	Nln	7	11.2	77.801	681	0	27.655	4.788382139	4.493291133	4.87883351	5.512982992	4.794865265	8.47159234
tr|Q6TXE9|Q6TXE9_RAT	Prolyl-tRNA synthetase (E	Eprs	10	8.1	166.78	1486	0	21.612	110.3147154	93.70689212	83.56265889	105.3192014	108.0745697	106.0587319
sp|F1LMY4|RYR1_RAT Ry	Ryanodine receptor 1 (RYI	Ryr1	216	47.2	565.48	5035	0	323.31	1159.440923	1480.208579	1670.887277	1184.049659	1200.253305	1318.444453
tr|M0R9L0|M0R9L0_RAT	Nascent polypeptide-asso	Naca	51	31.6	220.19	2163	0	323.31	47.21752793	41.75521857	61.17866039	62.86064393	59.4248345	52.0489399
tr|A0A0G2K654|A0A0G2K	H1.2 linker histone, cluster	Hist1h1c	3	16	21.316	212	0	60.756	31.7096481	29.90871281	35.00255458	43.17107767	70.12982555	30.60961275
tr|D3ZT98|D3ZT98_RAT B	BolA family member 3 (Sim	Bola3	3	40	12.297	110	0	45.232	6.150169561	5.516293001	5.987056403	6.827770845	5.791481057	9.487171499
tr|B2GV73|B2GV73_RAT	Actin-related protein 2/3 co	Arpc3	2	12.4	20.535	178	0.0015314	2.88	0.6844058	0.669250329	0.612289718	0.747996869	1.139852348	0.667167233
tr|D3ZEH3|D3ZEH3_RAT	SEC61 translocon subunit	Sec61a2	5	15.3	52.247	476	0	18.773	1.965916903	2.221571508	2.238948702	1.86419884	2.060751226	2.10065173
tr|Q6P2A5|Q6P2A5_RAT	GTP:AMP phosphotransfe	Ak3	11	52.4	25.494	227	0	129.26	18.49315548	15.0246921	15.75935947	18.4126441	16.08337271	21.15586718
sp|Q03346|MPPB_RAT M	Mitochondrial-processing p	Pmpcb	12	31.7	54.265	489	0	104.92	15.83350065	13.71027834	14.65388083	16.25238805	14.43960381	19.56076986
tr|B1H271|B1H271_RAT M	Mitochondrial coenzyme A	Slc25a42	8	34	35.185	318	0	323.31	13.45079913	16.5977438	13.44686465	12.47955492	12.25006911	14.34470003
sp|Q9R1T1|BAF_RAT Bar	Barrier-to-autointegration f	Banf1	3	41.6	10.044	89	0	9.5147	7.017153816	7.782829997	6.971330305	7.547458027	11.2836538	7.782042944
sp|Q9Z2Q7|STX8_RAT Sy	Syntaxin-8	Stx8	6	33.9	26.91	236	0	72.913	2.70285352	3.380012781	3.078691678	2.460673188	2.905555684	2.92837516
tr|D3ZTC5|D3ZTC5_RAT M	Myotilin (Predicted) (Myotili	Myot	8	19.8	55.122	496	0	48.308	8.946795857	7.416097203	9.55733618	8.918148553	9.66577102	9.913580635
tr|G3V7I0|G3V7I0_RAT Pe	Thioredoxin-dependent pe	Prdx3	12	46.7	28.299	257	0	323.31	111.142565	94.90630085	120.7885199	131.0845998	102.4335517	153.4036587
tr|F7FL53|F7FL53_RAT So	Solute carrier family 43 me	Slc43a1	3	7.1	67.146	606	0	45.312	2.867721012	3.526853974	2.966497259	3.607753023	4.18271235	2.994922214
tr|Q6AYI1|Q6AYI1_RAT D	DEAD box protein 5 (EC 3	Ddx5	2	2.9	69.238	615	0.0015593	3.0567	0.185664076	0.172621077	0.181770508	0.208613723	0.251235624	0.171782837
tr|G3V679|G3V679_RAT T	Transferrin receptor protein	Tfrc	19	27.3	85.875	761	0	71.338	25.6312255	13.03851073	14.28889451	23.69413269	30.65833938	17.54911846
tr|A0A0H2UHM3|A0A0H2U	Haptoglobin (Liver regener	Hp	2	6.6	38.432	346	0.0010477	3.1687	0.445232539	0.474867141	0.571994862	0.58823826	0.507993621	0.999882182
sp|Q64057|AL7A1_RAT A	Alpha-aminoadipic semiald	Aldh7a1	8	19.9	58.748	539	0	63.329	7.160146011	6.454102298	7.08841865	7.455481408	6.894877112	9.599234705
tr|G3V778|G3V778_RAT F	[Pyruvate dehydrogenase	Pdk4	10	28.6	46.609	412	0	104.09	23.47680977	25.13476991	17.66341721	15.15703013	19.62491812	21.42307138
sp|P20171|RASH_RAT GT	GTPase HRas (EC 3.6.5.2	Hras	3	19.6	21.298	189	0	35.704	6.767293771	7.874636591	8.223626747	7.865195439	7.888796433	9.761081204
sp|P31399|ATP5H_RAT A	ATP synthase subunit d, m	Atp5pd	21	88.2	18.763	161	0	323.31	992.8675595	911.0076944	684.0978046	783.7721903	802.0384233	623.307137
tr|F1LMM8|F1LMM8_RAT	[Pyruvate dehydrogenase	Pdk2	11	34.6	46.239	407	0	163.8	31.39255664	31.49706561	34.24559672	39.01242054	30.88484297	55.14261591
tr|F1M0U5|F1M0U5_RAT N	NBAS subunit of NRZ teth	Nbas	2	0.8	265.8	2357	0.0089076	2.1084	0.531629929	0.545656936	0.528406896	0.508941274	0.609941808	0.66949384
sp|Q4G064|COQ5_RAT 2	2-methoxy-6-polyprenyl-1,4	Coq5	11	42.2	37.3	327	0	168.39	14.09702351	14.04937044	17.86054641	17.43016658	14.99345901	22.64631798
sp|Q6AXT0|RM37_RAT 39	39S ribosomal protein L37	Mrpl37	9	28.6	48.365	423	0	55.64	3.545002288	3.495165891	3.168017278	3.636361336	3.380188562	4.457535017
tr|G3V886|G3V886_RAT A	ATP-dependent zinc meta	Yme1l1	4	7.6	79.795	715	0	16.599	1.773002871	1.769596785	1.642667156	1.890179249	1.7408958	2.659850179
tr|A0A0H2UHR5|A0A0H2U	[3-methyl-2-oxobutanoate	Bckdk	7	24	46.213	409	0	217.02	11.40124434	14.58342815	13.25293708	14.60517042	12.82549609	18.39645041
tr|D4A7U8|D4A7U8_RAT M	Myozenin 1 (Similar to myo	Myoz1	8	36.5	31.398	296	0	58.765	10.79014085	9.480758408	11.85931134	11.08019635	11.23349943	12.80344994
sp|P10860|DHE3_RAT Glu	Glutamate dehydrogenase	Glud1	26	48	61.415	558	0	323.31	94.63072939	85.57263043	101.930893	99.39447376	95.39036869	107.5014314
sp|P84850|D2HDH_RAT D	D-2-hydroxyglutarate dehy	D2hgdh	5	12.9	58.804	535	0	45.398	4.284648231	4.492155891	4.416517497	4.539523955	4.643431436	6.593746241
tr|Q6IRK4|Q6IRK4_RAT S	Solute carrier family 37 (Gl	Slc37a4	4	10.7	46.223	429	0	8.5592	2.892104944	2.714513689	2.615277961	2.657765943	3.125156307	2.998274967
tr|D4A9Z6|D4A9Z6_RAT M	Mitochondrial ribosomal pr	Mrps35	8	38.1	36.206	320	0	64.101	9.788192035	9.37562505	7.599765408	7.428605123	8.290031363	8.418253098
sp|Q68FV8|UBTD1_RAT U	Ubiquitin domain-containin	Ubtd1	2	7.9	25.958	227	0.002005	2.6889	1.3197427	1.495410962	1.521279473	1.523765899	1.446441134	1.674750586
sp|Q7M730|SCN4B_RAT S	Sodium channel subunit b	Scn4b	6	22.8	25.247	228	0	35.657	3.35846019	3.805087185	4.304140128	4.442172079	4.082349688	4.012330147
tr|D3ZEH2|D3ZEH2_RAT F	FAD-dependent oxidoredu	Foxred1	4	11.3	54.229	487	0	43.09	4.38218396	2.460466086	2.450759689	4.746769974	3.644065243	3.487167215
sp|Q99MZ8|LASP1_RAT L	LIM and SH3 domain prote	Lasp1	3	10.6	29.97	263	0	5.0972	0.665540865	0.443470273	0.422835287	0.675192	0.778633052	0.472443442
sp|Q6P791|LTOR1_RAT R	Ragulator complex protein	Lamtor1	4	38.5	17.721	161	0	95.146	3.096107142	3.530901361	3.939656916	3.713705311	3.685483043	5.63811023
sp|P35171|CX7A2_RAT C	Cytochrome c oxidase sub	Cox7a2	3	30.1	9.3529	83	0	74.778	106.8678509	104.3831106	89.23869937	112.1935578	102.5360176	107.176316
sp|Q32PX9|AFG1L_RAT A	AFG1-like ATPase (EC 3.6	Afg1l	2	1.7	54.502	480	0.0024814	2.5954	1.499712174	1.399606338	1.280013449	1.453170857	1.401895429	1.689787172
sp|P14408|FUMH_RAT Fu	Fumarate hydratase, mitoc	Fh	28	62.7	54.463	507	0	323.31	293.309604	233.1591346	238.2747826	280.594386	253.5060527	322.6363723
sp|O88656|ARC1B_RAT A	Actin-related protein 2/3 co	Arpc1b	2	7.3	41.056	372	0	4.6887	3.845386243	3.05780125	3.264843851	3.868871062	6.049803007	3.368906496

sp|Q5XJW2|G45IP_RAT G	Growth arrest and DNA da	Gadd45gip1	3	15.8	26.467	228	0	21.017	2.933798457	2.989736039	2.724453696	3.057505886	2.879993136	3.8961014
tr|F1M964|F1M964_RAT M	Mitochondrial-processing p	Pmpca	13	28.9	58.381	523	0	121.77	25.33821693	22.94671274	20.71777646	17.88288267	18.98693301	24.21398369
sp|P13803|ETFA_RAT Ele	Electron transfer flavoprote	Etfa	24	73	34.951	333	0	323.31	205.7331562	199.6349201	200.7013155	233.0651694	191.2930669	291.1205005
tr|Q6P3V9|Q6P3V9_RAT	60S ribosomal protein L4 (	Rpl4	16	35.9	47.3	421	0	132.99	32.74772126	31.55284059	32.25692436	34.82330361	39.57880573	31.25374759
tr|Q6GMM8|Q6GMM8_RA	Long-chain fatty acid trans	Slc27a1	8	19.3	71.346	646	0	36.118	4.016224286	4.447141044	4.229450571	4.411174764	4.253252039	5.463055919
tr|A0A0G2JZF6|A0A0G2J	NADH:ubiquinone oxidored	Ndufaf2	6	33.1	19.554	166	0	12.58	7.476735748	7.774439071	6.496116279	6.793727551	6.793489792	6.708552609
sp|P04182|OAT_RAT Orn	Ornithine aminotransferase	Oat	13	42.1	48.332	439	0	220.94	13.00626901	14.36526409	14.93928367	16.9033914	13.5195678	26.57310215
tr|B0BNG3|B0BNG3_RAT	Lectin, mannose-binding 2	Lman2	4	16.2	40.392	358	0	15.952	8.923214688	9.091320758	7.379767384	6.04417783	9.309297514	5.527571002
sp|Q68FU3|ETFB_RAT Ele	Electron transfer flavoprote	Etfb	20	81.6	27.687	255	0	323.31	153.4331337	156.7819711	151.2177684	174.5465386	147.0709376	222.2569997
sp|Q07984|SSRD_RAT Tr	Translocon-associated pro	Ssr4	3	19.7	18.979	173	0	19.07	2.347229422	2.298126361	2.015566093	2.106324304	2.54422853	2.72233329
tr|B2GV62|B2GV62_RAT	Mitochondrial ribosomal pr	Mrpl20	2	21.5	17.525	149	0	28.234	1.577530032	1.673989595	1.575615783	1.788646617	1.603213976	2.676309145
tr|Q5M9H2|Q5M9H2_RAT	Very long-chain specific ac	Acadvl	45	68.9	70.821	655	0	323.31	461.0720608	499.0626211	445.4251046	432.9052783	405.8135376	479.0270286
tr|F1M2E9|F1M2E9_RAT 6	60S ribosomal protein L13	Rpl13	10	36.2	24.069	210	0	29.252	19.78761114	16.62143583	19.7211534	25.59816816	26.44429403	16.27202462
sp|P46462|TERA_RAT Tra	Transitional endoplasmic re	Vcp	67	74.3	89.348	806	0	323.31	483.263446	453.5344477	523.0098546	523.2633478	502.546749	847.6672267
tr|E9PTI1|E9PTI1_RAT V-	V-type proton ATPase sub	Atp6v1h	2	8.6	50.825	441	0	47.893	0.712853721	0.723791344	0.633054604	0.659484304	0.775343357	0.970814836
sp|Q4V8F9|HSDL2_RAT H	Hydroxysteroid dehydroge	Hsdl2	21	37.8	58.343	524	0	194.67	46.18045822	43.87466758	51.45026545	51.30384146	46.04494391	63.91361919
tr|A0A0G2JZH8|A0A0G2J	Dihydrolipoamide acetyltra	Pdhx	17	43.2	54.169	502	0	251.95	77.43152785	80.63679202	78.26166701	101.9447346	69.36942065	114.3186945
sp|F1LU71|AUHM_RAT Me	Methylglutaconyl-CoA hydr	Auh	9	26.7	33.341	315	0	311.22	22.44576078	26.79321161	25.62954107	28.12095543	23.80444863	34.66034715
sp|Q6P742|PLP2_RAT Pr	Proteolipid protein 2	Plp2	2	17.9	16.556	151	0	90.596	2.102988719	2.664957872	2.717547313	2.492805279	2.761995552	4.264091367
tr|D3ZF12|D3ZF12_RAT S	Signal peptidase complex	Spcs3	2	11.7	20.327	180	0	5.1422	1.789760553	1.759626391	1.483454449	1.426115397	1.642097092	1.583108689
sp|P70470|LYPA1_RAT A	Acyl-protein thioesterase 1	Lypla1	3	13.9	24.708	230	0	6.6986	2.845093124	3.099805235	3.386551698	3.351831067	3.077752341	4.482325064
tr|D3ZZ21|D3ZZ21_RAT N	Complex I-B17 (NADH deh	Ndufb6	10	51.6	15.638	128	0	283.54	54.39222403	54.83716468	51.50515061	60.81207378	54.89476271	53.42153638
sp|P62909|RS3_RAT 40S	40S ribosomal protein S3 (	Rps3	13	50.2	26.674	243	0	79.19	21.95105796	16.1856013	19.95670222	22.52591018	28.42242538	18.29993179
tr|M0R6K0|M0R6K0_RAT	Laminin subunit beta-2 (La	Lamb2	6	4.9	196.53	1801	0	13.493	1.394901404	1.345558908	1.270682972	1.306008267	1.682112726	1.425935724
sp|Q6AXR4|HEXB_RAT B	Beta-hexosaminidase subu	Hexb	7	12.3	61.527	537	0	27.229	6.126086665	6.139689392	5.301906633	5.816147484	7.348962879	6.162460374
sp|Q4KM74|SC22B_RAT V	Vesicle-trafficking protein S	Sec22b	7	31.2	24.74	215	0	27.259	4.19338409	5.013331068	3.819595625	3.084083545	4.307936477	4.019950039
sp|P04636|MDHM_RAT M	Malate dehydrogenase, m	Mdh2	24	68.6	35.683	338	0	323.31	1373.728525	1159.033574	1205.003728	1463.025495	1152.202186	1758.061402
sp|Q63377|AT1B3_RAT S	Sodium/potassium-transpo	Atp1b3	7	30.8	31.829	279	0	52.996	4.184904904	4.914219433	5.972877736	5.774817731	4.866591401	6.934609394
sp|Q5U2U0|CLPX_RAT A	ATP-dependent Clp protea	Clpx	12	24.6	69.207	633	0	93.032	10.34109519	10.52715615	9.532637857	11.31790216	9.706757383	14.93092369
tr|D4ABV0|D4ABV0_RAT	RGD1566320 (Predicted) (	Smim26	2	25.2	12.108	107	0	5.9362	0.937627417	0.878283086	0.828628731	0.894621934	0.898356378	1.108745034
sp|P14841|CYTC_RAT Cy	Cystatin-C (Cystatin-3)	Cst3	2	20	15.437	140	0	49.73	5.683061724	5.423005655	5.367311451	5.439282246	6.241791764	5.65639797
tr|G3V9U2|G3V9U2_RAT	3-ketoacyl-CoA thiolase, m	Acaa2	34	91.2	41.885	397	0	323.31	263.4819338	245.6270624	263.1194658	301.4205205	237.4997991	336.3420174
sp|P39069|KAD1_RAT Ad	Adenylate kinase isoenzym	Ak1	13	70.6	21.584	194	0	137.09	18.04561499	18.17375701	20.08110858	25.17889812	23.90529666	16.65301921
tr|A0A0G2JYW3|A0A0G2J	Clathrin light chain A (Lca)	Clta	2	6.8	25.58	236	0.0010638	3.3839	1.058643969	0.907601966	1.048535279	0.953092785	1.584338677	1.141764565
sp|P35284|RAB12_RAT R	Ras-related protein Rab-12	Rab12	4	15.6	27.271	243	0.00055494	4.0477	1.093112614	1.24378192	1.042040535	0.906447499	1.039381827	1.146133303
tr|D4AE99|D4AE99_RAT C	Cell cycle progression 1 (C	Ccpg1	2	3	72.407	636	0.00054555	3.8243	2.546716077	3.400101644	3.345616515	2.411758349	2.478973924	3.223823759
tr|F1M953|F1M953_RAT S	Stress-70 protein, mitocho	Hspa9	42	57.4	73.744	679	0	323.31	195.4176491	173.8747795	196.3745353	221.556147	174.1542953	301.9001072
tr|D4AAB5|D4AAB5_RAT	Peptidase M20 domain-co	Pm20d2	3	6.5	46.683	431	0	26.491	6.273594403	37.77150339	26.7505705	7.361115786	8.414608337	21.56632534
sp|Q925F0|SMPX_RAT Sm	Small muscular protein (Str	Smpx	2	22.4	9.1204	85	0.0015464	2.9664	0.454675042	0.459013228	0.545878672	0.594885661	0.441369208	0.9451612
sp|P12007|IVD_RAT Isova	Isovaleryl-CoA dehydrogen	Ivd	19	42.5	46.435	424	0	323.31	72.27377429	62.36925408	80.11861498	96.17529209	59.65673103	128.9387266
tr|Q3B8N9|Q3B8N9_RAT	Biphenyl hydrolase-like (Bi	Bphl	5	17.5	32.822	291	0	9.6345	3.21035038	3.401187528	3.299467241	3.749898708	3.086003543	4.550904089
sp|P80067|CATC_RAT Dip	Dipeptidyl peptidase 1 (EC	Ctsc	5	14.3	52.235	462	0	20.129	3.680418406	4.011553305	4.855964693	4.323677526	4.298930263	6.014634476
tr|D4A678|D4A678_RAT S	Spectrin, alpha, erythrocyt	Spta1	115	55.5	280.07	2416	0	323.31	206.7115238	247.9222272	222.0653648	271.2474114	363.5059029	203.3596684
tr|D4AE68|D4AE68_RAT G	Guanine nucleotide-bindin	Gnaq	4	17	42.158	359	0	19.088	4.581068543	4.505630729	3.626537068	3.183525799	4.434239193	3.634078726
tr|G3V6W2|G3V6W2_RAT	Prolactin regulatory elemer	Preb	6	21.1	45.371	417	0	41.396	1.585256628	1.806319423	1.714612456	1.791692596	1.69678153	2.515377033
sp|Q497B0|NIT2_RAT Om	Omega-amidase NIT2 (EC	Nit2	6	24.3	30.701	276	0	28.422	4.148178487	4.764959679	5.260742761	5.133788475	4.627414397	7.453879611
tr|D3ZCZ9|D3ZCZ9_RAT N	NADH dehydrogenase [ub	LOC1009125	8	55.2	13.04	116	0	323.31	134.2069551	138.7562892	100.9155176	115.5501072	112.4806038	104.4534747
sp|P11275|KCC2A_RAT C	Calcium/calmodulin-depen	Camk2a	5	17.4	54.114	478	0	199.17	16.81989593	20.07553554	20.81245337	20.63979224	24.5211707	18.89733129
tr|G3V9X8|G3V9X8_RAT	Magnesium transporter pro	Magt1	3	7.8	37.94	335	0	5.6564	1.051067891	1.18756272	1.200292752	1.141047603	1.090560852	1.029294964
sp|P26772|CH10_RAT 10	10 kDa heat shock protein	Hspe1	11	77.5	10.902	102	0	117.91	145.2499664	127.4087967	135.7858904	157.2202937	125.5369178	191.8993513
sp|Q6RUV5|RAC1_RAT R	Ras-related C3 botulinum t	Rac1	5	24	21.45	192	0	91.985	39.92291909	44.74090722	42.60460687	43.74324391	42.3157241	53.94273697
tr|G3V734|G3V734_RAT 2	2,4-dienoyl-CoA reductase	Decr1	16	51.9	36.132	335	0	218.56	29.04146392	43.09974095	81.143138	65.44673978	49.20844404	173.7335296
sp|Q5XFV8|COX14_RAT C	Cytochrome c oxidase ass	Cox14	2	28.1	6.3613	57	0.0024988	2.6599	3.390219509	2.79328967	2.200620564	2.110624509	2.379204486	2.690634541
sp|B5DEH2|ERLN2_RAT E	Erlin-2 (Endoplasmic reticu	Erlin2	9	31	37.71	339	0	100.2	14.75127552	30.33467567	25.10401564	15.92569188	15.85147618	22.42076919
tr|F1LMC6|F1LMC6_RAT T	Troponin I, slow skeletal m	Tnni1	2	12.3	21.697	187	0	31.428	0.865228211	0.601974852	0.772508653	0.858488262	0.729772992	0.954620026
tr|F1M8K0|F1M8K0_RAT D	Alpha-dystroglycan (Beta-d	Dag1	5	6	96.705	893	0	17.641	10.28891558	11.77444251	14.27791747	11.93366771	14.61002079	14.12118321
tr|A0A0H2UHT6|A0A0H2U	40S ribosomal protein S18	Rps18	9	42.4	17.587	151	0	33.326	12.42025188	9.743345011	10.41080045	13.48950597	16.72944724	9.516127087
tr|G3V8A5|G3V8A5_RAT V	Vacuolar protein sorting-as	Vps35	2	3.9	91.726	796	0	13.47	0.144161219	0.137808608	0.126148971	0.136483747	0.233282518	0.143690837
tr|F8WFT7|F8WFT7_RAT A	Anion exchange protein	Slc4a1	2	2.3	103.24	928	0	323.31	129.1194433	167.9813884	131.0428976	176.8698441	246.9266628	124.6512675
tr|A0A0G2JSU4|A0A0G2J	Protein NDRG2 (Antidepre	Ndrg2	6	30.3	39.255	357	0	146.83	13.59579824	10.29714608	17.02034606	16.13891041	16.23113934	15.03861816
sp|P61354|RL27_RAT 60S	60S ribosomal protein L27	Rpl27	7	50.7	15.798	136	0	70.622	18.58597497	16.25865171	18.75243029	21.39591172	23.75753003	16.07390744
tr|F1LZW6|F1LZW6_RAT S	Solute carrier family 25 me	Slc25a13	2	6.4	54.099	484	0	48.128	0.297072556	0.287448421	0.28563153	0.246580954	0.28616032	0.290703946
tr|D3ZUX7|D3ZUX7_RAT A	Acyl-CoA synthetase family	Acsf3	10	23.5	65.265	583	0	61.912	7.973947205	7.202868984	7.77859956	7.969714324	7.729165332	13.3693539
tr|D4AD70|D4AD70_RAT U	60S ribosomal protein L38	Rpl38	4	52.9	8.2458	70	0	23.283	9.408886423	8.533077434	9.335508651	9.785356676	11.19359166	8.711669726
sp|Q5I0I4|DMAC2_RAT Di	Distal membrane-arm asse	Dmac2	3	24.8	28.507	254	0	6.0553	0.791925896	0.489768437	0.602867765	0.73342395	0.786075313	0.661498034
tr|G3V731|G3V731_RAT M	Myosin light chain kinase 2	Mylk2	3	10	65.948	610	0	13.901	1.891460616	1.770732028	1.727647681	2.348390042	3.655120776	1.475515819
tr|G3V7K1|G3V7K1_RAT	Myomesin 2	Myom2	55	44.3	164.71	1464	0	323.31	69.60960392	68.18367172	88.36511055	81.93683364	79.65915513	86.09969572

tr|A0A0G2JU49|A0A0G2J	Metaxin 1	Mtx1	7	17.9	51.925	463	0	29.978	6.526966538	6.614023463	5.728638766	5.96701303	5.766134227	6.190501575
tr|Q5RK10|Q5RK10_RAT	60S ribosomal protein L13	Rpl13a	9	35	23.446	203	0	26.258	16.94532768	14.67523475	15.16980135	17.87631291	18.89579228	14.52148151
sp|P97536|CAND1_RAT C	Cullin-associated NEDD8-d	Cand1	4	3.3	136.36	1230	0	7.208	1.33981178	1.147977296	1.20477504	1.211642645	1.82545714	1.291317638
sp|D3ZUI5|COBL_RAT Pro	Protein cordon-bleu	Cobl	5	7.1	122.27	1133	0	34.697	6.214892344	5.37858311	6.009467844	5.608423665	5.441479082	5.721421045
tr|D4A5L9|D4A5L9_RAT U	Cytochrome c, somatic	Cycs	13	72.4	11.635	105	0	252.96	292.8530324	353.3221205	307.3797751	340.6061437	308.1095965	404.7178452
tr|Q6IRH6|Q6IRH6_RAT P	Phosphate carrier protein,	Slc25a3	19	48.5	39.621	357	0	323.31	1431.226439	1343.239063	1016.015821	1149.468839	1275.215207	844.7716679
sp|A6ZIQ8|TM38A_RAT T	Trimeric intracellular cation	Tmem38a	6	11.4	33.276	297	0	104.18	60.31761989	78.80559597	89.36676475	91.49284602	79.9719458	85.18734069
sp|P63095|GNAS2_RAT G	Guanine nucleotide-bindin	Gnas	9	24.6	45.663	394	0	50.074	16.04021218	17.77444768	19.01130531	15.25677101	16.65933898	17.6801806
sp|Q5I0L3|SYYM_RAT Ty	Tyrosine--tRNA ligase, mito	Yars2	4	12.1	52.627	471	0	10.576	0.990007716	0.975667177	0.799082219	0.84128744	0.94236279	0.733145335
sp|P19836|PCY1A_RAT C	Choline-phosphate cytidyly	Pcyt1a	7	22.6	41.68	367	0	43.429	4.374658055	6.017280599	5.850300875	6.002370275	5.461972264	6.58399278
sp|P69736|EDF1_RAT En	Endothelial differentiation-	Edf1	2	11.5	16.369	148	0.0015369	2.9195	1.230937021	1.221965514	1.181906222	1.121099427	1.267988664	0.946268625
tr|D3ZIL6|D3ZIL6_RAT En	Enoyl CoA hydratase dom	Echdc2	5	26.4	31.779	296	0	54.248	5.323323466	6.350449692	6.578444022	7.054128888	5.481926152	9.435254637
sp|P61589|RHOA_RAT Tr	Transforming protein RhoA	Rhoa	3	18.7	21.782	193	0	29.069	18.52677119	19.3529288	21.75281914	19.300756	21.56691675	22.74182063
tr|A0A0G2K2A0|A0A0G2K	Elongin-C (EloC) (Elongin 1	Eloc	2	26.8	12.591	112	0	38.512	1.445525659	1.582725943	1.436344685	1.645784232	1.38765806	2.201335898
sp|Q32Q06|AP1M1_RAT A	AP-1 complex subunit mu-	Ap1m1	2	5	48.556	423	0	5.1304	0.809486341	0.718756789	0.768072102	0.72930292	0.832184972	0.900457836
sp|Q07969|CD36_RAT Pla	Platelet glycoprotein 4 (Ad	Cd36	7	19.1	52.73	472	0	190.3	44.8252936	70.05435448	58.01361605	48.50154083	51.03125863	50.05659223
tr|D3ZJH9|D3ZJH9_RAT M	Malic enzyme	Me2	9	22.3	65.351	584	0	53.972	9.052660254	9.944727218	8.863953635	8.827366435	10.01361582	13.54105546
tr|A0A0G2JXA8|A0A0G2J	Flotillin-2 (Reggie-1) (REG-	Flot2	15	48	41.658	379	0	263.89	14.88573835	29.78482327	21.04845956	15.24900675	17.64139383	19.02839343
tr|F1LMS5|F1LMS5_RAT T	Thrombospondin-4	Thbs4	4	7.5	105.83	959	0	48.771	1.963257749	2.329715727	2.621909918	2.320856781	2.362324576	3.475686578
tr|D4AB01|D4AB01_RAT H	Histidine triad nucleotide b	Hint2	5	44.2	17.38	163	0	60.151	5.823043557	6.15745841	6.176867588	6.566175006	5.720833509	9.482701162
tr|A0A0G2K1N8|A0A0G2K	Cysteine and glycine-rich p	Csrp3	3	17.3	20.799	191	0	23.582	1.939776926	2.460712878	6.523101483	8.83094994	1.936012464	7.454184406
tr|D3Z9L0|D3Z9L0_RAT A	Acylglycerol kinase	Agk	9	18.8	54.258	490	0	26.381	12.90592361	13.24186727	11.68322145	11.35672346	11.3677837	13.04017458
sp|Q5U2R7|MESD_RAT L	LRP chaperone MESD (LD	Mesd	2	9.8	25.215	224	0.001552	3.014	0.3064398	0.243563882	0.32593096	0.352754224	0.299049453	0.309377761
sp|Q8CG09|MRP1_RAT M	Multidrug resistance-assoc	Abcc1	5	5.7	171.49	1532	0	85.883	4.074223928	5.785296194	6.349298471	6.219172306	5.316362814	7.100113441
sp|Q6AYD9|NUD19_RAT N	Nucleoside diphosphate-lin	Nudt19	3	10.4	39.938	357	0	41.719	2.372867672	1.466092081	1.656434184	2.071504583	2.766363835	1.783969033
tr|A0A0G2QC50|A0A0G2C	CD55 molecule (Cromer bl	Cd55	2	7.3	42.182	382	0	17.728	0.682900619	0.869102426	1.04021103	0.963186323	0.885575104	4.360406798
tr|A0A0H2UHS3|A0A0H2U	Mitochondrial potassium ch	Ccdc51	11	31.1	42.308	379	0	136.36	17.40741825	17.15006412	14.84826578	16.19564923	16.14754873	14.02161662
tr|Q6AYS3|Q6AYS3_RAT	Carboxypeptidase (EC 3.4	Ctsa	4	10.7	51.215	456	0	21.887	2.67350249	2.078975136	2.467316712	2.160256048	2.862627861	3.240079528
sp|Q5I0E7|TMED9_RAT T	Transmembrane emp24 do	Tmed9	2	13.2	27.028	235	0	57.699	5.370485804	5.171771481	5.181616653	5.062178108	5.555270171	9.06086396
tr|C6ZII9|C6ZII9_RAT NAD	NAD-dependent protein de	Sirt3	4	12.2	35	320	0	10.437	1.209814314	1.31584516	1.185473758	1.328882971	1.17533791	1.970605579
tr|Q5FVC4|Q5FVC4_RAT	DnaJ (Hsp40) homolog, su	Dnajb12	6	14	42.521	378	0	16.392	3.338491455	3.122460733	4.280448033	4.60074216	3.826832068	48.01750922
sp|Q8R4Z9|MFN1_RAT Mi	Mitofusin-1 (EC 3.6.5.-) (Mi	Mfn1	10	16.1	83.846	741	0	34.754	7.829449829	7.308002342	6.761394561	7.11743747	8.520849306	7.691417033
tr|D3ZMR9|D3ZMR9_RAT	Mitochondrial ribosomal pr	Mrpl21	5	26.3	23.39	209	0	22.986	3.779308797	3.640180824	3.357188136	3.594075981	3.627023545	4.581383656
tr|F1M9Q8|F1M9Q8_RAT	Alkyldihydroxyacetonepho	Agps	8	15.5	71.575	644	0	51.258	8.121956669	8.672268077	7.918556723	8.254602943	8.566689318	8.618910245
tr|M0RAK2|M0RAK2_RAT	RCG22622 (Similar to isoc	LOC684270	4	35.4	22.929	209	0	85.277	3.649813059	5.275424087	6.776030604	7.414868355	3.953620149	9.857193437
tr|D4A830|D4A830_RAT F	Inorganic diphosphatase (	Ppa2	12	49.7	35.046	304	0	209.65	14.7658256	14.08688281	16.5940713	15.49328232	15.00532349	20.33088691
tr|F1LPY4|F1LPY4_RAT C	Chloride channel protein 1	Clcn1	4	3.7	110.07	994	0	33.305	1.200030638	1.342251896	1.598393125	1.445107972	1.471787965	1.587680624
sp|Q91ZW6|TMLH_RAT Tr	Trimethyllysine dioxygenas	Tmlhe	2	5.9	49.567	421	0	7.1218	1.624240816	1.720189042	1.787518246	1.966448172	1.530301392	2.432777408
tr|M0RCT5|M0RCT5_RAT	Echinoderm microtubule-as	Eml1	2	2.6	85.75	780	0	7.5845	0.654653389	0.652715271	0.674355688	0.87646551	0.96959715	0.537090603
tr|G3V9M6|G3V9M6_RAT	Fibrillin 1 (Fibrillin 1, isoform	Fbn1	54	24.9	311.95	2872	0	323.31	77.26094067	116.6239898	96.34175406	88.97842026	119.3458197	125.392937
sp|Q8CHN6|SGPL1_RAT	Sphingosine-1-phosphate	Sgpl1	2	3.3	63.758	568	0.0010805	3.698	0.942443996	0.805824978	1.017708113	0.917795264	1.061492892	0.992028614
tr|Q9ET45|Q9ET45_RAT B	BCL2-interacting protein 3	Bnip3	6	21.9	20.978	187	0	24.093	5.242043692	4.785344692	4.021435807	3.995488226	4.47770631	4.469726843
tr|D3ZT71|D3ZT71_RAT B	BCL2-like 13	Bcl2l13	4	11.8	46.734	434	0	21.371	4.34836756	4.175768649	3.257434355	3.743269224	4.313599067	5.184066286
sp|Q5FVN1|STBD1_RAT S	Starch-binding domain-con	Stbd1	7	18	35.494	333	0	32.931	16.45714731	24.21472963	17.64786641	11.67923888	13.54113957	22.16778879
tr|A0A0G2K4T8|A0A0G2K	Mitochondrial intermediate	Mipep	9	16.6	80.875	711	0	55.964	7.831958464	7.063184757	6.736238862	7.306168714	7.208746372	8.696937935
tr|D4ADS4|D4ADS4_RAT	Microsomal glutathione S-t	Mgst3	4	34.9	16.732	152	0	21.891	8.636226844	9.921035194	7.20367749	7.035614114	8.53271378	7.71691827
sp|P08010|GSTM2_RAT G	Glutathione S-transferase	Gstm2	6	32.1	25.702	218	0	29.029	2.774500135	3.021127971	3.536616878	2.909268244	3.455258323	4.048194437
sp|P14141|CAH3_RAT Ca	Carbonic anhydrase 3 (EC	Ca3	17	60	29.431	260	0	323.31	70.57793703	64.15109175	90.13515701	79.07600243	71.47266826	144.2597887
sp|Q8VID1|DHRS4_RAT D	Dehydrogenase/reductase	Dhrs4	7	31.2	29.821	279	0	210.39	10.22068071	10.54788667	10.92534884	8.887091512	9.117848052	11.34449469
sp|Q5XI64|ABHD6_RAT M	Monoacylglycerol lipase AB	Abhd6	2	8	38.311	337	0	4.6167	0.978467995	0.992350311	1.053703632	0.953451135	1.071200188	1.35308956
sp|P62804|H4_RAT Histor	Histone H4 [Cleaved into:	H4c2	6	51.5	11.367	103	0	66.227	14.30022294	12.75025777	12.86691144	13.78633961	34.4797785	12.68356365
sp|Q9Z1W6|LYRIC_RAT P	Protein LYRIC (Lysine-rich	Mtdh	10	23.9	63.968	581	0	78.027	8.372820169	9.126365211	7.692612807	6.940651241	9.152902179	6.64799987
sp|P0DN35|NDUB1_RAT N	NADH dehydrogenase [ub	Ndufb1	4	49.1	6.9981	57	0	13.187	37.2657729	35.26952691	35.43751949	34.28637516	37.14658862	32.10311151
sp|P20070|NB5R3_RAT N	NADH-cytochrome b5 redu	Cyb5r3	5	26.6	34.174	301	0	264.37	12.73583816	17.86279919	16.9576855	15.00831469	16.79955549	27.55251222
tr|G3V6Z7|G3V6Z7_RAT G	Golgin subfamily A membe	Golga5	2	3.2	82.405	729	0.0015544	3.0397	0.401577273	0.382818691	0.390425599	0.474694915	0.426689618	0.366171353
tr|M0R959|M0R959_RAT M	Corrinoid adenosyltransfer	Mmab	3	15.2	26.233	237	0	10.417	2.161640605	2.716339949	2.713385188	2.971621224	2.222269857	3.617416563
sp|Q6PDV7|RL10_RAT 60	60S ribosomal protein L10	Rpl10	12	49.1	24.604	214	0	323.31	31.42567062	28.08195902	29.89960666	37.43567849	37.64759299	25.83143269
tr|A0A0G2K791|A0A0G2K	39S ribosomal protein L33	Mrpl33	2	31.2	7.579	64	0	4.9013	2.211562442	2.630456361	2.067981422	2.092587536	2.126491196	2.196154372
sp|Q5FVL2|EMC8_RAT EI	ER membrane protein com	Emc8	6	30.4	23.405	207	0	34.563	8.296557665	9.172268508	8.834224173	9.158243364	8.3428822	12.73436293
tr|D3ZCA0|D3ZCA0_RAT P	Pyridoxal phosphate home	Plpbp	2	10.5	30.104	275	0.0010543	3.229	0.754547235	0.646841623	0.659216531	0.649390766	0.796645481	0.760160391
sp|P38718|MPC2_RAT Mi	Mitochondrial pyruvate car	Mpc2	5	22.8	14.258	127	0	34.475	9.45454358	7.742356122	6.395036106	9.329654336	7.575466468	9.512672736
sp|Q9ES21|SAC1_RAT Ph	Phosphatidylinositol-3-pho	Sacm1l	10	19.1	67.038	587	0	48.501	11.80061903	15.07010182	12.67801501	13.40469636	13.38042988	16.30250419
tr|B2RYC9|B2RYC9_RAT G	Glucosylceramidase (EC 3	Gba	8	20.6	57.491	515	0	30.489	3.483039004	4.246647288	4.194278329	3.239906272	3.147590948	4.34404943
tr|A0A0G2JWK0|A0A0G2J	Integrin beta	Itgb3	6	13.8	62.389	567	0	24.316	3.140560154	3.736577748	2.413163353	3.242773076	4.282212141	3.125273161
sp|P63322|RALA_RAT Ra	Ras-related protein Ral-A (	Rala	7	26.7	23.553	206	0	48.124	14.93892141	20.1777074	20.01433164	19.76661161	18.02591064	23.25184537
tr|F1LQ92|F1LQ92_RAT P	Protein sel-1 homolog 1 (S	Sel1l	2	2.4	88.441	792	0	7.5598	0.348589885	0.384146431	0.341495477	0.320454903	0.404292728	0.487855943
tr|D3ZK56|D3ZK56_RAT P	Ras-related protein Rap-2	Rap2c	3	28.4	20.745	183	0	66.98	7.364850627	8.588852408	9.745775236	8.952789097	9.357294703	9.346051105

sp|P15390|SCN4A_RAT S	Sodium channel protein ty	Scn4a	10	4.2	208.86	1840	0	90.316	10.37972817	10.93337565	10.74148355	10.83830979	11.37317665	10.5774256
tr|G3V7Q3|G3V7Q3_RAT	Coiled-coil domain-contain	Ccdc127	5	23.4	29.588	252	0	73.725	2.313212332	3.09018035	3.230998002	3.094595159	2.76215734	3.915913118
tr|M0R776|M0R776_RAT M	Mitochondrial ribosomal pr	Mrps36	5	54.4	11.413	103	0	64.687	30.33391267	31.68413389	33.8147482	36.86351225	26.97765606	56.77327272
tr|D3ZHA7|D3ZHA7_RAT S	Similar to Myosin light chai	RGD1560334	5	30	22.807	207	0	64.911	10.54730499	8.623403277	11.01042084	10.86219982	11.12995494	10.27770986
tr|D3ZHF8|D3ZHF8_RAT T	Translation factor GUF1, m	Guf1	2	4.9	73.206	659	0	10.055	0.671661934	0.484921444	0.497396779	0.573766873	0.593978698	0.658622795
tr|A0A0G2K5E4|A0A0G2K	DnaJ heat shock protein fa	Dnaja3	15	33.8	52.403	480	0	323.31	29.32644485	31.96942535	30.61219901	31.95470814	28.85979302	43.64877134
sp|P84903|STIM1_RAT St	Stromal interaction molecu	Stim1	14	24.7	77.448	685	0	270.29	7.28407258	9.04788538	10.0888075	8.911578794	9.721318328	10.12968076
tr|A0A0G2K6H2|A0A0G2K	Maleylacetoacetate isomer	Gstz1	6	35.8	23.862	215	0	39.994	7.380404164	8.521725006	10.10710255	10.28286657	6.770839433	19.8249261
sp|B2RYW9|FAHD2_RAT	Fumarylacetoacetate hydr	Fahd2	9	42.5	34.581	313	0	273.06	9.608573769	11.26851908	12.0651307	12.03520034	9.659838783	19.66440039
sp|D3ZX08|SYAM_RAT Al	Alanine--tRNA ligase, mito	Aars2	6	9.9	107.8	985	0	16.02	1.092159333	1.042300996	1.15139922	1.154664921	1.01937401	1.587579026
tr|Q5XFV6|Q5XFV6_RAT	39S ribosomal protein L34	Mrpl34	2	24.2	10.446	91	0	4.8941	0.427571749	0.527739842	0.42254714	0.442712136	0.338444899	0.466692964
sp|P52873|PYC_RAT Pyr	Pyruvate carboxylase, mito	Pc	27	30.6	129.78	1178	0	323.31	31.60679407	35.41167905	34.51224713	35.41637363	32.01466444	44.54893454
sp|P11730|KCC2G_RAT C	Calcium/calmodulin-depen	Camk2g	10	21.8	59.037	527	0	59.435	7.943341858	8.815407391	9.560995191	6.925122721	9.560608639	7.466376233
tr|D4AE17|D4AE17_RAT M	Mitogen-activated protein k	Map3k20	4	9.7	51.317	453	0	7.9335	0.990108061	0.976753062	0.886669789	0.954048386	1.335616164	0.928245041
tr|D4A604|D4A604_RAT P	Phosphatidylinositol glycan	Pigt	3	5.2	67.502	598	0	15.155	0.079573902	0.087872731	0.122252124	0.081715851	0.138922201	0.122294181
tr|A0A0A0MXW3|A0A0A0	Histone H2A.Z (H2A/z)	H2az1	2	18.9	13.421	127	0	5.4448	3.702945948	3.989736899	3.501902013	3.850774363	4.333391166	3.635602704
sp|P04762|CATA_RAT Ca	Catalase (EC 1.11.1.6)	Cat	19	44.8	59.756	527	0	323.31	37.12478761	41.15304628	46.13097852	42.17068263	39.02225406	73.82455826
sp|Q91ZW1|TFAM_RAT Tr	Transcription factor A, mito	Tfam	11	44.3	28.186	244	0	82.104	23.6539194	25.58787987	24.98052403	27.45442356	22.01830604	35.32378572
sp|P32089|TXTP_RAT Tri	Tricarboxylate transport pr	Slc25a1	3	10	33.835	311	0	13.631	0.610952967	0.633662934	0.551550139	0.518276304	0.679780415	0.907772932
sp|Q9QZ76|MYG_RAT My	Myoglobin	Mb	10	70.8	17.157	154	0	212.31	63.66915625	103.8697834	112.0526316	59.88036257	74.29317724	99.00271225
sp|P62836|RAP1A_RAT R	Ras-related protein Rap-1A	Rap1a	4	33.2	20.987	184	0	144.19	12.11570359	15.77592671	12.31028442	16.63343405	11.76740075	16.85418434
tr|D4A4P4|D4A4P4_RAT F	FAD synthase (EC 2.7.7.2	Flad1	2	6.5	54.604	490	0	7.4994	1.614356794	0.983860669	1.324882069	1.490439306	1.3820494	1.549377969
sp|Q5U2V8|EMC3_RAT E	ER membrane protein com	Emc3	6	29.9	29.98	261	0	65.906	5.899306061	6.208297546	5.460616226	5.624967512	6.035242062	7.382151031
tr|A0A0G2K8Q1|A0A0G2K	Apolipoprotein C-III (Apo-C	Apoc3	2	39	11.029	100	0	125.86	1.608938142	1.725717181	1.792503648	1.715483397	1.627482217	2.863148888
sp|Q9QX75|TRDN_RAT Tr	Triadin	Trdn	2	1.6	77.199	687	0	4.6242	5.971554749	2.35528337	2.483736523	5.798946662	6.214827051	2.734423518
tr|F1LNY3|F1LNY3_RAT N	Neural cell adhesion molec	Ncam1	5	7.2	92.72	842	0	63.814	4.66390367	5.397339296	5.437290032	5.103328686	4.851545091	10.83446994
sp|D3Z8L7|RRAS_RAT Ra	Ras-related protein R-Ras	Rras	5	21.6	23.909	218	0	127.07	6.82599583	7.650549528	7.020269574	6.897649185	7.528008574	8.157246409
tr|A0A0G2K7W8|A0A0G2	Histidine triad nucleotide-b	Hint3	3	29.2	14.317	130	0	17.21	3.695871597	3.401483678	4.044944952	3.799530247	4.020708354	3.833923084
sp|P97576|GRPE1_RAT G	GrpE protein homolog 1, m	Grpel1	10	45.6	24.297	217	0	99.489	14.61129368	13.25963629	13.77205923	14.25816772	13.4257306	16.55142065
tr|F1LPV8|F1LPV8_RAT S	Succinate--CoA ligase [GD	Suclg2	14	39.4	46.638	431	0	221.09	18.80071413	23.37761144	25.29565633	23.31487845	21.21637548	34.36367937
sp|Q6MGB5|DHB8_RAT E	Estradiol 17-beta-dehydro	Hsd17b8	5	30.1	26.79	259	0	67.362	3.47757018	4.484258549	4.418529953	4.738408463	3.475805434	6.873853458
tr|Q68FZ8|Q68FZ8_RAT P	Propionyl coenzyme A car	Pccb	3	8.5	58.678	541	0	323.31	47.20247612	45.74386958	52.79037815	51.43882013	45.69871699	61.28424858
tr|F1LWG4|F1LWG4_RAT	Complex I intermediate-as	Ndufaf1	5	17.4	37.781	328	0	23.436	7.146599382	6.333174257	5.428599881	6.794324801	7.016757615	6.44612354
tr|D3ZUX5|D3ZUX5_RAT M	Coiled-coil-helix-coiled-coil	Chchd3	20	63.9	26.434	227	0	323.31	171.8264456	165.8145554	147.0236273	184.8311969	149.7404442	183.7308275
sp|Q06647|ATPO_RAT AT	ATP synthase subunit O, m	Atp5po	16	66.7	23.397	213	0	323.31	931.4561748	876.7036175	723.7980716	833.7620801	844.6426698	625.0546321
sp|Q66HF1|NDUS1_RAT N	NADH-ubiquinone oxidore	Ndufs1	50	75	79.411	727	0	323.31	859.9099046	871.4222704	736.3759212	823.0712912	754.7423167	770.5539232
sp|Q9Z1E1|FLOT1_RAT F	Flotillin-1 (Reggie-2) (REG-	Flot1	21	59.3	47.499	428	0	212.31	25.95534114	37.57752494	26.83381299	24.56850783	26.23936221	29.43818141
tr|D4ABI7|D4ABI7_RAT Ve	Very-long-chain (3R)-3-hyd	Hacd3	2	8	43.104	362	0	33.831	0.768194209	1.062735367	0.972290642	0.904894647	1.056423526	1.095334025
tr|E9PT04|E9PT04_RAT V	VPS39 subunit of HOPS c	Vps39	2	2.7	100.61	875	0.0015361	2.9152	0.491742632	0.532034022	0.454087813	0.337279457	0.510981311	0.483385607
sp|Q9JHU5|ARFP1_RAT A	Arfaptin-1 (ADP-ribosylatio	Arfip1	2	6	40.779	366	0.00055617	4.0825	4.426887836	3.900941167	2.118475771	2.892366047	3.489934944	2.107966826
tr|A0A140TAB7|A0A140TA	Vesicle-associated membra	Vamp5	3	34.7	11.364	101	0	67.271	7.095423228	9.07108382	9.836793129	9.207217927	8.250662882	11.6177948
sp|O08776|NDUF3_RAT N	NADH dehydrogenase [ub	Ndufaf3	5	41.1	20.696	185	0	222.74	6.339822367	6.048869965	5.493089946	6.372665755	5.917675914	6.283769049
tr|A0A0G2JVH4|A0A0G2J	MICOS complex subunit M	Immt	65	66.1	86.229	776	0	323.31	606.1865609	600.7902506	504.6233254	568.6066266	513.9743944	540.2909574
tr|G3V7J0|G3V7J0_RAT A	Methylmalonate-semialdeh	Aldh6a1	28	58.9	57.747	535	0	323.31	134.0062643	170.7454579	175.8840748	121.2598246	109.5684148	185.2649657
sp|Q5NDF0|PMGT2_RAT	Protein O-linked-mannose	Pomgnt2	3	6.2	66.716	580	0	9.0712	2.177595524	3.222658253	3.274814656	3.033018604	2.914561898	3.771846367
tr|Q6MG85|Q6MG85_RAT	1-acyl-sn-glycerol-3-phosp	Agpat1	3	15.1	31.698	284	0	6.4464	2.397101086	2.208442177	1.968913705	2.185579481	2.383788487	2.335039597
tr|D3ZQL1|D3ZQL1_RAT E	ER membrane protein com	Emc7	4	19.5	26.44	241	0	7.1472	6.898746244	7.165850196	9.042330411	7.698562473	7.666607198	10.28075781
tr|D3ZM21|D3ZM21_RAT C	Catechol-O-methyltransfera	Comtd1	6	36.3	28.987	262	0	144.66	1.905157763	2.5223615	2.210499893	2.384045913	2.062854473	2.877474284
sp|P07340|AT1B1_RAT S	Sodium/potassium-transpo	Atp1b1	10	28	35.201	304	0	63.254	42.85852376	46.35887505	41.66744273	35.62063339	39.01632182	47.24637619
tr|F1LP30|F1LP30_RAT M	Methylcrotonoyl-CoA carbo	Mccc1	17	26.7	79.295	715	0	161.27	20.82066703	22.87267517	22.75081433	23.75983028	20.5659866	26.73261188
sp|P10759|AMPD1_RAT A	AMP deaminase 1 (EC 3.5	Ampd1	35	49.4	86.431	747	0	323.31	156.1725632	202.4878347	186.9022711	194.5186045	262.7118058	164.5185407
sp|Q4FZT8|SPRY4_RAT S	SPRY domain-containing p	Spryd4	5	32.4	23.249	207	0	15.138	5.039345984	4.462343427	4.393511467	4.839702194	4.464170024	5.335346535
tr|G3V6D3|G3V6D3_RAT	ATP synthase subunit beta	Atp5f1b	29	73.7	56.344	529	0	323.31	4299.198314	2874.582929	3233.74226	4045.955916	3536.745686	3919.773863
sp|Q32KJ6|GALNS_RAT N	N-acetylgalactosamine-6-s	Galns	4	7.6	58.302	524	0	16.951	2.019802382	2.144621787	1.665627449	1.86443774	1.98891723	2.594928702
sp|P14604|ECHM_RAT En	Enoyl-CoA hydratase, mito	Echs1	18	51	31.516	290	0	323.31	184.6706567	170.2617458	201.8493302	195.1576628	165.8168061	292.0348875
sp|P51868|CASQ2_RAT C	Calsequestrin-2 (Calseque	Casq2	6	17.4	47.838	413	0	60.219	21.69618064	11.3716781	14.48693846	10.44233253	17.10156027	11.53042005
sp|Q64578|AT2A1_RAT S	Sarcoplasmic/endoplasmic	Atp2a1	57	47	109.41	994	0	323.31	6891.722066	9514.322109	9206.528521	6050.150337	9096.276281	7725.960542
tr|A0A0H2UHY9|A0A0H2U	Troponin T, fast skeletal m	Tnnt3	2	3.6	29.741	250	0	323.31	69.02258333	53.08000621	91.6902366	96.76657035	81.04514137	67.69105348
sp|Q4QQW3|HOT_RAT Hy	Hydroxyacid-oxoacid trans	Adhfe1	7	19.1	50.225	467	0	79.802	4.097403715	4.535788702	4.47583921	3.598973437	3.657763318	4.838732796
tr|Q642E6|Q642E6_RAT T	Tripeptidyl-peptidase 1 (TF	Tpp1	3	8.7	61.312	563	0	48.444	5.641418383	7.086876782	9.253180908	5.303168795	5.797952588	7.915340249
sp|Q8K1Q0|NMT1_RAT G	Glycylpeptide N-tetradecar	Nmt1	2	5.2	56.86	496	0	38.78	0.966827928	1.0214224	0.988939141	0.677401828	1.228404465	0.584689526
tr|F1LRE1|F1LRE1_RAT G	Glutathione reductase (GR	Gsr	3	11.2	44.789	410	0	43.226	2.68298513	2.326951658	3.226287025	2.872358146	2.498550306	4.126933318
tr|F1LVY9|F1LVY9_RAT P	Peptide deformylase (EC 3	Pdf	6	30.3	25.879	231	0	38.137	5.758822501	5.082432805	4.219891405	5.091204495	5.415053663	5.652537225
tr|G3V6P2|G3V6P2_RAT	Dihydrolipoyllysine-residue	Dlst	20	47.1	48.899	454	0	323.31	163.3021038	151.7227367	179.3601351	180.8057267	142.5732235	240.4431411
tr|D4A4L5|D4A4L5_RAT Ir	Iron-sulfur cluster assembly	Isca2	4	36.4	16.68	154	0	51.57	5.027806263	5.590330577	6.345182084	5.604183185	5.045852813	10.5205304
sp|Q4V7F2|CREL1_RAT C	Protein disulfide isomerase	Creld1	9	27.6	45.696	420	0	61.883	6.976513929	8.352919333	9.191435101	7.598224343	8.119075082	13.11942145
sp|P18163|ACSL1_RAT L	Long-chain-fatty-acid--CoA	Acsl1	42	59.7	78.178	699	0	323.31	405.8570045	416.1948301	372.7571503	388.0099376	391.8835669	383.422788

tr|D3ZDQ9|D3ZDQ9_RAT	Sarcoglycan, alpha (Sarco	Sgca	7	20.9	43.156	387	0	127.32	8.261436775	9.731005415	9.358834845	8.725833803	9.436031665	10.89136513
sp|Q6P7S1|ASAH1_RAT /	Acid ceramidase (AC) (ACD	Asah1	6	19.3	44.443	394	0	31.51	2.44642085	2.973052905	2.979898386	2.97556308	2.434050712	4.434878539
sp|Q07803|EFGM_RAT El	Elongation factor G, mitoch	Gfm1	12	20.2	83.456	751	0	60.206	15.37241354	12.50445301	12.92865725	14.44570446	13.52711792	15.05385794
tr|Q5I0M1|Q5I0M1_RAT A	Beta-2-glycoprotein 1 (Apo	Apoh	14	40.6	38.457	345	0	160.63	43.9854026	59.25967589	48.83407274	57.84970994	43.8656558	68.9976109
tr|B5DF63|B5DF63_RAT S	Sarcolemmal membrane-as	Slmap	10	15.1	86.02	749	0	140.18	14.26209169	13.77938007	12.25311238	12.38518929	14.30316236	16.48233363
tr|A0A0G2K484|A0A0G2K	Myosin-4 (Myosin heavy ch	Myh4	74	29.5	222.69	1938	0	323.31	6727.657338	5435.838855	8516.804987	7256.5969	6028.231237	5084.6013
tr|G3V9W4|G3V9W4_RAT	Xaa-Pro aminopeptidase 3	Xpnpep3	4	13.2	56.484	506	0	6.457	0.219209543	0.281362532	0.330879772	0.297114342	0.226913453	0.503451321
tr|M0RDK9|M0RDK9_RAT	Acyl-CoA dehydrogenase	Acad8	9	27.5	45.155	411	0	55.519	16.29559122	15.17276726	13.75376418	14.44570446	13.19383407	15.51003546
tr|D3ZVN7|D3ZVN7_RAT F	Protoporphyrinogen oxidas	Ppox	3	8.2	50.784	477	0	7.4817	1.745407886	1.992449889	1.991508097	1.672122991	1.790133366	2.002405927
tr|O89035|O89035_RAT M	Mitochondrial dicarboxylate	Slc25a10	3	17.1	31.454	286	0	72.412	1.728600032	1.530554131	1.636126674	1.697028348	1.529276733	1.981578223
tr|D3ZD31|D3ZD31_RAT M	Mannose receptor, C type	Mrc1	18	13.4	164.99	1456	0	117.15	16.2654876	19.9852097	17.67393686	14.77478964	18.76096871	17.09192496
sp|P13852|PRIO_RAT Ma	Major prion protein (PrP) ((	Prnp	2	8.7	27.804	254	0	6.5278	0.814052057	0.970435189	0.954864603	1.006009203	0.865513358	1.010936105
tr|F1LQZ0|F1LQZ0_RAT T	Transmembrane protein 65	Tmem65	8	27	27.733	259	0	72.344	20.24117234	16.43436755	15.98438863	16.82156804	18.2173601	13.70158117
sp|P80432|COX7C_RAT C	Cytochrome c oxidase sub	Cox7c	3	31.7	7.3746	63	0	15.253	72.74539767	63.16392407	58.34750079	60.54928344	61.01035962	63.10286272
sp|P10252|CD48_RAT CD	CD48 antigen (BCM1 surfa	Cd48	4	19.6	27.679	240	0	20.655	3.002334366	3.487021758	2.899857525	3.126010549	4.034082852	3.010466793
tr|F1M1J2|F1M1J2_RAT N	Nebulin	Neb	12	1.9	623.38	5375	0	93.578	19.64160858	15.11106928	21.49119987	17.15961198	16.8076449	18.02358372
tr|B0BMT9|B0BMT9_RAT	Sqrdl protein (Sulfide quin	Sqor	8	26.9	50.201	450	0	38.563	3.884922331	5.007408062	4.063102793	4.487383963	4.246295143	5.107765771
sp|P18886|CPT2_RAT Ca	Carnitine O-palmitoyltransf	Cpt2	34	58.7	74.109	658	0	323.31	147.5378415	137.9912343	142.0107825	127.8056931	122.2580087	154.8158786
tr|D3ZFD2|D3ZFD2_RAT F	Polypeptide N-acetylgalact	Galnt2	3	9.5	53.783	473	0	4.7179	1.057590342	1.068510298	1.135939899	1.242819135	1.212818861	0.987781794
sp|P60203|MYPR_RAT My	Myelin proteolipid protein (	Plp1	3	11.2	30.077	277	0	10.157	1.218343673	1.257651626	1.254812012	1.095357919	1.97112052	1.258806101
tr|D3ZLF0|D3ZLF0_RAT G	GTP-binding protein 6 (put	Gtpbp6	2	5.8	56.551	513	0	12.165	0.252825252	0.31343561	0.277572558	0.260496897	0.253036867	0.53867554
sp|Q7TP48|APMAP_RAT	Adipocyte plasma membra	Apmap	8	23.9	42.062	376	0	26.26	3.116477258	3.757456344	3.559028319	3.316891897	3.550605548	4.247937197
tr|F1LRR4|F1LRR4_RAT W	WD repeat domain 62	Wdr62	2	2.1	169.48	1551	0.0048614	2.405	4.406467547	4.696598317	5.089684007	4.550573095	4.106510071	8.819872187
tr|D4AAE9|D4AAE9_RAT	CDGSH iron sulfur domain	Cisd2	7	45.9	15.293	135	0	64.785	14.83004665	19.74286004	18.86540225	18.08535068	17.78322822	21.3651602
tr|A0A0G2K0Z7|A0A0G2K	Glycerol-3-phosphate dehy	Gpd2	40	73.9	69.061	614	0	323.31	198.5684946	150.0544233	126.3913802	190.0033886	181.3970172	152.7026287
tr|D3ZTT7|D3ZTT7_RAT S	Sad1 and UNC84 domain-	Sun2	5	7.8	81.526	730	0	14.105	7.181218545	8.837618664	8.479757503	8.589660627	7.958904687	9.175263934
sp|Q62920|PDLI5_RAT PI	PDZ and LIM domain prote	Pdlim5	6	11.5	63.201	591	0	89.729	6.448195399	6.938308046	8.288574189	7.564778299	6.788636143	9.007727916
tr|Q642E2|Q642E2_RAT 6	60S ribosomal protein L28	Rpl28	8	40.9	15.733	137	0	31.725	16.81688557	15.05085205	15.76164635	18.16239603	19.92206925	13.63046218
tr|M0R5U4|M0R5U4_RAT	Adenylate cyclase 9 (Aden	Adcy9	3	1.9	151	1354	0	5.1815	0.395345824	0.413638066	0.484727454	0.415967246	0.419004675	0.582952191
tr|A0A096MJV9|A0A096M	Phosphorylase b kinase re	Phka1	15	16.7	132.47	1182	0	174.55	35.42744517	33.97535008	27.76091485	30.23761217	34.07207204	26.62898135
sp|P04639|APOA1_RAT A	Apolipoprotein A-I (Apo-AI	Apoa1	11	51.4	30.062	259	0	91.89	14.87169	13.30899467	12.41776786	14.06167222	15.20755883	13.14990102
tr|D3ZCR3|D3ZCR3_RAT H	High mobility group protein	Hmgb1	4	26.8	24.677	213	0	9.9608	4.667114723	4.068660956	4.588399529	4.860187896	5.820063653	3.86237068
sp|P63039|CH60_RAT 60	60 kDa heat shock protein	Hspd1	46	82.9	60.955	573	0	323.31	313.4439085	283.9735909	283.2623202	305.8939288	266.2172184	380.0192364
tr|F1M5N4|F1M5N4_RAT M	Malic enzyme	Me3	16	34.2	67.208	605	0	116.55	19.58290652	19.77938524	20.0362857	21.46817906	18.21574222	22.36793794
sp|Q64361|LXN_RAT Late	Latexin (Endogenous carb	Lxn	2	12.1	25.58	223	0.0015091	2.7403	0.530877338	0.279901524	0.416381706	0.419043088	0.59753804	0.396325805
tr|F1M8Y4|F1M8Y4_RAT D	DEP domain-containing MT	Deptor	2	4.9	46.226	409	0.0015345	2.9099	1.028440004	0.841461731	0.758421461	0.740770135	0.812446802	0.895459187
sp|Q5PQQ1|GTPB3_RAT	tRNA modification GTPase	Gtpbp3	5	15.7	52.017	492	0	55.397	2.277288678	2.289093777	2.3909806	2.446697519	2.061074802	3.056694136
tr|A0A0G2KA25|A0A0G2K	Transmembrane 9 superfa	Tm9sf4	3	4.7	74.728	643	0	4.9633	1.137716144	1.411945934	1.651311569	1.823645512	1.478097707	1.305439838
tr|Q5U2X8|Q5U2X8_RAT	Acyl-CoA thioesterase 9 (S	Acot9	8	20.3	50.443	439	0	14.11	4.191126318	7.77295832	5.558952141	7.255402399	4.593654576	8.714311288
tr|D3ZA38|D3ZA38_RAT M	Myosin binding protein C, f	Mybpc2	60	56	128.93	1153	0	323.31	181.720502	190.2222763	222.8886422	227.5943523	257.7556915	167.3734601
tr|G3V6E1|G3V6E1_RAT	Myosin heavy chain 2	Myh2	26	14.6	219.57	1916	0	323.31	160.9038488	130.0840212	163.8733719	160.1946025	142.6325459	181.6175775
sp|Q5RK00|RM46_RAT 39	39S ribosomal protein L46	Mrpl46	2	8.7	31.673	277	0	4.3976	0.826896268	0.999260485	1.122904673	0.941207494	0.979789811	1.299343924
tr|G3V936|G3V936_RAT C	Citrate synthase, mitochon	Cs	25	53.6	51.83	466	0	323.31	462.9685889	438.0161717	447.6525274	476.4269422	400.4367738	577.5369878
tr|A0A0G2K5P5|A0A0G2K	Myomesin 1	Myom1	38	28.3	187.6	1688	0	323.31	46.85728795	44.32679038	58.69510681	54.89869387	49.73749171	54.58483984
tr|D3Z8B0|D3Z8B0_RAT C	Cytochrome c oxidase ass	Cox16	3	31.1	12.252	106	0	11.208	3.727781434	4.072609626	3.71700611	3.322386604	4.107480801	3.586936996
sp|P85834|EFTU_RAT Elo	Elongation factor Tu, mitoc	Tufm	32	72.1	49.522	452	0	323.31	244.7675168	237.6507475	222.9389536	224.6917136	210.1899378	246.7219318
sp|Q6MG08|ABCF1_RAT	ATP-binding cassette sub-	Abcf1	5	6.8	95.251	839	0	10.756	1.147750684	0.92640751	0.992918315	0.969099105	1.076377413	1.194697412
tr|Q642E3|Q642E3_RAT C	CDK5 regulatory subunit-a	Cdk5rap3	2	4.2	57.134	504	0.0010852	3.7643	0.229163807	0.309694245	0.259373554	0.189113485	0.311426256	0.214322152
tr|Q5XIU4|Q5XIU4_RAT B	B-cell receptor-associated	Bcap29	11	32.8	28.234	241	0	33.169	9.585494327	12.19448236	13.28175179	9.269929258	10.80745697	12.3493044
tr|Q5M7T5|Q5M7T5_RAT	Antithrombin-III (Serpin C1	Serpinc1	3	11.8	52.233	465	0	17.531	3.755125556	1.739438812	2.374011937	3.532439701	2.90404566	2.684335431
tr|F1M6D0|F1M6D0_RAT M	Mitochondrial ribosomal pro	Mrps6	3	26.4	14.341	125	0	9.0698	4.295987261	4.352817172	3.283413331	3.706896652	3.274325099	4.147354627
tr|Q6IMX8|Q6IMX8_RAT A	Acyl-coenzyme A thioester	Acot2	10	28.5	49.628	453	0	318.69	21.7232739	24.98718834	26.9554751	25.42735444	20.13778696	40.60487863
tr|A0A0G2K7B6|A0A0G2K	Dysferlin	Dysf	88	44.3	242.46	2134	0	323.31	263.4367784	341.6537985	331.95918	241.9343449	245.7348225	362.5544447
sp|Q9WVJ4|SYJ2B_RAT S	Synaptojanin-2-binding pro	Synj2bp	5	51	15.825	145	0	85.94	8.402923789	7.74087537	6.533163763	6.922136467	7.828934771	6.732733065
sp|Q9EPB1|DPP2_RAT D	Dipeptidyl peptidase 2 (EC	Dpp7	7	17.4	55.114	500	0	48.223	6.172245549	5.205828766	6.158572534	5.910154756	5.397796247	7.640719354
sp|Q63704|CPT1B_RAT C	Carnitine O-palmitoyltransf	Cpt1b	29	44	88.216	772	0	323.31	103.2503992	114.2547874	94.33844566	88.81716255	99.67236511	108.5986958
sp|Q641Y2|NDUS2_RAT N	NADH dehydrogenase [ub	Ndufs2	35	74.1	52.561	463	0	323.31	366.7524022	360.9726701	324.6182896	380.0784474	351.9326481	350.2711794
tr|A0A0G2JYY6|A0A0G2J	Coenzyme Q10A	Coq10a	9	53.4	23.331	204	0	61.653	31.09503253	38.87120819	37.2318069	35.28915921	34.00627814	46.3665327
tr|D3ZDX7|D3ZDX7_RAT M	Mitochondrial ribosomal pro	Mrpl48	4	17.1	24.019	211	0	8.9966	2.43563372	2.340229063	1.927200982	2.076999291	2.154588427	2.163033243
tr|D3ZXK4|D3ZXK4_RAT A	Abhydrolase domain-conta	Abhd11	4	20.2	33.215	307	0	90.584	8.009569822	6.34896894	5.834750079	6.313537929	7.820306062	7.516362722
tr|Q7TP91|Q7TP91_RAT A	Surfeit locus protein 1	Surf1	6	14.2	83.262	733	0	93.388	6.633834389	6.043934127	6.287095288	6.419251315	6.568604085	6.324103675
tr|M0RAD5|M0RAD5_RAT	ATP-dependent Clp protea	Clpp	5	25	29.722	272	0	135.71	6.619786033	7.107607303	6.325057525	6.745947489	6.023377588	9.142346002
sp|Q6TEK3|VKORL_RAT	Vitamin K epoxide reducta	Vkorc1l1	3	15.9	19.778	176	0	7.6988	3.165245122	3.9500034	3.96645917	3.474924451	4.260963947	3.973519499
sp|P63031|MPC1_RAT Mi	Mitochondrial pyruvate car	Mpc1	4	40.4	12.454	109	0	6.528	83.5425627	64.27942355	52.54339492	62.06032789	74.13138896	40.48905627
tr|B5DF57|B5DF57_RAT E	Erythrocyte membrane pro	Epb42	21	30.2	76.5	689	0	269.98	36.84231531	42.86676938	35.45627192	39.96802177	52.58388681	33.60270619
sp|Q2TA68|OPA1_RAT Dy	Dynamin-like 120 kDa prot	Opa1	35	40.4	111.31	960	0	323.31	78.22425651	71.59433606	66.88214345	75.6537555	75.69534232	71.7895392
tr|B0BNJ4|B0BNJ4_RAT E	ETHE1, persulfide dioxyge	Ethe1	6	39.8	27.676	254	0	46.947	10.70284036	11.12241826	12.6240446	11.44213032	10.01469441	17.02690189

sp|P70584|ACDSB_RAT S	Short/branched chain spec	Acadsb	12	40	47.823	432	0	167.06	14.43669269	14.77493868	24.92015035	13.3557218	18.79224778	15.29871046
sp|Q6PCT8|DHSD_RAT S	Succinate dehydrogenase	Sdhd	2	5	16.976	159	0.0015448	2.9584	32.88569619	33.80210215	33.59703706	33.25850658	35.04495889	28.1448318
tr|D3ZQP6|D3ZQP6_RAT	Sema domain, immunoglob	Sema7a	4	10.1	75.143	666	0	17.41	1.817606401	2.075569408	2.421899241	2.335907501	2.129457315	2.177765034
tr|F1M983|F1M983_RAT C	Complement factor H	Cfh	2	2.4	140.01	1234	0	5.5016	1.662823622	1.694966908	1.705647879	1.630972413	1.516495459	2.4544179
tr|E9PT51|E9PT51_RAT D	DNA polymerase delta-inte	Poldip2	6	20.4	41.797	368	0	14.24	4.602893667	5.526658262	6.006723586	4.811511958	4.68344707	5.752205407
sp|P07632|SODC_RAT Su	Superoxide dismutase [Cu	Sod1	6	40.9	15.911	154	0	47.431	18.93818733	18.34897927	25.20097943	18.63243239	24.747135	23.95693935
tr|F1LSP2|F1LSP2_RAT A	Acyl-CoA dehydrogenase	Acad10	7	7.3	119.19	1069	0	21.429	1.977807832	2.040475597	2.017532811	2.121195848	1.820927068	2.387362853
tr|F1LNV5|F1LNV5_RAT C	Calcium uptake protein 1,	Micu1	6	13.2	54.226	477	0	21.42	5.784410578	5.699412606	5.282696826	4.343446526	4.982863243	6.361288747
tr|B0BMW2|B0BMW2_RAT	3-hydroxyacyl-CoA dehydr	Hsd17b10	19	79.3	27.249	261	0	323.31	68.28002738	74.0671911	73.73821492	73.52157024	65.0712454	91.46105148
sp|Q62658|FKB1A_RAT P	Peptidyl-prolyl cis-trans iso	Fkbp1a	2	25	11.922	108	0	117.23	7.933809045	8.753709411	10.38838901	9.354141617	9.214921018	9.801517429
tr|F1LM10|F1LM10_RAT P	Protein kinase C theta type	Prkcq	9	17	81.777	707	0	70.262	10.08872651	9.761114029	10.10435829	9.969309914	10.39597545	8.655485725
sp|P01946|HBA_RAT Hem	Hemoglobin subunit alpha	Hba1	2	7.7	15.328	142	0	323.31	139.2392769	148.0109862	120.5095203	120.5550687	197.5596662	132.7181928
sp|Q7TT47|SPG7_RAT Pa	Paraplegin (EC 3.4.24.-)	Spg7	9	13.1	86.102	781	0	108.73	3.49894375	3.745758407	3.430185401	3.248148333	3.607231445	3.576675542
sp|Q6PDU7|ATP5L_RAT A	ATP synthase subunit g, m	Atp5mg	8	65	11.433	103	0	135.58	75.4998789	77.44824041	82.29115264	81.63820825	68.03736383	103.5594075
tr|A0A0G2K9Y5|A0A0G2K	Histidine-rich glycoprotein (	Hrg	5	9.3	57.926	515	0	13.305	9.105843316	7.033076143	11.52039547	8.848270212	9.032639559	12.60838072
tr|D4A9W3|D4A9W3_RAT	D-glutamate cyclase	Dglucy	5	7.9	66.055	618	0	19.321	0.669303817	0.761402432	0.806308765	0.704158662	0.659826527	1.139630995
tr|F1LM47|F1LM47_RAT S	Succinate--CoA ligase [AD	Sucla2	30	61.5	50.306	465	0	323.31	401.6274459	417.3202013	371.1746282	412.8973774	363.6407265	467.6987896
sp|P62632|EF1A2_RAT E	Elongation factor 1-alpha 2	Eef1a2	13	29.4	50.454	463	0	299.78	176.2165568	174.4670801	145.7018096	182.8244343	263.9198249	121.2985152
tr|D4ADG2|D4ADG2_RAT	Iron-sulfur cluster assembly	Iba57	6	27.4	38.673	358	0	117.29	2.86787153	3.226754999	3.08386003	3.125950824	2.50006033	4.653112236
sp|P15999|ATPA_RAT AT	ATP synthase subunit alph	Atp5f1a	52	68.9	59.753	553	0	323.31	4300.502804	3522.70787	3323.799663	4326.245704	3885.399425	3530.041805
tr|D4ACG2|D4ACG2_RAT	2-hydroxyacyl-CoA lyase 2	Ilvbl	4	11.2	72.269	672	0	14.889	2.339854035	2.211946623	2.151498344	2.30037108	2.108694486	2.493228548
tr|A0A0G2JY53|A0A0G2J	Myosin, heavy chain 15	Myh15	2	1	220.29	1915	0.0093284	2.0737	4.567371395	3.880309362	3.504005944	4.209304003	3.621414884	3.527908235
tr|D3ZQN6|D3ZQN6_RAT	SH3 and cysteine rich dom	Stac3	7	18.3	41.086	361	0	22.485	4.819388867	5.817872728	5.029310329	5.513938593	6.77677167	4.528552407
tr|Q99JC6|Q99JC6_RAT T	TAP-binding protein (Tap-b	Tapbp	2	5.6	50.044	464	0	5.9713	1.012535258	1.7116994	1.773659742	1.193486221	1.900203325	1.936163669
sp|O88177|PEX12_RAT P	Peroxisome assembly prot	Pex12	2	6.7	40.681	359	0.00054855	3.863	1.383311511	1.399705055	1.002431743	0.974593812	1.45808989	1.048395493
sp|P40241|CD9_RAT CD9	CD9 antigen (CD antigen C	Cd9	3	15.5	25.215	226	0	91.693	1.775611852	2.593931157	3.532454753	2.123405676	2.623612645	4.534038729
tr|A0A0H2UI06|A0A0H2UI	NADH dehydrogenase (ub	Ndufaf6	3	11.9	38.124	329	0	7.7981	4.641125264	4.945216498	4.146665451	3.993397848	4.523114887	4.731647919
sp|P11960|ODBA_RAT 2-	2-oxoisovalerate dehydrog	Bckdha	11	36.1	50.164	441	0	126.11	16.32720002	16.93831665	18.16698857	17.35013498	15.13637199	22.60263061
tr|B4F7C9|B4F7C9_RAT S	Dolichyl-diphosphooligosac	Stt3a	3	4.4	80.597	705	0	4.5279	1.24764453	1.248569683	1.160272321	1.091416064	1.323967408	1.116568123
tr|A0A0G2KAM4|A0A0G2I	Fascin	Fscn1	2	5.3	53.881	492	0	5.6423	1.814295003	1.535736761	1.303065217	1.834754377	2.271561352	1.117888904
sp|P21913|SDHB_RAT Su	Succinate dehydrogenase	Sdhb	18	50.7	31.83	282	0	323.31	495.8718455	522.0142697	466.5696132	539.4906514	458.0873302	533.2806571
sp|Q5XIE6|HIBCH_RAT 3-	3-hydroxyisobutyryl-CoA hy	Hibch	12	35.6	43.024	385	0	81.978	23.07141435	24.13476905	23.75155378	23.51734646	20.25373522	33.64334561
tr|A0A0G2K913|A0A0G2K	5'-AMP-activated protein k	Prkag1	3	7.9	37.296	329	0	9.8549	3.322285673	3.430111541	3.20744312	3.03403393	4.28981619	3.25755448
tr|Q3KRE2|Q3KRE2_RAT	Methyltransferase like 7A,	Mettl7a	6	31.1	28.142	244	0	38.718	7.866577627	8.022711743	10.57774281	8.546061321	8.586643206	11.15856933
sp|Q6AYQ4|TM109_RAT T	Transmembrane protein 10	Tmem109	3	12.3	26.242	243	0	41.755	12.08309133	14.00840298	12.93002937	12.88807445	13.50284968	11.38716608
sp|P62246|RS15A_RAT 4	40S ribosomal protein S15	Rps15a	6	40	14.839	130	0	9.7411	12.82514556	8.765555423	12.71551987	11.56038598	19.31536321	8.701611469
sp|Q3KRE0|ATAD3_RAT A	ATPase family AAA domai	Atad3	15	27.4	66.758	591	0	323.31	15.193297	15.08639008	11.89635883	12.93943801	14.53505889	12.87050499
tr|B3DM90|B3DM90_RAT	Nicastrin	Ncstn	2	3.3	67.169	602	0.0010684	3.4409	0.531780447	0.529566102	0.491816788	0.457948003	0.572838363	0.460790088
tr|Q5U2Q8|Q5U2Q8_RAT	Aspartate-beta-hydroxylas	Asph	8	24.6	22.634	207	0	42.846	90.61189613	136.9892591	160.1000171	103.2228512	98.97128257	147.7039797
tr|G3V648|G3V648_RAT 1	1-acylglycerol-3-phosphate	Agpat3	2	5.3	43.245	376	0	5.149	1.871592227	1.224433433	1.129765319	1.473298209	2.174704103	1.137497426
sp|P19643|AOFB_RAT Ar	Amine oxidase [flavin-cont	Maob	3	5.8	58.458	520	0	5.7103	3.893250999	6.135740721	4.816630327	4.219516991	3.585713604	5.741029566
sp|P83883|RL36A_RAT 6	60S ribosomal protein L36	Rpl36a	4	26.4	12.441	106	0	7.9713	4.123343001	3.817722931	4.121509752	4.567415567	5.346455433	3.142951309
tr|M0RAM5|M0RAM5_RAT	Glutathione peroxidase 1 (	Gpx1	4	28.5	22.155	200	0	17.726	3.133285112	3.487515342	3.854539178	3.256330668	3.086758555	5.18518387
sp|Q68FQ0|TCPE_RAT T-	T-complex protein 1 subun	Cct5	10	24.2	59.536	541	0	69.041	5.606297494	4.775226223	5.040287361	5.113123599	6.717988595	4.595810651
sp|Q9ES71|GNPAT_RAT	Dihydroxyacetone phosph	Gnpat	4	8.1	77.075	678	0	54.098	3.080603778	2.804839532	2.901138179	3.25388194	3.191165924	2.646439169
sp|Q641X9|RM09_RAT 39	39S ribosomal protein L9,	Mrpl9	6	21.4	30.076	262	0	11.74	3.664262796	3.699805752	3.186586758	3.40044728	3.514257115	3.970471543
sp|P19527|NFL_RAT Neu	Neurofilament light polype	Nefl	2	3.7	61.335	542	0	7.8832	1.169074082	0.910563469	1.207565036	1.04208315	2.127192279	0.751991867
tr|F1LP21|F1LP21_RAT T	Mitochondrial import inner	Timm8a	3	34.7	10.888	95	0	46.669	3.02732037	2.622805812	2.616329927	2.239332051	2.508581179	3.107493413
tr|D3ZG43|D3ZG43_RAT N	Complex I-30kD (NADH de	Ndufs3	23	56.4	30.226	264	0	323.31	223.2133249	229.4868708	200.8248071	239.7125705	206.3879133	225.396395
sp|Q99M64|P4K2A_RAT P	Phosphatidylinositol 4-kina	Pi4k2a	3	6.9	54.304	478	0	6.5158	2.087334836	2.291512338	2.404153039	2.123883476	2.305860467	2.578063342
tr|D4A833|D4A833_RAT M	Mitochondrial ribosomal pr	Mrps30	5	17.3	50.001	440	0	76.548	3.086172947	3.832184938	3.285837426	3.576337633	2.706610031	4.896237579
sp|P0C2C0|RM22_RAT 39	39S ribosomal protein L22	Mrpl22	2	13.6	24.039	206	0	15.481	0.336347746	0.413845371	0.329882691	0.351308878	0.349953438	0.338526386
tr|B2RZD1|B2RZD1_RAT P	Protein transport protein S	Sec61b	3	37.5	9.9884	96	0	6.3936	3.669079375	3.223546704	4.184947852	3.97577895	4.245594061	3.376932782
sp|Q9WV97|TIM9_RAT Mi	Mitochondrial import inner	Timm9	5	59.6	10.376	89	0	17.838	7.139073477	6.692996877	7.12729564	6.747739241	6.840408392	7.974978601
sp|P62074|TIM10_RAT Mi	Mitochondrial import inner	Timm10	4	52.2	10.333	90	0	13.469	4.583978559	4.393784631	3.839262808	4.281631071	4.069568414	4.935861015
sp|Q5FWU3|ATG9A_RAT	Autophagy-related protein	Atg9a	2	2.3	94.487	839	0.00054585	3.8267	0.314422276	0.397754538	0.280001227	0.2740306	0.351058991	0.309855274
sp|Q5XIP9|TMM43_RAT T	Transmembrane protein 43	Tmem43	9	31.2	44.774	400	0	35.708	8.50979164	13.04838241	11.05021258	8.525754794	9.895510374	11.94189419
sp|P02401|RLA2_RAT 60	60S acidic ribosomal prote	Rplp2	9	92.2	11.692	115	0	319.67	26.73351972	23.39883554	29.5332482	26.18884917	34.64642043	23.94982745
tr|Q6P685|Q6P685_RAT E	Eukaryotic translation initia	Eif2s2	3	10.2	38.243	333	0	14.568	3.370501638	2.515253893	2.563594434	3.063597844	4.251094862	2.084294363
sp|Q68FY0|QCR1_RAT Cy	Cytochrome b-c1 complex	Uqcrc1	26	56.9	52.848	480	0	323.31	883.3907282	918.8556774	810.928266	903.1028949	812.0153671	842.302823
sp|P43278|H10_RAT Histo	Histone H1.0 (Histone H1')	H1-0	4	21.1	20.885	194	0	7.8565	4.900016396	5.094772401	5.640822507	4.900920399	6.520606896	4.997632937
sp|Q4QQV3|F162A_RAT P	Protein FAM162A (E2-indu	Fam162a	8	37.4	17.827	155	0	214.1	36.53576011	38.54149418	30.29752408	34.19439855	33.45296223	34.18283394
tr|A0A096MK54|A0A096M	RCG24674, isoform CRA_	Synm	12	12.4	140.76	1255	0	122.9	4.399443369	4.285492337	4.762202541	3.905064459	4.809156562	4.333686378
tr|A0A0G2JWU3|A0A0G2J	Mitochondrial ribosomal pr	LOC1003596	4	14.4	34.926	312	0	24.245	3.656837237	3.677002178	3.448068817	3.215598165	3.465397055	3.836361449
sp|Q5U2X7|TIM21_RAT M	Mitochondrial import inner	Timm21	9	32.7	27.876	245	0	80.308	15.53095927	14.83614308	13.5753874	15.0955133	13.89977025	16.0556197
sp|P57093|PAHX_RAT Ph	Phytanoyl-CoA dioxygenas	Phyh	4	10.9	38.588	338	0	17.228	3.33598282	4.081296702	2.698291768	2.773513143	3.706245872	2.991975856
sp|P49432|ODPB_RAT Py	Pyruvate dehydrogenase E	Pdhb	20	50.7	38.982	359	0	323.31	267.9573387	270.8195816	250.3861083	241.8029482	208.8794527	300.4269281

<table>
<tr><td>sp|Q99NA5|IDH3A_RAT Is</td><td>Isocitrate dehydrogenase</td><td>Idh3a</td><td>25</td><td>54.6</td><td>39.613</td><td>366</td><td>0</td><td>323.31</td><td>356.2913942</td><td>348.5935873</td><td>299.1835909</td><td>350.2457712</td><td>304.1026402</td><td>389.752378</td></tr>
<tr><td>tr|A0A0G2K261|A0A0G2K</td><td>Isoleucyl-tRNA synthetase</td><td>Iars2</td><td>21</td><td>26.3</td><td>112.68</td><td>1011</td><td>0</td><td>323.31</td><td>27.86692101</td><td>26.00643897</td><td>26.74874099</td><td>26.19243268</td><td>25.86563129</td><td>30.88900878</td></tr>
<tr><td>sp|B0K020|CISD1_RAT C</td><td>CDGSH iron-sulfur domain-</td><td>Cisd1</td><td>10</td><td>62</td><td>12.097</td><td>108</td><td>0</td><td>113.68</td><td>55.00934824</td><td>66.96451964</td><td>53.25232827</td><td>49.05041429</td><td>56.70679143</td><td>62.04420577</td></tr>
<tr><td>tr|A0A0G2K7E5|A0A0G2K</td><td>Voltage-dependent calcium</td><td>Cacna2d1</td><td>46</td><td>42.3</td><td>122.73</td><td>1084</td><td>0</td><td>323.31</td><td>231.5369758</td><td>295.7208864</td><td>338.9844808</td><td>264.43278</td><td>230.462009</td><td>319.5985755</td></tr>
<tr><td>tr|F1LM84|F1LM84_RAT N</td><td>Nidogen-1 (NID-1) (Entacti</td><td>Nid1</td><td>2</td><td>1.5</td><td>137.04</td><td>1246</td><td>0.0015129</td><td>2.7746</td><td>0.835174764</td><td>1.253801672</td><td>0.872033746</td><td>0.678894954</td><td>1.055291008</td><td>1.621919337</td></tr>
<tr><td>sp|Q920A6|RISC_RAT Re</td><td>Retinoid-inducible serine c</td><td>Scpep1</td><td>5</td><td>13.9</td><td>51.174</td><td>452</td><td>0</td><td>46.976</td><td>1.192755596</td><td>0.687957157</td><td>0.918000069</td><td>0.850664277</td><td>1.271817653</td><td>0.920066357</td></tr>
<tr><td>sp|P24049|RL17_RAT 60S</td><td>60S ribosomal protein L17</td><td>Rpl17</td><td>6</td><td>29.3</td><td>21.397</td><td>184</td><td>0</td><td>177.46</td><td>25.63373414</td><td>20.62736228</td><td>23.19950053</td><td>24.94597031</td><td>29.69462054</td><td>19.12389607</td></tr>
<tr><td>sp|A2VD12|PBIP1_RAT P</td><td>Pre-B-cell leukemia transcr</td><td>Pbxip1</td><td>23</td><td>39.8</td><td>80.275</td><td>722</td><td>0</td><td>323.31</td><td>41.18476249</td><td>47.87022877</td><td>50.9059876</td><td>42.87185504</td><td>45.56551131</td><td>58.47098458</td></tr>
<tr><td>tr|A0A0G2K471|A0A0G2K</td><td>Interferon gamma-induced</td><td>Igtp</td><td>2</td><td>5.7</td><td>50.437</td><td>442</td><td>0</td><td>6.384</td><td>0.956943906</td><td>0.965005767</td><td>0.833842821</td><td>0.966351752</td><td>1.027031988</td><td>0.845015504</td></tr>
<tr><td>tr|A0A0G2JU83|A0A0G2J</td><td>Delta(14)-sterol reductase</td><td>Lbr</td><td>2</td><td>2.9</td><td>70.619</td><td>620</td><td>0.0015674</td><td>3.1275</td><td>0.368448239</td><td>0.367675539</td><td>0.365471145</td><td>0.310200107</td><td>0.432918467</td><td>0.305120781</td></tr>
<tr><td>tr|B2RZD4|B2RZD4_RAT 6</td><td>60S ribosomal protein L34</td><td>Rpl34</td><td>3</td><td>20.5</td><td>13.293</td><td>117</td><td>0</td><td>9.1435</td><td>11.84226237</td><td>11.31935821</td><td>12.2082895</td><td>13.07202768</td><td>15.11911458</td><td>9.319940277</td></tr>
<tr><td>tr|D4A4P3|D4A4P3_RAT N</td><td>Complex I-B12 (NADH deh</td><td>Ndufb3</td><td>5</td><td>30.3</td><td>11.267</td><td>99</td><td>0</td><td>52.509</td><td>141.767981</td><td>133.2725728</td><td>118.5656708</td><td>155.2852012</td><td>132.4129196</td><td>120.4044479</td></tr>
<tr><td>sp|P21775|THIKA_RAT 3-</td><td>3-ketoacyl-CoA thiolase A,</td><td>Acaa1a</td><td>8</td><td>27.6</td><td>43.833</td><td>424</td><td>0</td><td>141.17</td><td>6.580651327</td><td>8.385002282</td><td>7.494111471</td><td>6.673082894</td><td>6.985478548</td><td>10.34273293</td></tr>
<tr><td>sp|Q63514|C4BPA_RAT C</td><td>C4b-binding protein alpha</td><td>C4bpa</td><td>6</td><td>11.3</td><td>62.265</td><td>558</td><td>0</td><td>9.4642</td><td>3.824865609</td><td>5.4827293</td><td>4.069826225</td><td>3.844204605</td><td>4.124037135</td><td>4.723418436</td></tr>
<tr><td>tr|B2RYW3|B2RYW3_RAT</td><td>Complex I-B22 (NADH deh</td><td>Ndufb9</td><td>14</td><td>67</td><td>21.892</td><td>179</td><td>0</td><td>323.31</td><td>162.0578209</td><td>165.8737854</td><td>134.4503514</td><td>163.9274199</td><td>139.898324</td><td>144.1886697</td></tr>
<tr><td>sp|B0BNM1|NNRE_RAT N</td><td>NAD(P)H-hydrate epimeras</td><td>Naxe</td><td>6</td><td>22.7</td><td>30.89</td><td>282</td><td>0</td><td>20.304</td><td>6.55004598</td><td>6.977794753</td><td>7.959263218</td><td>6.652179117</td><td>6.01852394</td><td>10.6404167</td></tr>
<tr><td>tr|M0R5K3|M0R5K3_RAT</td><td>Cytochrome c oxidase sub</td><td>LOC687508</td><td>2</td><td>18.8</td><td>9.0274</td><td>80</td><td>0</td><td>26.276</td><td>18.5122211</td><td>14.35687317</td><td>9.497877255</td><td>12.39892606</td><td>15.46534149</td><td>18.09470271</td></tr>
<tr><td>tr|Q6P722|Q6P722_RAT P</td><td>Popeye domain-containing</td><td>Popdc2</td><td>2</td><td>5.7</td><td>41.342</td><td>368</td><td>0.00054795</td><td>3.8588</td><td>0.75018221</td><td>0.809674932</td><td>0.775115698</td><td>0.713057699</td><td>0.809912119</td><td>0.868078376</td></tr>
<tr><td>sp|O35547|ACSL4_RAT L</td><td>Long-chain-fatty-acid--CoA</td><td>Acsl4</td><td>6</td><td>13.9</td><td>74.326</td><td>670</td><td>0</td><td>27.85</td><td>4.519255776</td><td>6.026165108</td><td>5.257541127</td><td>4.825666801</td><td>4.640465318</td><td>5.723656213</td></tr>
<tr><td>sp|P54313|GBB2_RAT Gu</td><td>Guanine nucleotide-bindin</td><td>Gnb2</td><td>5</td><td>21.8</td><td>37.331</td><td>340</td><td>0</td><td>323.31</td><td>18.6496943</td><td>19.48817077</td><td>19.08128389</td><td>16.6716581</td><td>21.36036704</td><td>17.46377568</td></tr>
<tr><td>sp|Q63362|NDUA5_RAT N</td><td>NADH dehydrogenase [ub</td><td>Ndufa5</td><td>13</td><td>74.1</td><td>13.412</td><td>116</td><td>0</td><td>323.31</td><td>241.3658077</td><td>258.8156226</td><td>233.0515447</td><td>264.7373779</td><td>243.5345019</td><td>237.3647048</td></tr>
<tr><td>tr|A0A0H2UI21|A0A0H2UI</td><td>Carnitine O-acetyltransfera</td><td>Crat</td><td>28</td><td>47.1</td><td>71.013</td><td>626</td><td>0</td><td>323.31</td><td>202.180929</td><td>257.1917318</td><td>249.1191758</td><td>224.7872737</td><td>209.9310765</td><td>315.4431946</td></tr>
<tr><td>tr|G3V8S6|G3V8S6_RAT</td><td>Histidine rich calcium bindi</td><td>Hrc</td><td>4</td><td>7.4</td><td>86.502</td><td>754</td><td>0</td><td>113.72</td><td>25.57754071</td><td>20.87514137</td><td>23.32665115</td><td>21.6933426</td><td>27.80115839</td><td>22.80887567</td></tr>
<tr><td>sp|Q63584|TMEDA_RAT T</td><td>Transmembrane emp24 do</td><td>Tmed10</td><td>6</td><td>35.2</td><td>24.857</td><td>219</td><td>0</td><td>38.467</td><td>21.35099247</td><td>24.68857012</td><td>21.14359384</td><td>21.64377079</td><td>24.61123284</td><td>22.49900008</td></tr>
<tr><td>tr|Q5RK08|Q5RK08_RAT</td><td>Glioblastoma amplified seq</td><td>Nipsnap2</td><td>15</td><td>47.3</td><td>32.941</td><td>281</td><td>0</td><td>323.31</td><td>154.7125376</td><td>153.2972692</td><td>141.4756521</td><td>152.7409129</td><td>136.6355937</td><td>172.6667448</td></tr>
<tr><td>sp|Q561R9|LACB2_RAT E</td><td>Endoribonuclease LACTB2</td><td>Lactb2</td><td>2</td><td>6.6</td><td>32.484</td><td>288</td><td>0.0015083</td><td>2.74</td><td>0.938480353</td><td>0.842300824</td><td>0.733448713</td><td>0.674236398</td><td>0.788124631</td><td>0.945313598</td></tr>
<tr><td>tr|F7FHT4|F7FHT4_RAT S</td><td>Semaphorin-3C</td><td>Sema3c</td><td>4</td><td>6.9</td><td>85.962</td><td>757</td><td>0</td><td>27.938</td><td>2.134697865</td><td>2.205579391</td><td>3.116059325</td><td>2.334712999</td><td>2.284989779</td><td>3.339646112</td></tr>
<tr><td>tr|G3V8B0|G3V8B0_RAT</td><td>Myosin-7 (Myosin heavy ch</td><td>Myh7</td><td>40</td><td>21.7</td><td>222.9</td><td>1935</td><td>0</td><td>323.31</td><td>391.0008681</td><td>309.0081633</td><td>473.0643573</td><td>427.8167017</td><td>317.4070296</td><td>504.8127419</td></tr>
<tr><td>tr|A0A0G2JXC3|A0A0G2J</td><td>40S ribosomal protein S21</td><td>Rps21</td><td>3</td><td>43.2</td><td>8.866</td><td>81</td><td>0</td><td>6.8124</td><td>1.629910331</td><td>1.438105878</td><td>1.434195016</td><td>1.49778549</td><td>1.410470207</td><td>1.710818073</td></tr>
<tr><td>tr|A0A0G2JT93|A0A0G2J</td><td>Catenin beta-1 (Beta-cater</td><td>Ctnnb1</td><td>2</td><td>3.2</td><td>85.47</td><td>781</td><td>0.0010799</td><td>3.6952</td><td>0.081214549</td><td>0.051915148</td><td>0.059536679</td><td>0.081936834</td><td>0.106300292</td><td>0.0306279</td></tr>
<tr><td>sp|P56574|IDHP_RAT Iso</td><td>Isocitrate dehydrogenase</td><td>Idh2</td><td>34</td><td>64.2</td><td>50.967</td><td>452</td><td>0</td><td>323.31</td><td>963.1151484</td><td>1306.812576</td><td>1338.694835</td><td>832.8662039</td><td>773.3479687</td><td>1663.168351</td></tr>
<tr><td>tr|G3V6H0|G3V6H0_RAT</td><td>Ras-related protein Rab-1B</td><td>Rab1b</td><td>8</td><td>31.3</td><td>22.177</td><td>201</td><td>0</td><td>237.17</td><td>55.84221506</td><td>56.45611967</td><td>55.24191538</td><td>57.69800824</td><td>61.99726811</td><td>51.33775001</td></tr>
<tr><td>sp|Q3BCU4|POPD1_RAT</td><td>Blood vessel epicardial sub</td><td>Bves</td><td>3</td><td>11.2</td><td>40.86</td><td>356</td><td>0</td><td>6.4088</td><td>2.263089804</td><td>2.593141423</td><td>2.129086903</td><td>1.698043675</td><td>2.400776257</td><td>2.566379508</td></tr>
<tr><td>tr|D3Z946|D3Z946_RAT S</td><td>Similar to RIKEN cDNA 221</td><td>Tmem70</td><td>3</td><td>10.7</td><td>28.399</td><td>253</td><td>0</td><td>5.4702</td><td>6.587675505</td><td>5.230507958</td><td>5.271719794</td><td>6.175573</td><td>7.227082377</td><td>4.616638354</td></tr>
<tr><td>tr|D3ZFJ6|D3ZFJ6_RAT La</td><td>Lactamase, beta (Lactama</td><td>Lactb</td><td>14</td><td>28.5</td><td>60.42</td><td>550</td><td>0</td><td>286.55</td><td>9.787188581</td><td>11.30553787</td><td>9.919578248</td><td>9.447312738</td><td>8.387643624</td><td>11.93986222</td></tr>
<tr><td>sp|P97546|NPTN_RAT Ne</td><td>Neuroplastin (Glycoprotein</td><td>Nptn</td><td>3</td><td>5.1</td><td>43.931</td><td>393</td><td>0</td><td>5.5684</td><td>3.951601849</td><td>3.957851383</td><td>3.428584584</td><td>2.985417717</td><td>4.106833648</td><td>3.841644574</td></tr>
<tr><td>sp|Q6AXX6|PXL2A_RAT P</td><td>Peroxiredoxin-like 2A (Per</td><td>Prxl2a</td><td>7</td><td>27.9</td><td>25.763</td><td>229</td><td>0</td><td>21.228</td><td>7.621734851</td><td>11.45015793</td><td>11.37129078</td><td>9.218565692</td><td>7.629935189</td><td>11.78949636</td></tr>
<tr><td>sp|P16970|ABCD3_RAT A</td><td>ATP-binding cassette sub-</td><td>Abcd3</td><td>4</td><td>6.5</td><td>75.315</td><td>659</td><td>0</td><td>10.69</td><td>3.183257122</td><td>3.379864706</td><td>2.826814522</td><td>3.110243129</td><td>3.951085466</td><td>2.744278578</td></tr>
<tr><td>sp|P27615|SCRB2_RAT L</td><td>Lysosome membrane prote</td><td>Scarb2</td><td>4</td><td>8.6</td><td>54.09</td><td>478</td><td>0</td><td>42.223</td><td>13.11915759</td><td>16.27691431</td><td>14.22120281</td><td>12.61274184</td><td>14.7594053</td><td>18.20646112</td></tr>
<tr><td>sp|P20760|IGG2A_RAT Ig</td><td>Ig gamma-2A chain C regi</td><td>Igg-2a</td><td>9</td><td>36.3</td><td>35.185</td><td>322</td><td>0</td><td>34.849</td><td>7.655852287</td><td>13.00938928</td><td>20.22518213</td><td>11.89604091</td><td>8.917769881</td><td>26.95206476</td></tr>
<tr><td>tr|D3ZCV0|D3ZCV0_RAT A</td><td>Actinin alpha 2 (RCG3055</td><td>Actn2</td><td>28</td><td>37.9</td><td>103.83</td><td>894</td><td>0</td><td>323.31</td><td>58.54652358</td><td>45.68019727</td><td>55.29680054</td><td>49.73725268</td><td>57.14361978</td><td>57.29244134</td></tr>
<tr><td>tr|G3V640|G3V640_RAT M</td><td>Mitochondrial import inner</td><td>Timm44</td><td>19</td><td>35.8</td><td>51.06</td><td>453</td><td>0</td><td>176.24</td><td>30.87778474</td><td>20.85737235</td><td>19.61824372</td><td>23.31487845</td><td>26.12125677</td><td>25.7745375</td></tr>
<tr><td>tr|A0A0G2JXY6|A0A0G2J</td><td>Sodium channel subunit b</td><td>Scn1b</td><td>4</td><td>19.5</td><td>21.426</td><td>185</td><td>0</td><td>6.6737</td><td>3.427447652</td><td>2.945165418</td><td>2.531303664</td><td>2.792923793</td><td>2.887327538</td><td>3.575151564</td></tr>
<tr><td>sp|P25886|RL29_RAT 60S</td><td>60S ribosomal protein L29</td><td>Rpl29</td><td>6</td><td>32.1</td><td>17.325</td><td>156</td><td>0</td><td>14.223</td><td>14.18181537</td><td>14.17770223</td><td>16.90828885</td><td>19.21296014</td><td>19.39463947</td><td>9.982261259</td></tr>
<tr><td>tr|Q66H18|Q66H18_RAT S</td><td>Synaptophysin-like 1 (Syna</td><td>Sypl1</td><td>2</td><td>10.5</td><td>28.628</td><td>258</td><td>0</td><td>17.514</td><td>2.296705513</td><td>3.654988339</td><td>3.114595721</td><td>3.118664365</td><td>2.947944213</td><td>3.426004884</td></tr>
<tr><td>tr|D3ZXA6|D3ZXA6_RAT P</td><td>Pyruvate dehydrogenase p</td><td>Pdpr</td><td>22</td><td>26.2</td><td>98.818</td><td>878</td><td>0</td><td>165.67</td><td>38.47443324</td><td>35.51533166</td><td>36.88648776</td><td>34.86152766</td><td>31.69378436</td><td>50.37053176</td></tr>
<tr><td>sp|Q68FX0|IDH3B_RAT Is</td><td>Isocitrate dehydrogenase</td><td>Idh3B</td><td>15</td><td>43.4</td><td>42.353</td><td>385</td><td>0</td><td>225.78</td><td>74.83759926</td><td>75.04448711</td><td>64.11501654</td><td>67.35196975</td><td>64.63441705</td><td>76.98427333</td></tr>
<tr><td>sp|P23711|HMOX2_RAT H</td><td>Heme oxygenase 2 (HO-2)</td><td>Hmox2</td><td>7</td><td>33.7</td><td>35.762</td><td>315</td><td>0</td><td>21.774</td><td>8.343218276</td><td>9.28826071</td><td>8.786657033</td><td>8.215184392</td><td>9.488343208</td><td>9.1705904</td></tr>
<tr><td>sp|Q7TS56|CBR4_RAT Ca</td><td>Carbonyl reductase family</td><td>Cbr4</td><td>5</td><td>32.6</td><td>25.286</td><td>236</td><td>0</td><td>27.058</td><td>3.884219913</td><td>4.71362696</td><td>4.114237468</td><td>4.320094021</td><td>3.399009932</td><td>5.702320517</td></tr>
<tr><td>tr|A0A0G2K0D8|A0A0G2K</td><td>Cytosolic 10-formyltetrahyd</td><td>Aldh1l1</td><td>3</td><td>3.8</td><td>98.802</td><td>902</td><td>0</td><td>5.309</td><td>1.149406383</td><td>1.332676369</td><td>1.188035065</td><td>1.048414008</td><td>1.221016134</td><td>1.559944219</td></tr>
<tr><td>sp|P05982|NQO1_RAT NA</td><td>NAD(P)H dehydrogenase [</td><td>Nqo1</td><td>2</td><td>6.9</td><td>30.946</td><td>274</td><td>0.0010858</td><td>3.7703</td><td>0.551598663</td><td>0.84545976</td><td>0.946082977</td><td>0.575600433</td><td>0.51376407</td><td>1.631063207</td></tr>
<tr><td>sp|P0C0A9|SVIP_RAT Sm</td><td>Small VCP/p97-interacting</td><td>Svip</td><td>2</td><td>31.6</td><td>8.2484</td><td>76</td><td>0</td><td>25.103</td><td>0.735431436</td><td>0.827937534</td><td>0.921247441</td><td>0.767407519</td><td>0.958218041</td><td>0.832325845</td></tr>
<tr><td>tr|A0A0G2K621|A0A0G2K</td><td>Calcium-independent phos</td><td>Pnpla8</td><td>4</td><td>6.6</td><td>80.841</td><td>711</td><td>0</td><td>24.609</td><td>2.142725497</td><td>2.357405781</td><td>2.497183388</td><td>2.313271697</td><td>2.2087875</td><td>2.626830648</td></tr>
<tr><td>sp|O35910|MOT4_RAT Mo</td><td>Monocarboxylate transport</td><td>Slc16a3</td><td>5</td><td>11.3</td><td>50.548</td><td>471</td><td>0</td><td>9.5424</td><td>3.046385996</td><td>2.719943111</td><td>2.259164736</td><td>2.688703533</td><td>2.981434386</td><td>2.01348017</td></tr>
<tr><td>tr|M0R4V3|M0R4V3_RAT</td><td>Synaptosomal-associated</td><td>Snap23</td><td>5</td><td>31</td><td>22.928</td><td>210</td><td>0</td><td>21.188</td><td>5.186351995</td><td>4.302323546</td><td>4.338763518</td><td>4.505361211</td><td>4.373137153</td><td>4.656668185</td></tr>
<tr><td>tr|D4A7L4|D4A7L4_RAT N</td><td>Complex I-ESSS (NADH de</td><td>Ndufb11</td><td>9</td><td>51</td><td>17.634</td><td>151</td><td>0</td><td>165.26</td><td>60.67886333</td><td>57.8480261</td><td>50.41202114</td><td>60.87777136</td><td>57.40787396</td><td>54.04027158</td></tr>
<tr><td>tr|D3ZVS2|D3ZVS2_RAT L</td><td>L-2-hydroxyglutarate dehy</td><td>L2hgdh</td><td>19</td><td>45.8</td><td>50.732</td><td>463</td><td>0</td><td>179.65</td><td>28.27783542</td><td>36.51483894</td><td>34.16647062</td><td>34.8913902</td><td>28.03359422</td><td>33.09471341</td></tr>
<tr><td>tr|D3ZU74|D3ZU74_RAT C</td><td>Cytoplasmic dynein 1 inter</td><td>Dync1i2</td><td>5</td><td>14.5</td><td>68.362</td><td>612</td><td>0</td><td>62.714</td><td>0.907172588</td><td>0.776555456</td><td>0.652035723</td><td>0.804317617</td><td>1.00335697</td><td>0.64420596</td></tr>
<tr><td>tr|Q05BA4|Q05BA4_RAT</td><td>Myeloid-associated differen</td><td>Myadm</td><td>4</td><td>12.9</td><td>35.118</td><td>318</td><td>0</td><td>28.505</td><td>1.722830171</td><td>1.367079163</td><td>1.836731941</td><td>1.885759593</td><td>1.956020281</td><td>1.311332554</td></tr>
<tr><td>tr|G3V8I4|G3V8I4_RAT Sy</td><td>Syntaxin-4</td><td>Stx4</td><td>5</td><td>22.1</td><td>34.195</td><td>298</td><td>0</td><td>159.67</td><td>5.097044589</td><td>6.103657771</td><td>6.387260708</td><td>5.980271997</td><td>5.246362419</td><td>6.917540837</td></tr>
<tr><td>tr|D3ZPE6|D3ZPE6_RAT M</td><td>39S ribosomal protein L51</td><td>Mrpl51</td><td>2</td><td>15.6</td><td>15.047</td><td>128</td><td>0</td><td>8.4292</td><td>0.744111313</td><td>0.657947259</td><td>0.755768678</td><td>0.668144441</td><td>0.626929577</td><td>0.966893131</td></tr>
<tr><td>tr|M0R4P2|M0R4P2_RAT</td><td>Elongation factor Ts, mitoc</td><td>Tsfm</td><td>3</td><td>14.6</td><td>34.058</td><td>314</td><td>0</td><td>78.016</td><td>1.88875129</td><td>1.632627269</td><td>1.670978752</td><td>1.70574821</td><td>1.627428288</td><td>1.977006288</td></tr>
<tr><td>sp|Q499N5|ACSF2_RAT A</td><td>Medium-chain acyl-CoA lig</td><td>Acsf2</td><td>8</td><td>15.9</td><td>67.886</td><td>615</td><td>0</td><td>33.922</td><td>3.732547841</td><td>6.304546395</td><td>5.676955239</td><td>3.306021933</td><td>3.389032988</td><td>7.574781892</td></tr>
<tr><td>tr|B2GUZ6|B2GUZ6_RAT</td><td>Reticulon 4-interacting pro</td><td>Rtn4ip1</td><td>8</td><td>27</td><td>43.605</td><td>396</td><td>0</td><td>44.366</td><td>11.79510003</td><td>12.69201487</td><td>12.80745251</td><td>11.30117914</td><td>12.32557031</td><td>14.5997124</td></tr>
<tr><td>sp|P07151|B2MG_RAT Be</td><td>Beta-2-microglobulin</td><td>B2m</td><td>4</td><td>26.1</td><td>13.72</td><td>119</td><td>0</td><td>48.469</td><td>5.148220743</td><td>9.223107643</td><td>8.363126534</td><td>5.306334224</td><td>7.437946432</td><td>8.650405797</td></tr>
<tr><td>tr|Q6WN19|Q6WN19_RAT</td><td>Reticulon</td><td>Rtn2</td><td>6</td><td>17.9</td><td>50.966</td><td>469</td><td>0</td><td>176.51</td><td>49.3669264</td><td>59.69402967</td><td>61.81441351</td><td>54.08046031</td><td>58.68600136</td><td>61.93955926</td></tr>
<tr><td>sp|Q5U316|RAB35_RAT R</td><td>Ras-related protein Rab-35</td><td>Rab35</td><td>9</td><td>47.8</td><td>23.025</td><td>201</td><td>0</td><td>43.056</td><td>6.067384606</td><td>5.954595452</td><td>5.60286027</td><td>5.659906682</td><td>6.621454923</td><td>5.640548595</td></tr>
</table>

tr|F1LUT4|F1LUT4_RAT P	Phospholipid-transporting	Atp8a1	7	9.7	110.95	985	0	20.535	2.660708452	2.718511718	2.517079259	2.526191597	2.543797094	2.954485989
tr|G3V837|G3V837_RAT	Antigen-presenting glycop	Cd1d	7	20.2	38.593	336	0	23.869	3.441194972	5.119945177	5.611550421	4.294591413	3.816369759	5.399760019
sp|Q5I0K3|CLYBL_RAT C	Citramalyl-CoA lyase, mito	Clybl	11	42.6	37.321	338	0	164.77	14.44221168	10.06417451	10.05130264	12.48433292	11.32248299	12.03231691
sp|P17764|THIL_RAT Ace	Acetyl-CoA acetyltransfera	Acat1	33	75.2	44.695	424	0	323.31	247.3965662	315.2519989	324.7692238	297.1860125	242.6230946	387.8016857
sp|P07896|ECHP_RAT Pe	Peroxisomal bifunctional e	Ehhadh	7	11.1	78.657	722	0	40.244	2.545411587	3.342056184	3.48571089	3.244684278	2.579768022	3.947815065
sp|Q9ET09|MRS2_RAT M	Magnesium transporter MR	Mrs2	2	4.6	49.365	434	0.001077	3.6662	2.992600862	2.3439803	2.181456495	2.158942097	2.688058309	2.436231758
tr|D4ACY1|D4ACY1_RAT	RGD1565784 (RGD15657	RGD1565784	4	33.8	15.658	136	0	43.459	5.759825955	5.405236637	4.952928479	5.231140351	5.386309279	5.301006223
tr|B2GV15|B2GV15_RAT	Dihydrolipoamide acetyltra	Dbt	23	51.5	53.273	482	0	166.59	46.61093999	54.88652306	61.74123329	53.14277659	44.19246813	73.83675009
sp|P15650|ACADL_RAT L	Long-chain specific acyl-C	Acadl	25	48.6	47.872	430	0	323.31	310.2830284	357.552134	354.3843424	300.673957	244.8665587	558.0199053
tr|G3V644|G3V644_RAT	Complex I-9kD (NADH deh	Ndufv3	11	30.6	49.299	458	0	323.31	19.47503522	12.7216299	14.35612883	14.51737456	14.92281146	19.08732059
tr|B5DEL8|B5DEL8_RAT	Complex I-15 kDa (NADH	Ndufs5	9	66	12.7	106	0	177.48	82.29827974	84.65456448	75.68663816	83.54343822	80.20384233	76.21415628
tr|G3V6S5|G3V6S5_RAT	C-1-tetrahydrofolate syntha	Mthfd1	5	6.1	100.96	935	0	16.691	1.241222424	1.384009089	1.209623229	1.043158201	1.533159651	1.128455154
tr|A0A0G2K0V8|A0A0G2K	Nuclear migration protein	Nudc	2	7.2	32.4	279	0.005332	2.3756	0.870245481	0.87295238	0.88433717	0.962409897	0.984427742	0.744311016
tr|F1LQ09|F1LQ09_RAT A	Atlastin GTPase 2	Atl2	14	33.1	66.194	583	0	206.79	17.78220832	22.16240802	22.00712039	19.90816004	21.64781089	21.61204469
tr|Q66HF3|Q66HF3_RAT	Electron transfer flavoprote	Etfdh	37	56.8	68.163	616	0	323.31	292.5620307	302.7593919	268.0774254	316.9729306	271.0331162	288.2655811
sp|Q9WVK7|HCDH_RAT H	Hydroxyacyl-coenzyme A	Hadh	20	70.1	34.447	314	0	323.31	191.2332459	197.1225784	219.6092538	200.4373596	162.6673276	270.4959937
tr|D4AE79|D4AE79_RAT	Charged multivesicular bod	Chmp1a	3	12.8	21.607	196	0.001061	3.3344	1.093513996	1.115499479	1.201207504	0.936608663	1.159590518	1.428374089
tr|G3V9N1|G3V9N1_RAT	Serine/threonine-protein p	Pgam5	7	25.3	32	288	0	15.597	10.62055713	11.61649569	9.574259105	9.696366311	9.525554512	11.87382316
sp|B2RZ37|REEP5_RAT	Receptor expression-enha	Reep5	6	16.9	21.431	189	0	18.117	13.69664536	26.82776248	25.2151581	20.15781086	21.24118301	27.90709118
sp|P29418|ATP5E_RAT A	ATP synthase subunit eps	Atp5f1e	5	68.6	5.7667	51	0	9.5001	384.3529853	321.5254495	306.5519239	354.0622037	301.017877	336.2912182
sp|P32551|QCR2_RAT Cy	Cytochrome b-c1 complex	Uqcrc2	25	48.7	48.396	452	0	323.31	853.5379717	941.7579676	804.9823735	929.6208292	795.5668922	833.7888641
tr|A0A0G2K6D5|A0A0G2K	Pitrilysin metalloproteinase	Pitrm1	16	21.5	117.42	1035	0	124.18	13.94098641	13.98767246	14.16403076	12.85283665	13.67326666	16.39597486
sp|P06907|MYP0_RAT My	Myelin protein P0 (Myelin	Mpz	11	35.5	27.57	248	0	142.63	247.1657718	296.8462575	308.6055437	268.9898034	450.4832812	192.2346265
tr|F1LWG8|F1LWG8_RAT	Sarcalumenin	Srl	33	43.5	98.363	904	0	323.31	177.1146481	186.1354021	169.8650021	138.5980145	192.1721166	189.0037925
tr|D3ZYM7|D3ZYM7_RAT	LYR motif-containing 4 (R	Lyrm4	2	23.1	10.816	91	0	9.0881	2.570297246	2.52083139	2.639930546	2.428063295	2.201237381	3.369516088
sp|Q63619|COQ7_RAT 5-	5-demethoxyubiquinone h	Coq7	5	34.6	20.14	179	0	22.381	7.433587226	7.865258498	8.660878537	7.749328788	7.612138478	8.996653673
tr|B2RYW4|B2RYW4_RAT	39S ribosomal protein L53	Mrpl53	4	42.9	12.735	119	0	14.929	3.775897054	4.038058757	4.068819997	3.966402113	3.508864172	4.66195131
sp|P10960|SAP_RAT Pros	Prosaposin (Sulfated glyco	Psap	14	31.4	61.123	554	0	124.42	37.10371507	33.69450087	46.6340925	26.01982721	35.4947303	50.21914992
tr|D4A2W1|D4A2W1_RAT	AP3A hydrolase (EC 3.6.1	Enpp4	2	8	41.503	364	0	24.295	1.020863926	1.214857907	1.35909382	0.94210337	1.314583688	1.467895927
sp|P02600|MYL1_RAT My	Myosin light chain 1/3, ske	Myl1	20	77.2	20.679	189	0	323.31	811.7441126	564.0182545	1033.944974	895.6969853	730.0426396	676.9613341
sp|Q2PS20|JPH2_RAT Ju	Junctophilin-2 (JP-2) (Junc	Jph2	16	27.3	74.258	692	0	267.09	27.70185283	29.85984801	36.29144113	28.15738773	31.03746324	32.63243999
sp|P97615|THIOM_RAT T	Thioredoxin, mitochondrial	Txn2	3	36.7	18.232	166	0	162.25	4.036293366	4.396104475	5.063613555	3.809086259	4.268406208	5.915067892
tr|D3ZGP9|D3ZGP9_RAT	Dehydrogenase/reductase	Dhrs7c	15	49.5	34.366	311	0	104.68	22.95551541	26.11305308	29.28717973	24.74589131	28.98221282	26.14638822
tr|A0JN29|A0JN29_RAT L	Endoplasmic reticulum junc	Lnpk	6	17	42.169	376	0	56.405	6.764785136	8.47335379	7.600680161	6.511825185	8.007980465	7.854482714
tr|G3V8F5|G3V8F5_RAT	Mitochondrial import recep	Tomm40	11	42.9	37.92	361	0	174.53	14.55610371	14.17177923	11.44401362	12.90300571	14.1025449	13.90071434
tr|Q5RKJ9|Q5RKJ9_RAT	Ras-related protein Rab-1	Rab10	12	41	22.541	200	0	236.46	47.72527566	54.73351207	46.73928906	49.27318883	51.71292658	46.17857537
tr|D4AEG6|D4AEG6_RAT	Mitochondrial ribosomal pr	Mrpl32	3	19.8	21.601	187	0	8.6925	2.512448123	2.589883769	2.171760116	2.289560841	2.446346622	2.649995119
tr|G3V7K3|G3V7K3_RAT	Ceruloplasmin (EC 1.16.3.	Cp	9	12.7	120.66	1059	0	53.027	5.556124794	6.747784683	7.197731598	5.36910528	6.149572445	8.750378775
tr|D3ZKG1|D3ZKG1_RAT	Methylmalonyl-CoA isomer	Mmut	17	28.9	92.035	833	0	281.16	23.61829678	22.19893323	26.07411088	22.65849985	20.17391967	27.44075381
sp|B0BN93|PSD13_RAT 2	26S proteasome non-ATP	Psmd13	2	5.3	42.817	376	0	5.6618	0.300715094	0.304620203	0.225093196	0.263698162	0.371670818	0.228484991
sp|Q8VHW7|CCG6_RAT V	Voltage-dependent calciu	Cacng6	5	14.6	28.255	260	0	20.701	0.559174741	0.595953129	0.648834088	0.677103202	0.520089991	0.642326387
sp|Q60587|ECHB_RAT Tr	Trifunctional enzyme subu	Hadhb	33	67.2	51.414	475	0	323.31	338.9517091	403.2678694	353.6891304	355.4060179	307.3815493	410.1736876
tr|B1H264|B1H264_RAT T	TBC1 domain family, mem	Tbc1d17	9	17.5	72.795	646	0	79.988	8.035157899	10.43337522	8.491649288	7.89744698	8.589339677	11.28963147
sp|Q6P6R2|DLDH_RAT Di	Dihydrolipoyl dehydrogena	Dld	27	56.6	54.037	509	0	323.31	392.1197193	373.0259874	395.2051815	387.8904875	321.5488095	482.7861751
tr|Q6AY58|Q6AY58_RAT	B-cell receptor-associated	Bcap31	14	42.4	27.911	245	0	68.675	22.84864756	33.06419431	29.75141672	23.63978287	25.97295085	33.39239718
tr|D4A4A9|D4A4A9_RAT	Mitochondrial ribosomal pr	Mrpl19	5	16.1	33.633	292	0	25.524	3.937804356	4.672708859	4.218199112	4.305760003	3.638672301	5.210685107
tr|D3ZDC2|D3ZDC2_RAT	L-type amino acid transpo	Slc43a2	2	3.5	62.501	568	0	12.168	1.303837954	1.769152559	2.080101896	1.490678206	1.950357691	1.54226607
tr|Q6AYL0|Q6AYL0_RAT	RCG35015, isoform CRA_	Slc25a19	3	11	35.638	318	0	7.753	3.889136837	3.911454502	2.436215121	3.394176147	3.330681349	3.188772258
tr|F1LSK3|F1LSK3_RAT V	Very-long-chain (3R)-3-hyd	Hacd1	3	6.2	24.744	211	0	13.941	3.825618199	5.017279739	5.214090374	4.691404827	3.943697134	5.085515687
sp|P84817|FIS1_RAT Mito	Mitochondrial fission 1 prot	Fis1	6	42.1	16.994	152	0	122.66	33.23138609	32.42993907	27.2513976	33.14323718	30.61465654	30.45111901
sp|Q3MIE0|ECHD3_RAT E	Enoyl-CoA hydratase dom	Echdc3	2	5	32.385	300	0.0010644	3.3898	1.409953214	1.361945891	1.297530963	1.220840306	1.229536983	1.72859782
sp|Q63524|TMED2_RAT T	Transmembrane emp24 do	Tmed2	3	23.4	22.733	201	0	42.277	3.358961917	3.501681198	3.628549524	2.959855384	3.983173474	3.350720355
sp|Q68FV1|TM38B_RAT T	Trimeric intracellular cation	Tmem38b	4	16.5	32.444	291	0	11.172	1.61019246	2.237909133	2.469146218	1.414767633	1.774439903	2.825252627
tr|D3ZRH1|D3ZRH1_RAT	OXA1L, mitochondrial inne	Oxa1l	6	14.8	48.023	433	0	25.545	12.51006101	11.78135269	12.23435995	11.87274813	12.49221224	12.33101666
sp|P15651|ACADS_RAT S	Short-chain specific acyl-C	Acads	19	57.5	44.765	412	0	323.31	100.2601063	87.63581088	100.7737309	108.6160257	88.95658817	95.38681966
sp|P19939|APOC1_RAT A	Apolipoprotein C-I (Apo-CI	Apoc1	6	40.9	9.8606	88	0	12.992	20.44537523	44.46894252	21.82599935	29.57765006	25.87102423	26.40343256
tr|G3V822|G3V822_RAT	Carboxylesterase 1D (Carb	Ces1d	3	7.8	62.081	564	0	9.8405	3.052507066	2.966735032	4.974425167	3.085457222	4.005554186	4.330638421
tr|G3V8L3|G3V8L3_RAT L	Prelamin-A/C [Cleaved int	Lmna	19	27.2	74.311	665	0	66.562	21.04945454	20.39735221	19.87163022	18.25556715	25.43904953	18.98369006
sp|Q68FT1|COQ9_RAT U	Ubiquinone biosynthesis p	Coq9	15	51.3	35.145	312	0	323.31	113.6010272	93.88951814	110.9915185	102.9301983	96.25863244	125.2811786
sp|Q4FZT0|STML2_RAT S	Stomatin-like protein 2, mit	Stoml2	15	55.2	38.413	353	0	236.7	14.9981252	16.61501924	13.84112306	13.21596512	13.10970416	18.09470271
sp|P68511|1433F_RAT 14	14-3-3 protein eta	Ywhah	2	9.8	28.211	246	0	35.772	0.773311824	0.701826863	0.666854716	0.68719674	0.772808674	0.704525022
tr|A1L1M0|A1L1M0_RAT	cAMP-dependent protein	Prkaca	3	8.5	40.605	351	0	15.486	0.354033623	0.318257925	0.241389515	0.250271964	0.408930658	0.217695225
sp|P04692|TPM1_RAT Tr	Tropomyosin alpha-1 chain	Tpm1	9	21.8	32.68	284	0	323.31	314.6229669	232.137416	408.4279318	357.2694403	328.5650278	235.8813659
sp|Q5I0H4|TMCO1_RAT C	Calcium load-activated cal	Tmco1	3	17.6	21.175	188	0	5.6617	1.192605078	1.594177088	1.624920954	1.351459051	1.488128581	1.491263595
sp|Q9ER34|ACON_RAT A	Aconitate hydratase, mitoc	Aco2	60	63.3	85.432	780	0	323.31	990.3589245	1011.353289	966.1617987	897.010937	821.6148049	1340.186543
sp|Q9QZ81|AGO2_RAT P	Protein argonaute-2 (Argo	Ago2	2	2.9	97.317	860	0	4.765	0.569811353	0.477048782	0.697133031	0.468011679	0.814496121	0.382457602
sp|P45479|PPT1_RAT Pa	Palmitoyl-protein thioestera	Ppt1	2	7.8	34.455	306	0	105.77	0.479570735	0.844176442	0.670467989	0.427398626	0.528972168	0.938120421

tr|A0A0G2K719|A0A0G2K	RNA helicase (EC 3.6.4.13	Ddx3x	3	7.3	73.145	662	0	28.457	5.430191317	5.558247628	5.405273688	4.944997506	6.279542362	4.92214521
sp|Q5I0P2|GCSH_RAT Gl	Glycine cleavage system H	Gcsh	3	21.8	18.485	170	0	189.53	4.421469184	4.763627003	4.998208737	4.275658564	4.014182894	6.290474554
tr|D3ZAQ0|D3ZAQ0_RAT	FUN14 domain-containing	Fundc2	9	39.7	16.344	151	0	97.049	74.20542324	69.77794753	64.61355676	66.12163315	76.13756361	68.42357906
sp|P0DP31|CALM3_RAT (	Calmodulin-3	Calm3	8	55	16.837	149	0	48.7	20.54572063	18.1495714	17.01302804	18.63004338	21.06051944	16.82472076
tr|F7EMK8|F7EMK8_RAT	Aldose reductase-related p	LOC1009107	3	15.9	33.766	295	0	22.023	6.451205761	3.003901895	8.553395095	7.362907538	4.673955491	5.051683368
tr|Q304F3|Q304F3_RAT R	RCG32327 (Skeletal tropo	Tnnc2	8	55	18.096	160	0	173.22	46.1930014	29.76952217	58.55332015	53.77466791	47.83324367	37.65445658
tr|A0A0G2JXD0|A0A0G2J	60S ribosomal protein L9	Rpl9	5	29.3	21.424	188	0	233.19	12.43279505	10.29418458	9.587980395	10.25121228	13.11617569	9.613052109
sp|P0C089|PTPM1_RAT P	Phosphatidylglycerophosp	Ptpmt1	5	31.6	21.886	193	0	45.642	6.41457969	6.71520815	5.827889434	6.270535873	6.497956537	6.049177985
sp|P62912|RL32_RAT 60S	60S ribosomal protein L32	Rpl32	6	34.8	15.86	135	0	41.73	10.91707778	10.79270426	11.912367	10.34617515	13.73690339	8.805546791
sp|Q5RJS6|MSPD1_RAT I	Motile sperm domain-conta	Mospd1	5	25.8	24.088	213	0	80.272	2.743794443	3.75390254	3.980271936	3.518882108	3.3456198	3.808320248
tr|Q5M7T6|Q5M7T6_RAT	V-type proton ATPase sub	Atp6v0d1	6	19.9	40.301	351	0	72.615	7.2640035	7.992603129	8.49759518	6.808061569	7.588948825	9.81340446
sp|P21571|ATP5J_RAT A	ATP synthase-coupling fac	Atp5pf	12	63.9	12.494	108	0	323.31	259.879534	192.1225741	165.2409272	216.5272955	195.5912422	191.3811987
tr|Q5XIH1|Q5XIH1_RAT A	Asporin	Aspn	8	29.6	42.767	375	0	103.25	9.075739696	14.81886764	16.70155474	9.689796552	11.69028167	17.7360598
tr|A0A0G2JSI4|A0A0G2JS	Adhesion G protein-couple	Adgre5	2	2.4	99.386	903	0.0010875	3.7848	0.146268472	0.184521383	0.165593107	0.138388977	0.153186535	0.216851957
sp|Q5XI37|RT15_RAT 28S	28S ribosomal protein S15	Mrps15	7	30.4	29.712	257	0	17.583	6.084945051	5.463973114	5.155088825	5.300481166	5.235684393	6.371753398
sp|P35738|ODBB_RAT 2-	2-oxoisovalerate dehydrog	Bckdhb	7	27.7	42.823	390	0	241.45	18.82630221	21.40673116	23.64132608	19.18369485	15.99708563	30.87478498
sp|P19945|RLA0_RAT 60	60S acidic ribosomal prote	Rplp0	12	44.8	34.215	317	0	323.31	57.42767237	53.10962124	58.63107412	56.6635699	68.98652172	46.6124012
tr|A0JPL2|A0JPL2_RAT C	Casein kinase I isoform alp	Csnk1a1	3	12.8	38.914	337	0	5.6686	0.589579397	0.79965518	0.575653872	0.543629599	0.73845563	0.642519424
sp|P20761|IGG2B_RAT Ig	Ig gamma-2B chain C regi	Igh-1a	3	11.4	36.497	333	0	18.19	3.194796843	5.309481372	3.837433302	3.252030463	3.597578078	5.130727044
sp|Q5RK30|SBDS_RAT R	Ribosome maturation prote	Sbds	3	14	28.752	250	0	9.3607	1.929391177	2.506665534	2.493753065	2.301505856	2.597618662	2.130013713
sp|Q9JHY2|SFXN3_RAT S	Sideroflexin-3	Sfxn3	5	22.7	35.433	321	0	49.622	1.77390598	1.960712448	1.766387458	2.0839274	1.690525716	1.783867435
tr|B2RYD7|B2RYD7_RAT	Dolichyl-diphosphooligosac	Stt3b	3	4.6	93.435	824	0	43.371	2.559710807	3.244770809	3.037985183	2.678848895	3.183292228	2.876153503
tr|D3ZTW9|D3ZTW9_RAT	Endonuclease G-like 1 (Pr	Exog	3	12	41.326	368	0	5.9318	0.801960436	0.612685621	0.580136161	0.64049173	0.616790845	0.70681099
tr|F1LUA1|F1LUA1_RAT E	Early endosome antigen 1	Eea1	4	3	161.1	1411	0	6.3049	1.565388239	1.556417924	1.2674356	1.240967658	1.529869956	1.68358966
sp|Q66HA6|ARL8B_RAT A	ADP-ribosylation factor-like	Arl8b	3	16.1	21.539	186	0	125.25	25.23435944	37.81493877	39.62571471	31.27205051	30.64593561	38.65012242
tr|Q0D2L2|Q0D2L2_RAT M	Mitochondrial ribosomal pro	Mrps22	6	17.5	41.235	359	0	17.669	7.886646707	7.333668702	6.591250559	7.177162547	7.118144936	7.685930711
sp|P00507|AATM_RAT As	Aspartate aminotransferas	Got2	30	60.7	47.314	430	0	323.31	800.4552552	896.2495376	824.3751307	728.4070436	721.0364254	1028.685373
tr|Q5M9H4|Q5M9H4_RAT	Sodium/potassium-transpo	Atp1b2	7	20.7	33.398	290	0	186.5	9.843382005	8.034064172	8.786657033	9.520177332	9.863692013	7.619790052
tr|A0A0G2JSS8|A0A0G2J	Peroxiredoxin-5, mitochond	Prdx5	13	48.4	22.206	213	0	323.31	28.72437245	31.10170496	32.10690492	26.71442986	25.99344403	37.76316703
tr|D3ZUD8|D3ZUD8_RAT T	Transmembrane 9 superfa	Tm9sf3	3	4.9	69.968	609	0	6.1244	1.121711053	1.064561627	0.895908792	1.022732225	1.185153065	0.910993606
sp|Q5BJQ0|COQ8A_RAT	Atypical kinase COQ8A, m	Coq8a	24	41.3	72.225	649	0	323.31	208.9341744	227.1571551	208.659664	191.2277527	187.8955131	257.0849844
tr|B1WBY5|B1WBY5_RAT	DnaJ (Hsp40) homolog, su	Dnajc11	9	19.9	63.204	559	0	68.975	10.07718679	10.02715572	7.801925754	8.772419364	9.36322694	9.485749119
sp|O35165|GOSR2_RAT (	Golgi SNAP receptor comp	Gosr2	3	17.5	24.607	212	0	5.1062	0.307071976	0.301456331	0.284648171	0.327317314	0.329627438	0.246528895
tr|B4F789|B4F789_RAT A	Apolipoprotein B editing co	Apobec2	3	18.3	25.66	224	0	7.8995	1.387877226	1.168609101	1.129216467	1.043158201	1.227541594	1.371580497
tr|A0A0A0MXU4|A0A0A0M	ER membrane protein com	Emc2	8	33.1	34.513	296	0	50.716	9.060687886	9.921528778	8.825076646	9.314723066	9.527711689	9.089819549
tr|D4A040|D4A040_RAT M	Mitochondrial ribosomal pro	Mrps11	2	15.7	20.168	191	0	5.3612	1.545369331	1.554344872	1.334029596	1.481062469	1.438136003	1.541046887
tr|A0A0G2K401|A0A0G2K	Propanoyl-CoA:carbon dio	Pcca	3	3.3	79.811	723	0	323.31	45.25477191	45.1387358	49.44695704	44.79918328	40.92650209	52.84140863
tr|O55158|O55158_RAT T	Tetraspanin	Tspan8	6	26.8	25.545	235	0	143.05	10.60801395	16.91956047	16.40608962	11.5030499	11.13750505	22.66054178
sp|Q641Z6|EHD1_RAT EH	EH domain-containing prot	Ehd1	14	34.8	60.602	534	0	323.31	74.68206389	73.90924427	67.51789658	71.38341247	79.16839735	64.00302592
tr|G3V945|G3V945_RAT A	Succinate-semialdehyde d	Aldh5a1	17	39.2	56.145	523	0	151.27	35.08175527	53.21820968	56.45853647	34.12511746	31.24455224	74.52050837
tr|A0A0G2K3W1|A0A0G2I	von Willebrand factor A do	Vwa8	8	19.8	53.76	481	0	67.237	8.219793434	7.80898994	8.195269414	7.023071848	7.591645296	9.376937066
tr|Q4PP99|Q4PP99_RAT	Cardiac troponin C (Tropor	Tnnc1	3	30.4	18.42	161	0	48.099	4.843471763	2.63139417	5.765228874	4.520889731	4.211348875	4.804392485
tr|D4A2Z6|D4A2Z6_RAT S	SEC63 homolog, protein tr	Sec63	8	12.9	83.591	727	0	21.535	2.406031827	2.698027989	2.433059224	2.351734646	2.654622065	2.493330146
tr|D3ZFF4|D3ZFF4_RAT Ly	Lysocardiolipin acyltransfe	Lclat1	2	5.1	44.566	376	0.00249	2.6147	0.821577962	1.017375012	0.928977102	0.914092309	1.094875206	0.723178517
sp|O70595|ABCB6_RAT A	ATP-binding cassette sub-	Abcb6	3	6.7	93.304	836	0	20.247	1.30413899	0.926999811	0.840337565	0.88751465	1.000498711	1.23279687
tr|D4A193|D4A193_RAT R	Receptor expression-enha	Reep1	2	14.4	22.24	201	0	6.152	1.680584758	1.801235509	1.494568694	1.587193931	1.983038923	1.456821685
tr|D3ZAI6|D3ZAI6_RAT 5-n	5'-nucleotidase domain-co	Nt5dc3	5	9.7	63.126	546	0	13.061	5.036837349	4.313281107	4.319599449	4.501299906	4.266572608	4.988895461
tr|Q8R4I6|Q8R4I6_RAT Ac	Actinin alpha 3 (Actinin alp	Actn3	39	44	103.01	900	0	323.31	156.1173732	134.2548046	186.9114186	164.9308012	162.1873557	154.5923618
sp|P00173|CYB5_RAT Cy	Cytochrome b5	Cyb5a	7	56	15.355	134	0	42.184	11.015918	12.41610151	13.31971402	10.76305619	12.30076277	13.99926494
sp|P24090|FETUA_RAT A	Alpha-2-HS-glycoprotein (5	Ahsg	2	8.5	37.982	352	0	12.184	6.66544319	5.548869535	4.734759961	5.402431873	7.835945596	4.025842755
tr|D4A520|D4A520_RAT P	Protein phosphatase (EC 3	Pptc7	3	15	32.847	307	0	8.3016	1.262746513	0.971422356	0.890557488	0.99221271	0.961777383	1.141256572
tr|Q5BKA0|Q5BKA0_RAT	C-type lectin domain family	Clec10a	2	5.6	34.214	306	0.0010604	3.3293	0.833167856	1.126950625	0.93789594	0.796314457	1.112725846	1.017204736
tr|G3V8U8|G3V8U8_RAT	Branched-chain-amino-acid	Bcat2	13	37.9	44.229	393	0	244.51	42.79329925	60.47882797	67.25719206	47.20670115	41.36117326	78.87705442
tr|E9PTA4|E9PTA4_RAT A	ADAM metallopeptidase do	Adam9	2	3.1	92.367	845	0	9.1321	1.38998448	1.630850367	1.457018096	1.262767311	1.484623168	1.767205272
tr|A0A0G2K0T6|A0A0G2K	Gamma-synuclein (Persyn)	Sncg	3	43.9	13.045	123	0	27.296	5.282683579	5.869699031	6.036453049	4.538747529	5.36840471	7.10021504
tr|F1MA54|F1MA54_RAT [	[Pyruvate dehydrogenase	Pdk1	8	22.8	49.098	434	0	63.619	9.815285293	9.849959121	10.40073817	8.972498373	8.469077057	12.36149623
sp|P23785|GRN_RAT Gra	Progranulin (PGRN) (Acrog	Grn	5	11.9	63.369	588	0	21.123	1.862460795	1.246595348	1.622999973	1.1770021	1.5154708	2.103191694
sp|P16409|MYL3_RAT My	Myosin light chain 3 (Myos	Myl3	12	60	22.156	200	0	323.31	53.9958597	36.65106808	56.38535625	44.89832691	38.60214383	65.42845365
tr|A0A0G2K075|A0A0G2K	Translocon-associated pro	Ssr1	4	21.2	32.867	292	0	191.28	7.40950433	6.677202194	5.960528575	5.642287784	9.361609057	5.298263062
tr|D4ACV3|D4ACV3_RAT	Histone H2A type 2-A (H2A	H2ac18	2	14.3	13.645	126	0	100.86	8.893612795	7.569601778	8.397887136	6.432390833	12.8999187	5.939756341
tr|A0A0G2K6J0|A0A0G2K	Metalloreductase STEAP4	Steap4	6	21.7	43.785	391	0	39.24	5.352925359	5.645118384	5.167437986	5.018757976	6.082160663	5.127272694
tr|F1LQS6|F1LQS6_RAT F	Xanthine dehydrogenase/	Xdh	7	7.8	146.26	1331	0	60.319	29.8828601	23.50446248	37.63749972	38.59972025	29.23675971	22.16169288
sp|Q5XI78|ODO1_RAT 2-o	2-oxoglutarate dehydrogen	Ogdh	55	51.1	116.29	1023	0	323.31	538.2025525	516.9797145	547.0678505	484.0717521	435.0972159	670.845101
tr|D3ZM09|D3ZM09_RAT S	Seryl-tRNA synthetase (EC	Sars2	10	22.2	58.286	518	0	55.98	8.683890909	9.503956849	8.809983226	8.215781643	8.629247452	10.0705504
sp|P07154|CATL1_RAT C	Procathepsin L (EC 3.4.22	Ctsl	2	8.1	37.66	334	0.0010678	3.431	0.545477594	0.420380421	0.491039248	0.350287579	0.571490127	0.525589646
sp|Q9WUH4|FHL1_RAT Fo	Four and a half LIM domai	Fhl1	14	50	31.904	280	0	201.89	55.59135156	72.93194827	78.93401024	57.27336294	69.26695474	82.12719221
tr|D3ZEG8|D3ZEG8_RAT T	Translocase of inner mitoc	Timm29	3	22.8	29.873	267	0	62.303	2.958734289	2.905234485	2.714803055	3.11908244	2.935594374	2.501254834

sp|Q62651|ECH1_RAT De	Delta(3,5)-Delta(2,4)-dieno	Ech1	15	49.5	36.171	327	0	126.01	50.51387432	49.30408983	56.55001174	47.52025781	56.76611379	51.71772861
tr|D4A3V2|D4A3V2_RAT N	NADH dehydrogenase [ub	Ndufa6	8	52.3	15.224	130	0	323.31	170.0101938	173.8747795	150.6414742	180.9849019	158.7143007	153.6881347
tr|D3ZTN2|D3ZTN2_RAT C	COX assembly mitochondr	Cmc1	3	28.3	12.572	106	0	7.7271	1.804210291	1.232281416	1.109732235	1.036946793	1.376225022	1.704518963
sp|P06685|AT1A1_RAT S	Sodium/potassium-transpo	Atp1a1	31	31.1	113.05	1023	0	323.31	79.40331496	98.27254264	109.5233404	86.58941717	84.75009294	114.6234901
tr|F1LRV9|F1LRV9_RAT M	Myosin heavy chain 4	Myh1	4	2.4	223.28	1943	0	323.31	899.0947833	850.0994485	957.5631234	928.5457778	738.3477712	1048.903485
sp|P35280|RAB8A_RAT R	Ras-related protein Rab-8A	Rab8a	2	7.2	23.668	207	0	15.551	3.793909053	3.36569885	2.674325248	3.311218014	3.885291566	2.679357101
sp|Q9Z270|VAPA_RAT Ve	Vesicle-associated membra	Vapa	11	43.4	27.841	249	0	82.418	41.55905083	45.07259556	49.50641597	41.78366413	48.43293889	46.18568727
sp|Q64428|ECHA_RAT Tri	Trifunctional enzyme subu	Hadha	62	76	82.664	763	0	323.31	855.7455705	1033.515204	984.1824269	958.8861171	815.6825681	1091.574879
tr|D4A2V6|D4A2V6_RAT J	Junctional sarcoplasmic re	Jsrp1	9	41.1	36.751	338	0	106.99	20.61646414	29.34602724	31.33531102	25.33418332	24.22779462	31.43459302
sp|Q5BK63|NDUA9_RAT N	NADH dehydrogenase [ub	Ndufa9	32	72.4	42.559	377	0	323.31	359.632896	377.8285582	329.928429	387.7949273	326.8015356	354.3859209
tr|M0R735|M0R735_RAT H	Heterogeneous nuclear rib	Syncrip	3	5.9	62.672	562	0.00055127	3.9222	0.782744292	0.845854627	0.745431973	0.788012671	0.924026785	0.655991392
tr|A0A0H2UHK5|A0A0H2U	Leucyl-cystinyl aminopepti	Lnpep	7	9.6	103.19	900	0	27.327	5.093030773	4.683222195	4.585655271	4.474005546	5.346293645	4.523878873
sp|P67779|PHB_RAT Pro	Prohibitin	Phb	21	80.5	29.82	272	0	323.31	138.2057193	149.9606424	132.794649	141.0586877	130.3743873	149.1873187
sp|Q5HZE0|MCATL_RAT M	Mitochondrial basic amino	Slc25a29	2	6.9	32.634	306	0.0010672	3.4163	0.741953887	0.832478505	0.631545262	0.705771239	0.764988908	0.738022066
tr|A0A0G2K4U8|A0A0G2K	Syntaxin-12 (Syntaxin-13)	Stx12	2	8.8	31.106	272	0	13.417	0.815005338	0.846496286	0.72750282	0.748355219	0.7816531	0.861159515
tr|Q2I6B2|Q2I6B2_RAT V-	V-type proton ATPase 116	Atp6v0a1	6	7.9	95.584	832	0	20.869	2.408088907	3.219252524	2.698474719	2.511320053	2.351862267	3.479547323
sp|P68136|ACTS_RAT Ac	Actin, alpha skeletal muscl	Acta1	2	5.8	42.051	377	0	323.31	841.0449694	578.5789778	1030.3317	869.2985011	839.4654449	745.4590801
tr|F8WG17|F8WG17_RAT	Troponin I, fast skeletal mu	Tnni2	10	49.5	21.311	182	0	281.27	48.45980398	36.19549019	60.34166167	52.4947595	57.07351152	35.18053176
tr|D3ZYX8|D3ZYX8_RAT C	Cytochrome c oxidase sub	Cox7a2l	5	35.8	13.273	120	0	58.235	20.50357557	24.30554906	20.99494653	23.04193485	20.32492207	22.47156847
sp|Q561S0|NDUAA_RAT N	NADH dehydrogenase [ub	Ndufa10	3	13	40.493	355	0	323.31	369.8631096	370.7357584	360.3393825	401.3226574	359.649949	340.3246808
sp|P20788|UCRI_RAT Cyt	Cytochrome b-c1 complex	Uqcrfs1	19	62.4	29.445	274	0	323.31	599.7644553	686.5257637	537.4172096	659.3648541	576.7752109	587.859401
tr|A0A0A6YYM0|A0A0A6Y	Long-chain-fatty-acid--CoA	Acsl6	31	47.6	80.995	722	0	323.31	104.3391468	79.55584341	68.8808781	81.88308107	96.98128675	73.86316571
