# Supplementary material for: Ultrastructural and proteomic profiling of mitochondria-associated endoplasmic reticulum membranes reveal aging signatures in striated muscle
Source: Cell Death Dis. 2022 Apr 2;13(4):296. doi: 10.1038/s41419-022-04746-4 (PMC8976840; doi:10.1038/s41419-022-04746-4)
Supplement: Supplementary file 6 — Supplementary Table 4. Differential expression analysis. [file 41419_2022_4746_MOESM6_ESM.pdf]

Supplementary Table 4a. Differential expression analysis of the heart samples								
Gene names	Protein names	EntrezID	logFC	AveExpr	t	P.Value	adj.P.Val	B
Sntb1	"syntrophin, beta 1"	299940	-1.1595	5.2978	-4.5992	0.0011607	0.016811	-0.72099
Nlrx1	NLR family member X1	315599	-0.86966	8.5762	-9.6039	3.7114E-06	0.0017822	4.9419
Tpm1	tropomyosin 1	24851	-0.83801	7.6217	-7.9366	1.8536E-05	0.0028798	3.4103
Dnajc11	DnaJ heat shock protein family (Hsp40) m	362666	-0.82836	7.0472	-5.9674	0.00017887	0.0076421	1.168
Ldb3	LIM domain binding 3	498587	-0.81825	8.7475	-4.7415	0.00094369	0.014661	-0.5112
Hspa9	heat shock protein family A (Hsp70) memb	291671	-0.81232	7.7269	-6.7176	7.1453E-05	0.0054266	2.0846
Crat	carnitine O-acetyltransferase	311849	-0.80353	8.0924	-7.5621	2.7579E-05	0.0034508	3.0231
Acadvl	"acyl-CoA dehydrogenase, very long chai	25363	-0.73046	11.296	-7.9544	1.8196E-05	0.0028798	3.4282
Mybpc3	myosin binding protein C3	295929	-0.7264	8.6688	-8.2546	1.3376E-05	0.0028798	3.7258
Pygm	"glycogen phosphorylase, muscle associa	24701	-0.72465	8.5888	-7.4897	2.9829E-05	0.0034508	2.9464
Gnai2	G protein subunit alpha i2	81664	-0.72084	5.7033	-6.2681	0.00012276	0.0067675	1.5452
Uqcrc1	ubiquinol-cytochrome c reductase core pr	301011	-0.70804	7.4042	-6.6512	7.7272E-05	0.0054266	2.0068
Ndufaf3	NADH:ubiquinone oxidoreductase comple	56769	-0.7	5.9258	-4.3342	0.0017197	0.019503	-1.1193
Rap1a	"RAP1A, member of RAS oncogene family	295347	-0.69956	6.4423	-5.469	0.00034285	0.010102	0.51308
Slc8a1	solute carrier family 8 member A1	29715	-0.69434	8.7299	-6.5377	8.8459E-05	0.0054266	1.8722
Flna	filamin A	293860	-0.68645	7.6717	-8.0205	1.6991E-05	0.0028798	3.4946
Mtx2	metaxin 2	288150	-0.66987	5.6815	-5.3509	0.00040204	0.010256	0.35228
Mrps7	mitochondrial ribosomal protein S7	113958	-0.66955	10.922	-3.6077	0.0053108	0.034554	-2.2589
Sdha	succinate dehydrogenase complex flavop	157074	-0.66633	7.9913	-7.2364	3.9433E-05	0.0039642	2.6723
Etfdh	electron transfer flavoprotein dehydrogen	295143	-0.66235	6.6433	-7.6909	2.4016E-05	0.0034203	3.1582
Coq5	"coenzyme Q5, methyltransferase"	304542	-0.65501	7.0804	-4.5459	0.0012552	0.017299	-0.80025
Txndc5	thioredoxin domain containing 5	100362805	-0.64897	6.3901	-4.2753	0.0018794	0.02051	-1.2093
Eef1a2	eukaryotic translation elongation factor 1	24799	-0.6479	6.0827	-4.8801	0.00077354	0.013771	-0.30978
Pdha1	pyruvate dehydrogenase E1 alpha 1 subu	29554	-0.63254	8.2335	-6.5852	0.00008358	0.0054266	1.9287
Ndufs5	NADH:ubiquinone oxidoreductase subuni	362588	-0.62981	5.0296	-4.4633	0.0014181	0.018158	-0.92393
Dlat	dihydrolipoamide S-acetyltransferase	81654	-0.622	9.0159	-5.802	0.00022115	0.0082162	0.95482
Pcca	propionyl-CoA carboxylase subunit alpha	687008	-0.62029	8.4517	-5.2472	0.00046308	0.010639	0.20944
Immt	inner membrane mitochondrial protein	312444	-0.61342	7.8132	-6.7868	6.5886E-05	0.0054266	2.1651
Nos3	nitric oxide synthase 3	24600	-0.59818	9.8847	-3.9688	0.0030058	0.026479	-1.6846
Gnb1	G protein subunit beta 1	24400	-0.59002	8.2979	-5.8692	0.0002028	0.0082162	1.0419
Hsp90b1	heat shock protein 90 beta family member	362862	-0.5826	5.0826	-5.3624	0.00039584	0.010256	0.36798
Hsp90ab1	heat shock protein 90 alpha family class E	301252	-0.5687	10.947	-5.4422	0.00035542	0.010145	0.47672
Atp1a1	ATPase Na+/K+ transporting subunit alph	24211	-0.56709	7.6489	-6.1885	0.00013546	0.0072171	1.4467
Cct8	chaperonin containing TCP1 subunit 8	288305	-0.56519	8.3368	-4.2563	0.0019341	0.020801	-1.2383
Hadh	hydroxyacyl-CoA dehydrogenase	113965	-0.56403	7.2109	-5.1693	0.00051553	0.010941	0.10096
Fkbp8	FKBP prolyl isomerase 8	290652	-0.55038	9.4746	-3.6181	0.0052234	0.034467	-2.2422
Actn2	actinin alpha 2	291245	-0.54474	7.7036	-5.8051	0.00022027	0.0082162	0.95884
Acsl1	acyl-CoA synthetase long-chain family me	25288	-0.53375	7.2801	-5.986	0.00017469	0.0076421	1.1918

Acadl	"acyl-CoA dehydrogenase, long chain"	25287	-0.52804	7.8894	-5.4865	0.00033491	0.010041	0.53672
L2hgdh	L-2-hydroxyglutarate dehydrogenase	314196	-0.52508	7.4899	-4.8601	0.00079598	0.013869	-0.33875
Oxct1	3-oxoacid CoA transferase 1	690163	-0.52382	6.5299	-5.1651	0.00051855	0.010941	0.095039
Scamp1	secretory carrier membrane protein 1	29521	-0.52374	7.1226	-3.3981	0.0074438	0.040544	-2.5982
Atp5pd	ATP synthase peripheral stalk subunit d	641434	-0.52014	7.1499	-5.0979	0.0005692	0.011603	0.00076529
Gpx4	glutathione peroxidase 4	29328	-0.51886	5.5802	-3.5077	0.0062353	0.037787	-2.4203
Ndufs7	NADH:ubiquinone oxidoreductase core su	362837	-0.51478	8.2663	-4.3381	0.0017096	0.019503	-1.1134
Gsn	gelsolin	296654	-0.50994	7.1358	-3.4467	0.0068808	0.039593	-2.5193
Fbn1	fibrillin 1	83727	-0.50574	6.2328	-5.7437	0.00023853	0.0083194	0.8787
Rps5	ribosomal protein S5	25538	-0.50493	9.7947	-4.5084	0.0013266	0.017654	-0.85636
Myom2	myomesin 2	306616	-0.49716	8.6367	-5.7565	0.00023459	0.0083194	0.89547
Hsdl2	hydroxysteroid dehydrogenase like 2	313200	-0.49715	5.9847	-5.1758	0.00051094	0.010941	0.11
Acsf2	acyl-CoA synthetase family member 2	619561	-0.49408	6.3586	-5.2679	0.00045018	0.010539	0.238
Cltc	clathrin heavy chain	54241	-0.49338	9.6208	-5.5505	0.00030754	0.0097332	0.6227
Nnt	nicotinamide nucleotide transhydrogenase	310378	-0.48757	6.7339	-5.5043	0.00032703	0.009998	0.56074
Ptgis	prostaglandin I2 synthase	25527	-0.48682	8.2012	-3.6587	0.0048963	0.033103	-2.1771
Hba1	"hemoglobin, alpha 1"	25632	-0.48674	4.6673	-4.8191	0.00084401	0.013869	-0.3981
Gpi	glucose-6-phosphate isomerase	292804	-0.48599	10.552	-3.8884	0.0034073	0.027996	-1.8113
Hspa8	heat shock protein family A (Hsp70) memb	24468	-0.48569	10.091	-5.0571	0.00060258	0.011603	-0.056901
LOC1009111	mitochondrial import inner membrane trans	100911130	-0.48329	5.2966	-3.1712	0.01078	0.051463	-2.9685
Mtdh	metadherin	170910	-0.47875	8.1621	-4.0332	0.0027204	0.024729	-1.5837
Apoe	apolipoprotein E	25728	-0.4781	8.0673	-2.6632	0.025041	0.085761	-3.8012
Add3	adducin 3	25230	-0.47676	8.412	-4.1615	0.002234	0.022591	-1.3843
Arl6ip5	ADP-ribosylation factor like GTPase 6 inte	66028	-0.4765	7.4691	-2.6843	0.024174	0.083622	-3.7667
Serpinh1	serpin family H member 1	29345	-0.47612	7.936	-4.2397	0.0019835	0.021055	-1.2639
Ak3	adenylate kinase 3	26956	-0.47392	8.0226	-4.0611	0.0026058	0.024084	-1.5401
Tgm2	transglutaminase 2	56083	-0.47115	8.8077	-4.3735	0.0016212	0.018848	-1.0596
Atp5f1c	ATP synthase F1 subunit gamma	116550	-0.47076	7.4849	-4.3795	0.0016068	0.018848	-1.0506
Rras2	RAS related 2	365355	-0.46908	9.0765	-4.0625	0.0026001	0.024084	-1.5379
Lap3	leucine aminopeptidase 3	289668	-0.46784	6.1402	-4.3271	0.0017381	0.019503	-1.1301
Snap23	synaptosome associated protein 23	64630	-0.46521	5.6998	-4.415	0.0015239	0.018341	-0.99687
Spg7	"SPG7 matrix AAA peptidase subunit, par	353231	-0.4649	10.055	-3.7467	0.0042581	0.030966	-2.0363
Isca2	iron-sulfur cluster assembly 2	500694	-0.46398	8.1081	-3.3063	0.0086427	0.044489	-2.7477
Ivd	isovaleryl-CoA dehydrogenase	24513	-0.46313	4.518	-4.0175	0.0027873	0.025071	-1.6083
Letm1	leucine zipper and EF-hand containing tra	305457	-0.46038	9.2587	-5.3373	0.0004095	0.010256	0.33372
Capn2	calpain 2	29154	-0.45964	3.8984	-3.6704	0.004806	0.032985	-2.1583
Pnpt1	polyribonucleotide nucleotidyltransferase	360992	-0.45657	8.7307	-4.5637	0.0012228	0.017157	-0.77375
Acadm	acyl-CoA dehydrogenase medium chain	24158	-0.45574	7.3951	-4.557	0.0012348	0.017157	-0.78367
Ndufs1	NADH:ubiquinone oxidoreductase core su	301458	-0.45412	7.8472	-5.3861	0.0003833	0.010256	0.40049
Mlec	malectin	304543	-0.45208	7.8094	-3.0836	0.012455	0.05661	-3.1122

Prdx3	peroxiredoxin 3	64371	-0.45143	8.8421	-3.8564	0.0035823	0.028758	-1.8619
Sod2	superoxide dismutase 2	24787	-0.44722	6.0572	-4.4314	0.0014871	0.018341	-0.97205
Psma5	proteasome 20S subunit alpha 5	29672	-0.44674	8.3655	-3.799	0.0039203	0.030271	-1.9529
Ndufb7	NADH:ubiquinone oxidoreductase subunit	361385	-0.4456	9.5038	-4.3995	0.0015596	0.018639	-1.0203
Dglucy	D-glutamate cyclase	362769	-0.44095	6.2543	-3.8702	0.0035056	0.028394	-1.84
Sdhc	succinate dehydrogenase complex subun	289217	-0.44067	7.65	-4.1318	0.002338	0.023096	-1.4304
Anxa5	annexin A5	25673	-0.43911	6.7291	-4.707	0.00099194	0.015272	-0.56173
Tln1	talin 1	313494	-0.43803	9.3624	-5.0097	0.00064398	0.012094	-0.12416
Mrps23	mitochondrial ribosomal protein S23	360594	-0.43799	4.6653	-3.7808	0.0040344	0.030541	-1.9819
Pdk2	pyruvate dehydrogenase kinase 2	81530	-0.43742	6.4984	-4.1052	0.002435	0.023511	-1.4716
Phb2	prohibitin 2	114766	-0.4368	11.247	-4.1849	0.0021558	0.022329	-1.3483
Cryab	"crystallin, alpha B"	25420	-0.43615	7.1406	-4.3488	0.0016824	0.019428	-1.0971
Cd36	CD36 molecule	29184	-0.43548	6.642	-4.7681	0.0009081	0.01437	-0.47225
Hbb	hemoglobin subunit beta	24440	-0.43067	7.2519	-4.1638	0.0022263	0.022591	-1.3808
Psmc1	"proteasome 26S subunit, ATPase 1"	117263	-0.42919	5.6645	-2.852	0.01828	0.070679	-3.4922
Hsd17b4	hydroxysteroid (17-beta) dehydrogenase 4	79244	-0.42797	5.2064	-4.4392	0.0014699	0.018341	-0.96033
Atp5f1b	ATP synthase F1 subunit beta	171374	-0.42406	8.1133	-4.8303	0.00083063	0.013869	-0.38191
D2hgdh	D-2-hydroxyglutarate dehydrogenase	301624	-0.42375	9.6561	-3.0231	0.013762	0.059708	-3.2114
Ckm	"creatine kinase, M-type"	24265	-0.42034	7.4941	-3.8821	0.0034408	0.028002	-1.8212
Idh3g	isocitrate dehydrogenase (NAD(+)) 3 non-	25179	-0.41871	8.1301	-4.5617	0.0012264	0.017157	-0.77674
Maoa	monoamine oxidase A	29253	-0.41801	13.988	-4.4986	0.0013459	0.017694	-0.871
Ndufc2	NADH:ubiquinone oxidoreductase subunit	293130	-0.41655	8.1434	-3.0258	0.013703	0.059708	-3.2071
Tpp1	tripeptidyl peptidase 1	83534	-0.4158	8.5783	-3.2178	0.0099876	0.048629	-2.8923
Myl2	myosin light chain 2	363925	-0.41013	5.4182	-4.2809	0.0018635	0.02051	-1.2007
Canx	calnexin	29144	-0.40699	7.7394	-3.4557	0.0067809	0.039392	-2.5046
Arf1	ADP-ribosylation factor 1	64310	-0.40692	8.8206	-2.8805	0.017435	0.068496	-3.4455
Got2	glutamic-oxaloacetic transaminase 2	25721	-0.40622	4.7538	-4.1799	0.0021722	0.022364	-1.3559
Scp2	sterol carrier protein 2	25541	-0.40401	7.3404	-4.2736	0.0018841	0.02051	-1.2118
Aldh7a1	"aldehyde dehydrogenase 7 family, memb	291450	-0.40265	6.0363	-3.7971	0.0039323	0.030271	-1.956
Vcl	vinculin	305679	-0.40209	7.0865	-4.6037	0.0011531	0.016811	-0.71427
Tfrc	transferrin receptor	64678	-0.40062	7.3299	-2.8921	0.017103	0.06765	-3.4265
Ndufaf6	NADH:ubiquinone oxidoreductase comple	297821	-0.40041	4.9781	-3.6386	0.0050551	0.033747	-2.2092
Rps17	ribosomal protein S17	29286	-0.39918	7.5339	-2.2842	0.04711	0.12986	-4.4118
Fam162a	"family with sequence similarity 162, memb	360721	-0.39479	5.3293	-3.777	0.0040588	0.030557	-1.988
Pacsin2	protein kinase C and casein kinase substr	124461	-0.39403	7.2649	-3.8833	0.0034342	0.028002	-1.8192
Ywhaz	"tyrosine 3-monooxygenase/tryptophan 5-	25578	-0.39372	5.7897	-3.454	0.0067997	0.039392	-2.5074
Acad10	"acyl-CoA dehydrogenase family, member	304500	-0.39294	6.5817	-3.7169	0.0044636	0.031653	-2.0838
Aco2	aconitase 2	79250	-0.39183	10.121	-4.5872	0.0011812	0.016964	-0.73869
Nckap1	NCK-associated protein 1	58823	-0.39101	7.264	-2.7721	0.020881	0.07658	-3.6232
Ldhb	lactate dehydrogenase B	24534	-0.38956	6.7316	-3.9358	0.0031642	0.027038	-1.7365

Clu	clusterin	24854	-0.38912	6.1887	-3.4593	0.0067418	0.039324	-2.4988
Mrpl38	mitochondrial ribosomal protein L38	303685	-0.38493	7.6583	-3.3171	0.0084914	0.044078	-2.7301
Cdh13	cadherin 13	192248	-0.38368	5.726	-4.0739	0.0025553	0.024084	-1.5203
Ssr4	signal sequence receptor subunit 4	29435	-0.38346	9.603	-3.7199	0.0044424	0.031634	-2.0791
Pnpla8	patatin-like phospholipase domain contair	314075	-0.38272	6.762	-3.0445	0.013284	0.058662	-3.1763
Gcdh	glutaryl-CoA dehydrogenase	364975	-0.3806	7.915	-3.0801	0.012527	0.056637	-3.118
Gfm1	"G elongation factor, mitochondrial 1"	114017	-0.38036	5.3454	-3.8132	0.0038337	0.029917	-1.9304
Ldha	lactate dehydrogenase A	24533	-0.37965	9.3832	-3.9315	0.0031853	0.027083	-1.7432
Rplp0	ribosomal protein lateral stalk subunit P0	64205	-0.37892	6.9843	-3.1083	0.011957	0.055108	-3.0717
Afg3l2	AFG3 like matrix AAA peptidase subunit 2	307350	-0.37829	7.7782	-4.2207	0.0020415	0.021536	-1.2931
Vdac1	voltage-dependent anion channel 1	83529	-0.37816	10.822	-4.0597	0.0026115	0.024084	-1.5424
Rpl30	ribosomal protein L30	64640	-0.37735	7.1431	-2.6143	0.027172	0.0908	-3.8809
Pebp1	phosphatidylethanolamine binding protein	29542	-0.37703	7.8785	-3.5409	0.0059111	0.036833	-2.3667
Bsg	basigin (Ok blood group)	25246	-0.3761	7.1242	-3.4214	0.0071682	0.040118	-2.5603
Cav1	caveolin 1	25404	-0.37594	6.812	-3.6805	0.0047291	0.032721	-2.1421
Ndufaf4	NADH:ubiquinone oxidoreductase comple	362495	-0.37457	8.0955	-3.0523	0.013115	0.058219	-3.1636
Atp1b1	ATPase Na+/K+ transporting subunit beta	25650	-0.37372	8.3821	-3.5586	0.0057456	0.035968	-2.3381
Ppif	peptidylprolyl isomerase F	282819	-0.37143	9.9008	-2.9602	0.015274	0.06351	-3.3147
Ank1	ankyrin 1	306570	-0.37123	7.3074	-4.0705	0.0025686	0.024084	-1.5256
Pccb	propionyl-CoA carboxylase subunit beta	24624	-0.36863	6.2307	-3.3918	0.0075201	0.040544	-2.6084
Ndufb4	NADH:ubiquinone oxidoreductase subunit	288088	-0.36808	7.4614	-3.7216	0.0044301	0.031634	-2.0762
Sgcd	"sarcoglycan, delta"	497892	-0.36802	9.0502	-3.666	0.0048392	0.033081	-2.1653
Slmap	sarcolemma associated protein	290533	-0.36692	6.9961	-3.5037	0.0062752	0.037895	-2.4267
C3	complement C3	24232	-0.36606	9.872	-3.7732	0.0040834	0.030559	-1.9941
Bcs1l	"BCS1 homolog, ubiquinol-cytochrome c r	301514	-0.36539	6.7156	-3.8379	0.0036879	0.029179	-1.8912
Flnb	filamin B	306204	-0.36187	10.151	-3.512	0.006192	0.037659	-2.4133
Afg1l	AFG1 like ATPase	502479	-0.36187	4.3849	-2.3636	0.041287	0.12018	-4.2856
Pecr	peroxisomal trans-2-enoyl-CoA reductase	113956	-0.36053	8.5154	-3.6083	0.0053058	0.034554	-2.258
Rrbp1	ribosome binding protein 1	311483	-0.35827	7.3677	-3.5213	0.0061005	0.037368	-2.3984
Timm50	translocase of inner mitochondrial membra	687295	-0.35662	7.7723	-3.5716	0.0056274	0.035357	-2.3172
Ndufs4	NADH:ubiquinone oxidoreductase subunit	499529	-0.35656	5.4386	-3.6069	0.0053176	0.034554	-2.2602
Gstz1	glutathione S-transferase zeta 1	681913	-0.35532	10.906	-3.5393	0.0059269	0.036833	-2.3693
Epb42	erythrocyte membrane protein band 4.2	362202	-0.35431	5.4355	-3.1911	0.010435	0.050256	-2.936
Srl	sarcalumenin	302948	-0.35376	9.6769	-3.975	0.0029773	0.026363	-1.6749
Septin7	septin 7	64551	-0.35114	9.242	-3.2711	0.0091537	0.046092	-2.8052
Napa	NSF attachment protein alpha	140673	-0.34984	7.0637	-3.4486	0.0068591	0.039593	-2.5161
Pdcd6ip	programmed cell death 6 interacting prote	501083	-0.34925	6.898	-3.2311	0.0097726	0.047992	-2.8705
Anxa4	annexin A4	79124	-0.34888	9.1807	-2.9261	0.016163	0.065302	-3.3707
Kif5b	kinesin family member 5B	117550	-0.34802	6.3351	-3.2202	0.0099481	0.048575	-2.8883
Dguok	deoxyguanosine kinase	297389	-0.34694	6.1718	-3.0082	0.014106	0.06057	-3.2359

C1qbp	complement C1q binding protein	29681	-0.34674	9.8581	-3.4987	0.0063268	0.038072	-2.435
Anxa11	annexin A11	290527	-0.3457	8.8954	-3.4085	0.0073196	0.040544	-2.5813
Camk2d	calcium/calmodulin-dependent protein kin	24246	-0.34521	8.5834	-2.7758	0.020753	0.07658	-3.6171
Csrp3	cysteine and glycine rich protein 3	117505	-0.34479	5.0574	-3.2601	0.0093186	0.046484	-2.823
Cox6a2	cytochrome c oxidase subunit 6A2	25278	-0.34378	5.8504	-3.423	0.0071495	0.040118	-2.5577
Actn4	actinin alpha 4	63836	-0.34179	5.589	-3.3357	0.0082385	0.042926	-2.6998
Pls3	plastin 3	81748	-0.34102	6.6598	-3.2888	0.0088923	0.045519	-2.7762
Lsamp	limbic system-associated membrane prote	29561	-0.33979	7.0194	-2.6391	0.026069	0.088164	-3.8405
Ipo5	importin 5	306182	-0.33902	10.877	-2.4597	0.035173	0.10829	-4.1313
Aifm1	"apoptosis inducing factor, mitochondria a	83533	-0.33886	11.514	-3.9785	0.0029609	0.026355	-1.6694
Atp2a2	ATPase sarcoplasmic/endoplasmic reticulu	29693	-0.33708	11.718	-3.7118	0.0044997	0.031777	-2.092
Ccdc141	coiled-coil domain containing 141	311134	-0.33615	9.2512	-3.3644	0.0078619	0.041688	-2.6529
Ndufa9	NADH:ubiquinone oxidoreductase subunit	362440	-0.33531	5.68	-3.8395	0.0036785	0.029179	-1.8886
Psmd1	"proteasome 26S subunit, non-ATPase 1"	83806	-0.33519	10.524	-2.6669	0.024887	0.085576	-3.7951
Rpl3l	ribosomal protein L3-like	287122	-0.33335	6.2376	-3.6581	0.0049006	0.033103	-2.178
Apoh	apolipoprotein H	287774	-0.3325	11.003	-2.8285	0.019011	0.072358	-3.5308
Plg	plasminogen	85253	-0.33132	5.8084	-3.3421	0.0081523	0.042653	-2.6893
Pdhx	"pyruvate dehydrogenase complex, comp	311254	-0.33115	6.0016	-3.0253	0.013713	0.059708	-3.2078
Itga7	integrin subunit alpha 7	81008	-0.3307	6.8043	-3.5951	0.0054189	0.034818	-2.2792
Tubb4b	"tubulin, beta 4B class IVb"	296554	-0.32936	4.9416	-3.4202	0.0071819	0.040118	-2.5622
Itga5	integrin subunit alpha 5	315346	-0.326	6.5696	-2.8812	0.017415	0.068496	-3.4444
Slc25a18	solute carrier family 25 member 18	681896	-0.32548	7.4631	-3.4395	0.0069608	0.039786	-2.5309
Dlst	dihydrolipoamide S-succinyltransferase	299201	-0.32303	9.7487	-3.4157	0.0072343	0.040272	-2.5695
Cyb5r1	cytochrome b5 reductase 1	304805	-0.32135	6.7399	-2.8373	0.018734	0.071777	-3.5164
Ctnnd1	catenin delta 1	311163	-0.32017	9.5039	-2.7388	0.022075	0.079592	-3.6777
Mdh2	malate dehydrogenase 2	81829	-0.31751	7.9219	-3.6956	0.004617	0.032471	-2.1179
Sgcb	"sarcoglycan, beta"	680229	-0.31473	8.3635	-3.3821	0.0076391	0.040925	-2.6241
Bckdhb	branched chain keto acid dehydrogenase	29711	-0.3147	6.0586	-2.8374	0.018731	0.071777	-3.5162
Prdx5	peroxiredoxin 5	113898	-0.31197	8.2831	-2.919	0.016355	0.065812	-3.3824
Acot2	acyl-CoA thioesterase 2	192272	-0.31159	5.3518	-3.0532	0.013096	0.058219	-3.1621
Acot9	acyl-CoA thioesterase 9	302640	-0.31124	7.3547	-3.108	0.011963	0.055108	-3.0722
Ap2a2	adaptor related protein complex 2 subunit	81637	-0.31119	7.0725	-2.4967	0.033066	0.10369	-4.0716
Vat1	vesicle amine transport 1	287721	-0.31063	9.0927	-3.0233	0.013758	0.059708	-3.2111
Idh2	isocitrate dehydrogenase (NADP(+)) 2	361596	-0.30587	7.0731	-3.5935	0.0054335	0.034818	-2.2819
Mtarc2	mitochondrial amidoxime reducing compor	171451	-0.30564	8.7946	-2.4069	0.03841	0.11456	-4.2162
Cfl1	cofilin 1	29271	-0.30519	6.2812	-2.7119	0.023086	0.081855	-3.7216
Fam210a	"family with sequence similarity 210, memb	307343	-0.30483	5.8958	-3.4849	0.0064689	0.038387	-2.4573
Ca14	carbonic anhydrase 14	791259	-0.30334	5.7316	-2.5761	0.028963	0.093922	-3.943
Park7	Parkinsonism associated deglycase	117287	-0.29996	12.133	-2.9774	0.014845	0.062029	-3.2865
Hrg	histidine-rich glycoprotein	171016	-0.29925	7.486	-2.4666	0.034769	0.10764	-4.1202

Lactb	"lactamase, beta"	300803	-0.29861	6.0123	-2.6832	0.024221	0.083622	-3.7686
Phb	prohibitin	25344	-0.29816	6.8819	-3.2562	0.0093796	0.046598	-2.8295
Msn	moesin	81521	-0.29797	6.2268	-3.352	0.0080224	0.042316	-2.6732
Cct3	chaperonin containing TCP1 subunit 3	295230	-0.29785	6.3611	-2.617	0.027052	0.09065	-3.8766
Pcyt1a	"phosphate cytidylyltransferase 1, choline	140544	-0.29782	5.6791	-2.4358	0.036605	0.11092	-4.1698
Sgca	"sarcoglycan, alpha"	303468	-0.29777	9.6186	-2.7436	0.0219	0.079127	-3.6699
Tufm	"Tu translation elongation factor, mitochor	293481	-0.2973	6.773	-3.4025	0.0073909	0.040544	-2.591
Mrpl45	mitochondrial ribosomal protein L45	287656	-0.29678	7.9167	-2.6916	0.023884	0.083302	-3.7549
Marcks	myristoylated alanine rich protein kinase C	25603	-0.29643	8.2942	-2.7834	0.020492	0.076134	-3.6047
Arhgap1	Rho GTPase activating protein 1	311193	-0.29629	8.8164	-2.4598	0.035165	0.10829	-4.1311
Vtn	vitronectin	29169	-0.29479	10.272	-2.836	0.018774	0.071777	-3.5185
Lama2	laminin subunit alpha 2	309368	-0.29426	7.7659	-2.8131	0.019505	0.073585	-3.5561
Rpn2	ribophorin II	64701	-0.29355	5.6105	-3.0148	0.013954	0.060096	-3.2251
Prkar1a	protein kinase cAMP-dependent type I reg	25725	-0.29341	8.3214	-2.423	0.037395	0.11212	-4.1904
Tomm22	translocase of outer mitochondrial membra	300075	-0.29299	5.8542	-3.1829	0.010576	0.050769	-2.9494
Mb	myoglobin	59108	-0.29208	6.4978	-3.0397	0.013391	0.058984	-3.1843
Plin4	perilipin 4	363331	-0.29119	7.6274	-3.3148	0.0085237	0.044078	-2.7339
Rrad	"RRAD, Ras related glycolysis inhibitor and	83521	-0.29014	8.9408	-2.7207	0.02275	0.081168	-3.7073
Suclg2	"succinate-CoA ligase, GDP-forming, beta	362404	-0.2901	9.3017	-3.3833	0.0076241	0.040925	-2.6222
Ociad1	OCIA domain containing 1	289590	-0.28903	11.669	-2.7803	0.020599	0.076362	-3.6098
Rpl8	ribosomal protein L8	26962	-0.28889	5.8809	-2.6521	0.025509	0.086867	-3.8193
Coq8a	coenzyme Q8A	360887	-0.28846	7.5976	-3.2753	0.0090912	0.046092	-2.7983
Trak1	trafficking kinesin protein 1	316085	-0.28814	9.5412	-2.2946	0.046304	0.12888	-4.3953
Cd47	Cd47 molecule	29364	-0.28591	10.326	-2.2973	0.046098	0.1287	-4.3911
Cs	citrate synthase	170587	-0.28577	11.353	-2.997	0.014371	0.061246	-3.2543
Art3	ADP-ribosyltransferase 3	305235	-0.28555	6.7837	-3.137	0.011406	0.053846	-3.0247
Vdac2	voltage-dependent anion channel 2	83531	-0.2846	10.137	-3.0226	0.013774	0.059708	-3.2122
Fga	fibrinogen alpha chain	361969	-0.28414	8.1176	-3.1274	0.011586	0.054099	-3.0403
Sdhb	succinate dehydrogenase complex iron su	298596	-0.28408	5.7843	-3.0064	0.014149	0.060605	-3.2389
Ech1	enoyl-CoA hydratase 1	64526	-0.28347	8.7277	-3.1976	0.010323	0.050122	-2.9253
Agk	acylglycerol kinase	502749	-0.28277	9.0354	-2.6991	0.023584	0.083069	-3.7426
Rps4x	"ribosomal protein S4, X-linked"	100362640	-0.28162	8.9937	-2.8261	0.019084	0.07236	-3.5347
Agps	alkylglycerone phosphate synthase	84114	-0.2787	7.8907	-2.4619	0.035045	0.10829	-4.1278
Pdp1	pyruvate dehyrogenase phosphatase cata	54705	-0.27767	10.681	-2.4968	0.033063	0.10369	-4.0715
Myl7	myosin light chain 7	289759	-0.27711	5.9588	-2.4257	0.037225	0.112	-4.186
Mtx1	Metaxin 1	295241	-0.2769	4.6572	-2.8908	0.01714	0.06765	-3.4287
Ndrg2	NDRG family member 2	171114	-0.2768	6.8246	-2.9019	0.016826	0.06672	-3.4104
Slc25a3	solute carrier family 25 member 3	245959	-0.27442	7.5176	-3.1908	0.010439	0.050256	-2.9364
Gstk1	glutathione S-transferase kappa 1	297029	-0.27413	6.1191	-2.7921	0.020199	0.075506	-3.5905
Jph2	junctophilin 2	296345	-0.27412	8.7925	-3.0984	0.012154	0.055685	-3.0879

Dpysl2	dihydropyrimidinase-like 2	25416	-0.27397	13.184	-2.7913	0.020224	0.075506	-3.5917
Stip1	stress-induced phosphoprotein 1	192277	-0.27312	5.164	-2.7626	0.021217	0.077477	-3.6388
Gyg1	glycogenin 1	81675	-0.27261	7.4721	-2.3281	0.043799	0.12434	-4.3422
Ppia	peptidylprolyl isomerase A	25518	-0.2718	7.7172	-2.6017	0.027752	0.09155	-3.9015
Mgll	monoglyceride lipase	29254	-0.2712	7.5797	-2.8436	0.018537	0.071352	-3.506
RGD1565784	RGD1565784	497874	-0.26801	4.3687	-2.5717	0.029173	0.094425	-3.9501
Uqcr10	"ubiquinol-cytochrome c reductase, compl	685322	-0.26729	5.0553	-2.964	0.015179	0.063269	-3.3085
Anxa1	annexin A1	25380	-0.2669	8.8125	-2.6125	0.027256	0.0908	-3.8839
Abhd16a	abhydrolase domain containing 16A	361796	-0.26667	8.7283	-2.3699	0.040855	0.11935	-4.2755
Ndufaf2	NADH:ubiquinone oxidoreductase comple	361894	-0.26662	11.324	-2.4937	0.033234	0.10383	-4.0765
Ndufv1	NADH:ubiquinone oxidoreductase core su	293655	-0.26591	7.877	-2.9464	0.015627	0.064197	-3.3373
Shmt2	serine hydroxymethyltransferase 2	299857	-0.26223	13.586	-2.271	0.048156	0.13168	-4.4327
Bdh1	3-hydroxybutyrate dehydrogenase 1	117099	-0.26211	6.7564	-3.0542	0.013073	0.058219	-3.1604
Atad1	"ATPase family, AAA domain containing 1	309532	-0.26124	5.2473	-2.6405	0.026009	0.088164	-3.8382
Pdia6	"protein disulfide isomerase family A, mem	286906	-0.26014	7.5146	-2.4076	0.038367	0.11456	-4.2151
Mrps22	mitochondrial ribosomal protein S22	683519	-0.25745	5.543	-2.3823	0.040019	0.11751	-4.2557
Ndufab1	NADH:ubiquinone oxidoreductase subuni	293453	-0.25663	5.8922	-2.5427	0.030623	0.097601	-3.9972
Rpsa	ribosomal protein SA	29236	-0.25443	9.1253	-2.6461	0.025768	0.087549	-3.8291
Cavin3	caveolae associated protein 3	85332	-0.25423	7.34	-2.5585	0.029825	0.09581	-3.9716
Pdia4	"protein disulfide isomerase family A, mem	116598	-0.25344	6.5032	-2.5507	0.030217	0.096524	-3.9842
Hadha	hydroxyacyl-CoA dehydrogenase trifunctio	170670	-0.25158	8.7169	-2.9536	0.015442	0.063744	-3.3255
Hspb1	heat shock protein family B (small) membe	24471	-0.25142	7.6426	-2.8186	0.019325	0.073069	-3.547
Rab11b	"RAB11B, member RAS oncogene family"	79434	-0.25118	8.1972	-2.7493	0.021692	0.078708	-3.6606
Suclg1	"succinate-CoA ligase, alpha subunit"	114597	-0.24729	8.4867	-2.3344	0.043341	0.12345	-4.3321
Mrpl50	mitochondrial ribosomal protein L50	362517	-0.24681	9.9928	-2.5675	0.029382	0.094645	-3.957
Naxe	NAD(P)HX epimerase	295229	-0.24589	7.1399	-2.4537	0.035526	0.109	-4.141
Slc25a5	solute carrier family 25 member 5	25176	-0.24141	6.8102	-2.7209	0.022743	0.081168	-3.707
Prodh1	proline dehydrogenase 1	680409	-0.24071	5.8422	-2.356	0.04181	0.1209	-4.2977
Txn2	thioredoxin 2	79462	-0.23837	6.3891	-2.3721	0.040702	0.11931	-4.2719
Txnl1	thioredoxin-like 1	140922	-0.2368	7.1414	-2.4673	0.034729	0.10764	-4.1191
Cyc1	cytochrome c-1	300047	-0.23649	6.253	-2.6084	0.027442	0.091065	-3.8905
Samm50	SAMM50 sorting and assembly machinery	300111	-0.23636	10.06	-2.657	0.025305	0.086491	-3.8114
Map3k20	mitogen-activated protein kinase kinase ki	311743	-0.23469	6.1045	-2.4321	0.03683	0.11101	-4.1758
Dpysl3	dihydropyrimidinase-like 3	25418	-0.23442	8.7928	-2.3085	0.045248	0.12677	-4.3733
Mrpl12	mitochondrial ribosomal protein L12	303746	-0.23395	8.0782	-2.3414	0.042841	0.12243	-4.321
Col6a1	collagen type VI alpha 1 chain	294337	-0.233	6.6519	-2.4587	0.035232	0.10829	-4.133
Ak4	adenylate kinase 4	29223	-0.23147	6.8695	-2.287	0.046894	0.12986	-4.4074
Gcsh	glycine cleavage system protein H	171133	-0.23087	8.8427	-2.5952	0.028054	0.091848	-3.912
Srprb	SRP receptor subunit beta	300965	-0.23019	8.3237	-2.6057	0.027567	0.091126	-3.895
Uqcrb	ubiquinol-cytochrome c reductase binding	362897	-0.22908	6.1605	-2.4503	0.035727	0.10942	-4.1464

Rtn4ip1	reticulon 4 interacting protein 1	309912	-0.22894	10.211	-2.581	0.028726	0.093332	-3.935
Tln2	talin 2	315776	-0.22805	8.4505	-2.6965	0.023688	0.083069	-3.7469
Anpep	"alanyl aminopeptidase, membrane"	81641	-0.22749	8.5428	-2.3952	0.039168	0.11561	-4.235
Pygb	glycogen phosphorylase B	25739	-0.2274	5.3573	-2.4947	0.033176	0.10383	-4.0748
Mfn1	mitofusin 1	192647	-0.22682	6.8245	-2.2846	0.047078	0.12986	-4.4112
Atpaf1	ATP synthase mitochondrial F1 complex a	313510	-0.22631	5.5161	-2.2773	0.047653	0.13072	-4.4227
Atic	5-aminoimidazole-4-carboxamide ribonucle	81643	-0.22497	4.7813	-2.2522	0.049683	0.13507	-4.4624
Hrc	histidine rich calcium binding protein	292905	-0.22469	7.6239	-2.2655	0.048595	0.13267	-4.4414
Hsd17b10	hydroxysteroid (17-beta) dehydrogenase	63864	-0.2244	6.9942	-2.5231	0.031643	0.099592	-4.029
Blvrb	biliverdin reductase B	292737	-0.22427	5.0892	-2.3133	0.044889	0.12597	-4.3657
Synj2bp	synaptojanin 2 binding protein	64531	-0.22348	6.4972	-2.4379	0.036475	0.11072	-4.1664
Cisd2	CDGSH iron sulfur domain 2	295457	-0.22119	8.4778	-2.3981	0.038977	0.11542	-4.2303
Cpt1a	carnitine palmitoyltransferase 1A	25757	-0.22095	6.3118	-2.289	0.046739	0.12986	-4.4043
Flot2	flotillin 2	83764	-0.21976	4.6437	-2.4396	0.036373	0.11072	-4.1637
Fech	ferrochelatase	361338	-0.21764	8.4828	-2.3524	0.04206	0.12121	-4.3034
Ndufa5	NADH:ubiquinone oxidoreductase subunit	25488	-0.21665	7.8998	-2.3192	0.044449	0.12515	-4.3563
Rab1b	"RAB1B, member RAS oncogene family"	100126191	-0.21568	5.0019	-2.3547	0.041904	0.12097	-4.2998
Erlin2	ER lipid raft associated 2	290823	-0.21554	6.1932	-2.3997	0.038873	0.11542	-4.2277
Hmgcl	3-hydroxy-3-methylglutaryl-CoA lyase	79238	-0.21348	6.8008	-2.2801	0.047432	0.13053	-4.4183
Rpl12	ribosomal protein L12	499782	-0.20968	10.507	-2.2965	0.046163	0.1287	-4.3924
Ndufs6	NADH:ubiquinone oxidoreductase subunit	29478	-0.19663	7.989	-2.32	0.044394	0.12515	-4.3551
Ndufa13	NADH:ubiquinone oxidoreductase subunit	100911483	0.21183	8.5495	2.2785	0.047561	0.13068	-4.4209
Uqcrq	"ubiquinol-cytochrome c reductase, compl	497902	0.21356	4.3667	2.3665	0.041083	0.11981	-4.2808
Ndufb5	NADH:ubiquinone oxidoreductase subunit	294964	0.21926	6.4896	2.3514	0.042129	0.12121	-4.305
Nipsnap2	nipsnap homolog 2	498174	0.22083	5.0798	2.4233	0.037372	0.11212	-4.1898
Apool	apolipoprotein O-like	317191	0.22215	6.1807	2.2763	0.047733	0.13073	-4.4243
Etfa	electron transfer flavoprotein subunit alph	300726	0.22593	7.1783	2.6733	0.024622	0.084836	-3.7847
Nln	neurolysin	117041	0.22828	7.3986	2.5542	0.030039	0.096315	-3.9785
Fgb	fibrinogen beta chain	24366	0.22883	4.3566	2.4338	0.036727	0.11101	-4.173
Hccs	holocytochrome c synthase	317444	0.2292	6.8284	2.5818	0.028686	0.093332	-3.9337
Tubb5	"tubulin, beta 5 class I"	29214	0.23037	7.8595	2.4693	0.034617	0.10756	-4.1159
Rac1	Rac family small GTPase 1	363875	0.23141	7.4058	2.3327	0.043461	0.12359	-4.3348
Slc25a46	"solute carrier family 25, member 46"	291709	0.23526	4.3759	2.3064	0.045408	0.12701	-4.3767
Cox20	cytochrome c oxidase assembly factor CO	289278	0.23567	5.1167	2.4409	0.036292	0.11072	-4.1616
Fhl1	four and a half LIM domains 1	25177	0.2368	4.5045	2.3707	0.040798	0.11935	-4.2742
Cox15	cytochrome c oxidase assembly homolog	309391	0.23707	8.0275	2.3606	0.04149	0.12018	-4.2903
Mmab	metabolism of cobalamin associated B	687861	0.24141	7.7333	2.2506	0.049812	0.13512	-4.4649
Prdx6	peroxiredoxin 6	94167	0.24362	11.646	2.2605	0.049002	0.13356	-4.4493
Cct7	chaperonin containing TCP1 subunit 7	297406	0.24674	6.3398	2.2518	0.049714	0.13507	-4.463
Clic1	chloride intracellular channel 1	406864	0.24739	7.0237	2.4747	0.034301	0.10678	-4.1071

Mrps21	mitochondrial ribosomal protein S21	689432	0.24845	6.7849	2.5519	0.030158	0.096517	-3.9823
Abcb10	ATP binding cassette subfamily B membe	361439	0.24939	6.4755	2.3263	0.043929	0.1245	-4.345
Myom1	myomesin 1	316740	0.25012	6.0617	2.316	0.044684	0.1256	-4.3613
Ganab	glucosidase II alpha subunit	293721	0.25098	9.5792	2.349	0.042301	0.1213	-4.3089
Gpd2	glycerol-3-phosphate dehydrogenase 2	25062	0.2525	8.493	2.8258	0.019096	0.07236	-3.5352
Plbd1	phospholipase B domain containing 1	297694	0.2529	6.4315	2.3363	0.043204	0.12327	-4.3291
Glrx5	glutaredoxin 5	362776	0.25368	7.0495	2.4783	0.0341	0.10634	-4.1014
Acad8	"acyl-CoA dehydrogenase family, member	367196	0.25385	7.3498	2.8645	0.017905	0.069545	-3.4718
Rplp2	ribosomal protein lateral stalk subunit P2	140662	0.25385	10.151	2.384	0.039907	0.11739	-4.253
Tmem126a	transmembrane protein 126A	293113	0.2564	7.6483	2.6226	0.026796	0.090097	-3.8673
Myl3	myosin light chain 3	24585	0.25885	9.6231	2.8693	0.017762	0.069463	-3.4639
Cp	ceruloplasmin	24268	0.26143	5.8176	2.6069	0.02751	0.091114	-3.8929
Cap2	cyclase associated actin cytoskeleton reg	116653	0.26227	6.218	2.3972	0.039037	0.11542	-4.2318
Cspg4	chondroitin sulfate proteoglycan 4	81651	0.26403	10.825	2.2862	0.046959	0.12986	-4.4087
Tbrg4	transforming growth factor beta regulator 4	360977	0.26448	7.565	2.7882	0.020329	0.075693	-3.5968
Vapb	VAMP associated protein B and C	60431	0.26474	7.5965	2.4461	0.035979	0.11	-4.1532
Prkcsh	protein kinase C substrate 80K-H	300445	0.26933	11.678	2.3431	0.042721	0.12229	-4.3183
Rcn2	reticulocalbin 2	29218	0.2702	5.7478	2.5307	0.031243	0.098697	-4.0167
Grpel1		79563	0.27133	4.6531	2.6383	0.026104	0.088164	-3.8418
Jup	junction plakoglobin	81679	0.27303	5.8988	2.9805	0.014768	0.061861	-3.2814
Etfb	electron transfer flavoprotein subunit beta	292845	0.27428	7.6114	2.9486	0.01557	0.064119	-3.3337
Rpl19	ribosomal protein L19	81767	0.27457	5.5001	2.7349	0.02222	0.07961	-3.6842
Atp5md	ATP synthase membrane subunit DAPIT	171069	0.27659	12.28	2.5974	0.027951	0.091685	-3.9084
Idh3a	isocitrate dehydrogenase (NAD(+)) 3 catal	114096	0.27722	5.7616	2.7958	0.020075	0.075403	-3.5845
Mrpl37	mitochondrial ribosomal protein L37	56281	0.27795	8.2999	2.5911	0.028248	0.092304	-3.9187
Rab18	"RAB18, member RAS oncogene family"	307039	0.27808	7.7949	2.7168	0.022899	0.081359	-3.7137
Ccdc127	coiled-coil domain containing 127	308060	0.27881	7.6214	2.8754	0.017583	0.068922	-3.4539
Epb41l2	erythrocyte membrane protein band 4.1-lik	309557	0.27966	5.9424	2.7761	0.020744	0.07658	-3.6167
Dld	dihydrolipoamide dehydrogenase	298942	0.28047	5.6362	3.0538	0.013082	0.058219	-3.1611
Aldh5a1	"aldehyde dehydrogenase 5 family, memb	291133	0.28062	6.7909	2.6128	0.027242	0.0908	-3.8834
Gja1	"gap junction protein, alpha 1"	24392	0.28075	7.0592	3.025	0.013719	0.059708	-3.2083
Hadhb	hydroxyacyl-CoA dehydrogenase trifunctio	171155	0.28354	9.9219	3.0626	0.012893	0.057834	-3.1466
Aldh9a1	"aldehyde dehydrogenase 9 family, memb	64040	0.28417	5.7063	2.5391	0.030807	0.097789	-4.003
Cisd1	CDGSH iron sulfur domain 1	294362	0.28437	10.947	2.8655	0.017876	0.069545	-3.4702
Itgav	integrin subunit alpha V	296456	0.28464	8.5636	2.3973	0.03903	0.11542	-4.2316
Atp5mf	ATP synthase membrane subunit f	690441	0.28574	7.016	2.9352	0.015921	0.065092	-3.3557
Mrps30	mitochondrial ribosomal protein S30	294767	0.28644	6.3118	3.0215	0.0138	0.059708	-3.2141
Ndufa7	NADH:ubiquinone oxidoreductase subunit	299643	0.28719	9.0623	2.7041	0.02339	0.082762	-3.7345
Flot1	flotillin 1	64665	0.288	8.356	3.0145	0.01396	0.060096	-3.2256
Rpl4	ribosomal protein L4	64302	0.29004	4.0624	3.116	0.011807	0.054831	-3.0591

Ndufb11	NADH:ubiquinone oxidoreductase subunit	299310	0.29045	9.1967	3.1227	0.011676	0.054373	-3.048
Mrpl19	mitochondrial ribosomal protein L19	297372	0.29221	7.8372	2.9291	0.016083	0.065289	-3.3658
Oat	ornithine aminotransferase	64313	0.29538	4.7462	2.7452	0.021841	0.079082	-3.6673
Acat1	acetyl-CoA acetyltransferase 1	25014	0.29596	4.4495	3.131	0.011518	0.054018	-3.0344
Slc25a11	solute carrier family 25 member 11	64201	0.29766	9.5366	2.988	0.014587	0.061635	-3.2691
Ndufs8	NADH:ubiquinone oxidoreductase core su	293652	0.29864	6.1855	2.917	0.016408	0.065823	-3.3855
Enpep	glutamyl aminopeptidase	64017	0.29867	8.6595	2.6845	0.024167	0.083622	-3.7664
Slc25a12	solute carrier family 25 member 12	362145	0.29943	8.4097	3.4246	0.0071311	0.040118	-2.5551
Mrpl15	mitochondrial ribosomal protein L15	297799	0.30114	4.8058	2.285	0.047047	0.12986	-4.4105
Niban1	niban apoptosis regulator 1	63912	0.30175	5.9576	2.5313	0.031209	0.098697	-4.0156
Pacsin3	protein kinase C and casein kinase substr	311187	0.30586	6.2101	3.1621	0.010943	0.051948	-2.9834
Cbr1	carbonyl reductase 1	29224	0.30644	6.9589	2.4324	0.036814	0.11101	-4.1753
Ncam1	neural cell adhesion molecule 1	24586	0.30875	8.1235	3.0504	0.013155	0.058244	-3.1666
Uqcc1	ubiquinol-cytochrome c reductase comple:	683512	0.31034	6.3857	2.6218	0.026834	0.090097	-3.8687
Slc12a7	solute carrier family 12 member 7	308069	0.3106	11.23	2.9152	0.016459	0.065876	-3.3886
Abcd3	ATP binding cassette subfamily D membe	25270	0.31072	6.0489	2.6006	0.027802	0.09155	-3.9032
Cd163	CD163 molecule	312701	0.31237	4.144	2.9307	0.016039	0.065275	-3.3631
Ckb	creatine kinase B	24264	0.31476	6.9537	2.6112	0.027313	0.090812	-3.8859
Uggt1	UDP-glucose glycoprotein glucosyltransfe	171129	0.31504	10.153	2.4019	0.038735	0.11533	-4.2243
Mtfp1	mitochondrial fission process 1	289745	0.3158	6.2608	2.9917	0.014497	0.061479	-3.263
Ccdc90b	coiled-coil domain containing 90B	308820	0.31615	10.416	2.631	0.026427	0.089079	-3.8538
Vdac3	voltage-dependent anion channel 3	83532	0.31645	8.6824	3.3989	0.0074344	0.040544	-2.5969
Ryr2	ryanodine receptor 2	689560	0.31784	6.8738	3.144	0.011274	0.053374	-3.0131
Mtif2	mitochondrial translational initiation factor	305606	0.31864	11.276	2.5384	0.030842	0.097789	-4.0041
Atp5me	ATP synthase membrane subunit e	140608	0.32052	7.1681	3.0704	0.012729	0.057246	-3.1339
Pyroxd2	pyridine nucleotide-disulphide oxidoreduct	309381	0.32109	5.0433	2.9832	0.014702	0.061734	-3.2769
Ppp1cc	protein phosphatase 1 catalytic subunit ga	24669	0.32231	10.486	2.5898	0.028304	0.092313	-3.9207
Acta1	"actin, alpha 1, skeletal muscle"	29437	0.32254	7.2992	2.791	0.020235	0.075506	-3.5923
Gstp1	glutathione S-transferase pi 1	24426	0.32367	6.1991	2.6956	0.023725	0.083069	-3.7484
Mpst	mercaptopyruvate sulfurtransferase	192172	0.32379	8.6804	2.9271	0.016137	0.065302	-3.3691
Cox7a2	cytochrome c oxidase subunit 7A2	29507	0.32395	6.0636	2.7588	0.021352	0.077639	-3.6451
Decr1	"2,4-dienoyl-CoA reductase 1"	117543	0.32531	8.1321	3.2606	0.0093124	0.046484	-2.8224
Cfh	complement factor H	155012	0.32627	7.4073	2.7739	0.020821	0.07658	-3.6203
Pmpcb	"peptidase, mitochondrial processing beta	64198	0.32676	6.7589	2.7175	0.022873	0.081359	-3.7126
Cox5b	cytochrome c oxidase subunit 5B	94194	0.3273	11.075	3.13	0.011537	0.054018	-3.036
Cd81	Cd81 molecule	25621	0.33051	6.8811	3.1035	0.012052	0.055367	-3.0795
Mvp	major vault protein	64681	0.33246	9.319	2.698	0.02363	0.083069	-3.7444
Hspd1	heat shock protein family D (Hsp60) memb	63868	0.33649	5.3823	3.608	0.005309	0.034554	-2.2586
Rpl23	ribosomal protein L23	29282	0.33664	6.179	3.2866	0.0089246	0.045529	-2.7798
Hnrnpu	heterogeneous nuclear ribonucleoprotein	117280	0.33695	8.3825	2.4388	0.036422	0.11072	-4.165

P4hb	prolyl 4-hydroxylase subunit beta	25506	0.33796	5.467	3.4201	0.0071832	0.040118	-2.5624
Rps3	ribosomal protein S3	140654	0.33817	4.0727	2.735	0.022213	0.07961	-3.6838
Aldh4a1	"aldehyde dehydrogenase 4 family, memb	641316	0.33832	8.3417	3.4254	0.0071209	0.040118	-2.5537
Ak2	adenylate kinase 2	24184	0.33837	3.8001	2.9186	0.016366	0.065812	-3.383
Pi4k2a	phosphatidylinositol 4-kinase type 2 alpha	114554	0.34121	6.6647	2.7362	0.022171	0.07961	-3.682
Acot7	acyl-CoA thioesterase 7	26759	0.34164	4.4505	2.3609	0.041473	0.12018	-4.2899
Rpl10	ribosomal protein L10	81764	0.34242	6.9844	2.9385	0.015834	0.064891	-3.3503
Pgam1	phosphoglycerate mutase 1	24642	0.34305	7.5054	2.6637	0.025022	0.085761	-3.8004
Dbt	dihydrolipoamide branched chain transacy	29611	0.34394	7.7224	3.4682	0.0066449	0.039159	-2.4842
Vwa8	von Willebrand factor A domain containing	290381	0.34721	6.5306	3.1332	0.011477	0.054018	-3.0309
Bcam	basal cell adhesion molecule (Lutheran bl	78958	0.34746	7.7244	3.3984	0.0074401	0.040544	-2.5977
Ehd4	EH-domain containing 4	192204	0.34758	7.15	3.5724	0.0056201	0.035357	-2.3159
Hebp1	heme binding protein 1	362454	0.34775	6.6782	2.3192	0.04445	0.12515	-4.3563
Acsf3	acyl-CoA synthetase family member 3	498962	0.34795	7.3002	3.0861	0.012404	0.056527	-3.1081
Mlycd	malonyl-CoA decarboxylase	85239	0.34855	8.6717	3.4864	0.0064532	0.038387	-2.4548
Anxa6	annexin A6	79125	0.34875	7.1204	3.691	0.004651	0.032488	-2.1253
Flnc	filamin C	362332	0.34944	5.1075	2.9548	0.015412	0.063744	-3.3236
Rexo2	RNA exonuclease 2	300689	0.35026	9.3474	2.35	0.042231	0.1213	-4.3073
Ndufa2	NADH:ubiquinone oxidoreductase subunit	291660	0.35077	6.32	3.535	0.0059673	0.036928	-2.3762
Gk	glycerol kinase	79223	0.35085	6.6541	2.9872	0.014606	0.061635	-3.2704
Hpx	hemopexin	58917	0.35088	8.5046	3.5928	0.0054396	0.034818	-2.283
Opa1	"OPA1, mitochondrial dynamin like GTPas	171116	0.3516	11.516	4.0573	0.0026212	0.024084	-1.5461
Sgcg	"sarcoglycan, gamma"	305941	0.35204	10.483	3.3668	0.0078319	0.041688	-2.6491
Clpx	caseinolytic mitochondrial matrix peptidase	300786	0.35286	6.2377	2.5681	0.029352	0.094645	-3.956
Dpp4	dipeptidylpeptidase 4	25253	0.35363	10.223	2.8081	0.019667	0.074032	-3.5642
Cox6b1	cytochrome c oxidase subunit 6B1	688869	0.35531	3.8031	3.9173	0.0032567	0.027274	-1.7656
Ndufv2	NADH:ubiquinone oxidoreductase core su	81728	0.35554	8.6071	3.8238	0.0037705	0.029695	-1.9136
Tomm70	translocase of outer mitochondrial membra	304017	0.35729	8.8532	3.4443	0.0069072	0.039612	-2.5231
Gnpat	glyceronephosphate O-acyltransferase	84470	0.35739	4.076	2.6945	0.023769	0.083069	-3.7502
Lpl	lipoprotein lipase	24539	0.36002	9.4066	2.9043	0.016759	0.066609	-3.4065
Pars2	"prolyl-tRNA synthetase 2, mitochondrial"	313429	0.36128	10.491	3.1095	0.011934	0.055108	-3.0697
Slc27a1	solute carrier family 27 member 1	94172	0.36222	9.8643	3.0706	0.012724	0.057246	-3.1335
Taco1	translational activator of cytochrome c oxid	360645	0.36418	5.8013	2.3618	0.04141	0.12018	-4.2885
Slc9a3r2	SLC9A3 regulator 2	116501	0.36519	5.4158	3.3918	0.0075205	0.040544	-2.6084
Copb1	COPI coat complex subunit beta 1	114023	0.36598	5.6257	3.6025	0.0053552	0.034667	-2.2673
Rmdn1	regulator of microtubule dynamics 1	500419	0.36656	9.1472	3.6721	0.0047926	0.032985	-2.1555
Actr3	actin related protein 3	81732	0.36886	8.7337	3.282	0.0089915	0.045733	-2.7873
Timm21	translocase of inner mitochondrial membra	307210	0.36926	5.5471	2.567	0.029407	0.094645	-3.9578
Apoa1	apolipoprotein A1	25081	0.36933	7.2028	2.9548	0.015412	0.063744	-3.3236
Ndufb6	NADH:ubiquinone oxidoreductase subunit	297990	0.36983	7.4197	3.7283	0.0043833	0.031634	-2.0655

Zadh2	"zinc binding alcohol dehydrogenase, dor	291403	0.37095	6.9427	3.3138	0.0085371	0.044078	-2.7354
Tubb2a	"tubulin, beta 2A class IIa"	498736	0.3713	6.8358	2.7651	0.021128	0.077317	-3.6347
Mrps36	mitochondrial ribosomal protein S36	294696	0.37158	9.1116	3.2726	0.0091303	0.046092	-2.8026
Nsf	"N-ethylmaleimide sensitive factor, vesicle	60355	0.37199	7.6817	2.6843	0.024175	0.083622	-3.7668
Mul1	mitochondrial E3 ubiquitin protein ligase 1	298576	0.37246	9.0004	3.6181	0.0052235	0.034467	-2.2422
Lamtor1	"late endosomal/lysosomal adaptor, MAPK	308869	0.37283	6.9787	2.386	0.039773	0.11719	-4.2497
Ldhd	lactate dehydrogenase D	307858	0.37318	7.1241	2.7731	0.020846	0.07658	-3.6215
Ehd2	EH-domain containing 2	361512	0.37345	6.8347	3.8906	0.0033956	0.027996	-1.8078
Dag1	dystroglycan 1	114489	0.37565	9.7493	3.7239	0.004414	0.031634	-2.0726
Prdx2	peroxiredoxin 2	29338	0.37739	6.6202	3.1953	0.010363	0.050171	-2.9291
Clpb	caseinolytic mitochondrial matrix peptidase	65041	0.37746	8.1007	2.5418	0.030668	0.097601	-3.9986
Ano6	anoctamin 6	315272	0.37887	4.2498	2.5981	0.027919	0.091685	-3.9073
Fn1	fibronectin 1	25661	0.38233	5.3318	3.4721	0.0066042	0.039054	-2.4781
Atp5mg	ATP synthase membrane subunit g	300677	0.38339	13.348	3.7673	0.0041214	0.030624	-2.0034
Ppib	peptidylprolyl isomerase B	64367	0.38549	8.7327	3.3953	0.0074776	0.040544	-2.6027
Arhgdia	Rho GDP dissociation inhibitor alpha	360678	0.38605	7.4913	3.3469	0.0080884	0.042533	-2.6814
Cacna2d1	calcium voltage-gated channel auxiliary su	25399	0.38651	10.069	3.5886	0.0054761	0.0349	-2.2898
Eci2	enoyl-CoA delta isomerase 2	291075	0.38686	10.654	3.7923	0.0039618	0.030362	-1.9635
Asph	aspartate-beta-hydroxylase	312981	0.38692	8.1796	3.5271	0.0060435	0.037152	-2.3889
Hsp90aa1	heat shock protein 90 alpha family class A	299331	0.38728	12.304	3.3631	0.007879	0.041688	-2.6551
Gnb3	G protein subunit beta 3	60449	0.38745	5.0902	3.4922	0.0063928	0.038334	-2.4454
Ndufs2	NADH:ubiquinone oxidoreductase core su	289218	0.38779	8.0085	4.2415	0.0019781	0.021055	-1.2611
Echs1	"enoyl-CoA hydratase, short chain 1"	140547	0.38784	8.7975	3.6324	0.0051057	0.033952	-2.2193
Actc1	"actin, alpha, cardiac muscle 1"	29275	0.3879	8.8478	4.166	0.0022188	0.022591	-1.3774
Clic5	chloride intracellular channel 5	94272	0.38841	7.045	3.2518	0.0094471	0.046733	-2.8367
Mrps27	mitochondrial ribosomal protein S27	361883	0.38863	6.8962	2.5284	0.031363	0.098893	-4.0204
Cpt1b	carnitine palmitoyltransferase 1B	25756	0.38928	7.6301	4.0388	0.0026969	0.024647	-1.5749
Ndufv3	NADH:ubiquinone oxidoreductase subunit	64539	0.38989	7.2401	3.6436	0.0050154	0.033613	-2.2013
Mrpl41	mitochondrial ribosomal protein L41	296551	0.38994	10.293	3.4205	0.0071781	0.040118	-2.5617
Glud1	glutamate dehydrogenase 1	24399	0.39189	4.4439	4.4842	0.0013748	0.017936	-0.89255
Rps2	ribosomal protein S2	83789	0.39672	7.2894	3.3711	0.0077775	0.041537	-2.6421
Gpx1	glutathione peroxidase 1	24404	0.39672	6.3323	3.18	0.010627	0.050873	-2.9542
Tmed10	transmembrane p24 trafficking protein 10	84599	0.39675	8.406	3.2491	0.0094889	0.046733	-2.8411
Rpl17	ribosomal protein L17	291434	0.39851	5.5258	2.4152	0.037884	0.11339	-4.2029
Pgk1	phosphoglycerate kinase 1	24644	0.39961	7.7931	4.1524	0.0022654	0.02276	-1.3985
Chchd3	coiled-coil-helix-coiled-coil-helix domain co	296966	0.39969	9.3706	4.2559	0.0019353	0.020801	-1.239
Gng12	G protein subunit gamma 12	114120	0.40004	11.116	3.1659	0.010875	0.051772	-2.9772
Cyb5a	cytochrome b5 type A	64001	0.40099	7.7879	3.856	0.0035845	0.028758	-1.8625
Atp5po	ATP synthase peripheral stalk subunit OS	192241	0.40148	7.4975	3.6901	0.0046574	0.032488	-2.1267
Ecsit	ECSIT signaling integrator	300447	0.4022	9.3293	3.5754	0.0055927	0.035357	-2.311

Oxsm	"3-oxoacyl-ACP synthase, mitochondrial"	289934	0.40234	4.9054	3.3414	0.0081613	0.042653	-2.6904
Ndufa10	NADH:ubiquinone oxidoreductase subunit	678759	0.40297	6.6468	4.128	0.0023516	0.023097	-1.4363
Snd1	staphylococcal nuclease and tudor domai	64635	0.40416	10.846	3.5171	0.0061418	0.037487	-2.4051
Ndufs3	NADH:ubiquinone oxidoreductase core su	295923	0.40418	9.1377	4.3276	0.001737	0.019503	-1.1295
Popdc2	popeye domain containing 2	360718	0.405	8.6571	3.9217	0.0032346	0.027231	-1.7587
Myl6	myosin light chain 6	685867	0.40763	10.279	3.2228	0.0099063	0.04851	-2.8841
Pdhb	pyruvate dehydrogenase E1 beta subunit	289950	0.41086	8.2855	4.4607	0.0014238	0.018158	-0.92797
Lamtor5	"late endosomal/lysosomal adaptor, MAPK	295357	0.41379	11.394	3.2501	0.0094728	0.046733	-2.8394
Cpt2	carnitine palmitoyltransferase 2	25413	0.41397	7.5332	4.6487	0.0010797	0.016045	-0.64764
Fermt2	fermitin family member 2	289992	0.41835	8.2925	2.9124	0.016534	0.06602	-3.3931
Mrps25	mitochondrial ribosomal protein S25	297459	0.41966	3.9228	2.6997	0.023562	0.083069	-3.7416
Lman1	"lectin, mannose-binding, 1"	116666	0.42003	6.1711	3.3924	0.0075123	0.040544	-2.6073
Sardh	sarcosine dehydrogenase	114123	0.42324	6.0927	2.76	0.021308	0.077639	-3.643
Sirt3	sirtuin 3	293615	0.42449	8.7682	3.2886	0.0088961	0.045519	-2.7766
Uqcrh	ubiquinol-cytochrome c reductase hinge p	366448	0.42588	6.3867	4.4656	0.0014133	0.018158	-0.92054
Bche	butyrylcholinesterase	65036	0.42671	7.9236	2.9053	0.016731	0.066609	-3.4048
Hint2	histidine triad nucleotide binding protein 2	313491	0.42805	12.226	3.9504	0.0030932	0.026723	-1.7136
Acaca	acetyl-CoA carboxylase alpha	60581	0.42885	5.0669	2.9984	0.014336	0.061246	-3.2519
Ckmt2	"creatine kinase, mitochondrial 2"	688698	0.42997	10.036	3.7608	0.0041637	0.030804	-2.0137
Coq6	coenzyme Q6 monooxygenase	299195	0.43148	6.5295	2.8658	0.017865	0.069545	-3.4695
Rhoa	ras homolog family member A	117273	0.43176	8.8376	3.7564	0.0041931	0.030888	-2.0208
Snta1	"syntrophin, alpha 1"	362242	0.43189	8.2397	3.27	0.0091699	0.046092	-2.8069
Col14a1	collagen type XIV alpha 1 chain	314981	0.43563	11.34	3.802	0.0039018	0.030271	-1.9481
Rack1	receptor for activated C kinase 1	83427	0.43664	7.384	4.0133	0.0028056	0.025104	-1.6149
Cars2	"cysteinyl-tRNA synthetase 2, mitochondri	361184	0.43779	12.626	3.7202	0.0044406	0.031634	-2.0786
Mdh1	malate dehydrogenase 1	24551	0.43847	8.1391	4.1451	0.0022907	0.02276	-1.4097
Ywhaq	"tyrosine 3-monooxygenase/tryptophan 5-	25577	0.43847	6.6116	3.7861	0.0040012	0.030527	-1.9735
Ckap4	cytoskeleton-associated protein 4	362859	0.43874	7.8735	3.853	0.0036011	0.028758	-1.8672
Tnnt2	"troponin T2, cardiac type"	24837	0.44146	4.7205	4.0258	0.0027519	0.024883	-1.5953
Ccdc51	coiled-coil domain containing 51	316008	0.4431	5.4713	3.5866	0.0054934	0.0349	-2.2929
Hk1	hexokinase 1	25058	0.44955	6.8096	4.4303	0.0014894	0.018341	-0.97365
Rpn1	ribophorin I	25596	0.45318	6.7322	3.7714	0.0040947	0.030559	-1.9968
Hk2	hexokinase 2	25059	0.45776	5.726	3.4034	0.0073798	0.040544	-2.5895
Cct2	chaperonin containing TCP1 subunit 2	299809	0.45825	9.8848	3.8203	0.0037911	0.02972	-1.9191
Sqor	sulfide quinone oxidoreductase	691966	0.45863	7.1163	3.4654	0.0066751	0.039202	-2.4888
Pbxip1	PBX homeobox interacting protein 1	310644	0.46167	5.3132	4.6899	0.0010169	0.015517	-0.58691
Acaa2	acetyl-CoA acyltransferase 2	170465	0.46284	9.7655	5.0783	0.00058499	0.011603	-0.02693
Septin2	septin 2	117515	0.46313	7.1382	3.7479	0.0042497	0.030966	-2.0343
Ywhag	"tyrosine 3-monooxygenase/tryptophan 5-	56010	0.46372	8.1696	3.0811	0.012505	0.056637	-3.1163
Ehd1	EH-domain containing 1	293692	0.46681	9.135	4.5725	0.0012071	0.017157	-0.76064

Ndufb10	NADH:ubiquinone oxidoreductase subunit	681418	0.47071	8.4538	4.8191	0.00084395	0.013869	-0.39803
Atp1a2	ATPase Na+/K+ transporting subunit alph	24212	0.47341	11.702	4.6611	0.0010603	0.015895	-0.62926
Coq9	coenzyme Q9	498909	0.47672	11.294	4.6134	0.0011368	0.016748	-0.69984
Surf1	"SURF1, cytochrome c oxidase assembly	64463	0.48062	12.789	3.961	0.0030427	0.026666	-1.6969
Ndufa12	NADH:ubiquinone oxidoreductase subunit	299739	0.4813	5.4683	4.1132	0.0024056	0.023359	-1.4592
Csrp1	cysteine and glycine-rich protein 1	29276	0.48164	8.0827	3.7801	0.0040388	0.030541	-1.983
Ndufa11	NADH:ubiquinone oxidoreductase subunit	301123	0.48229	8.9542	4.5303	0.0012843	0.01752	-0.82349
Tmem65	transmembrane protein 65	500874	0.48723	5.1994	2.9306	0.016042	0.065275	-3.3632
Abcb7	ATP binding cassette subfamily B membe	302395	0.48772	7.5431	3.9144	0.0032716	0.027274	-1.7702
Gnb2	G protein subunit beta 2	81667	0.48815	6.5236	5.1476	0.00053125	0.011072	0.070576
Cyb5r3	cytochrome b5 reductase 3	25035	0.49151	7.8489	4.1146	0.0024003	0.023359	-1.457
Lgals1	galectin 1	56646	0.49167	8.4075	4.1482	0.00228	0.02276	-1.405
Tomm40	translocase of outer mitochondrial membra	308416	0.49267	11.446	4.4158	0.001522	0.018341	-0.99558
Wdr1	WD repeat domain 1	360950	0.49344	11.877	4.5053	0.0013326	0.017654	-0.8609
Acads	acyl-CoA dehydrogenase short chain	64304	0.49439	8.0113	4.7723	0.00090268	0.01437	-0.46619
Ywhab	"tyrosine 3-monooxygenase/tryptophan 5-	56011	0.49517	5.2325	3.5331	0.0059854	0.036928	-2.3792
Por	cytochrome p450 oxidoreductase	29441	0.49592	6.903	3.9484	0.0031026	0.026723	-1.7166
Alb	albumin	24186	0.49782	8.4444	5.4036	0.00037436	0.010256	0.42431
Myo1c	myosin 1C	65261	0.50133	6.0787	4.7532	0.00092788	0.014548	-0.49408
Dbi	"diazepam binding inhibitor, acyl-CoA bind	25045	0.50146	5.6325	2.8321	0.018897	0.072088	-3.5249
Des	desmin	64362	0.50225	10.501	5.711	0.00024892	0.0083412	0.8358
Atp5f1a	ATP synthase F1 subunit alpha	65262	0.50256	6.241	5.3453	0.00040512	0.010256	0.34457
Pdia3	"protein disulfide isomerase family A, mem	29468	0.50486	12.085	5.0552	0.00060423	0.011603	-0.059666
Pfkm	"phosphofructokinase, muscle"	65152	0.50571	6.5968	5.2917	0.00043574	0.010353	0.27096
Mgst3	microsomal glutathione S-transferase 3	289197	0.50844	6.6791	4.1975	0.0021149	0.022038	-1.3288
Tmem120a	transmembrane protein 120A	288591	0.51036	7.4479	3.6836	0.0047059	0.032692	-2.1371
Nt5e	"5' nucleotidase, ecto"	58813	0.51041	5.2201	4.5211	0.0013019	0.01752	-0.83732
Myh6	myosin heavy chain 6	29556	0.51059	8.9681	5.0802	0.00058348	0.011603	-0.024301
Rab7a	"RAB7A, member RAS oncogene family"	29448	0.51258	10.854	3.7488	0.004244	0.030966	-2.033
Lias	lipoic acid synthetase	305348	0.51315	5.8084	2.8493	0.018362	0.070837	-3.4966
Vcp	valosin-containing protein	116643	0.51479	7.0451	5.291	0.00043618	0.010353	0.26993
Got1	glutamic-oxaloacetic transaminase 1	24401	0.51528	5.5039	4.0701	0.0025699	0.024084	-1.5261
Ndufb9	NADH:ubiquinone oxidoreductase subunit	299954	0.51719	12.731	4.3207	0.0017549	0.019503	-1.1399
Cct5	chaperonin containing TCP1 subunit 5	294864	0.51722	8.4469	4.7874	0.00088326	0.014241	-0.44415
Cdh2	cadherin 2	83501	0.52027	7.5008	4.942	0.00070847	0.012881	-0.22079
Gapdh	glyceraldehyde-3-phosphate dehydrogena	24383	0.52075	9.042	5.5969	0.00028919	0.0094423	0.68472
Dnaja3	DnaJ heat shock protein family (Hsp40) m	360481	0.52243	6.3174	4.1993	0.0021089	0.022038	-1.326
Cycs	"cytochrome c, somatic"	25309	0.52349	5.0962	5.3433	0.00040622	0.010256	0.34184
Trap1	TNF receptor-associated protein 1	287069	0.52567	10.29	3.6518	0.0049503	0.033307	-2.1881
Aldoa	"aldolase, fructose-bisphosphate A"	24189	0.53185	7.9128	5.503	0.00032761	0.009998	0.55894

Hibadh	3-hydroxyisobutyrate dehydrogenase	63938	0.53354	4.9734	4.8325	0.00082801	0.013869	-0.37871
Tnni3	"troponin I3, cardiac type"	29248	0.53379	7.1138	3.9269	0.0032082	0.027142	-1.7504
Lnpep	leucyl and cystinyl aminopeptidase	171105	0.53889	10.202	4.8294	0.00083163	0.013869	-0.38313
Anxa2	annexin A2	56611	0.54015	5.764	4.4374	0.0014738	0.018341	-0.963
Eno1	enolase 1	24333	0.54389	7.7167	4.9079	0.00074364	0.013378	-0.26985
Uqcrfs1	"ubiquinol-cytochrome c reductase, Rieske	291103	0.54504	7.453	5.767	0.00023141	0.0083194	0.9092
Lama4	laminin subunit alpha 4	309816	0.54716	5.7536	4.953	0.00069755	0.012818	-0.20507
Tpi1	triosephosphate isomerase 1	24849	0.55543	8.277	5.3246	0.00041664	0.010256	0.31624
Cavin1	caveolae associated protein 1	287710	0.55596	7.6422	5.8116	0.00021841	0.0082162	0.96738
Iars2	"isoleucyl-tRNA synthetase 2, mitochondri	364070	0.56257	4.434	4.3774	0.001612	0.018848	-1.0538
Hhatl	hedgehog acyltransferase-like	301073	0.57186	8.5749	4.8685	0.00078651	0.013857	-0.32662
Lonp1	"lon peptidase 1, mitochondrial"	170916	0.5741	8.0092	5.0598	0.00060031	0.011603	-0.053089
Mccc1	methylcrotonoyl-CoA carboxylase 1	294972	0.57765	4.8653	5.5874	0.00029283	0.0094423	0.67213
Bckdha	branched chain keto acid dehydrogenase	25244	0.57772	5.8422	5.1818	0.00050674	0.010941	0.11835
Capza2	capping actin protein of muscle Z-line sub	493810	0.57954	8.956	5.4359	0.00035841	0.010145	0.46826
Mecr	mitochondrial trans-2-enoyl-CoA reductase	29470	0.58779	9.9391	5.2151	0.00048398	0.010742	0.16481
Ywhae	"tyrosine 3-monooxygenase/tryptophan 5-	29753	0.58838	6.0533	4.4182	0.0015165	0.018341	-0.99191
Gnao1	G protein subunit alpha o1	50664	0.5907	4.7641	3.9473	0.0031078	0.026723	-1.7183
Myh7	myosin heavy chain 7	29557	0.59179	8.5415	5.9984	0.00017197	0.0076421	1.2075
Cap1	cyclase associated actin cytoskeleton regu	64185	0.60085	9.6108	3.6608	0.00488	0.033103	-2.1737
Acox1	acyl-CoA oxidase 1	50681	0.60132	4.3661	4.3794	0.001607	0.018848	-1.0507
Pitrm1	pitrilysin metallopeptidase 1	307081	0.60274	5.2863	4.5247	0.0012949	0.01752	-0.83186
Agl	"amylo-alpha-1, 6-glucosidase, 4-alpha-gl	362029	0.61068	8.3887	4.4274	0.0014959	0.018341	-0.97804
Syncrip	"synaptotagmin binding, cytoplasmic RNA	363113	0.61443	8.664	4.0623	0.0026011	0.024084	-1.5383
Ehbp1l1	EH domain binding protein 1-like 1	309169	0.62395	10.662	2.6898	0.023956	0.083384	-3.7579
Ptges2	prostaglandin E synthase 2	311865	0.62615	10.507	5.2316	0.00047314	0.010639	0.18773
Casq2	calsequestrin 2	29209	0.62634	6.6819	3.9076	0.0033065	0.027431	-1.781
Me3	malic enzyme 3	361602	0.63113	5.9379	5.0137	0.00064042	0.012094	-0.11855
Ctnnb1	catenin beta 1	84353	0.63644	5.7047	3.9466	0.0031117	0.026723	-1.7196
Slc25a4	solute carrier family 25 member 4	85333	0.64583	4.5032	6.0096	0.00016954	0.0076421	1.2218
Atp5pf	ATP synthase peripheral stalk subunit F6	94271	0.6459	11.768	6.0139	0.00016862	0.0076421	1.2273
Cavin4	caveolae associated protein 4	313225	0.64606	10.766	4.9568	0.00069387	0.012818	-0.1997
Plcd1	"phospholipase C, delta 1"	24655	0.66021	5.6	2.9946	0.014428	0.061339	-3.2583
Kyat3	kynurenine aminotransferase 3	541589	0.69853	6.7025	4.8417	0.00081717	0.013869	-0.36537
Mtch2	mitochondrial carrier 2	295922	0.69987	5.9151	6.0177	0.00016781	0.0076421	1.2321
Theg	theg spermatid protein	299599	0.70347	7.7084	5.7208	0.00024576	0.0083412	0.84866
Epb41	erythrocyte membrane protein band 4.1	313052	0.70576	8.1347	5.9451	0.00018402	0.0076707	1.1395
Inpp5a	inositol polyphosphate-5-phosphatase A	365382	0.71972	5.2782	2.3213	0.044299	0.12515	-4.353
Ndufb8	NADH:ubiquinone oxidoreductase subunit	293991	0.72773	9.4542	5.0955	0.00057112	0.011603	-0.0026385
Cacna2d2	calcium voltage-gated channel auxiliary su	300992	0.73231	6.9439	2.652	0.025516	0.086867	-3.8195

Ndufa6	NADH:ubiquinone oxidoreductase subunit	315167	0.74599	7.3553	6.1657	0.00013936	0.0072171	1.4183
Aldh6a1	"aldehyde dehydrogenase 6 family, memb	81708	0.74638	5.2395	6.0769	0.00015575	0.0076421	1.3069
Rpl9	ribosomal protein L9	29257	0.74673	8.0145	6.7571	6.8212E-05	0.0054266	2.1307
Speg	striated muscle enriched protein kinase	363256	0.7502	6.4069	3.4623	0.0067085	0.039263	-2.4938
Hspa5	heat shock protein family A (Hsp70) memb	25617	0.76265	9.3173	8.2388	1.3593E-05	0.0028798	3.7103
Fahd1	fumarylacetoacetate hydrolase domain co	302980	0.77391	8.4034	5.3186	0.00042009	0.010256	0.30791
Myh14	myosin heavy chain 14	308572	0.78618	8.1365	7.0025	5.1354E-05	0.0048758	2.4118
Sfxn3	sideroflexin 3	65042	0.80329	6.8454	3.089	0.012344	0.056408	-3.1034
Cd38	CD38 molecule	25668	0.84962	8.256	4.3198	0.0017575	0.019503	-1.1413
Itga6	integrin subunit alpha 6	114517	0.85418	5.5323	5.8492	0.00020807	0.0082162	1.0161
Hspa12b	heat shock protein family A (Hsp70) memb	311427	0.87909	8.4419	6.2864	0.00012002	0.0067675	1.5677
Mrpl47	mitochondrial ribosomal protein L47	294963	0.89452	7.7911	2.9844	0.014672	0.061734	-3.2749
Atp6v0a1	ATPase H+ transporting V0 subunit a1	29757	0.90382	5.8095	5.2406	0.00046733	0.010639	0.20022
Tagln2	transgelin 2	304983	0.92119	6.6076	4.7945	0.00087427	0.01423	-0.43378
Macrod1	mono-ADP ribosylhydrolase 1	246233	0.97183	12.102	4.0922	0.0024844	0.023853	-1.4919
Uqcrc2	ubiquinol cytochrome c reductase core pro	293448	1.0321	8.2866	6.5886	8.3241E-05	0.0054266	1.9328
Mospd1	motile sperm domain containing 1	317312	1.061	7.2355	4.6689	0.0010483	0.015855	-0.61776
Sptan1	"spectrin, alpha, non-erythrocytic 1"	64159	1.0616	9.9457	9.474	4.1713E-06	0.0017822	4.8329
Dnajc3	DnaJ heat shock protein family (Hsp40) m	63880	1.074	5.3413	3.4899	0.0064169	0.038345	-2.4492
Pecam1	platelet and endothelial cell adhesion mol	29583	1.0836	6.2168	2.8581	0.018095	0.070124	-3.4822
Abca8a	"ATP-binding cassette, subfamily A (ABC1	303638	1.2864	7.5866	6.3394	0.00011246	0.0066275	1.6327
Trim72	tripartite motif containing 72	365377	1.2986	5.8154	7.4757	3.0288E-05	0.0034508	2.9314
Hibch	3-hydroxyisobutyryl-CoA hydrolase	301384	1.3203	6.8133	5.4283	0.00036212	0.010145	0.45787
Septin11	septin 11	305227	1.3474	7.4765	6.5516	8.7003E-05	0.0054266	1.8887
Rpl6	ribosomal protein L6	117042	1.4132	6.7853	7.4132	3.2428E-05	0.0034637	2.8645
Mccc2	methylcrotonoyl-CoA carboxylase 2	361884	1.4217	8.1469	8.7893	7.8977E-06	0.0022495	4.2304
Psmd11	"proteasome 26S subunit, non-ATPase 1	303353	1.4414	8.462	3.2594	0.0093294	0.046484	-2.8242
Pc	pyruvate carboxylase	25104	1.5088	7.1073	8.7936	7.8648E-06	0.0022495	4.2344
Septin8	septin 8	303135	1.6232	6.8755	6.5335	8.8909E-05	0.0054266	1.8672
Abcb8	ATP binding cassette subfamily B membe	362302	1.6685	4.0187	6.7336	7.0116E-05	0.0054266	2.1033
Bcat2	branched chain amino acid transaminase	64203	1.9272	10.787	9.7886	3.1506E-06	0.0017822	5.0943
Gnas	GNAS complex locus	24896	1.9725	7.5926	9.6007	3.722E-06	0.0017822	4.9393
Clybl	citrate lyase beta like	306198	-0.21201	7.5768	-2.2472	0.050096	0.13552	-4.4703
Tns1	tensin 1	301509	-0.29013	6.1976	-2.2462	0.050178	0.13552	-4.4719
Cox7a2l	cytochrome c oxidase subunit 7A2 like	298762	0.23827	8.1416	2.246	0.050196	0.13552	-4.4722
Rdh13	retinol dehydrogenase 13	361504	0.21491	7.1043	2.2417	0.050558	0.13607	-4.479
Nedd4	NEDD4 E3 ubiquitin protein ligase	25489	-0.25447	4.9991	-2.2409	0.050624	0.13607	-4.4803
Rpl10a	ribosomal protein L10A	81729	-0.22926	5.5501	-2.2407	0.05064	0.13607	-4.4806
Capza1	capping actin protein of muscle Z-line sub	691149	0.24578	5.958	2.2341	0.0512	0.13706	-4.491
Dnajc10	DnaJ heat shock protein family (Hsp40) m	295690	0.25712	8.7256	2.2326	0.051324	0.13706	-4.4933

Eif4a1	eukaryotic translation initiation factor 4A1	287436	0.35135	4.5539	2.2319	0.051385	0.13706	-4.4945
Naca	nascent polypeptide associated complex s	288770	-0.28722	8.5251	-2.2317	0.051404	0.13706	-4.4948
Psmd2	"proteasome 26S subunit, non-ATPase 2"	287984	0.43691	4.305	2.2316	0.051407	0.13706	-4.4949
Mrc1	"mannose receptor, C type 1"	291327	-0.25131	5.384	-2.2295	0.051587	0.13732	-4.4982
Slc16a1	solute carrier family 16 member 1	25027	0.22911	5.0283	2.2279	0.051725	0.13744	-4.5007
Acss1	acyl-CoA synthetase short-chain family me	296259	0.2052	8.4468	2.2271	0.051793	0.13744	-4.502
Nucb1	nucleobindin 1	84595	0.24314	12.369	2.2197	0.052437	0.13894	-4.5137
Tmed9	transmembrane p24 trafficking protein 9	361207	0.25118	4.6393	2.2107	0.053223	0.1408	-4.5278
Mrps9	mitochondrial ribosomal protein S9	301371	0.21691	6.0016	2.2011	0.054077	0.14254	-4.5429
Tomm6	translocase of outer mitochondrial membra	681123	0.24798	6.9564	2.2006	0.05412	0.14254	-4.5436
B2m	beta-2 microglobulin	24223	0.19839	5.7	2.2002	0.054156	0.14254	-4.5442
Plxnb2	plexin B2	315217	-0.2274	5.1743	-2.1996	0.054212	0.14254	-4.5452
Maob	monoamine oxidase B	25750	0.39205	6.2247	2.1937	0.054736	0.14369	-4.5543
Tfam	"transcription factor A, mitochondrial"	83474	-0.24256	11.006	-2.1923	0.054866	0.14381	-4.5566
Rdh14	retinol dehydrogenase 14	500629	-0.20736	6.0174	-2.1838	0.055647	0.14564	-4.5699
Lrpap1	LDL receptor related protein associated p	116565	0.40767	8.5268	2.181	0.055899	0.14607	-4.5742
Adssl1	adenylosuccinate synthase like 1	684425	0.2111	9.2405	2.1796	0.056028	0.14619	-4.5764
Rab5c	"RAB5C, member RAS oncogene family"	287709	0.22312	6.7774	2.1716	0.05678	0.14778	-4.589
Cacnb2	calcium voltage-gated channel auxiliary su	116600	-0.24336	6.047	-2.1713	0.05681	0.14778	-4.5895
Ap2a1	adaptor related protein complex 2 subunit	308578	-0.31412	5.1677	-2.1689	0.057034	0.14813	-4.5932
Xpnpep2	X-prolyl aminopeptidase 2	117522	-0.26376	7.4169	-2.166	0.05731	0.14862	-4.5978
Trappc3	trafficking protein particle complex 3	362599	-0.50164	6.4377	-2.16	0.057878	0.14987	-4.6071
Dctn1	dynactin subunit 1	29167	0.19103	3.8724	2.1572	0.058149	0.15025	-4.6115
Slc25a42	"solute carrier family 25, member 42"	689414	0.25907	5.7867	2.1566	0.058201	0.15025	-4.6123
Rab14	"RAB14, member RAS oncogene family"	94197	-0.20338	6.3992	-2.1537	0.058486	0.15076	-4.6169
Cd48	Cd48 molecule	245962	0.24609	4.7716	2.1515	0.058693	0.15107	-4.6203
Rps11	ribosomal protein S11	81774	0.28177	6.5501	2.1467	0.059158	0.152	-4.6277
Alpl	"alkaline phosphatase, biomineralization a	25586	-0.2679	7.472	-2.146	0.059233	0.152	-4.6289
Pdpr	pyruvate dehydrogenase phosphatase re	307852	0.21269	7.7855	2.1416	0.059657	0.15285	-4.6356
Susd2	sushi domain containing 2	294335	0.19896	4.0357	2.1393	0.059892	0.15323	-4.6393
Mrpl20	mitochondrial ribosomal protein L20	680747	0.23636	11.443	2.136	0.06021	0.15381	-4.6443
Ndufa3	NADH:ubiquinone oxidoreductase subunit	691001	-0.22839	6.7135	-2.134	0.060413	0.1541	-4.6475
Sucla2	succinate-CoA ligase ADP-forming beta su	361071	-0.19092	7.7005	-2.1329	0.060524	0.15415	-4.6492
Adhfe1	"alcohol dehydrogenase, iron containing,	362474	0.39446	5.4511	2.1183	0.061993	0.15746	-4.6717
Clpp	caseinolytic mitochondrial matrix peptidase	301117	0.20672	7.8561	2.1174	0.062094	0.15746	-4.6733
Gda	guanine deaminase	83585	0.19753	7.8481	2.1173	0.0621	0.15746	-4.6734
Glg1	golgi glycoprotein 1	29476	-0.27069	7.6912	-2.1155	0.06228	0.15759	-4.6761
Txnrd2	thioredoxin reductase 2	50551	0.18907	4.9324	2.115	0.062336	0.15759	-4.6769
Trabd	TraB domain containing	300142	0.23007	5.8615	2.1111	0.062741	0.15838	-4.683
Parl	"presenilin associated, rhomboid-like"	287979	0.27238	8.6513	2.1067	0.063192	0.15928	-4.6897

LOC1083480	collagen alpha-1(XV) chain-like	108348074	-0.22459	6.1229	-2.1053	0.063337	0.15941	-4.6919
Aldh3a2	"aldehyde dehydrogenase 3 family, memb	65183	0.22049	11.792	2.0953	0.064393	0.16164	-4.7074
Erp29	endoplasmic reticulum protein 29	117030	0.20413	5.2906	2.0944	0.06448	0.16164	-4.7087
Uaca	uveal autoantigen with coiled-coil domains	315732	-0.22767	6.1501	-2.094	0.064526	0.16164	-4.7093
Tpm4	tropomyosin 4	24852	0.2891	11.366	2.0933	0.064598	0.16164	-4.7104
Rps6	ribosomal protein S6	29304	0.20565	9.6144	2.0915	0.064791	0.16172	-4.7132
Rpl23a	ribosomal protein L23a	360572	-0.2574	6.8537	-2.0913	0.064819	0.16172	-4.7136
Sdcbp	syndecan binding protein	83841	0.3222	9.2539	2.0884	0.065124	0.16214	-4.718
Cand1	cullin-associated and neddylation-dissocia	117152	0.21977	7.6946	2.0877	0.065202	0.16214	-4.7191
Mrpl21	mitochondrial ribosomal protein L21	309140	0.1897	5.9897	2.0868	0.065294	0.16214	-4.7204
Mylk3	myosin light chain kinase 3	291926	-0.18828	7.5926	-2.0861	0.06537	0.16214	-4.7215
Tpt1	"tumor protein, translationally-controlled 1'	116646	0.21923	5.2832	2.0842	0.065573	0.16241	-4.7244
Calr	calreticulin	64202	0.17703	6.0845	2.0808	0.065944	0.16309	-4.7297
Myof	myoferlin	309499	-0.31797	4.1087	-2.0777	0.066277	0.16368	-4.7344
Ddx1	DEAD-box helicase 1	84474	0.88458	7.7548	2.075	0.066575	0.16418	-4.7386
Rtn4	reticulon 4	83765	0.19842	6.5037	2.0708	0.067033	0.16507	-4.745
Atp5pb	ATP synthase peripheral stalk-membrane	171375	-0.20723	11.136	-2.0672	0.067433	0.16582	-4.7506
Rab6a	"RAB6A, member RAS oncogene family"	84379	0.23531	9.2908	2.0653	0.067648	0.16611	-4.7536
Iba57	iron-sulfur cluster assembly factor IBA57	363611	0.18989	8.9697	2.0567	0.068607	0.16803	-4.7667
Hspa4	heat shock protein family A (Hsp70) memb	266759	-0.20927	10.958	-2.0558	0.06871	0.16803	-4.7681
Clic4	chloride intracellular channel 4	83718	-0.17951	8.3599	-2.0556	0.068725	0.16803	-4.7683
Timm44	translocase of inner mitochondrial membra	29635	-0.19649	7.8161	-2.0542	0.068889	0.16819	-4.7705
Nt5c3a	"5'-nucleotidase, cytosolic IIIA"	312373	0.21109	4.6791	2.052	0.069139	0.16856	-4.7739
Pfn1	profilin 1	64303	-0.2533	6.7445	-2.0505	0.069312	0.16874	-4.7762
Stxbp1	syntaxin binding protein 1	25558	0.21969	7.056	2.0486	0.069521	0.16901	-4.7791
Rab12	"RAB12, member RAS oncogene family"	25530	-0.25006	9.6596	-2.0418	0.070305	0.17067	-4.7895
Chmp6	charged multivesicular body protein 6	287873	0.27767	5.5238	2.0343	0.071174	0.17253	-4.801
Card19	"caspase recruitment domain family, meml	361224	0.30243	6.9324	2.0319	0.071449	0.17296	-4.8046
Xdh	xanthine dehydrogenase	497811	0.29883	8.1805	2.0301	0.071658	0.17322	-4.8073
Lamc1	laminin subunit gamma 1	117036	0.20853	5.9494	2.0269	0.072045	0.17391	-4.8123
Preb	prolactin regulatory element binding	58842	0.85096	4.7946	2.0232	0.072483	0.17472	-4.8179
Lin7c	"lin-7 homolog C, crumbs cell polarity com	60442	-0.31188	7.1598	-2.0217	0.072655	0.17488	-4.8201
Igf2r	insulin-like growth factor 2 receptor	25151	0.21803	7.486	2.0171	0.0732	0.17584	-4.8271
Rhog	ras homolog family member G	308875	-0.22176	8.8561	-2.0163	0.073306	0.17584	-4.8284
Hars2	"histidyl-tRNA synthetase 2, mitochondrial	307491	0.19369	5.344	2.0158	0.073359	0.17584	-4.8291
Serbp1	Serpine1 mRNA binding protein 1	246303	0.68905	7.9264	2.0145	0.073518	0.17585	-4.8311
Colgalt1	collagen beta(1-O)galactosyltransferase 1	290637	-0.78755	7.0002	-2.0141	0.07357	0.17585	-4.8318
Aqp1	aquaporin 1	25240	-0.20742	4.6325	-2.0131	0.073687	0.17588	-4.8333
Emc3	ER membrane protein complex subunit 3	312640	0.22027	9.8701	2.0116	0.07386	0.17605	-4.8354
Podxl	podocalyxin-like	192181	-0.17931	7.9863	-2.0107	0.073976	0.17608	-4.8369

Ugp2	UDP-glucose pyrophosphorylase 2	289827	0.28443	6.7712	2.0044	0.07474	0.17765	-4.8464
Mpp7	membrane palmitoylated protein 7	307035	0.19596	7.2619	1.9999	0.075296	0.17872	-4.8533
Endog	endonuclease G	362100	-0.20754	7.1861	-1.9941	0.076013	0.18017	-4.8621
Apoo	apolipoprotein O	363474	-0.18815	5.9881	-1.9911	0.076384	0.18065	-4.8666
Serpina1	serpin family A member 1	24648	-0.22143	9.1985	-1.9907	0.076426	0.18065	-4.8671
Anp32b	acidic nuclear phosphoprotein 32 family m	170724	-0.34841	8.027	-1.9885	0.07671	0.181	-4.8706
Myo1b	myosin Ib	117057	-0.19702	7.822	-1.9874	0.076847	0.181	-4.8722
Dsg2	desmoglein 2	307562	-0.29879	6.075	-1.987	0.076892	0.181	-4.8728
Ndufb2	NADH:ubiquinone oxidoreductase subunit	362344	-0.20867	7.2981	-1.9833	0.077361	0.18186	-4.8784
Gdi2	GDP dissociation inhibitor 2	29662	0.20205	9.3172	1.9794	0.077849	0.18269	-4.8842
Rras	RAS related	361568	0.28474	7.4262	1.9787	0.077944	0.18269	-4.8854
Ptcd3	Pentatricopeptide repeat domain 3	500199	-0.22302	6.1764	-1.978	0.078035	0.18269	-4.8864
Mrps11	mitochondrial ribosomal protein S11	499185	0.23065	7.7742	1.9753	0.078371	0.18322	-4.8904
Mrpl51	mitochondrial ribosomal protein L51	297601	0.63217	6.3304	1.9724	0.078753	0.18368	-4.8949
Enpp1	ectonucleotide pyrophosphatase/phospho	85496	-0.22731	5.0246	-1.9721	0.078783	0.18368	-4.8953
Coa5	cytochrome C oxidase assembly factor 5	503252	-0.23294	8.5905	-1.9702	0.079026	0.184	-4.8981
Dcn	decorin	29139	-0.20811	6.0417	-1.9662	0.079549	0.18475	-4.9042
Rpl7a	ribosomal protein L7a	296596	-0.20232	7.1816	-1.9661	0.079565	0.18475	-4.9044
Rab3a	"RAB3A, member RAS oncogene family"	25531	0.19527	6.0904	1.9637	0.079869	0.18508	-4.9079
Gnai3	G protein subunit alpha i3	25643	0.25535	10.114	1.9633	0.079922	0.18508	-4.9085
Uba1	ubiquitin-like modifier activating enzyme 1	314432	-0.18245	7.3268	-1.9606	0.080276	0.18564	-4.9126
Mthfd1	"methylenetetrahydrofolate dehydrogenas	64300	0.1862	5.2816	1.9591	0.080473	0.18585	-4.9149
Stxbp3	syntaxin binding protein 3	114095	0.19522	6.069	1.9569	0.080757	0.18625	-4.9181
Nenf	neudesin neurotrophic factor	289380	0.22972	6.4202	1.9551	0.080998	0.18656	-4.9209
Ppa2	inorganic pyrophosphatase 2	310856	-0.17186	7.7232	-1.9488	0.081836	0.18797	-4.9304
Myh10	myosin heavy chain 10	79433	-0.25139	8.8005	-1.9486	0.081853	0.18797	-4.9306
Nfs1	NFS1 cysteine desulfurase	84594	-0.25132	6.7783	-1.948	0.081943	0.18797	-4.9316
Nap1l4	nucleosome assembly protein 1-like 4	361684	0.27712	4.0642	1.9442	0.082452	0.18864	-4.9373
Pgm1	phosphoglucomutase 1	24645	0.23574	6.6251	1.9441	0.082455	0.18864	-4.9373
Ehhadh	enoyl-CoA hydratase and 3-hydroxyacyl C	171142	-0.19721	5.6171	-1.9406	0.082929	0.18933	-4.9426
Ezr	ezrin	54319	-0.24802	6.1588	-1.9402	0.082978	0.18933	-4.9432
Rpl13	ribosomal protein L13	81765	0.37561	4.9858	1.9377	0.083324	0.18963	-4.947
Atp5f1d	ATP synthase F1 subunit delta	245965	-0.1713	10.577	-1.9371	0.083404	0.18963	-4.9479
Fhl2	four and a half LIM domains 2	63839	0.19825	6.704	1.9368	0.083439	0.18963	-4.9483
Mrpl44	mitochondrial ribosomal protein L44	301552	0.17603	5.7498	1.9355	0.083627	0.1898	-4.9503
Mrpl10	mitochondrial ribosomal protein L10	691075	0.20492	9.4087	1.9332	0.083934	0.19021	-4.9537
Psmd3	"proteasome 26S subunit, non-ATPase 3"	287670	0.21924	5.106	1.9315	0.084171	0.19021	-4.9563
Myh11	myosin heavy chain 11	24582	-0.1815	8.4132	-1.9311	0.084225	0.19021	-4.9569
Rpl37a-ps1	"ribosomal protein L37a, pseudogene 1"	363248	-0.19927	6.0985	-1.9309	0.084254	0.19021	-4.9572
Eprs	glutamyl-prolyl-tRNA synthetase	289352	-0.21561	9.2705	-1.9298	0.084396	0.19028	-4.9588

Ndufa4	"NDUFA4, mitochondrial complex associat	681024	-0.17428	8.8303	-1.9243	0.085148	0.19172	-4.9669
Supv3l1	Suv3 like RNA helicase	294385	-0.20118	5.8742	-1.9229	0.08535	0.19192	-4.9691
Hsdl1	hydroxysteroid dehydrogenase like 1	361418	0.24757	4.1358	1.92	0.085744	0.19237	-4.9734
Mcu	mitochondrial calcium uniporter	294560	0.23305	5.6706	1.9198	0.085772	0.19237	-4.9737
Hspe1	heat shock protein family E (Hsp10) memb	25462	-0.20772	5.0975	-1.918	0.086029	0.19266	-4.9764
Lamb1	laminin subunit beta 1	298941	-0.20543	7.0312	-1.917	0.086165	0.19266	-4.9779
Mrps14	mitochondrial ribosomal protein S14	289143	0.24914	4.5979	1.9165	0.086239	0.19266	-4.9786
Mettl7a	methyltransferase like 7A	315306	-0.17464	5.4273	-1.9136	0.086651	0.19314	-4.983
Exog	exo/endonuclease G	301062	0.268	7.5019	1.9134	0.08668	0.19314	-4.9833
Sacm1l	SAC1 like phosphatidylinositide phosphat	116482	-0.1851	8.1516	-1.91	0.087149	0.19393	-4.9883
Ube2m	ubiquitin-conjugating enzyme E2M	361509	-0.23069	5.1603	-1.9066	0.087632	0.1946	-4.9934
Slc2a4	solute carrier family 2 member 4	25139	-0.19963	5.9301	-1.9063	0.087679	0.1946	-4.9939
Hp	haptoglobin	24464	-0.22411	5.0667	-1.9039	0.088012	0.19509	-4.9973
Atp2b1	ATPase plasma membrane Ca2+ transpor	29598	0.18183	8.1527	1.9024	0.088227	0.19531	-4.9996
Agpat3	1-acylglycerol-3-phosphate O-acyltransfera	294324	-0.17544	5.089	-1.9013	0.088384	0.19541	-5.0012
Col4a2	collagen type IV alpha 2 chain	306628	-0.33451	7.8468	-1.8982	0.088833	0.19614	-5.0058
Perm1	"PPARGC1 and ESRR induced regulator,	313776	0.23272	11.01	1.8889	0.090178	0.19883	-5.0196
Psmb2	proteasome 20S subunit beta 2	29675	0.22594	6.8476	1.8874	0.090392	0.19883	-5.0218
Gstm2	glutathione S-transferase mu 2	24424	-0.17314	9.1338	-1.8871	0.090446	0.19883	-5.0223
Lmo7	LIM domain 7	361084	-0.20041	5.8724	-1.8866	0.090513	0.19883	-5.023
Npepps	aminopeptidase puromycin sensitive	50558	0.22946	7.5361	1.8857	0.090641	0.19885	-5.0243
Cyb5b	cytochrome b5 type B	80773	-0.16326	7.6851	-1.8775	0.091858	0.20126	-5.0365
Armc10	armadillo repeat containing 10	296758	-0.19483	8.2276	-1.8744	0.092325	0.20203	-5.0412
Idh1	isocitrate dehydrogenase (NADP(+)) 1	24479	0.33376	7.6964	1.8733	0.092486	0.20212	-5.0427
Itih4	inter-alpha-trypsin inhibitor heavy chain 4	54404	-0.19522	6.507	-1.869	0.093125	0.20326	-5.049
Hacl1	2-hydroxyacyl-CoA lyase 1	85255	0.23797	6.2191	1.8636	0.093945	0.20456	-5.057
Coq3	coenzyme Q3 methyltransferase	29309	-0.16614	8.2984	-1.8635	0.093961	0.20456	-5.0572
Dnm1l	dynamin 1-like	114114	-0.17987	5.0215	-1.8615	0.094264	0.20496	-5.0601
Vta1	vesicle trafficking 1	292640	-0.24144	8.1531	-1.8594	0.094579	0.20538	-5.0632
Pam	peptidylglycine alpha-amidating monooxyg	25508	-0.16041	8.0608	-1.8584	0.094734	0.20546	-5.0647
Cops4	COP9 signalosome subunit 4	360915	0.63704	7.7359	1.8561	0.09508	0.20595	-5.068
Rcn1	reticulocalbin 1	362182	-0.1987	5.909	-1.8514	0.095811	0.20727	-5.075
Niban2	niban apoptosis regulator 2	362115	-0.26701	7.2368	-1.847	0.096487	0.2083	-5.0814
Nudt19	nudix hydrolase 19	308518	0.15896	7.8045	1.8467	0.096531	0.2083	-5.0818
Ptpmt1	"protein tyrosine phosphatase, mitochond	29390	0.21634	4.7899	1.8448	0.096828	0.20852	-5.0846
Actr1b	actin related protein 1B	316333	0.26629	7.6956	1.8445	0.09688	0.20852	-5.0851
Akr1a1	aldo-keto reductase family 1 member A1	78959	0.17497	8.2001	1.8424	0.097206	0.20896	-5.0881
Rps27a	ribosomal protein S27a	100912032	-0.16431	7.2398	-1.8364	0.098149	0.21017	-5.0969
Acad9	"acyl-CoA dehydrogenase family, member	294973	0.16655	8.2599	1.8364	0.098154	0.21017	-5.097
Dhodh	dihydroorotate dehydrogenase (quinone)	65156	0.15903	3.8486	1.8358	0.098248	0.21017	-5.0978

Calm3	calmodulin 3	24244	-0.15944	4.9805	-1.8357	0.098261	0.21017	-5.098
Tmem143	transmembrane protein 143	308593	-0.17785	12.529	-1.8335	0.0986	0.21063	-5.1011
Hyou1	hypoxia up-regulated 1	192235	-0.19938	8.0512	-1.8322	0.098812	0.21082	-5.1031
Unc45b	unc-45 myosin chaperone B	303373	0.15665	4.9445	1.8314	0.098937	0.21083	-5.1042
Rps26	ribosomal protein S26	27139	-0.2488	14.418	-1.8235	0.1002	0.21325	-5.1157
Cds2	CDP-diacylglycerol synthase 2	114101	-0.17357	7.9736	-1.8219	0.10045	0.21328	-5.118
Slc25a24	solute carrier family 25 member 24	310791	0.19636	9.9443	1.8219	0.10046	0.21328	-5.1181
Rala	RAS like proto-oncogene A	81757	0.36157	5.0082	1.8198	0.10079	0.21368	-5.1211
Dnm2	dynamin 2	25751	-0.17261	5.2469	-1.8192	0.1009	0.21368	-5.122
Gna11	G protein subunit alpha 11	81662	0.19054	4.9968	1.8087	0.1026	0.21702	-5.1372
Abhd11	abhydrolase domain containing 11	360831	0.21715	5.5036	1.8024	0.10364	0.21867	-5.1463
Ndufa8	NADH:ubiquinone oxidoreductase subunit	296658	-0.1645	8.2483	-1.8021	0.1037	0.21867	-5.1468
Dmac2l	distal membrane arm assembly complex 2	362749	-0.23511	6.4711	-1.8017	0.10377	0.21867	-5.1474
Slc44a2	solute carrier family 44 member 2	363024	0.25141	5.3836	1.7991	0.1042	0.21931	-5.1512
Mrps35	mitochondrial ribosomal protein S35	297727	0.16362	6.1942	1.7982	0.10435	0.21936	-5.1525
LOC1009111	leucine-rich repeat-containing protein 10-li	100911101	0.20217	4.8433	1.7974	0.10448	0.21936	-5.1536
Sorbs1	sorbin and SH3 domain containing 1	686098	0.16952	8.1777	1.7879	0.10609	0.22247	-5.1675
Pkm	pyruvate kinase M1/2	25630	-0.16458	7.1651	-1.7857	0.10646	0.22252	-5.1706
Cbr4	carbonyl reductase 4	359725	0.17385	5.358	1.785	0.10657	0.22252	-5.1715
Nceh1	neutral cholesterol ester hydrolase 1	294930	-0.17795	10.935	-1.785	0.10658	0.22252	-5.1716
Rmc1	regulator of MON1-CCZ1	291784	0.17217	7.1705	1.7846	0.10664	0.22252	-5.1721
Cstb	cystatin B	25308	0.25459	5.3717	1.7817	0.10714	0.22303	-5.1763
Uqcc2	ubiquinol-cytochrome c reductase complex	361805	0.16964	6.6409	1.7809	0.10728	0.22303	-5.1775
Rpl24	ribosomal protein L24	64307	-0.22717	7.8918	-1.7807	0.10731	0.22303	-5.1778
Arpc5	"actin related protein 2/3 complex, subuni	360854	-0.22075	5.1667	-1.7801	0.10741	0.22303	-5.1786
Stat1	signal transducer and activator of transcrip	25124	0.20434	6.1838	1.7726	0.1087	0.22545	-5.1894
Slc25a51	"solute carrier family 25, member 51"	100909697	0.1759	3.8755	1.7719	0.10884	0.22545	-5.1905
Pgam2	phosphoglycerate mutase 2	24959	-0.17396	8.0026	-1.7698	0.10919	0.22591	-5.1934
Bcap29	B-cell receptor-associated protein 29	298943	0.18176	9.2763	1.7659	0.10988	0.22707	-5.1991
Rps24	ribosomal protein S24	81776	-0.20166	8.7684	-1.7636	0.11028	0.22762	-5.2024
Auh	AU RNA binding methylglutaconyl-CoA hy	361215	-0.17395	6.1904	-1.7604	0.11085	0.22851	-5.207
Mesd	mesoderm development LRP chaperone	308796	-0.17349	4.1412	-1.7579	0.11129	0.22914	-5.2106
Rab10	"RAB10, member RAS oncogene family"	50993	-0.17801	7.0554	-1.7562	0.11159	0.22949	-5.213
Psmd12	"proteasome 26S subunit, non-ATPase 12	287772	-0.17936	6.2366	-1.7554	0.11173	0.22951	-5.2142
Rasa3	RAS p21 protein activator 3	29372	0.22619	11.209	1.7538	0.11202	0.22982	-5.2164
Me2	malic enzyme 2	307270	0.23013	5.1711	1.7487	0.11292	0.2314	-5.2237
Ywhah	"tyrosine 3-monooxygenase/tryptophan 5-	25576	-0.19165	5.5629	-1.7475	0.11314	0.23156	-5.2254
Fabp3	fatty acid binding protein 3	79131	-0.17281	12.603	-1.7433	0.11391	0.23286	-5.2315
Anxa3	annexin A3	25291	0.16255	6.7995	1.7381	0.11484	0.23449	-5.2388
Lamp1	lysosomal-associated membrane protein 1	25328	-0.15406	8.1996	-1.737	0.11504	0.23462	-5.2404

Ncl	nucleolin	25135	-0.2285	5.0923	-1.7276	0.11678	0.23784	-5.2538
Psmc5	"proteasome 26S subunit, ATPase 5"	81827	0.1569	5.4874	1.7258	0.11712	0.23784	-5.2563
Pdk4	pyruvate dehydrogenase kinase 4	89813	-0.15394	10.751	-1.7252	0.11722	0.23784	-5.2571
Emc8	ER membrane protein complex subunit 8	361425	0.16309	6.28	1.7249	0.11728	0.23784	-5.2576
Gdi1	GDP dissociation inhibitor 1	25183	0.21709	5.1742	1.7247	0.11732	0.23784	-5.2579
Psmd13	"proteasome 26S subunit, non-ATPase 1	365388	-0.20062	7.7842	-1.7224	0.11774	0.2384	-5.2611
Poldip2	DNA polymerase delta interacting protein	287544	-0.24393	6.0119	-1.7167	0.11882	0.24031	-5.2692
Ghitm	growth hormone inducible transmembrane	290596	0.15995	9.897	1.7141	0.11931	0.24101	-5.2729
Nadk2	"NAD kinase 2, mitochondrial"	365699	0.24953	4.2774	1.7082	0.12043	0.24298	-5.2812
Coro1c	coronin 1C	501841	-0.20848	7.4728	-1.7011	0.12177	0.24541	-5.2911
Yme1l1	YME1-like 1 ATPase	114217	0.16193	7.8397	1.7	0.12199	0.24545	-5.2928
Cat	catalase	24248	0.14484	6.2169	1.6996	0.12208	0.24545	-5.2934
Suox	sulfite oxidase	81805	-0.17063	5.0433	-1.6978	0.12241	0.24582	-5.2958
Plgrkt	plasminogen receptor with a C-terminal lys	293888	0.2073	4.9843	1.6958	0.12279	0.24613	-5.2986
Entpd1	ectonucleoside triphosphate diphosphohy	64519	-0.18283	6.2442	-1.6956	0.12285	0.24613	-5.299
Pfkl	"phosphofructokinase, liver type"	25741	0.17486	8.9627	1.6945	0.12306	0.24626	-5.3005
Tecr	"trans-2,3-enoyl-CoA reductase"	191576	-0.1769	11.072	-1.6921	0.12353	0.24692	-5.3039
Bves	blood vessel epicardial substance	365603	-0.17944	8.1777	-1.6863	0.12465	0.24886	-5.3119
Atpaf2	ATP synthase mitochondrial F1 complex a	303190	-0.18214	7.7334	-1.6854	0.12483	0.24894	-5.3133
Thbd	thrombomodulin	83580	-0.15437	4.3339	-1.6825	0.1254	0.24977	-5.3173
Fabp4	fatty acid binding protein 4	79451	-0.18227	11.848	-1.681	0.1257	0.25009	-5.3194
Dsp	desmoplakin	306871	-0.16098	7.6011	-1.6788	0.12613	0.25021	-5.3225
Dtnb	"dystrobrevin, beta"	362715	-0.16005	9.4675	-1.6785	0.1262	0.25021	-5.3229
Atp1b3	ATPase Na+/K+ transporting subunit beta	25390	-0.19299	6.8054	-1.6785	0.1262	0.25021	-5.3229
Ncln	nicalin	314648	0.22037	5.8803	1.677	0.12649	0.25049	-5.325
Eef2	eukaryotic translation elongation factor 2	29565	-0.15969	6.9807	-1.6763	0.12664	0.25049	-5.326
Acaa1a	acetyl-CoA acyltransferase 1A	24157	-0.17302	12.702	-1.6743	0.12703	0.25097	-5.3287
Chid1	chitinase domain containing 1	293628	0.17887	5.1027	1.672	0.12749	0.25147	-5.332
Cct6a	chaperonin containing TCP1 subunit 6A	288620	0.16367	5.4725	1.6716	0.12758	0.25147	-5.3326
Sod1	superoxide dismutase 1	24786	-0.16075	5.8484	-1.6689	0.12811	0.25196	-5.3363
Acot13	acyl-CoA thioesterase 13	291135	0.24887	5.1793	1.6689	0.12812	0.25196	-5.3363
Prkar2a	protein kinase cAMP-dependent type II re	29699	-0.18222	5.837	-1.6646	0.12898	0.25337	-5.3423
Rock2	Rho-associated coiled-coil containing prot	25537	-0.17989	6.035	-1.6634	0.12924	0.25358	-5.344
Rps14	ribosomal protein S14	29284	-0.18825	9.2505	-1.6598	0.12997	0.25466	-5.3491
Lpcat3	lysophosphatidylcholine acyltransferase 3	362434	0.19453	8.7351	1.6592	0.13009	0.25466	-5.3498
Rps8	ribosomal protein S8	65136	-0.19271	10.44	-1.6568	0.13058	0.25534	-5.3532
Isoc1		364879	0.43505	8.2131	1.6544	0.13106	0.25596	-5.3564
Fgg	fibrinogen gamma chain	24367	-0.17606	9.7242	-1.6538	0.1312	0.25596	-5.3574
Pmpca	"peptidase, mitochondrial processing alph	296588	-0.18124	11.712	-1.6509	0.1318	0.25656	-5.3614
Ap2m1	adaptor related protein complex 2 subunit	116563	-0.16098	9.481	-1.6509	0.13181	0.25656	-5.3614

Adipoq	"adiponectin, C1Q and collagen domain c	246253	0.20974	4.0742	1.6485	0.13229	0.25719	-5.3647
Eif5a	eukaryotic translation initiation factor 5A	287444	0.18871	8.4942	1.6478	0.13243	0.25719	-5.3656
Rpl32	ribosomal protein L32	28298	-0.21649	7.9582	-1.6454	0.13294	0.25788	-5.369
Maip1	matrix AAA peptidase interacting protein 1	301418	0.27553	6.7776	1.6431	0.13341	0.2585	-5.3721
Mrps6	mitochondrial ribosomal protein S6	100360017	-1.0437	7.0123	-1.6417	0.1337	0.25861	-5.374
Dock1	dedicator of cyto-kinesis 1	309081	0.76294	5.6467	1.6412	0.13381	0.25861	-5.3748
Utrn	utrophin	25600	0.17706	9.1414	1.6407	0.13392	0.25861	-5.3755
Dap3	death associated protein 3	295238	0.46863	11.832	1.6387	0.13433	0.25912	-5.3782
Yars2	tyrosyl-tRNA synthetase 2	287924	0.16187	6.7713	1.6357	0.13497	0.25979	-5.3824
Sntb2	"syntrophin, beta 2"	689421	-0.17579	7.3701	-1.6356	0.13499	0.25979	-5.3825
Rps3a	ribosomal protein S3a	29288	-0.19177	8.3839	-1.6337	0.13539	0.26027	-5.3851
Efr3a	EFR3 homolog A	362923	0.21687	4.8478	1.6289	0.13642	0.26167	-5.3918
Mrpl39	mitochondrial ribosomal protein L39	684304	-0.15911	6.8005	-1.6289	0.13642	0.26167	-5.3918
Scn7a	sodium voltage-gated channel alpha subu	64155	0.16901	5.9561	1.6272	0.13678	0.26207	-5.3941
Lrp1	LDL receptor related protein 1	299858	0.16076	5.2475	1.6224	0.13781	0.26346	-5.4007
Rpl18a	ribosomal protein L18A	290641	-0.2144	6.8871	-1.6224	0.13782	0.26346	-5.4008
Abcc1	ATP binding cassette subfamily C membe	24565	0.16251	9.6796	1.6203	0.13825	0.264	-5.4035
Pa2g4	proliferation-associated 2G4	288778	-0.1792	7.3417	-1.6178	0.1388	0.26474	-5.407
Gpd1l	glycerol-3-phosphate dehydrogenase 1-lik	363159	-0.14654	6.0625	-1.6123	0.14	0.26673	-5.4146
Psma1	proteasome 20S subunit alpha 1	29668	-0.19983	5.1867	-1.6074	0.14106	0.26846	-5.4212
Palm	paralemmin	170673	-0.17042	11.063	-1.6057	0.14144	0.26887	-5.4235
Rer1	retention in endoplasmic reticulum sorting	298675	-0.17829	10.012	-1.6042	0.14178	0.269	-5.4256
Fh	fumarate hydratase	24368	-0.14039	10.004	-1.604	0.14182	0.269	-5.4259
Cavin2	caveolae associated protein 2	316384	-0.15926	7.6279	-1.6014	0.14239	0.26979	-5.4294
Slc30a9	solute carrier family 30 member 9	498358	-0.15753	8.6526	-1.5933	0.14419	0.27289	-5.4404
Agrn	agrin	25592	0.18584	8.8959	1.586	0.14585	0.27572	-5.4504
Rpl18	ribosomal protein L18	81766	0.14761	6.8677	1.5846	0.14616	0.27601	-5.4522
Cdh5	cadherin 5	307618	-0.23657	5.8757	-1.5809	0.147	0.27716	-5.4572
Col6a2	collagen type VI alpha 2 chain	361821	-0.13684	8.7474	-1.5805	0.14709	0.27716	-5.4578
Abcb6	ATP binding cassette subfamily B membe	140669	-0.14377	9.5931	-1.5766	0.14798	0.27852	-5.463
Atp6v1d	ATPase H+ transporting V1 subunit D	299159	0.17509	5.8358	1.5727	0.14889	0.27992	-5.4684
Timm10	translocase of inner mitochondrial membra	64464	0.1728	6.8514	1.5683	0.14991	0.28144	-5.4743
Rab5a	"RAB5A, member RAS oncogene family"	64633	-0.15361	12.219	-1.5673	0.15014	0.28144	-5.4756
Flad1	flavin adenine dinucleotide synthetase 1	751787	0.18261	6.0493	1.5671	0.15019	0.28144	-5.4759
Qdpr	quinoid dihydropteridine reductase	64192	0.18859	7.9452	1.5629	0.15117	0.28296	-5.4816
Mydgf	myeloid-derived growth factor	501282	0.2029	8.0099	1.5599	0.15186	0.28394	-5.4856
Tars2	"threonyl-tRNA synthetase 2, mitochondria	310672	0.17962	6.9127	1.5592	0.15202	0.28394	-5.4865
Mpc2	mitochondrial pyruvate carrier 2	100359982	-0.17801	9.0127	-1.5514	0.15387	0.28673	-5.497
Vapa	VAMP associated protein A	58857	0.15206	5.7359	1.5512	0.15393	0.28673	-5.4973
Selenbp1	selenium binding protein 1	140927	0.23079	6.3704	1.5499	0.15423	0.28673	-5.499

Stx7	syntaxin 7	60466	-0.16235	4.6171	-1.5497	0.15428	0.28673	-5.4993
Lima1	LIM domain and actin binding 1	300228	0.20018	6.1175	1.5494	0.15436	0.28673	-5.4997
Pnp	purine nucleoside phosphorylase	290029	0.1592	6.6856	1.5474	0.15482	0.28727	-5.5023
Timm23	translocase of inner mitochondrial membra	54312	-0.1367	7.2523	-1.5466	0.15501	0.28732	-5.5034
Ano10	anoctamin 10	301111	0.33719	7.0886	1.5443	0.15555	0.28802	-5.5064
Lypla1	lysophospholipase 1	25514	-0.153	5.4022	-1.5429	0.15591	0.28836	-5.5084
Vars1	valyl-tRNA synthetase 1	25009	0.20642	4.7246	1.539	0.15683	0.28976	-5.5135
Arpc4	"actin related protein 2/3 complex, subuni	297518	0.16736	7.1371	1.5362	0.15752	0.29071	-5.5173
Fxn	frataxin	499335	-0.15608	12.671	-1.532	0.15854	0.29228	-5.5229
Sirt5	sirtuin 5	306840	0.16104	9.9675	1.5259	0.16003	0.29471	-5.5309
Echdc2	enoyl CoA hydratase domain containing 2	298381	0.15035	7.1037	1.5235	0.16061	0.29533	-5.5341
Lrrc59	leucine rich repeat containing 59	287633	-0.13094	7.4689	-1.5231	0.16071	0.29533	-5.5346
Tmx4	thioredoxin-related transmembrane protei	296182	0.14753	7.6716	1.5222	0.16093	0.29542	-5.5358
Stom	stomatin	296655	0.15145	4.6271	1.5213	0.16116	0.29551	-5.537
Wfs1	wolframin ER transmembrane glycoprotein	83725	-0.31808	5.3205	-1.52	0.16149	0.29581	-5.5388
Tmed7	transmembrane p24 trafficking protein 7	252889	0.1691	5.0183	1.5179	0.16202	0.29645	-5.5416
Iqgap1	IQ motif containing GTPase activating pro	361598	0.13038	7.6406	1.5165	0.16235	0.29675	-5.5434
Sec63	"SEC63 homolog, protein translocation re	309858	0.15963	4.7019	1.5154	0.16264	0.29696	-5.5449
Fkbp3	FKBP prolyl isomerase 3	299104	0.15637	8.3487	1.5133	0.16316	0.29758	-5.5476
Lyn	"LYN proto-oncogene, Src family tyrosine	81515	-0.21364	5.5971	-1.511	0.16372	0.2983	-5.5506
Vnn1	vanin 1	29142	-0.14729	9.7847	-1.5072	0.16468	0.29973	-5.5556
Ssr1	signal sequence receptor subunit 1	361233	0.19381	4.206	1.5046	0.16534	0.3006	-5.5591
Tmx1	thioredoxin-related transmembrane protei	362751	-0.15528	7.1022	-1.5025	0.16588	0.30127	-5.5619
Vim	vimentin	81818	-0.14447	7.8561	-1.5017	0.16608	0.30129	-5.5629
Fundc1	FUN14 domain containing 1	363442	0.17097	6.7257	1.501	0.16625	0.30129	-5.5638
LOC684270	similar to isochorismatase domain contain	684270	0.15531	6.4665	1.5001	0.16648	0.30139	-5.565
Rtraf	"RNA transcription, translation and transp	302247	0.17477	7.6624	1.4949	0.1678	0.30322	-5.5717
Immp1l	inner mitochondrial membrane peptidase s	691145	0.16767	7.7297	1.4948	0.16785	0.30322	-5.572
Tmem186	transmembrane protein 186	497863	0.16965	4.9741	1.494	0.16804	0.30325	-5.5729
Kpnb1	karyopherin subunit beta 1	24917	0.16056	6.9824	1.4923	0.16847	0.30371	-5.5752
Tmem38a	transmembrane protein 38a	306327	-0.15848	6.3794	-1.4902	0.16901	0.30432	-5.5779
Parvb	"parvin, beta"	362973	-0.145	7.5442	-1.4896	0.16917	0.30432	-5.5787
Rhot1	ras homolog family member T1	303351	-0.13288	8.7121	-1.4878	0.16963	0.30484	-5.581
Ank3	ankyrin 3	361833	0.23101	6.6445	1.4858	0.17016	0.30547	-5.5837
Pgrmc1	progesterone receptor membrane compor	291948	0.18633	6.9562	1.484	0.17064	0.306	-5.5861
Tm9sf3	transmembrane 9 superfamily member 3	309475	0.28737	4.2922	1.4825	0.17101	0.30635	-5.588
C7	complement C7	117517	0.16965	5.879	1.4765	0.17258	0.30884	-5.5958
Adcy5	adenylate cyclase 5	64532	-0.16989	9.7451	-1.4755	0.17284	0.30898	-5.5971
Ndufaf1	NADH:ubiquinone oxidoreductase comple	296086	-0.21126	6.7644	-1.4719	0.17379	0.31035	-5.6017
Mfn2	mitofusin 2	64476	0.16495	5.461	1.4705	0.17418	0.31071	-5.6036

Klhl41	kelch-like family member 41	117537	0.17475	4.0872	1.4687	0.17464	0.31071	-5.6059
Ahsg	alpha-2-HS-glycoprotein	25373	-0.19018	6.4279	-1.468	0.17482	0.31071	-5.6068
Bgn	biglycan	25181	-0.12955	6.3982	-1.4677	0.17491	0.31071	-5.6072
Nfu1	NFU1 iron-sulfur cluster scaffold	297416	-0.15386	4.184	-1.4672	0.17504	0.31071	-5.6079
Timmdc1	translocase of inner mitochondrial membra	303922	-0.16207	7.1763	-1.4671	0.17508	0.31071	-5.608
Cfl2	cofilin 2	366624	0.12927	5.894	1.4657	0.17543	0.31095	-5.6098
Foxred1	FAD-dependent oxidoreductase domain c	315547	-0.15276	9.3665	-1.4652	0.17558	0.31095	-5.6105
Nid1	nidogen 1	25494	-0.13331	6.9164	-1.4615	0.17656	0.31226	-5.6152
Erp44	endoplasmic reticulum protein 44	298066	-0.16677	7.1671	-1.4611	0.17668	0.31226	-5.6158
Rps27	ribosomal protein S27	94266	0.13752	5.9456	1.4595	0.17711	0.31258	-5.6179
Cdc42	cell division cycle 42	64465	0.14402	6.7399	1.4584	0.17739	0.31258	-5.6192
Cyfip1	cytoplasmic FMR1 interacting protein 1	308666	0.15016	5.6741	1.4583	0.17741	0.31258	-5.6193
Actn1	"actinin, alpha 1"	81634	0.13224	7.2636	1.4559	0.17806	0.31326	-5.6224
Itgb1	integrin subunit beta 1	24511	0.12831	4.9698	1.4548	0.17836	0.31326	-5.6239
S1pr1	sphingosine-1-phosphate receptor 1	29733	0.20586	6.4192	1.4545	0.17846	0.31326	-5.6243
Tm9sf2	transmembrane 9 superfamily member 2	306197	-0.15402	5.9361	-1.4542	0.17853	0.31326	-5.6247
Dut	deoxyuridine triphosphatase	497778	0.13881	6.5834	1.452	0.17913	0.31398	-5.6275
Stim1	stromal interaction molecule 1	361618	0.18076	10.673	1.4504	0.17957	0.31443	-5.6296
Cacna1c	calcium voltage-gated channel subunit alp	24239	-0.13061	7.0576	-1.4473	0.1804	0.31557	-5.6335
Mrps34	mitochondrial ribosomal protein S34	287126	0.34269	6.0054	1.4459	0.18079	0.31592	-5.6353
Tnnc1	"troponin C1, slow skeletal and cardiac typ	290561	-0.14633	9.9784	-1.4407	0.1822	0.31806	-5.6419
Bckdk	branched chain ketoacid dehydrogenase	29603	-0.17139	6.4111	-1.4378	0.18301	0.31914	-5.6457
Actr2	actin related protein 2	289820	0.16457	10.428	1.4369	0.18327	0.31928	-5.6469
Psma7	proteasome 20S subunit alpha 7	29674	-0.12691	7.9586	-1.4338	0.18413	0.32045	-5.6508
Rdx	radixin	315655	-0.14244	10.144	-1.4325	0.18447	0.32072	-5.6524
Rtcb	"RNA 2',3'-cyclic phosphate and 5'-OH liga	362855	0.14753	5.7803	1.4302	0.18512	0.32152	-5.6554
Psap	prosaposin	25524	-0.16576	8.229	-1.4197	0.18807	0.32631	-5.6687
Timm13	translocase of inner mitochondrial membra	252928	-0.15801	6.5363	-1.4168	0.1889	0.32742	-5.6724
Atl1	atlastin GTPase 1	362750	0.25361	7.9606	1.4098	0.19091	0.33035	-5.6813
Dnpep	aspartyl aminopeptidase	301529	0.1454	7.0796	1.4095	0.19098	0.33035	-5.6816
Emc1	ER membrane protein complex subunit 1	362643	0.14522	7.2144	1.4086	0.19125	0.33044	-5.6828
Itga2b	integrin subunit alpha 2b	685269	-0.18557	4.5454	-1.408	0.19142	0.33044	-5.6836
Gimap4	"GTPase, IMAP family member 4"	286938	0.16221	6.6478	1.4061	0.19196	0.33089	-5.686
Lama5	laminin subunit alpha 5	140433	0.46894	4.9979	1.4057	0.19206	0.33089	-5.6864
Tst	thiosulfate sulfurtransferase	25274	-0.12677	5.7058	-1.4003	0.19363	0.33325	-5.6932
Bag3	BAG cochaperone 3	293524	0.1389	11.3	1.3974	0.19447	0.33435	-5.6968
Hdhd5	haloacid dehalogenase like hydrolase dor	312680	0.16782	6.5354	1.3955	0.19503	0.33498	-5.6992
Myo1d	myosin ID	25485	-0.14049	6.9386	-1.3945	0.19531	0.33511	-5.7005
Mcee	methylmalonyl CoA epimerase	293829	-0.16943	10.59	-1.3936	0.19557	0.33511	-5.7016
Obscn	"obscurin, cytoskeletal calmodulin and titir	338458	-0.17158	11.608	-1.3932	0.19569	0.33511	-5.7021

Ltbp4	latent transforming growth factor beta bind	292734	-0.17153	7.3789	-1.3911	0.19631	0.33584	-5.7048
Plcd3	"phospholipase C, delta 3"	287745	0.122	5.6083	1.3899	0.19666	0.33609	-5.7062
S100a10	S100 calcium binding protein A10	81778	0.14064	9.3771	1.3876	0.19736	0.33695	-5.7092
Copa	COPI coat complex subunit alpha	304978	-0.16504	7.2664	-1.3829	0.19875	0.33898	-5.7151
Mug1	murinoglobulin 1	497794	0.14984	6.5551	1.3809	0.19932	0.3393	-5.7175
Plcb4	"phospholipase C, beta 4"	25031	-0.12738	5.1594	-1.3803	0.19952	0.3393	-5.7183
Tmsb4x	"thymosin beta 4, X-linked"	81814	-0.15883	6.676	-1.3796	0.19972	0.3393	-5.7191
Tmed1	transmembrane p24 trafficking protein 1	315461	-0.30194	6.2617	-1.3796	0.19973	0.3393	-5.7192
Phyh	phytanoyl-CoA 2-hydroxylase	114209	-0.14867	10.562	-1.3735	0.20154	0.34204	-5.7267
Gpam	"glycerol-3-phosphate acyltransferase, mit	29653	-0.15689	8.7717	-1.3625	0.20485	0.34731	-5.7403
Rhot2	ras homolog family member T2	287156	0.144	4.1706	1.3562	0.20678	0.35012	-5.748
Bpnt1	"3'(2'), 5'-bisphosphate nucleotidase 1"	64473	0.11666	5.0282	1.3548	0.20722	0.35012	-5.7498
Eng	endoglin	497010	-0.11909	7.8403	-1.3546	0.20728	0.35012	-5.75
Rpl27a	ribosomal protein L27a	293418	0.14711	6.6706	1.3544	0.20733	0.35012	-5.7502
Tmod2	tropomodulin 2	58814	-0.20561	4.5393	-1.3516	0.20822	0.3504	-5.7538
Psmb4	proteasome 20S subunit beta 4	58854	-0.12901	8.8347	-1.3513	0.20831	0.3504	-5.7541
Mmaa	metabolism of cobalamin associated A	291939	-0.44446	5.5538	-1.3512	0.20833	0.3504	-5.7542
Gfm2	"G elongation factor, mitochondrial 2"	294672	-0.16474	8.0817	-1.3511	0.20837	0.3504	-5.7544
Grsf1	G-rich RNA sequence binding factor 1	305256	-0.17067	5.1199	-1.3506	0.20852	0.3504	-5.755
Cald1	caldesmon 1	25687	-0.17858	6.6346	-1.349	0.20901	0.35088	-5.7569
Mrrf	mitochondrial ribosome recycling factor	311903	0.39219	5.307	1.3482	0.20926	0.35095	-5.7579
Rps12	ribosomal protein S12	65139	-0.13774	4.0617	-1.3469	0.20965	0.35126	-5.7594
Cd99	CD99 molecule (Xg blood group)	652929	-0.15141	4.8206	-1.3457	0.21002	0.35154	-5.7609
Rplp1	ribosomal protein lateral stalk subunit P1	140661	-0.12246	9.8746	-1.3441	0.21053	0.35204	-5.7629
Coro1a	coronin 1A	155151	0.14779	3.9024	1.3415	0.21134	0.35306	-5.7661
Cct4	chaperonin containing TCP1 subunit 4	29374	-0.13445	8.705	-1.3395	0.21198	0.35378	-5.7686
Dhrs7	dehydrogenase/reductase 7	299135	-0.14256	8.8553	-1.3372	0.21268	0.3546	-5.7713
St13	"ST13, Hsp70 interacting protein"	81800	0.14954	4.2902	1.3356	0.2132	0.35512	-5.7733
Slc25a19	solute carrier family 25 member 19	303676	-0.18202	10.019	-1.3313	0.21455	0.35703	-5.7785
Cox4i1	cytochrome c oxidase subunit 4i1	29445	-0.11988	6.6143	-1.3292	0.21522	0.35749	-5.7811
Rasip1	Ras interacting protein 1	292912	0.17098	4.2734	1.3291	0.21525	0.35749	-5.7812
Trnt1	tRNA nucleotidyl transferase 1	312616	0.15891	6.0752	1.3266	0.21603	0.35845	-5.7842
Dhrs4	dehydrogenase/reductase 4	266686	-0.12895	11.892	-1.3255	0.21641	0.35872	-5.7856
Tmod1	tropomodulin 1	25566	-0.12972	9.6886	-1.317	0.21912	0.36268	-5.7959
Tsg101	tumor susceptibility 101	292925	-0.18972	5.2265	-1.3167	0.21922	0.36268	-5.7963
Arpc3	"actin related protein 2/3 complex, subuni	288669	0.12363	8.6466	1.3126	0.22054	0.36451	-5.8012
Myo18a	myosin XVIIIa	360570	0.16315	7.0148	1.3116	0.22086	0.36466	-5.8024
Ggt5	gamma-glutamyltransferase 5	29566	-0.14827	4.8032	-1.311	0.22106	0.36466	-5.8031
Actr1a	actin related protein 1A	294010	-0.13465	13.15	-1.3061	0.22264	0.36692	-5.8089
Eef1d	eukaryotic translation elongation factor 1 d	300033	0.15995	7.3322	1.3041	0.22329	0.36763	-5.8113

Cpq	carboxypeptidase Q	58952	0.15208	6.1298	1.3028	0.22373	0.368	-5.8129
Asah1	N-acylsphingosine amidohydrolase 1	84431	-0.1235	8.9828	-1.3	0.22465	0.36916	-5.8163
Ap2b1	adaptor related protein complex 2 subunit	140670	-0.12886	9.6804	-1.2991	0.22495	0.36923	-5.8174
Gpc1	glypican 1	58920	0.11422	6.4724	1.2985	0.22512	0.36923	-5.818
Prkaca	protein kinase cAMP-activated catalytic su	25636	0.12577	4.5291	1.2965	0.22579	0.36987	-5.8204
Eif4g1	"eukaryotic translation initiation factor 4 ga	287986	0.13031	7.5906	1.2961	0.22595	0.36987	-5.821
Hspb6	heat shock protein family B (small) membe	192245	-0.11887	7.6848	-1.2949	0.22634	0.37016	-5.8224
Timm9	translocase of inner mitochondrial membra	171139	0.12755	7.9053	1.2921	0.22726	0.37131	-5.8257
Fundc2	FUN14 domain containing 2	361288	0.13169	7.7867	1.2861	0.22926	0.37422	-5.8329
Rpl27	ribosomal protein L27	64306	0.12263	5.7267	1.2835	0.23014	0.3753	-5.836
Sparc	secreted protein acidic and cysteine rich	24791	0.13696	5.5654	1.2824	0.23049	0.37551	-5.8372
Ucp3	uncoupling protein 3	25708	0.12743	6.868	1.2802	0.23124	0.37615	-5.8399
Gng10	G protein subunit gamma 10	114119	0.16781	8.5012	1.2799	0.23133	0.37615	-5.8401
Gstm7	"glutathione S-transferase, mu 7"	81869	-0.23157	4.832	-1.2771	0.2323	0.37737	-5.8435
Cnp	"2',3'-cyclic nucleotide 3' phosphodiestera	25275	0.11579	8.4187	1.2753	0.23289	0.37782	-5.8456
Gys1	glycogen synthase 1	690987	0.11421	6.9708	1.2745	0.23317	0.37782	-5.8466
Usp9x	"ubiquitin specific peptidase 9, X-linked"	363445	-0.71027	6.1279	-1.2743	0.23324	0.37782	-5.8468
Psmd4	"proteasome 26S subunit, non-ATPase 4"	83499	0.11628	7.8434	1.2711	0.23433	0.37905	-5.8506
Sbds	"SBDS, ribosome maturation factor"	288615	-0.13136	5.5447	-1.2707	0.23444	0.37905	-5.851
Nudt8	nudix hydrolase 8	361692	0.11922	7.0087	1.2671	0.23568	0.3807	-5.8553
Ppp2r2a	"protein phosphatase 2, regulatory subun	117104	0.17262	6.1679	1.2613	0.23768	0.3835	-5.8621
Ctdsp1	CTD small phosphatase 1	363249	0.12634	4.8796	1.2605	0.23794	0.3835	-5.863
Aldh1a1	"aldehyde dehydrogenase 1 family, memb	24188	0.13618	7.2574	1.2596	0.23827	0.3835	-5.8641
Ephx2	epoxide hydrolase 2	65030	-0.14162	6.8723	-1.2591	0.23843	0.3835	-5.8646
Gnaq	G protein subunit alpha q	81666	-0.14134	8.702	-1.2588	0.23854	0.3835	-5.865
Sccpdh	saccharopine dehydrogenase (putative)	305021	-0.15578	7.0989	-1.2569	0.2392	0.3842	-5.8672
Manf	mesencephalic astrocyte-derived neurotro	315989	-0.11174	4.8509	-1.253	0.24056	0.38603	-5.8718
Sorbs2	sorbin and SH3 domain containing 2	114901	-0.10815	4.5833	-1.252	0.24091	0.38623	-5.873
Akr1c15	"aldo-keto reductase family 1, member C1	361267	-0.11262	5.423	-1.2501	0.24157	0.38692	-5.8752
Qrsl1	glutaminyl-tRNA amidotransferase subunit	309911	0.23882	7.0232	1.2456	0.24313	0.38897	-5.8804
Cmc1	C-x(9)-C motif containing 1	363162	0.15371	5.4459	1.2451	0.2433	0.38897	-5.8809
Lmcd1	LIM and cysteine-rich domains 1	494021	-0.1257	6.8871	-1.2437	0.24382	0.38938	-5.8826
Pabpc4	"poly(A) binding protein, cytoplasmic 4"	298510	-0.12128	4.6828	-1.2431	0.24402	0.38938	-5.8833
Serpinc1	serpin family C member 1	304917	-0.33523	7.5284	-1.242	0.24441	0.38964	-5.8846
Stoml2	stomatin like 2	298203	-0.10797	6.5152	-1.2379	0.24587	0.3916	-5.8893
Cdc37	cell division cycle 37	114562	0.1178	6.6719	1.2365	0.24633	0.39194	-5.8909
Pgs1	phosphatidylglycerophosphate synthase 1	303698	-0.18939	11.348	-1.236	0.24654	0.39194	-5.8915
Ppp2r1b	protein phosphatase 2 scaffold subunit A	315648	0.1338	6.8812	1.2339	0.24728	0.39275	-5.8939
Retsat	retinol saturase	246298	-0.12908	10.891	-1.2309	0.24834	0.39394	-5.8974
Abcd1	ATP binding cassette subfamily D membe	363516	0.11032	5.6225	1.2299	0.2487	0.39394	-5.8985

Prdx1	peroxiredoxin 1	117254	-0.11951	5.3616	-1.2298	0.24872	0.39394	-5.8986
Rab21	"RAB21, member RAS oncogene family"	299799	-0.12742	8.0526	-1.229	0.24904	0.39408	-5.8996
Ccdc47	coiled-coil domain containing 47	303606	0.11699	7.2612	1.2263	0.24997	0.3952	-5.9026
Tmx2	thioredoxin-related transmembrane protei	295701	-0.15792	9.0172	-1.2253	0.25035	0.39543	-5.9038
Slc38a2	"solute carrier family 38, member 2"	29642	0.14366	5.3796	1.2228	0.25123	0.39645	-5.9066
Glipr2	GLI pathogenesis-related 2	679819	0.11854	5.1892	1.22	0.25227	0.39773	-5.9099
Ndufb3	NADH:ubiquinone oxidoreductase subuni	301427	-0.11636	4.6546	-1.2193	0.25252	0.39775	-5.9107
Rhob	ras homolog family member B	64373	0.12907	11.492	1.2162	0.25364	0.39914	-5.9142
Aoc3	"amine oxidase, copper containing 3"	29473	-0.11442	7.2602	-1.2143	0.25432	0.39985	-5.9164
Slc43a1	solute carrier family 43 member 1	311168	0.1619	6.4024	1.2134	0.25467	0.40003	-5.9175
Cd34	CD34 molecule	305081	0.18656	13.594	1.212	0.25518	0.40046	-5.919
Cav3	caveolin 3	29161	0.10189	6.7316	1.2106	0.25566	0.40085	-5.9205
Eci1	enoyl-CoA delta isomerase 1	29740	-0.10485	7.273	-1.2088	0.25634	0.40155	-5.9227
Lamb2	laminin subunit beta 2	25473	-0.12064	7.6405	-1.2068	0.25707	0.40232	-5.9249
Fdxr	ferredoxin reductase	79122	0.12233	9.1311	1.2054	0.2576	0.40278	-5.9265
Mpz	myelin protein zero	24564	-0.12816	10.195	-1.2033	0.25835	0.40359	-5.9289
Fscn1	fascin actin-bundling protein 1	683788	-0.11764	4.9293	-1.2001	0.25954	0.40507	-5.9325
Tmlhe	"trimethyllysine hydroxylase, epsilon"	170898	0.14064	7.5543	1.1991	0.25992	0.40529	-5.9336
Rpl5	ribosomal protein L5	81763	-0.13235	12.213	-1.1967	0.26081	0.40631	-5.9364
Cmbl	carboxymethylenebutenolidase homolog	310201	0.12084	9.1034	1.1948	0.26153	0.40668	-5.9385
Spr	sepiapterin reductase	29270	0.12705	5.0767	1.194	0.26183	0.40668	-5.9394
Scfd1	sec1 family domain containing 1	54350	-0.32336	5.2092	-1.1934	0.26202	0.40668	-5.94
Pafah1b1	"platelet-activating factor acetylhydrolase	83572	-0.14613	7.6812	-1.193	0.26216	0.40668	-5.9404
Vps35	VPS35 retromer complex component	25479	-0.12537	11.659	-1.1928	0.26225	0.40668	-5.9407
Akr1b1	aldo-keto reductase family 1 member B	24192	-0.13819	8.7285	-1.1922	0.26247	0.40668	-5.9414
Mrpl1	mitochondrial ribosomal protein L1	289491	-0.14274	4.1391	-1.1861	0.26475	0.40983	-5.9482
Rpl15	ribosomal protein L15	245981	-0.12473	11.61	-1.1844	0.26542	0.41049	-5.9502
Epdr1	ependymin related 1	291180	-0.14776	6.6277	-1.1815	0.26651	0.41158	-5.9534
Elob	elongin B	81807	0.18197	6.9683	1.1812	0.2666	0.41158	-5.9537
Amacr	alpha-methylacyl-CoA racemase	25284	-0.1414	5.7099	-1.1782	0.26777	0.41279	-5.9571
Usp5	ubiquitin specific peptidase 5	297593	-0.13429	12.422	-1.1779	0.26787	0.41279	-5.9574
Serpina3n	"serine (or cysteine) peptidase inhibitor, cl	24795	-0.10886	7.223	-1.1703	0.27075	0.41685	-5.9658
Gsk3b	glycogen synthase kinase 3 beta	84027	-0.11748	6.0805	-1.1688	0.27133	0.41737	-5.9675
Isca1	iron-sulfur cluster assembly 1	290985	0.59219	8.17	1.1648	0.27287	0.41937	-5.9719
Dstn	"destrin, actin depolymerizing factor"	502674	0.10588	10.42	1.1633	0.27346	0.4199	-5.9736
Arpc1b	"actin related protein 2/3 complex, subuni	54227	-0.10153	6.6393	-1.1574	0.27575	0.42303	-5.9801
Lman2	"lectin, mannose-binding 2"	290994	0.10492	6.975	1.149	0.27902	0.42766	-5.9893
Coq10a	coenzyme Q10A	362810	-0.12567	9.5836	-1.1407	0.2823	0.4322	-5.9984
Rab8a	"RAB8A, member RAS oncogene family"	117103	-0.15375	8.5285	-1.1402	0.28248	0.4322	-5.9989
Pdf	peptide deformylase (mitochondrial)	690214	-0.10925	9.104	-1.1394	0.28281	0.43232	-5.9998

Spryd4	SPRY domain containing 4	288772	0.15059	7.0218	1.138	0.28335	0.43271	-6.0013
Rab5b	"RAB5B, member RAS oncogene family"	288779	0.38412	7.9077	1.1375	0.28358	0.43271	-6.0019
Erap1	endoplasmic reticulum aminopeptidase 1	80897	-0.1163	8.3917	-1.1348	0.28465	0.43377	-6.0048
Pigs	"phosphatidylinositol glycan anchor biosyr	303277	-0.14191	7.8483	-1.1341	0.28491	0.43377	-6.0055
Igtp	interferon gamma induced GTPase	303163	0.1014	4.966	1.1338	0.28503	0.43377	-6.0059
Rpl22	ribosomal protein L22	81768	-0.12911	10.039	-1.1283	0.28721	0.4367	-6.0118
Arhgdib	Rho GDP dissociation inhibitor beta	362456	-0.10508	6.6711	-1.1252	0.28847	0.43806	-6.0152
Anxa7	annexin A7	155423	0.30302	5.1	1.1245	0.28876	0.43806	-6.0159
Vps29	VPS29 retromer complex component	288666	0.19326	4.959	1.1242	0.28888	0.43806	-6.0162
Napg	NSF attachment protein gamma	307382	-0.13122	9.9388	-1.1215	0.28998	0.4391	-6.0192
Serhl2	serine hydrolase-like 2	500911	0.107	5.8774	1.1209	0.2902	0.4391	-6.0198
Mpdu1	mannose-P-dolichol utilization defect 1	303244	0.11191	5.3248	1.1206	0.29033	0.4391	-6.0201
Lcp1	lymphocyte cytosolic protein 1	306071	0.12257	7.0012	1.1191	0.29093	0.43961	-6.0217
Nmnat3	nicotinamide nucleotide adenylyltransfera	363118	-0.15139	13.187	-1.1178	0.29149	0.44007	-6.0232
Smyd1	SET and MYND domain containing 1	297333	0.15932	4.8171	1.1166	0.29194	0.44037	-6.0244
RGD1309362	similar to interferon-inducible GTPase	307415	-0.13185	7.8575	-1.1136	0.29319	0.44185	-6.0276
Tmem11	transmembrane protein 11	303196	0.19553	6.5298	1.1114	0.29407	0.44279	-6.0299
Sar1b	"secretion associated, Ras related GTPas	287276	-0.12152	5.6859	-1.107	0.29588	0.44512	-6.0347
Dpep1	dipeptidase 1	94199	0.10787	6.5032	1.1027	0.29764	0.44738	-6.0392
Emc7	ER membrane protein complex subunit 7	296050	0.16198	4.4409	1.0974	0.29984	0.45029	-6.0448
Rpl11	ribosomal protein L11	362631	-0.10342	7.2604	-1.0949	0.3009	0.45149	-6.0475
Itgb3	integrin subunit beta 3	29302	0.13645	7.5067	1.0888	0.30343	0.45487	-6.0539
Dync1li1	dynein cytoplasmic 1 light intermediate ch	252902	0.29644	7.142	1.0869	0.30422	0.45566	-6.0559
Snx2	sorting nexin 2	291464	0.20622	3.7709	1.0847	0.30517	0.45669	-6.0582
Set	SET nuclear proto-oncogene	307947	-0.1111	5.4359	-1.0834	0.3057	0.45708	-6.0596
Eps15	epidermal growth factor receptor pathway	313474	-0.10979	4.7903	-1.0715	0.31073	0.4642	-6.0719
Slc25a34	"solute carrier family 25, member 34"	298606	-0.10601	5.4156	-1.0645	0.31373	0.46771	-6.0792
Golga5	golgin A5	299258	-0.15494	7.4117	-1.0639	0.31401	0.46771	-6.0798
Add2	adducin 2	24171	0.12335	5.7087	1.0639	0.31401	0.46771	-6.0798
Magt1	magnesium transporter 1	116967	0.11048	6.7442	1.0635	0.31418	0.46771	-6.0802
Ndufb1	NADH:ubiquinone oxidoreductase subunit	100912357	-0.12126	6.312	-1.0622	0.31473	0.46812	-6.0815
Rpl13a	ribosomal protein L13A	317646	0.15602	4.6594	1.059	0.31612	0.46978	-6.0849
Tefm	"transcription elongation factor, mitochond	287554	0.092262	7.2427	1.0556	0.31759	0.47155	-6.0884
Tspan8	tetraspanin 8	171048	-0.16747	7.6583	-1.0529	0.31878	0.47266	-6.0912
Aprt	adenine phosphoribosyl transferase	292072	-0.15269	7.4567	-1.0526	0.31889	0.47266	-6.0914
Sh3glb1	SH3 domain -containing GRB2-like endop	292156	-0.10225	6.5639	-1.0495	0.32022	0.47423	-6.0945
Gbe1	"1,4-alpha-glucan branching enzyme 1"	288333	0.14951	3.9225	1.0431	0.32303	0.47762	-6.1011
LOC681355	similar to potassium channel tetramerisatio	681355	0.11815	7.0011	1.0431	0.32307	0.47762	-6.1012
Impdh2	inosine monophosphate dehydrogenase 2	301005	-0.10329	8.5473	-1.0381	0.32526	0.48045	-6.1062
Ola1	Obg-like ATPase 1	296488	-0.13068	5.764	-1.0343	0.32693	0.48249	-6.11

Nrp1	neuropilin 1	246331	0.12035	7.059	1.0335	0.3273	0.48263	-6.1109
Apmap	adipocyte plasma membrane associated p	366227	0.12543	5.7989	1.0323	0.32784	0.483	-6.1121
Fhit	fragile histidine triad diadenosine triphosp	60398	0.11051	7.5487	1.0265	0.3304	0.48635	-6.1179
Gpd1	glycerol-3-phosphate dehydrogenase 1	60666	0.10529	7.6713	1.024	0.33151	0.48757	-6.1203
Ppp5c	"protein phosphatase 5, catalytic subunit"	65179	0.11595	7.4656	1.0189	0.33382	0.49054	-6.1255
Qars1	glutaminyl-tRNA synthetase 1	290868	0.12563	6.5739	1.018	0.33424	0.49073	-6.1264
Mtfr1l	mitochondrial fission regulator 1-like	298549	-0.13661	7.2641	-1.0154	0.33542	0.49204	-6.129
Akap2	A-kinase anchoring protein 2	298024	0.1401	5.409	1.0131	0.33642	0.49309	-6.1312
Atp6v1a	ATPase H+ transporting V1 subunit A	685232	0.099636	6.1431	1.0106	0.33759	0.49438	-6.1338
Ran	"RAN, member RAS oncogene family"	84509	-0.13971	8.0025	-1.0072	0.33913	0.49621	-6.1371
Esyt2	extended synaptotagmin 2	299488	0.099565	4.922	1.0042	0.34047	0.49775	-6.14
Picalm	phosphatidylinositol binding clathrin asser	89816	0.091712	5.667	1.0008	0.34203	0.49939	-6.1433
Romo1	reactive oxygen species modulator 1	679572	-0.088166	7.4323	-0.99991	0.34246	0.49939	-6.1443
Cyp4b1	"cytochrome P450, family 4, subfamily b, p	24307	0.10085	5.9737	0.99987	0.34247	0.49939	-6.1443
Abce1	ATP binding cassette subfamily E membe	361390	0.10258	7.8442	0.99788	0.34339	0.5003	-6.1463
Timm10b	translocase of inner mitochondrial membra	84384	-0.10062	7.4488	-0.99618	0.34417	0.50101	-6.1479
P4ha2	prolyl 4-hydroxylase subunit alpha 2	360526	0.26295	5.2158	0.99432	0.34503	0.50122	-6.1497
Mtch1	mitochondrial carrier 1	294313	-0.14136	10.24	-0.99423	0.34507	0.50122	-6.1498
Psmc2	"proteasome 26S subunit, ATPase 2"	25581	0.085853	5.288	0.99398	0.34519	0.50122	-6.1501
Rap1b	"RAP1B, member of RAS oncogene family	171337	-0.1352	7.2854	-0.99294	0.34567	0.50149	-6.1511
Tmod3	tropomodulin 3	300838	-0.087664	8.3922	-0.99079	0.34666	0.5025	-6.1532
Tjp1	tight junction protein 1	292994	-0.102	6.683	-0.98903	0.34748	0.50326	-6.1549
Pisd	phosphatidylserine decarboxylase	681361	-0.1023	7.0236	-0.98777	0.34807	0.50368	-6.1561
Itga1	integrin subunit alpha 1	25118	-0.12102	8.2979	-0.9852	0.34926	0.5048	-6.1586
Tpd52l2	TPD52 like 2	296480	-0.092223	5.1173	-0.98483	0.34943	0.5048	-6.159
Mtor	mechanistic target of rapamycin kinase	56718	0.27746	5.7563	0.97961	0.35188	0.50725	-6.164
Myzap	myocardial zonula adherens protein	363091	0.12259	5.8987	0.97957	0.35189	0.50725	-6.1641
Atp2b4	ATPase plasma membrane Ca2+ transpor	29600	0.19746	5.1301	0.9793	0.35202	0.50725	-6.1643
Gba	glucosylceramidase beta	684536	-0.13848	6.2702	-0.97732	0.35295	0.50816	-6.1662
Mrpl3	mitochondrial ribosomal protein L3	300974	-0.10024	9.8738	-0.97415	0.35444	0.50988	-6.1693
Eif2s1	eukaryotic translation initiation factor 2 sul	54318	-0.11694	5.4777	-0.97202	0.35544	0.51089	-6.1713
Tomm40l	translocase of outer mitochondrial membra	304971	0.11495	4.323	0.96874	0.357	0.51236	-6.1745
Smpdl3b	"sphingomyelin phosphodiesterase, acid-l	362619	0.11336	3.9767	0.9686	0.35706	0.51236	-6.1746
Hexb	hexosaminidase subunit beta	294673	-0.1014	11.076	-0.96753	0.35757	0.51266	-6.1757
Grb10	growth factor receptor bound protein 10	498416	-0.32364	5.5347	-0.96537	0.35859	0.51369	-6.1777
Aimp1	aminoacyl tRNA synthetase complex-intera	114632	-0.085075	4.8428	-0.96319	0.35963	0.51475	-6.1798
Ist1	IST1 factor associated with ESCRT-III	307833	0.1443	5.7799	0.95893	0.36166	0.5172	-6.1838
Vldlr	very low density lipoprotein receptor	25696	-0.099584	4.2239	-0.95834	0.36195	0.5172	-6.1844
Hint3	histidine triad nucleotide binding protein 3	246769	-0.088181	8.1405	-0.95271	0.36465	0.52002	-6.1897
Copb2	COPI coat complex subunit beta 2	60384	0.1102	5.9722	0.95257	0.36471	0.52002	-6.1899

Gna13	G protein subunit alpha 13	303634	-0.10116	12.768	-0.95232	0.36484	0.52002	-6.1901
Mrps17	mitochondrial ribosomal protein S17	288621	0.12718	5.3048	0.95078	0.36558	0.5206	-6.1916
Dusp3	dual specificity phosphatase 3	498003	-0.1035	7.2908	-0.95021	0.36585	0.5206	-6.1921
Acot11	acyl-CoA thioesterase 11	100363074	0.088359	8.2071	0.94923	0.36633	0.52082	-6.193
Armc1	armadillo repeat containing 1	294948	0.10228	4.7379	0.94834	0.36675	0.52082	-6.1938
Prkaa2	protein kinase AMP-activated catalytic sub	78975	-0.095304	4.9428	-0.94799	0.36692	0.52082	-6.1942
Eif4e	eukaryotic translation initiation factor 4E	117045	-0.13392	8.2468	-0.94672	0.36754	0.52126	-6.1954
Mapre2	"microtubule-associated protein, RP/EB fa	679221	-0.087475	6.8851	-0.94359	0.36906	0.52297	-6.1983
Ubtd1	ubiquitin domain containing 1	309373	0.28734	7.5106	0.94298	0.36935	0.52297	-6.1989
Stt3a	STT3 oligosaccharyltransferase complex c	500972	-0.093485	6.6126	-0.9423	0.36968	0.523	-6.1995
Mipep	mitochondrial intermediate peptidase	81684	0.10976	4.1149	0.93068	0.37536	0.53059	-6.2103
Pon2	paraoxonase 2	296851	0.12987	11.519	0.92784	0.37675	0.53212	-6.213
Tcp1	t-complex 1	24818	-0.092707	5.1259	-0.92711	0.37712	0.5322	-6.2136
Tmem205	transmembrane protein 205	300441	-0.088175	4.6186	-0.92478	0.37826	0.53338	-6.2158
Gng2	G protein subunit gamma 2	80850	-0.10792	4.7834	-0.92346	0.37892	0.53386	-6.217
Mavs	mitochondrial antiviral signaling protein	311430	-0.090407	8.1218	-0.92157	0.37985	0.53474	-6.2187
Ppt1	palmitoyl-protein thioesterase 1	29411	-0.12824	4.2551	-0.92072	0.38027	0.53485	-6.2195
Agpat5	1-acylglycerol-3-phosphate O-acyltransfera	306582	0.11638	6.917	0.92014	0.38056	0.53485	-6.2201
Pptc7	PTC7 protein phosphatase homolog	304488	-0.10502	9.6761	-0.91698	0.38213	0.53662	-6.223
Psmd6	"proteasome 26S subunit, non-ATPase 6"	289924	-0.089082	8.8237	-0.91624	0.3825	0.5367	-6.2236
Tuba4a	"tubulin, alpha 4A"	316531	-0.080677	9.7153	-0.91288	0.38418	0.53766	-6.2267
Micu1	mitochondrial calcium uptake 1	365567	0.10595	8.3626	0.91253	0.38435	0.53766	-6.227
Trmt10c	"tRNA methyltransferase 10C, mitochondr	304012	0.11844	4.0954	0.91239	0.38442	0.53766	-6.2271
Mia2	MIA SH3 domain ER export factor 2	100912115	0.13266	8.3938	0.91233	0.38445	0.53766	-6.2272
Tpp2	tripeptidyl peptidase 2	81815	0.09759	7.4847	0.91129	0.38497	0.53795	-6.2281
Ubxn4	UBX domain protein 4	304766	-0.082709	7.3292	-0.90772	0.38676	0.54001	-6.2314
Rps19	ribosomal protein S19	108348115	0.079835	8.0427	0.90315	0.38906	0.54278	-6.2355
Eef1a1	eukaryotic translation elongation factor 1 a	171361	-0.089727	8.2989	-0.90103	0.39013	0.54382	-6.2374
Creld1	cysteine-rich with EGF-like domains 1	312638	0.12415	4.5086	0.89928	0.39101	0.54459	-6.239
Mrpl46	mitochondrial ribosomal protein L46	293054	-0.088543	6.6164	-0.89869	0.39131	0.54459	-6.2396
Fis1	"fission, mitochondrial 1"	288584	0.10025	8.1158	0.89535	0.393	0.54649	-6.2425
Mrpl27	mitochondrial ribosomal protein L27	287635	-0.097793	10.117	-0.89309	0.39415	0.54765	-6.2446
Bcap31	B-cell receptor-associated protein 31	293852	0.087314	6.6155	0.88898	0.39625	0.55011	-6.2482
Anp32e	acidic nuclear phosphoprotein 32 family m	361999	-0.079816	7.3451	-0.88741	0.39705	0.55077	-6.2496
Cdnf	cerebral dopamine neurotrophic factor	361276	-0.13585	7.2048	-0.88319	0.39921	0.55324	-6.2534
Minpp1	multiple inositol-polyphosphate phosphata	29688	0.088906	9.206	0.88268	0.39947	0.55324	-6.2538
Ergic1	endoplasmic reticulum-golgi intermediate c	287177	-0.11687	8.1137	-0.87842	0.40166	0.55582	-6.2576
Lum	lumican	81682	0.1045	10.684	0.87036	0.40583	0.56091	-6.2647
Cpne3	copine 3	313087	0.08856	7.9975	0.87005	0.406	0.56091	-6.265
Tgfbi	"transforming growth factor, beta induced"	116487	0.13154	6.1424	0.86776	0.40718	0.5621	-6.267

Psmd7	"proteasome 26S subunit, non-ATPase 7"	307821	-0.085357	6.1537	-0.86666	0.40776	0.56244	-6.2679
Pnkd	PNKD metallo-beta-lactamase domain con	100188944	-0.087645	8.5405	-0.86581	0.4082	0.56259	-6.2687
Plxnb1	plexin B1	316009	0.1019	4.2937	0.86471	0.40877	0.56269	-6.2696
Parva	"parvin, alpha"	57341	-0.15061	11.292	-0.86441	0.40893	0.56269	-6.2699
Mrpl55	mitochondrial ribosomal protein L55	287356	-0.10836	4.8442	-0.85756	0.41252	0.56717	-6.2758
Lamtor3	"late endosomal/lysosomal adaptor, MAPK	362045	0.088714	9.0019	0.85655	0.41305	0.56744	-6.2767
Fmo3	flavin containing dimethylaniline monoxyge	84493	-0.094926	7.1794	-0.85463	0.41406	0.56837	-6.2784
Vps4a	vacuolar protein sorting 4 homolog A	246772	0.11249	8.0159	0.85293	0.41495	0.56914	-6.2798
Atl3	atlastin GTPase 3	309187	0.093995	8.5043	0.85	0.4165	0.57081	-6.2824
Cpd	carboxypeptidase D	25306	0.10519	7.0523	0.84737	0.41789	0.57226	-6.2846
Zmpste24	zinc metallopeptidase STE24	313564	0.12051	4.8464	0.8453	0.41899	0.57292	-6.2864
Eef1g	eukaryotic translation elongation factor 1 g	293725	-0.092995	6.6999	-0.84519	0.41905	0.57292	-6.2865
Psma3	proteasome 20S subunit alpha 3	29670	0.15391	9.6402	0.84364	0.41987	0.57359	-6.2878
Ptgfrn	prostaglandin F2 receptor inhibitor	29602	0.10531	4.8549	0.83922	0.42222	0.57634	-6.2916
Capns1	"calpain, small subunit 1"	29156	0.12045	8.4874	0.83703	0.4234	0.57737	-6.2934
Emc4	ER membrane protein complex subunit 4	296049	-0.093332	12.231	-0.83654	0.42366	0.57737	-6.2938
Irgm	immunity-related GTPase M	303090	0.086317	4.1093	0.83338	0.42535	0.57922	-6.2965
Gaa	"glucosidase, alpha, acid"	367562	0.087909	8.7373	0.83151	0.42635	0.58013	-6.2981
Vps36	vacuolar protein sorting 36 homolog	290851	0.082406	5.4792	0.82902	0.42769	0.58137	-6.3002
Cryz	crystallin zeta	362061	-0.090405	4.7726	-0.82854	0.42795	0.58137	-6.3006
Fabp5	fatty acid binding protein 5	140868	-0.075999	7.0029	-0.82781	0.42834	0.58144	-6.3012
Enpp3	ectonucleotide pyrophosphatase/phospho	54410	-0.080332	6.4819	-0.82445	0.43015	0.58344	-6.304
Rpl14	ribosomal protein L14	65043	-0.084903	8.5514	-0.82257	0.43117	0.58436	-6.3056
Psma6	proteasome 20S subunit alpha 6	29673	0.092243	8.3527	0.82117	0.43193	0.5848	-6.3067
Psmc4	"proteasome 26S subunit, ATPase 4"	117262	0.13872	11.685	0.82037	0.43237	0.5848	-6.3074
Bcl2l13	BCL2 like 13	312682	0.11047	8.457	0.82007	0.43253	0.5848	-6.3077
Lars2	"leucyl-tRNA synthetase 2, mitochondrial"	363172	0.10279	7.9775	0.81663	0.4344	0.58653	-6.3105
Magmas-ps1	"mitochondria-associated protein involved	287065	0.12815	7.8347	0.81589	0.4348	0.58653	-6.3111
Cab39	calcium binding protein 39	301574	0.098915	5.0825	0.81582	0.43483	0.58653	-6.3112
Ero1a	endoplasmic reticulum oxidoreductase 1 a	171562	-0.13452	9.2717	-0.81314	0.4363	0.58804	-6.3134
Csnk1g2	"casein kinase 1, gamma 2"	65278	0.080953	6.081	0.81248	0.43665	0.58806	-6.3139
Hacd3	3-hydroxyacyl-CoA dehydratase 3	300783	0.13574	8.0291	0.80999	0.43802	0.58943	-6.316
Dctn4	dynactin subunit 4	84428	0.21593	4.9684	0.8083	0.43894	0.59021	-6.3174
Lyrm1	LYR motif containing 1	365361	0.13109	5.1449	0.80647	0.43995	0.59109	-6.3189
Mrps5	mitochondrial ribosomal protein S5	296134	-0.10703	5.8935	-0.80572	0.44036	0.59118	-6.3195
Mthfd1l	methylenetetrahydrofolate dehydrogenase	361472	-0.1061	7.4454	-0.79846	0.44435	0.59607	-6.3254
Mrpl35	mitochondrial ribosomal protein L35	297334	0.1851	7.5301	0.79544	0.44602	0.59785	-6.3279
Acadsb	"acyl-CoA dehydrogenase, short/branched	25618	0.071131	6.2011	0.79125	0.44835	0.60049	-6.3313
Dnaja4	DnaJ heat shock protein family (Hsp40) m	300721	-0.092252	7.5573	-0.79013	0.44897	0.60085	-6.3322
Ethe1	"ETHE1, persulfide dioxygenase"	292710	0.085409	5.5375	0.78355	0.45264	0.60529	-6.3375

Fitm2	fat storage-inducing transmembrane prote	311617	-0.073501	7.5856	-0.78279	0.45307	0.60539	-6.3381
Mrc2	"mannose receptor, C type 2"	498011	0.12166	4.2541	0.78102	0.45405	0.60623	-6.3395
Tagln	transgelin	25123	-0.27253	7.5441	-0.77896	0.45521	0.60724	-6.3411
Psma4	proteasome 20S subunit alpha 4	29671	-0.078672	5.4988	-0.77841	0.45552	0.60724	-6.3416
Stx4	syntaxin 4	81803	-0.071329	6.7794	-0.77171	0.45929	0.61179	-6.3469
Ptgr2	prostaglandin reductase 2	299194	0.076208	6.2755	0.77069	0.45987	0.61208	-6.3477
Cndp2	carnosine dipeptidase 2	291394	-0.082998	12.159	-0.7695	0.46054	0.61237	-6.3486
Lancl1	LanC like 1	114515	-0.083886	5.9955	-0.76904	0.4608	0.61237	-6.349
Ap1b1	adaptor related protein complex 1 subunit	29663	0.082397	5.2476	0.76585	0.46261	0.61429	-6.3515
Mpp6	membrane palmitoylated protein 6	362359	-0.097365	6.254	-0.76105	0.46533	0.61698	-6.3552
Grb2	growth factor receptor bound protein 2	81504	0.12667	7.2644	0.76102	0.46535	0.61698	-6.3552
Cyp27a1	"cytochrome P450, family 27, subfamily a,	301517	-0.080618	4.9406	-0.75818	0.46697	0.61864	-6.3575
Oxsr1	oxidative stress responsive kinase 1	316064	-0.084843	4.6723	-0.75466	0.46898	0.61982	-6.3602
Colec12	collectin sub-family member 12	361289	0.075681	6.6979	0.75435	0.46916	0.61982	-6.3604
Emc2	ER membrane protein complex subunit 2	362905	-0.072488	8.8149	-0.75431	0.46918	0.61982	-6.3605
Gpsm1	G-protein signaling modulator 1	246254	0.069487	5.1751	0.75409	0.46931	0.61982	-6.3606
Rpl3	ribosomal protein L3	300079	0.077599	10.972	0.75332	0.46975	0.61992	-6.3612
Scarb2	"scavenger receptor class B, member 2"	117106	0.085765	5.5623	0.75213	0.47043	0.62034	-6.3621
Cpox	coproporphyrinogen oxidase	304024	0.084504	6.6415	0.74672	0.47354	0.62396	-6.3663
Mcf2l	MCF.2 cell line derived transforming seque	117020	0.081284	4.0998	0.74506	0.47449	0.62474	-6.3676
Nid2	nidogen 2	302248	0.096075	8.1603	0.74359	0.47534	0.62538	-6.3687
Cops8	COP9 signalosome subunit 8	363283	0.10557	8.9988	0.74289	0.47575	0.62538	-6.3692
Bnip1	BCL2 interacting protein 1	140932	0.094608	6.9417	0.74231	0.47608	0.62538	-6.3697
Ctsc	cathepsin C	25423	-0.071686	7.0384	-0.74113	0.47677	0.6258	-6.3706
Hprt1	hypoxanthine phosphoribosyltransferase	24465	-0.078376	5.2986	-0.73919	0.47789	0.62679	-6.3721
Hmgb1	high mobility group box 1	25459	-0.06812	11.467	-0.73723	0.47902	0.6278	-6.3735
Mrpl23	mitochondrial ribosomal protein L23	64360	0.10846	5.4152	0.73643	0.47949	0.62793	-6.3741
Rps25	ribosomal protein s25	122799	-0.092478	11.085	-0.73435	0.4807	0.62903	-6.3757
Lrrc4b	leucine rich repeat containing 4B	308571	-0.094615	5.4782	-0.73278	0.48161	0.62974	-6.3769
Bphl	biphenyl hydrolase like	361239	-0.079663	9.7383	-0.72495	0.48618	0.63523	-6.3828
Mia3	MIA SH3 domain ER export factor 3	683007	-0.081057	5.2331	-0.72299	0.48733	0.63583	-6.3842
Ephx1	epoxide hydrolase 1	25315	0.083038	6.6155	0.72245	0.48765	0.63583	-6.3847
C9	complement C9	117512	-0.16864	7.3985	-0.72227	0.48775	0.63583	-6.3848
Slc25a15	solute carrier family 25 member 15	306574	-0.081025	3.9475	-0.71875	0.48982	0.63803	-6.3874
Coq7	"coenzyme Q7, hydroxylase"	25249	-0.074418	5.5422	-0.71632	0.49125	0.63941	-6.3892
Ahsa1	activator of Hsp90 ATPase activity 1	681996	0.075026	5.1739	0.71482	0.49213	0.64007	-6.3903
S100a8	S100 calcium binding protein A8	116547	0.070566	4.157	0.71376	0.49276	0.64039	-6.3911
Dlg1	discs large MAGUK scaffold protein 1	25252	-0.070413	13.013	-0.71048	0.4947	0.64213	-6.3935
Golim4	golgi integral membrane protein 4	310526	-0.067365	12.683	-0.70996	0.49501	0.64213	-6.3939
Rsu1	Ras suppressor protein 1	680419	0.15092	4.0849	0.7094	0.49534	0.64213	-6.3943

Mrpl33	mitochondrial ribosomal protein L33	100363539	0.07481	10.71	0.70848	0.49588	0.64213	-6.395
Dnajb4	DnaJ heat shock protein family (Hsp40) m	295549	-0.070745	6.1264	-0.70834	0.49597	0.64213	-6.3951
Plin5	perilipin 5	501283	0.18773	14.772	0.70721	0.49664	0.64251	-6.3959
Ca4	carbonic anhydrase 4	29242	0.07252	10.397	0.70558	0.4976	0.64316	-6.3971
Mtus1	microtubule associated scaffold protein 1	306487	-0.067294	6.6562	-0.70509	0.49789	0.64316	-6.3974
Slc25a35	"solute carrier family 25, member 35"	497933	0.34057	4.3725	0.69914	0.50144	0.64718	-6.4018
Tomm34	translocase of outer mitochondrial membra	311621	0.071425	5.0953	0.6986	0.50176	0.64718	-6.4021
Nomo1	nodal modulator 1	361578	-0.063525	6.6369	-0.69666	0.50292	0.64818	-6.4035
Hspb2	heat shock protein family B (small) membe	161476	-0.066557	8.0815	-0.69345	0.50484	0.65016	-6.4058
Adsl	adenylosuccinate lyase	315150	0.10728	7.8331	0.69038	0.50668	0.65192	-6.408
Mrpl53	mitochondrial ribosomal protein L53	362388	-0.11327	7.9651	-0.68991	0.50697	0.65192	-6.4084
Slc25a1	solute carrier family 25 member 1	29743	0.092496	9.1104	0.68821	0.50799	0.65274	-6.4096
Lims1	LIM zinc finger domain containing 1	499443	0.063634	6.157	0.68709	0.50866	0.65283	-6.4104
Tecrl	"trans-2,3-enoyl-CoA reductase-like"	364134	-0.082269	6.0079	-0.68632	0.50913	0.65283	-6.4109
Hagh	hydroxyacyl glutathione hydrolase	24439	-0.080949	9.2652	-0.68576	0.50946	0.65283	-6.4113
Capn1	calpain 1	29153	0.070348	8.7215	0.68556	0.50958	0.65283	-6.4115
Fmo1	flavin containing dimethylaniline monoxyg	25256	0.067018	7.6384	0.68347	0.51084	0.65396	-6.413
Slc28a2	solute carrier family 28 member 2	60423	0.076554	8.346	0.67286	0.51728	0.6617	-6.4204
Jak1	Janus kinase 1	84598	-0.069034	6.4751	-0.67217	0.51769	0.66173	-6.4209
Rars1	arginyl-tRNA synthetase 1	287191	0.079485	5.2676	0.66971	0.51919	0.66262	-6.4226
Ntn1	netrin 1	114523	-0.063391	8.5678	-0.66945	0.51935	0.66262	-6.4228
Acp6	"acid phosphatase 6, lysophosphatidic"	295305	0.076671	6.021	0.66912	0.51955	0.66262	-6.423
Rpl28	ribosomal protein L28	64638	0.084572	4.0993	0.66836	0.52001	0.66272	-6.4236
Cluh	clustered mitochondria homolog	303300	0.086236	9.2753	0.66441	0.52243	0.6653	-6.4263
Otub1	"OTU deubiquitinase, ubiquitin aldehyde b	293705	0.066251	7.7429	0.66084	0.52462	0.66759	-6.4288
Arf6	ADP-ribosylation factor 6	79121	-0.076964	6.8325	-0.66016	0.52503	0.66762	-6.4292
Ak1	adenylate kinase 1	24183	0.066715	7.4761	0.65893	0.52579	0.66809	-6.4301
Trim25	tripartite motif-containing 25	494338	0.065257	11.802	0.65532	0.52801	0.67041	-6.4325
Mrpl16	mitochondrial ribosomal protein L16	293754	-0.067484	10.511	-0.65429	0.52864	0.67071	-6.4332
Cox7c	cytochrome c oxidase subunit 7C	100188937	-0.10531	9.2232	-0.65277	0.52958	0.6714	-6.4343
Cdc42bpa	CDC42 binding protein kinase alpha	114116	0.065175	6.0115	0.65186	0.53014	0.67162	-6.4349
Rpl31	ribosomal protein L31	64298	-0.058565	5.5874	-0.64616	0.53367	0.67559	-6.4387
Eif2s3	eukaryotic translation initiation factor 2 su	299027	-0.07319	4.204	-0.64207	0.53621	0.6783	-6.4415
Akap12	A-kinase anchoring protein 12	83425	0.10195	5.4418	0.64133	0.53667	0.67837	-6.442
Nudt9	nudix hydrolase 9	305149	0.10082	6.3525	0.63332	0.54166	0.68364	-6.4473
Osbp	oxysterol binding protein	365410	0.076915	7.609	0.63309	0.5418	0.68364	-6.4475
Pgd	phosphogluconate dehydrogenase	100360180	0.066345	9.2193	0.63272	0.54203	0.68364	-6.4477
Pald1	"phosphatase domain containing, paladin	294508	0.073592	4.8495	0.62864	0.54459	0.68557	-6.4504
Coa7	cytochrome c oxidase assembly factor 7	298377	-0.08106	6.4606	-0.62842	0.54473	0.68557	-6.4505
Ccny	cyclin Y	361261	0.12419	11.633	0.62836	0.54477	0.68557	-6.4506

Vwf	von Willebrand factor	116669	-0.078082	7.0309	-0.62676	0.54577	0.68633	-6.4516
Sco1	synthesis of cytochrome C oxidase 1	497930	-0.055743	8.2588	-0.62521	0.54675	0.68705	-6.4526
Ttr	transthyretin	24856	0.053888	5.847	0.62337	0.5479	0.688	-6.4538
Steap4	STEAP4 metalloreductase	499991	-0.14174	4.3958	-0.62215	0.54867	0.68846	-6.4546
Steap3	STEAP3 metalloreductase	170824	0.061468	5.2763	0.61589	0.55262	0.69291	-6.4587
Chchd6	coiled-coil-helix-coiled-coil-helix domain co	297436	-0.065351	13.62	-0.61174	0.55525	0.69488	-6.4613
Dnajc28	DnaJ heat shock protein family (Hsp40) m	360699	0.060692	10.794	0.6116	0.55534	0.69488	-6.4614
Ilvbl	ilvB acetolactate synthase like	362843	0.063648	6.8846	0.61148	0.55541	0.69488	-6.4615
Pex14	peroxisomal biogenesis factor 14	64460	0.060419	7.7913	0.60906	0.55695	0.69629	-6.4631
Vps13a	vacuolar protein sorting 13 homolog A	309243	0.19161	11.718	0.60532	0.55933	0.69875	-6.4654
Gprin3	GPRIN family member 3	502784	0.054314	5.3536	0.60296	0.56083	0.70012	-6.4669
Stx5	syntaxin 5	65134	0.058015	6.0444	0.59918	0.56325	0.7026	-6.4693
Lamp2	lysosomal-associated membrane protein 2	24944	-0.066851	12.234	-0.59824	0.56385	0.7026	-6.4699
Csnk1a1	"casein kinase 1, alpha 1"	113927	0.090131	4.0878	0.59793	0.56405	0.7026	-6.4701
Slc25a10	solute carrier family 25 member 10	170943	-0.063666	5.9063	-0.59649	0.56497	0.70323	-6.471
Arih1	ariadne RBR E3 ubiquitin protein ligase 1	300756	0.079973	6.5733	0.59526	0.56576	0.7037	-6.4718
Luc7l2	LUC7-like 2 pre-mRNA splicing factor	312251	-0.13917	7.3978	-0.59315	0.56711	0.70486	-6.4731
Afg3l1	AFG3(ATPase family gene 3)-like 1 (S. ce	361436	-0.084994	5.2383	-0.59204	0.56782	0.70524	-6.4738
Cltb	"clathrin, light chain B"	116561	0.1778	6.3737	0.59122	0.56835	0.70538	-6.4743
Atp6v1e1	ATPase H+ transporting V1 subunit E1	297566	0.064184	8.4152	0.58873	0.56995	0.70686	-6.4758
Atp6v0d1	ATPase H+ transporting V0 subunit D1	291969	0.053438	9.1551	0.58749	0.57075	0.70733	-6.4766
Acsl6	acyl-CoA synthetase long-chain family me	117243	0.050849	8.1409	0.58412	0.57292	0.70951	-6.4787
Get3	"guided entry of tail-anchored proteins fac	288919	-0.081489	6.1988	-0.58348	0.57334	0.70951	-6.479
Ank2	ankyrin 2	362036	0.059275	7.4566	0.5796	0.57584	0.71176	-6.4814
Psme2	proteasome activator subunit 2	29614	-0.06778	12.362	-0.57931	0.57603	0.71176	-6.4816
Psmb5	proteasome 20S subunit beta 5	29425	-0.052831	7.8337	-0.5779	0.57694	0.71176	-6.4824
Rad23b	"RAD23 homolog B, nucleotide excision re	298012	0.10173	11.991	0.57749	0.57721	0.71176	-6.4827
Dnajc13	DnaJ heat shock protein family (Hsp40) m	363127	-0.095983	6.1763	-0.57745	0.57724	0.71176	-6.4827
Psmc6	"proteasome 26S subunit, ATPase 6"	289990	-0.062654	5.069	-0.57581	0.5783	0.71256	-6.4837
Tmx3	thioredoxin-related transmembrane protein	682967	-0.066609	3.9228	-0.57336	0.57989	0.71376	-6.4852
Tm9sf4	transmembrane 9 superfamily member 4	296279	0.060618	6.1296	0.57301	0.58012	0.71376	-6.4854
C1qb	complement C1q B chain	29687	-0.056386	4.6265	-0.56965	0.5823	0.71594	-6.4874
Tomm20	translocase of outer mitochondrial membra	266601	-0.054619	9.0008	-0.56773	0.58355	0.71669	-6.4886
Dnajc30	DnaJ heat shock protein family (Hsp40) m	368190	-0.054049	6.2588	-0.567	0.58403	0.71669	-6.489
Rab4b	"RAB4B, member RAS oncogene family"	50866	0.055351	7.6827	0.56679	0.58417	0.71669	-6.4891
Daam1	dishevelled associated activator of morph	314212	-0.065427	6.4666	-0.56472	0.58552	0.71783	-6.4904
Sdhd	succinate dehydrogenase complex subun	363061	-0.077503	9.6027	-0.56354	0.58629	0.71826	-6.4911
Pak2	p21 (RAC1) activated kinase 2	29432	0.055425	7.1279	0.56204	0.58727	0.71894	-6.492
Tpm3	tropomyosin 3	117557	-0.065741	7.6037	-0.56139	0.58769	0.71895	-6.4923
Pex3	peroxisomal biogenesis factor 3	83519	0.071141	5.799	0.5593	0.58907	0.72011	-6.4936

Tapbp	TAP binding protein	25217	0.1249	11.649	0.55827	0.58974	0.72031	-6.4942
Plxnd1	plexin D1	312652	0.055742	6.7743	0.55776	0.59007	0.72031	-6.4945
Pcyox1l	prenylcysteine oxidase 1 like	307396	-0.057885	7.1938	-0.55402	0.59253	0.72279	-6.4967
Sdhaf2	succinate dehydrogenase complex assem	361726	-0.054831	4.614	-0.55094	0.59456	0.72475	-6.4985
Sec23a	"Sec23 homolog A, COPII coat complex c	58817	-0.067801	7.6709	-0.54806	0.59646	0.72655	-6.5001
Cd200	Cd200 molecule	24560	-0.07251	7.0191	-0.54646	0.59751	0.72684	-6.5011
Fam98a	"family with sequence similarity 98, membe	313873	0.055128	6.5924	0.5464	0.59755	0.72684	-6.5011
Ubl3	ubiquitin-like 3	363869	0.058931	6.8319	0.5454	0.59821	0.72713	-6.5017
Sars2	"seryl-tRNA synthetase 2, mitochondrial"	292759	-0.05178	5.1483	-0.544	0.59914	0.72774	-6.5025
Ddah2	dimethylarginine dimethylaminohydrolase	294239	0.059	11.653	0.53883	0.60256	0.73113	-6.5054
Sec22b	"SEC22 homolog B, vesicle trafficking pro	310710	-0.056428	5.2918	-0.53849	0.60278	0.73113	-6.5056
Cops7a	COP9 signalosome subunit 7A	312710	-0.051576	8.2298	-0.53169	0.6073	0.73507	-6.5095
Eea1	early endosome antigen 1	314764	-0.053492	11.131	-0.53151	0.60742	0.73507	-6.5096
Cxadr	"CXADR, Ig-like cell adhesion molecule"	89843	-0.068616	6.9097	-0.53063	0.60801	0.73507	-6.5101
Ptrh2	peptidyl-tRNA hydrolase 2	287593	-0.050659	9.075	-0.53052	0.60808	0.73507	-6.5101
Ehd3	EH-domain containing 3	192249	0.052549	5.8491	0.53037	0.60819	0.73507	-6.5102
Neu3	neuraminidase 3	117185	0.084956	4.4834	0.5296	0.6087	0.73517	-6.5106
Ahcyl1	adenosylhomocysteinase-like 1	362013	0.15517	11.306	0.52698	0.61045	0.73676	-6.5121
C4bpa	"complement component 4 binding protei	24235	0.067081	7.5573	0.52392	0.61249	0.73851	-6.5138
Tmem182	transmembrane protein 182	501129	0.056825	6.9125	0.52352	0.61276	0.73851	-6.514
Pdgfrb	platelet derived growth factor receptor bet	24629	-0.057478	6.8808	-0.52136	0.61421	0.73973	-6.5152
Copg1	COPI coat complex subunit gamma 1	297428	-0.090978	3.7912	-0.52039	0.61486	0.73999	-6.5157
Prxl2a	peroxiredoxin like 2A	361118	-0.052424	5.1741	-0.51947	0.61547	0.74021	-6.5163
Ndufaf5	NADH:ubiquinone oxidoreductase comple	296190	0.055214	8.8979	0.51495	0.61851	0.74334	-6.5187
Skp1	S-phase kinase-associated protein 1	287280	-0.056987	4.1554	-0.51421	0.619	0.74341	-6.5191
Snx5	sorting nexin 5	296199	0.05789	7.2259	0.51338	0.61956	0.74356	-6.5196
Kyat1	kynurenine aminotransferase 1	311844	0.049083	10.63	0.51252	0.62014	0.74373	-6.52
F13a1	coagulation factor XIII A1 chain	60327	0.058008	9.3393	0.51031	0.62163	0.745	-6.5212
Mrpl40	mitochondrial ribosomal protein L40	287962	-0.054352	7.1509	-0.50867	0.62274	0.7458	-6.5221
Prelp	proline and arginine rich end leucine rich r	84400	0.05054	5.0971	0.50384	0.626	0.74918	-6.5247
Ctsa	cathepsin A	296370	0.10677	4.6545	0.49883	0.62939	0.75204	-6.5274
Syn2	synapsin II	29179	-0.054528	8.2042	-0.4987	0.62948	0.75204	-6.5275
Psmd8	"proteasome 26S subunit, non-ATPase 8"	292766	0.095182	8.4709	0.49836	0.62971	0.75204	-6.5276
Atxn10	ataxin 10	170821	-0.049458	7.2698	-0.49739	0.63037	0.7523	-6.5281
App	amyloid beta precursor protein	54226	0.063471	6.9889	0.49607	0.63126	0.75273	-6.5288
Mfge8	milk fat globule-EGF factor 8 protein	25277	-0.10726	7.3467	-0.49498	0.63201	0.75273	-6.5294
Bnip3	BCL2 interacting protein 3	84480	-0.055546	6.2994	-0.49492	0.63205	0.75273	-6.5294
Ppid	peptidylprolyl isomerase D	361967	0.052003	7.9457	0.49275	0.63352	0.75396	-6.5306
Pfkp	"phosphofructokinase, platelet"	60416	0.057859	5.5727	0.4918	0.63417	0.75421	-6.5311
Abca9	ATP binding cassette subfamily A membe	287788	-0.061284	7.6235	-0.48954	0.63571	0.75551	-6.5323

Golgb1	golgin B1	192243	0.050931	12.239	0.48745	0.63713	0.75621	-6.5333
Tmem242	transmembrane protein 242	292228	-0.049434	6.8661	-0.48739	0.63718	0.75621	-6.5334
Lrrc57	leucine rich repeat containing 57	311346	-0.079386	7.9323	-0.48383	0.63961	0.75856	-6.5352
Ralb	RAS like proto-oncogene B	116546	-0.044559	5.0264	-0.48008	0.64217	0.76108	-6.5371
Golm1	golgi membrane protein 1	680692	0.054361	4.8945	0.47391	0.64641	0.76556	-6.5402
Timm29	translocase of inner mitochondrial membra	315463	0.14846	6.5108	0.47292	0.64709	0.76575	-6.5407
Etf1	eukaryotic translation termination factor 1	307503	-0.044572	8.2632	-0.47203	0.6477	0.76575	-6.5412
Gbf1	golgi brefeldin A resistant guanine nucleo	309451	0.055773	8.581	0.47173	0.6479	0.76575	-6.5413
Rmnd1	required for meiotic nuclear division 1 hom	292268	-0.059037	9.0087	-0.47104	0.64838	0.76578	-6.5417
Pip4p2	"phosphatidylinositol-4,5-bisphosphate 4-	362490	0.04404	5.9752	0.46842	0.65018	0.76738	-6.543
Dctn2	dynactin subunit 2	299850	-0.051079	4.6674	-0.46653	0.65149	0.76839	-6.5439
Dhrs7b	dehydrogenase/reductase 7B	287380	-0.057559	9.9108	-0.46249	0.65427	0.77114	-6.5459
Pf4	platelet factor 4	360918	0.042727	5.2825	0.46148	0.65497	0.77143	-6.5464
Eif3a	"eukaryotic translation initiation factor 3, s	292148	0.043142	5.8686	0.4602	0.65586	0.77194	-6.5471
Pgm2	phosphoglucomutase 2	289632	0.098498	11.527	0.45953	0.65633	0.77196	-6.5474
Lclat1	lysocardiolipin acyltransferase 1	362702	0.042205	5.4025	0.45836	0.65713	0.77211	-6.548
Coa6	cytochrome c oxidase assembly factor 6	102556031	0.074046	8.7489	0.4579	0.65745	0.77211	-6.5482
Golga4	golgin A4	501069	0.079917	6.0902	0.45739	0.65781	0.77211	-6.5484
Galnt2	polypeptide N-acetylgalactosaminyltransfe	292090	-0.069317	7.3394	-0.45654	0.6584	0.77227	-6.5488
Wars1	tryptophanyl-tRNA synthetase 1	314442	0.056429	5.213	0.45542	0.65917	0.77264	-6.5494
Slc3a2	solute carrier family 3 member 2	50567	-0.049212	6.4271	-0.45433	0.65993	0.77295	-6.5499
Plod3	"procollagen-lysine, 2-oxoglutarate 5-diox	288583	0.08044	5.8876	0.45374	0.66034	0.77295	-6.5502
Mrpl42	mitochondrial ribosomal protein L42	299743	-0.051247	4.6165	-0.45149	0.6619	0.77425	-6.5513
Rpl26	ribosomal protein L26	287417	0.047303	5.1937	0.45078	0.66239	0.7743	-6.5516
Pde2a	phosphodiesterase 2A	81743	0.049264	5.6876	0.44913	0.66354	0.77468	-6.5524
Kras	"KRAS proto-oncogene, GTPase"	24525	0.045925	6.6453	0.44901	0.66362	0.77468	-6.5525
Prnp	prion protein	24686	0.045965	4.5296	0.4479	0.6644	0.77505	-6.553
Cdc42bpb	CDC42 binding protein kinase beta	113960	0.052142	10.106	0.44292	0.66787	0.77857	-6.5554
Cd59	CD59 molecule	25407	-0.050246	5.6167	-0.4413	0.669	0.77936	-6.5561
Gna12	G protein subunit alpha 12	81663	0.049813	8.5065	0.43987	0.66999	0.77999	-6.5568
Ptprc	"protein tyrosine phosphatase, receptor ty	24699	0.050262	6.2705	0.43647	0.67237	0.78222	-6.5584
Rps20	ribosomal protein S20	122772	-0.040316	7.3673	-0.43431	0.67388	0.78334	-6.5594
Ptgr1	prostaglandin reductase 1	192227	-0.05831	8.8739	-0.43379	0.67425	0.78334	-6.5596
Pcyox1	prenylcysteine oxidase 1	246302	-0.042327	12.678	-0.42986	0.67701	0.78506	-6.5615
Ada	adenosine deaminase	24165	0.062839	9.6504	0.42986	0.67701	0.78506	-6.5615
Fkbp11	FKBP prolyl isomerase 11	300211	0.047217	4.8573	0.42972	0.67711	0.78506	-6.5615
Timm22	translocase of inner mitochondrial membra	79463	-0.042926	3.7628	-0.42697	0.67904	0.78676	-6.5628
Fam20b	"FAM20B, glycosaminoglycan xylosylkinas	304885	-0.072083	7.3476	-0.42482	0.68055	0.78798	-6.5638
Ptprm	"protein tyrosine phosphatase, receptor ty	29616	-0.041156	4.8538	-0.42131	0.68303	0.79031	-6.5653
Lmna	lamin A/C	60374	-0.03913	6.4521	-0.42063	0.6835	0.79033	-6.5656

Tmed5	transmembrane p24 trafficking protein 5	289883	0.038861	10.461	0.41625	0.68659	0.79289	-6.5676
Pdlim5	PDZ and LIM domain 5	64353	-0.051821	8.496	-0.41618	0.68665	0.79289	-6.5676
Sec61a2	Sec61 translocon alpha 2 subunit	361273	0.1483	6.4263	0.41474	0.68766	0.79352	-6.5683
Ahcy	adenosylhomocysteinase	29443	0.038	5.1077	0.41394	0.68823	0.79364	-6.5686
Cd300lg	Cd300 molecule-like family member G	684984	0.043214	10.333	0.41267	0.68913	0.79414	-6.5692
Myadm	myeloid-associated differentiation marker	369016	-0.041457	11.567	-0.40875	0.6919	0.79655	-6.5709
Coq4	coenzyme Q4	366013	0.071518	8.8235	0.40819	0.6923	0.79655	-6.5712
Aars2	"alanyl-tRNA synthetase 2, mitochondrial"	301254	0.037677	5.3467	0.40737	0.69288	0.79655	-6.5715
Rpl38	ribosomal protein L38	689284	-0.040701	5.5923	-0.40709	0.69308	0.79655	-6.5716
Rps23	ribosomal protein S23	124323	0.058547	4.5298	0.40446	0.69494	0.79816	-6.5728
Clec10a	C-type lectin domain containing 10A	64195	0.048005	6.6879	0.4017	0.69691	0.79988	-6.574
Pcmt1	protein-L-isoaspartate (D-aspartate) O-me	25604	-0.03843	9.0755	-0.40023	0.69795	0.80012	-6.5746
Psmc3	"proteasome 26S subunit, ATPase 3"	29677	-0.051549	8.1292	-0.3989	0.6989	0.80012	-6.5752
Coro6	coronin 6	245982	0.047872	5.0722	0.39844	0.69923	0.80012	-6.5754
Adgrl2	adhesion G protein-coupled receptor L2	171447	0.04033	5.5123	0.39751	0.69989	0.80012	-6.5758
Dmtn	dematin actin binding protein	361069	0.052487	5.1001	0.3975	0.6999	0.80012	-6.5758
Eif3j	"eukaryotic translation initiation factor 3, s	691947	-0.036019	5.5672	-0.39745	0.69993	0.80012	-6.5758
Triobp	TRIO and F-actin binding protein	362956	0.04335	4.9395	0.39448	0.70205	0.802	-6.577
Rab9a	"RAB9A, member RAS oncogene family"	84589	0.08078	12.519	0.39253	0.70344	0.80306	-6.5779
Mrpl28	mitochondrial ribosomal protein L28	497876	-0.042222	7.634	-0.39182	0.70395	0.8031	-6.5782
Ogn	osteoglycin	291015	-0.072609	6.7732	-0.39065	0.70479	0.80352	-6.5787
Vps26a	VPS26 retromer complex component A	361846	0.035495	7.1095	0.38878	0.70612	0.80412	-6.5794
Mrps10	mitochondrial ribosomal protein S10	363187	-0.039693	4.7978	-0.38838	0.7064	0.80412	-6.5796
Fkbp4	FKBP prolyl isomerase 4	260321	-0.051297	11.019	-0.38794	0.70672	0.80412	-6.5798
Tmed2	transmembrane p24 trafficking protein 2	65165	-0.048008	7.4418	-0.38308	0.7102	0.80712	-6.5818
Psmb1	proteasome 20S subunit beta 1	94198	-0.039028	7.7765	-0.38293	0.71031	0.80712	-6.5819
Nmt1	N-myristoyltransferase 1	259274	-0.044468	9.8134	-0.37951	0.71276	0.80938	-6.5833
LOC1009111	lymphocyte antigen 6B-like	100911104	0.085293	9.7679	0.37812	0.71376	0.80997	-6.5838
Tmem43	transmembrane protein 43	362401	-0.043655	5.391	-0.376	0.71529	0.81112	-6.5847
Mrps15	mitochondrial ribosomal protein S15	298517	-0.040042	5.1886	-0.37539	0.71572	0.81112	-6.5849
Rcn3	reticulocalbin 3	494125	-0.039897	9.1214	-0.37454	0.71633	0.81128	-6.5853
Taz	tafazzin	363521	-0.035794	5.6409	-0.36442	0.72363	0.819	-6.5893
Cul4a	cullin 4A	361181	0.049573	6.2538	0.36322	0.72449	0.81943	-6.5897
Fxr1	FMR1 autosomal homolog 1	361927	-0.039681	6.02	-0.35938	0.72727	0.82202	-6.5912
Icam1	intercellular adhesion molecule 1	25464	0.031505	7.6962	0.35814	0.72817	0.8225	-6.5917
Rpl35a	ribosomal protein L35a	100359498	-0.045886	9.992	-0.35707	0.72894	0.82283	-6.5921
Hbs1l	HBS1-like translational GTPase	293408	-0.038277	11.37	-0.34829	0.73531	0.82947	-6.5954
Slc25a21	solute carrier family 25 member 21	171151	-0.04134	6.9977	-0.34724	0.73607	0.82978	-6.5958
Coro1b	coronin 1B	29474	-0.038447	5.9287	-0.34563	0.73725	0.83035	-6.5964
Wasf2	WASP family member 2	313024	-0.03614	5.1766	-0.34431	0.73821	0.83035	-6.5969

Glo1	glyoxalase 1	294320	-0.035524	4.6977	-0.34384	0.73855	0.83035	-6.5971
Cnpy2	canopy FGF signaling regulator 2	685814	-0.036195	6.0971	-0.34339	0.73888	0.83035	-6.5973
Usp24	ubiquitin specific peptidase 24	313427	-0.038598	7.7688	-0.34229	0.73968	0.83035	-6.5977
Fcer1g	Fc fragment of IgE receptor Ig	25441	0.045975	5.6984	0.34206	0.73985	0.83035	-6.5978
Mrpl43	mitochondrial ribosomal protein L43	309440	-0.04666	8.4255	-0.34155	0.74022	0.83035	-6.5979
Vps45	vacuolar protein sorting 45 homolog	64516	0.036037	7.504	0.34058	0.74092	0.83035	-6.5983
Vti1b	vesicle transport through interaction with t	100359512	-0.036123	5.1804	-0.34055	0.74095	0.83035	-6.5983
Tpd52l1	TPD52 like 1	689256	0.03187	7.0409	0.33719	0.7434	0.83254	-6.5995
Nppa	natriuretic peptide A	24602	0.033022	4.4605	0.33524	0.74482	0.83342	-6.6002
Lyrm9	LYR motif containing 9	497962	-0.041211	10.624	-0.3336	0.74602	0.83342	-6.6008
Plaa	"phospholipase A2, activating protein"	116645	0.032035	8.9934	0.33351	0.74608	0.83342	-6.6009
RT1-Ba	"RT1 class II, locus Ba"	309621	0.034011	6.0638	0.33345	0.74613	0.83342	-6.6009
Tapt1	transmembrane anterior posterior transfor	305386	-0.034419	9.3976	-0.3319	0.74726	0.83408	-6.6014
Sec11a	"SEC11 homolog A, signal peptidase com	65166	0.036571	6.4571	0.33132	0.74769	0.83408	-6.6016
Dnajc5	DnaJ heat shock protein family (Hsp40) m	79130	-0.032599	7.9552	-0.32783	0.75024	0.83638	-6.6029
Atp5if1	ATP synthase inhibitory factor subunit 1	25392	0.069115	6.5622	0.32511	0.75224	0.83805	-6.6038
Eepd1	endonuclease/exonuclease/phosphatase	315500	-0.041074	7.1083	-0.31645	0.75859	0.84458	-6.6068
Slirp	SRA stem-loop interacting RNA binding pr	688717	-0.032007	4.9812	-0.31555	0.75925	0.84477	-6.6071
Ptges3	prostaglandin E synthase 3	362809	-0.031914	14.656	-0.31344	0.76081	0.84508	-6.6079
Txndc12	thioredoxin domain containing 12	298370	-0.03207	11.316	-0.31321	0.76098	0.84508	-6.6079
Entpd2	ectonucleoside triphosphate diphosphohy	64467	-0.034646	7.1407	-0.31316	0.76101	0.84508	-6.6079
Mrpl4	mitochondrial ribosomal protein L4	363023	-0.033681	8.2033	-0.31073	0.7628	0.84651	-6.6088
Serpina6	serpin family A member 6	299270	0.03016	14.808	0.30863	0.76435	0.84731	-6.6095
Dcxr	dicarbonyl and L-xylulose reductase	171408	0.028339	7.0851	0.30796	0.76484	0.84731	-6.6097
Rps18	ribosomal protein S18	294282	-0.029588	7.55	-0.30759	0.76512	0.84731	-6.6098
Efnb3	ephrin B3	360546	-0.033523	10.834	-0.30707	0.7655	0.84731	-6.61
Ufl1	Ufm1-specific ligase 1	313115	-0.028035	7.865	-0.30354	0.76811	0.84913	-6.6111
Parp3	"poly (ADP-ribose) polymerase family, mer	300985	0.029309	6.6964	0.30324	0.76833	0.84913	-6.6112
Mrpl14	mitochondrial ribosomal protein L14	301250	-0.030585	10.871	-0.30281	0.76864	0.84913	-6.6114
Slc25a29	solute carrier family 25 member 29	314441	0.027825	4.8565	0.29732	0.7727	0.85258	-6.6132
Atp13a1	ATPase 13A1	290673	0.046353	5.4717	0.29723	0.77277	0.85258	-6.6132
Ifi47	interferon gamma inducible protein 47	246208	0.040998	7.5133	0.2963	0.77346	0.85258	-6.6135
Rps9	ribosomal protein S9	81772	-0.034885	8.3584	-0.2959	0.77376	0.85258	-6.6136
Nampt	nicotinamide phosphoribosyltransferase	297508	-0.026862	6.7271	-0.29465	0.77468	0.85305	-6.614
Fermt3	fermitin family member 3	309186	-0.035383	6.8686	-0.2927	0.77612	0.85408	-6.6146
Lbp	lipopolysaccharide binding protein	29469	0.033692	5.7707	0.29191	0.77671	0.85408	-6.6149
Ermp1	endoplasmic reticulum metallopeptidase 1	373544	0.02679	10.342	0.29136	0.77712	0.85408	-6.6151
Rnh1	ribonuclease/angiogenin inhibitor 1	100360501	-0.03939	5.8981	-0.28889	0.77895	0.85555	-6.6158
Iscu	iron-sulfur cluster assembly enzyme	288740	0.032173	7.9457	0.28724	0.78018	0.85634	-6.6163
Sars1	seryl-tRNA synthetase 1	266975	0.034913	6.484	0.2855	0.78147	0.8572	-6.6169

Uqcc3	ubiquinol-cytochrome c reductase comple:	690344	0.030179	7.0367	0.27528	0.78907	0.86499	-6.62
Mff	mitochondrial fission factor	301563	-0.027442	7.0754	-0.2731	0.7907	0.86597	-6.6206
Fastkd2	FAST kinase domains 2	301463	-0.027093	5.5442	-0.27273	0.79097	0.86597	-6.6207
Naxd	NAD(P)HX dehydratase	361185	0.026461	10.975	0.27033	0.79276	0.86737	-6.6214
Emilin1	elastin microfibril interfacer 1	298845	-0.030085	6.6473	-0.26643	0.79568	0.87	-6.6226
Dad1	defender against cell death 1	192275	0.043511	7.8343	0.26334	0.79799	0.87197	-6.6234
Smim8	small integral membrane protein 8	297971	0.043902	7.4449	0.2569	0.8028	0.87667	-6.6253
Abhd10	abhydrolase domain containing 10	303953	-0.025263	9.6863	-0.25508	0.80417	0.8776	-6.6258
Mcur1	mitochondrial calcium uniporter regulator 1	291034	-0.029435	8.5535	-0.25301	0.80572	0.87871	-6.6263
Scpep1	serine carboxypeptidase 1	114861	-0.023203	8.4754	-0.25236	0.80621	0.87871	-6.6265
Lgals3	galectin 3	83781	-0.027993	9.5458	-0.24895	0.80877	0.88076	-6.6274
Appl1	"adaptor protein, phosphotyrosine interac	290537	-0.049426	10.861	-0.24847	0.80913	0.88076	-6.6276
Man2a1	"mannosidase, alpha, class 2A, member 1	25478	0.023613	7.7775	0.24547	0.81138	0.88254	-6.6284
Usp14	ubiquitin specific peptidase 14	291796	0.021453	8.6621	0.24485	0.81185	0.88254	-6.6285
Aak1	AP2 associated kinase 1	500244	0.031308	6.4771	0.24424	0.81231	0.88254	-6.6287
Chp1	calcineurin-like EF-hand protein 1	64152	-0.023627	5.0942	-0.24039	0.8152	0.88512	-6.6297
Eml1	EMAP like 1	362783	-0.027825	6.6845	-0.23963	0.81578	0.88518	-6.6299
Rftn1	raftlin lipid raft linker 1	501095	-0.023453	11.039	-0.23763	0.81728	0.88589	-6.6304
Ctsl	cathepsin L	25697	-0.024814	8.1729	-0.23738	0.81747	0.88589	-6.6305
Tap1	"transporter 1, ATP binding cassette subfa	24811	-0.023904	6.1558	-0.2354	0.81896	0.88691	-6.631
Siglec1	sialic acid binding Ig like lectin 1	311426	-0.023475	4.3886	-0.23476	0.81944	0.88691	-6.6311
Dock9	dedicator of cytokinesis 9	259237	-0.022986	6.5897	-0.23322	0.8206	0.8876	-6.6315
Mgat2	"alpha-1,6-mannosyl-glycoprotein 2-beta-N	94273	-0.026638	6.8286	-0.23055	0.82262	0.88922	-6.6322
Emb	embigin	114511	-0.021917	4.9534	-0.22559	0.82636	0.89168	-6.6334
Rnpep	arginyl aminopeptidase	81761	-0.025471	5.9299	-0.22543	0.82648	0.89168	-6.6335
Mrpl48	mitochondrial ribosomal protein L48	293149	-0.023205	4.0053	-0.22483	0.82693	0.89168	-6.6336
Col24a1	collagen type XXIV alpha 1 chain	499723	0.020456	7.7684	0.22477	0.82698	0.89168	-6.6336
Tsfm	"Ts translation elongation factor, mitochor	679068	-0.023759	10.221	-0.22392	0.82762	0.8918	-6.6338
Sirt2	sirtuin 2	361532	-0.021355	7.1999	-0.22285	0.82843	0.89181	-6.6341
Deptor	DEP domain containing MTOR-interacting	314979	-0.029285	4.5031	-0.22199	0.82908	0.89181	-6.6343
Gmps	guanine monophosphate synthase	295088	0.025005	5.7462	0.22172	0.82929	0.89181	-6.6344
Sfxn1	sideroflexin 1	364678	-0.020453	6.0261	-0.22115	0.82971	0.89181	-6.6345
Crip1	cysteine rich protein 1	691657	0.031186	5.1525	0.22036	0.83031	0.89189	-6.6347
Acox3	"acyl-CoA oxidase 3, pristanoyl"	83522	0.028171	6.8129	0.21797	0.83212	0.89328	-6.6353
Smc1a	structural maintenance of chromosomes 1	63996	0.0263	6.8486	0.21649	0.83324	0.8935	-6.6356
Ephb4	EPH receptor B4	686310	0.045933	12.585	0.21632	0.83337	0.8935	-6.6356
Mrpl22	mitochondrial ribosomal protein L22	287302	-0.021203	7.1067	-0.21156	0.83698	0.89647	-6.6368
Ufsp2	UFM1-specific peptidase 2	361151	-0.021665	10.636	-0.21109	0.83734	0.89647	-6.6369
Plscr3	phospholipid scramblase 3	360549	-0.029207	8.8732	-0.21058	0.83772	0.89647	-6.637
Mapkapk3	MAPK activated protein kinase 3	315994	0.019538	6.2002	0.20973	0.83837	0.8966	-6.6372

Apoa4	apolipoprotein A4	25080	0.04261	4.5859	0.20898	0.83893	0.89665	-6.6373
Psmb6	proteasome 20S subunit beta 6	29666	0.031067	6.0761	0.20491	0.84202	0.89938	-6.6383
Lxn	latexin	59073	-0.024333	4.5207	-0.20414	0.84261	0.89945	-6.6384
Mrps24	mitochondrial ribosomal protein S24	498406	0.026428	5.3893	0.20244	0.8439	0.89991	-6.6388
Cnn3	calponin 3	54321	0.020659	8.0219	0.20218	0.8441	0.89991	-6.6389
Slc4a11	solute carrier family 4 member 11	311423	0.03203	5.5286	0.20025	0.84556	0.90091	-6.6393
Rab13	"RAB13, member RAS oncogene family"	81756	0.018102	7.2195	0.19774	0.84747	0.90184	-6.6398
Letmd1	LETM1 domain containing 1	681352	0.019743	5.2172	0.19727	0.84783	0.90184	-6.6399
Slc25a40	"solute carrier family 25, member 40"	296813	-0.028068	4.7635	-0.19644	0.84846	0.90184	-6.6401
L1cam	L1 cell adhesion molecule	50687	0.020015	7.835	0.19633	0.84854	0.90184	-6.6401
Cops2	COP9 signalosome subunit 2	261736	-0.03847	6.7138	-0.19556	0.84912	0.9019	-6.6403
Itga9	integrin subunit alpha 9	685004	0.021453	5.3302	0.18991	0.85343	0.90575	-6.6415
Tmem109	transmembrane protein 109	361732	-0.02404	6.8011	-0.18941	0.85381	0.90575	-6.6416
Cst3	cystatin C	25307	-0.024374	6.6068	-0.18615	0.85629	0.90782	-6.6422
Thnsl1	threonine synthase-like 1	498805	-0.02212	6.6808	-0.18224	0.85927	0.91042	-6.643
Lamtor2	"late endosomal/lysosomal adaptor, MAPI	295234	-0.051239	4.4721	-0.18096	0.86025	0.91088	-6.6433
Rab3d	"RAB3D, member RAS oncogene family"	140665	0.017909	7.5453	0.18009	0.86091	0.91102	-6.6434
Coa8	cytochrome c oxidase assembly factor 8	299341	0.036596	6.4306	0.17867	0.862	0.91161	-6.6437
Sirpa	signal-regulatory protein alpha	25528	-0.016531	7.3852	-0.17686	0.86338	0.9125	-6.6441
Metap2	methionyl aminopeptidase 2	64370	-0.026918	8.069	-0.17554	0.86439	0.91286	-6.6443
Rpl36	ribosomal protein L36	58927	0.023465	6.6177	0.17502	0.86479	0.91286	-6.6444
Snx6	sorting nexin 6	362738	-0.019722	4.6997	-0.17379	0.86572	0.91329	-6.6446
Lrrc8a	leucine rich repeat containing 8 VRAC sul	311846	0.016027	6.9653	0.17219	0.86695	0.91365	-6.6449
Ddx5	DEAD-box helicase 5	287765	0.018582	7.5262	0.16938	0.86909	0.91365	-6.6455
Hacd1	3-hydroxyacyl-CoA dehydratase 1	680115	-0.02101	5.4793	-0.16926	0.86918	0.91365	-6.6455
Ppm1f	"protein phosphatase, Mg2+/Mn2+ depen	287931	0.02312	7.3636	0.1691	0.86931	0.91365	-6.6455
Rps13	ribosomal protein S13	161477	-0.017487	9.5979	-0.16907	0.86933	0.91365	-6.6455
Esd	esterase D	290401	-0.018486	5.8684	-0.16875	0.86957	0.91365	-6.6456
Rock1	Rho-associated coiled-coil containing prot	81762	-0.017166	5.2791	-0.16844	0.86982	0.91365	-6.6456
Oxa1l	"OXA1L, mitochondrial inner membrane p	691393	-0.015412	4.7247	-0.16753	0.87051	0.91382	-6.6458
Eif4g2	"eukaryotic translation initiation factor 4, g	361628	0.023503	7.552	0.16378	0.87338	0.91627	-6.6465
Cand2	cullin-associated and neddylation-dissocia	192226	-0.022089	8.0526	-0.1626	0.87428	0.91647	-6.6467
Mrpl58	mitochondrial ribosomal protein L58	303673	-0.016028	6.4118	-0.16207	0.87469	0.91647	-6.6468
Prkcd	"protein kinase C, delta"	170538	0.017575	6.8265	0.1609	0.87558	0.91647	-6.647
Dnajb6	DnaJ heat shock protein family (Hsp40) m	362293	0.015187	6.8372	0.16073	0.87571	0.91647	-6.647
Tbl2	transducin (beta)-like 2	686610	0.015599	7.832	0.15613	0.87924	0.91959	-6.6478
Adprhl1	ADP-ribosylhydrolase like 1	290880	-0.014613	5.7957	-0.15408	0.88081	0.92055	-6.6482
Camk2g	calcium/calmodulin-dependent protein kin	171140	-0.016206	7.9029	-0.15353	0.88123	0.92055	-6.6482
Man1c1	"mannosidase, alpha, class 1C, member 1	362625	-0.017017	13.683	-0.15215	0.88229	0.92064	-6.6485
Tgm1	transglutaminase 1	60335	0.021553	5.8534	0.15202	0.88239	0.92064	-6.6485

Acad11	"acyl-CoA dehydrogenase family, member	315973	-0.024352	8.3087	-0.14944	0.88437	0.92214	-6.6489
Cers2	ceramide synthase 2	310667	0.015378	5.4998	0.14724	0.88606	0.92327	-6.6493
Nars2	"asparaginyl-tRNA synthetase 2, mitochon	293128	-0.016525	4.221	-0.14662	0.88654	0.92327	-6.6494
Dnajc16	DnaJ heat shock protein family (Hsp40) m	362652	-0.01336	6.6419	-0.14397	0.88857	0.92454	-6.6498
Tns2	tensin 2	315326	0.015803	5.6698	0.14363	0.88883	0.92454	-6.6498
Hspb7	heat shock protein family B (small) membe	50565	-0.017166	6.8094	-0.14225	0.88989	0.92508	-6.6501
Them4	thioesterase superfamily member 4	361992	-0.015363	6.6339	-0.14122	0.89068	0.92533	-6.6502
Dnajc15	DnaJ heat shock protein family (Hsp40) m	290370	-0.019039	9.2278	-0.14034	0.89136	0.92548	-6.6503
Lonp2	"lon peptidase 2, peroxisomal"	291922	0.026168	9.9018	0.13839	0.89286	0.92647	-6.6506
Mblac2	metallo-beta-lactamase domain containing	365627	0.01426	10.735	0.13749	0.89355	0.92663	-6.6508
Pgam5	"PGAM family member 5, mitochondrial se	288731	0.012302	5.2044	0.13064	0.89882	0.93153	-6.6518
Ece1	endothelin converting enzyme 1	94204	0.013136	9.5142	0.12697	0.90164	0.93388	-6.6523
Cfb	complement factor B	294257	0.012535	5.809	0.12488	0.90325	0.93394	-6.6526
Nucb2	nucleobindin 2	59295	0.012737	5.3312	0.12486	0.90327	0.93394	-6.6526
Mgat1	"alpha-1,3-mannosyl-glycoprotein 2-beta-N	81519	0.013989	5.0769	0.12461	0.90347	0.93394	-6.6526
Tnni3k	TNNI3 interacting kinase	295531	-0.013594	4.322	-0.12407	0.90388	0.93394	-6.6527
Dab2	DAB adaptor protein 2	79128	0.013213	5.3831	0.12272	0.90492	0.93444	-6.6529
Tmem177	transmembrane protein 177	304735	0.010922	11.155	0.1195	0.9074	0.93644	-6.6533
Ccz1b	"CCZ1 homolog B, vacuolar protein traffic	360768	0.017329	6.4862	0.11747	0.90897	0.93749	-6.6536
Dync1li2	"dynein, cytoplasmic 1 light intermediate c	81655	-0.013641	5.8296	-0.11673	0.90953	0.93751	-6.6536
Akap1	A-kinase anchoring protein 1	114124	-0.011714	5.9652	-0.11249	0.9128	0.94031	-6.6542
Rtn2	reticulon 2	308410	-0.024586	6.3127	-0.11138	0.91367	0.94064	-6.6543
Ap2s1	adaptor related protein complex 2 subunit	65046	-0.012046	3.9351	-0.10561	0.91812	0.94465	-6.655
Slc9a1	solute carrier family 9 member A1	24782	0.010463	4.7206	0.10154	0.92126	0.94731	-6.6555
Psma2	proteasome 20S subunit alpha 2	29669	0.0098897	12.555	0.09748	0.9244	0.94997	-6.6559
Spcs3	signal peptidase complex subunit 3	680782	-0.026721	5.4348	-0.09363	0.92737	0.95245	-6.6563
Tmem30a	transmembrane protein 30A	300857	-0.0086919	5.8119	-0.086723	0.93272	0.95736	-6.657
Efhd2	"EF-hand domain family, member D2"	298609	0.0093835	5.9759	0.080944	0.93719	0.96092	-6.6575
Abcc4	ATP binding cassette subfamily C membe	170924	0.00854	7.8933	0.080787	0.93731	0.96092	-6.6575
Arhgef1	Rho guanine nucleotide exchange factor	60323	-0.010565	6.3561	-0.079212	0.93853	0.9616	-6.6576
Hpcal1	hippocalcin-like 1	50871	-0.0082648	7.9335	-0.078449	0.93912	0.96163	-6.6577
Pck2	phosphoenolpyruvate carboxykinase 2 (m	361042	0.0072935	6.4356	0.076159	0.94089	0.96287	-6.6579
Cox16	cytochrome c oxidase assembly factor CO	100302447	0.0081601	7.8118	0.07467	0.94205	0.96347	-6.658
Chrm2	"cholinergic receptor, muscarinic 2"	81645	-0.0067658	12.436	-0.073444	0.943	0.96384	-6.6581
Ndrg1	N-myc downstream regulated 1	299923	-0.0083779	11.68	-0.072743	0.94354	0.96384	-6.6582
Apeh	acylaminoacyl-peptide hydrolase	24206	-0.0089489	7.148	-0.071943	0.94416	0.9639	-6.6582
Tmem33	transmembrane protein 33	59303	0.0077076	7.0021	0.069785	0.94583	0.9649	-6.6584
Dst	dystonin	316313	0.0088361	8.6336	0.068749	0.94663	0.9649	-6.6585
Lmf1	lipase maturation factor 1	360495	0.0095529	6.8843	0.068493	0.94683	0.9649	-6.6585
Kank2	KN motif and ankyrin repeat domains 2	100361376	-0.0061823	5.8482	-0.066136	0.94866	0.96618	-6.6587

Numbl	"NUMB-like, endocytic adaptor protein"	292732	0.0066142	8.9062	0.061299	0.95241	0.96911	-6.659
Hint1	histidine triad nucleotide binding protein 1	690660	-0.0080558	7.985	-0.060963	0.95267	0.96911	-6.659
Plpp7	phospholipid phosphatase 7 (inactive)	296635	-0.0053635	5.2494	-0.058635	0.95447	0.97037	-6.6592
Serpinf1	serpin family F member 1	287526	-0.0053726	8.1233	-0.052591	0.95916	0.97417	-6.6596
Stt3b	STT3 oligosaccharyltransferase complex c	363160	-0.0059176	8.6438	-0.052076	0.95956	0.97417	-6.6596
Tjp2	tight junction protein 2	115769	0.0055644	9.1053	0.051611	0.95992	0.97417	-6.6596
Elmo1	engulfment and cell motility 1	361251	0.0049772	6.9417	0.050471	0.96081	0.97449	-6.6597
Ssbp1	single stranded DNA binding protein 1	54304	-0.0050719	6.5757	-0.046992	0.96351	0.97665	-6.6599
Exoc7	exocyst complex component 7	64632	-0.0049586	9.5088	-0.045931	0.96433	0.9769	-6.6599
Ap3m1	adaptor related protein complex 3 subunit	171126	-0.0046556	4.6069	-0.044459	0.96547	0.97748	-6.66
Fgf1	fibroblast growth factor 1	25317	0.0053883	6.7793	0.04269	0.96684	0.97829	-6.6601
Eif2a	eukaryotic translation initiation factor 2A	502531	-0.0059308	6.6607	-0.041753	0.96757	0.97845	-6.6601
Impa1	inositol monophosphatase 1	83523	0.0039681	11.951	0.040605	0.96846	0.97866	-6.6602
Csnk2a1	casein kinase 2 alpha 1	116549	-0.0044186	4.343	-0.040013	0.96892	0.97866	-6.6602
Guf1	"GUF1 homolog, GTPase"	305317	0.0036733	5.7081	0.038287	0.97026	0.97943	-6.6603
Mylk	myosin light chain kinase	288057	-0.0035552	8.8426	-0.036838	0.97139	0.97999	-6.6603
Ppp2r5a	"protein phosphatase 2, regulatory subun	312754	0.0072856	5.5886	0.032489	0.97476	0.98281	-6.6605
Iqgap2	IQ motif containing GTPase activating pro	100360623	0.0028797	4.71	0.02788	0.97834	0.98584	-6.6606
Dpp7	dipeptidylpeptidase 7	83799	-0.0021726	11.218	-0.02225	0.98272	0.98966	-6.6608
Sh3gl2	"SH3 domain containing GRB2 like 2, end	116743	0.002616	7.491	0.02087	0.98379	0.99	-6.6608
Mrpl2	mitochondrial ribosomal protein L2	301240	-0.002752	7.6964	-0.01972	0.98468	0.99	-6.6608
Mpv17	mitochondrial inner membrane protein MP	360463	0.0018249	4.4303	0.019582	0.98479	0.99	-6.6609
Pfdn6	prefoldin subunit 6	309629	0.001056	6.001	0.0096739	0.99248	0.99715	-6.661
Mlip	muscular LMNA-interacting protein	681849	0.00082056	6.0916	0.0087416	0.99321	0.99729	-6.661
Ddb1	damage-specific DNA binding protein 1	64470	0.00089754	4.519	0.0075005	0.99417	0.99768	-6.661
Mapk1	mitogen activated protein kinase 1	116590	0.00059358	5.8117	0.0055302	0.9957	0.99863	-6.661
Man1a1	"mannosidase, alpha, class 1A, member 1	294410	0.00051906	4.4297	0.0040388	0.99686	0.99869	-6.6611
Ubr4	ubiquitin protein ligase E3 component n-re	313658	0.00040788	6.3229	0.0039418	0.99694	0.99869	-6.6611
Comt	catechol-O-methyltransferase	24267	-0.000256	6.7352	-0.0019766	0.99846	0.99918	-6.6611
Dbn1	drebrin 1	81653	0.00022553	5.3459	0.0018091	0.99859	0.99918	-6.6611
Tesc	tescalcin	288689	-1.482E-05	6.5535	-0.000162	0.99987	0.99987	-6.6611

Supplementary Table 4b. Differential expression analysis of the GA muscle samples								
Gene names	Protein names	EntrezID	logFC	AveExpr	t	P.Value	adj.P.Val	B
Slc25a11	solute carrier family 25 member	64201	-0.74149	10.809	-5.3262	0.00042023	0.10563	0.27447
Bdh1	3-hydroxybutyrate dehydrogena	117099	-0.56638	6.528	-5.2446	0.00046967	0.10563	0.18354
Psmc2	"proteasome 26S subunit, ATPa	25581	0.65448	5.3201	5.1996	0.0004996	0.10563	0.13285
C1qbp	complement C1q binding protei	29681	-0.75025	8.6537	-5.1827	0.00051132	0.10563	0.1138
Vim	vimentin	81818	0.81929	8.2903	4.8874	0.00077277	0.10563	-0.22865
Clic4	chloride intracellular channel 4	83718	0.59336	4.44	4.8177	0.00085344	0.10563	-0.31179
S100a10	S100 calcium binding protein A1	81778	0.70761	7.111	4.7931	0.00088396	0.10563	-0.34127
Ndufab1	NADH:ubiquinone oxidoreducta	293453	-1.0493	8.9104	-4.7431	0.00094989	0.10563	-0.40177
Rras2	RAS related 2	365355	-0.6215	5.6014	-4.7202	0.00098181	0.10563	-0.4296
Lamp2	lysosomal-associated membrane	24944	-0.60872	5.161	-4.6567	0.0010763	0.10563	-0.50716
Kcnj11	"potassium inwardly-rectifying ch	83535	-0.58457	3.9314	-4.6557	0.0010779	0.10563	-0.5084
Atp5md	ATP synthase membrane subur	171069	-1.1272	8.4729	-4.5072	0.0013396	0.10563	-0.69291
Actn1	"actinin, alpha 1"	81634	0.45919	4.6721	4.4589	0.0014388	0.10563	-0.75379
Ctsb	cathepsin B	64529	-0.57655	6.4036	-4.4557	0.0014456	0.10563	-0.75778
Cox6b1	cytochrome c oxidase subunit 6	688869	-0.90535	10.952	-4.4345	0.0014919	0.10563	-0.78471
Tppp3	tubulin polymerization-promoting	291966	0.52921	5.2823	4.4315	0.0014984	0.10563	-0.78844
Uqcr10	"ubiquinol-cytochrome c reducta	685322	-0.64853	9.1677	-4.4263	0.0015101	0.10563	-0.7951
Cct3	chaperonin containing TCP1 su	295230	0.53977	6.3632	4.4255	0.001512	0.10563	-0.79619
Slc12a2	solute carrier family 12 member	83629	-0.56489	7.8054	-4.4082	0.0015514	0.10563	-0.81816
Vdac1	voltage-dependent anion chanr	83529	-0.56802	13.756	-4.3011	0.0018209	0.10563	-0.95547
Vdac3	voltage-dependent anion chanr	83532	-0.60729	11.559	-4.2728	0.0019003	0.10563	-0.99217
Ndufa11	NADH:ubiquinone oxidoreducta	301123	-0.6266	9.179	-4.2388	0.0020005	0.10563	-1.0364
Aqp1	aquaporin 1	25240	0.76815	6.525	4.2182	0.0020639	0.10563	-1.0633
Nnt	nicotinamide nucleotide transhy	310378	-0.61808	13.031	-4.2153	0.0020729	0.10563	-1.067
Pet100	PET100 cytochrome c oxidase c	688786	-0.61547	4.0935	-4.2049	0.002106	0.10563	-1.0807
Sod2	superoxide dismutase 2	24787	-0.57354	9.9167	-4.1699	0.0022211	0.10563	-1.1266
Casq1	calsequestrin 1	686019	-0.66582	11.789	-4.1528	0.0022796	0.10563	-1.1491
Fn1	fibronectin 1	25661	0.49852	6.7342	4.135	0.0023423	0.10563	-1.1726
Samm50	SAMM50 sorting and assembly	300111	-0.52991	10.248	-4.1255	0.0023768	0.10563	-1.1852
Cd59	CD59 molecule	25407	-0.67342	8.9377	-4.1238	0.0023828	0.10563	-1.1874
Ganab	glucosidase II alpha subunit	293721	0.60567	5.813	4.1091	0.0024371	0.10563	-1.2069
Fitm1	fat storage-inducing transmemb	290223	-0.74342	7.2844	-4.0676	0.0025974	0.10563	-1.2621
Jph1	junctophilin 1	297748	-0.4843	8.8365	-4.063	0.0026156	0.10563	-1.2681
Dpysl2	dihydropyrimidinase-like 2	25416	0.46703	6.695	4.0512	0.0026634	0.10563	-1.2838
Cacna1s	calcium voltage-gated channel s	682930	-0.53475	10.07	-4.0185	0.002801	0.10563	-1.3276
Cav3	caveolin 3	29161	1.0229	7.5088	3.9797	0.0029744	0.10563	-1.3798
Coq3	coenzyme Q3 methyltransferase	29309	-0.48689	5.9017	-3.9595	0.0030691	0.10563	-1.407
Sdr39u1	"short chain dehydrogenase/rec	361044	-0.48945	6.5833	-3.9559	0.0030863	0.10563	-1.4119

Lum	lumican	81682	0.48499	5.6363	3.9243	0.0032412	0.10563	-1.4545
Bcs1l	"BCS1 homolog, ubiquinol-cytoc	301514	-0.50691	6.5326	-3.9186	0.0032703	0.10563	-1.4623
Tmem186	transmembrane protein 186	497863	-0.71693	4.2942	-3.9169	0.0032789	0.10563	-1.4646
Itgb2	integrin subunit beta 2	309684	1.43	4.5143	3.9059	0.0033356	0.10563	-1.4796
Cct7	chaperonin containing TCP1 su	297406	0.41762	6.2173	3.9018	0.0033572	0.10563	-1.4852
Cct2	chaperonin containing TCP1 su	299809	0.47948	6.5098	3.8779	0.0034845	0.10563	-1.5177
Sgcg	"sarcoglycan, gamma"	305941	-0.52567	6.1805	-3.8536	0.0036193	0.10563	-1.5508
Cyc1	cytochrome c-1	300047	-0.56253	11.742	-3.8343	0.0037309	0.10563	-1.5773
Mdh1	malate dehydrogenase 1	24551	0.51319	8.2843	3.834	0.0037325	0.10563	-1.5777
Nap1l4	nucleosome assembly protein 1	361684	0.45959	4.5885	3.8103	0.0038736	0.10563	-1.6101
Gapdh	glyceraldehyde-3-phosphate de	24383	0.47665	11.189	3.8032	0.0039175	0.10563	-1.62
Eepd1	endonuclease/exonuclease/pho	315500	-0.46281	4.0022	-3.7894	0.0040034	0.10563	-1.639
Tubb5	"tubulin, beta 5 class I"	29214	0.57775	6.8806	3.7885	0.004009	0.10563	-1.6402
Atp2a2	ATPase sarcoplasmic/endoplasi	29693	-0.41499	10.574	-3.7844	0.0040348	0.10563	-1.6458
Septin7	septin 7	64551	0.43543	6.2786	3.7837	0.0040391	0.10563	-1.6467
Myl6	myosin light chain 6	685867	0.51813	5.3413	3.7482	0.0042716	0.10664	-1.6957
Ndufs4	NADH:ubiquinone oxidoreducta	499529	-0.50826	9.47	-3.718	0.0044805	0.10664	-1.7376
Abcc9	ATP binding cassette subfamily	25560	-0.42745	6.2723	-3.7051	0.0045728	0.10664	-1.7554
Ahnak	AHNAK nucleoprotein	191572	0.43461	10.755	3.6957	0.0046419	0.10664	-1.7686
Ckb	creatine kinase B	24264	0.56685	6.0547	3.6918	0.0046706	0.10664	-1.774
Maip1	matrix AAA peptidase interacting	301418	-0.70494	5.0312	-3.6777	0.004776	0.10664	-1.7936
Lpcat3	lysophosphatidylcholine acyltran	362434	-0.60333	4.9998	-3.6761	0.0047884	0.10664	-1.7959
Eif2s1	eukaryotic translation initiation fa	54318	0.4816	4.9492	3.6634	0.0048856	0.10664	-1.8135
Acss3	acyl-CoA synthetase short-chain	314800	-0.5085	3.8397	-3.6388	0.0050807	0.10664	-1.8479
Anpep	"alanyl aminopeptidase, membra	81641	0.37122	6.6993	3.6347	0.0051142	0.10664	-1.8537
Ndufa8	NADH:ubiquinone oxidoreducta	296658	-0.49777	11.056	-3.6209	0.005228	0.10664	-1.873
Arl6ip5	ADP-ribosylation factor like GTP	66028	-0.65148	7.3492	-3.5998	0.0054068	0.10664	-1.9026
Lamp1	lysosomal-associated membrane	25328	-0.46438	6.6908	-3.5956	0.0054433	0.10664	-1.9085
Pdlim1	PDZ and LIM domain 1	54133	0.45681	5.5679	3.5884	0.0055062	0.10664	-1.9186
Cisd3	CDGSH iron sulfur domain 3	287661	-0.47453	7.2977	-3.5865	0.0055227	0.10664	-1.9212
Phb2	prohibitin 2	114766	-0.42634	11.252	-3.5828	0.0055554	0.10664	-1.9264
Ndufa12	NADH:ubiquinone oxidoreducta	299739	-0.41752	9.7579	-3.574	0.0056339	0.10664	-1.9387
Acss1	acyl-CoA synthetase short-chain	296259	-0.52139	6.6363	-3.5655	0.0057114	0.10664	-1.9508
Sypl2	synaptophysin-like 2	362018	-0.6068	8.007	-3.5516	0.0058403	0.10664	-1.9704
Ckap4	cytoskeleton-associated protein	362859	0.58704	7.3217	3.5452	0.0059002	0.10664	-1.9794
Epb41l2	erythrocyte membrane protein b	309557	0.41888	7.5928	3.5379	0.0059692	0.10664	-1.9896
Rps23	ribosomal protein S23	124323	0.44616	6.1648	3.5373	0.0059755	0.10664	-1.9906
Atp5f1c	ATP synthase F1 subunit gamm	116550	-0.58199	12.494	-3.5269	0.0060762	0.10664	-2.0053
Rab14	"RAB14, member RAS oncogen	94197	-0.5921	6.4863	-3.5266	0.0060789	0.10664	-2.0057
Anxa5	annexin A5	25673	0.49023	7.5934	3.5094	0.0062489	0.10664	-2.03

Vdac2	voltage-dependent anion chann	83531	-0.47024	11.598	-3.5003	0.0063409	0.10664	-2.0428
Rtraf	"RNA transcription, translation a	302247	0.55628	6.0554	3.4875	0.0064726	0.10664	-2.061
Mcu	mitochondrial calcium uniporter	294560	-0.38508	7.8225	-3.4865	0.0064832	0.10664	-2.0624
Abcb7	ATP binding cassette subfamily	302395	-0.48386	7.2416	-3.4826	0.0065242	0.10664	-2.068
Sdhc	succinate dehydrogenase comp	289217	-0.5339	6.6998	-3.4726	0.0066298	0.10664	-2.0821
Ghitm	growth hormone inducible trans	290596	-0.51238	5.265	-3.4705	0.0066525	0.10664	-2.0851
Psme1	proteasome activator subunit 1	29630	0.44063	5.8215	3.455	0.0068215	0.10664	-2.1072
Ndufs8	NADH:ubiquinone oxidoreducta	293652	-0.53911	10.051	-3.4392	0.0069977	0.10664	-2.1297
Hhatl	hedgehog acyltransferase-like	301073	-0.48656	9.0938	-3.4362	0.0070313	0.10664	-2.134
Unc45b	unc-45 myosin chaperone B	303373	0.36336	6.6949	3.4316	0.0070832	0.10664	-2.1404
Tomm22	translocase of outer mitochondr	300075	-0.44048	5.7505	-3.4227	0.0071856	0.10664	-2.1531
Cnst	"consortin, connexin sorting pro	498297	-0.4251	4.838	-3.4137	0.007291	0.10664	-2.166
Fkbp11	FKBP prolyl isomerase 11	300211	0.63322	5.6198	3.4095	0.0073409	0.10664	-2.172
Mrps18b	mitochondrial ribosomal protein	294230	-0.45077	4.2776	-3.4093	0.0073434	0.10664	-2.1723
Uqcc2	ubiquinol-cytochrome c reductas	361805	-0.43464	7.3334	-3.4093	0.0073441	0.10664	-2.1724
Ppid	peptidylprolyl isomerase D	361967	-0.37609	5.1213	-3.4046	0.0073995	0.10664	-2.179
Tmem11	transmembrane protein 11	303196	-0.50084	6.4333	-3.4045	0.0074007	0.10664	-2.1791
Ndufa4	"NDUFA4, mitochondrial comple	681024	-0.46041	12.745	-3.3994	0.0074624	0.10664	-2.1865
Niban2	niban apoptosis regulator 2	362115	-0.67488	4.1609	-3.3911	0.0075632	0.10664	-2.1983
Uqcc1	ubiquinol-cytochrome c reductas	683512	-0.48455	7.6119	-3.384	0.0076509	0.10664	-2.2085
Acad9	"acyl-CoA dehydrogenase family	294973	-0.34494	9.1864	-3.3832	0.0076611	0.10664	-2.2097
Uqcrh	ubiquinol-cytochrome c reductas	366448	-0.42733	10.233	-3.3776	0.0077303	0.10664	-2.2176
Rnh1	ribonuclease/angiogenin inhibito	100360501	0.36785	5.674	3.3743	0.0077714	0.10664	-2.2223
Mylpf	"myosin light chain, phosphoryla	24584	-0.69469	12.367	-3.342	0.0081906	0.11122	-2.2687
Tecr	"trans-2,3-enoyl-CoA reductase"	191576	-0.48654	8.9489	-3.3364	0.0082649	0.11122	-2.2767
Cox7b	cytochrome c oxidase subunit 7	303393	-0.5991	8.1958	-3.3262	0.0084034	0.11199	-2.2914
Por	cytochrome p450 oxidoreductas	29441	-0.58113	6.6785	-3.3035	0.008719	0.11403	-2.3239
Rtn4	reticulon 4	83765	-0.56477	8.2267	-3.3034	0.0087212	0.11403	-2.3242
Ndufc2	NADH:ubiquinone oxidoreducta	293130	-0.43185	9.78	-3.2777	0.0090942	0.11647	-2.3612
Atp5mf	ATP synthase membrane subur	690441	0.59532	10.812	3.2772	0.0091005	0.11647	-2.3618
Ndufv1	NADH:ubiquinone oxidoreducta	293655	-0.3991	12.227	-3.2681	0.0092378	0.11647	-2.3751
Tapt1	transmembrane anterior posteric	305386	-0.45218	5.5423	-3.2615	0.0093372	0.11647	-2.3845
Cacnb1	calcium voltage-gated channel a	50688	-0.44738	9.5567	-3.2614	0.0093381	0.11647	-2.3846
Tpm3	tropomyosin 3	117557	-0.46548	6.6296	-3.2566	0.009412	0.11647	-2.3916
Hpx	hemopexin	58917	0.36992	5.5446	3.2375	0.0097112	0.11846	-2.4193
Ndufs7	NADH:ubiquinone oxidoreducta	362837	-0.42481	9.9125	-3.2354	0.0097437	0.11846	-2.4222
Atp5pb	ATP synthase peripheral stalk-m	171375	-0.66332	11.679	-3.2245	0.0099194	0.11955	-2.438
Atp5if1	ATP synthase inhibitory factor s	25392	-0.8645	8.0372	-3.2128	0.010111	0.11987	-2.4549
Slc25a12	solute carrier family 25 member	362145	-0.45939	11.825	-3.2123	0.010119	0.11987	-2.4556
Oma1	OMA1 zinc metallopeptidase	298282	-0.33099	5.3125	-3.1817	0.010638	0.12447	-2.4999

Rock1	Rho-associated coiled-coil conta	81762	-0.35428	4.6092	-3.1728	0.010796	0.12447	-2.513
Ctsd	cathepsin D	171293	-0.54318	9.043	-3.1714	0.01082	0.12447	-2.5149
Lrrc57	leucine rich repeat containing 57	311346	-0.32028	3.9298	-3.1655	0.010926	0.12447	-2.5235
Mreg	melanoregulin	501162	-0.43098	3.9808	-3.1609	0.011008	0.12447	-2.5302
Hsp90ab1	heat shock protein 90 alpha fan	301252	0.42387	9.6746	3.1588	0.011046	0.12447	-2.5332
Dlat	dihydrolipoamide S-acetyltransfe	81654	-0.35895	11.996	-3.1433	0.011331	0.12665	-2.5558
Pdia4	"protein disulfide isomerase fam	116598	0.51703	7.7965	3.1321	0.011542	0.12687	-2.5721
Timm50	translocase of inner mitochondri	687295	-0.36511	7.6234	-3.1281	0.011618	0.12687	-2.5779
Gng12	G protein subunit gamma 12	114120	0.40562	5.3975	3.1277	0.011625	0.12687	-2.5784
Art1	ADP-ribosyltransferase 1	308873	-0.37745	4.472	-3.1077	0.012014	0.13	-2.6076
Gna13	G protein subunit alpha 13	303634	-0.35297	4.8431	-3.1004	0.01216	0.13	-2.6182
Snx2	sorting nexin 2	291464	0.69342	5.6339	3.0987	0.012194	0.13	-2.6207
Col1a1	collagen type I alpha 1 chain	29393	0.9195	6.1288	3.0901	0.012366	0.13084	-2.6332
Parl	"presenilin associated, rhomboid	287979	-0.51566	5.0294	-3.0823	0.012528	0.13154	-2.6446
Exoc4	exocyst complex component 4	116654	0.61072	4.2596	3.0426	0.013374	0.13937	-2.7026
Ppp2r1a	protein phosphatase 2 scaffold	117281	0.31722	5.1434	3.0339	0.013568	0.13937	-2.7153
Ifnl1	"interferon, lambda 1"	100911249	1.4148	5.0672	3.0336	0.013575	0.13937	-2.7158
Slc44a2	solute carrier family 44 member	363024	-0.38943	5.1102	-3.0276	0.013709	0.13971	-2.7245
Cyb5r1	cytochrome b5 reductase 1	304805	-0.51253	9.7733	-3.0159	0.013978	0.14072	-2.7416
Eef2	eukaryotic translation elongation	29565	0.40307	9.0174	3.014	0.01402	0.14072	-2.7443
Mrpl27	mitochondrial ribosomal protein	287635	-0.32844	5.1595	-3.0067	0.014192	0.14072	-2.7551
Lamtor5	"late endosomal/lysosomal adap	295357	0.58061	6.1419	3.0056	0.014218	0.14072	-2.7567
Lrp1	LDL receptor related protein 1	299858	0.30836	6.6884	3.0014	0.014315	0.14072	-2.7628
Pmpca	"peptidase, mitochondrial proce	296588	-0.39492	7.8295	-2.9907	0.014571	0.14124	-2.7784
Pcyox1	prenylcysteine oxidase 1	246302	-0.37114	7.3033	-2.987	0.01466	0.14124	-2.7839
Slc25a4	solute carrier family 25 member	85333	-0.56948	15.602	-2.9864	0.014674	0.14124	-2.7847
Cul1	cullin 1	362356	-0.36665	3.9863	-2.9805	0.014819	0.14165	-2.7934
Romo1	reactive oxygen species modula	679572	-0.42116	5.9799	-2.9725	0.015016	0.142	-2.8051
Mfn2	mitofusin 2	64476	-0.51975	3.8299	-2.9707	0.015061	0.142	-2.8077
Trdn	triadin	59299	-0.51081	11.587	-2.9404	0.015838	0.14832	-2.8523
Mrpl58	mitochondrial ribosomal protein	303673	-0.33936	4.5398	-2.9356	0.015963	0.14849	-2.8592
Calu	calumenin	64366	-0.54353	6.8999	-2.9295	0.016125	0.14899	-2.8682
Psmc3	"proteasome 26S subunit, ATPa	29677	0.3737	4.0549	2.9233	0.016291	0.14953	-2.8772
Apoo	apolipoprotein O	363474	-0.33508	8.8277	-2.9108	0.016631	0.15007	-2.8956
Dnajb4	DnaJ heat shock protein family (	295549	-0.39076	5.113	-2.9086	0.016693	0.15007	-2.8988
Pecr	peroxisomal trans-2-enoyl-CoA r	113956	-0.29851	7.0633	-2.9058	0.01677	0.15007	-2.9029
Tubb4b	"tubulin, beta 4B class IVb"	296554	0.3305	9.5364	2.9028	0.016856	0.15007	-2.9074
Ryr1	ryanodine receptor 1	114207	-0.42018	13.74	-2.9001	0.016929	0.15007	-2.9113
Fga	fibrinogen alpha chain	361969	0.36497	8.0137	2.8932	0.017124	0.15007	-2.9214
Rpn2	ribophorin II	64701	-0.31808	7.3079	-2.8873	0.017294	0.15007	-2.9301

Clpb	caseinolytic mitochondrial matrix	65041	0.44526	5.0791	2.8864	0.017321	0.15007	-2.9315
Tkt	transketolase	64524	0.36958	4.8861	2.8863	0.017324	0.15007	-2.9317
Ckm	"creatine kinase, M-type"	24265	0.3707	12.315	2.8805	0.01749	0.15013	-2.9401
Dhrs4	dehydrogenase/reductase 4	266686	-0.32882	6.78	-2.8785	0.017548	0.15013	-2.943
Myh11	myosin heavy chain 11	24582	0.34266	5.5416	2.8717	0.017748	0.15047	-2.9531
Ndufa7	NADH:ubiquinone oxidoreducta	299643	-0.35022	10.475	-2.8623	0.018025	0.15047	-2.9668
Slc25a42	"solute carrier family 25, membe	689414	-0.3614	7.1963	-2.8596	0.018106	0.15047	-2.9708
Rpl27a	ribosomal protein L27a	293418	-0.47017	6.1251	-2.851	0.018367	0.15047	-2.9834
Etf1	eukaryotic translation terminatio	307503	0.31728	4.0574	2.8486	0.018442	0.15047	-2.987
Rab2a	"RAB2A, member RAS oncogen	65158	-0.34778	7.7885	-2.847	0.01849	0.15047	-2.9893
Cd34	CD34 molecule	305081	0.41916	6.512	2.8466	0.018504	0.15047	-2.99
Nmnat3	nicotinamide nucleotide adenyly	363118	0.47598	5.0898	2.8449	0.018556	0.15047	-2.9925
Tmem143	transmembrane protein 143	308593	-0.3228	7.1447	-2.8446	0.018565	0.15047	-2.9929
Mgll	monoglyceride lipase	29254	-0.30768	5.8994	-2.8398	0.018712	0.15054	-2.9999
Prkcsh	protein kinase C substrate 80K-	300445	0.31262	6.43	2.8373	0.018791	0.15054	-3.0036
Ptges2	prostaglandin E synthase 2	311865	-0.36768	8.594	-2.8173	0.019427	0.15474	-3.033
App	amyloid beta precursor protein	54226	-0.35417	3.8213	-2.809	0.019696	0.15599	-3.0452
Ndufb10	NADH:ubiquinone oxidoreducta	681418	-0.39449	11.198	-2.8027	0.019903	0.15647	-3.0545
Myl2	myosin light chain 2	363925	-0.52611	8.1792	-2.8003	0.019982	0.15647	-3.058
St13	"ST13, Hsp70 interacting protei	81800	0.34866	5.3824	2.7928	0.020233	0.15694	-3.069
Uqcrb	ubiquinol-cytochrome c reductas	362897	-0.3735	12.413	-2.7918	0.020269	0.15694	-3.0706
Hccs	holocytochrome c synthase	317444	-0.30966	6.5201	-2.7809	0.020638	0.15891	-3.0865
Mrps35	mitochondrial ribosomal protein	297727	-0.35059	6.5299	-2.7742	0.020871	0.1592	-3.0964
Acadvl	"acyl-CoA dehydrogenase, very	25363	-0.3103	12.194	-2.7732	0.020905	0.1592	-3.0979
Plpp7	phospholipid phosphatase 7 (in	296635	-0.35439	5.0733	-2.767	0.02112	0.15996	-3.107
Mvp	major vault protein	64681	0.38147	7.7706	2.7595	0.021387	0.16044	-3.1181
Stx8	syntaxin 8	59074	-0.33677	5.1336	-2.7575	0.021457	0.16044	-3.1209
Gypc	glycophorin C (Gerbich blood gr	364837	0.41975	5.6352	2.7555	0.021531	0.16044	-3.124
Slc16a1	solute carrier family 16 member	25027	-0.39397	6.7804	-2.7438	0.021953	0.16271	-3.1411
Pvalb	parvalbumin	25269	0.39732	9.0142	2.7393	0.022119	0.16307	-3.1478
Ccpg1	cell cycle progression 1	363098	-0.38243	5.1238	-2.7293	0.02249	0.16493	-3.1625
Cox4i1	cytochrome c oxidase subunit 4	29445	-0.39068	13.271	-2.7076	0.023316	0.16637	-3.1943
Rab12	"RAB12, member RAS oncogen	25530	-0.30407	4.0246	-2.7054	0.023404	0.16637	-3.1977
Scp2	sterol carrier protein 2	25541	0.30035	8.4453	2.7035	0.023477	0.16637	-3.2004
Cox18	cytochrome c oxidase assembly	289522	-0.41332	5.6232	-2.697	0.023734	0.16637	-3.21
Rap2b	"RAP2B, member of RAS oncog	170923	0.41468	5.3972	2.6866	0.024146	0.16637	-3.2252
Ndufb5	NADH:ubiquinone oxidoreducta	294964	-0.30062	10.402	-2.6861	0.024168	0.16637	-3.226
Adhfe1	"alcohol dehydrogenase, iron co	362474	-0.32907	5.5951	-2.6836	0.024269	0.16637	-3.2297
Pdk4	pyruvate dehydrogenase kinase	89813	-0.44645	7.736	-2.6836	0.024269	0.16637	-3.2297
Ppbp	pro-platelet basic protein	246358	-0.32244	3.8876	-2.6832	0.024284	0.16637	-3.2303

Cct6a	chaperonin containing TCP1 su	288620	0.34823	6.6608	2.6811	0.024371	0.16637	-3.2334
Cavin1	caveolae associated protein 1	287710	0.3884	7.4804	2.6798	0.024423	0.16637	-3.2353
Hexa	hexosaminidase subunit alpha	300757	0.30818	4.2717	2.6783	0.024485	0.16637	-3.2375
Cox14	cytochrome c oxidase assembly	681219	-0.39186	4.9853	-2.6747	0.024632	0.16637	-3.2428
Flna	filamin A	293860	0.32282	8.3543	2.6724	0.024725	0.16637	-3.2461
Sec61a2	Sec61 translocon alpha 2 subu	361273	-0.29007	4.732	-2.6708	0.024793	0.16637	-3.2486
Capn2	calpain 2	29154	0.35417	6.3519	2.669	0.024867	0.16637	-3.2512
Agk	acylglycerol kinase	502749	-0.29438	7.0405	-2.667	0.024949	0.16637	-3.2541
Gnas	GNAS complex locus	24896	-0.3032	7.5007	-2.666	0.024991	0.16637	-3.2556
Cpt2	carnitine palmitoyltransferase 2	25413	-0.30101	10.486	-2.6639	0.025078	0.16637	-3.2586
Rps12	ribosomal protein S12	65139	0.53013	6.5635	2.6637	0.025087	0.16637	-3.259
Gba	glucosylceramidase beta	684536	-0.35744	5.4555	-2.6605	0.025222	0.1664	-3.2637
Atp1b1	ATPase Na+/K+ transporting su	25650	-0.32564	8.7764	-2.6579	0.025332	0.1664	-3.2675
Atp2a3	ATPase sarcoplasmic/endoplas	25391	-0.41822	13.039	-2.651	0.025623	0.16738	-3.2776
Sec31a	"SEC31 homolog A, COPII coat	93646	0.35397	4.7348	2.6487	0.025723	0.16738	-3.281
Ndufaf2	NADH:ubiquinone oxidoreducta	361894	-0.30495	6.2749	-2.6372	0.026222	0.16805	-3.298
Sec22b	"SEC22 homolog B, vesicle traf	310710	-0.38931	5.5505	-2.6353	0.026304	0.16805	-3.3007
Cobl	cordon-bleu WH2 repeat protein	305497	-0.27777	6.0066	-2.6342	0.026353	0.16805	-3.3024
Spcs3	signal peptidase complex subu	680782	-0.30088	4.4463	-2.6326	0.026423	0.16805	-3.3047
Parp14	"poly (ADP-ribose) polymerase f	303903	-0.84292	6.4182	-2.6319	0.026454	0.16805	-3.3058
Lman2	"lectin, mannose-binding 2"	290994	-0.50899	6.3812	-2.6296	0.026553	0.16805	-3.3091
Add2	adducin 2	24171	0.34763	5.113	2.6265	0.026693	0.16816	-3.3137
Rpsa	ribosomal protein SA	29236	0.49936	7.7883	2.604	0.027712	0.1738	-3.3467
Tln1	talin 1	313494	0.31182	8.0203	2.5999	0.027904	0.17421	-3.3528
Mybpc1	myosin binding protein C1	362867	0.34193	9.2009	2.5815	0.028774	0.17805	-3.3798
Msn	moesin	81521	0.33294	8.9557	2.5814	0.028776	0.17805	-3.3799
Gnaq	G protein subunit alpha q	81666	-0.37631	5.5267	-2.578	0.028941	0.17827	-3.3849
Bnip3	BCL2 interacting protein 3	84480	-0.31667	5.6854	-2.5632	0.029661	0.1819	-3.4066
Mtx1	Metaxin 1	295241	-0.28217	6.0967	-2.56	0.029823	0.18209	-3.4114
Cltc	clathrin heavy chain	54241	0.29458	7.7368	2.5508	0.030282	0.18408	-3.4248
Atp6v1a	ATPase H+ transporting V1 sub	685232	0.32697	5.8774	2.5464	0.030504	0.18462	-3.4312
Ap2m1	adaptor related protein complex	116563	0.31734	7.355	2.5325	0.031223	0.18815	-3.4517
Akap12	A-kinase anchoring protein 12	83425	0.36663	4.3968	2.5273	0.031493	0.18896	-3.4593
Pf4	platelet factor 4	360918	0.3196	5.9361	2.5138	0.03221	0.19193	-3.4791
Ankrd2	ankyrin repeat domain 2	309374	0.28775	4.4231	2.5127	0.032272	0.19193	-3.4808
Mrpl33	mitochondrial ribosomal protein	100363539	-0.29817	4.81	-2.5102	0.032405	0.19193	-3.4844
F13a1	coagulation factor XIII A1 chain	60327	0.29097	4.9244	2.5058	0.032641	0.19251	-3.4908
LOC1009125	"NADH dehydrogenase [ubiquin	100912599	-0.37136	10.244	-2.4995	0.032988	0.19373	-3.5001
Atp5pd	ATP synthase peripheral stalk s	641434	-0.43521	12.995	-2.4875	0.033654	0.19681	-3.5176
Edf1	endothelial differentiation-relate	296570	-0.30703	4.0987	-2.4827	0.033926	0.19757	-3.5247

Ehd4	EH-domain containing 4	192204	0.28309	6.7264	2.4714	0.034569	0.19971	-3.5412
Ppox	protoporphyrinogen oxidase	289219	-0.27017	4.6091	-2.4712	0.034582	0.19971	-3.5415
Tufm	"Tu translation elongation factor	293481	-0.26694	11.223	-2.4674	0.034803	0.19987	-3.5471
Anxa1	annexin A1	25380	0.25016	6.6448	2.4657	0.034899	0.19987	-3.5495
Eef1d	eukaryotic translation elongation	300033	0.48592	6.4281	2.4572	0.035395	0.20189	-3.5619
Acad8	"acyl-CoA dehydrogenase family	367196	-0.28075	7.2944	-2.4536	0.035607	0.202	-3.5671
Cdh13	cadherin 13	192248	0.35428	8.8209	2.4515	0.035736	0.202	-3.5703
Fabp3	fatty acid binding protein 3	79131	0.3005	7.3312	2.4495	0.035853	0.202	-3.5732
Cd36	CD36 molecule	29184	-0.39948	9.1151	-2.4403	0.036406	0.20374	-3.5866
Micu1	mitochondrial calcium uptake 1	365567	-0.31767	5.9234	-2.4381	0.036539	0.20374	-3.5898
Stbd1	starch binding domain 1	305234	-0.53971	7.5048	-2.436	0.036668	0.20374	-3.5929
Camk2g	calcium/calmodulin-dependent p	171140	-0.35265	6.5101	-2.4347	0.03675	0.20374	-3.5948
Pabpc1	"poly(A) binding protein, cytopla	171350	0.36241	5.4658	2.4293	0.03708	0.20441	-3.6027
Mtdh	metadherin	170910	-0.36453	6.445	-2.4279	0.037166	0.20441	-3.6047
Cox7c	cytochrome c oxidase subunit 7	100188937	-0.28237	9.3578	-2.4227	0.03749	0.20483	-3.6123
Yars2	tyrosyl-tRNA synthetase 2	287924	-0.3022	3.8306	-2.4216	0.03756	0.20483	-3.6139
Deptor	DEP domain containing MTOR-i	314979	-0.27668	3.7945	-2.4196	0.037685	0.20483	-3.6168
Ndufb1	NADH:ubiquinone oxidoreducta	100912357	-0.27735	8.5274	-2.4122	0.038151	0.20577	-3.6276
Mgst3	microsomal glutathione S-transfe	289197	-0.34596	6.4769	-2.4122	0.038155	0.20577	-3.6277
Aldh6a1	"aldehyde dehydrogenase 6 far	81708	-0.45112	10.569	-2.4056	0.038575	0.207	-3.6373
Man2a2	"mannosidase, alpha, class 2A,	308757	0.28709	4.1921	2.4001	0.038929	0.207	-3.6453
Timmdc1	translocase of inner mitochondri	303922	0.32553	4.1827	2.399	0.039	0.207	-3.6469
Magt1	magnesium transporter 1	116967	-0.26792	4.061	-2.3985	0.03903	0.207	-3.6475
Ppia	peptidylprolyl isomerase A	25518	0.3153	7.6934	2.3955	0.039229	0.207	-3.652
Tmx2	thioredoxin-related transmembra	295701	0.58947	6.4922	2.3947	0.039279	0.207	-3.6531
Ccdc51	coiled-coil domain containing 51	316008	-0.30404	7.4063	-2.3782	0.040372	0.21002	-3.6771
Hspb6	heat shock protein family B (sma	192245	0.30694	5.9691	2.3769	0.040459	0.21002	-3.679
Septin11	septin 11	305227	0.34042	5.2903	2.373	0.040724	0.21002	-3.6847
Rps2	ribosomal protein S2	83789	0.30448	7.4869	2.3729	0.040729	0.21002	-3.6848
C3	complement C3	24232	0.40704	5.3572	2.3697	0.040946	0.21002	-3.6895
Immt	inner membrane mitochondrial p	312444	-0.28853	12.485	-2.3689	0.041	0.21002	-3.6906
Itga2b	integrin subunit alpha 2b	685269	0.37756	5.5166	2.3688	0.04101	0.21002	-3.6908
Tgm2	transglutaminase 2	56083	0.26871	6.6995	2.3668	0.041146	0.21002	-3.6937
Ndufs1	NADH:ubiquinone oxidoreducta	301458	-0.28393	13.016	-2.3657	0.041219	0.21002	-3.6953
Pygm	"glycogen phosphorylase, musc	24701	0.3259	12.128	2.3636	0.041367	0.21002	-3.6984
LOC1003596	mitochondrial ribosomal protein	100359687	-0.24945	5.3853	-2.3584	0.041722	0.21105	-3.7059
Vps4a	vacuolar protein sorting 4 homo	246772	0.24414	5.2563	2.3488	0.042395	0.21367	-3.7198
Pcbp2	poly(rC) binding protein 2	363005	0.30118	5.9614	2.3357	0.043332	0.2176	-3.7389
Flot1	flotillin 1	64665	-0.36772	8.2095	-2.3314	0.043643	0.21826	-3.7452
Capzb	capping actin protein of muscle	298584	0.27319	7.1363	2.3295	0.043777	0.21826	-3.7478

Cpt1b	carnitine palmitoyltransferase 1B	25756	-0.28563	10.036	-2.3271	0.043954	0.21835	-3.7513
Pgm1	phosphoglucomutase 1	24645	0.30688	9.1248	2.3239	0.044185	0.21865	-3.7559
Prkar1a	protein kinase cAMP-dependen	25725	0.28179	4.1681	2.3201	0.044468	0.21865	-3.7615
Get3	"guided entry of tail-anchored p	288919	0.26451	4.9916	2.319	0.044549	0.21865	-3.7631
Eef1g	eukaryotic translation elongatior	293725	0.36522	8.2042	2.3177	0.044645	0.21865	-3.7649
Nmt1	N-myristoyltransferase 1	259274	-0.41229	3.8483	-2.3112	0.045129	0.21965	-3.7743
Acsl1	acyl-CoA synthetase long-chain	25288	-0.25256	11.989	-2.3091	0.045287	0.21965	-3.7774
Cox16	cytochrome c oxidase assembly	100302447	-0.27549	5.4556	-2.3086	0.045325	0.21965	-3.7781
Micu3	"mitochondrial calcium uptake fa	364601	0.65413	5.2698	2.3023	0.0458	0.22118	-3.7872
Gpd1	glycerol-3-phosphate dehydroge	60666	0.29516	8.8129	2.2987	0.046074	0.22173	-3.7924
Bgn	biglycan	25181	0.24207	6.3711	2.2947	0.046381	0.22244	-3.7982
Mrc1	"mannose receptor, C type 1"	291327	-0.30567	7.5267	-2.291	0.046669	0.22304	-3.8036
Rtca	RNA 3'-terminal phosphate cycla	295395	0.25123	5.1543	2.2888	0.046839	0.22309	-3.8067
Spg7	"SPG7 matrix AAA peptidase su	353231	-0.2451	5.3745	-2.2857	0.047084	0.22349	-3.8113
Mrpl48	mitochondrial ribosomal protein	293149	-0.26618	4.7902	-2.2808	0.047467	0.22423	-3.8183
Flnc	filamin C	362332	0.23346	10.242	2.2795	0.047564	0.22423	-3.8201
Rps17	ribosomal protein S17	29286	0.26264	6.8421	2.2663	0.048618	0.22842	-3.8392
Gpam	"glycerol-3-phosphate acyltrans	29653	0.25783	3.9479	2.2619	0.048976	0.22854	-3.8456
Pdhb	pyruvate dehydrogenase E1 be	289950	-0.30206	11.367	-2.2586	0.049246	0.22854	-3.8503
Ndufaf6	NADH:ubiquinone oxidoreducta	297821	-0.26214	5.687	-2.258	0.04929	0.22854	-3.8511
Hdlbp	high density lipoprotein binding	64474	0.31062	5.5471	2.2579	0.049304	0.22854	-3.8513
Flot2	flotillin 2	83764	-0.49815	7.6591	-2.2543	0.049601	0.22915	-3.8566
Septin2	septin 2	117515	0.28972	5.61	2.2514	0.049834	0.22947	-3.8606
Poldip2	DNA polymerase delta interactin	287544	-0.288	5.8812	-2.2474	0.050165	0.23023	-3.8664
Gfm2	"G elongation factor, mitochond	294672	0.25521	3.7748	2.2394	0.050833	0.23253	-3.8779
Hspa4	heat shock protein family A (Hsp	266759	0.28245	4.5067	2.2354	0.051173	0.23322	-3.8837
Synm	synemin	308709	-0.25766	5.6638	-2.2337	0.051322	0.23322	-3.8862
Phkg1	phosphorylase kinase catalytic s	29353	0.26369	6.3886	2.2293	0.051693	0.23342	-3.8924
Smtnl1	smoothelin-like 1	311167	0.40099	4.4653	2.2283	0.051783	0.23342	-3.894
Shmt2	serine hydroxymethyltransferase	299857	0.3143	6.366	2.2253	0.052034	0.23342	-3.8981
Stt3a	STT3 oligosaccharyltransferase	500972	-0.25316	4.1314	-2.2253	0.052039	0.23342	-3.8982
Slc25a3	solute carrier family 25 member	245959	-0.43469	13.548	-2.2191	0.052579	0.23392	-3.9072
Prdx4	peroxiredoxin 4	85274	0.28647	6.2996	2.2182	0.052653	0.23392	-3.9084
Dysf	dysferlin	312492	-0.37233	11.571	-2.2171	0.05275	0.23392	-3.91
Ptma	prothymosin alpha	29222	0.33097	4.4611	2.215	0.05293	0.23392	-3.913
Mrps6	mitochondrial ribosomal protein	100360017	-0.30059	5.4802	-2.2143	0.052995	0.23392	-3.914
Erp29	endoplasmic reticulum protein 2	117030	0.31213	4.5978	2.2121	0.053188	0.23403	-3.9172
Dnpep	aspartyl aminopeptidase	301529	0.22809	4.7047	2.207	0.053637	0.23525	-3.9245
Erlin2	ER lipid raft associated 2	290823	-0.53821	7.7264	-2.2047	0.053841	0.23541	-3.9277
Arhgdia	Rho GDP dissociation inhibitor a	360678	0.32357	6.6147	2.2006	0.054204	0.23625	-3.9336

Tpp1	tripeptidyl peptidase 1	83534	-0.40334	6.218	-2.1924	0.05495	0.23875	-3.9454
Gsta3	glutathione S-transferase alpha	494500	0.66587	5.3349	2.1882	0.055328	0.23964	-3.9513
Prkcq	"protein kinase C, theta"	85420	-0.26284	6.7356	-2.1754	0.056512	0.244	-3.9697
Bcap29	B-cell receptor-associated prote	298943	-0.32022	6.9119	-2.1686	0.057149	0.24599	-3.9793
Dnajb11	DnaJ heat shock protein family (	360734	0.35598	4.9746	2.1665	0.057349	0.24609	-3.9824
Fcer1g	Fc fragment of IgE receptor Ig	25441	0.30553	4.5858	2.1605	0.057918	0.24776	-3.9909
Synj2bp	synaptojanin 2 binding protein	64531	-0.28452	6.3385	-2.1581	0.058152	0.248	-3.9944
Sdhd	succinate dehydrogenase comp	363061	-0.27694	8.4229	-2.1555	0.058403	0.2483	-3.9981
Sgcd	"sarcoglycan, delta"	497892	0.40971	7.5847	2.1514	0.058796	0.24844	-4.0039
Rab22a	"RAB22A, member RAS oncoge	366265	0.25669	4.688	2.1498	0.058952	0.24844	-4.0062
Anxa6	annexin A6	79125	0.22516	9.7943	2.1475	0.059178	0.24844	-4.0095
Snap23	synaptosome associated protei	64630	-0.2393	5.706	-2.1456	0.059356	0.24844	-4.0121
Cadm4	cell adhesion molecule 4	365216	0.239	4.5198	2.145	0.059416	0.24844	-4.013
Atp5po	ATP synthase peripheral stalk s	192241	-0.35724	13.012	-2.1425	0.059663	0.24844	-4.0165
Uqcrc1	ubiquinol-cytochrome c reductas	301011	-0.24616	13.119	-2.1422	0.059689	0.24844	-4.0169
Atp2a1	ATPase sarcoplasmic/endoplası	116601	-0.38414	16.33	-2.135	0.060409	0.24953	-4.0273
Got1	glutamic-oxaloacetic transamina	24401	0.35998	7.2803	2.1343	0.060475	0.24953	-4.0282
Tmem65	transmembrane protein 65	500874	-0.32579	7.4806	-2.1341	0.060493	0.24953	-4.0284
Lrrc59	leucine rich repeat containing 5!	287633	0.25296	6.6593	2.1229	0.061618	0.25342	-4.0443
RGD1565784	RGD1565784	497874	-0.22982	5.9138	-2.1047	0.0635	0.26039	-4.0702
Zmpste24	zinc metallopeptidase STE24	313564	0.27738	4.1625	2.1019	0.063788	0.2608	-4.0741
Dnajc19	DnaJ heat shock protein family (	502525	0.32213	7.5672	2.0962	0.064391	0.2624	-4.0822
Serpinh1	serpin family H member 1	29345	0.57382	8.3941	2.0946	0.064558	0.2624	-4.0844
Gnb2	G protein subunit beta 2	81667	-0.26644	7.6344	-2.0894	0.065116	0.26367	-4.0919
Lactb	"lactamase, beta"	300803	-0.28306	6.7706	-2.0865	0.065426	0.26367	-4.0959
Slirp	SRA stem-loop interacting RNA	688717	0.27203	5.262	2.0863	0.065441	0.26367	-4.0961
Myh15	"myosin, heavy chain 15"	303965	-0.27728	5.4963	-2.083	0.065805	0.26383	-4.1009
Phka1	phosphorylase kinase regulator	64561	-0.30894	8.3543	-2.0824	0.065863	0.26383	-4.1017
Eif4a1	eukaryotic translation initiation fa	287436	0.24426	6.4257	2.0794	0.066195	0.26416	-4.106
Capza1	capping actin protein of muscle	691149	0.22602	4.0357	2.0782	0.066325	0.26416	-4.1077
Cand2	cullin-associated and neddylatio	192226	0.23071	6.9001	2.0723	0.066963	0.26593	-4.1159
Nucb1	nucleobindin 1	84595	0.20938	6.9599	2.0704	0.067174	0.26601	-4.1186
Nampt	nicotinamide phosphoribosyltran	297508	0.42074	5.0665	2.0662	0.067638	0.26708	-4.1245
Mcee	methylmalonyl CoA epimerase	293829	0.26933	4.6164	2.0614	0.068171	0.26803	-4.1312
Dnaja4	DnaJ heat shock protein family (	300721	0.33998	5.7881	2.0606	0.068264	0.26803	-4.1324
Cisd1	CDGSH iron sulfur domain 1	294362	-0.27668	9.2117	-2.0589	0.068458	0.26803	-4.1348
Fam162a	"family with sequence similarity 1	360721	-0.25703	8.496	-2.0542	0.068988	0.26839	-4.1414
Col1a2	collagen type I alpha 2 chain	84352	0.73095	5.6107	2.0536	0.069046	0.26839	-4.1422
Tmem109	transmembrane protein 109	361732	-0.26152	7.0994	-2.0529	0.06913	0.26839	-4.1432
Cavin4	caveolae associated protein 4	313225	0.29562	5.5636	2.0477	0.069727	0.26995	-4.1506

Vwa8	von Willebrand factor A domain	290381	-0.23806	6.4577	-2.0458	0.069939	0.27001	-4.1532
Oxa1l	"OXA1L, mitochondrial inner me	691393	-0.20906	7.036	-2.0433	0.070228	0.27038	-4.1567
Ldha	lactate dehydrogenase A	24533	0.29532	10.477	2.0409	0.070505	0.27069	-4.1601
Nptn	neuroplastin	56064	-0.26938	5.4372	-2.0372	0.070934	0.27091	-4.1653
Pmp2	peripheral myelin protein 2	688790	0.59558	8.0394	2.037	0.070953	0.27091	-4.1655
Prdx1	peroxiredoxin 1	117254	0.29817	7.1765	2.0243	0.072449	0.27531	-4.1833
Mmut	methylmalonyl-CoA mutase	688517	-0.25595	7.9585	-2.0238	0.072501	0.27531	-4.184
Pcca	propionyl-CoA carboxylase subu	687008	-0.23536	8.9161	-2.0176	0.073242	0.27666	-4.1927
Ugp2	UDP-glucose pyrophosphorylase	289827	0.3398	7.1171	2.0146	0.073597	0.27666	-4.1968
Mb	myoglobin	59108	-0.46437	9.7528	-2.0142	0.073648	0.27666	-4.1974
Pgam5	"PGAM family member 5, mitoch	288731	-0.24955	6.8211	-2.0132	0.07377	0.27666	-4.1988
Septin9	septin 9	83788	0.23671	4.8594	2.0097	0.074189	0.27666	-4.2036
Ptcd3	Pentatricopeptide repeat domai	500199	0.4664	5.7587	2.0078	0.074427	0.27666	-4.2064
Ndufs5	NADH:ubiquinone oxidoreducta	362588	-0.23092	9.7065	-2.0067	0.074555	0.27666	-4.2078
Tmsb4x	"thymosin beta 4, X-linked"	81814	0.40208	6.7501	2.0056	0.074687	0.27666	-4.2093
Sars2	"seryl-tRNA synthetase 2, mitoc	292759	-0.22369	6.6119	-2.0027	0.07504	0.27666	-4.2134
Apobec2	apolipoprotein B mRNA editing	301226	-0.22918	4.1489	-2.0012	0.075236	0.27666	-4.2156
Glo1	glyoxalase 1	294320	0.25923	5.4327	2.0005	0.075316	0.27666	-4.2165
Cfl2	cofilin 2	366624	0.23246	6.964	1.9996	0.075431	0.27666	-4.2178
Colec12	collectin sub-family member 12	361289	0.2533	4.1331	1.9994	0.075452	0.27666	-4.218
Bves	blood vessel epicardial substanc	365603	-0.28251	4.8324	-1.9932	0.076214	0.27868	-4.2266
Park7	Parkinsonism associated deglyc	117287	0.32773	7.0002	1.9895	0.076681	0.27868	-4.2318
Acsl4	acyl-CoA synthetase long-chain	113976	-0.26445	5.8645	-1.989	0.076738	0.27868	-4.2324
Sec63	"SEC63 homolog, protein transl	309858	-0.22146	4.9558	-1.9885	0.076808	0.27868	-4.2332
Ppp1r3a	"protein phosphatase 1, regulat	500036	0.235	6.3327	1.9822	0.077596	0.2808	-4.2419
Myh4	myosin heavy chain 4	360543	-0.37751	16.015	-1.9761	0.078377	0.28289	-4.2505
Pex12	peroxisomal biogenesis factor 1	116718	-0.3042	4.1337	-1.9706	0.079074	0.28467	-4.258
Casq2	calsequestrin 2	29209	-0.47201	7.2234	-1.9623	0.080147	0.28725	-4.2695
Tmed2	transmembrane p24 trafficking p	65165	-0.24697	5.3517	-1.9619	0.08021	0.28725	-4.2701
Ptpmt1	"protein tyrosine phosphatase,	29390	-0.2239	6.1325	-1.9603	0.080413	0.28725	-4.2723
Metap1	methionyl aminopeptidase 1	295500	0.29176	4.8208	1.9585	0.08065	0.28736	-4.2748
Aldoa	"aldolase, fructose-bisphosphat	24189	0.53371	12.567	1.9495	0.081844	0.29023	-4.2873
Phb	prohibitin	25344	-0.2171	10.504	-1.9492	0.081877	0.29023	-4.2876
Ano10	anoctamin 10	301111	0.39958	5.5935	1.9424	0.082791	0.29227	-4.297
Ndufb9	NADH:ubiquinone oxidoreducta	299954	-0.25823	10.614	-1.9415	0.082903	0.29227	-4.2982
Mia2	MIA SH3 domain ER export fact	100912115	0.23284	4.5233	1.9402	0.083084	0.29227	-4.3
Macrod1	mono-ADP ribosylhydrolase 1	246233	0.26839	10.538	1.9372	0.083493	0.29239	-4.3042
Csnk2a1	casein kinase 2 alpha 1	116549	0.25464	3.9571	1.9368	0.083539	0.29239	-4.3047
Fmod	fibromodulin	64507	0.21654	5.2596	1.9317	0.084231	0.29407	-4.3117
Uqcrc2	ubiquinol cytochrome c reductas	293448	-0.23874	13.115	-1.9279	0.084757	0.29516	-4.3169

Mrps11	mitochondrial ribosomal protein	499185	-0.20754	4.3553	-1.9199	0.085864	0.29826	-4.3279
Srl	sarcalumenin	302948	-0.26664	10.82	-1.9157	0.086455	0.29898	-4.3337
Marcksl1	MARCKS-like 1	81520	0.21184	4.7153	1.9153	0.086502	0.29898	-4.3342
Psmb7	proteasome 20S subunit beta 7	85492	0.20931	3.777	1.908	0.08753	0.30075	-4.3442
Hadhb	hydroxyacyl-CoA dehydrogenas	171155	-0.25206	11.862	-1.9072	0.087647	0.30075	-4.3453
Atp5f1e	ATP synthase F1 subunit epsilo	245958	-0.24221	11.75	-1.9071	0.087665	0.30075	-4.3455
Mthfd1	"methylenetetrahydrofolate deh	64300	-0.26042	4.176	-1.9048	0.087995	0.30114	-4.3487
Ube2m	ubiquitin-conjugating enzyme E2	361509	0.22788	3.8724	1.8997	0.088723	0.30288	-4.3556
Coq8a	coenzyme Q8A	360887	-0.24897	11.102	-1.8957	0.089302	0.30411	-4.3611
Ddx3x	"DEAD-box helicase 3, X-linked"	317335	-0.2436	5.9315	-1.8934	0.089624	0.30415	-4.3642
Myo15a	myosin XVA	501699	0.37989	4.9582	1.8922	0.0898	0.30415	-4.3658
Rab10	"RAB10, member RAS oncogen	50993	-0.2332	9.0074	-1.891	0.089972	0.30415	-4.3674
Lgals1	galectin 1	56646	0.33923	9.4695	1.8877	0.090459	0.30505	-4.372
Jph2	junctophilin 2	296345	-0.23997	8.3381	-1.8834	0.091093	0.30644	-4.3779
Atp8a1	ATPase phospholipid transporti	289615	-0.19825	5.0245	-1.879	0.091741	0.30788	-4.3839
Lnpk	"lunapark, ER junction formation	362151	-0.24342	6.3702	-1.8754	0.092277	0.30835	-4.3888
Ech1	enoyl-CoA hydratase 1	64526	-0.22011	9.082	-1.8721	0.092762	0.30835	-4.3932
Ogn	osteoglycin	291015	0.4218	5.3513	1.8716	0.09284	0.30835	-4.3939
Nt5dc3	5'-nucleotidase domain containi	691922	-0.20668	5.7087	-1.8703	0.093036	0.30835	-4.3957
Tap1	"transporter 1, ATP binding cas	24811	0.29513	4.0555	1.8694	0.093171	0.30835	-4.3969
Stoml2	stomatin like 2	298203	-0.25964	7.3116	-1.8691	0.093217	0.30835	-4.3973
Emc2	ER membrane protein complex s	362905	-0.20805	6.6595	-1.8643	0.093938	0.31	-4.4038
Napg	NSF attachment protein gamma	307382	0.31069	7.0068	1.8597	0.094629	0.31149	-4.41
Plpp3	phospholipid phosphatase 3	192270	0.19714	5.4397	1.858	0.094892	0.31149	-4.4123
Mrs2	magnesium transporter MRS2	79032	-0.24818	4.9313	-1.8556	0.095261	0.31149	-4.4156
C4bpa	"complement component 4 bind	24235	-0.27305	5.6357	-1.8554	0.095291	0.31149	-4.4158
Lnpep	leucyl and cystinyl aminopeptida	171105	-0.21999	5.7683	-1.8518	0.095853	0.31259	-4.4208
Ndufa10	NADH:ubiquinone oxidoreducta	678759	-0.21842	11.889	-1.8421	0.097347	0.31672	-4.4338
Mrpl14	mitochondrial ribosomal protein	301250	0.45449	5.0016	1.8404	0.097613	0.31684	-4.4361
Mrps7	mitochondrial ribosomal protein	113958	0.42091	6.916	1.8358	0.098347	0.31842	-4.4424
Fabp4	fatty acid binding protein 4	79451	0.25133	8.5209	1.8343	0.098587	0.31842	-4.4444
Pdk1	pyruvate dehydrogenase kinase	116551	-0.24591	6.7489	-1.83	0.099262	0.31842	-4.4501
Bzw2	basic leucine zipper and W2 do	171439	0.24436	3.9229	1.8298	0.099295	0.31842	-4.4504
Got2	glutamic-oxaloacetic transamina	25721	-0.25906	13.062	-1.8284	0.099515	0.31842	-4.4523
Mrps22	mitochondrial ribosomal protein	683519	-0.20249	6.3304	-1.8277	0.099637	0.31842	-4.4533
L2hgdh	L-2-hydroxyglutarate dehydroge	314196	-0.25637	8.4063	-1.8272	0.099706	0.31842	-4.4539
Prdx5	peroxiredoxin 5	113898	-0.25773	8.3075	-1.8243	0.10018	0.31869	-4.4578
Cacna2d1	calcium voltage-gated channel a	25399	-0.29979	11.486	-1.8238	0.10025	0.31869	-4.4585
Mrpl15	mitochondrial ribosomal protein	297799	0.36054	6.2741	1.8182	0.10115	0.32036	-4.466
Tmco1	transmembrane and coiled-coil d	289196	-0.22869	4.3337	-1.8174	0.10129	0.32036	-4.4671

Pptc7	PTC7 protein phosphatase hom	304488	-0.21918	3.9843	-1.816	0.10151	0.32036	-4.4689
Maob	monoamine oxidase B	25750	-0.33728	5.7337	-1.8135	0.10192	0.32036	-4.4723
Surf1	"SURF1, cytochrome c oxidase	64463	-0.19058	6.1509	-1.8135	0.10193	0.32036	-4.4724
Rpl14	ribosomal protein L14	65043	0.22237	7.2398	1.8079	0.10284	0.32248	-4.4798
Stt3b	STT3 oligosaccharyltransferase	363160	-0.22771	5.1435	-1.801	0.10398	0.32533	-4.489
Enpp3	ectonucleotide pyrophosphatas	54410	0.19857	4.1123	1.7981	0.10447	0.32594	-4.493
Vapa	VAMP associated protein A	58857	-0.21187	8.8872	-1.797	0.10465	0.32594	-4.4944
Tmem43	transmembrane protein 43	362401	-0.30637	6.8109	-1.7953	0.10494	0.32612	-4.4967
Gnb1	G protein subunit beta 1	24400	0.19459	7.3329	1.7904	0.10576	0.32784	-4.5032
Rrbp1	ribosome binding protein 1	311483	0.28492	6.5543	1.7887	0.10605	0.32784	-4.5055
Ehd1	EH-domain containing 1	293692	-0.22985	9.5413	-1.7862	0.10648	0.32784	-4.5088
Cltb	"clathrin, light chain B"	116561	0.20515	4.5578	1.785	0.10669	0.32784	-4.5105
Ppif	peptidylprolyl isomerase F	282819	0.25648	8.1879	1.7844	0.10678	0.32784	-4.5112
Tsfm	"Ts translation elongation factor	679068	-0.19169	4.5369	-1.782	0.10719	0.32784	-4.5144
Akr1b1	aldo-keto reductase family 1 me	24192	0.26588	7.0619	1.7814	0.10729	0.32784	-4.5152
Timm29	translocase of inner mitochondri	315463	-0.22259	5.1133	-1.779	0.1077	0.32784	-4.5183
Ipo5	importin 5	306182	0.19586	4.8482	1.778	0.10787	0.32784	-4.5197
Arfip1	ADP-ribosylation factor interactir	60382	-0.44415	5.1973	-1.7772	0.10802	0.32784	-4.5208
Phyh	phytanoyl-CoA 2-hydroxylase	114209	-0.29068	5.2692	-1.7767	0.1081	0.32784	-4.5214
Ndufa9	NADH:ubiquinone oxidoreducta	362440	-0.21473	11.844	-1.7728	0.10877	0.32784	-4.5266
Rps21	ribosomal protein S21	81775	-0.18902	4.3815	-1.7721	0.10889	0.32784	-4.5275
Steap4	STEAP4 metalloreductase	499991	-0.21425	5.927	-1.7714	0.10903	0.32784	-4.5285
Gyg1	glycogenin 1	81675	0.27207	6.693	1.7713	0.10904	0.32784	-4.5287
Adam9	ADAM metallopeptidase domain	290834	-0.21255	4.3635	-1.7675	0.1097	0.32911	-4.5337
Cox7a2l	cytochrome c oxidase subunit 7	298762	-0.2129	7.8542	-1.7629	0.1105	0.33058	-4.5397
Mrps15	mitochondrial ribosomal protein	298517	-0.20166	5.9745	-1.762	0.11067	0.33058	-4.541
Akr7a2	"aldo-keto reductase family 7, m	171445	0.20865	4.8893	1.7571	0.11154	0.33245	-4.5475
Rab11a	"RAB11a, member RAS oncoge	81830	0.18792	8.7205	1.7536	0.11215	0.33336	-4.5521
Pitrm1	pitrilysin metallopeptidase 1	307081	-0.19569	7.2402	-1.7527	0.11232	0.33336	-4.5533
Mrpl32	mitochondrial ribosomal protein	291206	-0.19626	4.9243	-1.7498	0.11283	0.33403	-4.5571
Pkm	pyruvate kinase M1/2	25630	0.22057	10.87	1.7487	0.11303	0.33403	-4.5586
Acadsb	"acyl-CoA dehydrogenase, shor	25618	-0.36816	7.4602	-1.7438	0.11392	0.33593	-4.565
Pnpla8	patatin-like phospholipase doma	314075	-0.19085	4.8817	-1.7414	0.11434	0.33601	-4.5681
Hspb7	heat shock protein family B (sma	50565	0.27058	5.5398	1.741	0.11443	0.33601	-4.5687
Ilvbl	ilvB acetolactate synthase like	362843	-0.1827	4.8363	-1.7391	0.11476	0.33613	-4.5711
Coq7	"coenzyme Q7, hydroxylase"	25249	-0.1943	6.4625	-1.7381	0.11495	0.33613	-4.5725
Ndufa6	NADH:ubiquinone oxidoreducta	315167	-0.21975	10.734	-1.7346	0.11559	0.33725	-4.5771
Flnb	filamin B	306204	0.19186	5.3206	1.732	0.11606	0.33725	-4.5805
Sncg	"synuclein, gamma"	64347	-0.24873	5.9898	-1.7287	0.11668	0.33725	-4.5849
Pura	purine rich element binding prot	307498	0.21283	4.251	1.7277	0.11685	0.33725	-4.5861

Hmox2	heme oxygenase 2	79239	-0.1934	6.5972	-1.7256	0.11726	0.33725	-4.589
Nedd4	NEDD4 E3 ubiquitin protein liga	25489	0.20227	5.1498	1.7255	0.11728	0.33725	-4.5891
Sfxn3	sideroflexin 3	65042	-0.20637	4.5954	-1.7244	0.11747	0.33725	-4.5905
Pnp	purine nucleoside phosphorylas	290029	0.19029	4.6384	1.7232	0.1177	0.33725	-4.5921
Ogdh	oxoglutarate dehydrogenase	360975	-0.25136	12.413	-1.7227	0.11779	0.33725	-4.5928
Fundc2	FUN14 domain containing 2	361288	-0.20167	9.5038	-1.7226	0.11781	0.33725	-4.5929
Dnajc11	DnaJ heat shock protein family (	362666	-0.22143	6.6504	-1.7215	0.11801	0.33725	-4.5943
Timm9	translocase of inner mitochondri	171139	-0.18045	6.2905	-1.7201	0.11828	0.33732	-4.5962
Psma1	proteasome 20S subunit alpha	29668	0.23546	4.8287	1.7148	0.11927	0.33943	-4.603
Scn4a	sodium voltage-gated channel a	25722	-0.18467	6.8678	-1.7094	0.12029	0.34121	-4.6101
Prdx6	peroxiredoxin 6	94167	0.1992	5.0622	1.7089	0.12038	0.34121	-4.6107
Hacd1	3-hydroxyacyl-CoA dehydratase	680115	-0.24287	5.7194	-1.7026	0.1216	0.34396	-4.619
Tpt1	"tumor protein, translationally-co	116646	0.21129	5.4477	1.7004	0.12201	0.34406	-4.6218
Eif2s3	eukaryotic translation initiation fa	299027	0.28949	5.9818	1.6998	0.12213	0.34406	-4.6226
Atic	5-aminoimidazole-4-carboxamide	81643	0.23078	5.1089	1.6974	0.1226	0.34411	-4.6258
Rps3a	ribosomal protein S3a	29288	0.29344	8.8273	1.6971	0.12265	0.34411	-4.6261
Hspa8	heat shock protein family A (Hsp	24468	0.21456	10.299	1.6947	0.12313	0.34475	-4.6293
Col14a1	collagen type XIV alpha 1 chain	314981	0.38124	7.9819	1.6916	0.12372	0.34527	-4.6333
Mpc1	mitochondrial pyruvate carrier 1	171087	-0.41345	9.3168	-1.6903	0.12398	0.34527	-4.635
Myo1c	myosin 1C	65261	0.19397	6.8407	1.6893	0.12418	0.34527	-4.6363
Idh2	isocitrate dehydrogenase (NADF	361596	-0.43326	13.478	-1.6886	0.12431	0.34527	-4.6372
Hdhd5	haloacid dehalogenase like hyd	312680	0.25696	4.9155	1.6849	0.12503	0.34658	-4.6419
Coro1c	coronin 1C	501841	0.19075	4.6178	1.6813	0.12575	0.34789	-4.6467
Iars2	"isoleucyl-tRNA synthetase 2, m	364070	-0.1794	8.1615	-1.6776	0.12648	0.34828	-4.6514
Mrpl53	mitochondrial ribosomal protein	362388	-0.19557	5.5359	-1.6773	0.12655	0.34828	-4.6519
Hadha	hydroxyacyl-CoA dehydrogenas	170670	-0.22489	13.264	-1.6768	0.12665	0.34828	-4.6525
Tuba8	"tubulin, alpha 8"	500377	0.27322	6.3221	1.6744	0.12712	0.3489	-4.6556
Slc16a3	solute carrier family 16 member	80878	-0.27403	4.9992	-1.6719	0.12763	0.34958	-4.6588
Rab35	"RAB35, member RAS oncogen	288700	-0.19616	6.0506	-1.6693	0.12814	0.35006	-4.6621
Myh1	myosin heavy chain 1	287408	-0.22405	13.181	-1.6685	0.1283	0.35006	-4.6632
Cyb5a	cytochrome b5 type A	64001	-0.20751	7.0417	-1.6626	0.12949	0.35261	-4.6708
Ralb	RAS like proto-oncogene B	116546	0.19976	5.9674	1.66	0.13004	0.35305	-4.6742
Lclat1	lysocardiolipin acyltransferase 1	362702	-0.23389	3.8663	-1.6593	0.13016	0.35305	-4.675
Echdc3	enoyl CoA hydratase domain co	684538	-0.19784	4.2702	-1.6565	0.13075	0.35389	-4.6787
Agps	alkylglycerone phosphate synth	84114	-0.1754	6.5156	-1.6553	0.13099	0.35389	-4.6802
Lmnb1	lamin B1	116685	0.26848	5.9905	1.6533	0.1314	0.35433	-4.6828
Ndufa5	NADH:ubiquinone oxidoreducta	25488	-0.19165	11.316	-1.6473	0.13265	0.35699	-4.6905
Vat1	vesicle amine transport 1	287721	0.17517	7.7267	1.6425	0.13365	0.35795	-4.6967
Eea1	early endosome antigen 1	314764	-0.20234	4.3452	-1.6422	0.13371	0.35795	-4.697
Etfdh	electron transfer flavoprotein de	295143	-0.19541	11.549	-1.6419	0.13378	0.35795	-4.6974

Timm21	translocase of inner mitochondri	307210	-0.18109	7.3054	-1.6395	0.13427	0.35856	-4.7004
Hspa5	heat shock protein family A (Hsp	25617	0.18738	10.838	1.6365	0.13491	0.35959	-4.7043
Emc10	ER membrane protein complex s	292878	0.1828	4.9023	1.6322	0.13581	0.36087	-4.7097
Snta1	"syntrophin, alpha 1"	362242	0.20483	5.7591	1.6318	0.13591	0.36087	-4.7103
Pi4k2a	phosphatidylinositol 4-kinase typ	114554	-0.17842	4.8513	-1.6298	0.13633	0.36112	-4.7128
Svip	small VCP interacting protein	499157	-0.18938	3.7894	-1.6269	0.13695	0.36112	-4.7166
Psmc1	"proteasome 26S subunit, ATPa	117263	0.17067	5.3764	1.6268	0.13698	0.36112	-4.7167
Sptan1	"spectrin, alpha, non-erythrocyti	64159	0.23519	9.1238	1.6262	0.1371	0.36112	-4.7174
Rtn4ip1	reticulon 4 interacting protein 1	309912	-0.1881	7.0749	-1.6246	0.13744	0.36112	-4.7195
Eif3a	"eukaryotic translation initiation	292148	0.17221	4.8508	1.624	0.13757	0.36112	-4.7202
Hnrnpk	heterogeneous nuclear ribonucl	117282	0.21245	5.8108	1.6224	0.13792	0.36134	-4.7223
Tmod1	tropomodulin 1	25566	0.22766	5.0587	1.6184	0.13877	0.36288	-4.7273
Tm9sf3	transmembrane 9 superfamily m	309475	-0.20501	3.9827	-1.6143	0.13966	0.36414	-4.7325
Mrpl45	mitochondrial ribosomal protein	287656	0.24181	5.431	1.6138	0.13977	0.36414	-4.7332
Actr3	actin related protein 3	81732	0.24032	6.487	1.6082	0.141	0.36609	-4.7403
Canx	calnexin	29144	0.17101	8.8625	1.608	0.14105	0.36609	-4.7406
Rack1	receptor for activated C kinase 1	83427	0.23051	7.2459	1.6063	0.14142	0.36634	-4.7427
Nudc	"nuclear distribution C, dynein c	29648	-0.19804	3.8383	-1.6051	0.14167	0.36634	-4.7442
Rpl32	ribosomal protein L32	28298	-0.26739	6.8914	-1.6028	0.14219	0.36676	-4.7471
Pm20d2	peptidase M20 domain containi	313130	-0.94615	7.266	-1.602	0.14236	0.36676	-4.7481
Ggt5	gamma-glutamyltransferase 5	29566	0.26076	5.504	1.6001	0.14279	0.36705	-4.7506
Chmp1a	charged multivesicular body pro	365024	-0.19222	4.0911	-1.5989	0.14305	0.36705	-4.752
Igtp	interferon gamma induced GTPa	303163	-0.18855	3.8859	-1.5979	0.14327	0.36705	-4.7533
Acads	acyl-CoA dehydrogenase short	64304	-0.19642	9.9716	-1.5934	0.14429	0.36898	-4.759
C9	complement C9	117512	0.2628	4.4048	1.5868	0.14577	0.37157	-4.7673
Prxl2a	peroxiredoxin like 2A	361118	-0.29787	6.7181	-1.5865	0.14584	0.37157	-4.7677
Marcks	myristoylated alanine rich protei	25603	0.23513	4.4354	1.5809	0.1471	0.3741	-4.7747
Clec10a	C-type lectin domain containing	64195	-0.20749	3.9203	-1.5795	0.14742	0.37421	-4.7764
Rps5	ribosomal protein S5	25538	0.28367	6.8432	1.5763	0.14815	0.37539	-4.7804
Asph	aspartate-beta-hydroxylase	312981	-0.33154	9.3234	-1.5726	0.149	0.37686	-4.785
Uqcrfs1	"ubiquinol-cytochrome c reducta	291103	-0.21067	12.613	-1.568	0.15007	0.37882	-4.7908
Nipsnap2	nipsnap homolog 2	498174	-0.18139	10.617	-1.5658	0.1506	0.37882	-4.7936
Itga7	integrin subunit alpha 7	81008	0.22025	5.0988	1.5649	0.15081	0.37882	-4.7948
Psap	prosaposin	25524	-0.32361	8.6109	-1.5646	0.15087	0.37882	-4.7951
Rab1b	"RAB1B, member RAS oncogen	100126191	-0.19029	9.1981	-1.5601	0.15191	0.38073	-4.8006
Stxbp3	syntaxin binding protein 3	114095	0.40511	7.3462	1.5563	0.15281	0.38136	-4.8054
Bcap31	B-cell receptor-associated prote	293852	-0.25906	8.1918	-1.5561	0.15286	0.38136	-4.8057
Tagln	transgelin	25123	0.33415	5.1202	1.5556	0.15298	0.38136	-4.8063
Snx5	sorting nexin 5	296199	0.1582	3.8822	1.5531	0.15356	0.38212	-4.8094
Fis1	"fission, mitochondrial 1"	288584	-0.19165	8.3518	-1.5468	0.15507	0.385	-4.8172

Map2k1	mitogen activated protein kinase	170851	0.29058	4.1667	1.5459	0.15528	0.385	-4.8183
Trim72	tripartite motif containing 72	365377	0.21088	12.63	1.5435	0.15586	0.38576	-4.8213
Coq9	coenzyme Q9	498909	-0.1929	10.112	-1.5397	0.15678	0.38647	-4.8261
Slc43a2	solute carrier family 43 member	287532	-0.24368	4.4887	-1.539	0.15695	0.38647	-4.8269
Dnajb12	DnaJ heat shock protein family	294513	1.121	6.0557	1.5386	0.15704	0.38647	-4.8274
Reep1	receptor accessory protein 1	362384	-0.20927	4.4801	-1.5377	0.15727	0.38647	-4.8285
Ssbp1	single stranded DNA binding pro	54304	0.45835	4.645	1.5329	0.15841	0.3886	-4.8344
Psmc5	"proteasome 26S subunit, ATPa	81827	0.17941	4.3295	1.5312	0.15883	0.38893	-4.8365
Ndufs2	NADH:ubiquinone oxidoreducta	289218	-0.17412	11.844	-1.5192	0.1618	0.39502	-4.8513
Ncl	nucleolin	25135	0.33082	5.6155	1.5188	0.16189	0.39502	-4.8517
Ufd1	ubiquitin recognition factor in EF	84478	0.16289	5.0707	1.5155	0.1627	0.39551	-4.8557
Agl	"amylo-alpha-1, 6-glucosidase, 4	362029	0.16412	8.661	1.513	0.16334	0.39551	-4.8589
Prdx2	peroxiredoxin 2	29338	0.16488	4.9184	1.5128	0.16337	0.39551	-4.8591
Tmem38b	transmembrane protein 38B	362521	-0.29705	4.6953	-1.5123	0.16351	0.39551	-4.8597
Cct8	chaperonin containing TCP1 su	288305	0.19677	6.8994	1.511	0.16384	0.39551	-4.8613
Sccpdh	saccharopine dehydrogenase (p	305021	0.22769	7.3644	1.5104	0.16399	0.39551	-4.8621
Cd47	Cd47 molecule	29364	0.16822	6.0346	1.51	0.16408	0.39551	-4.8625
Atp6v0d1	ATPase H+ transporting V0 sub	291969	-0.20249	6.4466	-1.5081	0.16456	0.39596	-4.8648
Gdi2	GDP dissociation inhibitor 2	29662	0.22611	6.6437	1.5069	0.16487	0.39604	-4.8664
Prnp	prion protein	24686	-0.16745	3.8903	-1.503	0.16585	0.3977	-4.8711
Opa1	"OPA1, mitochondrial dynamin l	171116	-0.17115	9.573	-1.4993	0.1668	0.39775	-4.8756
Neb	nebulin	311029	-0.20172	11.195	-1.4982	0.16708	0.39775	-4.877
Ndufs3	NADH:ubiquinone oxidoreducta	295923	-0.1769	11.157	-1.4976	0.16722	0.39775	-4.8776
Tmed10	transmembrane p24 trafficking p	84599	-0.18147	7.8997	-1.4965	0.1675	0.39775	-4.879
Aup1	"AUP1, lipid droplet regulating V	680423	0.17851	6.0637	1.4965	0.16751	0.39775	-4.879
Sptbn1	"spectrin, beta, non-erythrocytic	305614	0.20543	8.809	1.4961	0.1676	0.39775	-4.8794
Anxa11	annexin A11	290527	0.19207	8.0483	1.4935	0.16828	0.39869	-4.8827
Atp6v0a1	ATPase H+ transporting V0 sub	29757	-0.22146	5.0704	-1.4916	0.16875	0.39884	-4.8849
Abcf1	ATP binding cassette subfamily	85493	-0.16175	3.9998	-1.491	0.16892	0.39884	-4.8857
Sdhb	succinate dehydrogenase comp	298596	-0.17365	12.341	-1.489	0.16942	0.39906	-4.8881
Acad10	"acyl-CoA dehydrogenase family	304500	-0.1661	4.7227	-1.4884	0.16958	0.39906	-4.8888
Ndufaf3	NADH:ubiquinone oxidoreducta	56769	-0.16343	6.0848	-1.4866	0.17006	0.39933	-4.891
Ndufb11	NADH:ubiquinone oxidoreducta	299310	-0.18458	9.2085	-1.4857	0.17028	0.39933	-4.8921
Snd1	staphylococcal nuclease and tu	64635	0.21973	6.0916	1.4814	0.1714	0.40047	-4.8973
Myh9	"myosin, heavy chain 9, non-mu	100911597	0.24151	9.7059	1.4811	0.17148	0.40047	-4.8977
Ddah2	dimethylarginine dimethylaminoh	294239	0.16104	4.5176	1.4805	0.17163	0.40047	-4.8984
Tpm4	tropomyosin 4	24852	0.25541	6.303	1.4745	0.17322	0.40349	-4.9057
Pecam1	platelet and endothelial cell adh	29583	0.1775	5.4086	1.4716	0.17397	0.40405	-4.9091
Acsf2	acyl-CoA synthetase family mem	619561	-0.40687	5.7652	-1.4708	0.1742	0.40405	-4.9101
Timm10	translocase of inner mitochondri	64464	-0.1697	5.6436	-1.4689	0.17469	0.40405	-4.9124

Mrpl43	mitochondrial ribosomal protein	309440	0.20916	5.7605	1.4686	0.17478	0.40405	-4.9128
Acadm	acyl-CoA dehydrogenase mediu	24158	0.18788	10.336	1.4681	0.17491	0.40405	-4.9134
Calm3	calmodulin 3	24244	-0.19695	7.6275	-1.4636	0.17611	0.40529	-4.9188
Aldh1l1	"aldehyde dehydrogenase 1 fa	64392	-0.18046	4.1705	-1.4627	0.17634	0.40529	-4.9198
Tomm40	translocase of outer mitochondr	308416	-0.18386	7.1736	-1.4625	0.1764	0.40529	-4.9201
Tpi1	triosephosphate isomerase 1	24849	0.18454	10.248	1.4617	0.17662	0.40529	-4.9211
Atl2	atlastin GTPase 2	298757	-0.18226	7.7815	-1.4594	0.17723	0.40601	-4.9238
Vamp2	vesicle-associated membrane p	24803	0.2875	4.2091	1.4551	0.1784	0.40801	-4.929
Actn4	actinin alpha 4	63836	0.20197	6.6985	1.4509	0.17953	0.40909	-4.934
Atp1a1	ATPase Na+/K+ transporting su	24211	-0.2232	9.9414	-1.4499	0.17979	0.40909	-4.9351
Cyb5b	cytochrome b5 type B	80773	0.19388	4.9392	1.4493	0.17995	0.40909	-4.9359
Itgav	integrin subunit alpha V	296456	0.17852	5.2853	1.449	0.18005	0.40909	-4.9363
Rtn2	reticulon 2	308410	-0.17932	9.225	-1.4468	0.18064	0.40977	-4.9389
Adpgk	ADP-dependent glucokinase	315722	0.20822	4.1499	1.4437	0.1815	0.41105	-4.9426
Mrpl19	mitochondrial ribosomal protein	297372	-0.19274	5.6336	-1.4425	0.18182	0.4111	-4.944
Rpl21	ribosomal protein L21	79449	0.1506	6.8988	1.4406	0.18233	0.41158	-4.9462
Myh14	myosin heavy chain 14	308572	0.24543	5.6748	1.435	0.18388	0.41441	-4.9529
Rps9	ribosomal protein S9	81772	0.21525	7.207	1.4337	0.18426	0.41458	-4.9545
Pgk1	phosphoglycerate kinase 1	24644	0.17811	8.7149	1.4321	0.18469	0.41487	-4.9563
Sbds	"SBDS, ribosome maturation fa	288615	-0.19503	4.8631	-1.4268	0.18619	0.41724	-4.9627
Dhrs7c	dehydrogenase/reductase 7C	287411	-0.18457	8.1122	-1.4259	0.18642	0.41724	-4.9637
Eci1	enoyl-CoA delta isomerase 1	29740	0.3795	9.6439	1.4251	0.18664	0.41724	-4.9646
Hint3	histidine triad nucleotide binding	246769	-0.15589	5.4715	-1.4222	0.18748	0.41816	-4.9681
Atp1b2	ATPase Na+/K+ transporting su	24214	-0.20203	6.6023	-1.4191	0.18835	0.41816	-4.9718
Me3	malic enzyme 3	361602	-0.15932	7.7407	-1.419	0.18838	0.41816	-4.9718
Serbp1	Serpine1 mRNA binding protein	246303	0.30083	6.5498	1.4176	0.18877	0.41816	-4.9735
Serpina1	serpin family A member 1	24648	0.17999	5.8785	1.4165	0.18908	0.41816	-4.9748
Abcb6	ATP binding cassette subfamily	140669	-0.19422	3.9755	-1.4165	0.18909	0.41816	-4.9748
Yars1	tyrosyl-tRNA synthetase 1	313047	0.16937	4.1469	1.4162	0.18917	0.41816	-4.9751
Dld	dihydrolipoamide dehydrogenas	298942	-0.19597	11.976	-1.4095	0.19108	0.42171	-4.983
Synpo2	synaptopodin 2	499702	0.23533	5.3655	1.4079	0.19153	0.42171	-4.9849
Hibadh	3-hydroxyisobutyrate dehydroge	63938	0.2911	8.1178	1.4074	0.19169	0.42171	-4.9855
Slc25a51	"solute carrier family 25, membe	100909697	0.18375	5.7928	1.3987	0.1942	0.42655	-4.9957
Actn3	actinin alpha 3	171009	-0.18971	10.687	-1.3977	0.1945	0.42655	-4.9969
Anxa4	annexin A4	79124	0.21774	7.6341	1.3954	0.19516	0.42732	-4.9995
Plec	plectin	64204	0.1464	10.322	1.3926	0.19598	0.42845	-5.0028
Agpat1	1-acylglycerol-3-phosphate O-a	406165	-0.1554	4.8246	-1.39	0.19675	0.42883	-5.0058
Mlycd	malonyl-CoA decarboxylase	85239	-0.14286	7.2659	-1.3899	0.19678	0.42883	-5.006
LOC1083480	collagen alpha-1(XV) chain-like	108348074	0.31032	5.0453	1.3874	0.19751	0.42976	-5.0089
Taco1	translational activator of cytochr	360645	-0.14602	5.17	-1.3829	0.19883	0.43194	-5.014

Rps8	ribosomal protein S8	65136	0.21359	7.7025	1.3805	0.19955	0.43226	-5.0168
Fahd1	fumarylacetoacetate hydrolase (	302980	0.26004	7.3347	1.3803	0.1996	0.43226	-5.017
Lmna	lamin A/C	60374	-0.19987	7.7648	-1.3769	0.20064	0.43261	-5.021
Actn2	actinin alpha 2	291245	-0.16977	9.1305	-1.3759	0.20093	0.43261	-5.0222
Slc25a10	solute carrier family 25 member	170943	-0.15068	4.4927	-1.3755	0.20105	0.43261	-5.0226
Slc25a19	solute carrier family 25 member	303676	-0.2313	5.3044	-1.3747	0.20128	0.43261	-5.0235
Gcsh	glycine cleavage system protein	171133	-0.20241	5.7605	-1.3746	0.20132	0.43261	-5.0237
Abcb10	ATP binding cassette subfamily	361439	-0.14075	5.3157	-1.3712	0.20233	0.43411	-5.0276
Cox11	COX11 cytochrome c oxidase co	690300	0.18806	3.9698	1.3699	0.20271	0.43425	-5.029
Galnt2	polypeptide N-acetylgalactosam	292090	-0.16489	4.0608	-1.3662	0.20384	0.43537	-5.0333
Ephx1	epoxide hydrolase 1	25315	0.16772	4.0583	1.3645	0.20437	0.43537	-5.0353
Cst3	cystatin C	25307	-0.14955	5.9847	-1.3632	0.20475	0.43537	-5.0368
P4hb	prolyl 4-hydroxylase subunit bet	25506	0.16356	9.9061	1.3608	0.20547	0.43537	-5.0395
Fgg	fibrinogen gamma chain	24367	0.18485	6.7163	1.3606	0.20556	0.43537	-5.0398
Klhl40	kelch-like family member 40	316088	0.1636	4.0821	1.3603	0.20563	0.43537	-5.0401
Rras	RAS related	361568	-0.1505	6.3387	-1.36	0.20573	0.43537	-5.0405
Rab3a	"RAB3A, member RAS oncoger	25531	0.17478	4.3003	1.3598	0.20579	0.43537	-5.0407
Enpp4	ectonucleotide pyrophosphatas	301261	-0.18664	4.1429	-1.3589	0.20606	0.43537	-5.0417
Mrpl3	mitochondrial ribosomal protein	300974	0.20418	6.4835	1.3558	0.207	0.43669	-5.0452
Rps7	ribosomal protein S7	29258	0.19351	6.4839	1.3491	0.20908	0.43978	-5.0529
Stx4	syntaxin 4	81803	-0.17634	6.0528	-1.3482	0.20937	0.43978	-5.054
Spryd4	SPRY domain containing 4	288772	-0.14843	5.76	-1.348	0.20942	0.43978	-5.0542
Cmc1	C-x(9)-C motif containing 1	363162	-0.22074	4.2612	-1.3445	0.2105	0.44117	-5.0582
Anxa2	annexin A2	56611	0.16116	10.598	1.343	0.21098	0.44117	-5.0599
Atp5pf	ATP synthase peripheral stalk s	94271	-0.22417	11.027	-1.3428	0.21104	0.44117	-5.0601
Npepps	aminopeptidase puromycin sens	50558	0.20625	3.8971	1.3273	0.21592	0.45009	-5.0777
Eef1b2	eukaryotic translation elongatior	363241	0.3437	5.1491	1.3272	0.21595	0.45009	-5.0778
Sgpl1	sphingosine-1-phosphate lyase	286896	-0.14611	3.9091	-1.3255	0.21649	0.45049	-5.0797
Pdlim3	PDZ and LIM domain 3	114108	0.14198	6.5703	1.3242	0.21689	0.45049	-5.0812
Decr1	"2,4-dienoyl-CoA reductase 1"	117543	0.59818	9.3368	1.3235	0.21712	0.45049	-5.082
Timm23	translocase of inner mitochondri	54312	0.23833	5.835	1.3176	0.21903	0.45378	-5.0887
Grn	granulin precursor	29143	-0.21349	4.4139	-1.3129	0.22053	0.45572	-5.0939
Ubtd1	ubiquitin domain containing 1	309373	-0.14125	4.3649	-1.3126	0.22063	0.45572	-5.0942
Sucla2	succinate-CoA ligase ADP-formi	361071	-0.15741	12.03	-1.3063	0.22268	0.45928	-5.1013
Rplp0	ribosomal protein lateral stalk su	64205	-0.20651	9.2026	-1.3014	0.22427	0.46188	-5.1067
Dbi	"diazepam binding inhibitor, acy	25045	0.14905	6.141	1.2969	0.22575	0.46424	-5.1117
Rps13	ribosomal protein S13	161477	0.20197	7.3707	1.2949	0.22644	0.46436	-5.114
Ywhaq	"tyrosine 3-monooxygenase/tryp	25577	0.186	5.0426	1.2947	0.22648	0.46436	-5.1142
Rab7a	"RAB7A, member RAS oncoger	29448	-0.13665	8.5372	-1.293	0.22705	0.46484	-5.1161
Spart	spartin	295053	0.20849	3.9308	1.2902	0.22797	0.46603	-5.1192

Idh3a	isocitrate dehydrogenase (NAD(	114096	-0.16239	11.78	-1.2803	0.23131	0.47073	-5.1302
Sgcb	"sarcoglycan, beta"	680229	0.14092	6.4808	1.2802	0.23132	0.47073	-5.1302
B2m	beta-2 microglobulin	24223	-0.28453	6.3104	-1.2784	0.23192	0.47073	-5.1322
Emb	embigin	114511	0.19311	5.7228	1.2784	0.23196	0.47073	-5.1323
Aco2	aconitase 2	79250	-0.20575	13.325	-1.2783	0.23198	0.47073	-5.1324
Niban1	niban apoptosis regulator 1	63912	0.19351	5.3423	1.2773	0.23231	0.47073	-5.1335
Scn1b	sodium voltage-gated channel b	29686	-0.16618	5.1799	-1.2747	0.23319	0.47183	-5.1363
Arl8b	ADP-ribosylation factor like GTP	500282	-0.2257	8.4548	-1.2731	0.23373	0.47193	-5.1381
Mospd1	motile sperm domain containing	317312	-0.17991	5.3704	-1.2725	0.23392	0.47193	-5.1387
Rpl36	ribosomal protein L36	58927	0.17687	7.3846	1.2677	0.23556	0.47446	-5.144
Ddx1	DEAD-box helicase 1	84474	0.18101	7.0482	1.2669	0.23586	0.47446	-5.1449
Rab8a	"RAB8A, member RAS oncogen	117103	-0.21154	5.2777	-1.2634	0.23703	0.47613	-5.1487
Lmcd1	LIM and cysteine-rich domains 1	494021	0.21276	4.7559	1.2622	0.23748	0.47633	-5.1501
Txndc5	thioredoxin domain containing 5	100362805	0.17715	4.8697	1.259	0.23856	0.47782	-5.1535
Epb41	erythrocyte membrane protein b	313052	0.18085	8.5772	1.2568	0.23932	0.47865	-5.1559
Fhl1	four and a half LIM domains 1	25177	-0.20706	9.48	-1.2556	0.23975	0.47881	-5.1573
Hrc	histidine rich calcium binding pro	292905	-0.16875	7.9583	-1.2543	0.24019	0.47899	-5.1586
Ndufb6	NADH:ubiquinone oxidoreducta	297990	-0.14451	9.1612	-1.2527	0.24076	0.47945	-5.1604
Slc37a4	solute carrier family 37 member	29573	-0.13645	5.1042	-1.2501	0.24167	0.48056	-5.1632
Apoa1	apolipoprotein A1	25081	-0.15317	7.2074	-1.2444	0.24365	0.48381	-5.1694
Txn2	thioredoxin 2	79462	-0.18454	5.7011	-1.2403	0.2451	0.48599	-5.1738
Smim26	small integral membrane protein	296207	-0.13125	3.8769	-1.239	0.24557	0.48619	-5.1752
Higd1a	"HIG1 hypoxia inducible domain	140937	0.17032	5.2444	1.237	0.24625	0.48619	-5.1773
Atp1b4	ATPase Na+/K+ transporting fa	84396	0.2611	6.3757	1.2366	0.24641	0.48619	-5.1778
Mbp	myelin basic protein	24547	0.27279	10.104	1.236	0.2466	0.48619	-5.1784
Grpel1		79563	-0.13564	7.2542	-1.2349	0.24702	0.48622	-5.1797
Fyco1	FYVE and coiled-coil domain au	301085	-0.12714	4.7568	-1.2333	0.24756	0.48622	-5.1813
Rpl9	ribosomal protein L9	29257	-0.19156	6.8678	-1.233	0.24767	0.48622	-5.1816
Plg	plasminogen	85253	0.12892	6.8712	1.2291	0.24907	0.48827	-5.1858
Jsrp1	junctional sarcoplasmic reticulum	690423	-0.2076	8.1373	-1.2265	0.25	0.4894	-5.1886
Pgam2	phosphoglycerate mutase 2	24959	0.14244	9.5006	1.2221	0.25159	0.49172	-5.1933
Prx	periaxin	78960	0.22023	9.7443	1.2213	0.25189	0.49172	-5.1942
Sema7a	semaphorin 7A (John Milton Ha	315711	-0.15054	4.7765	-1.2196	0.25249	0.4922	-5.196
Ssr3	signal sequence receptor subun	81784	0.15251	5.3789	1.2153	0.25405	0.49369	-5.2006
Tbc1d17	"TBC1 domain family, member 1	292886	-0.18197	6.6236	-1.2149	0.2542	0.49369	-5.201
Sun2	Sad1 and UNC84 domain conta	315135	-0.14629	6.5142	-1.2137	0.25465	0.49369	-5.2023
H2az1	H2A.Z variant histone 1	58940	-0.14771	5.4827	-1.213	0.2549	0.49369	-5.2031
Mavs	mitochondrial antiviral signaling	311430	0.15299	5.4804	1.2121	0.25524	0.49369	-5.204
Tbrg4	transforming growth factor beta	360977	-0.12874	5.0747	-1.2116	0.25539	0.49369	-5.2045
Wasf2	WASP family member 2	313024	0.137	4.2205	1.206	0.25747	0.49702	-5.2105

Rhog	ras homolog family member G	308875	-0.12923	6.0205	-1.1968	0.26087	0.50227	-5.2202
Chchd6	coiled-coil-helix-coiled-coil-helix d	297436	0.14229	5.5639	1.1966	0.26092	0.50227	-5.2204
Ndufb3	NADH:ubiquinone oxidoreducta	301427	-0.16768	10.43	-1.1857	0.265	0.50942	-5.2318
Picalm	phosphatidylinositol binding clat	89816	0.1224	4.3613	1.1842	0.26558	0.50982	-5.2334
Ilk	integrin-linked kinase	170922	-0.1234	3.8651	-1.183	0.26603	0.50998	-5.2347
Myl1	"myosin, light chain 1"	56781	-0.24787	12.96	-1.1786	0.26768	0.51244	-5.2392
Bpnt1	"3'(2'), 5'-bisphosphate nucleoti	64473	0.14404	5.3252	1.1766	0.26843	0.51305	-5.2413
Aldh5a1	"aldehyde dehydrogenase 5 far	291133	-0.34922	8.8798	-1.1757	0.26879	0.51305	-5.2422
Fkbp1a	FKBP prolyl isomerase 1A	25639	-0.14401	6.6486	-1.1749	0.26911	0.51305	-5.2431
Afg1l	AFG1 like ATPase	502479	-0.12671	4.3328	-1.1725	0.27001	0.51406	-5.2456
S100a6	S100 calcium binding protein A6	85247	0.13409	7.0204	1.1711	0.27057	0.51437	-5.2471
Sec23a	"Sec23 homolog A, COPII coat	58817	0.14026	4.0929	1.1701	0.27092	0.51437	-5.248
Bcat2	branched chain amino acid tran	64203	-0.2737	9.1564	-1.168	0.27172	0.51518	-5.2502
Map4	microtubule-associated protein 4	367171	0.14209	7.0041	1.1667	0.27223	0.51545	-5.2515
Acsl6	acyl-CoA synthetase long-chain	117243	-0.20346	9.7591	-1.1636	0.27343	0.51546	-5.2548
Chp1	calcineurin-like EF-hand protein	64152	-0.12764	4.4313	-1.1636	0.27344	0.51546	-5.2548
Ufsp2	UFM1-specific peptidase 2	361151	0.16095	4.5758	1.1623	0.27393	0.51546	-5.2561
Tmx3	thioredoxin-related transmembra	682967	0.14191	5.9666	1.1619	0.27409	0.51546	-5.2565
Rpl6	ribosomal protein L6	117042	0.14067	8.8822	1.1619	0.2741	0.51546	-5.2565
Ssr1	signal sequence receptor subur	361233	-0.23641	6.1983	-1.1597	0.27494	0.51636	-5.2588
Sec61g	Sec61 translocon gamma subur	689134	0.15197	4.768	1.1573	0.27587	0.51739	-5.2613
Ldhd	lactate dehydrogenase D	307858	0.14064	5.7301	1.1556	0.27655	0.51796	-5.263
Glg1	golgi glycoprotein 1	29476	-0.12125	4.7416	-1.152	0.27792	0.51984	-5.2667
Cd55	CD55 molecule (Cromer blood g	64036	0.44506	4.1391	1.1449	0.28071	0.52414	-5.2739
Adsl	adenylosuccinate lyase	315150	0.1182	4.3932	1.1437	0.2812	0.52414	-5.2752
Apmap	adipocyte plasma membrane as	366227	-0.13823	5.3965	-1.1424	0.28172	0.52414	-5.2765
Scarb2	"scavenger receptor class B, me	117106	-0.1642	7.3002	-1.1423	0.28174	0.52414	-5.2766
Mrpl10	mitochondrial ribosomal protein	691075	0.12771	3.8617	1.1404	0.28249	0.52476	-5.2785
Sec61b	Sec61 translocon beta subunit	298068	-0.15587	5.4605	-1.1396	0.28283	0.52476	-5.2794
Abcd3	ATP binding cassette subfamily	25270	-0.17126	5.2484	-1.1381	0.28341	0.52514	-5.2809
Sgca	"sarcoglycan, alpha"	303468	-0.13535	6.6718	-1.1349	0.28469	0.52677	-5.2841
Hadh	hydroxyacyl-CoA dehydrogenas	113965	-0.18517	11.048	-1.1322	0.28575	0.52677	-5.2868
Grb10	growth factor receptor bound pr	498416	0.15803	5.2578	1.1322	0.28578	0.52677	-5.2869
Smyd1	SET and MYND domain contain	297333	0.15882	5.7366	1.1321	0.28581	0.52677	-5.287
Pbxip1	PBX homeobox interacting prot	310644	-0.15398	8.9538	-1.123	0.28944	0.53276	-5.2961
Oxnad1	oxidoreductase NAD-binding do	306270	-0.11861	4.6648	-1.121	0.29026	0.53355	-5.2981
Rpn1	ribophorin I	25596	-0.12637	9.3148	-1.1178	0.29154	0.53423	-5.3013
Gng2	G protein subunit gamma 2	80850	0.12228	3.9913	1.1176	0.29164	0.53423	-5.3015
Mrpl39	mitochondrial ribosomal protein	684304	0.13211	5.4103	1.1169	0.29192	0.53423	-5.3022
Tpm1	tropomyosin 1	24851	-0.25138	11.63	-1.1163	0.29217	0.53423	-5.3028

Ap2b1	adaptor related protein complex	140670	0.13435	7.6279	1.1149	0.29275	0.53434	-5.3042
Cp	ceruloplasmin	24268	-0.18375	6.1852	-1.1143	0.293	0.53434	-5.3049
Mfn1	mitofusin 1	192647	-0.13118	6.3726	-1.1117	0.29403	0.53551	-5.3074
Nsfl1c	NSFL1 cofactor	83809	0.1464	6.3612	1.1105	0.29454	0.53575	-5.3086
Vkorc1l1	"vitamin K epoxide reductase co	399684	-0.14335	5.4676	-1.1072	0.29587	0.53745	-5.3119
Aoc3	"amine oxidase, copper contain	29473	0.11841	7.4837	1.1048	0.29689	0.5386	-5.3143
Pdpr	pyruvate dehydrogenase phosp	307852	-0.16822	8.6194	-1.1016	0.29819	0.54025	-5.3174
Rhoa	ras homolog family member A	117273	-0.12467	7.7604	-1.0967	0.30021	0.54321	-5.3223
Napa	NSF attachment protein alpha	140673	-0.12061	6.2543	-1.0945	0.30116	0.54406	-5.3245
Dnaja2	DnaJ heat shock protein family (	84026	0.1543	4.5346	1.0936	0.30152	0.54406	-5.3254
Capn1	calpain 1	29153	0.11346	3.8592	1.0924	0.30203	0.54406	-5.3266
Prkag1	protein kinase AMP-activated no	25520	-0.15172	5.3343	-1.0918	0.30225	0.54406	-5.3271
Manf	mesencephalic astrocyte-derived	315989	0.14267	3.7917	1.0908	0.30269	0.54414	-5.3281
Aspn	asporin	306805	-0.26749	7.1103	-1.0867	0.30442	0.54597	-5.3322
Ehhadh	enoyl-CoA hydratase and 3-hyd	171142	-0.1698	5.2399	-1.0854	0.30496	0.54597	-5.3334
Mrps25	mitochondrial ribosomal protein	297459	0.18224	5.4119	1.0854	0.30496	0.54597	-5.3334
Mipep	mitochondrial intermediate pepti	81684	-0.12248	6.3601	-1.0846	0.30529	0.54597	-5.3342
Myadm	myeloid-associated differentiatio	369016	-0.17222	4.4834	-1.0826	0.3061	0.54673	-5.3361
Bckdhb	branched chain keto acid dehyd	29711	-0.21714	7.8082	-1.0747	0.30947	0.55202	-5.3438
Rpl8	ribosomal protein L8	26962	0.16045	7.8965	1.0731	0.31015	0.55237	-5.3454
Dync1h1	dynein cytoplasmic 1 heavy cha	29489	0.13113	8.371	1.0724	0.31046	0.55237	-5.3461
Hsd17b10	hydroxysteroid (17-beta) dehydr	63864	-0.13915	9.5869	-1.0691	0.31186	0.55358	-5.3493
Mlec	malectin	304543	0.1494	6.5545	1.0688	0.31199	0.55358	-5.3496
Plin4	perilipin 4	363331	0.21505	9.0889	1.0668	0.31284	0.55358	-5.3515
Ckmt2	"creatine kinase, mitochondrial 2	688698	0.14054	12.536	1.0657	0.31331	0.55358	-5.3526
Pdlim7	PDZ and LIM domain 7	286908	0.16579	5.6058	1.065	0.31359	0.55358	-5.3532
Mrpl50	mitochondrial ribosomal protein	362517	-0.111	4.9987	-1.0647	0.31374	0.55358	-5.3535
Srpra	SRP receptor subunit alpha	315548	0.16421	5.158	1.0631	0.31444	0.55358	-5.3551
Xdh	xanthine dehydrogenase	497811	-0.24164	8.2774	-1.0617	0.31502	0.55358	-5.3564
Mccc1	methylcrotonoyl-CoA carboxylas	294972	-0.12745	7.9133	-1.0614	0.31518	0.55358	-5.3568
Mrpl46	mitochondrial ribosomal protein	293054	-0.13252	3.9744	-1.0608	0.31544	0.55358	-5.3573
Hacd3	3-hydroxyacyl-CoA dehydratase	300783	-0.12972	3.9271	-1.0605	0.31553	0.55358	-5.3575
Ap1b1	adaptor related protein complex	29663	0.10815	4.0946	1.0495	0.32033	0.56128	-5.3681
Stab1	stabilin 1	100363145	0.11088	3.8387	1.0467	0.32155	0.56226	-5.3708
Txnrd2	thioredoxin reductase 2	50551	0.12983	7.0341	1.0459	0.32191	0.56226	-5.3716
Cbr4	carbonyl reductase 4	359725	-0.16751	5.6335	-1.0454	0.3221	0.56226	-5.372
Glud1	glutamate dehydrogenase 1	24399	-0.11469	9.9789	-1.0445	0.32252	0.56228	-5.3729
Clcn1	chloride voltage-gated channel	25688	-0.12062	4.3221	-1.0415	0.32385	0.5639	-5.3758
Eci2	enoyl-CoA delta isomerase 2	291075	0.1328	9.344	1.0404	0.32432	0.564	-5.3768
Coq6	coenzyme Q6 monooxygenase	299195	0.14904	6.6152	1.0247	0.33131	0.57471	-5.3916

Rpl3	ribosomal protein L3	300079	0.1572	5.6169	1.024	0.3316	0.57471	-5.3922
Gtpbp3	GTP binding protein 3	290633	-0.12848	4.9078	-1.0234	0.33188	0.57471	-5.3928
Myl3	myosin light chain 3	24585	-0.20787	8.9775	-1.0228	0.33214	0.57471	-5.3933
Abhd6	abhydrolase domain containing	305795	-0.11431	4.0123	-1.013	0.33656	0.57998	-5.4025
Rtcb	"RNA 2',3'-cyclic phosphate and	362855	0.1211	6.1385	1.0128	0.33664	0.57998	-5.4026
Timm8b	translocase of inner mitochondri	64372	0.11201	5.7521	1.0124	0.33685	0.57998	-5.4031
Slmap	sarcolemma associated protein	290533	-0.12594	7.2115	-1.0124	0.33686	0.57998	-5.4031
Vps13c	vacuolar protein sorting 13 hom	363087	0.10516	5.1595	1.0101	0.33786	0.58099	-5.4051
Lamb2	laminin subunit beta 2	25473	-0.11773	4.295	-1.0054	0.34001	0.58396	-5.4095
Vtn	vitronectin	29169	0.12769	4.302	1.0036	0.34085	0.58451	-5.4112
Ahsg	alpha-2-HS-glycoprotein	25373	-0.2246	5.9733	-1.0029	0.34118	0.58451	-5.4119
Pfkm	"phosphofructokinase, muscle"	65152	0.29734	11.499	1.0003	0.34235	0.5858	-5.4142
Eif2a	eukaryotic translation initiation fa	502531	0.14814	3.8876	0.99425	0.34514	0.58957	-5.4198
Cap1	cyclase associated actin cytoske	64185	0.14342	5.7824	0.99368	0.34541	0.58957	-5.4203
Farsa	phenylalanyl-tRNA synthetase s	288917	-0.11829	6.0629	-0.98832	0.34789	0.59308	-5.4252
Bckdha	branched chain keto acid dehyd	25244	-0.13715	7.5482	-0.98642	0.34877	0.59386	-5.4269
Clybl	citrate lyase beta like	306198	-0.14628	6.9711	-0.98482	0.34952	0.59424	-5.4284
Cd44	CD44 molecule (Indian blood gr	25406	0.10885	4.2995	0.9841	0.34985	0.59424	-5.429
Sqor	sulfide quinone oxidoreductase	691966	-0.12608	5.6753	-0.98217	0.35076	0.5945	-5.4308
Tmem33	transmembrane protein 33	59303	0.10449	5.5585	0.98193	0.35087	0.5945	-5.431
Atp6v1e1	ATPase H+ transporting V1 sub	297566	0.10989	4.3139	0.9795	0.352	0.5957	-5.4332
Farsb	phenylalanyl-tRNA synthetase s	301544	0.12244	7.209	0.97463	0.35429	0.59803	-5.4376
Lman1	"lectin, mannose-binding, 1"	116666	0.12614	5.5781	0.97317	0.35498	0.59803	-5.4389
Chchd3	coiled-coil-helix-coiled-coil-helix d	296966	-0.12264	10.753	-0.97271	0.3552	0.59803	-5.4393
Pip4k2b	phosphatidylinositol-5-phosphat	89812	0.11874	5.6159	0.97217	0.35545	0.59803	-5.4398
Dnajc13	DnaJ heat shock protein family (	363127	-0.10013	5.2494	-0.97199	0.35554	0.59803	-5.44
Acat1	acetyl-CoA acetyltransferase 1	25014	-0.1681	11.594	-0.97049	0.35624	0.59849	-5.4413
Myh2	myosin heavy chain 2	691644	-0.1243	10.656	-0.96615	0.3583	0.60113	-5.4452
Tln2	talin 2	315776	0.20572	7.493	0.96535	0.35868	0.60113	-5.4459
Dbt	dihydrolipoamide branched chai	29611	-0.17093	9.1613	-0.96209	0.36023	0.603	-5.4488
Dnm1l	dynamin 1-like	114114	0.15835	5.3823	0.95913	0.36165	0.60339	-5.4515
Atp5f1a	ATP synthase F1 subunit alpha	65262	-0.13667	15.259	-0.95799	0.36219	0.60339	-5.4525
Emc3	ER membrane protein complex s	312640	-0.11486	6.086	-0.95796	0.36221	0.60339	-5.4525
Rock2	Rho-associated coiled-coil conta	25537	0.17358	3.926	0.95795	0.36221	0.60339	-5.4525
Rdh14	retinol dehydrogenase 14	500629	0.12449	5.0012	0.9564	0.36295	0.6039	-5.4539
Tnni2	"troponin I2, fast skeletal type"	29389	-0.21756	8.9476	-0.95373	0.36423	0.6053	-5.4562
Cct5	chaperonin containing TCP1 su	294864	-0.14553	5.8974	-0.94977	0.36614	0.60772	-5.4597
Cox7a2	cytochrome c oxidase subunit 7	29507	-0.11323	10.069	-0.9489	0.36656	0.60772	-5.4605
Gpd1l	glycerol-3-phosphate dehydroge	363159	0.10299	5.5038	0.94738	0.36729	0.6082	-5.4618
Maoa	monoamine oxidase A	29253	0.19399	7.4287	0.9454	0.36825	0.60906	-5.4636

Higd2a	"HIG1 hypoxia inducible domain	290999	-0.10257	5.6015	-0.94358	0.36913	0.6098	-5.4652
Ndufaf5	NADH:ubiquinone oxidoreducta	296190	0.10654	6.088	0.93908	0.37132	0.61268	-5.4691
Lyrm9	LYR motif containing 9	497962	0.12276	4.661	0.93407	0.37377	0.61578	-5.4735
Trap1	TNF receptor-associated protein	287069	0.12005	7.2073	0.93341	0.37409	0.61578	-5.4741
Dpp7	dipeptidylpeptidase 7	83799	-0.12231	6.0778	-0.9315	0.37503	0.61607	-5.4757
Tomm20	translocase of outer mitochondr	266601	-0.097067	4.2371	-0.93124	0.37516	0.61607	-5.476
Myom1	myomesin 1	316740	-0.11999	9.0634	-0.93011	0.37571	0.61626	-5.4769
Cs	citrate synthase	170587	-0.12253	12.229	-0.92918	0.37617	0.61628	-5.4777
Ywhae	"tyrosine 3-monooxygenase/tryp	29753	0.13936	8.9844	0.92334	0.37905	0.61927	-5.4828
Pgrmc2	progesterone receptor membran	361940	0.12663	7.0991	0.92288	0.37928	0.61927	-5.4832
Glul	glutamate-ammonia ligase	24957	-0.096395	4.1838	-0.92245	0.37949	0.61927	-5.4836
Crat	carnitine O-acetyltransferase	311849	-0.14887	11.281	-0.92186	0.37978	0.61927	-5.4841
Hspb1	heat shock protein family B (sma	24471	0.12539	7.6769	0.91732	0.38203	0.62126	-5.488
Mrpl21	mitochondrial ribosomal protein	309140	-0.10585	5.4556	-0.91621	0.38259	0.62126	-5.4889
Sypl1	synaptophysin-like 1	366595	-0.14165	5.2036	-0.9157	0.38284	0.62126	-5.4894
Acta1	"actin, alpha 1, skeletal muscle"	29437	-0.17641	13.023	-0.91563	0.38288	0.62126	-5.4894
Daglb	"diacylglycerol lipase, beta"	304289	0.10747	3.87	0.91489	0.38325	0.62126	-5.49
Steap3	STEAP3 metalloreductase	170824	0.099193	4.0218	0.91342	0.38398	0.62172	-5.4913
Nt5c1a	"5'-nucleotidase, cytosolic IA"	313574	0.16445	4.5579	0.91184	0.38477	0.62201	-5.4927
Atp5mg	ATP synthase membrane subur	300677	-0.13017	9.7132	-0.91126	0.38506	0.62201	-5.4931
Ndufaf1	NADH:ubiquinone oxidoreducta	296086	-0.11626	6.1772	-0.90652	0.38744	0.62512	-5.4972
Ndufv3	NADH:ubiquinone oxidoreducta	64539	-0.14578	7.3834	-0.90563	0.38788	0.62512	-5.4979
Tfrc	transferrin receptor	64678	0.24401	7.7167	0.90317	0.38912	0.62571	-5.5
Tmem70	transmembrane protein 70	500384	-0.15859	6.022	-0.90225	0.38958	0.62571	-5.5008
Acaa1a	acetyl-CoA acyltransferase 1A	24157	-0.14522	6.3958	-0.90221	0.3896	0.62571	-5.5008
Rpl34	ribosomal protein L34	362041	-0.15987	7.0158	-0.89677	0.39235	0.62855	-5.5054
Pdf	peptide deformylase (mitochond	690214	-0.11454	5.8749	-0.89646	0.39251	0.62855	-5.5057
Mybph	myosin binding protein H	83708	0.10064	4.9132	0.89602	0.39273	0.62855	-5.5061
Sec62	"SEC62 homolog, preprotein tra	294912	0.10817	6.0422	0.8939	0.39381	0.62903	-5.5078
Ldhb	lactate dehydrogenase B	24534	0.11552	6.7271	0.89288	0.39433	0.62903	-5.5087
Rpl35	ribosomal protein L35	296709	0.12424	7.2835	0.89276	0.39439	0.62903	-5.5088
Ndufa2	NADH:ubiquinone oxidoreducta	291660	0.11865	10.806	0.8901	0.39575	0.63046	-5.511
Cfh	complement factor H	155012	-0.12296	4.545	-0.88883	0.39639	0.63077	-5.5121
Hspd1	heat shock protein family D (Hsp	63868	-0.11721	11.617	-0.88496	0.39837	0.63277	-5.5153
Ywhaz	"tyrosine 3-monooxygenase/tryp	25578	0.12321	7.3723	0.88458	0.39856	0.63277	-5.5156
Rpl22	ribosomal protein L22	81768	0.1285	5.6389	0.88126	0.40027	0.63476	-5.5184
Gpt2	glutamic--pyruvic transaminase 2	307759	0.088555	5.4323	0.87948	0.40119	0.63548	-5.5199
Lemd2	LEM domain containing 2	361807	-0.09543	4.2195	-0.87622	0.40287	0.63741	-5.5225
Pdlim5	PDZ and LIM domain 5	64353	-0.11589	6.3615	-0.87375	0.40414	0.63871	-5.5246
Rap1b	"RAP1B, member of RAS oncog	171337	-0.098714	9.2521	-0.8698	0.40619	0.64121	-5.5278

Sema3c	semaphorin 3C	296787	-0.13765	4.9708	-0.86783	0.40722	0.6421	-5.5294
Mrpl16	mitochondrial ribosomal protein	293754	0.11689	5.6318	0.86477	0.40881	0.64358	-5.532
Nt5e	"5' nucleotidase, ecto"	58813	0.15979	6.0822	0.864	0.40921	0.64358	-5.5326
Iqgap1	IQ motif containing GTPase acti	361598	0.10064	7.3816	0.86335	0.40955	0.64358	-5.5331
Hsp90aa1	heat shock protein 90 alpha fan	299331	0.10692	4.1374	0.86089	0.41084	0.64487	-5.5351
Cfl1	cofilin 1	29271	0.17774	6.2042	0.85817	0.41226	0.6457	-5.5373
Letm1	leucine zipper and EF-hand con	305457	-0.099969	10.335	-0.8581	0.4123	0.6457	-5.5374
Ctnnd1	catenin delta 1	311163	0.10118	5.236	0.85647	0.41316	0.64591	-5.5387
Rap2c	"RAP2C, member of RAS oncog	302495	-0.10727	6.5955	-0.85608	0.41336	0.64591	-5.539
Mettl7a	methyltransferase like 7A	315306	-0.12266	6.6242	-0.85428	0.41431	0.64666	-5.5405
Rplp2	ribosomal protein lateral stalk su	140662	-0.13875	8.1592	-0.85302	0.41497	0.64697	-5.5415
Cd48	Cd48 molecule	245962	-0.12168	5.2725	-0.85043	0.41634	0.64836	-5.5436
Tspan8	tetraspanin 8	171048	-0.22093	7.2584	-0.84915	0.41702	0.64869	-5.5446
Rps6	ribosomal protein S6	29304	0.1571	7.7711	0.84246	0.42056	0.65347	-5.55
Rpl17	ribosomal protein L17	291434	-0.14745	7.9618	-0.84072	0.42149	0.65374	-5.5514
Rps24	ribosomal protein S24	81776	0.14546	5.8213	0.84037	0.42168	0.65374	-5.5516
Ssr4	signal sequence receptor subur	29435	-0.098502	4.8694	-0.8375	0.42321	0.65539	-5.5539
Pccb	propionyl-CoA carboxylase subu	24624	-0.10403	9.0393	-0.83534	0.42437	0.65644	-5.5556
Slc25a5	solute carrier family 25 member	25176	-0.093982	8.6225	-0.83216	0.42607	0.65834	-5.5581
Abhd11	abhydrolase domain containing	360831	-0.11569	6.2619	-0.83104	0.42667	0.65853	-5.559
Cisd2	CDGSH iron sulfur domain 2	295457	-0.11358	7.6046	-0.82631	0.42922	0.66173	-5.5628
Scpep1	serine carboxypeptidase 1	114861	-0.13122	3.9153	-0.82506	0.42989	0.66204	-5.5637
Obscn	"obscurin, cytoskeletal calmodul	338458	-0.087918	3.8123	-0.82359	0.43068	0.66252	-5.5649
Stac3	SH3 and cysteine rich domain 3	362895	-0.13428	5.9225	-0.82129	0.43193	0.66363	-5.5667
Prkaa2	protein kinase AMP-activated ca	78975	0.10228	6.6387	0.81978	0.43275	0.66363	-5.5679
Mecr	mitochondrial trans-2-enoyl-CoA	29470	0.1589	6.2786	0.8196	0.43285	0.66363	-5.568
Clpp	caseinolytic mitochondrial matrix	301117	-0.11491	6.2629	-0.81037	0.43788	0.66828	-5.5752
Rpl38	ribosomal protein L38	689284	-0.10504	6.6851	-0.80783	0.43926	0.66828	-5.5771
Coq10a	coenzyme Q10A	362810	-0.11483	8.5924	-0.80755	0.43942	0.66828	-5.5774
Dguok	deoxyguanosine kinase	297389	-0.087122	5.9081	-0.80746	0.43946	0.66828	-5.5774
Oxct1	3-oxoacid CoA transferase 1	690163	0.12947	9.4739	0.80735	0.43952	0.66828	-5.5775
Ist1	IST1 factor associated with ESC	307833	0.099642	5.7175	0.80648	0.44	0.66828	-5.5782
Rab5c	"RAB5C, member RAS oncogen	287709	0.087111	4.8679	0.80635	0.44007	0.66828	-5.5783
Map3k20	mitogen-activated protein kinase	311743	-0.10856	3.9592	-0.80602	0.44026	0.66828	-5.5785
Plgrkt	plasminogen receptor with a C-t	293888	-0.086935	6.9146	-0.80556	0.44051	0.66828	-5.5789
Ano6	anoctamin 6	315272	0.11165	5.2128	0.80521	0.4407	0.66828	-5.5791
Bcas1	breast carcinoma amplified sequ	246755	0.15496	4.8626	0.80253	0.44217	0.66978	-5.5812
Gys1	glycogen synthase 1	690987	0.088366	6.2818	0.80116	0.44293	0.6702	-5.5823
Acadl	"acyl-CoA dehydrogenase, long	25287	-0.19436	11.791	-0.79965	0.44376	0.67062	-5.5834
Naxe	NAD(P)HX epimerase	295229	-0.14231	6.3394	-0.7989	0.44418	0.67062	-5.584

Rab5a	"RAB5A, member RAS oncogen	64633	0.087414	5.6463	0.79528	0.44617	0.6729	-5.5867
Ces1d	carboxylesterase 1D	113902	-0.14528	5.4294	-0.78929	0.4495	0.67718	-5.5913
Galns	galactosamine (N-acetyl)-6-sulfa	292073	-0.10368	4.7089	-0.78499	0.4519	0.67986	-5.5945
Mrpl41	mitochondrial ribosomal protein	296551	0.12421	6.1427	0.78434	0.45226	0.67986	-5.595
Myoz1	myozenin 1	498440	-0.090198	6.9131	-0.78285	0.45309	0.68001	-5.5961
Fkbp3	FKBP prolyl isomerase 3	299104	-0.085964	5.9517	-0.78241	0.45334	0.68001	-5.5965
Hibch	3-hydroxyisobutyryl-CoA hydrola	301384	-0.12452	8.0086	-0.78046	0.45443	0.68091	-5.5979
Rpl7a	ribosomal protein L7a	296596	-0.09512	7.4593	-0.77943	0.45501	0.68104	-5.5987
Csrp3	cysteine and glycine rich protein	117505	0.39555	5.563	0.77793	0.45585	0.68145	-5.5998
Scn4b	sodium voltage-gated channel b	315611	-0.096681	5.5348	-0.77697	0.45639	0.68145	-5.6005
Rdx	radixin	315655	0.093469	5.8125	0.77631	0.45676	0.68145	-5.601
Rps16	ribosomal protein S16	140655	0.11328	7.104	0.76744	0.46176	0.68818	-5.6076
Psma3	proteasome 20S subunit alpha	29670	0.083402	4.8953	0.76396	0.46374	0.69012	-5.6101
Eif4g1	"eukaryotic translation initiation	287986	0.087404	4.9644	0.76339	0.46406	0.69012	-5.6106
Tm9sf4	transmembrane 9 superfamily m	296279	-0.1153	4.3367	-0.75729	0.46753	0.69405	-5.615
Glrx5	glutaredoxin 5	362776	0.12043	6.7145	0.75652	0.46798	0.69405	-5.6156
Mrpl23	mitochondrial ribosomal protein	64360	0.091088	4.2855	0.75538	0.46863	0.69405	-5.6164
Minpp1	multiple inositol-polyphosphate	29688	-0.080126	4.1028	-0.75524	0.46871	0.69405	-5.6165
Arf1	ADP-ribosylation factor 1	64310	-0.08641	5.3514	-0.74898	0.4723	0.69778	-5.621
Atpaf1	ATP synthase mitochondrial F1	313510	0.09525	4.4321	0.74863	0.4725	0.69778	-5.6213
Comtd1	catechol-O-methyltransferase do	305685	-0.10179	4.8596	-0.74821	0.47274	0.69778	-5.6216
Oat	ornithine aminotransferase	64313	0.14823	7.4171	0.74734	0.47324	0.69778	-5.6222
Gpx1	glutathione peroxidase 1	24404	-0.12229	5.4089	-0.74336	0.47553	0.70041	-5.6251
Mrps30	mitochondrial ribosomal protein	294767	-0.12823	5.3723	-0.74207	0.47628	0.70069	-5.626
Nos3	nitric oxide synthase 3	24600	0.17896	7.7092	0.74125	0.47675	0.70069	-5.6266
Rps4x	"ribosomal protein S4, X-linked"	100362640	0.15671	8.1805	0.74041	0.47724	0.70069	-5.6272
Hmgb1	high mobility group box 1	25459	-0.11594	5.7218	-0.73904	0.47803	0.70093	-5.6282
Rpl15	ribosomal protein L15	245981	0.091182	5.4227	0.73613	0.47972	0.70093	-5.6302
Tcp1	t-complex 1	24818	0.082048	5.9535	0.73609	0.47974	0.70093	-5.6303
Aars2	"alanyl-tRNA synthetase 2, mito	301254	-0.091571	4.1045	-0.73527	0.48022	0.70093	-5.6308
Ecsit	ECSIT signaling integrator	300447	-0.088464	5.7863	-0.73455	0.48064	0.70093	-5.6314
Kif5b	kinesin family member 5B	117550	-0.085991	6.2822	-0.73386	0.48103	0.70093	-5.6318
Mpz	myelin protein zero	24564	-0.20184	11.523	-0.73327	0.48138	0.70093	-5.6323
Basp1	"brain abundant, membrane atta	64160	0.07771	4.2234	0.73315	0.48145	0.70093	-5.6323
Psma2	proteasome 20S subunit alpha	29669	0.082625	4.1298	0.73027	0.48313	0.70153	-5.6344
Rpl19	ribosomal protein L19	81767	0.10727	6.6702	0.72905	0.48384	0.70153	-5.6352
Dnajc28	DnaJ heat shock protein family	360699	-0.077244	4.3577	-0.7286	0.4841	0.70153	-5.6356
Vti1b	vesicle transport through interac	100359512	0.10948	4.031	0.72799	0.48446	0.70153	-5.636
Myoz3	myozenin 3	689274	-0.085046	5.9089	-0.72756	0.48471	0.70153	-5.6363
Rpl28	ribosomal protein L28	64638	-0.11613	7.4511	-0.72723	0.4849	0.70153	-5.6365

Sacm1l	SAC1 like phosphatidylinositide	116482	-0.095048	7.1966	-0.7245	0.4865	0.70297	-5.6384
Vapb	VAMP associated protein B and	60431	0.10322	7.5134	0.72362	0.48701	0.70297	-5.6391
Fam210a	"family with sequence similarity 2	307343	-0.083874	8.0206	-0.72262	0.4876	0.70297	-5.6398
Rac1	Rac family small GTPase 1	363875	-0.08637	8.856	-0.72208	0.48792	0.70297	-5.6401
Atp6v1b2	ATPase H+ transporting V1 sub	117596	0.099011	4.9798	0.71957	0.48939	0.70436	-5.6419
Slc27a1	solute carrier family 27 member	94172	-0.083051	5.6766	-0.71515	0.49199	0.70737	-5.645
Sptb	"spectrin, beta, erythrocytic"	314251	0.13188	10.911	0.71424	0.49253	0.7074	-5.6456
Endog	endonuclease G	362100	0.10679	5.4044	0.7097	0.49521	0.7105	-5.6487
Hexb	hexosaminidase subunit beta	294673	-0.091667	6.0964	-0.70861	0.49586	0.7105	-5.6495
Susd2	sushi domain containing 2	294335	0.10123	5.5297	0.70752	0.4965	0.7105	-5.6502
Skp1	S-phase kinase-associated prot	287280	0.074856	4.2768	0.70635	0.4972	0.7105	-5.651
Sar1b	"secretion associated, Ras relat	287276	0.085954	6.3375	0.70627	0.49725	0.7105	-5.6511
Cab39	calcium binding protein 39	301574	-0.085853	4.8	-0.70167	0.49998	0.71367	-5.6542
Rpl29	ribosomal protein L29	29283	-0.17159	7.3489	-0.69849	0.50188	0.71564	-5.6564
Rpl11	ribosomal protein L11	362631	0.13624	7.3792	0.69668	0.50296	0.71645	-5.6576
Sod1	superoxide dismutase 1	24786	-0.10654	7.8241	-0.69227	0.5056	0.71947	-5.6606
Ccdc47	coiled-coil domain containing 47	303606	-0.079077	7.5721	-0.69134	0.50616	0.71953	-5.6612
Timm44	translocase of inner mitochondri	29635	-0.11389	7.993	-0.68955	0.50723	0.72032	-5.6624
Grsf1	G-rich RNA sequence binding fa	305256	0.090629	4.9512	0.6866	0.50901	0.72209	-5.6644
Myh7	myosin heavy chain 7	29557	-0.1283	12.004	-0.6816	0.51203	0.72498	-5.6677
Dnase1l1	deoxyribonuclease 1-like 1	363522	0.088561	4.4805	0.6815	0.51209	0.72498	-5.6678
Mrpl2	mitochondrial ribosomal protein	301240	-0.072591	4.5397	-0.67875	0.51375	0.72658	-5.6696
Pc	pyruvate carboxylase	25104	-0.088256	8.5332	-0.67591	0.51547	0.72803	-5.6715
Myot	myotilin	291605	-0.080036	6.6219	-0.67533	0.51582	0.72803	-5.6718
Lgals3	galectin 3	83781	0.071914	5.8383	0.67253	0.51753	0.72968	-5.6737
Nubpl	nucleotide binding protein-like	299008	0.10799	4.8289	0.67128	0.51828	0.72968	-5.6745
Kcnma1	potassium calcium-activated cha	83731	0.091249	4.3807	0.67081	0.51857	0.72968	-5.6748
Dap3	death associated protein 3	295238	0.076008	5.3664	0.66683	0.521	0.73117	-5.6774
Slc25a46	"solute carrier family 25, membe	291709	-0.071653	5.8661	-0.66601	0.5215	0.73117	-5.6779
Ank1	ankyrin 1	306570	0.084871	9.4404	0.66511	0.52205	0.73117	-5.6785
Rpl13a	ribosomal protein L13A	317646	-0.09291	7.4367	-0.6646	0.52237	0.73117	-5.6789
Stim1	stromal interaction molecule 1	361618	-0.089668	6.6389	-0.6645	0.52242	0.73117	-5.6789
Psma7	proteasome 20S subunit alpha	29674	-0.073257	5.69	-0.66351	0.52303	0.73117	-5.6796
LOC1009107	aldose reductase-related protei	100910708	-0.20408	5.9699	-0.66304	0.52332	0.73117	-5.6799
Hsdl1	hydroxysteroid dehydrogenase	361418	-0.079821	4.6473	-0.65958	0.52544	0.7334	-5.6821
Rpl7	ribosomal protein L7	297755	0.094829	8.2349	0.65009	0.53129	0.74046	-5.6882
Acyp2	acylphosphatase 2	364224	0.10829	6.4999	0.64963	0.53157	0.74046	-5.6885
Rpl35a	ribosomal protein L35a	100359498	0.11677	5.7038	0.64748	0.5329	0.74157	-5.6898
Pdia3	"protein disulfide isomerase fam	29468	-0.07706	8.9721	-0.6459	0.53388	0.74218	-5.6908
Iba57	iron-sulfur cluster assembly facto	363611	-0.10976	5.2525	-0.64436	0.53484	0.7424	-5.6918

Ucp3	uncoupling protein 3	25708	0.10653	6.4338	0.64392	0.5351	0.7424	-5.6921
Itgb1	integrin subunit beta 1	24511	0.073422	6.5237	0.63579	0.54017	0.74867	-5.6972
Gstm2	glutathione S-transferase mu 2	24424	-0.082911	5.2824	-0.63111	0.54309	0.75198	-5.7001
Mrpl37	mitochondrial ribosomal protein	56281	-0.074029	5.4031	-0.62907	0.54437	0.75211	-5.7013
Nid1	nidogen 1	25494	-0.12355	3.9765	-0.62857	0.54468	0.75211	-5.7016
Pa2g4	proliferation-associated 2G4	288778	0.094569	6.6146	0.62835	0.54482	0.75211	-5.7018
LOC1009114	mitochondrial glutamate carrier 1	100911440	-0.066555	6.2371	-0.6263	0.54611	0.75314	-5.703
Oxsm	"3-oxoacyl-ACP synthase, mitoc	289934	0.098176	5.5767	0.62411	0.54748	0.75428	-5.7044
Capza2	capping actin protein of muscle	493810	0.11561	6.6451	0.62269	0.54838	0.75477	-5.7053
Nqo1	NAD(P)H quinone dehydrogena	24314	-0.1544	3.7441	-0.62146	0.54915	0.75508	-5.706
Mrpl12	mitochondrial ribosomal protein	303746	0.081491	7.2777	0.61862	0.55095	0.75562	-5.7077
Dhodh	dihydroorotate dehydrogenase	65156	0.071957	6.5036	0.61848	0.55103	0.75562	-5.7078
Fermt2	fermitin family member 2	289992	0.07781	4.9526	0.61802	0.55132	0.75562	-5.7081
Tmem126b	transmembrane protein 126B	293114	-0.069648	3.8728	-0.61738	0.55172	0.75562	-5.7085
Cpox	coproporphyrinogen oxidase	304024	0.068728	5.3649	0.61383	0.55397	0.75795	-5.7106
Foxred1	FAD-dependent oxidoreductase	315547	0.12553	5.3446	0.60815	0.55758	0.76213	-5.7141
Stom	stomatin	296655	0.077761	6.2952	0.60292	0.56091	0.76543	-5.7172
Fkbp8	FKBP prolyl isomerase 8	290652	0.067498	4.3948	0.60262	0.5611	0.76543	-5.7173
Prkar2a	protein kinase cAMP-dependen	29699	0.08288	5.0649	0.60049	0.56245	0.76559	-5.7186
Mrps16	mitochondrial ribosomal protein	688912	-0.065318	5.2538	-0.60046	0.56248	0.76559	-5.7186
Eif3j	"eukaryotic translation initiation	691947	0.093646	5.982	0.59985	0.56287	0.76559	-5.719
Pdk2	pyruvate dehydrogenase kinase	81530	0.10459	8.5672	0.5979	0.56411	0.76644	-5.7201
Tnnc1	"troponin C1, slow skeletal and	290561	-0.12916	5.6497	-0.59714	0.5646	0.76644	-5.7206
Mul1	mitochondrial E3 ubiquitin prote	298576	-0.074685	4.905	-0.59466	0.56619	0.76714	-5.722
Ndufb8	NADH:ubiquinone oxidoreducta	293991	0.074325	8.9541	0.59461	0.56622	0.76714	-5.7221
Vcp	valosin-containing protein	116643	0.10394	12.455	0.5921	0.56783	0.76856	-5.7235
Rps11	ribosomal protein S11	81774	0.13311	8.1754	0.58997	0.5692	0.76967	-5.7248
Emc4	ER membrane protein complex s	296049	-0.066734	5.2617	-0.58786	0.57056	0.77075	-5.726
Myof	myoferlin	309499	0.059669	4.299	0.58662	0.57135	0.77093	-5.7267
Epb42	erythrocyte membrane protein b	362202	-0.10517	8.7009	-0.58593	0.5718	0.77093	-5.7271
Cycs	"cytochrome c, somatic"	25309	-0.07671	11.749	-0.58397	0.57306	0.77188	-5.7283
Naxd	NAD(P)HX dehydratase	361185	0.076483	4.8712	0.58034	0.57541	0.77429	-5.7303
Pla2g6	phospholipase A2 group VI	360426	-0.063242	3.8583	-0.57185	0.58091	0.78094	-5.7352
Kyat3	kynurenine aminotransferase 3	541589	0.097613	7.0705	0.56517	0.58527	0.78603	-5.7389
Slc30a9	solute carrier family 30 member	498358	-0.063366	6.4399	-0.56219	0.58721	0.78787	-5.7406
Hras	"HRas proto-oncogene, GTPase	293621	-0.069067	6.4599	-0.55987	0.58873	0.78837	-5.7419
Fscn1	fascin actin-bundling protein 1	683788	-0.1151	4.4483	-0.55852	0.58962	0.78837	-5.7426
Rps20	ribosomal protein S20	122772	0.078856	6.3066	0.55825	0.58979	0.78837	-5.7428
Ethe1	"ETHE1, persulfide dioxygenase	292710	-0.093917	7.0096	-0.55815	0.58986	0.78837	-5.7428
Mrpl28	mitochondrial ribosomal protein	497876	-0.064856	6.0277	-0.55729	0.59042	0.78837	-5.7433

Ivd	isovaleryl-CoA dehydrogenase	24513	0.12746	9.705	0.55389	0.59266	0.79016	-5.7452
Isca1	iron-sulfur cluster assembly 1	290985	-0.062177	5.0705	-0.55322	0.5931	0.79016	-5.7455
Rmdn1	regulator of microtubule dynami	500419	-0.064436	6.1318	-0.55246	0.5936	0.79016	-5.746
Mybpc2	myosin binding protein C2	292879	-0.099237	11.057	-0.55178	0.59405	0.79016	-5.7463
Flad1	flavin adenine dinucleotide synt	751787	-0.074534	4.2789	-0.54981	0.59534	0.79091	-5.7474
Gpx4	glutathione peroxidase 4	29328	0.090048	6.267	0.54881	0.596	0.79091	-5.7479
Uba1	ubiquitin-like modifier activating	314432	0.070223	6.3662	0.54833	0.59632	0.79091	-5.7482
Tnnc2	"troponin C2, fast skeletal type"	296369	-0.12825	8.8625	-0.54665	0.59743	0.79162	-5.7491
Ran	"RAN, member RAS oncogene f	84509	0.068339	4.1039	0.54504	0.59849	0.79209	-5.75
Emc7	ER membrane protein complex s	296050	-0.077126	6.4651	-0.54438	0.59893	0.79209	-5.7503
Gadd45gip1	GADD45G interacting protein 1	288916	-0.06489	5.2019	-0.54327	0.59966	0.79224	-5.7509
Cav1	caveolin 1	25404	0.095673	8.1327	0.54248	0.60018	0.79224	-5.7514
Eif2s2	eukaryotic translation initiation fa	296302	-0.11849	5.1378	-0.54005	0.60179	0.79361	-5.7527
Pycr3	pyrroline-5-carboxylate reductas	300035	0.069412	4.5376	0.53524	0.60498	0.79639	-5.7552
Wdr1	WD repeat domain 1	360950	-0.059298	5.882	-0.53429	0.60561	0.79639	-5.7557
Ppa2	inorganic pyrophosphatase 2	310856	-0.068774	7.4074	-0.53393	0.60586	0.79639	-5.7559
Rab5b	"RAB5B, member RAS oncogen	288779	0.062989	6.3078	0.53341	0.6062	0.79639	-5.7562
Hba1	"hemoglobin, alpha 1"	25632	-0.099947	10.514	-0.5283	0.6096	0.8001	-5.7589
Slc2a4	solute carrier family 2 member 4	25139	0.07551	7.2877	0.52404	0.61245	0.80233	-5.7611
Plp1	proteolipid protein 1	24943	-0.086382	4.2358	-0.52403	0.61245	0.80233	-5.7611
Sntb1	"syntrophin, beta 1"	299940	0.055527	5.9354	0.51601	0.61783	0.8086	-5.7653
Aifm1	"apoptosis inducing factor, mito	83533	-0.059795	10.261	-0.51448	0.61886	0.80918	-5.7661
Pam16	presequence translocase assoc	679907	-0.055156	7.9699	-0.5119	0.62059	0.81069	-5.7674
Gde1	glycerophosphodiester phospho	60418	-0.055942	5.3014	-0.51007	0.62183	0.81094	-5.7683
Osbp	oxysterol binding protein	365410	0.053522	7.0799	0.50989	0.62195	0.81094	-5.7684
Tars2	"threonyl-tRNA synthetase 2, mi	310672	-0.05281	6.7281	-0.50827	0.62304	0.81097	-5.7692
Coa3	cytochrome C oxidase assembly	498000	-0.056095	7.5992	-0.5078	0.62336	0.81097	-5.7695
Cox19	cytochrome c oxidase assembly	304330	0.052543	4.3408	0.50725	0.62373	0.81097	-5.7698
Atp5me	ATP synthase membrane subun	140608	-0.063514	12.036	-0.50556	0.62487	0.81156	-5.7706
Apoa4	apolipoprotein A4	25080	-0.053651	4.6897	-0.50484	0.62536	0.81156	-5.771
Tmlhe	"trimethyllysine hydroxylase, eps	170898	-0.067273	4.5872	-0.50062	0.62822	0.81451	-5.7731
Ca3	carbonic anhydrase 3	54232	0.11547	9.7574	0.49928	0.62913	0.81456	-5.7738
Pdp1	pyruvate dehyrogenase phosph	54705	0.077536	7.8891	0.49882	0.62944	0.81456	-5.774
Rps27l	ribosomal protein S27-like	681429	-0.061587	3.9684	-0.49628	0.63116	0.81544	-5.7752
Ehd2	EH-domain containing 2	361512	-0.06677	9.7915	-0.49608	0.63129	0.81544	-5.7753
Rps19	ribosomal protein S19	108348115	0.08849	8.2992	0.49028	0.63524	0.81977	-5.7782
Gstz1	glutathione S-transferase zeta 1	681913	0.14418	6.7353	0.48673	0.63766	0.82159	-5.7799
Camk2a	calcium/calmodulin-dependent p	25400	-0.070964	7.7385	-0.48648	0.63783	0.82159	-5.78
LOC684270	similar to isochorismatase doma	684270	0.13973	6.0289	0.48239	0.64062	0.82442	-5.782
Dhrs7	dehydrogenase/reductase 7	299135	-0.055968	5.0609	-0.4808	0.64172	0.82491	-5.7828

Retsat	retinol saturase	246298	-0.055425	5.0186	-0.4801	0.6422	0.82491	-5.7831
Pcyt1a	"phosphate cytidylyltransferase	140544	-0.067294	5.9955	-0.47836	0.64338	0.82568	-5.784
Arpc1b	"actin related protein 2/3 comple	54227	0.093592	5.4781	0.47513	0.6456	0.82776	-5.7855
Apool	apolipoprotein O-like	317191	-0.052004	8.8261	-0.47283	0.64718	0.82784	-5.7866
Rpl18	ribosomal protein L18	81766	0.079777	7.5417	0.47275	0.64723	0.82784	-5.7866
Nfs1	NFS1 cysteine desulfurase	84594	0.055298	6.4875	0.47185	0.64785	0.82784	-5.787
Rps10	ribosomal protein S10	81773	0.066652	6.2678	0.47155	0.64806	0.82784	-5.7872
Bcl2l13	BCL2 like 13	312682	-0.066679	5.5811	-0.47064	0.64869	0.82788	-5.7876
Ddost	dolichyl-diphosphooligosacchari	313648	-0.051658	7.0618	-0.46969	0.64934	0.82795	-5.7881
Alb	albumin	24186	-0.05192	11.879	-0.4682	0.65037	0.82812	-5.7888
Sdha	succinate dehydrogenase comp	157074	-0.054392	12.539	-0.46725	0.65102	0.82812	-5.7892
Rps14	ribosomal protein S14	29284	-0.055681	5.6081	-0.46674	0.65138	0.82812	-5.7894
Eef1a1	eukaryotic translation elongatior	171361	0.064367	6.4071	0.46603	0.65186	0.82812	-5.7898
Pex14	peroxisomal biogenesis factor 1	64460	-0.050055	4.922	-0.46481	0.65271	0.82844	-5.7903
Tapbp	TAP binding protein	25217	-0.088727	4.4084	-0.46203	0.65462	0.83011	-5.7916
Vcl	vinculin	305679	0.060809	7.2654	0.45886	0.65682	0.83213	-5.7931
Rala	RAS like proto-oncogene A	81757	-0.067131	7.6691	-0.4531	0.66081	0.83642	-5.7957
Fech	ferrochelatase	361338	0.05963	8.6582	0.45055	0.66258	0.8379	-5.7969
Dnajc3	DnaJ heat shock protein family (	63880	0.046495	5.0641	0.44893	0.66371	0.83856	-5.7976
Dnajc30	DnaJ heat shock protein family (	368190	-0.056703	4.2663	-0.44708	0.665	0.83875	-5.7984
Erp44	endoplasmic reticulum protein 4	298066	0.048588	4.6511	0.44691	0.66512	0.83875	-5.7985
Ctsa	cathepsin A	296370	-0.061538	4.9802	-0.44525	0.66627	0.83875	-5.7993
Ndufa13	NADH:ubiquinone oxidoreducta	100911483	0.057954	11.062	0.44524	0.66628	0.83875	-5.7993
Psmd14	"proteasome 26S subunit, non-A	311078	-0.045774	3.8624	-0.43784	0.67145	0.84375	-5.8025
Eprs	glutamyl-prolyl-tRNA synthetase	289352	-0.055486	10.03	-0.43781	0.67147	0.84375	-5.8026
Gls	glutaminase	24398	-0.048701	4.8011	-0.43547	0.6731	0.84504	-5.8036
Agpat3	1-acylglycerol-3-phosphate O-ac	294324	-0.090655	4.3439	-0.434	0.67413	0.84556	-5.8042
Ap2a2	adaptor related protein complex	81637	0.048022	7.4075	0.43255	0.67515	0.84608	-5.8049
Cand1	cullin-associated and neddylatic	117152	-0.060907	4.2365	-0.43004	0.67691	0.84733	-5.8059
Pacsin3	protein kinase C and casein kina	311187	0.050521	9.1667	0.42938	0.67738	0.84733	-5.8062
Comt	catechol-O-methyltransferase	24267	-0.049818	6.1128	-0.423	0.68186	0.85031	-5.809
Nomo1	nodal modulator 1	361578	-0.046641	7.6356	-0.42231	0.68235	0.85031	-5.8092
Mylk2	myosin light chain kinase 2	117558	0.1013	4.7265	0.4214	0.68299	0.85031	-5.8096
Ccdc127	coiled-coil domain containing 12	308060	-0.062757	5.1909	-0.42048	0.68364	0.85031	-5.81
Nefl	neurofilament light	83613	-0.10381	4.0942	-0.41996	0.684	0.85031	-5.8102
Mpst	mercaptopyruvate sulfurtransfer	192172	0.05688	7.9666	0.41978	0.68413	0.85031	-5.8103
Ptrh2	peptidyl-tRNA hydrolase 2	287593	-0.045673	5.7246	-0.41955	0.6843	0.85031	-5.8104
Vamp5	vesicle-associated membrane pr	89818	-0.064276	6.6294	-0.41724	0.68592	0.85031	-5.8114
Eloc	elongin C	64525	-0.055665	4.4398	-0.41695	0.68613	0.85031	-5.8115
Bphl	biphenyl hydrolase like	361239	-0.053907	5.3771	-0.41652	0.68643	0.85031	-5.8117

Bsg	basigin (Ok blood group)	25246	-0.045237	8.4869	-0.41641	0.68651	0.85031	-5.8117
Hrg	histidine-rich glycoprotein	171016	-0.073791	6.695	-0.41324	0.68875	0.85154	-5.8131
Trabd	TraB domain containing	300142	0.044696	4.801	0.41096	0.69037	0.85154	-5.814
Nlrx1	NLR family member X1	315599	-0.044392	8.6749	-0.41094	0.69038	0.85154	-5.814
Pomgnt2	"protein O-linked mannose N-ac	316091	-0.064339	5.1894	-0.4108	0.69048	0.85154	-5.8141
Ndufb7	NADH:ubiquinone oxidoreducta	361385	0.04908	9.3584	0.41067	0.69057	0.85154	-5.8141
Reep5	receptor accessory protein 5	364838	-0.097324	7.8564	-0.40934	0.69151	0.85194	-5.8147
Abca2	ATP binding cassette subfamily	79248	-0.042865	4.0081	-0.40644	0.69357	0.85346	-5.8159
Uqcrq	"ubiquinol-cytochrome c reducta	497902	-0.055252	9.7607	-0.40586	0.69398	0.85346	-5.8161
Cops8	COP9 signalosome subunit 8	363283	-0.043836	3.9171	-0.40335	0.69576	0.85397	-5.8171
Atl3	atlastin GTPase 3	309187	0.044578	4.7437	0.40286	0.69611	0.85397	-5.8173
Dnaja1	DnaJ heat shock protein family (	65028	0.047538	4.0889	0.40243	0.69642	0.85397	-5.8175
Dad1	defender against cell death 1	192275	0.04511	4.9235	0.40182	0.69685	0.85397	-5.8177
Rpl10a	ribosomal protein L10A	81729	0.06299	8.6138	0.40018	0.69802	0.85415	-5.8184
Hk1	hexokinase 1	25058	0.049247	5.0532	0.39874	0.69904	0.85415	-5.819
Slc4a1	solute carrier family 4 member 1	24779	0.096076	10.676	0.39814	0.69947	0.85415	-5.8192
Klhl31	kelch-like family member 31	315833	-0.051051	5.7372	-0.39814	0.69947	0.85415	-5.8192
Bola3	bolA family member 3	297388	0.060072	6.1803	0.39616	0.70088	0.85477	-5.82
Fh	fumarate hydratase	24368	-0.051227	11.44	-0.39558	0.7013	0.85477	-5.8202
Stip1	stress-induced phosphoprotein	192277	-0.052347	5.5281	-0.39483	0.70183	0.85477	-5.8205
Cox5b	cytochrome c oxidase subunit 5	94194	-0.050008	12.776	-0.39295	0.70317	0.85566	-5.8213
Dnaja3	DnaJ heat shock protein family (	360481	-0.055105	8.4091	-0.38291	0.71035	0.86364	-5.8252
Ak3	adenylate kinase 3	26956	-0.04809	7.5287	-0.3777	0.71409	0.86742	-5.8272
Esyt2	extended synaptotagmin 2	299488	0.04318	5.0098	0.37683	0.71471	0.86742	-5.8275
Mtarc2	mitochondrial amidoxime reducir	171451	0.051117	6.805	0.37509	0.71596	0.86818	-5.8282
Rpl10	ribosomal protein L10	81764	-0.062915	8.3654	-0.37229	0.71798	0.86875	-5.8293
Acaa2	acetyl-CoA acyltransferase 2	170465	-0.0493	11.462	-0.37198	0.7182	0.86875	-5.8294
Cars2	"cysteinyl-tRNA synthetase 2, m	361184	0.045816	4.7898	0.37182	0.71832	0.86875	-5.8294
Dag1	dystroglycan 1	114489	-0.052545	7.0955	-0.3704	0.71934	0.86922	-5.83
Ncam1	neural cell adhesion molecule 1	24586	0.087917	6.0204	0.36876	0.72052	0.86922	-5.8306
Pacsin2	protein kinase C and casein kin	124461	-0.039327	5.8029	-0.36841	0.72077	0.86922	-5.8307
Col6a2	collagen type VI alpha 2 chain	361821	0.059208	7.2771	0.36702	0.72177	0.86922	-5.8312
Acsf3	acyl-CoA synthetase family mem	498962	0.064089	6.535	0.36693	0.72184	0.86922	-5.8313
Apoh	apolipoprotein H	287774	-0.063587	9.1122	-0.36574	0.7227	0.8695	-5.8317
Aldh4a1	"aldehyde dehydrogenase 4 far	641316	0.053934	8.7958	0.36429	0.72375	0.87	-5.8322
Pigs	"phosphatidylinositol glycan anc	303277	0.042842	5.0919	0.36148	0.72578	0.87124	-5.8333
Tagln2	transgelin 2	304983	0.063655	5.9114	0.36113	0.72603	0.87124	-5.8334
Pmpcb	"peptidase, mitochondrial proce	64198	-0.044458	7.3815	-0.35933	0.72733	0.87204	-5.8341
Tf	transferrin	24825	0.049924	8.7813	0.35744	0.7287	0.87293	-5.8347
Psma5	proteasome 20S subunit alpha	29672	-0.043475	4.2271	-0.3564	0.72945	0.87307	-5.8351

Tmem126a	transmembrane protein 126A	293113	-0.043856	7.1714	-0.35447	0.73085	0.87399	-5.8358
Rap1a	"RAP1A, member of RAS oncog	295347	-0.058379	7.2348	-0.3521	0.73257	0.87418	-5.8367
Abca8a	"ATP-binding cassette, subfamil	303638	-0.049269	8.8147	-0.35059	0.73367	0.87418	-5.8372
Rpl23	ribosomal protein L23	29282	0.039194	6.7093	0.34887	0.73492	0.87418	-5.8378
Atp5f1b	ATP synthase F1 subunit beta	171374	-0.05261	15.19	-0.34694	0.73632	0.87418	-5.8385
Camk2b	calcium/calmodulin-dependent p	24245	0.043467	7.2679	0.34663	0.73654	0.87418	-5.8386
Preb	prolactin regulatory element bin	58842	-0.045177	4.592	-0.34652	0.73663	0.87418	-5.8386
Ube2n	ubiquitin-conjugating enzyme E2	116725	0.049926	5.0634	0.34453	0.73807	0.87418	-5.8393
Gsr	glutathione-disulfide reductase	116686	-0.055363	5.1384	-0.3435	0.73882	0.87418	-5.8397
Tefm	"transcription elongation factor,	287554	0.0396	5.8983	0.34314	0.73909	0.87418	-5.8398
Gng5	G protein subunit gamma 5	79218	-0.036912	5.1508	-0.34234	0.73967	0.87418	-5.8401
Tfam	"transcription factor A, mitochon	83474	-0.052247	8.1078	-0.34211	0.73983	0.87418	-5.8402
Itga6	integrin subunit alpha 6	114517	-0.039427	4.1372	-0.34175	0.7401	0.87418	-5.8403
Tmed7	transmembrane p24 trafficking p	252889	0.036348	4.5884	0.34162	0.74019	0.87418	-5.8403
Eef1a2	eukaryotic translation elongation	24799	-0.087186	10.803	-0.34125	0.74046	0.87418	-5.8405
Mpc2	mitochondrial pyruvate carrier 2	100359982	-0.052111	6.4989	-0.34067	0.74088	0.87418	-5.8407
Tubb2a	"tubulin, beta 2A class IIa"	498736	0.039006	4.5785	0.33978	0.74153	0.87418	-5.841
Mrps31	mitochondrial ribosomal protein	290850	0.050868	4.7597	0.33951	0.74173	0.87418	-5.8411
Dlst	dihydrolipoamide S-succinyltrans	299201	-0.055982	10.815	-0.3358	0.74443	0.87662	-5.8423
Eif5a	eukaryotic translation initiation fa	287444	0.042882	6.2213	0.3346	0.74531	0.87691	-5.8427
Atad1	"ATPase family, AAA domain co	309532	-0.038804	7.4917	-0.33317	0.74636	0.8774	-5.8432
Anxa3	annexin A3	25291	0.040799	6.3441	0.32781	0.75028	0.88126	-5.845
Plp2	proteolipid protein 2	302562	0.054778	5.0805	0.32565	0.75186	0.88171	-5.8457
Camk2d	calcium/calmodulin-dependent p	24246	-0.04293	5.493	-0.32555	0.75194	0.88171	-5.8458
Emc1	ER membrane protein complex s	362643	-0.03705	6.9387	-0.32459	0.75264	0.88179	-5.8461
Tm9sf2	transmembrane 9 superfamily m	306197	0.035371	3.9338	0.31857	0.75706	0.88565	-5.848
Emc8	ER membrane protein complex s	361425	-0.045877	6.6652	-0.31836	0.75721	0.88565	-5.8481
Dhrs7b	dehydrogenase/reductase 7B	287380	-0.034491	4.0816	-0.31618	0.75881	0.88677	-5.8488
Gstp1	glutathione S-transferase pi 1	24426	0.048218	5.7795	0.31505	0.75964	0.88677	-5.8492
Mmab	metabolism of cobalamin associ	687861	-0.048825	5.0465	-0.31365	0.76067	0.88677	-5.8496
LOC687508	"similar to Cytochrome c oxidase	687508	-0.069156	7.2636	-0.31358	0.76073	0.88677	-5.8496
Gpd2	glycerol-3-phosphate dehydroge	25062	-0.055189	10.736	-0.31051	0.76299	0.88866	-5.8506
Gpi	glucose-6-phosphate isomerase	292804	0.042621	8.0406	0.30958	0.76367	0.88871	-5.8509
Prelp	proline and arginine rich end leu	84400	0.038285	6.6251	0.30767	0.76508	0.8896	-5.8515
Klhl41	kelch-like family member 41	117537	-0.03493	7.3126	-0.30443	0.76747	0.89091	-5.8525
Hspa9	heat shock protein family A (Hsp	291671	0.051789	11.063	0.30379	0.76794	0.89091	-5.8527
Add1	adducin 1	24170	0.044586	4.7267	0.30352	0.76814	0.89091	-5.8528
Lpl	lipoprotein lipase	24539	-0.040146	5.3801	-0.30187	0.76936	0.89116	-5.8533
Hyou1	hypoxia up-regulated 1	192235	0.036427	5.1702	0.30032	0.77051	0.89116	-5.8538
Hsdl2	hydroxysteroid dehydrogenase	313200	-0.039147	9.0293	-0.30007	0.77069	0.89116	-5.8538

Rpl3l	ribosomal protein L3-like	287122	0.039528	8.2785	0.29976	0.77092	0.89116	-5.8539
Ptp4a2	protein tyrosine phosphatase 4A	85237	0.032657	5.3229	0.29738	0.77268	0.89245	-5.8547
Pls3	plastin 3	81748	0.031851	4.9824	0.29627	0.7735	0.89265	-5.855
Rab21	"RAB21, member RAS oncogen	299799	0.036982	6.7645	0.29296	0.77595	0.89448	-5.856
Pdia6	"protein disulfide isomerase fam	286906	0.033131	6.563	0.29239	0.77637	0.89448	-5.8561
Psmd11	"proteasome 26S subunit, non-A	303353	0.037161	4.515	0.29073	0.77761	0.89515	-5.8566
Slc43a1	solute carrier family 43 member	311168	-0.043584	5.3068	-0.28828	0.77942	0.8965	-5.8574
Cyb5r3	cytochrome b5 reductase 3	25035	0.060002	7.524	0.28633	0.78087	0.89676	-5.8579
Asah1	N-acylsphingosine amidohydrola	84431	-0.049287	5.1704	-0.28623	0.78094	0.89676	-5.858
Acot8	acyl-CoA thioesterase 8	170588	-0.033802	3.9648	-0.27981	0.78572	0.9015	-5.8598
Slc25a24	solute carrier family 25 member	310791	0.034569	6.2969	0.27474	0.78949	0.90482	-5.8612
Rpl5	ribosomal protein L5	81763	0.047047	7.4447	0.27416	0.78992	0.90482	-5.8614
Sirt3	sirtuin 3	293615	-0.038053	4.2539	-0.27122	0.79211	0.90646	-5.8622
Spta1	"spectrin, alpha, erythrocytic 1"	289257	0.056291	11.322	0.27049	0.79266	0.90646	-5.8624
Rps15a	ribosomal protein S15a	117053	-0.072094	6.9983	-0.26894	0.79382	0.90703	-5.8628
Ndufaf4	NADH:ubiquinone oxidoreducta	362495	0.031681	7.7255	0.26761	0.79481	0.90704	-5.8632
Rpl27	ribosomal protein L27	64306	-0.043752	7.6512	-0.26718	0.79513	0.90704	-5.8633
Col6a1	collagen type VI alpha 1 chain	294337	0.035032	7.9346	0.25446	0.80465	0.91714	-5.8667
Rdh13	retinol dehydrogenase 13	361504	-0.029112	6.8954	-0.24876	0.80893	0.9207	-5.8682
Ndufb4	NADH:ubiquinone oxidoreducta	288088	0.031136	10.16	0.24853	0.8091	0.9207	-5.8682
Mrps23	mitochondrial ribosomal protein	360594	-0.026587	6.0356	-0.24425	0.81231	0.9226	-5.8693
Ampd1	adenosine monophosphate dea	25028	-0.046541	10.956	-0.24354	0.81285	0.9226	-5.8695
Aldh7a1	"aldehyde dehydrogenase 7 fam	291450	-0.030396	6.3481	-0.2431	0.81318	0.9226	-5.8696
Ubl3	ubiquitin-like 3	363869	-0.028621	4.7454	-0.24277	0.81343	0.9226	-5.8697
Myl12b	myosin light chain 12B	50685	0.037771	6.3124	0.23754	0.81737	0.92619	-5.8709
Lypla1	lysophospholipase 1	25514	-0.031281	5.3108	-0.23432	0.81979	0.92619	-5.8717
Naga	alpha-N-acetylgalactosaminidas	315165	-0.025666	4.9966	-0.23365	0.82029	0.92619	-5.8719
Mtx2	metaxin 2	288150	0.027375	7.6131	0.23346	0.82043	0.92619	-5.8719
Speg	striated muscle enriched protein	363256	0.025406	8.0218	0.23313	0.82068	0.92619	-5.872
Tmed9	transmembrane p24 trafficking p	361207	0.040199	6.0183	0.23279	0.82095	0.92619	-5.8721
Acot2	acyl-CoA thioesterase 2	192272	-0.048889	8.0941	-0.23234	0.82128	0.92619	-5.8722
Plxnb2	plexin B2	315217	0.027972	4.4093	0.23	0.82305	0.92744	-5.8727
Lamtor1	"late endosomal/lysosomal adap	308869	0.034496	5.4969	0.22381	0.82772	0.93115	-5.8742
Clta	"clathrin, light chain A"	83800	-0.032425	4.0489	-0.22257	0.82866	0.93115	-5.8744
Itgb3	integrin subunit beta 3	29302	-0.038858	5.2875	-0.22231	0.82885	0.93115	-5.8745
Banf1	BAF nuclear assembly factor 1	114087	0.036252	6.449	0.22148	0.82949	0.93115	-5.8747
Mrps36	mitochondrial ribosomal protein	294696	0.047084	8.519	0.22118	0.82971	0.93115	-5.8748
Dmac2l	distal membrane arm assembly c	362749	-0.029506	4.692	-0.21929	0.83114	0.93174	-5.8752
Mrpl57	mitochondrial ribosomal protein	691814	0.029059	5.3784	0.21838	0.83183	0.93174	-5.8754
Selenof	selenoprotein F	113922	0.022394	4.3378	0.21783	0.83224	0.93174	-5.8755

Trip10	thyroid hormone receptor interac	116717	0.028065	5.0954	0.2159	0.8337	0.93201	-5.8759
Cd99	CD99 molecule (Xg blood group	652929	-0.023583	4.7852	-0.21572	0.83384	0.93201	-5.876
Rpl24	ribosomal protein L24	64307	-0.032184	7.7913	-0.21484	0.83451	0.93201	-5.8762
Cat	catalase	24248	0.043851	8.8847	0.2129	0.83598	0.9325	-5.8766
Rnf170	ring finger protein 170	364654	0.02936	4.7979	0.21248	0.83629	0.9325	-5.8767
Erap1	endoplasmic reticulum aminopep	80897	0.022439	4.5731	0.21099	0.83743	0.93282	-5.877
Phkb	phosphorylase kinase regulatory	361377	0.031428	7.3477	0.20988	0.83826	0.93282	-5.8772
Acsl3	acyl-CoA synthetase long-chain	114024	-0.024166	6.309	-0.20929	0.83871	0.93282	-5.8774
Tmem38a	transmembrane protein 38a	306327	-0.032471	9.7026	-0.20855	0.83927	0.93282	-5.8775
Rpl31	ribosomal protein L31	64298	-0.024748	7.539	-0.20658	0.84077	0.93348	-5.8779
Mrpl13	mitochondrial ribosomal protein	299938	-0.023978	5.5467	-0.206	0.84121	0.93348	-5.8781
Me2	malic enzyme 2	307270	-0.029097	6.7517	-0.20118	0.84487	0.9357	-5.8791
Arhgap1	Rho GTPase activating protein	311193	-0.022306	4.8651	-0.20088	0.8451	0.9357	-5.8791
Psma6	proteasome 20S subunit alpha	29673	0.025434	4.2288	0.20069	0.84524	0.9357	-5.8792
Lias	lipoic acid synthetase	305348	0.029683	4.6727	0.19903	0.8465	0.93635	-5.8795
Myo1b	myosin Ib	117057	-0.028454	4.1308	-0.19547	0.84921	0.9386	-5.8802
Thbs4	thrombospondin 4	29220	-0.028644	4.9439	-0.19256	0.85142	0.9403	-5.8808
Itih4	inter-alpha-trypsin inhibitor heav	54404	0.023699	4.515	0.19111	0.85252	0.94076	-5.8811
Mrps34	mitochondrial ribosomal protein	287126	0.027116	6.5876	0.18819	0.85475	0.94247	-5.8816
Rpl26	ribosomal protein L26	287417	0.032358	7.2182	0.18712	0.85557	0.94262	-5.8819
Apoc3	apolipoprotein C3	24207	-0.029311	4.6049	-0.18484	0.85731	0.94333	-5.8823
Cox5a	cytochrome c oxidase subunit 5	252934	-0.023196	12.346	-0.18411	0.85786	0.94333	-5.8824
Vamp3	vesicle-associated membrane pr	29528	-0.020724	7.2211	-0.18359	0.85826	0.94333	-5.8825
Rpl23a	ribosomal protein L23a	360572	0.029364	8.3938	0.18113	0.86013	0.94464	-5.883
Copa	COPI coat complex subunit alph	304978	0.020217	5.4161	0.17997	0.86102	0.94487	-5.8832
Plbd1	phospholipase B domain contai	297694	-0.019326	5.7309	-0.17611	0.86396	0.94564	-5.8839
Rars1	arginyl-tRNA synthetase 1	287191	-0.020977	4.9247	-0.17531	0.86457	0.94564	-5.884
Ndufv2	NADH:ubiquinone oxidoreducta	81728	-0.021941	10.899	-0.17518	0.86468	0.94564	-5.8841
Ndufaf7	NADH:ubiquinone oxidoreducta	298748	0.019828	3.884	0.17484	0.86493	0.94564	-5.8841
Mrps27	mitochondrial ribosomal protein	361883	-0.019041	6.5687	-0.17459	0.86513	0.94564	-5.8842
Rpl13	ribosomal protein L13	81765	0.033638	7.7532	0.17141	0.86756	0.94754	-5.8847
Ldb3	LIM domain binding 3	498587	-0.019007	6.9109	-0.17009	0.86856	0.9479	-5.885
Slc3a2	solute carrier family 3 member 2	50567	0.017506	6.381	0.16636	0.87142	0.94916	-5.8856
Aprt	adenine phosphoribosyl transfe	292072	-0.018883	5.0085	-0.16547	0.8721	0.94916	-5.8858
Hmgcl	3-hydroxy-3-methylglutaryl-CoA	79238	0.019499	7.1261	0.16516	0.87234	0.94916	-5.8858
Afg3l2	AFG3 like matrix AAA peptidase	307350	0.021319	9.4697	0.16442	0.8729	0.94916	-5.8859
Rpl12	ribosomal protein L12	499782	0.021022	6.7931	0.16359	0.87354	0.94916	-5.8861
Cd163	CD163 molecule	312701	0.02408	8.2523	0.16266	0.87425	0.94916	-5.8862
Ctnna1	catenin alpha 1	307505	0.022475	4.6185	0.16217	0.87463	0.94916	-5.8863
Coq5	"coenzyme Q5, methyltransferas	304542	0.024929	7.4658	0.16142	0.8752	0.94916	-5.8864

Ctsc	cathepsin C	25423	-0.022897	5.685	-0.1592	0.87689	0.94995	-5.8868
Ubxn4	UBX domain protein 4	304766	-0.019046	4.82	-0.15659	0.8789	0.94995	-5.8872
Auh	AU RNA binding methylglutacon	361215	-0.022338	8.1319	-0.15614	0.87924	0.94995	-5.8873
Rps18	ribosomal protein S18	294282	0.031555	6.9914	0.15581	0.8795	0.94995	-5.8873
Mtfp1	mitochondrial fission process 1	289745	-0.018533	6.5969	-0.15538	0.87983	0.94995	-5.8874
Glipr2	GLI pathogenesis-related 2	679819	-0.021406	5.7793	-0.15509	0.88005	0.94995	-5.8875
Art3	ADP-ribosyltransferase 3	305235	-0.017519	7.1183	-0.15421	0.88073	0.94995	-5.8876
Pdhx	"pyruvate dehydrogenase comp	311254	0.025287	9.7983	0.15184	0.88254	0.95117	-5.888
Ywhab	"tyrosine 3-monooxygenase/tryp	56011	0.019751	5.1274	0.14847	0.88512	0.95281	-5.8885
Nceh1	neutral cholesterol ester hydrola	294930	0.015544	3.9756	0.14806	0.88544	0.95281	-5.8885
Cd9	CD9 molecule	24936	-0.035928	5.0682	-0.14407	0.8885	0.95416	-5.8891
Tomm70	translocase of outer mitochondr	304017	-0.016607	7.5391	-0.14366	0.88882	0.95416	-5.8892
Creld1	cysteine-rich with EGF-like doma	312638	-0.026433	6.5722	-0.14314	0.88922	0.95416	-5.8893
Cct4	chaperonin containing TCP1 su	29374	0.017535	5.4525	0.13946	0.89205	0.95416	-5.8898
Atp5f1d	ATP synthase F1 subunit delta	245965	-0.018092	10.558	-0.13787	0.89327	0.95416	-5.89
Echs1	"enoyl-CoA hydratase, short cha	140547	-0.024539	11.001	-0.13784	0.89329	0.95416	-5.89
Ehbp1l1	EH domain binding protein 1-like	309169	0.019156	3.8181	0.13726	0.89374	0.95416	-5.8901
Mrpl20	mitochondrial ribosomal protein	680747	0.019628	4.5631	0.13614	0.8946	0.95416	-5.8903
Ywhag	"tyrosine 3-monooxygenase/tryp	56010	-0.017939	7.5011	-0.13583	0.89483	0.95416	-5.8903
Fdxr	ferredoxin reductase	79122	0.015651	4.5548	0.13559	0.89502	0.95416	-5.8904
Acot13	acyl-CoA thioesterase 13	291135	0.015181	7.8825	0.13528	0.89526	0.95416	-5.8904
Mtif2	mitochondrial translational initiat	305606	-0.015278	5.3761	-0.13497	0.8955	0.95416	-5.8904
Mrpl38	mitochondrial ribosomal protein	303685	-0.015877	6.0825	-0.13479	0.89564	0.95416	-5.8905
Igf2r	insulin-like growth factor 2 recep	25151	0.014139	6.5504	0.1315	0.89817	0.95437	-5.8909
Dcn	decorin	29139	-0.019716	8.4953	-0.13039	0.89902	0.95437	-5.8911
Hspe1	heat shock protein family E (Hsp	25462	-0.018911	10.56	-0.12998	0.89934	0.95437	-5.8911
Kpnb1	karyopherin subunit beta 1	24917	0.018664	4.8115	0.12995	0.89936	0.95437	-5.8911
Smpdl3b	"sphingomyelin phosphodiester	362619	-0.014302	7.0009	-0.1295	0.89971	0.95437	-5.8912
Mmaa	metabolism of cobalamin associ	291939	-0.014554	6.154	-0.12916	0.89997	0.95437	-5.8912
Rab8b	"RAB8B, member RAS oncogen	266688	-0.014247	5.0523	-0.12529	0.90294	0.95614	-5.8917
Mia3	MIA SH3 domain ER export fact	683007	0.013149	4.6632	0.12519	0.90302	0.95614	-5.8917
Dmd	dystrophin	24907	0.01524	9.3767	0.12337	0.90443	0.9568	-5.892
Hsp90b1	heat shock protein 90 beta fami	362862	0.01485	8.2545	0.12187	0.90558	0.9568	-5.8922
Psma4	proteasome 20S subunit alpha	29671	-0.013753	4.5564	-0.12159	0.9058	0.9568	-5.8922
Nit2	"nitrilase family, member 2"	288174	0.019283	5.8664	0.12076	0.90643	0.9568	-5.8923
Sh3glb1	SH3 domain -containing GRB2-l	292156	-0.018206	6.6844	-0.1199	0.9071	0.9568	-5.8924
Rps27a	ribosomal protein S27a	100912032	0.014443	7.8125	0.11848	0.90819	0.95723	-5.8926
Mrps9	mitochondrial ribosomal protein	301371	0.015912	6.743	0.11719	0.90919	0.95732	-5.8927
Tuba4a	"tubulin, alpha 4A"	316531	0.018766	7.6952	0.116	0.91011	0.95732	-5.8929
Hsd17b12	hydroxysteroid (17-beta) dehydr	84013	-0.014207	5.192	-0.11568	0.91036	0.95732	-5.8929

Rps3	ribosomal protein S3	140654	0.02047	7.7885	0.11228	0.91297	0.95935	-5.8933
Tst	thiosulfate sulfurtransferase	25274	-0.014746	7.7588	-0.10965	0.91501	0.96076	-5.8936
Abcc1	ATP binding cassette subfamily	24565	-0.017948	6.0085	-0.10765	0.91655	0.96165	-5.8938
Mrpl4	mitochondrial ribosomal protein	363023	0.014362	5.3886	0.10536	0.91831	0.96258	-5.8941
Ak1	adenylate kinase 1	24183	-0.017991	7.7344	-0.10384	0.91949	0.96258	-5.8942
Ndrg2	NDRG family member 2	171114	0.017068	7.2791	0.10302	0.92012	0.96258	-5.8943
Gcdh	glutaryl-CoA dehydrogenase	364975	0.012527	7.4352	0.10258	0.92046	0.96258	-5.8944
Ppib	peptidylprolyl isomerase B	64367	-0.012382	7.5485	-0.10201	0.92091	0.96258	-5.8944
Iscu	iron-sulfur cluster assembly enzy	288740	-0.012393	5.4262	-0.099864	0.92256	0.96358	-5.8947
Ndufa3	NADH:ubiquinone oxidoreducta	691001	0.013007	7.3035	0.097597	0.92431	0.96468	-5.8949
Prdx3	peroxiredoxin 3	64371	0.014481	10.251	0.09516	0.9262	0.96587	-5.8951
Rpl18a	ribosomal protein L18A	290641	0.0126	7.7534	0.094076	0.92703	0.96587	-5.8952
Stub1	STIP1 homology and U-box cor	287155	0.009787	4.3382	0.092918	0.92793	0.96587	-5.8953
Ca14	carbonic anhydrase 14	791259	0.010066	4.2678	0.091813	0.92878	0.96587	-5.8955
Suclg1	"succinate-CoA ligase, alpha su	114597	-0.012287	10.85	-0.091614	0.92894	0.96587	-5.8955
Mrpl47	mitochondrial ribosomal protein	294963	-0.0098795	6.0109	-0.087498	0.93212	0.96811	-5.8958
Suclg2	"succinate-CoA ligase, GDP-for	362404	-0.015291	7.9818	-0.086228	0.9331	0.96811	-5.896
Parp3	"poly (ADP-ribose) polymerase f	300985	-0.01269	5.367	-0.084841	0.93418	0.96811	-5.8961
Abca9	ATP binding cassette subfamily	287788	0.014127	4.6378	0.083947	0.93487	0.96811	-5.8962
Atp1b3	ATPase Na+/K+ transporting su	25390	-0.012716	5.9237	-0.083028	0.93558	0.96811	-5.8962
Mrps26	mitochondrial ribosomal protein	362216	0.0096028	5.1765	0.082493	0.93599	0.96811	-5.8963
Wfs1	wolframin ER transmembrane gl	83725	0.0099392	4.2967	0.081955	0.93641	0.96811	-5.8963
Yme1l1	YME1-like 1 ATPase	114217	-0.010732	4.6271	-0.081616	0.93667	0.96811	-5.8964
Supv3l1	Suv3 like RNA helicase	294385	0.010269	5.0476	0.078795	0.93886	0.96964	-5.8966
Abhd16a	abhydrolase domain containing	361796	0.010082	6.2253	0.075099	0.94172	0.9709	-5.8969
Atp13a1	ATPase 13A1	290673	0.0079611	5.4908	0.07435	0.9423	0.9709	-5.8969
Naca	nascent polypeptide associated	288770	0.011726	9.1243	0.074153	0.94245	0.9709	-5.897
Isca2	iron-sulfur cluster assembly 2	500694	0.01607	6.1011	0.073601	0.94288	0.9709	-5.897
Rcn1	reticulocalbin 1	362182	-0.0096635	4.6339	-0.072012	0.94411	0.97145	-5.8971
Zadh2	"zinc binding alcohol dehydroge	291403	0.008098	5.1359	0.07067	0.94515	0.97179	-5.8972
Gpx7	glutathione peroxidase 7	298376	0.0082668	4.7413	0.066466	0.94841	0.97441	-5.8975
Cdnf	cerebral dopamine neurotrophic	361276	-0.0092878	6.499	-0.065573	0.9491	0.97441	-5.8976
Wdr62	WD repeat domain 62	308492	-0.013797	5.8551	-0.063111	0.95101	0.97564	-5.8977
Fbn1	fibrillin 1	83727	-0.01089	10.055	-0.060853	0.95276	0.97672	-5.8979
Eno3	enolase 3	25438	0.0084437	11.468	0.059917	0.95348	0.97674	-5.8979
Sco1	synthesis of cytochrome C oxida	497930	0.0072383	6.3745	0.056966	0.95577	0.97732	-5.8981
Apoe	apolipoprotein E	25728	0.0064398	6.9024	0.056715	0.95597	0.97732	-5.8981
Tmed1	transmembrane p24 trafficking p	315461	0.0072716	4.8392	0.056456	0.95617	0.97732	-5.8981
Mrpl40	mitochondrial ribosomal protein	287962	0.0064152	5.9943	0.05371	0.9583	0.97741	-5.8983
Gnai2	G protein subunit alpha i2	81664	-0.0066932	8.1747	-0.053369	0.95856	0.97741	-5.8983

Rps28	ribosomal protein S28	691531	0.0060334	4.1	0.052854	0.95896	0.97741	-5.8983
Bckdk	branched chain ketoacid dehyd	29603	-0.0077575	7.2297	-0.052704	0.95908	0.97741	-5.8983
Abcf2	ATP binding cassette subfamily	311959	0.0057347	5.3191	0.050761	0.96058	0.97823	-5.8985
Mdh2	malate dehydrogenase 2	81829	-0.0065807	13.753	-0.044045	0.9658	0.982	-5.8988
Echdc2	enoyl CoA hydratase domain co	298381	0.0073852	6.1945	0.043395	0.9663	0.982	-5.8988
Serpinc1	serpin family C member 1	304917	0.0092292	5.0734	0.043244	0.96642	0.982	-5.8988
Lamtor2	"late endosomal/lysosomal adap	295234	-0.0054685	5.4339	-0.041195	0.96801	0.9829	-5.8989
Scamp1	secretory carrier membrane prot	29521	0.0041928	4.3844	0.038938	0.96976	0.98396	-5.899
LOC683897	similar to Protein C6orf203	683897	-0.0041545	4.8853	-0.037513	0.97086	0.98436	-5.8991
Micu2	mitochondrial calcium uptake 2	171433	-0.0041328	3.9227	-0.031589	0.97546	0.9883	-5.8993
Clpx	caseinolytic mitochondrial matrix	300786	-0.0042508	6.8842	-0.029527	0.97707	0.9892	-5.8993
Gdpd1	glycerophosphodiester phospho	303407	-0.0032403	4.5015	-0.028054	0.97821	0.98963	-5.8994
Pebp1	phosphatidylethanolamine bindi	29542	0.0029886	6.4026	0.026703	0.97926	0.98997	-5.8994
Acot9	acyl-CoA thioesterase 9	302640	-0.0054766	6.1001	-0.022305	0.98267	0.9927	-5.8995
Nudt19	nudix hydrolase 19	308518	0.0028561	4.6806	0.015682	0.98782	0.99596	-5.8996
Hsd17b8	hydroxysteroid (17-beta) dehydr	361802	0.0028327	5.6809	0.014334	0.98886	0.99596	-5.8997
Nme2	NME/NM23 nucleoside diphospl	83782	0.0015145	6.9032	0.013766	0.98931	0.99596	-5.8997
Tnnt3	"troponin T3, fast skeletal type"	24838	0.002844	9.6083	0.013705	0.98935	0.99596	-5.8997
Ube2d2	ubiquitin-conjugating enzyme E2	641452	0.0017236	5.0354	0.01352	0.9895	0.99596	-5.8997
Mrpl44	mitochondrial ribosomal protein	301552	-0.001543	5.8968	-0.012294	0.99045	0.9962	-5.8997
Calr	calreticulin	64202	-0.0013009	9.7603	-0.0096968	0.99247	0.99676	-5.8997
Cmpk2	cytidine/uridine monophosphate	314004	0.0012055	4.9215	0.0096485	0.9925	0.99676	-5.8997
Hint2	histidine triad nucleotide binding	313491	-0.0013658	6.1879	-0.0088049	0.99316	0.99676	-5.8997
Hsd17b4	hydroxysteroid (17-beta) dehydr	79244	-0.0007887	8.0366	-0.0070185	0.99455	0.99743	-5.8997
Rpl30	ribosomal protein L30	64640	0.00077585	6.8135	0.0059886	0.99535	0.99751	-5.8998
Cpq	carboxypeptidase Q	58952	0.00045333	3.9507	0.0039061	0.99697	0.99841	-5.8998
Etfb	electron transfer flavoprotein su	292845	0.00025591	10.747	0.0018183	0.99859	0.99931	-5.8998
D2hgdh	D-2-hydroxyglutarate dehydroge	301624	1.6904E-05	5.7689	0.00012665	0.9999	0.9999	-5.8998

Supplementary Table 4c.Total list of the age-related MAM proteins in heart and GA muscle

Heart		GA muscle	
**Up-regulation**	**Down-regulation**	**Up-regulation**	**Down-regulation**
**298 hits**	**249 hits**	**111 hits**	**180 his**
**Prkcsh**	**Etfdh**	**Clic4**	**Bdh1**
**Rab18**	**Sdha**	**Actn1**	**Vdac1**
**Hadhb**	**Immt**	**Ganab**	**Vdac3**
**Aldh9a1**	**Hspa9**	**Mdh1**	**Samm50**
**Cisd1**	**Uqcrc1**	**Hsp90ab1**	**Cyc1**
**Rpl4**	**Gnai2**	**Eef2**	**Atp2a2**
**Acat1**	**Atp1a1**	**Lrp1**	**Phb2**
**Slc25a12**	**Acsl1**	**Prkcsh**	**Rab14**
**Pacsin3**	**Gnb1**	**Msn**	**Vdac2**
**Abcd3**	**Dlat**	**Cltc**	**Abcb7**
**Uggt1**	**Cltc**	**Atp6v1a**	**Ndufa4**
**Vdac3**	**Acadl**	**Pgm1**	**Ndufv1**
**Gstp1**	**Hsp90ab1**	Psmc2	**Ndufs7**
**Decr1**	**Ndufs1**	Vim	**Slc25a12**
**Cd81**	**Hsp90b1**	S100a10	**Dlat**
**Hspd1**	**Letm1**	Tppp3	**Gna13**
**P4hb**	**Hspa8**	Cct3	**Pcyox1**
**Rps3**	**Aco2**	Aqp1	**Rab2a**
**Rpl10**	**Hsd17b4**	Fn1	**Ndufb10**
**Anxa6**	**Ndufs7**	Dpysl2	**Slc16a1**
**Ndufv2**	**Lap3**	Cav3	**Cox4i1**
**Ndufb6**	**Cct8**	Lum	**Ndufb5**
**Ppib**	**Afg3l2**	Itgb2	**Gnas**
**Ndufs2**	**Phb2**	Cct7	**Atp1b1**
**Echs1**	**Got2**	Cct2	**Mtdh**
**Glud1**	**Vdac1**	Nap1l4	**Aldh6a1**
**Tmed10**	**Mtdh**	Gapdh	**Immt**
**Pgk1**	**Aifm1**	Tubb5	**Ndufs1**
**Snd1**	**Ldha**	Septin7	Slc25a11
**Ndufs3**	**Ndufa9**	Myl6	C1qbp
**Mdh1**	**Ndufb4**	Ahnak	Ndufab1
**Rpn1**	**Atp2a2**	Ckb	Rras2
**Acaa2**	**Mdh2**	Eif2s1	Lamp2
**Ndufb10**	**Idh2**	Anpep	Kcnj11
**Abcb7**	**Atp1b1**	Pdlim1	Atp5md
**Alb**	**Rrbp1**	Ckap4	Ctsb

**Pdia3**	**Canx**	Epb41l2	Cox6b1
**Rab7a**	**Msn**	Rps23	Uqcr10
**Vcp**	**Actn4**	Anxa5	Slc12a2
**Cycs**	**Phb**	Rtraf	Ndufa11
**Eno1**	**Slc25a3**	Psme1	Nnt
**Uqcrfs1**	**Rplp0**	Unc45b	Pet100
**Tpi1**	**Mlec**	Fkbp11	Sod2
**Mccc1**	**Vdac2**	Rnh1	Casq1
**Capza2**	**Sdhb**	Atp5mf	Cd59
**Acox1**	**Cs**	Hpx	Fitm1
**Aldh6a1**	**Park7**	Pdia4	Jph1
**Rpl9**	**Ndufv1**	Gng12	Cacna1s
**Uqcrc2**	**Prdx5**	Snx2	Coq3
**Rpl6**	**Ndrg2**	Col1a1	Sdr39u1
**Pc**	**Cfl1**	Exoc4	Bcs1l
**Gnas**	**Apoe**	Ppp2r1a	Tmem186
Cspg4	**Rpl8**	Ifnl1	Sgcg
Tbrg4	**Ap2a2**	Lamtor5	Eepd1
Vapb	Nlrx1	Psmc3	Ndufs4
Rcn2	Mybpc3	Tubb4b	Abcc9
Grpel1	Flna	Fga	Maip1
Jup	Acadvl	Clpb	Lpcat3
Etfb	Tpm1	Tkt	Acss3
Rpl19	Crat	Ckm	Ndufa8
Atp5md	Pygm	Myh11	Arl6ip5
Idh3a	Pdha1	Etf1	Lamp1
Mrpl37	Slc8a1	Cd34	Cisd3
Ccdc127	Dnajc11	Nmnat3	Ndufa12
Epb41l2	Actn2	St13	Acss1
Dld	Myom2	Mvp	Sypl2
Aldh5a1	Fbn1	Gypc	Atp5f1c
Gja1	Nnt	Pvalb	Mcu
Itgav	Rap1a	Scp2	Sdhc
Atp5mf	Mtx2	Rap2b	Ghitm
Mrps30	Acsf2	Cct6a	Ndufs8
Ndufa7	Pcca	Cavin1	Hhatl
Flot1	Hsdl2	Hexa	Tomm22
Ndufb11	Hadh	Flna	Cnst
Mrpl19	Oxct1	Capn2	Mrps18b
Oat	Atp5pd	Rps12	Uqcc2

Slc25a11	Tln1	Sec31a	Ppid
Ndufs8	Eef1a2	Add2	Tmem11
Enpep	L2hgdh	Rpsa	Niban2
Mrpl15	Atp5f1b	Tln1	Uqcc1
Niban1	Hba1	Mybpc1	Acad9
Cbr1	Cd36	Ap2m1	Uqcrh
Ncam1	Ldb3	Akap12	Mylpf
Uqcc1	Anxa5	Pf4	Tecr
Slc12a7	Vcl	Ankrd2	Cox7b
Cd163	Sntb1	F13a1	Por
Ckb	Pnpt1	Ehd4	Rtn4
Mtfp1	Idh3g	Eef1d	Ndufc2
Ccdc90b	Acadm	Cdh13	Tapt1
Ryr2	Coq5	Fabp3	Cacnb1
Mtif2	Rps5	Pabpc1	Tpm3
Atp5me	Maoa	Man2a2	Atp5pb
Pyroxd2	Ndufs5	Timmdc1	Atp5if1
Ppp1cc	Sod2	Ppia	Oma1
Acta1	Snap23	Tmx2	Rock1
Mpst	Ndufb7	Hspb6	Ctsd
Cox7a2	Atp5f1c	Septin11	Lrrc57
Cfh	Tgm2	Rps2	Mreg
Pmpcb	Cryab	C3	Timm50
Cox5b	Ndufaf3	Itga2b	Art1
Mvp	Myl2	Tgm2	Parl
Rpl23	Txndc5	Pygm	Slc44a2
Hnrnpu	Scp2	Pcbp2	Cyb5r1
Aldh4a1	Serpinh1	Capzb	Mrpl27
Ak2	Hbb	Prkar1a	Pmpca
Pi4k2a	Add3	Get3	Slc25a4
Acot7	Sdhc	Eef1g	Cul1
Pgam1	Pdk2	Micu3	Romo1
Dbt	Cdh13	Gpd1	Mfn2
Vwa8	Ank1	Hdlbp	Trdn
Bcam	Rras2	Septin2	Mrpl58
Ehd4	Ak3		Calu
Hebp1	Ivd		Apoo
Acsf3	Srl		Dnajb4
Mlycd	Nos3		Pecr
Flnc	Ldhb		Ryr1

Rexo2	Gpi		Rpn2
Ndufa2	Pacsin2		Dhrs4
Gk	Ckm		Ndufa7
Hpx	Dglucy		Slc25a42
Opa1	Prdx3		Rpl27a
Sgcg	Bcs1l		Tmem143
Clpx	Gfm1		Mgll
Dpp4	Psma5		Ptges2
Cox6b1	Aldh7a1		App
Tomm70	Mrps23		Myl2
Gnpat	Fam162a		Uqcrb
Lpl	C3		Hccs
Pars2	Spg7		Mrps35
Slc27a1	Ssr4		Acadvl
Taco1	Acad10		Plpp7
Slc9a3r2	Cav1		Stx8
Copb1	Capn2		Ccpg1
Rmdn1	Sgcd		Rab12
Actr3	Ptgis		Cox18
Timm21	Rpl3l		Adhfe1
Apoa1	Ndufaf6		Pdk4
Zadh2	Fkbp8		Ppbp
Tubb2a	Pecr		Cox14
Mrps36	Mrps7		Sec61a2
Nsf	Ndufs4		Agk
Mul1	Itga7		Cpt2
Lamtor1	Timm50		Gba
Ldhd	Pebp1		Atp2a3
Ehd2	Gstz1		Ndufaf2
Dag1	Flnb		Sec22b
Prdx2	Gpx4		Cobl
Clpb	Slmap		Spcs3
Ano6	C1qbp		Parp14
Fn1	Fam210a		Lman2
Atp5mg	Clu		Gnaq
Arhgdia	Ywhaz		Bnip3
Cacna2d1	Napa		Mtx1
Eci2	Gsn		Mrpl33
Asph	Slc25a18		LOC100912599
Hsp90aa1	Cox6a2		Atp5pd

Gnb3	Bsg		Edf1
Actc1	Tubb4b		Ppox
Clic5	Dlst		Tufm
Mrps27	Anxa11		Acad8
Cpt1b	Tufm		Cd36
Ndufv3	Scamp1		Micu1
Mrpl41	Pccb		Stbd1
Rps2	Suclg2		Camk2g
Gpx1	Sgcb		Cox7c
Rpl17	Ccdc141		Yars2
Chchd3	Plg		Deptor
Gng12	Mrpl38		Ndufb1
Cyb5a	Plin4		Mgst3
Atp5po	Isca2		Magt1
Ecsit	Pls3		Ccdc51
Oxsm	Coq8a		Flot1
Ndufa10	Septin7		Cpt1b
Popdc2	Csrp3		Nmt1
Myl6	Pdcd6ip		Cox16
Pdhb	Kif5b		Mrc1
Lamtor5	Tpp1		Mrpl48
Cpt2	Ech1		Pdhb
Fermt2	Epb42		Flot2
Mrps25	Tomm22		
Lman1	LOC100911130		
Sardh	Art3		
Sirt3	Fga		
Uqcrh	Acot9		
Bche	Jph2		
Hint2	Gcdh		
Acaca	Acot2		
Ckmt2	Ndufaf4		
Coq6	Pnpla8		
Rhoa	Mb		
Snta1	Ndufc2		
Col14a1	Pdhx		
Rack1	Vat1		
Cars2	D2hgdh		
Ywhaq	Rpn2		
Ckap4	Dguok		

Tnnt2	Uqcr10		
Ccdc51	Ppif		
Hk1	Anxa4		
Hk2	Tfrc		
Cct2	Mtx1		
Sqor	Itga5		
Pbxip1	Arf1		
Septin2	Psmc1		
Ywhag	Mgll		
Ehd1	Bckdhb		
Atp1a2	Cyb5r1		
Coq9	Vtn		
Surf1	Apoh		
Ndufa12	Rps4x		
Csrp1	Lama2		
Ndufa11	Gstk1		
Tmem65	Dpysl2		
Gnb2	Marcks		
Cyb5r3	Ociad1		
Lgals1	Camk2d		
Tomm40	Nckap1		
Wdr1	Stip1		
Acads	Sgca		
Ywhab	Ctnnd1		
Por	Rrad		
Myo1c	Agk		
Dbi	Mrpl45		
Des	Arl6ip5		
Atp5f1a	Lactb		
Pfkm	Psmd1		
Mgst3	Lsamp		
Tmem120a	Cct3		
Nt5e	Rpl30		
Myh6	Anxa1		
Lias	Ppia		
Got1	Ca14		
Ndufb9	RGD1565784		
Cct5	Pdp1		
Cdh2	Ndufaf2		
Gapdh	Hrg		

Dnaja3	Agps		
Trap1	Arhgap1		
Aldoa	Ipo5		
Hibadh	Pcyt1a		
Tnni3	Myl7		
Lnpep	Prkar1a		
Anxa2	Mtarc2		
Lama4	Abhd16a		
Cavin1	Afg1l		
Iars2	Gyg1		
Hhatl	Cd47		
Lonp1	Trak1		
Bckdha	Rps17		
Mecr			
Ywhae			
Gnao1			
Myh7			
Cap1			
Pitrm1			
Agl			
Syncrip			
Ehbp1l1			
Ptges2			
Casq2			
Me3			
Ctnnb1			
Slc25a4			
Atp5pf			
Cavin4			
Plcd1			
Kyat3			
Mtch2			
Theg			
Epb41			
Inpp5a			
Ndufb8			
Cacna2d2			
Ndufa6			
Speg			
Hspa5			

Fahd1			
Myh14			
Sfxn3			
Cd38			
Itga6			
Hspa12b			
Mrpl47			
Atp6v0a1			
Tagln2			
Macrod1			
Mospd1			
Sptan1			
Dnajc3			
Pecam1			
Abca8a			
Trim72			
Hibch			
Septin11			
Mccc2			
Psmd11			
Septin8			
Abcb8			
Bcat2			

The age-related MAM proteins was identified when the protein expression ratio >1.2 or <0.83 and P <0.05 (Student's t-test) between 4 mon and 24 mon. The deregulated consensus MAM proteins were highlited in bold.

Supplementary Table 4d. Differential expression analysis of combined dataset derived from heart and GA muscle samples			
**Gene names**	**EntrezID**	**CombinedTstat**	**CombinedPval**
Gapdh	24383	-33.517	0.0005981
Sptan1	64159	-34.117	0.0005981
Hspa5	25617	-32.656	0.00059831
Nnt	310378	30.678	0.0011072
Acadvl	25363	30.26	0.0011072
Trim72	365377	-29.465	0.0013392
Bdh1	117099	29.039	0.0013908
Rras2	365355	28.726	0.0013908
Nlrx1	315599	28.282	0.0013908
C1qbp	29681	28.088	0.0013908
Cavin1	287710	-28.301	0.0013908
Sod2	24787	27.432	0.0014181
Etfdh	295143	27.34	0.0014181
Immt	312444	27.243	0.0014181
Mdh1	24551	-27.113	0.0014181
Rpl6	117042	-27.189	0.0014181
Septin11	305227	-27.613	0.0014181
Bcs1l	301514	26.762	0.001431
Dlat	81654	26.744	0.001431
Cct2	299809	-26.908	0.001431
Tpm1	24851	26.292	0.0015582
Mccc2	361884	-26.169	0.0015582
Bcat2	64203	-26.246	0.0015582
Myh14	308572	-26.288	0.0015582
Vdac1	83529	25.458	0.0017563
Sdhc	289217	25.414	0.0017563
Uqcrc1	301011	25.244	0.0017563
Uqcr10	685322	25.132	0.0017563
Acsl1	25288	25.125	0.0017563
Crat	311849	25.117	0.0017563
Phb2	114766	25.097	0.0017563
Pc	25104	-25.138	0.0017563
Fn1	25661	-25.289	0.0017563
Gnas	24896	-25.344	0.0017563
Hspa12b	311427	-25.711	0.0017563
Flna	293860	24.818	0.0018906
Aldoa	24189	-24.87	0.0018906
Ckap4	362859	-24.66	0.0019803
Ndufs4	499529	24.471	0.0020255
Atp5f1c	116550	24.417	0.0020255
Atp2a2	29693	24.395	0.0020255
Tpi1	24849	-24.416	0.0020255
Epb41	313052	-24.289	0.0020777
Cd36	29184	24.221	0.0020948
Atp1a1	24211	24.15	0.002117

Ndufs7	362837	23.975	0.0022451
Dnajc11	362666	23.915	0.002259
Ndufs1	301458	23.793	0.0022925
Cavin4	313225	-23.83	0.0022925
Sdha	157074	23.683	0.0022972
Myl2	363925	23.624	0.0022972
Ndufab1	293453	23.618	0.0022972
Des	64362	-23.682	0.0022972
Atp5pd	641434	23.538	0.0023394
Pygm	24701	23.202	0.0026126
Tomm22	300075	23.188	0.0026126
Hpx	58917	-23.181	0.0026126
Atpaf2	303190	23.141	0.0026145
Snap23	64630	22.76	0.0029641
Actn2	291245	22.759	0.0029641
Myl6	685867	-22.778	0.0029641
Acsf2	619561	22.694	0.0029996
Timm50	687295	22.663	0.0029996
Pcca	687008	22.439	0.0032721
Anxa6	79125	-22.274	0.003476
Pecr	113956	22.11	0.0036893
L2hgdh	314196	21.811	0.0040332
Samm50	300111	21.788	0.0040332
Epb41l2	309557	-21.792	0.0040332
Agl	362029	-21.794	0.0040332
Mtdh	170910	21.679	0.0040502
Gng12	114120	-21.657	0.0040502
Abcb8	362302	-21.699	0.0040502
Fahd1	302980	-21.74	0.0040502
Myo1c	65261	-21.574	0.0041512
Hspa9	291671	21.466	0.0042481
Ganab	293721	-21.491	0.0042481
Macrod1	246233	-21.352	0.0043624
Lamtor5	295357	-21.356	0.0043624
Atp5mf	690441	-21.235	0.0045444
Cct7	297406	-21.19	0.0045817
Spg7	353231	21.135	0.0045852
Ehd4	192204	-21.135	0.0045852
Myom2	306616	21.095	0.0046037
Ndufs5	362588	21.071	0.0046037
Gnai2	81664	21.025	0.0046037
Vdac2	83531	20.933	0.0046037
Ndufaf6	297821	20.927	0.0046037
Anxa2	56611	-20.9	0.0046037
Pfkm	65152	-20.921	0.0046037
Tubb5	29214	-20.937	0.0046037
Mtch2	295922	-21.019	0.0046037
Atp1a2	24212	-20.771	0.0047793

Vcp	116643	-20.793	0.0047793
Atp5pf	94271	-20.743	0.0047906
Eno1	24333	-20.7	0.0048351
Cyc1	300047	20.654	0.0048367
Rpl9	29257	-20.658	0.0048367
Mgll	29254	20.527	0.0048612
Acadl	25287	20.522	0.0048612
Ckb	24264	-20.512	0.0048612
Ndufb8	293991	-20.532	0.0048612
Capza2	493810	-20.566	0.0048612
Mecr	29470	-20.612	0.0048612
Abca8a	303638	-20.482	0.0048818
S100a10	81778	-20.425	0.0049623
Fbn1	83727	20.334	0.0050482
Fxn	499335	20.285	0.0050482
Rpn2	64701	20.266	0.0050482
Myh7	29557	-20.3	0.0050482
Pgk1	24644	-20.306	0.0050482
Lgals1	56646	-20.344	0.0050482
Letm1	305457	20.235	0.005075
Atp1b1	25650	20.093	0.0052352
Coq3	29309	20.074	0.0052352
Arl6ip5	66028	20.055	0.0052352
Hadh	113965	20.035	0.0052352
Septin2	117515	-20.043	0.0052352
Actn1	81634	-20.056	0.0052352
Got1	24401	-20.071	0.0052352
Mtx1	295241	19.989	0.0052567
Uqcrc2	293448	-20.004	0.0052567
Rps2	83789	-19.911	0.0054042
Alb	24186	-19.851	0.0055079
Gnb1	24400	19.821	0.0055381
Rap1a	295347	19.769	0.0056266
Flnc	362332	-19.749	0.0056332
Psmc2	25581	-19.72	0.0056621
Ndufc2	293130	19.691	0.0056942
Hibadh	63938	-19.647	0.0057622
Ndufv1	293655	19.606	0.0058256
Ndufaf3	56769	19.526	0.0059965
Fam162a	360721	19.407	0.0062125
Hsdl2	313200	19.383	0.0062125
Slc25a4	85333	-19.382	0.0062125
Prkcsh	300445	-19.431	0.0062125
Rab14	94197	19.328	0.0062944
Col14a1	314981	-19.321	0.0062944
Ckmt2	688698	-19.196	0.0066113
Ndufa6	315167	-19.151	0.0066539
Rack1	83427	-19.162	0.0066539

Lonp1	170916	-19.065	0.0068688
Pdia3	29468	-19.037	0.0069061
Cltc	54241	18.99	0.0070067
Sdr39u1	361044	18.919	0.0071387
Itga6	114517	-18.918	0.0071387
Acad10	304500	18.86	0.0072437
Aco2	79250	18.856	0.0072437
Acaa2	170465	-18.837	0.0072559
Cyb5r1	304805	18.731	0.0075134
Mvp	64681	-18.742	0.0075134
Mtx2	288150	18.702	0.0075599
Uqcrfs1	291103	-18.66	0.0076558
Srl	302948	18.601	0.0078095
Tufm	293481	18.525	0.0079852
Arhgdia	360678	-18.524	0.0079852
Unc45b	303373	-18.475	0.0081092
Hsp90b1	362862	18.452	0.0081435
Ndufa9	362440	18.332	0.0084995
Lamp1	25328	18.329	0.0084995
Nap1l4	361684	-18.294	0.0085818
Agk	502749	18.266	0.0086348
Oxct1	690163	18.245	0.0086376
Atp5f1a	65262	-18.238	0.0086376
Eci2	291075	-18.187	0.0087882
Cycs	25309	-18.114	0.0090249
Tln1	313494	18.002	0.0093967
Ywhae	29753	-17.998	0.0093967
Got2	25721	17.97	0.009403
Kyat3	541589	-17.979	0.009403
Hba1	25632	17.945	0.0094547
Lamp2	24944	17.929	0.0094657
Ndufaf2	361894	17.893	0.0095347
Tppp3	291966	-17.888	0.0095347
Tpp1	83534	17.862	0.0095363
Snd1	64635	-17.873	0.0095363
Gfm1	114017	17.805	0.0097258
Atp5f1b	171374	17.767	0.0098344
Dglucy	362769	17.743	0.0098344
Niban2	362115	17.741	0.0098344
Aldh6a1	81708	-17.731	0.0098344
Clpb	65041	-17.63	0.010236
Idh2	361596	17.575	0.010433
Eef1a2	24799	17.421	0.011116
Rgd1565784	497874	17.348	0.0114
Slc25a3	245959	17.341	0.0114
Rab2a	65158	17.306	0.011495
Tagln2	304983	-17.275	0.011495
Hibch	301384	-17.277	0.011495

Ptges2	311865	-17.298	0.011495
Ndufa8	296658	17.209	0.011649
Hsp90ab1	301252	17.199	0.011649
Mccc1	294972	-17.212	0.011649
P4hb	25506	-17.224	0.011649
Vcl	305679	17.158	0.011741
Nt5e	58813	-17.169	0.011741
Rab12	25530	17.11	0.011939
Mb	59108	17.087	0.012001
Pnpla8	314075	17.025	0.012277
Abcc9	25560	16.914	0.012833
Phb	25344	16.901	0.012845
Anxa5	25673	16.872	0.012951
Wdr1	360950	-16.814	0.013225
Flot2	83764	16.766	0.013408
Atp5pb	171375	16.75	0.013408
Snta1	362242	-16.76	0.013408
Hspa8	24468	16.726	0.013491
Lum	81682	-16.71	0.013522
Coq8a	360887	16.691	0.013571
Pnpt1	360992	16.642	0.01374
Actr3	81732	-16.647	0.01374
Jph2	296345	16.628	0.013757
Uqcrb	362897	16.6	0.013796
Ndufa2	291660	-16.591	0.013796
Trap1	287069	-16.598	0.013796
Tecr	191576	16.565	0.013842
Cryab	25420	16.544	0.013842
Ech1	64526	16.532	0.013842
Lrp1	299858	-16.535	0.013842
Bckdha	25244	-16.553	0.013842
Lnpep	171105	-16.491	0.014033
Hk1	25058	-16.461	0.014092
Ywhaq	25577	-16.47	0.014092
Rtraf	302247	-16.401	0.014411
Lactb	300803	16.386	0.014444
Ldb3	498587	16.361	0.014541
Apoo	363474	16.314	0.014718
Ndufa4	681024	16.314	0.014718
Coq5	304542	16.295	0.014764
Gnb2	81667	-16.287	0.014764
Coq9	498909	-16.276	0.014777
Napa	140673	16.234	0.014826
Hsd17b4	79244	16.215	0.014826
Tmem143	308593	16.207	0.014826
Synj2bp	64531	16.191	0.014826
Atp6v0a1	29757	-16.182	0.014826
Clic4	83718	-16.192	0.014826

Cav3	29161	-16.224	0.014826
Vim	81818	-16.245	0.014826
Psme1	29630	-16.257	0.014826
Lama2	309368	16.129	0.01512
Acadm	24158	16.054	0.015563
Erlin2	290823	16.028	0.015685
Cap1	64185	-15.995	0.015846
Ssr4	29435	15.962	0.016015
Hint2	313491	-15.946	0.016063
Afg3l2	307350	15.927	0.016138
Pacsin2	124461	15.873	0.016462
Mrc1	291327	15.815	0.01669
Tomm40	308416	-15.817	0.01669
Acads	64304	-15.832	0.01669
Lap3	289668	15.787	0.016834
Sntb1	299940	15.756	0.016985
Cars2	361184	-15.749	0.016985
Prdx2	29338	-15.737	0.017003
Prdx5	113898	15.714	0.017057
Ak3	26956	15.707	0.017057
Ndufb9	299954	-15.704	0.017057
Lcp1	306071	-15.689	0.017101
Eepd1	315500	15.668	0.01719
Cct5	294864	-15.66	0.01719
Ndufb10	681418	-15.604	0.017559
Slmap	290533	15.591	0.017589
Fam210a	307343	15.528	0.017979
Ndufb7	361385	15.491	0.017979
Pgm1	24645	-15.483	0.017979
Cct3	295230	-15.496	0.017979
Nucb1	84595	-15.498	0.017979
Chchd3	296966	-15.506	0.017979
Cyb5r3	25035	-15.518	0.017979
Slc25a11	64201	15.47	0.018011
Scp2	25541	15.443	0.018159
Hhatl	301073	-15.403	0.018414
Ndufv2	81728	-15.343	0.018841
Rps5	25538	15.311	0.018976
Ehd1	293692	-15.313	0.018976
Me3	361602	-15.3	0.018998
Ank1	306570	15.287	0.019032
Pdk2	81530	15.275	0.019032
Cd59	25407	15.272	0.019032
Decr1	117543	-15.237	0.019259
Pmpca	296588	15.19	0.019591
Cpt2	25413	-15.18	0.019609
Mrpl41	296551	-15.169	0.019631
Psma5	29672	15.12	0.01992

Glud1	24399	-15.121	0.01992
Aldh7a1	291450	15.083	0.020115
Hsp90aa1	299331	-15.084	0.020115
Aifm1	83533	15.024	0.020571
Pbxip1	310644	-15.002	0.020627
Ndufa11	301123	-15.006	0.020627
Lman1	116666	-14.981	0.020751
Mrps23	360594	14.936	0.021091
Pdhb	289950	-14.903	0.021333
Gna13	303634	14.891	0.021374
Txndc5	100362805	14.866	0.021526
Tgm2	56083	14.846	0.021526
Serpinh1	29345	14.844	0.021526
Eif4a1	287436	-14.845	0.021526
Dpysl2	25416	-14.83	0.02159
Ldhb	24534	14.791	0.021888
Cct6a	288620	-14.764	0.021934
Atp5mg	300677	-14.769	0.021934
Cd34	305081	-14.776	0.021934
Mrps22	683519	14.711	0.022374
Mospd1	317312	-14.702	0.022392
Cdh13	192248	14.684	0.022495
Slc25a13	362322	14.656	0.022701
Ctsb	64529	14.635	0.022839
Gpi	292804	14.611	0.023006
Calu	64366	14.597	0.023074
Lrrc57	311346	14.573	0.023181
Dnaja3	360481	-14.572	0.023181
Ndufs3	295923	-14.553	0.023302
Ndufs2	289218	-14.499	0.023778
Rpl3l	287122	14.489	0.023809
Kif5b	117550	14.478	0.023851
Dhrs4	266686	14.44	0.024085
Ivd	24513	14.434	0.024085
Opa1	171116	-14.448	0.024085
Cct8	288305	14.427	0.02409
Ywhah	25576	14.411	0.024182
Mdh2	81829	14.397	0.024256
Hadha	170670	14.365	0.024452
Rps23	124323	-14.365	0.024452
Prdx6	94167	-14.341	0.024552
Pecam1	29583	-14.344	0.024552
Surf1	64463	-14.327	0.024626
Anpep	81641	-14.299	0.024859
Aldh4a1	641316	-14.273	0.025069
Rplp0	64205	14.254	0.025198
Ndufb4	288088	14.163	0.026055
Rtn4ip1	309912	14.16	0.026055

Pdk4	89813	14.157	0.026055
Echs1	140547	-14.145	0.026121
Cav1	25404	14.133	0.026157
Romo1	679572	14.127	0.026157
Aqp1	25240	-14.121	0.026157
Uqcrh	366448	-14.111	0.026203
Agps	84114	14.085	0.026355
Mrpl27	287635	14.084	0.026355
Dguok	297389	14.07	0.026444
Ctsd	171293	14.042	0.026683
Maoa	29253	14.016	0.026764
Pccb	24624	14.015	0.026764
Ldha	24533	14.015	0.026764
Ckm	24265	14.009	0.026764
Gcsh	171133	13.987	0.026942
Dag1	114489	-13.923	0.027634
Prdx3	64371	13.9	0.02778
Sdhb	298596	13.897	0.02778
Septin7	64551	-13.891	0.02778
Fitm1	290223	13.857	0.028037
Capza1	691149	-13.863	0.028037
Dbi	25045	-13.835	0.028224
Pebp1	29542	13.759	0.028942
Suclg2	362404	13.755	0.028942
Poldip2	287544	13.752	0.028942
Ehd2	361512	-13.756	0.028942
Dlst	299201	13.725	0.029202
Mesd	308796	13.684	0.029561
Cox5b	94194	-13.684	0.029561
Bsg	25246	13.663	0.029751
Oxsm	289934	-13.645	0.029915
Itga7	81008	13.637	0.029933
Pitrm1	307081	-13.619	0.030082
Rock1	81762	13.601	0.030242
Speg	363256	-13.589	0.030313
Bckdhb	29711	13.582	0.030321
Nos3	24600	13.572	0.030374
Nars2	293128	13.54	0.030634
Pacsin3	311187	-13.544	0.030634
Ndufa10	678759	-13.532	0.030655
St13	81800	-13.511	0.030779
Dnajc3	63880	-13.513	0.030779
Ak2	24184	-13.482	0.03109
Epb42	362202	13.396	0.03218
Gnaq	81666	13.382	0.032302
Vamp7	85491	13.365	0.032451
Dnajb4	295549	13.332	0.03274
Ugp2	289827	-13.336	0.03274

Txn2	79462	13.306	0.033001
Ldhd	307858	-13.302	0.033001
Rnpep	81761	13.268	0.033129
Rmdn1	500419	-13.263	0.033129
Rnh1	100360501	-13.275	0.033129
Mrpl15	297799	-13.279	0.033129
Rpl23	29282	-13.283	0.033129
Cpt1b	25756	-13.219	0.033682
Sgca	303468	13.209	0.033699
Atp5md	171069	13.192	0.033699
Tpm3	117557	13.191	0.033699
Tubb4b	296554	13.19	0.033699
Coq6	299195	-13.183	0.033699
Tomm70	304017	-13.188	0.033699
Art3	305235	13.157	0.033986
Mrpl38	303685	13.152	0.033986
Tapt1	305386	13.143	0.034021
Scamp1	29521	13.136	0.034021
Erp29	117030	-13.132	0.034021
Ociad1	289590	13.105	0.034251
Iars2	364070	-13.106	0.034251
Mgst3	289197	-13.071	0.034667
Sgcd	497892	13.058	0.034782
Bves	365603	13.042	0.034846
Ywhaz	25578	13.033	0.034846
Msn	81521	13.031	0.034846
Snx2	291464	-13.039	0.034846
Ppib	64367	-13.013	0.035043
Fkbp8	290652	13.002	0.03511
Ywhab	56011	-12.993	0.035167
Gpx4	29328	12.972	0.035403
Ppid	361967	12.959	0.035525
Stip1	192277	12.934	0.035813
Acox1	50681	-12.916	0.036016
Plin4	363331	12.908	0.036022
Popdc2	360718	-12.904	0.036022
Map3k20	311743	12.894	0.036039
C3	24232	12.892	0.036039
Mrpl50	362517	12.878	0.036165
Rab1b	100126191	12.866	0.036166
Cs	170587	12.865	0.036166
Itgav	296456	-12.862	0.036166
Rrbp1	311483	12.848	0.036199
Acot9	302640	12.843	0.036199
Mrps36	294696	-12.849	0.036199
Pls3	81748	12.824	0.036204
Tmem186	497863	12.822	0.036204
Eif2s1	54318	-12.824	0.036204

Ctnnb1	84353	-12.833	0.036204
Csrp3	117505	12.797	0.036333
Fkbp11	300211	-12.799	0.036333
Psmd2	287984	-12.799	0.036333
Gba	684536	12.78	0.036515
Flnb	306204	12.772	0.036562
Gpd1	60666	-12.764	0.036613
Tpt1	116646	-12.726	0.037128
Cox6b1	688869	-12.714	0.037152
Syncrip	363113	-12.718	0.037152
Ecsit	300447	-12.673	0.037725
Psmd13	365388	12.648	0.037982
Plg	85253	12.647	0.037982
Stt3a	500972	12.592	0.038529
Slc25a5	25176	12.578	0.038529
Arf1	64310	12.576	0.038529
Gstz1	681913	12.567	0.038529
Rhoa	117273	-12.568	0.038529
Ndufb6	297990	-12.583	0.038529
Cacna2d1	25399	-12.585	0.038529
Mrps25	297459	-12.589	0.038529
Cyb5a	64001	-12.596	0.038529
Tpm4	24852	-12.552	0.038696
Afg1l	502479	12.541	0.038796
Sgcb	680229	12.524	0.038992
Abcb7	302395	-12.486	0.039556
Mpst	192172	-12.468	0.039778
Mrps6	100360017	12.453	0.039941
Mrpl58	303673	12.448	0.039941
Acot2	192272	12.435	0.039989
Isca2	500694	12.43	0.039989
Rpn1	25596	-12.436	0.039989
Loc100911130	100911130	12.416	0.040149
Por	29441	-12.399	0.040348
Capn2	29154	12.393	0.040368
Dnpep	301529	-12.374	0.040604
Hsd17b10	63864	12.359	0.040716
Mfn1	192647	12.356	0.040716
Dnm2	25751	12.348	0.040716
Add2	24171	-12.353	0.040716
Niban1	63912	-12.328	0.040977
Ndufa5	25488	12.316	0.041065
Canx	29144	12.313	0.041065
Psmd11	303353	-12.296	0.041106
Eef1d	300033	-12.296	0.041106
Bcam	78958	-12.304	0.041106
Tomm40l	304971	12.258	0.04171
Sgcg	305941	12.245	0.041778

Actn4	63836	12.243	0.041778
Anxa11	290527	12.234	0.041778
Casq2	29209	-12.234	0.041778
Ndufa12	299739	-12.211	0.042118
Acsf3	498962	-12.199	0.042242
Gdi2	29662	-12.153	0.042999
Etfb	292845	-12.144	0.043064
Mul1	298576	-12.139	0.043071
Atp6v1a	685232	-12.131	0.04314
Fermt2	289992	-12.097	0.043684
Ndrg2	171114	12.091	0.043696
Hspd1	63868	-12.069	0.043766
Cfl2	366624	-12.071	0.043766
Ndufv3	64539	-12.072	0.043766
Ncam1	24586	-12.079	0.043766
Cox5a	252934	12.047	0.043932
Naxe	295229	12.041	0.043932
Lpcat3	362434	12.041	0.043932
Atp5po	192241	-12.048	0.043932
Zadh2	291403	-12.025	0.04415
Hrc	292905	12.005	0.044435
Calm3	24244	11.988	0.044676
Eef2	29565	-11.966	0.045005
Cox4i1	29445	11.942	0.045276
Dbt	29611	-11.944	0.045276
Vdac3	83532	11.913	0.045759
Mlycd	85239	-11.845	0.047025
Glg1	29476	11.837	0.047091
D2hgdh	301624	11.806	0.047624
Pdcd6ip	501083	11.798	0.047689
Vps4a	246772	-11.775	0.048063
Rab10	50993	11.76	0.048233
Asph	312981	-11.758	0.048233
Pcyox1	246302	11.749	0.048306
Maip1	301418	11.734	0.048538
Rpl19	81767	-11.725	0.048626
Fga	361969	11.713	0.048644
Clybl	306198	11.704	0.048644
Ddx1	84474	-11.701	0.048644
Rab7a	29448	-11.702	0.048644
Atp5me	140608	-11.709	0.048644
Sirt3	293615	-11.693	0.048717
Mrps7	113958	11.672	0.049023
Ndufa13	100911483	-11.67	0.049023
Plpp7	296635	11.641	0.04952
Sec22b	310710	11.62	0.049881
Camk2d	24246	11.613	0.049898
Gcdh	364975	11.61	0.049898

The differential expression proteins were identified from a combined proteomic profile of the heart and GA muscle MAM fraction (using Fisher’s method for P value combination) by NetworkAnalyst 3.0. An optimized cutoff of combined P <0.05 was used to determine age-related MAM proteins in the striated muscle.
