# Supplementary material for: Ultrastructural and proteomic profiling of mitochondria-associated endoplasmic reticulum membranes reveal aging signatures in striated muscle
Source: Cell Death Dis. 2022 Apr 2;13(4):296. doi: 10.1038/s41419-022-04746-4 (PMC8976840; doi:10.1038/s41419-022-04746-4)
Supplement: Supplementary file 7 — Supplementary Table 5. Consensus MAM proteins. [file 41419_2022_4746_MOESM7_ESM.pdf]

Supplementary Table 5a. Consensus MAM protein list

A - Rat heart in this work	B - Rat skeletal muscle in this work	A^B intersection	C - Mouse liver - Nat Cell Biol. 2019 [19]	D - Mouse liver - Sci Rep. 2016 [20]	E - Mouse liver & brain - J. Prot. 2013 [21]	A^B^C^D^E intersection	Other canonical MAM proteins from the literature		Consensus MAM proteins described by Carreras-Sureda A et al. [19]	A^B^Consensus MAM proteins intersection
1871 hits	1695 hits	1306 hits	1466 hits	1046 hits	292 hits	170 hits	19 hits	PMID	216 hits	176 hits
A1i3	March5	A1i3	Tspan9	Aadac	Abat	Abcb7	GRP78	28522876	Tmed10	Abcb7
A1m	A1i3	A1m	Faf2	Aadat	Abcb7	Abcd3	Calr	24434800	Mogs	Abcd3
Aak1	A1m	Aars2	Cyp3a11	Aass	Abcb8	Acaa2	Itpr1	28942427	Aldh1l1	Acaa2
Aars2	Aars2	Abca8a	Spcs3	Abat	Abcd3	Acadl	Itpr2	9278423	Itpr1	Acadl
Abca8a	Abca2	Abca9	Tars	Abca6	Acaa1	Acat1	Sigmar1	17981125	Apoc1	Acat1
Abca9	Abca8a	Abcb10	Cenpv	Abcb11	Acaa2	Aco2	ERo1a	21854214	Cdc42bpb	Aco2
Abcb10	Abca9	Abcb6	Alg9	Abcb6	Acadl	Acox1	Ero1b	21854214	Rplp0	Acox1
Abcb6	Abcb10	Abcb7	Alg2	Abcb7	Acat1	Acsl1	Tmbim6	PMC4046133	Anxa6	Acsl1
Abcb7	Abcb6	Abcb8	Nup210	Abcc3	Aco2	Actn1	Mfn1	PMC3167523	Ndrg2	Actn1
Abcb8	Abcb7	Abcc1	Inpp5b	Abcc6	Acox1	Actn4	Erp44	15652484	Rpl6	Actn4
Abcc1	Abcb8	Abcc9	Kdelr1	Abcd1	Acsl1	Afg3l2	Rab32	20670942	Clic4	Afg3l2
Abcc4	Abcc1	Abcd3	Serpinc1	Abcd3	Actc1	Aifm1	Apob	2332429	Rab5b	Aifm1
Abcc9	Abcc9	Abce1	Tmed10	Abhd12	Actg1	Alb	Apoc1	2332429	Calr	Alb
Abcd1	Abcd2	Abhd11	Pdia5	Abhd14b	Actn1	Aldh6a1	Ptdss1	10938271	Rab1A	Aldh6a1
Abcd3	Abcd3	Abhd16a	Mogs	Abhd6	Actn4	Aldh9a1	Acsl4	11319232	park7	Aldh9a1
Abce1	Abce1	Acaa1a	Proc	Acaa1a	Afg3l2	Anxa6	Dgat2	19049983	Ncstn	Anxa6
Abhd10	Abcf1	Acaa2	Yipf4	Acaa1b	Ahcy	Ap2a1	Mttp	7961664	Mdh1	Ap2a1
Abhd11	Abcf2	Acad10	Strn3	Acaa2	Aifm1	Ap2a2	Scd1	7961664	Pdia3	Ap2a2
Abhd16a	Abhd11	Acad8	Chid1	Acad10	Ak3	Ap2b1	Park7	23418303	Ndufb6	Ap2b1
Acaa1a	Abhd16a	Acad9	Ces1	Acad11	Alb	Apoe			GRP 78	Apoe
Acaa2	Abhd6	Acadl	Ica	Acad8	Alcam	Atp1a1			Alcam	Atp1a1
Acaca	Acaa1a	Acadm	Myo7b	Acad9	Aldh1l1	Atp1b1			Apob	Atp1b1
Acad10	Acaa2	Acads	Use1	Acadl	Aldh2	Atp2a2			Rpl10a	Atp2a2
Acad11	Acad10	Acadsb	Cyp4a12a	Acadm	Aldh6a1	Atp6v1a			Rpl18	Atp6v1a
Acad8	Acad8	Acadvl	Serpind1	Acads	Aldh9a1	Bcap31			Itgb1	Bcap31
Acad9	Acad9	Acat1	Lmnb1	Acadsb	Anxa5	Bdh1			Hspa8	Bdh1
Acadl	Acadl	Aco2	Abcb4	Acadvl	Anxa6	Canx			Cox5a	Canx
Acadm	Acadm	Acot13	Suox	Acat1	Ap2a1	Capza2			Erlin2	Capza2
Acads	Acads	Acot2	Tmod3	Acbd5	Ap2a2	Cct8			Cycs	Cct8
Acadsb	Acadsb	Acot8	Aldh1l1	Acly	Ap2b1	Cd81			Gstp1	Cd81
Acadvl	Acadvl	Acot9	Cpt1a	Acnat1	Apoe	Cfl1			Mttp	Cfl1
Acat1	Acat1	Acox1	Tmem259	Aco1	Arf3	Cisd1			Arpc4	Cisd1
Aco2	Ace	Acsf2	Lsr	Aco2	Arpc4	Clic4			Mccc1	Clic4
Acot11	Aco2	Acsf3	Tm9sf3	Acot1	Ass1	Cltc			Pacsin3	Cltc
Acot13	Acot13	Acsl1	Fgg	Acot12	Atp1a1	Cox4i1			Maob	Cox4i1
Acot2	Acot2	Acsl6	Kat2b	Acot4	Atp1b1	Cox5a			Pdia6	Cox5a
Acot7	Acot8	Acss1	Hist1h1e	Acot8	Atp2a2	Cs			Ppib	Cs
Acot8	Acot9	Acta1	Lima1	Acox1	Atp2b4	Cyc1			Ptprf	Cyc1
Acot9	Acox1	Actn1	Krt76	Acox2	Atp5a1	Cycs			Bdh1	Cycs
Acox1	Acsf2	Actn2	Arpc1b	Acox3	Atp5b	Ddost			Srprb	Ddost
Acox3	Acsf3	Actn4	Mccc2	Acsf2	Atp5c1	Decr1			Tmem30a	Decr1
Acp6	Acsl1	Actr3	Hmgb1	Acsf3	Atp5h	Dlat			Ndufa9	Dlat
Acsf2	Acsl3	Add1	Slc33a1	Acsl1	Atp5j	Dnm2			Rpl9	Dnm2
Acsf3	Acsl4	Add2	M6pr	Acsl4	Atp5o	Echs1			Uqcrc2	Echs1
Acsl1	Acsl6	Adhfe1	Itpr1	Acsl5	Atp6v1a	Eef1a1			Prkcsh	Eef1a1
Acsl6	Acss1	Adsl	Lpgat1	Acsm1	Auh	Eef2			Tmbim6	Eef2
Acss1	Acss3	Adssl1	Dnajc25	Acsm3	Baiap2	Eno1			Rab18	Eno1
Acta1	Acta1	Afg1l	Dipk2a	Acsm5	Bcap31	Erlin2			Lap3	Erlin2
Actc1	Actn1	Afg3l2	Apoc1	Acta2	Bdh1	Erp29			Acox1	Erp29

Actn1	Actn2	Agk	Cpne3	Actb	C10orf58	Etfa
Actn2	Actn3	Agl	Vapb	Actn1	C1qbp	Etfdh
Actn4	Actn4	Agpat3	Pigr	Actn2	Ca14	Fh
Actr1a	Actr3	Agps	Ces3b	Actn4	Calm1	Ganab
Actr1b	Acyp2	Ahsa1	Cdc42bpb	Actr2	Canx	Glud1
Actr2	Adam9	Ahsg	Emc4	Actr3	Capza2	Gna13
Actr3	Adcy9	Aifm1	Hist1h1c	Adh1	Cct7	Gnai2
Acy1a	Add1	Aimp1	Serpina1e	Adh5	Cct8	Gnas
Ada	Add2	Ak1	Ugt3a2	Adhfe1	Cd47	Gnb1
Adck1	Adgre5	Ak2	Capzb	Afg3l2	Cd81	Got2
Adcy5	Adh5	Ak3	Rps26	Agmat	Cdc42bpb	Gpd2
Add1	Adhfe1	Akap12	Daam1	Agpat2	Cdh2	Gstp1
Add2	Adpgk	Akr1b1	Aco1	Agps	Cfl1	Hadha
Add3	Adsl	Alb	Rplp0	Agxt	Cisd1	Hadhb
Adgrl2	Adssl1	Aldh3a2	Hacl1	Agxt2	Clic4	Hsd17b4
Adhfe1	Afg1l	Aldh4a1	Abca8b	Ahcy	Cltc	Hsp90ab1
Adipoq	Afg3l2	Aldh5a1	Abcg2	Ahsg	Cox2	Hsp90b1
Adprhl1	Agk	Aldh6a1	Pdia4	Aifm1	Cox4i1	Hspa8
Adsl	Agl	Aldh7a1	Rps27	Ak2	Cox5a	Hspa9
Adssl1	Ago2	Aldh9a1	Txndc5	Ak3	Cox7a2	Hspd1
Afg1l	Agpat1	Aldoa	Slc22a1	Akr1a1	Cs	Hyou1
Afg3l1	Agpat3	Ank1	Slc35c1	Akr1c6	Cyc1	Idh2
Afg3l2	Agps	Ank3	Anxa6	Akr7a2	Cycs	Immt
Agk	Ahnak	Ano10	Cyp2j6	Alb	Cyfip1	Itgb1
Agl	Ahsa1	Ano6	Nt5c3a	Alcam	Cyp2d6	Lap3
Agpat3	Ahsg	Anp32a	Npc1	Aldh1a1	Ddost	Ldha
Agpat5	Aifm1	Anpep	Esyt2	Aldh1b1	Ddx5	Letm1
Agps	Aimp1	Anxa1	Selo	Aldh1l1	Decr1	Lrp1
Agrn	Ak1	Anxa11	Atp13a1	Aldh2	Dlat	Maob
Ahcy	Ak2	Anxa2	Rab35	Aldh3a2	Dlst	Mccc1
Ahcyl1	Ak3	Anxa3	Krt18	Aldh4a1	Dnajc13	Mdh1
Ahsa1	Akap12	Anxa4	Stx7	Aldh5a1	Dnm2	Mdh2
Ahsg	Akap13	Anxa5	Taok3	Aldh6a1	Dync1h1	Mlec
Aifm1	Akr1b1	Anxa6	Mboat7	Aldh7a1	Echs1	Mogs
Aimp1	Akr7a2	Anxa7	Ndrg2	Aldh8a1	Eef1a1	Msn
Ak1	Alb	Aoc3	Rab5a	Aldh9a1	Eef2	Mtdh
Ak2	Aldh1l1	Ap1b1	Rpl6	Aldob	Efhd2	Mthfd1
Ak3	Aldh3a2	Ap2a1	Utp20	Alg2	Eif4a1	Myo1b
Ak4	Aldh4a1	Ap2a2	Vcl	Alg5	Eno1	Ndrg2
Akap1	Aldh5a1	Ap2b1	Hm13	Amacr	Erlin2	Ndufa13
Akap12	Aldh6a1	Ap2m1	Erlec1	Ambp	Erp29	Ndufa4
Akap2	Aldh7a1	Apmap	Hist1h1d	Amt	Esyt2	Ndufa9
Akr1a1	Aldh9a1	Apoa1	Ubxn4	Anp32b	Etf1	Ndufb10
Akr1b1	Aldoa	Apoa4	Timeless	Anpep	Etfa	Ndufb4
Akr1c15	Alg2	Apoe	Flnb	Anxa2	Etfdh	Ndufb5
Alb	Ampd1	Apoh	H2-Q8	Anxa4	Fam82a2	Ndufb6
Alcam	Ank1	Apoo	Slc16a10	Anxa6	Fh	Ndufs1
Aldh1a1	Ank3	Apool	Tex11	Aox3	Fkbp8	Ndufs2
Aldh3a2	Ankrd2	App	Slmap	Ap1b1	Ganab	Ndufs3
Aldh4a1	Ano10	Aprt	Tyk2	Ap2a1	Gcn1l1	Ndufs7
Aldh5a1	Ano6	Aqp1	Ergic1	Ap2a2	Glud1	Ndufv1
Aldh6a1	Anp32a	Arf1	Hist1h4a	Ap2b1	Gm10053	Ndufv2
Aldh7a1	Anpep	Arfip1	Clic4	Ap2m1	Gm9755	Nomo1
Aldh9a1	Anxa1	Arhgap1	Rpl31	Apmap	Gna13	P4hb
Aldoa	Anxa11	Arhgdia	Ces3a	Apoa1	Gnai2	Pa2g4
Alpl	Anxa2	Arl6ip5	Sec22b	Apob	Gnas	Pacsin3
Amacr	Anxa3	Arl8b	Ftcd	Apoe	Gnb1	Pc

Ero1b	Etfa
Uqcrfs1	Etfdh
Aldh9a1	Fh
Idh2	Ganab
Sptbn1	Glud1
Ndufb4	Gna13
Atp5h	Gnai2
Slc25a1	Gnas
Jup	Gnb1
Dnm2	Got2
Actn1	Gpd2
Lrp1	Gstp1
Capza2	Hadha
Hsp90b1	Hadhb
Itpr2	Hsd17b4
Uqcrc1	Hsp90ab1
Cyfip1	Hsp90b1
Rab1b	Hspa8
Acsl4	Hspa9
Cox4i1	Hspd1
Scd1	Hyou1
Cltc	Idh2
Vdac3	Immt
P4hb	Itgb1
Atp2a2	Lap3
Ndufb10	Ldha
Prdx5	Letm1
Pgk1	Lrp1
Glud1	Maob
Rps3a	Mccc1
Rps14	Mdh1
Apoe	Mdh2
Rpl8	Mlec
Decr1	Mogs
Ass1	Msn
Tst	Mtdh
Rab32	Mthfd1
Uggt1	Myo1b
Abcb7	Ndrg2
Ero1a	Ndufa13
Letm1	Ndufa4
Ndufv2	Ndufa9
Gnas	Ndufb10
Tpi1	Ndufb4
Etfa	Ndufb5
Ap2b1	Ndufb6
Ldha	Ndufs1
Hadhb	Ndufs2
Picalm	Ndufs3
Mdh2	Ndufs7
Prdx1	Ndufv1
Canx	Ndufv2
Atp6v1a	Nomo1
Etfdh	P4hb
Ganab	Pa2g4
Serpina3k	Pacsin3
Qdpr	Pc

Ank1	Anxa4	Armc10	Agmo	Apoh	Gnb2l1	Pcyox1
Ank2	Anxa5	Arpc1b	Rab5b	Apoo	Gng12	Pdia3
Ank3	Anxa6	Arpc3	Calr	Apool	Got1	Pdia6
Ano10	Anxa7	Art3	Gusb	Arf1	Got2	Pgk1
Ano6	Aoc3	Asah1	H2-T23	Arf6	Gpd2	Pgm1
Anp32a	Ap1b1	Asph	Scarb2	Arg1	Grhpr	Phb
Anp32b	Ap1m1	Atad1	Slc25a23	Arl6ip5	Gstm5	Phb2
Anp32e	Ap2a1	Atad3	Tsc2	Arl8b	Gstp1	Picalm
Anpep	Ap2a2	Atic	Polq	Arpc1b	Hadha	Ppib
Anxa1	Ap2b1	Atl3	Tomm7	Arpc2	Hadhb	Prdx1
Anxa11	Ap2m1	Atp13a1	Dbt	Arpc4	Hba1	Prdx5
Anxa2	Ap3d1	Atp1a1	Farsa	Asah1	Hba2	Prdx6
Anxa3	Apmap	Atp1a2	Tomm20	Asgr1	Hbb	Prkcsh
Anxa4	Apoa1	Atp1b1	Rab1A	Asl	Hbd	Qdpr
Anxa5	Apoa2	Atp1b3	Tkt	Asph	Hrsp12	Rab14
Anxa6	Apoa4	Atp2a2	Slc27a2	Ass1	Hsd17b4	Rab18
Anxa7	Apobec2	Atp2b1	Fas	Atad1	Hsp90aa1	Rab1b
Aoc3	Apoc1	Atp5f1a	Park7	Atad3	Hsp90ab1	Rab21
Ap1b1	Apoc3	Atp5f1b	Vamp8	Atl2	Hsp90b1	Rab2a
Ap2a1	Apoe	Atp5f1c	Pygl	Atl3	Hspa1a	Rab5b
Ap2a2	Apoh	Atp5f1d	Eps8l2	Atp11c	Hspa1b	Rab7a
Ap2b1	Apoo	Atp5if1	Ncstn	Atp12a	Hspa5	Rala
Ap2m1	Apool	Atp5md	Nceh1	Atp13a1	Hspa8	Rdx
Ap2s1	App	Atp5me	Rab11a	Atp1a1	Hspa9	Rpl10
Ap3b1	Aprt	Atp5mf	Crlf1	Atp1b1	Hspd1	Rpl10a
Ap3m1	Aqp1	Atp5mg	Krt1	Atp1b3	Hyou1	Rpl18
Apeh	Arf1	Atp5pb	Abcg8	Atp2a1	Idh1	Rpl4
Apmap	Arfip1	Atp5pd	Dlg1	Atp2a2	Idh2	Rpl5
Apoa1	Arhgap1	Atp5pf	Ap2s1	Atp5f1a	Immt	Rpl6
Apoa4	Arhgdia	Atp5po	Slc22a7	Atp5f1b	Itgb1	Rpl7
Apoe	Arl6ip5	Atp6v0a1	Gc	Atp5f1c	Jup	Rpl8
Apoh	Arl8b	Atp6v0d1	Tf	Atp5f1d	Kiaa0090	Rpl9
Apoo	Armc10	Atp6v1a	Cant1	Atp5me	Kpnb1	Rplp0
Apool	Arpc1b	Atp6v1b2	Pah	Atp5mf	Lap3	Rplp2
App	Arpc3	Atp6v1e1	Mdh1	Atp5mg	Ldha	Rpn1
Appl1	Art1	Atp6v1h	Steap4	Atp5mj	Letm1	Rps14
Aprt	Art3	Atpaf1	Snx3	Atp5pb	Lrp1	Rps24
Aqp1	Asah1	Atpaf2	Rab8a	Atp5pd	Lrpprc	Rps3
Arf1	Asph	Auh	B3gat3	Atp5po	Maob	Rps3a
Arf6	Aspn	B2m	Aldoa	Atp6v0c	Mccc1	Rps8
Arfip1	Aspscr1	Bcam	Napg	Atp6v0d1	Mdh1	Rps9
Arhgap1	Atad1	Bcap29	Stard10	Atp6v1a	Mdh2	Rrbp1
Arhgdia	Atad3	Bcap31	Pdia3	Atp6v1b2	Mlec	Samm50
Arhgdib	Atg9a	Bcat2	Anxa4	Auh	Mogs	Sdha
Arhgef1	Atic	Bckdha	Ecsit	Azgp1	Mrpl12	Sdhb
Arhgef15	Atl2	Bckdhb	Bhmt	B4galnt1	Msn	Slc16a1
Arih1	Atl3	Bckdk	C3	Baat	Mtdh	Slc25a1
Arl6ip5	Atp13a1	Bcl2l13	Ndufb6	Bcap31	Mthfd1	Slc25a12
Arl8a	Atp1a1	Bcs1l	Slc39a7	Bckdha	Myh9	Slc25a3
Arl8b	Atp1a2	Bdh1	Mia3	Bckdhb	Myl9	Snd1
Armc1	Atp1b1	Bgn	Rab5c	Bcs1l	Myo18a	Stoml2
Armc10	Atp1b2	Bnip3	Gdi2	Bdh1	Myo1b	Suclg1
Arpc1b	Atp1b3	Bphl	Creb3l3	Bhmt	Myo1d	Tmed10
Arpc2	Atp1b4	Bpnt1	Tmed5	Bphl	Ncstn	Tpi1
Arpc3	Atp2a1	Bsg	Serpina1a	Bsg	Ndrg2	Tst
Arpc4	Atp2a2	Bves	Hsdl2	Btd	Ndufa13	Uggt1
Arpc5	Atp2a3	Bzw2	Dhcr24	C1galt1	Ndufa4	Uqcrc1

Pgrmc1	Pcyox1
Eef1a1	Pdia3
Rps3	Pdia6
Gnb1	Pgk1
Vdac2	Pgm1
Dlat	Phb
Cfl1	Phb2
Sfxn1	Picalm
Hyou1	Ppib
Acsl1	Prdx1
Hspa9	Prdx5
Hsp90ab1	Prdx6
Slc25a3	Prkcsh
Myh9	Qdpr
Sigmar1	Rab14
Hspd1	Rab18
Alb	Rab1b
Rps8	Rab21
Sdhb	Rab2a
Afg3l2	Rab5b
Cyc1	Rab7a
Gna13	Rala
Mtdh	Rdx
Atp5a1	Rpl10
Erp44	Rpl10a
Samm50	Rpl18
Acaa2	Rpl4
Gpd2	Rpl5
Cisd1	Rpl6
Rpl7	Rpl7
Prdx6	Rpl8
Slc25a12	Rpl9
Vapa	Rplp0
Pgm1	Rplp2
Phb2	Rpn1
Ap2a2	Rps14
Stoml2	Rps24
Ptdss1	Rps3
Ndufs3	Rps3a
Ndufa4	Rps8
Acat1	Rps9
Atp5o	Rrbp1
Ahcy	Samm50
Rpl4	Sdha
Immt	Sdhb
Rpl10	Slc16a1
Atp5c1	Slc25a1
Rab2a	Slc25a12
Rpl5	Slc25a3
Dgat2	Snd1
Pa2g4	Stoml2
Pc	Suclg1
Myo1b	Tmed10
Ap2a1	Tpi1
Atp5b	Tst
Eef2	Uggt1
Rplp2	Uqcrc1

Art3	Atp2b1	C1qbp	Rab6a	C3	Ndufa6	Uqcrc2
Art4	Atp5f1a	C3	Slc4a4	C4b	Ndufa8	Uqcrfs1
Asah1	Atp5f1b	C4	Sel1l	C4bpa	Ndufa9	Vapa
Asph	Atp5f1c	C4bpa	Ces1f	C5	Ndufb10	Vcp
Atad1	Atp5f1d	C9	Ctsl	C6	Ndufb11	Vdac1
Atad3	Atp5f1e	Ca14	Alcam	C8a	Ndufb4	Vdac2
Atic	Atp5if1	Cab39	Mgst1	C8b	Ndufb5	Vdac3
Atl1	Atp5md	Cacna2d1	H1f0	C8g	Ndufb6	
Atl3	Atp5me	Calm3	Cttn	Ca3	Ndufb7	
Atp13a1	Atp5mf	Calr	Pi4ka	Ca5a	Ndufb8	
Atp1a1	Atp5mg	Calu	Pigt	Calr	Ndufs1	
Atp1a2	Atp5mpl	Camk2d	Map3k1	Calu	Ndufs2	
Atp1b1	Atp5pb	Camk2g	Lmna	Canx	Ndufs3	
Atp1b3	Atp5pd	Cand1	Ier3ip1	Cap1	Ndufs7	
Atp2a2	Atp5pf	Cand2	Slc9a3r1	Capza2	Ndufv1	
Atp2b1	Atp5po	Canx	Gjb1	Cat	Ndufv2	
Atp2b4	Atp6v0a1	Cap1	Pkm	Cbr4	Nnt	
Atp5f1a	Atp6v0d1	Cap2	Apob	Ccdc47	Nomo1	
Atp5f1b	Atp6v1a	Capn1	Rab9a	Cct2	P4hb	
Atp5f1c	Atp6v1b2	Capn2	Hacd3	Cct3	Pa2g4	
Atp5f1d	Atp6v1e1	Capns1	Ddx27	Cct5	Pacsin3	
Atp5if1	Atp6v1h	Capza1	Rpl10a	Cct6a	Pc	
Atp5md	Atp8a1	Capza2	H2-L	Cct8	Pcyox1	
Atp5me	Atp9a	Capzb	Ccdc47	Cd81	Pdhb	
Atp5mf	Atpaf1	Cars2	Slc35e1	Cdc42bpb	Pdia3	
Atp5mg	Atpaf2	Casq2	Aspm	Cdipt	Pdia6	
Atp5pb	Auh	Cat	Sts	Cept1	Pebp1	
Atp5pd	Aup1	Cav1	Reep6	Cers2	Pfn1	
Atp5pf	B2m	Cav3	Cyp3a25	Ces1	Pgam1	
Atp5po	Bag2	Cavin1	Aldob	Ces1b	Pgk1	
Atp6v0a1	Banf1	Cavin2	Synj2bp	Ces1c	Pgm1	
Atp6v0d1	Basp1	Cavin4	Rpl18	Ces1d	Pgrmc1	
Atp6v1a	Bcam	Cbr1	Atm	Ces1e	Phb	
Atp6v1b2	Bcap29	Cbr4	Pdzk1	Ces1f	Phb2	
Atp6v1d	Bcap31	Ccdc127	Pgap1	Ces2a	Pi4ka	
Atp6v1e1	Bcas1	Ccdc47	Rab17	Ces2b	Picalm	
Atp6v1h	Bcat2	Ccdc51	Mpc1	Ces2c	Plcb3	
Atpaf1	Bckdha	Ccdc90b	H2-D1	Ces2e	Plec	
Atpaf2	Bckdhb	Ccpg1	Dolpp1	Ces2g	Ppia	
Atxn10	Bckdk	Cct2	Myh10	Ces3a	Ppib	
Auh	Bcl2l13	Cct3	Cyp2b10	Ces3b	Ppp2r1a	
B2m	Bcs1l	Cct4	Stt3b	Cfb	Prdx1	
Bag3	Bdh1	Cct5	Gltpd2	Cfh	Prdx5	
Bcam	Bgn	Cct6a	Itgb1	Cfl1	Prdx6	
Bcap29	Bnip3	Cct7	Pex5	Chdh	Prkcsh	
Bcap31	Bola3	Cct8	Gulo	Cisd1	Ptprf	
Bcat2	Bphl	Cd163	Slc26a1	Cisd2	Qdpr	
Bche	Bpnt1	Cd1d	Hspa8	Ckmt2	Rab11b	
Bckdha	Bsg	Cd34	Qsox2	Clec4f	Rab14	
Bckdhb	Btf3	Cd36	Pex11g	Clic4	Rab18	
Bckdk	Btnl10	Cd47	Myh14	Clpp	Rab1a	
Bcl2l13	Bves	Cd48	Ndufb9	Clptm1	Rab1b	
Bcs1l	Bzw2	Cd59	Ldah	Clptm1l	Rab21	
Bdh1	C1qa	Cd81	Cox5a	Clpx	Rab2a	
Bgn	C1qbp	Cd9	Cml2	Cltc	Rab5b	
Blvrb	C3	Cd99	Arl8b	Clu	Rab5c	
Bnip1	C4	Cdc42	Colec12	Clybl	Rab6a	

Hadha	Uqcrc2
Ndufb5	Uqcrfs1
Hrsp12	Vapa
Idh1	Vcp
Rps9	Vdac1
Fh	Vdac2
Hsd17b4	Vdac3
Aifm1	GRP78
Msn	Mfn1
Mfn1	Erp44
Rps24	Park7
Rrbp1	Calr
Vdac1	Acsl4
Mthfd1	
Snd1	
Phb	
Eno1	
Ndufv1	
Cd81	
Rdx	
Actn4	
Ndufs1	
Pcyox1	
Cct8	
Mlec	
Rpn1	
Cs	
Suclg1	
Nomo1	
Got2	
Rala	
Aldh2	
Erp29	
Rab7a	
Ndufs7	
Slc16a1	
Rab14	
Rab21	
Ndufa13	
Abcd3	
Echs1	
Atp1a1	
Sdha	
Gnai2	
Aco2	
Acadl	
Bcap31	
Ndufs2	
Aldh6a1	
Vcp	
Atp1b1	
Ddost	
Mccc2	

Bnip3	C4bpa	Cdh13	Erlin2	Cml1	Rab7a
Bphl	C9	Cdnf	Cr1l	Cnpy2	Rala
Bpnt1	Ca1	Ces1d	Cycs	Coasy	Rdx
Bsg	Ca14	Cfh	Gsta1	Cobll1	Rpl10
Bves	Ca3	Cfl1	Emc7	Colgalt1	Rpl10a
Bzw2	Cab39	Cfl2	Tap1	Comt	Rpl13p12
C1qb	Cacna1s	Chchd3	Sec62	Coq8a	Rpl18
C1qbp	Cacna2d1	Chchd6	Rras	Coro1a	Rpl4
C3	Cacnb1	Chid1	Lamp1	Coro1b	Rpl5
C4	Cacng6	Chp1	Cpt2	Cox15	Rpl6
C4bpa	Cadm4	Cisd1	Atp6v0a1	Cox4i1	Rpl7
C7	Calm3	Cisd2	Ei24	Cox5a	Rpl8
C9	Calr	Ckap4	Lactb	Cox5b	Rpl9
Ca14	Calu	Ckb	Itih4	Cox6b1	Rplp0
Ca4	Camk2a	Ckm	Psma6	Cox6c	Rplp2
Cab39	Camk2b	Ckmt2	Glg1	Cp	Rpn1
Cacna1c	Camk2d	Clec10a	Helz2	Cpox	Rps14
Cacna2d1	Camk2g	Clic4	Coro1c	Cpq	Rps20
Cacna2d2	Cand1	Clpb	Rpl30	Cps1	Rps24
Cacnb2	Cand2	Clpp	Rps27a	Cpt1a	Rps3
Cald1	Canx	Clpx	Egfr	Cpt2	Rps3a
Calm3	Cap1	Cltb	Ogdh	Creld1	Rps6
Calr	Cap2	Cltc	Lemd2	Creld2	Rps8
Calu	Capg	Clybl	Derl1	Crot	Rps9
Camk2d	Capn1	Cmc1	Ggt5	Cryz	Rpsa-ps10
Camk2g	Capn2	Cndp2	Plec	Cs	Rrbp1
Cand1	Capns1	Cnp	Cldn3	Cth	Samm50
Cand2	Capza1	Coa3	Dhcr7	Ctnna1	Scamp3
Canx	Capza2	Cobl	F10	Ctnnb1	Sdha
Cap1	Capzb	Col14a1	Asph	Ctnnd1	Sdhb
Cap2	Cars2	Col6a1	Slc25a21	Ctsb	Serpina3k
Capn1	Casq1	Col6a2	Gm4952	Ctsc	Sfxn1
Capn2	Casq2	Colec12	Gstp1	Ctsd	Slc16a1
Capns1	Cat	Comt	Acad11	Ctsf	Slc25a1
Capza1	Cav1	Copa	Clcc1	Cyb5b	Slc25a12
Capza2	Cav3	Copg1	Krt5	Cyb5r3	Slc25a23
Capzb	Cavin1	Cops8	Clec2d	Cyc1	Slc25a3
Card19	Cavin2	Coq10a	Rps16	Cycs	Slc25a6
Cars2	Cavin4	Coq3	Ces2e	Cyfip1	Slc27a4
Casq2	Cbr1	Coq5	Hp	Cyp1a1	Snd1
Cat	Cbr4	Coq6	Txndc15	Cyp1a2	Snx2
Cav1	Ccdc127	Coq7	Mttp	Cyp27a1	Sptbn1
Cav3	Ccdc47	Coq8a	Rhog	Cyp2a12	Sptbn2
Cavin1	Ccdc51	Coq9	Rbp4	Cyp2a4	Srprb
Cavin2	Ccdc90b	Coro1c	Tln1	Cyp2a5	Ssr3
Cavin3	Ccpg1	Cox16	Galnt2	Cyp2b10	Stoml2
Cavin4	Cct2	Cox18	Arpc4	Cyp2c23	Suclg1
Cbr1	Cct3	Cox4i1	Acadm	Cyp2c29	Tars
Cbr4	Cct4	Cox5a	Lonp2	Cyp2c37	Timm13
Ccdc127	Cct5	Cox5b	Tmem33	Cyp2c38	Tmed10
Ccdc141	Cct6a	Cox6b1	Nucb1	Cyp2c39	Tmem30a
Ccdc47	Cct7	Cox6c2	Mccc1	Cyp2c40	Tomm70a
Ccdc51	Cct8	Cox7a2	Top2b	Cyp2c50	Tpi1
Ccdc58	Cd163	Cox7a2l	Pacsin3	Cyp2c54	Tpm3
Ccdc90b	Cd1d	Cox7c	Sema4d	Cyp2c67	Tst
Ccny	Cd34	Cp	Icam1	Cyp2c68	Tuba1c
Ccpg1	Cd36	Cpox	Myo1d	Cyp2c70	Tuba4a

Cct2	Cd44	Cpq	Mia2	Cyp2d10	Tubb3
Cct3	Cd47	Cpt1b	Slc6a13	Cyp2d22	Tubb4b
Cct4	Cd48	Cpt2	Ddb1	Cyp2d26	Uggt1
Cct5	Cd55	Crat	Insr	Cyp2d9	Uqcrc1
Cct6a	Cd59	Creld1	Fndc3a	Cyp2e1	Uqcrc2
Cct7	Cd81	Cryab	Ufsp2	Cyp2f2	Uqcrfs1
Cct8	Cd9	Cs	Calm1	Cyp2j5	Vapa
Ccz1b	Cd99	Csnk1a1	Tmx2	Cyp3a11	Vapb
Cd163	Cdc42	Csnk2a1	Bgn	Cyp3a13	Vcp
Cd1d	Cdh13	Csrp3	Farp1	Cyp3a16	Vdac1
Cd200	Cdk5rap3	Cst3	Maob	Cyp3a25	Vdac2
Cd300lg	Cdnf	Ctnnb1	Hint2	Cyp3a41a	Vdac3
Cd34	Cemip2	Ctnnd1	Ces1d	Cyp3a41b	Vps35
Cd36	Ces1d	Ctsa	Sdf2l1	Cyp4a10	Ywhab
Cd38	Cfh	Ctsb	Qsox1	Cyp4a12a	Ywhah
Cd47	Cfl1	Ctsc	Adcy5	Cyp4a12b	
Cd48	Cfl2	Ctsd	Ptrh2	Cyp4a14	
Cd59	Chchd3	Ctsl	Dhrs4	Cyp4f13	
Cd81	Chchd6	Cul4a	Mp68	Cyp4f14	
Cd9	Chid1	Cul5	Pfn1	Cyp4f15	
Cd99	Chmp1a	Cyb5a	Mgat1	Cyp4f3	
Cdc37	Chmp4bl1	Cyb5b	Dio1	Cyp4v2	
Cdc42	Chp1	Cyb5r1	Snap23	Cyp51a1	
Cdc42bpa	Cisd1	Cyb5r3	Tmed4	Cyp7a1	
Cdc42bpb	Cisd2	Cyc1	Krt8	Cyp7b1	
Cdh13	Cisd3	Cycs	Marc1	Cyp8b1	
Cdh2	Ckap4	D2hgdh	Pdia6	Dap3	
Cdh5	Ckap5	Dad1	Stom	Dbt	
Cdnf	Ckb	Dag1	Cdh2	Dcakd	
Cds2	Ckm	Dap3	Tcp1	Ddost	
Cers2	Ckmt2	Dars1	Slc1a2	Ddt	
Ces1d	Clcn1	Dbi	Ufl1	Decr1	
Cfb	Clec10a	Dbt	Ppib	Decr2	
Cfh	Clic4	Dcn	Psmb1	Dgat1	
Cfl1	Clip1	Dctn1	Sult1a1	Dglucy	
Cfl2	Clpb	Dctn2	Zmpste24	Dhcr24	
Chchd3	Clpp	Ddah2	Tmprss6	Dhcr7	
Chchd6	Clpx	Ddost	Rps10	Dhrs1	
Chid1	Clta	Ddx1	Pzp	Dhrs4	
Chmp4b	Cltb	Ddx3x	Atl2	Dhrs7	
Chmp6	Cltc	Ddx5	Nudt12	Dhrs7b	
Chp1	Clybl	Decr1	Ddx17	Dhtkd1	
Chrm2	Cmc1	Deptor	Col4a2	Dipk2a	
Cisd1	Cmpk2	Des	Efr3a	Dlat	
Cisd2	Cndp2	Dglucy	Gapdh	Dld	
Ckap4	Cnp	Dguok	Cyp2b19	Dlst	
Ckb	Cnst	Dhodh	Emc8	Dmgdh	
Ckm	Coa3	Dhrs4	Abca1	Dnaja3	
Ckmt2	Cobl	Dhrs7	Atad3	Dnajb11	
Clec10a	Col14a1	Dhrs7b	Bri3bp	Dnajc11	
Clec2d11	Col1a1	Dlat	Gcn1	Dnajc3	
Clic1	Col1a2	Dld	Ilvbl	Dnm2	
Clic4	Col6a1	Dlg1	Lbr	Dpp4	
Clic5	Col6a2	Dlst	Cd82	Dsp	
Clpb	Colec12	Dmac2l	Ptprf	Ech1	
Clpp	Comt	Dmtn	Abi1	Echdc2	
Clpx	Comtd1	Dnaja2	Preb	Echdc3	

Cltb	Copa	Dnaja3	Arrb2	Echs1
Cltc	Copg1	Dnaja4	Ddx5	Eci1
Clu	Cops3	Dnajb11	Igf1r	Eci2
Cluh	Cops8	Dnajb4	Serpina1b	Eef1a1
Clybl	Coq10a	Dnajc11	Abcb6	Eef2
Cmbl	Coq3	Dnajc13	Tomm40	Egfr
Cmc1	Coq5	Dnajc16	Bdh1	Ehd1
Cndp2	Coq6	Dnajc19	Srprb	Ehd3
Cnn3	Coq7	Dnajc28	Mrc1	Ehhadh
Cnp	Coq8a	Dnajc3	Tuba1b	Emc1
Cnpy2	Coq9	Dnajc30	Serping1	Emc3
Coa3	Coro1c	Dnm1l	Nin	Emc8
Coa5	Cox11	Dnm2	Aass	Endog
Coa6	Cox14	Dnpep	Esd	Eno1
Coa7	Cox16	Dpp4	Tmem30a	Enpep
Coa8	Cox18	Dpp7	Rpl37a	Enpp1
Cobl	Cox19	Dpysl2	Sbds	Entpd5
Col14a1	Cox4i1	Dstn	Mbl2	Epb41l5
Col24a1	Cox5a	Dusp3	Slc39a14	Ephx1
Col4a2	Cox5b	Ech1	Crot	Ephx2
Col6a1	Cox6b1	Echdc2	Fgb	Erap1
Col6a2	Cox6c2	Echs1	Fam20a	Ergic1
Col6a6	Cox7a2	Eci1	Coro1b	Erlin1
Colec12	Cox7a2l	Eci2	Enpp3	Erlin2
Colgalt1	Cox7b	Ecsit	Stim1	Ermp1
Comt	Cox7c	Eea1	Ndufa9	Ero1a
Copa	Cp	Eef1a1	Slco2a1	Ero1b
Copb1	Cpox	Eef1a2	Pon3	Erp29
Copb2	Cpq	Eef1d	Mat1a	Erp44
Copg1	Cpt1b	Eef1g	Gng12	Etfa
Cops2	Cpt2	Eef2	Hsd17b7	Etfb
Cops4	Crat	Eepd1	Tap2	Etfdh
Cops7a	Creld1	Ehbp1l1	Ctsd	Ethe1
Cops8	Cryab	Ehd1	Eef1b	Ezr
Coq10a	Cs	Ehd2	Slc29a1	F2
Coq3	Cse1l	Ehd4	Flot2	Faah
Coq4	Csnk1a1	Ehhadh	Mup3	Fabp1
Coq5	Csnk2a1	Eif2a	Cct3	Fads1
Coq6	Csrp3	Eif2s1	Rpl9	Fads2
Coq7	Cst3	Eif2s3	Uqcrc2	Fah
Coq8a	Ctnna1	Eif3a	Prkcsh	Fahd1
Coq9	Ctnnb1	Eif3j	Ank3	Fahd2
Coro1a	Ctnnd1	Eif4a1	Dnajb12	Fam162a
Coro1b	Ctsa	Eif4g1	Serpina3m	Fam210b
Coro1c	Ctsb	Eif5a	Arl1	Farp2
Coro6	Ctsc	Emb	Adtrp	Farsa
Cox15	Ctsd	Emc1	Cyp2c70	Farsb
Cox16	Ctsl	Emc2	Uqcrq	Fbp1
Cox18	Cul1	Emc3	Krt10	Fdxr
Cox20	Cul4a	Emc4	Napa	Fech
Cox4i1	Cul5	Emc7	Hcfc1r1	Fermt2
Cox5a	Cyb5a	Emc8	Tmbim6	Fga
Cox5b	Cyb5b	Eml1	Rpl18a	Fgb
Cox6a2	Cyb5r1	Endog	Iqgap2	Fgg
Cox6b1	Cyb5r3	Eno1	Rab18	Fh
Cox6c2	Cyc1	Eno3	Lap3	Fkbp11
Cox7a2	Cycs	Enpp3	Hsd3b7	Fkbp2

Cox7a2l	D2hgdh	Entpd2	Slc25a42	Flna
Cox7c	Dad1	Epb41	Cyp20a1	Flnb
Cp	Dag1	Epb41l2	Pxmp2	Flot1
Cpd	Daglb	Epb42	Lmf2	Flot2
Cpne3	Dap3	Ephx1	Dmd	Fmo1
Cpox	Dars1	Eprs	Ehhadh	Fmo5
Cpq	Dbi	Erap1	Hspe1	Fn1
Cpt1a	Dbt	Ergic1	Cobll1	Fndc3a
Cpt1b	Dcn	Erlin2	Rdh7	Ftcd
Cpt2	Dctn1	Erp29	Acox1	Ftl1
Cr1l	Dctn2	Erp44	Ambp	G6pc1
Crat	Ddah1	Esd	Lin7a	Gaa
Creld1	Ddah2	Esyt2	Por	Galnt2
Crip1	Ddost	Etf1	Agt	Ganab
Cryab	Ddx1	Etfa	Ero1b	Gapdh
Cryz	Ddx3x	Etfb	Ctnnd1	Gatd3a
Cs	Ddx5	Etfdh	Ndst1	Gatm
Csnk1a1	Decr1	Ethe1	Clptm1l	Gba
Csnk1g2	Deptor	Exog	Dld	Gc
Csnk2a1	Des	F13a1	Tmem19	Gcat
Cspg4	Dglucy	Fabp3	Ptgs1	Gcdh
Csrp1	Dguok	Fabp4	C1sa	Gdi2
Csrp3	Dhodh	Fahd1	Srp54	Gfm1
Cst3	Dhrs4	Fahd2	Uqcrfs1	Ggcx
Cstb	Dhrs7	Fam162a	Cxadr	Gldc
Ctdsp1	Dhrs7b	Fam210a	Tgm2	Glg1
Ctnnb1	Dhrs7c	Fam98a	Ctnna1	Glo1
Ctnnd1	Diaph1	Farsa	Tbc1d10a	Glod4
Ctsa	Dlat	Farsb	Aldh9a1	Glrx5
Ctsb	Dld	Fbn1	Pccb	Gls2
Ctsc	Dlg1	Fcer1g	Pxmp4	Gltpd2
Ctsd	Dlst	Fdxr	Os9	Glud1
Ctsl	Dmac2	Fech	Lman1	Glul
Cul4a	Dmac2l	Fermt2	Trim25	Glyat
Cul5	Dmd	Fga	Hmgn2	Glyctk
Cxadr	Dmtn	Fgb	Rack1	Gm4952
Cyb5a	Dnaja1	Fgg	Idh2	Gna11
Cyb5b	Dnaja2	Fh	Eif4a1	Gna13
Cyb5r1	Dnaja3	Fhl1	Dnajc5	Gnai2
Cyb5r3	Dnaja4	Fis1	Sptbn1	Gnaq
Cyc1	Dnajb11	Fitm1	Tapbp	Gnas
Cycs	Dnajb12	Fkbp11	Dnajc11	Gnb1
Cyfip1	Dnajb4	Fkbp1a	Mup20	Gnb2
Cyp27a1	Dnajc11	Fkbp3	Cdipt	Gnmt
Cyp4b1	Dnajc13	Fkbp8	Mup6	Gnpat
D2hgdh	Dnajc16	Flad1	Hp1bp3	Golim4
Daam1	Dnajc19	Flna	Ndufb4	Got2
Dab2	Dnajc28	Flnb	Abhd15	Gpam
Dad1	Dnajc3	Flnc	Serpinh1	Gpd1
Dag1	Dnajc30	Flot1	Sec61a1	Gpd2
Dap3	Dnase1l1	Flot2	Slco1a4	Gpt
Dars1	Dnm1l	Fn1	Kdsr	Gpt2
Dbi	Dnm2	Foxred1	Slc27a4	Gpx1
Dbn1	Dnpep	Fscn1	Bphl	Grhpr
Dbt	Dpp4	Fundc2	Man1b1	Grn
Dcakd	Dpp7	Fxn	Atp5h	Grpel1
Dcn	Dpysl2	Fxr1	Asl	Gsr

Dctn1	Dstn	Fyco1	Dpys	Gstk1
Dctn2	Dtna	Gadd45gip1	Adam10	Gstm1
Dctn4	Dusp29	Galnt2	Met	Gstp1
Dcxr	Dusp3	Ganab	Hbb-b1	Gstz1
Ddah2	Dync1h1	Gapdh	Mtco2	Gulo
Ddb1	Dync1i2	Gba	Apom	H2-D1
Ddost	Dynll2	Gcdh	Crp	H2-K1
Ddx1	Dysf	Gcsh	Phlda1	H2-Q10
Ddx3x	Ech1	Gda	Hist1h2bm	H6pd
Ddx5	Echdc2	Gdi1	Cdc42	Hacd3
Decr1	Echdc3	Gdi2	Fga	Hacl1
Deptor	Echs1	Get3	Slc25a1	Hadh
Des	Eci1	Gfm1	Phyh	Hadha
Dgat1	Eci2	Gfm2	Kng1	Hadhb
Dglucy	Ecpas	Ggt5	Ech1	Hagh
Dguok	Ecsit	Ghitm	Fmo5	Hao1
Dhodh	Edf1	Glg1	Atp6v0d1	Hdhd3
Dhrs4	Eea1	Glipr2	Ywhae	Hdlbp
Dhrs7	Eef1a1	Glo1	Tmem205	Hexb
Dhrs7b	Eef1a2	Glrx5	Rpl34	Hgd
Diablo	Eef1b2	Glud1	Flna	Hgsnat
Dlat	Eef1d	Gna13	Osbpl6	Hibadh
Dld	Eef1g	Gnai2	Yipf3	Hibch
Dlg1	Eef2	Gnai3	Rpl37	Hint2
Dlst	Eepd1	Gnaq	Rsu1	Hmgcl
Dmac2l	Ehbp1	Gnas	Aspdh	Hmgcs2
Dmtn	Ehbp1l1	Gnb1	Cyp39a1	Hoga1
Dnaja2	Ehd1	Gnb2	Fads6	Hp
Dnaja3	Ehd2	Gng12	Eci1	Hpd
Dnaja4	Ehd4	Gng2	Slc2a1	Hpn
Dnajb11	Ehhadh	Gng5	Csrp3	Hpx
Dnajb4	Eif2a	Gnpat	Jup	Hrg
Dnajb6	Eif2s1	Golga4	Masp2	Hsd11b1
Dnajc10	Eif2s2	Golga5	Dnm2	Hsd17b10
Dnajc11	Eif2s3	Golim4	Itgb3	Hsd17b11
Dnajc13	Eif3a	Got1	Pex6	Hsd17b12
Dnajc15	Eif3f	Got2	Rps20	Hsd17b13
Dnajc16	Eif3j	Gpam	Ugt2b17	Hsd17b2
Dnajc19	Eif4a1	Gpc1	Cyp2d11	Hsd17b4
Dnajc28	Eif4b	Gpd1	Actn1	Hsd17b6
Dnajc3	Eif4g1	Gpd1l	Itga1	Hsd17b7
Dnajc30	Eif5a	Gpd2	Slc9a3r2	Hsd17b8
Dnajc5	Eloc	Gpi	Cox15	Hsd3b2
Dnm1l	Emb	Gpx1	Sar1b	Hsd3b3
Dnm2	Emc1	Gpx4	Lrp1	Hsd3b4
Dnpep	Emc10	Grb10	Steap3	Hsd3b5
Dock1	Emc2	Grpel1	Sntb1	Hsd3b7
Dock9	Emc3	Grsf1	Ilk	Hsdl2
Dpep1	Emc4	Gstk1	Ugt1a6	Hsp90aa1
Dpp4	Emc7	Gstm2	Tln2	Hsp90ab1
Dpp7	Emc8	Gstp1	Cox5b	Hsp90b1
Dpysl2	Eml1	Gstz1	Tubb1	Hspa2
Dpysl3	Endog	Guf1	Tyw1	Hspa5
Dsg2	Eno1	Gyg1	Acp6	Hspa8
Dsp	Eno3	Gys1	Fitm2	Hspa9
Dst	Enpp3	H4c2	Eci2	Hspd1
Dstn	Enpp4	Hacd1	Kmo	Hspe1

Dtnb	Entpd2	Hacd3	Capza2	Htatip2
Dusp3	Epb41	Hadh	Hsp90b1	Hyou1
Dut	Epb41l2	Hadha	Glul	Iars2
Dync1li1	Epb42	Hadhb	Slc10a1	Ica
Dync1li2	Ephx1	Hars2	Tor1aip1	Icam1
Ece1	Eprs	Hba1	Shmt1	Ide
Ech1	Erap1	Hbs1l	Rps28	Idh1
Echdc2	Ergic1	Hccs	Rpl27	Idh2
Echs1	Erlec1	Hdhd5	Itpr2	Idh3a
Eci1	Erlin1	Hexb	Atp2b1	Idh3b
Eci2	Erlin2	Hhatl	Cmbl	Idh3g
Ecsit	Erp29	Hibadh	Uqcrc1	Ighg2c
Eea1	Erp44	Hibch	Kcnj14	Ighm
Eef1a1	Esd	Hint2	Aldh1a1	Igtp
Eef1a2	Esyt1	Hint3	Actr2	Ilvbl
Eef1d	Esyt2	Hk1	Sept9	Immt
Eef1g	Etf1	Hk2	Cyp2c37	Iqgap1
Eef2	Etfa	Hmgb1	Hpn	Iqgap2
Eepd1	Etfb	Hmgcl	Man2a1	Irgm1
Efhd2	Etfdh	Hnrnpk	Tmem168	Isoc2a
Efnb3	Ethe1	Hp	Sod2	Itgb1
Efr3a	Exoc4	Hpx	Serpinf1	Itih2
Ehbp1l1	Exog	Hrc	Plxnb2	Itih4
Ehd1	F13a1	Hrg	Fmo4	Itpr1
Ehd2	Fabp3	Hsd17b10	Cyfip1	Ivd
Ehd3	Fabp4	Hsd17b4	Stxbp3	Iyd
Ehd4	Fahd1	Hsd17b8	Pdlim5	Jup
Ehhadh	Fahd2	Hsdl1	Fn1	Keg1
Eif1b	Fam162a	Hsdl2	Nsf	Kif13b
Eif2a	Fam210a	Hsp90aa1	Stx12	Kmo
Eif2s1	Fam234a	Hsp90ab1	Vtn	Kng1
Eif2s3	Fam98a	Hsp90b1	Tram1	L2hgdh
Eif3a	Farsa	Hspa12b	Rab1b	Lactb
Eif3b	Farsb	Hspa1b	Tgm1	Lactb2
Eif3j	Fbn1	Hspa4	Vwa8	Lamp2
Eif4a1	Fbp2	Hspa5	Tmem38b	Lap3
Eif4e	Fbxo6	Hspa8	Rpl29	Lcp1
Eif4g1	Fcer1g	Hspa9	Abhd3	Ldha
Eif4g2	Fdxr	Hspb1	Cask	Ldhd
Eif5a	Fech	Hspb6	Scrib	Letm1
Elmo1	Fermt2	Hspb7	Gpatch8	Lgals9
Elob	Fga	Hspd1	Man1a1	Lipa
Emb	Fgb	Hspe1	Vimp	Lman1
Emc1	Fgg	Hyou1	Creld1	Lman2
Emc2	Fh	Iars2	Acsl4	Lmf1
Emc3	Fhl1	Iba57	Itga5	Lmf2
Emc4	Fhl3	Ide	Fis1	Lonp1
Emc7	Fis1	Idh2	Cml1	Lonp2
Emc8	Fitm1	Idh3a	C8g	Lpcat3
Emilin1	Fkbp11	Idh3B	Sec61g	Lpgat1
Eml1	Fkbp1a	Igf2r	Rpl17	Lrp1
Endog	Fkbp3	Igg-2a	Khk	Lrpap1
Eng	Fkbp8	Igh-1a	Golim4	Lrpprc
Eno1	Flad1	Ighm	Afdn	Lrrc59
Eno3	Flna	Igtp	Ptprd	Lsr
Enpep	Flnb	Ilk	Txn	Lss
Enpp1	Flnc	Ilvbl	H2afx	Lypla1

Enpp3	Flot1	Immt	Hist3h2a	M6pr
Entpd1	Flot2	Ipo5	Cox4i1	Magt1
Entpd2	Fmod	Iqgap1	Lgals8	Man1a1
Entpd5	Fn1	Irgm	Dnajb11	Man1a2
Epb41	Foxred1	Isca1	Hip1r	Man2a1
Epb41l2	Fscn1	Isca2	Scd1	Manf
Epb42	Fundc2	Iscu	Hibch	Maob
Epdr1	Fxn	Ist1	Arpc5	Marchf5
Ephb4	Fxr1	Itga2b	Cisd2	Mat1a
Ephx1	Fyco1	Itga6	Ugt1a9	Mbl2
Ephx2	Gadd45gip1	Itga7	Uqcrh	Mboat7
Eprs	Galns	Itgav	Cyp2d9	Mccc1
Eps15	Galnt2	Itgb1	Arpc1a	Mccc2
Erap1	Ganab	Itgb3	Fech	Mcfd2
Ergic1	Gapdh	Itih4	Cyb5b	Mdh1
Erlin2	Gatc	Ivd	Epb42	Mdh2
Ermp1	Gba	Jph2	Tm9sf2	Me1
Ero1a	Gcdh	Kars1	Pdcd11	Me2
Erp29	Gcsh	Kif5b	Iars2	Mecr
Erp44	Gda	Klhl41	Decr2	Mettl7a3
Esd	Gde1	Kpnb1	Rpl3	Mettl7b
Esyt2	Gdi1	Ktn1	Ndufa3	Mfn1
Etf1	Gdi2	Kyat3	Rtcb	Mgam
Etfa	Gdpd1	L2hgdh	Fbp1	Mgat1
Etfb	Get3	Lactb	Mup5	Mgat2
Etfdh	Gfm1	Lama2	Fam3a	Mgst1
Ethe1	Gfm2	Lamb2	Capn8	Mia2
Exoc5	Ggt5	Lamp1	Sil1	Mia3
Exoc7	Ghitm	Lamp2	Abcc6	Mlec
Exog	Glg1	Lamtor1	Hrg	Mlycd
Ezr	Glipr2	Lamtor2	Rps19	Mmut
F13a1	Glo1	Lamtor5	Cd302	Mogs
Fabp3	Glrx3	Lancl1	Rab5if	Mpc2
Fabp4	Glrx5	Lap3	Saysd1	Mpdu1
Fabp5	Gls	Lars2	Spcs2	Mpst
Fahd1	Glud1	Lclat1	Hadh	Mrc1
Fahd2	Glul	Lcp1	Gosr1	Mrpl37
Fam126a	Gna13	Ldb3	Ddrgk1	Mrpl39
Fam162a	Gnai2	Ldha	Tmx3	Mrps35
Fam173a	Gnai3	Ldhb	B4galt1	Mrps5
Fam20b	Gnaq	Ldhd	Col18a1	Mrps7
Fam210a	Gnas	Letm1	Ankrd11	Msn
Fam98a	Gnb1	Lgals1	Ppp2r1a	Msra
Farsa	Gnb2	Lgals3	Dpy19l1	Mtarc1
Farsb	Gng12	Lias	Slc25a11	Mtarc2
Fastkd2	Gng2	Lims1	Apoh	Mtch1
Fbn1	Gng5	Lman1	Sfxn2	Mtch2
Fcer1g	Gnpat	Lman2	Fetub	Mtco1
Fdxr	Golga4	Lmcd1	Cers2	Mtco2
Fech	Golga5	Lmna	Mtx1	Mtdh
Fermt2	Golim4	Lnpep	Cltc	Mthfd1
Fermt3	Gosr2	LOC100911130	Rp2	Mtnd1
Fga	Got1	LOC108348074	Ppm1l	Mtnd4
Fgb	Got2	LOC684270	Eef1g	Mtnd5
Fgf1	Gpam	Lonp1	Rpl38	Mttp
Fgg	Gpc1	Lpcat3	Vdac3	Mtx1
Fh	Gpd1	Lpl	Emc3	Mtx2

Fhit	Gpd1l	Lrp1	Edf1	Mug1
Fhl1	Gpd2	Lrpap1	P4hb	Mug2
Fhl2	Gpi	Lrrc57	Sqrdl	Mup1
Fis1	Gpt2	Lrrc59	Pigo	Mup17
Fitm1	Gpx1	Lum	Itih3	Mup20
Fitm2	Gpx4	Lxn	Rpl32	Mup3
Fkbp11	Gpx7	Lypla1	Hdlbp	Mydgf
Fkbp1a	Grb10	Lyrm4	Vps35	Myh10
Fkbp3	Grn	Lyrm9	Cyp2e1	Myh9
Fkbp4	Grpel1	Macrod1	Atp2a2	Myl6
Fkbp8	Grsf1	Magt1	Hsd17b10	Myo1a
Flad1	Gsr	Maip1	Rpl24	Myo1b
Flna	Gsta3	Manf	Actg1	Myo1c
Flnb	Gstk1	Maoa	Ndufb10	Nadk2
Flnc	Gstm2	Maob	Pon1	Nat8f2
Flot1	Gstp1	Map3k20	Fau	Naxe
Flot2	Gstz1	Marcks	Abhd12	Nceh1
Fmo1	Gtpbp3	Mavs	Eif5a	Nckap1
Fmo3	Gtpbp6	Mb	Prdx5	Ncln
Fn1	Guf1	Mcat	Lgals9	Ncstn
Foxred1	Gyg1	Mccc1	Cyp51a1	Ndrg2
Fscn1	Gypc	Mccc2	Pgk1	Ndufa10
Fundc1	Gys1	Mcee	Rusf1	Ndufa11
Fundc2	H1-0	Mcu	Glud1	Ndufa12
Fxn	H1-4	Mdh1	Hpx	Ndufa13
Fxr1	H1-5	Mdh2	Tex2	Ndufa4
Fyco1	H2ac18	Me2	Hsd17b6	Ndufa5
Gaa	H2az1	Me3	Tapbpl	Ndufa6
Gadd45gip1	H4c2	Mecr	Rps3a	Ndufa8
Galnt2	Hacd1	Mesd	Actr3	Ndufa9
Ganab	Hacd3	Mettl7a	Tmco1	Ndufb10
Gapdh	Hadh	Mfn1	Eng	Ndufb11
Gars1	Hadha	Mfn2	F9	Ndufb3
Gba	Hadhb	Mgll	Hsd17b2	Ndufb4
Gba2	Hars2	Mgst3	Rmdn3	Ndufb5
Gbe1	Hba1	Mia2	Rpl21	Ndufb6
Gbf1	Hbs1l	Mia3	Alg5	Ndufb7
Gcdh	Hccs	Micu1	Slco1a1	Ndufb8
Gcsh	Hdhd5	Minpp1	Cyp2f2	Ndufb9
Gda	Hdlbp	Mipep	Rps14	Ndufs1
Gdi1	Hectd1	Mlec	Surf4	Ndufs2
Gdi2	Hexa	Mlip	Rpl36	Ndufs3
Get3	Hexb	Mlycd	Arg1	Ndufs4
Gfm1	Hhatl	Mmaa	Pigk	Ndufs5
Gfm2	Hibadh	Mmab	Dag1	Ndufs6
Ggt5	Hibch	Mmut	Gprin3	Ndufs7
Ghitm	Higd1a	Mogs	Serbp1	Ndufs8
Gimap4	Higd2a	Mospd1	Col11a1	Ndufv1
Gja1	Hint2	Mpc1	Pipox	Ndufv2
Gk	Hint3	Mpc2	Rfwd3	Nfs1
Glg1	Hist1h1c	Mpst	Cep170b	Nipsnap1
Glipr2	Hist1h1d	Mpz	Dzip3	Nipsnap3b
Glo1	Hk1	Mrc1	Cds2	Nit2
Glrx5	Hk2	Mrpl10	St6gal1	Nlrx1
Glud1	Hmgb1	Mrpl11	Gpat4	Nme1
Gmps	Hmgcl	Mrpl12	Gar1	Nnt
Gna11	Hmox2	Mrpl13	Apoe	Nomo1

Gna12	Hnrnpk	Mrpl14	Ube2n	Nsdhl
Gna13	Hp	Mrpl15	Rpl19	Nsf
Gnai2	Hpx	Mrpl16	Magt1	Nucb1
Gnai3	Hras	Mrpl19	Rpl8	Nudt12
Gnao1	Hrc	Mrpl2	Tubb2b	Nudt19
Gnaq	Hrg	Mrpl20	H2afv	Nudt7
Gnas	Hsd17b10	Mrpl21	Actg2	Oat
Gnb1	Hsd17b12	Mrpl22	Mpp6	Ociad2
Gnb2	Hsd17b4	Mrpl23	Pm20d1	Ogdh
Gnb3	Hsd17b8	Mrpl24	Polk	Opa1
Gng10	Hsdl1	Mrpl27	Cyp2u1	Os9
Gng12	Hsdl2	Mrpl28	Dhrs1	Otc
Gng2	Hsp90aa1	Mrpl3	Decr1	P4hb
Gng5	Hsp90ab1	Mrpl32	Baat	Pa2g4
Gnpat	Hsp90b1	Mrpl33	Ampd2	Pabpc1
Golga4	Hspa12b	Mrpl37	Epb41l5	Pacsin3
Golga5	Hspa1b	Mrpl38	Odr4	Pafah2
Golgb1	Hspa4	Mrpl39	Hamp	Paics
Golim4	Hspa5	Mrpl4	Tm9sf4	Pals2
Golm1	Hspa8	Mrpl40	Rpl13	Paqr9
Got1	Hspa9	Mrpl41	Dpm3	Parva
Got2	Hspb1	Mrpl43	Tjp1	Pc
Gpam	Hspb6	Mrpl44	Haus5	Pcca
Gpc1	Hspb7	Mrpl45	Sorbs1	Pccb
Gpc4	Hspd1	Mrpl46	S1pr1	Pck1
Gpd1	Hspe1	Mrpl47	Tfam	Pcyox1
Gpd1l	Hyou1	Mrpl48	Cyp2c29	Pdha1
Gpd2	Iars2	Mrpl49	Atp5j	Pdhb
Gpi	Iba57	Mrpl50	Pex11b	Pdia3
Gprin3	Ide	Mrpl51	Fgl1	Pdia4
Gpsm1	Idh2	Mrpl53	Eif2a	Pdia5
Gpx1	Idh3a	Mrpl55	Paqr9	Pdia6
Gpx4	Idh3B	Mrpl58	Serpina1d	Pdzk1
Grb10	Ifnl1	Mrpl9	Lasp1	Pecr
Grb2	Igf2r	Mrps11	Ehd3	Pex1
Grpel1	Igg-2a	Mrps15	Cisd3	Pex11a
Grsf1	Igh-1a	Mrps21	Ass1	Pex11b
Gsk3b	Ighm	Mrps22	Tst	Pex11g
Gsn	Igtp	Mrps23	Sptlc2	Pex12
Gstk1	Ikbip	Mrps25	Lss	Pex13
Gstm2	Ilk	Mrps27	Rab32	Pex14
Gstm7	Ilvbl	Mrps30	Rhot1	Pex26
Gstp1	Immt	Mrps34	Kiaa1211	Pex3
Gstz1	Ipo5	Mrps35	F2	Pex5
Guf1	Iqgap1	Mrps36	F5	Pex6
Gyg1	Irgm	Mrps6	H3f3a	Pgap1
Gys1	Isca1	Mrps7	Hist1h3b	Pgk1
H4c2	Isca2	Mrps9	Tmc8	Pgm1
Hacd1	Iscu	Mrs2	Abcc2	Pgrmc1
Hacd3	Isoc2	Msn	Cox7c	Phb
Hacl1	Ist1	Mtarc2	Impad1	Phb2
Hadh	Itga2b	Mt-atp8	Ttn	Phyh
Hadha	Itga6	Mtch2	Ephx2	Picalm
Hadhb	Itga7	Mtco2	Plg	Pigr
Hagh	Itgav	Mtdh	Gnaq	Pigs
Hars2	Itgb1	Mtfp1	Cp	Pipox
Hba1	Itgb2	Mthfd1	Igf2r	Pitrm1

Hbb	Itgb3	Mtif2	Tpm3	Plbd1
Hbs1l	Itih3	Mtnd1	Uggt1	Plg
Hccs	Itih4	Mtnd4	Agpat2	Plpbp
Hdhd5	Ivd	Mtnd5	Pex3	Pls3
Hebp1	Jph1	Mtx1	Slc22a18	Plxnb2
Hexb	Jph2	Mtx2	Bmt2	Pm20d1
Hhatl	Jsrp1	Mul1	Mtch2	Pmpca
Hibadh	Kars1	Mvp	Abcb7	Pmpcb
Hibch	Kcnj11	Myadm	Iigp1	Pnpla8
Hint1	Kcnma1	Myh11	Pigs	Pnpt1
Hint2	Kif5b	Myh14	Rreb1	Poldip2
Hint3	Klhl31	Myh7	Clcn4	Pon1
Hip1	Klhl40	Myl2	Ero1a	Pon2
Hk1	Klhl41	Myl3	Sec11a	Pon3
Hk2	Kpnb1	Myl6	Amacr	Por
Hmgb1	Ktn1	Mylk	Haus3	Ppa2
Hmgcl	Kyat3	Myo1b	Sept2	Ppib
Hnrnpk	L2hgdh	Myo1c	Letm1	Ppt1
Hnrnpu	Lactb	Myo1d	Mtnd4	Prdx1
Hp	Lactb2	Myof	Rps13	Prdx3
Hpcal1	Lama2	Myom1	Cct4	Prdx4
Hprt1	Lamb2	Myom2	Rhoa	Prdx5
Hpx	Lamp1	Naca	Ugt1a1	Prdx6
Hrc	Lamp2	Nadk2	Rpl15	Preb
Hrg	Lamtor1	Nampt	Dysf	Prkcsh
Hsd17b10	Lamtor2	Nap1l4	Fmnl3	Prodh
Hsd17b11	Lamtor5	Napa	Ndufv2	Prodh2
Hsd17b4	Lancl1	Napg	Slc25a15	Prxl2a
Hsd17b8	Lap3	Nars2	Tmem109	Psap
Hsdl1	Lars2	Naxd	Dsc2	Ptcd3
Hsdl2	Lasp1	Naxe	Gnas	Ptges2
Hsp90aa1	Lbr	Ncam1	Zg16	Ptgs1
Hsp90ab1	Lclat1	Nceh1	Snta1	Ptpmt1
Hsp90b1	Lcp1	Ncl	Atp6v1b2	Ptprd
Hspa12b	Ldb3	Ncln	Sep15	Ptprf
Hspa1b	Ldha	Ndrg2	Farp2	Ptrh2
Hspa4	Ldhb	Ndufa10	Itm2b	Pxmp2
Hspa5	Ldhd	Ndufa11	Adh1	Pxmp4
Hspa8	Lemd2	Ndufa12	Tpi1	Pzp
Hspa9	Letm1	Ndufa13	Rpl11	Qdpr
Hspb1	Lgals1	Ndufa2	Cox7a2	Rab10
Hspb2	Lgals3	Ndufa3	Opa1	Rab14
Hspb6	Lias	Ndufa4	Spryd7	Rab18
Hspb7	Lims1	Ndufa5	Abhd6	Rab1A
Hspd1	Lipe	Ndufa6	Sgpl1	Rab1b
Hspe1	Lman1	Ndufa7	Agpat3	Rab21
Hyou1	Lman2	Ndufa8	Man1a2	Rab2a
Iars	Lmcd1	Ndufa9	Rmdn2	Rab5b
Iars2	Lmna	Ndufab1	Csrp1	Rab7a
Iba57	Lmnb1	Ndufaf1	Aldh16a1	Rab8a
Icam1	Lmod3	Ndufaf2	Ces1c	Rac1
Icam2	Lnpep	Ndufaf3	Folr2	Rack1
Ide	Lnpk	Ndufaf4	Pon2	Rala
Idh1	LOC100359687	Ndufaf5	Anxa5	Rap1a
Idh2	LOC100910708	Ndufaf6	Alg12	Rbp4
Idh3a	LOC100911130	Ndufb1	Tmx1	Rdh11
Idh3B	LOC100911440	Ndufb10	Slc4a1	Rdh14

Idh3g	LOC100912599	Ndufb11	Acly	Rdh16
Ifi47	LOC108348074	Ndufb3	Fam134c	Rdh7
ifitm3	LOC683897	Ndufb4	Pex11a	Rdh9
Igf2r	LOC684270	Ndufb5	Sept1	Rdx
Igg-2a	LOC687508	Ndufb6	Ehd1	Reep6
Igh-1a	Lonp1	Ndufb7	Itih1	Rer1
Ighm	Lpcat3	Ndufb8	Rdh11	Retsat
Igtp	Lpl	Ndufb9	Cyp4v2	Rgn
Ilk	Lrig1	Ndufc2	Nsdhl	Rhot1
Ilvbl	Lrp1	Ndufs1	Etfa	Rida
Immp1l	Lrpap1	Ndufs2	Rhot2	Rpl10
Immt	Lrrc47	Ndufs3	Lrpap1	Rpl10a
Impa1	Lrrc57	Ndufs4	Bche	Rpl11
Impdh2	Lrrc59	Ndufs5	Ap2b1	Rpl12
Inpp5a	Lum	Ndufs7	Pnp	Rpl13
Ipo5	Lxn	Ndufs8	Anpep	Rpl13a
Iqgap1	Lypla1	Ndufv1	Syvn1	Rpl14
Iqgap2	Lyrm4	Ndufv2	Adk	Rpl15
Irgm	Lyrm9	Ndufv3	Apoa1	Rpl17
Isca1	Macrod1	Nedd4	Ttc38	Rpl18
Isca2	Magt1	Nfs1	Cfh	Rpl18a
Iscu	Maip1	Niban1	Ldha	Rpl19
Isoc1	Man2a2	Niban2	Acat2	Rpl21
Ist1	Manf	Nid1	Baiap2	Rpl22
Itga1	Maoa	Nipsnap2	Hadhb	Rpl23
Itga2b	Maob	Nit2	Eif3a	Rpl23a
Itga5	Map2k1	Nln	Urah	Rpl24
Itga6	Map2k2	Nlrx1	Picalm	Rpl26
Itga7	Map3k20	Nme2	Ap1b1	Rpl27
Itga9	Map4	Nmnat3	Mdh2	Rpl27a
Itgav	Map7d1	Nmt1	Derl2	Rpl3
Itgb1	Marcks	Nnt	Ostc	Rpl30
Itgb3	Marcksl1	Nomo1	Iyd	Rpl31
Itih1	Mavs	Nos3	Ldlr	Rpl32
Itih4	Mb	Npepps	Myo18a	Rpl36
Itm2b	Mboat7l1	Nsfl1c	Cfb	Rpl4
Ivd	Mbp	Nt5e	Abhd16a	Rpl5
Jak1	Mcat	Nucb1	Erich5	Rpl6
Jph2	Mccc1	Nudt19	Rpl12	Rpl7
Jup	Mccc2	Oat	Prdx1	Rpl7a
Kank2	Mcee	Obscn	Cdh1	Rpl8
Kars1	Mcu	Ociad1	Canx	Rpl9
Kif5b	Mdh1	Ogn	St3gal5	Rplp0
Klhl41	Mdh2	Opa1	Atp6v1a	Rplp1
Kpnb1	Me2	Osbp	Etfdh	Rplp2
Kras	Me3	Oxa1l	Hist1h1a	Rpn1
Ktn1	Mecr	Oxct1	Adh5	Rpn2
Kyat1	Mesd	Oxnad1	Lmf1	Rps12
Kyat3	Metap1	Oxsm	Tfr2	Rps13
L1cam	Mettl7a	P4hb	Ganab	Rps14
L2hgdh	Mfn1	Pa2g4	Tkfc	Rps15a
Lactb	Mfn2	Pacsin2	Serpina3k	Rps16
Lama2	Mgll	Pacsin3	Sntb2	Rps18
Lama4	Mgst3	Park7	Qdpr	Rps24
Lama5	Mia2	Parl	Nipsnap1	Rps27l
Lamb1	Mia3	Parp3	Acox3	Rps3
Lamb2	Micu1	Pbxip1	Dmgdh	Rps3a

Lamc1	Micu2	Pc	Pgrmc1	Rps4x
Lamp1	Micu3	Pcca	Lman2	Rps7
Lamp2	Minpp1	Pccb	Mug1	Rps8
Lamtor1	Mipep	Pcmt1	Arf1	Rps9
Lamtor2	Mlec	Pcyox1	Arf6	Rpsa
Lamtor3	Mlip	Pcyt1a	Rab13	Rrbp1
Lamtor5	Mlycd	Pdcd6ip	Scpep1	Rtn4ip1
Lancl1	Mmaa	Pde2a	Slc30a6	Sacm1l
Lap3	Mmab	Pdf	Etfb	Samm50
Lars2	Mme	Pdhb	Mmgt1	Sardh
Lbp	Mmut	Pdhx	Eef1a1	Scarb1
Lclat1	Mogs	Pdia3	Il1rap	Scarb2
Lcp1	Mospd1	Pdia4	Gstk1	Scp2
Ldb3	Mpc1	Pdia6	Rps3	Sdf2l1
Ldha	Mpc2	Pdk2	Ces2a	Sdha
Ldhb	Mpst	Pdk4	Agxt	Sdhb
Ldhd	Mpz	Pdlim5	Rps12	Sdhc
Letm1	Mrc1	Pdp1	Gnb1	Sec11a
Letmd1	Mreg	Pdpr	Abcg5	Sec14l2
Lgals1	Mrpl10	Pebp1	Fkbp8	Sec14l4
Lgals3	Mrpl11	Pecam1	Fgd6	Sec22b
Lias	Mrpl12	Pecr	Tor1aip2	Sec23a
Lima1	Mrpl13	Pex14	Ptges2	Sec24a
Lims1	Mrpl14	Pf4	Selenbp2	Sec61a1
Lin7c	Mrpl15	Pfkm	Azgp1	Sec62
Lman1	Mrpl16	Pgam2	Acaa1a	Sec63
Lman2	Mrpl19	Pgam5	Mgat2	Sel1l
Lmcd1	Mrpl2	Pgk1	Cnpy3	Selenbp1
Lmf1	Mrpl20	Pgm1	Cat	Septin2
Lmna	Mrpl21	Phb	Cyp1a2	Serpina1a
Lmo7	Mrpl22	Phb2	Fmo1	Serpina1b
Lnpep	Mrpl23	Phyh	Amy2	Serpina1e
LOC100911101	Mrpl24	Pi4k2a	Vdac2	Serpina3c
LOC100911104	Mrpl27	Picalm	Dsp	Serpina3k
LOC100911130	Mrpl28	Pigs	Gpat3	Serpina3n
LOC100911516	Mrpl3	Pisd	Ywhag	Serpinc1
LOC108348074	Mrpl32	Pitrm1	Esyt1	Serpinf2
LOC681355	Mrpl33	Pkm	Dlat	Sfxn1
LOC684270	Mrpl34	Plbd1	Hspa2	Sfxn2
Lonp1	Mrpl37	Plcb4	Fasn	Sfxn5
Lonp2	Mrpl38	Plg	Fermt2	Sgpl1
Lpcat3	Mrpl39	Plgrkt	Cyp4f3	Shmt2
Lpl	Mrpl4	Plin4	Tecr	Slc16a1
Lrp1	Mrpl40	Plpp7	Plbd1	Slc1a2
Lrpap1	Mrpl41	Pls3	Cfl1	Slc22a1
Lrrc4b	Mrpl43	Plxnb2	Dnaja1	Slc22a18
Lrrc57	Mrpl44	Pmpca	Gpr107	Slc25a1
Lrrc59	Mrpl45	Pmpcb	Ugp2	Slc25a10
Lrrc8a	Mrpl46	Pnkd	Akr1c6	Slc25a11
Lsamp	Mrpl47	Pnp	Fdft1	Slc25a12
Ltbp4	Mrpl48	Pnpla8	Dhrs7	Slc25a13
Luc7l2	Mrpl49	Pnpt1	Hao1	Slc25a15
Lum	Mrpl50	Poldip2	Chdh	Slc25a17
Lxn	Mrpl51	Pon2	ORF11	Slc25a20
Lyn	Mrpl53	Popdc2	Prdx2	Slc25a22
Lypla1	Mrpl55	Por	Dnajc1	Slc25a3
Lyrm1	Mrpl57	Ppa2	Sfxn1	Slc25a4

Lyrm4	Mrpl58	Ppia	Clec4f	Slc25a42
Lyrm7	Mrpl9	Ppib	Asgr1	Slc25a5
Lyrm9	Mrps11	Ppid	Cd47	Slc26a1
Macf1	Mrps15	Ppif	Epn1	Slc27a2
Macrod1	Mrps16	Ppt1	Apoa5	Slc27a5
Magmas-ps1	Mrps18b	Pptc7	Gnmt	Slc29a1
Magt1	Mrps21	Prdx1	Mcfd2	Slc2a2
Maip1	Mrps22	Prdx2	Pgrmc2	Slc2a9
Man1a1	Mrps23	Prdx3	Ptprc	Slc33a1
Man1c1	Mrps25	Prdx4	Gpx1	Slc35b1
Man2a1	Mrps26	Prdx5	Dnaja2	Slc35d1
Manf	Mrps27	Prdx6	Cmas	Slc37a4
Maoa	Mrps30	Preb	Jak1	Slc38a3
Maob	Mrps31	Prelp	Hsd3b3	Slc39a14
Map1	Mrps34	Prkaa2	Slc3a2	Slc4a1
Map3k20	Mrps35	Prkaca	Slc12a7	Slc6a13
Mapk1	Mrps36	Prkag1	Aup1	Slc9a3r1
Mapkapk3	Mrps6	Prkar1a	Sucla2	Slco1a1
Mapre1	Mrps7	Prkar2a	Clptm1	Slco1a4
Mapre2	Mrps9	Prkcsh	Selt	Slco1b2
Marcks	Mrs2	Prnp	Lrrc59	Slco2b1
Mavs	Msn	Prxl2a	Gnb2	Smpd2
Mb	Mtarc2	Psap	Rpl7a	Snd1
Mblac2	Mt-atp8	Psma1	Skt	Sntb1
Mcam	Mtch2	Psma2	Fam234a	Soat2
Mcat	Mtco2	Psma3	Cyp2a12	Sod1
Mccc1	Mt-Cyb	Psma4	Hyou1	Sod2
Mccc2	Mtdh	Psma5	Acsl1	Sord
Mcee	Mtfp1	Psma6	Myo6	Spcs2
Mcf2l	Mthfd1	Psma7	Rpl35	Sptan1
Mcu	Mtif2	Psmc1	Rpn2	Sptbn1
Mcur1	Mtif3	Psmc2	Fads2	Sptbn2
Mdh1	Mtnd1	Psmc3	Atp5j2	Sptlc2
Mdh2	Mtnd4	Psmc5	Sardh	Sqor
Me2	Mtnd5	Psmc6	Plod3	Srprb
Me3	Mtx1	Psmd1	Abcb11	Ssbp1
Mecr	Mtx2	Psmd11	Hspa9	Ssr1
Mesd	Mul1	Psmd13	Ywhab	Ssr4
Metap2	Mvp	Psmd2	Hsp90ab1	St3gal5
Mettl7a	Myadm	Psmd4	Lin7c	St6gal1
Mff	Mybpc1	Psme1	Acaa1b	Steap4
Mfge8	Mybpc2	Ptcd3	Sdhc	Stim1
Mfn1	Mybph	Ptges2	Tbl2	Stom
Mfn2	Myh1	Ptpmt1	Faah	Stoml2
Mgat1	Myh11	Ptprc	Rps21	Sts
Mgat2	Myh14	Ptrh2	Zc3h3	Stt3a
Mgll	Myh15	Pygb	Aldh4a1	Stt3b
Mgst3	Myh2	Pygm	Otc	Stxbp3
Mia2	Myh4	Qdpr	Slc25a3	Sucla2
Mia3	Myh7	Rab10	Armc1	Suclg1
Micu1	Myh9	Rab12	Rpl22l1	Suclg2
Minpp1	Myl1	Rab14	Tmem97	Sult1a1
Mipep	Myl12b	Rab18	Dnajc3	Sult2a8
Mlec	Myl2	Rab1A	Myh9	Suox
Mlip	Myl3	Rab1b	Pecr	Surf4
Mlycd	Myl6	Rab21	Tmem56	Tapbp
Mmaa	Mylk	Rab2a	Plscr1	Tbl2

Mmab	Mylk2	Rab35	Sigmar1	Tcirg1
Mmut	Mylpf	Rab3a	Hspd1	Tecr
Mogs	Myo15a	Rab5a	Rpl36a	Tf
Mospd1	Myo1b	Rab5b	Alb	Tgm1
Mpc1	Myo1c	Rab5c	Sec14l2	Tgm2
Mpc2	Myo1d	Rab7a	Slc38a10	Timm44
Mpdu1	Myof	Rab8a	Acad9	Timm50
Mpp6	Myom1	Rac1	Hip1	Tkfc
Mpp7	Myom2	Rack1	Rps8	Tkt
Mpst	Myot	Rala	Cyp4a10	Tln1
Mpv17	Myoz1	Ralb	Slc25a20	Tm7sf2
Mpz	Myoz3	Ran	Sdhb	Tm9sf1
Mrc1	Naca	Rap1a	Apmap	Tm9sf2
Mrc2	Nadk2	Rap1b	Scarb1	Tm9sf3
Mrpl1	Naga	Rars1	Osbpl8	Tm9sf4
Mrpl10	Nampt	Rcn1	Trex1	Tmbim6
Mrpl11	Nap1l4	Rdh13	Asah1	Tmco1
Mrpl12	Napa	Rdh14	Ces2c	Tmed10
Mrpl13	Napg	Rdx	Afg3l2	Tmed2
Mrpl14	Nars	Retsat	Aadac	Tmed4
Mrpl15	Nars2	RGD1565784	Cyp4b1	Tmed7
Mrpl16	Naxd	Rhoa	Slc25a10	Tmed9
Mrpl19	Naxe	Rhog	Entpd1	Tmem135
Mrpl2	Nbas	Rmdn1	Cenpj	Tmem14c
Mrpl20	Ncam1	Rnh1	Atp6v1f	Tmem205
Mrpl21	Nceh1	Rnpep	Sec23a	Tmem214
Mrpl22	Ncl	Rock1	Cyc1	Tmem256
Mrpl23	Ncln	Rock2	Dstn	Tmem30a
Mrpl24	Ncstn	Romo1	Coq8a	Tmem33
Mrpl27	Ndrg2	Rpl10	Cct6a	Tomm40
Mrpl28	Ndufa10	Rpl10a	B4galnt1	Tor1aip2
Mrpl3	Ndufa11	Rpl11	Alg6	Tpi1
Mrpl32	Ndufa12	Rpl12	C5	Tpp1
Mrpl33	Ndufa13	Rpl13	Gna13	Tram1
Mrpl35	Ndufa2	Rpl13a	Myl6	Trap1
Mrpl37	Ndufa3	Rpl14	Mtdh	Trim14
Mrpl38	Ndufa4	Rpl15	Nol8	Tsfm
Mrpl39	Ndufa5	Rpl17	Abcc3	Tst
Mrpl4	Ndufa6	Rpl18	Srpra	Tuba1a
Mrpl40	Ndufa7	Rpl18a	Chp1	Tubb2a
Mrpl41	Ndufa8	Rpl19	Atp5a1	Tubb4a
Mrpl42	Ndufa9	Rpl21	Erp44	Tufm
Mrpl43	Ndufab1	Rpl22	Samm50	Txndc5
Mrpl44	Ndufaf1	Rpl23	Rps7	Txnrd1
Mrpl45	Ndufaf2	Rpl23a	Arpc3	Txnrd2
Mrpl46	Ndufaf3	Rpl24	Pex13	Tysnd1
Mrpl47	Ndufaf4	Rpl26	Gpx4	Ufsp2
Mrpl48	Ndufaf5	Rpl27	Acaa2	Ugdh
Mrpl49	Ndufaf6	Rpl27a	Farsb	Uggt1
Mrpl50	Ndufaf7	Rpl28	Pbld1	Ugt1a1
Mrpl51	Ndufb1	Rpl3	Soat2	Ugt1a5
Mrpl53	Ndufb10	Rpl30	Gpd2	Ugt1a6
Mrpl55	Ndufb11	Rpl31	Tomm22	Ugt1a6b
Mrpl58	Ndufb3	Rpl32	Hexa	Ugt1a9
Mrpl9	Ndufb4	Rpl35a	Scp2	Ugt2a3
Mrps10	Ndufb5	Rpl36	Rps5	Ugt2b1
Mrps11	Ndufb6	Rpl37a-ps1	Fgf7	Ugt2b17

Mrps14	Ndufb7	Rpl38	Mettl7b	Ugt2b34
Mrps15	Ndufb8	Rpl3l	Tmem43	Ugt2b35
Mrps17	Ndufb9	Rpl4	Txndc12	Ugt2b36
Mrps2	Ndufc2	Rpl5	Shprh	Ugt2b5
Mrps21	Ndufs1	Rpl6	Mpp1	Ugt3a1
Mrps22	Ndufs2	Rpl7	Cisd1	Ugt3a2
Mrps23	Ndufs3	Rpl7a	Dync1h1	Uox
Mrps24	Ndufs4	Rpl8	Ddx1	Uqcrb
Mrps25	Ndufs5	Rpl9	Ociad1	Uqcrc1
Mrps27	Ndufs7	Rplp0	Rpl7	Uqcrc2
Mrps30	Ndufs8	Rplp1	H2afy	Uqcrfs1
Mrps34	Ndufv1	Rplp2	Erlin1	Urah
Mrps35	Ndufv2	Rpn1	Cyp4f14	Uroc1
Mrps36	Ndufv3	Rpn2	Cyp2c54	Utrn
Mrps5	Neb	Rps11	Hgd	Vapa
Mrps6	Nedd4	Rps12	Rpl22	Vcp
Mrps7	Nefl	Rps13	G3bp1	Vdac1
Mrps9	Nefm	Rps14	Prdx6	Vdac2
Mrrf	Nfs1	Rps16	Slc25a12	Vdac3
Mrs2	Niban1	Rps17	Prdx4	Vkorc1l1
Msn	Niban2	Rps18	Cox6b1	Vtn
Mtarc2	Nid1	Rps19	Acsl5	Vwa8
Mt-atp8	Nipsnap2	Rps2	Tmem214	Xdh
Mtch1	Nit2	Rps20	Lamp2	Yars2
Mtch2	Nln	Rps23	Aqp11	Ywhae
Mtco2	Nlrx1	Rps24	Vapa	Ywhaz
Mtdh	Nme2	Rps27a	Rps18	Zadh2
Mtfp1	Nmnat3	Rps3	Ndufc2	Zmpste24
Mtfr1l	Nmt1	Rps3a	Pgm1	
Mthfd1	Nnt	Rps4x	Ece1	
Mthfd1l	Nol3	Rps5	C8a	
Mtif2	Nomo1	Rps6	Dcakd	
Mtnd1	Nos3	Rps8	Phb2	
Mtnd4	Npc1	Rps9	Drg1	
Mtnd5	Npepps	Rpsa	Maoa	
Mtor	Nptn	Rras	Ap2a2	
Mtpap	Nqo1	Rras2	Rdh10	
Mtus1	Nsfl1c	Rrbp1	Stoml2	
Mtx1	Nt5c1a	Rtcb	Ephx1	
Mtx2	Nt5dc3	Rtn2	Ptdss1	
Mug1	Nt5e	Rtn4	Ndufs3	
Mul1	Nubpl	Rtn4ip1	Ndufa4	
Mvp	Nucb1	Rtraf	Btd	
Myadm	Nudc	S100a10	Rtn4	
Mybpc3	Nudt19	Sacm1l	Rac1	
Mydgf	Oat	Samm50	Acat1	
Myh10	Obscn	Sar1b	Slc31a1	
Myh11	Ociad1	Sars2	H2-K1	
Myh14	Odr4	Sbds	Atp5o	
Myh6	Ogdh	Scamp1	Gsta3	
Myh7	Ogn	Scarb2	Ahcy	
Myl2	Oma1	Sccpdh	Dnajc10	
Myl3	Opa1	Scfd1	Sec63	
Myl4	Osbp	Sco1	Sdhd	
Myl6	Oxa1l	Scp2	Ggcx	
Myl7	Oxct1	Scpep1	Chchd3	
Mylk	Oxnad1	Sdha	Tysnd1	

Mylk3	Oxsm	Sdhaf2	Lpcat3
Myo18a	P4ha1	Sdhb	Cpt1b
Myo1b	P4hb	Sdhc	Slc16a7
Myo1c	Pa2g4	Sdhd	Cyp7b1
Myo1d	Pabpc1	Sdr39u1	Tgoln2
Myo6	Pacsin2	Sec22b	St3gal1
Myof	Pacsin3	Sec23a	Rpl4
Myom1	Pam16	Sec61a2	Timm50
Myom2	Park7	Sec63	Rps15
Myoz2	Parl	Selenbp1	Immt
Myzap	Parp14	Septin11	Myh11
Naca	Parp3	Septin2	Ywhaq
Nadk2	Pbxip1	Septin7	Atp5e
Nampt	Pc	Serbp1	Slco2b1
Nap1l1	Pcbp2	Serpina1	Fads1
Nap1l4	Pcca	Serpinc1	H6pd
Napa	Pccb	Serpinf1	B2m
Napg	Pcmt1	Serpinh1	Rpl10
Nars2	Pcyox1	Sfxn3	Ywhaz
Naxd	Pcyt1a	Sgca	Hmgcl
Naxe	Pdcd6ip	Sgcb	Atp5c1
Ncam1	Pde2a	Sgcd	Cept1
Nceh1	Pdf	Sgcg	Cyp4a14
Nckap1	Pdhb	Sh3glb1	Emc1
Ncl	Pdhx	Shmt2	Mug2
Ncln	Pdia3	Siglec1	Cyp2c50
Ndrg1	Pdia4	Sirt2	Ermp1
Ndrg2	Pdia6	Sirt3	Tdo2
Ndufa10	Pdk1	Skp1	Rab2a
Ndufa11	Pdk2	Slc16a1	Tmem135
Ndufa12	Pdk4	Slc25a1	Rab10
Ndufa13	Pdlim1	Slc25a10	Atp11c
Ndufa2	Pdlim3	Slc25a11	Dpp4
Ndufa3	Pdlim5	Slc25a12	Prodh
Ndufa4	Pdlim7	Slc25a13	Abcd1
Ndufa5	Pdp1	Slc25a19	Mapkapk2
Ndufa6	Pdpr	Slc25a24	Tubb4b
Ndufa7	Pebp1	Slc25a29	Susd2
Ndufa8	Pecam1	Slc25a3	Hmox2
Ndufa9	Pecr	Slc25a35	Ppia
Ndufab1	Pet100	Slc25a4	Atp5f1
Ndufaf1	Pex12	Slc25a42	Anxa11
Ndufaf2	Pex14	Slc25a46	Cps1
Ndufaf3	Pf4	Slc25a5	Fah
Ndufaf4	Pfkm	Slc25a51	Marc2
Ndufaf5	Pgam2	Slc27a1	Lyn
Ndufaf6	Pgam5	Slc2a4	P3h3
Ndufb1	Pgk1	Slc30a9	Tns2
Ndufb10	Pgm1	Slc3a2	Fkbp11
Ndufb11	Pgrmc2	Slc43a1	Nod2
Ndufb2	Phb	Slc44a2	Zfp62
Ndufb3	Phb2	Slirp	Atp5l
Ndufb4	Phka1	Slmap	Arf5
Ndufb5	Phkb	Smpdl3b	Uqcr10
Ndufb6	Phkg1	Smyd1	Rpl5
Ndufb7	Phyh	Snap23	Ces1e
Ndufb8	Pi4k2a	Snd1	Dgat2

Ndufb9	Picalm	Snta1	Ndufs4
Ndufc2	Pigs	Sntb1	Epb41
Ndufs1	Pigt	Snx2	Pa2g4
Ndufs2	Pip4k2b	Snx5	Tmem120a
Ndufs3	Pisd	Sod1	Pc
Ndufs4	Pitpna	Sod2	Fabp1
Ndufs5	Pitrm1	Spcs3	Flot1
Ndufs6	Pkm	Speg	Ak2
Ndufs7	Pla2g6	Spg7	Efcab6
Ndufs8	Plbd1	Spryd4	Myo1b
Ndufv1	Plcb4	Sptan1	Uroc1
Ndufv2	Plec	Sqor	Papss2
Ndufv3	Plg	Srl	Pck1
Nebl	Plgrkt	Ssbp1	Uqcrb
Nedd4	Plin4	Ssr1	Aqp1
Nenf	Plp1	Ssr4	Cap1
Neu3	Plp2	St13	Rpl14
Nfs1	Plpbp	Steap3	Exoc1
Nfu1	Plpp3	Steap4	Bmper
Niban1	Plpp7	Stim1	Ap2a1
Niban2	Pls3	Stip1	Clu
Nid1	Plxnb2	Stom	Vkorc1l1
Nid2	Pm20d2	Stoml2	Cyb5a
Nipsnap2	Pmp2	Stt3a	Naxd
Nit2	Pmpca	Stt3b	Atp5b
Nln	Pmpcb	Stx12	Slc25a22
Nlrx1	Pnkd	Stx4	Rps2
Nme2	Pnp	Stx7	Ca3
Nmes1	Pnpla8	Stxbp1	Coa3
Nmnat3	Pnpt1	Stxbp3	Vim
Nmt1	Poldip2	Sucla2	Anxa2
Nnt	Pomgnt2	Suclg1	Tapt1
Nomo1	Pon2	Suclg2	Aldh7a1
Nos3	Pon3	Supv3l1	Cct2
Npepps	Popdc2	Surf1	Eef2
Nppa	Por	Susd2	Rarres2
Nrp1	Ppa2	Syncrip	Ezr
Nsf	Ppbp	Synj2bp	Oat
Nsfl1c	Ppia	Sypl1	Alg10b
Nt5c3a	Ppib	Taco1	Armc10
Nt5e	Ppid	Tagln	Krt84
Ntn1	Ppif	Tagln2	Cox6c
Ntpcr	Ppox	Tap1	Mbl1
Nucb1	Ppp1r3a	Tapbp	Gstm1
Nucb2	Ppp2r1a	Tapt1	Rplp2
Nudt19	Ppp3ca	Tars2	Pacsin2
Nudt8	Ppt1	Tbrg4	Htatip2
Nudt9	Pptc7	Tcp1	Atl3
Numbl	Prdx1	Tecr	Rtn3
Oat	Prdx2	Tefm	Gpd1
Obscn	Prdx3	Tfam	Hadha
Ociad1	Prdx4	Tfrc	Ndufb5
Ogn	Prdx5	Tgfbi	Morc4
Ola1	Prdx6	Tgm2	Hrsp12
Opa1	Preb	Timm10	St3gal4
Osbp	Prelp	Timm13	Ugt1a2
Otub1	Prkaa2	Timm21	Idh1

Oxa1l	Prkaca	Timm23	Ttn
Oxct1	Prkag1	Timm29	Rps9
Oxnad1	Prkar1a	Timm44	Fh
Oxsm	Prkar2a	Timm50	Dhrs7b
Oxsr1	Prkcq	Timm9	Hsd17b4
P4ha2	Prkcsh	Timmdc1	Rap1b
P4hb	Prnp	Tln1	Jagn1
Pa2g4	Prx	Tln2	Aldh1a7
Pabpc4	Prxl2a	Tm9sf2	Fam213a
Pacsin2	Prxl2b	Tm9sf3	Fkbp2
Pacsin3	Psap	Tm9sf4	Ugt2a3
Pafah1b1	Psma1	Tmed1	Ighm
Pak2	Psma2	Tmed10	Cyp2d10
Pald1	Psma3	Tmed2	Gcdh
Palm	Psma4	Tmed4	Hsd17b12
Pam	Psma5	Tmed7	Itgav
Park7	Psma6	Tmed9	Rnf123
Parl	Psma7	Tmem109	Lclat1
Parp3	Psmb7	Tmem11	Acsm1
Pars2	Psmc1	Tmem126a	Entpd5
Parva	Psmc2	Tmem143	Cyp2c38
Parvb	Psmc3	Tmem186	Npm1
Pbxip1	Psmc5	Tmem33	Aifm1
Pc	Psmc6	Tmem38a	Cpox
Pcca	Psmd1	Tmem43	Nras
Pccb	Psmd11	Tmem65	Sdr42e1
Pck2	Psmd13	Tmem70	Msn
Pcmt1	Psmd14	Tmlhe	Pla2g12b
Pcyox1	Psmd2	Tmod1	Pcnt
Pcyox1l	Psmd4	Tmsb4x	Rps15a
Pcyt1a	Psmd5	Tmx2	Tubb5
Pdcd6ip	Psme1	Tmx3	Pex14
Pde2a	Ptcd3	Tnnc1	Tsku
Pdf	Ptges2	Tomm20	Ssr1
Pdgfrb	Ptma	Tomm22	Vat1
Pdha1	Ptp4a2	Tomm34	Cyp2a5
Pdhb	Ptpmt1	Tomm40	Comt
Pdhx	Ptprc	Tomm40l	Syne1
Pdia3	Ptrh2	Tomm6	Cyp8b1
Pdia4	Pura	Tomm70	Hltf
Pdia6	Pvalb	Tpd52l2	Mfn1
Pdk2	Pycr3	Tpi1	Hpd
Pdk4	Pygb	Tpm1	Ctnnb1
Pdlim5	Pygm	Tpm3	Rras2
Pdp1	Qdpr	Tpm4	Rps24
Pdpr	Rab10	Tpp1	Slc6a12
Pebp1	Rab11a	Tppp3	Arl6ip1
Pecam1	Rab12	Tpt1	Plscr2
Pecr	Rab14	Trabd	Dbi
Perm1	Rab18	Trap1	Rpl28
Pex14	Rab1A	Trim72	Sept7
Pex3	Rab1b	Tsfm	Rpl23a
Pf4	Rab21	Tspan8	Hsd17b13
Pfdn6	Rab22a	Tst	Nudt7
Pfkl	Rab2a	Tuba4a	Cct7
Pfkm	Rab35	Tuba8	Rps11
Pfkp	Rab3a	Tubb2a	Gpam

Pfn1	Rab5a	Tubb4b	Hsd11b1
Pgam1	Rab5b	Tubb5	Slc38a3
Pgam2	Rab5c	Tufm	Rrbp1
Pgam5	Rab7a	Txn	Dpm1
Pgd	Rab8a	Txn2	Vdac1
Pgk1	Rab8b	Txndc5	Haao
Pgm1	Rac1	Txnrd2	Emc2
Pgm2	Rack1	Uba1	Cyp2c40
Pgrmc1	Rala	Ube2m	Mthfd1
Pgs1	Ralb	Ubl3	Abhd2
Phb	Ran	Ubtd1	Cyp2c39
Phb2	Rap1a	Ubxn4	Myo1c
Phyh	Rap1b	Ucp3	Rgn
Pi4k2a	Rap2b	Ufsp2	C4b
Pi4ka	Rap2c	Uggt1	Ndufa5
Picalm	Rars1	Ugp2	Ugt3a1
Pigs	Rcn1	Unc45b	Pklr
Pip4p2	Rdh13	Uqcc1	Rap1a
Pisd	Rdh14	Uqcc2	Rap2a
Pitrm1	Rdx	Uqcr10	Erbin
Pkm	Reep1	Uqcrb	Snd1
Plaa	Reep5	Uqcrc1	Ttc7a
Plbd1	Retsat	Uqcrc2	Rps17
Plcb4	RGD1560334_pre	Uqcrfs1	Slc25a4
Plcd1	RGD1565784	Uqcrh	Tm7sf2
Plcd3	Rhoa	Uqcrq	Phb
Plg	Rhog	Usp14	Dgat1
Plgrkt	Rmdn1	Usp5	Bsg
Plin4	Rnf170	Vamp7	Ddx3x
Plin5	Rnh1	Vapa	Cyp3a13
Plod3	Rnpep	Vapb	Vamp2
Plpp7	Rock1	Vars1	Eno1
Pls3	Rock2	Vat1	Mtnd5
Plscr3	Romo1	Vcl	Ndufa7
Plxnb1	Rpl10	Vcp	Tmtc3
Plxnb2	Rpl10a	Vdac1	Ndufv1
Plxnd1	Rpl11	Vdac2	C8b
Pmpca	Rpl12	Vdac3	Cd81
Pmpcb	Rpl13	Vim	Serpina3n
Pnkd	Rpl13a	Vldlr	Rdx
Pnp	Rpl14	Vps35	Slco1b2
Pnpla8	Rpl15	Vps45	G6pc
Pnpt1	Rpl17	Vps4a	Slc25a5
Podxl	Rpl18	Vti1b	Cyp27a1
Poldip2	Rpl18a	Vtn	Jbts17
Pon2	Rpl19	Vwa8	Gabarapl2
Popdc2	Rpl21	Wasf2	Actn4
Por	Rpl22	Wdr1	Aldh3a2
Ppa1	Rpl23	Wfs1	Cd38
Ppa2	Rpl23a	Xdh	Rnf213
Ppia	Rpl24	Yars2	Rpl27a
Ppib	Rpl26	Yme1l1	Kras
Ppid	Rpl27	Ywhab	Ghdc
Ppif	Rpl27a	Ywhae	Slc30a5
Ppm1f	Rpl28	Ywhag	Tmem147
Ppp1cc	Rpl29	Ywhah	Cyb5r3
Ppp2cb	Rpl3	Ywhaq	Gna11

Ppp2r1b	Rpl30	Ywhaz	Ndufs1
Ppp2r2a	Rpl31	Zadh2	Mtnd2
Ppp2r5a	Rpl32	Zmpste24	Acadvl
Ppp5c	Rpl34		Tor1a
Ppt1	Rpl35		Pcyox1
Pptc7	Rpl35a		Clta
Prdx1	Rpl36		Pdha1
Prdx2	Rpl36a		Rpsa
Prdx3	Rpl37a-ps1		Pigu
Prdx4	Rpl38		Ndufa10
Prdx5	Rpl3l		Cfi
Prdx6	Rpl4		Ap2m1
Preb	Rpl5		Acnat1
Prelp	Rpl6		Aldh8a1
Prkaa2	Rpl7		Ktn1
Prkaca	Rpl7a		Colgalt1
Prkag1	Rpl8		Cct8
Prkar1a	Rpl9		Rpl35a
Prkar2a	Rplp0		Cyp2j5
Prkcd	Rplp1		Ppfibp1
Prkcsh	Rplp2		Zw10
Prnp	Rpn1		Anxa7
Prodh1	Rpn2		Mlec
Prps1	Rps10		Hmgcs2
Prxl2a	Rps11		Abhd14b
Psap	Rps12		Gna14
Psma1	Rps13		Tmed2
Psma2	Rps14		Srd5a1
Psma3	Rps15		Acsf2
Psma4	Rps15a		Anp32a
Psma5	Rps16		Rpn1
Psma6	Rps17		Cs
Psma7	Rps18		Manf
Psmb1	Rps19		Suclg1
Psmb2	Rps2		Me1
Psmb4	Rps20		Enpp1
Psmb5	Rps21		Nomo1
Psmb6	Rps23		Scamp3
Psmc1	Rps24		Cct5
Psmc2	Rps27a		Hsd3b4
Psmc3	Rps27l		Got2
Psmc4	Rps28		Ssr4
Psmc5	Rps3		Rala
Psmc6	Rps3a		Tomm70
Psmd1	Rps4x		Psma1
Psmd11	Rps5		Ssr3
Psmd12	Rps6		Hoxa11
Psmd13	Rps7		Aldh2
Psmd2	Rps8		Snx9
Psmd3	Rps9		Sec14l4
Psmd4	Rpsa		H2-Q10
Psmd6	Rras		Erp29
Psmd7	Rras2		Rab7a
Psmd8	Rrbp1		Cyp2d26
Psme1	Rtca		Nat8
Psme2	Rtcb		Eefsec
Ptcd3	Rtn2		Ndufs7

Ptges2	Rtn4
Ptges3	Rtn4ip1
Ptgfrn	Rtraf
Ptgis	Ryr1
Ptgr1	S100a10
Ptgr2	S100a6
Ptpmt1	Sacm1l
Ptpn11	Samm50
Ptprc	Sar1b
Ptprm	Sars2
Ptrh2	Sbds
Pygb	Sbk2
Pygm	Scamp1
Pyroxd2	Scamp3
Qars1	Scarb2
Qdpr	Sccpdh
Qrsl1	Scfd1
Rab10	Scn1b
Rab11b	Scn4a
Rab12	Scn4b
Rab13	Sco1
Rab14	Scp2
Rab18	Scpep1
Rab1A	Sdha
Rab1b	Sdhaf2
Rab21	Sdhb
Rab2a	Sdhc
Rab35	Sdhd
Rab3a	Sdr39u1
Rab3d	Sec22b
Rab4b	Sec23a
Rab5a	Sec31a
Rab5b	Sec61a2
Rab5c	Sec61b
Rab6a	Sec61g
Rab7a	Sec62
Rab8a	Sec63
Rab9a	Sel1l
Rac1	Selenbp1
Rack1	Selenof
Rad23b	Sema3c
Rala	Sema7a
Ralb	Septin11
Ran	Septin2
Rap1a	Septin7
Rap1b	Septin9
Rars1	Serbp1
Rasa3	Serpina1
Rasip1	Serpina3k
Rcn1	Serpinc1
Rcn2	Serpinf1
Rcn3	Serpinh1
Rdh13	Setd3
Rdh14	Sfxn3
Rdx	Sgca
Rer1	Sgcb
Retsat	Sgcd

Slc38a4
Pex1
Slc16a1
Retsat
Apool
Acads
Stab2
Rpl26
Ninj1
Cth
Rab14
Gstz1
Abca6
Rap2c
Rab21
Rps25
Rpl23
Slc7a2
Prodh2
Fmo2
Fam3c
Etf1
Tmed9
Cdk5rap3
Glyat
Sptan1
Mat2a
Ndst2
Ndufa13
Ncln
Irgm1
Nup155
Cpn2
Pi4k2a
Endog
Abcd3
Suclg2
Echs1
Adpgk
Abce1
Ahsg
Enpep
Blvrb
Stt3a
Rps6
Rps4x
Aqp9
Myl9
Slc2a2
Sord
Spen
Acot8
Rpl13a
Atp1a1
Sqle
Atp5d
Sdha

Rexo2	Sgcg
Rftn1	Sgpl1
RGD1309362	Sh3glb1
RGD1565784	Shmt2
Rhoa	Siglec1
Rhob	Sirt2
Rhog	Sirt3
Rhot1	Skp1
Rhot2	Slc12a2
Rlc-a	Slc16a1
Rmc1	Slc16a3
Rmdn1	Slc25a1
Rmnd1	Slc25a10
Rnh1	Slc25a11
Rnpep	Slc25a12
Rock1	Slc25a13
Rock2	Slc25a19
Romo1	Slc25a24
Rpl10	Slc25a29
Rpl10a	Slc25a3
Rpl11	Slc25a35
Rpl12	Slc25a4
Rpl13	Slc25a42
Rpl13a	Slc25a46
Rpl14	Slc25a5
Rpl15	Slc25a51
Rpl17	Slc27a1
Rpl18	Slc2a4
Rpl18a	Slc30a9
Rpl19	Slc37a4
Rpl21	Slc3a2
Rpl22	Slc43a1
Rpl23	Slc43a2
Rpl23a	Slc44a2
Rpl24	Slc4a1
Rpl26	Slirp
Rpl27	Slk
Rpl27a	Slmap
Rpl28	Smim26
Rpl3	Smpdl3b
Rpl30	Smpx
Rpl31	Smtnl1
Rpl32	Smtnl2
Rpl35a	Smyd1
Rpl36	Smyd2
Rpl37a-ps1	Snap23
Rpl38	Snapin
Rpl3l	Sncg
Rpl4	Snd1
Rpl5	Snta1
Rpl6	Sntb1
Rpl7	Snx2
Rpl7a	Snx5
Rpl8	Sod1
Rpl9	Sod2
Rplp0	Spag9
Rplp1	Spart

Hck
Gnai2
Aco2
Acadl
Ndufaf2
Spta1
Itih2
Erap1
Arpc2
Slc25a13
Hspg2
Mpc2
Bcap31
Atp1b3
Ndufs2
Aldh6a1
Acox2
Hsd3b5
Ndufa12
Vcp
Smpd2
Sacm1l
Atp1b1
Hsd17b11
Ddost
Ttr
Slc27a5
Tm9sf1
Uox
Ncl
Arhgap10
Ckmt2
Col7a1
Comtd1
Dip2b
Hivep3
Itgal
Keg1
Kif5a
Parva
Ptpmt1
Slc30a1
Tmtc4
Ywhaz
Zc3h3
Zfp62
Zg16
Zmpste24
Zw10

Rplp2	Spcs3
Rpn1	Speg
Rpn2	Spg7
Rps11	Spryd4
Rps12	Spryd7
Rps13	Spta1
Rps14	Sptan1
Rps16	Sptb
Rps17	Sptbn1
Rps18	Sqor
Rps19	Sri
Rps2	Srl
Rps20	Srpra
Rps23	Ssbp1
Rps24	Sspn
Rps25	Ssr1
Rps26	Ssr3
Rps27	Ssr4
Rps27a	St13
Rps3	Stab1
Rps3a	Stac3
Rps4x	Stat5b
Rps5	Stbd1
Rps6	Steap3
Rps8	Steap4
Rps9	Stim1
Rpsa	Stim2
Rrad	Stip1
Rras	Stom
Rras2	Stoml2
Rrbp1	Stt3a
Rsu1	Stt3b
RT1-Aw2	Stub1
RT1-Ba	Stx12
Rtcb	Stx4
Rtn2	Stx7
Rtn4	Stx8
Rtn4ip1	Stxbp1
Rtraf	Stxbp3
Ryr2	Sucla2
S100a10	Suclg1
S100a8	Suclg2
S1pr1	Sun2
Sacm1l	Supv3l1
Samm50	Surf1
Sar1b	Susd2
Sardh	Svip
Sars1	Syncrip
Sars2	Synj2bp
Sbds	Synm
Scamp1	Synpo2
Scarb2	Sypl1
Sccpdh	Sypl2
Scfd1	Taco1
Scn7a	Tagln
Sco1	Tagln2
Scp2	Tap1

Scpep1	Tapbp
Sdcbp	Tapt1
Sdha	Tars2
Sdhaf2	Tbc1d17
Sdhb	Tbrg4
Sdhc	Tcp1
Sdhd	Tecr
Sdr39u1	Tefm
Sec11a	Tf
Sec22b	Tfam
Sec23a	Tfrc
Sec24c	Tgfbi
Sec61a1	Tgm2
Sec61a2	Thbs4
Sec63	Timm10
Selenbp1	Timm13
Septin11	Timm21
Septin2	Timm23
Septin7	Timm29
Septin8	Timm44
Serbp1	Timm50
Serhl2	Timm8a
Serpina1	Timm8b
Serpina3l	Timm9
Serpina3n	Timmdc1
Serpina6	Tkt
Serpinb6a	Tln1
Serpinc1	Tln2
Serpinf1	Tm9sf2
Serpinh1	Tm9sf3
Set	Tm9sf4
Sfxn1	Tmco1
Sfxn3	Tmed1
Sgca	Tmed10
Sgcb	Tmed2
Sgcd	Tmed4
Sgcg	Tmed7
Sh3gl2	Tmed9
Sh3glb1	Tmem109
Shank3	Tmem11
Shmt2	Tmem126a
Siglec1	Tmem126b
Sirpa	Tmem131l
Sirt2	Tmem143
Sirt3	Tmem186
Sirt5	Tmem214
Skp1	Tmem33
Slc12a7	Tmem38a
Slc16a1	Tmem38b
Slc25a1	Tmem43
Slc25a10	Tmem65
Slc25a11	Tmem70
Slc25a12	Tmlhe
Slc25a13	Tmod1
Slc25a15	Tmsb4x
Slc25a18	Tmx2
Slc25a19	Tmx3

Slc25a21	Tnnc1
Slc25a24	Tnnc2
Slc25a29	Tnni1
Slc25a3	Tnni2
Slc25a34	Tnnt3
Slc25a35	Tomm20
Slc25a4	Tomm22
Slc25a40	Tomm34
Slc25a42	Tomm40
Slc25a46	Tomm40l
Slc25a5	Tomm5
Slc25a51	Tomm6
Slc27a1	Tomm70
Slc28a2	Tor1aip2
Slc2a4	Tpd52l2
Slc30a9	Tpi1
Slc38a2	Tpm1
Slc3a2	Tpm3
Slc43a1	Tpm4
Slc44a2	Tpp1
Slc4a11	Tppp3
Slc8a1	Tpt1
Slc9a1	Trabd
Slc9a3r2	Trap1
Slirp	Trappc8
Slmap	Trdn
Smc1a	Trim72
Smim8	Trip10
Smpdl3b	Tsfm
Smyd1	Tspan8
Snap23	Tst
Snd1	Tuba4a
Snta1	Tuba8
Sntb1	Tubb2a
Sntb2	Tubb4b
Snx2	Tubb5
Snx5	Tufm
Snx6	Txn
Sod1	Txn2
Sod2	Txndc5
Sorbs1	Txnrd2
Sorbs2	Uba1
Sparc	Ube2d2
Spcs3	Ube2m
Speg	Ube2n
Spg7	Ubl3
Spr	Ubtd1
Spryd4	Ubxn4
Sptan1	Ucp3
Sqor	Ufd1
Srl	Ufsp2
Srprb	Uggt1
Ssbp1	Ugp2
Ssr1	Unc45b
Ssr4	Uqcc1
St13	Uqcc2
Stat1	Uqcr10

Steap3	Uqcrb
Steap4	Uqcrc1
Stim1	Uqcrc2
Stip1	Uqcrfs1
Stom	Uqcrh
Stoml2	Uqcrq
Stt3a	Usp14
Stt3b	Usp5
Stx12	Vamp2
Stx4	Vamp3
Stx5	Vamp5
Stx7	Vamp7
Stxbp1	Vapa
Stxbp3	Vapb
Sucla2	Vars1
Suclg1	Vat1
Suclg2	Vcl
Suox	Vcp
Supv3l1	Vdac1
Surf1	Vdac2
Susd2	Vdac3
Syn2	Vim
Syncrip	Vkorc1l1
Synj2bp	Vldlr
Sypl1	Vps13c
Taco1	Vps35
Tagln	Vps39
Tagln2	Vps45
Taldo1	Vps4a
Tamm41	Vti1b
Tap1	Vtn
Tap2	Vwa8
Tapbp	Wasf2
Tapt1	Wdr1
Tars2	Wdr62
Taz	Wfs1
Tbl2	Xdh
Tbrg4	Xpnpep3
Tcp1	Xpo1
Tecr	Yars1
Tecrl	Yars2
Tefm	Yme1l1
Tesc	Ywhab
Tfam	Ywhae
Tfrc	Ywhag
Tgfbi	Ywhah
Tgm1	Ywhaq
Tgm2	Ywhaz
Thbd	Zadh2
Thbs1	Zmpste24
Theg	
Them4	
Thnsl1	
Timm10	
Timm10b	
Timm13	
Timm21	

Timm22
Timm23
Timm29
Timm44
Timm50
Timm9
Timmdc1
Tjp1
Tjp2
Tln1
Tln2
Tm9sf2
Tm9sf3
Tm9sf4
Tmed1
Tmed10
Tmed2
Tmed4
Tmed5
Tmed7
Tmed9
Tmem109
Tmem11
Tmem120a
Tmem126a
Tmem143
Tmem177
Tmem182
Tmem186
Tmem205
Tmem242
Tmem30a
Tmem33
Tmem38a
Tmem43
Tmem65
Tmem70
Tmlhe
Tmod1
Tmod2
Tmod3
Tmsb4x
Tmx1
Tmx2
Tmx3
Tmx4
Tnnc1
Tnni3
Tnni3k
Tnnt2
Tns1
Tns2
Tomm20
Tomm22
Tomm34
Tomm40
Tomm40l

Tomm6
Tomm70
Tpd52l1
Tpd52l2
Tpi1
Tpm1
Tpm3
Tpm4
Tpp1
Tpp2
Tppp3
Tpt1
Trabd
Trak1
Trap1
Trappc3
Trim25
Trim72
Triobp
Trmt10c
Trnt1
Tsfm
Tsg101
Tspan8
Tst
Ttc19
Ttr
Tuba4a
Tuba8
Tubb2a
Tubb4b
Tubb5
Tubb6
Tufm
Twf2
Txlnb
Txn
Txn2
Txndc12
Txndc5
Txnl1
Txnrd2
Uaca
Uba1
Ube2m
Ubl3
Ubr4
Ubtd1
Ubxn4
Ucp3
Ufl1
Ufsp2
Uggt1
Ugp2
Unc45b
Uqcc1
Uqcc2

Uqcc3
Uqcr10
Uqcrb
Uqcrc1
Uqcrc2
Uqcrfs1
Uqcrh
Uqcrq
Usp14
Usp24
Usp5
Usp9x
Utrn
Vamp7
Vapa
Vapb
Vars1
Vat1
Vcl
Vcp
Vdac1
Vdac2
Vdac3
Vim
Vldlr
Vnn1
Vps13a
Vps26a
Vps29
Vps35
Vps36
Vps45
Vps4a
Vta1
Vti1b
Vtn
Vwa8
Vwf
Wars1
Wars2
Wasf2
Wdr1
Wfs1
Xdh
Xpnpep2
Yars2
Yme1l1
Ywhab
Ywhae
Ywhag
Ywhah
Ywhaq
Ywhaz
Zadh2
Zmpste24

Supplementary Table 5b. The most significant cellular compartments (GOCC) of 176 identified consensus MAM proteins in this work				
**Term**	**PValue**	**FDR**	**Count**	**Proteins involved**
GO:0070062~extracellular exosome	7.56E-56	1.88E-53	116	RPL4, RPL5, ACAA2, CD81, CISD1, ENO1, PARK7, RPL10A, PHB2, RPL7, RPS14, CFL1, PDIA3, RAB2A, RPS9, RPS8, ACTN1, ACSL4, ACTN4, ATP1B1, SDHB, PDIA6, SND1, EEF1A1, MTHFD1, ERP29, VDAC3, VDAC2, VDAC1, SUCLG1, UQCRC2, LAP3, PPIB, ATP6V1A, RAB5B, VCP, TMED10, MAOB, NDUFB10, PHB, NDRG2, HSP90B1, ACAT1, LDHA, PRDX5, PRDX1, PGK1, PACSIN3, APOE, DECR1, HSPA9, HSPA8, RDX, GOT2, IDH2, MSN, ERLIN2, PA2G4, EEF2, PRDX6, CS, QDPR, ALDH6A1, GNB1, ALB, GNAS, HYOU1, CALR, SLC25A1, ITGB1, CLIC4, SLC25A3, HSP90AB1, COX4I1, RPLP0, CLTC, ETFA, ANXA6, RPLP2, PGM1, TPI1, RPS3A, DNM2, HADHB, TST, CANX, RAB7A, PCYOX1, ALDH9A1, FH, RALA, ECHS1, RAB1B, NDUFB4, GSTP1, ATP1A1, COX5A, GNAI2, HSPD1, RAB21, GNA13, GANAB, SAMM50, RPS3, CCT8, UGGT1, SLC16A1, MDH1, NDUFA4, MDH2, ERP44, MYO1B, RAB14, CAPZA2, RAB18, P4HB
GO:0005739~mitochondrion	5.43E-53	6.74E-51	94	SLC25A1, NDUFA13, CLIC4, SLC25A3, ABCD3, HSP90AB1, ACAA2, COX4I1, CLTC, CISD1, ETFA, PARK7, RPL10A, PHB2, RPS14, ACADL, AIFM1, ANXA6, UQCRFS1, DLAT, PDIA3, ACSL1, MCCC1, ACSL4, SDHA, SDHB, SND1, HADHB, AFG3L2, HADHA, MTHFD1, TST, NDUFS7, BDH1, ACOX1, GPD2, VDAC3, UQCRC1, NDUFS3, NDUFS2, VDAC2, NDUFS1, SUCLG1, VDAC1, LAP3, UQCRC2, SLC25A12, ALDH9A1, ATP6V1A, FH, STOML2, ECHS1, NDUFB6, MAOB, NDUFB10, NDUFB5, ABCB7, RAB1B, NDUFB4, GSTP1, ETFDH, HSD17B4, PHB, COX5A, ACAT1, HSPD1, LDHA, PRDX5, SAMM50, PRDX1, MFN1, CYC1, NDUFV2, NDUFV1, DECR1, NDUFA9, HSPA9, BCAP31, SLC16A1, MDH1, MDH2, NDUFA4, GOT2, IDH2, IMMT, PRDX6, LETM1, QDPR, CS, GLUD1, ALDH6A1, PC, CYCS, ACO2
GO:0005743~mitochondrial inner membrane	2.53E-49	2.09E-47	52	SLC25A1, NDUFA13, SLC25A3, ABCD3, ACAA2, COX4I1, PHB2, AIFM1, UQCRFS1, MCCC1, SDHA, SDHB, HADHB, AFG3L2, HADHA, TST, NDUFS7, BDH1, GPD2, VDAC3, UQCRC1, NDUFS3, VDAC2, SUCLG1, VDAC1, UQCRC2, SLC25A12, STOML2, MAOB, NDUFB10, NDUFB5, ABCB7, NDUFB4, ETFDH, PHB, COX5A, ACAT1, HSPD1, SAMM50, RPS3, CYC1, NDUFV1, NDUFA9, MDH2, NDUFA4, GOT2, IDH2, IMMT, LETM1, GLUD1, PC, CYCS
GO:0043209~myelin sheath	1.82E-38	1.13E-36	38	ATP6V1A, SLC25A3, RALA, VCP, CLTC, PHB, ENO1, ATP1A1, COX5A, HSPD1, PRDX1, UQCRFS1, DLAT, NDUFV2, HSPA9, PDIA3, HSPA8, MDH1, MDH2, RDX, GOT2, MSN, IMMT, SDHA, ATP1B1, EEF1A1, GNB1, CANX, ALB, UQCRC1, NDUFS3, VDAC2, CYCS, VDAC1, NDUFS1, UQCRC2, ACO2, SLC25A12
GO:0005925~focal adhesion	1.50E-26	7.44E-25	38	RPL4, ITGB1, RPL5, RALA, LRP1, CD81, RPLP0, CLTC, RPL10A, RPL8, RPL9, RPL6, RPL7, HSP90B1, RAB21, GNA13, RPS14, CFL1, ANXA6, RPS3, RPLP2, RPL18, HSPA9, PDIA3, HSPA8, RPS9, RPS8, ACTN1, RDX, MSN, RPS3A, ACTN4, DNM2, HYOU1, LAP3, CALR, P4HB, PPIB
GO:0016020~membrane	4.16E-24	1.72E-22	74	RPL4, ITGB1, RPL5, SLC25A3, ABCD3, HSP90AB1, CD81, COX4I1, RPLP0, CLTC, ATP2A2, AP2A1, ENO1, RPL10A, RPL8, RPL9, PHB2, RPL6, RPL7, RPS14, CFL1, ANXA6, RPLP2, RAB2A, RPS9, ACSL1, RPS8, ACSL4, SDHA, ATP1B1, DDOST, SND1, EEF1A1, MTHFD1, ACOX1, CANX, ERP29, VDAC1, PPIB, LRP1, RPL10, RPN1, HSD17B4, RRBP1, PHB, ATP1A1, GNAI2, HSP90B1, HSPD1, GNA13, LDHA, GANAB, PGK1, RPS3, MLEC, APOE, CYC1, RPL18, BCAP31, HSPA8, SLC16A1, MDH2, IMMT, PA2G4, AP2B1, EEF2, PRDX6, VAPA, CAPZA2, GNAS, HYOU1, CALR, RPS24, PICALM
GO:0042470~melanosome	7.06E-18	2.50E-16	18	RAB2A, PDIA3, ITGB1, HSPA8, HSP90AB1, TMED10, CLTC, RPN1, PDIA6, SND1, HSP90B1, GNA13, PRDX1, CANX, ERP29, ANXA6, P4HB, PPIB
GO:0005747~mitochondrial respiratory chain complex I	1.17E-13	3.64E-12	13	NDUFA9, NDUFB10, NDUFB6, NDUFA4, NDUFB5, NDUFB4, PARK7, NDUFS7, NDUFS3, NDUFS2, NDUFS1, NDUFV2, NDUFV1
GO:0005759~mitochondrial matrix	1.38E-13	3.81E-12	18	NDUFA9, ECHS1, ACAA2, MDH2, MCCC1, GOT2, PARK7, ACAT1, HSPD1, CS, GLUD1, PC, ACADL, BDH1, TST, PRDX1, RPS3, DLAT
GO:0005783~endoplasmic reticulum	2.69E-09	6.68E-08	29	CLIC4, VCP, TMED10, RPL10, PRKCSH, ATP2A2, RRBP1, ATP1A1, PARK7, MTDH, HSP90B1, MLEC, APOE, UGGT1, BCAP31, PDIA3, ACSL1, ACSL4, ERLIN2, DDOST, HADHB, ERP44, VAPA, CANX, ALB, ERP29, CALR, P4HB, PPIB
GO:0005790~smooth endoplasmic reticulum	7.21E-09	1.63E-07	8	PDIA3, CANX, ERP29, HYOU1, CALR, PPIB, PDIA6, HSP90B1
GO:0005788~endoplasmic reticulum lumen	8.97E-09	1.85E-07	10	PDIA3, ERP44, ERP29, HYOU1, P4HB, CALR, PPIB, UGGT1, PDIA6, HSP90B1
GO:0030529~intracellular ribonucleoprotein complex	1.65E-08	3.15E-07	12	RPL4, RPL5, HSPA8, RPS9, RPS8, RPLP0, RPS3, RPS3A, ACTN4, PA2G4, EEF2, RPL7
GO:0048471~perinuclear region of cytoplasm	3.37E-08	5.96E-07	24	RAB2A, ITGB1, HSPA8, VCP, CLIC4, LRP1, MSN, ATP2A2, PARK7, ACTN4, NDRG2, HSP90B1, DNM2, MTDH, MYO1B, PRDX5, VAPA, AIFM1, RAB14, GNAS, ANXA6, CALR, PPIB, PICALM
GO:0005829~cytosol	5.33E-08	8.82E-07	39	ATP6V1A, CLIC4, RALA, VCP, FH, HSP90AB1, GSTP1, PARK7, GNAI2, HSP90B1, HSPD1, LDHA, PRDX5, AIFM1, PRDX1, PGK1, RPS3, CCT8, PGM1, HSPA8, TPI1, MDH1, ACTN1, IDH2, RPS3A, ACTN4, EEF2, PRDX6, DNM2, QDPR, EEF1A1, MTHFD1, RAB14, GNAS, CYCS, SUCLG1, CALR, RAB7A, ALDH9A1

GO:0031966~mitochondrial membrane	3.11E-07	4.81E-06	10	NDUFA9, NDUFB6, ACADL, COX4I1, ETFDH, NDUFS3, UQCRFS1, ACSL4, SDHB, DNM2
GO:0005741~mitochondrial outer membrane	1.89E-06	2.76E-05	10	HADHB, SAMM50, MAOB, ACSL1, MFN1, VDAC3, CISD1, VDAC2, VDAC1, PHB2
GO:0034663~endoplasmic reticulum chaperone complex	3.43E-06	4.73E-05	5	HYOU1, P4HB, PPIB, PDIA6, HSP90B1
GO:0043234~protein complex	4.62E-06	6.04E-05	20	HSPA8, VCP, GSTP1, CLTC, GOT2, ATP2A2, AP2A1, ERLIN2, ATP1A1, ACTN4, PHB2, DDOST, DNM2, HSPD1, CANX, ALB, ANXA6, SUCLG1, VDAC1, CALR

Supplementary Table 5c. The most significant biological processes (GOBP) of 176 identified consensus MAM proteins in this work				
**Term**	**PValue**	**FDR**	**Count**	**Proteins involved**
GO:0006099~tricarboxylic acid cycle	5.19E-12	6.02E-09	10	CS, FH, MDH1, MDH2, IDH2, SUCLG1, ACO2, DLAT, SDHA, SDHB
GO:0006635~fatty acid beta-oxidation	1.26E-08	7.28E-06	9	HADHB, HADHA, ECHS1, ACAA2, ABCD3, ACOX1, HSD17B4, DECR1, ACAT1
GO:0055114~oxidation-reduction process	1.88E-08	7.28E-06	25	MAOB, NDUFB4, LDHA, PRDX5, ACADL, AIFM1, PRDX1, UQCRFS1, NDUFA9, PDIA3, NDUFA4, SDHA, PRDX6, QDPR, HADHB, GLUD1, ALDH6A1, NDUFS7, BDH1, MTHFD1, GPD2, NDUFS3, NDUFS2, PCYOX1, ALDH9A1
GO:0055115~oxidation-reduction process	1.88E-08	7.28E-06	25	MAOB, NDUFB4, LDHA, PRDX5, ACADL, AIFM1, PRDX1, UQCRFS1, NDUFA9, PDIA3, NDUFA4, SDHA, PRDX6, QDPR, HADHB, GLUD1, ALDH6A1, NDUFS7, BDH1, MTHFD1, GPD2, NDUFS3, NDUFS2, PCYOX1, ALDH9A2
GO:0006457~protein folding	1.43E-06	3.32E-04	10	HSPA9, HSPA8, ERP44, HSP90AB1, ERP29, CCT8, CALR, UGGT1, PDIA6, HSP90B1
GO:0045454~cell redox homeostasis	2.78E-06	5.37E-04	8	PDIA3, ERP44, PRDX5, AIFM1, PRDX1, P4HB, PRDX6, PDIA6
GO:0006107~oxaloacetate metabolic process	7.91E-06	0.00131242	5	CS, PC, MDH1, MDH2, GOT2
GO:0034976~response to endoplasmic reticulum stress	1.47E-05	0.00198285	8	PDIA3, ERP44, ERP29, ATP2A2, HYOU1, P4HB, EEF2, PDIA6
GO:0042493~response to drug	1.54E-05	0.00198285	18	ITGB1, HSPA8, HSP90AB1, ABCD3, MAOB, ACSL1, HSD17B4, PHB, PARK7, ATP1A1, EEF2, HSPD1, HADHA, LDHA, BDH1, GNAS, UQCRFS1, CALR
GO:0045471~response to ethanol	1.91E-05	0.00221877	11	PDIA3, HSPA8, MAOB, BDH1, GSTP1, GOT2, RPS3A, PHB, APOE, RPL10A, EEF2
GO:0005975~carbohydrate metabolic process	2.97E-05	0.00294628	9	CS, LDHA, GANAB, TPI1, MDH1, MDH2, PGK1, MLEC, PGM1
GO:0044794~positive regulation by host of viral process	3.05E-05	2.95E-03	4	PC, CFL1, APOE, PPIB
GO:0002931~response to ischemia	8.91E-05	0.00742689	6	PDIA3, AIFM1, CYCS, HYOU1, EEF2, HSPD1
GO:0006979~response to oxidative stress	8.96E-05	0.00742689	9	PRDX5, PRDX1, NDUFB4, ETFDH, RPS3, NDUFS2, CYCS, APOE, PARK7
GO:0006734~NADH metabolic process	1.85E-04	0.01429521	4	VCP, MDH1, MDH2, GPD2
GO:0050821~protein stabilization	2.77E-04	0.02006485	8	PHB, CCT8, PARK7, CALR, ATP1B1, PPIB, PHB2, HSPD1
GO:0042407~cristae formation	3.74E-04	0.02553416	4	LETM1, AFG3L2, SAMM50, IMMT
GO:0002181~cytoplasmic translation	4.17E-04	0.02689386	6	RPLP0, RPLP2, RPL8, RPL9, RPL6, RPL7
GO:0098869~cellular oxidant detoxification	5.50E-04	0.03360373	6	PRDX5, PRDX1, GSTP1, APOE, PARK7, PRDX6
