# Supplementary material for: Ultrastructural and proteomic profiling of mitochondria-associated endoplasmic reticulum membranes reveal aging signatures in striated muscle
Source: Cell Death Dis. 2022 Apr 2;13(4):296. doi: 10.1038/s41419-022-04746-4 (PMC8976840; doi:10.1038/s41419-022-04746-4)
Supplement: Supplementary file 8 — Supplementary Table 6. Subcellular mapping of the MAM proteome. [file 41419_2022_4746_MOESM8_ESM.pdf]

Supplementary Table 6a. Subcellular map of MAM proteome from heart in this work

| | SAFE | NMF | Uniprot/GO | MetaMass | HPA |
|---|---|---|---|---|---|
| Mitochondrion | 43.25 | 39.55 | 48.37 | 40.51 | 46.76 |
| ER | 20.17 | 20.43 | 10.01 | 14.70 | 10.00 |
| PM | 8.62 | 10.27 | 6.88 | 10.85 | 3.82 |
| Endosome/Lysosome | 10.57 | 4.90 | 2.53 | 3.67 | 0.00 |
| Cytosol, CS | 5.01 | 8.12 | 19.66 | 19.77 | 27.94 |
| Nucleus | 1.25 | 1.91 | 2.53 | 1.92 | 11.47 |
| Other | 11.13 | 14.81 | 10.01 | 8.57 | 0.00 |
| Proteins identified （Total 1876） | 804.00 | 938.00 | 879.00 | 1252.00 | 370.00 |

Supplementary Table 6b. Subcellular map of MAM proteome from GA muscle in this work

| | SAFE | NMF | Uniprot/GO | MetaMass | HPA |
|---|---|---|---|---|---|
| Mitochondrion | 46.89 | 37.85 | 48.37 | 40.51 | 46.76 |
| ER | 17.41 | 17.06 | 10.01 | 14.7 | 10 |
| PM | 12.69 | 10.02 | 6.88 | 10.85 | 3.82 |
| Endosome, lysosome | 4.98 | 4.26 | 2.53 | 3.67 | 0 |
| Cytosol, CS | 4.48 | 8.96 | 19.66 | 19.77 | 27.94 |
| Nucleus | 1.24 | 2.03 | 2.53 | 1.92 | 11.47 |
| Other | 12.31 | 19.83 | 10.01 | 8.57 | 0 |
| Proteins identified （Total 1695） | 719.00 | 837.00 | 829.00 | 1143.00 | 340.00 |

Supplementary Table 6c. SAFE-based map of MAM proteomics data from different tissues

| SAFE | A - Mouse Liver - Nat Cell Biol. 2019 [19] | B - Mouse Testis - Proteomics. 2018 [24] | C - MOUSE Brain - Proteomics. 2018 [24] | D - Mouse Brain - Sci Rep. 2017 [25] | E - MAM Consensus [19] |
|---|---|---|---|---|---|
| Mitochondrion | 23.25 | 19.27 | 20.26 | 24.6 | 13.2 |
| ER | 30.67 | 23.7 | 22.33 | 18.12 | 16.23 |
| PM | 15.83 | 15.32 | 17.82 | 21.84 | 10.5 |
| Endosome/Lysosome | 3.71 | 7.9 | 9.94 | 6.47 | 5.41 |
| Cytosol, Cs | 7.28 | 13.11 | 15.01 | 10.03 | 8.98 |
| Nucleus | 4.14 | 5.69 | 1.88 | 2.1 | 3.9 |
| Other | 15.12 | 15.01 | 12.76 | 16.83 | 41.79 |
| Proteins identified | 701 (Total 1466) | 633 (Total 2808) | 533 (Total 2478) | 618 (Total 1313) | 146 (Total 216) |

Supplementary Table 6d. NMF-based map of MAM proteomics data from different tissues

| NMF | A - Mouse Liver - Nat Cell Biol. 2019 [19] | B - Mouse Testis - Proteomics. 2018 [24] | C - MOUSE Brain - Proteomics. 2018 [24] | D - Mouse Brain - Sci Rep. 2017 [25] | E - MAM Consensus [19] |
|---|---|---|---|---|---|
| Mitochondrion | 24.71 | 18.41 | 19.27 | 19.27 | 39.84 |
| ER | 32.01 | 24.91 | 23.77 | 23.77 | 26.83 |
| PM | 13.76 | 13.18 | 15.42 | 15.42 | 10.57 |
| Endosome/Lysosome | 3.48 | 7.4 | 9.85 | 9.85 | 1.63 |
| Cytosol, Cs | 3.48 | 6.32 | 6.85 | 6.85 | 4.07 |
| Nucleus | 3.65 | 7.22 | 3.21 | 3.21 | 0.81 |
| Other | 18.91 | 22.56 | 21.63 | 21.63 | 16.26 |
| Proteins identified | 603 (Total 1466) | 554 (Total 2808) | 467 (Total 2478) | 515 (Total 1313) | 123 (Total 216) |

CS, cytoskeleton; PM, plasma membrane; HPA, the immunofluorescence-based location data from Human Protein Atlas; Uniprot/GO, proteins assigned with a single location in either Uniprot or Gene Ontology Consortium; SAFE, the BioID-based location prediction using spatial analysis of functional enrichment; NMF, the BioID-based location prediction using non-negative matrix factorization; Metamass, a clustering algorithm based subcellular location assignment tool.

| Supplementary Table 6e. Subcellular mapping of deregulated MAM proteins in heart. | | | | |
|---|---|---|---|---|
| **PANTHER: Cellular Compartment** | **Total** | **Hits** | **P.Value** | **FDR** |
| **Mitochondrion** | 1160 | 224 | 2.62E-127 | 1.34E-125 |
| **Mitochondrial inner membrane** | 222 | 64 | 1.35E-44 | 3.43E-43 |
| **Protein containing complex** | 544 | 52 | 2.69E-13 | 4.57E-12 |
| **Ribosome** | 261 | 30 | 4.72E-10 | 6.02E-09 |
| **Peroxisome** | 108 | 18 | 4.46E-09 | 4.55E-08 |
| **Cytosol** | 2640 | 122 | 9.32E-07 | 7.92E-06 |
| **Cytoskeleton** | 544 | 38 | 2.00E-06 | 1.45E-05 |
| **Cytoplasm** | 4330 | 169 | 0.000146 | 0.000859 |
| **Synapse** | 386 | 26 | 0.000152 | 0.000859 |
| **Endoplasmic reticulum** | 983 | 50 | 0.000284 | 0.00145 |
| **Neuron projection** | 341 | 21 | 0.00196 | 0.0091 |
| **Actin cytoskeleton** | 153 | 11 | 0.00747 | 0.0318 |
| **Heterotrimeric G protein complex** | 27 | 4 | 0.0087 | 0.0341 |
| **Endosome** | 343 | 19 | 0.00957 | 0.0349 |
| **Proton transporting ATP synthase complex** | 1 | 1 | 0.0306 | 0.104 |
| **Clathrin coated pit** | 43 | 4 | 0.0418 | 0.133 |

| Supplementary Table 6f. Subcellular mapping of deregulated MAM proteins in GA muscle. | | | | |
|---|---|---|---|---|
| **PANTHER: Cellular Compartment** | **Total** | **Hits** | **P.Value** | **FDR** |
| **Mitochondrion** | 1160 | 90 | 5.48E-38 | 2.79E-36 |
| **Mitochondrial inner membrane** | 222 | 39 | 4.62E-29 | 1.18E-27 |
| **Endoplasmic reticulum** | 983 | 36 | 4.88E-06 | 8.29E-05 |
| **Cytoskeleton** | 544 | 21 | 0.000257 | 0.00328 |
| **Cytosol** | 2640 | 64 | 0.000616 | 0.00628 |
| **Membrane** | 7090 | 139 | 0.00271 | 0.0231 |
| **Lysosome** | 257 | 11 | 0.00352 | 0.0256 |
| **Neuronal cell body** | 391 | 14 | 0.00528 | 0.0337 |
| **Protein containing complex** | 544 | 17 | 0.00848 | 0.0481 |
| **Ribosome** | 261 | 10 | 0.0111 | 0.0522 |
| **Endosome** | 343 | 12 | 0.0113 | 0.0522 |
| **Microvillus** | 55 | 4 | 0.0125 | 0.0532 |
| **Vesicle coat** | 2 | 1 | 0.0325 | 0.121 |
| **Clathrin coated pit** | 43 | 3 | 0.0332 | 0.121 |
| **Microtubule** | 234 | 8 | 0.0394 | 0.134 |
