# Supplementary material for: Ultrastructural and proteomic profiling of mitochondria-associated endoplasmic reticulum membranes reveal aging signatures in striated muscle
Source: Cell Death Dis. 2022 Apr 2;13(4):296. doi: 10.1038/s41419-022-04746-4 (PMC8976840; doi:10.1038/s41419-022-04746-4)
Supplement: Supplementary file 9 — Supplementary Table 7. GSEA summary. [file 41419_2022_4746_MOESM9_ESM.pdf]

<table>
<tr><th colspan="7">Supplementary Table 7a. Enrichment map of MAM proteome derived from the heart and GA muscle samples</th></tr>
<tr><th>Clusters</th><th>EnrichmentMap::Name</th><th>EnrichmentMap::ES</th><th>EnrichmentMap::NES</th><th>EnrichmentMap::pvalue</th><th>EnrichmentMap::gs_size</th><th>EnrichmentMap::Genes</th></tr>
<tr><td>Autophagy</td><td>GOBP_ORGANELLE_DISASSEMBLY</td><td>0.7324</td><td>1.8348</td><td>0</td><td>16</td><td>PHB2|CISD2|PARK7|FIS1|GBA|FUNDC2|GFM2|BNIP3|VCP|MFN2|MTIF2|KIF5B|VDAC1|DNM1L|HK2|MUL1</td></tr>
<tr><td>Autophagy</td><td>GOBP_AUTOPHAGOSOME_ORGANIZATION</td><td>0.6877</td><td>1.6139</td><td>0.0227</td><td>12</td><td>IRGM|MFN2|NSFL1C|SCFD1|GBA|SH3GLB1|RAB7A|RAB1B|STX12|SEC22B|RAB5A|RALB</td></tr>
<tr><td>Autophagy</td><td>GOBP_POSITIVE_REGULATION_OF_AUTOPHAGY</td><td>0.7617</td><td>1.7436</td><td>0</td><td>13</td><td>DCN|IRGM|PHB2|PARK7|SH3GLB1|RALB|MTDH|BNIP3|HMGB1|VDAC1|PRKAA2|ROCK1|FYCO1</td></tr>
<tr><td>Autophagy</td><td>GOBP_PROCESS_UTILIZING_AUTOPHAGIC_MECHANISM</td><td>0.3812</td><td>1.251</td><td>0.0263</td><td>69</td><td>ATP6V0A1|ATP6V0D1|ATP6V1H|ATP6V1A|PARK7|ATP6V1E1|ATP6V1B2|RAB12|SNX5|RAB8A|GAPDH|MFN2|MFN1|HK2|HSPA8|ANXA7|PHB2|HSP90AA1|SIRT2|EEF1A1|VCP|EIF4G1|TOMM70|CLTC|LAMTOR2|LAMTOR5|DNM1L|LAMP2|CTSA|PSAP|DCN|IRGM|PGAM5|FIS1|PRKACA|SH3GLB1|VPS35|ZMPSTE24|RAB5A|RALB|BNIP3|HMGB1|CSNK2A1|VDAC1|ROCK1|RAB7A|FYCO1|MUL1|RAB1B|SEC22B|LAMTOR1|CAPN1|CAPNS1|TOMM22|CISD2|GBA|NEDD4|STX12|TOMM40|EEF1A2|FUNDC2|TOMM20|MTDH|HSPB1|NSFL1C|SCFD1|PRKAA2|PRKAG1|VPS4A</td></tr>
<tr><td>Autophagy</td><td>GOBP_AUTOPHAGY_OF_MITOCHONDRION</td><td>0.7527</td><td>1.7486</td><td>0</td><td>12</td><td>BNIP3|MFN2|PHB2|CISD2|PARK7|FIS1|VDAC1|DNM1L|GBA|HK2|MUL1|FUNDC2</td></tr>
<tr><td>Autophagy</td><td>GOBP_REGULATION_OF_AUTOPHAGY</td><td>0.4171</td><td>1.3221</td><td>0.0444</td><td>44</td><td>ATP6V0A1|DCN|IRGM|ATP6V0D1|ATP6V1H|ATP6V1A|PARK7|ATP6V1E1|PRKACA|SH3GLB1|ATP6V1B2|SNX5|VPS35|ZMPSTE24|RALB|RAB8A|BNIP3|HMGB1|GAPDH|VDAC1|ROCK1|FYCO1|SEC22B|LAMTOR1|CAPN1|CAPNS1|PHB2|CISD2|GBA|NEDD4|SIRT2|EEF1A1|EEF1A2|MTDH|EIF4G1|LAMTOR2|HSPB1|LAMTOR5|SCFD1|DNM1L|PRKAA2|PRKAG1|CTSA|PSAP</td></tr>
<tr><td>Metabolism</td><td>REACTOME_RESPIRATORY_ELECTRON_TRANSPORT</td><td>0.4635</td><td>1.4689</td><td>0.0204</td><td>58</td><td>ECSIT|TMEM186|ETFA|ETFB|NDUFS4|ETFDH|ACAD9|COQ10A|NDUFAB1|NDUFA11|NDUFAF6|NDUFAF3|NDUFAF4|NDUFS7|NDUFA3|NDUFA6|NDUFA8|NDUFA10|NDUFB1|NDUFB3|NDUFC2|NDUFB4|NDUFB5|NDUFB6|NDUFB7|NDUFB8|NDUFB9|NDUFB10|NDUFS1|NDUFS2|NDUFV1|NDUFS5|NDUFS8|SDHA|NDUFV2|SDHB|NDUFV3|NDUFAF1|TIMMDC1|NDUFA2|NDUFB11|NDUFA12|UQCRC1|SDHC|SDHD|NDUFA5|NDUFS3|NDUFAF5|COX7A2L|COX7C|SCO1|COX16|SURF1|NDUFA4|TRAP1|CYCS|NDUFA7|NDUFA9</td></tr>
<tr><td>Metabolism</td><td>REACTOME_METABOLISM_OF_LIPIDS</td><td>0.4215</td><td>1.4056</td><td>0.0244</td><td>105</td><td>ACOT2|MGLL|CPT1B|PLBD1|CRAT|ECI1|DECR1|MMAA|ACSF3|DBI|GPD1L|ACSL6|GPD1|GPD2|PI4K2A|ESYT2|FDXR|AGK|MCAT|BDH1|HADHA|HADHB|HADH|HMGCL|PPT1|HSD17B4|ACADL|ACADS|ACADVL|GGT5|ACADM|ARF1|NUDT19|SLC27A1|ASAH1|NDUFAB1|OSBP|PNPLA8|PCCA|PCCB|ACOX1|ACAA2|AKR1B1|MECR|PHYH|PON2|SLC25A1|PECR|HEXB|SCP2|PRKACA|SAR1B|HSD17B8|RAB5A|MLYCD|PTGES2|OXCT1|ACSF2|ACAD10|GNPAT|CAV1|MCEE|VAPB|CBR4|CBR1|HACD1|TECR|KPNB1|FITM1|SACM1L|RAN|PISD|LCLAT1|GBA|GPX1|ACAT1|ACOT9|ACOT13|AGPS|FABP4|PRKAA2|HACD3|PTPMT1|DHRS7B|VAPA|FABP3|ACSL1|CD36|GPAM|LPCAT3|RAB14|GPX4|CTSA|PSAP|SEC23A|ECHS1|CSNK2A1|AGPAT3|APOA1|SLC44A2|ALDH3A2|ALB|PCYT1A|ACOT8|ECI2</td></tr>
<tr><td>Metabolism</td><td>GOBP_GLUCOSE_CATABOLIC_PROCESS</td><td>-0.7282</td><td>-1.58</td><td>0.0182</td><td>13</td><td>TPI1|SLC25A12|ENO1|ENO3|ALDOA|GAPDH|GPI|HK1|HK2|PFKM|PGAM2|PGK1|PKM</td></tr>
<tr><td>Metabolism</td><td>GOBP_MONOSACCHARIDE_CATABOLIC_PROCESS</td><td>-0.7177</td><td>-1.7221</td><td>0</td><td>14</td><td>TPI1|SLC25A12|PGM1|ENO1|ENO3|ALDOA|GAPDH|GPI|HK1|HK2|PFKM|PGAM2|PGK1|PKM</td></tr>
<tr><td>Metabolism</td><td>KEGG_CARDIAC_MUSCLE_CONTRACTION</td><td>0.6147</td><td>1.4757</td><td>0.0488</td><td>17</td><td>COX7A2|TPM3|ATP1B3|ATP1B1|COX7A2L|TPM4|COX7C|UQCRC1|ATP1A1|ATP1A2|ATP2A2|TNNC1|MYL2|MYH7|TPM1|MYL3|CACNA2D1</td></tr>
<tr><td>Metabolism</td><td>KEGG_PARKINSONS_DISEASE</td><td>0.4445</td><td>1.4683</td><td>0.0185</td><td>47</td><td>SLC25A5|SLC25A4|NDUFB10|NDUFS1|NDUFS2|PPID|NDUFV1|NDUFS5|NDUFS8|SDHA|NDUFV2|PARK7|SDHB|NDUFV3|NDUFA2|UBA1|UQCRC1|SDHC|NDUFS4|SDHD|VDAC1|VDAC3|NDUFA5|COX7A2|NDUFS3|COX7A2L|COX7C|VDAC2|NDUFAB1|NDUFA4|CYCS|NDUFA7|NDUFS7|NDUFA3|NDUFA6|NDUFA9|NDUFA8|NDUFA10|NDUFB1|NDUFB3|NDUFC2|NDUFB4|NDUFB5|NDUFB6|NDUFB7|NDUFB8|NDUFB9</td></tr>
<tr><td>Metabolism</td><td>REACTOME_PHOSPHOLIPID_METABOLISM</td><td>0.5199</td><td>1.3376</td><td>0.0426</td><td>24</td><td>HADHA|PTPMT1|HADHB|MGLL|SACM1L|PISD|PLBD1|LCLAT1|ARF1|SLC44A2|RAB5A|PNPLA8|GPD1L|CSNK2A1|AGPAT3|GPD1|GPD2|GPAM|PI4K2A|GNPAT|LPCAT3|RAB14|PCYT1A|AGK</td></tr>
<tr><td>Metabolism</td><td>GOBP_NUCLEOSIDE_PHOSPHATE_CATABOLIC_PROCESS</td><td>-0.7535</td><td>-1.612</td><td>0</td><td>12</td><td>ENTPD2|MLYCD|VCP|GDA|CNP|GPX1|ACAT1|NT5E|PNP|PDE2A|ENPP3|XDH</td></tr>
<tr><td>Metabolism</td><td>GOBP_GLUCOSE_METABOLIC_PROCESS</td><td>-0.5824</td><td>-1.6237</td><td>0.0435</td><td>33</td><td>SLC25A11|ZMPSTE24|BCKDK|ENO1|DLAT|ENO3|ALDOA|MLYCD|HMGB1|GAPDH|PDHB|GPD1|PDK4|GPI|HK1|HK2|PFKM|PGAM2|PGK1|PKM|TPI1|SLC25A12|PGM1|MDH1|MDH2|SLC25A13|SLC25A10|GOT1|GOT2|PC|PDK2|NLN|SLC25A1</td></tr>
<tr><td>Metabolism</td><td>GOBP_CARBOHYDRATE_METABOLIC_PROCESS</td><td>-0.4648</td><td>-1.4621</td><td>0.037</td><td>65</td><td>ENO1|ENO3|ALDOA|GPD1L|GAPDH|GPD1|GPD2|GPI|HK1|HK2|LDHA|PFKM|PGAM2|PGK1|PKM|TPI1|SLC25A12|PGM1|MDH1|MDH2|SLC25A13|CLTC|SLC25A10|AP2A1|AGL|AKR1B1|GOT1|GOT2|GYG1|GYS1|PC|PDK2|NLN|SLC25A1|UGP2|SLC25A11|HEXB|PYGM|PYGB|ZMPSTE24|BCKDK|DLAT|SLC2A4|MLYCD|HMGB1|PDHB|PDK4|CYB5A|LANCL1|APP|CS|PRKAA2|GANAB|PRKAG1|BPNT1|GLO1|IDH2|IDH3A|LDHB|COQ3|SLC3A2|SCARB2|CHID1|TPD52L2|MLEC</td></tr>
<tr><td>Metabolism</td><td>GOBP_NUCLEOBASE_CONTAINING_SMALL_MOLECULE_BIOSYNTHETIC_PROCESS</td><td>-0.6921</td><td>-1.5957</td><td>0</td><td>15</td><td>CNP|GPX1|ACAT1|PNP|PDE2A|ENPP3|XDH|ENTPD2|MLYCD|VCP|GDA|DHODH|APRT|NME2|NT5E</td></tr>
<tr><td>Metabolism</td><td>GOBP_LIPID_CATABOLIC_PROCESS</td><td>0.4421</td><td>1.4033</td><td>0</td><td>65</td><td>ILVBL|HINT2|MGLL|SMPDL3B|CPT1B|ABHD16A|PLBD1|CRAT|ECI1|DECR1|MMAA|FABP3|ECH1|ETFA|LPL|ETFB|ETFDH|GCDH|YWHAH|MCAT|APOE|HADHA|HADHB|HADH|PPT1|HSD17B4|ACADL|ACADS|ACADVL|ACADM|NUDT19|SIRT2|ASAH1|PNPLA8|PCCA|APOA4|PCCB|ACAA2|ACOX1|MECR|PHYH|ECHDC2|PECR|HSD17B10|HEXB|SCP2|PRDX6|ECHS1|IVD|AUH|MLYCD|APOA1|ACAD10|LRP1|RAB7A|MCEE|GBA|ACAT1|PLCB4|NCEH1|ALDH3A2|FABP4|ABCD3|ACOT8|ECI2</td></tr>
<tr><td>Metabolism</td><td>GOBP_DNA_METABOLIC_PROCESS</td><td>-0.5033</td><td>-1.4733</td><td>0.0179</td><td>37</td><td>CCT7|PPIA|PARK7|CCT6A|CCT2|RPS27A|ZMPSTE24|GNAS|PTPRC|CUL4A|PARP3|HMGB1|TCP1|CCT3|TFRC|NT5E|COPS8|DDX1|SLC30A9|FH|ANXA3|HSPD1|KPNB1|PHB|RAN|SUPV3L1|TEFM|HSP90AA1|EEPD1|LONP1|EXOG|RPS3|VCP|CCT4|HSP90AB1|CCT8|CCT5</td></tr>
</table>

<table>
<thead>
<tr><th></th><th></th><th></th><th></th><th></th><th></th><th></th></tr>
</thead>
<tbody>
<tr><td>Metabolism</td><td>REACTOME_RESPIRATORY_ELECTRON_TRANSPORT_ATP_SYNTHESIS_BY_CHEMIOSMOTIC_COUPLING_AND_HEAT_PRODUCTION_BY_UNCOUPLING_PROTEINS</td><td>0.4478</td><td>1.4728</td><td>0</td><td>59</td><td>ECSIT|TMEM186|ETFA|ETFB|NDUFS4|ETFDH|UCP3|ACAD9|COQ10A|NDUFAB1|NDUFA11|NDUFAF6|NDUFAF3|NDUFAF4|NDUFS7|NDUFA3|NDUFA6|NDUFA8|NDUFA10|NDUFB1|NDUFB3|NDUFC2|NDUFB4|NDUFB5|NDUFB6|NDUFB7|NDUFB8|NDUFB9|NDUFB10|NDUFS1|NDUFS2|NDUFV1|NDUFS5|NDUFS8|SDHA|NDUFV2|SDHB|NDUFV3|NDUFAF1|TIMMDC1|NDUFA2|NDUFB11|NDUFA12|UQCRC1|SDHC|SDHD|NDUFA5|NDUFS3|NDUFAF5|COX7A2L|COX7C|SCO1|COX16|SURF1|NDUFA4|TRAP1|CYCS|NDUFA7|NDUFA9</td></tr>
<tr><td>Metabolism</td><td>REACTOME_MITOCHONDRIAL_FATTY_ACID_BETA_OXIDATION</td><td>0.5002</td><td>1.3997</td><td>0.0208</td><td>24</td><td>ACOT2|HADHA|HADHB|HADH|ACADL|ACADS|ACADVL|ECI1|DECR1|ACADM|ECHS1|MMAA|ACOT9|NDUFAB1|ACOT13|DBI|ACSF2|PCCA|PCCB|ACAA2|ACAD10|MECR|MCEE|MCAT</td></tr>
<tr><td>Metabolism</td><td>GOBP_MONOSACCHARIDE_METABOLIC_PROCESS</td><td>-0.5929</td><td>-1.5463</td><td>0</td><td>35</td><td>SLC25A11|ZMPSTE24|BCKDK|ENO1|DLAT|ENO3|ALDOA|MLYCD|HMGB1|GAPDH|PDHB|GPD1|PDK4|GPI|HK1|HK2|PFKM|PGAM2|PGK1|PKM|TPI1|SLC25A12|PGM1|MDH1|CYB5A|MDH2|SLC25A13|SLC25A10|AKR1B1|GOT1|GOT2|PC|PDK2|NLN|SLC25A1</td></tr>
<tr><td>Metabolism</td><td>GOBP_NAD_METABOLIC_PROCESS</td><td>-0.6716</td><td>-1.7252</td><td>0</td><td>20</td><td>TPI1|SLC25A12|NADK2|ENO1|ENO3|ALDOA|GPD1L|GAPDH|GPD1|GPD2|GPI|HK1|HK2|NMNAT3|LDHA|NT5E|PFKM|PGAM2|PGK1|PKM</td></tr>
<tr><td>Metabolism</td><td>GOBP_CARBOHYDRATE_CATABOLIC_PROCESS</td><td>-0.6048</td><td>-1.5249</td><td>0</td><td>26</td><td>HEXB|PYGM|PYGB|ENO1|ENO3|ALDOA|HMGB1|GAPDH|GPD1|GPD2|GPI|HK1|HK2|LDHA|PFKM|PGAM2|PGK1|PKM|TPI1|SLC25A12|PGM1|AGL|APP|PRKAA2|PRKAG1|SCARB2</td></tr>
<tr><td>Metabolism</td><td>REACTOME_METABOLISM_OF_CARBOHYDRATES</td><td>-0.5746</td><td>-1.6288</td><td>0.0172</td><td>40</td><td>UGP2|SLC25A11|DCN|HEXB|GPC1|PYGM|PRKACA|PYGB|OGN|LUM|RPS27A|BGN|CHP1|PRELP|ENO1|ENO3|ALDOA|GAPDH|GPI|HK1|HK2|PFKM|PGAM2|PGK1|PKM|TPI1|SLC25A12|PGM1|MDH1|MDH2|SLC25A13|SLC25A10|AGL|AKR1B1|GOT1|GOT2|GYG1|GYS1|PC|SLC25A1</td></tr>
<tr><td>Metabolism</td><td>REACTOME_GLYCOLYSIS</td><td>-0.6992</td><td>-1.605</td><td>0</td><td>13</td><td>TPI1|PRKACA|ENO1|ENO3|ALDOA|GAPDH|GPI|HK1|HK2|PFKM|PGAM2|PGK1|PKM</td></tr>
<tr><td>Metabolism</td><td>GOBP_RESPIRATORY_ELECTRON_TRANSPORT_CHAIN</td><td>0.405</td><td>1.2616</td><td>0.0392</td><td>55</td><td>PARK7|ETFA|ETFB|ETFDH|NDUFS4|GPD1|GPD2|COQ9|SLC25A12|NDUFA11|NDUFAB1|SLC25A13|NDUFS7|NDUFA3|NDUFA6|NDUFA8|NDUFA10|NDUFB1|NDUFB3|NDUFC2|NDUFB4|NDUFB5|NDUFB6|NDUFB7|GHITM|NDUFB8|NDUFB9|NDUFB10|NDUFS1|NDUFS2|NDUFV1|NDUFS5|DLD|NDUFS8|NDUFV2|SDHA|NDUFV3|SDHB|NDUFAF1|NDUFA2|NDUFB11|NDUFA12|UQCRC1|SDHC|SDHD|NDUFA5|SDHAF2|NDUFS3|COX7A2L|ISCU|COX7C|NDUFA4|CYCS|NDUFA7|NDUFA9</td></tr>
<tr><td>Metabolism</td><td>REACTOME_GLUCOSE_METABOLISM</td><td>-0.6205</td><td>-1.5451</td><td>0.0196</td><td>23</td><td>TPI1|SLC25A11|SLC25A12|PRKACA|MDH1|ENO1|ENO3|MDH2|ALDOA|SLC25A13|GAPDH|SLC25A10|GPI|HK1|GOT1|HK2|GOT2|PFKM|PC|PGAM2|PGK1|PKM|SLC25A1</td></tr>
<tr><td>Metabolism</td><td>GOBP_CARBOHYDRATE_BIOSYNTHETIC_PROCESS</td><td>-0.6123</td><td>-1.6454</td><td>0</td><td>29</td><td>UGP2|SLC25A11|ENO1|ENO3|ALDOA|GAPDH|GPD1|GPI|PGAM2|PGK1|TPI1|SLC25A12|PGM1|MDH1|MDH2|SLC25A13|CLTC|SLC25A10|AP2A1|AGL|AKR1B1|GOT1|GOT2|GYG1|GYS1|PC|PDK2|NLN|SLC25A1</td></tr>
<tr><td>Metabolism</td><td>GOBP_NADH_METABOLIC_PROCESS</td><td>-0.7133</td><td>-1.6892</td><td>0</td><td>18</td><td>TPI1|SLC25A12|ENO1|MDH1|ENO3|MDH2|ALDOA|VCP|GAPDH|GPD1|GPD2|GPI|HK1|HK2|PFKM|PGAM2|PGK1|PKM</td></tr>
<tr><td>Organelle organization</td><td>GOBP_NEGATIVE_REGULATION_OF_ACTIN_FILAMENT_POLYMERIZATION</td><td>-0.7765</td><td>-1.6779</td><td>0</td><td>11</td><td>ADD1|ADD2|DMTN|RDX|SPTAN1|TMOD1|TMSB4X|CAPZA1|CAPZA2|CAPZB|MYADM</td></tr>
<tr><td>Organelle organization</td><td>GOBP_REGULATION_OF_ORGANELLE_ORGANIZATION</td><td>-0.4382</td><td>-1.4819</td><td>0</td><td>120</td><td>CCT7|WASF2|ADD1|ADD2|DMTN|RDX|SPTAN1|CCT6A|TMOD1|TMSB4X|CCT2|CAPZA1|CAPZA2|CAPZB|CAV3|MYADM|ENO1|ANXA1|ENO3|KIF5B|PRKAR1A|ARPC3|ARPC1B|ACTR3|DCTN1|DLG1|ARF1|MYO1C|RAC1|RPS3|PDCD6IP|CLTC|PPIF|ACAA2|VAT1|CCT4|CCT8|TAPT1|CCT5|FXN|ATL3|FAM162A|GHITM|ANK1|SLC25A5|ANXA2|DCN|IRGM|LCP1|MSN|NMT1|FIS1|SH3GLB1|SAR1B|TMED9|TMEM33|PARL|VPS35|RAB3A|RAB5A|RALB|SLC2A4|BNIP3|SSBP1|STXBP1|TCP1|CCT3|CDC42|TFRC|VDAC1|LMAN1|VDAC3|RAB7A|FYCO1|MUL1|CALR|RAB1B|MCU|ARHGDIA|SEC22B|RHOG|CFL2|DSTN|ACTN2|WDR1|GPX1|PDE2A|DHODH|PRKAA2|SCARB2|MAVS|PARK7|YWHAE|YWHAH|YWHAQ|YWHAZ|YWHAB|YWHAG|SIRT2|RHOA|DNM1L|DNM2|OPA1|PREB|FERMT2|S100A10|PARP3|FLNA|CLIC4|HRG|APOA1|LRP1|ROCK1|FSCN1|TPM1|CAPN2|ROCK2|CD47|CTNNB1|SCFD1</td></tr>
<tr><td>Organelle organization</td><td>GOBP_REGULATION_OF_ANATOMICAL_STRUCTURE_SIZE</td><td>-0.4664</td><td>-1.4413</td><td>0.0484</td><td>58</td><td>WASF2|ADD1|ADD2|DMTN|RDX|SPTAN1|FGA|TMOD1|FGB|TMSB4X|FGG|CAPZA1|CAPZA2|CAPZB|CAV3|MYADM|NOS3|ATP2B1|AP2M1|DPYSL2|ANO6|RAB21|FN1|APOE|GOLGA4|AQP1|RTN4|SCPEP1|IST1|DEPTOR|SNTA1|ARPC3|SOD2|ARPC1B|ACTR3|DLG1|ARF1|MYO1C|RAC1|RHOA|LAMTOR5|DNM2|MSN|GDI1|PICALM|RAB5A|LRP1|ROCK1|CAV1|ROCK2|CFL2|DSTN|ACTN2|WDR1|GPX1|SOD1|ATP1A2|HSP90AB1</td></tr>
<tr><td>Organelle organization</td><td>GOBP_NEGATIVE_REGULATION_OF_PROTEIN_POLYMERIZATION</td><td>-0.6915</td><td>-1.5098</td><td>0.037</td><td>12</td><td>ADD1|ADD2|DMTN|RDX|SPTAN1|TMOD1|TMSB4X|CAPZA1|CAPZA2|CAPZB|MYADM|VDAC2</td></tr>
<tr><td>Organelle organization</td><td>GOBP_REGULATION_OF_PROTEIN_DEPOLYMERIZATION</td><td>-0.6843</td><td>-1.6146</td><td>0.0222</td><td>13</td><td>ADD1|ADD2|DMTN|CFL2|RDX|DSTN|SPTAN1|TMOD1|ACTN2|WDR1|CAPZA1|CAPZA2|CAPZB</td></tr>
<tr><td>Organelle organization</td><td>GOBP_REGULATION_OF_ACTIN_FILAMENT_LENGTH</td><td>-0.6372</td><td>-1.5379</td><td>0.0196</td><td>22</td><td>WASF2|ADD1|ARPC3|ADD2|ARPC1B|DMTN|ACTR3|CFL2|RDX|DSTN|SPTAN1|DLG1|TMOD1|ACTN2|TMSB4X|WDR1|ARF1|CAPZA1|MYO1C|CAPZA2|CAPZB|MYADM</td></tr>
<tr><td>Organelle organization</td><td>GOBP_ACTIN_FILAMENT_DEPOLYMERIZATION</td><td>-0.6501</td><td>-1.4948</td><td>0.0444</td><td>14</td><td>ADD1|ADD2|DMTN|CFL2|RDX|DSTN|SPTAN1|TMOD1|ACTN2|WDR1|CAPZA1|CAPZA2|CAPZB|CFL1</td></tr>
<tr><td>Organelle organization</td><td>GOBP_MITOCHONDRIAL_MEMBRANE_ORGANIZATION</td><td>0.4537</td><td>1.3451</td><td>0.0263</td><td>45</td><td>SLC25A5|APOOL|ROMO1|NMT1|IMMT|SAMM50|MTX1|BNIP3|MFN2|MFN1|HSPA9|HK2|MUL1|CHCHD6|YWHAE|HSPA4|YWHAH|TIMM13|AGK|YWHAQ|TIMM10|TIMM9|LETM1|TOMM22|YWHAZ|YWHAB|YWHAG|HSP90AA1|CNP|VDAC2|DNAJC11|CALM3|CHCHD3|MTX2|TOMM70|OXA1L|TMEM11|PPIF|APOO|ACAA2|AFG3L2|OPA1|TIMM50|GHITM|SPG7</td></tr>
<tr><td>Organelle organization</td><td>GOBP_PROTEIN_POLYMERIZATION</td><td>-0.554</td><td>-1.567</td><td>0.0417</td><td>34</td><td>WASF2|ADD1|ADD2|DMTN|RDX|SPTAN1|FGA|TMOD1|FGB|TMSB4X|FGG|CAPZA1|CAPZA2|CAPZB|CAV3|MYADM|ARPC3|ARPC1B|ACTR3|DCTN1|DLG1|HSP90AA1|ARF1|MYO1C|TPPP3|RAC1|RPS3|VDAC2|DNM1L|DNM2|COBL|GPX4|OPA1|CASQ2</td></tr>
</tbody>
</table>

<table>
<tr><th></th><th></th><th></th><th></th><th></th><th></th><th></th><th></th></tr>
<tr><td>Organelle organization</td><td>GOBP_REGULATION_OF_CYTOSKELETON_ORGANIZATION</td><td>-0.5021</td><td>-1.5605</td><td>0</td><td>46</td><td>WASF2|ADD1|FERMT2|ADD2|DMTN|RDX|SPTAN1|TMOD1|TMSB4X|CAPZA1|CAPZA2|S100A10|CAPZB|CAV3|MYADM|PARP3|FLNA|CLIC4|HRG|CDC42|APOA1|LRP1|ROCK1|FSCN1|TPM1|CAPN2|ROCK2|ARHGDIA|CD47|RHOG|ARPC3|ARPC1B|ACTR3|CFL2|DSTN|DCTN1|DLG1|ACTN2|WDR1|ARF1|MYO1C|RAC1|RHOA|RPS3|CLTC|PRKAA2</td></tr>
<tr><td>Organelle organization</td><td>GOBP_PROTEIN_DEPOLYMERIZATION</td><td>-0.6185</td><td>-1.5195</td><td>0.0435</td><td>16</td><td>ADD1|ADD2|DMTN|CFL2|RDX|DSTN|SPTAN1|TMOD1|ACTN2|WDR1|CAPZA1|CAPZA2|CAPZB|CFL1|HSPA8|VPS4A</td></tr>
<tr><td>Organelle organization</td><td>GOBP_ACTIN_POLYMERIZATION_OR_DEPOLYMERIZATION</td><td>-0.5831</td><td>-1.4519</td><td>0.0484</td><td>25</td><td>WASF2|ADD1|ADD2|DMTN|RDX|SPTAN1|TMOD1|TMSB4X|CAPZA1|CAPZA2|CAPZB|MYADM|CFL1|ARPC3|ARPC1B|ACTR3|CFL2|DSTN|DLG1|ACTN2|WDR1|ARF1|MYO1C|RAC1|COBL</td></tr>
<tr><td>Organelle organization</td><td>GOBP_MEMBRANE_FUSION</td><td>0.5623</td><td>1.6361</td><td>0</td><td>32</td><td>ANXA2|VTI1B|PPIA|VAPA|STX4|STX7|CHP1|RAB3A|ANXA1|RAB8A|TAPBP|NAPA|STXBP1|TAP1|KIF5B|MFN1|MYOF|RAB7A|CALR|ANXA7|SEC22B|CD9|STX12|NAPG|NSFL1C|SNAP23|DNM1L|DNM2|OPA1|VPS4A|VAMP7|EEA1</td></tr>
<tr><td>Organelle organization</td><td>GOBP_REGULATION_OF_SUPRAMOLECULAR_FIBER_ORGANIZATION</td><td>-0.4732</td><td>-1.3706</td><td>0.0377</td><td>42</td><td>WASF2|ADD1|FERMT2|ADD2|DMTN|RDX|PARK7|SPTAN1|TMOD1|TMSB4X|CAPZA1|CAPZA2|S100A10|CAPZB|CAV3|MYADM|FLNA|CDC42|APOA1|ROCK1|HSPA8|TPM1|ROCK2|APOE|CD47|ARPC3|ARPC1B|ACTR3|CFL2|DSTN|DCTN1|DLG1|ACTN2|WDR1|ARF1|GPX1|MYO1C|RAC1|RHOA|RPS3|APP|CRYAB</td></tr>
<tr><td>Organelle organization</td><td>GOBP_NEGATIVE_REGULATION_OF_CYTOSKELETON_ORGANIZATION</td><td>-0.766</td><td>-1.7382</td><td>0</td><td>13</td><td>WASF2|ADD1|ADD2|DMTN|RDX|SPTAN1|TMOD1|TMSB4X|CAPZA1|CAPZA2|CAPZB|CAV3|MYADM</td></tr>
<tr><td>Organelle organization</td><td>GOBP_REGULATION_OF_PROTEIN_POLYMERIZATION</td><td>-0.65</td><td>-1.7003</td><td>0</td><td>25</td><td>WASF2|ADD1|ADD2|DMTN|RDX|SPTAN1|TMOD1|TMSB4X|CAPZA1|CAPZA2|CAPZB|CAV3|MYADM|ARPC3|ARPC1B|ACTR3|DCTN1|DLG1|HSP90AA1|ARF1|MYO1C|TPPP3|RAC1|RPS3|VDAC2</td></tr>
<tr><td>Organelle organization</td><td>REACTOME_CRISTAE_FORMATION</td><td>0.6683</td><td>1.5378</td><td>0</td><td>11</td><td>DNAJC11|SAMM50|IMMT|CHCHD3|MTX1|MTX2|APOOL|TMEM11|APOO|HSPA9|CHCHD6</td></tr>
<tr><td>Organelle organization</td><td>GOBP_LOCALIZATION_WITHIN_MEMBRANE</td><td>0.7195</td><td>1.5813</td><td>0.0192</td><td>12</td><td>PTPRC|FLNA|NAXE|DLG1|RALA|DNAJA3|ATP1B1|CD81|SYNJ2BP|RAC1|APOE|CDH13</td></tr>
<tr><td>Others</td><td>GOBP_DEPHOSPHORYLATION</td><td>0.5123</td><td>1.5008</td><td>0</td><td>33</td><td>PTPMT1|PPIA|HSP90B1|PGAM5|CHP1|GNAI2|LGALS3|CAMK2G|PTPRC|ROCK1|PPTC7|NT5E|YWHAE|PPA2|ROCK2|MYO1D|SDHAF2|PLPP7|MINPP1|SACM1L|YWHAB|DLG1|GBA|CALM3|FKBP1A|CYCS|ATP1A1|DUSP3|ATP1A2|HSP90AB1|BPNT1|PDP1|TIMM50</td></tr>
<tr><td>Others</td><td>GOBP_MIDBRAIN_DEVELOPMENT</td><td>-0.642</td><td>-1.4545</td><td>0.0213</td><td>15</td><td>CTNNB1|NDUFS3|CKB|CNP|SIRT2|GLUD1|HSPA5|RHOA|MAOB|NDRG2|CALM3|CDC42|YWHAE|YWHAH|YWHAQ</td></tr>
<tr><td>Others</td><td>GOBP_PROTEIN_DEPHOSPHORYLATION</td><td>0.5706</td><td>1.5009</td><td>0.0408</td><td>25</td><td>PTPMT1|PPIA|HSP90B1|PGAM5|GNAI2|LGALS3|CAMK2G|PTPRC|ROCK1|PPTC7|YWHAE|PPA2|ROCK2|SDHAF2|MINPP1|YWHAB|DLG1|GBA|CALM3|FKBP1A|CYCS|DUSP3|HSP90AB1|PDP1|TIMM50</td></tr>
<tr><td>Others</td><td>GOBP_NEURAL_NUCLEUS_DEVELOPMENT</td><td>-0.6693</td><td>-1.5753</td><td>0</td><td>14</td><td>NDUFS3|CKB|CNP|SIRT2|GLUD1|HSPA5|RHOA|MAOB|NDRG2|CALM3|CDC42|YWHAE|YWHAH|YWHAQ</td></tr>
<tr><td>Others</td><td>GOBP_SUBSTANTIA_NIGRA_DEVELOPMENT</td><td>-0.6693</td><td>-1.5234</td><td>0.0455</td><td>14</td><td>NDUFS3|CKB|CNP|SIRT2|GLUD1|HSPA5|RHOA|MAOB|NDRG2|CALM3|CDC42|YWHAE|YWHAH|YWHAQ</td></tr>
<tr><td>Others</td><td>REACTOME_SIGNALING_BY_RHO_GTPASES_MIRO_GTPASES_AND_RHOBTB3</td><td>-0.4663</td><td>-1.5202</td><td>0</td><td>81</td><td>CCT7|CKAP4|WASF2|HINT2|FLOT1|DBT|TPM3|CCT6A|SPTAN1|STOM|OBSCN|CCT2|TPM4|CAPZB|STIP1|BCAP31|MYLK|MFN2|TUBB2A|KIF5B|MFN1|CFL1|TUBA8|TUBB4B|YWHAE|YWHAH|YWHAQ|YWHAZ|ARPC3|ARPC1B|YWHAB|ACTR3|YWHAG|HSP90AA1|CKB|RAC1|RHOA|VCP|CLTC|MYL6|MYH14|MYH11|FERMT2|PICALM|SAMM50|MTX1|FLNA|CDC42|TFRC|LMAN1|ROCK1|RAB7A|CAV1|VAPB|KTN1|NDUFA5|ROCK2|ARHGDIA|LAMTOR1|ITGB1|LETM1|CTNNB1|ARHGAP1|RHOG|SHMT2|NDUFS3|VIM|ACTN1|ALDH3A2|NSFL1C|SCFD1|SNAP23|HSP90AB1|C1QBP|STEAP3|ABCD3|GNA13|EMC3|AKAP12|FLOT2|RRAS2</td></tr>
<tr><td>Others</td><td>REACTOME_RAC2_GTPASE_CYCLE</td><td>-0.6791</td><td>-1.5609</td><td>0.0377</td><td>13</td><td>WASF2|ARHGAP1|SAMM50|MTX1|CDC42|TFRC|LMAN1|RAB7A|CAV1|VAPB|ARHGDIA|LAMTOR1|ITGB1</td></tr>
<tr><td>Others</td><td>GOBP_POSITIVE_REGULATION_OF_PROTEIN_PHOSPHORYLATION</td><td>-0.4559</td><td>-1.4309</td><td>0.0172</td><td>55</td><td>HACD3|PPIA|FLOT1|MAVS|PARK7|PTPRC|PRDX2|DAG1|PRKAR1A|ERP29|PRKAR2A|PHB2|ACSL1|DLG1|EHD4|HPX|HSP90AA1|ITGB3|SLC27A1|PECAM1|PRNP|RAC1|RPLP1|RHOA|RPS3|C3|VLDLR|VTN|CALM3|FXR1|CD36|EIF4G1|LAMTOR2|CAB39|RACK1|IRGM|FERMT2|PRKACA|RAP1A|RPS27A|RALB|HMGB1|TFRC|MUL1|CAV1|ROCK2|COPS8|PHB|CD81|ILK|SOD1|APP|HSP90AB1|PRKAG1|DDX3X</td></tr>
<tr><td>Others</td><td>GOBP_POSITIVE_REGULATION_OF_PHOSPHORUS_METABOLIC_PROCESS</td><td>-0.4305</td><td>-1.3439</td><td>0.05</td><td>70</td><td>HACD3|PPIA|FLOT1|MAVS|PARK7|TMSB4X|FABP3|ITGA6|NOS3|ENO1|PTPRC|PRDX2|GPD1|DAG1|PRKAR1A|SLC25A12|ERP29|PRKAR2A|PHB2|ACSL1|DLG1|EHD4|HPX|HSP90AA1|ITGB3|SLC27A1|PECAM1|PRNP|RAC1|RPLP1|RHOA|RPS3|C3|VLDLR|VTN|CALM3|FXR1|VCP|CD36|EIF4G1|LAMTOR2|CAB39|RACK1|IRGM|FERMT2|PRKACA|RAP1A|RPS27A|CHP1|RALB|HMGB1|CDC42|TFRC|MUL1|CAV1|CAPN2|ROCK2|COPS8|PHB|GBA|CD81|ILK|EEF1A2|STOML2|SOD1|APP|HSP90AB1|PRKAA2|PRKAG1|DDX3X</td></tr>
<tr><td>Others</td><td>REACTOME_RHO_GTPASE_CYCLE</td><td>-0.4921</td><td>-1.5442</td><td>0.0154</td><td>59</td><td>CCT7|CKAP4|WASF2|HINT2|FLOT1|DBT|TPM3|CCT6A|SPTAN1|STOM|OBSCN|CCT2|TPM4|CAPZB|STIP1|BCAP31|HSP90AA1|CKB|RAC1|RHOA|VCP|CLTC|FERMT2|PICALM|SAMM50|MTX1|CDC42|TFRC|LMAN1|ROCK1|RAB7A|CAV1|VAPB|KTN1|NDUFA5|ROCK2|ARHGDIA|LAMTOR1|ITGB1|LETM1|ARHGAP1|RHOG|SHMT2|NDUFS3|VIM|ACTN1|ALDH3A2|NSFL1C|SCFD1|SNAP23|HSP90AB1|C1QBP|STEAP3|ABCD3|GNA13|EMC3|AKAP12|FLOT2|RRAS2</td></tr>
<tr><td>Others</td><td>REACTOME_RHOG_GTPASE_CYCLE</td><td>-0.6784</td><td>-1.6228</td><td>0.0364</td><td>16</td><td>LETM1|ARHGAP1|RHOG|SHMT2|NDUFS3|CDC42|TFRC|LMAN1|RAB7A|CAV1|VAPB|KTN1|NDUFA5|ARHGDIA|LAMTOR1|ITGB1</td></tr>
<tr><td>Others</td><td>GOBP_POSITIVE_REGULATION_OF_GTPASE_ACTIVITY</td><td>-0.6559</td><td>-1.5626</td><td>0</td><td>20</td><td>PREB|RACK1|ARHGAP1|HACD3|SEC23A|RHOG|FERMT2|PGAM5|CORO1C|GDI1|GDI2|GNAQ|LIMS1|RAP1A|ITGA6|S100A10|PICALM|DNM1L|ARHGDIA|ITGB1</td></tr>
<tr><td>Signaling</td><td>GOBP_POSITIVE_REGULATION_OF_CHEMOTAXIS</td><td>0.674</td><td>1.5415</td><td>0.04</td><td>11</td><td>HMGB1|HSPB1|STX4|DNM1L|C1QBP|TMSB4X|CALR|ANO6|RAC1|MCU|CDH13</td></tr>
<tr><td>Signaling</td><td>REACTOME_MTOR_SIGNALLING</td><td>-0.7048</td><td>-1.5748</td><td>0</td><td>10</td><td>FKBP1A|EIF4G1|LAMTOR2|LAMTOR5|YWHAB|CAB39|RPS6|PRKAA2|PRKAG1|LAMTOR1</td></tr>
</table>

<table>
<tr><td>Signaling</td><td>GOBP_IMMUNE_EFFECTOR_PROCESS</td><td>-0.3742</td><td>-1.3068</td><td>0.0357</td><td>156</td><td>WASF2|CKAP4|SPTAN1|STOM|CCT2|ANXA1|PTPRC|ALDOA|GPI|HK1|TUBB4B|HSPA8|PKM|IST1|PGM1|ARPC3|ARPC1B|ACTR3|HSP90AA1|MYO1C|ASAH1|RAC1|FCER1G|VCP|TOMM70|VAT1|AGL|CCT8|GYG1|ANXA2|NIT2|HEXB|LCP1|PYGB|RAB3A|ZMPSTE24|BNIP3|HMGB1|PTGES2|STXBP1|CDC42|TFRC|RAB7A|MUL1|LAMTOR1|DDX1|ANXA3|AIMP1|HSPD1|RHOG|KPNB1|PHB|EEF2|WDR1|PNP|RPS19|ENPP3|APRT|NME2|APP|MVP|ABCC9|DDX3X|LGALS1|MLEC|ATP6V0A1|PPIA|MAVS|VAPA|STX4|STX7|RAB10|AHSG|CTSC|GSTP1|ITGAV|LGALS3|PRDX2|STXBP3|CAT|B2M|PSMA2|PSMD11|ANO6|PSMA5|PSMC2|PSMC3|PHB2|CAP1|PSMD1|PRDX4|PSMD2|CST3|HPX|PSMD13|CTSB|CTSD|PECAM1|AP2A2|DDOST|EEF1A1|RHOA|C3|RAB18|ERP44|VTN|DNAJC13|METTL7A|CD36|ANPEP|LAMTOR2|DPP7|CFH|HP|CAB39|IGF2R|GPAM|RPS6|LAMP1|LAMP2|NDUFC2|DNAJC3|RAB14|CTSA|PSAP|RAB5B|RAB5C|VAMP7|C4BPA|C9|VCL|NLRX1|TXNDC5|CLPB|MAGT1|GDI2|SCAMP1|PRDX6|CD59|RAP1A|RAP1B|PARP3|FLNA|APOA1|LRP1|ROCK1|CD47|CAPN1|CAND1|CD81|PA2G4|SLC44A2|ABCE1|SNAP23|HSP90AB1|C1QBP</td></tr>
<tr><td>Signaling</td><td>GOBP_REGULATION_OF_CHEMOTAXIS</td><td>0.6298</td><td>1.4867</td><td>0</td><td>16</td><td>STX4|TMSB4X|GSTP1|RAC1|USP14|DPP4|HMGB1|DUSP3|HSPB1|HRG|DNM1L|C1QBP|CALR|ANO6|MCU|CDH13</td></tr>
<tr><td>Signaling</td><td>GOBP_CELLULAR_RESPONSE_TO_INSULIN_STIMULUS</td><td>-0.4919</td><td>-1.4088</td><td>0.04</td><td>29</td><td>ATP6V0A1|ATP6V0D1|ATP6V1H|ATP6V1A|ATP6V1E1|ATP6V1B2|RAB10|AHSG|RAB12|GSTP1|SNX5|GNAI2|GRB10|IDE|LPL|SLC2A4|RAB8A|ATP2B1|TRIM72|PDK4|PKM|YWHAG|CSRP3|SLC27A1|MYO1C|APRT|GOT1|OPA1|PDK2</td></tr>
<tr><td>Signaling</td><td>GOBP_RESPONSE_TO_INSULIN</td><td>-0.4976</td><td>-1.4546</td><td>0.0192</td><td>34</td><td>ATP6V0A1|ATP6V0D1|ATP6V1H|ATP6V1A|ATP6V1E1|ATP6V1B2|RAB10|AHSG|RAB12|GSTP1|FABP3|SNX5|GNAI2|GRB10|IDE|LPL|SLC2A4|RAB8A|ATP2B1|TRIM72|PDK4|STXBP3|UCP3|CAT|PKM|YWHAG|CSRP3|SLC27A1|MYO1C|APRT|GOT1|GOT2|OPA1|PDK2</td></tr>
<tr><td>Signaling</td><td>GOBP_IMMUNE_SYSTEM_DEVELOPMENT</td><td>-0.4277</td><td>-1.3475</td><td>0.05</td><td>67</td><td>WASF2|ADD1|ADD2|DMTN|FBN1|LGALS3|GNAS|ANXA1|PF4|PTPRC|PRDX2|PSMA4|CUL4A|PSMC5|PSME1|PSMC6|PSMA3|PSMC1|HSPA9|PSMA6|ITGA2B|PSMA7|B2M|PSMA2|PSMD11|PSMA1|PSMA5|PSMD4|PSMC2|PSMC3|PSMD1|PSMD2|PSMD13|FCER1G|RPS6|DNAJA3|ANXA2|PICALM|ZMPSTE24|PARP3|HMGB1|CDC42|TFRC|ITGB1|OCIAD1|HSPD1|PRDX3|EEF2|EPB42|GBA|SBDS|CD81|WDR1|GLRX5|BVES|RPL22|RPS14|RRAS|PNP|RPS19|CD34|SOD1|ACTN1|NME2|APP|GLO1|LGALS1</td></tr>
<tr><td>Signaling</td><td>REACTOME_APOPTOSIS</td><td>-0.4847</td><td>-1.4681</td><td>0.037</td><td>39</td><td>ADD1|NMT1|SPTAN1|RPS27A|BCAP31|PSMA4|PSMC5|HMGB1|PSME1|PSMC6|PSMA3|PSMC1|PSMA6|ROCK1|PSMA7|PSMA2|PSMD11|YWHAE|YWHAH|PSMA1|YWHAQ|PSMA5|PSMD4|PSMC2|CTNNB1|YWHAZ|KPNB1|PSMC3|YWHAB|YWHAG|PSMD1|PSMD2|PSMD13|LMNA|VIM|CYCS|DNM1L|C1QBP|OPA1</td></tr>
<tr><td>Signaling</td><td>GOBP_NEGATIVE_REGULATION_OF_WOUND_HEALING</td><td>0.7179</td><td>1.6522</td><td>0.0179</td><td>12</td><td>PLG|APOH|CD9|HRG|FGA|FGB|FGG|TSPAN8|CD34|APOE|NOS3|VTN</td></tr>
<tr><td>Signaling</td><td>REACTOME_FC_EPSILON_RECEPTOR_FCERI_SIGNALING</td><td>-0.5342</td><td>-1.307</td><td>0.037</td><td>22</td><td>PSMD4|PSMC2|SKP1|PSMC3|PSMD1|PSMD2|PSMD13|RPS27A|RAC1|PSMA4|FCER1G|PSMC5|PSME1|PSMC6|PSMA3|PSMC1|PSMA6|PSMA7|PSMA2|PSMD11|PSMA1|PSMA5</td></tr>
<tr><td>Signaling</td><td>GOBP_FC_RECEPTOR_SIGNALING_PATHWAY</td><td>-0.4874</td><td>-1.3823</td><td>0.0377</td><td>32</td><td>WASF2|RAP1A|PTPRC|PSMA4|PSMC5|PSME1|PSMC6|PSMA3|CDC42|PSMC1|PSMA6|PSMA7|PSMA2|PSMD11|PSMA1|CD47|PSMA5|PSMD4|PSMC2|SKP1|ARPC3|PSMC3|ARPC1B|ACTR3|PSMD1|PSMD2|HSP90AA1|PSMD13|MYO1C|RAC1|FCER1G|HSP90AB1</td></tr>
<tr><td>Signaling</td><td>REACTOME_HEDGEHOG_OFF_STATE</td><td>-0.5338</td><td>-1.5045</td><td>0.0179</td><td>27</td><td>PRKACA|RPS27A|GNAS|PSMA4|PSMC5|PSME1|PSMC6|TUBB2A|PSMA3|PSMC1|PSMA6|PSMA7|PSMA2|TUBB4B|PSMD11|PSMA1|PSMA5|PSMD4|PRKAR1A|PSMC2|SKP1|CSNK1A1|PSMC3|PRKAR2A|PSMD1|PSMD2|PSMD13</td></tr>
<tr><td>Signaling</td><td>GOBP_NEGATIVE_REGULATION_OF_COAGULATION</td><td>0.7166</td><td>1.6431</td><td>0.0182</td><td>13</td><td>PLG|ANXA5|CD9|FGA|FGB|FGG|CD34|NOS3|VTN|APOH|HRG|TSPAN8|APOE</td></tr>
<tr><td>Signaling</td><td>REACTOME_G2_M_CHECKPOINTS</td><td>-0.5827</td><td>-1.5732</td><td>0.0192</td><td>25</td><td>RPS27A|PSMA4|PSMC5|PSME1|PSMC6|PSMA3|PSMC1|PSMA6|PSMA7|PSMA2|PSMD11|YWHAE|YWHAH|PSMA1|YWHAQ|PSMA5|PSMD4|PSMC2|YWHAZ|PSMC3|YWHAB|YWHAG|PSMD1|PSMD2|PSMD13</td></tr>
<tr><td>Signaling</td><td>GOBP_NEGATIVE_REGULATION_OF_CELL_ADHESION</td><td>-0.5242</td><td>-1.3076</td><td>0.04</td><td>26</td><td>DMTN|CORO1C|RDX|LGALS3|MYADM|ANXA1|PTPRC|PRDX2|HMGB1|HRG|APOA1|LRP1|ARHGDIA|CDH13|PRKAR1A|PLG|TGFBI|CD9|DLG1|PRNP|PLXNB2|ACTN4|RHOA|DUSP3|MIA3|LGALS1</td></tr>
<tr><td>Signaling</td><td>GOBP_NEGATIVE_REGULATION_OF_RESPONSE_TO_WOUNDING</td><td>0.7179</td><td>1.7405</td><td>0</td><td>12</td><td>PLG|APOH|CD9|HRG|FGA|FGB|FGG|TSPAN8|CD34|APOE|NOS3|VTN</td></tr>
<tr><td>Signaling</td><td>REACTOME_CELL_CYCLE_CHECKPOINTS</td><td>-0.5827</td><td>-1.5805</td><td>0</td><td>25</td><td>RPS27A|PSMA4|PSMC5|PSME1|PSMC6|PSMA3|PSMC1|PSMA6|PSMA7|PSMA2|PSMD11|YWHAE|YWHAH|PSMA1|YWHAQ|PSMA5|PSMD4|PSMC2|YWHAZ|PSMC3|YWHAB|YWHAG|PSMD1|PSMD2|PSMD13</td></tr>
<tr><td>Transportation</td><td>GOBP_NEUROTRANSMITTER_SECRETION</td><td>0.702</td><td>1.6269</td><td>0</td><td>13</td><td>STX4|RAP1A|RAB3A|RAB5A|RAP1B|CALM3|NAPA|STXBP1|ATP2A2|STXBP3|SNAP23|DNM1L|HSPA8</td></tr>
<tr><td>Transportation</td><td>GOBP_CYTOSOLIC_TRANSPORT</td><td>0.5716</td><td>1.4172</td><td>0.0227</td><td>19</td><td>VTI1B|SNX2|MSN|RDX|DCTN1|SNX5|VPS35|RAB5A|DNAJC13|CLTC|FLNA|AP2A1|KIF5B|LAMP1|RAB7A|RAB14|RAB21|MYO1D|EEA1</td></tr>
<tr><td>Transportation</td><td>GOBP_SYNAPTIC_SIGNALING</td><td>0.4936</td><td>1.4245</td><td>0.0476</td><td>42</td><td>FLOT1|PARK7|PRKACA|STX4|GNAQ|ATAD1|RAP1A|GNB1|GNAI2|RAB3A|MPZ|RAB5A|RAP1B|RAB8A|NAPA|PSMC5|STXBP1|STXBP3|KIF5B|PACSIN2|VDAC1|HSPA8|DAG1|YWHAH|APOE|ITGB1|YWHAG|LAMA2|DLG1|CNP|ARF1|RPL22|PLCB4|CALM3|ATP1A2|ATP2A2|APP|SNAP23|DNM1L|SYNJ2BP|AKAP12|SYPL1</td></tr>
<tr><td>Transportation</td><td>GOBP_POSITIVE_REGULATION_OF_TRANSPORTER_ACTIVITY</td><td>0.6774</td><td>1.6753</td><td>0</td><td>15</td><td>ANK3|PARK7|ATP1B3|ACTN2|ATP1B1|TMSB4X|CHP1|ACTN4|CALM3|KIF5B|STIM1|DNM2|JPH2|GSTM2|CACNA2D1</td></tr>
<tr><td>Transportation</td><td>GOBP_POSITIVE_REGULATION_OF_TRANSMEMBRANE_TRANSPORT</td><td>0.6112</td><td>1.5369</td><td>0.0204</td><td>21</td><td>ANK3|ACSL1|ATP1B3|ACTN2|ATP1B1|TMSB4X|ARF1|RAP1A|CHP1|ACTN4|C3|CALM3|FLNA|KIF5B|STIM1|DNM2|ANO6|JPH2|GSTM2|ITGB1|CACNA2D1</td></tr>
<tr><td>Transportation</td><td>GOBP_REGULATION_OF_NEUROTRANSMITTER_LEVELS</td><td>0.5888</td><td>1.6026</td><td>0</td><td>23</td><td>FLOT1|PARK7|STX4|ALDH9A1|ITGB3|RAP1A|RAB3A|ALDH5A1|MAOB|RAB5A|RAP1B|MAOA|CALM3|COMT|NAPA|ATP1A2|STXBP1|ATP2A2|STXBP3|SNAP23|DNM1L|HSPA8|ITGB1</td></tr>
<tr><td>Transportation</td><td>GOBP_SYNAPTIC_VESICLE_EXOCYTOSIS</td><td>0.7731</td><td>1.6842</td><td>0</td><td>11</td><td>CALM3|NAPA|STXBP1|ATP2A2|STX4|SNAP23|DNM1L|RAP1A|RAB3A|RAB5A|RAP1B</td></tr>
<tr><td>Transportation</td><td>GOBP_POSITIVE_REGULATION_OF_CATION_TRANSMEMBRANE_TRANSPORT</td><td>0.6269</td><td>1.6158</td><td>0</td><td>17</td><td>ANK3|ATP1B3|ACTN2|ATP1B1|TMSB4X|ARF1|CHP1|ACTN4|CALM3|FLNA|KIF5B|STIM1|DNM2|ANO6|JPH2|GSTM2|CACNA2D1</td></tr>
</table>

**Transportation**	**GOBP_PROTEIN_IMPORT**	0.5931	1.6377	0.0217	31	ROMO1|MAVS|PEX14|CHP1|SAMM50|FLNA|HSPA8|RPL23|HSPA4|APOE|DNAJC19|TOMM22|HSPD1|KPNB1|GRPEL1|PHB2|RAN|HSP90AA1|TOMM40|IPO5|TOMM20|AIFM1|CD36|TIMM21|TOMM70|HSP90AB1|LAMP2|TIMM23|TIMM44|TOMM40L|TIMM50
**Transportation**	**GOBP_VESICLE_MEDIATED_TRANSPORT_IN_SYNAPSE**	0.6735	1.6609	0.0256	20	AP2B1|STX4|ATAD1|ITGB3|RAP1A|PICALM|RAB3A|RAB5A|RAP1B|SLC2A4|CALM3|RAB8A|NAPA|CANX|STXBP1|ATP2A2|SNAP23|DNM1L|DNM2|ROCK1
**Transportation**	**GOBP_SIGNAL_RELEASE**	0.4789	1.4138	0.0476	37	SLC25A4|SLC25A5|PARK7|STX4|FGA|FGB|SLC16A1|FGG|RAP1A|RAB3A|GNAS|RAB5A|CAMK2G|ANXA1|RAP1B|NAPA|OXCT1|STXBP1|STXBP3|KIF5B|LRP1|MPC2|HSPA8|SIRT3|MCU|UQCC2|AIMP1|HADH|GLUD1|RAC1|MAOB|CALM3|DPP4|ATP2A2|SNAP23|DNM1L|VAMP7
**Transportation**	**GOBP_NEUROTRANSMITTER_TRANSPORT**	0.6369	1.5323	0.0435	18	FLOT1|PARK7|STX4|ITGB3|RAP1A|RAB3A|RAB5A|RAP1B|CALM3|NAPA|ATP1A2|STXBP1|ATP2A2|STXBP3|SNAP23|DNM1L|HSPA8|ITGB1

Supplementary Table 7b. Gene-sets enriched in the 4-month-old heart samples

GS<br>follow link to MSigDB	GS DETAILS	SIZE	ES	NES	NOM p-val	FDR q-val	FWER p-val	RANK AT MAX	LEADING EDGE
REACTOME_TRANS_GOLGI_NETWORK_VESICLE_BUDDING	Details ...	24	0.6029296	2.19912	0	0.01280402	0.01	171	tags=29%, list=9%, signal=32%
REACTOME_PYRUVATE_METABOLISM_AND_CITRIC_ACID_TCA_C	Details ...	42	0.5136536	1.9421911	0	0.47302014	0.49	275	tags=55%, list=15%, signal=63%
REACTOME_LYSOSOME_VESICLE_BIOGENESIS	Details ...	10	0.7467183	1.8508468	0	0.86414456	0.89	149	tags=40%, list=8%, signal=43%
GOBP_POSITIVE_REGULATION_OF_REACTIVE_OXYGEN_SPECI	Details ...	22	0.5359739	1.8445214	0	0.7020984	0.9	118	tags=32%, list=6%, signal=34%
GOBP_INTRACELLULAR_RECEPTOR_SIGNALING_PATHWAY	Details ...	23	0.55662864	1.8357615	0	0.613008	0.92	364	tags=43%, list=19%, signal=53%
REACTOME_GOLGI_ASSOCIATED_VESICLE_BIOGENESIS	Details ...	19	0.5989545	1.8354051	0	0.51084	0.92	171	tags=32%, list=9%, signal=34%
GOBP_FATTY_ACID_BETA_OXIDATION_USING_ACYL_COA_DEH	Details ...	10	0.71456295	1.8145441	0	0.52165514	0.95	133	tags=60%, list=7%, signal=64%
REACTOME_HSF1_DEPENDENT_TRANSACTIVATION	Details ...	14	0.5952862	1.7823156	0.02222222	0.6064644	0.99	352	tags=50%, list=19%, signal=61%
REACTOME_PYRUVATE_METABOLISM	Details ...	19	0.57381123	1.7780385	0.02	0.56014293	1	180	tags=47%, list=10%, signal=52%
GOBP_POSITIVE_REGULATION_OF_SODIUM_ION_TRANSMEMBR	Details ...	10	0.65533936	1.7723538	0.03921569	0.53726286	1	407	tags=60%, list=22%, signal=76%
GOBP_POSITIVE_REGULATION_OF_REACTIVE_OXYGEN_SPECI	Details ...	11	0.6350908	1.7537982	0.02222222	0.5694325	1	244	tags=55%, list=13%, signal=62%
GOBP_NEGATIVE_REGULATION_OF_COAGULATION	Details ...	16	0.6283381	1.7522628	0	0.5314498	1	462	tags=69%, list=25%, signal=91%
GOBP_REGULATION_OF_PROTEIN_IMPORT	Details ...	12	0.6496886	1.738395	0.01960784	0.5659774	1	237	tags=42%, list=13%, signal=47%
GOBP_DETOXIFICATION	Details ...	32	0.47996372	1.7379538	0	0.52648467	1	244	tags=44%, list=13%, signal=49%
GOBP_CELLULAR_RESPONSE_TO_TOXIC_SUBSTANCE	Details ...	32	0.47996372	1.7367711	0	0.49649677	1	244	tags=44%, list=13%, signal=49%
GOBP_REGULATION_OF_REGULATED_SECRETORY_PATHWAY	Details ...	18	0.5272363	1.7364827	0	0.4654657	1	533	tags=61%, list=28%, signal=85%
REACTOME_ATTENUATION_PHASE	Details ...	10	0.59806466	1.7088945	0	0.55944675	1	352	tags=50%, list=19%, signal=61%
GOBP_RESPONSE_TO_REACTIVE_OXYGEN_SPECIES	Details ...	51	0.44189444	1.6888915	0.02083333	0.61185783	1	312	tags=39%, list=17%, signal=46%
GOBP_DEFENSE_RESPONSE_TO_BACTERIUM	Details ...	21	0.51634824	1.6850295	0.02083333	0.60045236	1	386	tags=38%, list=21%, signal=47%
REACTOME_RECYCLING_PATHWAY_OF_L1	Details ...	19	0.5323173	1.6833266	0	0.5805297	1	582	tags=58%, list=31%, signal=83%
GOBP_RESPONSE_TO_OSMOTIC_STRESS	Details ...	12	0.5740162	1.6680931	0.04	0.6221766	1	603	tags=58%, list=32%, signal=86%
GOBP_CLATHRIN_DEPENDENT_ENDOCYTOSIS	Details ...	16	0.5312063	1.6640399	0	0.61536485	1	582	tags=44%, list=31%, signal=63%
BIOCARTA_INTEGRIN_PATHWAY	Details ...	15	0.5739213	1.663275	0	0.59422874	1	238	tags=40%, list=13%, signal=45%
GOBP_REACTIVE_NITROGEN_SPECIES_METABOLIC_PROCESS	Details ...	22	0.48848405	1.6557362	0.02380952	0.6066638	1	265	tags=41%, list=14%, signal=47%
GOBP_CELLULAR_RESPONSE_TO_LIPID	Details ...	54	0.39623618	1.6474184	0	0.62160575	1	530	tags=46%, list=28%, signal=63%
GOBP_RELAXATION_OF_MUSCLE	Details ...	10	0.63676035	1.6473852	0.04545455	0.59769785	1	475	tags=90%, list=25%, signal=120%
REACTOME_RND1_GTPASE_CYCLE	Details ...	10	0.64762515	1.6357722	0	0.6349811	1	336	tags=60%, list=18%, signal=73%
GOBP_POSITIVE_REGULATION_OF_SODIUM_ION_TRANSPORT	Details ...	12	0.60173106	1.6347407	0.01960784	0.6150187	1	504	tags=58%, list=27%, signal=79%
GOBP_POSITIVE_REGULATION_OF_TRANSMEMBRANE_TRANSP	Details ...	35	0.43675008	1.6323591	0	0.60565895	1	490	tags=46%, list=26%, signal=61%
GOBP_REGULATION_OF_SODIUM_ION_TRANSPORT	Details ...	25	0.47649598	1.6294695	0.05769231	0.6029609	1	407	tags=48%, list=22%, signal=61%
GOBP_NEGATIVE_REGULATION_OF_RESPONSE_TO_WOUNDING	Details ...	17	0.55450296	1.6178851	0.05882353	0.6342517	1	462	tags=59%, list=25%, signal=77%
GOBP_TISSUE_REMODELING	Details ...	21	0.5146145	1.6168466	0.04347826	0.61964	1	212	tags=38%, list=11%, signal=42%
GOBP_REGULATION_OF_DNA_BINDING_TRANSCRIPTION_FACTO	Details ...	39	0.45406836	1.6043729	0	0.6601066	1	352	tags=41%, list=19%, signal=49%
GOBP_REGULATION_OF_PHOSPHATASE_ACTIVITY	Details ...	19	0.49941173	1.5981474	0.025	0.6755315	1	38	tags=16%, list=2%, signal=16%
GOBP_NEGATIVE_REGULATION_OF_WOUND_HEALING	Details ...	16	0.5559646	1.597671	0.02	0.65840435	1	462	tags=63%, list=25%, signal=82%
GOBP_REGULATION_OF_COAGULATION	Details ...	22	0.5348247	1.5950153	0.0212766	0.6531315	1	462	tags=64%, list=25%, signal=84%
BIOCARTA_MCALPAIN_PATHWAY	Details ...	10	0.59496903	1.5935535	0.03636364	0.639296	1	397	tags=60%, list=21%, signal=76%
GOBP_SODIUM_ION_HOMEOSTASIS	Details ...	10	0.62513226	1.5934176	0.07843138	0.6228107	1	150	tags=40%, list=8%, signal=43%
GOBP_CELLULAR_RESPONSE_TO_LIGHT_STIMULUS	Details ...	11	0.60977185	1.5923026	0.05128205	0.61045074	1	300	tags=27%, list=16%, signal=32%
GOBP_REGULATION_OF_PATTERN_RECOGNITION_RECEPTOR_	Details ...	13	0.55116206	1.5833937	0.04444445	0.63612777	1	352	tags=46%, list=19%, signal=56%
GOBP_CELLULAR_OXIDANT_DETOXIFICATION	Details ...	29	0.47891435	1.5819728	0.02040816	0.625927	1	244	tags=45%, list=13%, signal=51%
GOBP_REGULATION_OF_RESPONSE_TO_WOUNDING	Details ...	31	0.423621	1.5784512	0	0.6264962	1	462	tags=48%, list=25%, signal=63%
KEGG_LEUKOCYTE_TRANSENDOTHELIAL_MIGRATION	Details ...	25	0.45085433	1.5773182	0.02	0.61986136	1	338	tags=44%, list=18%, signal=53%
GOBP_RIBOSOMAL_SMALL_SUBUNIT_BIOGENESIS	Details ...	14	0.5593719	1.5760366	0.04545455	0.6104096	1	433	tags=57%, list=23%, signal=74%
GOBP_IMPORT_INTO_NUCLEUS	Details ...	16	0.5056203	1.5720216	0.02272727	0.61864805	1	237	tags=31%, list=13%, signal=35%
GOBP_INTRACELLULAR_STEROID_HORMONE_RECEPTOR_SIGN	Details ...	13	0.56079775	1.5704845	0	0.6112971	1	502	tags=46%, list=27%, signal=63%
GOBP_NEUROTRANSMITTER_SECRETION	Details ...	20	0.49576312	1.5690609	0.04347826	0.6031953	1	609	tags=65%, list=33%, signal=95%
GOBP_RESPONSE_TO_NUTRIENT	Details ...	34	0.41866732	1.5687275	0.04545455	0.5924694	1	186	tags=29%, list=10%, signal=32%
REACTOME_ASSEMBLY_AND_CELL_SURFACE_PRESENTATION_	Details ...	10	0.61594	1.5651045	0.01886793	0.597865	1	195	tags=40%, list=10%, signal=44%
GOBP_REGULATION_OF_REACTIVE_OXYGEN_SPECIES_BIOSYN	Details ...	22	0.49625438	1.5621426	0	0.6016793	1	265	tags=41%, list=14%, signal=47%
GOBP_AMYLOID_BETA_CLEARANCE		12	0.5761062	1.556099	0.04166667	0.6171557	1	156	tags=42%, list=8%, signal=45%
GOBP_RESPONSE_TO_ORGANOPHOSPHORUS		21	0.503577	1.549749	0.06382979	0.6303064	1	300	tags=43%, list=16%, signal=50%
GOBP_STEROID_HORMONE_MEDIATED_SIGNALING_PATHWAY		13	0.56079775	1.5471742	0.06896552	0.6275555	1	502	tags=46%, list=27%, signal=63%
GOBP_HUMORAL_IMMUNE_RESPONSE		28	0.42936262	1.5466936	0	0.6178345	1	256	tags=36%, list=14%, signal=41%

GOBP_ACETYL_COA_BIOSYNTHETIC_PROCESS_FROM_PYRUVATE	11	0.5442006	1.5438025	0	0.62078243	1	275	tags=55%, list=15%, signal=64%
GOBP_SYNAPTIC_VESICLE_EXOCYTOSIS	14	0.5310073	1.5420071	0.04081633	0.6156155	1	533	tags=64%, list=28%, signal=89%
GOBP_CAMERA_TYPE_EYE_DEVELOPMENT	25	0.4294751	1.5412192	0.0212766	0.6093178	1	93	tags=20%, list=5%, signal=21%
GOBP_HORMONE_MEDIATED_SIGNALING_PATHWAY	17	0.49093074	1.5410748	0.05882353	0.59946805	1	364	tags=35%, list=19%, signal=43%
GOBP_REGULATION_OF_CELL_DEATH	236	0.29763007	1.5409421	0	0.58974475	1	312	tags=27%, list=17%, signal=28%
GOBP_GLAND_MORPHOGENESIS	13	0.50541174	1.5404187	0.04545455	0.58183795	1	210	tags=31%, list=11%, signal=34%
GOBP_POSITIVE_REGULATION_OF_NF_KAPPAB_TRANSCRIPTION_FACTOR_A	20	0.48900017	1.5389718	0.02439024	0.57753605	1	352	tags=45%, list=19%, signal=55%
GOBP_MYELOID_CELL_HOMEOSTASIS	19	0.4875227	1.5386006	0	0.56967455	1	453	tags=47%, list=24%, signal=62%
GOBP_POSITIVE_REGULATION_OF_ERK1_AND_ERK2_CASCADE	21	0.5210301	1.5364666	0.02272727	0.5711533	1	256	tags=33%, list=14%, signal=38%
GOBP_VESICLE_MEDIATED_TRANSPORT_IN_SYNAPSE	31	0.4168538	1.5341533	0.0212766	0.57054746	1	609	tags=55%, list=33%, signal=80%
GOBP_REGULATION_OF_PHOSPHOPROTEIN_PHOSPHATASE_ACTIVITY	17	0.5301325	1.533552	0.01923077	0.56335825	1	38	tags=18%, list=2%, signal=18%
GOBP_REGULATION_OF_NITRIC_OXIDE_METABOLIC_PROCESS	17	0.5276979	1.526773	0.08	0.5839155	1	131	tags=35%, list=7%, signal=38%
GOBP_IMPORT_ACROSS_PLASMA_MEMBRANE	21	0.47138086	1.5251307	0.03636364	0.58127654	1	150	tags=29%, list=8%, signal=31%
GOBP_MITOCHONDRIAL_CALCIUM_ION_HOMEOSTASIS	12	0.5550949	1.5251247	0.0212766	0.57272834	1	134	tags=33%, list=7%, signal=36%
GOBP_SODIUM_ION_TRANSPORT	31	0.42439082	1.5227715	0.01923077	0.5747219	1	232	tags=32%, list=12%, signal=36%
GOBP_ERYTHROCYTE_HOMEOSTASIS	16	0.4827554	1.5211515	0.07142858	0.5723644	1	427	tags=44%, list=23%, signal=56%
GOBP_POSITIVE_REGULATION_OF_CELL_DEATH	83	0.34803596	1.51995	0	0.5691299	1	312	tags=30%, list=17%, signal=35%
GOBP_NEGATIVE_REGULATION_OF_DNA_BINDING_TRANSCRIPTION_FACTOR	13	0.5347759	1.5198377	0.04	0.56175673	1	321	tags=38%, list=17%, signal=46%
GOBP_SUPEROXIDE_METABOLIC_PROCESS	11	0.5839026	1.5195948	0.04878049	0.5552841	1	91	tags=36%, list=5%, signal=38%
GOBP_APOPTOTIC_CELL_CLEARANCE	14	0.52108043	1.5186673	0.03636364	0.55172634	1	386	tags=50%, list=21%, signal=63%
GOBP_PROTEIN_LOCALIZATION_TO_CELL_SURFACE	16	0.51436436	1.5108395	0.09433962	0.56906193	1	322	tags=38%, list=17%, signal=45%
GOBP_CELLULAR_RESPONSE_TO_REACTIVE_OXYGEN_SPECIES	31	0.43553755	1.5083811	0.05769231	0.571136	1	506	tags=52%, list=27%, signal=70%
GOBP_RESPONSE_TO_TOXIC_SUBSTANCE	44	0.39481857	1.5076686	0.025	0.5660267	1	337	tags=39%, list=18%, signal=46%
GOBP_ADENYLATE_CYCLASE_INHIBITING_G_PROTEIN_COUPLED_RECEPTO	10	0.5544454	1.5040642	0.11764706	0.57218915	1	18	tags=20%, list=1%, signal=20%
GOBP_REACTIVE_OXYGEN_SPECIES_METABOLIC_PROCESS	69	0.35801122	1.5028148	0	0.5691499	1	265	tags=29%, list=14%, signal=33%
GOBP_REGULATION_OF_THE_FORCE_OF_HEART_CONTRACTION	16	0.4739276	1.5010582	0.04761905	0.5696775	1	194	tags=44%, list=10%, signal=48%
GOBP_REPRODUCTIVE_SYSTEM_DEVELOPMENT	31	0.40892366	1.499757	0.01960784	0.56624734	1	460	tags=35%, list=25%, signal=46%
GOBP_RELEASE_OF_CYTOCHROME_C_FROM_MITOCHONDRIA	14	0.49752724	1.4925537	0.07407408	0.587139	1	165	tags=36%, list=9%, signal=39%
GOBP_RESPONSE_TO_CAMP	18	0.50119907	1.4917017	0.03921569	0.5832868	1	300	tags=44%, list=16%, signal=52%
GOBP_INTERLEUKIN_6_PRODUCTION	14	0.51668143	1.474351	0.07843138	0.64393234	1	91	tags=21%, list=5%, signal=22%
GOBP_POSITIVE_REGULATION_OF_NUCLEOCYTOPLASMIC_TRANSPORT	14	0.4621554	1.4706668	0.05769231	0.65183246	1	237	tags=36%, list=13%, signal=41%
GOBP_POSITIVE_REGULATION_OF_CATION_TRANSMEMBRANE_TRANSPORT	26	0.43352228	1.4695545	0.05	0.6485599	1	490	tags=50%, list=26%, signal=67%
GOBP_RESPONSE_TO_INTERLEUKIN_12	22	0.43208984	1.4684012	0.06382979	0.6454952	1	269	tags=36%, list=14%, signal=42%
GOBP_RESPONSE_TO_HYDROGEN_PEROXIDE	33	0.42150134	1.4680078	0.06122449	0.64033353	1	312	tags=42%, list=17%, signal=50%
GOBP_NUCLEAR_TRANSPORT	32	0.41326642	1.4668565	0.04347826	0.6390586	1	244	tags=28%, list=13%, signal=32%
GOBP_REGULATION_OF_ACTIN_FILAMENT_BASED_MOVEMENT	15	0.49139968	1.4653349	0.08928572	0.6381838	1	228	tags=40%, list=12%, signal=45%
GOBP_THIOESTER_METABOLIC_PROCESS	45	0.33839324	1.4628047	0.04761905	0.6422488	1	275	tags=31%, list=15%, signal=36%
GOBP_PROTEIN_PHOSPHOPANTETHEINYLATION	39	0.39227933	1.4573206	0.02173913	0.65555894	1	513	tags=44%, list=27%, signal=59%
GOBP_POSITIVE_REGULATION_OF_TRANSPORTER_ACTIVITY	27	0.40570375	1.4572986	0.05263158	0.64864755	1	490	tags=44%, list=26%, signal=59%
GOBP_NEGATIVE_REGULATION_OF_NEURON_APOPTOTIC_PROCESS	16	0.46142447	1.4568692	0.06666667	0.6437754	1	579	tags=56%, list=31%, signal=81%
REACTOME_SIGNALING_BY_NUCLEAR_RECEPTORS	41	0.39060232	1.4546573	0.02173913	0.64478916	1	174	tags=24%, list=9%, signal=26%
GOBP_NEGATIVE_REGULATION_OF_CELL_POPULATION_PROLIFERATION	57	0.3675846	1.4539713	0.02	0.6403333	1	365	tags=35%, list=20%, signal=42%
GOBP_ERK1_AND_ERK2_CASCADE	39	0.36419582	1.4525334	0.02272727	0.63962513	1	340	tags=31%, list=18%, signal=37%
GOBP_SODIUM_ION_TRANSMEMBRANE_TRANSPORT	28	0.41400087	1.4501771	0.04081633	0.6439805	1	407	tags=43%, list=22%, signal=54%
GOBP_RESPONSE_TO_OXIDATIVE_STRESS	90	0.33943203	1.4498357	0.02272727	0.63850355	1	325	tags=33%, list=17%, signal=38%
GOBP_POSITIVE_REGULATION_OF_MITOTIC_CELL_CYCLE	12	0.5198153	1.4487112	0.05357143	0.6367067	1	679	tags=67%, list=36%, signal=104%
KEGG_CITRATE_CYCLE_TCA_CYCLE	24	0.43205792	1.4480853	0.0877193	0.63280296	1	261	tags=50%, list=14%, signal=57%
REACTOME_NEUROTRANSMITTER_RELEASE_CYCLE	10	0.5401874	1.4431566	0.06	0.64460725	1	195	tags=40%, list=10%, signal=44%
GOBP_CELLULAR_RESPONSE_TO_VIRUS	11	0.52283347	1.4420174	0.09090909	0.64243555	1	227	tags=45%, list=12%, signal=51%
REACTOME_GENE_AND_PROTEIN_EXPRESSION_BY_JAK_STAT_SIGNALING_	18	0.46148282	1.4346715	0.06521739	0.6643241	1	269	tags=39%, list=14%, signal=45%
GOBP_CRISTAE_FORMATION	26	0.41047058	1.4331744	0.07317073	0.66322875	1	134	tags=31%, list=7%, signal=33%
GOBP_PATTERN_RECOGNITION_RECEPTOR_SIGNALING_PATHWAY	30	0.4268349	1.4280741	0.04255319	0.6770436	1	352	tags=33%, list=19%, signal=40%
REACTOME_THE_CITRIC_ACID_TCA_CYCLE_AND_RESPIRATORY_ELECTRON	128	0.3026835	1.4251705	0	0.68189913	1	293	tags=35%, list=16%, signal=39%
GOBP_RESPONSE_TO_OXYGEN_CONTAINING_COMPOUND	241	0.2800338	1.4229186	0	0.6841001	1	409	tags=31%, list=22%, signal=35%
GOBP_MUSCLE_CELL_PROLIFERATION	23	0.4527909	1.4222564	0.125	0.68005127	1	255	tags=35%, list=14%, signal=40%
GOBP_REGULATION_OF_INTRACELLULAR_STEROID_HORMONE_RECEPTOR_	11	0.53082615	1.4195887	0.13725491	0.6853412	1	244	tags=27%, list=13%, signal=31%
GOBP_REGULATION_OF_BODY_FLUID_LEVELS	95	0.31842038	1.418389	0	0.68549514	1	511	tags=43%, list=27%, signal=56%

GOBP_HYDROGEN_PEROXIDE_CATABOLIC_PROCESS		13	0.49486062	1.415349	0.10204082	0.69328	1	312	tags=46%, list=17%, signal=55%
GOBP_RESPONSE_TO_ETHANOL		22	0.42512107	1.4119824	0.02040816	0.7026579	1	485	tags=55%, list=26%, signal=73%
GOBP_REGULATION_OF_LIPID_METABOLIC_PROCESS		66	0.3300293	1.4108557	0.03225806	0.7019862	1	350	tags=32%, list=19%, signal=38%
GOBP_SENSORY_SYSTEM_DEVELOPMENT		28	0.40082762	1.4041423	0.06976745	0.72629017	1	156	tags=21%, list=8%, signal=23%
GOBP_REGULATION_OF_WOUND_HEALING		28	0.4062773	1.4004407	0.06521739	0.7357471	1	462	tags=50%, list=25%, signal=65%
GOBP_REGULATION_OF_PROTON_TRANSPORT		10	0.55364627	1.3952479	0.09259259	0.7536035	1	244	tags=40%, list=13%, signal=46%
GOBP_REGULATION_OF_ORGANELLE_ASSEMBLY		25	0.42309633	1.394636	0.04444445	0.7503681	1	635	tags=68%, list=34%, signal=102%
GOBP_REGULATION_OF_REACTIVE_OXYGEN_SPECIES_METABOLIC_PROCE		46	0.38220608	1.3919017	0.04878049	0.75767726	1	148	tags=24%, list=8%, signal=25%
GOBP_REGULATION_OF_NUCLEOCYTOPLASMIC_TRANSPORT		20	0.4530565	1.3912833	0.07272727	0.7547731	1	244	tags=35%, list=13%, signal=40%
GOBP_RENAL_SYSTEM_PROCESS		15	0.49030602	1.3910053	0.13725491	0.7495865	1	471	tags=53%, list=25%, signal=71%
GOBP_RESPONSE_TO_BACTERIUM		68	0.33575404	1.3891883	0.07142858	0.7510695	1	390	tags=31%, list=21%, signal=38%
GOBP_REGULATION_OF_ACTION_POTENTIAL		18	0.4493821	1.3887726	0.10638298	0.74692756	1	228	tags=33%, list=12%, signal=38%
KEGG_FOCAL_ADHESION		57	0.33201644	1.3855373	0.01960784	0.7546714	1	254	tags=28%, list=14%, signal=31%
GOBP_PLATELET_ACTIVATION		40	0.36716047	1.3817432	0.02439024	0.76589787	1	400	tags=38%, list=21%, signal=47%
GOBP_NEGATIVE_REGULATION_OF_IMMUNE_SYSTEM_PROCESS		37	0.38479614	1.3809263	0.02380952	0.7633505	1	337	tags=30%, list=18%, signal=36%
GOBP_REACTIVE_OXYGEN_SPECIES_BIOSYNTHETIC_PROCESS		31	0.3928106	1.3792199	0.0625	0.76719075	1	265	tags=35%, list=14%, signal=41%
GOBP_REGULATION_OF_CATION_TRANSMEMBRANE_TRANSPORT		66	0.3248918	1.3791683	0	0.7612968	1	504	tags=42%, list=27%, signal=56%
GOBP_RESPONSE_TO_ACTIVITY		18	0.43567404	1.3789532	0.09803922	0.7568742	1	74	tags=22%, list=4%, signal=23%
GOBP_REGULATION_OF_SODIUM_ION_TRANSMEMBRANE_TRANSPORTER_A		17	0.45371294	1.3789346	0.09756097	0.7510521	1	407	tags=47%, list=22%, signal=60%
GOBP_INNER_MITOCHONDRIAL_MEMBRANE_ORGANIZATION		43	0.3701109	1.3789049	0.02222222	0.745417	1	176	tags=26%, list=9%, signal=28%
GOBP_COAGULATION		78	0.30578065	1.3767822	0.01960784	0.7492208	1	462	tags=38%, list=25%, signal=49%
GOBP_ANIMAL_ORGAN_REGENERATION		12	0.47579092	1.3759377	0.13333334	0.7469452	1	339	tags=50%, list=18%, signal=61%
REACTOME_ACTIVATION_OF_NMDA_RECEPTORS_AND_POSTSYNAPTIC_EVE		19	0.43264383	1.3743886	0.0862069	0.74920654	1	233	tags=26%, list=12%, signal=30%
REACTOME_RHOF_GTPASE_CYCLE		10	0.5391189	1.3734366	0.08163265	0.7475195	1	253	tags=40%, list=14%, signal=46%
GOBP_FATTY_ACID_BETA_OXIDATION		44	0.34325305	1.3728462	0.03773585	0.74472785	1	133	tags=25%, list=7%, signal=26%
GOBP_NEGATIVE_REGULATION_OF_CELL_ACTIVATION		19	0.44016144	1.3704275	0.10869565	0.74917895	1	390	tags=32%, list=21%, signal=39%
GOBP_CYCLIC_NUCLEOTIDE_MEDIATED_SIGNALING		11	0.4779366	1.3704133	0.0877193	0.7438413	1	397	tags=55%, list=21%, signal=69%
REACTOME_CRISTAE_FORMATION		24	0.416595	1.3702787	0.06382979	0.73876834	1	103	tags=29%, list=6%, signal=30%
GOBP_POSITIVE_REGULATION_OF_POTASSIUM_ION_TRANSPORT		11	0.5288578	1.3687054	0.13636364	0.74013215	1	154	tags=36%, list=8%, signal=39%
GOBP_TRICARBOXYLIC_ACID_CYCLE		27	0.41765186	1.3665655	0.07692308	0.7437233	1	261	tags=48%, list=14%, signal=55%
REACTOME_REGULATION_OF_PYRUVATE_DEHYDROGENASE_PDH_COMPLE		10	0.58064944	1.3628458	0.13953489	0.75320756	1	275	tags=60%, list=15%, signal=70%
REACTOME_SIGNALING_BY_MODERATE_KINASE_ACTIVITY_BRAF_MUTANTS		19	0.4363182	1.3620913	0.1	0.7509896	1	256	tags=37%, list=14%, signal=42%
REACTOME_MITOCHONDRIAL_CALCIUM_ION_TRANSPORT		17	0.45120862	1.3606908	0.08888889	0.75099	1	260	tags=41%, list=14%, signal=47%
GOBP_SMOOTH_MUSCLE_CELL_PROLIFERATION		21	0.43353257	1.3597172	0.0882353	0.74994624	1	255	tags=33%, list=14%, signal=38%
GOBP_POSITIVE_REGULATION_OF_DNA_BINDING_TRANSCRIPTION_FACTOR		27	0.4220279	1.3593242	0.07142858	0.74714035	1	270	tags=37%, list=14%, signal=43%
GOBP_EPITHELIAL_CELL_DEVELOPMENT		34	0.3801366	1.3581041	0.04878049	0.74694973	1	611	tags=56%, list=33%, signal=81%
REACTOME_PLASMA_LIPOPROTEIN_CLEARANCE		10	0.5362331	1.3548039	0.13636364	0.75672406	1	582	tags=80%, list=31%, signal=116%
GOBP_PROTEIN_CONTAINING_COMPLEX_LOCALIZATION		23	0.41148323	1.3531861	0.0952381	0.7577975	1	595	tags=61%, list=32%, signal=88%
REACTOME_CITRIC_ACID_CYCLE_TCA_CYCLE		21	0.42692062	1.3528337	0.14	0.7545304	1	261	tags=52%, list=14%, signal=60%
GOBP_PROTEIN_LOCALIZATION_TO_SYNAPSE		10	0.5248028	1.3526376	0.10204082	0.7505496	1	553	tags=70%, list=30%, signal=99%
GOBP_RECEPTOR_CATABOLIC_PROCESS		12	0.47603962	1.3526145	0.08510638	0.7457794	1	232	tags=42%, list=12%, signal=47%
GOBP_ESTABLISHMENT_OF_ENDOTHELIAL_BARRIER		17	0.44613233	1.3525716	0.125	0.7410737	1	647	tags=59%, list=35%, signal=89%
KEGG_LONG_TERM_POTENTIATION		13	0.46512857	1.3517739	0.08695652	0.73941284	1	563	tags=54%, list=30%, signal=76%
GOBP_CARDIAC_MUSCLE_CELL_ACTION_POTENTIAL		26	0.3802348	1.3507358	0.13725491	0.7385909	1	228	tags=35%, list=12%, signal=39%
GOBP_HEPATICOBILIARY_SYSTEM_DEVELOPMENT		22	0.41659802	1.3478246	0.16	0.7443063	1	368	tags=32%, list=20%, signal=39%
GOBP_NEUROTRANSMITTER_TRANSPORT		26	0.37537447	1.3471605	0.08695652	0.74283797	1	564	tags=50%, list=30%, signal=71%
GOBP_NEGATIVE_REGULATION_OF_CELLULAR_RESPONSE_TO_GROWTH_F		10	0.5344721	1.3451295	0.11904762	0.7465985	1	366	tags=50%, list=20%, signal=62%
KEGG_FC_GAMMA_R_MEDIATED_PHAGOCYTOSIS		19	0.42888921	1.3439798	0.11627907	0.74733293	1	407	tags=37%, list=22%, signal=47%
GOBP_CELL_COMMUNICATION_BY_ELECTRICAL_COUPLING_INVOLVED_IN_C		12	0.47738537	1.3438451	0.11111111	0.74306256	1	192	tags=42%, list=10%, signal=46%
GOBP_CELLULAR_RESPONSE_TO_KETONE		16	0.47881135	1.3419374	0.13333334	0.7446828	1	604	tags=69%, list=32%, signal=101%
GOBP_VESICLE_CYTOSKELETAL_TRAFFICKING		13	0.47428915	1.3391981	0.18181819	0.7515962	1	516	tags=54%, list=28%, signal=74%
REACTOME_EPH_EPHRIN_MEDIATED_REPULSION_OF_CELLS		11	0.52001077	1.3376716	0.10869565	0.75324917	1	582	tags=55%, list=31%, signal=79%
GOBP_PROTEIN_IMPORT		40	0.37034866	1.3349324	0.12195122	0.7607829	1	237	tags=28%, list=13%, signal=31%
GOBP_FATTY_ACID_CATABOLIC_PROCESS		56	0.32753286	1.3326682	0.06896552	0.76592803	1	167	tags=25%, list=9%, signal=27%
REACTOME_INTERLEUKIN_12_FAMILY_SIGNALING		23	0.4210727	1.330652	0.12727273	0.77040005	1	269	tags=35%, list=14%, signal=40%
GOBP_REGULATION_OF_FATTY_ACID_METABOLIC_PROCESS		22	0.4356553	1.330399	0.17021276	0.7668463	1	350	tags=41%, list=19%, signal=50%
GOBP_KERATINOCYTE_DIFFERENTIATION		14	0.473976	1.3287886	0.1	0.77003515	1	600	tags=57%, list=32%, signal=83%

GOBP_RESPONSE_TO_MOLECULE_OF_BACTERIAL_ORIGIN		39	0.34867314	1.3274556	0.08510638	0.77052987	1	430	tags=33%, list=23%, signal=42%
REACTOME_HEMOSTASIS		146	0.26859757	1.3258299	0.01886793	0.77341306	1	409	tags=33%, list=22%, signal=39%
REACTOME_ION_TRANSPORT_BY_P_TYPE_ATPASES		10	0.5049766	1.3252743	0.14583333	0.7709799	1	194	tags=40%, list=10%, signal=44%
REACTOME_ESR_MEDIATED_SIGNALING		25	0.38581818	1.3226274	0.15	0.7772269	1	131	tags=20%, list=7%, signal=21%
GOBP_REGULATION_OF_NITRIC_OXIDE_SYNTHASE_ACTIVITY		12	0.47078595	1.3199884	0.12820514	0.784145	1	448	tags=50%, list=24%, signal=65%
GOBP_NEGATIVE_REGULATION_OF_MAPK_CASCADE		24	0.3997098	1.3178147	0.06818182	0.78771716	1	340	tags=38%, list=18%, signal=45%
GOBP_NEGATIVE_REGULATION_OF_CELL_DEATH		147	0.2837306	1.3174733	0	0.78474736	1	321	tags=28%, list=17%, signal=31%
GOBP_REGULATION_OF_SODIUM_ION_TRANSMEMBRANE_TRANSPORT		22	0.40528885	1.3171237	0.13461539	0.78230584	1	407	tags=45%, list=22%, signal=57%
GOBP_MONOCARBOXYLIC_ACID_CATABOLIC_PROCESS		60	0.31475526	1.316949	0.06666667	0.77853215	1	167	tags=23%, list=9%, signal=25%
REACTOME_TRANSMISSION_ACROSS_CHEMICAL_SYNAPSES		49	0.32709855	1.3169017	0.12195122	0.774516	1	233	tags=24%, list=12%, signal=27%
GOBP_POSITIVE_REGULATION_OF_PEPTIDYL_SERINE_PHOSPHORYLATION		10	0.5065604	1.3135506	0.08333334	0.78398556	1	244	tags=40%, list=13%, signal=46%
GOBP_POSITIVE_REGULATION_OF_PROTEIN_DEPHOSPHORYLATION		11	0.50388765	1.3131509	0.1923077	0.78167456	1	580	tags=55%, list=31%, signal=79%
GOBP_NEGATIVE_REGULATION_OF_MULTICELLULAR_ORGANISMAL_PROCE		115	0.2863328	1.3126779	0.07142858	0.77884007	1	462	tags=33%, list=25%, signal=41%
GOBP_CIRCULATORY_SYSTEM_DEVELOPMENT		158	0.26752418	1.3119038	0	0.77696556	1	366	tags=26%, list=20%, signal=30%
REACTOME_ONCOGENIC_MAPK_SIGNALING		26	0.3977945	1.3107072	0.10526316	0.7777794	1	258	tags=35%, list=14%, signal=40%
GOBP_POSITIVE_REGULATION_OF_SYNAPTIC_TRANSMISSION		12	0.45647144	1.3057277	0.08888889	0.79538935	1	380	tags=42%, list=20%, signal=52%
GOBP_POSITIVE_REGULATION_OF_SMOOTH_MUSCLE_CELL_PROLIFERATIO		14	0.44110158	1.3036658	0.09302326	0.7986691	1	227	tags=29%, list=12%, signal=32%
GOBP_REGULATION_OF_VASCULATURE_DEVELOPMENT		47	0.3315259	1.3030727	0.12765957	0.7982295	1	366	tags=32%, list=20%, signal=39%
REACTOME_CYTOKINE_SIGNALING_IN_IMMUNE_SYSTEM		104	0.2856237	1.3026296	0.09302326	0.7955267	1	269	tags=20%, list=14%, signal=22%
GOBP_CYTOKINE_MEDIATED_SIGNALING_PATHWAY		106	0.2901002	1.3019469	0.06060606	0.7937992	1	210	tags=19%, list=11%, signal=20%
GOBP_ANTIMICROBIAL_HUMORAL_RESPONSE		12	0.47888342	1.3002876	0.14893617	0.7966208	1	256	tags=42%, list=14%, signal=48%
REACTOME_NEUROTRANSMITTER_RECEPTORS_AND_POSTSYNAPTIC_SIGNA		36	0.34474775	1.2999723	0.15789473	0.79357	1	233	tags=25%, list=12%, signal=28%
GOBP_PEPTIDE_CROSS_LINKING		10	0.4751546	1.2970507	0.21276596	0.80222106	1	554	tags=60%, list=30%, signal=85%
REACTOME_G_PROTEIN_MEDIATED_EVENTS		14	0.48021057	1.2963997	0.11904762	0.80030644	1	536	tags=50%, list=29%, signal=70%
GOBP_RECEPTOR_INTERNALIZATION		25	0.37097764	1.2937856	0.17073171	0.80785155	1	291	tags=32%, list=16%, signal=37%
GOBP_REGULATION_OF_COMPLEMENT_ACTIVATION		14	0.4373403	1.2926761	0.14	0.80887324	1	254	tags=36%, list=14%, signal=41%
GOBP_RECEPTOR_METABOLIC_PROCESS		39	0.32350612	1.2923623	0.11764706	0.80597115	1	535	tags=46%, list=29%, signal=63%
GOBP_APOPTOTIC_PROCESS		273	0.24397774	1.2906976	0	0.80862665	1	311	tags=23%, list=17%, signal=23%
GOBP_MONOVALENT_INORGANIC_CATION_HOMEOSTASIS		27	0.4012471	1.290474	0.16981132	0.8052933	1	150	tags=22%, list=8%, signal=24%
GOBP_LIPID_OXIDATION		51	0.31283054	1.288212	0.06976745	0.8112735	1	167	tags=24%, list=9%, signal=25%
GOBP_PROTEIN_LOCALIZATION_TO_ENDOSOME		10	0.5077457	1.2859405	0.08333334	0.8166027	1	595	tags=60%, list=32%, signal=88%
GOBP_MITOCHONDRIAL_CALCIUM_ION_TRANSMEMBRANE_TRANSPORT		16	0.43433857	1.28582	0.1521739	0.81284344	1	227	tags=38%, list=12%, signal=42%
GOBP_EPITHELIAL_CELL_DIFFERENTIATION		74	0.30294248	1.2854096	0.11904762	0.8105192	1	611	tags=49%, list=33%, signal=69%
GOBP_METAL_ION_HOMEOSTASIS		98	0.2803202	1.2839919	0.05769231	0.8123126	1	194	tags=19%, list=10%, signal=20%
GOBP_REGULATION_OF_PROTEIN_LOCALIZATION_TO_NUCLEUS		24	0.4015921	1.2814801	0.15	0.8193334	1	244	tags=33%, list=13%, signal=38%
GOBP_EXOCYTIC_PROCESS		18	0.4150622	1.281248	0.16666667	0.81644136	1	518	tags=39%, list=28%, signal=53%
GOBP_REGULATION_OF_FATTY_ACID_OXIDATION		10	0.46409383	1.2795143	0.16666667	0.81994617	1	48	tags=20%, list=3%, signal=20%
GOBP_RESPONSE_TO_TOPOLOGICALLY_INCORRECT_PROTEIN		53	0.33300778	1.278145	0.06	0.82187665	1	118	tags=19%, list=6%, signal=20%
GOBP_REGENERATION		26	0.3950773	1.2777158	0.12820514	0.81963986	1	339	tags=38%, list=18%, signal=46%
GOBP_CELLULAR_RESPONSE_TO_CHEMICAL_STRESS		66	0.3244409	1.277277	0.02325581	0.8176797	1	407	tags=35%, list=22%, signal=43%
GOBP_REGULATION_OF_CELLULAR_RESPONSE_TO_GROWTH_FACTOR_STI		32	0.36612475	1.276078	0.07142858	0.8183972	1	439	tags=47%, list=23%, signal=60%
GOBP_CELLULAR_RESPONSE_TO_STEROID_HORMONE_STIMULUS		25	0.394601	1.2755055	0.13043478	0.81729287	1	382	tags=36%, list=20%, signal=45%
GOBP_POST_GOLGI_VESICLE_MEDIATED_TRANSPORT		23	0.39582357	1.2746878	0.1632653	0.81752753	1	535	tags=48%, list=29%, signal=66%
GOBP_REGULATION_OF_STRIATED_MUSCLE_CONTRACTION		30	0.3657586	1.2740719	0.11363637	0.8160079	1	228	tags=30%, list=12%, signal=34%
GOBP_IMPORT_INTO_CELL		30	0.36399055	1.2689564	0.09433962	0.83301204	1	407	tags=37%, list=22%, signal=46%
GOBP_FATTY_ACID_METABOLIC_PROCESS		120	0.2734806	1.2684519	0.04444445	0.83145005	1	180	tags=21%, list=10%, signal=22%
GOBP_RESPONSE_TO_PURINE_CONTAINING_COMPOUND		26	0.3965819	1.2670561	0.10638298	0.83376217	1	141	tags=27%, list=8%, signal=29%
GOBP_RESPONSE_TO_OXYGEN_LEVELS		89	0.2870708	1.2668862	0.08108108	0.83038026	1	434	tags=31%, list=23%, signal=39%
GOBP_NEGATIVE_REGULATION_OF_PROTEIN_SERINE_THREONINE_KINASE		17	0.4350006	1.265731	0.13953489	0.83206624	1	397	tags=47%, list=21%, signal=59%
GOBP_SYNAPTIC_SIGNALING		70	0.30198988	1.2656391	0.06122449	0.8289523	1	536	tags=43%, list=29%, signal=58%
REACTOME_L1CAM_INTERACTIONS		41	0.32793692	1.2626112	0.125	0.8387573	1	407	tags=32%, list=22%, signal=40%
GOBP_COMPLEMENT_ACTIVATION		15	0.4266535	1.2618601	0.20754717	0.8382987	1	254	tags=33%, list=14%, signal=38%
GOBP_POSITIVE_REGULATION_OF_DEPHOSPHORYLATION		11	0.50388765	1.2589611	0.21428572	0.84697795	1	580	tags=55%, list=31%, signal=79%
GOBP_POSITIVE_REGULATION_OF_I_KAPPAB_KINASE_NF_KAPPAB_SIGNAL		20	0.37750775	1.2576634	0.23636363	0.84857625	1	170	tags=30%, list=9%, signal=33%
REACTOME_SIGNALING_BY_BRAF_AND_RAF_FUSIONS		24	0.40021047	1.2574606	0.16	0.845691	1	258	tags=33%, list=14%, signal=38%
GOBP_REGULATION_OF_PEPTIDYL_SERINE_PHOSPHORYLATION		15	0.3846275	1.2541753	0.16363636	0.8554584	1	504	tags=47%, list=27%, signal=63%
GOBP_MACROMOLECULE_DEACYLATION		11	0.45190573	1.2538553	0.20754717	0.8535899	1	604	tags=64%, list=32%, signal=93%

GOBP_RESPONSE_TO_LIPID		104	0.27797106	1.2521974	0.1	0.85733473	1	530	tags=41%, list=28%, signal=54%
REACTOME_INTERLEUKIN_12_SIGNALING		21	0.4074863	1.2520356	0.125	0.85444736	1	269	tags=33%, list=14%, signal=38%
GOBP_REGULATION_OF_MEMBRANE_POTENTIAL		69	0.2916348	1.2512269	0.09756097	0.8541171	1	244	tags=28%, list=13%, signal=30%
GOBP_REGULATION_OF_G_PROTEIN_COUPLED_RECEPTOR_SIGNALING_PA		13	0.48682877	1.250109	0.17021276	0.85474354	1	449	tags=54%, list=24%, signal=70%
GOBP_POSITIVE_REGULATION_OF_VASCULATURE_DEVELOPMENT		25	0.36265615	1.2500091	0.13636364	0.8515797	1	338	tags=32%, list=18%, signal=39%
GOBP_REGULATION_OF_RNA_SPLICING		12	0.43505368	1.249912	0.21153846	0.8481699	1	389	tags=42%, list=21%, signal=52%
GOBP_WOUND_HEALING		108	0.25842094	1.2497916	0.04878049	0.84484214	1	410	tags=31%, list=22%, signal=37%
GOBP_PLACENTA_DEVELOPMENT		13	0.45850942	1.2493182	0.1875	0.84273905	1	132	tags=23%, list=7%, signal=25%
REACTOME_MITOCHONDRIAL_BIOGENESIS		35	0.33718878	1.2478868	0.10638298	0.844477	1	103	tags=23%, list=6%, signal=24%
REACTOME_PLATELET_ACTIVATION_SIGNALING_AND_AGGREGATION		86	0.28278717	1.2478824	0.10638298	0.84088343	1	472	tags=38%, list=25%, signal=49%
GOBP_SENSORY_ORGAN_DEVELOPMENT		38	0.33890432	1.2459589	0.18181819	0.84475434	1	93	tags=13%, list=5%, signal=14%
GOBP_PLATELET_DEGRANULATION		53	0.31589082	1.2453715	0.10204082	0.84399295	1	490	tags=43%, list=26%, signal=57%
REACTOME_DEGRADATION_OF_THE_EXTRACELLULAR_MATRIX		20	0.38714984	1.2435583	0.14893617	0.8479349	1	552	tags=55%, list=30%, signal=77%
GOBP_MUSCLE_CELL_DEVELOPMENT		42	0.32527187	1.240485	0.11111111	0.859134	1	233	tags=26%, list=12%, signal=29%
GOBP_ACETYL_COA_BIOSYNTHETIC_PROCESS		14	0.42127383	1.2387658	0.16363636	0.86347795	1	275	tags=43%, list=15%, signal=50%
GOBP_REGULATION_OF_EXOCYTOSIS		36	0.3542025	1.2382296	0.21428572	0.8616478	1	533	tags=53%, list=28%, signal=72%
REACTOME_MITOCHONDRIAL_FATTY_ACID_BETA_OXIDATION		29	0.37220848	1.2332721	0.20408164	0.8788232	1	319	tags=45%, list=17%, signal=53%
GOBP_AEROBIC_RESPIRATION		56	0.30664188	1.2331641	0.23255815	0.8756289	1	332	tags=39%, list=18%, signal=46%
KEGG_PEROXISOME		36	0.3475118	1.2325482	0.1590909	0.87486744	1	262	tags=31%, list=14%, signal=35%
KEGG_ARGININE_AND_PROLINE_METABOLISM		16	0.40493926	1.2325143	0.19565217	0.87155366	1	114	tags=38%, list=6%, signal=40%
GOBP_REGULATION_OF_CELLULAR_COMPONENT_MOVEMENT		149	0.25635695	1.2319295	0.05	0.87080485	1	400	tags=28%, list=21%, signal=32%
GOBP_THIOESTER_BIOSYNTHETIC_PROCESS		25	0.36753097	1.2310308	0.17777778	0.8706375	1	180	tags=28%, list=10%, signal=31%
GOBP_POSITIVE_REGULATION_OF_ATPASE_ACTIVITY		21	0.37973222	1.2279593	0.13333334	0.88065803	1	407	tags=33%, list=22%, signal=42%
GOBP_RUFFLE_ORGANIZATION		16	0.39732495	1.2265481	0.19607843	0.8831128	1	384	tags=31%, list=21%, signal=39%
GOBP_PROTEIN_LOCALIZATION_TO_CELL_JUNCTION		23	0.36155856	1.225631	0.22727273	0.8838075	1	553	tags=48%, list=30%, signal=67%
GOBP_CARDIAC_MUSCLE_CELL_CONTRACTION		21	0.39191628	1.224452	0.2037037	0.88591474	1	228	tags=33%, list=12%, signal=38%
GOBP_LIPOSACCHARIDE_METABOLIC_PROCESS		11	0.44849026	1.2240057	0.26	0.88401645	1	728	tags=64%, list=39%, signal=104%
GOBP_REGULATION_OF_BLOOD_PRESSURE		22	0.3652985	1.2229987	0.16363636	0.884558	1	95	tags=23%, list=5%, signal=24%
GOBP_RIBOSOME_BIOGENESIS		46	0.3175871	1.2192749	0.14285715	0.8978542	1	433	tags=37%, list=23%, signal=47%
GOBP_MITOCHONDRIAL_MEMBRANE_ORGANIZATION		75	0.28449675	1.2186022	0.13157895	0.8975808	1	403	tags=35%, list=22%, signal=42%
GOBP_SECOND_MESSENGER_MEDIATED_SIGNALING		48	0.30541787	1.217731	0.18604651	0.89801204	1	233	tags=23%, list=12%, signal=26%
GOBP_RECEPTOR_MEDIATED_ENDOCYTOSIS		69	0.31301042	1.2171556	0.1590909	0.89699113	1	312	tags=29%, list=17%, signal=34%
GOBP_SUPRAMOLECULAR_FIBER_ORGANIZATION		142	0.2446777	1.2171336	0.02439024	0.89361364	1	449	tags=27%, list=24%, signal=33%
GOBP_CELLULAR_RESPONSE_TO_OXYGEN_CONTAINING_COMPOUND		167	0.24830765	1.2170503	0.05555556	0.8905104	1	409	tags=29%, list=22%, signal=34%
GOBP_CELL_COMMUNICATION_INVOLVED_IN_CARDIAC_CONDUCTION		22	0.37076485	1.2162639	0.21153846	0.8896381	1	357	tags=41%, list=19%, signal=50%
GOBP_POSITIVE_REGULATION_OF_ENDOCYTOSIS		25	0.3788219	1.2156316	0.22222222	0.88921946	1	407	tags=44%, list=22%, signal=55%
GOBP_REGULATION_OF_CALCIUM_MEDIATED_SIGNALING		12	0.41739103	1.2153124	0.15555556	0.88713914	1	104	tags=17%, list=6%, signal=18%
GOBP_MEMBRANE_RAFT_ORGANIZATION		14	0.39734343	1.2147295	0.20408164	0.88566685	1	324	tags=36%, list=17%, signal=43%
GOBP_MUSCLE_CONTRACTION		95	0.26441568	1.2108228	0.09756097	0.8996268	1	246	tags=25%, list=13%, signal=28%
GOBP_BODY_FLUID_SECRETION		12	0.40554297	1.2097566	0.23809524	0.90118843	1	557	tags=67%, list=30%, signal=94%
KEGG_ENDOCYTOSIS		36	0.34925136	1.2089055	0.13461539	0.90111893	1	589	tags=50%, list=31%, signal=72%
KEGG_BUTANOATE_METABOLISM		16	0.4209188	1.2081164	0.17391305	0.90147114	1	375	tags=56%, list=20%, signal=70%
GOBP_POSITIVE_REGULATION_OF_AUTOPHAGY		18	0.40739587	1.2061063	0.2173913	0.9065404	1	366	tags=28%, list=20%, signal=34%
GOBP_POSITIVE_REGULATION_OF_RESPONSE_TO_WOUNDING		18	0.41076303	1.2049202	0.25	0.9081051	1	297	tags=39%, list=16%, signal=46%
GOBP_CELLULAR_RESPONSE_TO_MOLECULE_OF_BACTERIAL_ORIGIN		19	0.39269683	1.2047392	0.21153846	0.90572697	1	337	tags=26%, list=18%, signal=32%
GOBP_HEART_DEVELOPMENT		81	0.28896022	1.2042933	0.23076923	0.90426695	1	233	tags=19%, list=12%, signal=20%
GOBP_REGULATION_OF_ENDOTHELIAL_CELL_MIGRATION		30	0.32408574	1.200772	0.18	0.9171963	1	366	tags=33%, list=20%, signal=41%
GOBP_ENDOCYTOSIS		121	0.25983217	1.2005857	0.14	0.9145387	1	428	tags=29%, list=23%, signal=35%
GOBP_VASCULATURE_DEVELOPMENT		99	0.27208814	1.2002743	0.08163265	0.9124138	1	366	tags=28%, list=20%, signal=33%
GOBP_STRIATED_MUSCLE_CONTRACTION		53	0.29582584	1.200077	0.2	0.9099318	1	234	tags=26%, list=13%, signal=29%
GOBP_CELL_ADHESION_MEDIATED_BY_INTEGRIN		15	0.4303279	1.1988908	0.22222222	0.91156787	1	442	tags=40%, list=24%, signal=52%
GOBP_HYDROGEN_PEROXIDE_METABOLIC_PROCESS		19	0.38862857	1.1985755	0.1521739	0.90955937	1	312	tags=42%, list=17%, signal=50%
GOBP_AXO_DENDRITIC_TRANSPORT		15	0.385425	1.1963663	0.19512194	0.91666025	1	583	tags=53%, list=31%, signal=77%
GOBP_NEGATIVE_REGULATION_OF_SYNAPTIC_TRANSMISSION		11	0.4325123	1.195449	0.24489796	0.9172069	1	235	tags=36%, list=13%, signal=41%
GOBP_MUSCLE_SYSTEM_PROCESS		104	0.2649857	1.1949401	0.14285715	0.91643643	1	246	tags=24%, list=13%, signal=26%
REACTOME_BINDING_AND_UPTAKE_OF_LIGANDS_BY_SCAVENGER_RECEPT		17	0.3785004	1.1947429	0.2264151	0.9138551	1	312	tags=41%, list=17%, signal=49%
GOBP_POSITIVE_REGULATION_OF_PROTEIN_LOCALIZATION_TO_NUCLEUS		21	0.38251317	1.1935341	0.23913044	0.91517407	1	244	tags=33%, list=13%, signal=38%

GOBP_CELLULAR_RESPONSE_TO_RADIATION		20	0.36506018	1.1931913	0.2	0.91302115	1	407	tags=30%, list=22%, signal=38%
GOBP_NEGATIVE_REGULATION_OF_CATION_TRANSMEMBRANE_TRANSPORT		18	0.37232792	1.192086	0.2244898	0.91434294	1	242	tags=39%, list=13%, signal=44%
GOBP_EXTRACELLULAR_MATRIX_DISASSEMBLY		13	0.4123836	1.1917307	0.22222222	0.91270155	1	166	tags=23%, list=9%, signal=25%
GOBP_RESPONSE_TO_NITROGEN_COMPOUND		181	0.23337238	1.1871805	0.08	0.92853343	1	146	tags=14%, list=8%, signal=14%
GOBP_REGULATION_OF_MUSCLE_CONTRACTION		40	0.31586877	1.1854532	0.16666667	0.9333749	1	228	tags=25%, list=12%, signal=28%
GOBP_INTERLEUKIN_8_PRODUCTION		12	0.42309323	1.185267	0.18965517	0.9310117	1	352	tags=42%, list=19%, signal=51%
GOBP_LOCALIZATION_WITHIN_MEMBRANE		19	0.38882518	1.1852567	0.25	0.9278769	1	340	tags=42%, list=18%, signal=51%
GOBP_RESPONSE_TO_VITAMIN		13	0.43059772	1.1845683	0.2	0.92666334	1	74	tags=23%, list=4%, signal=24%
GOBP_ACTIN_FILAMENT_BUNDLE_ORGANIZATION		39	0.32699966	1.182431	0.175	0.93314034	1	434	tags=31%, list=23%, signal=39%
KEGG_REGULATION_OF_ACTIN_CYTOSKELETON		58	0.29142722	1.1824274	0.1521739	0.9299447	1	328	tags=28%, list=18%, signal=32%
GOBP_CARDIAC_MUSCLE_CONTRACTION		46	0.3203514	1.1819582	0.22	0.9287666	1	228	tags=26%, list=12%, signal=29%
GOBP_NEGATIVE_REGULATION_OF_IMMUNE_EFFECTOR_PROCESS		14	0.4423828	1.1812179	0.20754717	0.928911	1	297	tags=29%, list=16%, signal=34%
GOBP_B_CELL_MEDIATED_IMMUNITY		16	0.40136757	1.1805817	0.1754386	0.9290407	1	182	tags=25%, list=10%, signal=27%
GOBP_REGULATION_OF_VESICLE_MEDIATED_TRANSPORT		112	0.25894696	1.1805478	0.18421052	0.9260323	1	544	tags=40%, list=29%, signal=53%
REACTOME_FATTY_ACID_METABOLISM		75	0.28246003	1.180493	0.14583333	0.9231706	1	173	tags=21%, list=9%, signal=23%
REACTOME_HIV_LIFE_CYCLE		11	0.42515773	1.1802953	0.22807017	0.92105836	1	589	tags=64%, list=31%, signal=92%
GOBP_RESPONSE_TO_ENDOGENOUS_STIMULUS		216	0.22467674	1.1785604	0.11363637	0.9253904	1	485	tags=32%, list=26%, signal=39%
GOBP_REGULATION_OF_TRANSPORTER_ACTIVITY		56	0.28626984	1.1771954	0.13461539	0.92843044	1	504	tags=39%, list=27%, signal=52%
GOBP_REGULATION_OF_CARBOHYDRATE_BIOSYNTHETIC_PROCESS		10	0.45344403	1.1768836	0.2264151	0.9264563	1	309	tags=40%, list=17%, signal=48%
REACTOME_EXTRA_NUCLEAR_ESTROGEN_SIGNALING		15	0.39408115	1.1759037	0.2	0.9276128	1	131	tags=27%, list=7%, signal=28%
GOBP_REGULATION_OF_TRANSMEMBRANE_TRANSPORT		93	0.26940924	1.1758417	0.13333334	0.9246361	1	407	tags=31%, list=22%, signal=38%
REACTOME_SIGNALING_BY_PDGF		11	0.38366288	1.1753287	0.24	0.92359906	1	333	tags=27%, list=18%, signal=33%
GOBP_POSITIVE_REGULATION_OF_INFLAMMATORY_RESPONSE		16	0.36307293	1.1729436	0.23809524	0.92932415	1	265	tags=25%, list=14%, signal=29%
GOBP_REGULATION_OF_NEUROTRANSMITTER_LEVELS		30	0.33323437	1.1712368	0.2	0.9335407	1	244	tags=27%, list=13%, signal=30%
GOBP_BLOOD_VESSEL_MORPHOGENESIS		90	0.25753665	1.1707093	0.2	0.93320423	1	366	tags=28%, list=20%, signal=33%
GOBP_I_KAPPAB_KINASE_NF_KAPPAB_SIGNALING		32	0.33691898	1.1705574	0.18	0.9305881	1	170	tags=22%, list=9%, signal=24%
GOBP_BIOMINERALIZATION		14	0.4055508	1.1686089	0.2	0.93652767	1	509	tags=50%, list=27%, signal=68%
GOBP_ION_HOMEOSTASIS		121	0.25747693	1.1678768	0.14705883	0.9365361	1	150	tags=17%, list=8%, signal=17%
GOBP_INORGANIC_ION_IMPORT_ACROSS_PLASMA_MEMBRANE		13	0.4013168	1.167034	0.29411766	0.93732935	1	150	tags=23%, list=8%, signal=25%
GOBP_REGULATION_OF_CATION_CHANNEL_ACTIVITY		28	0.35002083	1.1668948	0.29411766	0.93473536	1	490	tags=43%, list=26%, signal=57%
REACTOME_INTERLEUKIN_4_AND_INTERLEUKIN_13_SIGNALING		17	0.4099265	1.1641123	0.20930232	0.94430864	1	114	tags=24%, list=6%, signal=25%
GOBP_ACTIN_MEDIATED_CELL_CONTRACTION		42	0.29735008	1.1634023	0.25581396	0.94390744	1	228	tags=26%, list=12%, signal=29%
GOBP_RESPONSE_TO_CARBOHYDRATE		38	0.31178346	1.1628578	0.1590909	0.94333816	1	154	tags=21%, list=8%, signal=22%
GOBP_ANATOMICAL_STRUCTURE_FORMATION_INVOLVED_IN_MORPHOGENE		143	0.24453087	1.1624099	0.19444445	0.94204235	1	449	tags=31%, list=24%, signal=38%
KEGG_ALZHEIMERS_DISEASE		79	0.2611343	1.161992	0.18	0.9404777	1	152	tags=23%, list=8%, signal=24%
REACTOME_SIGNALING_BY_RETINOIC_ACID		14	0.3942496	1.1617664	0.3125	0.9383625	1	374	tags=43%, list=20%, signal=53%
GOBP_UNSATURATED_FATTY_ACID_METABOLIC_PROCESS		24	0.34192637	1.1614848	0.32692307	0.93674666	1	135	tags=25%, list=7%, signal=27%
GOBP_ENDOTHELIAL_CELL_DEVELOPMENT		20	0.37480676	1.1612153	0.24193548	0.93502337	1	647	tags=55%, list=35%, signal=83%
GOBP_RESPONSE_TO_CYTOKINE		165	0.23087119	1.1609912	0.05405406	0.9328637	1	297	tags=21%, list=16%, signal=23%
GOBP_RESPONSE_TO_EXTRACELLULAR_STIMULUS		69	0.28214368	1.160962	0.19565217	0.93004644	1	186	tags=17%, list=10%, signal=19%
GOBP_POSITIVE_REGULATION_OF_CATION_CHANNEL_ACTIVITY		15	0.38768476	1.1565008	0.27272728	0.9468066	1	490	tags=47%, list=26%, signal=63%
GOBP_CELLULAR_RESPONSE_TO_DRUG		10	0.46214214	1.1564445	0.29787233	0.9440789	1	113	tags=30%, list=6%, signal=32%
GOBP_REGULATION_OF_MUSCLE_SYSTEM_PROCESS		52	0.29872903	1.155358	0.24528302	0.9459435	1	246	tags=23%, list=13%, signal=26%
GOBP_SYNAPTIC_VESICLE_RECYCLING		14	0.42592376	1.1529443	0.26923078	0.9528713	1	511	tags=43%, list=27%, signal=59%
GOBP_POSITIVE_REGULATION_OF_IMMUNE_EFFECTOR_PROCESS		25	0.3517863	1.1501616	0.23076923	0.96221596	1	401	tags=36%, list=21%, signal=45%
GOBP_REGULATION_OF_FATTY_ACID_BIOSYNTHETIC_PROCESS		11	0.41419438	1.1496631	0.2888889	0.9613004	1	350	tags=36%, list=19%, signal=44%
REACTOME_RESPONSE_TO_ELEVATED_PLATELET_CYTOSOLIC_CA2		58	0.29473582	1.1495142	0.2	0.95868665	1	490	tags=45%, list=26%, signal=59%
GOBP_CELLULAR_RESPONSE_TO_TOPOLOGICALLY_INCORRECT_PROTEIN		37	0.32452664	1.1494328	0.25531915	0.9562397	1	117	tags=19%, list=6%, signal=20%
GOBP_CYTOSOLIC_TRANSPORT		35	0.2977488	1.1492769	0.25581396	0.95407885	1	595	tags=40%, list=32%, signal=58%
GOBP_VESICLE_LOCALIZATION		43	0.302164	1.1491171	0.21621622	0.9519014	1	416	tags=35%, list=22%, signal=44%
GOBP_PROTEIN_FOLDING		82	0.24958809	1.149038	0.22727273	0.94923306	1	329	tags=27%, list=18%, signal=31%
REACTOME_THE_ROLE_OF_NEF_IN_HIV_1_REPLICATION_AND_DISEASE_PA		12	0.42893422	1.1489332	0.30769232	0.94688934	1	205	tags=25%, list=11%, signal=28%
GOBP_OLEFINIC_COMPOUND_METABOLIC_PROCESS		19	0.38503507	1.1488208	0.3	0.9445561	1	135	tags=32%, list=7%, signal=34%
GOBP_CELLULAR_RESPONSE_TO_CAMP		11	0.43633184	1.1484933	0.30612245	0.94299084	1	300	tags=36%, list=16%, signal=43%
GOBP_PEPTIDYL_SERINE_MODIFICATION		33	0.31283906	1.148308	0.19565217	0.9406473	1	609	tags=39%, list=33%, signal=57%
REACTOME_SIGNALING_BY_INTERLEUKINS		82	0.28657904	1.1462958	0.2195122	0.9456358	1	210	tags=18%, list=11%, signal=20%
GOBP_DEVELOPMENTAL_PROCESS_INVOLVED_IN_REPRODUCTION		57	0.26969683	1.1452051	0.2173913	0.94756305	1	464	tags=33%, list=25%, signal=43%

GOBP_DEVELOPMENT_OF_PRIMARY_SEXUAL_CHARACTERISTICS	11	0.41140315	1.1442897	0.2857143	0.9486122	1	227	tags=27%, list=12%, signal=31%
GOBP_REGULATION_OF_IMMUNE_EFFECTOR_PROCESS	49	0.30548793	1.1431805	0.20754717	0.95085716	1	337	tags=27%, list=18%, signal=32%
GOBP_REGULATION_OF_DEFENSE_RESPONSE_TO_VIRUS_BY_VIRUS	12	0.42893416	1.1428684	0.3275862	0.9492005	1	205	tags=25%, list=11%, signal=28%
KEGG_FATTY_ACID_METABOLISM	25	0.34842178	1.1428347	0.30769232	0.94658035	1	133	tags=28%, list=7%, signal=30%
GOBP_PROTON_TRANSMEMBRANE_TRANSPORT	51	0.29111105	1.1422606	0.2264151	0.9461234	1	292	tags=29%, list=16%, signal=34%
GOBP_CELL_KILLING	23	0.33419406	1.1418436	0.23913044	0.9451482	1	212	tags=22%, list=11%, signal=24%
GOBP_BIOLOGICAL_ADHESION	203	0.22733773	1.1417453	0.13793103	0.94289225	1	340	tags=24%, list=18%, signal=26%
GOBP_METAL_ION_EXPORT	13	0.41793904	1.1414747	0.26086956	0.9416475	1	150	tags=31%, list=8%, signal=33%
GOBP_VIRION_ASSEMBLY	13	0.41110715	1.1413754	0.26	0.9393091	1	365	tags=62%, list=20%, signal=76%
GOBP_HEART_MORPHOGENESIS	29	0.33413127	1.1411753	0.3	0.9372011	1	80	tags=17%, list=4%, signal=18%
REACTOME_PEROXISOMAL_PROTEIN_IMPORT	30	0.32704788	1.1401281	0.30952382	0.9392065	1	375	tags=37%, list=20%, signal=45%
GOBP_REGULATION_OF_DEPHOSPHORYLATION	25	0.33352667	1.1398202	0.2777778	0.93777126	1	38	tags=12%, list=2%, signal=12%
GOBP_FATTY_ACID_DERIVATIVE_METABOLIC_PROCESS	24	0.34020156	1.1390703	0.31707317	0.937753	1	513	tags=46%, list=27%, signal=62%
GOBP_MYOFIBRIL_ASSEMBLY	24	0.3389841	1.1385231	0.24489796	0.93730295	1	119	tags=21%, list=6%, signal=22%
GOBP_NEGATIVE_REGULATION_OF_IMMUNE_RESPONSE	14	0.38171032	1.1381176	0.32608697	0.9364975	1	337	tags=29%, list=18%, signal=35%
GOBP_POSITIVE_REGULATION_OF_TUMOR_NECROSIS_FACTOR_SUPERFAM	12	0.43371192	1.1380525	0.36	0.93421894	1	308	tags=25%, list=16%, signal=30%
REACTOME_POTENTIAL_THERAPEUTICS_FOR_SARS	21	0.3706156	1.1379381	0.23529412	0.9320227	1	205	tags=24%, list=11%, signal=26%
GOBP_POSITIVE_REGULATION_OF_TRANSPORT	158	0.23390867	1.1371703	0.15555556	0.93257546	1	321	tags=23%, list=17%, signal=26%
GOBP_CELLULAR_RESPONSE_TO_HORMONE_STIMULUS	89	0.25134075	1.1366291	0.15151516	0.9321415	1	538	tags=38%, list=29%, signal=51%
GOBP_NEGATIVE_REGULATION_OF_SUPRAMOLECULAR_FIBER_ORGANIZAT	37	0.3299655	1.1364068	0.23255815	0.9306823	1	146	tags=16%, list=8%, signal=17%
KEGG_LYSINE_DEGRADATION	12	0.42543507	1.1360983	0.27906978	0.929337	1	375	tags=50%, list=20%, signal=62%
GOBP_FATTY_ACID_BETA_OXIDATION_USING_ACYL_COA_OXIDASE	10	0.4474673	1.1354867	0.27450982	0.9287853	1	108	tags=30%, list=6%, signal=32%
GOBP_ACETYL_COA_METABOLIC_PROCESS	16	0.3992747	1.1352234	0.24074075	0.9273803	1	275	tags=44%, list=15%, signal=51%
GOBP_PROTEIN_AUTOPHOSPHORYLATION	25	0.32284218	1.1337106	0.21276596	0.930724	1	616	tags=44%, list=33%, signal=65%
GOBP_REPRODUCTION	97	0.2565553	1.1332511	0.20930232	0.92977506	1	272	tags=23%, list=15%, signal=25%
KEGG_HUNTINGTONS_DISEASE	86	0.2763202	1.1323388	0.31578946	0.9308946	1	341	tags=37%, list=18%, signal=43%
GOBP_CELLULAR_BIOGENIC_AMINE_METABOLIC_PROCESS	11	0.45420557	1.1322125	0.3392857	0.92897797	1	244	tags=36%, list=13%, signal=42%
GOBP_NEURON_DEATH	51	0.2908672	1.1319668	0.26086956	0.9274954	1	146	tags=18%, list=8%, signal=19%
GOBP_CELL_COMMUNICATION_BY_ELECTRICAL_COUPLING	15	0.37983644	1.1318274	0.30952382	0.9251148	1	192	tags=33%, list=10%, signal=37%
GOBP_POSITIVE_REGULATION_OF_RECEPTOR_MEDIATED_ENDOCYTOSIS	13	0.41306746	1.131793	0.2173913	0.92274344	1	254	tags=38%, list=14%, signal=44%
GOBP_MULTI_MULTICELLULAR_ORGANISM_PROCESS	26	0.33966428	1.1287826	0.3529412	0.9323673	1	228	tags=27%, list=12%, signal=30%
GOBP_NEGATIVE_REGULATION_OF_DEVELOPMENTAL_PROCESS	81	0.2538457	1.1287405	0.18604651	0.9300612	1	384	tags=30%, list=21%, signal=36%
GOBP_RESPONSE_TO_INORGANIC_SUBSTANCE	102	0.25430456	1.1285969	0.24	0.92814445	1	506	tags=39%, list=27%, signal=51%
GOBP_SENSORY_ORGAN_MORPHOGENESIS	10	0.41241327	1.1283464	0.28	0.9270902	1	44	tags=10%, list=2%, signal=10%
REACTOME_AMYLOID_FIBER_FORMATION	16	0.38993874	1.1282425	0.30952382	0.925088	1	488	tags=38%, list=26%, signal=50%
GOBP_NEGATIVE_REGULATION_OF_PEPTIDASE_ACTIVITY	38	0.30847794	1.127186	0.23404256	0.9275808	1	426	tags=37%, list=23%, signal=47%
GOBP_COLLAGEN_METABOLIC_PROCESS	16	0.37416375	1.1268141	0.31111112	0.92626524	1	78	tags=13%, list=4%, signal=13%
GOBP_POSITIVE_REGULATION_OF_LIPID_LOCALIZATION	23	0.34174618	1.1256161	0.22727273	0.92791456	1	156	tags=26%, list=8%, signal=28%
GOBP_REGULATION_OF_PHOSPHOLIPID_METABOLIC_PROCESS	11	0.42475188	1.1255937	0.31111112	0.9255604	1	149	tags=27%, list=8%, signal=29%
GOBP_ACTION_POTENTIAL	34	0.32274404	1.1255797	0.26190478	0.92311835	1	228	tags=29%, list=12%, signal=33%
GOBP_POSITIVE_REGULATION_OF_NEURON_PROJECTION_DEVELOPMENT	24	0.34032467	1.1254742	0.26086956	0.92119056	1	380	tags=38%, list=20%, signal=46%
GOBP_RESPONSE_TO_WOUNDING	119	0.2514086	1.1250737	0.18604651	0.9202823	1	410	tags=30%, list=22%, signal=36%
GOBP_MATERNAL_PROCESS_INVOLVED_IN_FEMALE_PREGNANCY	10	0.42659724	1.1248611	0.33333334	0.9186749	1	228	tags=30%, list=12%, signal=34%
GOBP_ASPARTATE_FAMILY_AMINO_ACID_METABOLIC_PROCESS	10	0.45906958	1.1248132	0.26190478	0.9164087	1	208	tags=40%, list=11%, signal=45%
GOBP_SARCOMERE_ORGANIZATION	15	0.389987	1.1234148	0.32608697	0.9195437	1	119	tags=27%, list=6%, signal=28%
GOBP_REGULATION_OF_INTRACELLULAR_TRANSPORT	73	0.25606737	1.1228247	0.22727273	0.9198126	1	316	tags=26%, list=17%, signal=30%
KEGG_COMPLEMENT_AND_COAGULATION_CASCADES	17	0.36091378	1.1220479	0.32608697	0.92050433	1	457	tags=41%, list=24%, signal=54%
GOBP_REGULATION_OF_TUBE_SIZE	23	0.33120024	1.1217353	0.27272728	0.91957855	1	146	tags=22%, list=8%, signal=23%
REACTOME_CARGO_RECOGNITION_FOR_CLATHRIN_MEDIATED_ENDOCYTOS	23	0.3561387	1.1212028	0.24193548	0.9189523	1	205	tags=17%, list=11%, signal=19%
GOBP_VESICLE_MEDIATED_TRANSPORT_BETWEEN_ENDOSOMAL_COMPART	13	0.3901223	1.1210142	0.27450982	0.917176	1	640	tags=62%, list=34%, signal=93%
GOBP_REGULATION_OF_ATPASE_ACTIVITY	28	0.30888954	1.1201204	0.30612245	0.9184817	1	171	tags=18%, list=9%, signal=19%
GOBP_REGULATION_OF_HUMORAL_IMMUNE_RESPONSE	16	0.3772325	1.1200572	0.3859649	0.9164585	1	254	tags=31%, list=14%, signal=36%
GOBP_FORMATION_OF_PRIMARY_GERM_LAYER	13	0.40981779	1.1199772	0.28846154	0.9143792	1	394	tags=54%, list=21%, signal=68%
GOBP_CELLULAR_RESPONSE_TO_OXYGEN_LEVELS	63	0.2737867	1.1196821	0.3	0.91315293	1	434	tags=30%, list=23%, signal=38%
GOBP_REGULATION_OF_INFLAMMATORY_RESPONSE	46	0.292244	1.1195966	0.23913044	0.9112542	1	453	tags=33%, list=24%, signal=42%
GOBP_RIBONUCLEOPROTEIN_COMPLEX_BIOGENESIS	56	0.27860063	1.1187433	0.25531915	0.9118864	1	433	tags=32%, list=23%, signal=41%
GOBP_REGULATION_OF_PHOSPHORUS_METABOLIC_PROCESS	187	0.22329138	1.1182727	0.20930232	0.9115482	1	309	tags=20%, list=17%, signal=22%

GOBP_REGULATION_OF_POTASSIUM_ION_TRANSPORT		20	0.34434325	1.1179177	0.2820513	0.91069967	1	232	tags=30%, list=12%, signal=34%
GOBP_REGULATION_OF_EPITHELIAL_CELL_MIGRATION		40	0.28113258	1.1178913	0.20833333	0.90850675	1	384	tags=30%, list=21%, signal=37%
GOBP_NEGATIVE_REGULATION_OF_PROTEOLYSIS		46	0.29004595	1.1172751	0.27450982	0.908304	1	426	tags=35%, list=23%, signal=44%
GOBP_RESPONSE_TO_BIOTIC_STIMULUS		190	0.21918692	1.1170095	0.21052632	0.9067654	1	337	tags=22%, list=18%, signal=24%
REACTOME_GLYOXYLATE_METABOLISM_AND_GLYCINE_DEGRADATION		14	0.40396914	1.1167483	0.37777779	0.9056177	1	349	tags=57%, list=19%, signal=70%
GOBP_LIPID_CATABOLIC_PROCESS		89	0.24631928	1.1167235	0.21428572	0.9033966	1	167	tags=18%, list=9%, signal=19%
KEGG_TRYPTOPHAN_METABOLISM		14	0.36932266	1.114212	0.275	0.91131043	1	133	tags=29%, list=7%, signal=31%
GOBP_REGULATION_OF_PROTEIN_STABILITY		67	0.27894	1.1133764	0.25	0.9124677	1	321	tags=25%, list=17%, signal=30%
GOBP_REGULATION_OF_CELLULAR_RESPONSE_TO_HEAT		16	0.38250184	1.1127537	0.27083334	0.91275424	1	192	tags=25%, list=10%, signal=28%
GOBP_RESPONSE_TO_TEMPERATURE_STIMULUS		42	0.2813915	1.1124783	0.2173913	0.9116931	1	194	tags=19%, list=10%, signal=21%
GOBP_NUCLEOSIDE_BISPHOSPHATE_BIOSYNTHETIC_PROCESS		27	0.34166571	1.1119399	0.27450982	0.911017	1	180	tags=26%, list=10%, signal=28%
REACTOME_NEURONAL_SYSTEM		56	0.26791087	1.1115001	0.23404256	0.91065544	1	270	tags=23%, list=14%, signal=26%
GOBP_CELLULAR_LIPID_CATABOLIC_PROCESS		78	0.2704854	1.1104288	0.2413793	0.9123319	1	135	tags=18%, list=7%, signal=19%
GOBP_PEROXISOME_ORGANIZATION		37	0.29622474	1.110212	0.2777778	0.91091734	1	380	tags=32%, list=20%, signal=40%
GOBP_REGULATION_OF_PROTEIN_LOCALIZATION_TO_PLASMA_MEMBRANE		24	0.3368807	1.1098031	0.39534885	0.9101611	1	493	tags=38%, list=26%, signal=50%
GOBP_NEGATIVE_REGULATION_OF_TRANSPORT		78	0.26019666	1.1090742	0.27659574	0.9109464	1	309	tags=26%, list=17%, signal=29%
GOBP_ESTABLISHMENT_OF_PROTEIN_LOCALIZATION_TO_VACUOLE		12	0.41847104	1.1089035	0.3488372	0.9091429	1	232	tags=25%, list=12%, signal=28%
GOBP_SECRETION		305	0.20542368	1.1087829	0.11428572	0.9074083	1	526	tags=35%, list=28%, signal=41%
GOBP_REGULATION_OF_CARDIAC_MUSCLE_CONTRACTION		26	0.33389527	1.1083547	0.2826087	0.9067829	1	500	tags=46%, list=27%, signal=62%
GOBP_CELLULAR_ION_HOMEOSTASIS		103	0.23897058	1.1079147	0.22857143	0.9066188	1	194	tags=17%, list=10%, signal=18%
GOBP_NEGATIVE_REGULATION_OF_MOLECULAR_FUNCTION		145	0.235796	1.1077743	0.2	0.9049962	1	434	tags=29%, list=23%, signal=35%
GOBP_CARDIAC_MUSCLE_CELL_DIFFERENTIATION		22	0.33800444	1.1075935	0.33333334	0.90350777	1	222	tags=23%, list=12%, signal=25%
GOBP_NEGATIVE_REGULATION_OF_PHOSPHORYLATION		66	0.26062688	1.1074184	0.24444444	0.9019011	1	452	tags=30%, list=24%, signal=39%
GOBP_DIVALENT_INORGANIC_CATION_HOMEOSTASIS		71	0.26334175	1.1065187	0.29545453	0.903118	1	194	tags=20%, list=10%, signal=21%
REACTOME_STRIATED_MUSCLE_CONTRACTION		19	0.34502742	1.1064428	0.28301886	0.901153	1	80	tags=21%, list=4%, signal=22%
GOBP_POLYSACCHARIDE_METABOLIC_PROCESS		18	0.3516138	1.1056604	0.2857143	0.90232635	1	309	tags=33%, list=17%, signal=40%
GOBP_PROTEIN_PHOSPHORYLATION		169	0.21410373	1.105574	0.16666667	0.9004952	1	482	tags=28%, list=26%, signal=34%
GOBP_PRODUCTION_OF_MOLECULAR_MEDIATOR_INVOLVED_IN_INFLAMMAT		11	0.4126737	1.1048753	0.38181818	0.90113425	1	91	tags=18%, list=5%, signal=19%
REACTOME_DISEASES_OF_PROGRAMMED_CELL_DEATH		11	0.39862677	1.1044729	0.3272727	0.9002388	1	182	tags=27%, list=10%, signal=30%
GOBP_NEGATIVE_REGULATION_OF_EPITHELIAL_CELL_MIGRATION		14	0.38919947	1.1041732	0.3137255	0.8990578	1	384	tags=43%, list=21%, signal=54%
GOBP_REGULATION_OF_PROTEIN_DEPHOSPHORYLATION		23	0.34749642	1.103515	0.25	0.899702	1	38	tags=13%, list=2%, signal=13%
GOBP_NEGATIVE_REGULATION_OF_BIOSYNTHETIC_PROCESS		105	0.23329137	1.1026087	0.26190478	0.900852	1	365	tags=28%, list=20%, signal=32%
GOBP_LYMPHOCYTE_MEDIATED_IMMUNITY		28	0.31275225	1.1021895	0.30555555	0.90024245	1	182	tags=18%, list=10%, signal=19%
GOBP_LONG_CHAIN_FATTY_ACID_METABOLIC_PROCESS		28	0.33029535	1.1016561	0.27083334	0.9002533	1	173	tags=29%, list=9%, signal=31%
GOBP_NEGATIVE_REGULATION_OF_SECRETION		17	0.36058226	1.101458	0.27659574	0.89922315	1	297	tags=29%, list=16%, signal=35%
GOBP_RAC_PROTEIN_SIGNAL_TRANSDUCTION		11	0.4457413	1.1004943	0.29545453	0.90081954	1	407	tags=36%, list=22%, signal=46%
GOBP_TUBE_DEVELOPMENT		120	0.22981896	1.1000054	0.275	0.9005651	1	366	tags=24%, list=20%, signal=28%
GOBP_EPITHELIUM_DEVELOPMENT		153	0.22740969	1.0984039	0.26666668	0.9042157	1	460	tags=28%, list=25%, signal=34%
GOBP_RESPONSE_TO_ORGANIC_CYCLIC_COMPOUND		137	0.22451615	1.0978118	0.26530612	0.90483034	1	502	tags=35%, list=27%, signal=44%
GOBP_NEGATIVE_REGULATION_OF_PROTEIN_CATABOLIC_PROCESS		16	0.38905612	1.097772	0.3478261	0.90301955	1	244	tags=25%, list=13%, signal=29%
GOBP_CARBOHYDRATE_TRANSPORT		17	0.35957992	1.0977678	0.33333334	0.90098184	1	406	tags=41%, list=22%, signal=52%
REACTOME_INTEGRIN_SIGNALING		11	0.39426887	1.0974694	0.38636363	0.9003764	1	533	tags=55%, list=28%, signal=76%
GOBP_POSITIVE_REGULATION_OF_PHAGOCYTOSIS		17	0.36790565	1.097454	0.33333334	0.8984114	1	453	tags=41%, list=24%, signal=54%
GOBP_REGULATION_OF_TRANSMEMBRANE_RECEPTOR_PROTEIN_SERINE_T		20	0.3470574	1.0970523	0.3181818	0.8980812	1	482	tags=45%, list=26%, signal=60%
GOBP_STEROL_TRANSPORT		18	0.36684927	1.0969801	0.3265306	0.89645225	1	146	tags=28%, list=8%, signal=30%
GOBP_NUCLEOSIDE_BISPHOSPHATE_METABOLIC_PROCESS		49	0.28390992	1.0969652	0.30769232	0.89448196	1	309	tags=31%, list=17%, signal=36%
REACTOME_GRB2_SOS_PROVIDES_LINKAGE_TO_MAPK_SIGNALING_FOR_IN		11	0.39426887	1.0967641	0.29545453	0.89324313	1	533	tags=55%, list=28%, signal=76%
GOBP_SIGNAL_RELEASE		60	0.25971755	1.0963603	0.2857143	0.8926886	1	471	tags=42%, list=25%, signal=54%
GOBP_MEMBRANE_ORGANIZATION		242	0.21266419	1.0961267	0.16666667	0.8915712	1	407	tags=26%, list=22%, signal=29%
GOBP_MEMBRANE_INVAGINATION		15	0.37776518	1.0956148	0.33962265	0.8916848	1	156	tags=27%, list=8%, signal=29%
GOBP_REGULATION_OF_PROTEIN_LOCALIZATION_TO_CELL_SURFACE		11	0.42069262	1.0952717	0.26086956	0.8908845	1	40	tags=18%, list=2%, signal=18%
GOBP_REGULATION_OF_ANATOMICAL_STRUCTURE_MORPHOGENESIS		184	0.22181338	1.0952524	0.25641027	0.8889517	1	385	tags=25%, list=21%, signal=28%
GOBP_REGULATION_OF_RELEASE_OF_CYTOCHROME_C_FROM_MITOCHOND		11	0.3844558	1.0951862	0.33333334	0.88711274	1	158	tags=27%, list=8%, signal=30%
GOBP_PROTEIN_STABILIZATION		55	0.27400768	1.094642	0.3529412	0.8872894	1	352	tags=29%, list=19%, signal=35%
GOBP_REGULATION_OF_SUPRAMOLECULAR_FIBER_ORGANIZATION		84	0.2473538	1.0930928	0.25	0.8908069	1	352	tags=21%, list=19%, signal=25%
KEGG_MAPK_SIGNALING_PATHWAY		32	0.33055612	1.0926051	0.33333334	0.8907246	1	352	tags=28%, list=19%, signal=34%
GOBP_CYTOSKELETON_DEPENDENT_INTRACELLULAR_TRANSPORT		31	0.30624124	1.0926038	0.3478261	0.8887869	1	583	tags=45%, list=31%, signal=65%

GOBP_REGULATION_OF_CELLULAR_KETONE_METABOLIC_PROCESS	57	0.2689678	1.0925677	0.32608697	0.88699746	1	385	tags=26%, list=21%, signal=32%
GOBP_MITOCHONDRIAL_RNA_METABOLIC_PROCESS	17	0.37056237	1.0895271	0.33333334	0.89653426	1	389	tags=35%, list=21%, signal=44%
GOBP_SEX_DIFFERENTIATION	13	0.38744414	1.0889385	0.32608697	0.8966911	1	227	tags=23%, list=12%, signal=26%
GOBP_REGULATION_OF_OXIDOREDUCTASE_ACTIVITY	26	0.33372062	1.0886886	0.34146342	0.8955102	1	448	tags=42%, list=24%, signal=55%
GOBP_CIRCULATORY_SYSTEM_PROCESS	118	0.23514567	1.0867584	0.24390244	0.90134054	1	194	tags=18%, list=10%, signal=19%
REACTOME_PROTEIN_LOCALIZATION	83	0.24901469	1.0865556	0.27906978	0.9002931	1	271	tags=25%, list=14%, signal=28%
GOBP_REGULATION_OF_BIOMINERALIZATION	10	0.44776756	1.0861815	0.4117647	0.89966875	1	434	tags=50%, list=23%, signal=65%
GOBP_REGULATION_OF_MONOOXYGENASE_ACTIVITY	14	0.40004852	1.0849689	0.40816328	0.90222335	1	448	tags=50%, list=24%, signal=65%
GOBP_ESTABLISHMENT_OF_PROTEIN_LOCALIZATION	409	0.19749217	1.0844063	0.11111111	0.90233976	1	428	tags=26%, list=23%, signal=27%
GOBP_CELLULAR_MONOVALENT_INORGANIC_CATION_HOMEOSTASIS	21	0.33342338	1.0838308	0.32692307	0.9025985	1	150	tags=19%, list=8%, signal=20%
GOBP_HOMEOSTATIC_PROCESS	273	0.21160188	1.0835168	0.2	0.9021874	1	154	tags=14%, list=8%, signal=13%
REACTOME_TRAFFICKING_OF_AMPA_RECEPTORS	10	0.42200753	1.0827078	0.33962265	0.9032538	1	205	tags=30%, list=11%, signal=34%
GOBP_PROTEIN_TARGETING_TO_VACUOLE	11	0.43637827	1.0813054	0.32258064	0.9067125	1	232	tags=27%, list=12%, signal=31%
GOBP_NEURAL_PRECURSOR_CELL_PROLIFERATION	13	0.39283663	1.0791457	0.34	0.91277736	1	18	tags=15%, list=1%, signal=15%
GOBP_POSITIVE_REGULATION_OF_WOUND_HEALING	16	0.36530805	1.0789917	0.2888889	0.91162	1	390	tags=44%, list=21%, signal=55%
GOBP_NEGATIVE_REGULATION_OF_HYDROLASE_ACTIVITY	62	0.26340115	1.0788158	0.26530612	0.91033304	1	434	tags=31%, list=23%, signal=39%
GOBP_REGULATION_OF_CHOLESTEROL_METABOLIC_PROCESS	13	0.38817492	1.0734708	0.35555556	0.9282226	1	546	tags=62%, list=29%, signal=86%
GOBP_REGULATION_OF_ACTIN_FILAMENT_BASED_PROCESS	91	0.23596328	1.0734222	0.31111112	0.9265229	1	228	tags=15%, list=12%, signal=17%
GOBP_STRIATED_MUSCLE_CELL_DEVELOPMENT	31	0.3085954	1.0727961	0.3243243	0.9266452	1	282	tags=29%, list=15%, signal=34%
GOBP_POSITIVE_REGULATION_OF_CATABOLIC_PROCESS	74	0.257993	1.0723253	0.3409091	0.9265409	1	392	tags=26%, list=21%, signal=31%
GOBP_RESPONSE_TO_HORMONE	134	0.22100611	1.0723108	0.32352942	0.92466795	1	409	tags=28%, list=22%, signal=33%
GOBP_REGULATION_OF_RESPONSE_TO_STRESS	196	0.2082232	1.0710199	0.25	0.9274698	1	467	tags=27%, list=25%, signal=32%
GOBP_PEPTIDE_SECRETION	69	0.24956742	1.0699787	0.35416666	0.92927325	1	471	tags=38%, list=25%, signal=49%
GOBP_CELL_MIGRATION	199	0.21586967	1.0698166	0.29411766	0.9276459	1	402	tags=25%, list=21%, signal=28%
KEGG_GLUTATHIONE_METABOLISM	14	0.37574637	1.0689206	0.33333334	0.92938805	1	475	tags=57%, list=25%, signal=76%
GOBP_NEURON_PROJECTION_GUIDANCE	35	0.292825	1.068752	0.3809524	0.92806363	1	406	tags=29%, list=22%, signal=36%
GOBP_CARDIAC_CELL_DEVELOPMENT	20	0.35317656	1.0686783	0.325	0.9263688	1	222	tags=25%, list=12%, signal=28%
GOBP_CARDIAC_MUSCLE_TISSUE_DEVELOPMENT	44	0.28953022	1.06822	0.29787233	0.9261382	1	274	tags=25%, list=15%, signal=29%
GOBP_PROTEIN_LOCALIZATION_TO_NUCLEUS	35	0.3056847	1.0677887	0.3617021	0.9260389	1	244	tags=26%, list=13%, signal=29%
GOBP_CELLULAR_KETONE_METABOLIC_PROCESS	82	0.25186637	1.0675348	0.325	0.92483914	1	353	tags=24%, list=19%, signal=29%
GOBP_PHAGOCYTOSIS	67	0.25942826	1.0672251	0.34210527	0.9242965	1	409	tags=28%, list=22%, signal=35%
GOBP_TUBE_MORPHOGENESIS	108	0.23655127	1.0659802	0.27659574	0.92709756	1	366	tags=26%, list=20%, signal=30%
GOBP_RRNA_METABOLIC_PROCESS	24	0.33119914	1.0659086	0.3	0.9256379	1	646	tags=54%, list=35%, signal=82%
GOBP_ENTRY_INTO_HOST	36	0.29703772	1.0656259	0.375	0.92502606	1	358	tags=33%, list=19%, signal=40%
GOBP_LIPID_MODIFICATION	69	0.26080465	1.0656224	0.38297874	0.9231305	1	167	tags=19%, list=9%, signal=20%
GOBP_NEGATIVE_REGULATION_OF_CELLULAR_PROTEIN_LOCALIZATION	21	0.3309127	1.0654813	0.37209302	0.92166305	1	493	tags=38%, list=26%, signal=51%
GOBP_FIBROBLAST_MIGRATION	10	0.41306975	1.0647808	0.3529412	0.9223615	1	17	tags=10%, list=1%, signal=10%
REACTOME_MAP2K_AND_MAPK_ACTIVATION	18	0.35462183	1.0646286	0.35849056	0.92105216	1	256	tags=28%, list=14%, signal=32%
REACTOME_PLATELET_AGGREGATION_PLUG_FORMATION	11	0.39426887	1.0637462	0.33333334	0.9223252	1	533	tags=55%, list=28%, signal=76%
REACTOME_PROCESSING_OF_SMDT1	12	0.38210508	1.0637193	0.375	0.92053396	1	227	tags=33%, list=12%, signal=38%
GOBP_POSITIVE_REGULATION_OF_ACTIN_FILAMENT_BUNDLE_ASSEMBLY	15	0.35162473	1.0625203	0.3617021	0.92294544	1	18	tags=13%, list=1%, signal=13%
GOBP_RESPONSE_TO_KETONE	25	0.32359204	1.0623505	0.31914893	0.9218819	1	604	tags=60%, list=32%, signal=87%
GOBP_PURINE_CONTAINING_COMPOUND_BIOSYNTHETIC_PROCESS	72	0.24701925	1.061673	0.35555556	0.92238504	1	296	tags=26%, list=16%, signal=30%
GOBP_CARDIOCYTE_DIFFERENTIATION	23	0.33648244	1.061494	0.41666666	0.92107034	1	222	tags=22%, list=12%, signal=24%
GOBP_LOCOMOTION	226	0.2089359	1.0604194	0.31707317	0.9234986	1	406	tags=25%, list=22%, signal=28%
GOBP_AMYLOID_PRECURSOR_PROTEIN_METABOLIC_PROCESS	14	0.3795278	1.0598747	0.37209302	0.9235161	1	434	tags=36%, list=23%, signal=46%
GOBP_EXOCYTOSIS	238	0.20723732	1.0591102	0.30555555	0.9246463	1	526	tags=34%, list=28%, signal=42%
REACTOME_RAC3_GTPASE_CYCLE	18	0.3551515	1.0587623	0.40816328	0.92427284	1	253	tags=33%, list=14%, signal=38%
GOBP_REGULATION_OF_CARDIAC_MUSCLE_CELL_ACTION_POTENTIAL	13	0.38656107	1.0584892	0.4	0.92352027	1	563	tags=54%, list=30%, signal=76%
GOBP_INORGANIC_ION_TRANSMEMBRANE_TRANSPORT	143	0.21643627	1.0571572	0.2857143	0.92600465	1	244	tags=20%, list=13%, signal=22%
GOBP_CATION_TRANSPORT	186	0.2110536	1.0568091	0.275	0.92558324	1	321	tags=23%, list=17%, signal=25%
REACTOME_PEROXISOMAL_LIPID_METABOLISM	16	0.34294936	1.0564126	0.41860464	0.925337	1	167	tags=25%, list=9%, signal=27%
GOBP_RESPONSE_TO_ALCOHOL	32	0.30283427	1.0562775	0.4	0.9238884	1	485	tags=47%, list=26%, signal=62%
GOBP_NEGATIVE_REGULATION_OF_PROTEIN_METABOLIC_PROCESS	141	0.21920034	1.0558885	0.31111112	0.92355	1	434	tags=28%, list=23%, signal=34%
GOBP_PROTEIN_LOCALIZATION_TO_PLASMA_MEMBRANE	72	0.24840862	1.0558734	0.3478261	0.9218065	1	518	tags=36%, list=28%, signal=48%
GOBP_MUSCLE_CELL_MIGRATION	14	0.3649029	1.0542698	0.42	0.92614174	1	328	tags=29%, list=18%, signal=34%
KEGG_PYRIMIDINE_METABOLISM	11	0.37402466	1.0537901	0.3962264	0.92602277	1	128	tags=27%, list=7%, signal=29%

GOBP_REGULATION_OF_TRANS_SYNAPTIC_SIGNALING		44	0.25774086	1.053323	0.27083334	0.9257045	1	533	tags=41%, list=28%, signal=56%
GOBP_NEGATIVE_REGULATION_OF_CYTOKINE_PRODUCTION		30	0.3057653	1.0532185	0.3783784	0.92424536	1	297	tags=23%, list=16%, signal=27%
GOBP_RESPONSE_TO_MONOSACCHARIDE		33	0.30824885	1.0527539	0.4	0.9244851	1	154	tags=21%, list=8%, signal=23%
GOBP_REGULATION_OF_LYASE_ACTIVITY		11	0.378021	1.0519962	0.38	0.9257407	1	35	tags=18%, list=2%, signal=18%
REACTOME_NERVOUS_SYSTEM_DEVELOPMENT		193	0.21442544	1.0519751	0.36363637	0.9240429	1	438	tags=30%, list=23%, signal=35%
REACTOME_INTERFERON_SIGNALING		22	0.3145723	1.0507333	0.37777779	0.92723334	1	232	tags=18%, list=12%, signal=21%
REACTOME_INTRA_GOLGI_TRAFFIC		11	0.3678619	1.0502764	0.37777779	0.92697054	1	171	tags=18%, list=9%, signal=20%
GOBP_REGULATION_OF_LIPID_LOCALIZATION		33	0.28733915	1.0497216	0.35714287	0.92732537	1	156	tags=21%, list=8%, signal=23%
GOBP_CHEMICAL_HOMEOSTASIS		177	0.21796952	1.0494598	0.38	0.9265914	1	154	tags=15%, list=8%, signal=14%
GOBP_DEFENSE_RESPONSE_TO_OTHER_ORGANISM		140	0.22629379	1.0478884	0.3030303	0.9309869	1	337	tags=21%, list=18%, signal=24%
REACTOME_INFLUENZA_INFECTION		72	0.23454759	1.047811	0.33333334	0.92944545	1	433	tags=36%, list=23%, signal=45%
GOBP_GLAND_DEVELOPMENT		50	0.27577764	1.0478039	0.3902439	0.92768925	1	460	tags=32%, list=25%, signal=41%
GOBP_ENDOTHELIAL_CELL_MIGRATION		40	0.28524905	1.0475394	0.45	0.92703897	1	366	tags=30%, list=20%, signal=36%
REACTOME_P130CAS_LINKAGE_TO_MAPK_SIGNALING_FOR_INTEGRINS		10	0.40970838	1.0460624	0.34615386	0.93108284	1	533	tags=60%, list=28%, signal=83%
GOBP_PROTEIN_LOCALIZATION_TO_CELL_CELL_JUNCTION		10	0.40138066	1.046026	0.35185185	0.9295273	1	111	tags=20%, list=6%, signal=21%
GOBP_ALPHA_AMINO_ACID_CATABOLIC_PROCESS		24	0.3034941	1.0457476	0.41304347	0.9288012	1	353	tags=38%, list=19%, signal=46%
GOBP_PEROXISOMAL_TRANSPORT		33	0.2989689	1.0454266	0.38	0.9284472	1	375	tags=33%, list=20%, signal=41%
GOBP_REGULATION_OF_ENDOCYTOSIS		49	0.25788283	1.0451833	0.3392857	0.9274416	1	254	tags=22%, list=14%, signal=25%
GOBP_CELLULAR_COMPONENT_ASSEMBLY_INVOLVED_IN_MORPHOGENESI		29	0.28723854	1.0446045	0.3125	0.92747176	1	119	tags=17%, list=6%, signal=18%
GOBP_POSITIVE_REGULATION_OF_OXIDOREDUCTASE_ACTIVITY		11	0.3823384	1.0444962	0.35714287	0.92620033	1	275	tags=36%, list=15%, signal=42%
GOBP_ORGANIC_HYDROXY_COMPOUND_TRANSPORT		26	0.2980097	1.0441381	0.33333334	0.9253941	1	324	tags=31%, list=17%, signal=37%
KEGG_ARRHYTHMOGENIC_RIGHT_VENTRICULAR_CARDIOMYOPATHY_ARVC		33	0.28420582	1.0420932	0.3809524	0.9323292	1	246	tags=33%, list=13%, signal=38%
GOBP_CELLULAR_LIPID_METABOLIC_PROCESS		212	0.20313486	1.0409955	0.27906978	0.93441415	1	523	tags=34%, list=28%, signal=42%
GOBP_LYSOSOMAL_TRANSPORT		22	0.30608037	1.0405566	0.31578946	0.934197	1	365	tags=32%, list=20%, signal=39%
GOBP_REGULATION_OF_ANION_TRANSMEMBRANE_TRANSPORT		20	0.33472425	1.0405325	0.41304347	0.9324509	1	156	tags=20%, list=8%, signal=22%
GOBP_MUCOPOLYSACCHARIDE_METABOLIC_PROCESS		13	0.40372795	1.0401038	0.4117647	0.9321561	1	645	tags=46%, list=34%, signal=70%
GOBP_GUANOSINE_CONTAINING_COMPOUND_METABOLIC_PROCESS		14	0.38931647	1.0393249	0.4473684	0.9328678	1	347	tags=36%, list=19%, signal=44%
GOBP_NUCLEOSIDE_PHOSPHATE_BIOSYNTHETIC_PROCESS		83	0.24639125	1.0384576	0.36956522	0.934324	1	296	tags=24%, list=16%, signal=27%
REACTOME_ORGANELLE_BIOGENESIS_AND_MAINTENANCE		60	0.25951612	1.0381027	0.38297874	0.93368113	1	219	tags=22%, list=12%, signal=24%
GOBP_RESPONSE_TO_PEPTIDE_HORMONE		74	0.23711483	1.0374981	0.35	0.9336259	1	471	tags=34%, list=25%, signal=43%
GOBP_REGULATION_OF_RESPONSE_TO_EXTERNAL_STIMULUS		148	0.21781322	1.0363953	0.36363637	0.9359814	1	462	tags=26%, list=25%, signal=31%
GOBP_REGULATION_OF_ION_TRANSPORT		207	0.20477799	1.0359719	0.34042552	0.93575174	1	365	tags=25%, list=20%, signal=28%
GOBP_ADENYLATE_CYCLASE_ACTIVATING_G_PROTEIN_COUPLED_RECEPT(		12	0.38554692	1.035935	0.43589744	0.9342857	1	128	tags=25%, list=7%, signal=27%
GOBP_REGULATION_OF_STEROID_METABOLIC_PROCESS		18	0.34395635	1.034082	0.3809524	0.9397783	1	146	tags=22%, list=8%, signal=24%
GOBP_NOTCH_SIGNALING_PATHWAY		11	0.39120913	1.0338095	0.37777779	0.9390635	1	548	tags=55%, list=29%, signal=77%
GOBP_VIRAL_LIFE_CYCLE		66	0.24657562	1.0327157	0.44	0.94109225	1	365	tags=30%, list=20%, signal=36%
GOBP_RESPONSE_TO_ALKALOID		20	0.3230245	1.0324053	0.42857143	0.9404648	1	58	tags=20%, list=3%, signal=20%
GOBP_NEGATIVE_REGULATION_OF_RESPONSE_TO_EXTERNAL_STIMULUS		49	0.26231748	1.0321041	0.3488372	0.93961185	1	462	tags=33%, list=25%, signal=42%
GOBP_TRANSPORT_ALONG_MICROTUBULE		26	0.31393516	1.031831	0.37254903	0.9388063	1	583	tags=42%, list=31%, signal=61%
GOBP_PROTEIN_DEPOLYMERIZATION		23	0.3419721	1.031658	0.4489796	0.9377974	1	72	tags=17%, list=4%, signal=18%
GOBP_TOLL_LIKE_RECEPTOR_SIGNALING_PATHWAY		23	0.30643433	1.0306224	0.35714287	0.93953216	1	482	tags=30%, list=26%, signal=40%
GOBP_POSITIVE_REGULATION_OF_COLD_INDUCED_THERMOGENESIS		15	0.37412235	1.0296218	0.3888889	0.9416635	1	131	tags=33%, list=7%, signal=36%
GOBP_ACTIN_FILAMENT_DEPOLYMERIZATION		21	0.2898342	1.0292494	0.34146342	0.94125056	1	66	tags=14%, list=4%, signal=15%
GOBP_MICROTUBULE_BASED_TRANSPORT		26	0.31393516	1.0291477	0.39130434	0.93984985	1	583	tags=42%, list=31%, signal=61%
REACTOME_DEVELOPMENTAL_BIOLOGY		207	0.2001536	1.0290796	0.5	0.9384514	1	438	tags=29%, list=23%, signal=34%
GOBP_REGULATION_OF_LIPID_BIOSYNTHETIC_PROCESS		34	0.29769123	1.0289825	0.4347826	0.9370623	1	156	tags=18%, list=8%, signal=19%
GOBP_FC_RECEPTOR_MEDIATED_STIMULATORY_SIGNALING_PATHWAY		26	0.28022793	1.0287273	0.36	0.9360681	1	337	tags=23%, list=18%, signal=28%
GOBP_CARDIAC_MUSCLE_CELL_ACTION_POTENTIAL_INVOLVED_IN_CONTRA		16	0.3212062	1.0286982	0.33333334	0.9345045	1	320	tags=31%, list=17%, signal=37%
KEGG_APOPTOSIS		10	0.41868415	1.0286878	0.4090909	0.9328792	1	397	tags=50%, list=21%, signal=63%
GOBP_G_PROTEIN_COUPLED_RECEPTOR_SIGNALING_PATHWAY		66	0.23581934	1.0267155	0.40677965	0.93922937	1	480	tags=33%, list=26%, signal=43%
GOBP_POSITIVE_REGULATION_OF_INTRACELLULAR_SIGNAL_TRANSDUCTIO		113	0.22143692	1.0254245	0.4090909	0.9418802	1	269	tags=19%, list=14%, signal=20%
GOBP_PRODUCTION_OF_MOLECULAR_MEDIATOR_OF_IMMUNE_RESPONSE		15	0.3685913	1.0242101	0.46938777	0.94469994	1	227	tags=33%, list=12%, signal=38%
GOBP_TRANSFERRIN_TRANSPORT		13	0.3634321	1.0241704	0.4117647	0.94322306	1	407	tags=38%, list=22%, signal=49%
GOBP_MITOCHONDRIAL_FUSION		15	0.3710177	1.023718	0.3653846	0.9429294	1	380	tags=40%, list=20%, signal=50%
GOBP_CELLULAR_MODIFIED_AMINO_ACID_METABOLIC_PROCESS		46	0.27432546	1.023473	0.4047619	0.94211507	1	289	tags=28%, list=15%, signal=33%
KEGG_CHEMOKINE_SIGNALING_PATHWAY		28	0.3043114	1.0233917	0.42222223	0.94074285	1	30	tags=11%, list=2%, signal=11%
REACTOME_CELLULAR_RESPONSE_TO_HEAT_STRESS		25	0.30555907	1.022679	0.42	0.9418359	1	93	tags=20%, list=5%, signal=21%

GOBP_ADAPTIVE_IMMUNE_RESPONSE_BASED_ON_SOMATIC_RECOMBINATI		28	0.2894934	1.0207835	0.4509804	0.947125	1	297	tags=21%, list=16%, signal=25%
GOBP_HOMOPHILIC_CELL_ADHESION_VIA_PLASMA_MEMBRANE_ADHESION		11	0.40708238	1.0199885	0.43636364	0.9480214	1	338	tags=45%, list=18%, signal=55%
GOBP_REGULATION_OF_CALCIUM_ION_TRANSPORT_INTO_CYTOSOL		18	0.33195874	1.0188253	0.37777779	0.95038074	1	478	tags=50%, list=26%, signal=67%
REACTOME_SIGNALING_BY_NTRKS		23	0.2985296	1.0187888	0.46341464	0.94889617	1	242	tags=22%, list=13%, signal=25%
GOBP_NEGATIVE_REGULATION_OF_CYSTEINE_TYPE_ENDOPEPTIDASE_ACT		14	0.34727487	1.0178059	0.39534885	0.9504975	1	300	tags=36%, list=16%, signal=42%
GOBP_CATION_TRANSMEMBRANE_TRANSPORT		146	0.21733184	1.0177038	0.42857143	0.94933087	1	244	tags=20%, list=13%, signal=21%
GOBP_REGULATION_OF_MULTICELLULAR_ORGANISMAL_DEVELOPMENT		136	0.2188879	1.0174598	0.38	0.9484587	1	409	tags=26%, list=22%, signal=31%
GOBP_NUCLEOSIDE_MONOPHOSPHATE_BIOSYNTHETIC_PROCESS		11	0.3924342	1.0160713	0.4	0.95141804	1	661	tags=55%, list=35%, signal=84%
GOBP_RIBOSOME_ASSEMBLY		25	0.30169234	1.0160701	0.53061223	0.9497663	1	360	tags=32%, list=19%, signal=39%
GOBP_CELL_REDOX_HOMEOSTASIS		15	0.3388125	1.0151094	0.375	0.9511474	1	468	tags=47%, list=25%, signal=62%
GOBP_AMYLOID_PRECURSOR_PROTEIN_CATABOLIC_PROCESS		12	0.40795666	1.0148469	0.46938777	0.9503431	1	434	tags=42%, list=23%, signal=54%
KEGG_PROSTATE_CANCER		11	0.39245	1.014823	0.40816328	0.9487677	1	38	tags=18%, list=2%, signal=18%
GOBP_VACUOLAR_TRANSPORT		29	0.27410328	1.0143988	0.52380955	0.94825864	1	365	tags=28%, list=20%, signal=34%
GOBP_HUMORAL_IMMUNE_RESPONSE_MEDIATED_BY_CIRCULATING_IMMUN		11	0.393583	1.0139567	0.4390244	0.94818556	1	182	tags=27%, list=10%, signal=30%
GOBP_BARBED_END_ACTIN_FILAMENT_CAPPING		10	0.3777531	1.0121298	0.5	0.9526952	1	66	tags=20%, list=4%, signal=21%
GOBP_NEGATIVE_REGULATION_OF_CATALYTIC_ACTIVITY		114	0.21740404	1.011605	0.35135135	0.95270056	1	434	tags=28%, list=23%, signal=34%
GOBP_POSITIVE_REGULATION_OF_INTRACELLULAR_TRANSPORT		47	0.2672025	1.0112901	0.4347826	0.95251685	1	237	tags=23%, list=13%, signal=26%
GOBP_RESPONSE_TO_FATTY_ACID		12	0.36610168	1.0103929	0.45652175	0.9543335	1	520	tags=58%, list=28%, signal=80%
KEGG_ADHERENS_JUNCTION		17	0.32332253	1.0095727	0.40425533	0.95564663	1	209	tags=24%, list=11%, signal=26%
REACTOME_RRNA_PROCESSING		66	0.23531824	1.00917	0.4878049	0.9553695	1	433	tags=36%, list=23%, signal=46%
GOBP_NEGATIVE_REGULATION_OF_PHOSPHORUS_METABOLIC_PROCESS		75	0.24383818	1.0088998	0.35897437	0.95441407	1	452	tags=29%, list=24%, signal=37%
GOBP_RESPONSE_TO_ABIOTIC_STIMULUS		195	0.20839325	1.0080569	0.46153846	0.9557273	1	150	tags=12%, list=8%, signal=12%
REACTOME_MHC_CLASS_II_ANTIGEN_PRESENTATION		36	0.2821566	1.0059646	0.47058824	0.9616967	1	205	tags=19%, list=11%, signal=21%
GOBP_CELL_MORPHOGENESIS		152	0.20363237	1.0048484	0.44444445	0.9638515	1	457	tags=29%, list=24%, signal=35%
GOBP_MYELOID_CELL_DEVELOPMENT		10	0.38133323	1.0028428	0.41666666	0.9696941	1	123	tags=20%, list=7%, signal=21%
GOBP_NEUROMUSCULAR_PROCESS		11	0.37790662	1.002637	0.525	0.9687196	1	678	tags=64%, list=36%, signal=99%
GOBP_NEGATIVE_REGULATION_OF_VASCULATURE_DEVELOPMENT		20	0.33188906	1.0022303	0.47727272	0.9685295	1	434	tags=40%, list=23%, signal=52%
GOBP_REGULATION_OF_AMYLOID_PRECURSOR_PROTEIN_CATABOLIC_PRO		12	0.40795666	1.0009359	0.4	0.9715105	1	434	tags=42%, list=23%, signal=54%
GOBP_RESPONSE_TO_GROWTH_FACTOR		76	0.2360179	1.0008512	0.45454547	0.9701372	1	439	tags=30%, list=23%, signal=38%
GOBP_CELLULAR_MODIFIED_AMINO_ACID_CATABOLIC_PROCESS		10	0.38997698	1.0003875	0.36363637	0.97015226	1	58	tags=20%, list=3%, signal=21%
GOBP_REGULATION_OF_CELL_PROJECTION_ASSEMBLY		37	0.26929083	0.99956584	0.46666667	0.9714731	1	539	tags=38%, list=29%, signal=52%
GOBP_ESTABLISHMENT_OR_MAINTENANCE_OF_BIPOLAR_CELL_POLARITY		11	0.36924538	0.99950874	0.4528302	0.9700429	1	539	tags=64%, list=29%, signal=89%
GOBP_REGULATION_OF_PRODUCTION_OF_MOLECULAR_MEDIATOR_OF_IMM		12	0.36209956	0.9971404	0.4871795	0.97675174	1	113	tags=25%, list=6%, signal=26%
KEGG_RENAL_CELL_CARCINOMA		11	0.4034566	0.9971202	0.4893617	0.9751686	1	14	tags=9%, list=1%, signal=9%
GOBP_REGULATION_OF_PROTEIN_DEPOLYMERIZATION		20	0.3145294	0.9946764	0.59090906	0.982536	1	66	tags=15%, list=4%, signal=15%
GOBP_NEGATIVE_REGULATION_OF_TRANSPORTER_ACTIVITY		15	0.34887677	0.99449897	0.5121951	0.98156226	1	242	tags=40%, list=13%, signal=46%
GOBP_CELL_POPULATION_PROLIFERATION		168	0.2059469	0.9939018	0.4473684	0.9821141	1	371	tags=23%, list=20%, signal=26%
GOBP_RUFFLE_ASSEMBLY		12	0.3572733	0.9936716	0.48979592	0.9811035	1	384	tags=33%, list=21%, signal=42%
GOBP_RESPONSE_TO_HEAT		30	0.28575304	0.9931037	0.4347826	0.9814129	1	194	tags=20%, list=10%, signal=22%
GOBP_ORGANIC_ANION_TRANSPORT		45	0.26964256	0.9928111	0.41304347	0.98071873	1	311	tags=27%, list=17%, signal=31%
GOBP_POST_TRANSLATIONAL_PROTEIN_MODIFICATION		88	0.22317994	0.9916371	0.48076922	0.98327255	1	426	tags=30%, list=23%, signal=36%
GOBP_LIPID_METABOLIC_PROCESS		253	0.19324812	0.99113935	0.42424244	0.9837608	1	523	tags=33%, list=28%, signal=40%
GOBP_REGULATION_OF_STEROID_BIOSYNTHETIC_PROCESS		11	0.38063452	0.9904933	0.47058824	0.98427135	1	600	tags=64%, list=32%, signal=93%
REACTOME_TRANSPORT_OF_SMALL_MOLECULES		150	0.20710975	0.99013317	0.5208333	0.9839188	1	234	tags=19%, list=13%, signal=20%
GOBP_IRON_SULFUR_CLUSTER_ASSEMBLY		10	0.395997	0.990044	0.44230768	0.98266417	1	387	tags=40%, list=21%, signal=50%
GOBP_DNA_CONFORMATION_CHANGE		17	0.3331889	0.98957	0.42105263	0.98288625	1	585	tags=47%, list=31%, signal=68%
GOBP_AMINOGLYCAN_BIOSYNTHETIC_PROCESS		12	0.3550279	0.9895338	0.38297874	0.9814947	1	554	tags=42%, list=30%, signal=59%
GOBP_CELL_CELL_SIGNALING		187	0.19424027	0.98901296	0.47826087	0.98147464	1	340	tags=22%, list=18%, signal=25%
GOBP_RESPONSE_TO_TRANSFORMING_GROWTH_FACTOR_BETA		26	0.28381962	0.9876537	0.4318182	0.9851544	1	482	tags=42%, list=26%, signal=56%
GOBP_TISSUE_MORPHOGENESIS		103	0.22198887	0.98721594	0.42857143	0.9849439	1	233	tags=17%, list=12%, signal=19%
GOBP_REGULATION_OF_PROTEASOMAL_PROTEIN_CATABOLIC_PROCESS		32	0.2764684	0.98662674	0.48	0.98569715	1	146	tags=16%, list=8%, signal=17%
GOBP_CELL_PART_MORPHOGENESIS		100	0.20952313	0.9863601	0.46153846	0.9849464	1	407	tags=26%, list=22%, signal=31%
REACTOME_RHOB_GTPASE_CYCLE		18	0.32309824	0.98577553	0.47916666	0.98539394	1	270	tags=28%, list=14%, signal=32%
GOBP_POSITIVE_REGULATION_OF_CALCIUM_ION_TRANSPORT		18	0.3255437	0.98576516	0.475	0.9838494	1	475	tags=39%, list=25%, signal=52%
GOBP_AMINOGLYCAN_METABOLIC_PROCESS		17	0.32195076	0.9854804	0.4716981	0.9832102	1	554	tags=35%, list=30%, signal=50%
KEGG_AXON_GUIDANCE		23	0.29752004	0.98307693	0.5416667	0.98970735	1	283	tags=17%, list=15%, signal=20%
GOBP_POSITIVE_REGULATION_OF_SIGNALING		207	0.1957412	0.9826949	0.4878049	0.98947	1	256	tags=16%, list=14%, signal=17%

GOBP_PLASMA_MEMBRANE_ORGANIZATION		28	0.2917579	0.9816374	0.5283019	0.99133193	1	194	tags=25%, list=10%, signal=27%
REACTOME_RAB_GERANYLGERANYLATION		21	0.3035529	0.9816091	0.5208333	0.9898298	1	518	tags=43%, list=28%, signal=59%
GOBP_REGULATION_OF_TRANSPORT		283	0.19079101	0.98149264	0.58536583	0.9886993	1	324	tags=22%, list=17%, signal=22%
GOBP_EPIDERMAL_CELL_DIFFERENTIATION		18	0.34698072	0.98123336	0.509434	0.98793805	1	500	tags=44%, list=27%, signal=60%
GOBP_REGULATION_OF_CELLULAR_RESPONSE_TO_STRESS		88	0.22549969	0.9808497	0.5945946	0.9877023	1	352	tags=23%, list=19%, signal=27%
GOBP_VASCULAR_ENDOTHELIAL_GROWTH_FACTOR_RECEPTOR_SIGNALIN(		23	0.28601277	0.98077106	0.48	0.9862542	1	434	tags=35%, list=23%, signal=45%
GOBP_TRANSMEMBRANE_RECEPTOR_PROTEIN_SERINE_THREONINE_KINAS		26	0.26965526	0.98011047	0.52380955	0.986737	1	482	tags=38%, list=26%, signal=51%
GOBP_POSITIVE_REGULATION_OF_PRODUCTION_OF_MOLECULAR_MEDIAT(		10	0.41684666	0.97938	0.47916666	0.98784065	1	113	tags=30%, list=6%, signal=32%
GOBP_SEQUESTERING_OF_CALCIUM_ION		23	0.3061838	0.9792154	0.5102041	0.98682725	1	478	tags=43%, list=26%, signal=58%
GOBP_GLYCEROLIPID_METABOLIC_PROCESS		74	0.23036602	0.97791076	0.5263158	0.9895336	1	511	tags=36%, list=27%, signal=48%
GOBP_NEGATIVE_REGULATION_OF_NEURON_DEATH		29	0.29349792	0.9777647	0.45652175	0.98845476	1	609	tags=48%, list=33%, signal=70%
GOBP_POSITIVE_REGULATION_OF_LIPID_TRANSPORT		16	0.32718614	0.9777294	0.54	0.98710066	1	146	tags=25%, list=8%, signal=27%
GOBP_RESPONSE_TO_PEPTIDE		86	0.22995517	0.9774811	0.54285717	0.9864757	1	557	tags=38%, list=30%, signal=52%
GOBP_PROTEIN_LOCALIZATION_TO_CELL_PERIPHERY		78	0.22651872	0.97738785	0.63461536	0.9853858	1	518	tags=36%, list=28%, signal=48%
REACTOME_GABA_B_RECEPTOR_ACTIVATION		10	0.36562687	0.97646976	0.44186047	0.987269	1	30	tags=20%, list=2%, signal=20%
GOBP_CELLULAR_RESPONSE_TO_HEAT		24	0.29216754	0.97631454	0.5	0.9862814	1	352	tags=29%, list=19%, signal=35%
GOBP_NEGATIVE_REGULATION_OF_TRANSMEMBRANE_TRANSPORT		26	0.30198905	0.9754064	0.5	0.9875122	1	309	tags=35%, list=17%, signal=41%
GOBP_NEGATIVE_REGULATION_OF_REACTIVE_OXYGEN_SPECIES_BIOSYNT		10	0.37482354	0.9750686	0.48076922	0.9871042	1	244	tags=30%, list=13%, signal=34%
GOBP_CELLULAR_COMPONENT_MORPHOGENESIS		126	0.21442375	0.9742391	0.5405405	0.9883218	1	449	tags=29%, list=24%, signal=35%
GOBP_EXTERNAL_ENCAPSULATING_STRUCTURE_ORGANIZATION		66	0.23501545	0.9737727	0.5208333	0.9887095	1	265	tags=21%, list=14%, signal=24%
GOBP_ARACHIDONIC_ACID_METABOLIC_PROCESS		10	0.38661787	0.97368544	0.53333336	0.98745114	1	264	tags=40%, list=14%, signal=46%
GOBP_REGULATION_OF_EPITHELIAL_CELL_DIFFERENTIATION		11	0.3543315	0.9732147	0.42857143	0.9873671	1	131	tags=27%, list=7%, signal=29%
GOBP_TUMOR_NECROSIS_FACTOR_SUPERFAMILY_CYTOKINE_PRODUCTION		17	0.3181767	0.97289866	0.47058824	0.9868049	1	308	tags=24%, list=16%, signal=28%
REACTOME_ANTIVIRAL_MECHANISM_BY_IFN_STIMULATED_GENES		14	0.3587652	0.9725153	0.42857143	0.98634374	1	232	tags=21%, list=12%, signal=24%
GOBP_CELL_JUNCTION_ORGANIZATION		118	0.2078742	0.97188026	0.59090906	0.9870257	1	232	tags=18%, list=12%, signal=19%
GOBP_MAMMARY_GLAND_DEVELOPMENT		16	0.336456	0.9711432	0.5217391	0.9879018	1	176	tags=25%, list=9%, signal=27%
GOBP_POSITIVE_REGULATION_OF_CELLULAR_COMPONENT_ORGANIZATION		193	0.20038049	0.9711259	0.57894737	0.9864437	1	384	tags=24%, list=21%, signal=27%
GOBP_EPIDERMIS_DEVELOPMENT		26	0.29831144	0.9703248	0.59090906	0.98776007	1	500	tags=38%, list=27%, signal=52%
GOBP_INFLAMMATORY_RESPONSE		83	0.2326019	0.969915	0.53488374	0.9874405	1	453	tags=30%, list=24%, signal=38%
REACTOME_ACTIVATION_OF_THE_MRNA_UPON_BINDING_OF_THE_CAP_BINI		32	0.29394826	0.96865505	0.47826087	0.9901407	1	529	tags=41%, list=28%, signal=56%
GOBP_POSITIVE_REGULATION_OF_ENDOTHELIAL_CELL_MIGRATION		19	0.30905318	0.9673229	0.47727272	0.9925244	1	308	tags=26%, list=16%, signal=31%
GOBP_POSITIVE_REGULATION_OF_CYTOSKELETON_ORGANIZATION		46	0.2377116	0.966475	0.5	0.993848	1	149	tags=13%, list=8%, signal=14%
GOBP_REGULATION_OF_HYDROLASE_ACTIVITY		183	0.19920868	0.96629643	0.5675676	0.99293876	1	434	tags=26%, list=23%, signal=31%
GOBP_BLOOD_VESSEL_ENDOTHELIAL_CELL_MIGRATION		19	0.30662543	0.9659437	0.45833334	0.992632	1	338	tags=37%, list=18%, signal=45%
GOBP_REGULATION_OF_EXTRINSIC_APOPTOTIC_SIGNALING_PATHWAY_VIA		12	0.329811	0.96380836	0.49019608	0.9986486	1	256	tags=25%, list=14%, signal=29%
REACTOME_UNFOLDED_PROTEIN_RESPONSE_UPR		21	0.31094396	0.96308166	0.4509804	0.99995905	1	99	tags=19%, list=5%, signal=20%
GOBP_REGULATION_OF_MAPK_CASCADE		74	0.2317597	0.96275634	0.5714286	0.99950475	1	340	tags=23%, list=18%, signal=27%
GOBP_CELLULAR_RESPONSE_TO_ORGANIC_CYCLIC_COMPOUND		79	0.23084484	0.96084297	0.4390244	1	1	480	tags=35%, list=26%, signal=46%
REACTOME_COPI_DEPENDENT_GOLGI_TO_ER_RETROGRADE_TRAFFIC		23	0.30242577	0.9604607	0.54	1	1	171	tags=22%, list=9%, signal=24%
GOBP_INTRINSIC_APOPTOTIC_SIGNALING_PATHWAY_IN_RESPONSE_TO_OX		12	0.3445646	0.96046025	0.5	1	1	308	tags=42%, list=16%, signal=50%
GOBP_MULTICELLULAR_ORGANISMAL_HOMEOSTASIS		68	0.21770748	0.959767	0.5	1	1	131	tags=15%, list=7%, signal=15%
GOBP_INTRACELLULAR_TRANSPORT		383	0.17827152	0.95969445	0.54285717	1	1	422	tags=25%, list=23%, signal=26%
GOBP_DEFENSE_RESPONSE_TO_VIRUS		23	0.29863885	0.9596226	0.4893617	0.9999483	1	227	tags=22%, list=12%, signal=24%
GOBP_NEGATIVE_REGULATION_OF_ERK1_AND_ERK2_CASCADE		14	0.325875	0.9594597	0.53488374	0.9989653	1	340	tags=36%, list=18%, signal=43%
GOBP_CELLULAR_RESPONSE_TO_BIOTIC_STIMULUS		25	0.29678014	0.9585906	0.5609756	1	1	337	tags=24%, list=18%, signal=29%
GOBP_LOW_DENSITY_LIPOPROTEIN_PARTICLE_CLEARANCE		13	0.34436268	0.9582597	0.47916666	0.9999071	1	205	tags=31%, list=11%, signal=34%
GOBP_POSITIVE_REGULATION_OF_ORGANELLE_ORGANIZATION		100	0.21456757	0.95771366	0.55	1	1	380	tags=26%, list=20%, signal=31%
GOBP_NEGATIVE_REGULATION_OF_CELL_ADHESION		36	0.24751627	0.957573	0.44680852	0.9991087	1	297	tags=22%, list=16%, signal=26%
GOBP_DENDRITIC_SPINE_DEVELOPMENT		14	0.34528792	0.9563462	0.5	1	1	202	tags=21%, list=11%, signal=24%
GOBP_VASCULAR_PROCESS_IN_CIRCULATORY_SYSTEM		44	0.26729384	0.95572925	0.53488374	1	1	146	tags=16%, list=8%, signal=17%
REACTOME_COMPLEMENT_CASCADE		11	0.35058314	0.955333	0.48979592	1	1	254	tags=27%, list=14%, signal=31%
GOBP_MYELOID_CELL_DIFFERENTIATION		42	0.2593365	0.95499915	0.5	1	1	128	tags=14%, list=7%, signal=15%
GOBP_CELLULAR_HOMEOSTASIS		156	0.20319161	0.954854	0.5555556	1	1	154	tags=14%, list=8%, signal=14%
REACTOME_SRP_DEPENDENT_COTRANSLATIONAL_PROTEIN_TARGETING_T		73	0.22937125	0.9541426	0.5531915	1	1	433	tags=34%, list=23%, signal=43%
GOBP_REGULATION_OF_CELLULAR_LOCALIZATION		155	0.19629005	0.95314264	0.6486486	1	1	371	tags=25%, list=20%, signal=28%
GOBP_NEGATIVE_REGULATION_OF_ESTABLISHMENT_OF_PROTEIN_LOCALI		18	0.32381278	0.95307374	0.5	1	1	467	tags=39%, list=25%, signal=51%
GOBP_DENDRITE_DEVELOPMENT		28	0.28800803	0.9509988	0.5	1	1	380	tags=25%, list=20%, signal=31%

GOBP_POSITIVE_REGULATION_OF_LOCOMOTION		87	0.22649223	0.9509848	0.5744681	1	1	454	tags=30%, list=24%, signal=38%
GOBP_CALCIUM_ION_TRANSPORT_INTO_CYTOSOL		24	0.27841425	0.9508019	0.48979592	1	1	478	tags=42%, list=26%, signal=55%
GOBP_REGULATION_OF_CELL_ADHESION		110	0.2087395	0.95044106	0.5	1	1	297	tags=20%, list=16%, signal=22%
GOBP_CELL_MORPHOGENESIS_INVOLVED_IN_DIFFERENTIATION		111	0.20754775	0.9497059	0.5208333	1	1	457	tags=29%, list=24%, signal=36%
GOBP_SPROUTING_ANGIOGENESIS		17	0.34592468	0.9494865	0.54385966	1	1	340	tags=29%, list=18%, signal=36%
GOBP_NEUTRAL_LIPID_METABOLIC_PROCESS		29	0.2883187	0.94862294	0.5	1	1	502	tags=41%, list=27%, signal=56%
GOBP_LIPID_STORAGE		18	0.31593367	0.9484348	0.5102041	1	1	339	tags=33%, list=18%, signal=40%
GOBP_RIBONUCLEOPROTEIN_COMPLEX_SUBUNIT_ORGANIZATION		31	0.26196688	0.9483213	0.5116279	1	1	168	tags=19%, list=9%, signal=21%
GOBP_VESICLE_TARGETING		22	0.30981612	0.9482975	0.53061223	1	1	361	tags=32%, list=19%, signal=39%
GOBP_NEGATIVE_REGULATION_OF_REACTIVE_OXYGEN_SPECIES_METABO		19	0.32683462	0.9477889	0.47916666	1	1	312	tags=32%, list=17%, signal=38%
GOBP_ENZYME_LINKED_RECEPTOR_PROTEIN_SIGNALING_PATHWAY		118	0.21029386	0.94773793	0.5416667	1	1	439	tags=28%, list=23%, signal=34%
GOBP_PHOSPHATIDYLINOSITOL_3_KINASE_SIGNALING		12	0.3377926	0.9471927	0.42222223	1	1	366	tags=33%, list=20%, signal=41%
REACTOME_METABOLISM_OF_LIPIDS		149	0.20350428	0.9467266	0.58536583	1	1	273	tags=19%, list=15%, signal=20%
REACTOME_SARS_COV_INFECTIONS		41	0.25864375	0.94599044	0.54901963	1	1	205	tags=17%, list=11%, signal=19%
GOBP_MEMBRANE_DEPOLARIZATION		21	0.29334342	0.94489306	0.53846157	1	1	366	tags=38%, list=20%, signal=47%
GOBP_HOMEOSTASIS_OF_NUMBER_OF_CELLS		32	0.2617514	0.9441036	0.54347825	1	1	579	tags=41%, list=31%, signal=58%
GOBP_CHAPERONE_MEDIATED_PROTEIN_FOLDING		22	0.2924614	0.9418443	0.4814815	1	1	352	tags=32%, list=19%, signal=39%
GOBP_CELLULAR_MODIFIED_AMINO_ACID_BIOSYNTHETIC_PROCESS		16	0.32380578	0.9408309	0.6	1	1	277	tags=31%, list=15%, signal=36%
REACTOME_PHASE_I_FUNCTIONALIZATION_OF_COMPOUNDS		20	0.28885624	0.9404256	0.6052632	1	1	220	tags=20%, list=12%, signal=22%
GOBP_RNA_PROCESSING		58	0.24006055	0.94041926	0.64705884	1	1	433	tags=29%, list=23%, signal=37%
GOBP_REGULATION_OF_ACTIN_FILAMENT_ORGANIZATION		66	0.21982852	0.9404167	0.5675676	1	1	149	tags=11%, list=8%, signal=11%
GOBP_POSITIVE_REGULATION_OF_ANION_TRANSPORT		82	0.21144602	0.9401887	0.5882353	1	1	295	tags=22%, list=16%, signal=25%
REACTOME_RHOV_GTPASE_CYCLE		10	0.38422933	0.9394309	0.59574467	1	1	336	tags=30%, list=18%, signal=36%
GOBP_NEGATIVE_REGULATION_OF_INTRACELLULAR_SIGNAL_TRANSDUCTI		69	0.22744064	0.9384445	0.61764705	1	1	342	tags=26%, list=18%, signal=31%
GOBP_CYTOKINE_PRODUCTION		71	0.22336641	0.9378464	0.6363636	1	1	352	tags=25%, list=19%, signal=30%
GOBP_COGNITION		24	0.25967857	0.9377381	0.5833333	1	1	235	tags=21%, list=13%, signal=24%
GOBP_FATTY_ACYL_COA_METABOLIC_PROCESS		16	0.32594088	0.9377044	0.53846157	1	1	133	tags=19%, list=7%, signal=20%
REACTOME_METABOLISM_OF_STEROIDS		19	0.3086216	0.9375529	0.6097561	1	1	108	tags=16%, list=6%, signal=17%
REACTOME_GAP_JUNCTION_TRAFFICKING_AND_REGULATION		13	0.32558206	0.9374338	0.5576923	1	1	127	tags=15%, list=7%, signal=16%
GOBP_REGULATION_OF_PROTEIN_CONTAINING_COMPLEX_DISASSEMBLY		27	0.27042317	0.937422	0.6226415	1	1	66	tags=11%, list=4%, signal=11%
GOBP_POSITIVE_REGULATION_OF_ION_TRANSPORT		113	0.19906174	0.9368003	0.5777778	1	1	321	tags=21%, list=17%, signal=24%
GOBP_ACTIN_FILAMENT_BASED_PROCESS		171	0.19123423	0.9364828	0.5714286	1	1	300	tags=19%, list=16%, signal=21%
GOBP_REGULATION_OF_TOLL_LIKE_RECEPTOR_SIGNALING_PATHWAY		10	0.36953467	0.9362872	0.59183675	1	1	337	tags=30%, list=18%, signal=36%
GOBP_HORMONE_TRANSPORT		43	0.24213998	0.9360038	0.5744681	1	1	339	tags=33%, list=18%, signal=39%
GOBP_INTRACELLULAR_PROTEIN_TRANSPORT		276	0.17871445	0.9353146	0.72	1	1	433	tags=26%, list=23%, signal=29%
REACTOME_ADRENALINE_NORADRENALINE_INHIBITS_INSULIN_SECRETION		12	0.31420928	0.9352727	0.5714286	1	1	30	tags=17%, list=2%, signal=17%
GOBP_NEGATIVE_REGULATION_OF_EPITHELIAL_CELL_PROLIFERATION		17	0.30194455	0.9348557	0.6101695	1	1	206	tags=24%, list=11%, signal=26%
GOBP_PLATELET_AGGREGATION		23	0.28426397	0.93455416	0.5869565	1	1	337	tags=35%, list=18%, signal=42%
GOBP_ACTIN_NUCLEATION		16	0.3266418	0.9331802	0.51785713	1	1	149	tags=19%, list=8%, signal=20%
GOBP_ADAPTIVE_IMMUNE_RESPONSE		39	0.24835178	0.933037	0.61702126	1	1	401	tags=23%, list=21%, signal=29%
GOBP_NEGATIVE_REGULATION_OF_KINASE_ACTIVITY		43	0.2604266	0.9329295	0.56	1	1	409	tags=33%, list=22%, signal=41%
GOBP_REGULATION_OF_CELL_PROJECTION_ORGANIZATION		91	0.20242572	0.932709	0.6041667	1	1	384	tags=24%, list=21%, signal=29%
REACTOME_CELL_SURFACE_INTERACTIONS_AT_THE_VASCULAR_WALL		30	0.27540165	0.93221796	0.56363636	1	1	446	tags=37%, list=24%, signal=47%
GOBP_REGULATION_OF_PROTEIN_PHOSPHORYLATION		137	0.2007029	0.9318742	0.61290324	1	1	365	tags=22%, list=20%, signal=25%
GOBP_CALCIUM_MEDIATED_SIGNALING		34	0.26327705	0.93162525	0.64	1	1	194	tags=18%, list=10%, signal=19%
GOBP_NEGATIVE_REGULATION_OF_INTRACELLULAR_TRANSPORT		12	0.36580506	0.9315007	0.6122449	1	1	253	tags=33%, list=14%, signal=38%
GOBP_MAINTENANCE_OF_LOCATION		63	0.23579098	0.93122846	0.57894737	1	1	490	tags=38%, list=26%, signal=50%
REACTOME_EUKARYOTIC_TRANSLATION_ELONGATION		68	0.23005635	0.92997336	0.67346936	1	1	433	tags=34%, list=23%, signal=42%
GOBP_CELLULAR_MACROMOLECULE_LOCALIZATION		412	0.16719829	0.9294826	0.6333333	1	1	434	tags=25%, list=23%, signal=26%
GOBP_SHORT_CHAIN_FATTY_ACID_METABOLIC_PROCESS		11	0.3651193	0.9289185	0.5416667	1	1	129	tags=27%, list=7%, signal=29%
GOBP_MRNA_METABOLIC_PROCESS		134	0.20051773	0.92880577	0.627451	1	1	434	tags=28%, list=23%, signal=34%
GOBP_PLASMA_LIPOPROTEIN_PARTICLE_CLEARANCE		17	0.31034857	0.92801684	0.44642857	1	1	205	tags=29%, list=11%, signal=33%
GOBP_ORGANOPHOSPHATE_BIOSYNTHETIC_PROCESS		131	0.20717855	0.927868	0.6315789	1	1	520	tags=35%, list=28%, signal=45%
GOBP_CELLULAR_HORMONE_METABOLIC_PROCESS		14	0.34265032	0.92783904	0.53061223	1	1	586	tags=36%, list=31%, signal=52%
GOBP_REGULATION_OF_NUCLEOTIDE_BIOSYNTHETIC_PROCESS		12	0.320075	0.92750895	0.5869565	1	1	344	tags=42%, list=18%, signal=51%
GOBP_POSITIVE_REGULATION_OF_CELLULAR_COMPONENT_BIOGENESIS		96	0.20835228	0.9266805	0.6666667	1	1	365	tags=22%, list=20%, signal=26%
GOBP_RESPONSE_TO_VIRUS		37	0.26825348	0.9252579	0.5769231	1	1	227	tags=22%, list=12%, signal=24%

GOBP_ADENYLATE_CYCLASE_MODULATING_G_PROTEIN_COUPLED_RECEP		23	0.32579386	0.92452794	0.54285717	1	1	128	tags=17%, list=7%, signal=18%
REACTOME_REGULATION_OF_INSULIN_LIKE_GROWTH_FACTOR_IGF_TRANS		35	0.26150206	0.9244832	0.5681818	1	1	457	tags=46%, list=24%, signal=59%
GOBP_POSITIVE_REGULATION_OF_PHOSPHORUS_METABOLIC_PROCESS		106	0.20037115	0.9244071	0.6666667	1	1	269	tags=17%, list=14%, signal=19%
GOBP_REGULATION_OF_LIPID_TRANSPORT		26	0.26379856	0.92375505	0.5217391	1	1	146	tags=19%, list=8%, signal=21%
GOBP_POSITIVE_REGULATION_OF_PROTEIN_KINASE_B_SIGNALING		22	0.27864492	0.92331296	0.5660377	1	1	244	tags=23%, list=13%, signal=26%
REACTOME_GABA_RECEPTOR_ACTIVATION		10	0.36562687	0.92267233	0.58928573	1	1	30	tags=20%, list=2%, signal=20%
GOBP_NEGATIVE_REGULATION_OF_TRANSCRIPTION_BY_RNA_POLYMERAS		36	0.25051567	0.92195296	0.6122449	1	1	482	tags=36%, list=26%, signal=48%
GOBP_NEGATIVE_REGULATION_OF_PROTEIN_POLYMERIZATION		23	0.27935714	0.9213748	0.5609756	1	1	584	tags=39%, list=31%, signal=56%
GOBP_POSITIVE_REGULATION_OF_SECRETION		44	0.23754372	0.92101353	0.5471698	1	1	380	tags=30%, list=20%, signal=36%
GOBP_REGULATION_OF_CALCIUM_ION_TRANSMEMBRANE_TRANSPORT		29	0.26495486	0.9209009	0.5869565	1	1	478	tags=41%, list=26%, signal=55%
GOBP_POSITIVE_REGULATION_OF_LIPASE_ACTIVITY		11	0.34762466	0.92075	0.5	1	1	227	tags=27%, list=12%, signal=31%
GOBP_FATTY_ACID_TRANSPORT		29	0.2786095	0.9206471	0.64	1	1	190	tags=24%, list=10%, signal=26%
REACTOME_INFECTIOUS_DISEASE		220	0.17636006	0.9206355	0.75	1	1	434	tags=27%, list=23%, signal=31%
REACTOME_ION_HOMEOSTASIS		18	0.31505015	0.92057234	0.5849057	1	1	194	tags=28%, list=10%, signal=31%
GOBP_POSITIVE_REGULATION_OF_MAPK_CASCADE		50	0.23068498	0.9204626	0.5769231	1	1	304	tags=20%, list=16%, signal=23%
GOBP_VERY_LONG_CHAIN_FATTY_ACID_METABOLIC_PROCESS		11	0.38362432	0.92001563	0.5471698	1	1	173	tags=27%, list=9%, signal=30%
GOBP_STEROID_METABOLIC_PROCESS		44	0.2459609	0.91995716	0.6923077	1	1	604	tags=48%, list=32%, signal=69%
GOBP_TELENCEPHALON_DEVELOPMENT		22	0.29853377	0.91992414	0.62222224	0.99904686	1	300	tags=27%, list=16%, signal=32%
REACTOME_CLATHRIN_MEDIATED_ENDOCYTOSIS		38	0.26094046	0.9191855	0.5	0.99971503	1	205	tags=16%, list=11%, signal=17%
GOBP_POSITIVE_REGULATION_OF_RESPONSE_TO_EXTERNAL_STIMULUS		86	0.2107117	0.91874844	0.6388889	0.99963963	1	390	tags=22%, list=21%, signal=27%
GOBP_REGULATION_OF_SMALL_MOLECULE_METABOLIC_PROCESS		88	0.20514163	0.91762376	0.6744186	1	1	523	tags=33%, list=28%, signal=44%
GOBP_RECEPTOR_CLUSTERING		12	0.35317537	0.91680735	0.5555556	1	1	340	tags=42%, list=18%, signal=51%
GOBP_POSITIVE_REGULATION_OF_EXOCYTOSIS		19	0.30439615	0.9150298	0.56	1	1	512	tags=47%, list=27%, signal=65%
GOBP_CARDIAC_CONDUCTION		37	0.24666353	0.91421664	0.54545456	1	1	228	tags=22%, list=12%, signal=24%
GOBP_MEMBRANE_DEPOLARIZATION_DURING_ACTION_POTENTIAL		11	0.3281519	0.9133875	0.53333336	1	1	364	tags=36%, list=19%, signal=45%
GOBP_NEGATIVE_REGULATION_OF_ION_TRANSPORT		52	0.24046706	0.9123707	0.6086956	1	1	309	tags=27%, list=17%, signal=31%
GOBP_REGULATION_OF_HEMATOPOIETIC_STEM_CELL_DIFFERENTIATION		32	0.2596277	0.9123102	0.627907	1	1	425	tags=25%, list=23%, signal=32%
GOBP_REGULATION_OF_POSTSYNAPTIC_MEMBRANE_NEUROTRANSMITTER		12	0.34587806	0.9114437	0.6304348	1	1	582	tags=67%, list=31%, signal=96%
GOBP_MULTI_ORGANISM_PROCESS		83	0.20785394	0.9113557	0.6666667	1	1	271	tags=22%, list=14%, signal=24%
GOBP_HEMATOPOIETIC_STEM_CELL_DIFFERENTIATION		33	0.2558404	0.91116303	0.5319149	1	1	425	tags=24%, list=23%, signal=31%
GOBP_POSITIVE_REGULATION_OF_NEURON_DEATH		11	0.36261353	0.91022426	0.6	1	1	257	tags=27%, list=14%, signal=31%
REACTOME_RESPONSE_OF_EIF2AK4_GCN2_TO_AMINO_ACID_DEFICIENCY		65	0.21265745	0.9099877	0.7619048	1	1	433	tags=34%, list=23%, signal=43%
REACTOME_INTEGRIN_CELL_SURFACE_INTERACTIONS		28	0.2608509	0.9088273	0.6511628	1	1	333	tags=32%, list=18%, signal=39%
GOBP_REGULATION_OF_GLUCOSE_TRANSMEMBRANE_TRANSPORT		12	0.31768513	0.908813	0.62222224	1	1	406	tags=33%, list=22%, signal=42%
GOBP_ESTABLISHMENT_OF_PROTEIN_LOCALIZATION_TO_ORGANELLE		199	0.18570271	0.9079609	0.75	1	1	433	tags=29%, list=23%, signal=33%
GOBP_NEGATIVE_REGULATION_OF_SIGNALING		179	0.1866821	0.90727407	0.8181818	1	1	425	tags=27%, list=23%, signal=32%
GOBP_REGULATION_OF_PEPTIDE_SECRETION		47	0.23255005	0.90682554	0.6818182	1	1	339	tags=30%, list=18%, signal=35%
GOBP_NEGATIVE_REGULATION_OF_MAP_KINASE_ACTIVITY		10	0.3343656	0.90655196	0.5576923	1	1	337	tags=30%, list=18%, signal=36%
GOBP_CELLULAR_RESPONSE_TO_INORGANIC_SUBSTANCE		27	0.2661561	0.9062066	0.6086956	1	1	453	tags=33%, list=24%, signal=43%
GOBP_ENDOPLASMIC_RETICULUM_UNFOLDED_PROTEIN_RESPONSE		31	0.2731117	0.90566367	0.6363636	1	1	117	tags=16%, list=6%, signal=17%
GOBP_CELL_PROJECTION_ORGANIZATION		185	0.17692025	0.90400535	0.6666667	1	1	407	tags=23%, list=22%, signal=27%
GOBP_REGULATION_OF_HORMONE_LEVELS		64	0.21947047	0.90387315	0.625	1	1	374	tags=28%, list=20%, signal=34%
REACTOME_BASIGIN_INTERACTIONS		10	0.372309	0.9033912	0.625	1	1	402	tags=50%, list=21%, signal=63%
GOBP_FATTY_ACID_DERIVATIVE_BIOSYNTHETIC_PROCESS		16	0.31438884	0.90331227	0.6969697	1	1	513	tags=50%, list=27%, signal=68%
GOBP_NEGATIVE_REGULATION_OF_PROTEIN_CONTAINING_COMPLEX_ASSE		36	0.24679331	0.90294707	0.59574467	1	1	609	tags=42%, list=33%, signal=61%
GOBP_LAMELLIPODIUM_ORGANIZATION		24	0.27062497	0.9025353	0.64444447	1	1	407	tags=29%, list=22%, signal=37%
REACTOME_SIGNALING_BY_RECEPTOR_TYROSINE_KINASES		85	0.20194642	0.9022543	0.70731705	1	1	407	tags=25%, list=22%, signal=30%
GOBP_POSITIVE_REGULATION_OF_DNA_METABOLIC_PROCESS		23	0.26047197	0.9014543	0.62222224	1	1	271	tags=22%, list=14%, signal=25%
GOBP_ANIMAL_ORGAN_MORPHOGENESIS		125	0.19146281	0.9013596	0.8	1	1	300	tags=18%, list=16%, signal=20%
REACTOME_MAPK_FAMILY_SIGNALING_CASCADES		74	0.21288374	0.89998645	0.72727275	1	1	457	tags=26%, list=24%, signal=33%
GOBP_GOLGI_ORGANIZATION		26	0.2681662	0.8987535	0.6122449	1	1	170	tags=15%, list=9%, signal=17%
GOBP_LONG_CHAIN_FATTY_ACID_TRANSPORT		17	0.29343474	0.89637613	0.6	1	1	469	tags=47%, list=25%, signal=62%
GOBP_REGULATION_OF_PEPTIDE_TRANSPORT		101	0.20062152	0.8953992	0.73170733	1	1	380	tags=27%, list=20%, signal=32%
GOBP_DNA_BIOSYNTHETIC_PROCESS		24	0.26575813	0.89485514	0.6458333	1	1	286	tags=29%, list=15%, signal=34%
GOBP_LAMELLIPODIUM_ASSEMBLY		20	0.28837314	0.8946111	0.6730769	1	1	212	tags=20%, list=11%, signal=22%
GOBP_NCRNA_PROCESSING		35	0.24175362	0.89302456	0.71428573	1	1	433	tags=31%, list=23%, signal=40%
GOBP_PEPTIDYL_PROLINE_MODIFICATION		13	0.32877213	0.8929048	0.6862745	1	1	269	tags=23%, list=14%, signal=27%

GOBP_CELL_SUBSTRATE_ADHESION		78	0.20423712	0.89287317	0.7948718	1	1	256	tags=18%, list=14%, signal=20%
GOBP_GLYCEROLIPID_BIOSYNTHETIC_PROCESS		43	0.23986167	0.8915377	0.7234042	1	1	511	tags=35%, list=27%, signal=47%
GOBP_ER_NUCLEUS_SIGNALING_PATHWAY		13	0.31443298	0.8905968	0.65384614	1	1	350	tags=31%, list=19%, signal=38%
GOBP_REGULATION_OF_ACTIN_FILAMENT_BUNDLE_ASSEMBLY		23	0.3016375	0.890577	0.6976744	1	1	18	tags=9%, list=1%, signal=9%
GOBP_NEGATIVE_REGULATION_OF_ANION_TRANSPORT		31	0.2492437	0.88935846	0.55	1	1	467	tags=32%, list=25%, signal=42%
GOBP_REGULATION_OF_ANION_TRANSPORT		136	0.19019084	0.8888992	0.82051283	1	1	324	tags=23%, list=17%, signal=26%
GOBP_REGULATION_OF_PROTEOLYSIS		110	0.20084897	0.88830936	0.6904762	1	1	439	tags=28%, list=23%, signal=35%
REACTOME_RHOJ_GTPASE_CYCLE		16	0.29108572	0.8866216	0.54545456	1	1	253	tags=25%, list=14%, signal=29%
GOBP_REGULATION_OF_PROTEIN_CATABOLIC_PROCESS		69	0.22412805	0.8862562	0.6818182	1	1	244	tags=17%, list=13%, signal=19%
GOBP_NEGATIVE_REGULATION_OF_RESPONSE_TO_STIMULUS		206	0.17892691	0.8858477	0.7297297	1	1	425	tags=26%, list=23%, signal=30%
GOBP_NEGATIVE_REGULATION_OF_CELL_DIFFERENTIATION		48	0.22079702	0.88517994	0.7173913	1	1	384	tags=27%, list=21%, signal=33%
REACTOME_SIGNAL_AMPLIFICATION		12	0.33788276	0.8847431	0.5531915	1	1	30	tags=17%, list=2%, signal=17%
GOBP_ACTIVATION_OF_PROTEIN_KINASE_ACTIVITY		36	0.23047474	0.8841991	0.67391306	1	1	482	tags=31%, list=26%, signal=40%
GOBP_AXON_EXTENSION		24	0.27183497	0.8837953	0.61904764	1	1	283	tags=21%, list=15%, signal=24%
REACTOME_REGULATION_OF_INSULIN_SECRETION		24	0.26783437	0.8829909	0.65957445	1	1	397	tags=38%, list=21%, signal=47%
GOBP_PROTEIN_REFOLDING		11	0.32488173	0.88249093	0.64	1	1	72	tags=18%, list=4%, signal=19%
GOBP_MORPHOGENESIS_OF_AN_EPITHELIUM		84	0.19959739	0.8822933	0.6666667	1	1	210	tags=15%, list=11%, signal=17%
REACTOME_RAC2_GTPASE_CYCLE		21	0.27303132	0.88217974	0.5263158	1	1	253	tags=29%, list=14%, signal=33%
GOBP_PROCESS_UTILIZING_AUTOPHAGIC_MECHANISM		98	0.19884875	0.8820401	0.7346939	1	1	380	tags=22%, list=20%, signal=27%
GOBP_CELL_DEATH_IN_RESPONSE_TO_OXIDATIVE_STRESS		16	0.2977441	0.8819428	0.59090906	1	1	522	tags=56%, list=28%, signal=77%
GOBP_NEGATIVE_REGULATION_OF_CATABOLIC_PROCESS		31	0.24150786	0.8815851	0.6666667	1	1	312	tags=23%, list=17%, signal=27%
GOBP_REGULATION_OF_HEMATOPOIETIC_PROGENITOR_CELL_DIFFERENTIA		32	0.2596277	0.88066745	0.6851852	1	1	425	tags=25%, list=23%, signal=32%
GOBP_MUSCLE_ADAPTATION		22	0.27948958	0.88042873	0.65217394	1	1	246	tags=23%, list=13%, signal=26%
GOBP_OSSIFICATION		44	0.23266934	0.8804272	0.6458333	1	1	333	tags=27%, list=18%, signal=32%
GOBP_REGULATION_OF_INTRACELLULAR_SIGNAL_TRANSDUCTION		201	0.1775746	0.87890375	0.81578946	1	1	269	tags=16%, list=14%, signal=17%
GOBP_MACROMOLECULE_CATABOLIC_PROCESS		262	0.16835804	0.8783641	0.9148936	1	1	437	tags=24%, list=23%, signal=27%
KEGG_RIBOSOME		63	0.20745106	0.87779844	0.7111111	1	1	433	tags=33%, list=23%, signal=42%
GOBP_CELL_RECOGNITION		27	0.25471324	0.87587893	0.6530612	1	1	260	tags=22%, list=14%, signal=25%
GOBP_ESTABLISHMENT_OF_ORGANELLE_LOCALIZATION		70	0.20140141	0.8755999	0.78571427	1	1	416	tags=27%, list=22%, signal=34%
GOBP_SMOOTH_MUSCLE_CELL_MIGRATION		12	0.32588252	0.8754997	0.627451	1	1	328	tags=25%, list=18%, signal=30%
GOBP_REGULATION_OF_PROTEIN_LOCALIZATION_TO_CELL_PERIPHERY		29	0.24507079	0.8751918	0.6976744	1	1	493	tags=34%, list=26%, signal=46%
GOBP_PEPTIDE_HORMONE_SECRETION		36	0.24661775	0.8751912	0.6981132	1	1	471	tags=42%, list=25%, signal=55%
GOBP_HEMATOPOIETIC_PROGENITOR_CELL_DIFFERENTIATION		40	0.24159275	0.87421966	0.65957445	1	1	683	tags=43%, list=37%, signal=66%
GOBP_NEGATIVE_REGULATION_OF_LYMPHOCYTE_ACTIVATION		10	0.34205553	0.8734573	0.63265306	1	1	337	tags=30%, list=18%, signal=36%
GOBP_MAINTENANCE_OF_LOCATION_IN_CELL		44	0.22116221	0.8731622	0.6976744	1	1	490	tags=39%, list=26%, signal=51%
GOBP_CYTOSOLIC_CALCIUM_ION_TRANSPORT		34	0.23595603	0.87193376	0.6481481	1	1	478	tags=38%, list=26%, signal=50%
GOBP_CELLULAR_MACROMOLECULE_CATABOLIC_PROCESS		225	0.17126301	0.8715293	0.925	1	1	437	tags=26%, list=23%, signal=30%
GOBP_ESTABLISHMENT_OR_MAINTENANCE_OF_CELL_POLARITY		46	0.23458555	0.8708258	0.7173913	1	1	539	tags=43%, list=29%, signal=60%
GOBP_LEUKOCYTE_MEDIATED_CYTOTOXICITY		11	0.31354463	0.869956	0.63414633	1	1	709	tags=55%, list=38%, signal=87%
REACTOME_PHOSPHOLIPID_METABOLISM		33	0.2493682	0.86954325	0.68	1	1	511	tags=42%, list=27%, signal=57%
KEGG_ADIPOCYTOKINE_SIGNALING_PATHWAY		12	0.34684822	0.86943763	0.5681818	1	1	381	tags=33%, list=20%, signal=42%
GOBP_NEURON_APOPTOTIC_PROCESS		28	0.24645878	0.8691159	0.6486486	1	1	321	tags=25%, list=17%, signal=30%
GOBP_REGULATION_OF_HEMOPOIESIS		27	0.24648099	0.86638826	0.6	1	1	522	tags=37%, list=28%, signal=51%
GOBP_EXTRINSIC_APOPTOTIC_SIGNALING_PATHWAY_VIA_DEATH_DOMAIN_		14	0.29858944	0.8662825	0.6956522	1	1	704	tags=57%, list=38%, signal=91%
GOBP_OSTEOBLAST_DIFFERENTIATION		27	0.26006314	0.86599857	0.63265306	1	1	374	tags=37%, list=20%, signal=46%
GOBP_MITOCHONDRIAL_ELECTRON_TRANSPORT_CYTOCHROME_C_TO_OXY		11	0.32707223	0.8640932	0.6956522	1	1	137	tags=27%, list=7%, signal=29%
GOBP_NEGATIVE_REGULATION_OF_TRANSFERASE_ACTIVITY		48	0.2249211	0.8630356	0.81578946	1	1	409	tags=29%, list=22%, signal=36%
GOBP_RESPONSE_TO_RADIATION		40	0.21924694	0.8626281	0.75609756	1	1	407	tags=25%, list=22%, signal=31%
GOBP_AXON_DEVELOPMENT		72	0.20158632	0.8625396	0.79545456	1	1	407	tags=24%, list=22%, signal=29%
GOBP_ANTIGEN_PROCESSING_AND_PRESENTATION_OF_PEPTIDE_OR_POL		29	0.24158153	0.86214536	0.7755102	1	1	205	tags=17%, list=11%, signal=19%
GOBP_POSITIVE_REGULATION_OF_IMMUNE_RESPONSE		106	0.18940829	0.86145353	0.74418604	1	1	337	tags=19%, list=18%, signal=22%
GOBP_POSITIVE_REGULATION_OF_DNA_BIOSYNTHETIC_PROCESS		13	0.29964983	0.8601877	0.61538464	1	1	38	tags=15%, list=2%, signal=16%
GOBP_PROTEIN_CATABOLIC_PROCESS		154	0.17796987	0.8600878	0.88372093	1	1	244	tags=14%, list=13%, signal=15%
GOBP_REGULATION_OF_STEM_CELL_DIFFERENTIATION		33	0.2357629	0.86004734	0.76744187	1	1	425	tags=24%, list=23%, signal=31%
GOBP_TRIGLYCERIDE_METABOLIC_PROCESS		26	0.2622784	0.85970634	0.6727273	1	1	502	tags=38%, list=27%, signal=52%
GOBP_CELL_MORPHOGENESIS_INVOLVED_IN_NEURON_DIFFERENTIATION		80	0.20337981	0.859465	0.8095238	1	1	453	tags=28%, list=24%, signal=35%
GOBP_REGULATION_OF_EPITHELIAL_CELL_APOPTOTIC_PROCESS		11	0.33455056	0.8593234	0.6097561	1	1	457	tags=45%, list=24%, signal=60%

GOBP_SARCOPLASMIC_RETICULUM_CALCIUM_ION_TRANSPORT		14	0.30103242	0.8592128	0.5686275	1	1	478	tags=50%, list=26%, signal=67%
GOBP_PROTEIN_LOCALIZATION_TO_MEMBRANE		205	0.17540248	0.8590165	0.8717949	1	1	448	tags=28%, list=24%, signal=33%
REACTOME_INNATE_IMMUNE_SYSTEM		234	0.16580804	0.8589702	0.8292683	1	1	128	tags=10%, list=7%, signal=9%
GOBP_SYNAPTIC_VESICLE_LOCALIZATION		10	0.3424348	0.8586201	0.58	1	1	407	tags=40%, list=22%, signal=51%
GOBP_REGULATION_OF_POSTSYNAPSE_ORGANIZATION		13	0.31166005	0.858308	0.6511628	1	1	202	tags=23%, list=11%, signal=26%
GOBP_DEFENSE_RESPONSE		203	0.17396295	0.85737824	0.7948718	1	1	338	tags=19%, list=18%, signal=21%
GOBP_CARBOHYDRATE_TRANSMEMBRANE_TRANSPORT		15	0.29244784	0.8570399	0.74418604	1	1	406	tags=33%, list=22%, signal=42%
GOBP_INTEGRIN_MEDIATED_SIGNALING_PATHWAY		26	0.25879472	0.8570251	0.76744187	1	1	265	tags=23%, list=14%, signal=27%
REACTOME_NONSENSE_MEDIATED_DECAY_NMD		66	0.2097483	0.8568254	0.76	1	1	433	tags=33%, list=23%, signal=42%
GOBP_NUCLEAR_TRANSCRIBED_MRNA_CATABOLIC_PROCESS		68	0.19216363	0.85615414	0.75555557	1	1	433	tags=31%, list=23%, signal=39%
GOBP_REGULATION_OF_CARDIAC_CONDUCTION		20	0.27140078	0.85553545	0.5813953	1	1	194	tags=20%, list=10%, signal=22%
REACTOME_VISUAL_PHOTOTRANSDUCTION		13	0.29790154	0.85547197	0.6938776	1	1	146	tags=15%, list=8%, signal=17%
GOBP_REGULATION_OF_MICROTUBULE_BASED_PROCESS		22	0.25838596	0.85522383	0.6086956	1	1	609	tags=45%, list=33%, signal=67%
GOBP_REGULATION_OF_RESPONSE_TO_BIOTIC_STIMULUS		67	0.21648774	0.8549832	0.71428573	1	1	212	tags=13%, list=11%, signal=15%
GOBP_REGULATION_OF_MUSCLE_ADAPTATION		18	0.28653896	0.8548628	0.6481481	1	1	246	tags=22%, list=13%, signal=25%
GOBP_REGULATION_OF_IMMUNE_RESPONSE		128	0.18063869	0.85385966	0.8	1	1	337	tags=17%, list=18%, signal=20%
GOBP_ACUTE_INFLAMMATORY_RESPONSE		16	0.30313036	0.85340947	0.6730769	1	1	426	tags=50%, list=23%, signal=64%
GOBP_MAPK_CASCADE		119	0.18526927	0.85295874	0.7631579	1	1	340	tags=19%, list=18%, signal=22%
GOBP_ORGANELLE_ASSEMBLY		126	0.18296613	0.8524561	0.82222223	1	1	539	tags=34%, list=29%, signal=45%
GOBP_CALCIUM_ION_IMPORT		19	0.29002264	0.8519536	0.6603774	1	1	194	tags=21%, list=10%, signal=23%
GOBP_INTRINSIC_APOPTOTIC_SIGNALING_PATHWAY		50	0.21442802	0.85110766	0.78571427	1	1	453	tags=30%, list=24%, signal=39%
GOBP_REGULATION_OF_CYTOSKELETON_ORGANIZATION		96	0.1927262	0.85096025	0.8095238	1	1	66	tags=7%, list=4%, signal=7%
GOBP_ACTIN_FILAMENT_ORGANIZATION		96	0.18965848	0.8500232	0.82222223	1	1	449	tags=23%, list=24%, signal=29%
GOBP_UBIQUINONE_METABOLIC_PROCESS		11	0.32845902	0.8495085	0.6888889	1	1	245	tags=27%, list=13%, signal=31%
GOBP_REGULATION_OF_ORGANELLE_ORGANIZATION		186	0.17768614	0.8493604	0.9032258	1	1	381	tags=23%, list=20%, signal=26%
GOBP_POSITIVE_REGULATION_OF_CELL_PROJECTION_ORGANIZATION		55	0.20591198	0.84904194	0.75	1	1	407	tags=25%, list=22%, signal=32%
REACTOME_COPII_MEDIATED_VESICLE_TRANSPORT		20	0.26752496	0.8489311	0.7446808	1	1	361	tags=30%, list=19%, signal=37%
GOBP_AMINE_METABOLIC_PROCESS		43	0.21976596	0.84884596	0.7209302	1	1	683	tags=47%, list=37%, signal=72%
GOBP_ORGANELLE_TRANSPORT_ALONG_MICROTUBULE		16	0.291534	0.84878707	0.75555557	1	1	564	tags=38%, list=30%, signal=53%
GOBP_AMYLOID_BETA_METABOLIC_PROCESS		11	0.337406	0.8473675	0.7291667	1	1	146	tags=18%, list=8%, signal=20%
GOBP_REGULATION_OF_RESPONSE_TO_OXIDATIVE_STRESS		15	0.30354345	0.8472174	0.66101694	1	1	522	tags=60%, list=28%, signal=83%
GOBP_BIOLOGICAL_PROCESS_INVOLVED_IN_INTERACTION_WITH_HOST		50	0.21240512	0.8468628	0.78571427	1	1	365	tags=28%, list=20%, signal=34%
REACTOME_OPIOID_SIGNALLING		23	0.24338645	0.8455859	0.75	1	1	574	tags=43%, list=31%, signal=62%
GOBP_VIRAL_GENE_EXPRESSION		67	0.19410412	0.8445539	0.8372093	1	1	433	tags=31%, list=23%, signal=39%
GOBP_REGULATION_OF_MITOCHONDRIAL_MEMBRANE_POTENTIAL		18	0.31035542	0.8441386	0.6666667	1	1	465	tags=50%, list=25%, signal=66%
GOBP_MONOCARBOXYLIC_ACID_TRANSPORT		20	0.254797	0.8431208	0.64444447	1	1	297	tags=25%, list=16%, signal=29%
REACTOME_IRE1ALPHA_ACTIVATES_CHAPERONES		17	0.28025976	0.84298795	0.66	1	1	418	tags=41%, list=22%, signal=53%
GOBP_RESPONSE_TO_ENDOPLASMIC_RETICULUM_STRESS		66	0.19802614	0.8424709	0.8333333	1	1	351	tags=23%, list=19%, signal=27%
GOBP_INNATE_IMMUNE_RESPONSE		120	0.175566	0.83937097	0.9032258	1	1	297	tags=17%, list=16%, signal=19%
GOBP_EPITHELIAL_CELL_PROLIFERATION		34	0.23329832	0.838874	0.7017544	1	1	206	tags=18%, list=11%, signal=19%
GOBP_REGULATION_OF_NEUROTRANSMITTER_TRANSPORT		16	0.29629818	0.83862877	0.627451	1	1	609	tags=56%, list=33%, signal=83%
GOBP_NEGATIVE_REGULATION_OF_MITOCHONDRION_ORGANIZATION		14	0.31591034	0.8385472	0.71428573	1	1	380	tags=43%, list=20%, signal=53%
GOBP_STEROID_BIOSYNTHETIC_PROCESS		29	0.23763187	0.8382886	0.7708333	1	1	604	tags=48%, list=32%, signal=70%
GOBP_CYTOSKELETON_ORGANIZATION		211	0.16981353	0.8378741	0.9142857	1	1	304	tags=17%, list=16%, signal=18%
GOBP_REGULATION_OF_CARDIAC_MUSCLE_CONTRACTION_BY_CALCIUM_IO		16	0.28907788	0.8373963	0.7	1	1	478	tags=50%, list=26%, signal=67%
REACTOME_HIV_INFECTION		58	0.20304033	0.8368558	0.74358976	1	1	590	tags=36%, list=32%, signal=51%
GOBP_POSITIVE_REGULATION_OF_PROTEIN_PHOSPHORYLATION		86	0.19547583	0.8367785	0.7916667	1	1	269	tags=17%, list=14%, signal=19%
GOBP_NEGATIVE_REGULATION_OF_CANONICAL_WNT_SIGNALING_PATHWAY		38	0.22225082	0.8358131	0.73913044	1	1	209	tags=16%, list=11%, signal=17%
REACTOME_RHOG_GTPASE_CYCLE		27	0.24581899	0.8355641	0.7234042	1	1	378	tags=33%, list=20%, signal=41%
GOBP_REGULATION_OF_CELLULAR_RESPONSE_TO_TRANSFORMING_GROW		16	0.29413557	0.8352584	0.65384614	1	1	482	tags=44%, list=26%, signal=58%
GOBP_REGULATION_OF_ANIMAL_ORGAN_MORPHOGENESIS		47	0.2210479	0.8340886	0.8	1	1	205	tags=15%, list=11%, signal=16%
GOBP_REGULATION_OF_IMMUNE_SYSTEM_PROCESS		177	0.17052004	0.8338036	0.8947368	1	1	401	tags=21%, list=21%, signal=24%
GOBP_REGULATION_OF_PROTEIN_SERINE_THREONINE_KINASE_ACTIVITY		54	0.2061439	0.8335485	0.78571427	1	1	482	tags=30%, list=26%, signal=39%
GOBP_POSITIVE_REGULATION_OF_EPITHELIAL_CELL_PROLIFERATION		13	0.29641476	0.8330094	0.6981132	1	1	128	tags=15%, list=7%, signal=16%
GOBP_REGULATION_OF_SECRETION		84	0.18829471	0.8328094	0.7818182	1	1	518	tags=38%, list=28%, signal=50%
GOBP_AMEBOIDAL_TYPE_CELL_MIGRATION		69	0.19574806	0.8325463	0.7234042	1	1	384	tags=23%, list=21%, signal=28%
GOBP_POSITIVE_REGULATION_OF_PROTEOLYSIS_INVOLVED_IN_CELLULAR		21	0.25923797	0.8324411	0.73214287	1	1	131	tags=14%, list=7%, signal=15%

GOBP_ORGANIC_HYDROXY_COMPOUND_BIOSYNTHETIC_PROCESS	33	0.22537796	0.8322768	0.7058824	1	1	604	tags=48%, list=32%, signal=70%
REACTOME_PCP_CE_PATHWAY	42	0.22607289	0.83208686	0.76086956	1	1	721	tags=50%, list=39%, signal=80%
GOBP_POSITIVE_REGULATION_OF_DEVELOPMENTAL_PROCESS	149	0.17140725	0.8318781	0.8367347	1	1	384	tags=21%, list=21%, signal=24%
GOBP_NEUTRAL_LIPID_BIOSYNTHETIC_PROCESS	10	0.31525704	0.83173805	0.754717	1	1	156	tags=20%, list=8%, signal=22%
GOBP_NEGATIVE_REGULATION_OF_GENE_EXPRESSION	186	0.16344371	0.8311646	0.9268293	1	1	434	tags=25%, list=23%, signal=30%
GOBP_NUCLEAR_EXPORT	16	0.28355992	0.83040553	0.72	1	1	244	tags=19%, list=13%, signal=21%
REACTOME_EXTRACELLULAR_MATRIX_ORGANIZATION	62	0.21204194	0.83040076	0.79545456	1	1	265	tags=21%, list=14%, signal=24%
GOBP_RNA_CATABOLIC_PROCESS	123	0.1855286	0.83033204	0.87234044	1	1	434	tags=28%, list=23%, signal=35%
GOBP_DNA_GEOMETRIC_CHANGE	10	0.2939734	0.83004004	0.6363636	1	1	585	tags=40%, list=31%, signal=58%
REACTOME_REGULATION_OF_EXPRESSION_OF_SLITS_AND_ROBOS	97	0.18611617	0.8279801	0.8043478	1	1	433	tags=29%, list=23%, signal=36%
REACTOME_RAC1_GTPASE_CYCLE	27	0.2459979	0.8271262	0.7962963	1	1	131	tags=15%, list=7%, signal=16%
GOBP_INTERFERON_GAMMA_MEDIATED_SIGNALING_PATHWAY	13	0.29401714	0.82535976	0.65957445	1	1	192	tags=15%, list=10%, signal=17%
REACTOME_VEGFR2_MEDIATED_VASCULAR_PERMEABILITY	11	0.3038066	0.82497096	0.65909094	1	1	338	tags=36%, list=18%, signal=44%
KEGG_PURINE_METABOLISM	29	0.23578103	0.8243066	0.74545455	1	1	471	tags=31%, list=25%, signal=41%
GOBP_REGULATION_OF_DENDRITE_DEVELOPMENT	14	0.31004012	0.8242199	0.7169811	1	1	609	tags=50%, list=33%, signal=74%
GOBP_INOSITOL_LIPID_MEDIATED_SIGNALING	19	0.27353072	0.8242082	0.72727275	1	1	366	tags=32%, list=20%, signal=39%
GOBP_IRE1_MEDIATED_UNFOLDED_PROTEIN_RESPONSE	21	0.24522844	0.82395595	0.7962963	1	1	418	tags=33%, list=22%, signal=42%
GOBP_PROTEASOMAL_UBIQUITIN_INDEPENDENT_PROTEIN_CATABOLIC_PRO	12	0.32104087	0.8238399	0.7111111	1	1	796	tags=58%, list=43%, signal=101%
GOBP_NEGATIVE_REGULATION_OF_PROTEIN_CONTAINING_COMPLEX_DISA	16	0.27378693	0.82199264	0.74509805	1	1	66	tags=13%, list=4%, signal=13%
GOBP_CELL_SURFACE_RECEPTOR_SIGNALING_PATHWAY_INVOLVED_IN_CE	79	0.1846546	0.8205723	0.8378378	1	1	609	tags=39%, list=33%, signal=56%
GOBP_REGULATION_OF_MYELOID_CELL_DIFFERENTIATION	19	0.2833042	0.8201731	0.68	1	1	128	tags=16%, list=7%, signal=17%
GOBP_CELL_ACTIVATION	251	0.16524863	0.82009286	0.97619045	1	1	131	tags=10%, list=7%, signal=10%
GOBP_CELLULAR_RESPONSE_TO_CARBOHYDRATE_STIMULUS	19	0.26614782	0.8188973	0.7592593	1	1	471	tags=37%, list=25%, signal=49%
GOBP_REGULATION_OF_CELL_SUBSTRATE_ADHESION	50	0.2141349	0.81879133	0.85106385	1	1	256	tags=20%, list=14%, signal=23%
GOBP_POSITIVE_REGULATION_OF_EPITHELIAL_CELL_MIGRATION	27	0.25000754	0.8181671	0.78	1	1	308	tags=22%, list=16%, signal=26%
BIOCARTA_PROTEASOME_PATHWAY	15	0.27507982	0.8181372	0.6935484	1	1	656	tags=47%, list=35%, signal=71%
GOBP_REGULATION_OF_CELLULAR_COMPONENT_BIOGENESIS	170	0.16921112	0.8180762	0.8888889	1	1	371	tags=19%, list=20%, signal=22%
KEGG_GLYCEROPHOSPHOLIPID_METABOLISM	14	0.3004404	0.8177935	0.6818182	1	1	504	tags=50%, list=27%, signal=68%
GOBP_ANTIGEN_PROCESSING_AND_PRESENTATION_OF_PEPTIDE_ANTIGEN	72	0.19401905	0.8177383	0.7777778	1	1	205	tags=14%, list=11%, signal=15%
REACTOME_METABOLISM_OF_RNA	117	0.1827837	0.8157219	0.8604651	1	1	433	tags=28%, list=23%, signal=34%
REACTOME_EUKARYOTIC_TRANSLATION_INITIATION	71	0.1900847	0.81416345	0.8857143	1	1	433	tags=31%, list=23%, signal=39%
GOBP_REGULATION_OF_LIPASE_ACTIVITY	15	0.28125927	0.8140966	0.7209302	1	1	297	tags=27%, list=16%, signal=31%
GOBP_MODULATION_BY_HOST_OF_VIRAL_PROCESS	10	0.31208742	0.81403923	0.71153843	1	1	227	tags=40%, list=12%, signal=45%
GOBP_REGULATION_OF_NIK_NF_KAPPAB_SIGNALING	13	0.2850491	0.81399536	0.75510204	1	1	342	tags=31%, list=18%, signal=37%
GOBP_VASCULOGENESIS	11	0.28977767	0.81360066	0.7358491	1	1	131	tags=18%, list=7%, signal=19%
REACTOME_RHOQ_GTPASE_CYCLE	17	0.28386217	0.8134019	0.7446808	1	1	253	tags=24%, list=14%, signal=27%
GOBP_TISSUE_MIGRATION	55	0.22252107	0.8133946	0.8372093	1	1	384	tags=25%, list=21%, signal=31%
GOBP_POSITIVE_REGULATION_OF_NIK_NF_KAPPAB_SIGNALING	11	0.32772616	0.81290007	0.64444447	1	1	227	tags=27%, list=12%, signal=31%
REACTOME_SELENOAMINO_ACID_METABOLISM	70	0.19748887	0.8128315	0.8636364	1	1	433	tags=31%, list=23%, signal=39%
GOBP_PHOSPHATIDYLCHOLINE_METABOLIC_PROCESS	13	0.28974092	0.810397	0.6956522	1	1	196	tags=23%, list=10%, signal=26%
GOBP_VESICLE_TARGETING_TO_FROM_OR_WITHIN_GOLGI	20	0.25734138	0.8102273	0.7457627	1	1	361	tags=30%, list=19%, signal=37%
GOBP_GRANULOCYTE_CHEMOTAXIS	20	0.26142457	0.8100114	0.7948718	1	1	380	tags=25%, list=20%, signal=31%
GOBP_NEGATIVE_REGULATION_OF_CELL_DEVELOPMENT	18	0.27140296	0.8091606	0.74418604	1	1	384	tags=28%, list=21%, signal=35%
REACTOME_NEGATIVE_REGULATION_OF_NOTCH4_SIGNALING	33	0.213494	0.8080955	0.79591835	1	1	714	tags=48%, list=38%, signal=77%
GOBP_REGULATION_OF_POTASSIUM_ION_TRANSMEMBRANE_TRANSPORTE	17	0.2661521	0.80809194	0.76	1	1	407	tags=35%, list=22%, signal=45%
REACTOME_CARDIAC_CONDUCTION	23	0.25233427	0.8079577	0.775	1	1	194	tags=22%, list=10%, signal=24%
GOBP_NEURON_PROJECTION_ORGANIZATION	16	0.28631443	0.8079569	0.7234042	1	1	202	tags=19%, list=11%, signal=21%
GOBP_RESPONSE_TO_CADMIUM_ION	10	0.33366153	0.8064643	0.74358976	1	1	74	tags=20%, list=4%, signal=21%
GOBP_INFLAMMATORY_RESPONSE_TO_ANTIGENIC_STIMULUS	11	0.29463422	0.8064048	0.64285713	1	1	426	tags=36%, list=23%, signal=47%
REACTOME_VESICLE_MEDIATED_TRANSPORT	167	0.16416696	0.80547136	0.84615386	1	1	205	tags=13%, list=11%, signal=13%
GOBP_SULFUR_COMPOUND_BIOSYNTHETIC_PROCESS	43	0.21619567	0.80509865	0.7755102	1	1	275	tags=21%, list=15%, signal=24%
GOBP_CELLULAR_RESPONSE_TO_ABIOTIC_STIMULUS	45	0.20892833	0.8050317	0.76086956	1	1	407	tags=22%, list=22%, signal=28%
REACTOME_HSP90_CHAPERONE_CYCLE_FOR_STEROID_HORMONE_RECEPT	23	0.25611874	0.8046103	0.74358976	1	1	211	tags=17%, list=11%, signal=19%
KEGG_ANTIGEN_PROCESSING_AND_PRESENTATION	17	0.27368605	0.803781	0.7407407	1	1	117	tags=18%, list=6%, signal=19%
GOBP_NEGATIVE_REGULATION_OF_DEFENSE_RESPONSE	27	0.23929879	0.8037521	0.725	1	1	182	tags=15%, list=10%, signal=16%
GOBP_RAS_PROTEIN_SIGNAL_TRANSDUCTION	56	0.21218112	0.8027723	0.80851066	1	1	434	tags=25%, list=23%, signal=32%
GOBP_NEGATIVE_REGULATION_OF_INTRINSIC_APOPTOTIC_SIGNALING_PAT	19	0.27558097	0.80196667	0.75555557	1	1	420	tags=47%, list=22%, signal=60%

GOBP_REGULATION_OF_PROTEASOMAL_UBIQUITIN_DEPENDENT_PROTEIN_		23	0.2598383	0.8014238	0.7380952	1	1	244	tags=17%, list=13%, signal=20%
GOBP_GLIAL_CELL_DEVELOPMENT		14	0.29703096	0.80091256	0.70212764	1	1	598	tags=43%, list=32%, signal=63%
GOBP_POSITIVE_REGULATION_OF_TRANSFERASE_ACTIVITY		73	0.18749481	0.80066967	0.8888889	1	1	118	tags=10%, list=6%, signal=10%
GOBP_COTRANSLATIONAL_PROTEIN_TARGETING_TO_MEMBRANE		66	0.19178654	0.80066	0.82	1	1	433	tags=32%, list=23%, signal=40%
GOBP_REGULATION_OF_CYTOSOLIC_CALCIUM_ION_CONCENTRATION		42	0.21218288	0.8003811	0.9148936	1	1	478	tags=36%, list=26%, signal=47%
GOBP_REGULATION_OF_DENDRITIC_SPINE_DEVELOPMENT		10	0.32911685	0.7997194	0.6956522	1	1	380	tags=30%, list=20%, signal=37%
GOBP_NEGATIVE_REGULATION_OF_LOCOMOTION		40	0.2250913	0.7995598	0.7659575	1	1	384	tags=25%, list=21%, signal=31%
GOBP_CARBOHYDRATE_HOMEOSTASIS		35	0.2156922	0.79939425	0.75555557	1	1	471	tags=34%, list=25%, signal=45%
GOBP_CELL_CELL_ADHESION_VIA_PLASMA_MEMBRANE_ADHESION_MOLEC		21	0.26888397	0.7977396	0.64102566	1	1	338	tags=29%, list=18%, signal=34%
GOBP_ACTIN_CYTOSKELETON_REORGANIZATION		21	0.24635473	0.7968523	0.6875	1	1	57	tags=10%, list=3%, signal=10%
REACTOME_PLASMA_LIPOPROTEIN_ASSEMBLY_REMODELING_AND_CLEARA		17	0.26664513	0.79659086	0.8035714	1	1	582	tags=53%, list=31%, signal=76%
GOBP_REGULATION_OF_RELEASE_OF_SEQUESTERED_CALCIUM_ION_INTO_		16	0.2995993	0.7961893	0.65217394	1	1	478	tags=50%, list=26%, signal=67%
GOBP_RIBONUCLEOSIDE_METABOLIC_PROCESS		19	0.25976157	0.79607725	0.78431374	1	1	347	tags=26%, list=19%, signal=32%
REACTOME_N_GLYCAN_TRIMMING_IN_THE_ER_AND_CALNEXIN_CALRETICUL		12	0.29430255	0.79428434	0.7826087	1	1	126	tags=25%, list=7%, signal=27%
REACTOME_TOLL_LIKE_RECEPTOR_CASCADES		22	0.26201427	0.7941362	0.78	1	1	482	tags=27%, list=26%, signal=36%
GOBP_STRESS_ACTIVATED_PROTEIN_KINASE_SIGNALING_CASCADE		27	0.2431932	0.7937437	0.8235294	1	1	337	tags=22%, list=18%, signal=27%
GOBP_CELL_PROJECTION_ASSEMBLY		74	0.18358059	0.79248077	0.8666667	1	1	539	tags=34%, list=29%, signal=46%
GOBP_COPII_COATED_VESICLE_BUDDING		21	0.23982114	0.7923814	0.7647059	1	1	361	tags=29%, list=19%, signal=35%
GOBP_NUCLEAR_TRANSCRIBED_MRNA_CATABOLIC_PROCESS_NONSENSE_		65	0.19483949	0.79129064	0.8867925	1	1	433	tags=32%, list=23%, signal=41%
REACTOME_CDC42_GTPASE_CYCLE		23	0.24624553	0.790054	0.84090906	1	1	253	tags=17%, list=14%, signal=20%
GOBP_GLYCEROLIPID_CATABOLIC_PROCESS		11	0.29365537	0.78949875	0.77272725	1	1	469	tags=45%, list=25%, signal=60%
GOBP_ORGANIC_ACID_TRANSPORT		45	0.20097408	0.78944695	0.87234044	1	1	190	tags=16%, list=10%, signal=17%
GOBP_DENDRITE_MORPHOGENESIS		16	0.29002354	0.7890688	0.75	1	1	661	tags=50%, list=35%, signal=77%
GOBP_HORMONE_METABOLIC_PROCESS		23	0.24183816	0.7888536	0.6938776	1	1	61	tags=9%, list=3%, signal=9%
GOBP_POSITIVE_REGULATION_OF_CELLULAR_PROTEIN_CATABOLIC_PROC		30	0.22256109	0.78706706	0.81632656	1	1	352	tags=23%, list=19%, signal=28%
GOBP_TRIGLYCERIDE_BIOSYNTHETIC_PROCESS		10	0.31525704	0.7866576	0.7380952	1	1	156	tags=20%, list=8%, signal=22%
GOBP_REGULATION_OF_PHAGOCYTOSIS		23	0.23359396	0.78641343	0.74	1	1	453	tags=30%, list=24%, signal=40%
GOBP_VASCULAR_TRANSPORT		16	0.26565585	0.78591007	0.8181818	1	1	146	tags=19%, list=8%, signal=20%
GOBP_RNA_DEPENDENT_DNA_BIOSYNTHETIC_PROCESS		14	0.28553092	0.78557444	0.76	1	1	269	tags=29%, list=14%, signal=33%
GOBP_RESPONSE_TO_AXON_INJURY		12	0.27182698	0.78370494	0.7906977	1	1	453	tags=42%, list=24%, signal=55%
GOBP_REGULATION_OF_AUTOPHAGY		60	0.19731213	0.78369105	0.9	1	1	380	tags=20%, list=20%, signal=24%
REACTOME_TRANSCRIPTIONAL_REGULATION_BY_RUNX1		41	0.20125237	0.7834803	0.82051283	1	1	683	tags=41%, list=37%, signal=64%
GOBP_AUTOPHAGY_OF_MITOCHONDRION		15	0.27932978	0.7831339	0.84782606	1	1	380	tags=33%, list=20%, signal=41%
GOBP_REGULATION_OF_RECEPTOR_MEDIATED_ENDOCYTOSIS		26	0.24673508	0.78303397	0.85365856	1	1	254	tags=23%, list=14%, signal=26%
GOBP_NEURON_DEVELOPMENT		132	0.17240645	0.78165144	0.875	1	1	407	tags=24%, list=22%, signal=29%
GOBP_NEGATIVE_REGULATION_OF_CELLULAR_COMPONENT_ORGANIZATIO		116	0.17725419	0.78146875	0.94871795	1	1	384	tags=22%, list=21%, signal=26%
GOBP_TRANSMEMBRANE_RECEPTOR_PROTEIN_TYROSINE_KINASE_SIGNAL		97	0.17309777	0.780933	0.9111111	1	1	409	tags=24%, list=22%, signal=29%
GOBP_NUCLEOSIDE_METABOLIC_PROCESS		24	0.22866799	0.7790924	0.80851066	1	1	347	tags=29%, list=19%, signal=35%
GOBP_NEGATIVE_REGULATION_OF_ACTIN_FILAMENT_POLYMERIZATION		19	0.24363767	0.7786681	0.8113208	1	1	66	tags=11%, list=4%, signal=11%
GOBP_REGULATION_OF_SUBSTRATE_ADHESION_DEPENDENT_CELL_SPREA		23	0.21851707	0.77824897	0.8979592	1	1	457	tags=30%, list=24%, signal=40%
GOBP_CELL_CELL_SIGNALING_BY_WNT		77	0.17449377	0.7780711	0.8717949	1	1	609	tags=39%, list=33%, signal=55%
GOBP_ACTIVATION_OF_IMMUNE_RESPONSE		89	0.18155518	0.7779893	0.8913044	1	1	337	tags=18%, list=18%, signal=21%
REACTOME_DOWNSTREAM_SIGNALING_EVENTS_OF_B_CELL_RECEPTOR_B		35	0.20282811	0.77798337	0.8	1	1	769	tags=54%, list=41%, signal=90%
REACTOME_ARACHIDONIC_ACID_METABOLISM		14	0.282576	0.77777344	0.72727275	1	1	62	tags=14%, list=3%, signal=15%
GOBP_REGULATION_OF_ACTIN_FILAMENT_LENGTH		47	0.18920013	0.77739114	0.90384614	1	1	149	tags=11%, list=8%, signal=11%
GOBP_POSITIVE_REGULATION_OF_CALCIUM_ION_TRANSMEMBRANE_TRANS		12	0.28443065	0.774158	0.8043478	1	1	475	tags=42%, list=25%, signal=55%
GOBP_CELLULAR_COMPONENT_DISASSEMBLY		154	0.16424908	0.77355313	1	1	1	247	tags=14%, list=13%, signal=15%
GOBP_POSITIVE_REGULATION_OF_PROTEIN_KINASE_ACTIVITY		60	0.18338698	0.77315164	0.8979592	1	1	482	tags=27%, list=26%, signal=35%
REACTOME_UB_SPECIFIC_PROCESSING_PROTEASES		46	0.18868963	0.7725529	0.9189189	1	1	683	tags=41%, list=37%, signal=63%
GOBP_NUCLEOSIDE_MONOPHOSPHATE_METABOLIC_PROCESS		19	0.24222814	0.7725007	0.65	1	1	344	tags=26%, list=18%, signal=32%
GOBP_NEGATIVE_REGULATION_OF_ACTIN_FILAMENT_DEPOLYMERIZATION		16	0.27378693	0.77237594	0.84444445	1	1	66	tags=13%, list=4%, signal=13%
GOBP_HOMOTYPIC_CELL_CELL_ADHESION		33	0.22015202	0.7699037	0.9047619	1	1	457	tags=36%, list=24%, signal=47%
REACTOME_BIOLOGICAL_OXIDATIONS		35	0.21654533	0.76944476	0.8727273	1	1	240	tags=17%, list=13%, signal=19%
GOBP_REGULATION_OF_ANATOMICAL_STRUCTURE_SIZE		101	0.17716458	0.76831067	0.93877554	1	1	149	tags=11%, list=8%, signal=11%
REACTOME_POST_TRANSLATIONAL_PROTEIN_MODIFICATION		208	0.15116933	0.7675844	0.9512195	1	1	394	tags=21%, list=21%, signal=24%
REACTOME_GLYCOGEN_METABOLISM		11	0.29372254	0.76704127	0.78723407	1	1	299	tags=27%, list=16%, signal=32%
REACTOME_THE_ROLE_OF_GTSE1_IN_G2_M_PROGRESSION_AFTER_G2_CH		39	0.20627172	0.76687557	0.8666667	1	1	185	tags=13%, list=10%, signal=14%

GOBP_ANTIGEN_PROCESSING_AND_PRESENTATION_OF_PEPTIDE_ANTIGEN		49	0.19469196	0.76656705	0.8947368	1	1	185	tags=12%, list=10%, signal=13%
GOBP_REGULATION_OF_DEFENSE_RESPONSE		101	0.1693364	0.76540047	0.9318182	1	1	338	tags=17%, list=18%, signal=19%
GOBP_REGULATION_OF_INNATE_IMMUNE_RESPONSE		60	0.18469538	0.7647763	0.8863636	1	1	185	tags=10%, list=10%, signal=11%
GOBP_NEGATIVE_REGULATION_OF_WNT_SIGNALING_PATHWAY		41	0.18960038	0.76413554	0.8913044	1	1	425	tags=24%, list=23%, signal=31%
REACTOME_FCERI_MEDIATED_NF_KB_ACTIVATION		34	0.19822632	0.7640344	0.8095238	1	1	714	tags=47%, list=38%, signal=75%
GOBP_NON_CANONICAL_WNT_SIGNALING_PATHWAY		47	0.19530034	0.7639143	0.8333333	1	1	205	tags=15%, list=11%, signal=16%
GOBP_POSITIVE_REGULATION_OF_LIPID_METABOLIC_PROCESS		25	0.23507956	0.7631026	0.78723407	1	1	339	tags=24%, list=18%, signal=29%
GOBP_FATTY_ACYL_COA_BIOSYNTHETIC_PROCESS		11	0.3044413	0.76299846	0.7173913	1	1	133	tags=18%, list=7%, signal=19%
GOBP_FOREBRAIN_DEVELOPMENT		29	0.22984774	0.7629602	0.84090906	1	1	328	tags=24%, list=18%, signal=29%
GOBP_PHOSPHOLIPID_BIOSYNTHETIC_PROCESS		40	0.20142943	0.76266694	0.84090906	1	1	511	tags=33%, list=27%, signal=44%
GOBP_GLYCEROPHOSPHOLIPID_METABOLIC_PROCESS		54	0.19194892	0.7619554	0.88372093	1	1	511	tags=35%, list=27%, signal=47%
BIOCARTA_UCALPAIN_PATHWAY		12	0.29476264	0.7611836	0.79591835	1	1	97	tags=17%, list=5%, signal=17%
REACTOME_AUTOPHAGY		38	0.20250729	0.7611353	0.84444445	1	1	244	tags=18%, list=13%, signal=21%
GOBP_FC_RECEPTOR_SIGNALING_PATHWAY		58	0.19177762	0.7604537	0.81578946	1	1	337	tags=17%, list=18%, signal=20%
GOBP_NEGATIVE_REGULATION_OF_GROWTH		21	0.24749129	0.760426	0.775	1	1	352	tags=33%, list=19%, signal=41%
GOBP_NEUROGENESIS		164	0.16382067	0.7604039	0.9811321	1	1	407	tags=23%, list=22%, signal=27%
GOBP_REGULATION_OF_GROWTH		67	0.17807907	0.7591848	0.9130435	1	1	513	tags=33%, list=27%, signal=44%
GOBP_CELLULAR_ALDEHYDE_METABOLIC_PROCESS		17	0.26137882	0.7584105	0.75	1	1	244	tags=24%, list=13%, signal=27%
KEGG_ECM_RECEPTOR_INTERACTION		27	0.22893386	0.7576592	0.8333333	1	1	400	tags=37%, list=21%, signal=46%
GOBP_POSITIVE_REGULATION_OF_ACTIN_FILAMENT_POLYMERIZATION		23	0.23809963	0.7573319	0.8604651	1	1	328	tags=22%, list=18%, signal=26%
GOBP_REGULATION_OF_REPRODUCTIVE_PROCESS		10	0.30473313	0.7570108	0.76	1	1	233	tags=20%, list=12%, signal=23%
REACTOME_AUF1_HNRNP_D0_BINDS_AND_DESTABILIZES_MRNA		34	0.21845315	0.7567909	0.88	1	1	683	tags=44%, list=37%, signal=68%
REACTOME_REGULATION_OF_MRNA_STABILITY_BY_PROTEINS_THAT_BIND_		39	0.19993286	0.75656104	0.85365856	1	1	683	tags=44%, list=37%, signal=67%
REACTOME_ADAPTIVE_IMMUNE_SYSTEM		140	0.15839358	0.75578415	1	1	1	276	tags=14%, list=15%, signal=16%
GOBP_NEGATIVE_REGULATION_OF_ORGANELLE_ORGANIZATION		53	0.18689813	0.75560343	0.8684211	1	1	407	tags=25%, list=22%, signal=30%
GOBP_POSTSYNAPSE_ORGANIZATION		23	0.23574004	0.75534046	0.8292683	1	1	380	tags=26%, list=20%, signal=32%
GOBP_NEURON_DIFFERENTIATION		142	0.15850484	0.7544252	0.9736842	1	1	407	tags=23%, list=22%, signal=27%
GOBP_ENDOCYTIC_RECYCLING		14	0.24629955	0.753406	0.8181818	1	1	535	tags=43%, list=29%, signal=60%
GOBP_POSITIVE_REGULATION_OF_CALCIUM_ION_TRANSMEMBRANE_TRANS		12	0.28443065	0.751383	0.754717	1	1	475	tags=42%, list=25%, signal=55%
REACTOME_COLLAGEN_BIOSYNTHESIS_AND_MODIFYING_ENZYMES		12	0.28526253	0.7509959	0.73333335	1	1	333	tags=33%, list=18%, signal=40%
GOBP_ESTABLISHMENT_OF_CELL_POLARITY		24	0.21712877	0.7504926	0.78	1	1	539	tags=42%, list=29%, signal=58%
GOBP_CORTICAL_ACTIN_CYTOSKELETON_ORGANIZATION		15	0.26360062	0.750078	0.8604651	1	1	291	tags=27%, list=16%, signal=31%
GOBP_MORPHOGENESIS_OF_A_POLARIZED_EPITHELIUM		47	0.19474863	0.7496084	0.8947368	1	1	590	tags=36%, list=32%, signal=52%
GOBP_RELEASE_OF_SEQUESTERED_CALCIUM_ION_INTO_CYTOSOL_BY_EN		12	0.27332893	0.74772006	0.8627451	1	1	478	tags=50%, list=26%, signal=67%
GOBP_NEGATIVE_REGULATION_OF_INFLAMMATORY_RESPONSE		24	0.23940863	0.74733466	0.8490566	1	1	146	tags=13%, list=8%, signal=13%
GOBP_SMOOTH_MUSCLE_CONTRACTION		14	0.28205124	0.74701965	0.82978725	1	1	453	tags=36%, list=24%, signal=47%
GOBP_REGULATION_OF_CELLULAR_CATABOLIC_PROCESS		163	0.157601	0.74439615	1	1	1	392	tags=20%, list=21%, signal=23%
REACTOME_SIGNALING_BY_ROBO_RECEPTORS		109	0.16146626	0.74391025	0.92156863	1	1	433	tags=28%, list=23%, signal=34%
GOBP_GOLGI_VESICLE_TRANSPORT		83	0.1724803	0.7430583	0.9166667	1	1	571	tags=34%, list=31%, signal=46%
GOBP_NEGATIVE_REGULATION_OF_ENDOTHELIAL_CELL_PROLIFERATION		13	0.26889604	0.741326	0.82978725	1	1	340	tags=31%, list=18%, signal=37%
GOBP_PROTEIN_MATURATION		53	0.18853924	0.7405035	0.9142857	1	1	349	tags=23%, list=19%, signal=27%
GOBP_NEGATIVE_REGULATION_OF_BINDING		14	0.25504568	0.74005413	0.8780488	1	1	244	tags=21%, list=13%, signal=24%
GOBP_CEREBRAL_CORTEX_DEVELOPMENT		12	0.26133338	0.7391603	0.9	1	1	394	tags=33%, list=21%, signal=42%
REACTOME_TNFR2_NON_CANONICAL_NF_KB_PATHWAY		33	0.20395188	0.7388965	0.8627451	1	1	714	tags=48%, list=38%, signal=77%
GOBP_REGULATION_OF_UBIQUITIN_DEPENDENT_PROTEIN_CATABOLIC_PRO		27	0.21150492	0.7388332	0.8684211	1	1	244	tags=15%, list=13%, signal=17%
REACTOME_DECTIN_1_MEDIATED_NONCANONICAL_NF_KB_SIGNALING		33	0.20395188	0.7388246	0.7619048	1	1	714	tags=48%, list=38%, signal=77%
GOBP_PEPTIDYL_THREONINE_MODIFICATION		20	0.24041052	0.73834956	0.81632656	1	1	1425	tags=100%, list=76%, signal=415%
GOBP_DE_NOVO_PROTEIN_FOLDING		14	0.27237082	0.73725396	0.67391306	1	1	72	tags=14%, list=4%, signal=15%
GOBP_NEGATIVE_REGULATION_OF_PROTEIN_BINDING		13	0.28250748	0.73696864	0.82222223	1	1	244	tags=23%, list=13%, signal=26%
GOBP_REGULATION_OF_PROTEIN_KINASE_ACTIVITY		93	0.17254458	0.7354613	0.98	1	1	482	tags=27%, list=26%, signal=34%
GOBP_REGULATION_OF_CELL_KILLING		10	0.2865788	0.73517156	0.72	1	1	38	tags=10%, list=2%, signal=10%
GOBP_REGULATION_OF_STEROL_TRANSPORT		12	0.2758042	0.734636	0.7755102	1	1	146	tags=25%, list=8%, signal=27%
GOBP_PALLIUM_DEVELOPMENT		16	0.25427124	0.7343925	0.85714287	1	1	277	tags=25%, list=15%, signal=29%
GOBP_QUINONE_METABOLIC_PROCESS		14	0.2629413	0.7330654	0.9230769	1	1	245	tags=21%, list=13%, signal=24%
REACTOME_DISEASES_OF_SIGNAL_TRANSDUCTION_BY_GROWTH_FACTOR_		81	0.16938564	0.7327703	0.97727275	1	1	400	tags=22%, list=21%, signal=27%
GOBP_MICROTUBULE_BASED_MOVEMENT		34	0.20756748	0.7325348	0.8378378	1	1	592	tags=35%, list=32%, signal=51%
GOBP_ANTIGEN_PROCESSING_AND_PRESENTATION		82	0.16979644	0.7324944	0.9787234	1	1	205	tags=12%, list=11%, signal=13%

GOBP_FOCAL_ADHESION_ASSEMBLY		22	0.22933604	0.7319847	0.89361703	1	1	212	tags=14%, list=11%, signal=15%
BIOCARTA_PTDINS_PATHWAY		11	0.27244443	0.73195934	0.7777778	1	1	609	tags=55%, list=33%, signal=80%
GOBP_POLYSACCHARIDE_BIOSYNTHETIC_PROCESS		13	0.2762586	0.7246022	0.8666667	1	1	309	tags=31%, list=17%, signal=37%
GOBP_MRNA_PROCESSING		19	0.23570096	0.7231897	0.8888889	1	1	389	tags=26%, list=21%, signal=33%
REACTOME_FCGAMMA_RECEPTOR_FCGR_DEPENDENT_PHAGOCYTOSIS		23	0.21466616	0.7226412	0.877551	1	1	337	tags=22%, list=18%, signal=26%
GOBP_ACTIN_POLYMERIZATION_OR_DEPOLYMERIZATION		50	0.18095441	0.7223518	0.93333334	1	1	202	tags=12%, list=11%, signal=13%
REACTOME_SIGNALING_BY_NOTCH		39	0.20259376	0.72231686	0.8611111	1	1	769	tags=54%, list=41%, signal=90%
REACTOME_FACTORS_INVOLVED_IN_MEGAKARYOCYTE_DEVELOPMENT_AN		26	0.21922153	0.72209924	0.85	1	1	233	tags=19%, list=12%, signal=22%
REACTOME_CLEC7A_DECTIN_1_SIGNALING		35	0.19982488	0.72205687	0.9361702	1	1	714	tags=46%, list=38%, signal=73%
GOBP_REGULATION_OF_CATABOLIC_PROCESS		176	0.1524559	0.7203032	1	1	1	392	tags=20%, list=21%, signal=23%
GOBP_REGULATION_OF_CELL_MATRIX_ADHESION		26	0.21201819	0.7195542	0.85365856	1	1	212	tags=15%, list=11%, signal=17%
REACTOME_SIGNALING_BY_NOTCH4		33	0.213494	0.7125761	0.9047619	1	1	714	tags=48%, list=38%, signal=77%
GOBP_PROTEIN_LOCALIZATION_TO_VACUOLE		17	0.2278956	0.7121897	0.8888889	1	1	232	tags=18%, list=12%, signal=20%
REACTOME_HOST_INTERACTIONS_OF_HIV_FACTORS		51	0.17855531	0.711953	0.9574468	1	1	690	tags=43%, list=37%, signal=66%
GOBP_REGULATION_OF_CARDIAC_MUSCLE_CONTRACTION_BY_REGULATIO		12	0.27332893	0.7118763	0.8666667	1	1	478	tags=50%, list=26%, signal=67%
REACTOME_NCAM_SIGNALING_FOR_NEURITE_OUT_GROWTH		13	0.2591044	0.71032244	0.8666667	1	1	563	tags=38%, list=30%, signal=55%
GOBP_POSITIVE_REGULATION_OF_PROTEIN_CONTAINING_COMPLEX_ASSE		59	0.17291753	0.7100008	0.9318182	1	1	352	tags=20%, list=19%, signal=24%
GOBP_ARP2_3_COMPLEX_MEDIATED_ACTIN_NUCLEATION		14	0.24401765	0.70967484	0.8965517	1	1	328	tags=21%, list=18%, signal=26%
GOBP_RESPONSE_TO_UV		10	0.2797548	0.7096393	0.88095236	1	1	300	tags=20%, list=16%, signal=24%
GOBP_REGULATION_OF_SIGNALING_RECEPTOR_ACTIVITY		14	0.24340643	0.7088846	0.81632656	1	1	40	tags=7%, list=2%, signal=7%
GOBP_CELLULAR_GLUCAN_METABOLIC_PROCESS		16	0.24332045	0.70792925	0.90697676	1	1	309	tags=25%, list=17%, signal=30%
REACTOME_S_PHASE		34	0.19060662	0.7078007	0.96153843	1	1	714	tags=47%, list=38%, signal=75%
REACTOME_CELL_EXTRACELLULAR_MATRIX_INTERACTIONS		10	0.29201865	0.7075726	0.8108108	1	1	18	tags=10%, list=1%, signal=10%
REACTOME_GLYCEROPHOSPHOLIPID_BIOSYNTHESIS		27	0.20425566	0.70616376	0.9	1	1	504	tags=37%, list=27%, signal=50%
GOBP_GLUCOSE_IMPORT		13	0.24745117	0.7058734	0.8888889	1	1	406	tags=31%, list=22%, signal=39%
GOBP_FILOPODIUM_ASSEMBLY		14	0.24103151	0.7055161	0.90697676	1	1	539	tags=36%, list=29%, signal=50%
GOBP_TAXIS		84	0.16205537	0.7021994	0.9756098	1	1	406	tags=21%, list=22%, signal=26%
GOBP_MAINTENANCE_OF_BLOOD_BRAIN_BARRIER		10	0.27649418	0.7017666	0.81632656	1	1	338	tags=30%, list=18%, signal=36%
GOBP_EPITHELIAL_CELL_APOPTOTIC_PROCESS		13	0.2583423	0.7009565	0.8333333	1	1	457	tags=38%, list=24%, signal=51%
GOBP_REGULATION_OF_STRESS_ACTIVATED_PROTEIN_KINASE_SIGNALING		19	0.2381021	0.7009022	0.85714287	1	1	337	tags=21%, list=18%, signal=25%
GOBP_CELL_MATRIX_ADHESION		51	0.18642533	0.7008859	0.9130435	1	1	256	tags=16%, list=14%, signal=18%
GOBP_RESPONSE_TO_INTERLEUKIN_1		42	0.19024727	0.7002482	0.9583333	1	1	425	tags=24%, list=23%, signal=30%
GOBP_PHOSPHATIDYLGLYCEROL_METABOLIC_PROCESS		14	0.24328087	0.69815475	0.87234044	1	1	502	tags=36%, list=27%, signal=48%
REACTOME_REGULATION_OF_PTEN_STABILITY_AND_ACTIVITY		33	0.19357052	0.69760126	0.9	1	1	683	tags=42%, list=37%, signal=66%
GOBP_TRANSITION_METAL_ION_HOMEOSTASIS		24	0.20943792	0.69635254	0.89361703	1	1	149	tags=13%, list=8%, signal=13%
GOBP_AUTOPHAGOSOME_ORGANIZATION		15	0.23618048	0.695525	0.8666667	1	1	760	tags=67%, list=41%, signal=111%
GOBP_VESICLE_DOCKING		18	0.2198311	0.6951944	0.85365856	1	1	518	tags=33%, list=28%, signal=46%
GOBP_REGULATION_OF_LIPID_STORAGE		10	0.27209345	0.69439065	0.9056604	1	1	339	tags=30%, list=18%, signal=36%
GOBP_REGULATION_OF_PROTEIN_AUTOPHOSPHORYLATION		11	0.2602745	0.69435716	0.877551	1	1	611	tags=45%, list=33%, signal=67%
REACTOME_CLASS_A_1_RHODOPSIN_LIKE_RECEPTORS		10	0.2594849	0.69429445	0.8863636	1	1	460	tags=30%, list=25%, signal=40%
REACTOME_TRANSCRIPTIONAL_REGULATION_BY_RUNX2		37	0.19238268	0.6932252	0.925	1	1	714	tags=46%, list=38%, signal=73%
GOBP_ENDOTHELIAL_CELL_PROLIFERATION		22	0.21573758	0.6924844	0.8717949	1	1	206	tags=18%, list=11%, signal=20%
GOBP_REGULATION_OF_HEART_RATE_BY_CARDIAC_CONDUCTION		10	0.28380528	0.6918889	0.7647059	1	1	320	tags=40%, list=17%, signal=48%
GOBP_CHOLESTEROL_EFFLUX		10	0.2712252	0.69187516	0.85106385	1	1	324	tags=30%, list=17%, signal=36%
GOBP_CELL_MATURATION		12	0.23829743	0.6914093	0.9166667	1	1	156	tags=17%, list=8%, signal=18%
GOBP_PROTEIN_LOCALIZATION_TO_LYSOSOME		13	0.26265797	0.6889102	0.92156863	1	1	232	tags=23%, list=12%, signal=26%
GOBP_REGULATION_OF_GLUCOSE_IMPORT		10	0.27715236	0.68756664	0.86	1	1	14	tags=10%, list=1%, signal=10%
GOBP_NIK_NF_KAPPAB_SIGNALING		45	0.1801448	0.68621594	0.93877554	1	1	718	tags=47%, list=38%, signal=74%
GOBP_REGULATION_OF_MRNA_METABOLIC_PROCESS		57	0.16813701	0.68292624	0.9787234	1	1	185	tags=12%, list=10%, signal=13%
GOBP_PIGMENT_BIOSYNTHETIC_PROCESS		14	0.24543291	0.6823114	0.8372093	1	1	557	tags=36%, list=30%, signal=50%
GOBP_VESICLE_ORGANIZATION		74	0.16193801	0.6796783	0.9777778	1	1	407	tags=26%, list=22%, signal=32%
GOBP_PIGMENT_METABOLIC_PROCESS		16	0.22742464	0.67952067	0.877193	1	1	557	tags=38%, list=30%, signal=53%
GOBP_L_AMINO_ACID_TRANSPORT		12	0.23479679	0.6785318	0.8372093	1	1	190	tags=17%, list=10%, signal=18%
GOBP_MITOTIC_CYTOKINESIS		13	0.24259642	0.67639416	0.85714287	1	1	434	tags=31%, list=23%, signal=40%
REACTOME_RUNX1_REGULATES_TRANSCRIPTION_OF_GENES_INVOLVED_IN		32	0.18706767	0.67562115	0.9259259	1	1	714	tags=47%, list=38%, signal=75%
GOBP_REGULATION_OF_PROTEIN_CONTAINING_COMPLEX_ASSEMBLY		102	0.15200244	0.67507875	1	1	1	260	tags=15%, list=14%, signal=16%
REACTOME_TRIGLYCERIDE_METABOLISM		11	0.25304356	0.6748416	0.8518519	1	1	502	tags=45%, list=27%, signal=62%

GOBP_ANTIGEN_PROCESSING_AND_PRESENTATION_OF_EXOGENOUS_PEP	42	0.17818083	0.67436296	0.9807692	1	1	185	tags=12%, list=10%, signal=13%
GOBP_POSITIVE_REGULATION_OF_MACROAUTOPHAGY	11	0.25954345	0.6735907	0.9019608	1	1	761	tags=55%, list=41%, signal=91%
GOBP_MITOTIC_NUCLEAR_DIVISION	21	0.20470311	0.6730371	0.925	1	1	98	tags=10%, list=5%, signal=10%
GOBP_CELL_CYCLE_ARREST	21	0.20653117	0.6725068	0.9433962	1	1	83	tags=10%, list=4%, signal=10%
GOBP_RETINA_DEVELOPMENT_IN_CAMERA_TYPE_EYE	14	0.23958015	0.671601	0.8979592	1	1	30	tags=7%, list=2%, signal=7%
REACTOME_APOPTOTIC_CLEAVAGE_OF_CELLULAR_PROTEINS	13	0.23003568	0.6699921	0.94	1	1	228	tags=15%, list=12%, signal=17%
REACTOME_MATURATION_OF_SARS_COV_2_SPIKE_PROTEIN	13	0.2391429	0.668969	0.8888889	1	1	201	tags=15%, list=11%, signal=17%
GOBP_CELL_CHEMOTAXIS	43	0.17121962	0.6689553	0.9777778	1	1	380	tags=21%, list=20%, signal=26%
GOBP_REGULATION_OF_PHOSPHATIDYLINOSITOL_3_KINASE_SIGNALING	10	0.25679797	0.6679892	0.92105263	1	1	366	tags=30%, list=20%, signal=37%
REACTOME_SCF_SKP2_MEDIATED_DEGRADATION_OF_P27_P21	32	0.18055382	0.6665905	0.9302326	1	1	714	tags=47%, list=38%, signal=75%
GOBP_VESICLE_DOCKING_INVOLVED_IN_EXOCYTOSIS	12	0.26779795	0.6657748	0.90697676	1	1	518	tags=33%, list=28%, signal=46%
GOBP_NEURON_PROJECTION_EXTENSION	29	0.19375657	0.6636423	0.93333334	1	1	283	tags=17%, list=15%, signal=20%
REACTOME_SIGNALING_BY_THE_B_CELL_RECEPTOR_BCR	40	0.18748146	0.66272116	0.8918919	1	1	769	tags=50%, list=41%, signal=83%
GOBP_MORPHOGENESIS_OF_A_BRANCHING_STRUCTURE	16	0.23410149	0.66078174	0.78723407	1	1	98	tags=13%, list=5%, signal=13%
GOBP_REGULATION_OF_MUSCLE_HYPERTROPHY	10	0.23969303	0.6603417	0.90909094	1	1	575	tags=30%, list=31%, signal=43%
REACTOME_ASSEMBLY_OF_THE_PRE_REPLICATIVE_COMPLEX	31	0.17188455	0.66022867	0.94871795	1	1	714	tags=45%, list=38%, signal=72%
GOBP_ESTABLISHMENT_OF_TISSUE_POLARITY	44	0.184194	0.6600006	0.9756098	1	1	721	tags=48%, list=39%, signal=76%
KEGG_GLYCEROLIPID_METABOLISM	13	0.24131119	0.65883684	0.85106385	1	1	549	tags=46%, list=29%, signal=65%
REACTOME_MAPK6_MAPK4_SIGNALING	37	0.1733574	0.6585225	0.975	1	1	683	tags=41%, list=37%, signal=63%
GOBP_L_ALPHA_AMINO_ACID_TRANSMEMBRANE_TRANSPORT	10	0.26378724	0.6584831	0.9583333	1	1	190	tags=20%, list=10%, signal=22%
GOBP_POSITIVE_REGULATION_OF_CELL_CYCLE	29	0.19784321	0.6580597	0.9791667	1	1	304	tags=17%, list=16%, signal=20%
GOBP_HETEROTYPIC_CELL_CELL_ADHESION	22	0.22002982	0.6576915	0.95454544	1	1	265	tags=23%, list=14%, signal=26%
GOBP_MICROTUBULE_BASED_PROCESS	82	0.15100306	0.6555693	1	1	1	157	tags=10%, list=8%, signal=10%
REACTOME_TCR_SIGNALING	34	0.17238198	0.65437883	0.96428573	1	1	714	tags=44%, list=38%, signal=70%
GOBP_LYMPHOCYTE_ACTIVATION	62	0.15731473	0.65335906	0.9767442	1	1	337	tags=18%, list=18%, signal=21%
GOBP_REGULATION_OF_CELLULAR_COMPONENT_SIZE	80	0.14617485	0.65104353	1	1	1	328	tags=16%, list=18%, signal=19%
REACTOME_ORC1_REMOVAL_FROM_CHROMATIN	32	0.18055382	0.6500905	0.94	1	1	714	tags=47%, list=38%, signal=75%
GOBP_REGULATION_OF_NUCLEOTIDE_METABOLIC_PROCESS	18	0.23010953	0.6489885	0.8684211	1	1	344	tags=28%, list=18%, signal=34%
GOBP_REGULATION_OF_ACTOMYOSIN_STRUCTURE_ORGANIZATION	22	0.19407074	0.64894146	0.9019608	1	1	1	tags=5%, list=0%, signal=4%
REACTOME_REGULATION_OF_HMOX1_EXPRESSION_AND_ACTIVITY	34	0.17549708	0.646714	1	1	1	714	tags=44%, list=38%, signal=70%
GOBP_MITOTIC_CELL_CYCLE	114	0.13665153	0.64620566	0.9787234	1	1	434	tags=20%, list=23%, signal=25%
REACTOME_INTERLEUKIN_1_FAMILY_SIGNALING	35	0.17695837	0.64256287	0.9767442	1	1	718	tags=46%, list=38%, signal=73%
GOBP_REGULATION_OF_MRNA_CATABOLIC_PROCESS	51	0.1579186	0.63568425	0.9189189	1	1	185	tags=12%, list=10%, signal=13%
GOBP_NEGATIVE_REGULATION_OF_AUTOPHAGY	10	0.23911862	0.63563454	0.8958333	1	1	1425	tags=100%, list=76%, signal=417%
REACTOME_APC_C_MEDIATED_DEGRADATION_OF_CELL_CYCLE_PROTEINS	32	0.18055382	0.6346289	0.95652175	1	1	714	tags=47%, list=38%, signal=75%
GOBP_RESPONSE_TO_TYPE_I_INTERFERON	10	0.27141896	0.63424385	0.9361702	1	1	289	tags=20%, list=15%, signal=24%
GOBP_SMALL_GTPASE_MEDIATED_SIGNAL_TRANSDUCTION	70	0.1590626	0.6336278	1	1	1	253	tags=14%, list=14%, signal=16%
REACTOME_RND3_GTPASE_CYCLE	12	0.23626354	0.6335682	0.9142857	1	1	592	tags=42%, list=32%, signal=61%
REACTOME_SWITCHING_OF_ORIGINS_TO_A_POST_REPLICATIVE_STATE	32	0.18055382	0.6330725	0.95652175	1	1	714	tags=47%, list=38%, signal=75%
REACTOME_STABILIZATION_OF_P53	31	0.17188455	0.6320669	0.97619045	1	1	714	tags=45%, list=38%, signal=72%
GOBP_NEUTROPHIL_CHEMOTAXIS	17	0.2249426	0.63144743	0.9318182	1	1	380	tags=24%, list=20%, signal=29%
REACTOME_CYCLIN_A_CDK2_ASSOCIATED_EVENTS_AT_S_PHASE_ENTRY	32	0.18055382	0.6313679	0.9148936	1	1	714	tags=47%, list=38%, signal=75%
REACTOME_INTERLEUKIN_1_SIGNALING	34	0.18126653	0.6311612	0.98	1	1	718	tags=47%, list=38%, signal=75%
REACTOME_TCF_DEPENDENT_SIGNALING_IN_RESPONSE_TO_WNT	42	0.1663872	0.63081723	0.9787234	1	1	185	tags=12%, list=10%, signal=13%
GOBP_REGULATION_OF_NERVOUS_SYSTEM_PROCESS	12	0.22976641	0.62720555	0.95	1	1	264	tags=17%, list=14%, signal=19%
GOBP_NEGATIVE_REGULATION_OF_CELL_CELL_ADHESION	18	0.20712547	0.6270639	0.9574468	1	1	718	tags=50%, list=38%, signal=80%
REACTOME_REGULATION_OF_RUNX2_EXPRESSION_AND_ACTIVITY	34	0.17186265	0.6264237	0.97959185	1	1	714	tags=47%, list=38%, signal=75%
REACTOME_DNA_REPLICATION	32	0.18055382	0.6257335	0.97619045	1	1	714	tags=47%, list=38%, signal=75%
GOBP_REGULATION_OF_CELL_JUNCTION_ASSEMBLY	37	0.16808972	0.6252558	0.98	1	1	212	tags=11%, list=11%, signal=12%
GOBP_REGULATION_OF_CELL_MORPHOGENESIS_INVOLVED_IN_DIFFERENT	28	0.18920588	0.624687	0.9591837	1	1	457	tags=29%, list=24%, signal=37%
REACTOME_CROSS_PRESENTATION_OF_SOLUBLE_EXOGENOUS_ANTIGENS	32	0.17156103	0.62405825	0.98039216	1	1	425	tags=22%, list=23%, signal=28%
REACTOME_UCH_PROTEINASES	31	0.17188455	0.62347406	0.9111111	1	1	714	tags=45%, list=38%, signal=72%
REACTOME_CELL_CYCLE	78	0.1457261	0.6227728	1	1	1	127	tags=9%, list=7%, signal=9%
GOBP_REGULATION_OF_LEUKOCYTE_MIGRATION	28	0.18229201	0.6213316	0.93333334	1	1	446	tags=29%, list=24%, signal=37%
GOBP_PORPHYRIN_CONTAINING_COMPOUND_METABOLIC_PROCESS	11	0.22003339	0.6208805	0.9411765	1	1	534	tags=45%, list=29%, signal=63%
GOBP_DEVELOPMENTAL_GROWTH_INVOLVED_IN_MORPHOGENESIS	34	0.17166394	0.6206767	0.9782609	1	1	283	tags=15%, list=15%, signal=17%
GOBP_PROTEIN_KINASE_A_SIGNALING	10	0.25083694	0.61990404	0.91803277	1	1	538	tags=40%, list=29%, signal=56%

GOBP_PRIMARY_ALCOHOL_METABOLIC_PROCESS		15	0.21012932	0.6193479	0.9285714	1	1	1480	tags=100%, list=79%, signal=475%
GOBP_INTERLEUKIN_1_MEDIATED_SIGNALING_PATHWAY		32	0.18055382	0.6185319	1	1	1	714	tags=47%, list=38%, signal=75%
GOBP_IMMUNE_SYSTEM_DEVELOPMENT		104	0.1309143	0.6166854	1	1	1	128	tags=8%, list=7%, signal=8%
GOBP_MACROPHAGE_ACTIVATION		11	0.22971839	0.61540496	0.9	1	1	118	tags=9%, list=6%, signal=10%
REACTOME_DEGRADATION_OF_GLI1_BY_THE_PROTEASOME		33	0.16620259	0.61442417	0.9777778	1	1	714	tags=45%, list=38%, signal=72%
GOBP_ANAPHASE_PROMOTING_COMPLEX_DEPENDENT_CATABOLIC_PROCE		31	0.17188455	0.61399513	1	1	1	714	tags=45%, list=38%, signal=72%
REACTOME_CLASS_I_MHC_MEDIATED_ANTIGEN_PROCESSING_PRESENTATI		72	0.14558391	0.61227584	1	1	1	482	tags=24%, list=26%, signal=31%
GOBP_REGULATION_OF_CHEMOTAXIS		28	0.1812966	0.60930824	0.97959185	1	1	380	tags=21%, list=20%, signal=26%
GOBP_POSITIVE_REGULATION_OF_PLASMA_MEMBRANE_BOUNDED_CELL_F		22	0.18094903	0.60840666	0.9423077	1	1	539	tags=32%, list=29%, signal=44%
GOBP_ORGANELLE_DISASSEMBLY		23	0.18952297	0.6075225	1	1	1	380	tags=26%, list=20%, signal=32%
REACTOME_CELL_CYCLE_MITOTIC		74	0.1392916	0.60518724	1	1	1	127	tags=8%, list=7%, signal=8%
GOBP_NEGATIVE_REGULATION_OF_CELL_CYCLE_PROCESS		42	0.15567033	0.6050328	0.9791667	1	1	233	tags=12%, list=12%, signal=13%
REACTOME_REGULATION_OF_RUNX3_EXPRESSION_AND_ACTIVITY		31	0.17188455	0.6048886	0.9777778	1	1	714	tags=45%, list=38%, signal=72%
REACTOME_C_TYPE_LECTIN_RECEPTORS_CLRS		42	0.15835792	0.60270447	0.9591837	1	1	714	tags=40%, list=38%, signal=64%
REACTOME_APC_C_CDH1_MEDIATED_DEGRADATION_OF_CDC20_AND_OTHE		31	0.17188455	0.6015587	0.975	1	1	714	tags=45%, list=38%, signal=72%
REACTOME_DEGRADATION_OF_DVL		31	0.17188455	0.60096705	0.9583333	1	1	714	tags=45%, list=38%, signal=72%
REACTOME_ASYMMETRIC_LOCALIZATION_OF_PCP_PROTEINS		31	0.17188455	0.600821	1	1	1	714	tags=45%, list=38%, signal=72%
GOBP_GLYCOSYL_COMPOUND_METABOLIC_PROCESS		30	0.17947038	0.59853834	0.9491525	1	1	347	tags=23%, list=19%, signal=28%
REACTOME_G1_S_DNA_DAMAGE_CHECKPOINTS		31	0.17188455	0.5959155	0.98	1	1	714	tags=45%, list=38%, signal=72%
GOBP_ENDOSOMAL_TRANSPORT		46	0.1626352	0.59471905	1	1	1	535	tags=33%, list=29%, signal=45%
GOBP_IN_UTERO_EMBRYONIC_DEVELOPMENT		23	0.1889114	0.5924434	0.9583333	1	1	16	tags=4%, list=1%, signal=4%
GOBP_REGULATION_OF_CELLULAR_AMINO_ACID_METABOLIC_PROCESS		32	0.17049882	0.5908612	0.9375	1	1	425	tags=22%, list=23%, signal=28%
REACTOME_DNA_REPLICATION_PRE_INITIATION		31	0.17188455	0.5884397	0.9767442	1	1	714	tags=45%, list=38%, signal=72%
REACTOME_APOPTOTIC_EXECUTION_PHASE		17	0.20680717	0.58823264	0.95454544	1	1	530	tags=29%, list=28%, signal=41%
GOBP_RNA_SPLICING		18	0.2049253	0.5880895	1	1	1	389	tags=28%, list=21%, signal=35%
REACTOME_CELLULAR_RESPONSE_TO_HYPOXIA		32	0.15576848	0.5873307	1	1	1	714	tags=44%, list=38%, signal=70%
GOBP_INTRA_GOLGI_VESICLE_MEDIATED_TRANSPORT		11	0.20168571	0.5864074	0.9607843	1	1	177	tags=18%, list=9%, signal=20%
REACTOME_DEGRADATION_OF_AXIN		31	0.17188455	0.5859483	0.97727275	1	1	714	tags=45%, list=38%, signal=72%
GOBP_POSITIVE_REGULATION_OF_CHEMOTAXIS		19	0.18377368	0.58535606	0.9583333	1	1	380	tags=21%, list=20%, signal=26%
GOBP_NEGATIVE_REGULATION_OF_CYTOSKELETON_ORGANIZATION		31	0.1683899	0.58500904	1	1	1	66	tags=6%, list=4%, signal=7%
REACTOME_FC_EPSILON_RECEPTOR_FCERI_SIGNALING		40	0.15953082	0.58497	0.9787234	1	1	714	tags=40%, list=38%, signal=63%
GOBP_MICROTUBULE_POLYMERIZATION_OR_DEPOLYMERIZATION		12	0.22206579	0.58385384	0.95652175	1	1	352	tags=25%, list=19%, signal=31%
REACTOME_ANTIGEN_PROCESSING_CROSS_PRESENTATION		53	0.14246827	0.5824397	1	1	1	482	tags=23%, list=26%, signal=30%
GOBP_POSITIVE_REGULATION_OF_WNT_SIGNALING_PATHWAY		39	0.14514999	0.5817538	1	1	1	185	tags=10%, list=10%, signal=11%
GOBP_SIGNAL_TRANSDUCTION_BY_P53_CLASS_MEDIATOR		17	0.18645132	0.5763786	1	1	1	701	tags=35%, list=37%, signal=56%
GOBP_REGULATION_OF_CELLULAR_AMINE_METABOLIC_PROCESS		35	0.15387054	0.5752702	0.9583333	1	1	683	tags=43%, list=37%, signal=66%
GOBP_CELLULAR_RESPONSE_TO_CORTICOSTEROID_STIMULUS		10	0.23965062	0.57341087	0.94	1	1	529	tags=40%, list=28%, signal=55%
GOBP_GLIOGENESIS		36	0.1677445	0.5701331	1	1	1	598	tags=36%, list=32%, signal=52%
GOBP_REGULATION_OF_LAMELLIPODIUM_ORGANIZATION		17	0.18941952	0.56875294	1	1	1	407	tags=24%, list=22%, signal=30%
REACTOME_DEUBIQUITINATION		51	0.14342172	0.56729925	1	1	1	271	tags=14%, list=14%, signal=16%
GOBP_STEM_CELL_DIFFERENTIATION		43	0.15384439	0.56616414	1	1	1	425	tags=21%, list=23%, signal=26%
GOBP_LYTIC_VACUOLE_ORGANIZATION		13	0.20317645	0.5645226	0.95	1	1	149	tags=15%, list=8%, signal=17%
REACTOME_ANTIGEN_PROCESSING_UBIQUITINATION_PROTEASOME_DEGRA		44	0.1490718	0.56396574	1	1	1	437	tags=23%, list=23%, signal=29%
REACTOME_HEDGEHOG_ON_STATE		32	0.1658698	0.5630999	1	1	1	714	tags=44%, list=38%, signal=70%
GOBP_SCF_DEPENDENT_PROTEASOMAL_UBIQUITIN_DEPENDENT_PROTEIN_		33	0.15297236	0.5576198	1	1	1	714	tags=42%, list=38%, signal=67%
GOBP_DEVELOPMENTAL_CELL_GROWTH		36	0.15679568	0.555038	1	1	1	283	tags=14%, list=15%, signal=16%
GOBP_CELLULAR_TRANSITION_METAL_ION_HOMEOSTASIS		19	0.16885167	0.5494637	0.98275864	1	1	534	tags=32%, list=29%, signal=44%
REACTOME_REGULATION_OF_RAS_BY_GAPS		33	0.1481087	0.54033476	1	1	1	714	tags=42%, list=38%, signal=67%
REACTOME_METABOLISM_OF_POLYAMINES		30	0.15541016	0.53863204	1	1	1	714	tags=43%, list=38%, signal=69%
REACTOME_INTRACELLULAR_SIGNALING_BY_SECOND_MESSENGERS		58	0.13589513	0.5380623	1	1	1	482	tags=22%, list=26%, signal=29%
GOBP_FC_EPSILON_RECEPTOR_SIGNALING_PATHWAY		37	0.15043353	0.5327572	1	1	1	714	tags=41%, list=38%, signal=64%
REACTOME_TRANSLATION_OF_SARS_COV_2_STRUCTURAL_PROTEINS		15	0.19476625	0.52804655	0.9791667	1	1	201	tags=13%, list=11%, signal=15%
REACTOME_DEFECTIVE_CFTR_CAUSES_CYSTIC_FIBROSIS		33	0.1461538	0.5274297	1	1	1	683	tags=42%, list=37%, signal=66%
GOBP_PROTEIN_MODIFICATION_BY_SMALL_PROTEIN_REMOVAL		47	0.13746953	0.52357113	0.9807692	1	1	803	tags=55%, list=43%, signal=94%
GOBP_REGULATION_OF_TRANSCRIPTION_FROM_RNA_POLYMERASE_II_PRO		33	0.15169217	0.5212061	1	1	1	714	tags=42%, list=38%, signal=67%
GOBP_NEGATIVE_REGULATION_OF_CELL_CYCLE		64	0.12868254	0.52109396	1	1	1	385	tags=17%, list=21%, signal=21%
GOBP_NEGATIVE_REGULATION_OF_MITOTIC_CELL_CYCLE		41	0.14168957	0.5207832	1	1	1	425	tags=20%, list=23%, signal=25%

GOBP_CELL_CYCLE_G1_S_PHASE_TRANSITION		18	0.17323245	0.52059686	0.9777778	1	1	1549	tags=100%, list=83%, signal=575%
GOBP_LYMPHOCYTE_MIGRATION		10	0.21883303	0.5195648	0.97959185	1	1	733	tags=60%, list=39%, signal=98%
GOBP_POSITIVE_REGULATION_OF_CANONICAL_WNT_SIGNALING_PATHWAY		36	0.1471569	0.5170522	1	1	1	185	tags=11%, list=10%, signal=12%
GOBP_REGULATION_OF_RESPONSE_TO_DNA_DAMAGE_STIMULUS		15	0.18411168	0.5152336	0.98	1	1	304	tags=13%, list=16%, signal=16%
GOBP_REGULATION_OF_CELL_CYCLE_G1_S_PHASE_TRANSITION		15	0.17295241	0.5138618	1	1	1	1549	tags=100%, list=83%, signal=576%
KEGG_PROTEASOME		30	0.15541016	0.5125207	1	1	1	714	tags=43%, list=38%, signal=69%
GOBP_ENSHEATHMENT_OF_NEURONS		18	0.17130817	0.5122538	1	1	1	134	tags=11%, list=7%, signal=12%
GOBP_AMINOGLYCAN_CATABOLIC_PROCESS		12	0.1990317	0.5111288	0.93877554	1	1	1500	tags=100%, list=80%, signal=501%
GOBP_ISOPRENOID_METABOLIC_PROCESS		19	0.15818557	0.50603557	1	1	1	586	tags=37%, list=31%, signal=53%
REACTOME_GLYCOSAMINOGLYCAN_METABOLISM		12	0.1990318	0.5006174	1	1	1	1500	tags=100%, list=80%, signal=501%
REACTOME_DISEASES_ASSOCIATED_WITH_GLYCOSAMINOGLYCAN_METABO		10	0.19881782	0.4905914	0.9811321	1	1	1500	tags=100%, list=80%, signal=502%
REACTOME_ABC_TRANSPORTER_DISORDERS		37	0.13297364	0.4797771	1	1	1	683	tags=43%, list=37%, signal=67%
GOBP_CELL_CYCLE_PHASE_TRANSITION		72	0.10679995	0.47481927	1	1	1	717	tags=40%, list=38%, signal=63%
GOBP_INNATE_IMMUNE_RESPONSE_ACTIVATING_SIGNAL_TRANSDUCTION		39	0.12694296	0.4658068	1	1	1	425	tags=18%, list=23%, signal=23%
REACTOME_NEDDYLATION		44	0.12251449	0.46517733	1	1	1	796	tags=50%, list=43%, signal=85%
REACTOME_PTEN_REGULATION		39	0.11743727	0.45858896	1	1	1	683	tags=38%, list=37%, signal=59%
REACTOME_MITOTIC_G1_PHASE_AND_G1_S_TRANSITION		36	0.1359521	0.4535028	1	1	1	714	tags=44%, list=38%, signal=70%
GOBP_T_CELL_RECEPTOR_SIGNALING_PATHWAY		43	0.1179932	0.4489957	1	1	1	714	tags=40%, list=38%, signal=62%
GOBP_EPHRIN_RECEPTOR_SIGNALING_PATHWAY		22	0.14041772	0.4457967	1	1	1	669	tags=41%, list=36%, signal=63%
GOBP_NEGATIVE_REGULATION_OF_CELL_CYCLE_G2_M_PHASE_TRANSITIO		33	0.12863417	0.44313645	1	1	1	714	tags=42%, list=38%, signal=67%
GOBP_EAR_DEVELOPMENT		13	0.15093875	0.4094389	1	1	1	863	tags=54%, list=46%, signal=99%
GOBP_NEGATIVE_REGULATION_OF_CELL_CYCLE_PHASE_TRANSITION		38	0.10023647	0.37683752	1	1	1	185	tags=8%, list=10%, signal=9%
REACTOME_HCMV_INFECTION		15	0.12382998	0.3593253	1	0.9999695	1	701	tags=40%, list=37%, signal=63%

Supplementary Table 7c. Gene-sets enriched in the 24-month-old heart samples

GS<br>follow link to MSigDB	GS DETAILS	SIZE	ES	NES	NOM p-val	FDR q-val	FWER p-val	RANK AT MAX	LEADING EDGE
GOBP_MIDBRAIN_DEVELOPMENT	Details ...	19	-0.6381298	-2.0175836	0	0.17532253	0.21	346	tags=63%, list=18%, signal=77%
REACTOME_BRANCHED_CHAIN_AMINO_ACID_CATABOLISM	Details ...	18	-0.6377184	-2.0050607	0	0.1141233	0.26	296	tags=61%, list=16%, signal=72%
GOBP_GLYCOLYTIC_PROCESS_THROUGH_FRUCTOSE_6_PHOSI	Details ...	15	-0.6632592	-1.981748	0	0.10673431	0.33	223	tags=60%, list=12%, signal=68%
REACTOME_GLYCOLYSIS	Details ...	18	-0.6452442	-1.9541165	0	0.10698417	0.42	223	tags=56%, list=12%, signal=62%
GOBP_SUBSTANTIA_NIGRA_DEVELOPMENT	Details ...	17	-0.6477265	-1.8616593	0	0.26366565	0.83	281	tags=59%, list=15%, signal=69%
GOBP_BRANCHED_CHAIN_AMINO_ACID_METABOLIC_PROCESS	Details ...	18	-0.6488011	-1.8562979	0	0.2300649	0.87	44	tags=39%, list=2%, signal=39%
GOBP_NEURAL_NUCLEUS_DEVELOPMENT	Details ...	17	-0.6477265	-1.8397883	0	0.2506233	0.92	281	tags=59%, list=15%, signal=69%
REACTOME_GLUCOSE_METABOLISM	Details ...	29	-0.574389	-1.8390974	0	0.21929535	0.92	308	tags=52%, list=16%, signal=61%
REACTOME_GLUCONEOGENESIS	Details ...	20	-0.5642526	-1.785044	0.01666667	0.33290517	0.99	308	tags=55%, list=16%, signal=65%
GOBP_MONOSACCHARIDE_CATABOLIC_PROCESS	Details ...	19	-0.582432	-1.7633883	0	0.37906873	1	306	tags=53%, list=16%, signal=62%
GOBP_NAD_METABOLIC_PROCESS	Details ...	25	-0.5440847	-1.7274474	0	0.48885822	1	392	tags=56%, list=21%, signal=70%
GOBP_GLUCOSE_CATABOLIC_PROCESS	Details ...	16	-0.6298874	-1.7245817	0	0.4596317	1	306	tags=63%, list=16%, signal=74%
GOBP_NADH_METABOLIC_PROCESS	Details ...	22	-0.5906485	-1.7201991	0	0.43907845	1	223	tags=50%, list=12%, signal=56%
GOBP_FAT_CELL_DIFFERENTIATION	Details ...	19	-0.5383534	-1.6406734	0.04081633	0.81324154	1	318	tags=47%, list=17%, signal=56%
GOBP_RIBONUCLEOTIDE_CATABOLIC_PROCESS	Details ...	11	-0.6221769	-1.6184815	0.03703704	0.9079257	1	356	tags=45%, list=19%, signal=56%
GOBP_CYTOCHROME_COMPLEX_ASSEMBLY	Details ...	21	-0.5137969	-1.6152303	0.04081633	0.87786084	1	413	tags=43%, list=22%, signal=54%
GOBP_POSITIVE_REGULATION_OF_TYPE_I_INTERFERON_PROD	Details ...	10	-0.6053417	-1.6147916	0.03571429	0.82801825	1	448	tags=60%, list=24%, signal=78%
GOBP_MONOSACCHARIDE_BIOSYNTHETIC_PROCESS	Details ...	30	-0.4533139	-1.6115313	0.01886793	0.7977466	1	318	tags=40%, list=17%, signal=47%
GOBP_MONOSACCHARIDE_METABOLIC_PROCESS	Details ...	58	-0.4019314	-1.5895991	0	0.89585567	1	318	tags=34%, list=17%, signal=40%
GOBP_SMALL_MOLECULE_CATABOLIC_PROCESS	Details ...	141	-0.3474543	-1.5842955	0	0.88553226	1	398	tags=39%, list=21%, signal=46%
REACTOME_METABOLISM_OF_WATER_SOLUBLE_VITAMINS_ANI	Details ...	29	-0.4765216	-1.5838331	0	0.84592724	1	165	tags=31%, list=9%, signal=34%
KEGG_VIBRIO_CHOLERAE_INFECTION	Details ...	16	-0.5578188	-1.5703806	0.01851852	0.8866732	1	15	tags=13%, list=1%, signal=12%
KEGG_AMINOACYL_TRNA_BIOSYNTHESIS	Details ...	21	-0.4862461	-1.5670128	0.04444445	0.86905116	1	694	tags=57%, list=37%, signal=90%
GOBP_PURINE_CONTAINING_COMPOUND_CATABOLIC_PROCES	Details ...	16	-0.5491474	-1.5530432	0.04166667	0.9226755	1	356	tags=38%, list=19%, signal=46%
GOBP_PHAGOSOME_MATURATION	Details ...	10	-0.5639513	-1.5379791	0.06	0.9871695	1	96	tags=20%, list=5%, signal=21%
KEGG_VALINE_LEUCINE_AND_ISOLEUCINE_DEGRADATION	Details ...	33	-0.4426412	-1.5371629	0.05882353	0.95300347	1	296	tags=45%, list=16%, signal=53%
GOBP_REGULATION_OF_PROTEIN_TARGETING	Details ...	16	-0.540116	-1.5360535	0.0212766	0.924525	1	496	tags=50%, list=27%, signal=67%
REACTOME_RHO_GTPASES_ACTIVATE_PKNS	Details ...	14	-0.5844949	-1.5290233	0.06557377	0.94021845	1	183	tags=50%, list=10%, signal=55%
GOBP_PH_REDUCTION	Details ...	10	-0.5809586	-1.5289676	0.02083333	0.9077971	1	96	tags=20%, list=5%, signal=21%
GOBP_NUCLEOSIDE_DIPHOSPHATE_METABOLIC_PROCESS	Details ...	32	-0.426252	-1.5163828	0.07272727	0.95396495	1	223	tags=31%, list=12%, signal=35%
REACTOME_METABOLISM_OF_CARBOHYDRATES	Details ...	59	-0.3745205	-1.510771	0	0.96317226	1	174	tags=22%, list=9%, signal=24%
GOBP_ORGANIC_ACID_CATABOLIC_PROCESS	Details ...	98	-0.3325064	-1.4928972	0	1	1	345	tags=37%, list=18%, signal=43%
KEGG_NATURAL_KILLER_CELL_MEDIATED_CYTOTOXICITY	Details ...	10	-0.5662302	-1.4873443	0.14893617	1	1	420	tags=30%, list=22%, signal=38%
GOBP_GLUCOSE_METABOLIC_PROCESS	Details ...	51	-0.3911829	-1.4864713	0.03921569	1	1	318	tags=35%, list=17%, signal=41%
GOBP_ATP_METABOLIC_PROCESS	Details ...	137	-0.3309245	-1.486299	0	1	1	322	tags=35%, list=17%, signal=39%
KEGG_GALACTOSE_METABOLISM	Details ...	10	-0.6016882	-1.4800131	0.08510638	1	1	390	tags=50%, list=21%, signal=63%
GOBP_REGULATION_OF_ESTABLISHMENT_OF_PROTEIN_LOCAL	Details ...	13	-0.5286171	-1.4766209	0.05084746	1	1	155	tags=38%, list=8%, signal=42%
REACTOME_METABOLISM_OF_VITAMINS_AND_COFACTORS	Details ...	48	-0.3890511	-1.4764636	0.04477612	0.99379843	1	222	tags=29%, list=12%, signal=32%
GOBP_SENSORY_PERCEPTION_OF_MECHANICAL_STIMULUS	Details ...	18	-0.5187973	-1.4728707	0.07692308	0.9926983	1	195	tags=33%, list=10%, signal=37%
GOBP_CELLULAR_AMINO_ACID_CATABOLIC_PROCESS	Details ...	32	-0.4143483	-1.4649662	0.05769231	1	1	320	tags=41%, list=17%, signal=48%
GOBP_WATER_SOLUBLE_VITAMIN_METABOLIC_PROCESS	Details ...	20	-0.4764658	-1.4623669	0.07407408	1	1	165	tags=30%, list=9%, signal=33%
REACTOME_MITOTIC_PROMETAPHASE	Details ...	23	-0.44201	-1.4545739	0.125	1	1	337	tags=30%, list=18%, signal=37%
KEGG_VASOPRESSIN_REGULATED_WATER_REABSORPTION	Details ...	15	-0.4951281	-1.4536126	0.08888889	1	1	563	tags=60%, list=30%, signal=85%
GOBP_POSITIVE_REGULATION_OF_AXON_EXTENSION	Details ...	12	-0.5296054	-1.4494306	0.08	1	1	692	tags=58%, list=37%, signal=92%
GOBP_REGULATION_OF_MITOCHONDRIAL_OUTER_MEMBRANE	Details ...	12	-0.5837694	-1.4490714	0.08474577	1	1	211	tags=50%, list=11%, signal=56%
GOBP_GENERATION_OF_PRECURSOR_METABOLITES_AND_ENE	Details ...	203	-0.3034529	-1.4469807	0	1	1	356	tags=35%, list=19%, signal=39%
GOBP_POSITIVE_REGULATION_OF_VIRAL_PROCESS	Details ...	14	-0.5073184	-1.4430112	0.06	1	1	208	tags=29%, list=11%, signal=32%
GOBP_REGULATION_OF_MEMBRANE_REPOLARIZATION	Details ...	11	-0.5385385	-1.4397128	0.08163265	1	1	203	tags=45%, list=11%, signal=51%
GOBP_NEGATIVE_REGULATION_OF_CALCIUM_ION_TRANSPORT	Details ...	11	-0.5403338	-1.4394659	0.05882353	0.9833501	1	121	tags=36%, list=6%, signal=39%
GOBP_MAINTENANCE_OF_PROTEIN_LOCALIZATION_IN_ORGANE	Details ...	10	-0.5494078	-1.4324584	0.125	1	1	330	tags=50%, list=18%, signal=60%
GOBP_NUCLEOTIDE_PHOSPHORYLATION		25	-0.4662123	-1.4288664	0.05357143	1	1	223	tags=36%, list=12%, signal=40%
REACTOME_CILIUM_ASSEMBLY		25	-0.4229789	-1.4287306	0.11764706	0.9933461	1	193	tags=28%, list=10%, signal=31%
GOBP_CARBOHYDRATE_CATABOLIC_PROCESS		37	-0.4126356	-1.4283237	0.04761905	0.97806	1	235	tags=30%, list=13%, signal=33%
GOBP_PROTEIN_LOCALIZATION_TO_MITOCHONDRION		59	-0.3618234	-1.4227961	0.03571429	0.98985237	1	264	tags=27%, list=14%, signal=31%

REACTOME_DEATH_RECEPTOR_SIGNALLING		12	-0.5495479	-1.4181945	0.10344828	0.99925536	1	179	tags=33%, list=10%, signal=37%
GOBP_MITOCHONDRIAL_OUTER_MEMBRANE_PERMEABILIZATION		16	-0.4802765	-1.4158071	0.075	0.9970956	1	211	tags=38%, list=11%, signal=42%
REACTOME_CA2_PATHWAY		10	-0.590473	-1.4149731	0.06779661	0.9842883	1	173	tags=50%, list=9%, signal=55%
GOBP_TRNA_METABOLIC_PROCESS		32	-0.4124544	-1.4129173	0.07142858	0.97893816	1	553	tags=47%, list=30%, signal=65%
GOBP_MACROMOLECULE_METHYLATION		12	-0.5304154	-1.409394	0.13207547	0.9839128	1	56	tags=17%, list=3%, signal=17%
GOBP_APOPTOTIC_MITOCHONDRIAL_CHANGES		36	-0.3752873	-1.4076087	0.06	0.97718865	1	249	tags=33%, list=13%, signal=38%
GOBP_POSITIVE_REGULATION_OF_NERVOUS_SYSTEM_DEVELOPMENT		36	-0.3818969	-1.4068446	0.01666667	0.96582365	1	647	tags=47%, list=35%, signal=71%
GOBP_SENSORY_PERCEPTION		37	-0.3708561	-1.4034075	0.05555556	0.97135293	1	249	tags=24%, list=13%, signal=28%
KEGG_ABC_TRANSPORTERS		12	-0.5277245	-1.4005655	0.11111111	0.9713805	1	383	tags=33%, list=20%, signal=42%
GOBP_CELLULAR_AMINO_ACID_METABOLIC_PROCESS		107	-0.3195391	-1.4000053	0	0.95906866	1	320	tags=23%, list=17%, signal=27%
REACTOME_TRANSCRIPTIONAL_REGULATION_BY_TP53		39	-0.3970467	-1.3996211	0.03508772	0.9459447	1	273	tags=33%, list=15%, signal=38%
REACTOME_INTRINSIC_PATHWAY_FOR_APOPTOSIS		12	-0.5531084	-1.3992987	0.11864407	0.93288356	1	155	tags=42%, list=8%, signal=45%
REACTOME_TRNA_AMINOACYLATION		24	-0.4157675	-1.398001	0.12244898	0.9269472	1	543	tags=38%, list=29%, signal=52%
GOBP_VITAMIN_METABOLIC_PROCESS		22	-0.4478604	-1.3974607	0.05357143	0.91669333	1	165	tags=27%, list=9%, signal=30%
GOBP_POSITIVE_REGULATION_OF_CELL_GROWTH		26	-0.4310446	-1.3966348	0.03448276	0.907847	1	627	tags=50%, list=34%, signal=74%
KEGG_CELL_ADHESION_MOLECULES_CAMS		19	-0.4676336	-1.3944834	0.09259259	0.90724206	1	344	tags=32%, list=18%, signal=38%
REACTOME_TP53_REGULATES_METABOLIC_GENES		32	-0.4099178	-1.3819561	0.07272727	0.9627314	1	273	tags=38%, list=15%, signal=43%
REACTOME_METABOLISM_OF_AMINO_ACIDS_AND_DERIVATIVES		155	-0.3016349	-1.3808366	0.03703704	0.95606345	1	311	tags=25%, list=17%, signal=28%
GOBP_AMINO_ACID_ACTIVATION		25	-0.430644	-1.3766443	0.07317073	0.9683647	1	543	tags=40%, list=29%, signal=56%
GOBP_CHEMOKINE_PRODUCTION		12	-0.4906925	-1.3755631	0.10416666	0.96201235	1	318	tags=33%, list=17%, signal=40%
GOBP_POSITIVE_REGULATION_OF_DEVELOPMENTAL_GROWTH		21	-0.4371163	-1.3709677	0.0877193	0.9764904	1	627	tags=52%, list=34%, signal=78%
BIOCARTA_AGR_PATHWAY		11	-0.5325053	-1.3626604	0.08	1	1	591	tags=73%, list=32%, signal=106%
GOBP_POSITIVE_REGULATION_OF_NEUROGENESIS		33	-0.3949842	-1.3584212	0.15686275	1	1	647	tags=48%, list=35%, signal=73%
GOBP_ORGANOPHOSPHATE_CATABOLIC_PROCESS		37	-0.3556499	-1.3580801	0.09259259	1	1	682	tags=54%, list=36%, signal=83%
GOBP_ORGANIC_ACID_METABOLIC_PROCESS		300	-0.2698515	-1.357655	0	1	1	320	tags=26%, list=17%, signal=26%
GOBP_MESENCHYMAL_CELL_DIFFERENTIATION		22	-0.4481726	-1.3549385	0.06122449	1	1	477	tags=50%, list=25%, signal=66%
GOBP_REGULATION_OF_MUSCLE_CELL_DIFFERENTIATION		13	-0.4892946	-1.3535951	0.07142858	0.99877936	1	80	tags=23%, list=4%, signal=24%
GOBP_TOXIN_TRANSPORT		12	-0.5013836	-1.3518881	0.13953489	0.99471164	1	128	tags=33%, list=7%, signal=36%
GOBP_KIDNEY_EPITHELIUM_DEVELOPMENT		13	-0.4867928	-1.3499078	0.15789473	0.9935328	1	621	tags=62%, list=33%, signal=91%
GOBP_NUCLEOSIDE_PHOSPHATE_CATABOLIC_PROCESS		24	-0.4193912	-1.3472066	0.14893617	0.99810266	1	251	tags=25%, list=13%, signal=29%
GOBP_VIRAL_RELEASE_FROM_HOST_CELL		11	-0.5273174	-1.3360639	0.1632653	1	1	96	tags=18%, list=5%, signal=19%
REACTOME_DISEASES_OF_METABOLISM		39	-0.3641336	-1.3303874	0.04081633	1	1	94	tags=13%, list=5%, signal=13%
KEGG_PATHWAYS_IN_CANCER		37	-0.3782537	-1.3303295	0.08928572	1	1	448	tags=43%, list=24%, signal=56%
GOBP_REGULATION_OF_AXONOGENESIS		23	-0.4425821	-1.3289207	0.18	1	1	647	tags=52%, list=35%, signal=79%
REACTOME_SIGNALING_BY_INSULIN_RECEPTOR		14	-0.4958297	-1.3284298	0.16981132	1	1	15	tags=7%, list=1%, signal=7%
REACTOME_RECRUITMENT_OF_NUMA_TO_MITOTIC_CENTROSOMES		14	-0.456975	-1.322693	0.14516129	1	1	337	tags=36%, list=18%, signal=43%
GOBP_NEGATIVE_REGULATION_OF_RESPONSE_TO_ENDOPLASMIC_RETICUL		12	-0.4616657	-1.3224877	0.10714286	1	1	249	tags=33%, list=13%, signal=38%
GOBP_CHAPERONE_MEDIATED_PROTEIN_COMPLEX_ASSEMBLY		11	-0.4994187	-1.3174454	0.10909091	1	1	212	tags=45%, list=11%, signal=51%
GOBP_CARBOHYDRATE_BIOSYNTHETIC_PROCESS		45	-0.3471095	-1.3148698	0.0483871	1	1	447	tags=38%, list=24%, signal=48%
GOBP_CHROMOSOME_LOCALIZATION		10	-0.5070571	-1.3143772	0.12068965	1	1	653	tags=70%, list=35%, signal=107%
GOBP_SKELETAL_MUSCLE_CONTRACTION		10	-0.5350257	-1.3116683	0.1509434	1	1	75	tags=30%, list=4%, signal=31%
GOBP_POSITIVE_REGULATION_OF_HEMOPOIESIS		10	-0.5626063	-1.3108989	0.16	1	1	4	tags=10%, list=0%, signal=10%
GOBP_REGULATION_OF_MITOCHONDRIAL_MEMBRANE_PERMEABILITY		22	-0.4174325	-1.3096393	0.11538462	1	1	211	tags=32%, list=11%, signal=35%
GOBP_CELLULAR_CARBOHYDRATE_METABOLIC_PROCESS		44	-0.3801981	-1.3094437	0.11764706	1	1	447	tags=43%, list=24%, signal=55%
GOBP_OXIDATIVE_PHOSPHORYLATION		95	-0.294864	-1.3078867	0.07017544	1	1	346	tags=37%, list=18%, signal=43%
GOBP_MITOCHONDRIAL_ELECTRON_TRANSPORT_UBIQUINOL_TO_CYTOCHR		10	-0.4950584	-1.3077964	0.16	1	1	140	tags=40%, list=7%, signal=43%
REACTOME_MTORC1_MEDIATED_SIGNALLING		10	-0.5026417	-1.3072064	0.16666667	1	1	469	tags=50%, list=25%, signal=66%
GOBP_REGULATION_OF_RHO_PROTEIN_SIGNAL_TRANSDUCTION		11	-0.4944806	-1.3058058	0.10169491	1	1	453	tags=55%, list=24%, signal=72%
GOBP_SMALL_MOLECULE_BIOSYNTHETIC_PROCESS		149	-0.2853773	-1.3035527	0.01612903	1	1	318	tags=26%, list=17%, signal=28%
GOBP_RESPIRATORY_GASEOUS_EXCHANGE_BY_RESPIRATORY_SYSTEM		10	-0.5550324	-1.3022003	0.12727273	1	1	459	tags=50%, list=25%, signal=66%
GOBP_POSITIVE_REGULATION_OF_GTPASE_ACTIVITY		39	-0.3483423	-1.290532	0.15384616	1	1	647	tags=54%, list=35%, signal=81%
GOBP_CELLULAR_AMINO_ACID_BIOSYNTHETIC_PROCESS		12	-0.4986064	-1.2873626	0.18367347	1	1	306	tags=42%, list=16%, signal=49%
GOBP_NUCLEUS_ORGANIZATION		13	-0.4669972	-1.2872956	0.18367347	1	1	653	tags=62%, list=35%, signal=94%
REACTOME_TRANSLOCATION_OF_SLC2A4_GLUT4_TO_THE_PLASMA_MEMBR		27	-0.3776756	-1.2870758	0.1147541	1	1	187	tags=26%, list=10%, signal=28%
GOBP_MYOTUBE_DIFFERENTIATION		18	-0.4528122	-1.2865533	0.20754717	1	1	497	tags=61%, list=27%, signal=82%
GOBP_INORGANIC_ANION_TRANSMEMBRANE_TRANSPORT		13	-0.4919606	-1.2834553	0.1875	1	1	464	tags=54%, list=25%, signal=71%
GOBP_POSITIVE_REGULATION_OF_AXONOGENESIS		18	-0.4598105	-1.2752303	0.13461539	1	1	692	tags=61%, list=37%, signal=96%

KEGG_MELANOGENESIS		15	-0.4541147	-1.2726666	0.19642857	1	1	56	tags=20%, list=3%, signal=20%
GOBP_MITOCHONDRIAL_ELECTRON_TRANSPORT_NADH_TO_UBIQUINONE		42	-0.3410975	-1.2719868	0.0877193	1	1	335	tags=43%, list=18%, signal=51%
GOBP_MULTICELLULAR_ORGANISMAL_MOVEMENT		11	-0.5012673	-1.2699312	0.14285715	1	1	75	tags=27%, list=4%, signal=28%
KEGG_SMALL_CELL_LUNG_CANCER		14	-0.4527731	-1.2669127	0.18367347	1	1	344	tags=43%, list=18%, signal=52%
REACTOME_AMINO_ACIDS_REGULATE_MTORC1		11	-0.4674718	-1.2645142	0.18367347	1	1	494	tags=36%, list=26%, signal=49%
REACTOME_MITOCHONDRIAL_TRNA_AMINOACYLATION		15	-0.4429404	-1.2541478	0.18	1	1	651	tags=53%, list=35%, signal=81%
GOBP_EMBRYONIC_MORPHOGENESIS		48	-0.3350049	-1.2541016	0.16666667	1	1	344	tags=31%, list=18%, signal=37%
GOBP_BIOLOGICAL_PROCESS_INVOLVED_IN_INTERACTION_WITH_SYMBIONT		22	-0.414014	-1.2528572	0.1875	1	1	212	tags=32%, list=11%, signal=35%
GOBP_SMALL_MOLECULE_METABOLIC_PROCESS		446	-0.2442215	-1.2509255	0.04109589	1	1	376	tags=26%, list=20%, signal=25%
KEGG_OOCYTE_MEIOSIS		19	-0.4433363	-1.250755	0.23404256	1	1	261	tags=32%, list=14%, signal=36%
GOBP_REGULATION_OF_HEART_CONTRACTION		60	-0.3096803	-1.2465827	0.10869565	1	1	203	tags=25%, list=11%, signal=27%
GOBP_RESPIRATORY_CHAIN_COMPLEX_IV_ASSEMBLY		14	-0.4323466	-1.2464824	0.16393442	1	1	399	tags=36%, list=21%, signal=45%
GOBP_NEPHRON_EPITHELIUM_DEVELOPMENT		11	-0.48568	-1.2459029	0.1764706	1	1	621	tags=64%, list=33%, signal=95%
GOBP_MONOCARBOXYLIC_ACID_METABOLIC_PROCESS		179	-0.2648238	-1.2457	0.08333334	1	1	320	tags=28%, list=17%, signal=31%
REACTOME_PEPTIDE_HORMONE_METABOLISM		13	-0.4861456	-1.2432147	0.24074075	1	1	314	tags=38%, list=17%, signal=46%
GOBP_POSITIVE_REGULATION_OF_GROWTH		34	-0.3390885	-1.2424723	0.14814815	1	1	627	tags=44%, list=34%, signal=65%
GOBP_REGULATION_OF_VASOCONSTRICTION		11	-0.4692193	-1.2409871	0.26086956	1	1	330	tags=45%, list=18%, signal=55%
KEGG_NEUROTROPHIN_SIGNALING_PATHWAY		22	-0.3937339	-1.2385491	0.17021276	1	1	179	tags=27%, list=10%, signal=30%
KEGG_GLYCOLYSIS_GLUCONEOGENESIS		29	-0.3805766	-1.2369398	0.2	1	1	296	tags=41%, list=16%, signal=48%
GOBP_NEPHRON_DEVELOPMENT		16	-0.4292712	-1.2362214	0.18644068	1	1	448	tags=38%, list=24%, signal=49%
GOBP_MESENCHYME_DEVELOPMENT		27	-0.3961923	-1.2342504	0.1923077	1	1	477	tags=48%, list=25%, signal=64%
GOBP_CELL_DIVISION		56	-0.3105518	-1.2334443	0.14285715	1	1	360	tags=29%, list=19%, signal=34%
GOBP_CELL_CELL_JUNCTION_ASSEMBLY		27	-0.3446753	-1.233238	0.21428572	1	1	148	tags=26%, list=8%, signal=28%
KEGG_OXIDATIVE_PHOSPHORYLATION		75	-0.3051786	-1.232391	0.15789473	1	1	346	tags=39%, list=18%, signal=46%
REACTOME_P75_NTR_RECEPTOR_MEDIATED_SIGNALLING		11	-0.4930976	-1.2318949	0.26415095	1	1	477	tags=45%, list=25%, signal=61%
GOBP_PROSTANOID_METABOLIC_PROCESS		13	-0.4580949	-1.2316056	0.25490198	1	1	286	tags=38%, list=15%, signal=45%
GOBP_EMBRYO_DEVELOPMENT_ENDING_IN_BIRTH_OR_EGG_HATCHING		37	-0.3377494	-1.2300892	0.16363636	1	1	292	tags=22%, list=16%, signal=25%
GOBP_MITOCHONDRIAL_TRANSMEMBRANE_TRANSPORT		60	-0.3129825	-1.2295753	0.20408164	1	1	389	tags=37%, list=21%, signal=45%
GOBP_LIPID_HOMEOSTASIS		27	-0.3870169	-1.2287093	0.18181819	1	1	234	tags=26%, list=13%, signal=29%
GOBP_INTERLEUKIN_1_PRODUCTION		11	-0.4893754	-1.2286831	0.31578946	1	1	236	tags=45%, list=13%, signal=52%
GOBP_CYTOSKELETON_DEPENDENT_CYTOKINESIS		20	-0.4026955	-1.2284853	0.25	1	1	216	tags=25%, list=12%, signal=28%
GOBP_MEMBRANE_REPOLARIZATION		15	-0.4268408	-1.227996	0.16981132	1	1	203	tags=40%, list=11%, signal=45%
GOBP_ATP_BIOSYNTHETIC_PROCESS		26	-0.3983813	-1.2277795	0.26923078	1	1	315	tags=42%, list=17%, signal=50%
KEGG_VIRAL_MYOCARDITIS		18	-0.405273	-1.2269142	0.2	1	1	246	tags=33%, list=13%, signal=38%
GOBP_CELLULAR_RESPONSE_TO_INSULIN_STIMULUS		43	-0.3424276	-1.2256181	0.14	1	1	249	tags=21%, list=13%, signal=24%
GOBP_DETECTION_OF_STIMULUS		25	-0.3888657	-1.2234105	0.1754386	1	1	538	tags=48%, list=29%, signal=66%
GOBP_MITOCHONDRIAL_TRANSPORT		116	-0.2724378	-1.2219741	0.10714286	1	1	324	tags=27%, list=17%, signal=30%
GOBP_PYRUVATE_METABOLIC_PROCESS		43	-0.3340271	-1.2210616	0.16666667	1	1	174	tags=26%, list=9%, signal=28%
GOBP_ELECTRON_TRANSPORT_CHAIN		94	-0.2960636	-1.221058	0.11864407	1	1	356	tags=38%, list=19%, signal=45%
GOBP_EMBRYONIC_ORGAN_MORPHOGENESIS		15	-0.4429605	-1.218521	0.2264151	1	1	56	tags=13%, list=3%, signal=14%
GOBP_REGULATION_OF_BLOOD_CIRCULATION		66	-0.3099008	-1.2171732	0.1764706	1	1	203	tags=24%, list=11%, signal=26%
GOBP_REGULATION_OF_SYSTEM_PROCESS		101	-0.2842253	-1.2150007	0.15	1	1	330	tags=28%, list=18%, signal=32%
KEGG_DILATED_CARDIOMYOPATHY		39	-0.3215745	-1.2131758	0.18181819	1	1	274	tags=33%, list=15%, signal=38%
GOBP_INTRACELLULAR_LIPID_TRANSPORT		12	-0.4737791	-1.2116779	0.28301886	1	1	283	tags=42%, list=15%, signal=49%
GOBP_ALPHA_AMINO_ACID_METABOLIC_PROCESS		40	-0.3121899	-1.211044	0.2	1	1	320	tags=33%, list=17%, signal=38%
GOBP_RESPONSE_TO_ANTIBIOTIC		13	-0.4518957	-1.2090358	0.23529412	1	1	229	tags=31%, list=12%, signal=35%
GOBP_ACTIVATION_OF_CYSTEINE_TYPE_ENDOPEPTIDASE_ACTIVITY_INVOL		15	-0.3912506	-1.2066274	0.25	1	1	212	tags=33%, list=11%, signal=37%
GOBP_DNA_METABOLIC_PROCESS		62	-0.2960137	-1.2063177	0.13793103	1	1	366	tags=26%, list=20%, signal=31%
GOBP_REGULATION_OF_LIPID_CATABOLIC_PROCESS		10	-0.4683206	-1.2059658	0.23636363	1	1	447	tags=40%, list=24%, signal=52%
GOBP_POSITIVE_REGULATION_OF_CYTOKINE_PRODUCTION		38	-0.3427986	-1.2027538	0.20930232	1	1	328	tags=32%, list=18%, signal=38%
GOBP_MUSCLE_ORGAN_DEVELOPMENT		61	-0.2905335	-1.2020888	0.1923077	1	1	329	tags=31%, list=18%, signal=37%
REACTOME_PLATELET_HOMEOSTASIS		20	-0.3921146	-1.2012395	0.19672132	1	1	173	tags=30%, list=9%, signal=33%
KEGG_PYRUVATE_METABOLISM		23	-0.3691105	-1.1995869	0.1724138	1	1	349	tags=43%, list=19%, signal=53%
GOBP_CILIUM_ORGANIZATION		31	-0.3471451	-1.1995647	0.18367347	1	1	337	tags=35%, list=18%, signal=43%
GOBP_EMBRYONIC_ORGAN_DEVELOPMENT		21	-0.4111503	-1.1995459	0.26086956	1	1	274	tags=19%, list=15%, signal=22%
GOBP_POLYOL_METABOLIC_PROCESS		20	-0.3943502	-1.1980159	0.25581396	1	1	235	tags=25%, list=13%, signal=28%
GOBP_EPITHELIAL_TUBE_MORPHOGENESIS		27	-0.3645204	-1.1979531	0.18644068	1	1	329	tags=30%, list=18%, signal=35%

REACTOME_ANCHORING_OF_THE_BASAL_BODY_TO_THE_PLASMA_MEMBRA	13	-0.4431489	-1.197494	0.1875	1	1	337	tags=38%, list=18%, signal=47%
GOBP_POSITIVE_REGULATION_OF_TRANSCRIPTION_BY_RNA_POLYMERASE	49	-0.3311189	-1.1969099	0.18965517	1	1	545	tags=37%, list=29%, signal=50%
REACTOME_TOLL_LIKE_RECEPTOR_9_TLR9_CASCADE	11	-0.4223371	-1.1952063	0.2173913	1	1	169	tags=18%, list=9%, signal=20%
REACTOME_INFECTION_WITH_MYCOBACTERIUM_TUBERCULOSIS	11	-0.4470215	-1.1949855	0.24074075	1	1	451	tags=45%, list=24%, signal=60%
GOBP_REGULATION_OF_PROTEIN_MODIFICATION_BY_SMALL_PROTEIN_CON	25	-0.3882425	-1.1943206	0.21818182	1	1	229	tags=32%, list=12%, signal=36%
GOBP_ORGANIC_HYDROXY_COMPOUND_CATABOLIC_PROCESS	12	-0.4576524	-1.1938783	0.34146342	1	1	392	tags=50%, list=21%, signal=63%
GOBP_CELLULAR_CARBOHYDRATE_CATABOLIC_PROCESS	14	-0.420751	-1.1917064	0.22	1	1	392	tags=43%, list=21%, signal=54%
GOBP_REGULATION_OF_CELLULAR_RESPONSE_TO_INSULIN_STIMULUS	14	-0.4174736	-1.1904905	0.2	1	1	249	tags=29%, list=13%, signal=33%
GOBP_CARDIAC_VENTRICLE_DEVELOPMENT	17	-0.3732861	-1.1867015	0.2244898	1	1	105	tags=29%, list=6%, signal=31%
GOBP_MITOCHONDRIAL_RESPIRATORY_CHAIN_COMPLEX_ASSEMBLY	69	-0.2783409	-1.1867014	0.14814815	1	1	335	tags=33%, list=18%, signal=39%
KEGG_TIGHT_JUNCTION	31	-0.3300384	-1.1866182	0.19148937	1	1	148	tags=23%, list=8%, signal=24%
REACTOME_CELL_JUNCTION_ORGANIZATION	18	-0.3820871	-1.1861378	0.20930232	1	1	182	tags=28%, list=10%, signal=30%
GOBP_RESPONSE_TO_MECHANICAL_STIMULUS	29	-0.3329246	-1.1853758	0.25	1	1	330	tags=34%, list=18%, signal=41%
GOBP_ORGANIC_ACID_BIOSYNTHETIC_PROCESS	68	-0.3021468	-1.1828183	0.21428572	1	1	318	tags=31%, list=17%, signal=36%
GOBP_REGULATION_OF_TRANSLATIONAL_INITIATION	13	-0.4112168	-1.1799247	0.28301886	1	1	514	tags=46%, list=27%, signal=63%
GOBP_MITOCHONDRIAL_CYTOCHROME_C_OXIDASE_ASSEMBLY	12	-0.4340246	-1.1767348	0.2	1	1	361	tags=33%, list=19%, signal=41%
GOBP_REGULATION_OF_NEUROGENESIS	40	-0.3347814	-1.1751635	0.20833333	1	1	647	tags=45%, list=35%, signal=67%
GOBP_CELLULAR_RESPIRATION	115	-0.272394	-1.1744633	0.12068965	1	1	349	tags=36%, list=19%, signal=41%
GOBP_FATTY_ACID_BIOSYNTHETIC_PROCESS	41	-0.3241412	-1.1738816	0.26	1	1	318	tags=37%, list=17%, signal=43%
GOBP_ENDOPLASMIC_RETICULUM_ORGANIZATION	24	-0.3530104	-1.1726557	0.24528302	1	1	724	tags=63%, list=39%, signal=101%
GOBP_REGULATION_OF_NERVOUS_SYSTEM_DEVELOPMENT	46	-0.3225311	-1.1701629	0.23728813	1	1	647	tags=43%, list=35%, signal=65%
REACTOME_GLUCAGON_SIGNALING_IN_METABOLIC_REGULATION	12	-0.4550023	-1.1697599	0.28070176	1	1	173	tags=33%, list=9%, signal=36%
REACTOME_NON_INTEGRIN_MEMBRANE_ECM_INTERACTIONS	18	-0.3800024	-1.1690166	0.27659574	1	1	614	tags=61%, list=33%, signal=90%
GOBP_RIBONUCLEOSIDE_TRIPHOSPHATE_BIOSYNTHETIC_PROCESS	28	-0.3634	-1.1682906	0.23636363	1	1	315	tags=39%, list=17%, signal=47%
REACTOME_RESPIRATORY_ELECTRON_TRANSPORT_ATP_SYNTHESIS_BY_C	90	-0.2908659	-1.166425	0.15517241	1	1	346	tags=39%, list=18%, signal=45%
GOBP_REGULATION_OF_HORMONE_SECRETION	33	-0.3160886	-1.163702	0.2037037	1	1	377	tags=36%, list=20%, signal=45%
GOBP_INSULIN_RECEPTOR_SIGNALING_PATHWAY	25	-0.3274064	-1.1632907	0.13043478	1	1	15	tags=8%, list=1%, signal=8%
GOBP_ENERGY_DERIVATION_BY_OXIDATION_OF_ORGANIC_COMPOUNDS	133	-0.2609119	-1.163029	0.1923077	1	1	349	tags=33%, list=19%, signal=38%
GOBP_ESTABLISHMENT_OF_PROTEIN_LOCALIZATION_TO_PLASMA_MEMBRA	10	-0.4523548	-1.1620711	0.2881356	1	1	197	tags=30%, list=11%, signal=33%
GOBP_ICOSANOID_METABOLIC_PROCESS	23	-0.3560318	-1.1607636	0.23214285	1	1	286	tags=30%, list=15%, signal=35%
GOBP_SKIN_DEVELOPMENT	21	-0.3575664	-1.160201	0.2542373	1	1	19	tags=10%, list=1%, signal=10%
GOBP_POSITIVE_REGULATION_OF_CYSTEINE_TYPE_ENDOPEPTIDASE_ACTI	27	-0.3116061	-1.1566513	0.18604651	1	1	212	tags=26%, list=11%, signal=29%
GOBP_POSITIVE_REGULATION_OF_PROTEIN_MODIFICATION_BY_SMALL_PRO	10	-0.4545828	-1.1561655	0.25	1	1	211	tags=40%, list=11%, signal=45%
REACTOME_AURKA_ACTIVATION_BY_TPX2	11	-0.4198886	-1.1553806	0.26666668	1	1	337	tags=36%, list=18%, signal=44%
GOBP_PURINE_CONTAINING_COMPOUND_METABOLIC_PROCESS	129	-0.2618381	-1.1551101	0.19672132	1	1	315	tags=26%, list=17%, signal=29%
REACTOME_ASSEMBLY_OF_COLLAGEN_FIBRILS_AND_OTHER_MULTIMERIC_	10	-0.4783149	-1.1535908	0.31707317	1	1	142	tags=20%, list=8%, signal=22%
GOBP_LEUKOCYTE_DIFFERENTIATION	37	-0.3031496	-1.1526619	0.2777778	1	1	469	tags=30%, list=25%, signal=39%
REACTOME_RHOH_GTPASE_CYCLE	17	-0.3963159	-1.151258	0.26086956	1	1	419	tags=47%, list=22%, signal=60%
GOBP_MITOCHONDRION_ORGANIZATION	221	-0.2370883	-1.1508904	0.16666667	1	1	324	tags=27%, list=17%, signal=28%
GOBP_PROTEIN_LIPID_COMPLEX_SUBUNIT_ORGANIZATION	11	-0.4463318	-1.1494095	0.24489796	1	1	222	tags=36%, list=12%, signal=41%
GOBP_POSITIVE_REGULATION_OF_MEMBRANE_PERMEABILITY	19	-0.3884794	-1.1453178	0.33333334	1	1	211	tags=32%, list=11%, signal=35%
GOBP_CYTOKINESIS	24	-0.3396404	-1.145002	0.2777778	1	1	216	tags=21%, list=12%, signal=23%
GOBP_CARBOHYDRATE_METABOLIC_PROCESS	104	-0.2671191	-1.144801	0.21568628	1	1	318	tags=25%, list=17%, signal=28%
GOBP_DICARBOXYLIC_ACID_METABOLIC_PROCESS	34	-0.3175145	-1.1434194	0.27118644	1	1	349	tags=44%, list=19%, signal=53%
GOBP_PEPTIDYL_LYSINE_MODIFICATION	16	-0.3967102	-1.1422347	0.24528302	1	1	211	tags=31%, list=11%, signal=35%
GOBP_REGULATION_OF_CELL_DIVISION	10	-0.4394135	-1.1414843	0.33333334	1	1	653	tags=60%, list=35%, signal=92%
KEGG_LONG_TERM_DEPRESSION	14	-0.4275273	-1.1408174	0.24528302	1	1	146	tags=21%, list=8%, signal=23%
GOBP_BONE_DEVELOPMENT	21	-0.3683504	-1.1371375	0.30357143	1	1	330	tags=33%, list=18%, signal=40%
GOBP_NUCLEAR_CHROMOSOME_SEGREGATION	16	-0.3968668	-1.1370635	0.29090908	1	1	591	tags=56%, list=32%, signal=82%
KEGG_WNT_SIGNALING_PATHWAY	18	-0.3546719	-1.1358994	0.2857143	1	1	148	tags=17%, list=8%, signal=18%
GOBP_PHOSPHOLIPID_TRANSPORT	11	-0.4142782	-1.1346802	0.34	1	1	198	tags=27%, list=11%, signal=30%
REACTOME_MTOR_SIGNALLING	13	-0.4372938	-1.1337186	0.29411766	1	1	505	tags=46%, list=27%, signal=63%
REACTOME_RESPIRATORY_ELECTRON_TRANSPORT	76	-0.2748209	-1.1334403	0.22807017	1	1	361	tags=38%, list=19%, signal=45%
GOBP_RESPONSE_TO_CORTICOSTEROID	25	-0.3444802	-1.1331732	0.29310346	1	1	318	tags=28%, list=17%, signal=33%
KEGG_PARKINSONS_DISEASE	70	-0.2808919	-1.133135	0.21311475	1	1	346	tags=39%, list=18%, signal=46%
GOBP_PROTEIN_TRANSMEMBRANE_IMPORT_INTO_INTRACELLULAR_ORGANE	16	-0.3989559	-1.1331292	0.2777778	1	1	264	tags=31%, list=14%, signal=36%
GOBP_NUCLEOBASE_CONTAINING_COMPOUND_TRANSPORT	24	-0.346934	-1.1325307	0.27586207	1	1	517	tags=46%, list=28%, signal=63%

GOBP_PROTEIN_IMPORT_INTO_MITOCHONDRIAL_MATRIX		12	-0.4190985	-1.1288041	0.23913044	1	1	264	tags=33%, list=14%, signal=39%
REACTOME_REGULATION_OF_HSF1_MEDIATED_HEAT_SHOCK_RESPONSE		16	-0.38977	-1.127387	0.33333334	1	1	40	tags=19%, list=2%, signal=19%
GOBP_PEPTIDYL_ASPARAGINE_MODIFICATION		11	-0.4493211	-1.1271209	0.30612245	1	1	298	tags=27%, list=16%, signal=32%
GOBP_INOSITOL_PHOSPHATE_MEDIATED_SIGNALING		10	-0.4404875	-1.1268569	0.29166666	1	1	482	tags=40%, list=26%, signal=54%
GOBP_NUCLEOSIDE_TRIPHOSPHATE_BIOSYNTHETIC_PROCESS		30	-0.3319264	-1.126434	0.27272728	1	1	315	tags=37%, list=17%, signal=43%
KEGG_VASCULAR_SMOOTH_MUSCLE_CONTRACTION		22	-0.3520494	-1.1257126	0.29310346	1	1	261	tags=18%, list=14%, signal=21%
GOBP_ATP_SYNTHESIS_COUPLED_ELECTRON_TRANSPORT		69	-0.2816469	-1.1245284	0.3	1	1	346	tags=36%, list=18%, signal=43%
GOBP_CILIARY_BASAL_BODY_PLASMA_MEMBRANE_DOCKING		11	-0.4198885	-1.1229696	0.31914893	1	1	337	tags=36%, list=18%, signal=44%
REACTOME_VASOPRESSIN_REGULATES_RENAL_WATER_HOMEOSTASIS_VIA		13	-0.4229307	-1.12286	0.35849056	1	1	173	tags=31%, list=9%, signal=34%
GOBP_REGULATION_OF_CYSTEINE_TYPE_ENDOPEPTIDASE_ACTIVITY		40	-0.3057565	-1.1228092	0.24489796	1	1	212	tags=25%, list=11%, signal=28%
REACTOME_CARGO_CONCENTRATION_IN_THE_ER		15	-0.3885195	-1.1208577	0.3	1	1	207	tags=20%, list=11%, signal=22%
REACTOME_ANTIGEN_PRESENTATION_FOLDING_ASSEMBLY_AND_PEPTIDE_		13	-0.4052983	-1.1203448	0.3181818	1	1	97	tags=15%, list=5%, signal=16%
GOBP_POSITIVE_REGULATION_OF_CELL_DEVELOPMENT		58	-0.2844527	-1.1186215	0.28333333	1	1	603	tags=43%, list=32%, signal=62%
GOBP_RESPIRATORY_ELECTRON_TRANSPORT_CHAIN		78	-0.2573364	-1.1174973	0.27586207	1	1	346	tags=35%, list=18%, signal=41%
REACTOME_REGULATED_NECROSIS		11	-0.4273163	-1.1165882	0.34920636	1	1	328	tags=45%, list=18%, signal=55%
GOBP_LEUKOCYTE_CELL_CELL_ADHESION		48	-0.2880443	-1.1165689	0.1904762	1	1	260	tags=21%, list=14%, signal=24%
GOBP_NUCLEOBASE_CONTAINING_SMALL_MOLECULE_METABOLIC_PROCES		154	-0.2481901	-1.1160846	0.2264151	1	1	364	tags=27%, list=19%, signal=30%
REACTOME_RHO_GTPASE_EFFECTORS		58	-0.2879995	-1.1157292	0.24	1	1	261	tags=21%, list=14%, signal=23%
GOBP_REGULATION_OF_PEPTIDASE_ACTIVITY		73	-0.2642305	-1.1153927	0.30769232	1	1	212	tags=21%, list=11%, signal=22%
GOBP_CARTILAGE_DEVELOPMENT		10	-0.4055784	-1.1145766	0.31111112	1	1	4	tags=10%, list=0%, signal=10%
GOBP_NUCLEOBASE_CONTAINING_SMALL_MOLECULE_BIOSYNTHETIC_PROC		29	-0.3283231	-1.113444	0.23404256	1	1	251	tags=21%, list=13%, signal=24%
GOBP_METHYLATION		18	-0.3785969	-1.1132563	0.30769232	1	1	56	tags=11%, list=3%, signal=11%
REACTOME_GPCR_LIGAND_BINDING		18	-0.3860435	-1.1130446	0.26785713	1	1	257	tags=28%, list=14%, signal=32%
REACTOME_SEMA4D_IN_SEMAPHORIN_SIGNALING		11	-0.4604532	-1.1093453	0.25	1	1	420	tags=45%, list=22%, signal=58%
GOBP_GLUTAMINE_FAMILY_AMINO_ACID_METABOLIC_PROCESS		15	-0.3842549	-1.1070973	0.3090909	1	1	320	tags=47%, list=17%, signal=56%
GOBP_RESPONSE_TO_COLD		11	-0.4321785	-1.1070518	0.3888889	1	1	224	tags=45%, list=12%, signal=51%
GOBP_MUSCLE_CELL_DIFFERENTIATION		62	-0.2775037	-1.1070273	0.25	1	1	279	tags=27%, list=15%, signal=31%
REACTOME_G_ALPHA_12_13_SIGNALLING_EVENTS		18	-0.364463	-1.1060253	0.31343284	1	1	173	tags=22%, list=9%, signal=24%
REACTOME_ION_CHANNEL_TRANSPORT		24	-0.3502622	-1.105524	0.33333334	1	1	509	tags=46%, list=27%, signal=62%
GOBP_DNA_TEMPLATED_TRANSCRIPTION_INITIATION		12	-0.4288467	-1.1047271	0.27868852	1	1	63	tags=17%, list=3%, signal=17%
GOBP_REGULATION_OF_MEMBRANE_PERMEABILITY		26	-0.3569976	-1.1046591	0.3392857	1	1	211	tags=27%, list=11%, signal=30%
GOBP_STEROL_BIOSYNTHETIC_PROCESS		15	-0.356662	-1.1043278	0.3392857	1	1	198	tags=27%, list=11%, signal=30%
GOBP_POSITIVE_REGULATION_OF_PEPTIDASE_ACTIVITY		37	-0.3011502	-1.1013688	0.3220339	1	1	212	tags=24%, list=11%, signal=27%
KEGG_PPAR_SIGNALING_PATHWAY		25	-0.3217897	-1.1004239	0.34	1	1	235	tags=32%, list=13%, signal=36%
GOBP_TEMPERATURE_HOMEOSTASIS		24	-0.3219514	-1.1003203	0.2962963	1	1	354	tags=42%, list=19%, signal=51%
GOBP_MUSCLE_STRUCTURE_DEVELOPMENT		103	-0.2563774	-1.0988315	0.3508772	1	1	279	tags=25%, list=15%, signal=28%
REACTOME_CYTOSOLIC_TRNA_AMINOACYLATION		12	-0.3938019	-1.095642	0.26666668	1	1	777	tags=58%, list=42%, signal=99%
GOBP_SKELETAL_SYSTEM_MORPHOGENESIS		10	-0.4426041	-1.0953908	0.4	1	1	231	tags=30%, list=12%, signal=34%
REACTOME_AQUAPORIN_MEDIATED_TRANSPORT		13	-0.4229307	-1.0952334	0.3469388	1	1	173	tags=31%, list=9%, signal=34%
GOBP_MUSCLE_FILAMENT_SLIDING		19	-0.3555168	-1.0949796	0.29310346	1	1	379	tags=53%, list=20%, signal=65%
GOBP_ANION_TRANSMEMBRANE_TRANSPORT		102	-0.2425803	-1.0949179	0.25925925	1	1	389	tags=27%, list=21%, signal=33%
REACTOME_G_ALPHA_Z_SIGNALLING_EVENTS		12	-0.4143983	-1.094683	0.43103448	1	1	173	tags=33%, list=9%, signal=36%
GOBP_POSITIVE_REGULATION_OF_LEUKOCYTE_PROLIFERATION		15	-0.3761333	-1.0945482	0.32608697	1	1	260	tags=27%, list=14%, signal=31%
REACTOME_G_ALPHA_S_SIGNALLING_EVENTS		12	-0.4119606	-1.0943861	0.4347826	1	1	173	tags=33%, list=9%, signal=36%
KEGG_EPITHELIAL_CELL_SIGNALING_IN_HELICOBACTER_PYLORI_INFECTIO		12	-0.4086001	-1.0931457	0.35416666	1	1	15	tags=8%, list=1%, signal=8%
GOBP_PHOSPHATIDYLINOSITOL_METABOLIC_PROCESS		18	-0.3458981	-1.0920805	0.41379312	1	1	370	tags=33%, list=20%, signal=41%
GOBP_NEGATIVE_REGULATION_OF_ERBB_SIGNALING_PATHWAY		12	-0.3601226	-1.0897464	0.3043478	1	1	309	tags=25%, list=17%, signal=30%
KEGG_CARDIAC_MUSCLE_CONTRACTION		39	-0.3189357	-1.0853792	0.30952382	1	1	274	tags=38%, list=15%, signal=44%
GOBP_ORGANONITROGEN_COMPOUND_CATABOLIC_PROCESS		243	-0.220801	-1.0844487	0.234375	1	1	320	tags=19%, list=17%, signal=20%
GOBP_EPITHELIAL_TO_MESENCHYMAL_TRANSITION		13	-0.3758595	-1.0822409	0.32692307	1	1	477	tags=46%, list=25%, signal=62%
GOBP_RIBONUCLEOSIDE_TRIPHOSPHATE_METABOLIC_PROCESS		33	-0.3190041	-1.0821524	0.3448276	1	1	360	tags=39%, list=19%, signal=48%
GOBP_POSITIVE_REGULATION_OF_PROTEIN_LOCALIZATION_TO_MEMBRANE		30	-0.3337572	-1.0738294	0.3478261	1	1	155	tags=20%, list=8%, signal=21%
GOBP_NUCLEOSIDE_TRIPHOSPHATE_METABOLIC_PROCESS		38	-0.2918561	-1.073199	0.30645162	1	1	315	tags=32%, list=17%, signal=37%
GOBP_PROTEIN_HOMOOLIGOMERIZATION		26	-0.3239055	-1.0717182	0.3	1	1	125	tags=19%, list=7%, signal=20%
KEGG_GNRH_SIGNALING_PATHWAY		15	-0.3765213	-1.0715185	0.34	1	1	4	tags=7%, list=0%, signal=7%
GOBP_STRIATED_MUSCLE_CELL_DIFFERENTIATION		55	-0.2671632	-1.0712322	0.3653846	1	1	279	tags=27%, list=15%, signal=31%
GOBP_REGULATION_OF_CELLULAR_AMIDE_METABOLIC_PROCESS		76	-0.2595435	-1.0700899	0.36842105	1	1	275	tags=21%, list=15%, signal=24%

GOBP_REGULATION_OF_EXTRINSIC_APOPTOTIC_SIGNALING_PATHWAY	24	-0.3228023	-1.0700717	0.3272727	1	1	344	tags=29%, list=18%, signal=35%
GOBP_HEART_PROCESS	73	-0.2603913	-1.069275	0.27868852	1	1	203	tags=23%, list=11%, signal=25%
KEGG_PROPANOATE_METABOLISM	22	-0.3215712	-1.0681322	0.42	1	1	293	tags=36%, list=16%, signal=43%
GOBP_POSITIVE_REGULATION_OF_LEUKOCYTE_CELL_CELL_ADHESION	30	-0.3093683	-1.0669234	0.3508772	1	1	260	tags=23%, list=14%, signal=27%
GOBP_CARDIAC_CHAMBER_DEVELOPMENT	24	-0.3242682	-1.0660338	0.4	1	1	105	tags=21%, list=6%, signal=22%
GOBP_POTASSIUM_ION_TRANSPORT	31	-0.29728	-1.0620805	0.3773585	1	1	197	tags=23%, list=11%, signal=25%
GOBP_CARBOHYDRATE_DERIVATIVE_METABOLIC_PROCESS	196	-0.2131269	-1.0611917	0.22807017	1	1	400	tags=24%, list=21%, signal=27%
GOBP_ANATOMICAL_STRUCTURE_HOMEOSTASIS	62	-0.2634253	-1.0603747	0.37254903	1	1	128	tags=15%, list=7%, signal=15%
GOBP_SKELETAL_MUSCLE_ORGAN_DEVELOPMENT	27	-0.3318121	-1.0579243	0.35416666	1	1	423	tags=41%, list=23%, signal=52%
GOBP_CELL_CELL_JUNCTION_ORGANIZATION	39	-0.2760598	-1.0576917	0.4117647	1	1	150	tags=21%, list=8%, signal=22%
REACTOME_COLLAGEN_FORMATION	16	-0.3763609	-1.057582	0.40350878	1	1	208	tags=25%, list=11%, signal=28%
GOBP_REGULATION_OF_LEUKOCYTE_PROLIFERATION	21	-0.3436825	-1.0568306	0.34545454	1	1	260	tags=24%, list=14%, signal=27%
GOBP_REGULATION_OF_CELL_SHAPE	34	-0.3025218	-1.0563444	0.33333334	1	1	210	tags=24%, list=11%, signal=26%
GOBP_SKELETAL_SYSTEM_DEVELOPMENT	33	-0.3139597	-1.055191	0.3773585	1	1	258	tags=21%, list=14%, signal=24%
GOBP_HEAD_DEVELOPMENT	74	-0.26728	-1.0543724	0.3148148	1	1	175	tags=19%, list=9%, signal=20%
GOBP_DETECTION_OF_ABIOTIC_STIMULUS	12	-0.4069584	-1.0542012	0.4375	1	1	82	tags=25%, list=4%, signal=26%
GOBP_REGULATION_OF_BIOLOGICAL_PROCESS_INVOLVED_IN_SYMBIOTIC_	22	-0.3248624	-1.0541548	0.32692307	1	1	363	tags=27%, list=19%, signal=33%
GOBP_LEUKOCYTE_PROLIFERATION	29	-0.3123711	-1.0535816	0.38	1	1	260	tags=24%, list=14%, signal=28%
REACTOME_FORMATION_OF_ATP_BY_CHEMIOSMOTIC_COUPLING	13	-0.3914466	-1.0528961	0.375	1	1	315	tags=54%, list=17%, signal=64%
GOBP_APOPTOTIC_SIGNALING_PATHWAY	103	-0.2408946	-1.052702	0.3220339	1	1	249	tags=19%, list=13%, signal=21%
GOBP_REGULATION_OF_VACUOLE_ORGANIZATION	11	-0.3998092	-1.0523585	0.3859649	1	1	66	tags=18%, list=4%, signal=19%
GOBP_PROTEIN_TRANSMEMBRANE_TRANSPORT	26	-0.3385317	-1.0512209	0.3968254	1	1	264	tags=27%, list=14%, signal=31%
GOBP_CERAMIDE_METABOLIC_PROCESS	10	-0.4146102	-1.0504248	0.3846154	1	1	144	tags=20%, list=8%, signal=22%
GOBP_AMINO_ACID_TRANSPORT	17	-0.342938	-1.0502481	0.42	1	1	584	tags=53%, list=31%, signal=76%
REACTOME_INTEGRATION_OF_ENERGY_METABOLISM	30	-0.2919648	-1.0489546	0.3432836	1	1	173	tags=23%, list=9%, signal=25%
GOBP_NUCLEOBASE_METABOLIC_PROCESS	13	-0.3827232	-1.047749	0.390625	1	1	485	tags=38%, list=26%, signal=52%
GOBP_GLYCOSYL_COMPOUND_CATABOLIC_PROCESS	10	-0.427755	-1.0468668	0.38181818	1	1	559	tags=40%, list=30%, signal=57%
REACTOME_MUSCLE_CONTRACTION	56	-0.2611755	-1.0463175	0.3888889	1	1	279	tags=27%, list=15%, signal=31%
GOBP_MITOTIC_SISTER_CHROMATID_SEGREGATION	10	-0.4227654	-1.0440129	0.41509435	1	1	653	tags=60%, list=35%, signal=92%
REACTOME_RECRUITMENT_OF_MITOTIC_CENTROSOME_PROTEINS_AND_COI	11	-0.4198886	-1.0421305	0.37777779	1	1	337	tags=36%, list=18%, signal=44%
BIOCARTA_PPARA_PATHWAY	11	-0.3982177	-1.0416871	0.46	1	1	222	tags=36%, list=12%, signal=41%
GOBP_NEGATIVE_REGULATION_OF_EXTRINSIC_APOPTOTIC_SIGNALING_PAT	17	-0.3529271	-1.0399694	0.375	1	1	344	tags=35%, list=18%, signal=43%
GOBP_POSITIVE_REGULATION_OF_ESTABLISHMENT_OF_PROTEIN_LOCALIZ	69	-0.2409592	-1.0394166	0.3508772	1	1	420	tags=32%, list=22%, signal=40%
GOBP_POSITIVE_REGULATION_OF_MULTICELLULAR_ORGANISMAL_PROCES	162	-0.2218298	-1.0364861	0.37142858	1	1	330	tags=24%, list=18%, signal=27%
GOBP_ORGANOPHOSPHATE_ESTER_TRANSPORT	21	-0.350309	-1.0360497	0.3888889	1	1	389	tags=33%, list=21%, signal=42%
GOBP_MYELOID_LEUKOCYTE_DIFFERENTIATION	18	-0.3273701	-1.0359224	0.38	1	1	54	tags=11%, list=3%, signal=11%
GOBP_PROTEIN_COMPLEX_OLIGOMERIZATION	38	-0.2878928	-1.0341893	0.37096775	1	1	513	tags=37%, list=27%, signal=50%
REACTOME_RNA_POLYMERASE_II_TRANSCRIPTION	96	-0.236537	-1.0317253	0.3962264	1	1	238	tags=18%, list=13%, signal=19%
GOBP_NEGATIVE_REGULATION_OF_ENDOCYTOSIS	13	-0.3560237	-1.031355	0.44642857	1	1	420	tags=46%, list=22%, signal=59%
GOBP_PROTEIN_CONTAINING_COMPLEX_SUBUNIT_ORGANIZATION	420	-0.1975856	-1.0305853	0.3125	1	1	253	tags=16%, list=14%, signal=15%
GOBP_TRANSMEMBRANE_TRANSPORT	284	-0.207182	-1.0295937	0.42857143	1	1	389	tags=25%, list=21%, signal=27%
GOBP_CARBOHYDRATE_DERIVATIVE_BIOSYNTHETIC_PROCESS	115	-0.2326294	-1.0267051	0.41935483	1	1	315	tags=23%, list=17%, signal=26%
REACTOME_SIGNALING_BY_RHO_GTPASES_MIRO_GTPASES_AND_RHOBTB3	148	-0.2230123	-1.0260361	0.43103448	1	1	333	tags=23%, list=18%, signal=26%
GOBP_EMBRYO_DEVELOPMENT	80	-0.2521478	-1.0258533	0.37254903	1	1	344	tags=25%, list=18%, signal=29%
GOBP_ORGANOPHOSPHATE_METABOLIC_PROCESS	219	-0.2115286	-1.0252601	0.42857143	1	1	401	tags=26%, list=21%, signal=30%
REACTOME_MITOCHONDRIAL_PROTEIN_IMPORT	41	-0.2762972	-1.0237067	0.45454547	1	1	264	tags=29%, list=14%, signal=33%
GOBP_RESPONSE_TO_INSULIN	51	-0.2768977	-1.0231539	0.35714287	1	1	249	tags=18%, list=13%, signal=20%
GOBP_POSITIVE_REGULATION_OF_GENE_EXPRESSION	116	-0.2297471	-1.0228548	0.4090909	1	1	556	tags=37%, list=30%, signal=49%
REACTOME_RHO_GTPASES_ACTIVATE_FORMINS	21	-0.3294319	-1.0217426	0.43636364	1	1	636	tags=52%, list=34%, signal=78%
GOBP_NUCLEOBASE_CONTAINING_SMALL_MOLECULE_CATABOLIC_PROCES	12	-0.3762815	-1.0204836	0.43636364	1	1	398	tags=25%, list=21%, signal=32%
GOBP_SULFUR_COMPOUND_METABOLIC_PROCESS	102	-0.2205582	-1.0199319	0.42857143	1	1	386	tags=27%, list=21%, signal=33%
GOBP_REGULATION_OF_RESPONSE_TO_CYTOKINE_STIMULUS	17	-0.3451728	-1.0190325	0.4	1	1	318	tags=35%, list=17%, signal=42%
GOBP_REGULATION_OF_PH	17	-0.3484314	-1.0189538	0.42105263	1	1	96	tags=12%, list=5%, signal=12%
GOBP_POSITIVE_REGULATION_OF_APOPTOTIC_SIGNALING_PATHWAY	19	-0.3448006	-1.0181233	0.43137255	1	1	137	tags=16%, list=7%, signal=17%
GOBP_REGULATION_OF_GTPASE_ACTIVITY	52	-0.2774178	-1.0167013	0.4107143	1	1	627	tags=44%, list=34%, signal=65%
GOBP_MITOCHONDRIAL_ATP_SYNTHESIS_COUPLED_PROTON_TRANSPORT	15	-0.3686444	-1.0146977	0.41509435	1	1	315	tags=47%, list=17%, signal=56%
GOBP_ADAPTIVE_THERMOGENESIS	23	-0.3356419	-1.013382	0.4509804	1	1	354	tags=39%, list=19%, signal=48%

REACTOME_RHOC_GTPASE_CYCLE		23	-0.3276092	-1.0132606	0.39344263	1	1	363	tags=35%, list=19%, signal=43%
GOBP_REGULATION_OF_VIRAL_LIFE_CYCLE		17	-0.3409767	-1.0131791	0.42105263	1	1	208	tags=18%, list=11%, signal=20%
GOBP_REGULATION_OF_LYMPHOCYTE_ACTIVATION		39	-0.2866105	-1.0128598	0.5	1	1	260	tags=21%, list=14%, signal=23%
GOBP_REGULATION_OF_BINDING		49	-0.2611672	-1.0123097	0.42553192	1	1	160	tags=14%, list=9%, signal=15%
GOBP_SISTER_CHROMATID_SEGREGATION		12	-0.3869761	-1.0122674	0.48076922	1	1	309	tags=33%, list=17%, signal=40%
GOBP_REGULATION_OF_PROTEIN_TYROSINE_KINASE_ACTIVITY		10	-0.3782097	-1.0104309	0.37037036	1	1	309	tags=30%, list=17%, signal=36%
GOBP_ATP_SYNTHESIS_COUPLED_PROTON_TRANSPORT		16	-0.359803	-1.00973	0.45833334	1	1	315	tags=44%, list=17%, signal=52%
GOBP_RESPONSE_TO_DOPAMINE		14	-0.3657645	-1.0083954	0.44067797	1	1	36	tags=14%, list=2%, signal=14%
REACTOME_AGGREPHAGY		12	-0.3676186	-1.0073724	0.4651163	1	1	193	tags=25%, list=10%, signal=28%
GOBP_RETROGRADE_TRANSPORT_ENDOSOME_TO_GOLGI		16	-0.342462	-1.0069218	0.43103448	1	1	521	tags=38%, list=28%, signal=52%
GOBP_ORGANELLE_MEMBRANE_FUSION		28	-0.2966568	-1.0067903	0.4262295	1	1	96	tags=11%, list=5%, signal=11%
GOBP_TOR_SIGNALING		11	-0.3834645	-1.0063236	0.4375	1	1	469	tags=36%, list=25%, signal=48%
GOBP_MITOCHONDRIAL_TRANSLATION		90	-0.2379881	-1.0051867	0.4262295	1	1	553	tags=39%, list=30%, signal=53%
GOBP_RESPONSE_TO_DRUG		60	-0.2569903	-1.0044625	0.4489796	1	1	283	tags=23%, list=15%, signal=27%
GOBP_NUCLEOTIDE_TRANSPORT		10	-0.4031383	-1.0041499	0.4318182	1	1	484	tags=50%, list=26%, signal=67%
GOBP_CENTRAL_NERVOUS_SYSTEM_DEVELOPMENT		97	-0.228728	-1.0036231	0.43396226	1	1	364	tags=28%, list=19%, signal=33%
GOBP_SPHINGOLIPID_BIOSYNTHETIC_PROCESS		13	-0.3644104	-1.0029439	0.3962264	1	1	599	tags=46%, list=32%, signal=67%
GOBP_ESTABLISHMENT_OF_PROTEIN_LOCALIZATION_TO_MITOCHONDRIAL_		27	-0.2851745	-1.0022172	0.44444445	1	1	230	tags=22%, list=12%, signal=25%
GOBP_CALCIUM_ION_TRANSPORT		74	-0.2391956	-1.0021785	0.37037036	1	1	393	tags=30%, list=21%, signal=36%
GOBP_INORGANIC_ANION_TRANSPORT		19	-0.3208233	-1.0018691	0.43396226	1	1	464	tags=42%, list=25%, signal=55%
REACTOME_THROMBIN_SIGNALLING_THROUGH_PROTEINASE_ACTIVATED_R		12	-0.3471718	-1.0015372	0.4528302	1	1	173	tags=25%, list=9%, signal=27%
GOBP_PROTEIN_MODIFICATION_BY_SMALL_PROTEIN_CONJUGATION		80	-0.2489851	-0.999399	0.52	1	1	238	tags=16%, list=13%, signal=18%
GOBP_REGULATION_OF_RESPONSE_TO_ENDOPLASMIC_RETICULUM_STRES		17	-0.33938	-0.9981889	0.45614034	1	1	249	tags=24%, list=13%, signal=27%
GOBP_TISSUE_HOMEOSTASIS		37	-0.2749952	-0.9980068	0.51724136	1	1	126	tags=14%, list=7%, signal=14%
KEGG_BETA_ALANINE_METABOLISM		10	-0.3669855	-0.9979328	0.43137255	1	1	293	tags=40%, list=16%, signal=47%
GOBP_REGULATION_OF_HEART_RATE		28	-0.291885	-0.9975415	0.4814815	1	1	313	tags=32%, list=17%, signal=38%
GOBP_REGULATION_OF_RAS_PROTEIN_SIGNAL_TRANSDUCTION		23	-0.333814	-0.9973582	0.46551725	1	1	507	tags=39%, list=27%, signal=53%
GOBP_POSITIVE_REGULATION_OF_CHROMOSOME_ORGANIZATION		11	-0.4096595	-0.996944	0.47761193	1	1	128	tags=27%, list=7%, signal=29%
REACTOME_ANTI_INFLAMMATORY_RESPONSE_FAVOURING_LEISHMANIA_PA		19	-0.3457999	-0.995677	0.47916666	1	1	173	tags=26%, list=9%, signal=29%
GOBP_2_OXOGLUTARATE_METABOLIC_PROCESS		12	-0.3511779	-0.9949249	0.43103448	1	1	310	tags=50%, list=17%, signal=60%
GOBP_REGULATION_OF_PEPTIDYL_TYROSINE_PHOSPHORYLATION		29	-0.2832787	-0.9944427	0.4814815	1	1	372	tags=31%, list=20%, signal=38%
GOBP_CHROMOSOME_SEGREGATION		23	-0.3096601	-0.9942936	0.47272727	1	1	309	tags=26%, list=17%, signal=31%
REACTOME_COMPLEX_I_BIOGENESIS		45	-0.2687222	-0.9941184	0.3888889	1	1	335	tags=38%, list=18%, signal=45%
GOBP_PROTEIN_TARGETING_TO_MITOCHONDRION		42	-0.2703576	-0.9931347	0.4909091	1	1	606	tags=45%, list=32%, signal=65%
GOBP_ACTIN_FILAMENT_BASED_MOVEMENT		50	-0.2645575	-0.9924843	0.43137255	1	1	333	tags=34%, list=18%, signal=40%
GOBP_POSITIVE_REGULATION_OF_CELLULAR_PROTEIN_LOCALIZATION		65	-0.242517	-0.9924556	0.4814815	1	1	160	tags=17%, list=9%, signal=18%
GOBP_NEGATIVE_REGULATION_OF_NUCLEOBASE_CONTAINING_COMPOUND		72	-0.2378657	-0.9912036	0.49056605	1	1	132	tags=14%, list=7%, signal=14%
GOBP_POSITIVE_REGULATION_OF_MAP_KINASE_ACTIVITY		22	-0.3162604	-0.9910722	0.42857143	1	1	428	tags=27%, list=23%, signal=35%
GOBP_ACTOMYOSIN_STRUCTURE_ORGANIZATION		54	-0.2568197	-0.9897487	0.47540984	1	1	199	tags=19%, list=11%, signal=20%
GOBP_EPITHELIAL_TUBE_FORMATION		10	-0.3743555	-0.9886817	0.47368422	1	1	562	tags=50%, list=30%, signal=71%
GOBP_ORGANIC_ACID_TRANSMEMBRANE_TRANSPORT		34	-0.2834277	-0.988232	0.3859649	1	1	308	tags=26%, list=16%, signal=31%
GOBP_ORGANONITROGEN_COMPOUND_BIOSYNTHETIC_PROCESS		401	-0.1927207	-0.9881234	0.5147059	1	1	315	tags=19%, list=17%, signal=18%
GOBP_RESPONSE_TO_CATECHOLAMINE		21	-0.2976969	-0.9875352	0.43636364	1	1	36	tags=10%, list=2%, signal=10%
REACTOME_GLUCAGON_LIKE_PEPTIDE_1_GLP1_REGULATES_INSULIN_SECI		14	-0.3699026	-0.9864366	0.5471698	1	1	173	tags=29%, list=9%, signal=31%
GOBP_PROTEIN_MODIFICATION_BY_SMALL_PROTEIN_CONJUGATION_OR_RE		91	-0.2396898	-0.9859189	0.4716981	1	1	238	tags=15%, list=13%, signal=17%
GOBP_PROTEOLYSIS		236	-0.1992783	-0.9856934	0.5217391	1	1	325	tags=18%, list=17%, signal=19%
GOBP_REGULATION_OF_NEURON_PROJECTION_DEVELOPMENT		58	-0.2324888	-0.9855447	0.46296296	1	1	179	tags=14%, list=10%, signal=15%
REACTOME_SENSORY_PROCESSING_OF_SOUND		17	-0.3192789	-0.9850556	0.5	1	1	509	tags=47%, list=27%, signal=64%
GOBP_CELL_CELL_ADHESION		122	-0.2228539	-0.9837077	0.45454547	1	1	344	tags=21%, list=18%, signal=24%
KEGG_HYPERTROPHIC_CARDIOMYOPATHY_HCM		38	-0.2785727	-0.9835491	0.47916666	1	1	344	tags=37%, list=18%, signal=44%
GOBP_CARDIAC_MUSCLE_CELL_MEMBRANE_REPOLARIZATION		11	-0.3851394	-0.9811513	0.4716981	1	1	203	tags=36%, list=11%, signal=41%
GOBP_TYPE_I_INTERFERON_PRODUCTION		13	-0.3779215	-0.980587	0.47540984	1	1	328	tags=38%, list=18%, signal=46%
GOBP_POSITIVE_REGULATION_OF_INSULIN_SECRETION		12	-0.3833068	-0.9801446	0.48979592	1	1	420	tags=50%, list=22%, signal=64%
GOBP_NEGATIVE_REGULATION_OF_CELLULAR_AMIDE_METABOLIC_PROCES		21	-0.3162567	-0.9795702	0.4814815	1	1	137	tags=19%, list=7%, signal=20%
GOBP_MITOCHONDRIAL_FISSION		15	-0.3514364	-0.9794493	0.47826087	1	1	425	tags=40%, list=23%, signal=51%
GOBP_POSITIVE_REGULATION_OF_NUCLEOBASE_CONTAINING_COMPOUND_		111	-0.2171024	-0.9785199	0.4716981	1	1	128	tags=12%, list=7%, signal=12%
GOBP_REGULATION_OF_CHROMOSOME_ORGANIZATION		13	-0.3782879	-0.97768	0.4375	1	1	366	tags=38%, list=20%, signal=47%

GOBP_ZYMOGEN_ACTIVATION		11	-0.3771134	-0.9771531	0.44186047	1	1	409	tags=45%, list=22%, signal=58%
KEGG_PROXIMAL_TUBULE_BICARBONATE_RECLAMATION		10	-0.3742706	-0.9765922	0.5510204	1	1	145	tags=30%, list=8%, signal=32%
REACTOME_LEISHMANIA_INFECTION		43	-0.2509707	-0.9757207	0.46296296	1	1	216	tags=19%, list=12%, signal=21%
GOBP_REGULATION_OF_SMALL_GTPASE_MEDIATED_SIGNAL_TRANSDUCTIO		36	-0.2749946	-0.975528	0.44827586	1	1	507	tags=36%, list=27%, signal=49%
GOBP_ALCOHOL_BIOSYNTHETIC_PROCESS		25	-0.3145423	-0.974565	0.509434	1	1	198	tags=20%, list=11%, signal=22%
REACTOME_PROTEIN_FOLDING		27	-0.301252	-0.9724595	0.5471698	1	1	187	tags=22%, list=10%, signal=24%
GOBP_B_CELL_ACTIVATION		15	-0.35816	-0.9707313	0.51666665	1	1	260	tags=27%, list=14%, signal=31%
GOBP_CELLULAR_RESPONSE_TO_MECHANICAL_STIMULUS		14	-0.3411322	-0.9693933	0.5416667	1	1	420	tags=36%, list=22%, signal=46%
GOBP_LYMPHOCYTE_ACTIVATION_INVOLVED_IN_IMMUNE_RESPONSE		18	-0.3233537	-0.9677322	0.5090909	1	1	469	tags=33%, list=25%, signal=44%
GOBP_NEGATIVE_REGULATION_OF_DEPHOSPHORYLATION		11	-0.3762359	-0.9676044	0.5423729	1	1	62	tags=18%, list=3%, signal=19%
GOBP_GASTRULATION		20	-0.3064926	-0.9667211	0.5074627	1	1	344	tags=40%, list=18%, signal=48%
GOBP_MORPHOGENESIS_OF_EMBRYONIC_EPITHELIUM		11	-0.4029771	-0.9665095	0.5625	1	1	562	tags=55%, list=30%, signal=78%
GOBP_MONOCARBOXYLIC_ACID_BIOSYNTHETIC_PROCESS		50	-0.2518533	-0.9660054	0.56	1	1	318	tags=30%, list=17%, signal=35%
KEGG_HEMATOPOIETIC_CELL_LINEAGE		13	-0.331404	-0.9657306	0.44230768	1	1	53	tags=15%, list=3%, signal=16%
REACTOME_RESOLUTION_OF_SISTER_CHROMATID_COHESION		15	-0.3277135	-0.9646256	0.58928573	1	1	261	tags=20%, list=14%, signal=23%
GOBP_CELLULAR_CARBOHYDRATE_BIOSYNTHETIC_PROCESS		15	-0.3616252	-0.9645794	0.5	1	1	654	tags=53%, list=35%, signal=81%
GOBP_GLUCAN_BIOSYNTHETIC_PROCESS		11	-0.3503645	-0.9641033	0.5185185	1	1	654	tags=64%, list=35%, signal=97%
GOBP_RESPONSE_TO_XENOBIOTIC_STIMULUS		19	-0.327714	-0.963073	0.4814815	1	1	236	tags=26%, list=13%, signal=30%
REACTOME_COOPERATION_OF_PDCL_PHLP1_AND_TRIC_CCT_IN_G_PROTEII		18	-0.3252039	-0.9619502	0.5576923	1	1	173	tags=28%, list=9%, signal=30%
REACTOME_DNA_REPAIR		12	-0.3579455	-0.9619029	0.58	1	1	268	tags=17%, list=14%, signal=19%
GOBP_NEGATIVE_REGULATION_OF_NERVOUS_SYSTEM_DEVELOPMENT		12	-0.3656834	-0.961242	0.5744681	1	1	477	tags=42%, list=25%, signal=56%
GOBP_NERVOUS_SYSTEM_PROCESS		77	-0.2325534	-0.9605785	0.48076922	1	1	249	tags=18%, list=13%, signal=20%
REACTOME_COSTIMULATION_BY_THE_CD28_FAMILY		11	-0.3667426	-0.9601866	0.48979592	1	1	605	tags=45%, list=32%, signal=67%
KEGG_PENTOSE_PHOSPHATE_PATHWAY		10	-0.3873851	-0.958652	0.44	1	1	84	tags=20%, list=4%, signal=21%
GOBP_CARDIAC_VENTRICLE_MORPHOGENESIS		13	-0.35097	-0.9574463	0.4680851	1	1	92	tags=31%, list=5%, signal=32%
GOBP_REGULATION_OF_PLASMA_LIPOPROTEIN_PARTICLE_LEVELS		25	-0.2886601	-0.9571409	0.5531915	1	1	318	tags=32%, list=17%, signal=38%
GOBP_RIBOSE_PHOSPHATE_BIOSYNTHETIC_PROCESS		67	-0.2402513	-0.9570603	0.56363636	1	1	315	tags=28%, list=17%, signal=33%
GOBP_APICAL_JUNCTION_ASSEMBLY		17	-0.3303464	-0.9569892	0.58	1	1	148	tags=24%, list=8%, signal=25%
GOBP_DOPAMINE_RECEPTOR_SIGNALING_PATHWAY		11	-0.3894261	-0.9568975	0.55932206	1	1	36	tags=18%, list=2%, signal=18%
GOBP_CELLULAR_AMIDE_METABOLIC_PROCESS		325	-0.189371	-0.9554991	0.68115944	1	1	314	tags=18%, list=17%, signal=18%
GOBP_BIOLOGICAL_PROCESS_INVOLVED_IN_SYMBIOTIC_INTERACTION		237	-0.1920108	-0.9541231	0.65625	1	1	265	tags=16%, list=14%, signal=17%
GOBP_PROTEIN_N_LINKED_GLYCOSYLATION		15	-0.3566667	-0.9534104	0.5090909	1	1	298	tags=20%, list=16%, signal=24%
GOBP_STRIATED_MUSCLE_ADAPTATION		12	-0.3418948	-0.9530269	0.5714286	1	1	482	tags=42%, list=26%, signal=56%
GOBP_NADH_DEHYDROGENASE_COMPLEX_ASSEMBLY		50	-0.2624329	-0.9516764	0.5423729	1	1	335	tags=36%, list=18%, signal=43%
GOBP_REGULATION_OF_NEURON_DIFFERENTIATION		13	-0.3574733	-0.9511977	0.54901963	1	1	501	tags=46%, list=27%, signal=63%
GOBP_CARDIAC_CHAMBER_MORPHOGENESIS		17	-0.3126791	-0.9507476	0.5102041	1	1	92	tags=24%, list=5%, signal=25%
REACTOME_REGULATION_OF_PLK1_ACTIVITY_AT_G2_M_TRANSITION		14	-0.3335847	-0.9487647	0.537037	1	1	337	tags=29%, list=18%, signal=35%
GOBP_POSITIVE_REGULATION_OF_CELL_DIFFERENTIATION		92	-0.2305305	-0.9481514	0.53846157	1	1	627	tags=43%, list=34%, signal=62%
REACTOME_ADORA2B_MEDIATED_ANTI_INFLAMMATORY_CYTOKINES_PRODU		14	-0.3514602	-0.9476088	0.5185185	1	1	173	tags=29%, list=9%, signal=31%
GOBP_MAINTENANCE_OF_PROTEIN_LOCATION_IN_CELL		17	-0.3103253	-0.9476054	0.48076922	1	1	416	tags=35%, list=22%, signal=45%
GOBP_CARBOHYDRATE_DERIVATIVE_CATABOLIC_PROCESS		41	-0.2683313	-0.9470225	0.55172414	1	1	705	tags=37%, list=38%, signal=57%
GOBP_REGULATION_OF_SYNAPSE_STRUCTURE_OR_ACTIVITY		33	-0.265926	-0.9464144	0.55172414	1	1	249	tags=18%, list=13%, signal=21%
GOBP_DNA_REPAIR		26	-0.2726214	-0.9463973	0.53846157	1	1	268	tags=15%, list=14%, signal=18%
GOBP_JNK_CASCADE		13	-0.3496409	-0.9457812	0.5686275	1	1	269	tags=23%, list=14%, signal=27%
GOBP_ALPHA_AMINO_ACID_BIOSYNTHETIC_PROCESS		11	-0.3725539	-0.9441821	0.5	1	1	482	tags=55%, list=26%, signal=73%
GOBP_POSITIVE_REGULATION_OF_BIOSYNTHETIC_PROCESS		143	-0.2073789	-0.943637	0.5925926	1	1	267	tags=17%, list=14%, signal=18%
GOBP_POSITIVE_REGULATION_OF_PEPTIDE_HORMONE_SECRETION		16	-0.323122	-0.9430013	0.5714286	1	1	420	tags=44%, list=22%, signal=56%
GOBP_EXTRINSIC_APOPTOTIC_SIGNALING_PATHWAY		27	-0.2964759	-0.9426249	0.4893617	1	1	344	tags=26%, list=18%, signal=31%
REACTOME_SLC_MEDIATED_TRANSMEMBRANE_TRANSPORT		20	-0.3154963	-0.9406925	0.57894737	1	1	369	tags=35%, list=20%, signal=43%
GOBP_FATTY_ACID_TRANSMEMBRANE_TRANSPORT		17	-0.3385102	-0.9405348	0.50793654	1	1	306	tags=41%, list=16%, signal=49%
GOBP_ESTABLISHMENT_OF_PROTEIN_LOCALIZATION_TO_MEMBRANE		126	-0.2146986	-0.9394692	0.53846157	1	1	269	tags=17%, list=14%, signal=18%
GOBP_PEPTIDYL_AMINO_ACID_MODIFICATION		116	-0.2107431	-0.9393134	0.5714286	1	1	318	tags=20%, list=17%, signal=22%
GOBP_CELLULAR_RESPONSE_TO_STARVATION		16	-0.3292262	-0.9386065	0.5555556	1	1	103	tags=13%, list=6%, signal=13%
GOBP_RESPIRATORY_SYSTEM_DEVELOPMENT		16	-0.3245438	-0.9378283	0.54	1	1	591	tags=31%, list=32%, signal=45%
GOBP_CELL_CYCLE		174	-0.1934166	-0.9378134	0.58928573	1	1	419	tags=23%, list=22%, signal=27%
GOBP_REGULATION_OF_CELL_CELL_ADHESION		55	-0.2436329	-0.9371752	0.5873016	1	1	328	tags=22%, list=18%, signal=26%
GOBP_NEGATIVE_REGULATION_OF_CELLULAR_PROTEIN_CATABOLIC_PROC		10	-0.3402056	-0.9369717	0.5645161	1	1	269	tags=30%, list=14%, signal=35%

GOBP_NEGATIVE_REGULATION_OF_PROTEIN_MODIFICATION_PROCESS	82	-0.2231758	-0.936327	0.5714286	1	1	318	tags=21%, list=17%, signal=24%
GOBP_PROTEIN_TARGETING	170	-0.2010987	-0.9362608	0.6212121	1	1	269	tags=17%, list=14%, signal=18%
GOBP_MUSCLE_ORGAN_MORPHOGENESIS	16	-0.3288492	-0.9353957	0.5740741	1	1	274	tags=38%, list=15%, signal=44%
GOBP_REGULATION_OF_APOPTOTIC_SIGNALING_PATHWAY	64	-0.2424707	-0.934922	0.54901963	1	1	393	tags=28%, list=21%, signal=34%
GOBP_GLOMERULUS_DEVELOPMENT	10	-0.366403	-0.9343993	0.53061223	1	1	318	tags=30%, list=17%, signal=36%
GOBP_LEARNING	10	-0.386894	-0.9337537	0.5652174	1	1	215	tags=30%, list=11%, signal=34%
GOBP_POSITIVE_REGULATION_OF_CELL_CELL_ADHESION	39	-0.2580556	-0.9333625	0.5483871	1	1	451	tags=31%, list=24%, signal=40%
REACTOME_CELLULAR_RESPONSES_TO_EXTERNAL_STIMULI	190	-0.1983724	-0.9302121	0.6268657	1	1	258	tags=16%, list=14%, signal=17%
GOBP_RHYTHMIC_PROCESS	27	-0.2771275	-0.9298873	0.54761904	1	1	450	tags=30%, list=24%, signal=38%
GOBP_PEPTIDYL_TYROSINE_MODIFICATION	32	-0.2693496	-0.9296175	0.55172414	1	1	372	tags=28%, list=20%, signal=35%
REACTOME_RHOD_GTPASE_CYCLE	14	-0.3388169	-0.9290196	0.55932206	1	1	149	tags=21%, list=8%, signal=23%
GOBP_SPERM_EGG_RECOGNITION	10	-0.3792764	-0.9288352	0.6041667	1	1	128	tags=30%, list=7%, signal=32%
GOBP_SULFUR_COMPOUND_CATABOLIC_PROCESS	18	-0.3025623	-0.9286919	0.5283019	1	1	372	tags=28%, list=20%, signal=34%
GOBP_REGULATION_OF_CALCIUM_ION_TRANSMEMBRANE_TRANSPORTER_A	22	-0.2857453	-0.9283247	0.5254237	1	1	274	tags=23%, list=15%, signal=26%
GOBP_ENERGY_RESERVE_METABOLIC_PROCESS	17	-0.3237486	-0.9277865	0.5740741	1	1	45	tags=12%, list=2%, signal=12%
GOBP_POSITIVE_REGULATION_OF_CELL_ACTIVATION	39	-0.265446	-0.926471	0.6333333	1	1	260	tags=18%, list=14%, signal=20%
GOBP_POSITIVE_REGULATION_OF_SMALL_MOLECULE_METABOLIC_PROCES	23	-0.2926703	-0.926268	0.5090909	1	1	318	tags=26%, list=17%, signal=31%
GOBP_CELLULAR_RESPONSE_TO_HYDROGEN_PEROXIDE	15	-0.319085	-0.9251182	0.53333336	1	1	137	tags=20%, list=7%, signal=21%
GOBP_VASOCONSTRICTION	12	-0.3489682	-0.9251114	0.5272727	1	1	330	tags=42%, list=18%, signal=50%
GOBP_REGULATION_OF_MYELOID_LEUKOCYTE_DIFFERENTIATION	10	-0.3779513	-0.9250966	0.5625	1	1	4	tags=10%, list=0%, signal=10%
GOBP_IMMUNE_EFFECTOR_PROCESS	233	-0.1883872	-0.9238399	0.72131145	1	1	469	tags=29%, list=25%, signal=34%
GOBP_STEROL_HOMEOSTASIS	14	-0.3288318	-0.9228979	0.5964912	1	1	234	tags=29%, list=13%, signal=32%
GOBP_REGULATION_OF_T_CELL_ACTIVATION	35	-0.2561755	-0.9221544	0.5576923	1	1	260	tags=20%, list=14%, signal=23%
GOBP_LYMPHOCYTE_COSTIMULATION	11	-0.3555725	-0.9196636	0.475	1	1	420	tags=36%, list=22%, signal=47%
GOBP_CARDIAC_MUSCLE_TISSUE_MORPHOGENESIS	15	-0.3307416	-0.9184515	0.6181818	1	1	274	tags=40%, list=15%, signal=46%
GOBP_POSITIVE_REGULATION_OF_HYDROLASE_ACTIVITY	112	-0.2088654	-0.9181743	0.6315789	1	1	212	tags=16%, list=11%, signal=17%
GOBP_CELLULAR_RESPONSE_TO_NITROGEN_COMPOUND	117	-0.2169831	-0.917462	0.68421054	1	1	330	tags=21%, list=18%, signal=24%
REACTOME_THROMBOXANE_SIGNALLING_THROUGH_TP_RECEPTOR	10	-0.3537205	-0.9156368	0.6122449	1	1	173	tags=30%, list=9%, signal=33%
GOBP_REGULATION_OF_CELLULAR_CARBOHYDRATE_METABOLIC_PROCESS	17	-0.3131437	-0.9153572	0.5	1	1	447	tags=41%, list=24%, signal=54%
GOBP_MOVEMENT_IN_HOST_ENVIRONMENT	45	-0.2379723	-0.9127435	0.5714286	1	1	291	tags=20%, list=16%, signal=23%
GOBP_VESICLE_MEDIATED_TRANSPORT_TO_THE_PLASMA_MEMBRANE	15	-0.3248798	-0.9127255	0.5576923	1	1	197	tags=20%, list=11%, signal=22%
REACTOME_RHO_GTPASE_CYCLE	100	-0.2076454	-0.9124007	0.6451613	1	1	444	tags=30%, list=24%, signal=37%
REACTOME_RHO_GTPASES_ACTIVATE_IQGAPS	10	-0.3737065	-0.9111692	0.5869565	1	1	187	tags=20%, list=10%, signal=22%
GOBP_RESPONSE_TO_STARVATION	23	-0.2824301	-0.9102539	0.5869565	1	1	129	tags=13%, list=7%, signal=14%
GOBP_DICARBOXYLIC_ACID_TRANSPORT	14	-0.334606	-0.9101459	0.59574467	1	1	584	tags=57%, list=31%, signal=82%
REACTOME_TRANSCRIPTIONAL_ACTIVATION_OF_MITOCHONDRIAL_BIOGENE	10	-0.3623441	-0.9101186	0.537037	1	1	144	tags=30%, list=8%, signal=32%
GOBP_MITOCHONDRIAL_GENE_EXPRESSION	97	-0.2129961	-0.9098325	0.6666667	1	1	553	tags=38%, list=30%, signal=51%
KEGG_CALCIUM_SIGNALING_PATHWAY	30	-0.2711067	-0.9097975	0.6111111	1	1	130	tags=13%, list=7%, signal=14%
GOBP_NADP_METABOLIC_PROCESS	11	-0.3405661	-0.9097445	0.56363636	1	1	349	tags=36%, list=19%, signal=44%
GOBP_MUSCLE_FIBER_DEVELOPMENT	12	-0.3347718	-0.9084896	0.59615386	1	1	279	tags=33%, list=15%, signal=39%
REACTOME_COPI_INDEPENDENT_GOLGI_TO_ER_RETROGRADE_TRAFFIC	19	-0.3161913	-0.9082131	0.6458333	1	1	563	tags=42%, list=30%, signal=60%
GOBP_REGULATION_OF_PROTEIN_MODIFICATION_PROCESS	168	-0.1977586	-0.9077667	0.71666664	1	1	280	tags=19%, list=15%, signal=20%
GOBP_CELLULAR_RESPONSE_TO_PEPTIDE_HORMONE_STIMULUS	55	-0.2304131	-0.9058245	0.6	1	1	249	tags=16%, list=13%, signal=18%
GOBP_POSITIVE_REGULATION_OF_PROTEIN_SERINE_THREONINE_KINASE_A	35	-0.2559938	-0.9056698	0.6557377	1	1	428	tags=29%, list=23%, signal=36%
GOBP_MODULATION_BY_HOST_OF_SYMBIONT_PROCESS	13	-0.3479879	-0.9050242	0.6226415	1	1	190	tags=23%, list=10%, signal=26%
GOBP_GLYCOPROTEIN_BIOSYNTHETIC_PROCESS	28	-0.2711434	-0.9043058	0.5686275	1	1	134	tags=11%, list=7%, signal=11%
GOBP_REGULATION_OF_INSULIN_RECEPTOR_SIGNALING_PATHWAY	11	-0.3541924	-0.903002	0.60714287	1	1	3	tags=9%, list=0%, signal=9%
GOBP_RNA_LOCALIZATION	21	-0.2887057	-0.9024329	0.5185185	1	1	522	tags=48%, list=28%, signal=65%
GOBP_RESPONSE_TO_STEROID_HORMONE	46	-0.2440753	-0.9007664	0.69491524	1	1	318	tags=24%, list=17%, signal=28%
GOBP_REGULATION_OF_METAL_ION_TRANSPORT	46	-0.2549238	-0.9006341	0.6086956	1	1	276	tags=22%, list=15%, signal=25%
GOBP_VENTRICULAR_CARDIAC_MUSCLE_TISSUE_DEVELOPMENT	15	-0.3209013	-0.900391	0.5714286	1	1	92	tags=27%, list=5%, signal=28%
GOBP_MODULATION_OF_PROCESS_OF_OTHER_ORGANISM	18	-0.2999262	-0.89988	0.5849057	1	1	210	tags=22%, list=11%, signal=25%
GOBP_PROTEIN_HOMOTETRAMERIZATION	11	-0.3401273	-0.8987812	0.55737704	1	1	490	tags=55%, list=26%, signal=73%
KEGG_PRION_DISEASES	11	-0.3481031	-0.8977004	0.5714286	1	1	562	tags=45%, list=30%, signal=65%
GOBP_CONNECTIVE_TISSUE_DEVELOPMENT	14	-0.3140594	-0.8965187	0.625	1	1	4	tags=7%, list=0%, signal=7%
GOBP_MUSCLE_TISSUE_DEVELOPMENT	69	-0.2177064	-0.894011	0.6792453	1	1	460	tags=35%, list=25%, signal=44%
REACTOME_SMOOTH_MUSCLE_CONTRACTION	18	-0.2817631	-0.8937331	0.6551724	1	1	231	tags=22%, list=12%, signal=25%

GOBP_PROTEIN_LOCALIZATION_TO_ORGANELLE		257	-0.1819371	-0.8936509	0.78333336	1	1	264	tags=16%, list=14%, signal=16%
GOBP_PROTEIN_TETRAMERIZATION		15	-0.3118582	-0.8936119	0.6	1	1	513	tags=47%, list=27%, signal=64%
GOBP_PROTEIN_KINASE_B_SIGNALING		32	-0.2611918	-0.8935157	0.66	1	1	477	tags=38%, list=25%, signal=49%
REACTOME_G_ALPHA_I_SIGNALLING_EVENTS		32	-0.2498367	-0.8930531	0.7222222	1	1	173	tags=16%, list=9%, signal=17%
GOBP_CELL_ACTIVATION_INVOLVED_IN_IMMUNE_RESPONSE		179	-0.1892024	-0.8930518	0.8135593	1	1	469	tags=29%, list=25%, signal=35%
GOBP_POSITIVE_REGULATION_OF_CELL_ADHESION		75	-0.2194137	-0.8924868	0.7222222	1	1	352	tags=25%, list=19%, signal=30%
GOBP_SYNAPSE_ASSEMBLY		17	-0.2986765	-0.8920689	0.5833333	1	1	129	tags=12%, list=7%, signal=13%
REACTOME_CELL_CYCLE_CHECKPOINTS		47	-0.2360194	-0.8917888	0.6896552	1	1	261	tags=17%, list=14%, signal=19%
GOBP_TRANSLATIONAL_TERMINATION		71	-0.2183368	-0.891663	0.75	1	1	552	tags=37%, list=30%, signal=50%
GOBP_CELL_JUNCTION_ASSEMBLY		73	-0.2200792	-0.886628	0.6551724	1	1	182	tags=15%, list=10%, signal=16%
GOBP_RIBOSOMAL_LARGE_SUBUNIT_ASSEMBLY		12	-0.3166355	-0.8862361	0.5849057	1	1	6	tags=8%, list=0%, signal=8%
GOBP_POSITIVE_REGULATION_OF_PROTEIN_MODIFICATION_PROCESS		99	-0.2029168	-0.886125	0.6964286	1	1	269	tags=19%, list=14%, signal=21%
REACTOME_SULFUR_AMINO_ACID_METABOLISM		10	-0.3427424	-0.8861117	0.6181818	1	1	282	tags=30%, list=15%, signal=35%
GOBP_REGULATION_OF_OXIDATIVE_STRESS_INDUCED_CELL_DEATH		13	-0.3172985	-0.885896	0.6923077	1	1	208	tags=31%, list=11%, signal=34%
GOBP_PROTEIN_LOCALIZATION_TO_CHROMOSOME		11	-0.3440172	-0.883994	0.59183675	1	1	128	tags=27%, list=7%, signal=29%
GOBP_PYRIMIDINE_CONTAINING_COMPOUND_METABOLIC_PROCESS		12	-0.3479982	-0.8822603	0.66071427	1	1	52	tags=17%, list=3%, signal=17%
GOBP_METAL_ION_TRANSPORT		81	-0.2103018	-0.8821357	0.703125	1	1	393	tags=27%, list=21%, signal=33%
GOBP_DEPHOSPHORYLATION		60	-0.2130347	-0.8814764	0.6666667	1	1	192	tags=13%, list=10%, signal=14%
GOBP_REGULATION_OF_INTRINSIC_APOPTOTIC_SIGNALING_PATHWAY		33	-0.2513491	-0.8813989	0.68421054	1	1	249	tags=24%, list=13%, signal=27%
GOBP_POSITIVE_REGULATION_OF_MITOCHONDRION_ORGANIZATION		23	-0.2957323	-0.8800212	0.70454544	1	1	249	tags=26%, list=13%, signal=30%
GOBP_PROTEIN_INSERTION_INTO_MEMBRANE		32	-0.2508874	-0.880016	0.66101694	1	1	230	tags=19%, list=12%, signal=21%
GOBP_SPINDLE_ORGANIZATION		21	-0.2766853	-0.8794374	0.6730769	1	1	337	tags=29%, list=18%, signal=34%
REACTOME_CELL_CELL_COMMUNICATION		26	-0.2683982	-0.8790324	0.6363636	1	1	182	tags=19%, list=10%, signal=21%
REACTOME_ABC_FAMILY_PROTEINS_MEDIATED_TRANSPORT		47	-0.2256807	-0.8779783	0.6730769	1	1	412	tags=21%, list=22%, signal=27%
GOBP_POSITIVE_REGULATION_OF_HORMONE_SECRETION		18	-0.3120489	-0.8759521	0.6666667	1	1	420	tags=39%, list=22%, signal=50%
REACTOME_IRON_UPTAKE_AND_TRANSPORT		13	-0.3345923	-0.8748763	0.64705884	1	1	494	tags=31%, list=26%, signal=42%
GOBP_REGULATION_OF_DNA_BIOSYNTHETIC_PROCESS		18	-0.3093951	-0.8742014	0.625	1	1	366	tags=39%, list=20%, signal=48%
GOBP_MITOCHONDRIAL_TRANSLATIONAL_TERMINATION		67	-0.2195154	-0.8739514	0.6545454	1	1	552	tags=37%, list=30%, signal=51%
REACTOME_LAMININ_INTERACTIONS		14	-0.316821	-0.873951	0.61403507	1	1	48	tags=14%, list=3%, signal=15%
GOBP_ORGANIC_CYCLIC_COMPOUND_CATABOLIC_PROCESS		167	-0.1804021	-0.8714446	0.7536232	1	1	259	tags=14%, list=14%, signal=15%
GOBP_CELLULAR_RESPONSE_TO_PEPTIDE		68	-0.2123022	-0.8713377	0.7	1	1	249	tags=16%, list=13%, signal=18%
GOBP_POSITIVE_REGULATION_OF_PEPTIDYL_TYROSINE_PHOSPHORYLATIC		19	-0.2918851	-0.870957	0.5813953	1	1	447	tags=37%, list=24%, signal=48%
GOBP_CELL_AGING		15	-0.3025524	-0.8689029	0.7	1	1	501	tags=33%, list=27%, signal=45%
GOBP_POSITIVE_REGULATION_OF_T_CELL_PROLIFERATION		11	-0.3342944	-0.8677259	0.627451	1	1	260	tags=27%, list=14%, signal=31%
GOBP_POSITIVE_REGULATION_OF_BINDING		34	-0.2573066	-0.8655277	0.66101694	1	1	160	tags=15%, list=9%, signal=16%
GOBP_CELLULAR_PROTEIN_CONTAINING_COMPLEX_ASSEMBLY		226	-0.1806754	-0.8631965	0.83928573	1	1	250	tags=15%, list=13%, signal=16%
GOBP_EXPORT_ACROSS_PLASMA_MEMBRANE		15	-0.3001024	-0.8628067	0.65384614	1	1	330	tags=33%, list=18%, signal=40%
GOBP_MYELOID_LEUKOCYTE_MEDIATED_IMMUNITY		163	-0.1842168	-0.862802	0.8103448	1	1	468	tags=29%, list=25%, signal=35%
GOBP_TRANSLATIONAL_INITIATION		81	-0.2086177	-0.8623258	0.7358491	1	1	311	tags=19%, list=17%, signal=21%
GOBP_MODULATION_OF_PROCESS_OF_OTHER_ORGANISM_INVOLVED_IN_S		18	-0.2999262	-0.8623167	0.59574467	1	1	210	tags=22%, list=11%, signal=25%
GOBP_REGULATION_OF_CELL_MORPHOGENESIS		67	-0.209772	-0.8603267	0.73333335	1	1	271	tags=19%, list=14%, signal=22%
GOBP_POSTTRANSCRIPTIONAL_REGULATION_OF_GENE_EXPRESSION		109	-0.1987789	-0.8600302	0.75438595	1	1	275	tags=17%, list=15%, signal=18%
REACTOME_DETOXIFICATION_OF_REACTIVE_OXYGEN_SPECIES		16	-0.3011144	-0.859326	0.64	1	1	236	tags=31%, list=13%, signal=35%
GOBP_LIPOPROTEIN_METABOLIC_PROCESS		16	-0.3027964	-0.8578479	0.6415094	1	1	198	tags=19%, list=11%, signal=21%
KEGG_PATHOGENIC_ESCHERICHIA_COLI_INFECTION		20	-0.2900122	-0.8577631	0.6	1	1	187	tags=20%, list=10%, signal=22%
GOBP_ALCOHOL_METABOLIC_PROCESS		63	-0.2142402	-0.8577474	0.76363635	1	1	235	tags=17%, list=13%, signal=19%
REACTOME_SARS_COV_2_INFECTION		20	-0.271792	-0.8568934	0.68333334	1	1	400	tags=25%, list=21%, signal=31%
GOBP_REGULATION_OF_INTRACELLULAR_PROTEIN_TRANSPORT		47	-0.2361209	-0.8567962	0.6849315	1	1	416	tags=30%, list=22%, signal=37%
GOBP_MAINTENANCE_OF_PROTEIN_LOCATION		20	-0.2774741	-0.8566414	0.63793105	1	1	124	tags=20%, list=7%, signal=21%
GOBP_MITOTIC_SPINDLE_ORGANIZATION		16	-0.2747147	-0.8555396	0.75	1	1	337	tags=31%, list=18%, signal=38%
GOBP_POSITIVE_REGULATION_OF_CATALYTIC_ACTIVITY		180	-0.1875768	-0.855352	0.8095238	1	1	224	tags=16%, list=12%, signal=16%
REACTOME_APOPTOSIS		62	-0.209048	-0.8544676	0.7413793	1	1	249	tags=15%, list=13%, signal=16%
GOBP_CELLULAR_RESPONSE_TO_EXTERNAL_STIMULUS		40	-0.2554158	-0.8538806	0.7358491	1	1	236	tags=18%, list=13%, signal=20%
REACTOME_HSF1_ACTIVATION		12	-0.3372304	-0.8534503	0.62068963	1	1	193	tags=25%, list=10%, signal=28%
GOBP_SENSORY_PERCEPTION_OF_LIGHT_STIMULUS		11	-0.3155359	-0.8532427	0.6545454	1	1	249	tags=27%, list=13%, signal=31%
GOBP_NEGATIVE_REGULATION_OF_PROTEIN_MODIFICATION_BY_SMALL_PR		14	-0.2940726	-0.8518937	0.7090909	1	1	229	tags=21%, list=12%, signal=24%
GOBP_VESICLE_BUDDING_FROM_MEMBRANE		36	-0.2281674	-0.8516092	0.7058824	1	1	363	tags=25%, list=19%, signal=30%

GOBP_PROTEIN_TARGETING_TO_MEMBRANE		81	-0.2042344	-0.8501672	0.8	1	1	311	tags=17%, list=17%, signal=20%
GOBP_REGULATION_OF_PROTEIN_LOCALIZATION		163	-0.1844892	-0.8500617	0.8064516	1	1	428	tags=27%, list=23%, signal=32%
GOBP_MONONUCLEAR_CELL_DIFFERENTIATION		22	-0.273843	-0.8495656	0.73770493	1	1	584	tags=36%, list=31%, signal=52%
REACTOME_RAB_GEFS_EXCHANGE_GTP_FOR_GDP_ON_RABS		22	-0.2588906	-0.8492219	0.6730769	1	1	479	tags=41%, list=26%, signal=54%
GOBP_UROGENITAL_SYSTEM_DEVELOPMENT		33	-0.2439627	-0.8483546	0.75	1	1	329	tags=21%, list=18%, signal=25%
GOBP_ORGANELLE_FUSION		44	-0.2296598	-0.8454804	0.72727275	1	1	249	tags=16%, list=13%, signal=18%
GOBP_ACTIVATION_OF_MAPK_ACTIVITY		17	-0.2891071	-0.8453864	0.6851852	1	1	428	tags=29%, list=23%, signal=38%
GOBP_REGULATION_OF_ATP_METABOLIC_PROCESS		22	-0.2764948	-0.8449485	0.704918	1	1	528	tags=36%, list=28%, signal=50%
REACTOME_SARS_COV_1_INFECTION		12	-0.3221939	-0.8435722	0.6888889	1	1	400	tags=33%, list=21%, signal=42%
GOBP_REGULATION_OF_CELLULAR_PROTEIN_LOCALIZATION		111	-0.1899299	-0.8435551	0.75	1	1	160	tags=14%, list=9%, signal=14%
GOBP_PEPTIDE_METABOLIC_PROCESS		261	-0.1772023	-0.84264	0.89855075	1	1	424	tags=23%, list=23%, signal=25%
REACTOME_RHOBTB1_GTPASE_CYCLE		10	-0.3541639	-0.8414769	0.7058824	1	1	366	tags=30%, list=20%, signal=37%
GOBP_PROTEIN_ACYLATION		11	-0.315459	-0.8414084	0.67391306	1	1	296	tags=27%, list=16%, signal=32%
GOBP_POSITIVE_REGULATION_OF_PROTEOLYSIS		60	-0.2137189	-0.8411807	0.74603176	1	1	212	tags=17%, list=11%, signal=18%
GOBP_ESTABLISHMENT_OF_PROTEIN_LOCALIZATION_TO_ENDOPLASMIC_RE		73	-0.2042238	-0.8384378	0.8039216	1	1	311	tags=18%, list=17%, signal=21%
GOBP_MICROTUBULE_CYTOSKELETON_ORGANIZATION		54	-0.2192988	-0.8382965	0.71428573	1	1	420	tags=26%, list=22%, signal=32%
GOBP_PEPTIDE_BIOSYNTHETIC_PROCESS		221	-0.1769943	-0.8380449	0.8548387	1	1	576	tags=32%, list=31%, signal=41%
GOBP_POSITIVE_REGULATION_OF_MUSCLE_CELL_DIFFERENTIATION		10	-0.3420885	-0.8375513	0.65957445	1	1	80	tags=20%, list=4%, signal=21%
GOBP_RESPONSE_TO_NERVE_GROWTH_FACTOR		12	-0.3285959	-0.83398	0.63265306	1	1	125	tags=17%, list=7%, signal=18%
REACTOME_COPI_MEDIATED_ANTEROGRADE_TRANSPORT		36	-0.2262128	-0.8339741	0.71428573	1	1	495	tags=33%, list=26%, signal=44%
GOBP_CELL_CELL_RECOGNITION		14	-0.3041211	-0.833585	0.7346939	1	1	366	tags=36%, list=20%, signal=44%
GOBP_ESTABLISHMENT_OF_RNA_LOCALIZATION		13	-0.3236551	-0.8333605	0.6721311	1	1	517	tags=46%, list=28%, signal=63%
GOBP_MALE_GAMETE_GENERATION		23	-0.2727509	-0.8327262	0.65957445	1	1	505	tags=39%, list=27%, signal=53%
GOBP_AMIDE_BIOSYNTHETIC_PROCESS		254	-0.1681608	-0.8321502	0.91525424	1	1	311	tags=17%, list=17%, signal=18%
GOBP_REGULATION_OF_PROTEIN_BINDING		33	-0.2358565	-0.8318062	0.6896552	1	1	328	tags=21%, list=18%, signal=25%
GOBP_MULTICELLULAR_ORGANISMAL_SIGNALING		44	-0.2242739	-0.8314442	0.7647059	1	1	330	tags=25%, list=18%, signal=30%
REACTOME_G2_M_CHECKPOINTS		38	-0.2278077	-0.8313109	0.7222222	1	1	155	tags=13%, list=8%, signal=14%
GOBP_GLUTATHIONE_METABOLIC_PROCESS		19	-0.2579597	-0.8312905	0.7708333	1	1	386	tags=32%, list=21%, signal=39%
GOBP_REGULATION_OF_PEPTIDE_HORMONE_SECRETION		29	-0.2587692	-0.8310777	0.7647059	1	1	420	tags=38%, list=22%, signal=48%
GOBP_ERAD_PATHWAY		17	-0.2721201	-0.831004	0.6909091	1	1	85	tags=12%, list=5%, signal=12%
GOBP_POSITIVE_REGULATION_OF_MOLECULAR_FUNCTION		233	-0.1764098	-0.8298423	0.8507463	1	1	212	tags=14%, list=11%, signal=14%
GOBP_POSITIVE_REGULATION_OF_LIPID_BIOSYNTHETIC_PROCESS		16	-0.2947041	-0.8294421	0.68	1	1	224	tags=25%, list=12%, signal=28%
GOBP_ACIDIC_AMINO_ACID_TRANSPORT		11	-0.3353448	-0.8294206	0.6875	1	1	584	tags=64%, list=31%, signal=92%
REACTOME_PROGRAMMED_CELL_DEATH		70	-0.1997879	-0.8283023	0.79310346	1	1	328	tags=19%, list=18%, signal=22%
GOBP_MYELOID_LEUKOCYTE_MIGRATION		34	-0.2309574	-0.8282939	0.78431374	1	1	260	tags=18%, list=14%, signal=20%
REACTOME_SIGNALING_BY_HEDGEHOG		45	-0.2180488	-0.8277273	0.78846157	1	1	238	tags=13%, list=13%, signal=15%
GOBP_POSITIVE_REGULATION_OF_CELL_SUBSTRATE_ADHESION		38	-0.2307469	-0.8265647	0.75555557	1	1	352	tags=26%, list=19%, signal=32%
REACTOME_RHOA_GTPASE_CYCLE		32	-0.2424272	-0.8262878	0.67741936	1	1	363	tags=28%, list=19%, signal=34%
GOBP_MODIFICATION_DEPENDENT_MACROMOLECULE_CATABOLIC_PROCES		90	-0.1944296	-0.825828	0.86764705	1	1	146	tags=9%, list=8%, signal=9%
GOBP_UBIQUITIN_DEPENDENT_ERAD_PATHWAY		15	-0.3031451	-0.8257077	0.779661	1	1	85	tags=13%, list=5%, signal=14%
GOBP_NEGATIVE_REGULATION_OF_NEURON_PROJECTION_DEVELOPMENT		19	-0.2787085	-0.8255442	0.6964286	1	1	179	tags=21%, list=10%, signal=23%
GOBP_REGULATION_OF_TELOMERE_MAINTENANCE_VIA_TELOMERE_LENGT		10	-0.3275357	-0.8248603	0.62711865	1	1	366	tags=40%, list=20%, signal=49%
GOBP_CORTICAL_CYTOSKELETON_ORGANIZATION		20	-0.2704912	-0.8239497	0.67241377	1	1	303	tags=25%, list=16%, signal=30%
GOBP_TRNA_PROCESSING		10	-0.3188329	-0.8221037	0.6551724	1	1	553	tags=50%, list=30%, signal=71%
GOBP_MICROTUBULE_CYTOSKELETON_ORGANIZATION_INVOLVED_IN_MITOS		20	-0.2572117	-0.8217089	0.7708333	1	1	419	tags=35%, list=22%, signal=45%
GOBP_CALCIUM_ION_TRANSMEMBRANE_TRANSPORT		57	-0.2027374	-0.8209093	0.8490566	1	1	387	tags=28%, list=21%, signal=34%
GOBP_POSITIVE_REGULATION_OF_IMMUNE_SYSTEM_PROCESS		140	-0.1823123	-0.8207657	0.8679245	1	1	271	tags=15%, list=14%, signal=16%
GOBP_POSITIVE_REGULATION_OF_PROTEIN_METABOLIC_PROCESS		184	-0.17556	-0.8205181	0.875	1	1	276	tags=17%, list=15%, signal=18%
GOBP_CELLULAR_IRON_ION_HOMEOSTASIS		12	-0.2879513	-0.8175254	0.75	1	1	426	tags=33%, list=23%, signal=43%
GOBP_ADHERENS_JUNCTION_ORGANIZATION		15	-0.2889273	-0.815704	0.7777778	1	1	150	tags=20%, list=8%, signal=22%
REACTOME_SIGNALING_BY_GPCR		51	-0.2146522	-0.8155479	0.8	1	1	173	tags=12%, list=9%, signal=13%
GOBP_REGULATION_OF_MITOCHONDRIAL_GENE_EXPRESSION		11	-0.3328282	-0.8153005	0.7291667	1	1	263	tags=27%, list=14%, signal=32%
KEGG_T_CELL_RECEPTOR_SIGNALING_PATHWAY		10	-0.3278849	-0.8135711	0.75	1	1	148	tags=10%, list=8%, signal=11%
GOBP_REGULATION_OF_PROTEIN_LOCALIZATION_TO_MEMBRANE		49	-0.2179116	-0.8125454	0.754717	1	1	260	tags=18%, list=14%, signal=21%
GOBP_CELL_CYCLE_PROCESS		139	-0.1767974	-0.811991	0.8923077	1	1	376	tags=19%, list=20%, signal=23%
GOBP_SEXUAL_REPRODUCTION		48	-0.217554	-0.8119764	0.77272725	1	1	128	tags=13%, list=7%, signal=13%
GOBP_NEGATIVE_REGULATION_OF_CELLULAR_CATABOLIC_PROCESS		28	-0.2378254	-0.811716	0.75	1	1	269	tags=18%, list=14%, signal=21%

REACTOME_SENSORY_PERCEPTION		30	-0.2407988	-0.8116055	0.85714287	1	1	222	tags=20%, list=12%, signal=22%
REACTOME_BETA_CATENIN_INDEPENDENT_WNT_SIGNALING		52	-0.2057059	-0.8114217	0.8043478	1	1	173	tags=13%, list=9%, signal=14%
GOBP_MEMBRANE_DOCKING		38	-0.2258206	-0.8085524	0.7083333	1	1	468	tags=26%, list=25%, signal=34%
GOBP_CELLULAR_GLUCOSE_HOMEOSTASIS		21	-0.2682713	-0.8082753	0.68	1	1	249	tags=24%, list=13%, signal=27%
GOBP_POSITIVE_REGULATION_OF_CELL_POPULATION_PROLIFERATION		82	-0.1902567	-0.8070792	0.877193	1	1	507	tags=34%, list=27%, signal=45%
GOBP_ERBB_SIGNALING_PATHWAY		23	-0.2542873	-0.807054	0.75384617	1	1	737	tags=57%, list=39%, signal=92%
GOBP_MEMBRANE_BIOGENESIS		13	-0.2991729	-0.8059511	0.7288136	1	1	80	tags=15%, list=4%, signal=16%
GOBP_REGULATION_OF_DNA_METABOLIC_PROCESS		31	-0.2247668	-0.805733	0.8035714	1	1	366	tags=26%, list=20%, signal=32%
GOBP_REGULATION_OF_MITOCHONDRION_ORGANIZATION		37	-0.2287708	-0.8055905	0.7446808	1	1	249	tags=22%, list=13%, signal=24%
KEGG_GAP_JUNCTION		19	-0.2512167	-0.8055388	0.6730769	1	1	360	tags=21%, list=19%, signal=26%
GOBP_KETONE_BIOSYNTHETIC_PROCESS		13	-0.2994795	-0.8055097	0.7291667	1	1	162	tags=23%, list=9%, signal=25%
GOBP_MYELOID_LEUKOCYTE_ACTIVATION		171	-0.1715547	-0.8050903	0.94285715	1	1	468	tags=27%, list=25%, signal=33%
GOBP_SYNAPSE_ORGANIZATION		57	-0.2023913	-0.8045357	0.8727273	1	1	148	tags=12%, list=8%, signal=13%
GOBP_NEUTROPHIL_MIGRATION		21	-0.2523954	-0.8044208	0.76785713	1	1	420	tags=29%, list=22%, signal=36%
GOBP_ORGANELLE_FISSION		43	-0.2059442	-0.804264	0.7704918	1	1	563	tags=42%, list=30%, signal=59%
GOBP_LEUKOCYTE_MEDIATED_IMMUNITY		181	-0.1772951	-0.8031775	0.9130435	1	1	468	tags=28%, list=25%, signal=34%
REACTOME_MITOCHONDRIAL_TRANSLATION		71	-0.2026821	-0.8009425	0.8103448	1	1	491	tags=32%, list=26%, signal=42%
GOBP_SPINDLE_ASSEMBLY		10	-0.3230194	-0.7999071	0.78571427	1	1	254	tags=30%, list=14%, signal=35%
GOBP_CELLULAR_PROTEIN_CATABOLIC_PROCESS		131	-0.1757812	-0.7990984	0.9122807	1	1	269	tags=14%, list=14%, signal=15%
GOBP_TRANSFORMING_GROWTH_FACTOR_BETA_RECEPTOR_SIGNALING_PA		21	-0.2792373	-0.7990108	0.754717	1	1	150	tags=19%, list=8%, signal=20%
GOBP_NEGATIVE_REGULATION_OF_APOPTOTIC_SIGNALING_PATHWAY		41	-0.2257108	-0.7981103	0.7846154	1	1	393	tags=32%, list=21%, signal=39%
GOBP_POSITIVE_REGULATION_OF_INTRINSIC_APOPTOTIC_SIGNALING_PATH		11	-0.2933627	-0.7970998	0.7446808	1	1	137	tags=18%, list=7%, signal=20%
GOBP_CYTOPLASMIC_TRANSLATION		36	-0.2220243	-0.7957494	0.8245614	1	1	22	tags=6%, list=1%, signal=6%
GOBP_PROTEASOMAL_PROTEIN_CATABOLIC_PROCESS		79	-0.2044554	-0.7945013	0.8245614	1	1	159	tags=10%, list=8%, signal=11%
REACTOME_NEUTROPHIL_DEGRANULATION		155	-0.1791589	-0.7935422	0.9056604	1	1	466	tags=28%, list=25%, signal=35%
GOBP_REGULATION_OF_GLUCOSE_METABOLIC_PROCESS		15	-0.2792381	-0.7923323	0.7083333	1	1	447	tags=40%, list=24%, signal=52%
GOBP_MULTICELLULAR_ORGANISM_GROWTH		16	-0.2723867	-0.7899361	0.78	1	1	4	tags=6%, list=0%, signal=6%
GOBP_ORGAN_GROWTH		16	-0.2711136	-0.7874649	0.84126985	1	1	447	tags=31%, list=24%, signal=41%
GOBP_LIPID_BIOSYNTHETIC_PROCESS		121	-0.1784333	-0.7868028	0.9076923	1	1	318	tags=21%, list=17%, signal=23%
GOBP_T_CELL_MEDIATED_IMMUNITY		11	-0.3139339	-0.786552	0.75510204	1	1	449	tags=27%, list=24%, signal=36%
GOBP_MACROAUTOPHAGY		66	-0.1852953	-0.7855159	0.8958333	1	1	234	tags=12%, list=13%, signal=13%
GOBP_CENTRAL_NERVOUS_SYSTEM_NEURON_DIFFERENTIATION		10	-0.3073289	-0.7851915	0.7818182	1	1	449	tags=40%, list=24%, signal=52%
REACTOME_CARGO_TRAFFICKING_TO_THE_PERICILIARY_MEMBRANE		10	-0.3282606	-0.7847411	0.74545455	1	1	128	tags=20%, list=7%, signal=21%
GOBP_GROWTH		106	-0.183146	-0.7846847	0.9	1	1	269	tags=16%, list=14%, signal=18%
GOBP_REGULATION_OF_CELL_SIZE		35	-0.2431929	-0.7834452	0.7826087	1	1	627	tags=46%, list=34%, signal=67%
GOBP_CELLULAR_RESPONSE_TO_IONIZING_RADIATION		10	-0.3222706	-0.782655	0.75	1	1	23	tags=10%, list=1%, signal=10%
REACTOME_ASPARAGINE_N_LINKED_GLYCOSYLATION		75	-0.1892712	-0.7822317	0.88	1	1	501	tags=28%, list=27%, signal=37%
GOBP_TELOMERE_MAINTENANCE_VIA_TELOMERE_LENGTHENING		13	-0.2894878	-0.781107	0.74	1	1	366	tags=38%, list=20%, signal=47%
GOBP_POSITIVE_REGULATION_OF_PROTEIN_LOCALIZATION_TO_CELL_PERI		15	-0.274807	-0.7795752	0.8148148	1	1	584	tags=47%, list=31%, signal=67%
GOBP_DEVELOPMENTAL_GROWTH		74	-0.1911708	-0.7784463	0.90163934	1	1	260	tags=15%, list=14%, signal=17%
GOBP_REGULATION_OF_DNA_BINDING		10	-0.3169702	-0.7780717	0.7916667	1	1	160	tags=20%, list=9%, signal=22%
REACTOME_INTERFERON_GAMMA_SIGNALING		10	-0.301902	-0.7776368	0.8113208	1	1	449	tags=30%, list=24%, signal=39%
REACTOME_PARASITE_INFECTION		18	-0.2633383	-0.7769766	0.7692308	1	1	641	tags=50%, list=34%, signal=75%
REACTOME_GOLGI_TO_ER_RETROGRADE_TRANSPORT		37	-0.2038099	-0.7757815	0.78431374	1	1	359	tags=24%, list=19%, signal=30%
GOBP_T_CELL_PROLIFERATION		18	-0.2652026	-0.7756261	0.828125	1	1	260	tags=22%, list=14%, signal=26%
REACTOME_TRANSLATION		183	-0.1614674	-0.7731718	1	1	1	424	tags=22%, list=23%, signal=25%
GOBP_T_CELL_DIFFERENTIATION		18	-0.253271	-0.773114	0.877193	1	1	469	tags=28%, list=25%, signal=37%
GOBP_CELL_SUBSTRATE_JUNCTION_ORGANIZATION		31	-0.2303873	-0.7711884	0.8269231	1	1	150	tags=13%, list=8%, signal=14%
GOBP_MICROTUBULE_POLYMERIZATION		10	-0.2953981	-0.7708771	0.73214287	1	1	420	tags=30%, list=22%, signal=38%
GOBP_INSULIN_SECRETION		30	-0.2348601	-0.7708553	0.8148148	1	1	528	tags=47%, list=28%, signal=64%
GOBP_GAMETE_GENERATION		31	-0.2202519	-0.7706687	0.8095238	1	1	505	tags=35%, list=27%, signal=48%
GOBP_FERTILIZATION		17	-0.2553749	-0.7706029	0.7222222	1	1	128	tags=18%, list=7%, signal=19%
GOBP_BRANCHING_MORPHOGENESIS_OF_AN_EPITHELIAL_TUBE		13	-0.3019006	-0.76886	0.78431374	1	1	453	tags=31%, list=24%, signal=40%
GOBP_PROTEIN_LOCALIZATION_TO_ENDOPLASMIC_RETICULUM		83	-0.1812675	-0.7684405	0.96666664	1	1	330	tags=17%, list=18%, signal=20%
GOBP_CARBOHYDRATE_DERIVATIVE_TRANSPORT		14	-0.2809094	-0.7661937	0.87234044	1	1	336	tags=21%, list=18%, signal=26%
GOBP_LOCOMOTORY_BEHAVIOR		20	-0.2368523	-0.7649649	0.84615386	1	1	325	tags=20%, list=17%, signal=24%
GOBP_POSITIVE_REGULATION_OF_CELLULAR_AMIDE_METABOLIC_PROCES		35	-0.2175204	-0.7648113	0.92	1	1	267	tags=17%, list=14%, signal=20%

GOBP_REGULATION_OF_SYNAPSE_ASSEMBLY		10	-0.2971521	-0.7631491	0.79545456	1	1	1319	tags=100%, list=70%, signal=337%
GOBP_MULTICELLULAR_ORGANISM_REPRODUCTION		45	-0.2008203	-0.7631354	0.8148148	1	1	30	tags=7%, list=2%, signal=7%
GOBP_RESPONSE_TO_INTERFERON_GAMMA		30	-0.2286124	-0.7630223	0.8	1	1	545	tags=40%, list=29%, signal=56%
GOBP_COVALENT_CHROMATIN_MODIFICATION		17	-0.2624297	-0.7620329	0.84444445	1	1	144	tags=12%, list=8%, signal=13%
GOBP_POSITIVE_REGULATION_OF_PROTEIN_BINDING		22	-0.2463476	-0.7614844	0.84615386	1	1	146	tags=14%, list=8%, signal=15%
KEGG_STARCH_AND_SUCROSE_METABOLISM		14	-0.2971263	-0.7606449	0.7924528	1	1	124	tags=21%, list=7%, signal=23%
GOBP_DIGESTIVE_SYSTEM_PROCESS		10	-0.2888272	-0.759643	0.7368421	1	1	198	tags=20%, list=11%, signal=22%
GOBP_REGULATION_OF_CELL_DIFFERENTIATION		175	-0.1585165	-0.7594167	0.9649123	1	1	450	tags=23%, list=24%, signal=28%
GOBP_MITOCHONDRION_LOCALIZATION		12	-0.3010227	-0.7589199	0.70454544	1	1	249	tags=25%, list=13%, signal=29%
GOBP_PURINE_NUCLEOSIDE_MONOPHOSPHATE_METABOLIC_PROCESS		16	-0.2579233	-0.7586833	0.7708333	1	1	95	tags=13%, list=5%, signal=13%
GOBP_SINGLE_FERTILIZATION		16	-0.2703458	-0.7581873	0.7846154	1	1	128	tags=19%, list=7%, signal=20%
REACTOME_FORMATION_OF_TUBULIN_FOLDING_INTERMEDIATES_BY_CCT_T		13	-0.2782026	-0.7578163	0.7818182	1	1	187	tags=23%, list=10%, signal=25%
GOBP_RESPONSE_TO_IONIZING_RADIATION		16	-0.2647778	-0.7573185	0.88	1	1	159	tags=13%, list=8%, signal=14%
GOBP_REGULATION_OF_TELOMERE_MAINTENANCE		10	-0.3275357	-0.7572938	0.84444445	1	1	366	tags=40%, list=20%, signal=49%
REACTOME_SIGNALING_BY_WNT		67	-0.1849949	-0.7559893	0.8627451	1	1	173	tags=12%, list=9%, signal=13%
REACTOME_COOPERATION_OF_PREFOLDIN_AND_TRIC_CCT_IN_ACTIN_AND_		14	-0.2749481	-0.7556171	0.77272725	1	1	187	tags=21%, list=10%, signal=24%
GOBP_REGULATION_OF_LEUKOCYTE_DIFFERENTIATION		19	-0.256854	-0.7550597	0.75	1	1	4	tags=5%, list=0%, signal=5%
GOBP_MEMBRANE_LIPID_BIOSYNTHETIC_PROCESS		15	-0.2748273	-0.7545689	0.7647059	1	1	599	tags=40%, list=32%, signal=58%
GOBP_CELLULAR_RESPONSE_TO_ALCOHOL		10	-0.3239739	-0.7533917	0.76	1	1	313	tags=40%, list=17%, signal=48%
GOBP_ENDOMEMBRANE_SYSTEM_ORGANIZATION		94	-0.1691231	-0.7525552	0.95555556	1	1	489	tags=28%, list=26%, signal=36%
REACTOME_SELECTIVE_AUTOPHAGY		26	-0.2253415	-0.7514473	0.84444445	1	1	230	tags=19%, list=12%, signal=22%
REACTOME_SIGNAL_TRANSDUCTION_BY_L1		12	-0.3007602	-0.751288	0.82608694	1	1	627	tags=50%, list=34%, signal=75%
GOBP_RIBOSOMAL_LARGE_SUBUNIT_BIOGENESIS		19	-0.2430516	-0.7499765	0.7407407	1	1	6	tags=5%, list=0%, signal=5%
REACTOME_MYD88_INDEPENDENT_TLR4_CASCADE		10	-0.283794	-0.7498685	0.7735849	1	1	146	tags=10%, list=8%, signal=11%
GOBP_REGULATION_OF_CELLULAR_PROTEIN_CATABOLIC_PROCESS		47	-0.1969636	-0.7486848	0.9285714	1	1	269	tags=17%, list=14%, signal=19%
REACTOME_CELLULAR_RESPONSE_TO_STARVATION		76	-0.1829917	-0.7486646	0.91071427	1	1	311	tags=17%, list=17%, signal=20%
GOBP_POSITIVE_REGULATION_OF_STRESS_FIBER_ASSEMBLY		13	-0.2559871	-0.7470673	0.82258064	1	1	627	tags=62%, list=34%, signal=92%
REACTOME_G_ALPHA_Q_SIGNALLING_EVENTS		17	-0.258742	-0.7467715	0.8	1	1	173	tags=18%, list=9%, signal=19%
GOBP_REGULATION_OF_MAP_KINASE_ACTIVITY		33	-0.207488	-0.7457746	0.8695652	1	1	428	tags=24%, list=23%, signal=31%
GOBP_NEGATIVE_REGULATION_OF_CELL_GROWTH		19	-0.244942	-0.7450413	0.875	1	1	330	tags=32%, list=18%, signal=38%
GOBP_PROTEIN_DEPHOSPHORYLATION		45	-0.202288	-0.7448297	0.88135594	1	1	146	tags=11%, list=8%, signal=12%
GOBP_PURINE_NUCLEOSIDE_METABOLIC_PROCESS		20	-0.2460875	-0.743471	0.852459	1	1	360	tags=25%, list=19%, signal=31%
GOBP_RESPONSE_TO_CALCIUM_ION		30	-0.2166402	-0.7430911	0.8035714	1	1	68	tags=10%, list=4%, signal=10%
GOBP_ENDOSOME_ORGANIZATION		15	-0.272104	-0.7420573	0.8235294	1	1	396	tags=33%, list=21%, signal=42%
GOBP_REGULATION_OF_TRANSFERASE_ACTIVITY		116	-0.1674733	-0.7411649	0.9677419	1	1	318	tags=19%, list=17%, signal=21%
GOBP_DIGESTION		10	-0.2888272	-0.7406533	0.79310346	1	1	198	tags=20%, list=11%, signal=22%
GOBP_REGULATION_OF_GENERATION_OF_PRECURSOR_METABOLITES_AND		33	-0.2158488	-0.7401791	0.84615386	1	1	565	tags=39%, list=30%, signal=55%
GOBP_REGULATION_OF_MEMBRANE_DEPOLARIZATION		10	-0.2983317	-0.7394225	0.8113208	1	1	416	tags=40%, list=22%, signal=51%
GOBP_RESPONSE_TO_ACID_CHEMICAL		17	-0.2399113	-0.7392355	0.84	1	1	482	tags=41%, list=26%, signal=55%
GOBP_POLYOL_BIOSYNTHETIC_PROCESS		10	-0.2836316	-0.7391304	0.8076923	1	1	61	tags=10%, list=3%, signal=10%
GOBP_RNA_SPLICING_VIA_TRANSESTERIFICATION_REACTIONS		11	-0.291326	-0.7387644	0.8	1	1	401	tags=36%, list=21%, signal=46%
REACTOME_SEMAPHORIN_INTERACTIONS		24	-0.224163	-0.7385186	0.82978725	1	1	193	tags=17%, list=10%, signal=18%
GOBP_MITOCHONDRION_MORPHOGENESIS		10	-0.2971386	-0.7384886	0.82258064	1	1	249	tags=20%, list=13%, signal=23%
GOBP_TELOMERE_ORGANIZATION		15	-0.265852	-0.7367462	0.8035714	1	1	254	tags=27%, list=14%, signal=31%
GOBP_PHOSPHOLIPID_METABOLIC_PROCESS		65	-0.1821844	-0.7366022	0.94827586	1	1	401	tags=25%, list=21%, signal=30%
GOBP_MEMBRANE_FUSION		41	-0.2002169	-0.7347175	0.877551	1	1	508	tags=29%, list=27%, signal=39%
GOBP_LIPID_LOCALIZATION		77	-0.1890686	-0.7338246	0.96	1	1	344	tags=22%, list=18%, signal=26%
GOBP_RESPONSE_TO_ESTRADIOL		15	-0.2607716	-0.7328332	0.9230769	1	1	501	tags=40%, list=27%, signal=54%
REACTOME_TRANSPORT_TO_THE_GOLGI_AND_SUBSEQUENT_MODIFICATION		54	-0.1802304	-0.7293713	0.9230769	1	1	495	tags=26%, list=26%, signal=34%
GOBP_REGULATION_OF_DEVELOPMENTAL_GROWTH		35	-0.2104582	-0.7282766	0.8545455	1	1	640	tags=43%, list=34%, signal=64%
GOBP_ORGANIC_HYDROXY_COMPOUND_METABOLIC_PROCESS		83	-0.1765596	-0.7271232	0.9137931	1	1	310	tags=19%, list=17%, signal=22%
GOBP_CHROMOSOME_ORGANIZATION		60	-0.1890401	-0.7269282	0.9245283	1	1	309	tags=18%, list=17%, signal=21%
GOBP_STEROL_METABOLIC_PROCESS		27	-0.2241446	-0.7241991	0.8833333	1	1	198	tags=19%, list=11%, signal=20%
GOBP_POSITIVE_REGULATION_OF_PROTEIN_CATABOLIC_PROCESS		42	-0.1881544	-0.7236089	0.92156863	1	1	330	tags=19%, list=18%, signal=23%
GOBP_ENDOTHELIUM_DEVELOPMENT		29	-0.2051549	-0.7230271	0.90384614	1	1	159	tags=14%, list=8%, signal=15%
GOBP_CANONICAL_WNT_SIGNALING_PATHWAY		47	-0.187555	-0.7229959	0.96153843	1	1	85	tags=9%, list=5%, signal=9%
GOBP_REGULATION_OF_CELL_DEVELOPMENT		73	-0.1758937	-0.7227621	0.983871	1	1	603	tags=37%, list=32%, signal=52%

REACTOME_SPHINGOLIPID_METABOLISM		14	-0.2477114	-0.7223274	0.8333333	1	1	1412	tags=100%, list=75%, signal=405%
GOBP_RESPONSE_TO_LIGHT_STIMULUS		24	-0.2323984	-0.7212655	0.90909094	1	1	450	tags=29%, list=24%, signal=38%
GOBP_REGULATION_OF_ADAPTIVE_IMMUNE_RESPONSE		15	-0.2410218	-0.7203243	0.8545455	1	1	316	tags=27%, list=17%, signal=32%
GOBP_POSITIVE_REGULATION_OF_INTRACELLULAR_PROTEIN_TRANSPORT		35	-0.222247	-0.720233	0.84615386	1	1	155	tags=14%, list=8%, signal=15%
REACTOME_RAB_REGULATION_OF_TRAFFICKING		24	-0.2241962	-0.7200037	0.91071427	1	1	479	tags=38%, list=26%, signal=50%
GOBP_CELLULAR_RESPONSE_TO_EXTRACELLULAR_STIMULUS		27	-0.2230694	-0.719337	0.9433962	1	1	236	tags=15%, list=13%, signal=17%
GOBP_GLIAL_CELL_DIFFERENTIATION		24	-0.2270095	-0.7191683	0.8545455	1	1	236	tags=17%, list=13%, signal=19%
REACTOME_DISEASES_OF_GLYCOSYLATION		17	-0.2495524	-0.7189651	0.9111111	1	1	686	tags=47%, list=37%, signal=74%
GOBP_GLYCOSYLATION		23	-0.2297231	-0.7183204	0.9245283	1	1	298	tags=13%, list=16%, signal=15%
GOBP_GRANULOCYTE_MIGRATION		24	-0.2228783	-0.7166369	0.8448276	1	1	227	tags=17%, list=12%, signal=19%
GOBP_PEPTIDYL_CYSTEINE_MODIFICATION		10	-0.2952709	-0.7164046	0.7755102	1	1	141	tags=20%, list=8%, signal=22%
GOBP_REGULATION_OF_MONONUCLEAR_CELL_MIGRATION		14	-0.2489187	-0.7158045	0.875	1	1	148	tags=14%, list=8%, signal=15%
REACTOME_CELLULAR_RESPONSE_TO_CHEMICAL_STRESS		68	-0.177365	-0.713836	0.91525424	1	1	273	tags=18%, list=15%, signal=20%
GOBP_RHO_PROTEIN_SIGNAL_TRANSDUCTION		24	-0.2341338	-0.7123845	0.9183673	1	1	198	tags=17%, list=11%, signal=18%
GOBP_RIBONUCLEOSIDE_MONOPHOSPHATE_METABOLIC_PROCESS		17	-0.2485784	-0.7082523	0.8269231	1	1	95	tags=12%, list=5%, signal=12%
GOBP_NEURAL_TUBE_DEVELOPMENT		11	-0.2759705	-0.7082419	0.8235294	1	1	292	tags=27%, list=16%, signal=32%
GOBP_AMINO_ACID_TRANSMEMBRANE_TRANSPORT		14	-0.2579714	-0.7063168	0.90384614	1	1	584	tags=43%, list=31%, signal=62%
GOBP_NEGATIVE_REGULATION_OF_CELL_PROJECTION_ORGANIZATION		25	-0.2199334	-0.7060093	0.9056604	1	1	179	tags=16%, list=10%, signal=17%
GOBP_ENDOPLASMIC_RETICULUM_TO_GOLGI_VESICLE_MEDIATED_TRANSP		55	-0.1858565	-0.7058568	0.98214287	1	1	500	tags=29%, list=27%, signal=39%
GOBP_POSITIVE_REGULATION_OF_PEPTIDE_SECRETION		27	-0.2203667	-0.7057674	0.90909094	1	1	420	tags=33%, list=22%, signal=42%
GOBP_CELLULAR_RESPONSE_TO_CALCIUM_ION		12	-0.261998	-0.7041031	0.8039216	1	1	23	tags=8%, list=1%, signal=8%
GOBP_ANATOMICAL_STRUCTURE_MATURATION		17	-0.2505521	-0.7023493	0.9148936	1	1	562	tags=47%, list=30%, signal=67%
GOBP_POSITIVE_REGULATION_OF_PROTEIN_POLYMERIZATION		31	-0.2069855	-0.7012195	0.9074074	1	1	572	tags=39%, list=31%, signal=55%
GOBP_REGULATION_OF_WNT_SIGNALING_PATHWAY		52	-0.1857075	-0.6998448	0.94736844	1	1	137	tags=10%, list=7%, signal=10%
GOBP_POSITIVE_REGULATION_OF_ADAPTIVE_IMMUNE_RESPONSE		10	-0.2803206	-0.697798	0.8363636	1	1	260	tags=30%, list=14%, signal=35%
GOBP_REGULATION_OF_MICROTUBULE_POLYMERIZATION_OR_DEPOLYMER		10	-0.2874413	-0.6961475	0.8367347	1	1	420	tags=30%, list=22%, signal=38%
GOBP_POSITIVE_REGULATION_OF_LAMELLIPODIUM_ASSEMBLY		12	-0.2554893	-0.695784	0.8545455	1	1	591	tags=58%, list=32%, signal=85%
GOBP_GENE_SILENCING		10	-0.2622246	-0.6944636	0.9032258	1	1	1384	tags=100%, list=74%, signal=382%
GOBP_POSITIVE_REGULATION_OF_CELL_MATRIX_ADHESION		16	-0.2455524	-0.6939817	0.92727274	1	1	627	tags=56%, list=34%, signal=84%
GOBP_CHROMATIN_ORGANIZATION		25	-0.2197604	-0.6938568	0.9411765	1	1	254	tags=16%, list=14%, signal=18%
GOBP_REGULATION_OF_CELL_ACTIVATION		59	-0.1775702	-0.6926634	0.9423077	1	1	260	tags=14%, list=14%, signal=15%
GOBP_POSITIVE_REGULATION_OF_PROTEASOMAL_UBIQUITIN_DEPENDENT_		14	-0.2553378	-0.6919956	0.8490566	1	1	137	tags=14%, list=7%, signal=15%
GOBP_POSITIVE_REGULATION_OF_CELL_JUNCTION_ASSEMBLY		14	-0.2527622	-0.6909367	0.93442625	1	1	627	tags=50%, list=34%, signal=75%
GOBP_NCRNA_METABOLIC_PROCESS		60	-0.1739071	-0.6887493	0.94827586	1	1	553	tags=33%, list=30%, signal=46%
GOBP_CELLULAR_PROTEIN_COMPLEX_DISASSEMBLY		97	-0.1664242	-0.6876494	0.98	1	1	552	tags=34%, list=30%, signal=46%
REACTOME_SIGNALING_BY_VEGF		29	-0.207825	-0.686987	0.9322034	1	1	627	tags=45%, list=34%, signal=66%
GOBP_RESPONSE_TO_METAL_ION		66	-0.1740398	-0.6849704	0.9830508	1	1	317	tags=18%, list=17%, signal=21%
REACTOME_ASSOCIATION_OF_TRIC_CCT_WITH_TARGET_PROTEINS_DURING		11	-0.2766028	-0.68471	0.9423077	1	1	128	tags=18%, list=7%, signal=19%
GOBP_STRESS_FIBER_ASSEMBLY		21	-0.213145	-0.6842366	0.9423077	1	1	627	tags=48%, list=34%, signal=71%
GOBP_PROTEIN_CONTAINING_COMPLEX_DISASSEMBLY		114	-0.1532975	-0.6831484	1	1	1	540	tags=31%, list=29%, signal=41%
REACTOME_ER_TO_GOLGI_ANTEROGRADE_TRANSPORT		49	-0.1852469	-0.6831367	0.91525424	1	1	495	tags=29%, list=26%, signal=38%
GOBP_TUBE_FORMATION		11	-0.2631417	-0.6828716	0.9	1	1	292	tags=27%, list=16%, signal=32%
REACTOME_INTRA_GOLGI_AND_RETROGRADE_GOLGI_TO_ER_TRAFFIC		47	-0.1890774	-0.6818647	0.96666664	1	1	495	tags=28%, list=26%, signal=37%
GOBP_NUCLEIC_ACID_PHOSPHODIESTER_BOND_HYDROLYSIS		14	-0.2520967	-0.679689	0.7962963	1	1	347	tags=29%, list=19%, signal=35%
REACTOME_MEMBRANE_TRAFFICKING		150	-0.1487281	-0.6789069	0.96666664	1	1	479	tags=25%, list=26%, signal=30%
GOBP_IMMUNE_RESPONSE_REGULATING_SIGNALING_PATHWAY		73	-0.1632919	-0.6787233	0.9677419	1	1	271	tags=14%, list=14%, signal=15%
GOBP_ORGANELLE_LOCALIZATION		112	-0.153587	-0.6775298	0.98507464	1	1	337	tags=18%, list=18%, signal=20%
GOBP_POSITIVE_REGULATION_OF_LEUKOCYTE_CHEMOTAXIS		11	-0.2650966	-0.6762649	0.82978725	1	1	501	tags=36%, list=27%, signal=49%
KEGG_LYSOSOME		24	-0.2036499	-0.6753856	0.93333334	1	1	15	tags=4%, list=1%, signal=4%
GOBP_LEUKOCYTE_MIGRATION		71	-0.1681636	-0.6739083	0.96875	1	1	451	tags=23%, list=24%, signal=29%
GOBP_MUSCLE_HYPERTROPHY		17	-0.2404631	-0.6737283	0.94827586	1	1	92	tags=12%, list=5%, signal=12%
GOBP_MONONUCLEAR_CELL_MIGRATION		24	-0.2152602	-0.6731073	0.94545454	1	1	148	tags=13%, list=8%, signal=13%
REACTOME_RHOBTB_GTPASE_CYCLE		16	-0.2263766	-0.6702469	0.94545454	1	1	366	tags=25%, list=20%, signal=31%
GOBP_IRON_ION_TRANSPORT		20	-0.2179906	-0.6698717	0.962963	1	1	101	tags=10%, list=5%, signal=10%
GOBP_REGULATION_OF_LEUKOCYTE_CHEMOTAXIS		14	-0.2548091	-0.6698679	0.8888889	1	1	501	tags=36%, list=27%, signal=48%
GOBP_CARBOXYLIC_ACID_TRANSPORT		32	-0.2004618	-0.668588	0.9285714	1	1	584	tags=41%, list=31%, signal=58%
REACTOME_CYTOPROTECTION_BY_HMOX1		53	-0.1741954	-0.6681309	0.94736844	1	1	412	tags=23%, list=22%, signal=28%

GOBP_CELL_GROWTH		64	-0.1704334	-0.6671212	0.96428573	1	1	330	tags=19%, list=18%, signal=22%
REACTOME_METABOLISM_OF_FAT_SOLUBLE_VITAMINS		10	-0.2647506	-0.666163	0.89361703	1	1	520	tags=40%, list=28%, signal=55%
REACTOME_MITOTIC_G2_G2_M_PHASES		51	-0.1759835	-0.6650001	0.9423077	1	1	238	tags=14%, list=13%, signal=15%
GOBP_RESPONSE_TO_RETINOIC_ACID		11	-0.2517988	-0.6647698	0.8627451	1	1	462	tags=36%, list=25%, signal=48%
GOBP_REGULATION_OF_MACROAUTOPHAGY		39	-0.195415	-0.6647598	0.9433962	1	1	234	tags=10%, list=13%, signal=11%
GOBP_POSITIVE_REGULATION_OF_UBIQUITIN_DEPENDENT_PROTEIN_CATAB		15	-0.2456781	-0.662566	0.9361702	1	1	137	tags=13%, list=7%, signal=14%
GOBP_REGULATION_OF_SYNAPTIC_PLASTICITY		16	-0.223267	-0.6623018	0.9464286	1	1	155	tags=13%, list=8%, signal=14%
GOBP_TRANSLATIONAL_ELONGATION		79	-0.160226	-0.6614468	1	1	1	552	tags=34%, list=30%, signal=46%
GOBP_DEVELOPMENTAL_MATURATION		23	-0.2112219	-0.6610594	0.91071427	1	1	502	tags=39%, list=27%, signal=53%
GOBP_REGULATION_OF_ERBB_SIGNALING_PATHWAY		16	-0.2176117	-0.658585	0.91071427	1	1	309	tags=19%, list=17%, signal=22%
GOBP_REGULATION_OF_CELL_CYCLE_G2_M_PHASE_TRANSITION		45	-0.1659762	-0.6585663	0.98039216	1	1	412	tags=20%, list=22%, signal=25%
GOBP_POSITIVE_REGULATION_OF_CELL_MORPHOGENESIS_INVOLVED_IN_D		26	-0.2040481	-0.6557288	0.8958333	1	1	317	tags=23%, list=17%, signal=27%
GOBP_POSITIVE_REGULATION_OF_RESPONSE_TO_BIOTIC_STIMULUS		51	-0.1842281	-0.6556565	0.98039216	1	1	243	tags=14%, list=13%, signal=15%
GOBP_BEHAVIOR		50	-0.1713331	-0.655157	0.9464286	1	1	369	tags=20%, list=20%, signal=24%
REACTOME_RHOBTB2_GTPASE_CYCLE		11	-0.2434337	-0.6535177	0.90697676	1	1	642	tags=55%, list=34%, signal=83%
REACTOME_FATTY_ACYL_COA_BIOSYNTHESIS		12	-0.2256485	-0.6504385	0.9122807	1	1	228	tags=17%, list=12%, signal=19%
GOBP_PROTEIN_PROCESSING		35	-0.1965203	-0.6502581	0.9056604	1	1	317	tags=20%, list=17%, signal=24%
GOBP_CELLULAR_RESPONSE_TO_DNA_DAMAGE_STIMULUS		49	-0.170705	-0.6494889	0.98	1	1	424	tags=16%, list=23%, signal=21%
REACTOME_MET_PROMOTES_CELL_MOTILITY		13	-0.2527317	-0.648959	0.9444444	1	1	442	tags=38%, list=24%, signal=50%
GOBP_POSITIVE_REGULATION_OF_SUBSTRATE_ADHESION_DEPENDENT_CE		21	-0.2050562	-0.6464133	0.89285713	1	1	317	tags=24%, list=17%, signal=28%
REACTOME_NEGATIVE_REGULATION_OF_THE_PI3K_AKT_NETWORK		11	-0.2619801	-0.6446471	0.88372093	1	1	420	tags=18%, list=22%, signal=23%
REACTOME_MITOPHAGY		12	-0.236942	-0.6353447	0.9347826	1	1	525	tags=42%, list=28%, signal=58%
GOBP_SUBSTRATE_ADHESION_DEPENDENT_CELL_SPREADING		36	-0.1833174	-0.6348457	0.94	1	1	344	tags=25%, list=18%, signal=30%
REACTOME_ECM_PROTEOGLYCANS		26	-0.1878834	-0.6301248	0.9574468	1	1	344	tags=23%, list=18%, signal=28%
GOBP_NUCLEOTIDE_EXCISION_REPAIR		10	-0.2590005	-0.6289024	0.9354839	1	1	1390	tags=100%, list=74%, signal=387%
REACTOME_EPH_EPHRIN_SIGNALING		29	-0.1965748	-0.6282305	0.9264706	1	1	269	tags=17%, list=14%, signal=20%
GOBP_EPIDERMAL_GROWTH_FACTOR_RECEPTOR_SIGNALING_PATHWAY		17	-0.2219817	-0.6276647	0.92727274	1	1	309	tags=18%, list=17%, signal=21%
GOBP_GLYCOPROTEIN_METABOLIC_PROCESS		38	-0.1754058	-0.6264097	1	1	1	400	tags=18%, list=21%, signal=23%
GOBP_RESPONSE_TO_AMYLOID_BETA		10	-0.2565812	-0.6263199	0.9375	1	1	560	tags=40%, list=30%, signal=57%
GOBP_VACUOLE_ORGANIZATION		31	-0.1851119	-0.6260293	0.9259259	1	1	96	tags=10%, list=5%, signal=10%
GOBP_POSITIVE_REGULATION_OF_LEUKOCYTE_MIGRATION		18	-0.2138616	-0.6244853	0.9259259	1	1	501	tags=28%, list=27%, signal=38%
KEGG_N_GLYCAN_BIOSYNTHESIS		13	-0.2379564	-0.6213511	0.93877554	1	1	557	tags=23%, list=30%, signal=33%
KEGG_INSULIN_SIGNALING_PATHWAY		26	-0.1892592	-0.6212921	0.9811321	1	1	562	tags=38%, list=30%, signal=54%
REACTOME_PHASE_II_CONJUGATION_OF_COMPOUNDS		14	-0.2137221	-0.6203366	0.9259259	1	1	364	tags=21%, list=19%, signal=26%
GOBP_POSITIVE_REGULATION_OF_CELL_SUBSTRATE_JUNCTION_ORGANIZA		12	-0.2257928	-0.6200709	0.98245615	1	1	627	tags=42%, list=34%, signal=62%
GOBP_TISSUE_REGENERATION		11	-0.248497	-0.6184195	0.9444444	1	1	260	tags=27%, list=14%, signal=31%
GOBP_REGULATION_OF_LYMPHOCYTE_MEDIATED_IMMUNITY		13	-0.2323372	-0.6177502	0.9183673	1	1	260	tags=23%, list=14%, signal=27%
GOBP_VENTRICULAR_CARDIAC_MUSCLE_CELL_ACTION_POTENTIAL		11	-0.2416246	-0.6152409	0.9791667	1	1	313	tags=27%, list=17%, signal=33%
GOBP_NEUROMUSCULAR_JUNCTION_DEVELOPMENT		14	-0.2316056	-0.6122829	0.9310345	1	1	519	tags=43%, list=28%, signal=59%
GOBP_POSITIVE_REGULATION_OF_LEUKOCYTE_MEDIATED_IMMUNITY		12	-0.227371	-0.6096636	0.95555556	1	1	260	tags=25%, list=14%, signal=29%
GOBP_VIRAL_GENOME_REPLICATION		20	-0.2027998	-0.608745	0.9607843	1	1	190	tags=15%, list=10%, signal=17%
GOBP_TRANSITION_METAL_ION_TRANSPORT		24	-0.1962097	-0.6078746	0.9433962	1	1	101	tags=8%, list=5%, signal=9%
REACTOME_DISORDERS_OF_TRANSMEMBRANE_TRANSPORTERS		42	-0.1609444	-0.605814	0.9811321	1	1	412	tags=21%, list=22%, signal=27%
GOBP_PROTEIN_POLYUBIQUITINATION		44	-0.1574343	-0.6056542	1	1	1	73	tags=7%, list=4%, signal=7%
GOBP_CELLULAR_RESPONSE_TO_AMYLOID_BETA		10	-0.2565812	-0.6026315	0.9607843	1	1	560	tags=40%, list=30%, signal=57%
GOBP_AGING		50	-0.1554124	-0.5992823	1	1	1	296	tags=14%, list=16%, signal=16%
GOBP_IRON_ION_HOMEOSTASIS		17	-0.1986358	-0.5984837	0.9814815	1	1	449	tags=29%, list=24%, signal=38%
REACTOME_IMMUNOREGULATORY_INTERACTIONS_BETWEEN_A_LYMPHOID_		14	-0.2276639	-0.5965914	0.9811321	1	1	584	tags=36%, list=31%, signal=52%
REACTOME_HEDGEHOG_OFF_STATE		43	-0.164899	-0.5953253	1	1	1	60	tags=5%, list=3%, signal=5%
GOBP_TETRAPYRROLE_METABOLIC_PROCESS		16	-0.2186483	-0.5922861	0.96	1	1	560	tags=44%, list=30%, signal=62%
GOBP_REGULATION_OF_OXIDATIVE_PHOSPHORYLATION		10	-0.2404593	-0.5922458	0.962963	1	1	528	tags=40%, list=28%, signal=55%
GOBP_T_CELL_ACTIVATION		50	-0.1595466	-0.5855163	1	1	1	260	tags=14%, list=14%, signal=16%
GOBP_CIRCADIAN_RHYTHM		18	-0.2000989	-0.5844978	0.9830508	1	1	450	tags=33%, list=24%, signal=43%
REACTOME_TOLL_LIKE_RECEPTOR_TLR1_TLR2_CASCADE		15	-0.2139821	-0.5795921	0.9811321	1	1	317	tags=20%, list=17%, signal=24%
GOBP_POSITIVE_REGULATION_OF_TRANSLATION		29	-0.1601498	-0.5785415	1	1	1	267	tags=14%, list=14%, signal=16%
GOBP_ADULT_LOCOMOTORY_BEHAVIOR		10	-0.2207878	-0.5778418	0.9818182	1	1	325	tags=20%, list=17%, signal=24%
GOBP_REGULATION_OF_CELL_CYCLE		115	-0.1254713	-0.5764957	1	1	1	419	tags=19%, list=22%, signal=23%

GOBP_REGULATION_OF_CARBOHYDRATE_METABOLIC_PROCESS		24	-0.1817848	-0.5743405	1	1	1	537	tags=38%, list=29%, signal=52%
REACTOME_SIGNALING_BY_MET		20	-0.1834492	-0.5678517	0.962963	1	1	442	tags=25%, list=24%, signal=32%
GOBP_ACTIVATION_OF_INNATE_IMMUNE_RESPONSE		43	-0.1539101	-0.5639285	1	1	1	238	tags=12%, list=13%, signal=13%
GOBP_REGULATION_OF_DNA_TEMPLATED_TRANSCRIPTION_IN_RESPONSE_		36	-0.1587312	-0.5619554	0.96153843	1	1	60	tags=6%, list=3%, signal=6%
GOBP_REGULATION_OF_SIGNAL_TRANSDUCTION_BY_P53_CLASS_MEDIATO		11	-0.2258065	-0.5615	0.9148936	1	1	1452	tags=100%, list=78%, signal=444%
REACTOME_TRANSCRIPTIONAL_REGULATION_BY_RUNX3		33	-0.155402	-0.5585786	1	1	1	60	tags=6%, list=3%, signal=6%
REACTOME_HEDGEHOG_LIGAND_BIOGENESIS		33	-0.1539613	-0.5575998	0.9807692	1	1	238	tags=12%, list=13%, signal=14%
REACTOME_NUCLEAR_ENVELOPE_NE_REASSEMBLY		14	-0.2019671	-0.5563579	0.97959185	1	1	653	tags=43%, list=35%, signal=65%
GOBP_ADULT_BEHAVIOR		11	-0.2084128	-0.5562828	0.96363634	1	1	325	tags=18%, list=17%, signal=22%
GOBP_POSITIVE_REGULATION_OF_CELL_CYCLE_PROCESS		20	-0.1679896	-0.5536044	0.98	1	1	613	tags=40%, list=33%, signal=59%
GOBP_PROTEIN_POLYMERIZATION		63	-0.1416176	-0.5480242	1	1	1	465	tags=24%, list=25%, signal=31%
GOBP_PROTEIN_INSERTION_INTO_MITOCHONDRIAL_INNER_MEMBRANE		10	-0.2038322	-0.5475273	1	1	1	630	tags=50%, list=34%, signal=75%
GOBP_POSITIVE_REGULATION_OF_LAMELLIPODIUM_ORGANIZATION		14	-0.1921358	-0.5472691	0.96428573	1	1	591	tags=50%, list=32%, signal=73%
GOBP_REGULATION_OF_MICROTUBULE_CYTOSKELETON_ORGANIZATION		17	-0.1770176	-0.5454027	1	1	1	420	tags=24%, list=22%, signal=30%
GOBP_MEMBRANE_LIPID_METABOLIC_PROCESS		24	-0.165546	-0.5438022	1	1	1	1416	tags=96%, list=76%, signal=389%
GOBP_ANTIGEN_RECEPTOR_MEDIATED_SIGNALING_PATHWAY		46	-0.1476341	-0.5417809	1	1	1	260	tags=11%, list=14%, signal=12%
REACTOME_METABOLISM_OF_NUCLEOTIDES		24	-0.1733128	-0.5416182	1	1	1	95	tags=8%, list=5%, signal=9%
GOBP_REGULATION_OF_EXTENT_OF_CELL_GROWTH		19	-0.1777545	-0.5411255	0.98	1	1	627	tags=42%, list=34%, signal=63%
GOBP_REGULATION_OF_PROTEIN_POLYMERIZATION		53	-0.1424004	-0.540909	1	1	1	607	tags=34%, list=32%, signal=49%
GOBP_RETROGRADE_VESICLE_MEDIATED_TRANSPORT_GOLGI_TO_ENDOPL		22	-0.1704737	-0.5385947	1	1	1	285	tags=18%, list=15%, signal=21%
GOBP_SPHINGOLIPID_METABOLIC_PROCESS		22	-0.1816244	-0.537841	1	1	1	599	tags=32%, list=32%, signal=46%
GOBP_POSITIVE_REGULATION_OF_DEFENSE_RESPONSE		64	-0.1333966	-0.5365708	1	1	1	260	tags=13%, list=14%, signal=14%
GOBP_LEUKOCYTE_CHEMOTAXIS		30	-0.1495667	-0.5328324	0.96	1	1	501	tags=27%, list=27%, signal=36%
REACTOME_RHO_GTPASES_ACTIVATE_WASPS_AND_WAVES		14	-0.1930477	-0.5326774	1	1	1	641	tags=50%, list=34%, signal=75%
REACTOME_M_PHASE		69	-0.1295466	-0.5322013	1	1	1	655	tags=36%, list=35%, signal=54%
GOBP_REGULATION_OF_FILOPODIUM_ASSEMBLY		13	-0.1910657	-0.5313758	0.9818182	1	1	1517	tags=100%, list=81%, signal=525%
GOBP_REGULATION_OF_CELL_CYCLE_PROCESS		83	-0.1265063	-0.5289608	1	1	1	419	tags=19%, list=22%, signal=24%
REACTOME_DEGRADATION_OF_BETA_CATENIN_BY_THE_DESTRUCTION_COM		38	-0.1462399	-0.5271398	1	1	1	146	tags=8%, list=8%, signal=8%
GOBP_TUMOR_NECROSIS_FACTOR_MEDIATED_SIGNALING_PATHWAY		40	-0.1417764	-0.519613	1	1	1	448	tags=23%, list=24%, signal=29%
GOBP_REGULATION_OF_CELL_SUBSTRATE_JUNCTION_ORGANIZATION		22	-0.1606156	-0.5129936	0.98	1	1	150	tags=9%, list=8%, signal=10%
GOBP_PEPTIDE_CATABOLIC_PROCESS		11	-0.2085418	-0.5114431	0.9649123	1	1	664	tags=55%, list=35%, signal=84%
REACTOME_EPHB_MEDIATED_FORWARD_SIGNALING		16	-0.1765842	-0.5061988	0.9583333	1	1	641	tags=44%, list=34%, signal=66%
GOBP_CELL_CYCLE_G2_M_PHASE_TRANSITION		50	-0.1357805	-0.5049471	1	1	1	412	tags=18%, list=22%, signal=22%
GOBP_REGULATION_OF_LEUKOCYTE_MEDIATED_IMMUNITY		19	-0.1697779	-0.5043681	0.98275864	1	1	449	tags=26%, list=24%, signal=34%
GOBP_REGULATION_OF_MITOTIC_CELL_CYCLE		74	-0.120572	-0.5033555	0.98333335	1	1	337	tags=14%, list=18%, signal=16%
GOBP_CARDIOLIPIN_METABOLIC_PROCESS		12	-0.1961488	-0.4992171	0.9791667	1	1	373	tags=25%, list=20%, signal=31%
REACTOME_MITOTIC_METAPHASE_AND_ANAPHASE		55	-0.1283316	-0.4947883	1	1	1	655	tags=36%, list=35%, signal=54%
GOBP_CELLULAR_PROCESS_INVOLVED_IN_REPRODUCTION_IN_MULTICELLU		11	-0.1915587	-0.4869407	0.98	1	1	562	tags=36%, list=30%, signal=52%
GOBP_RESPONSE_TO_TUMOR_NECROSIS_FACTOR		53	-0.1290511	-0.4831701	1	1	1	238	tags=11%, list=13%, signal=13%
GOBP_TERPENOID_METABOLIC_PROCESS		18	-0.1601507	-0.4778411	0.9818182	1	1	520	tags=33%, list=28%, signal=46%
GOBP_CELLULAR_COMPONENT_MAINTENANCE		10	-0.188319	-0.4634432	1	1	1	459	tags=30%, list=25%, signal=40%
GOBP_REGULATION_OF_LAMELLIPODIUM_ASSEMBLY		15	-0.1685263	-0.4561499	1	1	1	591	tags=47%, list=32%, signal=68%
GOBP_REGULATION_OF_CELL_CYCLE_PHASE_TRANSITION		61	-0.1104882	-0.4436048	1	1	1	412	tags=16%, list=22%, signal=20%
GOBP_ALPHA_BETA_T_CELL_ACTIVATION		11	-0.1596774	-0.4427097	1	1	1	1575	tags=100%, list=84%, signal=628%
REACTOME_SEPARATION_OF_SISTER_CHROMATIDS		46	-0.1177372	-0.430863	1	1	1	261	tags=11%, list=14%, signal=12%
GOBP_PROTEIN_DNA_COMPLEX_SUBUNIT_ORGANIZATION		18	-0.1328909	-0.3806517	1	0.9998131	1	1390	tags=94%, list=74%, signal=364%

Supplementary Table 7d. Gene-sets enriched in the 4-month-old GA muscle samples									
GS<br>follow link to MSigDB	GS DETAILS	SIZE	ES	NES	NOM p-val	FDR q-val	FWER p-val	RANK AT MAX	LEADING EDGE
GOBP_ATP_SYNTHESIS_COUPLED_ELECTRON_TRANSPORT	Details ...	63	0.47930884	3.803851	0	0	0	145	tags=33%, list=9%, signal=35%
GOBP_RESPIRATORY_ELECTRON_TRANSPORT_CHAIN	Details ...	71	0.4358229	3.6842196	0	0	0	145	tags=31%, list=9%, signal=32%
GOBP_MITOCHONDRIAL_ELECTRON_TRANSPORT_NADH_TO_UB	Details ...	39	0.40289062	2.8454015	0	0	0	166	tags=31%, list=10%, signal=33%
KEGG_CARDIAC_MUSCLE_CONTRACTION	Details ...	35	0.5367643	2.6643112	0	0	0	145	tags=37%, list=9%, signal=40%
GOBP_REGULATION_OF_CARDIAC_CONDUCTION	Details ...	19	0.53463143	2.615916	0	0	0	191	tags=58%, list=11%, signal=65%
REACTOME_CARDIAC_CONDUCTION	Details ...	28	0.40654644	2.545328	0	0	0	191	tags=43%, list=11%, signal=48%
KEGG_HUNTINGTONS_DISEASE	Details ...	84	0.42181298	2.5336585	0	0	0	168	tags=37%, list=10%, signal=39%
GOBP_PROTON_TRANSMEMBRANE_TRANSPORT	Details ...	49	0.32755536	2.3650398	0	0	0	168	tags=35%, list=10%, signal=37%
GOBP_MITOCHONDRIAL_ELECTRON_TRANSPORT_UBIQUINOL_T	Details ...	10	0.71461666	2.185764	0	0.00334186	0.02	145	tags=50%, list=9%, signal=54%
REACTOME_MITOCHONDRIAL_CALCIUM_ION_TRANSPORT	Details ...	18	0.487632	2.135845	0	0.00300767	0.02	139	tags=44%, list=8%, signal=48%
GOBP_MITOCHONDRIAL_ELECTRON_TRANSPORT_CYTOCHROMI	Details ...	10	0.5948924	2.1078653	0	0.00273425	0.02	133	tags=40%, list=8%, signal=43%
REACTOME_ION_HOMEOSTASIS	Details ...	23	0.40914413	2.026274	0	0.00615903	0.05	191	tags=48%, list=11%, signal=53%
REACTOME_COMPLEX_I_BIOGENESIS	Details ...	46	0.32998535	1.9868805	0	0.00781671	0.07	208	tags=33%, list=12%, signal=36%
GOBP_LYTIC_VACUOLE_ORGANIZATION	Details ...	11	0.5006805	1.8968738	0	0.01599774	0.16	157	tags=36%, list=9%, signal=40%
GOBP_RESPIRATORY_CHAIN_COMPLEX_IV_ASSEMBLY	Details ...	12	0.491946	1.8953323	0	0.01591123	0.16	117	tags=33%, list=7%, signal=36%
GOBP_CYTOCHROME_COMPLEX_ASSEMBLY	Details ...	16	0.55094653	1.8388368	0	0.01983268	0.2	117	tags=38%, list=7%, signal=40%
KEGG_LYSINE_DEGRADATION	Details ...	12	0.43850267	1.8160747	0	0.02806058	0.29	956	tags=100%, list=56%, signal=228%
GOBP_NEGATIVE_REGULATION_OF_LIPID_BIOSYNTHETIC_PRO	Details ...	11	0.45486942	1.801959	0.07692308	0.03143262	0.34	928	tags=100%, list=55%, signal=220%
GOBP_REGULATION_OF_CARDIAC_MUSCLE_CONTRACTION_BY_	Details ...	12	0.48187748	1.7999	0	0.03055196	0.35	883	tags=100%, list=52%, signal=207%
GOBP_MITOCHONDRIAL_CALCIUM_ION_TRANSMEMBRANE_TRAI	Details ...	17	0.40181592	1.7791303	0	0.03595403	0.38	139	tags=35%, list=8%, signal=38%
GOBP_CRISTAE_FORMATION	Details ...	25	0.3259802	1.7633604	0	0.03697145	0.41	179	tags=32%, list=11%, signal=35%
GOBP_ACETYL_COA_BIOSYNTHETIC_PROCESS	Details ...	14	0.39678764	1.7462026	0	0.03922745	0.46	1027	tags=100%, list=61%, signal=252%
GOBP_MITOCHONDRIAL_CYTOCHROME_C_OXIDASE_ASSEMBLY	Details ...	12	0.491946	1.7448982	0	0.03752191	0.46	117	tags=33%, list=7%, signal=36%
GOBP_CARBOXYLIC_ACID_TRANSPORT	Details ...	30	0.2406935	1.7437979	0	0.0359585	0.46	131	tags=23%, list=8%, signal=25%
GOBP_INNER_MITOCHONDRIAL_MEMBRANE_ORGANIZATION	Details ...	41	0.33212605	1.732142	0	0.03855705	0.5	220	tags=37%, list=13%, signal=41%
REACTOME_CRISTAE_FORMATION	Details ...	25	0.3349687	1.7304473	0	0.03762838	0.51	220	tags=40%, list=13%, signal=45%
GOBP_INORGANIC_ANION_TRANSPORT	Details ...	16	0.34147123	1.7287722	0	0.03623474	0.51	149	tags=44%, list=9%, signal=48%
KEGG_CALCIUM_SIGNALING_PATHWAY	Details ...	32	0.29822505	1.66177	0	0.05049335	0.64	205	tags=44%, list=12%, signal=49%
REACTOME_CITRIC_ACID_CYCLE_TCA_CYCLE	Details ...	21	0.37570775	1.6530752	0	0.05087689	0.65	836	tags=95%, list=49%, signal=186%
GOBP_FATTY_ACYL_COA_METABOLIC_PROCESS	Details ...	14	0.46103513	1.652374	0.09090909	0.04964326	0.66	919	tags=100%, list=54%, signal=217%
GOBP_ENDOPLASMIC_RETICULUM_ORGANIZATION	Details ...	32	0.25040329	1.64729	0	0.05066676	0.68	99	tags=19%, list=6%, signal=20%
REACTOME_GLYOXYLATE_METABOLISM_AND_GLYCINE_DEGRA	Details ...	14	0.39916715	1.6468773	0	0.04908342	0.68	1023	tags=100%, list=60%, signal=250%
GOBP_ACETYL_COA_METABOLIC_PROCESS	Details ...	15	0.3970238	1.6406022	0	0.05014619	0.69	1027	tags=100%, list=61%, signal=251%
GOBP_NADH_DEHYDROGENASE_COMPLEX_ASSEMBLY	Details ...	51	0.2842781	1.6297717	0	0.05234476	0.69	208	tags=29%, list=12%, signal=33%
REACTOME_FATTY_ACYL_COA_BIOSYNTHESIS	Details ...	13	0.46076098	1.6238904	0	0.05369078	0.72	919	tags=100%, list=54%, signal=217%
GOBP_POSITIVE_REGULATION_OF_CALCIUM_ION_TRANSMEMB	Details ...	12	0.4278434	1.582763	0	0.06573678	0.79	206	tags=42%, list=12%, signal=47%
GOBP_CARDIAC_CONDUCTION	Details ...	33	0.32658577	1.5800825	0	0.06475628	0.79	206	tags=42%, list=12%, signal=47%
REACTOME_MITOCHONDRIAL_BIOGENESIS	Details ...	34	0.2744502	1.5780212	0	0.06345513	0.8	220	tags=35%, list=13%, signal=40%
GOBP_FATTY_ACYL_COA_BIOSYNTHETIC_PROCESS	Details ...	13	0.46076098	1.5671624	0	0.06566897	0.83	919	tags=100%, list=54%, signal=217%
REACTOME_PROCESSING_OF_SMDT1	Details ...	14	0.36550766	1.5597248	0	0.06659589	0.83	139	tags=36%, list=8%, signal=39%
GOBP_REGULATION_OF_CARDIAC_MUSCLE_CONTRACTION_BY_	Details ...	10	0.48130563	1.5288721	0	0.079617	0.86	883	tags=100%, list=52%, signal=208%
GOBP_AEROBIC_RESPIRATION	Details ...	53	0.29694724	1.5150951	0	0.08600482	0.89	180	tags=26%, list=11%, signal=29%
GOBP_ESTABLISHMENT_OF_PROTEIN_LOCALIZATION_TO_MITO	Details ...	25	0.24823408	1.4977963	0	0.09485717	0.93	140	tags=28%, list=8%, signal=30%
GOBP_IMPORT_ACROSS_PLASMA_MEMBRANE	Details ...	20	0.36119404	1.4940344	0	0.09574927	0.93	1089	tags=100%, list=64%, signal=276%
GOBP_INORGANIC_ION_IMPORT_ACROSS_PLASMA_MEMBRANE	Details ...	12	0.4291711	1.4895729	0.14285715	0.09864324	0.95	206	tags=42%, list=12%, signal=47%
GOBP_POSITIVE_REGULATION_OF_LIPID_LOCALIZATION	Details ...	20	0.24300306	1.4865276	0	0.09813391	0.95	42	tags=20%, list=2%, signal=20%
GOBP_THIOESTER_BIOSYNTHETIC_PROCESS	Details ...	27	0.39988002	1.471599	0	0.10542164	0.95	1027	tags=100%, list=61%, signal=250%
GOBP_MITOCHONDRIAL_ATP_SYNTHESIS_COUPLED_PROTON_T	Details ...	16	0.34950447	1.4689913	0.07692308	0.10538875	0.95	168	tags=31%, list=10%, signal=34%
KEGG_CITRATE_CYCLE_TCA_CYCLE	Details ...	21	0.2764131	1.4679843	0	0.10445093	0.95	836	tags=95%, list=49%, signal=186%
REACTOME_FORMATION_OF_ATP_BY_CHEMIOSMOTIC_COUPLIN	Details ...	14	0.3838533	1.4476854	0.07692308	0.11330967	0.95	168	tags=36%, list=10%, signal=39%
GOBP_REGULATION_OF_CALCIUM_ION_TRANSPORT_INTO_CYT	Details ...	21	0.24555314	1.4332529	0	0.12186595	0.97	132	tags=19%, list=8%, signal=20%
GOBP_ORGANIC_ANION_TRANSPORT	Details ...	43	0.19526641	1.4182143	0	0.13099375	0.97	167	tags=26%, list=10%, signal=28%
GOBP_MITOCHONDRIAL_TRANSMEMBRANE_TRANSPORT	Details ...	56	0.24936154	1.4094894	0	0.13654168	0.97	168	tags=25%, list=10%, signal=27%
KEGG_BUTANOATE_METABOLISM	Details ...	15	0.31190485	1.4029676	0	0.13959157	0.98	1170	tags=100%, list=69%, signal=320%

GOBP_AMINO_ACID_TRANSMEMBRANE_TRANSPORT	Details ...	10	0.35905036	1.3671198	0.14285715	0.1707994	1	1089	tags=100%, list=64%, signal=278%
GOBP_NUCLEOSIDE_BISPHOSPHATE_BIOSYNTHETIC_PROCESS	Details ...	27	0.39988002	1.342383	0	0.19362685	1	1027	tags=100%, list=61%, signal=250%
GOBP_POSITIVE_REGULATION_OF_CATION_CHANNEL_ACTIVITY	Details ...	15	0.35261753	1.3244796	0.125	0.21572599	1	68	tags=20%, list=4%, signal=21%
REACTOME_REGULATION_OF_PYRUVATE_DEHYDROGENASE_P	Details ...	10	0.39584577	1.3221352	0.15384616	0.21531175	1	1027	tags=100%, list=61%, signal=252%
REACTOME_MITOCHONDRIAL_PROTEIN_IMPORT	Details ...	41	0.24558197	1.316687	0	0.21779343	1	155	tags=24%, list=9%, signal=26%
GOBP_NEGATIVE_REGULATION_OF_SMALL_MOLECULE_METAB	Details ...	11	0.44655585	1.3134577	0	0.21785194	1	942	tags=100%, list=56%, signal=224%
GOBP_FATTY_ACID_DERIVATIVE_BIOSYNTHETIC_PROCESS	Details ...	18	0.31533387	1.3103487	0.2	0.21819589	1	924	tags=94%, list=55%, signal=205%
GOBP_REGULATION_OF_RELEASE_OF_SEQUESTERED_CALCIU	Details ...	19	0.28282192	1.3097	0	0.21604416	1	132	tags=21%, list=8%, signal=23%
GOBP_MULTICELLULAR_ORGANISMAL_SIGNALING	Details ...	39	0.28481963	1.3096462	0	0.21261488	1	206	tags=38%, list=12%, signal=43%
KEGG_TRYPTOPHAN_METABOLISM	Details ...	13	0.31866825	1.2917767	0.14285715	0.2321285	1	1158	tags=100%, list=68%, signal=313%
GOBP_DICARBOXYLIC_ACID_TRANSPORT	Details ...	12	0.36143562	1.2830039	0.125	0.24098915	1	93	tags=33%, list=5%, signal=35%
GOBP_ATP_SYNTHESIS_COUPLED_PROTON_TRANSPORT	Details ...	17	0.28850266	1.282589	0.1	0.23842356	1	103	tags=24%, list=6%, signal=25%
GOBP_APOPTOTIC_MITOCHONDRIAL_CHANGES	Details ...	31	0.23509248	1.2803384	0	0.2384515	1	180	tags=32%, list=11%, signal=35%
GOBP_FATTY_ACID_DERIVATIVE_METABOLIC_PROCESS	Details ...	21	0.26207337	1.2286998	0.25	0.31948137	1	924	tags=90%, list=55%, signal=196%
REACTOME_PINK1_PRKN_MEDIATED_MITOPHAGY	Details ...	10	0.3371286	1.2233394	0.08333334	0.32352537	1	95	tags=30%, list=6%, signal=32%
GOBP_AUTOPHAGY_OF_MITOCHONDRION	Details ...	15	0.34152466	1.2202711	0.25	0.32493687	1	210	tags=47%, list=12%, signal=53%
GOBP_POSITIVE_REGULATION_OF_CALCIUM_ION_TRANSMEMB	Details ...	14	0.30311587	1.2093353	0.16666667	0.33698305	1	206	tags=36%, list=12%, signal=40%
GOBP_FATTY_ACID_TRANSMEMBRANE_TRANSPORT	Details ...	13	0.31926274	1.2057667	0.21428572	0.33682474	1	1157	tags=100%, list=68%, signal=313%
GOBP_ORGANELLE_TRANSPORT_ALONG_MICROTUBULE	Details ...	15	0.30464068	1.2053069	0.11111111	0.33375812	1	90	tags=27%, list=5%, signal=28%
GOBP_ACETYL_COA_BIOSYNTHETIC_PROCESS_FROM_PYRUVA	Details ...	11	0.39608073	1.2028972	0.2	0.33304247	1	1027	tags=100%, list=61%, signal=252%
GOBP_ORGANIC_ACID_TRANSMEMBRANE_TRANSPORT	Details ...	27	0.25687495	1.1959066	0.33333334	0.34136125	1	237	tags=37%, list=14%, signal=42%
REACTOME_MITOPHAGY	Details ...	12	0.2520555	1.1763314	0.27272728	0.37432146	1	95	tags=25%, list=6%, signal=26%
KEGG_ADIPOCYTOKINE_SIGNALING_PATHWAY	Details ...	10	0.31869435	1.1683023	0.2	0.38485605	1	1157	tags=100%, list=68%, signal=313%
KEGG_FATTY_ACID_METABOLISM	Details ...	26	0.2318753	1.1623042	0.33333334	0.39237565	1	1307	tags=100%, list=77%, signal=430%
GOBP_CYTOSOLIC_CALCIUM_ION_TRANSPORT	Details ...	38	0.23879828	1.1564817	0	0.39864296	1	139	tags=26%, list=8%, signal=28%
GOBP_TRANSPORT_ALONG_MICROTUBULE	Details ...	24	0.20227289	1.1486285	0.25	0.41243947	1	106	tags=21%, list=6%, signal=22%
GOBP_REGULATION_OF_PATTERN_RECOGNITION_RECEPTOR_S	Details ...	11	0.31425083	1.1452328	0.33333334	0.415664	1	146	tags=36%, list=9%, signal=40%
GOBP_ORGANELLE_DISASSEMBLY	Details ...	20	0.2568339	1.1391373	0.125	0.4251643	1	180	tags=35%, list=11%, signal=39%
GOBP_VERY_LONG_CHAIN_FATTY_ACID_METABOLIC_PROCESS	Details ...	10	0.3305638	1.1345748	0.2777778	0.42923623	1	1137	tags=100%, list=67%, signal=302%
GOBP_PROTEIN_TARGETING_TO_MITOCHONDRION	Details ...	39	0.19102891	1.1336876	0	0.42607307	1	155	tags=23%, list=9%, signal=25%
GOBP_PROTEIN_LOCALIZATION_TO_MITOCHONDRION	Details ...	56	0.18696404	1.1175876	0	0.45701563	1	155	tags=23%, list=9%, signal=25%
GOBP_IRON_SULFUR_CLUSTER_ASSEMBLY	Details ...	11	0.3218527	1.1082262	0.30769232	0.47328228	1	1152	tags=100%, list=68%, signal=310%
REACTOME_BRANCHED_CHAIN_AMINO_ACID_CATABOLISM	Details ...	18	0.24854398	1.1060518	0.14285715	0.472038	1	1003	tags=94%, list=59%, signal=229%
GOBP_LYSOSOME_LOCALIZATION	Details ...	10	0.29690388	1.1038644	0.3846154	0.47307163	1	38	tags=20%, list=2%, signal=20%
GOBP_REGULATION_OF_CALCIUM_ION_TRANSMEMBRANE_TRA	Details ...	34	0.19407967	1.1033535	0	0.46906263	1	148	tags=21%, list=9%, signal=22%
REACTOME_MITOCHONDRIAL_TRNA_AMINOACYLATION	Details ...	11	0.3028503	1.0890998	0.46153846	0.49927083	1	1184	tags=100%, list=70%, signal=330%
GOBP_REGULATION_OF_RYANODINE_SENSITIVE_CALCIUM_REL	Details ...	13	0.30110186	1.0728576	0.375	0.53675765	1	132	tags=23%, list=8%, signal=25%
GOBP_VASCULAR_TRANSPORT	Details ...	15	0.2711638	1.0546707	0.375	0.571055	1	131	tags=33%, list=8%, signal=36%
GOBP_MONOCARBOXYLIC_ACID_TRANSPORT	Details ...	20	0.24008149	1.0156173	0.2	0.6708249	1	167	tags=35%, list=10%, signal=38%
GOBP_POSITIVE_REGULATION_OF_TRANSPORTER_ACTIVITY	Details ...	24	0.19933094	0.9979423	0.44444445	0.7383368	1	68	tags=13%, list=4%, signal=13%
GOBP_REGULATION_OF_THE_FORCE_OF_HEART_CONTRACTIO	Details ...	10	0.32166177	0.9955188	0.375	0.7368614	1	1152	tags=100%, list=68%, signal=310%
GOBP_REGULATION_OF_ORGANIC_ACID_TRANSPORT	Details ...	10	0.28356695	0.99419755	0.5	0.7326174	1	42	tags=20%, list=2%, signal=20%
KEGG_HYPERTROPHIC_CARDIOMYOPATHY_HCM	Details ...	30	0.1873031	0.9827098	0.5	0.7494985	1	127	tags=23%, list=7%, signal=25%
GOBP_FATTY_ACID_BIOSYNTHETIC_PROCESS	Details ...	41	0.1588157	0.9531221	1	0.8230199	1	1157	tags=93%, list=68%, signal=285%
GOBP_REGULATION_OF_LIPID_LOCALIZATION	Details ...	32	0.20531759	0.9517762	1	0.8194652	1	42	tags=16%, list=2%, signal=16%
GOBP_UBIQUINONE_METABOLIC_PROCESS	Details ...	10	0.24925812	0.93932885	0.5833333	0.8472865	1	1274	tags=100%, list=75%, signal=400%
GOBP_PROTEIN_LIPID_COMPLEX_SUBUNIT_ORGANIZATION		13	0.23127231	0.92372656	0.6	0.8786102	1	1305	tags=100%, list=77%, signal=431%
GOBP_CALCIUM_ION_TRANSPORT_INTO_CYTOSOL		26	0.17502117	0.92229027	0.25	0.8731546	1	132	tags=19%, list=8%, signal=21%
GOBP_WATER_SOLUBLE_VITAMIN_METABOLIC_PROCESS		17	0.26287133	0.9188754	0.8	0.8727486	1	855	tags=94%, list=50%, signal=188%
GOBP_PROTEIN_N_LINKED_GLYCOSYLATION		11	0.26900235	0.9128355	0.5714286	0.8776013	1	1241	tags=100%, list=73%, signal=371%
GOBP_BRANCHED_CHAIN_AMINO_ACID_METABOLIC_PROCESS		18	0.24269532	0.9078844	0.5	0.8827101	1	1287	tags=100%, list=76%, signal=411%
GOBP_AMYLOID_PRECURSOR_PROTEIN_CATABOLIC_PROCESS		12	0.2389032	0.9075487	0.75	0.87464374	1	135	tags=25%, list=8%, signal=27%
GOBP_POSITIVE_REGULATION_OF_CALCIUM_ION_TRANSPORT		20	0.19641794	0.9073317	0.8	0.86728776	1	1365	tags=100%, list=81%, signal=508%
GOBP_CELL_COMMUNICATION_BY_ELECTRICAL_COUPLING_INVOLVED_IN_C		11	0.21615186	0.8924244	0.7777778	0.898762	1	1330	tags=100%, list=78%, signal=461%
REACTOME_SLC_MEDIATED_TRANSMEMBRANE_TRANSPORT		19	0.20955881	0.8917699	0.6	0.89092636	1	131	tags=21%, list=8%, signal=23%
GOBP_REGULATION_OF_STRIATED_MUSCLE_CONTRACTION		27	0.15786836	0.88154227	0.6	0.907349	1	148	tags=19%, list=9%, signal=20%
GOBP_LIPOPROTEIN_BIOSYNTHETIC_PROCESS		11	0.25475055	0.8733558	0.5882353	0.9181301	1	1265	tags=100%, list=75%, signal=392%

GOBP_ACIDIC_AMINO_ACID_TRANSPORT		10	0.27165827	0.8710441	0.8	0.9152105	1	93	tags=30%, list=5%, signal=32%
REACTOME_ION_TRANSPORT_BY_P_TYPE_ATPASES		17	0.2169249	0.8691298	0.75	0.9119001	1	1330	tags=100%, list=78%, signal=460%
GOBP_PROTEIN_TRANSMEMBRANE_IMPORT_INTO_INTRACELLULAR_ORGAN		16	0.22229506	0.8664015	0.8181818	0.90932786	1	784	tags=88%, list=46%, signal=161%
GOBP_AMINO_ACID_TRANSPORT		15	0.22458029	0.8523659	0.6666667	0.9306255	1	93	tags=20%, list=5%, signal=21%
GOBP_LIPID_STORAGE		14	0.21885769	0.84127444	0.7777778	0.9454425	1	157	tags=29%, list=9%, signal=31%
GOBP_POSITIVE_REGULATION_OF_CATION_TRANSMEMBRANE_TRANSPORT		28	0.15912427	0.8392849	0.6666667	0.9425306	1	68	tags=11%, list=4%, signal=11%
REACTOME_PYRUVATE_METABOLISM_AND_CITRIC_ACID_TCA_CYCLE		42	0.16245352	0.83178884	1	0.9493318	1	1027	tags=90%, list=61%, signal=224%
GOBP_PHOSPHATIDYLINOSITOL_METABOLIC_PROCESS		12	0.21806306	0.82693255	0.54545456	0.95027137	1	1327	tags=100%, list=78%, signal=457%
GOBP_REGULATION_OF_AMYLOID_PRECURSOR_PROTEIN_CATABOLIC_PRO		11	0.2467343	0.8213635	0.8333333	0.9521136	1	135	tags=27%, list=8%, signal=29%
REACTOME_SIGNALING_BY_RETINOIC_ACID		11	0.22446561	0.81904316	0.78571427	0.9479822	1	1316	tags=100%, list=78%, signal=444%
GOBP_MICROTUBULE_BASED_TRANSPORT		24	0.20227289	0.817305	0.6666667	0.9437232	1	106	tags=21%, list=6%, signal=22%
GOBP_PROTEIN_LIPID_COMPLEX_ASSEMBLY		11	0.2309976	0.816244	0.61538464	0.9376511	1	1305	tags=100%, list=77%, signal=432%
GOBP_ENDOPLASMIC_RETICULUM_CALCIUM_ION_HOMEOSTASIS		10	0.23211205	0.8085417	0.9444444	0.94866264	1	148	tags=30%, list=9%, signal=33%
GOBP_REGULATION_OF_CATION_CHANNEL_ACTIVITY		36	0.18853915	0.805995	1	0.9464002	1	132	tags=19%, list=8%, signal=21%
GOBP_CELL_COMMUNICATION_BY_ELECTRICAL_COUPLING		13	0.216409	0.79562336	0.8333333	0.9592343	1	1330	tags=100%, list=78%, signal=461%
GOBP_PORPHYRIN_CONTAINING_COMPOUND_METABOLIC_PROCESS		11	0.24343348	0.7801159	0.6666667	0.97798413	1	55	tags=18%, list=3%, signal=19%
GOBP_NEGATIVE_REGULATION_OF_LIPID_METABOLIC_PROCESS		13	0.23127235	0.77202815	0.7777778	0.98324776	1	1305	tags=100%, list=77%, signal=431%
GOBP_PROTEIN_IMPORT_INTO_MITOCHONDRIAL_MATRIX		12	0.19582365	0.7619708	0.9166667	0.99024457	1	155	tags=25%, list=9%, signal=27%
GOBP_TETRAPYRROLE_METABOLIC_PROCESS		16	0.183816	0.76024526	0.875	0.9850272	1	55	tags=13%, list=3%, signal=13%
GOBP_AMYLOID_PRECURSOR_PROTEIN_METABOLIC_PROCESS		14	0.19928545	0.75870824	0.9	0.97911423	1	135	tags=21%, list=8%, signal=23%
GOBP_TRICARBOXYLIC_ACID_CYCLE		25	0.16779943	0.75432116	0.6666667	0.9776095	1	836	tags=88%, list=49%, signal=171%
REACTOME_PYRUVATE_METABOLISM		19	0.1623564	0.73961896	1	0.99080706	1	167	tags=26%, list=10%, signal=29%
GOBP_MITOCHONDRIAL_FUSION		14	0.21415825	0.73790413	1	0.985472	1	1334	tags=100%, list=79%, signal=466%
GOBP_STEROL_BIOSYNTHETIC_PROCESS		15	0.1942698	0.7347349	0.8333333	0.98293215	1	85	tags=20%, list=5%, signal=21%
GOBP_RELAXATION_OF_MUSCLE		11	0.21615201	0.7316156	0.84615386	0.9794928	1	1330	tags=100%, list=78%, signal=461%
GOBP_IMPORT_INTO_CELL		29	0.20102349	0.73128456	1	0.9723432	1	146	tags=21%, list=9%, signal=22%
GOBP_POSITIVE_REGULATION_OF_CYSTEINE_TYPE_ENDOPEPTIDASE_ACTI		21	0.1592881	0.71664965	0.8	0.9784713	1	1015	tags=90%, list=60%, signal=223%
GOBP_RESPONSE_TO_ALKALOID		14	0.17627676	0.6792974	1	1	1	132	tags=21%, list=8%, signal=23%
GOBP_REGULATION_OF_STEROID_METABOLIC_PROCESS		20	0.15699941	0.6376237	1	1	1	85	tags=15%, list=5%, signal=16%
GOBP_MEMBRANE_PROTEIN_PROTEOLYSIS		12	0.17367269	0.6325892	0.90909094	1	1	1078	tags=92%, list=64%, signal=250%
GOBP_MITOCHONDRIAL_OUTER_MEMBRANE_PERMEABILIZATION		13	0.1898002	0.6229491	1	1	1	180	tags=31%, list=11%, signal=34%
GOBP_STRIATED_MUSCLE_ADAPTATION		11	0.18230407	0.6170147	1	1	1	1387	tags=100%, list=82%, signal=547%
REACTOME_MITOCHONDRIAL_FATTY_ACID_BETA_OXIDATION		25	0.13762332	0.6097297	1	1	1	2	tags=4%, list=0%, signal=4%
GOBP_POSITIVE_REGULATION_OF_LEUKOCYTE_MIGRATION		14	0.15638372	0.53300935	1	1	1	139	tags=21%, list=8%, signal=23%
KEGG_PROPANOATE_METABOLISM		22	0.11775101	0.53147125	1	1	1	70	tags=9%, list=4%, signal=9%
GOBP_GLUTAMATE_METABOLIC_PROCESS		11	0.13738696	0.51254916	1	0.99845666	1	1050	tags=91%, list=62%, signal=237%
REACTOME_THE_CITRIC_ACID_TCA_CYCLE_AND_RESPIRATORY_ELECTRON		134	0.30867946	---	---	1	0	181	tags=28%, list=11%, signal=28%
REACTOME_RESPIRATORY_ELECTRON_TRANSPORT_ATP_SYNTHESIS_BY_C		96	0.3719079	---	---	1	0	175	tags=31%, list=10%, signal=33%
REACTOME_RESPIRATORY_ELECTRON_TRANSPORT		81	0.3756085	---	---	1	0	145	tags=28%, list=9%, signal=30%
REACTOME_PROTEIN_LOCALIZATION		78	0.1136509	---	---	1	0	106	tags=12%, list=6%, signal=12%
GOBP_TEMPERATURE_HOMEOSTASIS		22	0.16258211	---	---	1	0	1422	tags=100%, list=84%, signal=613%
GOBP_HEART_PROCESS		65	0.17454228	---	---	1	0	148	tags=22%, list=9%, signal=23%
GOBP_GENERATION_OF_PRECURSOR_METABOLITES_AND_ENERGY		186	0.19350891	---	---	1	0	138	tags=19%, list=8%, signal=19%
GOBP_OXIDATIVE_PHOSPHORYLATION		89	0.41308048	---	---	1	0	168	tags=33%, list=10%, signal=34%
GOBP_CATION_TRANSPORT		188	0.15953943	---	---	1	0	139	tags=18%, list=8%, signal=18%
GOBP_MITOCHONDRIAL_TRANSPORT		106	0.1883304	---	---	1	0	168	tags=23%, list=10%, signal=24%
GOBP_MITOCHONDRION_ORGANIZATION		203	0.20674773	---	---	1	0	180	tags=24%, list=11%, signal=23%
GOBP_MITOCHONDRIAL_MEMBRANE_ORGANIZATION		69	0.2582185	---	---	1	0	180	tags=30%, list=11%, signal=33%
GOBP_REGULATION_OF_HEART_CONTRACTION		51	0.19697866	---	---	1	0	148	tags=24%, list=9%, signal=25%
GOBP_ORGANIC_ACID_TRANSPORT		42	0.15656056	---	---	1	0	131	tags=17%, list=8%, signal=18%
GOBP_ENERGY_DERIVATION_BY_OXIDATION_OF_ORGANIC_COMPOUNDS		120	0.23609407	---	---	1	0	226	tags=31%, list=13%, signal=33%
GOBP_ELECTRON_TRANSPORT_CHAIN		89	0.31272456	---	---	1	0	145	tags=25%, list=9%, signal=26%
GOBP_MITOCHONDRIAL_RESPIRATORY_CHAIN_COMPLEX_ASSEMBLY		68	0.3285151	---	---	1	0	175	tags=29%, list=10%, signal=31%
GOBP_CELLULAR_RESPIRATION		105	0.30644098	---	---	1	0	166	tags=25%, list=10%, signal=26%
GOBP_ATP_METABOLIC_PROCESS		128	0.23247086	---	---	1	0	168	tags=26%, list=10%, signal=26%
GOBP_RIBOSE_PHOSPHATE_BIOSYNTHETIC_PROCESS		63	0.11523709	---	---	1	0	103	tags=13%, list=6%, signal=13%
GOBP_PROTEIN_INSERTION_INTO_MEMBRANE		33	0.17056565	---	---	1	0	140	tags=21%, list=8%, signal=23%

GOBP_CALCIUM_ION_TRANSMEMBRANE_TRANSPORT		63	0.20847249	---	---	1	0	148	tags=25%, list=9%, signal=27%
GOBP_CATION_TRANSMEMBRANE_TRANSPORT		153	0.19255088	---	---	1	0	139	tags=20%, list=8%, signal=20%
GOBP_ANION_TRANSMEMBRANE_TRANSPORT		84	0.16045019	---	---	1	0	167	tags=23%, list=10%, signal=24%
GOBP_INORGANIC_ION_TRANSMEMBRANE_TRANSPORT		150	0.19814865	---	---	1	0	149	tags=22%, list=9%, signal=22%
KEGG_OXIDATIVE_PHOSPHORYLATION		73	0.43690848	---	---	1	0	168	tags=34%, list=10%, signal=36%
KEGG_ALZHEIMERS_DISEASE		82	0.40290663	---	---	1	0	168	tags=37%, list=10%, signal=39%
KEGG_PARKINSONS_DISEASE		70	0.5612652	---	---	1	0	168	tags=41%, list=10%, signal=44%

Supplementary Table 7e. Gene-sets enriched in the 24-month-old GA muscle samples									
GS<br>follow link to MSigDB	GS DETAILS	SIZE	ES	NES	NOM p-val	FDR q-val	FWER p-val	RANK AT MAX	LEADING EDGE
GOBP_INTERLEUKIN_8_PRODUCTION	Details ...	11	-0.7059808	-1.7642498	0	0.0497223	0.75	391	tags=82%, list=23%, signal=106%
REACTOME_TRANSCRIPTIONAL_REGULATION_BY_RUNX1	Details ...	33	-0.5637052	-1.7411722	0	0.06475087	0.87	192	tags=39%, list=11%, signal=44%
GOBP_CELLULAR_CARBOHYDRATE_BIOSYNTHETIC_PROCESS	Details ...	10	-0.6946212	-1.7411417	0.01111111	0.06266214	0.87	281	tags=80%, list=17%, signal=95%
GOBP_PEPTIDYL_TYROSINE_MODIFICATION	Details ...	28	-0.5782918	-1.7340722	0	0.06762491	0.9	341	tags=57%, list=20%, signal=70%
GOBP_TUMOR_NECROSIS_FACTOR_MEDIATED_SIGNALING_PATH	Details ...	30	-0.5809924	-1.7338082	0	0.06557567	0.9	355	tags=57%, list=21%, signal=70%
GOBP_NEGATIVE_REGULATION_OF_PROTEOLYSIS	Details ...	43	-0.5457006	-1.7325318	0	0.0650553	0.91	357	tags=47%, list=21%, signal=57%
GOBP_PLATELET_ACTIVATION	Details ...	34	-0.5507049	-1.7283592	0	0.06649341	0.92	393	tags=56%, list=23%, signal=71%
GOBP_REGULATION_OF_CELLULAR_AMINE_METABOLIC_PROCES	Details ...	26	-0.5551544	-1.7250441	0	0.06731112	0.92	192	tags=42%, list=11%, signal=47%
REACTOME_ACTIVATION_OF_THE_MRNA_UPON_BINDING_OF_TH	Details ...	38	-0.5423907	-1.7245581	0	0.06653421	0.92	638	tags=79%, list=38%, signal=124%
GOBP_RESPONSE_TO_TUMOR_NECROSIS_FACTOR	Details ...	42	-0.5416831	-1.7069466	0	0.08071592	0.95	355	tags=52%, list=21%, signal=65%
GOBP_REGULATION_OF_PEPTIDYL_TYROSINE_PHOSPHORYLATI	Details ...	26	-0.5532089	-1.7008636	0.01020408	0.08381743	0.97	341	tags=54%, list=20%, signal=66%
REACTOME_SEMAPHORIN_INTERACTIONS	Details ...	18	-0.6168653	-1.6987792	0	0.08363947	0.97	306	tags=56%, list=18%, signal=67%
REACTOME_ASSEMBLY_OF_THE_PRE_REPLICATIVE_COMPLEX	Details ...	22	-0.5322292	-1.6899062	0	0.09192234	1	192	tags=41%, list=11%, signal=46%
GOBP_NEGATIVE_REGULATION_OF_PEPTIDASE_ACTIVITY	Details ...	34	-0.5673047	-1.6873585	0	0.09271438	1	356	tags=47%, list=21%, signal=58%
GOBP_AMINOGLYCAN_BIOSYNTHETIC_PROCESS	Details ...	10	-0.6595679	-1.6805129	0.01111111	0.10062499	1	260	tags=60%, list=15%, signal=70%
REACTOME_THE_ROLE_OF_GTSE1_IN_G2_M_PROGRESSION_AF	Details ...	28	-0.5363382	-1.6704079	0	0.1086091	1	355	tags=54%, list=21%, signal=67%
GOBP_RNA_DEPENDENT_DNA_BIOSYNTHETIC_PROCESS	Details ...	12	-0.6506195	-1.6657826	0	0.11132423	1	376	tags=67%, list=22%, signal=85%
GOBP_POSITIVE_REGULATION_OF_WNT_SIGNALING_PATHWAY	Details ...	27	-0.5437916	-1.6612045	0.02040816	0.11558933	1	192	tags=41%, list=11%, signal=45%
GOBP_REGULATION_OF_CYTOSKELETON_ORGANIZATION	Details ...	63	-0.4870496	-1.6532469	0	0.12684831	1	570	tags=59%, list=34%, signal=85%
GOBP_ACTOMYOSIN_STRUCTURE_ORGANIZATION	Details ...	37	-0.5298339	-1.644702	0	0.13682595	1	406	tags=49%, list=24%, signal=63%
GOBP_RNA_SPLICING	Details ...	20	-0.5785548	-1.6331404	0	0.15581772	1	482	tags=75%, list=28%, signal=104%
GOBP_REGULATION_OF_CELL_CYCLE_PROCESS	Details ...	71	-0.4788321	-1.6309885	0	0.1542378	1	548	tags=56%, list=32%, signal=80%
GOBP_NEGATIVE_REGULATION_OF_CYSTEINE_TYPE_ENDOPEPT	Details ...	12	-0.6408202	-1.6274903	0.02173913	0.1572455	1	156	tags=42%, list=9%, signal=46%
GOBP_REGULATION_OF_NUCLEOCYTOPLASMIC_TRANSPORT	Details ...	12	-0.6244877	-1.6263193	0	0.15718043	1	513	tags=75%, list=30%, signal=107%
GOBP_CYTOKINESIS	Details ...	22	-0.5574437	-1.6257335	0	0.15566787	1	399	tags=55%, list=24%, signal=70%
GOBP_REGULATION_OF_NITRIC_OXIDE_METABOLIC_PROCESS	Details ...	12	-0.6068478	-1.6241529	0.02197802	0.1559895	1	386	tags=67%, list=23%, signal=86%
GOBP_RNA_PROCESSING	Details ...	63	-0.4740672	-1.6235863	0	0.15385443	1	601	tags=62%, list=35%, signal=92%
GOBP_FORMATION_OF_PRIMARY_GERM_LAYER	Details ...	11	-0.6530993	-1.6229179	0	0.15213817	1	325	tags=64%, list=19%, signal=78%
REACTOME_PROTEIN_FOLDING	Details ...	21	-0.5740211	-1.6191759	0	0.15875134	1	376	tags=57%, list=22%, signal=73%
GOBP_CELL_DIVISION	Details ...	47	-0.4971299	-1.6183602	0	0.15767089	1	518	tags=55%, list=31%, signal=77%
REACTOME_INTEGRIN_CELL_SURFACE_INTERACTIONS	Details ...	22	-0.566081	-1.6182709	0.01030928	0.15516287	1	393	tags=64%, list=23%, signal=82%
GOBP_POSITIVE_REGULATION_OF_PROTEIN_LOCALIZATION_TO	Details ...	18	-0.5992746	-1.617854	0.01041667	0.15354055	1	395	tags=67%, list=23%, signal=86%
GOBP_FERTILIZATION	Details ...	15	-0.5912094	-1.6157382	0	0.15465164	1	213	tags=47%, list=13%, signal=53%
GOBP_REGULATION_OF_LYMPHOCYTE_DIFFERENTIATION	Details ...	10	-0.6341083	-1.6126268	0.01176471	0.15882505	1	313	tags=60%, list=18%, signal=73%
REACTOME_MITOTIC_G2_G2_M_PHASES	Details ...	41	-0.5132318	-1.6122345	0	0.15706284	1	560	tags=63%, list=33%, signal=92%
GOBP_REGULATION_OF_CELLULAR_AMINO_ACID_METABOLIC_P	Details ...	23	-0.5613453	-1.6099111	0	0.15791184	1	192	tags=43%, list=11%, signal=48%
GOBP_CYTOSKELETON_DEPENDENT_CYTOKINESIS	Details ...	16	-0.5790783	-1.6085414	0.01075269	0.15814888	1	388	tags=56%, list=23%, signal=72%
REACTOME_COOPERATION_OF_PDCL_PHLP1_AND_TRIC_CCT_IN	Details ...	15	-0.6124173	-1.6048679	0.04347826	0.16230665	1	376	tags=60%, list=22%, signal=76%
GOBP_REGULATION_OF_ANATOMICAL_STRUCTURE_MORPHOGE	Details ...	142	-0.4544969	-1.6039147	0	0.16160375	1	548	tags=51%, list=32%, signal=70%
GOBP_CYTOKINE_PRODUCTION	Details ...	70	-0.4747247	-1.6014274	0.01	0.16389105	1	391	tags=43%, list=23%, signal=53%
GOBP_HEMATOPOIETIC_PROGENITOR_CELL_DIFFERENTIATION	Details ...	28	-0.5370939	-1.6002421	0.01030928	0.163326	1	205	tags=39%, list=12%, signal=44%
GOBP_NEGATIVE_REGULATION_OF_CELL_CYCLE_G2_M_PHASE	Details ...	26	-0.5188479	-1.5990989	0	0.16278194	1	193	tags=38%, list=11%, signal=43%

GOBP_POSITIVE_REGULATION_OF_DNA_BIOSYNTHETIC_PROCES	Details ...	11	-0.6358489	-1.5957242	0.01136364	0.16794446	1	376	tags=64%, list=22%, signal=81%
GOBP_CELLULAR_RESPONSE_TO_OXYGEN_LEVELS	Details ...	50	-0.4890432	-1.5956678	0	0.1656119	1	557	tags=58%, list=33%, signal=84%
GOBP_EXTERNAL_ENCAPSULATING_STRUCTURE_ORGANIZATION	Details ...	49	-0.4900272	-1.595275	0	0.16360731	1	393	tags=49%, list=23%, signal=62%
GOBP_POSITIVE_REGULATION_OF_CYTOKINE_PRODUCTION	Details ...	39	-0.5083357	-1.592845	0	0.16490711	1	429	tags=49%, list=25%, signal=64%
GOBP_REGULATION_OF_ANIMAL_ORGAN_MORPHOGENESIS	Details ...	32	-0.5326991	-1.5901818	0.01010101	0.16796197	1	206	tags=41%, list=12%, signal=45%
REACTOME_AUF1_HNRNP_D0_BINDS_AND_DESTABILIZES_MRNA	Details ...	26	-0.5379937	-1.5899088	0	0.16651118	1	548	tags=69%, list=32%, signal=101%
GOBP_PROTEIN_REFOLDING	Details ...	11	-0.6441542	-1.589412	0.02298851	0.16509809	1	405	tags=64%, list=24%, signal=83%
GOBP_PROTEIN_FOLDING	Details ...	71	-0.4795335	-1.5891235	0	0.1633538	1	605	tags=62%, list=36%, signal=92%
REACTOME_SEPARATION_OF_SISTER_CHROMATIDS	Details ...	34	-0.5109808	-1.5885239	0	0.16177367	1	560	tags=65%, list=33%, signal=95%
GOBP_ANAPHASE_PROMOTING_COMPLEX_DEPENDENT_CATABO	Details ...	22	-0.5322292	-1.5874283	0.01052632	0.16215768	1	192	tags=41%, list=11%, signal=46%
GOBP_NEGATIVE_REGULATION_OF_SUPRAMOLECULAR_FIBER_(	Details ...	21	-0.5458059	-1.58694	0	0.16158186	1	353	tags=57%, list=21%, signal=71%
GOBP_CYTOPLASMIC_TRANSLATION	Details ...	40	-0.4806845	-1.586668	0.03030303	0.16008206	1	591	tags=58%, list=35%, signal=86%
GOBP_FC_RECEPTOR_SIGNALING_PATHWAY	Details ...	40	-0.5024871	-1.5860438	0	0.15896355	1	420	tags=52%, list=25%, signal=68%
GOBP_STEM_CELL_DIFFERENTIATION	Details ...	31	-0.5271244	-1.5825173	0	0.16267726	1	355	tags=48%, list=21%, signal=60%
REACTOME_APC_C_CDH1_MEDIATED_DEGRADATION_OF_CDC20_	Details ...	22	-0.5322292	-1.5812625	0	0.16268319	1	192	tags=41%, list=11%, signal=46%
GOBP_CARTILAGE_DEVELOPMENT	Details ...	12	-0.6335909	-1.5810798	0.02197802	0.16123636	1	341	tags=58%, list=20%, signal=73%
GOBP_GASTRULATION	Details ...	18	-0.5612528	-1.5803242	0.03092784	0.16059831	1	460	tags=56%, list=27%, signal=75%
REACTOME_STABILIZATION_OF_P53	Details ...	22	-0.5322292	-1.5799226	0.0106383	0.15931861	1	192	tags=41%, list=11%, signal=46%
GOBP_REGULATION_OF_UBIQUITIN_DEPENDENT_PROTEIN_CATA	Details ...	23	-0.5502565	-1.5797693	0.02083333	0.1577454	1	724	tags=83%, list=43%, signal=142%
KEGG_ANTIGEN_PROCESSING_AND_PRESENTATION	Details ...	15	-0.5847088	-1.5793635	0.01111111	0.15631081	1	435	tags=60%, list=26%, signal=80%
GOBP_CELL_MATRIX_ADHESION	Details ...	40	-0.5108684	-1.5791179	0	0.15501457	1	504	tags=55%, list=30%, signal=76%
GOBP_CELL_CYCLE_G2_M_PHASE_TRANSITION	Details ...	42	-0.4882146	-1.5781986	0.01	0.15468633	1	548	tags=57%, list=32%, signal=82%
REACTOME_REGULATION_OF_RAS_BY_GAPS	Details ...	23	-0.5222604	-1.5778468	0.02150538	0.15405463	1	192	tags=39%, list=11%, signal=44%
GOBP_VASCULATURE_DEVELOPMENT	Details ...	78	-0.4724799	-1.5774792	0	0.15282458	1	344	tags=41%, list=20%, signal=49%
REACTOME_RHO_GTPASE_EFFECTORS	Details ...	46	-0.4686452	-1.5737009	0	0.15819755	1	704	tags=67%, list=42%, signal=112%
GOBP_REGULATION_OF_CELL_CYCLE_G2_M_PHASE_TRANSITIO	Details ...	40	-0.5029286	-1.5736765	0	0.15664929	1	548	tags=60%, list=32%, signal=87%
GOBP_HOMOTYPIC_CELL_CELL_ADHESION	Details ...	26	-0.5381221	-1.572367	0	0.15681627	1	659	tags=77%, list=39%, signal=124%
REACTOME_CROSS_PRESENTATION_OF_SOLUBLE_EXOGENOUS	Details ...	22	-0.5419182	-1.5701307	0.01075269	0.1586561	1	192	tags=41%, list=11%, signal=46%
REACTOME_DNA_REPLICATION_PRE_INITIATION	Details ...	22	-0.5322292	-1.5681169	0.0106383	0.16055079	1	192	tags=41%, list=11%, signal=46%
GOBP_REGULATION_OF_STEM_CELL_DIFFERENTIATION	Details ...	23	-0.5235947	-1.5672663	0.02061856	0.16048528	1	192	tags=39%, list=11%, signal=44%
GOBP_NEGATIVE_REGULATION_OF_CELLULAR_PROTEIN_LOCAL	Details ...	19	-0.5736986	-1.5665255	0.02105263	0.16013403	1	455	tags=63%, list=27%, signal=85%
GOBP_PROTEIN_POLYUBIQUITINATION	Details ...	36	-0.4891177	-1.5659232	0.01020408	0.15960008	1	355	tags=44%, list=21%, signal=55%
GOBP_MORPHOGENESIS_OF_A_POLARIZED_EPITHELIUM	Details ...	32	-0.5098076	-1.5654879	0.01010101	0.15833081	1	206	tags=38%, list=12%, signal=42%
REACTOME_MITOTIC_METAPHASE_AND_ANAPHASE	Details ...	44	-0.4943709	-1.564751	0.01020408	0.15829156	1	560	tags=61%, list=33%, signal=89%
GOBP_POSITIVE_REGULATION_OF_NEURON_DEATH	Details ...	11	-0.624827	-1.5636007	0.01123596	0.15906934	1	434	tags=55%, list=26%, signal=73%
REACTOME_REGULATION_OF_MRNA_STABILITY_BY_PROTEINS_T	Details ...	30	-0.5160239	-1.5627615	0.01041667	0.1586617	1	548	tags=67%, list=32%, signal=97%
REACTOME_UCH_PROTEINASES	Details ...	23	-0.5312573	-1.5610839	0.03191489	0.16050483	1	192	tags=39%, list=11%, signal=44%
GOBP_KERATINOCYTE_DIFFERENTIATION	Details ...	11	-0.612618	-1.5604601	0.02272727	0.16035159	1	294	tags=45%, list=17%, signal=55%
REACTOME_CELL_CELL_COMMUNICATION	Details ...	20	-0.5461031	-1.5565492	0.01098901	0.16727115	1	651	tags=75%, list=38%, signal=120%
REACTOME_DEGRADATION_OF_AXIN	Details ...	22	-0.5322292	-1.5562453	0.02040816	0.16592565	1	192	tags=41%, list=11%, signal=46%
GOBP_REGULATION_OF_DNA_TEMPLATED_TRANSCRIPTION_IN_F	Details ...	28	-0.5304996	-1.5560188	0.01041667	0.16451688	1	548	tags=68%, list=32%, signal=99%
REACTOME_DEGRADATION_OF_BETA_CATENIN_BY_THE_DESTRU	Details ...	27	-0.5102211	-1.5558941	0	0.16339125	1	192	tags=37%, list=11%, signal=41%
GOBP_SKIN_DEVELOPMENT	Details ...	19	-0.5808935	-1.5557035	0	0.1622866	1	294	tags=42%, list=17%, signal=50%
GOBP_ACTIVATION_OF_INNATE_IMMUNE_RESPONSE	Details ...	31	-0.5026668	-1.5548441	0.01020408	0.1617881	1	548	tags=58%, list=32%, signal=84%
GOBP_REGULATION_OF_CELL_CYCLE_PHASE_TRANSITION	Details ...	51	-0.482672	-1.5536128	0.01010101	0.16263418	1	548	tags=59%, list=32%, signal=84%
REACTOME_BETA_CATENIN_INDEPENDENT_WNT_SIGNALING	Details ...	40	-0.4959212	-1.5535048	0.01020408	0.16131458	1	367	tags=45%, list=22%, signal=56%

REACTOME_ASYMMETRIC_LOCALIZATION_OF_PCP_PROTEINS	Details ...	22	-0.5322292	-1.5531584	0.01086957	0.16034618	1	192	tags=41%, list=11%, signal=46%
GOBP_NEGATIVE_REGULATION_OF_CYTOSKELETON_ORGANIZA	Details ...	18	-0.5714421	-1.5525247	0.01086957	0.1601287	1	352	tags=61%, list=21%, signal=76%
GOBP_PATTERN_SPECIFICATION_PROCESS	Details ...	10	-0.6430431	-1.5516315	0	0.16047774	1	472	tags=60%, list=28%, signal=83%
REACTOME_NERVOUS_SYSTEM_DEVELOPMENT	Details ...	172	-0.4323175	-1.5511065	0	0.16018067	1	819	tags=72%, list=48%, signal=124%
REACTOME_CELL_CYCLE	Details ...	67	-0.4552829	-1.5491691	0	0.16219693	1	576	tags=54%, list=34%, signal=78%
GOBP_REGULATION_OF_PROTEASOMAL_PROTEIN_CATABOLIC_	Details ...	27	-0.5111279	-1.5475672	0	0.16307293	1	736	tags=74%, list=43%, signal=129%
REACTOME_PLATELET_AGGREGATION_PLUG_FORMATION	Details ...	11	-0.6070418	-1.5475429	0	0.16174713	1	393	tags=64%, list=23%, signal=82%
GOBP_CELLULAR_RESPONSE_TO_KETONE	Details ...	11	-0.626256	-1.5470723	0.02325581	0.1609082	1	367	tags=73%, list=22%, signal=92%
REACTOME_TNFR2_NON_CANONICAL_NF_KB_PATHWAY	Details ...	25	-0.5188606	-1.5462495	0.01010101	0.16108285	1	192	tags=40%, list=11%, signal=44%
REACTOME_DECTIN_1_MEDIATED_NONCANONICAL_NF_KB_SIGN	Details ...	25	-0.5188606	-1.545139	0.01030928	0.1617859	1	192	tags=40%, list=11%, signal=44%
GOBP_ACTIN_FILAMENT_BUNDLE_ORGANIZATION	Details ...	25	-0.5345056	-1.5447793	0.01030928	0.1611955	1	294	tags=44%, list=17%, signal=52%
GOBP_MUCOPOLYSACCHARIDE_METABOLIC_PROCESS	Details ...	16	-0.582689	-1.5446076	0	0.16016169	1	341	tags=56%, list=20%, signal=70%
GOBP_NEGATIVE_REGULATION_OF_GENE_EXPRESSION		186	-0.4219234	-1.5441471	0	0.15936778	1	566	tags=50%, list=33%, signal=67%
REACTOME_PTEN_REGULATION		29	-0.5133851	-1.5438207	0.01041667	0.15880793	1	355	tags=48%, list=21%, signal=60%
GOBP_REGULATION_OF_MRNA_CATABOLIC_PROCESS		40	-0.4846374	-1.5437602	0	0.15766944	1	548	tags=58%, list=32%, signal=83%
GOBP_NEGATIVE_REGULATION_OF_ACTIN_FILAMENT_POLYMERIZATION		16	-0.5558106	-1.5436143	0.0212766	0.15669426	1	624	tags=81%, list=37%, signal=127%
REACTOME_CELL_CYCLE_MITOTIC		61	-0.4661416	-1.5428873	0	0.15754324	1	560	tags=54%, list=33%, signal=78%
GOBP_REGULATION_OF_PROTEASOMAL_UBIQUITIN_DEPENDENT_PROTEIN_C		18	-0.5566048	-1.542652	0.02105263	0.15679896	1	628	tags=72%, list=37%, signal=114%
GOBP_REGULATION_OF_CELL_SHAPE		27	-0.5194959	-1.541643	0	0.15749033	1	547	tags=59%, list=32%, signal=86%
GOBP_REGULATION_OF_ORGANELLE_ASSEMBLY		23	-0.5202321	-1.5412586	0.03157895	0.15661417	1	539	tags=65%, list=32%, signal=94%
GOBP_POSITIVE_REGULATION_OF_CANONICAL_WNT_SIGNALING_PATHWAY		26	-0.5235883	-1.5408404	0.01052632	0.15610361	1	192	tags=38%, list=11%, signal=43%
REACTOME_PCP_CE_PATHWAY		31	-0.5058454	-1.5405955	0.01020408	0.15553002	1	206	tags=39%, list=12%, signal=43%
GOBP_ANTIGEN_PROCESSING_AND_PRESENTATION_OF_EXOGENOUS_PEPT		33	-0.5047183	-1.5400226	0.01	0.1551722	1	355	tags=42%, list=21%, signal=53%
GOBP_ESTABLISHMENT_OF_TISSUE_POLARITY		30	-0.4936104	-1.5397242	0.01010101	0.15440603	1	206	tags=37%, list=12%, signal=41%
GOBP_INNATE_IMMUNE_RESPONSE_ACTIVATING_SIGNAL_TRANSDUCTION		27	-0.5197267	-1.5396574	0.01075269	0.15331095	1	192	tags=37%, list=11%, signal=41%
REACTOME_TRANSCRIPTIONAL_REGULATION_BY_RUNX3		23	-0.5262591	-1.5376706	0.03092784	0.15521252	1	192	tags=39%, list=11%, signal=44%
REACTOME_APC_C_MEDIATED_DEGRADATION_OF_CELL_CYCLE_PROTEINS		24	-0.499663	-1.5376068	0	0.15426156	1	548	tags=63%, list=32%, signal=91%
GOBP_CELL_CYCLE_PHASE_TRANSITION		59	-0.4667378	-1.5372548	0	0.15372516	1	548	tags=56%, list=32%, signal=80%
GOBP_REGULATION_OF_T_CELL_DIFFERENTIATION		10	-0.6341083	-1.5371417	0.02272727	0.15273164	1	313	tags=60%, list=18%, signal=73%
GOBP_FC_EPSILON_RECEPTOR_SIGNALING_PATHWAY		27	-0.5215514	-1.5370083	0.01030928	0.15188302	1	548	tags=63%, list=32%, signal=92%
GOBP_PROTEIN_DEPOLYMERIZATION		22	-0.5297033	-1.5367305	0.01041667	0.15137248	1	624	tags=77%, list=37%, signal=121%
REACTOME_HEDGEHOG_ON_STATE		23	-0.5302674	-1.5360922	0	0.15100102	1	192	tags=39%, list=11%, signal=44%
REACTOME_C_TYPE_LECTIN_RECEPTORS_CLRS		32	-0.4827972	-1.5355525	0.02040816	0.15076008	1	576	tags=59%, list=34%, signal=88%
GOBP_REGULATION_OF_HEMOPOIESIS		23	-0.5261749	-1.5336406	0.0212766	0.15167958	1	313	tags=43%, list=18%, signal=53%
GOBP_REGULATION_OF_LEUKOCYTE_DIFFERENTIATION		16	-0.5822918	-1.5331596	0.01041667	0.15144072	1	313	tags=50%, list=18%, signal=61%
GOBP_POSITIVE_REGULATION_OF_AXONOGENESIS		12	-0.6188059	-1.5328305	0	0.15082516	1	547	tags=75%, list=32%, signal=110%
REACTOME_METABOLISM_OF_POLYAMINES		22	-0.5421715	-1.5318844	0.01052632	0.15216315	1	192	tags=41%, list=11%, signal=46%
GOBP_MODIFICATION_DEPENDENT_MACROMOLECULE_CATABOLIC_PROCESS		80	-0.4476846	-1.5298663	0	0.15416934	1	828	tags=78%, list=49%, signal=144%
REACTOME_INTERLEUKIN_4_AND_INTERLEUKIN_13_SIGNALING		13	-0.5960476	-1.5298581	0.01149425	0.15317468	1	353	tags=62%, list=21%, signal=77%
GOBP_MRNA_METABOLIC_PROCESS		128	-0.4337854	-1.528993	0	0.15348706	1	819	tags=73%, list=48%, signal=130%
REACTOME_METABOLISM_OF_RNA		116	-0.4424552	-1.5283929	0	0.15373108	1	819	tags=74%, list=48%, signal=134%
REACTOME_REGULATION_OF_RUNX3_EXPRESSION_AND_ACTIVITY		22	-0.5322292	-1.5272769	0.03125	0.15428178	1	192	tags=41%, list=11%, signal=46%
GOBP_EPIDERMAL_CELL_DIFFERENTIATION		13	-0.5731481	-1.5269117	0.02197802	0.1535536	1	294	tags=38%, list=17%, signal=46%
REACTOME_SWITCHING_OF_ORIGINS_TO_A_POST_REPLICATIVE_STATE		24	-0.499663	-1.5269022	0.02150538	0.15265408	1	548	tags=63%, list=32%, signal=91%
GOBP_RIBOSOMAL_SMALL_SUBUNIT_BIOGENESIS		18	-0.5304354	-1.5267448	0.02150538	0.15200454	1	638	tags=72%, list=38%, signal=115%
GOBP_NEGATIVE_REGULATION_OF_CELL_DEVELOPMENT		18	-0.5314246	-1.5266145	0.02222222	0.15142214	1	347	tags=44%, list=20%, signal=55%

GOBP_RNA_CATABOLIC_PROCESS		118	-0.4429427	-1.5265409	0	0.15061106	1	819	tags=74%, list=48%, signal=133%
GOBP_NEGATIVE_REGULATION_OF_NEURON_PROJECTION_DEVELOPMENT		15	-0.5737354	-1.5264918	0.02150538	0.14975165	1	301	tags=47%, list=18%, signal=56%
REACTOME_MITOTIC_G1_PHASE_AND_G1_S_TRANSITION		25	-0.5171114	-1.5254407	0.01041667	0.15018554	1	192	tags=40%, list=11%, signal=44%
GOBP_RHYTHMIC_PROCESS		14	-0.5561056	-1.5253338	0.04347826	0.14933941	1	405	tags=57%, list=24%, signal=74%
REACTOME_REGULATION_OF_HMOX1_EXPRESSION_AND_ACTIVITY		25	-0.5176506	-1.5252955	0.03030303	0.14850296	1	192	tags=40%, list=11%, signal=44%
REACTOME_TCR_SIGNALING		28	-0.4833928	-1.5242137	0.01052632	0.14962378	1	819	tags=86%, list=48%, signal=163%
GOBP_PLATELET_AGGREGATION		21	-0.5595524	-1.523464	0.02150538	0.15010375	1	393	tags=57%, list=23%, signal=73%
KEGG_SYSTEMIC_LUPUS_ERYTHEMATOSUS		10	-0.6122716	-1.523429	0.03488372	0.14927742	1	434	tags=50%, list=26%, signal=67%
REACTOME_M_PHASE		57	-0.4639204	-1.5233904	0.01	0.14846118	1	560	tags=54%, list=33%, signal=78%
REACTOME_SEMA4D_IN_SEMAPHORIN_SIGNALING		10	-0.6181619	-1.5224649	0.03571429	0.1488843	1	294	tags=50%, list=17%, signal=60%
GOBP_POLYSACCHARIDE_METABOLIC_PROCESS		15	-0.560166	-1.5220178	0.03333334	0.14863575	1	281	tags=53%, list=17%, signal=63%
GOBP_INTEGRIN_MEDIATED_SIGNALING_PATHWAY		23	-0.5286837	-1.5211723	0.01041667	0.14916334	1	386	tags=52%, list=23%, signal=67%
GOBP_CELL_CELL_ADHESION		99	-0.4516259	-1.5207878	0	0.1488603	1	659	tags=60%, list=39%, signal=92%
GOBP_CONNECTIVE_TISSUE_DEVELOPMENT		16	-0.5755232	-1.5207753	0.02197802	0.14801452	1	341	tags=56%, list=20%, signal=70%
GOBP_DNA_TEMPLATED_TRANSCRIPTION_INITIATION		10	-0.6132278	-1.5199349	0.02325581	0.14880505	1	192	tags=40%, list=11%, signal=45%
GOBP_REGULATION_OF_CELL_CYCLE		94	-0.4417243	-1.5192807	0	0.1493181	1	558	tags=52%, list=33%, signal=73%
GOBP_REGULATION_OF_TRANSCRIPTION_FROM_RNA_POLYMERASE_II_PROM		25	-0.5079725	-1.5183038	0	0.14993656	1	548	tags=64%, list=32%, signal=93%
GOBP_RIBONUCLEOPROTEIN_COMPLEX_BIOGENESIS		62	-0.4626738	-1.5177997	0.02	0.15000837	1	737	tags=71%, list=43%, signal=121%
GOBP_REGULATION_OF_HEMATOPOIETIC_STEM_CELL_DIFFERENTIATION		23	-0.5235947	-1.5177435	0	0.1491796	1	192	tags=39%, list=11%, signal=44%
REACTOME_IMMUNOREGULATORY_INTERACTIONS_BETWEEN_A_LYMPHOID_A		12	-0.5769543	-1.5155044	0.03448276	0.15142384	1	259	tags=50%, list=15%, signal=59%
GOBP_CIRCULATORY_SYSTEM_DEVELOPMENT		121	-0.4224225	-1.5152582	0	0.15127842	1	459	tags=41%, list=27%, signal=53%
GOBP_AMINOGLYCAN_CATABOLIC_PROCESS		13	-0.5890449	-1.5151006	0.0212766	0.15071763	1	341	tags=54%, list=20%, signal=67%
GOBP_POSITIVE_REGULATION_OF_CHROMOSOME_ORGANIZATION		13	-0.5655249	-1.5143119	0.02173913	0.15068193	1	576	tags=69%, list=34%, signal=104%
GOBP_ANTIGEN_PROCESSING_AND_PRESENTATION_OF_PEPTIDE_ANTIGEN_		41	-0.4790382	-1.5139031	0	0.15059693	1	355	tags=41%, list=21%, signal=51%
GOBP_AMINOGLYCAN_METABOLIC_PROCESS		17	-0.5617412	-1.5125002	0	0.15236174	1	341	tags=53%, list=20%, signal=66%
GOBP_AMINE_METABOLIC_PROCESS		34	-0.4703557	-1.5123155	0.03061225	0.15160209	1	568	tags=59%, list=34%, signal=87%
GOBP_HEMATOPOIETIC_STEM_CELL_DIFFERENTIATION		24	-0.5113298	-1.5120531	0.03191489	0.151004	1	192	tags=38%, list=11%, signal=42%
GOBP_NEGATIVE_REGULATION_OF_CELL_ADHESION		31	-0.4990965	-1.5117159	0.03061225	0.15076631	1	663	tags=68%, list=39%, signal=109%
GOBP_POSITIVE_REGULATION_OF_NEUROGENESIS		21	-0.5201933	-1.510234	0.02105263	0.15214239	1	547	tags=57%, list=32%, signal=83%
GOBP_CELLULAR_RESPONSE_TO_STEROID_HORMONE_STIMULUS		20	-0.5096618	-1.5101308	0.0212766	0.15155062	1	279	tags=40%, list=16%, signal=47%
GOBP_RESPONSE_TO_WOUNDING		105	-0.4397351	-1.5101264	0	0.15076539	1	519	tags=49%, list=31%, signal=66%
GOBP_REGULATION_OF_ACTIN_FILAMENT_LENGTH		29	-0.4826948	-1.5100092	0.01041667	0.15043393	1	786	tags=79%, list=46%, signal=145%
GOBP_REGULATION_OF_HEMATOPOIETIC_PROGENITOR_CELL_DIFFERENTIAT		23	-0.5235947	-1.5098947	0.02040816	0.1499588	1	192	tags=39%, list=11%, signal=44%
GOBP_SUPRAMOLECULAR_FIBER_ORGANIZATION		109	-0.4404392	-1.509807	0	0.14934109	1	534	tags=50%, list=32%, signal=69%
GOBP_REGULATION_OF_CELL_SIZE		27	-0.4922867	-1.5097457	0.03061225	0.14858301	1	570	tags=63%, list=34%, signal=93%
REACTOME_G2_M_CHECKPOINTS		30	-0.4898316	-1.5092264	0.01	0.1484152	1	819	tags=87%, list=48%, signal=165%
REACTOME_DOWNSTREAM_SIGNALING_EVENTS_OF_B_CELL_RECEPTOR_BC		28	-0.4998817	-1.5090779	0	0.14791125	1	548	tags=61%, list=32%, signal=88%
GOBP_REGULATION_OF_ORGANELLE_ORGANIZATION		152	-0.4261844	-1.5087702	0	0.14760476	1	643	tags=57%, list=38%, signal=83%
GOBP_ACTIN_FILAMENT_BASED_PROCESS		137	-0.4222336	-1.5082216	0	0.1477298	1	452	tags=42%, list=27%, signal=52%
GOBP_REGULATION_OF_CELL_MORPHOGENESIS		52	-0.450765	-1.5082163	0	0.14699847	1	547	tags=54%, list=32%, signal=77%
GOBP_NIK_NF_KAPPAB_SIGNALING		37	-0.4793918	-1.5072545	0	0.14731517	1	192	tags=32%, list=11%, signal=36%
GOBP_ACTIN_CYTOSKELETON_REORGANIZATION		10	-0.6052411	-1.5063996	0.04444445	0.14767659	1	285	tags=50%, list=17%, signal=60%
GOBP_PROTEIN_MODIFICATION_BY_SMALL_PROTEIN_REMOVAL		33	-0.507121	-1.5056531	0.03061225	0.14808124	1	192	tags=39%, list=11%, signal=44%
GOBP_REGULATION_OF_WNT_SIGNALING_PATHWAY		40	-0.4884365	-1.5053467	0	0.14773695	1	355	tags=45%, list=21%, signal=56%
GOBP_REGULATION_OF_PROTEIN_POLYMERIZATION		34	-0.5042749	-1.5052274	0	0.147349	1	668	tags=76%, list=39%, signal=124%
GOBP_RRNA_METABOLIC_PROCESS		26	-0.5144206	-1.5048128	0.01030928	0.14724162	1	714	tags=77%, list=42%, signal=131%

GOBP_WOUND_HEALING		92	-0.4445769	-1.5045829	0	0.14708792	1	519	tags=51%, list=31%, signal=70%
GOBP_NON_CANONICAL_WNT_SIGNALING_PATHWAY		35	-0.4952142	-1.5042012	0.01	0.14675471	1	367	tags=46%, list=22%, signal=57%
GOBP_CYTOKINE_MEDIATED_SIGNALING_PATHWAY		95	-0.4584782	-1.5041236	0	0.14614986	1	576	tags=56%, list=34%, signal=80%
GOBP_REGULATION_OF_CELLULAR_COMPONENT_SIZE		59	-0.4707429	-1.5035785	0	0.14623275	1	570	tags=59%, list=34%, signal=86%
GOBP_REGULATION_OF_PROTEIN_DEPOLYMERIZATION		18	-0.5127934	-1.5010633	0.06315789	0.14911	1	786	tags=89%, list=46%, signal=164%
GOBP_HINDBRAIN_DEVELOPMENT		10	-0.6262133	-1.5009449	0.01190476	0.14841321	1	405	tags=50%, list=24%, signal=65%
GOBP_REGULATION_OF_PROTEIN_CONTAINING_COMPLEX_ASSEMBLY		74	-0.4398144	-1.4993029	0	0.15005094	1	643	tags=61%, list=38%, signal=94%
REACTOME_REGULATION_OF_PTEN_STABILITY_AND_ACTIVITY		25	-0.5300522	-1.4992261	0	0.1494905	1	355	tags=52%, list=21%, signal=65%
GOBP_POSITIVE_REGULATION_OF_PROTEIN_CONTAINING_COMPLEX_ASSEM		39	-0.4747244	-1.4990163	0	0.14915486	1	707	tags=72%, list=42%, signal=120%
REACTOME_AQUAPORIN_MEDIATED_TRANSPORT		12	-0.5803826	-1.4990116	0.03333334	0.14847067	1	519	tags=58%, list=31%, signal=83%
GOBP_ACTIN_FILAMENT_ORGANIZATION		65	-0.4607436	-1.4961532	0	0.15283942	1	668	tags=63%, list=39%, signal=100%
REACTOME_CYTOKINE_SIGNALING_IN_IMMUNE_SYSTEM		90	-0.4389411	-1.495759	0	0.1528884	1	576	tags=56%, list=34%, signal=80%
GOBP_BLOOD_VESSEL_MORPHOGENESIS		67	-0.4700074	-1.495127	0	0.15310887	1	344	tags=40%, list=20%, signal=49%
REACTOME_RUNX1_REGULATES_TRANSCRIPTION_OF_GENES_INVOLVED_IN_		25	-0.5097023	-1.4950384	0.03157895	0.15250605	1	192	tags=36%, list=11%, signal=40%
GOBP_CELL_CYCLE_PROCESS		120	-0.4273574	-1.4938806	0	0.15350394	1	548	tags=49%, list=32%, signal=68%
REACTOME_COOPERATION_OF_PREFOLDIN_AND_TRIC_CCT_IN_ACTIN_AND_T		12	-0.5815281	-1.4936575	0.07777778	0.1532041	1	196	tags=50%, list=12%, signal=56%
GOBP_MORPHOGENESIS_OF_AN_EPITHELIUM		54	-0.4574207	-1.4936131	0	0.15256576	1	548	tags=50%, list=32%, signal=72%
GOBP_REGULATION_OF_PROTEIN_LOCALIZATION_TO_NUCLEUS		22	-0.5279068	-1.4931457	0	0.15218954	1	395	tags=55%, list=23%, signal=70%
REACTOME_SENSORY_PROCESSING_OF_SOUND		14	-0.6011699	-1.4927427	0.01086957	0.15223897	1	534	tags=86%, list=32%, signal=124%
REACTOME_FORMATION_OF_TUBULIN_FOLDING_INTERMEDIATES_BY_CCT_TF		12	-0.5815281	-1.4921781	0.03225806	0.15254262	1	196	tags=50%, list=12%, signal=56%
REACTOME_DEGRADATION_OF_DVL		22	-0.5322292	-1.4917862	0.01075269	0.15225385	1	192	tags=41%, list=11%, signal=46%
REACTOME_SIGNALING_BY_INTERLEUKINS		70	-0.4591519	-1.4917003	0	0.15163356	1	576	tags=59%, list=34%, signal=85%
REACTOME_RHOF_GTPASE_CYCLE		13	-0.5738415	-1.4911242	0	0.15180852	1	443	tags=62%, list=26%, signal=83%
REACTOME_HEDGEHOG_OFF_STATE		33	-0.4851764	-1.4903098	0.03	0.15235598	1	560	tags=58%, list=33%, signal=84%
GOBP_REGULATION_OF_AXONOGENESIS		15	-0.5523826	-1.4896882	0.05681818	0.15294084	1	547	tags=67%, list=32%, signal=98%
REACTOME_SENSORY_PERCEPTION		29	-0.4859994	-1.4867107	0.01041667	0.15672563	1	391	tags=48%, list=23%, signal=62%
GOBP_HETEROTYPIC_CELL_CELL_ADHESION		16	-0.5406944	-1.4855852	0.04395605	0.15773639	1	429	tags=56%, list=25%, signal=75%
REACTOME_SIGNALING_BY_THE_B_CELL_RECEPTOR_BCR		30	-0.4977702	-1.4854047	0.03061225	0.1573525	1	548	tags=60%, list=32%, signal=87%
GOBP_BIOLOGICAL_ADHESION		155	-0.4204658	-1.4844625	0	0.15814634	1	663	tags=55%, list=39%, signal=83%
GOBP_TELOMERE_ORGANIZATION		13	-0.5582889	-1.484267	0.05681818	0.15776445	1	376	tags=54%, list=22%, signal=69%
REACTOME_INTRACELLULAR_SIGNALING_BY_SECOND_MESSENGERS		41	-0.4631801	-1.483049	0	0.15895583	1	192	tags=32%, list=11%, signal=35%
GOBP_CELL_CYCLE		145	-0.428244	-1.4825772	0	0.15881473	1	560	tags=50%, list=33%, signal=69%
GOBP_NEGATIVE_REGULATION_OF_ACTIN_FILAMENT_DEPOLYMERIZATION		14	-0.557842	-1.4817724	0.0326087	0.15939088	1	624	tags=86%, list=37%, signal=135%
GOBP_CHAPERONE_MEDIATED_PROTEIN_FOLDING		18	-0.5307273	-1.4816945	0.03333334	0.15885139	1	540	tags=67%, list=32%, signal=97%
GOBP_RESPONSE_TO_OXYGEN_LEVELS		72	-0.443194	-1.481151	0	0.15898912	1	636	tags=58%, list=38%, signal=89%
GOBP_REGULATION_OF_RESPONSE_TO_STRESS		174	-0.4174123	-1.4809045	0	0.15865268	1	637	tags=56%, list=38%, signal=81%
REACTOME_CELL_CYCLE_CHECKPOINTS		38	-0.490128	-1.4808638	0	0.15800512	1	819	tags=84%, list=48%, signal=159%
GOBP_ORGANIC_CYCLIC_COMPOUND_CATABOLIC_PROCESS		150	-0.4306209	-1.4806083	0	0.15775359	1	820	tags=69%, list=48%, signal=121%
GOBP_NEGATIVE_REGULATION_OF_PROTEIN_METABOLIC_PROCESS		120	-0.42315	-1.4804302	0	0.15742485	1	520	tags=46%, list=31%, signal=61%
GOBP_POSTTRANSCRIPTIONAL_REGULATION_OF_GENE_EXPRESSION		95	-0.4243461	-1.479632	0	0.15822631	1	550	tags=52%, list=32%, signal=72%
GOBP_POSITIVE_REGULATION_OF_CYTOSKELETON_ORGANIZATION		32	-0.4645161	-1.4795134	0.03092784	0.1578225	1	521	tags=53%, list=31%, signal=75%
GOBP_TUBE_MORPHOGENESIS		80	-0.4459123	-1.4792067	0	0.15784624	1	357	tags=38%, list=21%, signal=45%
GOBP_SCF_DEPENDENT_PROTEASOMAL_UBIQUITIN_DEPENDENT_PROTEIN_C		27	-0.5082479	-1.4783611	0.02020202	0.15867233	1	548	tags=63%, list=32%, signal=92%
GOBP_NEGATIVE_REGULATION_OF_CANONICAL_WNT_SIGNALING_PATHWAY		27	-0.4839646	-1.4779881	0.01030928	0.15838595	1	820	tags=85%, list=48%, signal=162%
GOBP_TELOMERE_MAINTENANCE_VIA_TELOMERE_LENGTHENING		11	-0.6358489	-1.4778812	0.01111111	0.15794902	1	376	tags=64%, list=22%, signal=81%
REACTOME_HSP90_CHAPERONE_CYCLE_FOR_STEROID_HORMONE_RECEPTO		20	-0.5235021	-1.477845	0.0326087	0.15744066	1	560	tags=70%, list=33%, signal=103%

GOBP_REGULATION_OF_MRNA_METABOLIC_PROCESS		45	-0.4674763	-1.4772321	0.01	0.15787908	1	548	tags=58%, list=32%, signal=83%
REACTOME_VASOPRESSIN_REGULATES_RENAL_WATER_HOMEOSTASIS_VIA_		12	-0.5803826	-1.476155	0.02352941	0.15857568	1	519	tags=58%, list=31%, signal=83%
GOBP_CELL_ACTIVATION		215	-0.4026838	-1.4750675	0	0.15941666	1	656	tags=53%, list=39%, signal=75%
REACTOME_DEVELOPMENTAL_BIOLOGY		184	-0.4219616	-1.4749504	0	0.15898466	1	851	tags=72%, list=50%, signal=129%
GOBP_POSITIVE_REGULATION_OF_NUCLEOBASE_CONTAINING_COMPOUND_		108	-0.4314104	-1.4743545	0	0.15937634	1	576	tags=52%, list=34%, signal=74%
GOBP_NEGATIVE_REGULATION_OF_EXTRINSIC_APOPTOTIC_SIGNALING_PAT		16	-0.542777	-1.4742782	0.03125	0.15894836	1	683	tags=81%, list=40%, signal=135%
REACTOME_METABOLISM_OF_CARBOHYDRATES		52	-0.4550777	-1.4742155	0	0.15837596	1	496	tags=52%, list=29%, signal=71%
REACTOME_REGULATION_OF_HSF1_MEDIATED_HEAT_SHOCK_RESPONSE		11	-0.6014978	-1.4742029	0.02325581	0.15777147	1	513	tags=82%, list=30%, signal=117%
GOBP_T_CELL_RECEPTOR_SIGNALING_PATHWAY		34	-0.4675812	-1.4739156	0.02040816	0.15764722	1	819	tags=79%, list=48%, signal=151%
REACTOME_TCF_DEPENDENT_SIGNALING_IN_RESPONSE_TO_WNT		32	-0.4908868	-1.4731833	0.02020202	0.15854073	1	819	tags=81%, list=48%, signal=154%
GOBP_DEFENSE_RESPONSE_TO_VIRUS		22	-0.5065323	-1.4727733	0.05208333	0.15852067	1	401	tags=55%, list=24%, signal=71%
GOBP_CYTOSKELETON_ORGANIZATION		168	-0.4169537	-1.4722375	0	0.15846586	1	702	tags=60%, list=41%, signal=92%
REACTOME_G1_S_DNA_DAMAGE_CHECKPOINTS		22	-0.5322292	-1.4721445	0.03061225	0.1580519	1	192	tags=41%, list=11%, signal=46%
GOBP_NEGATIVE_REGULATION_OF_TRANSFERASE_ACTIVITY		38	-0.4492347	-1.4717159	0.03125	0.15814312	1	687	tags=68%, list=41%, signal=112%
REACTOME_CYCLIN_A_CDK2_ASSOCIATED_EVENTS_AT_S_PHASE_ENTRY		24	-0.499663	-1.4715207	0.02173913	0.15784186	1	548	tags=63%, list=32%, signal=91%
GOBP_CELL_SURFACE_RECEPTOR_SIGNALING_PATHWAY_INVOLVED_IN_CEL		63	-0.4534953	-1.470064	0.01020408	0.15949832	1	548	tags=54%, list=32%, signal=77%
REACTOME_HEDGEHOG_LIGAND_BIOGENESIS		26	-0.4897393	-1.4692285	0.04123711	0.15993835	1	819	tags=88%, list=48%, signal=169%
REACTOME_SIGNALING_BY_WNT		52	-0.4558131	-1.468988	0	0.15981118	1	548	tags=52%, list=32%, signal=74%
GOBP_CELL_CELL_SIGNALING_BY_WNT		62	-0.4472775	-1.4680972	0	0.16074036	1	548	tags=53%, list=32%, signal=76%
REACTOME_S_PHASE		24	-0.499663	-1.4677399	0.02061856	0.16068083	1	548	tags=63%, list=32%, signal=91%
GOBP_REGULATION_OF_LAMELLIPODIUM_ORGANIZATION		11	-0.5761029	-1.4671948	0.04545455	0.16104087	1	341	tags=55%, list=20%, signal=68%
REACTOME_REGULATION_OF_RUNX2_EXPRESSION_AND_ACTIVITY		25	-0.4840924	-1.4670287	0.04123711	0.16070274	1	819	tags=88%, list=48%, signal=168%
GOBP_REGULATION_OF_CHROMOSOME_ORGANIZATION		13	-0.5655249	-1.4643024	0.03225806	0.16387191	1	576	tags=69%, list=34%, signal=104%
GOBP_SINGLE_FERTILIZATION		14	-0.5394498	-1.4636153	0.03296704	0.16425094	1	213	tags=43%, list=13%, signal=49%
GOBP_ANATOMICAL_STRUCTURE_FORMATION_INVOLVED_IN_MORPHOGENES		116	-0.4290812	-1.4630446	0	0.1646277	1	357	tags=34%, list=21%, signal=41%
GOBP_REGULATION_OF_OXIDATIVE_STRESS_INDUCED_CELL_DEATH		13	-0.5658304	-1.4626455	0.03370787	0.16462365	1	561	tags=77%, list=33%, signal=114%
GOBP_PROTEIN_POLYMERIZATION		46	-0.4636679	-1.4618315	0.01030928	0.16550918	1	668	tags=70%, list=39%, signal=112%
REACTOME_ORC1_REMOVAL_FROM_CHROMATIN		24	-0.499663	-1.4604985	0.04123711	0.1672417	1	548	tags=63%, list=32%, signal=91%
GOBP_NEGATIVE_REGULATION_OF_WNT_SIGNALING_PATHWAY		30	-0.5062707	-1.45968	0.02083333	0.16773759	1	548	tags=63%, list=32%, signal=92%
GOBP_REGULATION_OF_RESPONSE_TO_OXIDATIVE_STRESS		14	-0.5332252	-1.4586263	0.03296704	0.1687054	1	561	tags=71%, list=33%, signal=106%
GOBP_PROTEASOMAL_PROTEIN_CATABOLIC_PROCESS		71	-0.4406975	-1.458616	0	0.16818088	1	548	tags=52%, list=32%, signal=74%
GOBP_COAGULATION		67	-0.4433622	-1.4585456	0.01010101	0.16769321	1	537	tags=52%, list=32%, signal=73%
REACTOME_MAPK6_MAPK4_SIGNALING		29	-0.5005758	-1.4584714	0.03030303	0.16724272	1	548	tags=59%, list=32%, signal=85%
GOBP_ORGAN_GROWTH		11	-0.5981382	-1.4580517	0.03614458	0.16726303	1	407	tags=55%, list=24%, signal=71%
GOBP_POSITIVE_REGULATION_OF_CELLULAR_COMPONENT_BIOGENESIS		69	-0.4391016	-1.4578537	0.01010101	0.16701646	1	643	tags=58%, list=38%, signal=90%
GOBP_CELLULAR_GLUCAN_METABOLIC_PROCESS		13	-0.5569426	-1.4577236	0.03488372	0.16667208	1	281	tags=54%, list=17%, signal=64%
REACTOME_SIGNALING_BY_ROBO_RECEPTORS		105	-0.4109082	-1.457561	0.01	0.16636385	1	851	tags=72%, list=50%, signal=136%
GOBP_SULFUR_COMPOUND_CATABOLIC_PROCESS		15	-0.5562532	-1.457268	0.04255319	0.16638595	1	260	tags=40%, list=15%, signal=47%
GOBP_ALPHA_BETA_T_CELL_ACTIVATION		11	-0.6029257	-1.4555691	0.01123596	0.16831157	1	573	tags=64%, list=34%, signal=96%
REACTOME_REGULATION_OF_EXPRESSION_OF_SLITS_AND_ROBOS		97	-0.4240114	-1.4533954	0	0.17110531	1	819	tags=72%, list=48%, signal=132%
REACTOME_SIGNALING_BY_HEDGEHOG		37	-0.4610212	-1.4526777	0.02040816	0.17153575	1	819	tags=78%, list=48%, signal=148%
GOBP_IMMUNE_SYSTEM_DEVELOPMENT		90	-0.4376718	-1.4515022	0	0.17267948	1	355	tags=38%, list=21%, signal=45%
REACTOME_CILIUM_ASSEMBLY		25	-0.5216754	-1.4507301	0.03125	0.17336015	1	567	tags=64%, list=33%, signal=95%
GOBP_CORTICAL_ACTIN_CYTOSKELETON_ORGANIZATION		11	-0.5894921	-1.4504709	0.04395605	0.17303622	1	402	tags=64%, list=24%, signal=83%
GOBP_REGULATION_OF_ANATOMICAL_STRUCTURE_SIZE		76	-0.4415679	-1.4503168	0.01	0.17271443	1	683	tags=64%, list=40%, signal=103%
GOBP_POSITIVE_REGULATION_OF_DEVELOPMENTAL_GROWTH		13	-0.5520688	-1.4499843	0.01086957	0.1726844	1	499	tags=62%, list=29%, signal=87%

GOBP_ENTRY_INTO_HOST		27	-0.4612222	-1.4499195	0.07216495	0.17223887	1	619	tags=59%, list=37%, signal=92%
GOBP_RIBOSOME_BIOGENESIS		50	-0.4401181	-1.4496567	0.01	0.17236692	1	737	tags=70%, list=43%, signal=120%
GOBP_IMMUNE_RESPONSE_REGULATING_SIGNALING_PATHWAY		52	-0.4481303	-1.4496235	0.02020202	0.17179805	1	824	tags=75%, list=49%, signal=141%
GOBP_REGULATION_OF_ACTIN_FILAMENT_ORGANIZATION		40	-0.4656633	-1.4490505	0.01	0.17227592	1	668	tags=65%, list=39%, signal=105%
REACTOME_NEGATIVE_REGULATION_OF_NOTCH4_SIGNALING		25	-0.5059825	-1.448385	0.02083333	0.17246762	1	548	tags=64%, list=32%, signal=93%
GOBP_POSITIVE_REGULATION_OF_NERVOUS_SYSTEM_DEVELOPMENT		21	-0.5201933	-1.4483118	0.08080808	0.17206053	1	547	tags=57%, list=32%, signal=83%
REACTOME_SIGNALING_BY_NOTCH4		26	-0.5014617	-1.4479665	0.01041667	0.17200181	1	548	tags=62%, list=32%, signal=90%
GOBP_CELL_SUBSTRATE_ADHESION		59	-0.4391155	-1.4469302	0	0.17294183	1	504	tags=47%, list=30%, signal=65%
REACTOME_CELLULAR_RESPONSE_TO_HYPOXIA		25	-0.5018137	-1.4468663	0.01030928	0.17253779	1	355	tags=48%, list=21%, signal=60%
GOBP_NEGATIVE_REGULATION_OF_CELL_PROJECTION_ORGANIZATION		22	-0.5172469	-1.446854	0.04123711	0.17198122	1	309	tags=41%, list=18%, signal=49%
GOBP_NEGATIVE_REGULATION_OF_CELLULAR_CATABOLIC_PROCESS		28	-0.4640165	-1.4464288	0.04123711	0.17220117	1	520	tags=50%, list=31%, signal=71%
GOBP_REGULATION_OF_PROTEIN_CATABOLIC_PROCESS		61	-0.4359627	-1.4464166	0.02	0.1716799	1	775	tags=67%, list=46%, signal=119%
GOBP_POSITIVE_REGULATION_OF_VASCULATURE_DEVELOPMENT		19	-0.5133643	-1.4461126	0.06315789	0.17156164	1	344	tags=42%, list=20%, signal=52%
GOBP_REGULATION_OF_EXTRINSIC_APOPTOTIC_SIGNALING_PATHWAY		21	-0.5128555	-1.4457754	0.03191489	0.17162633	1	683	tags=71%, list=40%, signal=118%
GOBP_EPIDERMIS_DEVELOPMENT		17	-0.5096383	-1.445673	0.07291666	0.1713267	1	294	tags=35%, list=17%, signal=42%
REACTOME_MITOTIC_PROMETAPHASE		19	-0.5075553	-1.4456613	0.04255319	0.17078452	1	560	tags=63%, list=33%, signal=93%
GOBP_POSITIVE_REGULATION_OF_CELLULAR_PROTEIN_LOCALIZATION		63	-0.438562	-1.4450574	0.02020202	0.17127666	1	629	tags=60%, list=37%, signal=92%
GOBP_NEGATIVE_REGULATION_OF_KINASE_ACTIVITY		32	-0.4656481	-1.4447759	0.04040404	0.17113128	1	687	tags=69%, list=41%, signal=113%
GOBP_REGULATION_OF_CELL_ADHESION		87	-0.4191274	-1.4443426	0.01	0.1712594	1	663	tags=59%, list=39%, signal=91%
GOBP_REACTIVE_NITROGEN_SPECIES_METABOLIC_PROCESS		17	-0.5115116	-1.4443392	0.04347826	0.17072421	1	386	tags=53%, list=23%, signal=68%
GOBP_RESPONSE_TO_VIRUS		36	-0.4797245	-1.4439664	0.02	0.17082088	1	564	tags=61%, list=33%, signal=90%
GOBP_REGULATION_OF_NEUROGENESIS		30	-0.4633423	-1.4436433	0.05102041	0.17073992	1	547	tags=50%, list=32%, signal=73%
REACTOME_SIGNALING_BY_RHO_GTPASES_MIRO_GTPASES_AND_RHOBTB3		113	-0.4060564	-1.4423267	0	0.17265517	1	707	tags=59%, list=42%, signal=95%
GOBP_PROTEIN_MODIFICATION_BY_SMALL_PROTEIN_CONJUGATION_OR_REI		81	-0.4310844	-1.4421223	0	0.17265631	1	750	tags=68%, list=44%, signal=116%
GOBP_TUBE_DEVELOPMENT		89	-0.4324846	-1.4413462	0.01	0.1733658	1	357	tags=37%, list=21%, signal=45%
GOBP_TOXIN_TRANSPORT		12	-0.5646775	-1.4409904	0.06593407	0.17342308	1	447	tags=58%, list=26%, signal=79%
GOBP_NEGATIVE_REGULATION_OF_CELL_CYCLE_PHASE_TRANSITION		30	-0.4660653	-1.4407991	0.03157895	0.17333394	1	548	tags=57%, list=32%, signal=82%
GOBP_REGULATION_OF_MICROTUBULE_BASED_PROCESS		17	-0.5112556	-1.4404016	0.05494506	0.17359732	1	539	tags=65%, list=32%, signal=94%
GOBP_POSITIVE_REGULATION_OF_ORGANELLE_ORGANIZATION		87	-0.4245324	-1.4398371	0	0.174356	1	586	tags=52%, list=35%, signal=75%
GOBP_POSITIVE_REGULATION_OF_BIOSYNTHETIC_PROCESS		138	-0.4097631	-1.4391885	0.01	0.17472984	1	384	tags=37%, list=23%, signal=44%
REACTOME_L1CAM_INTERACTIONS		34	-0.4574614	-1.4391261	0.03030303	0.17423089	1	800	tags=79%, list=47%, signal=147%
GOBP_ANTIGEN_PROCESSING_AND_PRESENTATION_OF_PEPTIDE_ANTIGEN		62	-0.4302143	-1.4384396	0.02	0.1749519	1	355	tags=37%, list=21%, signal=45%
GOBP_MONONUCLEAR_CELL_DIFFERENTIATION		21	-0.519848	-1.4377972	0.05208333	0.1756964	1	460	tags=52%, list=27%, signal=71%
GOBP_ACTIN_POLYMERIZATION_OR_DEPOLYMERIZATION		32	-0.473186	-1.4376357	0.06122449	0.17537248	1	668	tags=69%, list=39%, signal=111%
GOBP_CELLULAR_MACROMOLECULE_CATABOLIC_PROCESS		219	-0.4012048	-1.4366074	0	0.1768544	1	820	tags=67%, list=48%, signal=112%
GOBP_POSITIVE_REGULATION_OF_RESPONSE_TO_WOUNDING		19	-0.523792	-1.436358	0.05263158	0.17672758	1	504	tags=58%, list=30%, signal=81%
GOBP_CELLULAR_MODIFIED_AMINO_ACID_BIOSYNTHETIC_PROCESS		13	-0.5462673	-1.4356858	0.02197802	0.17720218	1	389	tags=54%, list=23%, signal=69%
GOBP_NEGATIVE_REGULATION_OF_CATALYTIC_ACTIVITY		101	-0.4230227	-1.4356842	0.01	0.17667793	1	687	tags=58%, list=41%, signal=92%
GOBP_CELL_MORPHOGENESIS		121	-0.4225894	-1.4343708	0	0.17865053	1	570	tags=50%, list=34%, signal=71%
REACTOME_SCF_SKP2_MEDIATED_DEGRADATION_OF_P27_P21		24	-0.499663	-1.4327961	0.01041667	0.18030284	1	548	tags=63%, list=32%, signal=91%
REACTOME_CLASS_I_MHC_MEDIATED_ANTIGEN_PROCESSING_PRESENTATIC		64	-0.4317224	-1.4314847	0	0.181749	1	644	tags=55%, list=38%, signal=85%
GOBP_POSITIVE_REGULATION_OF_CELL_DIFFERENTIATION		70	-0.421786	-1.4301544	0	0.1832977	1	570	tags=53%, list=34%, signal=76%
REACTOME_ECM_PROTEOGLYCANS		21	-0.5080617	-1.4299352	0.03061225	0.18309964	1	325	tags=48%, list=19%, signal=58%
GOBP_NEGATIVE_REGULATION_OF_PROTEIN_CONTAINING_COMPLEX_DISAS		14	-0.557842	-1.4296442	0.01136364	0.18295956	1	624	tags=86%, list=37%, signal=135%
GOBP_NEGATIVE_REGULATION_OF_UBIQUITIN_DEPENDENT_PROTEIN_CATAE		10	-0.586865	-1.4294881	0.04651163	0.18256871	1	172	tags=50%, list=10%, signal=55%
REACTOME_CLEC7A_DECTIN_1_SIGNALING		28	-0.4894987	-1.4292728	0.03092784	0.18220876	1	819	tags=89%, list=48%, signal=170%

GOBP_REGULATION_OF_ACTIN_FILAMENT_BASED_PROCESS	60	-0.4271362	-1.4292212	0.01	0.1817941	1	688	tags=58%, list=41%, signal=95%
GOBP_BIOLOGICAL_PROCESS_INVOLVED_IN_SYMBIOTIC_INTERACTION	216	-0.4006636	-1.429174	0	0.18132757	1	629	tags=53%, list=37%, signal=73%
GOBP_ANTIGEN_RECEPTOR_MEDIATED_SIGNALING_PATHWAY	35	-0.4695125	-1.4284527	0.01041667	0.18188094	1	819	tags=80%, list=48%, signal=152%
GOBP_REPRODUCTIVE_SYSTEM_DEVELOPMENT	26	-0.4904663	-1.4276922	0.02040816	0.18232235	1	475	tags=50%, list=28%, signal=68%
GOBP_ESTABLISHMENT_OR_MAINTENANCE_OF_CELL_POLARITY	33	-0.4670844	-1.4274276	0.02105263	0.18237813	1	651	tags=61%, list=38%, signal=96%
GOBP_NEGATIVE_REGULATION_OF_HYDROLASE_ACTIVITY	58	-0.4450457	-1.4270616	0.02020202	0.18235113	1	604	tags=50%, list=36%, signal=75%
GOBP_MACROMOLECULE_CATABOLIC_PROCESS	255	-0.3960187	-1.4268626	0	0.18207991	1	820	tags=65%, list=48%, signal=107%
GOBP_INTERLEUKIN_1_MEDIATED_SIGNALING_PATHWAY	25	-0.4886113	-1.4267576	0.08333334	0.18167444	1	576	tags=64%, list=34%, signal=96%
GOBP_RESPONSE_TO_CYTOKINE	144	-0.4059381	-1.4263964	0	0.18194798	1	576	tags=49%, list=34%, signal=68%
GOBP_NEGATIVE_REGULATION_OF_PROTEOLYSIS_INVOLVED_IN_CELLULAR	10	-0.586865	-1.4263698	0.03370787	0.1815182	1	172	tags=50%, list=10%, signal=55%
GOBP_EXTRINSIC_APOPTOTIC_SIGNALING_PATHWAY	21	-0.5128555	-1.4260275	0.05208333	0.18157649	1	683	tags=71%, list=40%, signal=118%
GOBP_IMPORT_INTO_NUCLEUS	13	-0.5570655	-1.4259299	0.06593407	0.18136513	1	395	tags=54%, list=23%, signal=70%
GOBP_NCRNA_PROCESSING	36	-0.4721367	-1.4258407	0.04123711	0.1809936	1	714	tags=69%, list=42%, signal=117%
GOBP_LAMELLIPODIUM_ORGANIZATION	15	-0.5441471	-1.4237473	0.02173913	0.18425624	1	341	tags=53%, list=20%, signal=66%
GOBP_COLLAGEN_METABOLIC_PROCESS	11	-0.5720301	-1.4236523	0.08045977	0.18379885	1	259	tags=45%, list=15%, signal=53%
REACTOME_ANCHORING_OF_THE_BASAL_BODY_TO_THE_PLASMA_MEMBRAN	15	-0.5415563	-1.4235882	0.05376344	0.18347704	1	515	tags=60%, list=30%, signal=85%
GOBP_TISSUE_MORPHOGENESIS	68	-0.4303913	-1.4234143	0.05	0.18336861	1	705	tags=56%, list=42%, signal=92%
GOBP_REGULATION_OF_SUPRAMOLECULAR_FIBER_ORGANIZATION	53	-0.4542164	-1.4227405	0.01	0.18381448	1	668	tags=64%, list=39%, signal=103%
REACTOME_INTERLEUKIN_1_SIGNALING	28	-0.4743824	-1.4227105	0.04123711	0.18333712	1	199	tags=36%, list=12%, signal=40%
REACTOME_DISEASES_OF_GLYCOSYLATION	14	-0.5492216	-1.4225415	0.06593407	0.18315102	1	260	tags=43%, list=15%, signal=50%
REACTOME_DEFECTIVE_CFTR_CAUSES_CYSTIC_FIBROSIS	27	-0.4646822	-1.421781	0.03061225	0.18398649	1	192	tags=33%, list=11%, signal=37%
GOBP_NEGATIVE_REGULATION_OF_CATABOLIC_PROCESS	34	-0.4478384	-1.4217405	0.02040816	0.18356493	1	520	tags=47%, list=31%, signal=67%
GOBP_NUCLEAR_ENVELOPE_ORGANIZATION	10	-0.5778706	-1.4216866	0.05952381	0.18314561	1	193	tags=40%, list=11%, signal=45%
GOBP_ANIMAL_ORGAN_MORPHOGENESIS	87	-0.4235587	-1.4205904	0.01	0.1845729	1	355	tags=33%, list=21%, signal=40%
REACTOME_ANTIGEN_PROCESSING_UBIQUITINATION_PROTEASOME_DEGRAD	37	-0.4671072	-1.4188628	0.04040404	0.18684761	1	355	tags=43%, list=21%, signal=54%
GOBP_NEGATIVE_REGULATION_OF_PROTEIN_POLYMERIZATION	17	-0.5228242	-1.4185468	0.04395605	0.18696567	1	624	tags=76%, list=37%, signal=120%
GOBP_POSITIVE_REGULATION_OF_UBIQUITIN_DEPENDENT_PROTEIN_CATAB	13	-0.5469536	-1.4181114	0.06593407	0.18713512	1	699	tags=77%, list=41%, signal=130%
REACTOME_PLATELET_ACTIVATION_SIGNALING_AND_AGGREGATION	75	-0.4196447	-1.4177525	0	0.18732865	1	425	tags=41%, list=25%, signal=53%
GOBP_ENDOPLASMIC_RETICULUM_UNFOLDED_PROTEIN_RESPONSE	30	-0.4430853	-1.4158543	0.06185567	0.18987763	1	636	tags=57%, list=38%, signal=89%
REACTOME_MAPK_FAMILY_SIGNALING_CASCADES	62	-0.4236028	-1.4157472	0.01	0.18944904	1	857	tags=74%, list=51%, signal=145%
REACTOME_EXTRACELLULAR_MATRIX_ORGANIZATION	49	-0.4415807	-1.4156362	0.02040816	0.18912506	1	503	tags=51%, list=30%, signal=70%
GOBP_PROTEIN_MODIFICATION_BY_SMALL_PROTEIN_CONJUGATION	73	-0.4334112	-1.4155182	0	0.18892972	1	750	tags=68%, list=44%, signal=118%
GOBP_RESPONSE_TO_INTERLEUKIN_1	30	-0.4789801	-1.4154792	0	0.18850759	1	576	tags=63%, list=34%, signal=94%
GOBP_T_CELL_DIFFERENTIATION	18	-0.5271892	-1.4153954	0.03333334	0.18818797	1	460	tags=50%, list=27%, signal=68%
GOBP_NAD_METABOLIC_PROCESS	23	-0.4698162	-1.415351	0.08247422	0.18774448	1	552	tags=61%, list=33%, signal=89%
REACTOME_DNA_REPLICATION	24	-0.499663	-1.4136364	0.04081633	0.1898444	1	548	tags=63%, list=32%, signal=91%
GOBP_REGULATION_OF_CELLULAR_COMPONENT_BIOGENESIS	126	-0.3937322	-1.4130943	0	0.19032738	1	653	tags=54%, list=39%, signal=81%
GOBP_NUCLEAR_EXPORT	14	-0.5201701	-1.4125407	0.08888889	0.19065781	1	536	tags=64%, list=32%, signal=93%
GOBP_IRE1_MEDIATED_UNFOLDED_PROTEIN_RESPONSE	21	-0.4993564	-1.4121441	0.04301075	0.19108765	1	636	tags=62%, list=38%, signal=98%
GOBP_POSITIVE_REGULATION_OF_ORGANELLE_ASSEMBLY	11	-0.5793002	-1.4120619	0.06097561	0.19081634	1	515	tags=73%, list=30%, signal=104%
GOBP_INTERLEUKIN_2_PRODUCTION	10	-0.58644	-1.4119405	0.04705882	0.19049628	1	352	tags=50%, list=21%, signal=63%
GOBP_NEGATIVE_REGULATION_OF_CELL_CYCLE	53	-0.4270679	-1.4119004	0.04040404	0.19010438	1	267	tags=32%, list=16%, signal=37%
GOBP_FOCAL_ADHESION_ASSEMBLY	18	-0.5019277	-1.4104629	0.08791209	0.19226043	1	558	tags=56%, list=33%, signal=82%
GOBP_CANONICAL_WNT_SIGNALING_PATHWAY	35	-0.4619256	-1.4098637	0.02020202	0.19265483	1	548	tags=54%, list=32%, signal=79%
GOBP_REGULATION_OF_DNA_BIOSYNTHETIC_PROCESS	13	-0.5367093	-1.4078431	0.1	0.1959002	1	376	tags=54%, list=22%, signal=69%
GOBP_RESPONSE_TO_TOPOLOGICALLY_INCORRECT_PROTEIN	54	-0.4385343	-1.407715	0	0.19562156	1	636	tags=59%, list=38%, signal=92%

REACTOME_DEGRADATION_OF_GLI1_BY_THE_PROTEASOME		25	-0.4961679	-1.4075828	0.05102041	0.19529502	1	548	tags=60%, list=32%, signal=87%
GOBP_ANTIGEN_PROCESSING_AND_PRESENTATION		69	-0.4191504	-1.4073266	0.01	0.19518906	1	355	tags=35%, list=21%, signal=42%
GOBP_POSITIVE_REGULATION_OF_I_KAPPAB_KINASE_NF_KAPPAB_SIGNALI		18	-0.5171022	-1.4068383	0.05263158	0.19559643	1	406	tags=50%, list=24%, signal=65%
GOBP_DNA_BIOSYNTHETIC_PROCESS		19	-0.5080614	-1.4059992	0.04255319	0.19643642	1	376	tags=47%, list=22%, signal=60%
GOBP_POSITIVE_REGULATION_OF_GENE_EXPRESSION		110	-0.4073636	-1.4055024	0	0.19652249	1	725	tags=61%, list=43%, signal=100%
REACTOME_ASSOCIATION_OF_TRIC_CCT_WITH_TARGET_PROTEINS_DURING_		10	-0.5953875	-1.4049729	0.04761905	0.19701792	1	376	tags=60%, list=22%, signal=77%
REACTOME_INFECTIOUS_DISEASE		196	-0.3933252	-1.404933	0	0.1966448	1	819	tags=65%, list=48%, signal=111%
GOBP_RNA_LOCALIZATION		22	-0.5167272	-1.4039501	0.05154639	0.19776116	1	567	tags=68%, list=33%, signal=101%
GOBP_POSITIVE_REGULATION_OF_PROTEOLYSIS_INVOLVED_IN_CELLULAR_		19	-0.4944358	-1.403514	0.05376344	0.19805843	1	699	tags=74%, list=41%, signal=124%
GOBP_RESPONSE_TO_CORTICOSTEROID		17	-0.4908116	-1.4031808	0.03191489	0.19825998	1	706	tags=65%, list=42%, signal=110%
GOBP_POSITIVE_REGULATION_OF_CELLULAR_COMPONENT_ORGANIZATION		163	-0.391436	-1.4026244	0	0.19869865	1	629	tags=49%, list=37%, signal=71%
REACTOME_GLYCOSAMINOGLYCAN_METABOLISM		12	-0.5675718	-1.401302	0.03614458	0.20034909	1	341	tags=50%, list=20%, signal=62%
GOBP_REGULATION_OF_INTRINSIC_APOPTOTIC_SIGNALING_PATHWAY		33	-0.469066	-1.400294	0.03092784	0.20161033	1	714	tags=67%, list=42%, signal=113%
GOBP_POSITIVE_REGULATION_OF_BINDING		32	-0.4641603	-1.400126	0.03191489	0.20127998	1	520	tags=53%, list=31%, signal=75%
GOBP_REGULATION_OF_CELLULAR_CATABOLIC_PROCESS		139	-0.4073116	-1.3996052	0.01	0.20177749	1	581	tags=49%, list=34%, signal=68%
GOBP_CILIUM_ORGANIZATION		28	-0.4722295	-1.3994908	0.03125	0.20149499	1	515	tags=54%, list=30%, signal=76%
GOBP_REGULATION_OF_ACTOMYOSIN_STRUCTURE_ORGANIZATION		13	-0.5420687	-1.39939	0.05376344	0.20104927	1	504	tags=54%, list=30%, signal=76%
GOBP_POSITIVE_REGULATION_OF_PROTEIN_POLYMERIZATION		18	-0.5156627	-1.3990319	0.04301075	0.20102707	1	668	tags=78%, list=39%, signal=127%
GOBP_NEGATIVE_REGULATION_OF_CELL_CYCLE_PROCESS		34	-0.4807566	-1.3989266	0.06122449	0.20072491	1	193	tags=35%, list=11%, signal=39%
REACTOME_INTERLEUKIN_1_FAMILY_SIGNALING		28	-0.4743824	-1.3989149	0.07142858	0.20026104	1	199	tags=36%, list=12%, signal=40%
GOBP_NEGATIVE_REGULATION_OF_PROTEIN_CATABOLIC_PROCESS		18	-0.5070934	-1.3982441	0.11111111	0.20089273	1	520	tags=56%, list=31%, signal=79%
GOBP_PLATELET_DEGRANULATION		47	-0.4345526	-1.3981739	0.05102041	0.20045383	1	425	tags=45%, list=25%, signal=58%
GOBP_ESTABLISHMENT_OF_PROTEIN_LOCALIZATION_TO_VACUOLE		10	-0.5572947	-1.3974359	0.05813954	0.20129243	1	461	tags=60%, list=27%, signal=82%
GOBP_ACTIN_FILAMENT_DEPOLYMERIZATION		19	-0.5144001	-1.396661	0.03225806	0.20175557	1	786	tags=89%, list=46%, signal=165%
GOBP_REGULATION_OF_MITOTIC_CELL_CYCLE		60	-0.4231343	-1.3958138	0.02	0.20290814	1	558	tags=52%, list=33%, signal=74%
GOBP_REGULATION_OF_VASCULATURE_DEVELOPMENT		37	-0.4546967	-1.3956331	0.05102041	0.20260647	1	344	tags=38%, list=20%, signal=46%
GOBP_TRANSLATIONAL_INITIATION		89	-0.4065017	-1.3955415	0.02	0.20235345	1	638	tags=56%, list=38%, signal=85%
BIOCARTA_INTEGRIN_PATHWAY		16	-0.4960764	-1.3951484	0.05494506	0.20258072	1	221	tags=31%, list=13%, signal=36%
GOBP_REGULATION_OF_CELLULAR_PROTEIN_CATABOLIC_PROCESS		44	-0.4589386	-1.3950734	0.04	0.20221426	1	775	tags=75%, list=46%, signal=135%
GOBP_PROTEIN_TARGETING_TO_MEMBRANE		92	-0.4026024	-1.3949689	0.01	0.2019855	1	817	tags=66%, list=48%, signal=121%
REACTOME_FCERI_MEDIATED_NF_KB_ACTIVATION		28	-0.4850871	-1.3945278	0.06122449	0.20228136	1	192	tags=36%, list=11%, signal=40%
REACTOME_RESPONSE_TO_ELEVATED_PLATELET_CYTOSOLIC_CA2		52	-0.442879	-1.3934788	0.05	0.20386654	1	425	tags=46%, list=25%, signal=60%
GOBP_NEGATIVE_REGULATION_OF_APOPTOTIC_SIGNALING_PATHWAY		38	-0.4377666	-1.3931732	0.02040816	0.20388354	1	683	tags=68%, list=40%, signal=112%
GOBP_MEMBRANE_DOCKING		38	-0.4301445	-1.3929473	0.05208333	0.20369859	1	640	tags=53%, list=38%, signal=83%
GOBP_POSITIVE_REGULATION_OF_CELL_DEVELOPMENT		42	-0.4399169	-1.3922749	0.03030303	0.20450468	1	570	tags=55%, list=34%, signal=80%
GOBP_REGULATION_OF_PROTEIN_CONTAINING_COMPLEX_DISASSEMBLY		25	-0.4808103	-1.392169	0.05376344	0.2041393	1	786	tags=80%, list=46%, signal=147%
GOBP_REGULATION_OF_CATABOLIC_PROCESS		150	-0.3977712	-1.3916491	0	0.20453899	1	705	tags=56%, list=42%, signal=87%
GOBP_PEPTIDYL_AMINO_ACID_MODIFICATION		105	-0.4142732	-1.3915988	0	0.20413017	1	564	tags=50%, list=33%, signal=70%
GOBP_PROTEIN_LOCALIZATION_TO_NUCLEUS		36	-0.4527933	-1.391347	0.05154639	0.2039912	1	520	tags=56%, list=31%, signal=78%
GOBP_TELENCEPHALON_DEVELOPMENT		16	-0.5063234	-1.3907483	0.07526882	0.20447564	1	237	tags=31%, list=14%, signal=36%
REACTOME_TRANSCRIPTIONAL_REGULATION_BY_RUNX2		27	-0.4877925	-1.3906895	0.03092784	0.20413664	1	819	tags=89%, list=48%, signal=169%
GOBP_POSITIVE_REGULATION_OF_SIGNALING		169	-0.3878832	-1.3899614	0	0.20488526	1	406	tags=38%, list=24%, signal=45%
GOBP_POSITIVE_REGULATION_OF_GTPASE_ACTIVITY		24	-0.4712169	-1.3889635	0.06382979	0.20600295	1	362	tags=42%, list=21%, signal=52%
GOBP_ESTABLISHMENT_OF_CELL_POLARITY		17	-0.4918985	-1.3889049	0.03333334	0.20568459	1	473	tags=53%, list=28%, signal=73%
GOBP_NEGATIVE_REGULATION_OF_CYTOKINE_PRODUCTION		27	-0.4576944	-1.3879267	0.0625	0.20653538	1	391	tags=44%, list=23%, signal=57%
GOBP_IMMUNE_EFFECTOR_PROCESS		197	-0.3862239	-1.3872058	0	0.20742407	1	656	tags=52%, list=39%, signal=75%

GOBP_REGULATION_OF_CELLULAR_PROTEIN_LOCALIZATION		104	-0.4072335	-1.3870767	0.01020408	0.20719256	1	607	tags=54%, list=36%, signal=79%
REACTOME_EUKARYOTIC_TRANSLATION_INITIATION		81	-0.4106625	-1.3857342	0	0.20894931	1	817	tags=70%, list=48%, signal=129%
GOBP_VIRAL_LIFE_CYCLE		58	-0.4197127	-1.3857218	0.05154639	0.2084755	1	619	tags=53%, list=37%, signal=81%
GOBP_SEXUAL_REPRODUCTION		40	-0.441351	-1.3827063	0.04040404	0.21330787	1	242	tags=30%, list=14%, signal=34%
GOBP_REGULATION_OF_CELL_DEVELOPMENT		59	-0.4138932	-1.3824503	0.02	0.2134559	1	653	tags=56%, list=39%, signal=88%
REACTOME_G_ALPHA_Q_SIGNALLING_EVENTS		14	-0.5397882	-1.3816969	0.07692308	0.21414407	1	367	tags=43%, list=22%, signal=54%
GOBP_REPRODUCTION		75	-0.4200428	-1.3816694	0.01	0.21370603	1	518	tags=43%, list=31%, signal=59%
GOBP_POSITIVE_REGULATION_OF_ACTIN_FILAMENT_BUNDLE_ASSEMBLY		11	-0.5553963	-1.3815963	0.10227273	0.21335596	1	504	tags=55%, list=30%, signal=77%
GOBP_NEGATIVE_REGULATION_OF_ORGANELLE_ORGANIZATION		38	-0.4446944	-1.3814795	0.05102041	0.21307229	1	570	tags=55%, list=34%, signal=81%
GOBP_GENE_SILENCING		21	-0.4844117	-1.3812217	0.06451613	0.2130684	1	518	tags=52%, list=31%, signal=74%
GOBP_NEGATIVE_REGULATION_OF_MITOTIC_CELL_CYCLE		33	-0.4444324	-1.3811878	0.06315789	0.21265824	1	193	tags=30%, list=11%, signal=34%
KEGG_REGULATION_OF_ACTIN_CYTOSKELETON		43	-0.434396	-1.3809559	0.03092784	0.21263482	1	688	tags=67%, list=41%, signal=111%
GOBP_REGULATION_OF_RESPONSE_TO_ENDOPLASMIC_RETICULUM_STRESS		15	-0.5222178	-1.3797026	0.08695652	0.21431676	1	619	tags=67%, list=37%, signal=104%
GOBP_RIBONUCLEOPROTEIN_COMPLEX_SUBUNIT_ORGANIZATION		35	-0.455425	-1.377945	0.05102041	0.21728916	1	737	tags=69%, list=43%, signal=119%
GOBP_AMYLOID_BETA_CLEARANCE		13	-0.5132206	-1.3778131	0.12222222	0.2171706	1	537	tags=69%, list=32%, signal=101%
GOBP_CELLULAR_RESPONSE_TO_RADIATION		17	-0.5068475	-1.3776828	0.09375	0.21686171	1	475	tags=53%, list=28%, signal=73%
REACTOME_CELLULAR_RESPONSES_TO_EXTERNAL_STIMULI		192	-0.3836335	-1.3775948	0	0.21657546	1	819	tags=65%, list=48%, signal=112%
GOBP_REGULATION_OF_CELL_DIFFERENTIATION		142	-0.3980507	-1.3775133	0	0.21624818	1	550	tags=46%, list=32%, signal=62%
GOBP_NCRNA_METABOLIC_PROCESS		57	-0.4277461	-1.3767827	0.02020202	0.21697497	1	601	tags=56%, list=35%, signal=84%
KEGG_PROTEASOME		20	-0.5168135	-1.3761194	0.02083333	0.21765499	1	548	tags=65%, list=32%, signal=95%
GOBP_CARBOHYDRATE_DERIVATIVE_CATABOLIC_PROCESS		30	-0.4712156	-1.375573	0.06185567	0.21795434	1	495	tags=43%, list=29%, signal=60%
REACTOME_RESOLUTION_OF_SISTER_CHROMATID_COHESION		12	-0.5311052	-1.375332	0.06976745	0.21789856	1	759	tags=83%, list=45%, signal=150%
GOBP_CARDIAC_CHAMBER_DEVELOPMENT		14	-0.5302595	-1.3751659	0.08510638	0.21761335	1	115	tags=21%, list=7%, signal=23%
BIOCARTA_UCALPAIN_PATHWAY		12	-0.5257343	-1.3733623	0.11363637	0.22074094	1	293	tags=33%, list=17%, signal=40%
REACTOME_G_ALPHA_I_SIGNALLING_EVENTS		26	-0.4641832	-1.3730936	0.09375	0.22078401	1	367	tags=38%, list=22%, signal=48%
REACTOME_SMOOTH_MUSCLE_CONTRACTION		16	-0.5080563	-1.3729793	0.07291666	0.22049403	1	459	tags=63%, list=27%, signal=85%
REACTOME_DEUBIQUITINATION		38	-0.4236394	-1.3729322	0.06060606	0.22010215	1	548	tags=53%, list=32%, signal=76%
REACTOME_FC_EPSILON_RECEPTOR_FCERI_SIGNALING		31	-0.4647149	-1.372761	0.07070707	0.21987766	1	576	tags=58%, list=34%, signal=86%
GOBP_DNA_METABOLIC_PROCESS		55	-0.413059	-1.372576	0.03030303	0.21973555	1	753	tags=67%, list=44%, signal=117%
GOBP_REGULATION_OF_PROTEIN_MODIFICATION_BY_SMALL_PROTEIN_CON		28	-0.4630156	-1.3723946	0.06315789	0.21973756	1	724	tags=71%, list=43%, signal=123%
GOBP_POSITIVE_REGULATION_OF_RESPONSE_TO_BIOTIC_STIMULUS		40	-0.4384709	-1.3715103	0.05050505	0.22084814	1	564	tags=50%, list=33%, signal=73%
GOBP_POSITIVE_REGULATION_OF_DNA_METABOLIC_PROCESS		19	-0.4760322	-1.371458	0.08421053	0.22037826	1	726	tags=74%, list=43%, signal=127%
GOBP_RESPONSE_TO_ACID_CHEMICAL		14	-0.5221421	-1.3711317	0.06818182	0.22054334	1	259	tags=43%, list=15%, signal=50%
GOBP_REGULATION_OF_CELL_CELL_ADHESION		48	-0.4131254	-1.3709704	0.01	0.22046243	1	659	tags=58%, list=39%, signal=93%
GOBP_RNA_SPLICING_VIA_TRANSESTERIFICATION_REACTIONS		12	-0.5766435	-1.3709556	0.07608695	0.2200576	1	401	tags=67%, list=24%, signal=87%
GOBP_PEPTIDYL_SERINE_MODIFICATION		30	-0.4511864	-1.3708274	0.06185567	0.21981637	1	564	tags=53%, list=33%, signal=79%
GOBP_CARDIAC_MUSCLE_CELL_MEMBRANE_REPOLARIZATION		11	-0.5557105	-1.3702941	0.09302326	0.2201208	1	287	tags=36%, list=17%, signal=43%
GOBP_POSITIVE_REGULATION_OF_RESPONSE_TO_EXTERNAL_STIMULUS		67	-0.4126294	-1.3702611	0.01	0.21967845	1	564	tags=52%, list=33%, signal=75%
GOBP_CHROMOSOME_ORGANIZATION		54	-0.4137546	-1.3701006	0.03061225	0.21964112	1	576	tags=50%, list=34%, signal=73%
REACTOME_INFLUENZA_INFECTION		82	-0.4096993	-1.3688208	0	0.22173375	1	852	tags=74%, list=50%, signal=142%
GOBP_POSITIVE_REGULATION_OF_TRANSCRIPTION_BY_RNA_POLYMERASE_		43	-0.4484706	-1.3685578	0.02040816	0.22159225	1	405	tags=42%, list=24%, signal=54%
GOBP_REGULATION_OF_PROTEOLYSIS		99	-0.3932001	-1.3677709	0.02	0.22235385	1	520	tags=41%, list=31%, signal=56%
KEGG_GAP_JUNCTION		14	-0.5199558	-1.366872	0.06741573	0.22351171	1	199	tags=36%, list=12%, signal=40%
GOBP_PROTEIN_TARGETING_TO_VACUOLE		10	-0.5572947	-1.3666755	0.05555556	0.22340782	1	461	tags=60%, list=27%, signal=82%
GOBP_STEROID_HORMONE_MEDIATED_SIGNALING_PATHWAY		11	-0.5252276	-1.3666056	0.10714286	0.22306482	1	491	tags=55%, list=29%, signal=76%
REACTOME_HCMV_INFECTION		13	-0.5232621	-1.3663917	0.07777778	0.22304153	1	759	tags=77%, list=45%, signal=138%

GOBP_CARBOHYDRATE_BIOSYNTHETIC_PROCESS		34	-0.4465852	-1.3655173	0.07070707	0.22418623	1	281	tags=41%, list=17%, signal=48%
GOBP_REGULATION_OF_REACTIVE_OXYGEN_SPECIES_BIOSYNTHETIC_PROC		16	-0.5026015	-1.3642882	0.05434782	0.22607921	1	619	tags=75%, list=37%, signal=117%
GOBP_REGULATION_OF_BODY_FLUID_LEVELS		83	-0.3922207	-1.3641216	0	0.22577305	1	537	tags=46%, list=32%, signal=64%
REACTOME_UB_SPECIFIC_PROCESSING_PROTEASES		35	-0.4353128	-1.3637997	0.0625	0.22588184	1	548	tags=54%, list=32%, signal=79%
REACTOME_CELL_SURFACE_INTERACTIONS_AT_THE_VASCULAR_WALL		29	-0.4613089	-1.3629906	0.02020202	0.22685531	1	432	tags=45%, list=25%, signal=59%
GOBP_CELL_SUBSTRATE_JUNCTION_ORGANIZATION		24	-0.4671418	-1.3619167	0.0625	0.22782399	1	558	tags=54%, list=33%, signal=80%
GOBP_REGULATION_OF_UBIQUITIN_PROTEIN_TRANSFERASE_ACTIVITY		12	-0.5278139	-1.360907	0.06818182	0.22918041	1	724	tags=92%, list=43%, signal=159%
REACTOME_ANTIGEN_PROCESSING_CROSS_PRESENTATION		43	-0.4501075	-1.3606787	0.03	0.22908573	1	393	tags=37%, list=23%, signal=47%
GOBP_NEGATIVE_REGULATION_OF_CELLULAR_PROTEIN_CATABOLIC_PROCE		12	-0.5466754	-1.3604105	0.08602151	0.22901152	1	520	tags=67%, list=31%, signal=95%
KEGG_FC_GAMMA_R_MEDIATED_PHAGOCYTOSIS		16	-0.480285	-1.3602291	0.09782609	0.22881986	1	851	tags=94%, list=50%, signal=187%
GOBP_POSITIVE_REGULATION_OF_MULTICELLULAR_ORGANISMAL_PROCESS		138	-0.3813097	-1.3598638	0.01	0.22901757	1	552	tags=45%, list=33%, signal=61%
GOBP_CELL_POPULATION_PROLIFERATION		140	-0.384191	-1.3593254	0.01	0.22942749	1	278	tags=29%, list=16%, signal=32%
GOBP_REGULATION_OF_APOPTOTIC_SIGNALING_PATHWAY		57	-0.4200442	-1.3588109	0.01	0.22991285	1	683	tags=61%, list=40%, signal=99%
GOBP_ENERGY_RESERVE_METABOLIC_PROCESS		14	-0.5439643	-1.3583931	0.09574468	0.23028013	1	281	tags=50%, list=17%, signal=59%
GOBP_CELL_CELL_JUNCTION_ORGANIZATION		28	-0.4456208	-1.358301	0.06122449	0.22993399	1	653	tags=61%, list=39%, signal=97%
GOBP_REGULATION_OF_CELLULAR_RESPONSE_TO_STRESS		80	-0.4098709	-1.3579687	0.03030303	0.23010914	1	662	tags=59%, list=39%, signal=92%
GOBP_CELLULAR_RESPONSE_TO_TOPOLOGICALLY_INCORRECT_PROTEIN		38	-0.4417819	-1.3579049	0.05	0.22970751	1	636	tags=58%, list=38%, signal=91%
GOBP_ESTABLISHMENT_OF_PROTEIN_LOCALIZATION_TO_ENDOPLASMIC_RE		80	-0.3971733	-1.3577278	0.02	0.22963274	1	817	tags=66%, list=48%, signal=122%
GOBP_FC_RECEPTOR_MEDIATED_STIMULATORY_SIGNALING_PATHWAY		15	-0.5123041	-1.3577118	0.14444445	0.2291762	1	386	tags=53%, list=23%, signal=68%
REACTOME_INNATE_IMMUNE_SYSTEM		199	-0.3779927	-1.3576623	0	0.22875954	1	656	tags=51%, list=39%, signal=73%
REACTOME_DISEASES_OF_SIGNAL_TRANSDUCTION_BY_GROWTH_FACTOR_R		64	-0.3970093	-1.3570673	0.04081633	0.2293145	1	420	tags=38%, list=25%, signal=48%
GOBP_ACUTE_INFLAMMATORY_RESPONSE		11	-0.5112998	-1.3568214	0.0952381	0.22943233	1	656	tags=73%, list=39%, signal=118%
GOBP_NUCLEOTIDE_PHOSPHORYLATION		25	-0.4492528	-1.356677	0.09574468	0.22935969	1	873	tags=84%, list=52%, signal=171%
REACTOME_ADAPTIVE_IMMUNE_SYSTEM		120	-0.3932424	-1.3566177	0.02	0.22902182	1	644	tags=51%, list=38%, signal=76%
GOBP_REGULATION_OF_PEPTIDASE_ACTIVITY		65	-0.4118597	-1.3565986	0.03	0.22864738	1	356	tags=32%, list=21%, signal=39%
GOBP_REGULATION_OF_ACTIN_FILAMENT_BUNDLE_ASSEMBLY		14	-0.5324301	-1.3562487	0.07692308	0.22884178	1	534	tags=57%, list=32%, signal=83%
GOBP_PROTEIN_PHOSPHOPANTETHEINYLATION		31	-0.4387449	-1.356083	0.04166667	0.22873344	1	448	tags=45%, list=26%, signal=60%
GOBP_REGULATION_OF_MULTICELLULAR_ORGANISMAL_DEVELOPMENT		109	-0.3868184	-1.3555589	0.03	0.22907566	1	655	tags=50%, list=39%, signal=77%
GOBP_EXOCYTOSIS		204	-0.391531	-1.3548975	0	0.22986594	1	658	tags=52%, list=39%, signal=75%
REACTOME_NEDDYLATION		34	-0.4423707	-1.3545495	0.07	0.22999872	1	355	tags=41%, list=21%, signal=51%
REACTOME_RECYCLING_PATHWAY_OF_L1		14	-0.5005166	-1.353892	0.08421053	0.23080319	1	800	tags=93%, list=47%, signal=174%
GOBP_POSITIVE_REGULATION_OF_PROTEIN_CATABOLIC_PROCESS		37	-0.4407178	-1.3538588	0.03030303	0.23035592	1	699	tags=65%, list=41%, signal=108%
GOBP_POSITIVE_REGULATION_OF_INTRACELLULAR_SIGNAL_TRANSDUCTION		96	-0.3944764	-1.3522081	0.01	0.23269702	1	406	tags=39%, list=24%, signal=48%
REACTOME_GLYCOGEN_METABOLISM		11	-0.5671948	-1.3493867	0.07228915	0.23768835	1	240	tags=55%, list=14%, signal=63%
GOBP_POSITIVE_REGULATION_OF_CELLULAR_PROTEIN_CATABOLIC_PROCE		28	-0.4381633	-1.348584	0.08163265	0.23873012	1	699	tags=68%, list=41%, signal=114%
GOBP_POSITIVE_REGULATION_OF_PROTEASOMAL_UBIQUITIN_DEPENDENT_I		12	-0.539112	-1.348453	0.11111111	0.23851158	1	699	tags=75%, list=41%, signal=127%
GOBP_CELLULAR_RESPONSE_TO_LIPID		44	-0.4360965	-1.3482343	0.05102041	0.23847818	1	313	tags=34%, list=18%, signal=41%
GOBP_REGULATION_OF_DNA_BINDING		13	-0.5146106	-1.3482187	0.12222222	0.23803979	1	366	tags=46%, list=22%, signal=58%
REACTOME_APOPTOSIS		50	-0.4229772	-1.3469709	0.05102041	0.23984568	1	570	tags=50%, list=34%, signal=73%
GOBP_SKELETAL_SYSTEM_DEVELOPMENT		35	-0.421411	-1.346791	0.09090909	0.23969965	1	459	tags=40%, list=27%, signal=54%
REACTOME_LEISHMANIA_INFECTION		28	-0.4440337	-1.3460711	0.09183674	0.24052465	1	367	tags=43%, list=22%, signal=54%
KEGG_APOPTOSIS		11	-0.5324522	-1.3457552	0.08988764	0.24087238	1	557	tags=55%, list=33%, signal=81%
GOBP_REGULATION_OF_RNA_SPLICING		11	-0.510518	-1.3455836	0.11235955	0.24078028	1	468	tags=73%, list=28%, signal=100%
GOBP_GLYCOLYTIC_PROCESS_THROUGH_FRUCTOSE_6_PHOSPHATE		14	-0.5050565	-1.3454903	0.10227273	0.24045172	1	847	tags=100%, list=50%, signal=198%
REACTOME_HIV_INFECTION		45	-0.4223777	-1.3454072	0.06	0.24008784	1	548	tags=51%, list=32%, signal=74%
REACTOME_APOPTOTIC_EXECUTION_PHASE		16	-0.4841647	-1.3441718	0.13043478	0.24181025	1	570	tags=50%, list=34%, signal=75%

KEGG_FOCAL_ADHESION		45	-0.4285586	-1.3435228	0.04	0.2427878	1	456	tags=47%, list=27%, signal=62%
GOBP_POSITIVE_REGULATION_OF_ESTABLISHMENT_OF_PROTEIN_LOCALIZA		62	-0.3950214	-1.3433193	0.07070707	0.24274684	1	595	tags=47%, list=35%, signal=69%
GOBP_CELL_ACTIVATION_INVOLVED_IN_IMMUNE_RESPONSE		153	-0.379171	-1.3423277	0.01	0.2445633	1	656	tags=52%, list=39%, signal=77%
GOBP_REGULATION_OF_RESPONSE_TO_EXTERNAL_STIMULUS		119	-0.3913488	-1.3418438	0.01	0.24502513	1	620	tags=52%, list=37%, signal=76%
GOBP_NEGATIVE_REGULATION_OF_CELL_DEATH		123	-0.3819984	-1.3414532	0	0.24532168	1	683	tags=58%, list=40%, signal=90%
GOBP_REGULATION_OF_SIGNAL_TRANSDUCTION_BY_P53_CLASS_MEDIATOR		15	-0.4935905	-1.3413833	0.08888889	0.24504344	1	724	tags=80%, list=43%, signal=138%
GOBP_ENDOSOMAL_TRANSPORT		36	-0.4287163	-1.3395921	0.06122449	0.24827448	1	425	tags=33%, list=25%, signal=44%
GOBP_CARDIAC_MUSCLE_CELL_ACTION_POTENTIAL_INVOLVED_IN_CONTRAC		10	-0.5380194	-1.3395865	0.07594936	0.247813	1	629	tags=60%, list=37%, signal=95%
REACTOME_AGGREPHAGY		13	-0.5163273	-1.3392575	0.0989011	0.24785326	1	805	tags=92%, list=47%, signal=174%
GOBP_NEGATIVE_REGULATION_OF_PROTEIN_CONTAINING_COMPLEX_ASSEM		28	-0.4517244	-1.3392226	0.11340206	0.24744792	1	640	tags=61%, list=38%, signal=96%
GOBP_REGULATION_OF_RESPONSE_TO_BIOTIC_STIMULUS		55	-0.4081825	-1.3389032	0	0.24761268	1	618	tags=55%, list=36%, signal=83%
GOBP_CELLULAR_PROTEIN_CATABOLIC_PROCESS		121	-0.385365	-1.3388515	0.02	0.24719132	1	828	tags=69%, list=49%, signal=125%
GOBP_RESPONSE_TO_TRANSFORMING_GROWTH_FACTOR_BETA		21	-0.4777178	-1.338646	0.0927835	0.24693057	1	628	tags=62%, list=37%, signal=97%
GOBP_CELLULAR_RESPONSE_TO_ABIOTIC_STIMULUS		33	-0.4239309	-1.3380603	0.08602151	0.24755564	1	405	tags=36%, list=24%, signal=47%
REACTOME_EUKARYOTIC_TRANSLATION_ELONGATION		77	-0.3971539	-1.3374791	0.01	0.24833696	1	850	tags=71%, list=50%, signal=137%
REACTOME_CELLULAR_RESPONSE_TO_STARVATION		82	-0.3955167	-1.3374503	0.03	0.2478997	1	850	tags=71%, list=50%, signal=135%
GOBP_NUCLEAR_TRANSCRIBED_MRNA_CATABOLIC_PROCESS_NONSENSE_M		74	-0.4017009	-1.3374268	0.03	0.24749912	1	817	tags=69%, list=48%, signal=127%
KEGG_ENDOCYTOSIS		29	-0.43589	-1.3373748	0.07368421	0.24715267	1	537	tags=55%, list=32%, signal=79%
GOBP_NEGATIVE_REGULATION_OF_MOLECULAR_FUNCTION		131	-0.3926781	-1.3366741	0	0.24796331	1	674	tags=54%, list=40%, signal=83%
REACTOME_NUCLEAR_ENVELOPE_NE_REASSEMBLY		15	-0.5044479	-1.3364064	0.06315789	0.24805391	1	759	tags=80%, list=45%, signal=144%
GOBP_MITOTIC_CELL_CYCLE		98	-0.3930354	-1.3358954	0.02	0.24845846	1	560	tags=47%, list=33%, signal=66%
GOBP_POSITIVE_REGULATION_OF_CELL_GROWTH		15	-0.4989116	-1.3354963	0.10869565	0.24861743	1	516	tags=60%, list=30%, signal=85%
GOBP_MUSCLE_FIBER_DEVELOPMENT		16	-0.455154	-1.3354932	0.09574468	0.24816786	1	324	tags=38%, list=19%, signal=46%
GOBP_REGULATION_OF_INNATE_IMMUNE_RESPONSE		45	-0.4133628	-1.3351138	0.08163265	0.2484301	1	618	tags=53%, list=36%, signal=82%
GOBP_REGULATION_OF_DEFENSE_RESPONSE		81	-0.3920198	-1.3339062	0.03	0.25040725	1	564	tags=49%, list=33%, signal=70%
GOBP_AXON_EXTENSION		16	-0.4921299	-1.3338399	0.07608695	0.25006068	1	399	tags=50%, list=24%, signal=65%
REACTOME_GLUCAGON_LIKE_PEPTIDE_1_GLP1_REGULATES_INSULIN_SECR		11	-0.5157948	-1.3333267	0.15476191	0.25071588	1	558	tags=55%, list=33%, signal=81%
KEGG_INSULIN_SIGNALING_PATHWAY		25	-0.4538603	-1.3331223	0.08080808	0.25055984	1	672	tags=64%, list=40%, signal=104%
GOBP_EPITHELIUM_DEVELOPMENT		106	-0.3872383	-1.3330512	0.03	0.2502839	1	710	tags=55%, list=42%, signal=88%
GOBP_INNATE_IMMUNE_RESPONSE		100	-0.3970872	-1.3327386	0.01	0.25033498	1	640	tags=53%, list=38%, signal=80%
REACTOME_CELL_JUNCTION_ORGANIZATION		13	-0.5242404	-1.3326675	0.12765957	0.25004324	1	651	tags=77%, list=38%, signal=124%
GOBP_SENSORY_ORGAN_DEVELOPMENT		25	-0.4515889	-1.332641	0.07142858	0.2496327	1	189	tags=24%, list=11%, signal=27%
GOBP_BIOLOGICAL_PROCESS_INVOLVED_IN_INTERACTION_WITH_SYMBIONT		20	-0.4539575	-1.3320726	0.15555556	0.2501802	1	138	tags=25%, list=8%, signal=27%
KEGG_RIBOSOME		71	-0.3920505	-1.3318444	0.04	0.25019628	1	858	tags=72%, list=51%, signal=139%
GOBP_TRIGLYCERIDE_METABOLIC_PROCESS		23	-0.4423393	-1.3317273	0.11827957	0.2500418	1	274	tags=30%, list=16%, signal=36%
GOBP_MEMBRANE_RAFT_ORGANIZATION		13	-0.4988756	-1.3315845	0.10227273	0.2500418	1	205	tags=31%, list=12%, signal=35%
GOBP_POST_TRANSLATIONAL_PROTEIN_MODIFICATION		75	-0.3939955	-1.3314896	0.03	0.24978743	1	828	tags=67%, list=49%, signal=125%
KEGG_VASCULAR_SMOOTH_MUSCLE_CONTRACTION		20	-0.471787	-1.3312923	0.11702128	0.2497029	1	331	tags=35%, list=20%, signal=43%
GOBP_MONOSACCHARIDE_CATABOLIC_PROCESS		16	-0.4607231	-1.3311602	0.15053764	0.24955148	1	631	tags=69%, list=37%, signal=108%
REACTOME_RECRUITMENT_OF_NUMA_TO_MITOTIC_CENTROSOMES		14	-0.5122362	-1.3309243	0.10752688	0.24948464	1	560	tags=64%, list=33%, signal=95%
GOBP_DEFENSE_RESPONSE		180	-0.373461	-1.330424	0.01	0.25014138	1	640	tags=49%, list=38%, signal=71%
GOBP_REGULATION_OF_GTPASE_ACTIVITY		33	-0.4526565	-1.3300989	0.07	0.25037652	1	607	tags=52%, list=36%, signal=79%
GOBP_POSITIVE_REGULATION_OF_INTRACELLULAR_PROTEIN_TRANSPORT		30	-0.42015	-1.3294379	0.06060606	0.2513334	1	687	tags=57%, list=41%, signal=94%
GOBP_NUCLEAR_TRANSCRIBED_MRNA_CATABOLIC_PROCESS		77	-0.3937331	-1.3294367	0.02	0.2509122	1	850	tags=70%, list=50%, signal=134%
GOBP_SKELETAL_SYSTEM_MORPHOGENESIS		10	-0.5446157	-1.3289795	0.10869565	0.25151217	1	337	tags=40%, list=20%, signal=50%
GOBP_REGULATION_OF_PEPTIDYL_SERINE_PHOSPHORYLATION		15	-0.5026703	-1.3286806	0.09782609	0.2515598	1	619	tags=73%, list=37%, signal=114%

GOBP_REGULATION_OF_RESPONSE_TO_CYTOKINE_STIMULUS		15	-0.514241	-1.3281965	0.08602151	0.25194025	1	656	tags=73%, list=39%, signal=119%
GOBP_EPITHELIAL_CELL_DEVELOPMENT		22	-0.4636552	-1.3280848	0.06315789	0.2516375	1	653	tags=59%, list=39%, signal=95%
KEGG_N_GLYCAN_BIOSYNTHESIS		10	-0.5443683	-1.3277085	0.125	0.251917	1	60	tags=20%, list=4%, signal=21%
GOBP_RESPONSE_TO_AXON_INJURY		13	-0.4822355	-1.3273693	0.13953489	0.25223005	1	301	tags=31%, list=18%, signal=37%
GOBP_POSITIVE_REGULATION_OF_DEVELOPMENTAL_PROCESS		116	-0.3828844	-1.3271515	0.03	0.25217673	1	570	tags=46%, list=34%, signal=64%
REACTOME_HOST_INTERACTIONS_OF_HIV_FACTORS		42	-0.4142795	-1.3269262	0.06060606	0.25220564	1	548	tags=50%, list=32%, signal=72%
GOBP_ADULT_BEHAVIOR		10	-0.5549666	-1.3268383	0.12903225	0.25198752	1	189	tags=40%, list=11%, signal=45%
GOBP_POSITIVE_REGULATION_OF_INTRACELLULAR_TRANSPORT		42	-0.4143348	-1.325567	0.03092784	0.2541731	1	595	tags=50%, list=35%, signal=75%
GOBP_UROGENITAL_SYSTEM_DEVELOPMENT		24	-0.4429938	-1.3253533	0.08333334	0.25411665	1	654	tags=58%, list=39%, signal=94%
GOBP_BIOLOGICAL_PROCESS_INVOLVED_IN_INTERACTION_WITH_HOST		38	-0.4264994	-1.3249354	0.09183674	0.2544053	1	619	tags=55%, list=37%, signal=85%
REACTOME_APOPTOTIC_CLEAVAGE_OF_CELLULAR_PROTEINS		10	-0.5369816	-1.3248465	0.15116279	0.254119	1	570	tags=50%, list=34%, signal=75%
GOBP_REGULATION_OF_DNA_METABOLIC_PROCESS		25	-0.4531942	-1.3247857	0.10204082	0.25376856	1	576	tags=60%, list=34%, signal=90%
GOBP_CELLULAR_RESPONSE_TO_LIPOPROTEIN_PARTICLE_STIMULUS		11	-0.5218073	-1.3240813	0.11904762	0.25475654	1	144	tags=36%, list=8%, signal=39%
GOBP_NEGATIVE_REGULATION_OF_PHOSPHORYLATION		54	-0.4205018	-1.3240007	0.05	0.25443855	1	519	tags=48%, list=31%, signal=67%
GOBP_ACTIVATION_OF_IMMUNE_RESPONSE		64	-0.4109873	-1.3233953	0.05050505	0.2550652	1	599	tags=53%, list=35%, signal=79%
REACTOME_PROGRAMMED_CELL_DEATH		55	-0.4065763	-1.3233553	0.05102041	0.2547314	1	570	tags=49%, list=34%, signal=72%
REACTOME_RRNA_PROCESSING		73	-0.3866132	-1.3231739	0.03	0.25460905	1	850	tags=70%, list=50%, signal=134%
GOBP_POSITIVE_REGULATION_OF_WOUND_HEALING		16	-0.4868179	-1.3212271	0.1	0.25774014	1	504	tags=56%, list=30%, signal=79%
REACTOME_NONSENSE_MEDIATED_DECAY_NMD		75	-0.4012456	-1.3209058	0.04	0.25785577	1	850	tags=72%, list=50%, signal=138%
GOBP_INTRACELLULAR_STEROID_HORMONE_RECEPTOR_SIGNALING_PATHW		10	-0.5303529	-1.3202214	0.16091955	0.25893888	1	447	tags=50%, list=26%, signal=68%
GOBP_NEGATIVE_REGULATION_OF_CELLULAR_COMPONENT_ORGANIZATION		96	-0.3797127	-1.3200445	0.03	0.25882697	1	414	tags=36%, list=24%, signal=46%
GOBP_CELL_MORPHOGENESIS_INVOLVED_IN_DIFFERENTIATION		92	-0.391071	-1.31821	0.04	0.26196453	1	553	tags=46%, list=33%, signal=64%
GOBP_VESICLE_MEDIATED_TRANSPORT_BETWEEN_ENDOSOMAL_COMPARTM		12	-0.5217714	-1.318143	0.11627907	0.26157525	1	537	tags=58%, list=32%, signal=85%
REACTOME_RECRUITMENT_OF_MITOTIC_CENTROSOME_PROTEINS_AND_COM		12	-0.5041744	-1.3180671	0.13253012	0.2612835	1	515	tags=58%, list=30%, signal=83%
REACTOME_SELENOAMINO_ACID_METABOLISM		76	-0.3896017	-1.3177869	0.07	0.2611693	1	850	tags=71%, list=50%, signal=136%
GOBP_REGULATION_OF_IMMUNE_SYSTEM_PROCESS		146	-0.3751194	-1.3173692	0.01	0.2615181	1	643	tags=51%, list=38%, signal=76%
GOBP_SYNCYTIUM_FORMATION		10	-0.5215673	-1.3167654	0.15116279	0.26232773	1	551	tags=50%, list=33%, signal=74%
GOBP_REGULATION_OF_IMMUNE_RESPONSE		102	-0.3849489	-1.3160348	0.01	0.26346865	1	643	tags=53%, list=38%, signal=80%
KEGG_STARCH_AND_SUCROSE_METABOLISM		10	-0.5217227	-1.3153421	0.11111111	0.26430452	1	725	tags=90%, list=43%, signal=156%
GOBP_MOVEMENT_IN_HOST_ENVIRONMENT		33	-0.4429989	-1.3152076	0.06	0.26421732	1	619	tags=55%, list=37%, signal=84%
GOBP_STRESS_FIBER_ASSEMBLY		12	-0.5251705	-1.31428	0.13580246	0.26539844	1	504	tags=50%, list=30%, signal=71%
GOBP_NEGATIVE_REGULATION_OF_PROTEIN_MODIFICATION_PROCESS		67	-0.4000917	-1.313091	0.03	0.26752383	1	687	tags=60%, list=41%, signal=96%
GOBP_RESPONSE_TO_ABIOTIC_STIMULUS		154	-0.3809063	-1.313011	0.01	0.2672739	1	374	tags=33%, list=22%, signal=39%
GOBP_REGULATION_OF_RESPONSE_TO_WOUNDING		33	-0.4330702	-1.312967	0.07070707	0.266883	1	637	tags=58%, list=38%, signal=90%
GOBP_POSITIVE_REGULATION_OF_CELL_PROJECTION_ORGANIZATION		46	-0.3936189	-1.3128309	0.13	0.26671356	1	405	tags=35%, list=24%, signal=44%
GOBP_DEFENSE_RESPONSE_TO_OTHER_ORGANISM		124	-0.386535	-1.312657	0.01	0.2665767	1	434	tags=39%, list=26%, signal=48%
GOBP_NEGATIVE_REGULATION_OF_CELLULAR_AMIDE_METABOLIC_PROCESS		21	-0.4472332	-1.3124384	0.11340206	0.26651925	1	308	tags=33%, list=18%, signal=40%
GOBP_CILIARY_BASAL_BODY_PLASMA_MEMBRANE_DOCKING		13	-0.5224684	-1.3122549	0.13333334	0.26636755	1	515	tags=62%, list=30%, signal=88%
GOBP_PHAGOCYTOSIS		51	-0.419342	-1.3119494	0.03030303	0.266467	1	391	tags=43%, list=23%, signal=54%
GOBP_VIRAL_GENE_EXPRESSION		74	-0.4034245	-1.3114777	0.07	0.26686105	1	817	tags=69%, list=48%, signal=127%
GOBP_RESPONSE_TO_HYDROGEN_PEROXIDE		26	-0.4367089	-1.3114588	0.0927835	0.2664441	1	336	tags=38%, list=20%, signal=47%
GOBP_RESPONSE_TO_GROWTH_FACTOR		61	-0.3874396	-1.3112234	0.07142858	0.26657298	1	656	tags=56%, list=39%, signal=88%
GOBP_NUCLEAR_TRANSPORT		27	-0.4477958	-1.3111681	0.08080808	0.2662513	1	536	tags=56%, list=32%, signal=80%
GOBP_DEVELOPMENTAL_CELL_GROWTH		27	-0.4200344	-1.3111302	0.13541667	0.26589984	1	406	tags=41%, list=24%, signal=53%
REACTOME_GLYCOLYSIS		15	-0.4970563	-1.31106	0.06451613	0.2656567	1	847	tags=93%, list=50%, signal=185%
GOBP_CELL_DEATH_IN_RESPONSE_TO_OXIDATIVE_STRESS		15	-0.5023077	-1.3105826	0.10526316	0.26609358	1	561	tags=67%, list=33%, signal=99%

GOBP_NUCLEOSIDE_DIPHOSPHATE_METABOLIC_PROCESS		29	-0.43975	-1.3099848	0.11702128	0.26691785	1	873	tags=83%, list=52%, signal=168%
GOBP_COTRANSLATIONAL_PROTEIN_TARGETING_TO_MEMBRANE		76	-0.392701	-1.309383	0.03	0.2677987	1	817	tags=66%, list=48%, signal=121%
GOBP_VESICLE_DOCKING		18	-0.4748274	-1.3086516	0.05376344	0.26875505	1	425	tags=39%, list=25%, signal=51%
GOBP_CELL_CELL_RECOGNITION		14	-0.5019164	-1.3083805	0.11363637	0.26877162	1	629	tags=71%, list=37%, signal=113%
GOBP_NEGATIVE_REGULATION_OF_INTRINSIC_APOPTOTIC_SIGNALING_PATH		19	-0.4794661	-1.307085	0.12222222	0.27084306	1	681	tags=68%, list=40%, signal=113%
REACTOME_RHO_GTPASE_CYCLE		77	-0.3882754	-1.3066305	0.04	0.27108625	1	504	tags=43%, list=30%, signal=58%
GOBP_REGULATION_OF_MICROTUBULE_CYTOSKELETON_ORGANIZATION		13	-0.5076853	-1.3065892	0.12765957	0.27077755	1	539	tags=62%, list=32%, signal=90%
GOBP_VASCULAR_ENDOTHELIAL_GROWTH_FACTOR_RECEPTOR_SIGNALING_		15	-0.4998468	-1.3059838	0.09574468	0.27153793	1	350	tags=53%, list=21%, signal=67%
GOBP_CORTICAL_CYTOSKELETON_ORGANIZATION		15	-0.5130334	-1.3055664	0.15053764	0.27176294	1	402	tags=47%, list=24%, signal=61%
GOBP_POSITIVE_REGULATION_OF_IMMUNE_RESPONSE		83	-0.3939554	-1.305438	0.02	0.271759	1	601	tags=52%, list=35%, signal=76%
GOBP_G_PROTEIN_COUPLED_RECEPTOR_SIGNALING_PATHWAY		51	-0.4058383	-1.3054316	0.05050505	0.27135995	1	294	tags=29%, list=17%, signal=35%
GOBP_CELL_JUNCTION_ASSEMBLY		53	-0.4116753	-1.304639	0.07070707	0.27238858	1	653	tags=53%, list=39%, signal=83%
GOBP_PROTEIN_LOCALIZATION_TO_LYSOSOME		11	-0.5136242	-1.3040957	0.16666667	0.27294528	1	353	tags=55%, list=21%, signal=68%
REACTOME_RESPONSE_OF_EIF2AK4_GCN2_TO_AMINO_ACID_DEFICIENCY		74	-0.3908497	-1.3036284	0.06	0.27331772	1	850	tags=70%, list=50%, signal=135%
GOBP_CAMERA_TYPE_EYE_DEVELOPMENT		19	-0.4723781	-1.303463	0.11578947	0.2732362	1	189	tags=26%, list=11%, signal=29%
GOBP_SIGNAL_TRANSDUCTION_BY_P53_CLASS_MEDIATOR		18	-0.4579317	-1.3023643	0.07368421	0.27489877	1	849	tags=83%, list=50%, signal=165%
REACTOME_NEUTROPHIL_DEGRANULATION		133	-0.3740204	-1.3009323	0.03	0.2772085	1	656	tags=50%, list=39%, signal=76%
GOBP_POSITIVE_REGULATION_OF_PEPTIDYL_SERINE_PHOSPHORYLATION		10	-0.5304049	-1.3001491	0.15384616	0.27839738	1	707	tags=90%, list=42%, signal=153%
GOBP_NEGATIVE_REGULATION_OF_ESTABLISHMENT_OF_PROTEIN_LOCALIZA		17	-0.4573446	-1.2994406	0.13402061	0.2794027	1	455	tags=53%, list=27%, signal=72%
GOBP_RESPONSE_TO_STEROID_HORMONE		34	-0.4289285	-1.2991867	0.12121213	0.2793415	1	852	tags=68%, list=50%, signal=133%
KEGG_PROXIMAL_TUBULE_BICARBONATE_RECLAMATION		11	-0.5085489	-1.2978609	0.11111111	0.2811956	1	45	tags=18%, list=3%, signal=19%
GOBP_REGULATION_OF_MACROAUTOPHAGY		34	-0.41385	-1.2964942	0.09183674	0.28359506	1	578	tags=50%, list=34%, signal=74%
GOBP_CELL_PROJECTION_ASSEMBLY		53	-0.3920996	-1.2963494	0.08163265	0.28345343	1	697	tags=57%, list=41%, signal=93%
GOBP_NEGATIVE_REGULATION_OF_PROTEIN_MODIFICATION_BY_SMALL_PRO		14	-0.4862545	-1.2962354	0.14893617	0.28320798	1	724	tags=79%, list=43%, signal=136%
GOBP_NEGATIVE_REGULATION_OF_CELL_DIFFERENTIATION		43	-0.396667	-1.2928803	0.11	0.28911233	1	373	tags=37%, list=22%, signal=46%
GOBP_ORGANELLE_ASSEMBLY		111	-0.3750319	-1.2928386	0.03	0.28871047	1	539	tags=44%, list=32%, signal=60%
GOBP_VESICLE_DOCKING_INVOLVED_IN_EXOCYTOSIS		13	-0.4898527	-1.2924565	0.12903225	0.28893322	1	425	tags=46%, list=25%, signal=61%
GOBP_CELLULAR_CARBOHYDRATE_CATABOLIC_PROCESS		12	-0.4976839	-1.2917919	0.15730338	0.2896709	1	446	tags=50%, list=26%, signal=67%
GOBP_POSITIVE_REGULATION_OF_DEFENSE_RESPONSE		51	-0.4220997	-1.2916725	0.07142858	0.28963965	1	564	tags=53%, list=33%, signal=77%
GOBP_LEUKOCYTE_CELL_CELL_ADHESION		42	-0.3943524	-1.2910261	0.1	0.29055163	1	658	tags=57%, list=39%, signal=91%
REACTOME_ABC_TRANSPORTER_DISORDERS		31	-0.4232388	-1.2903476	0.04081633	0.2915337	1	192	tags=29%, list=11%, signal=32%
GOBP_AMEBOIDAL_TYPE_CELL_MIGRATION		53	-0.3936192	-1.2889848	0.14285715	0.29411733	1	337	tags=32%, list=20%, signal=39%
GOBP_REGULATION_OF_NEURON_PROJECTION_DEVELOPMENT		47	-0.4099614	-1.2881372	0.07142858	0.29544413	1	558	tags=43%, list=33%, signal=62%
GOBP_REGULATION_OF_TRANSLATIONAL_INITIATION		11	-0.5106666	-1.287622	0.13953489	0.29616514	1	605	tags=55%, list=36%, signal=84%
GOBP_REGULATION_OF_PHAGOCYTOSIS		19	-0.4683325	-1.2867068	0.11827957	0.29757193	1	467	tags=47%, list=28%, signal=65%
GOBP_RESPONSE_TO_BIOTIC_STIMULUS		166	-0.368827	-1.2866585	0.02	0.2973094	1	640	tags=50%, list=38%, signal=72%
REACTOME_RHOD_GTPASE_CYCLE		17	-0.4736451	-1.2862495	0.17204301	0.29751465	1	619	tags=65%, list=37%, signal=101%
GOBP_CARDIAC_CELL_DEVELOPMENT		11	-0.5234751	-1.2856979	0.0989011	0.29823032	1	451	tags=45%, list=27%, signal=62%
REACTOME_BINDING_AND_UPTAKE_OF_LIGANDS_BY_SCAVENGER_RECEPTO		17	-0.4719454	-1.2854828	0.14285715	0.29802668	1	306	tags=41%, list=18%, signal=50%
REACTOME_AURKA_ACTIVATION_BY_TPX2		12	-0.5041744	-1.2853068	0.1764706	0.29779434	1	515	tags=58%, list=30%, signal=83%
GOBP_NUCLEOSIDE_PHOSPHATE_CATABOLIC_PROCESS		13	-0.5308056	-1.2851064	0.18888889	0.29769278	1	699	tags=69%, list=41%, signal=117%
REACTOME_RHO_GTPASES_ACTIVATE_PKNS		16	-0.4776379	-1.2843341	0.18085106	0.29901087	1	704	tags=63%, list=42%, signal=106%
GOBP_SYNAPTIC_VESICLE_RECYCLING		12	-0.5083422	-1.2842506	0.14814815	0.29877803	1	748	tags=75%, list=44%, signal=133%
GOBP_RESPONSE_TO_INTERLEUKIN_12		20	-0.4372526	-1.2842304	0.08247422	0.29837254	1	567	tags=55%, list=33%, signal=82%
GOBP_DEVELOPMENTAL_GROWTH		58	-0.3973276	-1.2834423	0.07	0.29962805	1	574	tags=45%, list=34%, signal=65%
GOBP_SPERM_EGG_RECOGNITION		10	-0.5251138	-1.2830013	0.1882353	0.30012888	1	376	tags=50%, list=22%, signal=64%

GOBP_RENAL_SYSTEM_PROCESS		14	-0.4974017	-1.2829084	0.08695652	0.29983848	1	602	tags=64%, list=36%, signal=99%
GOBP_SENSORY_PERCEPTION_OF_LIGHT_STIMULUS		10	-0.5457916	-1.2823688	0.12345679	0.3005822	1	663	tags=70%, list=39%, signal=114%
GOBP_CELLULAR_RESPONSE_TO_CALCIUM_ION		12	-0.5049921	-1.2820674	0.125	0.30084944	1	570	tags=67%, list=34%, signal=100%
GOBP_GLYCOPROTEIN_METABOLIC_PROCESS		36	-0.3932537	-1.2816437	0.1010101	0.30123118	1	262	tags=22%, list=15%, signal=26%
GOBP_CELLULAR_RESPONSE_TO_ORGANIC_CYCLIC_COMPOUND		64	-0.3863685	-1.2808049	0.08	0.30265412	1	447	tags=39%, list=26%, signal=51%
REACTOME_VESICLE_MEDIATED_TRANSPORT		141	-0.3610445	-1.2807534	0.04	0.30234808	1	828	tags=65%, list=49%, signal=116%
GOBP_REGULATION_OF_PROTEIN_LOCALIZATION		144	-0.3634108	-1.2792984	0.05	0.30520332	1	659	tags=51%, list=39%, signal=77%
GOBP_REGULATION_OF_ADAPTIVE_IMMUNE_RESPONSE		15	-0.4616188	-1.2792805	0.14130434	0.3047804	1	323	tags=33%, list=19%, signal=41%
GOBP_ENDOTHELIAL_CELL_DEVELOPMENT		14	-0.4511309	-1.2786251	0.14444445	0.30597913	1	653	tags=57%, list=39%, signal=92%
GOBP_SECRETION		264	-0.3521176	-1.2785484	0	0.30564126	1	658	tags=48%, list=39%, signal=66%
GOBP_POSITIVE_REGULATION_OF_PROTEIN_BINDING		19	-0.4541667	-1.2785013	0.16494845	0.30536112	1	619	tags=63%, list=37%, signal=98%
GOBP_NEGATIVE_REGULATION_OF_PHOSPHORUS_METABOLIC_PROCESS		65	-0.3864709	-1.2782912	0.11	0.30530804	1	519	tags=45%, list=31%, signal=62%
REACTOME_IRE1ALPHA_ACTIVATES_CHAPERONES		18	-0.4680807	-1.2778984	0.13402061	0.30577815	1	636	tags=56%, list=38%, signal=88%
GOBP_I_KAPPAB_KINASE_NF_KAPPAB_SIGNALING		26	-0.4232107	-1.277725	0.13265306	0.30558363	1	406	tags=42%, list=24%, signal=55%
REACTOME_SIGNALING_BY_NOTCH		35	-0.4216491	-1.2773066	0.09375	0.30593944	1	548	tags=46%, list=32%, signal=66%
GOBP_TRANSFERRIN_TRANSPORT		12	-0.4783401	-1.2767656	0.10869565	0.30679867	1	875	tags=83%, list=52%, signal=171%
GOBP_RESPONSE_TO_MECHANICAL_STIMULUS		16	-0.4455961	-1.2767648	0.10869565	0.30635077	1	217	tags=25%, list=13%, signal=28%
REACTOME_ER_TO_GOLGI_ANTEROGRADE_TRANSPORT		44	-0.3879667	-1.2740158	0.11	0.3116339	1	892	tags=77%, list=53%, signal=159%
GOBP_PROTEIN_DNA_COMPLEX_SUBUNIT_ORGANIZATION		14	-0.4724487	-1.2736144	0.16666667	0.31183785	1	267	tags=36%, list=16%, signal=42%
GOBP_REGULATION_OF_INTRACELLULAR_PROTEIN_TRANSPORT		42	-0.4169118	-1.2734535	0.13402061	0.31165048	1	595	tags=52%, list=35%, signal=79%
REACTOME_NON_INTEGRIN_MEMBRANE_ECM_INTERACTIONS		13	-0.4930626	-1.2728173	0.15053764	0.3127453	1	325	tags=46%, list=19%, signal=57%
KEGG_PRION_DISEASES		10	-0.5359486	-1.2727165	0.14285715	0.31250048	1	434	tags=50%, list=26%, signal=67%
GOBP_RESPONSE_TO_CALCIUM_ION		31	-0.4248159	-1.2724085	0.11458334	0.31257766	1	637	tags=58%, list=38%, signal=91%
GOBP_CELLULAR_RESPONSE_TO_HYDROGEN_PEROXIDE		12	-0.5121539	-1.2719967	0.1764706	0.31301486	1	557	tags=67%, list=33%, signal=99%
REACTOME_EPH_EPHRIN_SIGNALING		23	-0.4371474	-1.2717003	0.17204301	0.3133123	1	691	tags=65%, list=41%, signal=109%
GOBP_REGULATION_OF_AUTOPHAGY		51	-0.3837949	-1.2713526	0.10204082	0.31367788	1	578	tags=45%, list=34%, signal=66%
GOBP_CELL_JUNCTION_ORGANIZATION		87	-0.369814	-1.2710049	0.03030303	0.31382257	1	659	tags=51%, list=39%, signal=78%
GOBP_MYELOID_LEUKOCYTE_MEDIATED_IMMUNITY		142	-0.3629943	-1.2702396	0.03	0.3150998	1	656	tags=50%, list=39%, signal=75%
GOBP_PROTEIN_LOCALIZATION_TO_ENDOPLASMIC_RETICULUM		86	-0.3697423	-1.2699984	0.05	0.31514382	1	817	tags=63%, list=48%, signal=115%
GOBP_CELLULAR_RESPONSE_TO_BIOTIC_STIMULUS		21	-0.4379613	-1.2695538	0.19791667	0.3155058	1	698	tags=57%, list=41%, signal=96%
GOBP_ADENYLATE_CYCLASE_MODULATING_G_PROTEIN_COUPLED_RECEPT		17	-0.4436375	-1.269337	0.19565217	0.31560433	1	406	tags=41%, list=24%, signal=54%
GOBP_PROTEIN_LOCALIZATION_TO_VACUOLE		15	-0.4649126	-1.2684275	0.14583333	0.31722617	1	551	tags=60%, list=33%, signal=88%
GOBP_ESTABLISHMENT_OF_RNA_LOCALIZATION		14	-0.489249	-1.2683823	0.17391305	0.3168975	1	536	tags=64%, list=32%, signal=93%
GOBP_PROTEIN_CATABOLIC_PROCESS		139	-0.3617393	-1.268257	0.05	0.3166933	1	828	tags=64%, list=49%, signal=115%
REACTOME_ANTI_INFLAMMATORY_RESPONSE_FAVOURING_LEISHMANIA_PAR		15	-0.4613586	-1.2680898	0.12371134	0.3166664	1	578	tags=53%, list=34%, signal=80%
REACTOME_UNFOLDED_PROTEIN_RESPONSE_UPR		23	-0.4311141	-1.2680389	0.19354838	0.3162708	1	636	tags=52%, list=38%, signal=82%
KEGG_LONG_TERM_DEPRESSION		12	-0.5005829	-1.2670984	0.17777778	0.31810063	1	199	tags=33%, list=12%, signal=38%
GOBP_NEURON_DEATH		42	-0.3942068	-1.2668024	0.13131313	0.31818083	1	561	tags=45%, list=33%, signal=66%
GOBP_REGULATION_OF_CELL_MATRIX_ADHESION		19	-0.4489847	-1.266307	0.15789473	0.31883177	1	294	tags=37%, list=17%, signal=44%
GOBP_CARBOHYDRATE_CATABOLIC_PROCESS		35	-0.3901013	-1.2662711	0.14583333	0.3185438	1	552	tags=49%, list=33%, signal=71%
REACTOME_GLUCAGON_SIGNALING_IN_METABOLIC_REGULATION		10	-0.5327879	-1.2660234	0.18181819	0.31863642	1	519	tags=50%, list=31%, signal=72%
GOBP_REGULATION_OF_BIOLOGICAL_PROCESS_INVOLVED_IN_SYMBIOTIC_I		16	-0.4684048	-1.2659981	0.18947369	0.31825528	1	619	tags=56%, list=37%, signal=88%
GOBP_DNA_CONFORMATION_CHANGE		18	-0.4552908	-1.2657157	0.12765957	0.3184836	1	805	tags=72%, list=47%, signal=136%
GOBP_DEVELOPMENTAL_PROCESS_INVOLVED_IN_REPRODUCTION		46	-0.3929058	-1.265575	0.14141414	0.31827903	1	651	tags=50%, list=38%, signal=79%
GOBP_T_CELL_ACTIVATION		47	-0.4103732	-1.2650453	0.11111111	0.31913972	1	629	tags=51%, list=37%, signal=79%
GOBP_MICROTUBULE_CYTOSKELETON_ORGANIZATION		52	-0.3942547	-1.2648005	0.07	0.319406	1	560	tags=48%, list=33%, signal=70%

GOBP_POSITIVE_REGULATION_OF_IMMUNE_SYSTEM_PROCESS		110	-0.3687981	-1.2643619	0.05	0.31990063	1	668	tags=54%, list=39%, signal=83%
GOBP_MYELOID_LEUKOCYTE_ACTIVATION		146	-0.3666455	-1.2642022	0.06	0.31976193	1	656	tags=50%, list=39%, signal=75%
GOBP_RECEPTOR_MEDIATED_ENDOCYTOSIS		62	-0.3817253	-1.2638097	0.1010101	0.32006702	1	820	tags=66%, list=48%, signal=123%
GOBP_CELLULAR_RESPONSE_TO_HORMONE_STIMULUS		73	-0.3862448	-1.2626665	0.06060606	0.32219067	1	675	tags=51%, list=40%, signal=81%
REACTOME_HCMV_EARLY_EVENTS		10	-0.5273615	-1.2621166	0.15053764	0.3228922	1	759	tags=80%, list=45%, signal=144%
GOBP_INTRACELLULAR_LIPID_TRANSPORT		12	-0.4975495	-1.2620627	0.17582418	0.32252392	1	768	tags=75%, list=45%, signal=136%
REACTOME_GPCR_LIGAND_BINDING		15	-0.4778799	-1.2611722	0.14285715	0.3239575	1	367	tags=40%, list=22%, signal=51%
KEGG_TIGHT_JUNCTION		33	-0.4046989	-1.260932	0.15	0.3239219	1	402	tags=33%, list=24%, signal=43%
GOBP_ENDOTHELIAL_CELL_MIGRATION		33	-0.4144913	-1.2604401	0.1632653	0.32457802	1	294	tags=30%, list=17%, signal=36%
GOBP_CELLULAR_RESPONSE_TO_HEAT		18	-0.4552816	-1.2603389	0.15730338	0.32436872	1	107	tags=28%, list=6%, signal=29%
REACTOME_ANTIGEN_PRESENTATION_FOLDING_ASSEMBLY_AND_PEPTIDE_L		12	-0.4789114	-1.2601156	0.17045455	0.3245185	1	618	tags=58%, list=36%, signal=91%
KEGG_COMPLEMENT_AND_COAGULATION_CASCADES		14	-0.4732728	-1.2599366	0.16091955	0.32437655	1	637	tags=71%, list=38%, signal=113%
GOBP_CELL_RECOGNITION		24	-0.4230108	-1.2589118	0.15625	0.32612506	1	629	tags=58%, list=37%, signal=91%
GOBP_SENSORY_SYSTEM_DEVELOPMENT		23	-0.4554524	-1.2574432	0.1368421	0.3292948	1	189	tags=26%, list=11%, signal=29%
GOBP_VIRAL_GENOME_REPLICATION		20	-0.4371647	-1.2573787	0.16666667	0.32898858	1	734	tags=60%, list=43%, signal=105%
GOBP_EXTRACELLULAR_MATRIX_DISASSEMBLY		13	-0.4852459	-1.257287	0.1954023	0.32870916	1	341	tags=38%, list=20%, signal=48%
GOBP_POSITIVE_REGULATION_OF_GROWTH		21	-0.4388154	-1.2571284	0.15151516	0.32848302	1	516	tags=52%, list=30%, signal=74%
GOBP_ENDOCYTOSIS		105	-0.3645763	-1.2563145	0.11	0.3297411	1	820	tags=62%, list=48%, signal=112%
GOBP_ACTIN_FILAMENT_BASED_MOVEMENT		50	-0.3985036	-1.2560122	0.07070707	0.329895	1	366	tags=30%, list=22%, signal=37%
GOBP_NEGATIVE_REGULATION_OF_EPITHELIAL_CELL_MIGRATION		10	-0.5289558	-1.2560095	0.16091955	0.32944554	1	227	tags=30%, list=13%, signal=34%
GOBP_MAINTENANCE_OF_PROTEIN_LOCATION		16	-0.464413	-1.2556708	0.17525773	0.32985964	1	687	tags=75%, list=41%, signal=125%
GOBP_POSITIVE_REGULATION_OF_PEPTIDYL_TYROSINE_PHOSPHORYLATIOI		15	-0.4607351	-1.2554773	0.14893617	0.3298812	1	341	tags=47%, list=20%, signal=58%
GOBP_ADAPTIVE_IMMUNE_RESPONSE		30	-0.4015147	-1.2536868	0.14285715	0.3328899	1	637	tags=50%, list=38%, signal=79%
GOBP_CELLULAR_RESPONSE_TO_DNA_DAMAGE_STIMULUS		41	-0.3908331	-1.2525369	0.15	0.33501753	1	853	tags=76%, list=50%, signal=149%
KEGG_GLYCOLYSIS_GLUCONEOGENESIS		24	-0.4103133	-1.2523395	0.20618556	0.3348898	1	893	tags=83%, list=53%, signal=174%
GOBP_MYOFIBRIL_ASSEMBLY		20	-0.4305121	-1.25199	0.15789473	0.33530736	1	406	tags=35%, list=24%, signal=45%
GOBP_REGULATION_OF_PHOSPHOPROTEIN_PHOSPHATASE_ACTIVITY		11	-0.4850534	-1.2518378	0.18604651	0.33507514	1	294	tags=36%, list=17%, signal=44%
GOBP_RECEPTOR_INTERNALIZATION		22	-0.4377856	-1.2514335	0.17391305	0.33555618	1	748	tags=73%, list=44%, signal=128%
GOBP_HEART_DEVELOPMENT		57	-0.3860726	-1.2510773	0.12	0.33594564	1	452	tags=37%, list=27%, signal=49%
GOBP_PEPTIDE_BIOSYNTHETIC_PROCESS		216	-0.3507873	-1.2510328	0.01	0.3356494	1	862	tags=63%, list=51%, signal=111%
GOBP_LEUKOCYTE_DIFFERENTIATION		36	-0.4115163	-1.2505366	0.14141414	0.33623102	1	460	tags=42%, list=27%, signal=56%
GOBP_ZYMOGEN_ACTIVATION		11	-0.4968633	-1.2500426	0.20224719	0.33700582	1	637	tags=64%, list=38%, signal=101%
GOBP_REGULATION_OF_VASOCONSTRICTION		10	-0.5023747	-1.2493132	0.15555556	0.3385119	1	655	tags=80%, list=39%, signal=130%
KEGG_VASOPRESSIN_REGULATED_WATER_REABSORPTION		15	-0.4540848	-1.2488967	0.17204301	0.3389704	1	690	tags=60%, list=41%, signal=100%
REACTOME_TRANSPORT_TO_THE_GOLGI_AND_SUBSEQUENT_MODIFICATION		45	-0.3925841	-1.2485532	0.1010101	0.3394037	1	892	tags=78%, list=53%, signal=160%
REACTOME_CELLULAR_RESPONSE_TO_HEAT_STRESS		20	-0.4649537	-1.2476989	0.15957446	0.34064385	1	719	tags=75%, list=42%, signal=129%
GOBP_RESPONSE_TO_CAMP		13	-0.4872217	-1.2474288	0.17021276	0.34076598	1	225	tags=38%, list=13%, signal=44%
GOBP_REGULATION_OF_INTRACELLULAR_SIGNAL_TRANSDUCTION		165	-0.3516042	-1.2470497	0.05	0.3410796	1	681	tags=51%, list=40%, signal=77%
GOBP_POSITIVE_REGULATION_OF_LEUKOCYTE_MEDIATED_IMMUNITY		14	-0.4682988	-1.2465119	0.17977528	0.34190336	1	384	tags=43%, list=23%, signal=55%
GOBP_RESPONSE_TO_ENDOPLASMIC_RETICULUM_STRESS		69	-0.3668243	-1.2457418	0.08	0.34315798	1	636	tags=48%, list=38%, signal=73%
GOBP_PEPTIDYL_PROLINE_MODIFICATION		11	-0.5077131	-1.2456068	0.20224719	0.34293288	1	472	tags=55%, list=28%, signal=75%
GOBP_HORMONE_MEDIATED_SIGNALING_PATHWAY		13	-0.4840216	-1.2454435	0.18478261	0.34288633	1	491	tags=46%, list=29%, signal=64%
GOBP_CELL_CELL_ADHESION_VIA_PLASMA_MEMBRANE_ADHESION_MOLECU		13	-0.4950134	-1.2454002	0.17777778	0.34261113	1	607	tags=54%, list=36%, signal=83%
GOBP_TRNA_METABOLIC_PROCESS		25	-0.4209511	-1.2452224	0.14141414	0.3427041	1	590	tags=52%, list=35%, signal=79%
GOBP_NEGATIVE_REGULATION_OF_CELL_POPULATION_PROLIFERATION		45	-0.3985765	-1.2435697	0.14583333	0.34576008	1	202	tags=27%, list=12%, signal=29%
GOBP_LEUKOCYTE_MEDIATED_IMMUNITY		155	-0.3543621	-1.2434837	0.05	0.34544426	1	656	tags=50%, list=39%, signal=74%

GOBP_REGULATION_OF_EXTENT_OF_CELL_GROWTH		14	-0.4790339	-1.2434471	0.20879121	0.3450916	1	499	tags=57%, list=29%, signal=80%
GOBP_MULTI_ORGANISM_PROCESS		67	-0.3862901	-1.2431233	0.07	0.34528145	1	640	tags=48%, list=38%, signal=74%
REACTOME_HEMOSTASIS		128	-0.3611806	-1.2424728	0.06	0.34651875	1	655	tags=49%, list=39%, signal=74%
GOBP_PHOSPHATIDYLGLYCEROL_METABOLIC_PROCESS		10	-0.5111697	-1.2422702	0.20454545	0.34642962	1	210	tags=20%, list=12%, signal=23%
GOBP_INFLAMMATORY_RESPONSE		73	-0.3679598	-1.2421116	0.09090909	0.3463168	1	673	tags=53%, list=40%, signal=85%
GOBP_CELLULAR_CARBOHYDRATE_METABOLIC_PROCESS		35	-0.4057494	-1.2414078	0.2	0.34755924	1	446	tags=43%, list=26%, signal=57%
GOBP_PLACENTA_DEVELOPMENT		10	-0.5033681	-1.2412127	0.17582418	0.34764546	1	475	tags=50%, list=28%, signal=69%
GOBP_INSULIN_RECEPTOR_SIGNALING_PATHWAY		18	-0.441346	-1.2410508	0.20652173	0.34746876	1	497	tags=44%, list=29%, signal=62%
GOBP_NADH_METABOLIC_PROCESS		21	-0.4300452	-1.240421	0.15463917	0.3484916	1	860	tags=81%, list=51%, signal=162%
GOBP_CELL_PART_MORPHOGENESIS		84	-0.3658173	-1.2391404	0.1	0.35102195	1	558	tags=44%, list=33%, signal=62%
GOBP_CARDIAC_MUSCLE_CELL_DIFFERENTIATION		12	-0.4757176	-1.2389276	0.2	0.35099044	1	451	tags=42%, list=27%, signal=56%
GOBP_PROTEIN_LOCALIZATION_TO_CHROMOSOME		13	-0.4687665	-1.2389246	0.1978022	0.3505483	1	133	tags=31%, list=8%, signal=33%
GOBP_POSITIVE_REGULATION_OF_ADAPTIVE_IMMUNE_RESPONSE		10	-0.5208744	-1.2389185	0.22222222	0.3500948	1	323	tags=40%, list=19%, signal=49%
GOBP_ANATOMICAL_STRUCTURE_HOMEOSTASIS		45	-0.4020945	-1.2384859	0.15	0.35053623	1	728	tags=64%, list=43%, signal=110%
GOBP_REGULATION_OF_NIK_NF_KAPPAB_SIGNALING		13	-0.4553087	-1.2382741	0.21839081	0.35060462	1	347	tags=31%, list=20%, signal=38%
GOBP_ANTIGEN_PROCESSING_AND_PRESENTATION_OF_PEPTIDE_OR_POLY		26	-0.4116823	-1.2376897	0.18181819	0.3514779	1	800	tags=69%, list=47%, signal=129%
GOBP_APOPTOTIC_CELL_CLEARANCE		12	-0.5084374	-1.2375627	0.21978022	0.3513593	1	368	tags=50%, list=22%, signal=63%
BIOCARTA_MCALPAIN_PATHWAY		10	-0.5078695	-1.2366195	0.22222222	0.3532056	1	221	tags=40%, list=13%, signal=46%
GOBP_PROTEIN_PHOSPHORYLATION		148	-0.3528421	-1.2360526	0.08	0.3538996	1	564	tags=42%, list=33%, signal=57%
GOBP_RESPONSE_TO_KETONE		20	-0.4371571	-1.2355744	0.16483517	0.35441884	1	367	tags=40%, list=22%, signal=50%
GOBP_REGULATION_OF_CELLULAR_KETONE_METABOLIC_PROCESS		45	-0.389904	-1.235252	0.17171717	0.35465539	1	192	tags=24%, list=11%, signal=27%
GOBP_REGULATION_OF_PROTEIN_PHOSPHORYLATION		118	-0.3581049	-1.2351005	0.04	0.3545342	1	576	tags=43%, list=34%, signal=61%
GOBP_LYMPHOCYTE_ACTIVATION		57	-0.3894641	-1.2343887	0.12121213	0.35573727	1	629	tags=51%, list=37%, signal=78%
GOBP_POSITIVE_REGULATION_OF_CELL_POPULATION_PROLIFERATION		68	-0.3633263	-1.2326761	0.09	0.35908332	1	332	tags=32%, list=20%, signal=39%
GOBP_GROWTH		84	-0.3647839	-1.2311033	0.09	0.36212844	1	574	tags=42%, list=34%, signal=60%
GOBP_MAPK_CASCADE		104	-0.3604163	-1.230163	0.05	0.36364752	1	869	tags=68%, list=51%, signal=131%
GOBP_REGULATION_OF_PROTEIN_TARGETING_TO_MEMBRANE		10	-0.5004175	-1.2299985	0.20987654	0.3636259	1	122	tags=30%, list=7%, signal=32%
GOBP_HUMORAL_IMMUNE_RESPONSE_MEDIATED_BY_CIRCULATING_IMMUNO		10	-0.5208185	-1.229581	0.21348314	0.36430088	1	573	tags=80%, list=34%, signal=120%
GOBP_PROTEIN_LOCALIZATION_TO_CELL_SURFACE		13	-0.4700162	-1.2292525	0.23333333	0.36452276	1	495	tags=46%, list=29%, signal=65%
REACTOME_OPIOID_SIGNALLING		19	-0.4379865	-1.2286847	0.22916667	0.36538845	1	367	tags=37%, list=22%, signal=46%
REACTOME_FCGAMMA_RECEPTOR_FCGR_DEPENDENT_PHAGOCYTOSIS		13	-0.4564556	-1.2276907	0.17977528	0.36713928	1	668	tags=69%, list=39%, signal=113%
GOBP_REGULATION_OF_TRANSFERASE_ACTIVITY		101	-0.3545373	-1.2274486	0.05	0.3671982	1	853	tags=63%, list=50%, signal=120%
GOBP_INTRACELLULAR_RECEPTOR_SIGNALING_PATHWAY		19	-0.4337736	-1.2271707	0.1827957	0.36740223	1	576	tags=58%, list=34%, signal=87%
GOBP_MAINTENANCE_OF_PROTEIN_LOCATION_IN_CELL		12	-0.4816482	-1.2270658	0.20731707	0.36709687	1	659	tags=75%, list=39%, signal=122%
GOBP_BONE_DEVELOPMENT		20	-0.4428449	-1.2259384	0.16494845	0.36891964	1	459	tags=45%, list=27%, signal=61%
GOBP_REGULATION_OF_COAGULATION		20	-0.4382141	-1.2256556	0.19587629	0.3692046	1	683	tags=60%, list=40%, signal=99%
GOBP_RETROGRADE_TRANSPORT_ENDOSOME_TO_GOLGI		12	-0.5101761	-1.2251704	0.24175824	0.36974257	1	206	tags=33%, list=12%, signal=38%
GOBP_CARDIAC_MUSCLE_TISSUE_DEVELOPMENT		24	-0.41562	-1.2247639	0.21649484	0.37027952	1	171	tags=21%, list=10%, signal=23%
REACTOME_MAP2K_AND_MAPK_ACTIVATION		17	-0.4524499	-1.224194	0.20212767	0.3712836	1	199	tags=35%, list=12%, signal=40%
GOBP_NUCLEUS_ORGANIZATION		14	-0.4523301	-1.2236906	0.17894737	0.3719736	1	275	tags=36%, list=16%, signal=42%
KEGG_ECM_RECEPTOR_INTERACTION		19	-0.4486716	-1.2236477	0.21052632	0.37161738	1	456	tags=53%, list=27%, signal=71%
KEGG_PURINE_METABOLISM		19	-0.4278284	-1.2234446	0.19791667	0.37156078	1	620	tags=58%, list=37%, signal=90%
REACTOME_ABC_FAMILY_PROTEINS_MEDIATED_TRANSPORT		43	-0.4000007	-1.2231075	0.15463917	0.3719238	1	192	tags=26%, list=11%, signal=28%
GOBP_CELLULAR_COMPONENT_MORPHOGENESIS		107	-0.3522218	-1.2223697	0.11	0.37327933	1	558	tags=41%, list=33%, signal=57%
GOBP_NEUTRAL_LIPID_METABOLIC_PROCESS		25	-0.4075975	-1.2216761	0.16666667	0.3745109	1	274	tags=28%, list=16%, signal=33%
REACTOME_RHO_GTPASES_ACTIVATE_FORMINS		17	-0.4502305	-1.221623	0.20879121	0.37416533	1	759	tags=71%, list=45%, signal=127%

KEGG_ADHERENS_JUNCTION		14	-0.4749271	-1.2211664	0.17582418	0.37469044	1	653	tags=57%, list=39%, signal=92%
GOBP_PEPTIDYL_THREONINE_MODIFICATION		17	-0.4117707	-1.2205074	0.1875	0.37584415	1	564	tags=47%, list=33%, signal=70%
GOBP_CELLULAR_RESPONSE_TO_CARBOHYDRATE_STIMULUS		19	-0.4290092	-1.2202148	0.20618556	0.37604508	1	557	tags=53%, list=33%, signal=78%
REACTOME_MEMBRANE_TRAFFICKING		124	-0.3526093	-1.2198174	0.06	0.3764119	1	828	tags=64%, list=49%, signal=115%
REACTOME_SIGNALING_BY_MODERATE_KINASE_ACTIVITY_BRAF_MUTANTS		21	-0.4082769	-1.2192711	0.21052632	0.3773829	1	199	tags=29%, list=12%, signal=32%
GOBP_REGULATION_OF_CELL_SUBSTRATE_ADHESION		39	-0.3954006	-1.2188363	0.12244898	0.3777941	1	504	tags=46%, list=30%, signal=64%
KEGG_WNT_SIGNALING_PATHWAY		18	-0.4234943	-1.2186501	0.17525773	0.3777196	1	420	tags=33%, list=25%, signal=44%
GOBP_NEGATIVE_REGULATION_OF_RESPONSE_TO_STIMULUS		173	-0.3414868	-1.2183763	0.03	0.37807143	1	687	tags=51%, list=41%, signal=77%
GOBP_REGULATION_OF_CELL_PROJECTION_ORGANIZATION		75	-0.3618572	-1.2178763	0.11111111	0.37894282	1	558	tags=41%, list=33%, signal=59%
GOBP_REGULATION_OF_GENE_SILENCING		12	-0.5056211	-1.2176511	0.20454545	0.37887928	1	704	tags=75%, list=42%, signal=127%
GOBP_CLATHRIN_DEPENDENT_ENDOCYTOSIS		11	-0.4878783	-1.2176142	0.21686748	0.378486	1	800	tags=91%, list=47%, signal=171%
REACTOME_SRP_DEPENDENT_COTRANSLATIONAL_PROTEIN_TARGETING_TO		82	-0.3622719	-1.217259	0.12	0.37883496	1	850	tags=65%, list=50%, signal=123%
REACTOME_HSF1_DEPENDENT_TRANSACTIVATION		11	-0.4680367	-1.2171997	0.14942528	0.37847808	1	502	tags=55%, list=30%, signal=77%
GOBP_MITOTIC_CYTOKINESIS		10	-0.473284	-1.2161518	0.22727273	0.3804212	1	388	tags=40%, list=23%, signal=52%
GOBP_PURINE_CONTAINING_COMPOUND_CATABOLIC_PROCESS		10	-0.4968362	-1.21606	0.2159091	0.3801802	1	620	tags=50%, list=37%, signal=78%
GOBP_CELLULAR_KETONE_METABOLIC_PROCESS		68	-0.3670707	-1.215766	0.11	0.3804186	1	796	tags=56%, list=47%, signal=101%
GOBP_POSITIVE_REGULATION_OF_CATABOLIC_PROCESS		69	-0.3755159	-1.2157184	0.07	0.38007343	1	699	tags=57%, list=41%, signal=92%
GOBP_COVALENT_CHROMATIN_MODIFICATION		14	-0.4697004	-1.215519	0.20212767	0.38007903	1	576	tags=64%, list=34%, signal=97%
GOBP_REGULATION_OF_WOUND_HEALING		29	-0.4026872	-1.2151438	0.23958333	0.380318	1	683	tags=59%, list=40%, signal=97%
REACTOME_METABOLISM_OF_STEROIDS		14	-0.4502203	-1.2150867	0.2	0.3800324	1	854	tags=86%, list=50%, signal=171%
REACTOME_SIGNALING_BY_RECEPTOR_TYROSINE_KINASES		59	-0.3702743	-1.2142911	0.08	0.38159603	1	879	tags=71%, list=52%, signal=143%
GOBP_REGULATION_OF_NITRIC_OXIDE_SYNTHASE_ACTIVITY		10	-0.4916883	-1.2136343	0.14606741	0.38251603	1	857	tags=90%, list=51%, signal=181%
KEGG_T_CELL_RECEPTOR_SIGNALING_PATHWAY		10	-0.5076618	-1.2127094	0.12790698	0.38422415	1	309	tags=40%, list=18%, signal=49%
GOBP_CYTOSOLIC_TRANSPORT		28	-0.4049659	-1.2113534	0.21276596	0.3870856	1	537	tags=46%, list=32%, signal=67%
GOBP_RESPONSE_TO_INSULIN		40	-0.3835293	-1.2108933	0.16161616	0.387649	1	904	tags=68%, list=53%, signal=141%
GOBP_REGULATION_OF_NERVOUS_SYSTEM_DEVELOPMENT		33	-0.4043278	-1.2091103	0.20618556	0.39144662	1	547	tags=45%, list=32%, signal=66%
GOBP_NUCLEIC_ACID_PHOSPHODIESTER_BOND_HYDROLYSIS		16	-0.4317139	-1.2090707	0.17894737	0.39101142	1	814	tags=63%, list=48%, signal=119%
BIOCARTA_NFAT_PATHWAY		10	-0.4880523	-1.2089493	0.22105263	0.390877	1	519	tags=40%, list=31%, signal=57%
GOBP_PROTEOLYSIS		212	-0.3392154	-1.208822	0.05	0.39068496	1	829	tags=58%, list=49%, signal=99%
GOBP_VASOCONSTRICTION		10	-0.5023747	-1.2082764	0.21839081	0.39156324	1	655	tags=80%, list=39%, signal=130%
GOBP_PEPTIDE_METABOLIC_PROCESS		252	-0.3359955	-1.2080767	0.03	0.39157826	1	862	tags=60%, list=51%, signal=104%
GOBP_POSITIVE_REGULATION_OF_CELL_ADHESION		61	-0.3634291	-1.2076274	0.14	0.392086	1	393	tags=34%, list=23%, signal=43%
GOBP_TRANSMEMBRANE_RECEPTOR_PROTEIN_SERINE_THREONINE_KINASE		24	-0.4007159	-1.2058624	0.1734694	0.3963497	1	259	tags=29%, list=15%, signal=34%
GOBP_RESPONSE_TO_INTERFERON_GAMMA		26	-0.407833	-1.2056986	0.19587629	0.39618748	1	386	tags=35%, list=23%, signal=44%
GOBP_NUCLEOBASE_CONTAINING_SMALL_MOLECULE_BIOSYNTHETIC_PROC		16	-0.4545693	-1.2042006	0.20879121	0.3991006	1	844	tags=75%, list=50%, signal=148%
GOBP_REGULATION_OF_CELL_MORPHOGENESIS_INVOLVED_IN_DIFFERENTIA		23	-0.3986152	-1.2041848	0.22340426	0.3986723	1	504	tags=48%, list=30%, signal=67%
GOBP_CARBOHYDRATE_METABOLIC_PROCESS		87	-0.3561834	-1.2041012	0.1	0.3983705	1	575	tags=45%, list=34%, signal=64%
REACTOME_CYTOPROTECTION_BY_HMOX1		46	-0.3789874	-1.2034174	0.21	0.3995499	1	192	tags=24%, list=11%, signal=26%
GOBP_CHROMATIN_ORGANIZATION		22	-0.4104772	-1.2031078	0.21052632	0.39974827	1	704	tags=59%, list=42%, signal=100%
GOBP_REGULATION_OF_PROTEIN_SERINE_THREONINE_KINASE_ACTIVITY		46	-0.3841432	-1.203064	0.17	0.3993098	1	820	tags=65%, list=48%, signal=123%
GOBP_REGULATION_OF_BINDING		50	-0.3817999	-1.2014929	0.16161616	0.40262008	1	670	tags=54%, list=40%, signal=87%
GOBP_INTRINSIC_APOPTOTIC_SIGNALING_PATHWAY_BY_P53_CLASS_MEDIA		10	-0.4871699	-1.2012415	0.21839081	0.40282604	1	849	tags=90%, list=50%, signal=179%
GOBP_POSITIVE_REGULATION_OF_CELL_CELL_ADHESION		33	-0.3988939	-1.2010092	0.23232323	0.40288392	1	659	tags=58%, list=39%, signal=92%
GOBP_CELLULAR_RESPONSE_TO_INORGANIC_SUBSTANCE		27	-0.382369	-1.2009898	0.21649484	0.40244403	1	748	tags=59%, list=44%, signal=104%
GOBP_TRANSFORMING_GROWTH_FACTOR_BETA_RECEPTOR_SIGNALING_PA		16	-0.4478278	-1.200829	0.18478261	0.40240043	1	628	tags=56%, list=37%, signal=89%
GOBP_REGULATION_OF_VIRAL_LIFE_CYCLE		13	-0.4757957	-1.2008175	0.24468085	0.40196195	1	448	tags=46%, list=26%, signal=62%

GOBP_CELL_PROJECTION_ORGANIZATION		152	-0.340119	-1.200519	0.05	0.40222368	1	559	tags=39%, list=33%, signal=54%
GOBP_EMBRYONIC_MORPHOGENESIS		38	-0.3778411	-1.2001894	0.24	0.40237164	1	468	tags=34%, list=28%, signal=46%
GOBP_MONOSACCHARIDE_BIOSYNTHETIC_PROCESS		26	-0.4175521	-1.1987865	0.18947369	0.4049699	1	241	tags=35%, list=14%, signal=40%
GOBP_REGULATION_OF_HYDROLASE_ACTIVITY		148	-0.343378	-1.1970092	0.08	0.4087977	1	362	tags=28%, list=21%, signal=32%
GOBP_RESPONSE_TO_MOLECULE_OF_BACTERIAL_ORIGIN		29	-0.378077	-1.1966136	0.24	0.40929684	1	698	tags=55%, list=41%, signal=92%
KEGG_PATHOGENIC_ESCHERICHIA_COLI_INFECTION		15	-0.437032	-1.1960804	0.2371134	0.40995058	1	668	tags=67%, list=39%, signal=109%
GOBP_POSITIVE_REGULATION_OF_LOCOMOTION		71	-0.3561295	-1.195388	0.15	0.41129902	1	405	tags=37%, list=24%, signal=46%
GOBP_STRIATED_MUSCLE_CELL_DEVELOPMENT		31	-0.3961356	-1.1944585	0.2020202	0.41357076	1	451	tags=39%, list=27%, signal=52%
GOBP_REGULATION_OF_INTRACELLULAR_TRANSPORT		69	-0.3581854	-1.1944072	0.15306123	0.41322413	1	595	tags=45%, list=35%, signal=66%
GOBP_REGULATION_OF_PROTEIN_MODIFICATION_PROCESS		151	-0.3446695	-1.1937943	0.09	0.41421688	1	707	tags=52%, list=42%, signal=81%
GOBP_MULTICELLULAR_ORGANISM_REPRODUCTION		36	-0.3786035	-1.1933905	0.20618556	0.41468313	1	518	tags=36%, list=31%, signal=51%
GOBP_POSITIVE_REGULATION_OF_PHAGOCYTOSIS		15	-0.4580469	-1.1933397	0.25531915	0.41439205	1	467	tags=47%, list=28%, signal=64%
REACTOME_G_ALPHA_12_13_SIGNALLING_EVENTS		11	-0.4982986	-1.1931568	0.2345679	0.41441283	1	406	tags=45%, list=24%, signal=59%
REACTOME_DNA_REPAIR		10	-0.4785751	-1.1912092	0.2173913	0.4186279	1	828	tags=80%, list=49%, signal=155%
GOBP_REGULATION_OF_CELLULAR_RESPONSE_TO_INSULIN_STIMULUS		12	-0.4483629	-1.1881478	0.24444444	0.42567274	1	469	tags=42%, list=28%, signal=57%
GOBP_ENZYME_LINKED_RECEPTOR_PROTEIN_SIGNALING_PATHWAY		85	-0.3529044	-1.1881292	0.16	0.4252596	1	518	tags=41%, list=31%, signal=56%
REACTOME_TRANSLOCATION_OF_SLC2A4_GLUT4_TO_THE_PLASMA_MEMBRA		28	-0.4084586	-1.1878262	0.2	0.4256765	1	560	tags=46%, list=33%, signal=68%
GOBP_REGULATION_OF_SUBSTRATE_ADHESION_DEPENDENT_CELL_SPREAD		19	-0.435515	-1.1864899	0.2371134	0.4287566	1	504	tags=53%, list=30%, signal=74%
GOBP_REGULATION_OF_CELL_SUBSTRATE_JUNCTION_ORGANIZATION		16	-0.4535301	-1.1861913	0.31182796	0.42894918	1	558	tags=56%, list=33%, signal=83%
GOBP_RESPONSE_TO_RADIATION		33	-0.3784636	-1.1858287	0.21649484	0.42957062	1	405	tags=36%, list=24%, signal=47%
GOBP_RESPONSE_TO_LIPID		82	-0.3398116	-1.1847701	0.13131313	0.4318418	1	706	tags=51%, list=42%, signal=84%
GOBP_CELLULAR_PROTEIN_COMPLEX_DISASSEMBLY		90	-0.3511806	-1.1846284	0.19	0.43157873	1	799	tags=58%, list=47%, signal=103%
GOBP_CELL_ADHESION_MEDIATED_BY_INTEGRIN		10	-0.4775666	-1.1845624	0.20481928	0.4311625	1	607	tags=60%, list=36%, signal=93%
REACTOME_TRANSLATION		185	-0.3365835	-1.1845235	0.11	0.43079096	1	862	tags=60%, list=51%, signal=109%
GOBP_GLUCOSE_CATABOLIC_PROCESS		15	-0.4542111	-1.184513	0.22340426	0.43035465	1	847	tags=87%, list=50%, signal=172%
GOBP_REGULATION_OF_PROTEIN_AUTOPHOSPHORYLATION		10	-0.5024506	-1.183811	0.25	0.4316379	1	205	tags=30%, list=12%, signal=34%
GOBP_REGULATION_OF_DEVELOPMENTAL_GROWTH		29	-0.408787	-1.1832341	0.1875	0.43256867	1	186	tags=28%, list=11%, signal=30%
GOBP_MRNA_PROCESSING		19	-0.4349506	-1.1827266	0.22826087	0.4333552	1	468	tags=47%, list=28%, signal=65%
GOBP_NEGATIVE_REGULATION_OF_NEURON_DEATH		20	-0.4179651	-1.1826779	0.25555557	0.43293938	1	640	tags=55%, list=38%, signal=87%
GOBP_REGULATION_OF_CELL_CYCLE_G1_S_PHASE_TRANSITION		12	-0.455907	-1.1826535	0.18888889	0.43249208	1	714	tags=67%, list=42%, signal=114%
REACTOME_METABOLISM_OF_AMINO_ACIDS_AND_DERIVATIVES		152	-0.3349298	-1.1825932	0.1	0.43214366	1	822	tags=59%, list=48%, signal=103%
GOBP_REGULATION_OF_MAP_KINASE_ACTIVITY		30	-0.4013641	-1.1825733	0.17171717	0.43170914	1	820	tags=73%, list=48%, signal=140%
GOBP_POSITIVE_REGULATION_OF_PRODUCTION_OF_MOLECULAR_MEDIATOR		11	-0.4518609	-1.1825055	0.1954023	0.43142754	1	725	tags=64%, list=43%, signal=110%
GOBP_LOCALIZATION_WITHIN_MEMBRANE		14	-0.4497298	-1.1821725	0.24175824	0.43169984	1	205	tags=29%, list=12%, signal=32%
GOBP_ENDOSOME_ORGANIZATION		13	-0.4664537	-1.1815786	0.22580644	0.4327096	1	621	tags=54%, list=37%, signal=84%
GOBP_NEGATIVE_REGULATION_OF_RESPONSE_TO_ENDOPLASMIC_RETICUL		10	-0.4750567	-1.1814721	0.29069766	0.43246034	1	605	tags=60%, list=36%, signal=93%
GOBP_TISSUE_MIGRATION		40	-0.3725472	-1.1813543	0.22	0.4322764	1	294	tags=28%, list=17%, signal=32%
GOBP_POSITIVE_REGULATION_OF_PROTEIN_MODIFICATION_PROCESS		95	-0.3438532	-1.1809115	0.18	0.43282765	1	629	tags=45%, list=37%, signal=68%
GOBP_REGULATION_OF_T_CELL_ACTIVATION		32	-0.3561988	-1.1790018	0.29591838	0.43709987	1	629	tags=50%, list=37%, signal=78%
GOBP_STRIATED_MUSCLE_CELL_DIFFERENTIATION		48	-0.3558302	-1.1782483	0.19	0.43846324	1	466	tags=35%, list=27%, signal=47%
GOBP_CELLULAR_RESPONSE_TO_NITROGEN_COMPOUND		99	-0.3420503	-1.1779968	0.15	0.43858555	1	418	tags=32%, list=25%, signal=40%
GOBP_MUSCLE_FILAMENT_SLIDING		26	-0.4143355	-1.1770176	0.21212122	0.4404933	1	270	tags=19%, list=16%, signal=23%
GOBP_POSITIVE_REGULATION_OF_VIRAL_PROCESS		12	-0.4849756	-1.1768858	0.30337077	0.44031337	1	600	tags=50%, list=35%, signal=77%
GOBP_RESPONSE_TO_INORGANIC_SUBSTANCE		91	-0.3471245	-1.176748	0.12	0.44020835	1	753	tags=56%, list=44%, signal=95%
GOBP_POSITIVE_REGULATION_OF_PROTEIN_SERINE_THREONINE_KINASE_A		30	-0.3906533	-1.1760486	0.24210526	0.44144288	1	576	tags=47%, list=34%, signal=69%
GOBP_REGULATION_OF_MEMBRANE_REPOLARIZATION		11	-0.4801069	-1.1760067	0.27586207	0.44111156	1	287	tags=36%, list=17%, signal=43%

REACTOME_REGULATION_OF_PLK1_ACTIVITY_AT_G2_M_TRANSITION	16	-0.4419462	-1.1757787	0.23157895	0.44112283	1	515	tags=50%, list=30%, signal=71%
GOBP_VESICLE_BUDDING_FROM_MEMBRANE	30	-0.3704386	-1.1757675	0.25773194	0.44068626	1	666	tags=50%, list=39%, signal=81%
REACTOME_COPI_MEDIATED_ANTEROGRADE_TRANSPORT	32	-0.3864058	-1.175482	0.17894737	0.44097647	1	892	tags=75%, list=53%, signal=155%
GOBP_NUCLEOBASE_CONTAINING_COMPOUND_TRANSPORT	21	-0.4099334	-1.1750125	0.19587629	0.4417146	1	536	tags=52%, list=32%, signal=76%
GOBP_REGULATION_OF_STRESS_ACTIVATED_PROTEIN_KINASE_SIGNALING_	17	-0.4160433	-1.1749957	0.26595744	0.44125742	1	278	tags=35%, list=16%, signal=42%
REACTOME_STRIATED_MUSCLE_CONTRACTION	21	-0.4137209	-1.1739028	0.19587629	0.44344926	1	270	tags=19%, list=16%, signal=22%
GOBP_CELL_CELL_SIGNALING	161	-0.3312638	-1.1724904	0.12	0.446314	1	548	tags=39%, list=32%, signal=52%
GOBP_REGULATION_OF_CELL_DEATH	202	-0.3319591	-1.1720725	0.07	0.44694662	1	640	tags=48%, list=38%, signal=68%
GOBP_CELL_MORPHOGENESIS_INVOLVED_IN_NEURON_DIFFERENTIATION	67	-0.3465512	-1.1718944	0.24	0.44680393	1	553	tags=42%, list=33%, signal=60%
GOBP_TYPE_I_INTERFERON_PRODUCTION	11	-0.4522563	-1.1696777	0.23255815	0.4515941	1	384	tags=36%, list=23%, signal=47%
GOBP_CARDIOCYTE_DIFFERENTIATION	13	-0.4591003	-1.169577	0.27058825	0.4513515	1	451	tags=38%, list=27%, signal=52%
GOBP_MESENCHYME_DEVELOPMENT	20	-0.4177546	-1.1685961	0.31578946	0.45362356	1	504	tags=45%, list=30%, signal=63%
REACTOME_DEREGULATED_CDK5_TRIGGERS_MULTIPLE_NEURODEGENERAT	10	-0.507166	-1.1685122	0.23809524	0.45344242	1	313	tags=50%, list=18%, signal=61%
GOBP_INTRINSIC_APOPTOTIC_SIGNALING_PATHWAY_IN_RESPONSE_TO_OXI	11	-0.4598262	-1.1681584	0.28089887	0.45388302	1	522	tags=55%, list=31%, signal=78%
GOBP_B_CELL_MEDIATED_IMMUNITY	14	-0.4439623	-1.1680136	0.24719101	0.45371222	1	573	tags=64%, list=34%, signal=96%
GOBP_LOCOMOTION	186	-0.3358643	-1.1668602	0.07	0.45613965	1	560	tags=40%, list=33%, signal=54%
GOBP_ENDOTHELIUM_DEVELOPMENT	19	-0.4215303	-1.1667653	0.22340426	0.4559356	1	653	tags=47%, list=39%, signal=76%
GOBP_CARDIAC_VENTRICLE_DEVELOPMENT	12	-0.4623569	-1.166309	0.30952382	0.45677134	1	115	tags=17%, list=7%, signal=18%
GOBP_LOW_DENSITY_LIPOPROTEIN_PARTICLE_CLEARANCE	13	-0.4377627	-1.1662936	0.2826087	0.45630467	1	531	tags=54%, list=31%, signal=78%
KEGG_CELL_ADHESION_MOLECULES_CAMS	12	-0.4685559	-1.1649984	0.2840909	0.45917016	1	836	tags=83%, list=49%, signal=163%
KEGG_CHEMOKINE_SIGNALING_PATHWAY	22	-0.4009942	-1.1646215	0.2783505	0.45976803	1	367	tags=32%, list=22%, signal=40%
GOBP_CELLULAR_RESPONSE_TO_MOLECULE_OF_BACTERIAL_ORIGIN	16	-0.4255751	-1.1644875	0.23655914	0.45971766	1	698	tags=56%, list=41%, signal=95%
GOBP_RECEPTOR_CATABOLIC_PROCESS	13	-0.4292509	-1.1635426	0.27272728	0.46159738	1	974	tags=100%, list=57%, signal=233%
GOBP_GLYCOSYL_COMPOUND_METABOLIC_PROCESS	23	-0.3921261	-1.1613772	0.29787233	0.4669039	1	629	tags=48%, list=37%, signal=75%
GOBP_POSITIVE_REGULATION_OF_PROTEIN_METABOLIC_PROCESS	169	-0.3297228	-1.1611145	0.12	0.4673149	1	576	tags=41%, list=34%, signal=56%
GOBP_POSITIVE_REGULATION_OF_PROTEIN_PHOSPHORYLATION	78	-0.3483288	-1.1611093	0.23	0.46681947	1	576	tags=42%, list=34%, signal=61%
GOBP_REGULATION_OF_RESPONSE_TO_DNA_DAMAGE_STIMULUS	14	-0.4391404	-1.1605479	0.27586207	0.46772823	1	853	tags=86%, list=50%, signal=171%
GOBP_MYOTUBE_DIFFERENTIATION	22	-0.4070568	-1.1602682	0.21276596	0.46794954	1	373	tags=36%, list=22%, signal=46%
GOBP_EPITHELIAL_CELL_PROLIFERATION	28	-0.3832396	-1.1596668	0.24742268	0.46892765	1	278	tags=25%, list=16%, signal=29%
GOBP_PALLIUM_DEVELOPMENT	12	-0.4791191	-1.1595961	0.2826087	0.46859804	1	237	tags=33%, list=14%, signal=38%
GOBP_POSITIVE_REGULATION_OF_PROTEIN_MODIFICATION_BY_SMALL_PRO	14	-0.4363454	-1.1594867	0.28089887	0.4684452	1	699	tags=64%, list=41%, signal=108%
GOBP_REGULATION_OF_CELLULAR_RESPONSE_TO_GROWTH_FACTOR_STIM	26	-0.3885858	-1.1589992	0.26041666	0.46911982	1	472	tags=42%, list=28%, signal=58%
GOBP_NEGATIVE_REGULATION_OF_CELL_CELL_ADHESION	17	-0.437019	-1.1589184	0.2826087	0.46893582	1	658	tags=59%, list=39%, signal=95%
GOBP_RESPONSE_TO_HEAT	27	-0.3618765	-1.1586285	0.2371134	0.46917468	1	719	tags=59%, list=42%, signal=101%
GOBP_MONOSACCHARIDE_METABOLIC_PROCESS	47	-0.3606644	-1.1583483	0.25	0.46946535	1	281	tags=30%, list=17%, signal=35%
GOBP_REGULATION_OF_CELLULAR_COMPONENT_MOVEMENT	119	-0.3400742	-1.1576203	0.12	0.47096038	1	728	tags=52%, list=43%, signal=85%
REACTOME_GLUCOSE_METABOLISM	27	-0.3817335	-1.1570361	0.26262626	0.47190657	1	552	tags=48%, list=33%, signal=70%
GOBP_EPITHELIAL_CELL_DIFFERENTIATION	55	-0.3390785	-1.1568776	0.22	0.4720391	1	653	tags=45%, list=39%, signal=72%
GOBP_REGULATION_OF_MONOOXYGENASE_ACTIVITY	12	-0.4554476	-1.1567341	0.2967033	0.47194615	1	306	tags=42%, list=18%, signal=50%
GOBP_ORGANOPHOSPHATE_CATABOLIC_PROCESS	27	-0.3956937	-1.1559982	0.20618556	0.47321513	1	887	tags=70%, list=52%, signal=145%
GOBP_REGULATION_OF_SMALL_MOLECULE_METABOLIC_PROCESS	69	-0.3509907	-1.1559716	0.2	0.47277278	1	820	tags=61%, list=48%, signal=113%
GOBP_PROTEIN_AUTOPHOSPHORYLATION	19	-0.431411	-1.1551521	0.22727273	0.4744795	1	205	tags=26%, list=12%, signal=30%
REACTOME_METABOLISM_OF_NUCLEOTIDES	17	-0.4371464	-1.1548711	0.23913044	0.4745174	1	614	tags=59%, list=36%, signal=91%
REACTOME_EXTRA_NUCLEAR_ESTROGEN_SIGNALING	13	-0.4564239	-1.1543126	0.2967033	0.47547212	1	683	tags=69%, list=40%, signal=115%
GOBP_SPINDLE_ORGANIZATION	24	-0.3887711	-1.1540741	0.2173913	0.47547716	1	892	tags=71%, list=53%, signal=147%
GOBP_NEGATIVE_REGULATION_OF_ANION_TRANSPORT	29	-0.382253	-1.153515	0.23958333	0.4764707	1	455	tags=41%, list=27%, signal=56%

GOBP_REGULATION_OF_DNA_BINDING_TRANSCRIPTION_FACTOR_ACTIVITY	36	-0.3644191	-1.1533252	0.2783505	0.4764753	1	384	tags=36%, list=23%, signal=46%
GOBP_POSITIVE_REGULATION_OF_INTRINSIC_APOPTOTIC_SIGNALING_PATH	11	-0.4702583	-1.1530359	0.3068182	0.47685587	1	810	tags=73%, list=48%, signal=138%
GOBP_PEPTIDE_SECRETION	58	-0.3562035	-1.1525462	0.19	0.47751075	1	393	tags=33%, list=23%, signal=41%
GOBP_RIBOSOMAL_SMALL_SUBUNIT_ASSEMBLY	11	-0.4682216	-1.1510466	0.25287357	0.48096317	1	638	tags=64%, list=38%, signal=101%
GOBP_SMALL_GTPASE_MEDIATED_SIGNAL_TRANSDUCTION	49	-0.3556121	-1.1507754	0.24242425	0.4811758	1	406	tags=35%, list=24%, signal=44%
GOBP_STEROL_HOMEOSTASIS	14	-0.4318871	-1.1507605	0.23913044	0.48069957	1	941	tags=86%, list=56%, signal=191%
GOBP_REGULATION_OF_PHOSPHOLIPID_METABOLIC_PROCESS	11	-0.4618392	-1.1497694	0.31034482	0.4826009	1	210	tags=27%, list=12%, signal=31%
GOBP_POSITIVE_REGULATION_OF_CELL_MATRIX_ADHESION	11	-0.4600429	-1.1490577	0.3253012	0.48383015	1	182	tags=27%, list=11%, signal=30%
GOBP_AMIDE_BIOSYNTHETIC_PROCESS	247	-0.3147334	-1.1485698	0.07	0.48461476	1	866	tags=58%, list=51%, signal=101%
GOBP_RESPONSE_TO_NITROGEN_COMPOUND	163	-0.318031	-1.1484846	0.15	0.48428798	1	384	tags=29%, list=23%, signal=34%
KEGG_MAPK_SIGNALING_PATHWAY	27	-0.384631	-1.1483899	0.3030303	0.4840426	1	353	tags=33%, list=21%, signal=41%
GOBP_VIRION_ASSEMBLY	10	-0.4568407	-1.1483643	0.2962963	0.4836065	1	388	tags=40%, list=23%, signal=52%
GOBP_CELLULAR_RESPONSE_TO_INSULIN_STIMULUS	34	-0.3663036	-1.1481328	0.2755102	0.48376408	1	941	tags=68%, list=56%, signal=149%
GOBP_LAMELLIPODIUM_ASSEMBLY	12	-0.447474	-1.1480114	0.26966292	0.48351938	1	330	tags=42%, list=19%, signal=51%
REACTOME_SIGNALING_BY_BRAF_AND_RAF_FUSIONS	24	-0.3861223	-1.1480052	0.25263157	0.48309526	1	719	tags=58%, list=42%, signal=100%
GOBP_ENDOPLASMIC_RETICULUM_TO_GOLGI_VESICLE_MEDIATED_TRANSPO	51	-0.3489687	-1.1477225	0.24242425	0.4833433	1	892	tags=73%, list=53%, signal=149%
GOBP_SUBSTRATE_ADHESION_DEPENDENT_CELL_SPREADING	27	-0.3988786	-1.1467416	0.26041666	0.48553243	1	504	tags=44%, list=30%, signal=62%
GOBP_ORGANONITROGEN_COMPOUND_CATABOLIC_PROCESS	223	-0.3271876	-1.1461434	0.12	0.48661777	1	832	tags=56%, list=49%, signal=96%
GOBP_PH_REDUCTION	11	-0.4680727	-1.1457784	0.26086956	0.486911	1	497	tags=36%, list=29%, signal=51%
GOBP_POSITIVE_REGULATION_OF_APOPTOTIC_SIGNALING_PATHWAY	15	-0.4201377	-1.1451514	0.31111112	0.48791093	1	864	tags=73%, list=51%, signal=148%
GOBP_REGULATION_OF_ENDOCYTOSIS	41	-0.3560613	-1.1448623	0.29	0.48809344	1	768	tags=61%, list=45%, signal=109%
REACTOME_RNA_POLYMERASE_II_TRANSCRIPTION	92	-0.3343388	-1.144776	0.2	0.48783705	1	548	tags=39%, list=32%, signal=55%
GOBP_CELL_MIGRATION	152	-0.3310654	-1.1445235	0.11	0.48794892	1	432	tags=32%, list=25%, signal=39%
GOBP_REGULATION_OF_PHOSPHORUS_METABOLIC_PROCESS	164	-0.3187951	-1.1430786	0.13	0.4910796	1	811	tags=55%, list=48%, signal=95%
GOBP_REGULATION_OF_PROTEIN_LOCALIZATION_TO_MEMBRANE	46	-0.3585563	-1.1429694	0.29	0.4908207	1	687	tags=54%, list=41%, signal=89%
GOBP_CELL_KILLING	21	-0.3913001	-1.1427542	0.28282827	0.4909397	1	323	tags=38%, list=19%, signal=46%
GOBP_NEGATIVE_REGULATION_OF_CELL_ACTIVATION	17	-0.423345	-1.1426535	0.3125	0.49068138	1	313	tags=35%, list=18%, signal=43%
GOBP_MUSCLE_ORGAN_DEVELOPMENT	68	-0.3412071	-1.1423526	0.2	0.49093938	1	456	tags=34%, list=27%, signal=44%
GOBP_RESPONSE_TO_CARBOHYDRATE	34	-0.3685493	-1.1420585	0.33	0.49127552	1	391	tags=35%, list=23%, signal=45%
GOBP_INTRACELLULAR_PROTEIN_TRANSPORT	267	-0.3192701	-1.1416719	0.09	0.49167976	1	735	tags=49%, list=43%, signal=74%
GOBP_REACTIVE_OXYGEN_SPECIES_BIOSYNTHETIC_PROCESS	24	-0.4080973	-1.1407839	0.25773194	0.4937871	1	386	tags=42%, list=23%, signal=53%
GOBP_BIOMINERALIZATION	14	-0.431275	-1.1407318	0.26086956	0.49338967	1	683	tags=50%, list=40%, signal=83%
GOBP_REGULATION_OF_PEPTIDE_TRANSPORT	91	-0.329482	-1.1406506	0.24	0.49306166	1	399	tags=33%, list=24%, signal=41%
GOBP_ACTIVATION_OF_MAPK_ACTIVITY	16	-0.4179583	-1.1394882	0.2967033	0.49570397	1	313	tags=38%, list=18%, signal=46%
GOBP_AXON_DEVELOPMENT	62	-0.3352524	-1.1394825	0.26	0.49519765	1	659	tags=47%, list=39%, signal=74%
GOBP_NEGATIVE_REGULATION_OF_LOCOMOTION	30	-0.387621	-1.1389534	0.29896906	0.496183	1	227	tags=20%, list=13%, signal=23%
GOBP_RESPONSE_TO_IONIZING_RADIATION	14	-0.4418379	-1.1383619	0.25	0.49720547	1	252	tags=36%, list=15%, signal=42%
GOBP_TISSUE_REMODELING	22	-0.3788193	-1.1379108	0.26595744	0.49787438	1	309	tags=32%, list=18%, signal=38%
GOBP_PROTEIN_IMPORT	36	-0.3651119	-1.1378728	0.25510204	0.49748504	1	395	tags=33%, list=23%, signal=43%
GOBP_CELLULAR_ALDEHYDE_METABOLIC_PROCESS	15	-0.4207245	-1.1376632	0.33333334	0.49761447	1	949	tags=87%, list=56%, signal=195%
GOBP_RIBOSOMAL_LARGE_SUBUNIT_BIOGENESIS	18	-0.4376224	-1.1369199	0.2795699	0.49894428	1	737	tags=72%, list=43%, signal=126%
GOBP_MEMBRANE_BIOGENESIS	11	-0.458215	-1.1368032	0.25301206	0.49867246	1	659	tags=64%, list=39%, signal=103%
KEGG_LONG_TERM_POTENTIATION	15	-0.4243958	-1.1358552	0.30769232	0.50067097	1	309	tags=27%, list=18%, signal=32%
GOBP_NEGATIVE_REGULATION_OF_IMMUNE_EFFECTOR_PROCESS	19	-0.3882622	-1.1358353	0.34444445	0.50025165	1	601	tags=58%, list=35%, signal=89%
GOBP_ADAPTIVE_IMMUNE_RESPONSE_BASED_ON_SOMATIC_RECOMBINATIO	23	-0.3981789	-1.1358316	0.29166666	0.49974582	1	573	tags=48%, list=34%, signal=71%
GOBP_POSITIVE_REGULATION_OF_PHOSPHORUS_METABOLIC_PROCESS	94	-0.3343331	-1.1354752	0.23	0.50013363	1	576	tags=41%, list=34%, signal=59%

GOBP_REGULATION_OF_PROTEIN_KINASE_ACTIVITY		78	-0.3396638	-1.1349005	0.25252524	0.50120133	1	657	tags=46%, list=39%, signal=72%
GOBP_ESTABLISHMENT_OF_PROTEIN_LOCALIZATION		389	-0.3131978	-1.134877	0.07	0.50073475	1	735	tags=48%, list=43%, signal=66%
REACTOME_ONCOGENIC_MAPK_SIGNALING		25	-0.3857463	-1.134792	0.34736842	0.50050163	1	719	tags=56%, list=42%, signal=96%
GOBP_FOREBRAIN_DEVELOPMENT		20	-0.3955799	-1.1340617	0.3043478	0.5018761	1	237	tags=25%, list=14%, signal=29%
GOBP_RECEPTOR_METABOLIC_PROCESS		34	-0.3679819	-1.1339847	0.30612245	0.50163245	1	974	tags=85%, list=57%, signal=196%
GOBP_SEX_DIFFERENTIATION		12	-0.4400477	-1.1336974	0.30337077	0.5018619	1	407	tags=42%, list=24%, signal=54%
GOBP_RAS_PROTEIN_SIGNAL_TRANSDUCTION		41	-0.352427	-1.1328813	0.27272728	0.5032399	1	406	tags=34%, list=24%, signal=44%
REACTOME_PLATELET_HOMEOSTASIS		17	-0.4085899	-1.1310471	0.3263158	0.50740665	1	683	tags=59%, list=40%, signal=98%
GOBP_LEUKOCYTE_MIGRATION		60	-0.3395858	-1.1308572	0.32	0.5074375	1	386	tags=33%, list=23%, signal=42%
GOBP_NEGATIVE_REGULATION_OF_SIGNALING		144	-0.3318449	-1.1303422	0.16	0.50829506	1	705	tags=52%, list=42%, signal=82%
GOBP_MUSCLE_CELL_PROLIFERATION		14	-0.4604861	-1.1301085	0.35632184	0.5085178	1	241	tags=36%, list=14%, signal=41%
GOBP_EPITHELIAL_TUBE_MORPHOGENESIS		16	-0.4139606	-1.1300212	0.29347825	0.508279	1	39	tags=13%, list=2%, signal=13%
GOBP_NEUTRAL_LIPID_CATABOLIC_PROCESS		12	-0.4457078	-1.1297545	0.29411766	0.5085876	1	391	tags=42%, list=23%, signal=54%
GOBP_NEGATIVE_REGULATION_OF_MULTICELLULAR_ORGANISMAL_PROCES		106	-0.337092	-1.1290694	0.25	0.50971085	1	393	tags=30%, list=23%, signal=37%
GOBP_ERK1_AND_ERK2_CASCADE		37	-0.3425694	-1.1289167	0.30612245	0.5096059	1	657	tags=49%, list=39%, signal=78%
REACTOME_DEGRADATION_OF_THE_EXTRACELLULAR_MATRIX		20	-0.4025101	-1.1284447	0.25	0.51051384	1	341	tags=35%, list=20%, signal=43%
GOBP_POSITIVE_REGULATION_OF_SECRETION		38	-0.3607659	-1.1284277	0.29896906	0.5100449	1	393	tags=34%, list=23%, signal=44%
GOBP_EMBRYO_DEVELOPMENT		69	-0.342821	-1.1282501	0.3	0.50989157	1	629	tags=45%, list=37%, signal=69%
GOBP_TUMOR_NECROSIS_FACTOR_SUPERFAMILY_CYTOKINE_PRODUCTION		11	-0.4468215	-1.1279854	0.32608697	0.5101299	1	502	tags=55%, list=30%, signal=77%
GOBP_NEGATIVE_REGULATION_OF_COAGULATION		13	-0.4482632	-1.127347	0.32291666	0.5113095	1	683	tags=62%, list=40%, signal=102%
GOBP_GLAND_DEVELOPMENT		40	-0.3551949	-1.1271642	0.30208334	0.5113741	1	344	tags=28%, list=20%, signal=34%
GOBP_DEVELOPMENTAL_GROWTH_INVOLVED_IN_MORPHOGENESIS		24	-0.3833452	-1.1268315	0.2783505	0.5115995	1	399	tags=38%, list=24%, signal=48%
GOBP_CELLULAR_AMINO_ACID_METABOLIC_PROCESS		90	-0.3291262	-1.126371	0.24	0.5122055	1	919	tags=62%, list=54%, signal=129%
GOBP_MUSCLE_CELL_APOPTOTIC_PROCESS		10	-0.4668742	-1.1258066	0.3448276	0.51318824	1	147	tags=30%, list=9%, signal=33%
GOBP_POSITIVE_REGULATION_OF_REACTIVE_OXYGEN_SPECIES_METABOLI		16	-0.4114331	-1.1257627	0.30769232	0.51283383	1	306	tags=38%, list=18%, signal=45%
GOBP_MULTICELLULAR_ORGANISM_GROWTH		14	-0.4326101	-1.124332	0.32978722	0.516087	1	115	tags=21%, list=7%, signal=23%
GOBP_CYTOPLASMIC_MICROTUBULE_ORGANIZATION		11	-0.4366343	-1.123718	0.31460676	0.51732934	1	735	tags=73%, list=43%, signal=128%
REACTOME_ADORA2B_MEDIATED_ANTI_INFLAMMATORY_CYTOKINES_PRODU		12	-0.4627198	-1.1236523	0.29545453	0.5170386	1	578	tags=50%, list=34%, signal=75%
REACTOME_SIGNALING_BY_GPCR		42	-0.3536558	-1.1236368	0.32	0.5165594	1	620	tags=43%, list=37%, signal=66%
REACTOME_DISORDERS_OF_TRANSMEMBRANE_TRANSPORTERS		37	-0.3742625	-1.1235406	0.26	0.5162602	1	192	tags=24%, list=11%, signal=27%
REACTOME_ASPARAGINE_N_LINKED_GLYCOSYLATION		65	-0.3400892	-1.1222849	0.27272728	0.51910776	1	905	tags=69%, list=53%, signal=143%
GOBP_NEGATIVE_REGULATION_OF_CELL_GROWTH		20	-0.3957118	-1.1217715	0.32222223	0.5199629	1	308	tags=25%, list=18%, signal=30%
GOBP_T_CELL_PROLIFERATION		23	-0.3874939	-1.1216865	0.3469388	0.5197265	1	629	tags=52%, list=37%, signal=82%
GOBP_REGULATION_OF_ENDOTHELIAL_CELL_MIGRATION		26	-0.3784988	-1.1206589	0.36734694	0.5216802	1	294	tags=27%, list=17%, signal=32%
GOBP_NEGATIVE_REGULATION_OF_PROTEIN_SERINE_THREONINE_KINASE_A		15	-0.4375435	-1.1198689	0.32291666	0.5232539	1	657	tags=67%, list=39%, signal=108%
GOBP_NUCLEOSIDE_METABOLIC_PROCESS		18	-0.4141037	-1.1195792	0.31182796	0.52348435	1	629	tags=50%, list=37%, signal=79%
REACTOME_CLATHRIN_MEDIATED_ENDOCYTOSIS		28	-0.3883291	-1.1194752	0.29591838	0.5233021	1	828	tags=82%, list=49%, signal=158%
GOBP_MICROTUBULE_CYTOSKELETON_ORGANIZATION_INVOLVED_IN_MITOS		23	-0.3843983	-1.1185914	0.32	0.5251497	1	892	tags=70%, list=53%, signal=145%
REACTOME_POST_TRANSLATIONAL_PROTEIN_MODIFICATION		177	-0.3294691	-1.1184341	0.19	0.52506	1	759	tags=55%, list=45%, signal=89%
GOBP_SECOND_MESSENGER_MEDIATED_SIGNALING		41	-0.3602699	-1.1175823	0.26	0.52668715	1	309	tags=27%, list=18%, signal=32%
GOBP_POSITIVE_REGULATION_OF_MAP_KINASE_ACTIVITY		20	-0.3867796	-1.1175742	0.30208334	0.52619493	1	810	tags=70%, list=48%, signal=132%
GOBP_PROTEIN_LOCALIZATION_TO_MEMBRANE		198	-0.3194732	-1.1171176	0.18	0.5269157	1	640	tags=44%, list=38%, signal=62%
GOBP_REGULATION_OF_BLOOD_PRESSURE		18	-0.4137497	-1.1161752	0.34042552	0.52886266	1	683	tags=56%, list=40%, signal=92%
KEGG_ARGININE_AND_PROLINE_METABOLISM		19	-0.3956356	-1.1157635	0.3548387	0.5293092	1	910	tags=79%, list=54%, signal=169%
GOBP_ESTABLISHMENT_OF_ORGANELLE_LOCALIZATION		65	-0.3289446	-1.1143059	0.29	0.5327531	1	536	tags=43%, list=32%, signal=61%
GOBP_MICROTUBULE_BASED_PROCESS		76	-0.3366682	-1.1122575	0.24242425	0.5380105	1	560	tags=43%, list=33%, signal=62%

GOBP_VESICLE_ORGANIZATION		70	-0.3268246	-1.1120929	0.29	0.538002	1	666	tags=46%, list=39%, signal=72%
GOBP_MITOCHONDRION_MORPHOGENESIS		10	-0.4707841	-1.1120056	0.3548387	0.5376597	1	814	tags=70%, list=48%, signal=134%
GOBP_REGULATION_OF_HUMORAL_IMMUNE_RESPONSE		13	-0.4196871	-1.111065	0.32258064	0.5395848	1	573	tags=62%, list=34%, signal=92%
GOBP_NEGATIVE_REGULATION_OF_DNA_BINDING_TRANSCRIPTION_FACTOR_		12	-0.4406527	-1.1106946	0.35955057	0.5399535	1	366	tags=42%, list=22%, signal=53%
GOBP_POSITIVE_REGULATION_OF_EXOCYTOSIS		15	-0.4326915	-1.1106197	0.34375	0.5395917	1	640	tags=67%, list=38%, signal=106%
GOBP_ORGANELLE_LOCALIZATION		103	-0.3161141	-1.1105486	0.24	0.5393691	1	640	tags=45%, list=38%, signal=67%
GOBP_RESPONSE_TO_OXIDATIVE_STRESS		78	-0.3245329	-1.1103905	0.29	0.53934956	1	820	tags=58%, list=48%, signal=107%
REACTOME_INTERLEUKIN_12_FAMILY_SIGNALING		21	-0.3828916	-1.1090596	0.3723404	0.5422128	1	567	tags=52%, list=33%, signal=78%
GOBP_LYMPHOCYTE_ACTIVATION_INVOLVED_IN_IMMUNE_RESPONSE		15	-0.4188496	-1.1089475	0.34736842	0.54200655	1	301	tags=40%, list=18%, signal=48%
GOBP_MUSCLE_STRUCTURE_DEVELOPMENT		105	-0.3254248	-1.108875	0.28	0.5417183	1	466	tags=34%, list=27%, signal=44%
GOBP_RESPONSE_TO_ENDOGENOUS_STIMULUS		182	-0.3174071	-1.1077101	0.25	0.54402107	1	683	tags=46%, list=40%, signal=68%
REACTOME_IRON_UPTAKE_AND_TRANSPORT		16	-0.3974013	-1.1077017	0.39361703	0.5435202	1	890	tags=69%, list=53%, signal=143%
GOBP_REGULATION_OF_DENDRITE_DEVELOPMENT		11	-0.4346107	-1.1074059	0.2747253	0.5438814	1	733	tags=55%, list=43%, signal=95%
REACTOME_MHC_CLASS_II_ANTIGEN_PRESENTATION		33	-0.3484063	-1.1070442	0.3030303	0.54432493	1	759	tags=61%, list=45%, signal=108%
GOBP_CELL_CELL_JUNCTION_ASSEMBLY		21	-0.3927684	-1.1070379	0.33333334	0.5438162	1	653	tags=52%, list=39%, signal=84%
GOBP_PROTEIN_CONTAINING_COMPLEX_DISASSEMBLY		105	-0.3230375	-1.1069838	0.31	0.5434089	1	755	tags=52%, list=45%, signal=89%
GOBP_REGULATION_OF_CELLULAR_LOCALIZATION		153	-0.3156461	-1.1069359	0.19	0.54299337	1	659	tags=46%, list=39%, signal=69%
GOBP_MUSCLE_CELL_DEVELOPMENT		37	-0.3460985	-1.1067446	0.3298969	0.543126	1	466	tags=35%, list=27%, signal=47%
REACTOME_INTEGRATION_OF_ENERGY_METABOLISM		25	-0.3693062	-1.1066797	0.30927834	0.54283017	1	558	tags=44%, list=33%, signal=65%
GOBP_TRANSMEMBRANE_RECEPTOR_PROTEIN_TYROSINE_KINASE_SIGNALIN		63	-0.3270089	-1.1064825	0.28282827	0.5428712	1	691	tags=51%, list=41%, signal=83%
GOBP_NEGATIVE_REGULATION_OF_NUCLEOBASE_CONTAINING_COMPOUND_		69	-0.3245757	-1.1055306	0.29	0.5449048	1	629	tags=46%, list=37%, signal=71%
REACTOME_REGULATION_OF_INSULIN_LIKE_GROWTH_FACTOR_IGF_TRANSP		32	-0.3597762	-1.1050595	0.3131313	0.54574287	1	820	tags=63%, list=48%, signal=119%
GOBP_CELLULAR_RESPONSE_TO_EXTRACELLULAR_STIMULUS		20	-0.3795535	-1.105027	0.33333334	0.54527307	1	669	tags=60%, list=39%, signal=98%
GOBP_NEURON_PROJECTION_EXTENSION		20	-0.41185	-1.1049408	0.3469388	0.5449759	1	399	tags=40%, list=24%, signal=52%
GOBP_CELL_GROWTH		50	-0.3404316	-1.1046041	0.32	0.54528636	1	558	tags=40%, list=33%, signal=58%
GOBP_POLYOL_METABOLIC_PROCESS		14	-0.42829	-1.1037688	0.34408602	0.5468281	1	426	tags=43%, list=25%, signal=57%
REACTOME_INTERFERON_SIGNALING		16	-0.3996852	-1.1037401	0.33695653	0.5463951	1	836	tags=75%, list=49%, signal=147%
GOBP_LOCOMOTORY_BEHAVIOR		14	-0.4112195	-1.1033458	0.37777779	0.5468932	1	189	tags=29%, list=11%, signal=32%
GOBP_REGULATION_OF_CELLULAR_AMIDE_METABOLIC_PROCESS		70	-0.3358513	-1.1031005	0.28282827	0.5471297	1	866	tags=64%, list=51%, signal=126%
GOBP_CELLULAR_RESPONSE_TO_CHEMICAL_STRESS		61	-0.335789	-1.1021595	0.33	0.54905057	1	814	tags=61%, list=48%, signal=112%
REACTOME_COPI_INDEPENDENT_GOLGI_TO_ER_RETROGRADE_TRAFFIC		16	-0.4065703	-1.1020274	0.3655914	0.5489147	1	234	tags=31%, list=14%, signal=36%
GOBP_REGULATION_OF_CYSTEINE_TYPE_ENDOPEPTIDASE_ACTIVITY		32	-0.373062	-1.1012706	0.36363637	0.5505083	1	158	tags=19%, list=9%, signal=20%
GOBP_POSITIVE_REGULATION_OF_LEUKOCYTE_CELL_CELL_ADHESION		26	-0.3677402	-1.1012499	0.33673468	0.55006516	1	619	tags=50%, list=37%, signal=78%
GOBP_APOPTOTIC_SIGNALING_PATHWAY		85	-0.3304637	-1.1010559	0.29	0.54998237	1	687	tags=51%, list=41%, signal=81%
GOBP_MITOTIC_SPINDLE_ORGANIZATION		21	-0.3947229	-1.0994358	0.4021739	0.55408436	1	515	tags=43%, list=30%, signal=61%
GOBP_CELLULAR_RESPONSE_TO_OXYGEN_CONTAINING_COMPOUND		139	-0.3186102	-1.0992619	0.25	0.5541774	1	699	tags=48%, list=41%, signal=75%
GOBP_REGULATION_OF_CELLULAR_RESPONSE_TO_HEAT		12	-0.4333529	-1.0983452	0.37634408	0.5562679	1	353	tags=42%, list=21%, signal=52%
KEGG_VIRAL_MYOCARDITIS		21	-0.3866808	-1.0972854	0.34375	0.55856115	1	619	tags=48%, list=37%, signal=74%
GOBP_OSTEOBLAST_DIFFERENTIATION		25	-0.3722667	-1.0971365	0.39583334	0.5583727	1	536	tags=40%, list=32%, signal=58%
GOBP_REGULATION_OF_EXOCYTOSIS		31	-0.3536044	-1.097085	0.33333334	0.5580325	1	393	tags=35%, list=23%, signal=45%
GOBP_MODULATION_OF_PROCESS_OF_OTHER_ORGANISM_INVOLVED_IN_SY		17	-0.3859421	-1.0970708	0.33684212	0.55756813	1	878	tags=71%, list=52%, signal=145%
REACTOME_CELLULAR_RESPONSE_TO_CHEMICAL_STRESS		62	-0.3231438	-1.0967883	0.34	0.5577826	1	548	tags=39%, list=32%, signal=55%
GOBP_REGULATION_OF_PROTEIN_TARGETING		16	-0.3895046	-1.0955176	0.35555556	0.5607409	1	455	tags=44%, list=27%, signal=59%
GOBP_REGULATION_OF_CELLULAR_RESPONSE_TO_TRANSFORMING_GROWT		11	-0.4337538	-1.0955101	0.36666667	0.5602574	1	805	tags=73%, list=47%, signal=138%
GOBP_GRANULOCYTE_MIGRATION		19	-0.3690297	-1.0945071	0.33695653	0.5624075	1	362	tags=37%, list=21%, signal=46%
GOBP_SPROUTING_ANGIOGENESIS		11	-0.442176	-1.0940362	0.3409091	0.5631488	1	344	tags=36%, list=20%, signal=45%

GOBP_POSITIVE_REGULATION_OF_CELL_CYCLE_PROCESS		18	-0.3918138	-1.093264	0.34065935	0.56478417	1	534	tags=50%, list=32%, signal=72%
GOBP_NEURON_DEVELOPMENT		108	-0.3207421	-1.0930622	0.31	0.56491894	1	558	tags=36%, list=33%, signal=50%
GOBP_CERAMIDE_METABOLIC_PROCESS		12	-0.444678	-1.0925753	0.36263737	0.5659044	1	308	tags=33%, list=18%, signal=40%
REACTOME_LYSOSOME_VESICLE_BIOGENESIS		12	-0.4338068	-1.0925022	0.31182796	0.565613	1	368	tags=50%, list=22%, signal=63%
GOBP_RESPONSE_TO_BACTERIUM		60	-0.3458629	-1.0918999	0.2929293	0.5668245	1	728	tags=55%, list=43%, signal=93%
GOBP_LIPID_HOMEOSTASIS		21	-0.3856939	-1.091572	0.4347826	0.567143	1	274	tags=29%, list=16%, signal=34%
REACTOME_TRANS_GOLGI_NETWORK_VESICLE_BUDDING		22	-0.3886942	-1.090229	0.36842105	0.57029605	1	368	tags=41%, list=22%, signal=52%
GOBP_CELLULAR_MACROMOLECULE_LOCALIZATION		389	-0.3010333	-1.0896242	0.17	0.5713784	1	735	tags=48%, list=43%, signal=65%
GOBP_MESENCHYMAL_CELL_DIFFERENTIATION		15	-0.4131965	-1.0895985	0.31914893	0.57091606	1	504	tags=47%, list=30%, signal=66%
GOBP_REGULATION_OF_GROWTH		55	-0.3416275	-1.0881999	0.30612245	0.5740112	1	186	tags=22%, list=11%, signal=24%
GOBP_REGULATION_OF_LEUKOCYTE_PROLIFERATION		21	-0.3809435	-1.0877597	0.34375	0.57450634	1	810	tags=67%, list=48%, signal=126%
GOBP_POSITIVE_REGULATION_OF_CELL_SUBSTRATE_ADHESION		28	-0.3815157	-1.0873609	0.41237113	0.5749996	1	182	tags=25%, list=11%, signal=28%
GOBP_CELLULAR_RESPONSE_TO_EXTERNAL_STIMULUS		28	-0.3659585	-1.0868301	0.34	0.5758786	1	669	tags=54%, list=39%, signal=87%
GOBP_REGULATION_OF_PROTEIN_LOCALIZATION_TO_CELL_PERIPHERY		29	-0.3644974	-1.0868231	0.32291666	0.5753709	1	425	tags=41%, list=25%, signal=54%
GOBP_POSITIVE_REGULATION_OF_SUBSTRATE_ADHESION_DEPENDENT_CEL		17	-0.4025583	-1.0867	0.3814433	0.5751009	1	182	tags=29%, list=11%, signal=33%
GOBP_POSITIVE_REGULATION_OF_ENDOCYTOSIS		22	-0.3832235	-1.0863662	0.34042552	0.575496	1	508	tags=45%, list=30%, signal=64%
GOBP_REGULATION_OF_PROTEIN_DEPHOSPHORYLATION		15	-0.4160328	-1.0863403	0.38297874	0.57499	1	294	tags=33%, list=17%, signal=40%
GOBP_GAMETE_GENERATION		25	-0.3703522	-1.0839661	0.35789475	0.5806283	1	518	tags=36%, list=31%, signal=51%
GOBP_MODULATION_BY_HOST_OF_SYMBIONT_PROCESS		12	-0.4481977	-1.0838993	0.3846154	0.5803367	1	878	tags=75%, list=52%, signal=154%
GOBP_IRON_ION_TRANSPORT		20	-0.3813894	-1.0833286	0.375	0.58129364	1	875	tags=65%, list=52%, signal=133%
GOBP_ESTABLISHMENT_OF_PROTEIN_LOCALIZATION_TO_MEMBRANE		141	-0.3173627	-1.0829874	0.32	0.58158547	1	687	tags=47%, list=41%, signal=72%
GOBP_INTRINSIC_APOPTOTIC_SIGNALING_PATHWAY		47	-0.3385142	-1.0829766	0.36734694	0.58109355	1	714	tags=51%, list=42%, signal=86%
GOBP_REGULATION_OF_VESICLE_MEDIATED_TRANSPORT		98	-0.3093721	-1.0829185	0.3	0.5806896	1	399	tags=32%, list=24%, signal=39%
GOBP_RESPONSE_TO_OXYGEN_CONTAINING_COMPOUND		198	-0.3069064	-1.0827776	0.24	0.5805731	1	844	tags=57%, list=50%, signal=100%
KEGG_UBIQUITIN_MEDIATED_PROTEOLYSIS		11	-0.4258504	-1.0820484	0.34117648	0.5818459	1	718	tags=73%, list=42%, signal=125%
GOBP_CELL_MATURATION		10	-0.4380635	-1.0817543	0.3604651	0.58212763	1	518	tags=40%, list=31%, signal=57%
REACTOME_GOLGI_ASSOCIATED_VESICLE_BIOGENESIS		18	-0.3840922	-1.0816367	0.38709676	0.5819838	1	368	tags=44%, list=22%, signal=56%
GOBP_NEURON_APOPTOTIC_PROCESS		25	-0.3748967	-1.0815088	0.39795917	0.5817799	1	167	tags=20%, list=10%, signal=22%
GOBP_REGULATION_OF_VACUOLE_ORGANIZATION		12	-0.4305743	-1.0815055	0.3846154	0.5812822	1	552	tags=50%, list=33%, signal=74%
GOBP_NEGATIVE_REGULATION_OF_LEUKOCYTE_CELL_CELL_ADHESION		11	-0.4431385	-1.0806253	0.3655914	0.5830601	1	767	tags=73%, list=45%, signal=132%
GOBP_CELL_CYCLE_G1_S_PHASE_TRANSITION		17	-0.3869709	-1.0805238	0.35955057	0.5827694	1	714	tags=59%, list=42%, signal=101%
GOBP_REGULATION_OF_PRODUCTION_OF_MOLECULAR_MEDIATOR_OF_IMMU		14	-0.3988653	-1.0793482	0.43010753	0.58525735	1	725	tags=57%, list=43%, signal=99%
GOBP_UNSATURATED_FATTY_ACID_METABOLIC_PROCESS		19	-0.3846704	-1.0791678	0.39784947	0.5851464	1	727	tags=58%, list=43%, signal=100%
GOBP_ESTABLISHMENT_OF_ENDOTHELIAL_BARRIER		12	-0.4280019	-1.0784013	0.3846154	0.5868176	1	653	tags=58%, list=39%, signal=94%
REACTOME_PLASMA_LIPOPROTEIN_CLEARANCE		13	-0.4281501	-1.0782145	0.37931034	0.58676577	1	206	tags=31%, list=12%, signal=35%
GOBP_HEART_MORPHOGENESIS		17	-0.3856359	-1.0781325	0.36666667	0.58649075	1	168	tags=18%, list=10%, signal=19%
REACTOME_GENE_AND_PROTEIN_EXPRESSION_BY_JAK_STAT_SIGNALING_A		18	-0.4075804	-1.0779914	0.40860215	0.58642197	1	567	tags=50%, list=33%, signal=74%
GOBP_REGULATION_OF_IMMUNE_EFFECTOR_PROCESS		48	-0.3354191	-1.0774761	0.4	0.5870565	1	391	tags=35%, list=23%, signal=45%
REACTOME_SELECTIVE_AUTOPHAGY		27	-0.3633934	-1.0770754	0.39795917	0.5876483	1	576	tags=48%, list=34%, signal=72%
GOBP_POSITIVE_REGULATION_OF_TRANSPORT		145	-0.3062599	-1.0767691	0.31	0.5879389	1	659	tags=45%, list=39%, signal=67%
GOBP_ACTIN_MEDIATED_CELL_CONTRACTION		41	-0.3396546	-1.0736091	0.33333334	0.5956765	1	287	tags=20%, list=17%, signal=23%
GOBP_RESPONSE_TO_HORMONE		110	-0.3100742	-1.0735902	0.37	0.5951636	1	683	tags=45%, list=40%, signal=70%
GOBP_NEUROGENESIS		135	-0.2981327	-1.0720835	0.32	0.5986692	1	560	tags=35%, list=33%, signal=48%
REACTOME_RAF_ACTIVATION		10	-0.4761285	-1.0713832	0.32941177	0.60021347	1	199	tags=30%, list=12%, signal=34%
REACTOME_PLASMA_LIPOPROTEIN_ASSEMBLY_REMODELING_AND_CLEARAN		21	-0.376914	-1.0701913	0.39784947	0.6029752	1	974	tags=81%, list=57%, signal=188%
GOBP_PROTEIN_LOCALIZATION_TO_PLASMA_MEMBRANE		60	-0.3095241	-1.0698849	0.36734694	0.60313106	1	659	tags=45%, list=39%, signal=71%

GOBP_IN_UTERO_EMBRYONIC_DEVELOPMENT		18	-0.3832602	-1.0697725	0.39130434	0.60296285	1	587	tags=50%, list=35%, signal=76%
GOBP_CELLULAR_AMIDE_METABOLIC_PROCESS		311	-0.2943755	-1.0687156	0.29	0.60516936	1	866	tags=55%, list=51%, signal=92%
GOBP_GLIAL_CELL_DIFFERENTIATION		21	-0.3497911	-1.0677179	0.41666666	0.6072614	1	535	tags=38%, list=32%, signal=55%
GOBP_MODULATION_OF_PROCESS_OF_OTHER_ORGANISM		17	-0.3859421	-1.0665028	0.37777779	0.60996306	1	878	tags=71%, list=52%, signal=145%
GOBP_ICOSANOID_METABOLIC_PROCESS		18	-0.3880987	-1.0663712	0.42708334	0.60974723	1	746	tags=61%, list=44%, signal=108%
GOBP_POSITIVE_REGULATION_OF_TRANSFERASE_ACTIVITY		63	-0.3260267	-1.0657396	0.39	0.610719	1	629	tags=43%, list=37%, signal=66%
GOBP_ESTABLISHMENT_OF_PROTEIN_LOCALIZATION_TO_ORGANELLE		194	-0.2997939	-1.065594	0.28	0.6105452	1	601	tags=40%, list=35%, signal=55%
GOBP_REGULATION_OF_PROTEIN_STABILITY		61	-0.3248193	-1.0644636	0.42	0.61333454	1	733	tags=54%, list=43%, signal=92%
REACTOME_ESR_MEDIATED_SIGNALING		22	-0.3745648	-1.0643773	0.39361703	0.6130656	1	704	tags=55%, list=42%, signal=92%
GOBP_NEGATIVE_REGULATION_OF_BIOSYNTHETIC_PROCESS		96	-0.3019878	-1.0642568	0.36	0.61281395	1	724	tags=49%, list=43%, signal=81%
GOBP_REGULATION_OF_POTASSIUM_ION_TRANSPORT		19	-0.3980024	-1.0641023	0.39784947	0.61268973	1	659	tags=53%, list=39%, signal=85%
GOBP_PROTEIN_STABILIZATION		50	-0.3345584	-1.0639739	0.33333334	0.61249787	1	733	tags=54%, list=43%, signal=92%
GOBP_CELLULAR_COMPONENT_ASSEMBLY_INVOLVED_IN_MORPHOGENESIS		25	-0.3660114	-1.0637674	0.41836736	0.61255133	1	189	tags=20%, list=11%, signal=22%
GOBP_NEGATIVE_REGULATION_OF_MAP_KINASE_ACTIVITY		10	-0.4342737	-1.0635335	0.42222223	0.61262053	1	820	tags=80%, list=48%, signal=154%
GOBP_HEAD_DEVELOPMENT		67	-0.3113079	-1.0632957	0.35	0.61267364	1	547	tags=36%, list=32%, signal=51%
REACTOME_SIGNALING_BY_NTRKS		18	-0.3778744	-1.0628757	0.4226804	0.61337316	1	800	tags=67%, list=47%, signal=125%
GOBP_REGULATION_OF_CELL_JUNCTION_ASSEMBLY		26	-0.3615197	-1.0622412	0.40206185	0.6145498	1	653	tags=50%, list=39%, signal=80%
GOBP_REGULATION_OF_PROTEIN_LOCALIZATION_TO_PLASMA_MEMBRANE		23	-0.3714839	-1.0611148	0.39795917	0.6170581	1	629	tags=57%, list=37%, signal=89%
GOBP_RESPONSE_TO_MONOSACCHARIDE		29	-0.357338	-1.0593063	0.37894738	0.6212138	1	656	tags=52%, list=39%, signal=83%
GOBP_POSITIVE_REGULATION_OF_CELL_MORPHOGENESIS_INVOLVED_IN_D		21	-0.3647059	-1.0592806	0.43157893	0.6207479	1	852	tags=71%, list=50%, signal=142%
GOBP_RESPONSE_TO_CATECHOLAMINE		11	-0.4082463	-1.0574421	0.48913044	0.6249362	1	775	tags=64%, list=46%, signal=116%
GOBP_ORGANIC_HYDROXY_COMPOUND_TRANSPORT		29	-0.3494078	-1.0572277	0.40206185	0.6251112	1	213	tags=24%, list=13%, signal=27%
GOBP_ACTIVATION_OF_PROTEIN_KINASE_ACTIVITY		32	-0.3349336	-1.0559386	0.4	0.62792414	1	839	tags=59%, list=49%, signal=115%
GOBP_RESPONSE_TO_METAL_ION		63	-0.330546	-1.0559272	0.4	0.6274042	1	682	tags=48%, list=40%, signal=77%
REACTOME_COLLAGEN_FORMATION		13	-0.4107488	-1.0558653	0.37634408	0.6270101	1	778	tags=77%, list=46%, signal=141%
GOBP_NEGATIVE_REGULATION_OF_INTRACELLULAR_SIGNAL_TRANSDUCTIOI		56	-0.3234448	-1.0555762	0.4	0.6272576	1	681	tags=48%, list=40%, signal=78%
GOBP_REGULATION_OF_CELL_ACTIVATION		49	-0.3258473	-1.0555462	0.35353535	0.6267894	1	864	tags=65%, list=51%, signal=129%
GOBP_PHOSPHATIDYLCHOLINE_BIOSYNTHETIC_PROCESS		10	-0.4189105	-1.0555078	0.4408602	0.626339	1	179	tags=30%, list=11%, signal=33%
GOBP_LOW_DENSITY_LIPOPROTEIN_RECEPTOR_PARTICLE_METABOLIC_PRO		10	-0.4284867	-1.0544281	0.41379312	0.6287382	1	974	tags=100%, list=57%, signal=234%
REACTOME_RHOQ_GTPASE_CYCLE		13	-0.4251551	-1.0537702	0.43373495	0.6298391	1	921	tags=85%, list=54%, signal=184%
GOBP_NEGATIVE_REGULATION_OF_BINDING		16	-0.4031485	-1.0533748	0.44680852	0.630324	1	670	tags=63%, list=40%, signal=102%
GOBP_DE_NOVO_PROTEIN_FOLDING		13	-0.4293264	-1.0525924	0.41111112	0.63182837	1	577	tags=54%, list=34%, signal=81%
GOBP_CELLULAR_PROTEIN_CONTAINING_COMPLEX_ASSEMBLY		209	-0.2917871	-1.0503305	0.32	0.6370061	1	643	tags=42%, list=38%, signal=59%
GOBP_RESPONSE_TO_OSMOTIC_STRESS		12	-0.4101916	-1.0499337	0.4065934	0.6373347	1	463	tags=42%, list=27%, signal=57%
GOBP_ERAD_PATHWAY		24	-0.3475649	-1.0495894	0.40625	0.6376629	1	811	tags=63%, list=48%, signal=118%
GOBP_EXOCYTIC_PROCESS		19	-0.3798235	-1.0493386	0.43617022	0.6378507	1	425	tags=37%, list=25%, signal=49%
GOBP_LEUKOCYTE_PROLIFERATION		29	-0.3716642	-1.0484723	0.40816328	0.6394985	1	656	tags=52%, list=39%, signal=83%
GOBP_APOPTOTIC_PROCESS		229	-0.2930582	-1.0479313	0.32	0.6402204	1	699	tags=47%, list=41%, signal=69%
GOBP_POSITIVE_REGULATION_OF_ANION_TRANSPORT		73	-0.3149559	-1.0476835	0.3939394	0.64040446	1	659	tags=47%, list=39%, signal=73%
GOBP_GLYCEROLIPID_CATABOLIC_PROCESS		18	-0.369632	-1.0471163	0.42105263	0.6412726	1	391	tags=33%, list=23%, signal=43%
GOBP_POSITIVE_REGULATION_OF_IMMUNE_EFFECTOR_PROCESS		25	-0.3456731	-1.0462914	0.43010753	0.64292884	1	725	tags=56%, list=43%, signal=96%
GOBP_RIBONUCLEOSIDE_MONOPHOSPHATE_METABOLIC_PROCESS		11	-0.4038184	-1.0461786	0.3647059	0.6425929	1	1008	tags=91%, list=59%, signal=223%
GOBP_RESPONSE_TO_DRUG		48	-0.3184592	-1.046149	0.42	0.6421424	1	301	tags=23%, list=18%, signal=27%
GOBP_REGULATION_OF_TRANSMEMBRANE_RECEPTOR_PROTEIN_SERINE_TH		16	-0.3824943	-1.0458001	0.375	0.6425801	1	628	tags=50%, list=37%, signal=79%
GOBP_NEGATIVE_REGULATION_OF_NERVOUS_SYSTEM_DEVELOPMENT		13	-0.4202172	-1.0456142	0.4408602	0.64251596	1	309	tags=31%, list=18%, signal=37%
REACTOME_INTRA_GOLGI_AND_RETROGRADE_GOLGI_TO_ER_TRAFFIC		38	-0.3296524	-1.0447977	0.45454547	0.64414704	1	276	tags=24%, list=16%, signal=28%

GOBP_CARBOHYDRATE_TRANSPORT		12	-0.4284217	-1.0441831	0.40860215	0.64541525	1	350	tags=33%, list=21%, signal=42%
GOBP_PROTEIN_TARGETING		168	-0.2967159	-1.0441805	0.37	0.6448911	1	729	tags=47%, list=43%, signal=74%
GOBP_NEGATIVE_REGULATION_OF_IMMUNE_SYSTEM_PROCESS		36	-0.3346594	-1.0440933	0.47474748	0.64447373	1	391	tags=36%, list=23%, signal=46%
GOBP_DNA_PACKAGING		10	-0.4302546	-1.0439935	0.41758242	0.6440818	1	734	tags=60%, list=43%, signal=105%
GOBP_NEGATIVE_REGULATION_OF_ERK1_AND_ERK2_CASCADE		10	-0.4148313	-1.0427529	0.36363637	0.64703155	1	944	tags=80%, list=56%, signal=179%
GOBP_APICAL_JUNCTION_ASSEMBLY		12	-0.4238001	-1.0416393	0.41666666	0.6496438	1	653	tags=58%, list=39%, signal=94%
GOBP_RESPONSE_TO_REACTIVE_OXYGEN_SPECIES		40	-0.3287035	-1.0414102	0.41836736	0.6496551	1	336	tags=30%, list=20%, signal=37%
GOBP_CELLULAR_RESPONSE_TO_PEPTIDE_HORMONE_STIMULUS		46	-0.3177337	-1.0407304	0.43434343	0.65080655	1	669	tags=43%, list=39%, signal=70%
GOBP_REGULATION_OF_CELL_DIVISION		12	-0.3973722	-1.040669	0.41860464	0.65041935	1	882	tags=75%, list=52%, signal=155%
GOBP_NEGATIVE_REGULATION_OF_DEVELOPMENTAL_PROCESS		71	-0.316073	-1.0392054	0.41	0.6534671	1	518	tags=37%, list=31%, signal=51%
GOBP_CELLULAR_MODIFIED_AMINO_ACID_METABOLIC_PROCESS		37	-0.3259322	-1.0390017	0.45360824	0.6536051	1	389	tags=30%, list=23%, signal=38%
GOBP_SYNAPSE_ORGANIZATION		42	-0.3356835	-1.0388759	0.4489796	0.65332973	1	547	tags=40%, list=32%, signal=58%
GOBP_NEURON_DIFFERENTIATION		115	-0.3026379	-1.0387621	0.34	0.6530469	1	558	tags=35%, list=33%, signal=48%
GOBP_NEUROMUSCULAR_PROCESS		10	-0.4230267	-1.0380213	0.48314607	0.65434283	1	186	tags=30%, list=11%, signal=33%
KEGG_PPAR_SIGNALING_PATHWAY		21	-0.3642773	-1.0371869	0.45360824	0.65611446	1	431	tags=29%, list=25%, signal=38%
GOBP_UBIQUITIN_DEPENDENT_ERAD_PATHWAY		22	-0.3657816	-1.0364672	0.44329897	0.6573649	1	811	tags=64%, list=48%, signal=120%
GOBP_REGULATION_OF_INFLAMMATORY_RESPONSE		38	-0.3367537	-1.0363116	0.4226804	0.65719265	1	274	tags=29%, list=16%, signal=34%
REACTOME_INTERLEUKIN_12_SIGNALING		20	-0.3672245	-1.034507	0.48979592	0.661036	1	567	tags=50%, list=33%, signal=74%
GOBP_MITOTIC_NUCLEAR_DIVISION		19	-0.3845346	-1.0344537	0.44329897	0.66057855	1	518	tags=42%, list=31%, signal=60%
KEGG_GLYCEROLIPID_METABOLISM		12	-0.3973917	-1.0331632	0.37078652	0.6633438	1	289	tags=25%, list=17%, signal=30%
GOBP_INTRACELLULAR_TRANSPORT		369	-0.2851507	-1.033123	0.37	0.6629011	1	735	tags=46%, list=43%, signal=64%
GOBP_POSITIVE_REGULATION_OF_SMALL_MOLECULE_METABOLIC_PROCES		17	-0.3741322	-1.0325267	0.41836736	0.66387254	1	699	tags=53%, list=41%, signal=89%
GOBP_POSITIVE_REGULATION_OF_PROTEIN_LOCALIZATION_TO_MEMBRANE		32	-0.3399683	-1.0321884	0.43010753	0.6640485	1	687	tags=53%, list=41%, signal=88%
GOBP_TUBE_FORMATION		10	-0.4069091	-1.0312599	0.52272725	0.66590214	1	673	tags=40%, list=40%, signal=66%
GOBP_POSITIVE_REGULATION_OF_PROTEIN_KINASE_ACTIVITY		52	-0.3207268	-1.0312284	0.44444445	0.66538674	1	576	tags=38%, list=34%, signal=56%
REACTOME_AUTOPHAGY		34	-0.3281101	-1.0310663	0.43	0.6652086	1	759	tags=59%, list=45%, signal=104%
REACTOME_PHASE_II_CONJUGATION_OF_COMPOUNDS		12	-0.409881	-1.0300837	0.46666667	0.6670152	1	791	tags=75%, list=47%, signal=140%
REACTOME_BIOLOGICAL_OXIDATIONS		26	-0.3478293	-1.0299137	0.45454547	0.66701996	1	791	tags=69%, list=47%, signal=128%
REACTOME_MTOR_SIGNALLING		11	-0.416665	-1.0291495	0.47126436	0.66823167	1	839	tags=73%, list=49%, signal=143%
GOBP_PROTEIN_COMPLEX_OLIGOMERIZATION		35	-0.3358258	-1.0285283	0.45918366	0.6692336	1	388	tags=26%, list=23%, signal=33%
REACTOME_METABOLISM_OF_WATER_SOLUBLE_VITAMINS_AND_COFACTORS		25	-0.3354808	-1.0284617	0.43434343	0.6688461	1	153	tags=16%, list=9%, signal=17%
GOBP_PHOSPHOLIPID_TRANSPORT		15	-0.3938752	-1.0280495	0.5	0.66941494	1	940	tags=73%, list=55%, signal=163%
GOBP_CENTRAL_NERVOUS_SYSTEM_DEVELOPMENT		86	-0.3086852	-1.0278823	0.43	0.6694345	1	547	tags=36%, list=32%, signal=51%
GOBP_POSITIVE_REGULATION_OF_NEURON_PROJECTION_DEVELOPMENT		21	-0.350378	-1.027647	0.43617022	0.66950846	1	1002	tags=67%, list=59%, signal=161%
GOBP_POSITIVE_REGULATION_OF_HYDROLASE_ACTIVITY		82	-0.3033074	-1.0271941	0.41	0.67030674	1	447	tags=29%, list=26%, signal=38%
GOBP_NEGATIVE_REGULATION_OF_NEURON_APOPTOTIC_PROCESS		13	-0.3946608	-1.0271364	0.44705883	0.6698876	1	167	tags=23%, list=10%, signal=25%
GOBP_MEMBRANE_FUSION		43	-0.3227141	-1.0269774	0.49	0.66974676	1	469	tags=33%, list=28%, signal=44%
GOBP_PRIMARY_ALCOHOL_METABOLIC_PROCESS		16	-0.3822607	-1.0258582	0.47252747	0.6723524	1	380	tags=38%, list=22%, signal=48%
GOBP_SENSORY_PERCEPTION_OF_MECHANICAL_STIMULUS		19	-0.364648	-1.0254605	0.49473685	0.672891	1	487	tags=37%, list=29%, signal=51%
GOBP_POSITIVE_REGULATION_OF_PROTEOLYSIS		55	-0.3136746	-1.0244402	0.45	0.6751179	1	342	tags=25%, list=20%, signal=31%
REACTOME_N_GLYCAN_TRIMMING_IN_THE_ER_AND_CALNEXIN_CALRETICULI		12	-0.3912678	-1.0244018	0.4	0.67464155	1	440	tags=42%, list=26%, signal=56%
GOBP_GLUCOSE_METABOLIC_PROCESS		42	-0.3201404	-1.0242752	0.48	0.6743953	1	496	tags=38%, list=29%, signal=53%
GOBP_CELLULAR_COMPONENT_DISASSEMBLY		143	-0.2920329	-1.0242321	0.41	0.6739759	1	786	tags=51%, list=46%, signal=87%
GOBP_REGULATION_OF_MUSCLE_ADAPTATION		20	-0.361885	-1.0238432	0.5154639	0.6744796	1	309	tags=25%, list=18%, signal=30%
REACTOME_DISEASES_OF_METABOLISM		29	-0.3511225	-1.0235996	0.5050505	0.67461306	1	260	tags=24%, list=15%, signal=28%
GOBP_DEPHOSPHORYLATION		46	-0.319739	-1.0234135	0.49484536	0.6746351	1	813	tags=54%, list=48%, signal=102%

GOBP_TRANSITION_METAL_ION_TRANSPORT		23	-0.3619948	-1.0230647	0.5	0.67496425	1	809	tags=57%, list=48%, signal=107%
GOBP_SMALL_MOLECULE_CATABOLIC_PROCESS		119	-0.2928201	-1.0228056	0.54	0.67503333	1	910	tags=54%, list=54%, signal=108%
GOBP_REGULATION_OF_MEMBRANE_DEPOLARIZATION		10	-0.4175103	-1.0225828	0.41860464	0.6749924	1	659	tags=50%, list=39%, signal=81%
REACTOME_NEUROTRANSMITTER_RECEPTORS_AND_POSTSYNAPTIC_SIGNAL		31	-0.3323663	-1.0222828	0.45454547	0.6751946	1	759	tags=52%, list=45%, signal=92%
GOBP_CELLULAR_RESPONSE_TO_REACTIVE_OXYGEN_SPECIES		25	-0.3433969	-1.0206829	0.5102041	0.6787967	1	336	tags=32%, list=20%, signal=39%
REACTOME_GLUCONEOGENESIS		20	-0.356319	-1.0205348	0.4631579	0.67862713	1	552	tags=45%, list=33%, signal=66%
GOBP_PLASMA_MEMBRANE_ORGANIZATION		28	-0.3431476	-1.0204749	0.48421052	0.6781835	1	867	tags=71%, list=51%, signal=144%
REACTOME_RHO_GTPASES_ACTIVATE_IQGAPS		10	-0.4250633	-1.0202881	0.47777778	0.6781947	1	851	tags=80%, list=50%, signal=160%
GOBP_DNA_RECOMBINATION		12	-0.4040895	-1.0199832	0.45555556	0.67837906	1	853	tags=75%, list=50%, signal=150%
GOBP_REGULATION_OF_EPITHELIAL_CELL_MIGRATION		31	-0.3421658	-1.0196614	0.4387755	0.6785855	1	294	tags=26%, list=17%, signal=31%
GOBP_MEMBRANE_REPOLARIZATION		16	-0.3898307	-1.019288	0.47826087	0.67899495	1	287	tags=25%, list=17%, signal=30%
GOBP_MODULATION_BY_HOST_OF_VIRAL_PROCESS		10	-0.4313283	-1.0187678	0.4512195	0.67984056	1	878	tags=70%, list=52%, signal=144%
KEGG_AXON_GUIDANCE		17	-0.368375	-1.0184648	0.4408602	0.68006086	1	607	tags=47%, list=36%, signal=73%
GOBP_POSITIVE_REGULATION_OF_ERK1_AND_ERK2_CASCADE		22	-0.3558832	-1.0181015	0.41836736	0.68028045	1	393	tags=32%, list=23%, signal=41%
GOBP_CELLULAR_AMINO_ACID_BIOSYNTHETIC_PROCESS		14	-0.3755674	-1.0173168	0.49438202	0.68170005	1	646	tags=43%, list=38%, signal=69%
GOBP_RIBOSOME_ASSEMBLY		26	-0.3485788	-1.0169213	0.47916666	0.6821047	1	737	tags=58%, list=43%, signal=101%
GOBP_NEGATIVE_REGULATION_OF_LYMPHOCYTE_ACTIVATION		11	-0.4141434	-1.0168701	0.45054945	0.68171614	1	906	tags=82%, list=53%, signal=175%
GOBP_PRODUCTION_OF_MOLECULAR_MEDIATOR_OF_IMMUNE_RESPONSE		18	-0.3504293	-1.0161039	0.42105263	0.6830226	1	725	tags=50%, list=43%, signal=86%
GOBP_REGULATION_OF_MYELOID_CELL_DIFFERENTIATION		15	-0.3780084	-1.0159642	0.50526315	0.68281263	1	267	tags=27%, list=16%, signal=31%
GOBP_CELLULAR_GLUCOSE_HOMEOSTASIS		21	-0.3632321	-1.0153548	0.48387095	0.68396837	1	631	tags=52%, list=37%, signal=82%
GOBP_REGULATION_OF_LYMPHOCYTE_ACTIVATION		35	-0.3350756	-1.0153108	0.4489796	0.6835255	1	853	tags=66%, list=50%, signal=130%
GOBP_DNA_REPAIR		21	-0.3628053	-1.0152997	0.5416667	0.68299073	1	853	tags=76%, list=50%, signal=151%
GOBP_MUSCLE_SYSTEM_PROCESS		112	-0.2907885	-1.0150238	0.54	0.6833227	1	688	tags=40%, list=41%, signal=63%
GOBP_SENSORY_PERCEPTION		34	-0.3397652	-1.0148531	0.45918366	0.68315214	1	487	tags=35%, list=29%, signal=49%
GOBP_REGULATION_OF_PROTEIN_BINDING		31	-0.3416722	-1.0147506	0.5	0.6828353	1	670	tags=55%, list=40%, signal=89%
BIOCARTA_PROTEASOME_PATHWAY		11	-0.4177845	-1.0146173	0.4827586	0.68263435	1	819	tags=73%, list=48%, signal=140%
KEGG_VIBRIO_CHOLERAE_INFECTION		15	-0.3816085	-1.0132515	0.4945055	0.68533283	1	329	tags=27%, list=19%, signal=33%
GOBP_NEUROTRANSMITTER_SECRETION		18	-0.3482907	-1.0123484	0.4574468	0.6869247	1	640	tags=44%, list=38%, signal=71%
GOBP_MYELOID_LEUKOCYTE_MIGRATION		25	-0.3428692	-1.0123247	0.45833334	0.6864364	1	606	tags=52%, list=36%, signal=80%
GOBP_REGULATION_OF_ANION_TRANSPORT		125	-0.2955722	-1.0118581	0.47	0.6869408	1	459	tags=34%, list=27%, signal=43%
GOBP_MUSCLE_ADAPTATION		22	-0.3496216	-1.0117147	0.48453608	0.68672216	1	309	tags=23%, list=18%, signal=27%
GOBP_GOLGI_VESICLE_TRANSPORT		82	-0.3018633	-1.0115905	0.53	0.68643475	1	921	tags=67%, list=54%, signal=140%
GOBP_REGULATION_OF_POTASSIUM_ION_TRANSMEMBRANE_TRANSPORTER		16	-0.3853352	-1.0101328	0.5	0.68940175	1	748	tags=56%, list=44%, signal=100%
GOBP_CHROMOSOME_SEGREGATION		21	-0.3548256	-1.0095569	0.45360824	0.69031554	1	518	tags=38%, list=31%, signal=54%
KEGG_PATHWAYS_IN_CANCER		26	-0.3382118	-1.0094157	0.45833334	0.69029397	1	325	tags=31%, list=19%, signal=37%
GOBP_PLASMA_LIPOPROTEIN_PARTICLE_CLEARANCE		20	-0.3791168	-1.0091507	0.49462366	0.69031805	1	531	tags=45%, list=31%, signal=65%
GOBP_PROTEIN_DEPHOSPHORYLATION		30	-0.3263547	-1.0089135	0.45454547	0.6902965	1	687	tags=47%, list=41%, signal=77%
GOBP_CARDIAC_MUSCLE_CELL_CONTRACTION		15	-0.3763604	-1.0082674	0.44680852	0.69117683	1	629	tags=47%, list=37%, signal=74%
GOBP_MACROAUTOPHAGY		61	-0.313206	-1.0070957	0.53535354	0.6936822	1	578	tags=41%, list=34%, signal=60%
GOBP_PROTEIN_LOCALIZATION_TO_CELL_JUNCTION		16	-0.3772313	-1.0063581	0.4787234	0.69503146	1	653	tags=50%, list=39%, signal=81%
GOBP_PURINE_NUCLEOSIDE_METABOLIC_PROCESS		15	-0.3706897	-1.0060427	0.5319149	0.6953037	1	629	tags=47%, list=37%, signal=74%
GOBP_DEFENSE_RESPONSE_TO_BACTERIUM		23	-0.3494501	-1.0045614	0.5319149	0.69847673	1	728	tags=61%, list=43%, signal=105%
GOBP_NEGATIVE_REGULATION_OF_IMMUNE_RESPONSE		16	-0.3722062	-1.0039319	0.48314607	0.69951487	1	252	tags=31%, list=15%, signal=36%
GOBP_NEGATIVE_REGULATION_OF_INFLAMMATORY_RESPONSE		17	-0.3634425	-1.0037707	0.51724136	0.6994105	1	205	tags=24%, list=12%, signal=26%
GOBP_PROTEIN_CONTAINING_COMPLEX_SUBUNIT_ORGANIZATION		383	-0.2767434	-1.0036119	0.46	0.6992302	1	643	tags=40%, list=38%, signal=49%
REACTOME_PEPTIDE_HORMONE_METABOLISM		10	-0.4004697	-1.0032666	0.46666667	0.69949746	1	606	tags=50%, list=36%, signal=77%

GOBP_PROTEIN_LOCALIZATION_TO_CELL_PERIPHERY		66	-0.3106645	-1.0027583	0.46	0.7001443	1	659	tags=45%, list=39%, signal=71%
GOBP_POSITIVE_REGULATION_OF_CELLULAR_AMIDE_METABOLIC_PROCESS		30	-0.3399431	-1.0024129	0.43157893	0.7003962	1	714	tags=60%, list=42%, signal=102%
GOBP_POSITIVE_REGULATION_OF_LEUKOCYTE_PROLIFERATION		14	-0.3646857	-1.0016435	0.46067417	0.7016548	1	252	tags=29%, list=15%, signal=33%
GOBP_ORGANELLE_FISSION		37	-0.3229499	-0.9994968	0.5416667	0.70636225	1	930	tags=65%, list=55%, signal=141%
GOBP_REGENERATION		29	-0.3387387	-0.9994515	0.4848485	0.70592743	1	344	tags=28%, list=20%, signal=34%
GOBP_ESTABLISHMENT_OR_MAINTENANCE_OF_BIPOLAR_CELL_POLARITY		10	-0.4201012	-0.9994273	0.52873564	0.7054178	1	629	tags=50%, list=37%, signal=79%
GOBP_REGULATION_OF_TUBE_SIZE		19	-0.3485466	-0.9992599	0.5	0.70526373	1	683	tags=58%, list=40%, signal=96%
GOBP_SMOOTH_MUSCLE_CELL_PROLIFERATION		13	-0.3897758	-0.9988036	0.4597701	0.7059641	1	241	tags=31%, list=14%, signal=36%
GOBP_REGULATION_OF_OXIDOREDUCTASE_ACTIVITY		24	-0.3345808	-0.998665	0.5	0.7057647	1	869	tags=71%, list=51%, signal=143%
GOBP_POSITIVE_REGULATION_OF_TRANSLATION		25	-0.3423809	-0.9966696	0.47368422	0.71043205	1	714	tags=60%, list=42%, signal=102%
GOBP_NEGATIVE_REGULATION_OF_CELLULAR_RESPONSE_TO_GROWTH_FA		10	-0.3994607	-0.9965403	0.4875	0.71020657	1	255	tags=30%, list=15%, signal=35%
GOBP_ORGANONITROGEN_COMPOUND_BIOSYNTHETIC_PROCESS		381	-0.2765445	-0.9964107	0.47	0.7098993	1	822	tags=51%, list=48%, signal=77%
REACTOME_RAC3_GTPASE_CYCLE		16	-0.3643035	-0.9950803	0.48387095	0.7125311	1	921	tags=75%, list=54%, signal=163%
GOBP_MUSCLE_HYPERTROPHY		14	-0.3833792	-0.9940019	0.47191012	0.7147538	1	544	tags=43%, list=32%, signal=63%
GOBP_POSITIVE_REGULATION_OF_PEPTIDE_SECRETION		24	-0.3412519	-0.9928896	0.5151515	0.717124	1	393	tags=29%, list=23%, signal=37%
GOBP_REGULATION_OF_MAPK_CASCADE		67	-0.2930671	-0.9916698	0.54545456	0.71939945	1	885	tags=61%, list=52%, signal=123%
GOBP_NEGATIVE_REGULATION_OF_MAPK_CASCADE		22	-0.3390014	-0.9914724	0.48387095	0.71940064	1	1030	tags=82%, list=61%, signal=206%
GOBP_PROTEIN_CONTAINING_COMPLEX_LOCALIZATION		25	-0.3392908	-0.9894423	0.5208333	0.72373766	1	775	tags=60%, list=46%, signal=109%
KEGG_OOCYTE_MEIOSIS		20	-0.3639346	-0.9893137	0.5106383	0.72345144	1	513	tags=40%, list=30%, signal=57%
GOBP_POSITIVE_REGULATION_OF_T_CELL_PROLIFERATION		14	-0.3646857	-0.9879665	0.516129	0.7261553	1	252	tags=29%, list=15%, signal=33%
GOBP_MUSCLE_CONTRACTION		99	-0.291727	-0.987812	0.55	0.72597945	1	373	tags=24%, list=22%, signal=29%
GOBP_GLUTAMINE_FAMILY_AMINO_ACID_CATABOLIC_PROCESS		11	-0.3898628	-0.9876077	0.50537634	0.7259011	1	703	tags=55%, list=41%, signal=93%
REACTOME_RHOJ_GTPASE_CYCLE		11	-0.3966931	-0.9874714	0.5060241	0.725755	1	921	tags=73%, list=54%, signal=158%
GOBP_CARBOHYDRATE_HOMEOSTASIS		33	-0.3418541	-0.9872942	0.54	0.7255576	1	669	tags=55%, list=39%, signal=88%
REACTOME_MATURATION_OF_SARS_COV_2_SPIKE_PROTEIN		10	-0.4178377	-0.986669	0.5308642	0.7265371	1	587	tags=40%, list=35%, signal=61%
GOBP_POSITIVE_REGULATION_OF_CATALYTIC_ACTIVITY		144	-0.2808225	-0.9864671	0.54	0.7263539	1	869	tags=56%, list=51%, signal=104%
GOBP_RESPONSE_TO_ORGANOPHOSPHORUS		17	-0.3741201	-0.9861695	0.47777778	0.72651374	1	225	tags=29%, list=13%, signal=34%
GOBP_REGULATION_OF_SYNAPSE_STRUCTURE_OR_ACTIVITY		21	-0.3724589	-0.9850281	0.5851064	0.7287396	1	851	tags=67%, list=50%, signal=132%
REACTOME_GOLGI_TO_ER_RETROGRADE_TRANSPORT		30	-0.321094	-0.9848058	0.47959185	0.72872555	1	892	tags=60%, list=53%, signal=124%
REACTOME_RHOH_GTPASE_CYCLE		13	-0.3981057	-0.9847987	0.5168539	0.72816324	1	747	tags=62%, list=44%, signal=109%
GOBP_REGULATION_OF_GENE_EXPRESSION_EPIGENETIC		10	-0.4036548	-0.9846621	0.4431818	0.72803175	1	704	tags=50%, list=42%, signal=85%
GOBP_ALPHA_AMINO_ACID_BIOSYNTHETIC_PROCESS		12	-0.3939178	-0.9842755	0.52380955	0.72850096	1	217	tags=25%, list=13%, signal=28%
GOBP_NEUTROPHIL_CHEMOTAXIS		14	-0.378192	-0.9841308	0.51086956	0.7282656	1	170	tags=29%, list=10%, signal=31%
GOBP_AMINO_ACID_ACTIVATION		19	-0.3621523	-0.9834514	0.5102041	0.7293767	1	590	tags=47%, list=35%, signal=72%
GOBP_PROCESS_UTILIZING_AUTOPHAGIC_MECHANISM		90	-0.2961392	-0.983434	0.54	0.7288456	1	632	tags=41%, list=37%, signal=62%
GOBP_GLIOGENESIS		29	-0.3299949	-0.9826966	0.4949495	0.72997713	1	535	tags=34%, list=32%, signal=50%
GOBP_LIPID_CATABOLIC_PROCESS		81	-0.2839252	-0.9819871	0.52	0.7310542	1	992	tags=58%, list=59%, signal=133%
REACTOME_DISEASES_OF_PROGRAMMED_CELL_DEATH		14	-0.374326	-0.9809	0.5505618	0.73326576	1	313	tags=36%, list=18%, signal=43%
GOBP_ENDOMEMBRANE_SYSTEM_ORGANIZATION		98	-0.2801947	-0.9806398	0.6	0.73342574	1	659	tags=43%, list=39%, signal=66%
GOBP_PROTEIN_LOCALIZATION_TO_ORGANELLE		247	-0.2739477	-0.9805861	0.57	0.7329967	1	735	tags=46%, list=43%, signal=70%
GOBP_GOLGI_TO_PLASMA_MEMBRANE_TRANSPORT		13	-0.3818251	-0.9802191	0.47368422	0.73328894	1	392	tags=38%, list=23%, signal=50%
GOBP_POSITIVE_REGULATION_OF_PLASMA_MEMBRANE_BOUNDED_CELL_P		13	-0.3894704	-0.9791132	0.49438202	0.73520595	1	330	tags=31%, list=19%, signal=38%
GOBP_GOLGI_ORGANIZATION		23	-0.3342826	-0.9786051	0.54347825	0.73587114	1	883	tags=61%, list=52%, signal=125%
GOBP_NEGATIVE_REGULATION_OF_EPITHELIAL_CELL_PROLIFERATION		12	-0.3714058	-0.9772553	0.45348838	0.73852426	1	131	tags=17%, list=8%, signal=18%
GOBP_RESPONSE_TO_ORGANIC_CYCLIC_COMPOUND		105	-0.2926715	-0.977199	0.61	0.73810774	1	682	tags=44%, list=40%, signal=69%
GOBP_GLYCEROLIPID_BIOSYNTHETIC_PROCESS		34	-0.3157901	-0.9765571	0.5625	0.7392169	1	225	tags=21%, list=13%, signal=23%

REACTOME_G_PROTEIN_MEDIATED_EVENTS		12	-0.3956027	-0.9763697	0.5632184	0.7391001	1	64	tags=17%, list=4%, signal=17%
GOBP_TRANSLATIONAL_TERMINATION		66	-0.2940771	-0.9751042	0.58	0.7414783	1	899	tags=58%, list=53%, signal=118%
GOBP_COMPLEMENT_ACTIVATION		12	-0.3736483	-0.9745845	0.47252747	0.74225247	1	573	tags=58%, list=34%, signal=87%
GOBP_GRANULOCYTE_CHEMOTAXIS		15	-0.3867431	-0.9744175	0.54444444	0.742141	1	362	tags=40%, list=21%, signal=50%
GOBP_RESPONSE_TO_PEPTIDE		76	-0.2919943	-0.9738034	0.54	0.7427675	1	373	tags=26%, list=22%, signal=32%
GOBP_RIBOSOMAL_LARGE_SUBUNIT_ASSEMBLY		11	-0.3768637	-0.9737862	0.5180723	0.7422404	1	737	tags=64%, list=43%, signal=112%
GOBP_VESICLE_MEDIATED_TRANSPORT_TO_THE_PLASMA_MEMBRANE		18	-0.3504043	-0.9737572	0.5625	0.7417725	1	399	tags=33%, list=24%, signal=43%
GOBP_DENDRITE_MORPHOGENESIS		12	-0.3936501	-0.9720495	0.52873564	0.7455867	1	733	tags=58%, list=43%, signal=102%
GOBP_DEVELOPMENT_OF_PRIMARY_SEXUAL_CHARACTERISTICS		11	-0.3846304	-0.9713241	0.5212766	0.7465805	1	407	tags=36%, list=24%, signal=48%
GOBP_EMBRYO_DEVELOPMENT_ENDING_IN_BIRTH_OR_EGG_HATCHING		35	-0.3092982	-0.9707986	0.59183675	0.7471211	1	709	tags=46%, list=42%, signal=77%
GOBP_OSSIFICATION		40	-0.3095587	-0.9705344	0.5408163	0.7471601	1	536	tags=33%, list=32%, signal=46%
GOBP_CELLULAR_RESPONSE_TO_PEPTIDE		57	-0.2963583	-0.9696756	0.64646465	0.7486081	1	373	tags=26%, list=22%, signal=33%
GOBP_PYRUVATE_METABOLIC_PROCESS		42	-0.3029703	-0.9691591	0.53608245	0.7492044	1	847	tags=57%, list=50%, signal=111%
GOBP_POSITIVE_REGULATION_OF_RECEPTOR_MEDIATED_ENDOCYTOSIS		11	-0.4092271	-0.9683512	0.505618	0.7506977	1	748	tags=64%, list=44%, signal=113%
GOBP_TERPENOID_METABOLIC_PROCESS		17	-0.3563386	-0.9677524	0.55789477	0.7516456	1	1004	tags=82%, list=59%, signal=200%
GOBP_ANIMAL_ORGAN_REGENERATION		12	-0.3642689	-0.9670532	0.52747256	0.75248307	1	850	tags=67%, list=50%, signal=133%
REACTOME_G_ALPHA_S_SIGNALLING_EVENTS		10	-0.3998731	-0.9669514	0.48863637	0.75211215	1	620	tags=50%, list=37%, signal=78%
GOBP_POSITIVE_REGULATION_OF_ENDOTHELIAL_CELL_MIGRATION		17	-0.3606236	-0.9660342	0.6292135	0.75355613	1	725	tags=53%, list=43%, signal=92%
GOBP_POSITIVE_REGULATION_OF_MAPK_CASCADE		46	-0.2939249	-0.9655407	0.622449	0.7541525	1	406	tags=30%, list=24%, signal=39%
GOBP_PROTEIN_KINASE_B_SIGNALING		22	-0.330998	-0.9653385	0.53125	0.75404096	1	629	tags=45%, list=37%, signal=71%
GOBP_POSITIVE_REGULATION_OF_MOLECULAR_FUNCTION		195	-0.2723266	-0.9649788	0.59	0.75432634	1	578	tags=36%, list=34%, signal=49%
GOBP_GLUTATHIONE_METABOLIC_PROCESS		16	-0.3589077	-0.9643543	0.5714286	0.7551869	1	305	tags=31%, list=18%, signal=38%
GOBP_FAT_CELL_DIFFERENTIATION		12	-0.388288	-0.9641286	0.54651165	0.7551825	1	722	tags=58%, list=43%, signal=101%
GOBP_NEGATIVE_REGULATION_OF_INTRACELLULAR_TRANSPORT		13	-0.3651366	-0.9633407	0.5483871	0.75631577	1	455	tags=38%, list=27%, signal=52%
GOBP_RESPONSE_TO_TEMPERATURE_STIMULUS		36	-0.308178	-0.9632196	0.5510204	0.7561241	1	719	tags=53%, list=42%, signal=90%
GOBP_NEUTROPHIL_MIGRATION		18	-0.334689	-0.9630268	0.53125	0.7560189	1	362	tags=33%, list=21%, signal=42%
GOBP_NEGATIVE_REGULATION_OF_ION_TRANSPORT		48	-0.3211823	-0.9628083	0.5555556	0.75592786	1	391	tags=33%, list=23%, signal=42%
GOBP_REGULATION_OF_PHOSPHATASE_ACTIVITY		15	-0.3520663	-0.9623347	0.5591398	0.75635296	1	438	tags=33%, list=26%, signal=45%
GOBP_TRANSLATIONAL_ELONGATION		77	-0.2977882	-0.9613524	0.61	0.7580975	1	899	tags=57%, list=53%, signal=116%
GOBP_MULTICELLULAR_ORGANISMAL_HOMEOSTASIS		55	-0.2906959	-0.9612994	0.5102041	0.7576473	1	728	tags=45%, list=43%, signal=77%
GOBP_RHO_PROTEIN_SIGNAL_TRANSDUCTION		18	-0.3491457	-0.9612297	0.5326087	0.7572262	1	406	tags=33%, list=24%, signal=43%
GOBP_POSITIVE_REGULATION_OF_PROTEIN_LOCALIZATION_TO_CELL_PERIP		16	-0.3629024	-0.9606616	0.5591398	0.7579652	1	629	tags=56%, list=37%, signal=89%
REACTOME_CARGO_RECOGNITION_FOR_CLATHRIN_MEDIATED_ENDOCYTOSIS		18	-0.3484977	-0.9593155	0.5652174	0.7606616	1	828	tags=78%, list=49%, signal=150%
GOBP_ADENYLATE_CYCLASE_ACTIVATING_G_PROTEIN_COUPLED_RECEPTO		11	-0.40667	-0.9585071	0.54545456	0.7619106	1	406	tags=36%, list=24%, signal=48%
GOBP_NEGATIVE_REGULATION_OF_REACTIVE_OXYGEN_SPECIES_METABOL		14	-0.3513458	-0.958265	0.56666666	0.7619939	1	866	tags=79%, list=51%, signal=159%
KEGG_GLUTATHIONE_METABOLISM		14	-0.3622526	-0.9572728	0.5280899	0.7637389	1	791	tags=64%, list=47%, signal=120%
GOBP_HUMORAL_IMMUNE_RESPONSE		27	-0.3273489	-0.9569675	0.5744681	0.7640569	1	728	tags=59%, list=43%, signal=102%
GOBP_HYDROGEN_PEROXIDE_CATABOLIC_PROCESS		12	-0.3659826	-0.9567427	0.5591398	0.76402444	1	313	tags=33%, list=18%, signal=41%
GOBP_REGULATION_OF_ACTION_POTENTIAL		10	-0.3719374	-0.9554819	0.56666666	0.7663764	1	168	tags=20%, list=10%, signal=22%
GOBP_REGULATION_OF_SECRETION		70	-0.2828613	-0.9551326	0.57	0.76644963	1	393	tags=27%, list=23%, signal=34%
KEGG_GNRH_SIGNALING_PATHWAY		16	-0.3553479	-0.9550378	0.6210526	0.766153	1	199	tags=19%, list=12%, signal=21%
GOBP_ALCOHOL_METABOLIC_PROCESS		55	-0.2866127	-0.9541563	0.66	0.76753134	1	426	tags=29%, list=25%, signal=38%
GOBP_VESICLE_LOCALIZATION		39	-0.3085313	-0.9536447	0.5858586	0.7679149	1	455	tags=36%, list=27%, signal=48%
GOBP_NUCLEOSIDE_MONOPHOSPHATE_METABOLIC_PROCESS		14	-0.3709124	-0.9534034	0.5425532	0.7678024	1	1066	tags=93%, list=63%, signal=248%
KEGG_MELANOGENESIS		16	-0.3741987	-0.9531543	0.5934066	0.7679164	1	199	tags=19%, list=12%, signal=21%
GOBP_PURINE_NUCLEOSIDE_MONOPHOSPHATE_METABOLIC_PROCESS		12	-0.3699016	-0.9525881	0.6	0.7685543	1	1066	tags=92%, list=63%, signal=245%

GOBP_NEGATIVE_REGULATION_OF_ENDOCYTOSIS		12	-0.3841393	-0.9523339	0.6111111	0.7686327	1	946	tags=83%, list=56%, signal=187%
GOBP_REGULATION_OF_DEPHOSPHORYLATION		19	-0.3290005	-0.9520317	0.5979381	0.76887363	1	599	tags=42%, list=35%, signal=64%
REACTOME_SIGNALING_BY_VEGF		17	-0.3540723	-0.9517708	0.5670103	0.7688454	1	683	tags=53%, list=40%, signal=88%
GOBP_NUCLEAR_CHROMOSOME_SEGREGATION		14	-0.3601713	-0.950019	0.56179774	0.77236897	1	518	tags=43%, list=31%, signal=61%
GOBP_RESPONSE_TO_ISCHEMIA		13	-0.378423	-0.9498754	0.5632184	0.7721767	1	294	tags=31%, list=17%, signal=37%
GOBP_ERBB_SIGNALING_PATHWAY		11	-0.3870881	-0.9492948	0.54651165	0.77290756	1	306	tags=36%, list=18%, signal=44%
GOBP_HEPATICOBILIARY_SYSTEM_DEVELOPMENT		15	-0.3363082	-0.9476541	0.6105263	0.77616644	1	1040	tags=80%, list=61%, signal=205%
GOBP_CELL_AGING		14	-0.3488914	-0.9459289	0.5869565	0.77927256	1	199	tags=21%, list=12%, signal=24%
GOBP_GLIAL_CELL_DEVELOPMENT		14	-0.3566755	-0.9458484	0.5681818	0.77894163	1	518	tags=36%, list=31%, signal=51%
GOBP_CELL_REDOX_HOMEOSTASIS		16	-0.3645915	-0.9456164	0.5913978	0.7789341	1	554	tags=44%, list=33%, signal=64%
GOBP_CELLULAR_LIPID_CATABOLIC_PROCESS		75	-0.2751752	-0.9449643	0.66	0.7798051	1	992	tags=57%, list=59%, signal=132%
GOBP_VESICLE_MEDIATED_TRANSPORT_IN_SYNAPSE		25	-0.3171344	-0.9440542	0.556701	0.78116596	1	666	tags=44%, list=39%, signal=71%
REACTOME_DDX58_IFIH1_MEDIATED_INDUCTION_OF_INTERFERON_ALPHA_BE		10	-0.401088	-0.9437076	0.56179774	0.78135383	1	384	tags=40%, list=23%, signal=51%
GOBP_ENDOTHELIAL_CELL_PROLIFERATION		16	-0.3570411	-0.9434296	0.5638298	0.7815762	1	278	tags=25%, list=16%, signal=30%
GOBP_CHROMOSOME_LOCALIZATION		10	-0.393347	-0.9421563	0.6136364	0.7839974	1	515	tags=40%, list=30%, signal=57%
GOBP_REGULATION_OF_ATPASE_ACTIVITY		23	-0.3234953	-0.9409006	0.5744681	0.78616995	1	447	tags=35%, list=26%, signal=47%
GOBP_PROTEIN_HOMOOLIGOMERIZATION		23	-0.3180354	-0.9406935	0.5531915	0.7859476	1	388	tags=26%, list=23%, signal=33%
GOBP_REGULATION_OF_LYMPHOCYTE_MEDIATED_IMMUNITY		14	-0.3552057	-0.940203	0.5280899	0.78634655	1	323	tags=29%, list=19%, signal=35%
GOBP_POSITIVE_REGULATION_OF_LIPID_METABOLIC_PROCESS		21	-0.3322961	-0.9401708	0.5483871	0.7858524	1	559	tags=38%, list=33%, signal=56%
GOBP_POSITIVE_REGULATION_OF_ION_TRANSPORT		105	-0.2699653	-0.939271	0.69	0.7873753	1	659	tags=42%, list=39%, signal=64%
REACTOME_TRNA_AMINOACYLATION		18	-0.3368073	-0.9375268	0.53932583	0.79057616	1	590	tags=44%, list=35%, signal=67%
GOBP_DEVELOPMENTAL_MATURATION		22	-0.3234076	-0.9374202	0.59183675	0.7902125	1	518	tags=36%, list=31%, signal=52%
GOBP_POSITIVE_REGULATION_OF_CELL_ACTIVATION		31	-0.3007551	-0.9373236	0.56122446	0.78990525	1	640	tags=48%, list=38%, signal=76%
REACTOME_FACTORS_INVOLVED_IN_MEGAKARYOCYTE_DEVELOPMENT_AND		20	-0.3519519	-0.937197	0.6105263	0.7896124	1	234	tags=25%, list=14%, signal=29%
GOBP_HOMEOSTATIC_PROCESS		258	-0.2606956	-0.9352692	0.7	0.79335743	1	573	tags=34%, list=34%, signal=44%
GOBP_MUSCLE_TISSUE_DEVELOPMENT		59	-0.2934999	-0.9350289	0.67	0.7933265	1	360	tags=24%, list=21%, signal=29%
GOBP_POSITIVE_REGULATION_OF_NF_KAPPAB_TRANSCRIPTION_FACTOR_AC		19	-0.3273883	-0.9341492	0.5824176	0.79452187	1	170	tags=21%, list=10%, signal=23%
GOBP_MYELOID_CELL_DIFFERENTIATION		38	-0.300389	-0.9340491	0.6082474	0.7941497	1	851	tags=61%, list=50%, signal=119%
GOBP_MITOCHONDRIAL_TRANSLATIONAL_TERMINATION		63	-0.2769048	-0.9340281	0.65	0.7936256	1	899	tags=56%, list=53%, signal=114%
GOBP_MACROPHAGE_ACTIVATION		10	-0.3730469	-0.933883	0.5777778	0.7933518	1	549	tags=40%, list=32%, signal=59%
GOBP_POSITIVE_REGULATION_OF_ATPASE_ACTIVITY		18	-0.3458749	-0.931955	0.61290324	0.7967975	1	447	tags=39%, list=26%, signal=52%
GOBP_RESPONSE_TO_XENOBIOTIC_STIMULUS		17	-0.339267	-0.9315131	0.64044946	0.79708886	1	305	tags=29%, list=18%, signal=36%
GOBP_CARDIAC_MUSCLE_CELL_ACTION_POTENTIAL		19	-0.3237262	-0.931064	0.56179774	0.7975662	1	659	tags=42%, list=39%, signal=68%
GOBP_ORGANIC_ACID_METABOLIC_PROCESS		262	-0.2526442	-0.9304373	0.74	0.79830575	1	924	tags=53%, list=55%, signal=99%
GOBP_POSITIVE_REGULATION_OF_LIPID_BIOSYNTHETIC_PROCESS		13	-0.3735813	-0.929965	0.59770113	0.79874104	1	308	tags=31%, list=18%, signal=37%
GOBP_REGULATION_OF_CARBOHYDRATE_METABOLIC_PROCESS		20	-0.3303578	-0.9298174	0.5979381	0.7985545	1	281	tags=25%, list=17%, signal=30%
REACTOME_RAB_REGULATION_OF_TRAFFICKING		21	-0.3291626	-0.9297143	0.622449	0.79823035	1	630	tags=43%, list=37%, signal=67%
GOBP_LYMPHOCYTE_MEDIATED_IMMUNITY		23	-0.3215303	-0.9290936	0.5652174	0.7989746	1	434	tags=39%, list=26%, signal=52%
REACTOME_MITOTIC_PROPHASE		11	-0.3726609	-0.9285489	0.5833333	0.79950976	1	195	tags=18%, list=12%, signal=20%
GOBP_POSITIVE_REGULATION_OF_DNA_BINDING		10	-0.3968082	-0.9281159	0.63414633	0.79996383	1	518	tags=40%, list=31%, signal=57%
GOBP_NEGATIVE_REGULATION_OF_GROWTH		23	-0.3270846	-0.9277002	0.63829786	0.8003398	1	308	tags=22%, list=18%, signal=26%
GOBP_RESPONSE_TO_PEPTIDE_HORMONE		64	-0.2870775	-0.9275111	0.66	0.80019414	1	669	tags=42%, list=39%, signal=67%
GOBP_REGULATION_OF_REACTIVE_OXYGEN_SPECIES_METABOLIC_PROCES		34	-0.2946998	-0.9273003	0.625	0.80007553	1	656	tags=50%, list=39%, signal=80%
REACTOME_VISUAL_PHOTOTRANSDUCTION		13	-0.3477976	-0.9249142	0.60465115	0.8042898	1	391	tags=31%, list=23%, signal=40%
GOBP_REGULATION_OF_RAS_PROTEIN_SIGNAL_TRANSDUCTION		14	-0.3398117	-0.9245984	0.5777778	0.80438125	1	253	tags=29%, list=15%, signal=33%
GOBP_CELL_CYCLE_ARREST		18	-0.3230161	-0.9245218	0.5934066	0.80401397	1	1040	tags=78%, list=61%, signal=199%

GOBP_NEGATIVE_REGULATION_OF_TRANSPORTER_ACTIVITY		16	-0.3424659	-0.9244865	0.58064514	0.80353755	1	659	tags=50%, list=39%, signal=81%
GOBP_AGING		47	-0.2831667	-0.9239891	0.62	0.80400544	1	335	tags=23%, list=20%, signal=28%
GOBP_CELLULAR_BIOGENIC_AMINE_METABOLIC_PROCESS		11	-0.3920904	-0.923909	0.61627907	0.8036113	1	982	tags=91%, list=58%, signal=215%
GOBP_REGULATION_OF_SMALL_GTPASE_MEDIATED_SIGNAL_TRANSDUCTION		23	-0.3084162	-0.9231011	0.5714286	0.80454963	1	283	tags=26%, list=17%, signal=31%
GOBP_CALCIUM_MEDIATED_SIGNALING		28	-0.3174213	-0.9226935	0.625	0.8048795	1	309	tags=21%, list=18%, signal=26%
REACTOME_COPII_MEDIATED_VESICLE_TRANSPORT		18	-0.3196077	-0.9226582	0.62637365	0.80443823	1	455	tags=33%, list=27%, signal=45%
GOBP_ANTIMICROBIAL_HUMORAL_RESPONSE		13	-0.3553361	-0.9213895	0.6666667	0.80645066	1	138	tags=23%, list=8%, signal=25%
GOBP_REGULATION_OF_LEUKOCYTE_MEDIATED_IMMUNITY		19	-0.3211263	-0.9209426	0.60638297	0.8069069	1	384	tags=32%, list=23%, signal=40%
GOBP_GLYCEROLIPID_METABOLIC_PROCESS		62	-0.2881675	-0.9206826	0.7	0.8069613	1	274	tags=21%, list=16%, signal=24%
GOBP_REGULATION_OF_CYTOSOLIC_CALCIUM_ION_CONCENTRATION		41	-0.2840534	-0.9196155	0.6363636	0.8085122	1	418	tags=24%, list=25%, signal=32%
GOBP_PATTERN_RECOGNITION_RECEPTOR_SIGNALING_PATHWAY		27	-0.301836	-0.9180702	0.59375	0.81131095	1	134	tags=19%, list=8%, signal=20%
GOBP_REGULATION_OF_TRANSPORT		259	-0.2602333	-0.916533	0.79	0.8136097	1	467	tags=29%, list=28%, signal=34%
GOBP_VESICLE_TARGETING		20	-0.3257701	-0.9161224	0.6421053	0.8138503	1	455	tags=30%, list=27%, signal=41%
GOBP_POSITIVE_REGULATION_OF_CELL_DEATH		73	-0.2664189	-0.9158715	0.7	0.81376445	1	619	tags=40%, list=37%, signal=60%
GOBP_RESPONSE_TO_STARVATION		16	-0.3311982	-0.9155084	0.6813187	0.8138817	1	614	tags=50%, list=36%, signal=78%
REACTOME_TRANSMISSION_ACROSS_CHEMICAL_SYNAPSES		42	-0.2942908	-0.9154787	0.66	0.81339645	1	640	tags=43%, list=38%, signal=67%
GOBP_RESPONSE_TO_LIGHT_STIMULUS		20	-0.3111422	-0.9154584	0.6210526	0.81289154	1	101	tags=15%, list=6%, signal=16%
GOBP_NEGATIVE_REGULATION_OF_VASCULATURE_DEVELOPMENT		15	-0.3390184	-0.9149757	0.60215056	0.81332153	1	294	tags=27%, list=17%, signal=32%
GOBP_METAL_ION_EXPORT		10	-0.3742311	-0.9134194	0.61904764	0.81569564	1	861	tags=60%, list=51%, signal=121%
GOBP_REGULATION_OF_ION_TRANSPORT		200	-0.2578951	-0.9133326	0.77	0.8153117	1	467	tags=29%, list=28%, signal=35%
KEGG_PYRUVATE_METABOLISM		20	-0.3210535	-0.913316	0.59375	0.81474626	1	913	tags=65%, list=54%, signal=139%
REACTOME_RHOG_GTPASE_CYCLE		22	-0.3126478	-0.9127973	0.622449	0.8153415	1	969	tags=68%, list=57%, signal=157%
GOBP_OLEFINIC_COMPOUND_METABOLIC_PROCESS		17	-0.3222565	-0.9108374	0.6082474	0.8186585	1	250	tags=24%, list=15%, signal=27%
GOBP_REGULATION_OF_CELL_PROJECTION_ASSEMBLY		25	-0.3085886	-0.9105067	0.65625	0.818691	1	537	tags=40%, list=32%, signal=58%
GOBP_NEURAL_TUBE_DEVELOPMENT		11	-0.3566735	-0.9101389	0.62068963	0.81898654	1	673	tags=36%, list=40%, signal=60%
KEGG_HEMATOPOIETIC_CELL_LINEAGE		13	-0.3349913	-0.9097971	0.64044946	0.8191402	1	129	tags=23%, list=8%, signal=25%
GOBP_SMOOTH_MUSCLE_CONTRACTION		11	-0.3666807	-0.9097046	0.69411767	0.81874925	1	655	tags=64%, list=39%, signal=103%
GOBP_TISSUE_REGENERATION		11	-0.3715165	-0.9093117	0.5730337	0.8190105	1	574	tags=45%, list=34%, signal=68%
REACTOME_G_ALPHA_Z_SIGNALLING_EVENTS		10	-0.3894996	-0.9080263	0.6395349	0.82096106	1	367	tags=30%, list=22%, signal=38%
GOBP_REGULATION_OF_POSTSYNAPTIC_MEMBRANE_NEUROTRANSMITTER_F		12	-0.3870765	-0.9068298	0.6043956	0.8224612	1	1040	tags=92%, list=61%, signal=236%
REACTOME_RAC2_GTPASE_CYCLE		19	-0.3227014	-0.9063825	0.5979381	0.82269317	1	851	tags=68%, list=50%, signal=136%
GOBP_POSITIVE_REGULATION_OF_INFLAMMATORY_RESPONSE		16	-0.3307475	-0.9034922	0.64772725	0.8274685	1	620	tags=56%, list=37%, signal=88%
GOBP_TAXIS		67	-0.2718884	-0.9032158	0.67676765	0.8275279	1	606	tags=39%, list=36%, signal=58%
KEGG_LYSOSOME		29	-0.298061	-0.9024985	0.6631579	0.8284495	1	297	tags=21%, list=18%, signal=25%
KEGG_LEUKOCYTE_TRANSENDOTHELIAL_MIGRATION		25	-0.3005022	-0.9007779	0.67741936	0.8311561	1	680	tags=48%, list=40%, signal=79%
GOBP_AXO_DENDRITIC_TRANSPORT		13	-0.3451562	-0.9000427	0.6630435	0.8321677	1	515	tags=38%, list=30%, signal=55%
GOBP_BEHAVIOR		38	-0.2953248	-0.8995248	0.6969697	0.8324114	1	322	tags=24%, list=19%, signal=29%
GOBP_ISOPRENOID_METABOLIC_PROCESS		18	-0.3369433	-0.899096	0.6530612	0.83261395	1	1004	tags=78%, list=59%, signal=189%
GOBP_RIBONUCLEOSIDE_METABOLIC_PROCESS		15	-0.342219	-0.8979978	0.7222222	0.8341153	1	844	tags=53%, list=50%, signal=105%
KEGG_ARRHYTHMOGENIC_RIGHT_VENTRICULAR_CARDIOMYOPATHY_ARVC		26	-0.2962847	-0.8960592	0.6770833	0.83729357	1	660	tags=42%, list=39%, signal=68%
GOBP_CELLULAR_AMINO_ACID_CATABOLIC_PROCESS		32	-0.2934363	-0.8932642	0.6458333	0.84223026	1	935	tags=59%, list=55%, signal=130%
REACTOME_SIGNALING_BY_MET		12	-0.3420762	-0.8923868	0.6931818	0.8434293	1	259	tags=25%, list=15%, signal=29%
GOBP_MEMBRANE_INVAGINATION		12	-0.3560163	-0.8918079	0.6511628	0.8439562	1	467	tags=42%, list=28%, signal=57%
GOBP_LYSOSOMAL_TRANSPORT		21	-0.3097299	-0.8910524	0.64893615	0.8448476	1	353	tags=29%, list=21%, signal=36%
REACTOME_RAB_GEFS_EXCHANGE_GTP_FOR_GDP_ON_RABS		19	-0.3136667	-0.8910135	0.6458333	0.84434414	1	803	tags=53%, list=47%, signal=99%
GOBP_MORPHOGENESIS_OF_EMBRYONIC_EPITHELIUM		10	-0.3926917	-0.8904335	0.625	0.8448825	1	673	tags=40%, list=40%, signal=66%

GOBP_REGULATION_OF_SODIUM_ION_TRANSMEMBRANE_TRANSPORT	22	-0.3026793	-0.8903223	0.6666667	0.84459823	1	861	tags=55%, list=51%, signal=109%
GOBP_SYNAPTIC_SIGNALING	66	-0.2680849	-0.8891191	0.7171717	0.84652627	1	640	tags=38%, list=38%, signal=58%
GOBP_REGULATION_OF_SODIUM_ION_TRANSMEMBRANE_TRANSPORTER_AC	17	-0.3090001	-0.8886665	0.6292135	0.84675044	1	830	tags=53%, list=49%, signal=103%
REACTOME_CDC42_GTPASE_CYCLE	15	-0.3360601	-0.8884144	0.6111111	0.8465847	1	969	tags=73%, list=57%, signal=170%
REACTOME_POTENTIAL_THERAPEUTICS_FOR_SARS	16	-0.3344468	-0.8872004	0.6494845	0.84824353	1	982	tags=69%, list=58%, signal=162%
REACTOME_REGULATION_OF_INSULIN_SECRETION	20	-0.31604	-0.8869503	0.6956522	0.84812945	1	367	tags=30%, list=22%, signal=38%
GOBP_REGULATION_OF_RECEPTOR_MEDIATED_ENDOCYTOSIS	21	-0.3149549	-0.8852962	0.7368421	0.85042375	1	974	tags=71%, list=57%, signal=166%
GOBP_MALE_GAMETE_GENERATION	19	-0.3283444	-0.8850167	0.68085104	0.8503283	1	852	tags=53%, list=50%, signal=105%
GOBP_REGULATION_OF_GLUCOSE_METABOLIC_PROCESS	12	-0.3465874	-0.8837269	0.5955056	0.8519596	1	281	tags=25%, list=17%, signal=30%
REACTOME_MYD88_INDEPENDENT_TLR4_CASCADE	10	-0.3468223	-0.8834137	0.625	0.8519229	1	805	tags=70%, list=47%, signal=133%
GOBP_POSITIVE_REGULATION_OF_CELL_CYCLE	25	-0.2987997	-0.8829014	0.6262626	0.85230696	1	534	tags=40%, list=32%, signal=58%
REACTOME_TOLL_LIKE_RECEPTOR_CASCADES	21	-0.3177614	-0.8827904	0.65591395	0.851934	1	811	tags=62%, list=48%, signal=117%
REACTOME_MUSCLE_CONTRACTION	62	-0.2774354	-0.8825148	0.77	0.8518512	1	373	tags=23%, list=22%, signal=28%
GOBP_PROTEIN_ACYLATION	11	-0.3588187	-0.8819979	0.67741936	0.85226095	1	156	tags=18%, list=9%, signal=20%
GOBP_MYELOID_LEUKOCYTE_DIFFERENTIATION	16	-0.3220273	-0.8819411	0.67045456	0.8518434	1	923	tags=69%, list=54%, signal=150%
GOBP_CARBOHYDRATE_DERIVATIVE_METABOLIC_PROCESS	176	-0.2573185	-0.8813399	0.87	0.852298	1	951	tags=57%, list=56%, signal=117%
KEGG_VEGF_SIGNALING_PATHWAY	10	-0.3650425	-0.8797052	0.6117647	0.8548232	1	851	tags=70%, list=50%, signal=140%
GOBP_REGULATION_OF_COMPLEMENT_ACTIVATION	11	-0.3431144	-0.8788648	0.6666667	0.8558249	1	573	tags=55%, list=34%, signal=82%
REACTOME_RHOC_GTPASE_CYCLE	21	-0.3200953	-0.8785387	0.6666667	0.8559033	1	619	tags=52%, list=37%, signal=81%
GOBP_POSITIVE_REGULATION_OF_DNA_BINDING_TRANSCRIPTION_FACTOR_	26	-0.3204098	-0.876666	0.7419355	0.85876197	1	810	tags=62%, list=48%, signal=116%
GOBP_POSITIVE_REGULATION_OF_MUSCLE_CELL_DIFFERENTIATION	10	-0.3698946	-0.8766108	0.64634144	0.85828257	1	39	tags=20%, list=2%, signal=20%
GOBP_VESICLE_CYTOSKELETAL_TRAFFICKING	12	-0.3276714	-0.8750978	0.6703297	0.86026603	1	515	tags=50%, list=30%, signal=71%
GOBP_REGULATION_OF_PLASMA_LIPOPROTEIN_PARTICLE_LEVELS	28	-0.2885595	-0.8748862	0.74747473	0.8601126	1	974	tags=71%, list=57%, signal=165%
GOBP_COPII_COATED_VESICLE_BUDDING	19	-0.3017552	-0.8736576	0.6105263	0.8618305	1	883	tags=58%, list=52%, signal=119%
GOBP_RESPONSE_TO_EXTRACELLULAR_STIMULUS	53	-0.2681726	-0.8736571	0.7	0.8612462	1	669	tags=43%, list=39%, signal=69%
REACTOME_RHOB_GTPASE_CYCLE	14	-0.3328258	-0.8732101	0.70652175	0.861464	1	804	tags=64%, list=47%, signal=121%
GOBP_NEGATIVE_REGULATION_OF_RESPONSE_TO_EXTERNAL_STIMULUS	41	-0.2766132	-0.8722705	0.7113402	0.86258096	1	683	tags=46%, list=40%, signal=76%
GOBP_NUCLEOBASE_CONTAINING_SMALL_MOLECULE_METABOLIC_PROCESS	132	-0.2488661	-0.8720548	0.84	0.86240745	1	951	tags=58%, list=56%, signal=121%
KEGG_ALANINE_ASPARTATE_AND_GLUTAMATE_METABOLISM	10	-0.347031	-0.8712717	0.65909094	0.863229	1	1000	tags=70%, list=59%, signal=170%
GOBP_HYDROGEN_PEROXIDE_METABOLIC_PROCESS	18	-0.320543	-0.8704431	0.6875	0.8639654	1	313	tags=28%, list=18%, signal=34%
GOBP_NEGATIVE_REGULATION_OF_WOUND_HEALING	14	-0.3229055	-0.8703263	0.68817204	0.8635829	1	683	tags=50%, list=40%, signal=83%
GOBP_RESPONSE_TO_TOXIC_SUBSTANCE	48	-0.2704991	-0.8698683	0.7346939	0.8638177	1	820	tags=58%, list=48%, signal=110%
REACTOME_TRANSLATION_OF_SARS_COV_2_STRUCTURAL_PROTEINS	12	-0.3265551	-0.8688577	0.6630435	0.86501163	1	160	tags=17%, list=9%, signal=18%
GOBP_PEROXISOME_ORGANIZATION	30	-0.2754055	-0.8688369	0.67676765	0.8644677	1	912	tags=53%, list=54%, signal=113%
GOBP_VACUOLAR_TRANSPORT	26	-0.2893025	-0.8685756	0.6666667	0.8643651	1	461	tags=31%, list=27%, signal=42%
GOBP_AUTOPHAGOSOME_ORGANIZATION	17	-0.3322353	-0.8681894	0.76404494	0.86439806	1	630	tags=47%, list=37%, signal=74%
GOBP_ESTABLISHMENT_OF_PROTEIN_LOCALIZATION_TO_PLASMA_MEMBRAN	15	-0.3242532	-0.8681494	0.6236559	0.86392635	1	399	tags=33%, list=24%, signal=43%
REACTOME_RAC1_GTPASE_CYCLE	18	-0.3247018	-0.8680041	0.7263158	0.86361665	1	969	tags=72%, list=57%, signal=167%
GOBP_RESPONSE_TO_ALCOHOL	28	-0.2928099	-0.867523	0.65	0.86388874	1	793	tags=54%, list=47%, signal=99%
GOBP_CELL_CHEMOTAXIS	28	-0.2903431	-0.8663924	0.7157895	0.86539316	1	656	tags=46%, list=39%, signal=74%
GOBP_POSTSYNAPSE_ORGANIZATION	17	-0.3227291	-0.8660846	0.65591395	0.8653865	1	851	tags=59%, list=50%, signal=117%
GOBP_REGULATION_OF_NEUROTRANSMITTER_LEVELS	30	-0.280459	-0.8649318	0.71276593	0.86661685	1	640	tags=40%, list=38%, signal=63%
GOBP_INTERLEUKIN_6_PRODUCTION	11	-0.3558997	-0.8645402	0.6888889	0.866603	1	429	tags=36%, list=25%, signal=48%
GOBP_SARCOMERE_ORGANIZATION	14	-0.3222819	-0.8638643	0.7282609	0.86725265	1	406	tags=29%, list=24%, signal=37%
GOBP_PHOSPHOLIPID_CATABOLIC_PROCESS	11	-0.3573671	-0.8627415	0.70454544	0.86852527	1	1038	tags=82%, list=61%, signal=210%
GOBP_REGULATION_OF_SODIUM_ION_TRANSPORT	23	-0.301442	-0.8603824	0.7113402	0.8718828	1	861	tags=52%, list=51%, signal=105%

GOBP_MULTI_MULTICELLULAR_ORGANISM_PROCESS		17	-0.3199689	-0.8600221	0.7083333	0.871981	1	241	tags=18%, list=14%, signal=20%
GOBP_POSITIVE_REGULATION_OF_PEPTIDE_HORMONE_SECRETION		17	-0.3172544	-0.8600124	0.6292135	0.871418	1	866	tags=59%, list=51%, signal=119%
GOBP_NEGATIVE_REGULATION_OF_TRANSPORT		67	-0.2612331	-0.8597767	0.77	0.87115675	1	391	tags=28%, list=23%, signal=35%
REACTOME_ACTIVATION_OF_NMDA_RECEPTORS_AND_POSTSYNAPTIC_EVEN		18	-0.322172	-0.8592511	0.7222222	0.8714411	1	759	tags=50%, list=45%, signal=90%
REACTOME_NEURONAL_SYSTEM		50	-0.266634	-0.8592174	0.7373737	0.8709181	1	640	tags=42%, list=38%, signal=65%
GOBP_GLYCOSYLATION		18	-0.3176214	-0.8583949	0.6989247	0.87181664	1	65	tags=11%, list=4%, signal=11%
GOBP_PHOSPHATIDYLCHOLINE_METABOLIC_PROCESS		16	-0.317288	-0.8572752	0.7282609	0.87325275	1	431	tags=31%, list=25%, signal=42%
REACTOME_ORGANELLE_BIOGENESIS_AND_MAINTENANCE		59	-0.2613504	-0.8562541	0.77	0.87426424	1	567	tags=34%, list=33%, signal=49%
REACTOME_GLYCEROPHOSPHOLIPID_BIOSYNTHESIS		21	-0.2930874	-0.8560388	0.70212764	0.8740155	1	225	tags=14%, list=13%, signal=16%
GOBP_NEGATIVE_REGULATION_OF_RESPONSE_TO_WOUNDING		14	-0.3229055	-0.854535	0.7011494	0.87602824	1	683	tags=50%, list=40%, signal=83%
REACTOME_SARS_COV_INFECTIONS		31	-0.2804761	-0.8539915	0.78125	0.8762954	1	861	tags=52%, list=51%, signal=103%
REACTOME_TOLL_LIKE_RECEPTOR_TLR1_TLR2_CASCADE		14	-0.3272213	-0.8528053	0.6666667	0.87772775	1	805	tags=64%, list=47%, signal=121%
GOBP_ORGANIC_HYDROXY_COMPOUND_METABOLIC_PROCESS		72	-0.2625268	-0.8526075	0.81	0.87747043	1	426	tags=28%, list=25%, signal=36%
GOBP_ORGANOPHOSPHATE_METABOLIC_PROCESS		182	-0.2374473	-0.852303	0.85	0.87737244	1	956	tags=57%, list=56%, signal=117%
GOBP_IRON_ION_HOMEOSTASIS		22	-0.2880657	-0.8517964	0.71428573	0.87763846	1	420	tags=27%, list=25%, signal=36%
GOBP_PROTEIN_EXIT_FROM_ENDOPLASMIC_RETICULUM		13	-0.3352213	-0.8512477	0.7692308	0.8780309	1	868	tags=62%, list=51%, signal=125%
GOBP_MAINTENANCE_OF_LOCATION		57	-0.2493952	-0.8512411	0.79	0.8774633	1	420	tags=26%, list=25%, signal=34%
KEGG_AMINOACYL_TRNA_BIOSYNTHESIS		16	-0.3181998	-0.8510581	0.70526314	0.877144	1	590	tags=44%, list=35%, signal=66%
GOBP_ACTION_POTENTIAL		27	-0.2853795	-0.850403	0.7755102	0.87747806	1	659	tags=37%, list=39%, signal=60%
GOBP_REGULATION_OF_G_PROTEIN_COUPLED_RECEPTOR_SIGNALING_PAT		12	-0.3477804	-0.8487507	0.7032967	0.87953043	1	294	tags=25%, list=17%, signal=30%
GOBP_GLYCEROPHOSPHOLIPID_METABOLIC_PROCESS		46	-0.2638055	-0.8481297	0.77	0.87997127	1	537	tags=30%, list=32%, signal=43%
GOBP_CELLULAR_HOMEOSTASIS		155	-0.2433154	-0.8476275	0.87	0.88027793	1	573	tags=33%, list=34%, signal=45%
GOBP_RELEASE_OF_CYTOCHROME_C_FROM_MITOCHONDRIA		12	-0.3395347	-0.8467114	0.6923077	0.8810722	1	362	tags=25%, list=21%, signal=32%
REACTOME_MITOCHONDRIAL_TRANSLATION		68	-0.2527011	-0.846008	0.78	0.8815806	1	899	tags=53%, list=53%, signal=108%
GOBP_LEUKOCYTE_CHEMOTAXIS		21	-0.3085087	-0.8459461	0.70212764	0.8810832	1	606	tags=48%, list=36%, signal=73%
GOBP_TISSUE_HOMEOSTASIS		25	-0.2922176	-0.8453406	0.71875	0.88136953	1	728	tags=52%, list=43%, signal=90%
GOBP_NEUROTRANSMITTER_TRANSPORT		24	-0.2961658	-0.8447794	0.69072163	0.8816428	1	640	tags=42%, list=38%, signal=66%
REACTOME_RHOA_GTPASE_CYCLE		30	-0.2810151	-0.8444241	0.7113402	0.881664	1	619	tags=43%, list=37%, signal=67%
GOBP_NEGATIVE_REGULATION_OF_TRANSMEMBRANE_RECEPTOR_PROTEIN_		11	-0.3469853	-0.8433009	0.71428573	0.8829709	1	1016	tags=82%, list=60%, signal=203%
GOBP_HORMONE_METABOLIC_PROCESS		22	-0.2945758	-0.8426043	0.7010309	0.88346237	1	967	tags=68%, list=57%, signal=157%
GOBP_GOLGI_TO_PLASMA_MEMBRANE_PROTEIN_TRANSPORT		11	-0.3309108	-0.8418406	0.7032967	0.88409233	1	392	tags=36%, list=23%, signal=47%
GOBP_MACROMOLECULE_METHYLATION		14	-0.3368304	-0.8417901	0.6956522	0.883602	1	516	tags=43%, list=30%, signal=61%
GOBP_POSITIVE_REGULATION_OF_MITOTIC_CELL_CYCLE		11	-0.3415814	-0.8408156	0.6896552	0.8846661	1	534	tags=45%, list=32%, signal=66%
GOBP_POSITIVE_REGULATION_OF_POTASSIUM_ION_TRANSPORT		10	-0.3392893	-0.8401103	0.6385542	0.8851628	1	861	tags=50%, list=51%, signal=101%
GOBP_VASCULAR_PROCESS_IN_CIRCULATORY_SYSTEM		36	-0.2832778	-0.8393461	0.7979798	0.8856586	1	707	tags=50%, list=42%, signal=84%
GOBP_RESPONSE_TO_PURINE_CONTAINING_COMPOUND		24	-0.2797525	-0.8391076	0.7222222	0.88546926	1	225	tags=21%, list=13%, signal=24%
GOBP_SODIUM_ION_TRANSMEMBRANE_TRANSPORT		27	-0.270071	-0.8389074	0.7244898	0.8851804	1	861	tags=52%, list=51%, signal=104%
GOBP_NEGATIVE_REGULATION_OF_PROTEIN_BINDING		13	-0.3229704	-0.8384877	0.7	0.8852986	1	670	tags=54%, list=40%, signal=88%
GOBP_SKELETAL_MUSCLE_CELL_DIFFERENTIATION		10	-0.3310919	-0.8376125	0.7173913	0.8860629	1	1016	tags=90%, list=60%, signal=223%
GOBP_LIPOSACCHARIDE_METABOLIC_PROCESS		13	-0.3219001	-0.8358508	0.6818182	0.8882517	1	988	tags=77%, list=58%, signal=183%
GOBP_POSITIVE_REGULATION_OF_EPITHELIAL_CELL_MIGRATION		21	-0.2937549	-0.8354784	0.68085104	0.8881811	1	725	tags=48%, list=43%, signal=82%
GOBP_PEPTIDYL_LYSINE_MODIFICATION		16	-0.3155002	-0.8347012	0.742268	0.88870376	1	539	tags=50%, list=32%, signal=73%
GOBP_CHOLESTEROL_EFFLUX		14	-0.3067798	-0.8343763	0.73333335	0.8885943	1	916	tags=64%, list=54%, signal=139%
GOBP_PYRIMIDINE_CONTAINING_COMPOUND_METABOLIC_PROCESS		10	-0.3459813	-0.8343148	0.7241379	0.88811064	1	844	tags=60%, list=50%, signal=119%
REACTOME_METABOLISM_OF_FAT_SOLUBLE_VITAMINS		10	-0.3469673	-0.8322152	0.6593407	0.8905608	1	1004	tags=80%, list=59%, signal=195%
REACTOME_PHASE_I_FUNCTIONALIZATION_OF_COMPOUNDS		13	-0.3281648	-0.8319764	0.7222222	0.89032006	1	692	tags=62%, list=41%, signal=103%

GOBP_MUSCLE_CELL_DIFFERENTIATION		58	-0.2566703	-0.8316712	0.81	0.89022905	1	466	tags=29%, list=27%, signal=39%
GOBP_ALPHA_AMINO_ACID_CATABOLIC_PROCESS		24	-0.2812552	-0.8307853	0.7717391	0.8909041	1	1029	tags=63%, list=61%, signal=157%
GOBP_BLOOD_VESSEL_ENDOTHELIAL_CELL_MIGRATION		14	-0.313584	-0.8303835	0.7340425	0.8909311	1	683	tags=43%, list=40%, signal=71%
GOBP_CYTOPLASMIC_PATTERN_RECOGNITION_RECEPTOR_SIGNALING_PATH		10	-0.3390108	-0.8302743	0.6896552	0.89047927	1	576	tags=50%, list=34%, signal=75%
GOBP_MULTICELLULAR_ORGANISMAL_MOVEMENT		20	-0.3014289	-0.8300031	0.70652175	0.89034486	1	286	tags=15%, list=17%, signal=18%
GOBP_SKELETAL_MUSCLE_CONTRACTION		20	-0.3014289	-0.8280901	0.73913044	0.8926396	1	286	tags=15%, list=17%, signal=18%
GOBP_SKELETAL_MUSCLE_TISSUE_REGENERATION		10	-0.3480164	-0.8279133	0.73170733	0.89233625	1	551	tags=40%, list=33%, signal=59%
GOBP_MEMBRANE_DEPOLARIZATION		18	-0.3073213	-0.8277356	0.78125	0.89202744	1	308	tags=22%, list=18%, signal=27%
GOBP_ORGANOPHOSPHATE_ESTER_TRANSPORT		23	-0.2902971	-0.8276413	0.75510204	0.891607	1	507	tags=35%, list=30%, signal=49%
GOBP_VESICLE_TARGETING_TO_FROM_OR_WITHIN_GOLGI		17	-0.2877271	-0.826783	0.69473684	0.8924505	1	883	tags=59%, list=52%, signal=122%
GOBP_REGULATION_OF_ATP_METABOLIC_PROCESS		19	-0.2883607	-0.8266006	0.72727275	0.8921175	1	552	tags=37%, list=33%, signal=54%
GOBP_ORGANELLE_MEMBRANE_FUSION		31	-0.2675904	-0.8262045	0.75757575	0.8920745	1	469	tags=29%, list=28%, signal=39%
GOBP_NEUROMUSCULAR_JUNCTION_DEVELOPMENT		10	-0.3411666	-0.8259919	0.72619045	0.89185977	1	287	tags=30%, list=17%, signal=36%
GOBP_CELLULAR_RESPONSE_TO_STARVATION		11	-0.3337873	-0.8258393	0.752809	0.8915333	1	539	tags=45%, list=32%, signal=66%
GOBP_QUINONE_METABOLIC_PROCESS		13	-0.322277	-0.8257718	0.6956522	0.89109033	1	773	tags=38%, list=46%, signal=70%
GOBP_MYELOID_CELL_HOMEOSTASIS		19	-0.2947211	-0.8242271	0.76344085	0.89280826	1	601	tags=42%, list=35%, signal=65%
REACTOME_TRANSCRIPTIONAL_REGULATION_BY_TP53		43	-0.2636291	-0.822932	0.83838385	0.8939861	1	539	tags=35%, list=32%, signal=50%
GOBP_SMALL_MOLECULE_METABOLIC_PROCESS		394	-0.2256547	-0.8221097	0.98	0.8945224	1	956	tags=55%, list=56%, signal=97%
GOBP_POSITIVE_REGULATION_OF_PROTEIN_KINASE_B_SIGNALING		13	-0.3083785	-0.8208576	0.8235294	0.8955988	1	607	tags=46%, list=36%, signal=71%
GOBP_NEURAL_NUCLEUS_DEVELOPMENT		21	-0.2780834	-0.8206778	0.7628866	0.89537656	1	535	tags=38%, list=32%, signal=55%
GOBP_SUBSTANTIA_NIGRA_DEVELOPMENT		21	-0.2780834	-0.8202229	0.70652175	0.8955482	1	535	tags=38%, list=32%, signal=55%
GOBP_ALPHA_AMINO_ACID_METABOLIC_PROCESS		36	-0.2636783	-0.8195397	0.7676768	0.89587975	1	935	tags=53%, list=55%, signal=115%
GOBP_POST_GOLGI_VESICLE_MEDIATED_TRANSPORT		27	-0.2654049	-0.8188117	0.73195875	0.8963698	1	461	tags=33%, list=27%, signal=45%
GOBP_PROTEIN_LOCALIZATION_TO_SYNAPSE		11	-0.3052913	-0.8187946	0.7078652	0.89581585	1	1078	tags=73%, list=64%, signal=198%
GOBP_MEMBRANE_LIPID_METABOLIC_PROCESS		27	-0.2724089	-0.8187001	0.73195875	0.8954161	1	934	tags=59%, list=55%, signal=130%
GOBP_LONG_CHAIN_FATTY_ACID_METABOLIC_PROCESS		24	-0.2721781	-0.8182736	0.6969697	0.89542127	1	250	tags=17%, list=15%, signal=19%
GOBP_NEGATIVE_REGULATION_OF_TRANSMEMBRANE_TRANSPORT		23	-0.2878799	-0.8171278	0.7916667	0.8963821	1	366	tags=30%, list=22%, signal=38%
GOBP_SODIUM_ION_TRANSPORT		28	-0.269506	-0.816713	0.78350514	0.896429	1	861	tags=50%, list=51%, signal=100%
GOBP_MUSCLE_CELL_MIGRATION		10	-0.3326107	-0.8162532	0.7176471	0.8964948	1	656	tags=50%, list=39%, signal=81%
GOBP_LONG_CHAIN_FATTY_ACID_TRANSPORT		15	-0.3006391	-0.8160312	0.7419355	0.89626056	1	559	tags=33%, list=33%, signal=49%
GOBP_PEPTIDE_HORMONE_SECRETION		31	-0.2711984	-0.8159619	0.8181818	0.8957882	1	393	tags=29%, list=23%, signal=37%
GOBP_CARBOHYDRATE_TRANSMEMBRANE_TRANSPORT		10	-0.3308474	-0.8155417	0.74418604	0.8957014	1	350	tags=30%, list=21%, signal=38%
BIOCARTA_PPARA_PATHWAY		11	-0.3338023	-0.8148658	0.8021978	0.8960667	1	603	tags=45%, list=36%, signal=70%
GOBP_ERYTHROCYTE_HOMEOSTASIS		15	-0.2987689	-0.81414	0.76344085	0.89652354	1	781	tags=60%, list=46%, signal=110%
GOBP_DETOXIFICATION		35	-0.2529817	-0.8138304	0.7878788	0.8964549	1	820	tags=57%, list=48%, signal=108%
GOBP_FATTY_ACID_BETA_OXIDATION		36	-0.2618899	-0.8136982	0.78350514	0.8959904	1	992	tags=53%, list=59%, signal=125%
GOBP_ANATOMICAL_STRUCTURE_MATURATION		13	-0.3158061	-0.8129596	0.7553192	0.89642155	1	518	tags=31%, list=31%, signal=44%
GOBP_MONOCARBOXYLIC_ACID_CATABOLIC_PROCESS		48	-0.2565265	-0.8128159	0.79	0.8960733	1	992	tags=52%, list=59%, signal=122%
GOBP_STRESS_ACTIVATED_PROTEIN_KINASE_SIGNALING_CASCADE		28	-0.2902269	-0.8124301	0.7653061	0.89595646	1	278	tags=25%, list=16%, signal=29%
GOBP_NEGATIVE_REGULATION_OF_MITOCHONDRION_ORGANIZATION		14	-0.3113399	-0.8119911	0.76086956	0.89607084	1	414	tags=29%, list=24%, signal=37%
GOBP_MAINTENANCE_OF_LOCATION_IN_CELL		42	-0.2670749	-0.8104065	0.83838385	0.89781123	1	461	tags=29%, list=27%, signal=38%
GOBP_NEURON_PROJECTION_GUIDANCE		29	-0.2736532	-0.8094097	0.8061224	0.8987439	1	553	tags=34%, list=33%, signal=50%
KEGG_NEUROTROPHIN_SIGNALING_PATHWAY		20	-0.290722	-0.8086158	0.75	0.8991772	1	1077	tags=75%, list=64%, signal=203%
GOBP_REGULATION_OF_HEART_RATE		20	-0.2855874	-0.8059807	0.76842105	0.9024787	1	619	tags=30%, list=37%, signal=47%
GOBP_PIGMENT_METABOLIC_PROCESS		10	-0.3206541	-0.8052026	0.74698794	0.90284854	1	153	tags=20%, list=9%, signal=22%
GOBP_NEGATIVE_REGULATION_OF_DEFENSE_RESPONSE		23	-0.2855049	-0.8044721	0.8	0.9031828	1	205	tags=22%, list=12%, signal=24%

GOBP_ORGANIC_ACID_CATABOLIC_PROCESS		87	-0.236947	-0.8044324	0.87	0.90268105	1	1000	tags=52%, list=59%, signal=120%
GOBP_SISTER_CHROMATID_SEGREGATION		10	-0.3306048	-0.8031652	0.7977528	0.90383583	1	399	tags=30%, list=24%, signal=39%
GOBP_EXPORT_ACROSS_PLASMA_MEMBRANE		11	-0.3259106	-0.8023398	0.7303371	0.904264	1	1049	tags=73%, list=62%, signal=190%
GOBP_POSITIVE_REGULATION_OF_LIPID_TRANSPORT		15	-0.3075421	-0.8011342	0.7888889	0.90535057	1	640	tags=53%, list=38%, signal=85%
GOBP_REGULATION_OF_PH		17	-0.2981736	-0.7993819	0.7894737	0.90688264	1	123	tags=12%, list=7%, signal=13%
GOBP_REGULATION_OF_ACTIN_FILAMENT_BASED_MOVEMENT		11	-0.3202592	-0.798303	0.78723407	0.9079248	1	927	tags=64%, list=55%, signal=140%
GOBP_REGULATION_OF_MEMBRANE_PERMEABILITY		20	-0.282713	-0.7982917	0.7368421	0.90736157	1	535	tags=35%, list=32%, signal=51%
GOBP_CIRCULATORY_SYSTEM_PROCESS		101	-0.2335932	-0.7978201	0.9	0.90734124	1	688	tags=40%, list=41%, signal=63%
REACTOME_METABOLISM_OF_VITAMINS_AND_COFACTORS		42	-0.2484487	-0.7978025	0.8080808	0.906803	1	967	tags=57%, list=57%, signal=130%
GOBP_PURINE_CONTAINING_COMPOUND_METABOLIC_PROCESS		113	-0.2296296	-0.7967451	0.94	0.90765554	1	911	tags=53%, list=54%, signal=107%
GOBP_REACTIVE_OXYGEN_SPECIES_METABOLIC_PROCESS		56	-0.2396482	-0.7940124	0.85714287	0.91063535	1	313	tags=23%, list=18%, signal=28%
GOBP_MEMBRANE_LIPID_BIOSYNTHETIC_PROCESS		16	-0.2774197	-0.7929867	0.7395833	0.91139966	1	988	tags=63%, list=58%, signal=148%
GOBP_POSITIVE_REGULATION_OF_CHEMOTAXIS		12	-0.3136973	-0.7922258	0.7717391	0.9118148	1	502	tags=42%, list=30%, signal=59%
REACTOME_COPI_DEPENDENT_GOLGI_TO_ER_RETROGRADE_TRAFFIC		18	-0.2956981	-0.7917703	0.80851066	0.9118822	1	883	tags=56%, list=52%, signal=115%
GOBP_REGULATION_OF_TRANS_SYNAPTIC_SIGNALING		42	-0.2403614	-0.7899651	0.7777778	0.9136534	1	640	tags=36%, list=38%, signal=56%
GOBP_TOLL_LIKE_RECEPTOR_SIGNALING_PATHWAY		18	-0.2843851	-0.789413	0.7368421	0.91378427	1	134	tags=17%, list=8%, signal=18%
GOBP_PROTEIN_PROCESSING		34	-0.2471779	-0.7893253	0.81632656	0.9133048	1	790	tags=44%, list=47%, signal=81%
GOBP_REGULATION_OF_TRANSMEMBRANE_TRANSPORT		90	-0.232274	-0.7888995	0.87	0.91326874	1	372	tags=21%, list=22%, signal=26%
GOBP_BODY_FLUID_SECRETION		11	-0.3096237	-0.7884955	0.8023256	0.9132149	1	13	tags=9%, list=1%, signal=9%
GOBP_NEURON_PROJECTION_ORGANIZATION		13	-0.3120342	-0.7868889	0.84444445	0.91458964	1	851	tags=62%, list=50%, signal=123%
GOBP_DENDRITE_DEVELOPMENT		20	-0.2687134	-0.786615	0.79347825	0.9143139	1	851	tags=50%, list=50%, signal=99%
GOBP_REGULATION_OF_SYNAPTIC_PLASTICITY		19	-0.2805476	-0.7864716	0.7916667	0.91397876	1	1055	tags=68%, list=62%, signal=179%
REACTOME_ASSEMBLY_AND_CELL_SURFACE_PRESENTATION_OF_NMDA_RE		11	-0.3251455	-0.7853295	0.78494626	0.914754	1	759	tags=55%, list=45%, signal=98%
GOBP_SULFUR_COMPOUND_BIOSYNTHETIC_PROCESS		44	-0.2361943	-0.7850887	0.7755102	0.91455585	1	911	tags=52%, list=54%, signal=110%
GOBP_TRANSITION_METAL_ION_HOMEOSTASIS		29	-0.2638133	-0.7831713	0.8484849	0.91631943	1	544	tags=31%, list=32%, signal=45%
REACTOME_TRANSPORT_OF_SMALL_MOLECULES		158	-0.2255252	-0.7830032	0.94	0.91594857	1	820	tags=48%, list=48%, signal=84%
GOBP_REGULATION_OF_ESTABLISHMENT_OF_PROTEIN_LOCALIZATION_TO_M		13	-0.3069339	-0.7828309	0.81609195	0.91558313	1	513	tags=46%, list=30%, signal=66%
GOBP_EPHRIN_RECEPTOR_SIGNALING_PATHWAY		14	-0.3156081	-0.7827873	0.7826087	0.9150583	1	691	tags=50%, list=41%, signal=84%
REACTOME_SPHINGOLIPID_METABOLISM		14	-0.2999735	-0.7816709	0.7096774	0.9158726	1	684	tags=43%, list=40%, signal=71%
GOBP_INTERFERON_GAMMA_MEDIATED_SIGNALING_PATHWAY		11	-0.3235305	-0.780623	0.8202247	0.9167443	1	90	tags=18%, list=5%, signal=19%
GOBP_MONOCARBOXYLIC_ACID_METABOLIC_PROCESS		161	-0.2230741	-0.7804084	0.94	0.9163919	1	916	tags=50%, list=54%, signal=99%
GOBP_CELLULAR_RESPONSE_TO_TOXIC_SUBSTANCE		35	-0.2529817	-0.778849	0.85	0.9177421	1	820	tags=57%, list=48%, signal=108%
GOBP_FATTY_ACID_CATABOLIC_PROCESS		46	-0.2425439	-0.7777507	0.81632656	0.91846937	1	992	tags=50%, list=59%, signal=117%
GOBP_CELLULAR_RESPONSE_TO_VIRUS		11	-0.3132319	-0.7775697	0.8068182	0.9181698	1	326	tags=27%, list=19%, signal=34%
GOBP_RESPONSE_TO_ESTRADIOL		16	-0.283542	-0.7765061	0.79347825	0.9189312	1	252	tags=25%, list=15%, signal=29%
GOBP_REGULATION_OF_PEPTIDE_SECRETION		39	-0.2493623	-0.7761154	0.8061224	0.91875595	1	393	tags=26%, list=23%, signal=33%
GOBP_B_CELL_ACTIVATION		11	-0.3130631	-0.7759374	0.74712646	0.9184272	1	707	tags=55%, list=42%, signal=93%
GOBP_NEGATIVE_REGULATION_OF_SECRETION		14	-0.3010999	-0.7754133	0.78723407	0.9184478	1	318	tags=29%, list=19%, signal=35%
GOBP_PHOSPHOLIPID_BIOSYNTHETIC_PROCESS		35	-0.2442571	-0.7751013	0.82653064	0.9183502	1	225	tags=17%, list=13%, signal=19%
GOBP_DETECTION_OF_STIMULUS		20	-0.2773682	-0.7738742	0.8131868	0.919304	1	842	tags=50%, list=50%, signal=98%
REACTOME_DETOXIFICATION_OF_REACTIVE_OXYGEN_SPECIES		17	-0.2988497	-0.773645	0.80851066	0.91901094	1	470	tags=35%, list=28%, signal=48%
GOBP_REGULATION_OF_CELLULAR_CARBOHYDRATE_METABOLIC_PROCESS		14	-0.2988076	-0.7732326	0.8023256	0.91893065	1	281	tags=21%, list=17%, signal=25%
GOBP_GLUTAMINE_FAMILY_AMINO_ACID_METABOLIC_PROCESS		16	-0.2829746	-0.7715675	0.7741935	0.92009413	1	703	tags=44%, list=41%, signal=74%
GOBP_CHEMICAL_HOMEOSTASIS		178	-0.2180861	-0.771198	0.98	0.9199316	1	669	tags=35%, list=39%, signal=52%
GOBP_NEGATIVE_REGULATION_OF_TRANSCRIPTION_BY_RNA_POLYMERASE_		30	-0.2634993	-0.7696427	0.86	0.9212755	1	629	tags=43%, list=37%, signal=68%
GOBP_MONOVALENT_INORGANIC_CATION_HOMEOSTASIS		26	-0.2538398	-0.7682024	0.8125	0.92214745	1	143	tags=12%, list=8%, signal=12%

GOBP_REGULATION_OF_NUCLEOTIDE_METABOLIC_PROCESS		18	-0.2877952	-0.7681825	0.7659575	0.92161864	1	791	tags=56%, list=47%, signal=103%
GOBP_SARCOPLASMIC_RETICULUM_CALCIUM_ION_TRANSPORT		19	-0.268745	-0.7667065	0.78125	0.9225771	1	115	tags=11%, list=7%, signal=11%
GOBP_CALCIUM_ION_IMPORT		17	-0.2874359	-0.7665721	0.8426966	0.9221606	1	173	tags=18%, list=10%, signal=19%
GOBP_STRIATED_MUSCLE_CONTRACTION		52	-0.2364645	-0.7619097	0.89	0.926951	1	496	tags=23%, list=29%, signal=32%
GOBP_MIDBRAIN_DEVELOPMENT		23	-0.2532067	-0.7616332	0.79569894	0.926714	1	535	tags=35%, list=32%, signal=50%
GOBP_MITOCHONDRIAL_RNA_METABOLIC_PROCESS		13	-0.2999907	-0.7547343	0.8172043	0.9337178	1	996	tags=69%, list=59%, signal=167%
REACTOME_TOLL_LIKE_RECEPTOR_9_TLR9_CASCADE		11	-0.2887789	-0.7546673	0.7586207	0.9332194	1	805	tags=55%, list=47%, signal=103%
GOBP_SMALL_MOLECULE_BIOSYNTHETIC_PROCESS		130	-0.2190837	-0.7545923	0.93	0.93273306	1	916	tags=55%, list=54%, signal=110%
GOBP_SKELETAL_MUSCLE_ORGAN_DEVELOPMENT		38	-0.2430477	-0.7538266	0.81	0.93292034	1	407	tags=24%, list=24%, signal=30%
GOBP_ORGANOPHOSPHATE_BIOSYNTHETIC_PROCESS		114	-0.2102703	-0.7502956	0.92	0.9359437	1	237	tags=15%, list=14%, signal=16%
GOBP_SYNAPTIC_VESICLE_EXOCYTOSIS		13	-0.2782424	-0.7498754	0.84782606	0.93583655	1	640	tags=38%, list=38%, signal=61%
GOBP_JNK_CASCADE		15	-0.2807942	-0.749503	0.8636364	0.93566513	1	1109	tags=93%, list=65%, signal=268%
REACTOME_SIGNALING_BY_NUCLEAR_RECEPTORS		36	-0.2479977	-0.7485645	0.89	0.9360665	1	896	tags=53%, list=53%, signal=110%
GOBP_POTASSIUM_ION_TRANSPORT		31	-0.2402773	-0.7478895	0.8913044	0.93621117	1	659	tags=39%, list=39%, signal=62%
GOBP_REGULATION_OF_MUSCLE_CELL_DIFFERENTIATION		13	-0.2969452	-0.7460126	0.8333333	0.9373577	1	39	tags=15%, list=2%, signal=16%
GOBP_SUPEROXIDE_METABOLIC_PROCESS		13	-0.2743598	-0.7459418	0.8111111	0.93683624	1	915	tags=69%, list=54%, signal=149%
GOBP_REGULATION_OF_CELL_KILLING		10	-0.312311	-0.7415995	0.8414634	0.94081634	1	323	tags=30%, list=19%, signal=37%
GOBP_PEROXISOMAL_TRANSPORT		27	-0.2477929	-0.7400585	0.79787236	0.94162077	1	912	tags=52%, list=54%, signal=110%
GOBP_POSITIVE_REGULATION_OF_SYNAPTIC_TRANSMISSION		12	-0.2969632	-0.7383986	0.83516484	0.942691	1	640	tags=42%, list=38%, signal=66%
GOBP_MEMBRANE_ORGANIZATION		229	-0.2053835	-0.7365379	0.98	0.9440168	1	513	tags=28%, list=30%, signal=35%
GOBP_NERVOUS_SYSTEM_PROCESS		71	-0.2221319	-0.733565	0.9292929	0.9462399	1	487	tags=27%, list=29%, signal=36%
GOBP_CELLULAR_LIPID_METABOLIC_PROCESS		187	-0.2043757	-0.7334284	0.98	0.94585425	1	1052	tags=61%, list=62%, signal=143%
KEGG_GLYCEROPHOSPHOLIPID_METABOLISM		14	-0.2818194	-0.7330274	0.86170214	0.94562584	1	289	tags=21%, list=17%, signal=26%
GOBP_POSITIVE_REGULATION_OF_PEPTIDASE_ACTIVITY		32	-0.2332938	-0.7309834	0.82653064	0.9470555	1	322	tags=19%, list=19%, signal=23%
GOBP_HOMEOSTASIS_OF_NUMBER_OF_CELLS		27	-0.2471785	-0.730913	0.8369565	0.9465541	1	786	tags=52%, list=46%, signal=95%
REACTOME_RAB_GERANYLGERANYLATION		19	-0.2692317	-0.7305049	0.8064516	0.9463486	1	883	tags=53%, list=52%, signal=109%
GOBP_REGULATION_OF_GENERATION_OF_PRECURSOR_METABOLITES_AND_		26	-0.2489797	-0.729245	0.8367347	0.9470042	1	335	tags=23%, list=20%, signal=28%
GOBP_CELLULAR_MONOVALENT_INORGANIC_CATION_HOMEOSTASIS		22	-0.2472319	-0.7288266	0.875	0.946776	1	497	tags=23%, list=29%, signal=32%
GOBP_STEROL_TRANSPORT		20	-0.2618936	-0.727281	0.8913044	0.94756407	1	916	tags=65%, list=54%, signal=140%
REACTOME_EPHB_MEDIATED_FORWARD_SIGNALING		10	-0.3052375	-0.7238509	0.8791209	0.95024705	1	668	tags=50%, list=39%, signal=82%
GOBP_REGULATION_OF_LIPID_BIOSYNTHETIC_PROCESS		30	-0.2310436	-0.7228081	0.9157895	0.9505886	1	308	tags=20%, list=18%, signal=24%
GOBP_REGULATION_OF_MITOCHONDRIAL_MEMBRANE_POTENTIAL		17	-0.2574007	-0.7210943	0.83146065	0.95151305	1	308	tags=24%, list=18%, signal=28%
GOBP_TRANSMEMBRANE_TRANSPORT		270	-0.2028522	-0.7209534	0.99	0.9510864	1	372	tags=19%, list=22%, signal=21%
REACTOME_SARS_COV_2_INFECTION		15	-0.2705852	-0.7199305	0.8736842	0.9514675	1	160	tags=13%, list=9%, signal=15%
GOBP_REGULATION_OF_NUCLEOTIDE_BIOSYNTHETIC_PROCESS		12	-0.2913353	-0.7182003	0.8586956	0.95255035	1	888	tags=67%, list=52%, signal=139%
GOBP_REGULATION_OF_DEFENSE_RESPONSE_TO_VIRUS_BY_VIRUS		10	-0.2992002	-0.7152479	0.8202247	0.95451224	1	1077	tags=80%, list=64%, signal=218%
GOBP_LIPID_OXIDATION		43	-0.2321894	-0.7151929	0.90909094	0.95398706	1	992	tags=51%, list=59%, signal=120%
GOBP_REGULATION_OF_MITOCHONDRIAL_MEMBRANE_PERMEABILITY		19	-0.2509036	-0.7136031	0.9247312	0.95479006	1	535	tags=32%, list=32%, signal=46%
GOBP_POSITIVE_REGULATION_OF_HORMONE_SECRETION		18	-0.2551347	-0.7124116	0.8645833	0.95532763	1	393	tags=28%, list=23%, signal=36%
GOBP_SIGNAL_RELEASE		50	-0.2225503	-0.710907	0.9183673	0.9561641	1	393	tags=26%, list=23%, signal=33%
GOBP_DICARBOXYLIC_ACID_METABOLIC_PROCESS		36	-0.2389725	-0.7084367	0.9	0.9577408	1	1029	tags=58%, list=61%, signal=145%
GOBP_CYTOSKELETON_DEPENDENT_INTRACELLULAR_TRANSPORT		29	-0.2429543	-0.7080598	0.90625	0.9575939	1	515	tags=34%, list=30%, signal=49%
KEGG_BETA_ALANINE_METABOLISM		10	-0.2946778	-0.7074904	0.76744187	0.9575265	1	975	tags=60%, list=58%, signal=140%
REACTOME_DEATH_RECEPTOR_SIGNALLING		11	-0.2840007	-0.7073472	0.9230769	0.95703596	1	513	tags=36%, list=30%, signal=52%
GOBP_RELEASE_OF_SEQUESTERED_CALCIUM_ION_INTO_CYTOSOL_BY_END		16	-0.2579973	-0.7072455	0.875	0.95656323	1	6	tags=6%, list=0%, signal=6%
GOBP_RESPONSE_TO_ETHANOL		18	-0.2454254	-0.7061204	0.86021507	0.9569961	1	793	tags=50%, list=47%, signal=93%

GOBP_METHYLATION		20	-0.2491165	-0.7020676	0.9111111	0.9598407	1	525	tags=35%, list=31%, signal=50%
GOBP_REGULATION_OF_LEUKOCYTE_MIGRATION		19	-0.2529179	-0.7003666	0.8854167	0.9607452	1	658	tags=47%, list=39%, signal=77%
GOBP_REGULATION_OF_CHEMOTAXIS		19	-0.2496105	-0.7001427	0.86170214	0.9603683	1	656	tags=42%, list=39%, signal=68%
GOBP_PROTEIN_MATURATION		52	-0.2171586	-0.6993788	0.959596	0.9603918	1	673	tags=38%, list=40%, signal=62%
GOBP_REGULATION_OF_HORMONE_LEVELS		57	-0.206575	-0.6991885	0.9183673	0.95995337	1	393	tags=25%, list=23%, signal=31%
GOBP_STEROID_BIOSYNTHETIC_PROCESS		27	-0.2327405	-0.6970735	0.8969072	0.96115136	1	988	tags=70%, list=58%, signal=166%
GOBP_POSITIVE_REGULATION_OF_MACROAUTOPHAGY		10	-0.3000278	-0.6970574	0.82978725	0.96061003	1	578	tags=50%, list=34%, signal=75%
GOBP_GLYCOPROTEIN_BIOSYNTHETIC_PROCESS		23	-0.2447542	-0.6968196	0.8924731	0.9602916	1	65	tags=9%, list=4%, signal=9%
GOBP_REGULATION_OF_FATTY_ACID_METABOLIC_PROCESS		20	-0.24684	-0.6961916	0.8804348	0.96017903	1	916	tags=55%, list=54%, signal=118%
GOBP_REGULATION_OF_LIPID_METABOLIC_PROCESS		59	-0.215301	-0.6956496	0.94	0.96004397	1	916	tags=53%, list=54%, signal=110%
REACTOME_THE_ROLE_OF_NEF_IN_HIV_1_REPLICATION_AND_DISEASE_PAT		10	-0.2992002	-0.6941758	0.9101124	0.96051794	1	1077	tags=80%, list=64%, signal=218%
GOBP_ION_HOMEOSTASIS		128	-0.1976925	-0.6927416	0.99	0.96107596	1	372	tags=19%, list=22%, signal=22%
GOBP_SPHINGOLIPID_METABOLIC_PROCESS		24	-0.2426063	-0.691672	0.86734694	0.9613541	1	934	tags=54%, list=55%, signal=119%
GOBP_AMYLOID_BETA_METABOLIC_PROCESS		13	-0.2680567	-0.6893191	0.8804348	0.96247864	1	475	tags=31%, list=28%, signal=42%
GOBP_REGULATION_OF_TRANSPORTER_ACTIVITY		58	-0.2062983	-0.6887796	0.86	0.9623364	1	659	tags=33%, list=39%, signal=52%
GOBP_HORMONE_TRANSPORT		35	-0.2166712	-0.6870498	0.9191919	0.96303356	1	393	tags=26%, list=23%, signal=33%
GOBP_ATP_BIOSYNTHETIC_PROCESS		25	-0.2333289	-0.6857574	0.8888889	0.9635713	1	209	tags=16%, list=12%, signal=18%
GOBP_PROTEIN_HOMOTETRAMERIZATION		10	-0.2957802	-0.6847869	0.9047619	0.9637168	1	209	tags=20%, list=12%, signal=23%
GOBP_RECEPTOR_LOCALIZATION_TO_SYNAPSE		10	-0.293939	-0.6816585	0.90361446	0.9653056	1	1078	tags=70%, list=64%, signal=191%
GOBP_GLYCOSPHINGOLIPID_METABOLIC_PROCESS		10	-0.2872299	-0.6807392	0.8965517	0.9654224	1	1076	tags=80%, list=63%, signal=218%
GOBP_REGULATION_OF_LIPASE_ACTIVITY		15	-0.2321028	-0.6787061	0.87234044	0.9661943	1	391	tags=20%, list=23%, signal=26%
GOBP_POSITIVE_REGULATION_OF_SODIUM_ION_TRANSPORT		13	-0.2648891	-0.6750059	0.8764045	0.96839404	1	1129	tags=77%, list=67%, signal=229%
GOBP_LIPID_MODIFICATION		58	-0.2048099	-0.6738219	0.96	0.96866095	1	992	tags=52%, list=59%, signal=120%
GOBP_REGULATION_OF_MUSCLE_SYSTEM_PROCESS		52	-0.2071059	-0.6710405	0.94	0.9700218	1	719	tags=35%, list=42%, signal=58%
GOBP_RESPONSE_TO_OXYGEN_RADICAL		10	-0.2729923	-0.6707995	0.94382024	0.9696333	1	683	tags=50%, list=40%, signal=83%
GOBP_KETONE_BIOSYNTHETIC_PROCESS		11	-0.2754199	-0.6706734	0.88297874	0.96914953	1	773	tags=36%, list=46%, signal=66%
GOBP_REGULATION_OF_ANION_TRANSMEMBRANE_TRANSPORT		14	-0.2478895	-0.670322	0.8791209	0.9687959	1	902	tags=57%, list=53%, signal=121%
GOBP_ORGANIC_HYDROXY_COMPOUND_BIOSYNTHETIC_PROCESS		28	-0.2220195	-0.6698266	0.8947368	0.96851534	1	988	tags=68%, list=58%, signal=160%
GOBP_CELLULAR_TRANSITION_METAL_ION_HOMEOSTASIS		24	-0.2336882	-0.6695553	0.9255319	0.9681232	1	123	tags=13%, list=7%, signal=13%
GOBP_MITOCHONDRIAL_CALCIUM_ION_HOMEOSTASIS		14	-0.2448508	-0.6693227	0.8901099	0.96774846	1	337	tags=21%, list=20%, signal=27%
GOBP_DENDRITIC_SPINE_DEVELOPMENT		10	-0.2769142	-0.6665757	0.90909094	0.9691043	1	1144	tags=80%, list=67%, signal=245%
GOBP_REGULATION_OF_MEMBRANE_POTENTIAL		62	-0.1995504	-0.666214	0.9292929	0.9687628	1	309	tags=18%, list=18%, signal=21%
GOBP_STEROID_METABOLIC_PROCESS		46	-0.2042194	-0.6658943	0.96907216	0.96836036	1	988	tags=61%, list=58%, signal=142%
GOBP_CELLULAR_OXIDANT_DETOXIFICATION		32	-0.2232437	-0.6652237	0.9263158	0.9683067	1	820	tags=53%, list=48%, signal=101%
GOBP_PHOSPHOLIPID_METABOLIC_PROCESS		55	-0.2032014	-0.6648473	0.96	0.96802294	1	495	tags=25%, list=29%, signal=35%
GOBP_LIPID_METABOLIC_PROCESS		222	-0.1831617	-0.6615185	1	0.96954775	1	916	tags=48%, list=54%, signal=91%
GOBP_CARBOHYDRATE_DERIVATIVE_BIOSYNTHETIC_PROCESS		102	-0.1992551	-0.6611905	0.99	0.9692349	1	345	tags=18%, list=20%, signal=21%
GOBP_DIVALENT_INORGANIC_CATION_HOMEOSTASIS		73	-0.195547	-0.6605213	0.95	0.9691071	1	372	tags=19%, list=22%, signal=24%
REACTOME_PEROXISOMAL_PROTEIN_IMPORT		24	-0.2314072	-0.6595467	0.8924731	0.9691691	1	912	tags=50%, list=54%, signal=107%
REACTOME_CARGO_CONCENTRATION_IN_THE_ER		14	-0.2516692	-0.6585629	0.90217394	0.96937716	1	658	tags=43%, list=39%, signal=69%
GOBP_INORGANIC_ANION_TRANSMEMBRANE_TRANSPORT		12	-0.2681014	-0.6579778	0.875	0.9691995	1	4	tags=8%, list=0%, signal=8%
GOBP_METAL_ION_HOMEOSTASIS		105	-0.1898383	-0.6552128	0.99	0.97045106	1	420	tags=21%, list=25%, signal=26%
GOBP_REGULATION_OF_NEURON_DIFFERENTIATION		12	-0.2617311	-0.6548137	0.9431818	0.97014976	1	51	tags=8%, list=3%, signal=9%
GOBP_POSITIVE_REGULATION_OF_SODIUM_ION_TRANSMEMBRANE_TRANSPO		10	-0.2738039	-0.6538783	0.9375	0.9701556	1	1129	tags=80%, list=67%, signal=238%
GOBP_INSULIN_SECRETION		25	-0.2253596	-0.6531162	0.94623655	0.9699887	1	366	tags=24%, list=22%, signal=30%
GOBP_POSITIVE_REGULATION_OF_AUTOPHAGY		16	-0.2379808	-0.6527183	0.9157895	0.96962756	1	578	tags=38%, list=34%, signal=56%

GOBP_NEGATIVE_REGULATION_OF_RESPONSE_TO_BIOTIC_STIMULUS	10	-0.2800033	-0.652415	0.90361446	0.9692215	1	601	tags=50%, list=35%, signal=77%
GOBP_CELLULAR_HORMONE_METABOLIC_PROCESS	13	-0.2557416	-0.6517757	0.9222222	0.96911687	1	967	tags=69%, list=57%, signal=160%
GOBP_REGULATION_OF_SYSTEM_PROCESS	100	-0.1875879	-0.6514342	0.97	0.96879584	1	402	tags=20%, list=24%, signal=25%
GOBP_NEGATIVE_REGULATION_OF_DEPHOSPHORYLATION	11	-0.2449397	-0.6500922	0.9230769	0.96894747	1	687	tags=45%, list=41%, signal=76%
GOBP_REGULATION_OF_NEURONAL_SYNAPTIC_PLASTICITY	10	-0.2655915	-0.6497129	0.8636364	0.9685768	1	820	tags=50%, list=48%, signal=96%
GOBP_RIBONUCLEOSIDE_TRIPHOSPHATE_BIOSYNTHETIC_PROCESS	26	-0.2214178	-0.6495091	0.9183673	0.968095	1	209	tags=15%, list=12%, signal=17%
GOBP_CELLULAR_IRON_ION_HOMEOSTASIS	17	-0.2370773	-0.6483197	0.8842105	0.96836865	1	809	tags=47%, list=48%, signal=89%
REACTOME_BASIGIN_INTERACTIONS	11	-0.269076	-0.6467218	0.90804595	0.9688353	1	861	tags=55%, list=51%, signal=110%
GOBP_SEQUESTERING_OF_CALCIUM_ION	26	-0.2088127	-0.6460424	0.92783505	0.96871465	1	418	tags=19%, list=25%, signal=25%
GOBP_LIPID_LOCALIZATION	74	-0.1933988	-0.6409166	0.99	0.9712189	1	640	tags=35%, list=38%, signal=54%
GOBP_VACUOLE_ORGANIZATION	30	-0.2075991	-0.6372619	0.93877554	0.972703	1	630	tags=37%, list=37%, signal=57%
GOBP_NEGATIVE_REGULATION_OF_CATION_TRANSMEMBRANE_TRANSPORT	19	-0.2125724	-0.6360014	0.9255319	0.9729459	1	927	tags=58%, list=55%, signal=126%
GOBP_ALCOHOL_BIOSYNTHETIC_PROCESS	22	-0.2294674	-0.6357652	0.9166667	0.972558	1	241	tags=18%, list=14%, signal=21%
GOBP_SULFUR_COMPOUND_METABOLIC_PROCESS	95	-0.1829528	-0.6305182	0.98	0.97503704	1	955	tags=51%, list=56%, signal=109%
GOBP_FATTY_ACID_TRANSPORT	27	-0.2170766	-0.6298927	0.9183673	0.9748359	1	640	tags=33%, list=38%, signal=53%
GOBP_ENSHEATHMENT_OF_NEURONS	19	-0.2271353	-0.6290855	0.9347826	0.9746625	1	1145	tags=79%, list=68%, signal=241%
GOBP_STEROL_METABOLIC_PROCESS	26	-0.2132024	-0.6285253	0.9139785	0.97434616	1	395	tags=23%, list=23%, signal=30%
GOBP_MITOCHONDRIAL_GENE_EXPRESSION	88	-0.1859051	-0.6258245	1	0.97508734	1	1027	tags=58%, list=61%, signal=139%
GOBP_ORGANELLE_FUSION	46	-0.1950858	-0.625346	0.96875	0.9747878	1	469	tags=24%, list=28%, signal=32%
GOBP_MONONUCLEAR_CELL_MIGRATION	17	-0.2204357	-0.6251226	0.8979592	0.97433966	1	852	tags=65%, list=50%, signal=129%
GOBP_MITOCHONDRIAL_TRANSLATION	82	-0.1852952	-0.6249359	0.99	0.97388643	1	899	tags=48%, list=53%, signal=96%
GOBP_MITOCHONDRIAL_FISSION	12	-0.2416907	-0.6239321	0.89361703	0.97380257	1	1031	tags=67%, list=61%, signal=169%
REACTOME_STIMULI_SENSING_CHANNELS	11	-0.2404226	-0.6234736	0.9101124	0.9735427	1	593	tags=45%, list=35%, signal=69%
GOBP_2_OXOGLUTARATE_METABOLIC_PROCESS	13	-0.2284878	-0.6219593	0.87777776	0.97388166	1	862	tags=38%, list=51%, signal=78%
GOBP_RESPONSE_TO_NUTRIENT	28	-0.205242	-0.6210285	0.96875	0.9738139	1	669	tags=39%, list=39%, signal=64%
GOBP_REGULATION_OF_BLOOD_CIRCULATION	57	-0.1901912	-0.6207242	0.96	0.97339994	1	660	tags=33%, list=39%, signal=53%
GOBP_POSITIVE_REGULATION_OF_MITOCHONDRION_ORGANIZATION	20	-0.2217187	-0.6161912	0.9270833	0.9750662	1	513	tags=30%, list=30%, signal=43%
GOBP_CELLULAR_ION_HOMEOSTASIS	110	-0.1785413	-0.6137227	1	0.97588605	1	619	tags=28%, list=37%, signal=42%
REACTOME_NCAM_SIGNALING_FOR_NEURITE_OUT_GROWTH	11	-0.249335	-0.6132276	0.94382024	0.9756095	1	836	tags=64%, list=49%, signal=125%
REACTOME_PEROXISOMAL_LIPID_METABOLISM	12	-0.2518379	-0.6123827	0.9120879	0.9754649	1	865	tags=50%, list=51%, signal=101%
GOBP_CALCIUM_ION_TRANSPORT	79	-0.1810387	-0.6121234	0.99	0.9750408	1	467	tags=23%, list=28%, signal=30%
GOBP_RESPONSE_TO_ACTIVITY	12	-0.2373478	-0.6120725	0.9318182	0.974519	1	1023	tags=67%, list=60%, signal=167%
GOBP_REGULATION_OF_CATION_TRANSMEMBRANE_TRANSPORT	72	-0.1802893	-0.609538	0.98	0.9753206	1	629	tags=29%, list=37%, signal=44%
GOBP_PROTEIN_TRANSMEMBRANE_TRANSPORT	26	-0.2079985	-0.6085547	0.94	0.975209	1	511	tags=23%, list=30%, signal=33%
GOBP_RETROGRADE_VESICLE_MEDIATED_TRANSPORT_GOLGI_TO_ENDOPLA	18	-0.2217464	-0.6070467	0.94623655	0.97536415	1	950	tags=56%, list=56%, signal=125%
GOBP_NUCLEOSIDE_TRIPHOSPHATE_METABOLIC_PROCESS	34	-0.2056057	-0.605988	0.9361702	0.9752859	1	209	tags=15%, list=12%, signal=16%
GOBP_ER_NUCLEUS_SIGNALING_PATHWAY	14	-0.2236995	-0.60563	0.93333334	0.97483104	1	602	tags=36%, list=36%, signal=55%
GOBP_SHORT_CHAIN_FATTY_ACID_METABOLIC_PROCESS	10	-0.2504726	-0.605307	0.9166667	0.9744742	1	135	tags=10%, list=8%, signal=11%
GOBP_REGULATION_OF_CALCIUM_ION_TRANSMEMBRANE_TRANSPORTER_AC	26	-0.1932089	-0.6025472	0.97	0.9752298	1	89	tags=8%, list=5%, signal=8%
GOBP_INTERLEUKIN_1_PRODUCTION	10	-0.2456513	-0.6022005	0.9310345	0.97487867	1	656	tags=40%, list=39%, signal=65%
GOBP_CALCIUM_ION_REGULATED_EXOCYTOSIS	10	-0.2409432	-0.6016104	0.98888886	0.97465366	1	354	tags=30%, list=21%, signal=38%
GOBP_SPHINGOLIPID_BIOSYNTHETIC_PROCESS	13	-0.2306912	-0.6014577	0.9673913	0.9741458	1	866	tags=46%, list=51%, signal=94%
GOBP_REGULATION_OF_LIPID_TRANSPORT	27	-0.2010227	-0.6005604	0.9591837	0.974046	1	946	tags=63%, list=56%, signal=140%
GOBP_MICROTUBULE_BASED_MOVEMENT	29	-0.2000694	-0.599762	0.93939394	0.9739134	1	515	tags=31%, list=30%, signal=44%
GOBP_REGULATION_OF_EPITHELIAL_CELL_DIFFERENTIATION	10	-0.2447771	-0.5972229	0.9302326	0.97463346	1	653	tags=40%, list=39%, signal=65%
GOBP_REGULATION_OF_STEROL_TRANSPORT	15	-0.2295053	-0.5935255	0.9347826	0.9757499	1	916	tags=67%, list=54%, signal=144%

GOBP_REGULATION_OF_HORMONE_SECRETION			29	-0.1923423	-0.590883	0.96	0.9763601	1	393	tags=24%, list=23%, signal=31%
KEGG_SNARE_INTERACTIONS_IN_VESICULAR_TRANSPORT			12	-0.2401269	-0.5899282	0.9285714	0.9762541	1	425	tags=25%, list=25%, signal=33%
GOBP_REGULATION_OF_REGULATED_SECRETORY_PATHWAY			16	-0.2161359	-0.5894106	0.9368421	0.9759204	1	362	tags=25%, list=21%, signal=31%
REACTOME_AMYLOID_FIBER_FORMATION			13	-0.2182107	-0.5856539	0.9468085	0.9767193	1	915	tags=46%, list=54%, signal=100%
GOBP_MAMMARY_GLAND_DEVELOPMENT			14	-0.2229025	-0.5809401	0.94186044	0.978134	1	221	tags=14%, list=13%, signal=16%
KEGG_DILATED_CARDIOMYOPATHY			30	-0.1925391	-0.5791968	0.9791667	0.97832996	1	456	tags=23%, list=27%, signal=31%
GOBP_POST_EMBRYONIC_DEVELOPMENT			10	-0.2211031	-0.5777491	0.94186044	0.9782934	1	213	tags=10%, list=13%, signal=11%
GOBP_NUCLEOSIDE_TRIPHOSPHATE_BIOSYNTHETIC_PROCESS			29	-0.1906721	-0.5770411	0.92783505	0.978003	1	209	tags=14%, list=12%, signal=15%
GOBP_FATTY_ACID_METABOLIC_PROCESS			111	-0.1696493	-0.5762343	0.99	0.97784245	1	992	tags=50%, list=59%, signal=114%
GOBP_NUCLEOSIDE_BISPHOSPHATE_METABOLIC_PROCESS			44	-0.1784139	-0.5755168	1	0.9775526	1	1057	tags=57%, list=62%, signal=147%
GOBP_VITAMIN_METABOLIC_PROCESS			19	-0.2119413	-0.5744916	0.9677419	0.97739184	1	248	tags=11%, list=15%, signal=12%
GOBP_POSITIVE_REGULATION_OF_EPITHELIAL_CELL_PROLIFERATION			10	-0.2450039	-0.5726125	0.954023	0.97748524	1	278	tags=20%, list=16%, signal=24%
GOBP_METAL_ION_TRANSPORT			86	-0.1613785	-0.566022	0.98	0.97946423	1	467	tags=21%, list=28%, signal=27%
KEGG_AMYOTROPHIC_LATERAL_SCLEROSIS_ALS			12	-0.228397	-0.5649979	0.95454544	0.979228	1	309	tags=17%, list=18%, signal=20%
GOBP_REGULATION_OF_PEPTIDE_HORMONE_SECRETION			26	-0.1897256	-0.5586729	0.9591837	0.9808963	1	393	tags=23%, list=23%, signal=30%
GOBP_COGNITION			18	-0.2030731	-0.5574476	0.97849464	0.9807082	1	12	tags=6%, list=1%, signal=6%
GOBP_ORGANIC_ACID_BIOSYNTHETIC_PROCESS			65	-0.1689507	-0.5560783	0.99	0.9805847	1	916	tags=49%, list=54%, signal=103%
GOBP_POSITIVE_REGULATION_OF_COLD_INDUCED_THERMOGENESIS			14	-0.2034981	-0.5546929	0.96842104	0.9804936	1	695	tags=36%, list=41%, signal=60%
GOBP_LIPOPROTEIN_METABOLIC_PROCESS			21	-0.1926564	-0.554063	0.9479167	0.98013395	1	1000	tags=62%, list=59%, signal=149%
GOBP_POSITIVE_REGULATION_OF_INSULIN_SECRETION			12	-0.2194612	-0.5518757	0.96511626	0.98020923	1	1078	tags=67%, list=64%, signal=182%
GOBP_PROTEIN_TETRAMERIZATION			14	-0.2097036	-0.5510652	0.9655172	0.97995764	1	209	tags=14%, list=12%, signal=16%
REACTOME_ION_CHANNEL_TRANSPORT			33	-0.1773216	-0.5504232	0.9680851	0.97958285	1	875	tags=48%, list=52%, signal=98%
GOBP_REGULATION_OF_MITOCHONDRION_ORGANIZATION			33	-0.1837643	-0.5502638	0.97959185	0.9790852	1	707	tags=39%, list=42%, signal=66%
GOBP_REGULATION_OF_CHOLESTEROL_METABOLIC_PROCESS			10	-0.2231071	-0.5493757	0.9655172	0.97880775	1	52	tags=10%, list=3%, signal=10%
REACTOME_FATTY_ACID_METABOLISM			62	-0.164004	-0.5478844	0.98	0.97879773	1	992	tags=50%, list=59%, signal=116%
GOBP_POSITIVE_REGULATION_OF_NIK_NF_KAPPAB_SIGNALING			11	-0.2248184	-0.5445389	0.9767442	0.9792165	1	347	tags=18%, list=20%, signal=23%
REACTOME_P75_NTR_RECEPTOR_MEDIATED_SIGNALLING			10	-0.2101955	-0.5361372	0.9777778	0.980857	1	513	tags=30%, list=30%, signal=43%
GOBP_CARDIAC_MUSCLE_CONTRACTION			33	-0.1727978	-0.5336483	0.96842104	0.98099625	1	842	tags=33%, list=50%, signal=65%
GOBP_NEGATIVE_REGULATION_OF_CALCIUM_ION_TRANSPORT			10	-0.223858	-0.5273052	0.978022	0.98211735	1	39	tags=10%, list=2%, signal=10%
GOBP_REGULATION_OF_STEROID_BIOSYNTHETIC_PROCESS			10	-0.2212143	-0.5263115	0.97590363	0.9817965	1	52	tags=10%, list=3%, signal=10%
GOBP_REGULATION_OF_NEUROTRANSMITTER_TRANSPORT			16	-0.1975031	-0.525372	0.9782609	0.98149693	1	640	tags=38%, list=38%, signal=60%
GOBP_RIBONUCLEOSIDE_TRIPHOSPHATE_METABOLIC_PROCESS			31	-0.1757521	-0.5247075	0.989899	0.9810809	1	209	tags=13%, list=12%, signal=14%
GOBP_ACTIVATION_OF_CYSTEINE_TYPE_ENDOPEPTIDASE_ACTIVITY_INVOLV			12	-0.2084884	-0.5219467	0.96666664	0.98121375	1	1136	tags=75%, list=67%, signal=226%
REACTOME_TP53_REGULATES_METABOLIC_GENES			36	-0.1734866	-0.5198454	0.99	0.9811441	1	539	tags=28%, list=32%, signal=40%
REACTOME_INTRINSIC_PATHWAY_FOR_APOPTOSIS			10	-0.2127286	-0.5110523	0.96875	0.98255455	1	690	tags=50%, list=41%, signal=84%
GOBP_POSITIVE_REGULATION_OF_TRANSMEMBRANE_TRANSPORT			32	-0.1677691	-0.5084928	0.99	0.9826125	1	372	tags=19%, list=22%, signal=24%
REACTOME_PHOSPHOLIPID_METABOLISM			29	-0.1636228	-0.5001446	0.9895833	0.98381734	1	225	tags=10%, list=13%, signal=12%
GOBP_REGULATION_OF_CARDIAC_MUSCLE_CONTRACTION			18	-0.1869152	-0.4972494	0.9893617	0.9838577	1	39	tags=6%, list=2%, signal=6%
GOBP_THIOESTER_METABOLIC_PROCESS			42	-0.1622506	-0.4930485	0.99	0.9839613	1	1057	tags=55%, list=62%, signal=142%
REACTOME_METABOLISM_OF_LIPIDS			127	-0.144823	-0.4895253	1	0.9840124	1	992	tags=50%, list=59%, signal=112%
GOBP_LIPID_BIOSYNTHETIC_PROCESS			107	-0.1422936	-0.4870013	1	0.9838566	1	941	tags=49%, list=56%, signal=102%
KEGG_VALINE_LEUCINE_AND_ISOLEUCINE_DEGRADATION			33	-0.1618354	-0.480357	0.97938144	0.98432046	1	975	tags=39%, list=58%, signal=91%
GOBP_REGULATION_OF_TISSUE_REMODELING			10	-0.1947021	-0.4788093	0.97619045	0.9840114	1	946	tags=60%, list=56%, signal=135%
GOBP_EMBRYONIC_ORGAN_DEVELOPMENT			12	-0.1873277	-0.4714106	1	0.9845232	1	1177	tags=67%, list=69%, signal=217%
GOBP_ADAPTIVE_THERMOGENESIS			20	-0.1626402	-0.4588649	0.9893617	0.98548394	1	695	tags=30%, list=41%, signal=50%
GOBP_REGULATION_OF_METAL_ION_TRANSPORT			50	-0.1401834	-0.4581899	1	0.9849895	1	719	tags=32%, list=42%, signal=54%

GOBP_NUCLEOSIDE_PHOSPHATE_BIOSYNTHETIC_PROCESS		74	-0.1373154	-0.4578731	1	0.98447454	1	951	tags=47%, list=56%, signal=103%
KEGG_ABC_TRANSPORTERS		11	-0.1788611	-0.4538098	1	0.98440945	1	940	tags=55%, list=55%, signal=122%
GOBP_REGULATION_OF_MUSCLE_CONTRACTION		39	-0.1398217	-0.4502909	1	0.9842596	1	688	tags=28%, list=41%, signal=46%
GOBP_MONOCARBOXYLIC_ACID_BIOSYNTHETIC_PROCESS		48	-0.1404237	-0.4475353	1	0.98395735	1	941	tags=52%, list=56%, signal=114%
GOBP_REGULATION_OF_MITOCHONDRIAL_OUTER_MEMBRANE_PERMEABILIZ		11	-0.1698027	-0.4452821	1	0.9836177	1	989	tags=64%, list=58%, signal=152%
KEGG_PEROXISOME		32	-0.1477046	-0.4446307	0.9895833	0.98312604	1	912	tags=38%, list=54%, signal=80%
GOBP_POSITIVE_REGULATION_OF_MEMBRANE_PERMEABILITY		15	-0.1650499	-0.4300081	1	0.9837097	1	513	tags=27%, list=30%, signal=38%
GOBP_PURINE_CONTAINING_COMPOUND_BIOSYNTHETIC_PROCESS		66	-0.1215911	-0.4067687	1	0.9844189	1	237	tags=11%, list=14%, signal=12%
GOBP_CELL_COMMUNICATION_INVOLVED_IN_CARDIAC_CONDUCTION		16	-0.1413755	-0.4044142	1	0.9839694	1	366	tags=13%, list=22%, signal=16%
GOBP_REGULATION_OF_FATTY_ACID_BIOSYNTHETIC_PROCESS		12	-0.1549126	-0.3805662	0.9883721	0.9841671	1	916	tags=50%, list=54%, signal=108%

Supplementary Table 7f. GSEA results of the consensus MAM proteins in heart samples

GS<br>follow link to MSigDB	SIZE	ES	NES	NOM p-val	FDR q-val	FWER p-val	RANK AT MAX	LEADING EDGE		
GOBP_POSITIVE_REGULATION_OF_CELLULAR_PROTEIN_LOCA	10	-0.7765363	-1.9745874	0.0001	0.00605686	0.02	51	tags=100%,	list=27%,	signal=130%
GOBP_REGULATION_OF_CELLULAR_LOCALIZATION	15	-0.7050968	-1.867319	0.0001	0.02295208	0.1	65	tags=93%,	list=34%,	signal=131%
GOBP_REGULATION_OF_CELLULAR_PROTEIN_LOCALIZATION	14	-0.727508	-1.8262976	0.0001	0.03119494	0.15	51	tags=86%,	list=27%,	signal=109%
GOBP_POSITIVE_REGULATION_OF_GENE_EXPRESSION	18	-0.6840641	-1.8218657	0.0001	0.03058553	0.18	47	tags=72%,	list=25%,	signal=87%
GOBP_PROTEIN_LOCALIZATION_TO_MEMBRANE	31	-0.593526	-1.753968	0.0001	0.06795135	0.42	58	tags=65%,	list=31%,	signal=78%
GOBP_REGULATION_OF_PROTEIN_LOCALIZATION	23	-0.6048293	-1.742857	0.0001	0.06404139	0.45	51	tags=61%,	list=27%,	signal=73%
REACTOME_NERVOUS_SYSTEM_DEVELOPMENT	31	-0.6133353	-1.7326982	0.0001	0.06912897	0.52	61	tags=68%,	list=32%,	signal=84%
GOBP_MACROMOLECULE_CATABOLIC_PROCESS	43	-0.5800871	-1.7309667	0.0001	0.063147	0.52	64	tags=63%,	list=34%,	signal=73%
GOBP_CELL_MORPHOGENESIS	18	-0.6349624	-1.7295144	0.01162791	0.05740636	0.52	44	tags=67%,	list=23%,	signal=79%
REACTOME_DEVELOPMENTAL_BIOLOGY	31	-0.6133353	-1.7271109	0.0001	0.0526225	0.52	61	tags=68%,	list=32%,	signal=84%
GOBP_MITOCHONDRIAL_TRANSMEMBRANE_TRANSPORT	14	0.40863025	1.7378658	0.0001	0.04372472	0.25	12	tags=29%,	list=6%,	signal=28%
REACTOME_MITOCHONDRIAL_BIOGENESIS	11	0.47568184	1.748053	0.0001	0.04246624	0.22	33	tags=55%,	list=17%,	signal=62%
GOBP_TRANSMEMBRANE_TRANSPORT	43	0.32956558	1.8282999	0.0001	0.02919591	0.13	19	tags=35%,	list=10%,	signal=30%
REACTOME_RESPIRATORY_ELECTRON_TRANSPORT_ATP_SYNT	29	0.38582534	1.8558328	0.0001	0.02800744	0.11	26	tags=31%,	list=14%,	signal=30%
KEGG_ALZHEIMERS_DISEASE	30	0.382357	1.9436026	0.0001	0.01068665	0.04	20	tags=30%,	list=11%,	signal=28%
REACTOME_MITOCHONDRIAL_PROTEIN_IMPORT	10	0.5954829	1.9697438	0.0001	0.01246776	0.04	11	tags=40%,	list=6%,	signal=40%
KEGG_OXIDATIVE_PHOSPHORYLATION	26	0.40024167	1.9910747	0.0001	0.01496131	0.04	34	tags=42%,	list=18%,	signal=44%
GOBP_MITOCHONDRIAL_TRANSPORT	19	0.4402359	2.1773381	0.0001	0	0	12	tags=32%,	list=6%,	signal=30%
KEGG_HUNTINGTONS_DISEASE	33	0.4297726	2.2142997	0.0001	0	0	20	tags=33%,	list=11%,	signal=31%
KEGG_PARKINSONS_DISEASE	30	0.50324994	2.5427394	0.0001	0	0	20	tags=37%,	list=11%,	signal=34%

Supplementary Table 7g. GSEA results of the consensus MAM proteins in GA muslce samples

GS<br>follow link to MSigDB	SIZE	ES	NES	NOM p-val	FDR q-val	FWER p-val	RANK AT MAX	LEADING EDGE		
GOBP_CYTOSKELETON_ORGANIZATION	20	-0.6137304	-2.2091665	0	0	0	77	tags=90%,	list=41%,	signal=136%
GOBP_POSITIVE_REGULATION_OF_GENE_EXPRESSION	18	-0.6840641	-2.053529	0	0	0	47	tags=72%,	list=25%,	signal=87%
REACTOME_INNATE_IMMUNE_SYSTEM	28	-0.5957114	-2.0021996	0	0	0	74	tags=79%,	list=39%,	signal=110%
GOBP_RNA_PROCESSING	10	-0.6877635	-1.992478	0	0	0	57	tags=90%,	list=30%,	signal=122%
GOBP_POSITIVE_REGULATION_OF_PROTEIN_METABOLIC_PRO	30	-0.5357416	-1.9203359	0	0.00370807	0.1	64	tags=57%,	list=34%,	signal=72%
GOBP_CELLULAR_MACROMOLECULE_CATABOLIC_PROCESS	38	-0.5497288	-1.9051716	0	0.00355975	0.1	75	tags=68%,	list=40%,	signal=91%
GOBP_REGULATION_OF_PROTEIN_LOCALIZATION	23	-0.6048293	-1.8853871	0	0.00342284	0.1	51	tags=61%,	list=27%,	signal=73%
REACTOME_DEVELOPMENTAL_BIOLOGY	31	-0.6133353	-1.8715467	0	0.00329606	0.1	61	tags=68%,	list=32%,	signal=84%
GOBP_REGULATION_OF_CELLULAR_PROTEIN_LOCALIZATION	14	-0.727508	-1.8370565	0	0.00637681	0.2	51	tags=86%,	list=27%,	signal=109%
GOBP_MYELOID_LEUKOCYTE_MEDIATED_IMMUNITY	20	-0.5810301	-1.8108518	0	0.00615692	0.2	64	tags=70%,	list=34%,	signal=95%
GOBP_NADH_DEHYDROGENASE_COMPLEX_ASSEMBLY	12	0.3163842	1.6076403	0	0	0	132	tags=100%,	list=70%,	signal=311%
GOBP_GLYCEROLIPID_METABOLIC_PROCESS	10	0.3211047	1.6422205	0	0	0	14	tags=20%,	list=7%,	signal=20%
REACTOME_MITOCHONDRIAL_PROTEIN_IMPORT	10	0.5954829	1.674459	0	0	0	11	tags=40%,	list=6%,	signal=40%
GOBP_PROTON_TRANSMEMBRANE_TRANSPORT	17	0.39274812	1.7758845	0	0	0	19	tags=47%,	list=10%,	signal=48%
GOBP_TRANSMEMBRANE_TRANSPORT	43	0.32956558	1.7820942	0	0	0	19	tags=35%,	list=10%,	signal=30%
REACTOME_RESPIRATORY_ELECTRON_TRANSPORT_ATP_SYNT	29	0.38582534	1.8549207	0	0	0	26	tags=31%,	list=14%,	signal=30%
REACTOME_PROTEIN_LOCALIZATION	14	0.47437775	2.0827785	0	0	0	11	tags=29%,	list=6%,	signal=28%
GOBP_MITOCHONDRIAL_TRANSMEMBRANE_TRANSPORT	14	0.40863025	2.2346964	0	0	0	12	tags=29%,	list=6%,	signal=28%
GOBP_MITOCHONDRIAL_TRANSPORT	19	0.4402359	2.3526711	0	0	0	12	tags=32%,	list=6%,	signal=30%
KEGG_PARKINSONS_DISEASE	30	0.50324994	2.5416343	0	0	0	20	tags=37%,	list=11%,	signal=34%
