# Supplementary material for: Ultrastructural and proteomic profiling of mitochondria-associated endoplasmic reticulum membranes reveal aging signatures in striated muscle
Source: Cell Death Dis. 2022 Apr 2;13(4):296. doi: 10.1038/s41419-022-04746-4 (PMC8976840; doi:10.1038/s41419-022-04746-4)
Supplement: Supplementary file 10 — Supplementary Table 8. STRING interaction network. [file 41419_2022_4746_MOESM10_ESM.pdf]

Supplementary Table 8a. STRING interactions

#Node1	Node2	Node1_String_Id	Node2_String_Id	Neighborhood_On_Chromosome	Gene_Fusion	Phylogenetic_Cooccurrence	Homology	Coexpression	Experimentally_Determined_Interaction	Database_Annotated	Automated_Textmining	Combined_Score
Acaa2	Acads	10116.ENSRNC	10116.ENSRNC	0.068	0	0	0	0.296	0.513	0.914	0.755	0.992
Acaa2	Ehhadh	10116.ENSRNC	10116.ENSRNC	0.068	0.032	0	0	0.192	0.284	0.99	0.759	0.998
Acaa2	Decr1	10116.ENSRNC	10116.ENSRNC	0.045	0	0	0	0.687	0.507	0.156	0.473	0.922
Acaa2	Acadm	10116.ENSRNC	10116.ENSRNC	0.068	0	0	0	0.792	0.511	0.914	0.786	0.997
Acaa2	Hadh	10116.ENSRNC	10116.ENSRNC	0.09	0.024	0.21	0	0.474	0.424	0.994	0.676	0.999
Acaa2	Aldh6a1	10116.ENSRNC	10116.ENSRNC	0	0	0	0	0.23	0	0.9	0.054	0.92
Acaa2	Acadl	10116.ENSRNC	10116.ENSRNC	0.068	0	0	0	0.458	0.512	0.914	0.648	0.991
Acaa2	Hsd17b4	10116.ENSRNC	10116.ENSRNC	0.09	0	0	0	0.374	0.316	0.911	0.39	0.974
Acaa2	Pccb	10116.ENSRNC	10116.ENSRNC	0.09	0	0	0	0.185	0	0.917	0.167	0.942
Acaa2	Acadvl	10116.ENSRNC	10116.ENSRNC	0.068	0	0	0	0.317	0.508	0.914	0.719	0.991
Acaa2	ENSRNOG0000	10116.ENSRNC	10116.ENSRNC	0.068	0	0	0	0.586	0.435	0.18	0.666	0.929
Acaa2	Ech1	10116.ENSRNC	10116.ENSRNC	0	0	0	0	0.575	0.502	0	0.574	0.902
Acaa2	Mecr	10116.ENSRNC	10116.ENSRNC	0.045	0	0	0	0.084	0	0.9	0.226	0.923
Acaa2	Cs	10116.ENSRNC	10116.ENSRNC	0.076	0	0	0	0.102	0.055	0.962	0.502	0.982
Acaa2	Hadha	10116.ENSRNC	10116.ENSRNC	0.068	0.016	0	0	0.463	0.505	0.981	0.849	0.999
Acaa2	Hsd17b10	10116.ENSRNC	10116.ENSRNC	0	0.001	0	0	0.227	0.133	0.994	0.573	0.998
Acaa2	Acox1	10116.ENSRNC	10116.ENSRNC	0.068	0	0	0	0.223	0.09	0.965	0.59	0.989
Acaa2	Oxct1	10116.ENSRNC	10116.ENSRNC	0.1	0.164	0	0	0.075	0	0.849	0.491	0.937
Acadl	Ehhadh	10116.ENSRNC	10116.ENSRNC	0.052	0	0	0	0.149	0.193	0.922	0.645	0.978
Acadl	Decr1	10116.ENSRNC	10116.ENSRNC	0.044	0	0	0	0.427	0.51	0.145	0.635	0.901
Acadl	Acadm	10116.ENSRNC	10116.ENSRNC	0	0	0.416	0.797	0.606	0.51	0.8	0.852	0.968
Acadl	Bckdhb	10116.ENSRNC	10116.ENSRNC	0.043	0	0	0	0.48	0.057	0.827	0.342	0.936
Acadl	Acsl1	10116.ENSRNC	10116.ENSRNC	0.066	0	0	0	0.17	0.123	0.91	0.598	0.97
Acadl	Hadh	10116.ENSRNC	10116.ENSRNC	0.084	0.002	0.248	0	0.404	0.505	0.201	0.654	0.93
Acadl	Cpt2	10116.ENSRNC	10116.ENSRNC	0	0	0	0	0.264	0.512	0.9	0.728	0.988
Acadl	Dbt	10116.ENSRNC	10116.ENSRNC	0.045	0	0	0	0.549	0.129	0.738	0.333	0.922
Acadl	Mccc1	10116.ENSRNC	10116.ENSRNC	0.084	0	0	0	0.559	0.104	0.74	0.299	0.922
Acadl	Bckdha	10116.ENSRNC	10116.ENSRNC	0.044	0.025	0	0	0.55	0	0.807	0.226	0.927
Acadl	Mccc2	10116.ENSRNC	10116.ENSRNC	0.084	0.001	0	0	0.58	0	0.74	0.382	0.93
Acadl	Pccb	10116.ENSRNC	10116.ENSRNC	0.084	0	0	0	0.586	0	0.74	0.474	0.941
Acadl	Acox1	10116.ENSRNC	10116.ENSRNC	0	0	0	0	0.104	0	0.8	0.714	0.944
Acadl	Acadvl	10116.ENSRNC	10116.ENSRNC	0	0	0.356	0.711	0.347	0.512	0.8	0.785	0.951
Acadl	Hadha	10116.ENSRNC	10116.ENSRNC	0.052	0	0	0	0.179	0.508	0.922	0.697	0.989
Acadl	ENSRNOG0000	10116.ENSRNC	10116.ENSRNC	0.052	0	0	0	0.158	0.164	0.982	0.498	0.992
Acadm	Acads	10116.ENSRNC	10116.ENSRNC	0	0	0.444	0.871	0.171	0.51	0.8	0.822	0.925
Acadm	Ehhadh	10116.ENSRNC	10116.ENSRNC	0.052	0	0	0	0.225	0.193	0.964	0.684	0.992
Acadm	Gcdh	10116.ENSRNC	10116.ENSRNC	0	0	0.306	0.676	0.332	0	0.9	0.515	0.946
Acadm	Decr1	10116.ENSRNC	10116.ENSRNC	0.044	0	0	0	0.701	0.507	0.145	0.493	0.928
Acadm	Acsl1	10116.ENSRNC	10116.ENSRNC	0.066	0	0	0	0.578	0.123	0.452	0.643	0.92
Acadm	Acsf3	10116.ENSRNC	10116.ENSRNC	0.074	0	0	0	0.27	0	0.67	0.714	0.927
Acadm	Mccc2	10116.ENSRNC	10116.ENSRNC	0.084	0.001	0	0	0.203	0	0.916	0.153	0.941
Acadm	Hadh	10116.ENSRNC	10116.ENSRNC	0.084	0	0.369	0	0.403	0.504	0.201	0.66	0.942
Acadm	Mccc1	10116.ENSRNC	10116.ENSRNC	0.084	0	0	0	0.2	0.062	0.916	0.19	0.945
Acadm	Ech1	10116.ENSRNC	10116.ENSRNC	0	0	0	0	0.451	0.502	0.67	0.537	0.952
Acadm	Acadvl	10116.ENSRNC	10116.ENSRNC	0	0	0.431	0.756	0.398	0.508	0.8	0.812	0.953
Acadm	Acox1	10116.ENSRNC	10116.ENSRNC	0	0	0	0	0.208	0	0.8	0.796	0.964
Acadm	Dbt	10116.ENSRNC	10116.ENSRNC	0.045	0	0	0	0.12	0.129	0.972	0.477	0.987
Acadm	Cpt2	10116.ENSRNC	10116.ENSRNC	0	0	0	0	0.251	0.505	0.9	0.772	0.99
Acadm	ENSRNOG0000	10116.ENSRNC	10116.ENSRNC	0.052	0	0	0	0.279	0.436	0.965	0.524	0.992
Acadm	Hadha	10116.ENSRNC	10116.ENSRNC	0.052	0	0	0	0.37	0.504	0.99	0.65	0.998
Acads	Gcdh	10116.ENSRNC	10116.ENSRNC	0	0	0.322	0.769	0.165	0	0.9	0.641	0.93
Acads	Acsf3	10116.ENSRNC	10116.ENSRNC	0.074	0	0	0	0.118	0	0.771	0.727	0.942
Acads	Ech1	10116.ENSRNC	10116.ENSRNC	0	0	0	0	0.266	0.505	0.756	0.429	0.942

Acads	Pccb	10116.ENSRNO	10116.ENSRNO	0.084	0	0	0	0.182	0	0.916	0.241	0.946
Acads	Aldh6a1	10116.ENSRNO	10116.ENSRNO	0	0	0	0	0.589	0.07	0.9	0.318	0.97
Acads	Dbt	10116.ENSRNO	10116.ENSRNO	0.045	0	0	0	0.097	0.129	0.974	0.088	0.979
Acads	Hadh	10116.ENSRNO	10116.ENSRNO	0.084	0.003	0.372	0	0.609	0.133	0.833	0.665	0.986
Acads	Ehhadh	10116.ENSRNO	10116.ENSRNO	0.052	0	0	0	0.156	0.18	0.965	0.612	0.989
Acads	Hadha	10116.ENSRNO	10116.ENSRNO	0.052	0	0	0	0.186	0.507	0.979	0.702	0.997
Acads	ENSRNOG0000	10116.ENSRNO	10116.ENSRNO	0.052	0	0.247	0	0.594	0.435	0.968	0.642	0.997
Acadvl	Ehhadh	10116.ENSRNO	10116.ENSRNO	0.052	0	0	0	0.168	0.487	0.965	0.567	0.992
Acadvl	Hadh	10116.ENSRNO	10116.ENSRNO	0.084	0	0.352	0	0.184	0.504	0.201	0.717	0.933
Acadvl	Cpt2	10116.ENSRNO	10116.ENSRNO	0	0	0	0	0.284	0.508	0.9	0.789	0.991
Acadvl	Ech1	10116.ENSRNO	10116.ENSRNO	0	0	0	0	0.599	0.504	0.214	0.461	0.904
Acadvl	Acox1	10116.ENSRNO	10116.ENSRNO	0	0	0	0	0.101	0	0.8	0.723	0.945
Acadvl	ENSRNOG0000	10116.ENSRNO	10116.ENSRNO	0.052	0	0	0	0.14	0.402	0.939	0.586	0.985
Acadvl	Hadha	10116.ENSRNO	10116.ENSRNO	0.052	0	0	0	0.255	0.79	0.99	0.737	0.999
Aco2	Mdh2	10116.ENSRNO	10116.ENSRNO	0.084	0	0	0	0.572	0.8	0	0.886	0.989
Aco2	Atp5b	10116.ENSRNO	10116.ENSRNO	0.044	0	0	0	0.629	0.054	0	0.784	0.918
Aco2	Dlst	10116.ENSRNO	10116.ENSRNO	0.08	0	0	0	0.589	0.497	0.154	0.786	0.959
Aco2	Pdhb	10116.ENSRNO	10116.ENSRNO	0.044	0	0	0	0.679	0.497	0	0.754	0.957
Aco2	Sdhb	10116.ENSRNO	10116.ENSRNO	0.089	0	0	0	0.469	0.696	0	0.83	0.971
Aco2	Ndufs1	10116.ENSRNO	10116.ENSRNO	0.103	0	0	0	0.596	0.46	0	0.645	0.921
Aco2	Sdha	10116.ENSRNO	10116.ENSRNO	0.087	0	0	0	0.667	0.453	0.211	0.782	0.966
Aco2	Idh2	10116.ENSRNO	10116.ENSRNO	0.103	0	0	0	0.215	0.054	0.99	0.793	0.998
Aco2	Uqcrfs1	10116.ENSRNO	10116.ENSRNO	0	0	0	0	0.705	0.462	0	0.437	0.903
Aco2	Cs	10116.ENSRNO	10116.ENSRNO	0.12	0	0	0	0.584	0.803	0.994	0.849	0.999
Acot2	Tecr	10116.ENSRNO	10116.ENSRNO	0	0	0	0	0.062	0	0.9	0	0.902
Acox1	Ehhadh	10116.ENSRNO	10116.ENSRNO	0.052	0	0	0	0.445	0.507	0.99	0.82	0.999
Acox1	Gcdh	10116.ENSRNO	10116.ENSRNO	0	0	0	0	0.135	0	0.9	0.404	0.943
Acox1	Decr1	10116.ENSRNO	10116.ENSRNO	0.044	0	0	0	0.275	0.163	0.713	0.565	0.914
Acox1	Acsl1	10116.ENSRNO	10116.ENSRNO	0.066	0	0	0	0.419	0.258	0.91	0.733	0.988
Acox1	Hadh	10116.ENSRNO	10116.ENSRNO	0.084	0	0	0	0.156	0.133	0.833	0.559	0.941
Acox1	Scp2	10116.ENSRNO	10116.ENSRNO	0	0	0	0	0.472	0.693	0.8	0.653	0.987
Acox1	Aldh6a1	10116.ENSRNO	10116.ENSRNO	0	0	0	0	0.235	0.07	0.9	0.14	0.93
Acox1	Cpt2	10116.ENSRNO	10116.ENSRNO	0	0	0	0	0.167	0.139	0.9	0.808	0.984
Acox1	Dbt	10116.ENSRNO	10116.ENSRNO	0.045	0	0	0	0.086	0.129	0.915	0.088	0.93
Acox1	Hsd17b4	10116.ENSRNO	10116.ENSRNO	0.084	0	0	0	0.3	0.507	0.859	0.74	0.986
Acox1	Pccb	10116.ENSRNO	10116.ENSRNO	0.084	0	0	0	0.118	0	0.916	0.153	0.935
Acox1	Eci2	10116.ENSRNO	10116.ENSRNO	0.118	0	0	0	0.246	0.492	0.771	0.664	0.969
Acox1	ENSRNOG0000	10116.ENSRNO	10116.ENSRNO	0.052	0	0	0	0.157	0.164	0.965	0.286	0.98
Acox1	Ech1	10116.ENSRNO	10116.ENSRNO	0	0	0	0	0.234	0.5	0.759	0.592	0.957
Acox1	Hadha	10116.ENSRNO	10116.ENSRNO	0.052	0	0	0	0.172	0.234	0.971	0.654	0.993
Acsf3	Mlycd	10116.ENSRNO	10116.ENSRNO	0.044	0.292	0	0	0.081	0	0.8	0.355	0.905
Acsf3	Aldh7a1	10116.ENSRNO	10116.ENSRNO	0.068	0	0	0	0.059	0.517	0.942	0.128	0.974
Actn1	Vcl	10116.ENSRNO	10116.ENSRNO	0	0	0	0	0.132	0.86	0.924	0.74	0.997
Actn1	Tln1	10116.ENSRNO	10116.ENSRNO	0	0	0	0	0.064	0.503	0.637	0.516	0.907
Actn1	Actn4	10116.ENSRNO	10116.ENSRNO	0	0	0	0.984	0.12	0.935	0.924	0.825	0.995
Actn1	Ldb3	10116.ENSRNO	10116.ENSRNO	0	0	0	0	0.546	0.703	0.312	0.426	0.939
Actn2	Vcl	10116.ENSRNO	10116.ENSRNO	0	0	0	0	0.096	0.545	0.637	0.457	0.908
Actn2	Tpm4	10116.ENSRNO	10116.ENSRNO	0	0	0	0	0.461	0.447	0.6	0.278	0.902
Actn2	Actn4	10116.ENSRNO	10116.ENSRNO	0	0	0	0.98	0	0.734	0.637	0.636	0.9
Actn2	Myh7	10116.ENSRNO	10116.ENSRNO	0	0	0	0	0.492	0.615	0	0.64	0.923
Actn2	Tpm1	10116.ENSRNO	10116.ENSRNO	0	0	0	0	0.533	0.486	0.6	0.606	0.957
Actn2	Ldb3	10116.ENSRNO	10116.ENSRNO	0	0	0	0	0.563	0.703	0.312	0.787	0.978
Actn4	Vcl	10116.ENSRNO	10116.ENSRNO	0	0	0	0	0.096	0.696	0.924	0.67	0.992
Actn4	Ldb3	10116.ENSRNO	10116.ENSRNO	0	0	0	0	0.546	0.703	0.312	0.426	0.939
Actr3	Arpc1b	10116.ENSRNO	10116.ENSRNO	0	0	0	0	0.418	0.928	0.959	0.701	0.999
Adck3	Coq5	10116.ENSRNO	10116.ENSRNO	0	0	0	0	0.108	0.533	0	0.835	0.925
Adck3	Coq3	10116.ENSRNO	10116.ENSRNO	0	0	0	0	0.087	0.375	0.34	0.853	0.937

Adck3	Coq6	10116.ENSRNC	10116.ENSRNC	0	0	0	0	0.064	0.363	0	0.872	0.917
Adck3	Coq9	10116.ENSRNC	10116.ENSRNC	0.053	0	0	0	0.125	0.507	0	0.884	0.946
Afg3l2	Phb2	10116.ENSRNC	10116.ENSRNC	0	0	0	0	0.377	0.425	0.6	0.631	0.94
Afg3l2	Pmpca	10116.ENSRNC	10116.ENSRNC	0.043	0	0	0	0.189	0.082	0.9	0.524	0.959
Agk	Mgll	10116.ENSRNC	10116.ENSRNC	0.091	0	0	0	0	0	0.9	0.24	0.924
Agl	Pgm1	10116.ENSRNC	10116.ENSRNC	0	0	0	0	0.594	0	0.9	0.326	0.97
Agl	Pygm	10116.ENSRNC	10116.ENSRNC	0.12	0	0	0	0.671	0	0.981	0.673	0.997
Agl	Ugp2	10116.ENSRNC	10116.ENSRNC	0	0	0	0	0.208	0	0.9	0.508	0.957
Ak3	Nt5e	10116.ENSRNC	10116.ENSRNC	0	0	0	0	0	0	0.9	0.044	0.9
Alb	Fn1	10116.ENSRNC	10116.ENSRNC	0	0	0	0	0.06	0	0	0.931	0.933
Alb	Hpx	10116.ENSRNC	10116.ENSRNC	0	0	0	0	0.508	0.5	0	0.908	0.975
Aldh6a1	Ehhadh	10116.ENSRNC	10116.ENSRNC	0	0	0	0	0.197	0.144	0.9	0.365	0.95
Aldh6a1	Hibadh	10116.ENSRNC	10116.ENSRNC	0	0	0.239	0	0.865	0.485	0.994	0.746	0.999
Aldh6a1	Hadh	10116.ENSRNC	10116.ENSRNC	0	0.006	0	0	0.629	0	0.9	0.231	0.969
Aldh6a1	Mlycd	10116.ENSRNC	10116.ENSRNC	0	0	0	0	0.102	0	0.9	0.054	0.907
Aldh6a1	Hibch	10116.ENSRNC	10116.ENSRNC	0	0	0	0	0.508	0.521	0	0.798	0.948
Aldh6a1	Dbt	10116.ENSRNC	10116.ENSRNC	0	0	0	0	0.171	0	0.9	0.503	0.955
Aldh6a1	Aldh7a1	10116.ENSRNC	10116.ENSRNC	0	0	0	0.676	0.104	0.814	0.9	0.774	0.986
Aldh6a1	Pccb	10116.ENSRNC	10116.ENSRNC	0	0	0	0	0.509	0.287	0.99	0.584	0.998
Aldh7a1	Hibadh	10116.ENSRNC	10116.ENSRNC	0.083	0	0	0	0.135	0.201	0.915	0.388	0.961
Aldh7a1	Glud1	10116.ENSRNC	10116.ENSRNC	0.075	0	0	0	0.173	0.079	0.8	0.437	0.906
Aldh7a1	Maoa	10116.ENSRNC	10116.ENSRNC	0	0	0	0	0	0	0.967	0.284	0.975
Aldoa	Pdhb	10116.ENSRNC	10116.ENSRNC	0.044	0	0	0	0.077	0	0.845	0.381	0.904
Aldoa	Pfkm	10116.ENSRNC	10116.ENSRNC	0	0	0	0	0.213	0	0.879	0.666	0.965
Aldoa	Ldha	10116.ENSRNC	10116.ENSRNC	0	0	0	0	0.156	0.129	0.6	0.737	0.912
Aldoa	LOC500959	10116.ENSRNC	10116.ENSRNC	0	0	0	0	0.195	0	0.939	0.728	0.985
Aldoa	Eno1	10116.ENSRNC	10116.ENSRNC	0	0	0	0	0.343	0.162	0.646	0.74	0.942
Aldoa	Gpi	10116.ENSRNC	10116.ENSRNC	0	0	0	0	0.786	0.311	0.863	0.565	0.99
Aldoa	Gapdh	10116.ENSRNC	10116.ENSRNC	0.044	0	0	0	0.631	0.163	0.914	0.456	0.983
Anpep	Lap3	10116.ENSRNC	10116.ENSRNC	0	0	0	0	0.105	0.137	0.8	0.64	0.937
Anxa2	Plg	10116.ENSRNC	10116.ENSRNC	0	0	0	0	0	0.077	0.9	0.981	0.998
Anxa2	S100a10	10116.ENSRNC	10116.ENSRNC	0	0	0	0	0.764	0.808	0.8	0.989	0.999
Anxa5	Tagln2	10116.ENSRNC	10116.ENSRNC	0	0	0	0	0.344	0	0.9	0.109	0.936
Anxa5	Vcl	10116.ENSRNC	10116.ENSRNC	0	0	0	0	0.094	0	0.9	0.376	0.938
Anxa5	Wdr1	10116.ENSRNC	10116.ENSRNC	0	0	0	0	0.064	0	0.9	0.108	0.909
Anxa5	Tln1	10116.ENSRNC	10116.ENSRNC	0	0	0	0	0.07	0	0.9	0.114	0.91
Anxa5	Flna	10116.ENSRNC	10116.ENSRNC	0	0	0	0	0.151	0.09	0.9	0.234	0.932
Apoo	Ndufa12	10116.ENSRNC	10116.ENSRNC	0	0	0	0	0.687	0.742	0	0	0.916
Apoo	Ndufv2	10116.ENSRNC	10116.ENSRNC	0	0	0	0	0.531	0.787	0	0.237	0.917
Arhgdia	Rhoa	10116.ENSRNC	10116.ENSRNC	0	0	0	0	0.085	0.926	0.919	0.952	0.999
Atp1a1	Atp1b1	10116.ENSRNC	10116.ENSRNC	0	0	0	0	0.119	0.987	0.83	0.84	0.999
Atp1a1	Atp1a2	10116.ENSRNC	10116.ENSRNC	0	0	0.449	0.984	0	0.514	0.8	0.754	0.9
Atp1a2	Atp1b1	10116.ENSRNC	10116.ENSRNC	0	0	0	0	0.082	0.823	0.83	0.73	0.991
Atp2a2	Hrc	10116.ENSRNC	10116.ENSRNC	0	0	0	0	0.099	0	0.8	0.687	0.938
Atp5a1	Atp5i	10116.ENSRNC	10116.ENSRNC	0	0	0	0	0.3	0.809	0.965	0.503	0.997
Atp5a1	Mdh2	10116.ENSRNC	10116.ENSRNC	0.085	0	0	0	0.744	0.428	0	0.653	0.947
Atp5a1	Atp5j	10116.ENSRNC	10116.ENSRNC	0	0	0	0	0.182	0.69	0.9	0.442	0.983
Atp5a1	Atp5o	10116.ENSRNC	10116.ENSRNC	0.128	0	0	0	0.746	0.821	0.965	0.767	0.999
Atp5a1	Atp5b	10116.ENSRNC	10116.ENSRNC	0.09	0	0.447	0.592	0.768	0.984	0.965	0.918	0.999
Atp5a1	Atp5pd	10116.ENSRNC	10116.ENSRNC	0	0	0	0	0.68	0.69	0.941	0.749	0.998
Atp5a1	Sdhb	10116.ENSRNC	10116.ENSRNC	0.106	0	0	0	0.549	0.372	0	0.753	0.929
Atp5a1	Ndufs3	10116.ENSRNC	10116.ENSRNC	0.11	0	0	0	0.649	0.367	0	0.611	0.913
Atp5a1	Cyc1	10116.ENSRNC	10116.ENSRNC	0	0	0	0	0.887	0.46	0	0.379	0.958
Atp5a1	Sdha	10116.ENSRNC	10116.ENSRNC	0.078	0	0	0	0.771	0	0	0.683	0.927
Atp5a1	Uqcrc2	10116.ENSRNC	10116.ENSRNC	0	0	0	0	0.68	0.481	0	0.765	0.957
Atp5a1	Atp5f1	10116.ENSRNC	10116.ENSRNC	0	0	0	0	0.73	0.811	0.443	0.714	0.99
Atp5a1	Ndufa9	10116.ENSRNC	10116.ENSRNC	0.043	0	0	0	0.58	0.316	0	0.69	0.903

Atp5a1	Ndufv1	10116.ENSRNO	10116.ENSRNO	0	0	0	0	0.786	0.084	0	0.554	0.905
Atp5a1	Cox5a	10116.ENSRNO	10116.ENSRNO	0	0	0	0	0.569	0.369	0	0.733	0.921
Atp5a1	Uqcrc1	10116.ENSRNO	10116.ENSRNO	0	0	0	0	0.782	0.447	0	0.749	0.967
Atp5a1	Ndufs2	10116.ENSRNO	10116.ENSRNO	0.118	0	0	0	0.826	0.538	0	0.649	0.971
Atp5a1	Uqcrfs1	10116.ENSRNO	10116.ENSRNO	0	0	0	0	0.873	0.372	0	0.686	0.972
Atp5a1	Usmg5	10116.ENSRNO	10116.ENSRNO	0	0	0	0	0.204	0.676	0.941	0.156	0.985
Atp5a1	Atp5j2	10116.ENSRNO	10116.ENSRNO	0	0	0	0	0.597	0.73	0.9	0.369	0.992
Atp5a1	Atp5l	10116.ENSRNO	10116.ENSRNO	0	0	0	0	0.323	0.809	0.924	0.624	0.995
Atp5a1	Atp5c1	10116.ENSRNO	10116.ENSRNO	0.128	0	0.435	0	0.948	0.984	0.965	0.619	0.999
Atp5b	Atp5i	10116.ENSRNO	10116.ENSRNO	0	0	0	0	0.28	0.924	0.975	0.555	0.999
Atp5b	Mdh2	10116.ENSRNO	10116.ENSRNO	0.071	0	0	0	0.695	0.504	0	0.802	0.968
Atp5b	Atp5j	10116.ENSRNO	10116.ENSRNO	0	0	0	0	0.172	0.8	0.9	0.65	0.993
Atp5b	Atp5o	10116.ENSRNO	10116.ENSRNO	0.1	0	0	0	0.999	0.937	0.975	0.851	0.999
Atp5b	P4hb	10116.ENSRNO	10116.ENSRNO	0.042	0	0	0	0.692	0.506	0	0.416	0.903
Atp5b	Cox5b	10116.ENSRNO	10116.ENSRNO	0	0	0	0	0.729	0.052	0	0.702	0.917
Atp5b	Ndufa12	10116.ENSRNO	10116.ENSRNO	0	0	0	0	0.552	0.696	0	0.445	0.917
Atp5b	Slc25a3	10116.ENSRNO	10116.ENSRNO	0	0	0	0	0.847	0	0	0.479	0.917
Atp5b	Ndufv1	10116.ENSRNO	10116.ENSRNO	0	0	0	0	0.72	0.054	0	0.728	0.921
Atp5b	Hspd1	10116.ENSRNO	10116.ENSRNO	0.044	0	0	0	0.706	0.053	0.5	0.497	0.921
Atp5b	Ndufs3	10116.ENSRNO	10116.ENSRNO	0.097	0	0	0	0.438	0.472	0	0.746	0.923
Atp5b	Uqcrc1	10116.ENSRNO	10116.ENSRNO	0	0	0	0	0.707	0.16	0	0.727	0.927
Atp5b	Sdha	10116.ENSRNO	10116.ENSRNO	0.061	0	0	0	0.723	0	0	0.754	0.93
Atp5b	Sdhb	10116.ENSRNO	10116.ENSRNO	0.091	0	0	0	0.592	0.452	0	0.713	0.934
Atp5b	Ndufs1	10116.ENSRNO	10116.ENSRNO	0.069	0	0	0	0.582	0.491	0	0.722	0.937
Atp5b	Cox5a	10116.ENSRNO	10116.ENSRNO	0	0	0	0	0.731	0.474	0	0.717	0.956
Atp5b	Uqcrfs1	10116.ENSRNO	10116.ENSRNO	0	0	0	0	0.809	0.445	0	0.672	0.962
Atp5b	Uqcrc2	10116.ENSRNO	10116.ENSRNO	0	0	0	0	0.793	0.53	0	0.684	0.966
Atp5b	Cyc1	10116.ENSRNO	10116.ENSRNO	0	0	0	0	0.805	0.438	0	0.742	0.969
Atp5b	Ndufs2	10116.ENSRNO	10116.ENSRNO	0.099	0	0	0	0.721	0.696	0	0.685	0.972
Atp5b	Usmg5	10116.ENSRNO	10116.ENSRNO	0	0	0	0	0.182	0.796	0.965	0.138	0.994
Atp5b	Atp5j2	10116.ENSRNO	10116.ENSRNO	0	0	0	0	0.681	0.816	0.9	0.471	0.996
Atp5b	Atp5f1	10116.ENSRNO	10116.ENSRNO	0	0	0	0	0.778	0.928	0.67	0.815	0.998
Atp5b	Atp5pd	10116.ENSRNO	10116.ENSRNO	0	0	0	0	0.663	0.799	0.965	0.765	0.999
Atp5b	Atp5l	10116.ENSRNO	10116.ENSRNO	0	0	0	0	0.595	0.926	0.937	0.706	0.999
Atp5b	Atp5c1	10116.ENSRNO	10116.ENSRNO	0.1	0	0.429	0	0.804	0.984	0.975	0.753	0.999
Atp5c1	Atp5i	10116.ENSRNO	10116.ENSRNO	0	0	0	0	0.788	0.804	0.975	0.515	0.999
Atp5c1	Mdh2	10116.ENSRNO	10116.ENSRNO	0.093	0	0	0	0.855	0.462	0	0.405	0.952
Atp5c1	Atp5j	10116.ENSRNO	10116.ENSRNO	0	0	0	0	0.846	0.696	0.9	0.326	0.996
Atp5c1	Atp5o	10116.ENSRNO	10116.ENSRNO	0.155	0	0	0	0.913	0.927	0.975	0.712	0.999
Atp5c1	Sdhc	10116.ENSRNO	10116.ENSRNO	0.136	0	0	0	0.925	0	0	0.202	0.943
Atp5c1	Atp5pd	10116.ENSRNO	10116.ENSRNO	0	0	0	0	0.828	0.738	0.965	0.553	0.999
Atp5c1	Pdhb	10116.ENSRNO	10116.ENSRNO	0	0	0	0	0.799	0.467	0	0.235	0.91
Atp5c1	Sdhb	10116.ENSRNO	10116.ENSRNO	0.132	0	0	0	0.79	0.46	0	0.504	0.944
Atp5c1	Ndufs3	10116.ENSRNO	10116.ENSRNO	0.139	0	0	0	0.904	0.483	0	0.358	0.969
Atp5c1	Ndufb9	10116.ENSRNO	10116.ENSRNO	0	0	0	0	0.888	0.383	0	0.505	0.962
Atp5c1	Ndufs1	10116.ENSRNO	10116.ENSRNO	0.103	0	0	0	0.77	0.551	0	0.472	0.944
Atp5c1	Uqcrh	10116.ENSRNO	10116.ENSRNO	0	0	0	0	0.913	0.441	0	0.468	0.972
Atp5c1	Ndufv2	10116.ENSRNO	10116.ENSRNO	0.109	0	0	0	0.914	0.462	0	0.437	0.973
Atp5c1	Cyc1	10116.ENSRNO	10116.ENSRNO	0	0	0	0	0.899	0.467	0	0.329	0.96
Atp5c1	Sdha	10116.ENSRNO	10116.ENSRNO	0.094	0	0	0	0.741	0.447	0	0.489	0.925
Atp5c1	Uqcrc2	10116.ENSRNO	10116.ENSRNO	0	0	0	0	0.89	0.515	0	0.507	0.971
Atp5c1	Atp5f1	10116.ENSRNO	10116.ENSRNO	0	0	0	0	0.787	0.928	0.67	0.607	0.997
Atp5c1	Ndufa10	10116.ENSRNO	10116.ENSRNO	0	0	0	0	0.885	0.451	0	0.302	0.952
Atp5c1	Cox5b	10116.ENSRNO	10116.ENSRNO	0	0	0	0	0.84	0	0	0.474	0.912
Atp5c1	Ndufv1	10116.ENSRNO	10116.ENSRNO	0	0	0	0	0.689	0.444	0	0.544	0.914
Atp5c1	Uqcrfs1	10116.ENSRNO	10116.ENSRNO	0	0	0	0	0.925	0.46	0	0.529	0.979
Atp5c1	Cox5a	10116.ENSRNO	10116.ENSRNO	0	0	0	0	0.828	0.465	0	0.425	0.942

Atp5c1	Usmg5	10116.ENSRNO	10116.ENSRNO	0	0	0	0	0.443	0.736	0.965	0.069	0.994
Atp5c1	Atp5j2	10116.ENSRNO	10116.ENSRNO	0	0	0	0	0.663	0.696	0.9	0.379	0.992
Atp5c1	Ndufa9	10116.ENSRNO	10116.ENSRNO	0.044	0	0	0	0.873	0.442	0	0.273	0.944
Atp5c1	Ndufb7	10116.ENSRNO	10116.ENSRNO	0	0	0	0	0.84	0.466	0	0.094	0.916
Atp5c1	Ndufb6	10116.ENSRNO	10116.ENSRNO	0	0	0	0	0.823	0.453	0	0.359	0.932
Atp5c1	Ndufs7	10116.ENSRNO	10116.ENSRNO	0.143	0	0	0	0.695	0.459	0	0.509	0.921
Atp5c1	Atp5l	10116.ENSRNO	10116.ENSRNO	0	0	0	0	0.771	0.8	0.937	0.612	0.998
Atp5c1	Uqcrc1	10116.ENSRNO	10116.ENSRNO	0	0	0	0	0.782	0.508	0	0.522	0.944
Atp5c1	Ndufa8	10116.ENSRNO	10116.ENSRNO	0	0	0	0	0.741	0.46	0	0.383	0.906
Atp5c1	Ndufs2	10116.ENSRNO	10116.ENSRNO	0.15	0	0	0	0.919	0.464	0	0.488	0.978
Atp5f1	Atp5i	10116.ENSRNO	10116.ENSRNO	0	0	0	0	0.648	0.928	0.67	0.606	0.996
Atp5f1	Atp5j	10116.ENSRNO	10116.ENSRNO	0	0	0	0	0.857	0.786	0	0.725	0.99
Atp5f1	Atp5o	10116.ENSRNO	10116.ENSRNO	0	0	0	0	0.82	0.928	0.67	0.786	0.998
Atp5f1	Sdhc	10116.ENSRNO	10116.ENSRNO	0	0	0	0	0.866	0	0	0.366	0.911
Atp5f1	Atp5pd	10116.ENSRNO	10116.ENSRNO	0	0	0	0	0.645	0.786	0.443	0.749	0.987
Atp5f1	Ndufa5	10116.ENSRNO	10116.ENSRNO	0	0	0	0	0.672	0.794	0	0.624	0.972
Atp5f1	Ndufa12	10116.ENSRNO	10116.ENSRNO	0	0	0	0	0.706	0.576	0	0.351	0.912
Atp5f1	Sdhb	10116.ENSRNO	10116.ENSRNO	0	0	0	0	0.664	0.735	0	0.54	0.955
Atp5f1	Ndufs3	10116.ENSRNO	10116.ENSRNO	0	0	0	0	0.86	0.412	0	0.335	0.94
Atp5f1	Ndufb9	10116.ENSRNO	10116.ENSRNO	0	0	0	0	0.829	0.797	0	0.399	0.977
Atp5f1	Ndufs4	10116.ENSRNO	10116.ENSRNO	0	0	0	0	0.69	0.798	0	0.305	0.952
Atp5f1	Ndufc2	10116.ENSRNO	10116.ENSRNO	0	0	0	0	0.704	0.698	0	0.53	0.954
Atp5f1	Uqcrh	10116.ENSRNO	10116.ENSRNO	0	0	0	0	0.668	0.719	0	0.414	0.94
Atp5f1	Ndufv2	10116.ENSRNO	10116.ENSRNO	0	0	0	0	0.731	0.794	0	0.466	0.967
Atp5f1	Cyc1	10116.ENSRNO	10116.ENSRNO	0	0	0	0	0.712	0.793	0	0.589	0.973
Atp5f1	Ndufb10	10116.ENSRNO	10116.ENSRNO	0	0	0	0	0.736	0.649	0	0.241	0.923
Atp5f1	Uqcrc2	10116.ENSRNO	10116.ENSRNO	0	0	0	0	0.768	0.722	0	0.597	0.971
Atp5f1	Ndufab1	10116.ENSRNO	10116.ENSRNO	0	0	0	0	0.645	0.66	0	0.257	0.902
Atp5f1	Uqcrb	10116.ENSRNO	10116.ENSRNO	0	0	0	0	0.619	0.58	0	0.517	0.916
Atp5f1	Ndufv1	10116.ENSRNO	10116.ENSRNO	0	0	0	0	0.395	0.797	0	0.435	0.924
Atp5f1	Cox5b	10116.ENSRNO	10116.ENSRNO	0	0	0	0	0.777	0.504	0	0.414	0.929
Atp5f1	Ndufa9	10116.ENSRNO	10116.ENSRNO	0	0	0	0	0.64	0.691	0	0.432	0.931
Atp5f1	Ndufb6	10116.ENSRNO	10116.ENSRNO	0	0	0	0	0.764	0.521	0	0.477	0.935
Atp5f1	Cox4i1	10116.ENSRNO	10116.ENSRNO	0	0	0	0	0.673	0.792	0	0.27	0.946
Atp5f1	Usmg5	10116.ENSRNO	10116.ENSRNO	0	0	0	0	0.538	0.798	0.443	0.143	0.949
Atp5f1	Ndufs7	10116.ENSRNO	10116.ENSRNO	0	0	0	0	0.718	0.798	0	0.361	0.96
Atp5f1	Ndufa8	10116.ENSRNO	10116.ENSRNO	0	0	0	0	0.708	0.792	0	0.408	0.96
Atp5f1	Ndufb7	10116.ENSRNO	10116.ENSRNO	0	0	0	0	0.692	0.712	0	0.596	0.961
Atp5f1	Ndufa10	10116.ENSRNO	10116.ENSRNO	0	0	0	0	0.675	0.79	0	0.541	0.966
Atp5f1	Cox5a	10116.ENSRNO	10116.ENSRNO	0	0	0	0	0.818	0.552	0	0.642	0.968
Atp5f1	Ndufs2	10116.ENSRNO	10116.ENSRNO	0	0	0	0	0.721	0.798	0	0.517	0.97
Atp5f1	Atp5j2	10116.ENSRNO	10116.ENSRNO	0	0	0	0	0.667	0.811	0	0.604	0.972
Atp5f1	Atp5l	10116.ENSRNO	10116.ENSRNO	0	0	0	0	0.767	0.809	0.272	0.667	0.987
Atp5f1	Uqcrfs1	10116.ENSRNO	10116.ENSRNO	0	0	0	0	0.919	0.793	0	0.418	0.989
Atp5i	Ndufs1	10116.ENSRNO	10116.ENSRNO	0	0	0	0	0.15	0.855	0	0.285	0.904
Atp5i	Ndufa8	10116.ENSRNO	10116.ENSRNO	0	0	0	0	0.735	0.46	0	0.427	0.91
Atp5i	Ndufb9	10116.ENSRNO	10116.ENSRNO	0	0	0	0	0.834	0	0	0.499	0.913
Atp5i	Ndufs4	10116.ENSRNO	10116.ENSRNO	0	0	0	0	0.708	0.46	0	0.512	0.916
Atp5i	Ndufc2	10116.ENSRNO	10116.ENSRNO	0	0	0	0	0.782	0.462	0	0.351	0.917
Atp5i	Cox5a	10116.ENSRNO	10116.ENSRNO	0	0	0	0	0.744	0.462	0	0.447	0.917
Atp5i	Ndufa5	10116.ENSRNO	10116.ENSRNO	0	0	0	0	0.698	0.466	0	0.693	0.946
Atp5i	Ndufv2	10116.ENSRNO	10116.ENSRNO	0	0	0	0	0.855	0.46	0	0.418	0.95
Atp5i	Ndufb7	10116.ENSRNO	10116.ENSRNO	0	0	0	0	0.944	0.462	0	0.289	0.976
Atp5i	Ndufa6	10116.ENSRNO	10116.ENSRNO	0	0	0	0	0.845	0.855	0	0.389	0.985
Atp5i	Uqcrh	10116.ENSRNO	10116.ENSRNO	0	0	0	0	0.95	0.446	0	0.596	0.988
Atp5i	Atp5j2	10116.ENSRNO	10116.ENSRNO	0	0	0	0	0.689	0.779	0.9	0.614	0.996
Atp5i	Atp5j	10116.ENSRNO	10116.ENSRNO	0	0	0	0	0.793	0.751	0.9	0.639	0.997

Atp5i	Usmg5	10116.ENSRNO	10116.ENSRNO	0	0	0	0	0.609	0.779	0.965	0.141	0.997
Atp5i	Atp5pd	10116.ENSRNO	10116.ENSRNO	0	0	0	0	0.717	0.751	0.965	0.627	0.998
Atp5i	Atp5l	10116.ENSRNO	10116.ENSRNO	0	0	0	0	0.745	0.925	0.937	0.793	0.999
Atp5i	Atp5o	10116.ENSRNO	10116.ENSRNO	0	0	0	0	0.812	0.926	0.975	0.625	0.999
Atp5j	Uqcrh	10116.ENSRNO	10116.ENSRNO	0	0	0	0	0.784	0	0	0.589	0.907
Atp5j	Cox5b	10116.ENSRNO	10116.ENSRNO	0	0	0	0	0.845	0	0	0.437	0.909
Atp5j	Ndufa6	10116.ENSRNO	10116.ENSRNO	0	0	0	0	0.85	0	0	0.426	0.91
Atp5j	Uqcrb	10116.ENSRNO	10116.ENSRNO	0	0	0	0	0.9	0	0	0.234	0.92
Atp5j	Ndufb6	10116.ENSRNO	10116.ENSRNO	0	0	0	0	0.911	0	0	0.191	0.925
Atp5j	Ndufb9	10116.ENSRNO	10116.ENSRNO	0	0	0	0	0.854	0	0	0.521	0.927
Atp5j	Ndufa12	10116.ENSRNO	10116.ENSRNO	0	0	0	0	0.925	0	0	0.247	0.941
Atp5j	Ndufa5	10116.ENSRNO	10116.ENSRNO	0	0	0	0	0.904	0.352	0	0.576	0.971
Atp5j	Atp5j2	10116.ENSRNO	10116.ENSRNO	0	0	0	0	0.617	0.69	0.9	0.478	0.992
Atp5j	Usmg5	10116.ENSRNO	10116.ENSRNO	0	0	0	0	0.852	0.542	0.9	0.121	0.993
Atp5j	Atp5l	10116.ENSRNO	10116.ENSRNO	0	0	0	0	0.745	0.726	0.9	0.621	0.997
Atp5j	Atp5o	10116.ENSRNO	10116.ENSRNO	0	0	0	0	0.764	0.803	0.9	0.619	0.997
Atp5j	Atp5pd	10116.ENSRNO	10116.ENSRNO	0	0	0	0	0.852	0.562	0.9	0.672	0.997
Atp5j2	Atp5o	10116.ENSRNO	10116.ENSRNO	0	0	0	0	0.742	0.821	0.9	0.381	0.996
Atp5j2	Atp5pd	10116.ENSRNO	10116.ENSRNO	0	0	0	0	0.644	0.69	0.9	0.35	0.991
Atp5j2	Uqcrh	10116.ENSRNO	10116.ENSRNO	0	0	0	0	0.723	0.375	0	0.528	0.911
Atp5j2	Cyc1	10116.ENSRNO	10116.ENSRNO	0	0	0	0	0.554	0.434	0	0.637	0.9
Atp5j2	Usmg5	10116.ENSRNO	10116.ENSRNO	0	0	0	0	0.572	0.521	0.9	0.122	0.979
Atp5j2	Ndufb7	10116.ENSRNO	10116.ENSRNO	0	0	0	0	0.712	0.379	0	0.56	0.914
Atp5j2	Atp5l	10116.ENSRNO	10116.ENSRNO	0	0	0	0	0.632	0.726	0.9	0.625	0.995
Atp5l	Atp5o	10116.ENSRNO	10116.ENSRNO	0	0	0	0	0.783	0.925	0.937	0.703	0.999
Atp5l	Atp5pd	10116.ENSRNO	10116.ENSRNO	0	0	0	0	0.909	0.729	0.92	0.67	0.999
Atp5l	Ndufa4	10116.ENSRNO	10116.ENSRNO	0	0	0	0	0.758	0.349	0	0.627	0.936
Atp5l	Ndufa5	10116.ENSRNO	10116.ENSRNO	0	0	0	0	0.841	0.814	0	0.595	0.987
Atp5l	Ndufa12	10116.ENSRNO	10116.ENSRNO	0	0	0	0	0.772	0.813	0	0.583	0.98
Atp5l	Sdhb	10116.ENSRNO	10116.ENSRNO	0	0	0	0	0.398	0.808	0	0.265	0.907
Atp5l	Ndufa6	10116.ENSRNO	10116.ENSRNO	0	0	0	0	0.88	0.323	0	0.444	0.95
Atp5l	Ndufb9	10116.ENSRNO	10116.ENSRNO	0	0	0	0	0.856	0.814	0	0.526	0.986
Atp5l	Ndufs4	10116.ENSRNO	10116.ENSRNO	0	0	0	0	0.71	0.814	0	0.316	0.959
Atp5l	Ndufc2	10116.ENSRNO	10116.ENSRNO	0	0	0	0	0.815	0.814	0	0.521	0.982
Atp5l	Uqcrh	10116.ENSRNO	10116.ENSRNO	0	0	0	0	0.846	0.812	0	0.595	0.987
Atp5l	Cyc1	10116.ENSRNO	10116.ENSRNO	0	0	0	0	0.629	0.815	0	0.542	0.965
Atp5l	Ndufb8	10116.ENSRNO	10116.ENSRNO	0	0	0	0	0.858	0.193	0	0.402	0.925
Atp5l	Ndufb10	10116.ENSRNO	10116.ENSRNO	0	0	0	0	0.847	0.706	0	0.258	0.963
Atp5l	Uqcrc2	10116.ENSRNO	10116.ENSRNO	0	0	0	0	0.624	0.805	0	0.585	0.966
Atp5l	Ndufa10	10116.ENSRNO	10116.ENSRNO	0	0	0	0	0.6	0.814	0	0.402	0.951
Atp5l	Cox5b	10116.ENSRNO	10116.ENSRNO	0	0	0	0	0.859	0.54	0	0.407	0.958
Atp5l	Ndufa2	10116.ENSRNO	10116.ENSRNO	0	0	0	0	0.848	0.714	0	0.348	0.969
Atp5l	Cox4i1	10116.ENSRNO	10116.ENSRNO	0	0	0	0	0.812	0.548	0	0.311	0.936
Atp5l	Ndufab1	10116.ENSRNO	10116.ENSRNO	0	0	0	0	0.642	0.714	0	0.542	0.949
Atp5l	Cox5a	10116.ENSRNO	10116.ENSRNO	0	0	0	0	0.792	0.813	0	0.445	0.976
Atp5l	Usmg5	10116.ENSRNO	10116.ENSRNO	0	0	0	0	0.629	0.599	0.92	0.116	0.988
Atp5l	Cox6b1	10116.ENSRNO	10116.ENSRNO	0	0	0	0	0.842	0.654	0	0.239	0.955
Atp5l	Ndufa9	10116.ENSRNO	10116.ENSRNO	0	0	0	0	0.504	0.814	0	0.152	0.915
Atp5l	Ndufb7	10116.ENSRNO	10116.ENSRNO	0	0	0	0	0.852	0.813	0	0.577	0.987
Atp5l	Ndufs7	10116.ENSRNO	10116.ENSRNO	0	0	0	0	0.701	0.814	0	0.594	0.975
Atp5l	Ndufs5	10116.ENSRNO	10116.ENSRNO	0	0	0	0	0.887	0.521	0	0.32	0.96
Atp5l	Ndufs2	10116.ENSRNO	10116.ENSRNO	0	0	0	0	0.219	0.814	0	0.547	0.928
Atp5l	Ndufa11	10116.ENSRNO	10116.ENSRNO	0	0	0	0	0.906	0	0	0.558	0.956
Atp5o	Mdh2	10116.ENSRNO	10116.ENSRNO	0.09	0	0	0	0.739	0.51	0	0.49	0.933
Atp5o	Ndufv1	10116.ENSRNO	10116.ENSRNO	0	0	0	0	0.516	0.453	0	0.664	0.903
Atp5o	Cox6b1	10116.ENSRNO	10116.ENSRNO	0	0	0	0	0.785	0.416	0	0.321	0.907
Atp5o	Ndufa12	10116.ENSRNO	10116.ENSRNO	0	0	0	0	0.807	0	0	0.575	0.914

Atp5o	Ndufab1	10116.ENSRNO	10116.ENSRNO	0.044	0	0	0	0.717	0.434	0	0.512	0.915
Atp5o	Ndufb10	10116.ENSRNO	10116.ENSRNO	0	0	0	0	0.784	0.42	0	0.461	0.926
Atp5o	Ndufc2	10116.ENSRNO	10116.ENSRNO	0	0	0	0	0.762	0.473	0	0.459	0.926
Atp5o	Ndufb7	10116.ENSRNO	10116.ENSRNO	0	0	0	0	0.784	0.462	0	0.429	0.927
Atp5o	Sdhc	10116.ENSRNO	10116.ENSRNO	0.121	0	0	0	0.828	0	0	0.57	0.929
Atp5o	Sdhb	10116.ENSRNO	10116.ENSRNO	0.124	0	0	0	0.667	0.46	0	0.62	0.932
Atp5o	Ndufa8	10116.ENSRNO	10116.ENSRNO	0	0	0	0	0.788	0.513	0	0.447	0.938
Atp5o	Uqcrc1	10116.ENSRNO	10116.ENSRNO	0	0	0	0	0.725	0.32	0	0.706	0.94
Atp5o	Ndufa5	10116.ENSRNO	10116.ENSRNO	0	0	0	0	0.824	0.473	0	0.469	0.946
Atp5o	Ndufb9	10116.ENSRNO	10116.ENSRNO	0	0	0	0	0.878	0.115	0	0.562	0.948
Atp5o	Cox5a	10116.ENSRNO	10116.ENSRNO	0	0	0	0	0.86	0.057	0	0.645	0.949
Atp5o	Ndufa6	10116.ENSRNO	10116.ENSRNO	0	0	0	0	0.848	0.462	0	0.462	0.952
Atp5o	Ndufs3	10116.ENSRNO	10116.ENSRNO	0.121	0	0	0	0.824	0.099	0	0.705	0.953
Atp5o	Ndufv2	10116.ENSRNO	10116.ENSRNO	0	0	0	0	0.787	0.46	0	0.636	0.954
Atp5o	Cox5b	10116.ENSRNO	10116.ENSRNO	0	0	0	0	0.846	0	0	0.732	0.957
Atp5o	Cyc1	10116.ENSRNO	10116.ENSRNO	0	0	0	0	0.843	0.485	0	0.56	0.961
Atp5o	Ndufs4	10116.ENSRNO	10116.ENSRNO	0	0	0	0	0.877	0.537	0	0.401	0.963
Atp5o	Uqcrc2	10116.ENSRNO	10116.ENSRNO	0	0	0	0	0.819	0.534	0	0.602	0.963
Atp5o	Uqcrfs1	10116.ENSRNO	10116.ENSRNO	0	0	0	0	0.806	0.46	0	0.755	0.972
Atp5o	Usmg5	10116.ENSRNO	10116.ENSRNO	0	0	0	0	0.648	0.796	0.965	0.363	0.998
Atp5o	Atp5pd	10116.ENSRNO	10116.ENSRNO	0	0	0	0	0.748	0.803	0.965	0.814	0.999
Atp5pd	Ndufv2	10116.ENSRNO	10116.ENSRNO	0	0	0	0	0.76	0.378	0	0.393	0.901
Atp5pd	Cox5a	10116.ENSRNO	10116.ENSRNO	0	0	0	0	0.677	0.046	0	0.704	0.901
Atp5pd	Ndufc2	10116.ENSRNO	10116.ENSRNO	0	0	0	0	0.711	0.396	0	0.496	0.904
Atp5pd	Cox4i1	10116.ENSRNO	10116.ENSRNO	0	0	0	0	0.777	0.378	0	0.368	0.905
Atp5pd	Ndufa2	10116.ENSRNO	10116.ENSRNO	0	0	0	0	0.695	0.33	0	0.59	0.909
Atp5pd	Uqcrc2	10116.ENSRNO	10116.ENSRNO	0	0	0	0	0.627	0.381	0	0.658	0.914
Atp5pd	Sdhb	10116.ENSRNO	10116.ENSRNO	0	0	0	0	0.737	0.377	0	0.538	0.918
Atp5pd	Ndufb7	10116.ENSRNO	10116.ENSRNO	0	0	0	0	0.768	0.38	0	0.493	0.92
Atp5pd	Uqcrc1	10116.ENSRNO	10116.ENSRNO	0	0	0	0	0.687	0.411	0	0.62	0.924
Atp5pd	Ndufa5	10116.ENSRNO	10116.ENSRNO	0	0	0	0	0.805	0.381	0	0.451	0.928
Atp5pd	Ndufa4	10116.ENSRNO	10116.ENSRNO	0	0	0	0	0.787	0.386	0	0.543	0.935
Atp5pd	Uqcrh	10116.ENSRNO	10116.ENSRNO	0	0	0	0	0.805	0.347	0	0.563	0.939
Atp5pd	Ndufb10	10116.ENSRNO	10116.ENSRNO	0	0	0	0	0.854	0.444	0	0.371	0.944
Atp5pd	Ndufa6	10116.ENSRNO	10116.ENSRNO	0	0	0	0	0.831	0.381	0	0.527	0.946
Atp5pd	Uqcrfs1	10116.ENSRNO	10116.ENSRNO	0	0	0	0	0.842	0.512	0	0.426	0.952
Atp5pd	Cox6b1	10116.ENSRNO	10116.ENSRNO	0	0	0	0	0.917	0.325	0	0.248	0.954
Atp5pd	Ndufs3	10116.ENSRNO	10116.ENSRNO	0	0	0	0	0.841	0.374	0	0.616	0.958
Atp5pd	Cox5b	10116.ENSRNO	10116.ENSRNO	0	0	0	0	0.913	0	0	0.597	0.963
Atp5pd	Usmg5	10116.ENSRNO	10116.ENSRNO	0	0	0	0	0.538	0.479	0.941	0.216	0.987
Atp6v0a1	Atp6v1a	10116.ENSRNO	10116.ENSRNO	0.098	0	0	0	0.305	0.805	0.862	0.738	0.994
Bcat2	Bckdhb	10116.ENSRNO	10116.ENSRNO	0.106	0	0	0	0.07	0.079	0.994	0.698	0.998
Bcat2	Dbt	10116.ENSRNO	10116.ENSRNO	0.052	0	0	0	0.068	0.063	0.952	0.637	0.982
Bcat2	Bckdha	10116.ENSRNO	10116.ENSRNO	0.119	0	0	0	0.115	0	0.994	0.802	0.999
Bckdha	Ep300	10116.ENSRNO	10116.ENSRNO	0	0	0	0	0	0	0	0.965	0.965
Bckdha	Dlst	10116.ENSRNO	10116.ENSRNO	0.109	0	0.212	0	0.512	0.514	0.683	0.236	0.95
Bckdha	Pdhb	10116.ENSRNO	10116.ENSRNO	0.128	0.826	0.412	0	0.589	0.621	0.475	0.36	0.994
Bckdha	Bckdhb	10116.ENSRNO	10116.ENSRNO	0.128	0.891	0.446	0	0.73	0.925	0.981	0.862	0.999
Bckdha	Ivd	10116.ENSRNO	10116.ENSRNO	0.044	0.007	0	0	0.577	0	0.91	0.575	0.982
Bckdha	Ldha	10116.ENSRNO	10116.ENSRNO	0.044	0	0	0	0.064	0	0.914	0.188	0.929
Bckdha	Ldhb	10116.ENSRNO	10116.ENSRNO	0.044	0	0	0	0.064	0	0.914	0.188	0.929
Bckdha	Dbt	10116.ENSRNO	10116.ENSRNO	0.109	0	0.404	0	0.568	0.514	0.994	0.742	0.999
Bckdha	Pccb	10116.ENSRNO	10116.ENSRNO	0.097	0	0	0	0.572	0	0.91	0.501	0.98
Bckdha	Dlat	10116.ENSRNO	10116.ENSRNO	0.109	0	0.268	0	0.497	0.514	0.34	0.259	0.903
Bckdhb	Dlst	10116.ENSRNO	10116.ENSRNO	0.095	0.049	0.228	0	0.48	0.45	0.644	0.51	0.957
Bckdhb	Mccc2	10116.ENSRNO	10116.ENSRNO	0.09	0	0	0	0.672	0	0.447	0.609	0.926
Bckdhb	Ldhb	10116.ENSRNO	10116.ENSRNO	0.043	0	0	0	0.111	0.091	0.913	0.166	0.933

Bckdhb	Gcsh	10116.ENSRNO	10116.ENSRNO	0.042	0	0	0	0.149	0	0.9	0.281	0.933
Bckdhb	Ldha	10116.ENSRNO	10116.ENSRNO	0.043	0	0	0	0.111	0.091	0.913	0.166	0.933
Bckdhb	Mccc1	10116.ENSRNO	10116.ENSRNO	0.083	0	0	0	0.611	0.218	0.449	0.663	0.938
Bckdhb	Dlat	10116.ENSRNO	10116.ENSRNO	0.095	0	0.297	0	0.479	0.45	0.622	0.309	0.941
Bckdhb	Ivd	10116.ENSRNO	10116.ENSRNO	0.043	0	0	0	0.513	0.057	0.91	0.694	0.985
Bckdhb	Pccb	10116.ENSRNO	10116.ENSRNO	0.09	0	0	0	0.627	0	0.91	0.647	0.987
Bckdhb	Dbt	10116.ENSRNO	10116.ENSRNO	0.095	0	0.408	0	0.645	0.45	0.994	0.661	0.999
Bdh1	Oxct1	10116.ENSRNO	10116.ENSRNO	0	0	0	0	0.049	0	0.994	0.859	0.999
Bsg	Cav1	10116.ENSRNO	10116.ENSRNO	0	0	0	0	0	0.078	0.8	0.882	0.976
Bsg	Ppib	10116.ENSRNO	10116.ENSRNO	0	0	0	0	0.091	0.118	0.629	0.895	0.965
C3	Plg	10116.ENSRNO	10116.ENSRNO	0	0	0	0	0.281	0.163	0.818	0.51	0.939
Cam	Cav1	10116.ENSRNO	10116.ENSRNO	0	0	0	0	0	0.044	0.8	0.583	0.913
Cam	Nos3	10116.ENSRNO	10116.ENSRNO	0	0	0	0	0	0.685	0.8	0.607	0.973
Cam	Camk2d	10116.ENSRNO	10116.ENSRNO	0	0	0	0	0	0.667	0.8	0.337	0.952
Cam	Hsp90ab1	10116.ENSRNO	10116.ENSRNO	0	0	0	0	0.055	0.422	0.8	0.266	0.909
Camk2d	Ywhab	10116.ENSRNO	10116.ENSRNO	0	0	0	0	0.056	0.046	0.9	0.188	0.917
Canx	Pdia3	10116.ENSRNO	10116.ENSRNO	0	0	0	0	0.606	0.512	0.6	0.986	0.998
Canx	Hspa5	10116.ENSRNO	10116.ENSRNO	0	0	0	0	0.646	0.648	0	0.919	0.989
Canx	Hsp90b1	10116.ENSRNO	10116.ENSRNO	0	0	0	0	0.768	0.328	0	0.849	0.974
Canx	Ganab	10116.ENSRNO	10116.ENSRNO	0	0	0	0	0.156	0.806	0	0.491	0.909
Canx	Vcp	10116.ENSRNO	10116.ENSRNO	0	0	0	0	0.128	0.831	0	0.58	0.932
Canx	Rpn2	10116.ENSRNO	10116.ENSRNO	0	0	0	0	0.362	0.731	0	0.52	0.91
Canx	Rpn1	10116.ENSRNO	10116.ENSRNO	0	0	0	0	0.127	0.778	0	0.704	0.937
Canx	Rab10	10116.ENSRNO	10116.ENSRNO	0	0	0	0	0.855	0.471	0	0.265	0.938
Canx	P4hb	10116.ENSRNO	10116.ENSRNO	0	0	0	0	0.762	0.159	0	0.908	0.98
Capn2	Tln1	10116.ENSRNO	10116.ENSRNO	0	0	0	0	0.057	0.193	0.924	0.398	0.96
Capza1	Hsp90aa1	10116.ENSRNO	10116.ENSRNO	0	0	0	0	0.064	0	0.9	0.059	0.904
Capza1	Hsp90ab1	10116.ENSRNO	10116.ENSRNO	0	0	0	0	0.064	0	0.9	0.088	0.907
Capza1	Capza2	10116.ENSRNO	10116.ENSRNO	0	0	0	0.983	0.125	0.936	0.8	0.616	0.988
Capza2	Hsp90aa1	10116.ENSRNO	10116.ENSRNO	0	0	0	0	0.064	0	0.9	0.104	0.908
Capza2	Hsp90ab1	10116.ENSRNO	10116.ENSRNO	0	0	0	0	0.064	0	0.9	0.043	0.902
Casq2	Jph2	10116.ENSRNO	10116.ENSRNO	0	0	0	0	0.316	0	0.72	0.6	0.916
Cav1	Hsp90ab1	10116.ENSRNO	10116.ENSRNO	0	0	0	0	0	0.07	0.872	0.39	0.921
Cav1	Flna	10116.ENSRNO	10116.ENSRNO	0	0	0	0	0.101	0.059	0	0.926	0.932
Cav1	Dnm2	10116.ENSRNO	10116.ENSRNO	0	0	0	0	0	0.06	0.8	0.773	0.953
Cav1	Ctnnb1	10116.ENSRNO	10116.ENSRNO	0	0	0	0	0	0.621	0	0.909	0.964
Cav1	Rhoa	10116.ENSRNO	10116.ENSRNO	0	0	0	0	0.057	0.197	0.147	0.965	0.974
Cav1	Ptrf	10116.ENSRNO	10116.ENSRNO	0	0	0	0	0.179	0.653	0	0.916	0.974
Cav1	Hsp90aa1	10116.ENSRNO	10116.ENSRNO	0	0	0	0	0	0.07	0.872	0.858	0.981
Cav1	Nos3	10116.ENSRNO	10116.ENSRNO	0	0	0	0	0.057	0.372	0.891	0.989	0.999
Cav3	Jph2	10116.ENSRNO	10116.ENSRNO	0	0	0	0	0.292	0.114	0	0.864	0.908
Cav3	Trim72	10116.ENSRNO	10116.ENSRNO	0	0	0	0	0.221	0.114	0.8	0.977	0.996
Cav3	Nos3	10116.ENSRNO	10116.ENSRNO	0	0	0	0	0.07	0.413	0.8	0.983	0.998
Cct2	Cct6a	10116.ENSRNO	10116.ENSRNO	0	0	0.287	0.731	0.623	0.807	0.6	0.698	0.975
Cct2	Cct8	10116.ENSRNO	10116.ENSRNO	0	0	0	0.705	0.94	0.942	0.951	0.901	0.999
Cct2	Eif2s1	10116.ENSRNO	10116.ENSRNO	0	0	0	0	0.878	0	0	0.403	0.924
Cct2	Cct5	10116.ENSRNO	10116.ENSRNO	0	0	0.235	0.8	0.915	0.984	0.951	0.852	0.999
Cct2	Cct7	10116.ENSRNO	10116.ENSRNO	0	0	0.202	0.829	0.954	0.942	0.951	0.836	0.999
Cct2	Cct3	10116.ENSRNO	10116.ENSRNO	0	0	0.253	0.769	0.694	0.925	0.931	0.807	0.998
Cct2	Hsp90ab1	10116.ENSRNO	10116.ENSRNO	0	0	0	0	0.491	0.193	0.72	0.267	0.904
Cct3	Cct6a	10116.ENSRNO	10116.ENSRNO	0	0	0.263	0.808	0.563	0.696	0.6	0.781	0.952
Cct3	Cct8	10116.ENSRNO	10116.ENSRNO	0	0	0.219	0.736	0.718	0.927	0.931	0.871	0.998
Cct3	Cct5	10116.ENSRNO	10116.ENSRNO	0	0	0.299	0.83	0.722	0.928	0.931	0.874	0.998
Cct3	Cct7	10116.ENSRNO	10116.ENSRNO	0	0	0.3	0.822	0.922	0.925	0.931	0.854	0.999
Cct3	Stip1	10116.ENSRNO	10116.ENSRNO	0	0	0	0	0.636	0.044	0.72	0.307	0.923
Cct5	Cct6a	10116.ENSRNO	10116.ENSRNO	0	0	0.301	0.78	0.602	0.806	0.6	0.745	0.973
Cct5	Cct8	10116.ENSRNO	10116.ENSRNO	0	0	0.238	0.749	0.883	0.984	0.951	0.889	0.999

Cct5	Hsp90ab1	10116.ENSRNC	10116.ENSRNC	0	0	0	0	0.494	0.193	0.72	0.428	0.926
Cct5	Stip1	10116.ENSRNC	10116.ENSRNC	0	0	0	0	0.714	0.044	0.72	0.22	0.932
Cct5	Cct7	10116.ENSRNC	10116.ENSRNC	0	0	0.298	0.782	0.916	0.984	0.951	0.89	0.999
Cct6a	Cct7	10116.ENSRNC	10116.ENSRNC	0	0	0.316	0.779	0.623	0.807	0.6	0.797	0.975
Cct6a	Cct8	10116.ENSRNC	10116.ENSRNC	0	0	0.251	0.667	0.608	0.807	0.633	0.739	0.978
Cct7	Cct8	10116.ENSRNC	10116.ENSRNC	0	0	0.346	0.726	0.913	0.942	0.951	0.876	0.999
Cd34	Nt5e	10116.ENSRNC	10116.ENSRNC	0	0	0	0	0	0	0	0.929	0.929
Cd34	Pecam1	10116.ENSRNC	10116.ENSRNC	0	0	0	0	0.064	0	0	0.935	0.937
Chchd3	Samm50	10116.ENSRNC	10116.ENSRNC	0	0	0	0	0.148	0.579	0	0.797	0.921
Chchd3	Immt	10116.ENSRNC	10116.ENSRNC	0	0	0	0	0.122	0.709	0	0.823	0.95
Ckb	Ckm	10116.ENSRNC	10116.ENSRNC	0	0	0.448	0.98	0	0.833	0.969	0.62	0.994
Cltc	Hspa8	10116.ENSRNC	10116.ENSRNC	0	0	0	0	0.113	0.327	0.6	0.634	0.901
Cltc	Dnm2	10116.ENSRNC	10116.ENSRNC	0	0	0	0	0.121	0.519	0.6	0.81	0.963
Coq3	Coq5	10116.ENSRNC	10116.ENSRNC	0	0	0.237	0	0.313	0.375	0	0.957	0.984
Coq3	Coq9	10116.ENSRNC	10116.ENSRNC	0	0	0	0	0.267	0.507	0	0.948	0.979
Coq3	Coq6	10116.ENSRNC	10116.ENSRNC	0.104	0	0.343	0	0.129	0.697	0.91	0.942	0.999
Coq5	Coq9	10116.ENSRNC	10116.ENSRNC	0	0	0	0	0.145	0.456	0	0.912	0.955
Coq5	Coq6	10116.ENSRNC	10116.ENSRNC	0	0	0	0	0.139	0.193	0.99	0.921	0.999
Coq6	Coq9	10116.ENSRNC	10116.ENSRNC	0	0	0	0	0.125	0.53	0	0.941	0.974
Cox4i1	Ndufa4	10116.ENSRNC	10116.ENSRNC	0	0	0	0	0.553	0.824	0.8	0.548	0.991
Cox4i1	Ndufa5	10116.ENSRNC	10116.ENSRNC	0	0	0	0	0.34	0.81	0	0.445	0.924
Cox4i1	Ndufa12	10116.ENSRNC	10116.ENSRNC	0	0	0	0	0.663	0.814	0	0.374	0.957
Cox4i1	Ndufa6	10116.ENSRNC	10116.ENSRNC	0	0	0	0	0.76	0.696	0	0.39	0.951
Cox4i1	Ndufs3	10116.ENSRNC	10116.ENSRNC	0	0	0	0	0.765	0.819	0	0.624	0.982
Cox4i1	Ndufb9	10116.ENSRNC	10116.ENSRNC	0	0	0	0	0.806	0.853	0	0.364	0.98
Cox4i1	Ndufs1	10116.ENSRNC	10116.ENSRNC	0	0	0	0	0.333	0.852	0	0.345	0.929
Cox4i1	Ndufc2	10116.ENSRNC	10116.ENSRNC	0	0	0	0	0.542	0.72	0	0.329	0.906
Cox4i1	Uqcrh	10116.ENSRNC	10116.ENSRNC	0	0	0	0	0.683	0.813	0.947	0.492	0.998
Cox4i1	Ndufv2	10116.ENSRNC	10116.ENSRNC	0	0	0	0	0.525	0.808	0	0.465	0.947
Cox4i1	Cyc1	10116.ENSRNC	10116.ENSRNC	0	0	0	0	0.79	0.929	0.695	0.483	0.997
Cox4i1	Ndufb8	10116.ENSRNC	10116.ENSRNC	0	0	0	0	0.707	0.696	0	0.557	0.957
Cox4i1	Ndufb10	10116.ENSRNC	10116.ENSRNC	0	0	0	0	0.83	0.804	0	0.37	0.977
Cox4i1	Uqcrc2	10116.ENSRNC	10116.ENSRNC	0	0	0	0	0.577	0.929	0.923	0.684	0.999
Cox4i1	Ndufa10	10116.ENSRNC	10116.ENSRNC	0	0	0	0	0.586	0.929	0.181	0.302	0.98
Cox4i1	Cox5b	10116.ENSRNC	10116.ENSRNC	0	0	0	0	0.877	0.546	0.882	0.727	0.997
Cox4i1	Ndufa2	10116.ENSRNC	10116.ENSRNC	0	0	0	0	0.514	0.812	0	0.473	0.947
Cox4i1	Ndufa11	10116.ENSRNC	10116.ENSRNC	0	0	0	0	0.635	0.696	0	0.257	0.91
Cox4i1	Ndufab1	10116.ENSRNC	10116.ENSRNC	0	0	0	0	0.466	0.81	0	0.295	0.922
Cox4i1	Ndufv1	10116.ENSRNC	10116.ENSRNC	0	0	0	0	0.601	0.696	0	0.416	0.923
Cox4i1	Ndufb6	10116.ENSRNC	10116.ENSRNC	0	0	0	0	0.532	0.808	0	0.328	0.934
Cox4i1	Ndufb7	10116.ENSRNC	10116.ENSRNC	0	0	0	0	0.673	0.807	0	0.331	0.954
Cox4i1	Ndufs7	10116.ENSRNC	10116.ENSRNC	0	0	0	0	0.689	0.808	0	0.356	0.958
Cox4i1	Uqcrb	10116.ENSRNC	10116.ENSRNC	0	0	0	0	0.554	0.543	0.663	0.479	0.959
Cox4i1	Ndufa8	10116.ENSRNC	10116.ENSRNC	0	0	0	0	0.694	0.809	0	0.362	0.959
Cox4i1	Ndufs2	10116.ENSRNC	10116.ENSRNC	0	0	0	0	0.398	0.929	0	0.475	0.975
Cox4i1	Cox6b1	10116.ENSRNC	10116.ENSRNC	0	0	0	0	0.795	0.813	0.191	0.647	0.987
Cox4i1	Ndufa9	10116.ENSRNC	10116.ENSRNC	0	0	0	0	0.582	0.928	0	0.734	0.991
Cox4i1	Uqcrc1	10116.ENSRNC	10116.ENSRNC	0	0	0	0	0.734	0.852	0.841	0.624	0.997
Cox4i1	Uqcrfs1	10116.ENSRNC	10116.ENSRNC	0	0	0	0	0.734	0.852	0.912	0.579	0.998
Cox4i1	Cox5a	10116.ENSRNC	10116.ENSRNC	0	0	0	0	0.699	0.939	0.835	0.792	0.999
Cox5a	Mdh2	10116.ENSRNC	10116.ENSRNC	0	0	0	0	0.738	0.499	0	0.307	0.901
Cox5a	Ndufa4	10116.ENSRNC	10116.ENSRNC	0	0	0	0	0.567	0.825	0.8	0.491	0.991
Cox5a	Ndufa5	10116.ENSRNC	10116.ENSRNC	0	0	0	0	0.724	0.696	0	0.498	0.954
Cox5a	Ndufa12	10116.ENSRNC	10116.ENSRNC	0	0	0	0	0.795	0.924	0	0.307	0.988
Cox5a	Sdhb	10116.ENSRNC	10116.ENSRNC	0	0	0	0	0.726	0.453	0	0.675	0.947
Cox5a	Ndufa6	10116.ENSRNC	10116.ENSRNC	0	0	0	0	0.818	0.696	0	0.622	0.977
Cox5a	Ndufs3	10116.ENSRNC	10116.ENSRNC	0	0	0	0	0.795	0.811	0	0.535	0.98

Cox5a	Ndufb9	10116.ENSRNO	10116.ENSRNO	0	0	0	0	0.789	0.924	0	0.471	0.99
Cox5a	Ndufs4	10116.ENSRNO	10116.ENSRNO	0	0	0	0	0.716	0.917	0	0.312	0.982
Cox5a	Ndufs1	10116.ENSRNO	10116.ENSRNO	0	0	0	0	0.177	0.803	0	0.49	0.91
Cox5a	Ndufc2	10116.ENSRNO	10116.ENSRNO	0	0	0	0	0.844	0.923	0	0.41	0.992
Cox5a	Uqcrh	10116.ENSRNO	10116.ENSRNO	0	0	0	0	0.663	0.871	0.975	0.738	0.999
Cox5a	Ndufv2	10116.ENSRNO	10116.ENSRNO	0	0	0	0	0.723	0.924	0	0.537	0.989
Cox5a	Cyc1	10116.ENSRNO	10116.ENSRNO	0	0	0	0	0.784	0.927	0.789	0.731	0.998
Cox5a	Ndufb8	10116.ENSRNO	10116.ENSRNO	0	0	0	0	0.785	0.696	0	0.564	0.969
Cox5a	Ndufb10	10116.ENSRNO	10116.ENSRNO	0	0	0	0	0.757	0.804	0	0.541	0.976
Cox5a	Uqcrc2	10116.ENSRNO	10116.ENSRNO	0	0	0	0	0.78	0.926	0.969	0.791	0.999
Cox5a	Ndufa10	10116.ENSRNO	10116.ENSRNO	0	0	0	0	0.646	0.924	0	0.582	0.987
Cox5a	Cox5b	10116.ENSRNO	10116.ENSRNO	0	0	0	0	0.874	0.808	0.969	0.886	0.999
Cox5a	Ndufa2	10116.ENSRNO	10116.ENSRNO	0	0	0	0	0.313	0.807	0	0.47	0.923
Cox5a	Ndufab1	10116.ENSRNO	10116.ENSRNO	0	0	0	0	0.72	0.807	0	0.586	0.975
Cox5a	Ndufv1	10116.ENSRNO	10116.ENSRNO	0	0	0	0	0.344	0.924	0	0.318	0.963
Cox5a	Uqcrfs1	10116.ENSRNO	10116.ENSRNO	0	0	0	0	0.833	0.927	0.971	0.674	0.999
Cox5a	Ndufa11	10116.ENSRNO	10116.ENSRNO	0	0	0	0	0.739	0.536	0	0.398	0.92
Cox5a	Ndufs5	10116.ENSRNO	10116.ENSRNO	0	0	0	0	0.586	0.696	0	0.423	0.921
Cox5a	rCG_38845	10116.ENSRNO	10116.ENSRNO	0	0	0	0	0.672	0.804	0	0.126	0.938
Cox5a	Ndufb7	10116.ENSRNO	10116.ENSRNO	0	0	0	0	0.73	0.696	0	0.494	0.954
Cox5a	Ndufa8	10116.ENSRNO	10116.ENSRNO	0	0	0	0	0.728	0.696	0	0.495	0.954
Cox5a	Ndufa9	10116.ENSRNO	10116.ENSRNO	0	0	0	0	0.731	0.81	0	0.488	0.971
Cox5a	Ndufs7	10116.ENSRNO	10116.ENSRNO	0	0	0	0	0.599	0.923	0	0.394	0.979
Cox5a	Ndufs2	10116.ENSRNO	10116.ENSRNO	0	0	0	0	0.583	0.924	0	0.465	0.981
Cox5a	Ndufb6	10116.ENSRNO	10116.ENSRNO	0	0	0	0	0.821	0.86	0	0.461	0.985
Cox5a	Cox6b1	10116.ENSRNO	10116.ENSRNO	0	0	0	0	0.686	0.819	0.299	0.673	0.985
Cox5a	Uqcrb	10116.ENSRNO	10116.ENSRNO	0	0	0	0	0.697	0.807	0.814	0.684	0.996
Cox5a	Uqcrc1	10116.ENSRNO	10116.ENSRNO	0	0	0	0	0.667	0.81	0.903	0.8	0.998
Cox5b	Ndufb4	10116.ENSRNO	10116.ENSRNO	0	0	0	0	0.866	0	0	0.298	0.902
Cox5b	Ndufa4	10116.ENSRNO	10116.ENSRNO	0	0	0	0	0.778	0.529	0.8	0.547	0.989
Cox5b	Ndufa5	10116.ENSRNO	10116.ENSRNO	0	0	0	0	0.76	0.696	0	0.43	0.954
Cox5b	Ndufa12	10116.ENSRNO	10116.ENSRNO	0	0	0	0	0.895	0.605	0	0.426	0.974
Cox5b	Sdhb	10116.ENSRNO	10116.ENSRNO	0	0	0	0	0.818	0.48	0	0.52	0.95
Cox5b	Ndufs3	10116.ENSRNO	10116.ENSRNO	0	0	0	0	0.839	0	0	0.637	0.939
Cox5b	Ndufb9	10116.ENSRNO	10116.ENSRNO	0	0	0	0	0.913	0.6	0	0.28	0.972
Cox5b	LOC679794	10116.ENSRNO	10116.ENSRNO	0	0	0	0	0.643	0.458	0	0.554	0.906
Cox5b	Ndufs4	10116.ENSRNO	10116.ENSRNO	0	0	0	0	0.574	0.696	0	0.289	0.9
Cox5b	Ndufc2	10116.ENSRNO	10116.ENSRNO	0	0	0	0	0.75	0.6	0	0.367	0.931
Cox5b	Uqcrh	10116.ENSRNO	10116.ENSRNO	0	0	0	0	0.839	0.789	0.914	0.587	0.998
Cox5b	Ndufv2	10116.ENSRNO	10116.ENSRNO	0	0	0	0	0.74	0.445	0	0.414	0.908
Cox5b	Cyc1	10116.ENSRNO	10116.ENSRNO	0	0	0	0	0.884	0.809	0.9	0.656	0.999
Cox5b	Ndufb8	10116.ENSRNO	10116.ENSRNO	0	0	0	0	0.81	0	0	0.532	0.907
Cox5b	Ndufb10	10116.ENSRNO	10116.ENSRNO	0	0	0	0	0.921	0.52	0	0.275	0.97
Cox5b	Uqcrc2	10116.ENSRNO	10116.ENSRNO	0	0	0	0	0.678	0.808	0.827	0.642	0.995
Cox5b	Ndufa2	10116.ENSRNO	10116.ENSRNO	0	0	0	0	0.755	0.54	0	0.286	0.912
Cox5b	Ndufs7	10116.ENSRNO	10116.ENSRNO	0	0	0	0	0.888	0	0	0.505	0.942
Cox5b	Ndufa8	10116.ENSRNO	10116.ENSRNO	0	0	0	0	0.875	0	0	0.589	0.946
Cox5b	Ndufs2	10116.ENSRNO	10116.ENSRNO	0	0	0	0	0.736	0.696	0	0.392	0.947
Cox5b	Ndufa11	10116.ENSRNO	10116.ENSRNO	0	0	0	0	0.898	0.379	0	0.448	0.962
Cox5b	Ndufb6	10116.ENSRNO	10116.ENSRNO	0	0	0	0	0.904	0.6	0	0.238	0.968
Cox5b	Ndufb7	10116.ENSRNO	10116.ENSRNO	0	0	0	0	0.908	0.595	0	0.653	0.986
Cox5b	Uqcrc1	10116.ENSRNO	10116.ENSRNO	0	0	0	0	0.795	0.685	0.709	0.709	0.993
Cox5b	Uqcrb	10116.ENSRNO	10116.ENSRNO	0	0	0	0	0.889	0.696	0.74	0.579	0.995
Cox5b	Cox6b1	10116.ENSRNO	10116.ENSRNO	0	0	0	0	0.935	0.774	0.299	0.653	0.995
Cox5b	Uqcrfs1	10116.ENSRNO	10116.ENSRNO	0	0	0	0	0.925	0.855	0.903	0.714	0.999
Cox6b1	Ndufb4	10116.ENSRNO	10116.ENSRNO	0	0	0	0	0.816	0.443	0	0.127	0.902
Cox6b1	Ndufa4	10116.ENSRNO	10116.ENSRNO	0	0	0	0	0.87	0.714	0	0.298	0.971

Cox6b1	Ndufa5	10116.ENSRNC	10116.ENSRNC	0	0	0	0	0.642	0.728	0	0.451	0.942
Cox6b1	Ndufa12	10116.ENSRNC	10116.ENSRNC	0	0	0	0	0.909	0.728	0	0.415	0.984
Cox6b1	Sdhb	10116.ENSRNC	10116.ENSRNC	0	0	0	0	0.744	0.551	0	0.387	0.923
Cox6b1	Ndufa6	10116.ENSRNC	10116.ENSRNC	0	0	0	0	0.775	0.712	0	0.136	0.939
Cox6b1	Ndufs3	10116.ENSRNC	10116.ENSRNC	0	0	0	0	0.733	0.697	0	0.263	0.935
Cox6b1	Ndufb9	10116.ENSRNC	10116.ENSRNC	0	0	0	0	0.914	0.722	0	0.412	0.984
Cox6b1	Ndufc2	10116.ENSRNC	10116.ENSRNC	0	0	0	0	0.654	0.703	0	0.358	0.928
Cox6b1	Uqcrh	10116.ENSRNC	10116.ENSRNC	0	0	0	0	0.911	0.804	0.776	0.701	0.998
Cox6b1	Ndufv2	10116.ENSRNC	10116.ENSRNC	0	0	0	0	0.661	0.696	0	0.374	0.93
Cox6b1	Cyc1	10116.ENSRNC	10116.ENSRNC	0	0	0	0	0.818	0.774	0.776	0.433	0.994
Cox6b1	Ndufb10	10116.ENSRNC	10116.ENSRNC	0	0	0	0	0.901	0.541	0	0.198	0.96
Cox6b1	Uqcrc2	10116.ENSRNC	10116.ENSRNC	0	0	0	0	0.41	0.803	0.762	0.349	0.979
Cox6b1	Ndufa2	10116.ENSRNC	10116.ENSRNC	0	0	0	0	0.834	0.616	0	0.271	0.949
Cox6b1	Uqcrfs1	10116.ENSRNC	10116.ENSRNC	0	0	0	0	0.727	0.809	0.776	0.426	0.992
Cox6b1	Ndufa9	10116.ENSRNC	10116.ENSRNC	0	0	0	0	0.527	0.722	0	0.346	0.906
Cox6b1	Ndufs5	10116.ENSRNC	10116.ENSRNC	0	0	0	0	0.791	0.53	0	0.147	0.909
Cox6b1	Ndufb6	10116.ENSRNC	10116.ENSRNC	0	0	0	0	0.706	0.696	0	0.229	0.925
Cox6b1	Ndufa11	10116.ENSRNC	10116.ENSRNC	0	0	0	0	0.848	0.543	0	0.168	0.937
Cox6b1	Ndufs7	10116.ENSRNC	10116.ENSRNC	0	0	0	0	0.712	0.728	0	0.279	0.938
Cox6b1	Uqcrc1	10116.ENSRNC	10116.ENSRNC	0	0	0	0	0.588	0.709	0.416	0.334	0.947
Cox6b1	Ndufa8	10116.ENSRNC	10116.ENSRNC	0	0	0	0	0.793	0.696	0	0.359	0.956
Cox6b1	Ndufb7	10116.ENSRNC	10116.ENSRNC	0	0	0	0	0.917	0.683	0	0.309	0.98
Cox6b1	Uqcrb	10116.ENSRNC	10116.ENSRNC	0	0	0	0	0.893	0.629	0.67	0.606	0.994
Cpt1b	Cpt2	10116.ENSRNC	10116.ENSRNC	0	0	0.419	0.699	0.156	0	0.9	0.864	0.942
Cpt2	Ech1	10116.ENSRNC	10116.ENSRNC	0	0	0	0	0.529	0.504	0	0.614	0.902
Cpt2	Crat	10116.ENSRNC	10116.ENSRNC	0	0	0.4	0.773	0.655	0.435	0.663	0.748	0.945
Cs	Mdh2	10116.ENSRNC	10116.ENSRNC	0.094	0	0	0	0.607	0.853	0.994	0.848	0.999
Cs	Sdhc	10116.ENSRNC	10116.ENSRNC	0.155	0	0	0	0.576	0	0.67	0.651	0.953
Cs	L2hgdh	10116.ENSRNC	10116.ENSRNC	0.116	0	0	0	0.409	0	0.886	0.41	0.96
Cs	Dlst	10116.ENSRNC	10116.ENSRNC	0.107	0	0	0	0.604	0.434	0.34	0.61	0.939
Cs	Pdhb	10116.ENSRNC	10116.ENSRNC	0.086	0	0	0	0.233	0.924	0.67	0.601	0.991
Cs	Sdhb	10116.ENSRNC	10116.ENSRNC	0.155	0	0	0	0.575	0.549	0.684	0.759	0.985
Cs	Mdh1	10116.ENSRNC	10116.ENSRNC	0.094	0	0	0	0.409	0.371	0.99	0.848	0.999
Cs	Glud1	10116.ENSRNC	10116.ENSRNC	0	0	0	0	0.209	0.136	0.8	0.653	0.946
Cs	Hadh	10116.ENSRNC	10116.ENSRNC	0.087	0	0	0	0.077	0.054	0.66	0.759	0.922
Cs	Got2	10116.ENSRNC	10116.ENSRNC	0	0	0	0	0.117	0	0.8	0.578	0.919
Cs	Ldha	10116.ENSRNC	10116.ENSRNC	0.094	0	0	0	0.387	0.371	0.66	0.595	0.943
Cs	Ldhb	10116.ENSRNC	10116.ENSRNC	0.094	0	0	0	0.401	0.371	0.66	0.572	0.941
Cs	Sdha	10116.ENSRNC	10116.ENSRNC	0.155	0	0	0	0.569	0.518	0.704	0.761	0.985
Cs	Me3	10116.ENSRNC	10116.ENSRNC	0.119	0	0	0	0.135	0.045	0.931	0.282	0.957
Cs	Pc	10116.ENSRNC	10116.ENSRNC	0.131	0	0	0	0.064	0.043	0.9	0.84	0.985
Cs	Dlat	10116.ENSRNC	10116.ENSRNC	0.107	0	0	0	0.416	0.696	0.982	0.596	0.998
Cs	Suclg2	10116.ENSRNC	10116.ENSRNC	0.12	0	0	0	0.382	0.499	0.44	0.528	0.915
Cs	Uqcrc1	10116.ENSRNC	10116.ENSRNC	0	0	0	0	0.503	0.797	0	0.439	0.938
Ctnnb1	Ep300	10116.ENSRNC	10116.ENSRNC	0	0	0	0	0.05	0.855	0.924	0.942	0.999
Ctnnb1	Vcl	10116.ENSRNC	10116.ENSRNC	0	0	0	0	0.064	0.485	0.637	0.98	0.996
Ctnnb1	Rhoa	10116.ENSRNC	10116.ENSRNC	0	0	0	0	0.062	0.061	0.615	0.808	0.926
Ctnnb1	Rock1	10116.ENSRNC	10116.ENSRNC	0	0	0	0	0.064	0.136	0.819	0.587	0.931
Ctnnb1	Ywhaz	10116.ENSRNC	10116.ENSRNC	0	0	0	0	0.064	0.506	0.922	0.706	0.988
Ctnnb1	Pecam1	10116.ENSRNC	10116.ENSRNC	0	0	0	0	0	0.487	0.8	0.903	0.989
Ctsb	Lamp1	10116.ENSRNC	10116.ENSRNC	0	0	0	0	0.533	0.164	0	0.802	0.916
Ctsb	Ctsd	10116.ENSRNC	10116.ENSRNC	0	0	0	0	0.249	0.696	0.3	0.896	0.981
Cyb5a	Por	10116.ENSRNC	10116.ENSRNC	0	0	0	0	0.085	0.368	0	0.952	0.97
Cyb5a	Cyb5r3	10116.ENSRNC	10116.ENSRNC	0	0	0	0	0.109	0.486	0.9	0.835	0.991
Cyb5r1	Hba1	10116.ENSRNC	10116.ENSRNC	0	0	0	0	0.064	0.131	0.9	0.063	0.913
Cyc1	Mdh2	10116.ENSRNC	10116.ENSRNC	0	0	0	0	0.946	0.492	0	0.291	0.979
Cyc1	Sdhc	10116.ENSRNC	10116.ENSRNC	0	0	0	0	0.667	0.119	0.894	0.473	0.981

Cyc1	Slc25a11	10116.ENSRNO	10116.ENSRNO	0	0	0	0	0.689	0.434	0	0.528	0.909
Cyc1	Ndufa5	10116.ENSRNO	10116.ENSRNO	0	0	0	0	0.409	0.853	0.783	0.338	0.985
Cyc1	Ndufa12	10116.ENSRNO	10116.ENSRNO	0	0	0	0	0.777	0.93	0	0.375	0.989
Cyc1	Sdhb	10116.ENSRNO	10116.ENSRNO	0	0	0	0	0.842	0.462	0.9	0.535	0.995
Cyc1	Ndufa6	10116.ENSRNO	10116.ENSRNO	0	0	0	0	0.515	0.855	0	0.168	0.936
Cyc1	Slc25a3	10116.ENSRNO	10116.ENSRNO	0	0	0	0	0.874	0.138	0	0.207	0.906
Cyc1	Ndufs3	10116.ENSRNO	10116.ENSRNO	0	0	0	0	0.898	0.932	0.783	0.477	0.999
Cyc1	Ndufb9	10116.ENSRNO	10116.ENSRNO	0	0	0	0	0.931	0.929	0.783	0.199	0.999
Cyc1	LOC679794	10116.ENSRNO	10116.ENSRNO	0	0	0	0	0.69	0.524	0.27	0.572	0.947
Cyc1	Ndufs4	10116.ENSRNO	10116.ENSRNO	0	0	0	0	0.592	0.939	0.783	0.175	0.994
Cyc1	Ndufs1	10116.ENSRNO	10116.ENSRNO	0	0	0	0	0.744	0.925	0.898	0.345	0.998
Cyc1	Ndufc2	10116.ENSRNO	10116.ENSRNO	0	0	0	0	0.508	0.92	0	0.52	0.979
Cyc1	Uqcrh	10116.ENSRNO	10116.ENSRNO	0	0	0	0	0.788	0.984	0.979	0.688	0.999
Cyc1	Ndufv2	10116.ENSRNO	10116.ENSRNO	0	0	0	0	0.875	0.93	0.898	0.261	0.999
Cyc1	Pmpca	10116.ENSRNO	10116.ENSRNO	0	0	0	0	0.582	0.523	0.349	0.323	0.9
Cyc1	rCG_38845	10116.ENSRNO	10116.ENSRNO	0	0	0	0	0.657	0.776	0	0.091	0.924
Cyc1	Ndufb10	10116.ENSRNO	10116.ENSRNO	0	0	0	0	0.718	0.822	0	0.089	0.95
Cyc1	Ndufa11	10116.ENSRNO	10116.ENSRNO	0	0	0	0	0.796	0.696	0	0.342	0.955
Cyc1	Ndufab1	10116.ENSRNO	10116.ENSRNO	0	0	0	0	0.576	0.815	0.301	0.342	0.959
Cyc1	Ndufa2	10116.ENSRNO	10116.ENSRNO	0	0	0	0	0.27	0.813	0.67	0.225	0.96
Cyc1	Ndufb8	10116.ENSRNO	10116.ENSRNO	0	0	0	0	0.751	0.81	0	0.309	0.964
Cyc1	Ndufb6	10116.ENSRNO	10116.ENSRNO	0	0	0	0	0.727	0.924	0	0.228	0.982
Cyc1	Ndufa10	10116.ENSRNO	10116.ENSRNO	0	0	0	0	0.698	0.93	0	0.496	0.988
Cyc1	Ndufb7	10116.ENSRNO	10116.ENSRNO	0	0	0	0	0.83	0.93	0	0.341	0.991
Cyc1	Sdha	10116.ENSRNO	10116.ENSRNO	0	0	0	0	0.73	0.477	0.893	0.652	0.994
Cyc1	Ndufa9	10116.ENSRNO	10116.ENSRNO	0	0	0	0	0.873	0.864	0.67	0.414	0.996
Cyc1	Ndufa8	10116.ENSRNO	10116.ENSRNO	0	0	0	0	0.911	0.855	0.783	0.298	0.997
Cyc1	Uqcrb	10116.ENSRNO	10116.ENSRNO	0	0	0	0	0.703	0.812	0.961	0.587	0.998
Cyc1	Uqcrc1	10116.ENSRNO	10116.ENSRNO	0	0	0	0	0.923	0.984	0.961	0.716	0.999
Cyc1	Ndufv1	10116.ENSRNO	10116.ENSRNO	0	0	0	0	0.927	0.93	0.909	0.652	0.999
Cyc1	Uqcrc2	10116.ENSRNO	10116.ENSRNO	0	0	0	0	0.812	0.984	0.978	0.743	0.999
Cyc1	Uqcrfs1	10116.ENSRNO	10116.ENSRNO	0.155	0	0	0	0.927	0.984	0.972	0.844	0.999
Cyc1	Ndufs2	10116.ENSRNO	10116.ENSRNO	0	0	0	0	0.909	0.93	0.783	0.53	0.999
Cyc1	Ndufs7	10116.ENSRNO	10116.ENSRNO	0	0	0	0	0.897	0.93	0.898	0.581	0.999
D2hgdh	L2hgdh	10116.ENSRNO	10116.ENSRNO	0.109	0	0	0	0.064	0	0	0.947	0.952
Dag1	Lama2	10116.ENSRNO	10116.ENSRNO	0	0	0	0	0.058	0.968	0.924	0.687	0.999
Dbt	Pdhb	10116.ENSRNO	10116.ENSRNO	0.095	0	0.303	0	0.467	0.45	0.886	0.344	0.983
Dbt	Hibadh	10116.ENSRNO	10116.ENSRNO	0.081	0	0	0	0.134	0	0.8	0.515	0.912
Dbt	Ivd	10116.ENSRNO	10116.ENSRNO	0.045	0	0	0	0.55	0.129	0.994	0.655	0.999
Dbt	Gcsh	10116.ENSRNO	10116.ENSRNO	0.091	0	0	0	0.088	0.301	0.9	0.236	0.947
Dbt	Pccb	10116.ENSRNO	10116.ENSRNO	0.045	0	0	0	0.396	0	0.99	0.631	0.997
Ddx1	RGD1304704	10116.ENSRNO	10116.ENSRNO	0	0	0	0	0.242	0.811	0.67	0.633	0.98
Decr1	Ehhadh	10116.ENSRNO	10116.ENSRNO	0.044	0	0	0	0.167	0.234	0.911	0.637	0.976
Decr1	Ech1	10116.ENSRNO	10116.ENSRNO	0	0	0	0	0.641	0.5	0.288	0.56	0.936
Decr1	Eci2	10116.ENSRNO	10116.ENSRNO	0.044	0	0	0	0.182	0.46	0.83	0.584	0.964
Des	Vim	10116.ENSRNO	10116.ENSRNO	0	0	0	0.961	0.086	0.696	0.8	0	0.939
Dhrs4	Eci2	10116.ENSRNO	10116.ENSRNO	0.044	0	0	0	0.145	0.46	0.821	0.33	0.937
Dlat	Pdk2	10116.ENSRNO	10116.ENSRNO	0	0	0	0	0.081	0.696	0.9	0.349	0.979
Dlat	Dlst	10116.ENSRNO	10116.ENSRNO	0	0	0.419	0.715	0.56	0.809	0.5	0.869	0.969
Dlat	Pdhb	10116.ENSRNO	10116.ENSRNO	0.095	0.002	0.435	0	0.814	0.819	0.982	0.843	0.999
Dlat	Pdk4	10116.ENSRNO	10116.ENSRNO	0	0	0	0	0.064	0.521	0.9	0.188	0.958
Dlat	Gcsh	10116.ENSRNO	10116.ENSRNO	0.091	0	0	0	0.061	0.301	0.9	0.536	0.967
Dlat	Pc	10116.ENSRNO	10116.ENSRNO	0.076	0	0	0	0.072	0	0.8	0.55	0.912
Dlat	Suclg2	10116.ENSRNO	10116.ENSRNO	0.089	0	0	0	0.288	0.193	0.835	0.606	0.96
Dlst	Gcdh	10116.ENSRNO	10116.ENSRNO	0.045	0	0	0	0.064	0.129	0.915	0.174	0.935
Dlst	Sdha	10116.ENSRNO	10116.ENSRNO	0.109	0	0	0	0.733	0.471	0.274	0.467	0.942
Dlst	Sdhb	10116.ENSRNO	10116.ENSRNO	0.109	0	0	0	0.709	0.696	0.189	0.396	0.954

Dlst	Gcsh	10116.ENSRNO	10116.ENSRNO	0.091	0	0	0	0.06	0.716	0.9	0.31	0.98
Dlst	Pdhb	10116.ENSRNO	10116.ENSRNO	0.095	0.055	0.317	0	0.489	0.45	0.886	0.515	0.988
Dlst	Suclg2	10116.ENSRNO	10116.ENSRNO	0.089	0	0	0	0.288	0.193	0.99	0.215	0.995
Dnaja3	Hspa8	10116.ENSRNO	10116.ENSRNO	0.044	0	0.393	0	0.204	0.507	0.51	0.475	0.927
Dnaja3	Hspa9	10116.ENSRNO	10116.ENSRNO	0.044	0	0.406	0	0.155	0.809	0.194	0.739	0.976
Dnajb11	Dnajc3	10116.ENSRNO	10116.ENSRNO	0	0	0	0.599	0.886	0	0	0.732	0.919
Dnajb11	Hsp90b1	10116.ENSRNO	10116.ENSRNO	0	0	0	0	0.865	0.322	0.72	0.71	0.991
Dnajb11	Hspa5	10116.ENSRNO	10116.ENSRNO	0.044	0	0.392	0	0.849	0.88	0.764	0.713	0.999
Dnajb4	Hspa8	10116.ENSRNO	10116.ENSRNO	0.044	0	0.436	0	0.403	0.459	0.194	0.451	0.905
Dnajb4	Stip1	10116.ENSRNO	10116.ENSRNO	0	0	0	0	0.576	0.696	0	0.4	0.915
Dnajc3	Hsp90b1	10116.ENSRNO	10116.ENSRNO	0	0	0	0	0.786	0.178	0	0.709	0.944
Dnajc3	Hspa5	10116.ENSRNO	10116.ENSRNO	0.044	0	0	0	0.74	0.164	0.194	0.844	0.969
ENSRNOG0000	Ehhadh	10116.ENSRNO	10116.ENSRNO	0	0	0.216	0.739	0.11	0.318	0.982	0.644	0.99
ENSRNOG0000	Gcdh	10116.ENSRNO	10116.ENSRNO	0.052	0	0.212	0	0.26	0.164	0.982	0.329	0.993
ENSRNOG0000	Ivd	10116.ENSRNO	10116.ENSRNO	0.052	0	0	0	0.123	0.164	0.924	0.255	0.953
ENSRNOG0000	Hadh	10116.ENSRNO	10116.ENSRNO	0.084	0.679	0.265	0	0.578	0.396	0.982	0.666	0.999
ENSRNOG0000	Mccc1	10116.ENSRNO	10116.ENSRNO	0.084	0	0	0	0.135	0.104	0.916	0.447	0.961
ENSRNOG0000	Mccc2	10116.ENSRNO	10116.ENSRNO	0.084	0	0	0	0.555	0	0.916	0.331	0.974
ENSRNOG0000	Ech1	10116.ENSRNO	10116.ENSRNO	0	0	0.333	0.714	0.214	0.368	0.764	0.543	0.901
ENSRNOG0000	Mecr	10116.ENSRNO	10116.ENSRNO	0.053	0	0	0	0.147	0.079	0.912	0.302	0.946
ENSRNOG0000	Hadha	10116.ENSRNO	10116.ENSRNO	0	0	0	0.701	0.134	0.318	0.984	0.684	0.991
ENSRNOG0000	Hibch	10116.ENSRNO	10116.ENSRNO	0	0	0.378	0.698	0.124	0.435	0.986	0.68	0.994
ENSRNOG0000	Hsd17b10	10116.ENSRNO	10116.ENSRNO	0	0	0	0	0.674	0.134	0.986	0.584	0.998
ENSRNOG0000	Vdac1	10116.ENSRNO	10116.ENSRNO	0	0	0	0	0.064	0.071	0.8	0.736	0.948
ENSRNOG0000	Hsp90aa1	10116.ENSRNO	10116.ENSRNO	0	0	0	0	0.454	0.422	0.6	0.767	0.966
ENSRNOG0000	Vdac2	10116.ENSRNO	10116.ENSRNO	0	0	0	0	0.064	0.071	0.8	0.523	0.906
ENSRNOG0000	Vdac3	10116.ENSRNO	10116.ENSRNO	0	0	0	0	0.124	0.071	0.8	0.499	0.907
Ech1	Ehhadh	10116.ENSRNO	10116.ENSRNO	0	0	0	0.616	0.281	0.261	0.814	0.755	0.923
Ech1	Gcdh	10116.ENSRNO	10116.ENSRNO	0	0	0	0	0.177	0.139	0.783	0.437	0.902
Ech1	Hadh	10116.ENSRNO	10116.ENSRNO	0	0.046	0	0	0.277	0.497	0.898	0.609	0.983
Ech1	Pecr	10116.ENSRNO	10116.ENSRNO	0	0	0	0	0.124	0.502	0.48	0.629	0.904
Ech1	Eci2	10116.ENSRNO	10116.ENSRNO	0	0	0.295	0.63	0.74	0.659	0.427	0.768	0.964
Ech1	Hadha	10116.ENSRNO	10116.ENSRNO	0	0	0	0.657	0.24	0.5	0.746	0.577	0.915
Eci2	Ehhadh	10116.ENSRNO	10116.ENSRNO	0	0	0.234	0.566	0.136	0.375	0.885	0.732	0.957
Eci2	Scp2	10116.ENSRNO	10116.ENSRNO	0	0	0	0	0.559	0.933	0.67	0.291	0.992
Eci2	Pecr	10116.ENSRNO	10116.ENSRNO	0.044	0	0	0	0.087	0.46	0.821	0.736	0.973
Eci2	Hsd17b4	10116.ENSRNO	10116.ENSRNO	0.084	0	0	0	0.712	0.519	0.507	0.523	0.964
Eci2	Hadha	10116.ENSRNO	10116.ENSRNO	0	0	0	0	0.077	0.369	0.843	0.62	0.96
Eci2	Hsdl2	10116.ENSRNO	10116.ENSRNO	0.044	0	0	0	0.461	0.926	0.755	0.489	0.994
Ecsit	Ndufs3	10116.ENSRNO	10116.ENSRNO	0	0	0	0	0.392	0.965	0.6	0.272	0.992
Ecsit	Ndufs1	10116.ENSRNO	10116.ENSRNO	0	0	0	0	0.109	0.923	0	0.214	0.941
Ecsit	Ndufa11	10116.ENSRNO	10116.ENSRNO	0	0	0	0	0.659	0	0.6	0.379	0.908
Ecsit	Ndufa8	10116.ENSRNO	10116.ENSRNO	0	0	0	0	0.658	0.482	0.6	0.066	0.925
Ecsit	Ndufs7	10116.ENSRNO	10116.ENSRNO	0	0	0	0	0.755	0.116	0.6	0.343	0.935
Ecsit	Ndufaf3	10116.ENSRNO	10116.ENSRNO	0	0	0	0	0.244	0.801	0.6	0.591	0.972
Ecsit	rCG_38845	10116.ENSRNO	10116.ENSRNO	0	0	0	0	0.567	0.927	0.6	0.29	0.989
Ecsit	Ndufs2	10116.ENSRNO	10116.ENSRNO	0	0	0	0	0.219	0.962	0.6	0.362	0.991
Eef1d	Rps5	10116.ENSRNO	10116.ENSRNO	0	0	0	0	0.805	0.817	0	0.196	0.968
Eef2	Tpt1	10116.ENSRNO	10116.ENSRNO	0	0	0	0	0.62	0.696	0	0.436	0.929
Eef2	Rplp0	10116.ENSRNO	10116.ENSRNO	0.084	0	0	0	0.648	0.696	0	0.466	0.94
Eef2	Gnb2l1	10116.ENSRNO	10116.ENSRNO	0	0	0	0	0.873	0.932	0	0.392	0.994
Eef2	Mrps7	10116.ENSRNO	10116.ENSRNO	0.098	0	0	0	0.625	0.809	0	0.268	0.946
Eef2	Rpl19	10116.ENSRNO	10116.ENSRNO	0	0	0	0	0.572	0.518	0.72	0.142	0.943
Eef2	Rpl3l	10116.ENSRNO	10116.ENSRNO	0.086	0	0	0	0.623	0.73	0	0.289	0.924
Eef2	Rps23	10116.ENSRNO	10116.ENSRNO	0.086	0	0	0	0.476	0.73	0.72	0.211	0.966
Eef2	Rps5	10116.ENSRNO	10116.ENSRNO	0.098	0	0	0	0.884	0.932	0	0.445	0.995
Eef2	Rpl6	10116.ENSRNO	10116.ENSRNO	0.045	0	0	0	0.581	0.644	0.72	0.209	0.962

Ehhadh	Hibadh	10116.ENSRNO	10116.ENSRNO	0.083	0	0	0	0.135	0.193	0.842	0.27	0.913
Ehhadh	Oxct1	10116.ENSRNO	10116.ENSRNO	0.09	0	0	0	0.064	0.054	0.9	0.309	0.934
Ehhadh	Hsdl2	10116.ENSRNO	10116.ENSRNO	0.044	0	0	0	0.198	0.502	0.829	0.168	0.935
Ehhadh	Mccc2	10116.ENSRNO	10116.ENSRNO	0.084	0	0	0	0.152	0.044	0.916	0.144	0.937
Ehhadh	Mccc1	10116.ENSRNO	10116.ENSRNO	0.084	0	0	0	0.092	0.104	0.916	0.221	0.942
Ehhadh	Ivd	10116.ENSRNO	10116.ENSRNO	0.052	0	0	0	0.151	0.193	0.922	0.359	0.961
Ehhadh	Gcdh	10116.ENSRNO	10116.ENSRNO	0.052	0	0.2	0	0.208	0.164	0.922	0.341	0.968
Ehhadh	Scp2	10116.ENSRNO	10116.ENSRNO	0	0	0	0	0.233	0.52	0.928	0.552	0.986
Ehhadh	Hadha	10116.ENSRNO	10116.ENSRNO	0	0	0.305	0.744	0.061	0	0.99	0.644	0.992
Ehhadh	Hibch	10116.ENSRNO	10116.ENSRNO	0	0	0	0.597	0.094	0.078	0.99	0.41	0.992
Ehhadh	Hadh	10116.ENSRNO	10116.ENSRNO	0.084	0	0	0.778	0.185	0.263	0.99	0.718	0.995
Ehhadh	Hsd17b10	10116.ENSRNO	10116.ENSRNO	0	0	0	0	0.19	0.134	0.99	0.359	0.995
Eif2s1	Gnb2l1	10116.ENSRNO	10116.ENSRNO	0	0	0	0	0.717	0.856	0	0.427	0.974
Eif2s1	Rps23	10116.ENSRNO	10116.ENSRNO	0	0	0	0	0.461	0.678	0.6	0.059	0.925
Eif2s1	Rps5	10116.ENSRNO	10116.ENSRNO	0	0	0	0	0.637	0.856	0.6	0.263	0.982
Eif2s1	Eif4a1	10116.ENSRNO	10116.ENSRNO	0	0	0	0	0.598	0.728	0.848	0.538	0.991
Eif4a1	Gnb2l1	10116.ENSRNO	10116.ENSRNO	0	0	0	0	0.549	0.696	0	0.385	0.908
Eif4a1	Rps5	10116.ENSRNO	10116.ENSRNO	0	0	0	0	0.59	0.696	0.6	0.205	0.955
Eno1	Pgk1	10116.ENSRNO	10116.ENSRNO	0.074	0	0	0	0.438	0.307	0.186	0.751	0.914
Eno1	LOC500959	10116.ENSRNO	10116.ENSRNO	0.074	0	0	0	0.417	0.316	0.186	0.739	0.907
Eno1	Plg	10116.ENSRNO	10116.ENSRNO	0	0	0	0	0.055	0.072	0	0.982	0.983
Eno1	Gpi	10116.ENSRNO	10116.ENSRNO	0.087	0	0	0	0.769	0.193	0.835	0.632	0.987
Etfb	Etfdh	10116.ENSRNO	10116.ENSRNO	0.155	0	0.437	0	0.493	0	0.9	0.892	0.996
Etfb	Hsd17b10	10116.ENSRNO	10116.ENSRNO	0	0	0	0	0.911	0	0	0.269	0.932
Fahd1	Gstz1	10116.ENSRNO	10116.ENSRNO	0.079	0	0	0	0.106	0	0.911	0.128	0.927
Fga	Pebp1	10116.ENSRNO	10116.ENSRNO	0	0	0	0	0.047	0	0.9	0.064	0.903
Fga	Fn1	10116.ENSRNO	10116.ENSRNO	0	0	0	0	0.062	0.487	0.785	0.27	0.914
Fga	Plg	10116.ENSRNO	10116.ENSRNO	0	0	0	0	0.939	0.326	0.229	0.367	0.977
Fkbp8	Tomm70a	10116.ENSRNO	10116.ENSRNO	0	0	0	0	0.079	0	0.9	0.22	0.921
Fkbp8	Vdac1	10116.ENSRNO	10116.ENSRNO	0	0	0	0	0.064	0	0.9	0.069	0.905
Fkbp8	Vdac2	10116.ENSRNO	10116.ENSRNO	0	0	0	0	0.064	0	0.9	0.113	0.909
Fkbp8	Mul1	10116.ENSRNO	10116.ENSRNO	0	0	0	0	0.058	0	0.9	0.315	0.929
Fkbp8	Vdac3	10116.ENSRNO	10116.ENSRNO	0	0	0	0	0.064	0	0.9	0.13	0.911
Flna	Tagln2	10116.ENSRNO	10116.ENSRNO	0	0	0	0	0.524	0.16	0.9	0.307	0.968
Flna	Vcl	10116.ENSRNO	10116.ENSRNO	0	0	0	0	0.359	0.159	0.915	0.678	0.983
Flna	Tln1	10116.ENSRNO	10116.ENSRNO	0	0	0	0	0.142	0.055	0.917	0.58	0.968
Flna	Wdr1	10116.ENSRNO	10116.ENSRNO	0	0	0	0	0.131	0.088	0.9	0.339	0.94
Flna	Flnb	10116.ENSRNO	10116.ENSRNO	0	0	0	0.975	0.125	0.724	0.8	0.843	0.948
Fn1	Pebp1	10116.ENSRNO	10116.ENSRNO	0	0	0	0	0	0	0.9	0.136	0.909
Fn1	Vcl	10116.ENSRNO	10116.ENSRNO	0	0	0	0	0.084	0.043	0.5	0.938	0.969
Fn1	Tgm2	10116.ENSRNO	10116.ENSRNO	0	0	0	0	0.104	0.805	0	0.856	0.972
Fn1	Itga7	10116.ENSRNO	10116.ENSRNO	0	0	0	0	0.058	0.329	0.729	0.485	0.9
Fn1	Plg	10116.ENSRNO	10116.ENSRNO	0	0	0	0	0	0.21	0.163	0.872	0.908
Fn1	Itga6	10116.ENSRNO	10116.ENSRNO	0	0	0	0	0.057	0.329	0.729	0.53	0.908
Ganab	Prkcsh	10116.ENSRNO	10116.ENSRNO	0	0.115	0	0	0.154	0.924	0.981	0.659	0.999
Gapdh	Pgk1	10116.ENSRNO	10116.ENSRNO	0.044	0.002	0	0	0.248	0.237	0.915	0.72	0.984
Gapdh	LOC500959	10116.ENSRNO	10116.ENSRNO	0.044	0.002	0	0	0.251	0.218	0.915	0.379	0.965
Gapdh	Gpi	10116.ENSRNO	10116.ENSRNO	0.068	0	0	0	0.735	0	0.819	0.32	0.965
Gcdh	Hadha	10116.ENSRNO	10116.ENSRNO	0.052	0	0	0	0.123	0.164	0.922	0.428	0.963
Gcsh	Pdhb	10116.ENSRNO	10116.ENSRNO	0.042	0	0	0	0.11	0	0.9	0.188	0.921
Gcsh	Ndufab1	10116.ENSRNO	10116.ENSRNO	0	0	0	0	0.2	0.054	0.9	0	0.917
Gdi2	Ywhae	10116.ENSRNO	10116.ENSRNO	0	0	0	0	0.192	0	0.9	0.096	0.92
Gdi2	Rab7a	10116.ENSRNO	10116.ENSRNO	0	0	0	0	0.617	0.801	0.6	0.476	0.981
Gfm1	Mrps7	10116.ENSRNO	10116.ENSRNO	0.098	0	0.38	0	0.569	0.929	0	0.206	0.983
Gfm1	Tufm	10116.ENSRNO	10116.ENSRNO	0.116	0.057	0.398	0	0.783	0.446	0	0.644	0.973
Gfm1	Ict1	10116.ENSRNO	10116.ENSRNO	0	0	0	0	0.146	0.789	0	0.594	0.92
Gfm1	Mrps22	10116.ENSRNO	10116.ENSRNO	0	0	0	0	0.163	0.708	0	0.703	0.921

Glud1	Mdh2	10116.ENSRNO	10116.ENSRNO	0.071	0	0	0	0.262	0.045	0.8	0.708	0.954
Glud1	Mdh1	10116.ENSRNO	10116.ENSRNO	0.071	0	0	0	0.251	0	0.8	0.548	0.928
Glud1	Pc	10116.ENSRNO	10116.ENSRNO	0	0	0	0	0.102	0	0.8	0.596	0.921
Glud1	Sirt3	10116.ENSRNO	10116.ENSRNO	0.041	0	0	0	0.063	0.104	0.9	0.316	0.934
Glud1	Sod2	10116.ENSRNO	10116.ENSRNO	0.122	0	0	0	0.081	0	0.9	0.369	0.942
Glud1	Got2	10116.ENSRNO	10116.ENSRNO	0	0	0	0	0.206	0.097	0.9	0.578	0.965
Glud1	Got1	10116.ENSRNO	10116.ENSRNO	0	0	0	0	0.171	0.097	0.9	0.695	0.974
Glud1	Idh2	10116.ENSRNO	10116.ENSRNO	0	0	0	0	0.12	0.08	0.945	0.555	0.977
Gna13	Gnb2	10116.ENSRNO	10116.ENSRNO	0	0	0	0	0.12	0.373	0.793	0.34	0.914
Gna13	Gnaq	10116.ENSRNO	10116.ENSRNO	0	0	0	0.921	0.058	0.504	0.825	0.881	0.917
Gna13	Gnb1	10116.ENSRNO	10116.ENSRNO	0	0	0	0	0.12	0.5	0.848	0.349	0.95
Gna13	Rhoa	10116.ENSRNO	10116.ENSRNO	0	0	0	0	0	0.308	0.959	0.776	0.993
Gnai2	Gnb2	10116.ENSRNO	10116.ENSRNO	0	0	0	0	0.213	0.634	0.806	0.607	0.975
Gnai2	Nos3	10116.ENSRNO	10116.ENSRNO	0	0	0	0	0.06	0.151	0.922	0.102	0.937
Gnai2	Gnaq	10116.ENSRNO	10116.ENSRNO	0	0	0	0.937	0.058	0.47	0.822	0.847	0.908
Gnai2	Gnb1	10116.ENSRNO	10116.ENSRNO	0	0	0	0	0.372	0.941	0.845	0.5	0.996
Gnaq	Gnb2	10116.ENSRNO	10116.ENSRNO	0	0	0	0	0.118	0.528	0.793	0.432	0.944
Gnaq	Gnas	10116.ENSRNO	10116.ENSRNO	0	0	0	0	0	0	0.9	0	0.9
Gnaq	Gnb1	10116.ENSRNO	10116.ENSRNO	0	0	0	0	0.14	0.809	0.962	0.409	0.995
Gnas	Gnb1	10116.ENSRNO	10116.ENSRNO	0	0	0	0	0	0	0.9	0.096	0.905
Gnb1	Gnb2	10116.ENSRNO	10116.ENSRNO	0	0	0.449	0.985	0.081	0	0.9	0.515	0.905
Gnb1	Gnb2l1	10116.ENSRNO	10116.ENSRNO	0	0	0.419	0.679	0.06	0.855	0.629	0.45	0.958
Gnb1	Gng12	10116.ENSRNO	10116.ENSRNO	0	0	0	0	0.057	0.965	0.713	0.559	0.995
Gnb2	Gng12	10116.ENSRNO	10116.ENSRNO	0	0	0	0	0.057	0.965	0.713	0.55	0.995
Gnb2l1	Rplp0	10116.ENSRNO	10116.ENSRNO	0	0	0	0	0.876	0.696	0	0.454	0.977
Gnb2l1	Rpl3l	10116.ENSRNO	10116.ENSRNO	0	0	0	0	0.624	0.73	0	0.186	0.91
Gnb2l1	Rps2	10116.ENSRNO	10116.ENSRNO	0	0	0	0	0.548	0.528	0.54	0.319	0.924
Gnb2l1	Rpl19	10116.ENSRNO	10116.ENSRNO	0	0	0	0	0.848	0.528	0	0	0.925
Gnb2l1	Rps23	10116.ENSRNO	10116.ENSRNO	0	0	0	0	0.851	0.806	0.54	0.199	0.987
Gnb2l1	Rps5	10116.ENSRNO	10116.ENSRNO	0	0	0	0	0.98	0.94	0.54	0.492	0.999
Got1	Mpst	10116.ENSRNO	10116.ENSRNO	0.095	0	0	0	0.051	0	0.99	0.328	0.993
Got1	Mdh2	10116.ENSRNO	10116.ENSRNO	0	0	0	0	0.205	0.145	0.991	0.634	0.997
Got1	Lap3	10116.ENSRNO	10116.ENSRNO	0	0	0	0	0.086	0.294	0.8	0.387	0.91
Got1	Mdh1	10116.ENSRNO	10116.ENSRNO	0	0	0	0	0.602	0.217	0.99	0.707	0.999
Got1	Ldha	10116.ENSRNO	10116.ENSRNO	0	0	0	0	0.119	0.145	0.915	0.691	0.977
Got1	Ldhb	10116.ENSRNO	10116.ENSRNO	0	0	0	0	0.152	0.145	0.915	0.744	0.982
Got1	Pc	10116.ENSRNO	10116.ENSRNO	0	0	0	0	0.064	0.114	0.931	0.547	0.97
Got2	Mpst	10116.ENSRNO	10116.ENSRNO	0.095	0	0	0	0.075	0	0.922	0.11	0.934
Got2	Mdh2	10116.ENSRNO	10116.ENSRNO	0	0	0	0	0.584	0.444	0.978	0.616	0.997
Got2	Lap3	10116.ENSRNO	10116.ENSRNO	0	0	0	0	0.12	0.4	0.8	0.34	0.921
Got2	Sdhb	10116.ENSRNO	10116.ENSRNO	0	0	0	0	0.183	0.362	0.8	0.212	0.906
Got2	Mdh1	10116.ENSRNO	10116.ENSRNO	0	0	0	0	0.271	0.145	0.965	0.576	0.989
Got2	Sdha	10116.ENSRNO	10116.ENSRNO	0	0	0	0	0.179	0.042	0.862	0.292	0.913
Got2	Idh2	10116.ENSRNO	10116.ENSRNO	0	0	0	0	0.565	0.054	0.8	0.44	0.947
Got2	Ldha	10116.ENSRNO	10116.ENSRNO	0	0	0	0	0.119	0.145	0.916	0.507	0.964
Got2	Ldhb	10116.ENSRNO	10116.ENSRNO	0	0	0	0	0.119	0.145	0.916	0.617	0.972
Got2	Pc	10116.ENSRNO	10116.ENSRNO	0	0	0	0	0.064	0.07	0.953	0.483	0.976
Gpi	Pgk1	10116.ENSRNO	10116.ENSRNO	0.111	0	0	0	0.614	0.321	0.244	0.701	0.938
Gpi	Pdhb	10116.ENSRNO	10116.ENSRNO	0.082	0	0	0	0.141	0	0.831	0.474	0.92
Gpi	Pfkm	10116.ENSRNO	10116.ENSRNO	0.12	0	0	0	0.596	0.163	0.99	0.67	0.998
Gpi	Pgm1	10116.ENSRNO	10116.ENSRNO	0.128	0	0	0	0.774	0.162	0.982	0.722	0.999
Gpi	Ldha	10116.ENSRNO	10116.ENSRNO	0.094	0	0	0	0.228	0	0.8	0.642	0.943
Gpi	Ldhb	10116.ENSRNO	10116.ENSRNO	0.094	0	0	0	0.196	0	0.8	0.629	0.938
Gpi	LOC500959	10116.ENSRNO	10116.ENSRNO	0.095	0	0	0	0.632	0.452	0.8	0.643	0.984
Gpx4	Prdx6	10116.ENSRNO	10116.ENSRNO	0	0	0	0	0.064	0.193	0.8	0.726	0.953
Gpx4	Sod2	10116.ENSRNO	10116.ENSRNO	0	0	0	0	0.064	0.445	0.67	0.808	0.962
Hadh	Hibadh	10116.ENSRNO	10116.ENSRNO	0	0	0	0	0.489	0.505	0.875	0.185	0.971

Hadh	Hsd17b10	10116.ENSRNO	10116.ENSRNO	0	0	0	0	0.152	0	0.8	0.499	0.907
Hadh	Pccb	10116.ENSRNO	10116.ENSRNO	0.069	0	0	0	0.601	0	0.8	0.258	0.937
Hadh	Oxct1	10116.ENSRNO	10116.ENSRNO	0.128	0	0	0	0.106	0	0.9	0.417	0.948
Hadh	Scp2	10116.ENSRNO	10116.ENSRNO	0	0	0	0	0.363	0	0.954	0.313	0.978
Hadh	Hibch	10116.ENSRNO	10116.ENSRNO	0.084	0	0	0	0.16	0.133	0.988	0.297	0.993
Hadh	Hadha	10116.ENSRNO	10116.ENSRNO	0.084	0	0.27	0.793	0.221	0.263	0.99	0.832	0.995
Hadha	Ivd	10116.ENSRNO	10116.ENSRNO	0.052	0	0	0	0.155	0.193	0.922	0.309	0.959
Hadha	Scp2	10116.ENSRNO	10116.ENSRNO	0	0	0	0	0.188	0.52	0.928	0.151	0.973
Hadha	Mccc1	10116.ENSRNO	10116.ENSRNO	0.084	0	0	0	0.099	0.104	0.916	0.262	0.946
Hadha	Hsd17b4	10116.ENSRNO	10116.ENSRNO	0.084	0	0	0	0.195	0.133	0.911	0.328	0.954
Hadha	Mccc2	10116.ENSRNO	10116.ENSRNO	0.084	0	0	0	0.152	0.044	0.916	0.385	0.955
Hadha	Mecr	10116.ENSRNO	10116.ENSRNO	0.053	0	0	0	0.089	0.079	0.912	0.14	0.929
Hadha	Hibch	10116.ENSRNO	10116.ENSRNO	0	0	0	0.586	0	0.078	0.99	0.456	0.992
Hadha	Hsdl2	10116.ENSRNO	10116.ENSRNO	0.044	0	0	0	0.181	0.501	0.829	0.168	0.934
Hadha	Hsd17b10	10116.ENSRNO	10116.ENSRNO	0	0	0	0	0.171	0.134	0.994	0.515	0.998
Hba1	Hpx	10116.ENSRNO	10116.ENSRNO	0	0	0	0	0.05	0	0.9	0.241	0.921
Hibadh	Pccb	10116.ENSRNO	10116.ENSRNO	0.092	0	0	0	0.163	0.218	0.8	0.49	0.928
Hibadh	Mccc2	10116.ENSRNO	10116.ENSRNO	0.092	0	0.206	0	0.676	0.5	0	0.514	0.933
Hibadh	Hibch	10116.ENSRNO	10116.ENSRNO	0.083	0.769	0	0	0.159	0.512	0.994	0.825	0.999
Hibch	Mrps6	10116.ENSRNO	10116.ENSRNO	0	0	0	0	0.064	0.923	0	0.057	0.926
Hibch	Mrpl27	10116.ENSRNO	10116.ENSRNO	0	0	0	0	0.073	0.909	0	0.296	0.935
Hibch	Mrpl15	10116.ENSRNO	10116.ENSRNO	0	0	0	0	0.081	0.909	0	0	0.912
Hibch	Sod2	10116.ENSRNO	10116.ENSRNO	0	0	0	0	0.075	0.853	0	0.484	0.923
Hibch	Hsd17b10	10116.ENSRNO	10116.ENSRNO	0	0	0	0	0.674	0.134	0.783	0.538	0.967
Hpx	Lrp1	10116.ENSRNO	10116.ENSRNO	0	0	0	0	0.056	0.046	0.6	0.905	0.961
Hsd17b10	Scp2	10116.ENSRNO	10116.ENSRNO	0	0	0	0	0.193	0	0.954	0.165	0.966
Hsd17b4	Scp2	10116.ENSRNO	10116.ENSRNO	0	0	0	0.593	0.885	0.717	0.982	0.846	0.999
Hsd17b4	Hsdl2	10116.ENSRNO	10116.ENSRNO	0.07	0	0	0.565	0.519	0.519	0.71	0.609	0.947
Hsp90aa1	Tomm70a	10116.ENSRNO	10116.ENSRNO	0	0	0	0	0.113	0.451	0	0.939	0.967
Hsp90aa1	Hsp90ab1	10116.ENSRNO	10116.ENSRNO	0	0	0.449	0.984	0.376	0.478	0.8	0.849	0.93
Hsp90aa1	Hspa9	10116.ENSRNO	10116.ENSRNO	0	0	0	0	0.405	0.21	0.226	0.839	0.933
Hsp90aa1	Hspa5	10116.ENSRNO	10116.ENSRNO	0	0	0	0	0.558	0.248	0.226	0.798	0.941
Hsp90aa1	Hspd1	10116.ENSRNO	10116.ENSRNO	0	0	0	0	0.556	0.395	0	0.855	0.957
Hsp90aa1	Rhoa	10116.ENSRNO	10116.ENSRNO	0	0	0	0	0	0.066	0.946	0.399	0.967
Hsp90aa1	Stip1	10116.ENSRNO	10116.ENSRNO	0.044	0	0	0	0.604	0.71	0.6	0.769	0.988
Hsp90aa1	Hspa8	10116.ENSRNO	10116.ENSRNO	0	0	0	0	0.581	0.431	0.552	0.937	0.992
Hsp90aa1	Nos3	10116.ENSRNO	10116.ENSRNO	0	0	0	0	0.05	0.455	0.91	0.989	0.999
Hsp90ab1	Nos3	10116.ENSRNO	10116.ENSRNO	0	0	0	0	0.05	0.369	0.903	0.532	0.969
Hsp90ab1	Hspa5	10116.ENSRNO	10116.ENSRNO	0	0	0	0	0.527	0.248	0.483	0.701	0.937
Hsp90ab1	Hspa8	10116.ENSRNO	10116.ENSRNO	0	0	0	0	0.388	0.367	0.821	0.789	0.983
Hsp90ab1	Stip1	10116.ENSRNO	10116.ENSRNO	0.044	0	0	0	0.629	0.629	0.72	0.709	0.987
Hsp90b1	Pdia3	10116.ENSRNO	10116.ENSRNO	0	0	0	0	0.846	0.434	0	0.799	0.981
Hsp90b1	Ppib	10116.ENSRNO	10116.ENSRNO	0	0	0	0	0.205	0.532	0.72	0.667	0.96
Hsp90b1	Hspa5	10116.ENSRNO	10116.ENSRNO	0	0	0	0	0.974	0.752	0.774	0.967	0.999
Hsp90b1	P4hb	10116.ENSRNO	10116.ENSRNO	0	0	0	0	0.617	0.698	0.72	0.892	0.996
Hspa12b	St13	10116.ENSRNO	10116.ENSRNO	0	0	0	0	0.132	0.207	0.9	0.141	0.932
Hspa5	Pdia3	10116.ENSRNO	10116.ENSRNO	0	0	0	0	0.691	0.529	0.6	0.867	0.991
Hspa5	Ppib	10116.ENSRNO	10116.ENSRNO	0	0.003	0	0	0.222	0.713	0.72	0.553	0.968
Hspa5	Hspd1	10116.ENSRNO	10116.ENSRNO	0.044	0	0	0	0.599	0.384	0	0.639	0.903
Hspa5	St13	10116.ENSRNO	10116.ENSRNO	0	0	0	0	0.139	0.265	0.9	0.177	0.941
Hspa5	P4hb	10116.ENSRNO	10116.ENSRNO	0	0	0	0	0.656	0.671	0.72	0.894	0.996
Hspa8	Lamp2	10116.ENSRNO	10116.ENSRNO	0	0	0	0	0.05	0.356	0.6	0.737	0.927
Hspa8	Txndc5	10116.ENSRNO	10116.ENSRNO	0	0	0	0	0.064	0.156	0.9	0.208	0.929
Hspa8	St13	10116.ENSRNO	10116.ENSRNO	0	0	0	0	0.132	0.464	0.9	0.82	0.99
Hspa8	Stip1	10116.ENSRNO	10116.ENSRNO	0	0	0	0	0.455	0.715	0.72	0.587	0.979
Hspa8	Snx2	10116.ENSRNO	10116.ENSRNO	0	0	0	0	0.057	0.054	0.9	0.086	0.907
Hspa9	Vdac1	10116.ENSRNO	10116.ENSRNO	0	0	0	0	0.145	0.064	0	0.955	0.961

Hspa9	St13	10116.ENSRNO	10116.ENSRNO	0	0	0	0	0.148	0.207	0.9	0.229	0.941
Hspa9	Hspd1	10116.ENSRNO	10116.ENSRNO	0.044	0	0	0	0.6	0.516	0	0.787	0.955
Hspd1	St13	10116.ENSRNO	10116.ENSRNO	0	0	0	0	0.869	0	0	0.331	0.908
Ict1	Mrpl27	10116.ENSRNO	10116.ENSRNO	0.052	0	0	0	0.581	0.853	0.8	0.376	0.991
Ict1	Mrps7	10116.ENSRNO	10116.ENSRNO	0.069	0	0	0	0.566	0.809	0	0.309	0.939
Ict1	Mrpl50	10116.ENSRNO	10116.ENSRNO	0	0	0	0	0.118	0.929	0.8	0	0.986
Ict1	Mrpl38	10116.ENSRNO	10116.ENSRNO	0	0	0	0	0.169	0.853	0.8	0.319	0.981
Ict1	Mrpl41	10116.ENSRNO	10116.ENSRNO	0	0	0	0	0.384	0.875	0.8	0.074	0.983
Ict1	Mrps22	10116.ENSRNO	10116.ENSRNO	0	0	0	0	0.299	0.813	0	0.51	0.93
Idh2	Mdh2	10116.ENSRNO	10116.ENSRNO	0.085	0	0	0	0.585	0.16	0	0.802	0.928
Idh2	Sirt3	10116.ENSRNO	10116.ENSRNO	0.044	0	0	0	0	0.083	0.9	0.682	0.968
Idh2	Sod2	10116.ENSRNO	10116.ENSRNO	0.088	0	0	0	0.093	0.146	0.9	0.524	0.96
Immt	Mtx2	10116.ENSRNO	10116.ENSRNO	0	0	0	0	0.064	0.927	0	0.295	0.947
Immt	Samm50	10116.ENSRNO	10116.ENSRNO	0	0	0	0	0.139	0.861	0	0.842	0.979
Itga6	Lama2	10116.ENSRNO	10116.ENSRNO	0	0	0	0	0.06	0.193	0.845	0.41	0.921
Itga7	Lama2	10116.ENSRNO	10116.ENSRNO	0	0	0	0	0.083	0.193	0.845	0.406	0.923
Ivd	Pccb	10116.ENSRNO	10116.ENSRNO	0.084	0	0	0	0.59	0	0.74	0.737	0.97
Ivd	Mccc2	10116.ENSRNO	10116.ENSRNO	0.084	0.004	0.292	0	0.65	0	0.994	0.78	0.999
Ivd	Mccc1	10116.ENSRNO	10116.ENSRNO	0.084	0	0	0	0.563	0.156	0.994	0.608	0.999
LOC500959	Pgk1	10116.ENSRNO	10116.ENSRNO	0.088	0.399	0.219	0	0.541	0.375	0.281	0.774	0.974
LOC679794	Slc25a4	10116.ENSRNO	10116.ENSRNO	0	0	0	0	0.181	0.244	0.8	0.401	0.916
LOC679794	Slc25a5	10116.ENSRNO	10116.ENSRNO	0	0	0	0	0.448	0.189	0.8	0.323	0.931
LOC679794	Uqcrfs1	10116.ENSRNO	10116.ENSRNO	0.1	0	0	0	0.784	0.527	0.28	0.375	0.951
LOC687295	Tomm40l	10116.ENSRNO	10116.ENSRNO	0	0	0	0	0.568	0.804	0	0.503	0.954
LOC687295	Tomm22	10116.ENSRNO	10116.ENSRNO	0	0	0	0	0.174	0.39	0	0.835	0.909
LOC687295	Tomm40	10116.ENSRNO	10116.ENSRNO	0	0	0	0	0.577	0.804	0	0.752	0.977
LOC687295	rCG_22860	10116.ENSRNO	10116.ENSRNO	0	0	0	0	0.275	0.696	0	0.763	0.943
Lamp1	Rab7a	10116.ENSRNO	10116.ENSRNO	0	0	0	0	0.071	0.043	0	0.923	0.926
Ldha	Mpst	10116.ENSRNO	10116.ENSRNO	0	0	0	0	0.066	0	0.9	0.158	0.914
Ldha	Pdhb	10116.ENSRNO	10116.ENSRNO	0.043	0	0	0	0.111	0.091	0.913	0.442	0.955
Ldha	Ldhb	10116.ENSRNO	10116.ENSRNO	0	0	0.449	0.978	0.064	0.561	0.8	0.954	0.913
Ldha	Ldhd	10116.ENSRNO	10116.ENSRNO	0.067	0	0	0	0.06	0	0.9	0.475	0.947
Ldha	Me3	10116.ENSRNO	10116.ENSRNO	0.084	0	0	0	0.141	0.066	0.915	0.319	0.95
Ldha	Pc	10116.ENSRNO	10116.ENSRNO	0.065	0	0	0	0.064	0.096	0.921	0.649	0.974
Ldhb	Mpst	10116.ENSRNO	10116.ENSRNO	0	0	0	0	0.066	0	0.9	0.279	0.926
Ldhb	Pdhb	10116.ENSRNO	10116.ENSRNO	0.043	0	0	0	0.136	0.091	0.913	0.429	0.955
Ldhb	Me3	10116.ENSRNO	10116.ENSRNO	0.084	0	0	0	0.173	0.066	0.915	0.399	0.957
Ldhb	Pc	10116.ENSRNO	10116.ENSRNO	0.065	0	0	0	0.064	0.096	0.921	0.49	0.962
Ldhb	Ldhd	10116.ENSRNO	10116.ENSRNO	0.067	0	0	0	0.06	0	0.9	0.685	0.968
Ldhd	Pdhb	10116.ENSRNO	10116.ENSRNO	0	0	0	0	0.092	0	0.9	0.167	0.917
Ldhd	Me3	10116.ENSRNO	10116.ENSRNO	0.045	0	0	0	0.07	0	0.9	0.071	0.906
Ldhd	Pc	10116.ENSRNO	10116.ENSRNO	0.042	0	0	0	0.102	0	0.9	0.243	0.926
Lman1	Sec22b	10116.ENSRNO	10116.ENSRNO	0	0	0	0	0.171	0	0.6	0.768	0.916
MGC94335	Pmpca	10116.ENSRNO	10116.ENSRNO	0	0	0	0	0.054	0.099	0.9	0	0.907
Mccc1	Pccb	10116.ENSRNO	10116.ENSRNO	0.116	0.075	0	0	0.573	0.684	0.765	0.701	0.99
Mccc1	Mccc2	10116.ENSRNO	10116.ENSRNO	0.116	0.129	0	0	0.597	0.928	0.97	0.833	0.999
Mdh1	Mdh2	10116.ENSRNO	10116.ENSRNO	0	0	0	0	0.613	0	0.981	0.877	0.999
Mdh1	Pgm1	10116.ENSRNO	10116.ENSRNO	0	0	0	0	0.606	0.463	0	0.616	0.911
Mdh1	Me3	10116.ENSRNO	10116.ENSRNO	0.084	0	0	0	0.171	0.066	0.963	0.611	0.987
Mdh1	Pc	10116.ENSRNO	10116.ENSRNO	0.065	0	0	0	0.104	0.096	0.99	0.637	0.996
Mdh2	Ndufv1	10116.ENSRNO	10116.ENSRNO	0.044	0	0	0	0.872	0.046	0	0.354	0.915
Mdh2	Uqcrfs1	10116.ENSRNO	10116.ENSRNO	0	0	0	0	0.792	0.147	0	0.645	0.931
Mdh2	Uqcrc1	10116.ENSRNO	10116.ENSRNO	0	0	0	0	0.849	0.312	0	0.538	0.947
Mdh2	Sdhb	10116.ENSRNO	10116.ENSRNO	0.094	0	0	0	0.69	0.522	0.194	0.76	0.969
Mdh2	Sdha	10116.ENSRNO	10116.ENSRNO	0.094	0	0	0	0.636	0.513	0.176	0.809	0.97
Mdh2	Me3	10116.ENSRNO	10116.ENSRNO	0.084	0	0	0	0.159	0.101	0.975	0.662	0.993
Mdh2	Pc	10116.ENSRNO	10116.ENSRNO	0.065	0	0	0	0.09	0.16	0.99	0.719	0.997

Me3	Pdhb	10116.ENSRNO	10116.ENSRNO	0	0	0	0	0.099	0	0.925	0.292	0.948
Me3	Pc	10116.ENSRNO	10116.ENSRNO	0.043	0	0	0	0.075	0.083	0.941	0.593	0.977
Mecr	Oxsm	10116.ENSRNO	10116.ENSRNO	0	0	0	0	0.12	0	0.9	0.423	0.944
Mfn1	Vdac1	10116.ENSRNO	10116.ENSRNO	0	0	0	0	0	0.097	0.8	0.709	0.942
Mfn1	Tomm40	10116.ENSRNO	10116.ENSRNO	0	0	0	0	0.056	0.104	0.72	0.633	0.901
Mfn1	Opa1	10116.ENSRNO	10116.ENSRNO	0	0	0	0	0.119	0.256	0	0.88	0.914
Mrpl15	Mrps6	10116.ENSRNO	10116.ENSRNO	0	0	0	0	0.445	0.926	0	0.507	0.977
Mrpl15	Mrpl27	10116.ENSRNO	10116.ENSRNO	0	0	0	0	0.662	0.926	0.177	0.171	0.98
Mrpl15	Mrps7	10116.ENSRNO	10116.ENSRNO	0.085	0	0	0	0.562	0.81	0	0.161	0.927
Mrpl15	Mrpl50	10116.ENSRNO	10116.ENSRNO	0	0	0	0	0.145	0.939	0	0	0.945
Mrpl15	Mrpl38	10116.ENSRNO	10116.ENSRNO	0	0	0	0	0.199	0.942	0	0	0.951
Mrpl15	Rps5	10116.ENSRNO	10116.ENSRNO	0.085	0	0	0	0.554	0.808	0	0.161	0.925
Mrpl15	Sod2	10116.ENSRNO	10116.ENSRNO	0	0	0	0	0.53	0.92	0	0.322	0.972
Mrpl27	Mrps6	10116.ENSRNO	10116.ENSRNO	0.042	0	0	0	0.574	0.926	0	0.379	0.978
Mrpl27	Sod2	10116.ENSRNO	10116.ENSRNO	0	0	0	0	0.555	0.81	0	0	0.911
Mrpl27	Ndufb7	10116.ENSRNO	10116.ENSRNO	0	0	0	0	0.921	0	0	0	0.921
Mrpl27	Ndufb10	10116.ENSRNO	10116.ENSRNO	0	0	0	0	0.93	0	0	0	0.93
Mrpl27	Rps5	10116.ENSRNO	10116.ENSRNO	0.052	0	0	0	0.631	0.808	0	0.309	0.947
Mrpl27	Mrps7	10116.ENSRNO	10116.ENSRNO	0.052	0	0	0	0.69	0.809	0	0.309	0.956
Mrpl27	Mrpl41	10116.ENSRNO	10116.ENSRNO	0	0	0	0	0.602	0.809	0.8	0	0.983
Mrpl27	Mrpl38	10116.ENSRNO	10116.ENSRNO	0	0	0	0	0.489	0.853	0.8	0.129	0.985
Mrpl27	Mrpl50	10116.ENSRNO	10116.ENSRNO	0	0	0	0	0.155	0.927	0.8	0	0.986
Mrpl38	Mrpl50	10116.ENSRNO	10116.ENSRNO	0	0	0	0	0.083	0.936	0.8	0	0.987
Mrpl38	Mrpl41	10116.ENSRNO	10116.ENSRNO	0	0	0	0	0.088	0.696	0.8	0.144	0.946
Mrpl41	Mrpl50	10116.ENSRNO	10116.ENSRNO	0	0	0	0	0.155	0.811	0.8	0	0.965
Mrps22	Mrps6	10116.ENSRNO	10116.ENSRNO	0	0	0	0	0.064	0.858	0.8	0	0.971
Mrps22	Mrps7	10116.ENSRNO	10116.ENSRNO	0	0	0	0	0.143	0.858	0.8	0.31	0.98
Mrps22	Mrps23	10116.ENSRNO	10116.ENSRNO	0	0	0	0	0.146	0.939	0.8	0.306	0.991
Mrps22	Mrps25	10116.ENSRNO	10116.ENSRNO	0	0	0	0	0.122	0.934	0.8	0.144	0.988
Mrps23	Mrps6	10116.ENSRNO	10116.ENSRNO	0	0	0	0	0.112	0.856	0.8	0	0.972
Mrps23	Mrps7	10116.ENSRNO	10116.ENSRNO	0	0	0	0	0.229	0.856	0.8	0.356	0.983
Mrps23	Mrps25	10116.ENSRNO	10116.ENSRNO	0	0	0	0	0.567	0.808	0.8	0	0.981
Mrps25	Mrps6	10116.ENSRNO	10116.ENSRNO	0	0	0	0	0.064	0.808	0.8	0	0.96
Mrps25	Mrps7	10116.ENSRNO	10116.ENSRNO	0	0	0	0	0.225	0.931	0.8	0.405	0.992
Mrps6	Sod2	10116.ENSRNO	10116.ENSRNO	0	0	0	0	0.189	0.81	0	0.461	0.909
Mrps6	Rps5	10116.ENSRNO	10116.ENSRNO	0.115	0	0	0	0.564	0.809	0.21	0.309	0.952
Mrps6	Mrps7	10116.ENSRNO	10116.ENSRNO	0.115	0	0	0	0.564	0.858	0.835	0.309	0.992
Mrps7	Tufm	10116.ENSRNO	10116.ENSRNO	0.128	0	0.318	0	0.604	0.551	0	0.309	0.913
Msn	Rhoa	10116.ENSRNO	10116.ENSRNO	0	0	0	0	0.097	0.475	0.504	0.77	0.938
Mtch2	Ndufs1	10116.ENSRNO	10116.ENSRNO	0	0	0	0	0.139	0.87	0	0.271	0.911
Mtdh	Snd1	10116.ENSRNO	10116.ENSRNO	0	0	0	0	0	0.526	0	0.919	0.96
Mtx1	Samm50	10116.ENSRNO	10116.ENSRNO	0	0	0	0	0.132	0.723	0	0.643	0.906
Mul1	Tomm70a	10116.ENSRNO	10116.ENSRNO	0	0	0	0	0	0	0.9	0.168	0.913
Mul1	Vdac1	10116.ENSRNO	10116.ENSRNO	0	0	0	0	0	0	0.9	0.172	0.913
Mul1	Vdac2	10116.ENSRNO	10116.ENSRNO	0	0	0	0	0.058	0	0.9	0.137	0.911
Mul1	Vdac3	10116.ENSRNO	10116.ENSRNO	0	0	0	0	0.048	0	0.9	0.124	0.909
Myh7	Myl2	10116.ENSRNO	10116.ENSRNO	0	0	0	0	0.329	0.326	0.8	0.743	0.973
Myl2	Tpm1	10116.ENSRNO	10116.ENSRNO	0	0	0	0	0.261	0.134	0.6	0.715	0.917
Myl2	Rock1	10116.ENSRNO	10116.ENSRNO	0	0	0	0	0	0.148	0.859	0.586	0.946
Napa	Vamp7	10116.ENSRNO	10116.ENSRNO	0	0	0	0	0.098	0.803	0.9	0.693	0.993
Napa	Sec22b	10116.ENSRNO	10116.ENSRNO	0	0	0	0	0.077	0.749	0.9	0.783	0.994
Ndufa10	Ndufv3	10116.ENSRNO	10116.ENSRNO	0	0	0	0	0.099	0.804	0.768	0.5	0.976
Ndufa10	Ndufb4	10116.ENSRNO	10116.ENSRNO	0	0	0	0	0.109	0.782	0.759	0.524	0.974
Ndufa10	Ndufa4	10116.ENSRNO	10116.ENSRNO	0	0	0	0	0.167	0.484	0.768	0.579	0.952
Ndufa10	Ndufa5	10116.ENSRNO	10116.ENSRNO	0	0	0	0	0.501	0.94	0.932	0.652	0.999
Ndufa10	Ndufa12	10116.ENSRNO	10116.ENSRNO	0	0	0	0	0.231	0.929	0.827	0.319	0.992
Ndufa10	Ndufa6	10116.ENSRNO	10116.ENSRNO	0	0	0	0	0.591	0.967	0.903	0.702	0.999

Ndufa10	Ndufs3	10116.ENSRNO	10116.ENSRNO	0	0	0	0	0.588	0.94	0.903	0.771	0.999
Ndufa10	Ndufb9	10116.ENSRNO	10116.ENSRNO	0	0	0	0	0.676	0.931	0.903	0.495	0.998
Ndufa10	Ndufs4	10116.ENSRNO	10116.ENSRNO	0	0	0	0	0.573	0.937	0.932	0.61	0.999
Ndufa10	Ndufs1	10116.ENSRNO	10116.ENSRNO	0	0	0	0	0.712	0.941	0.932	0.835	0.999
Ndufa10	Ndufc2	10116.ENSRNO	10116.ENSRNO	0	0	0	0	0.677	0.94	0.903	0.544	0.999
Ndufa10	Uqcrh	10116.ENSRNO	10116.ENSRNO	0	0	0	0	0.589	0.923	0.821	0.582	0.997
Ndufa10	Ndufv2	10116.ENSRNO	10116.ENSRNO	0	0	0	0	0.61	0.941	0.932	0.57	0.999
Ndufa10	Ndufb8	10116.ENSRNO	10116.ENSRNO	0	0	0	0	0.738	0.931	0.903	0.609	0.999
Ndufa10	Ndufb10	10116.ENSRNO	10116.ENSRNO	0	0	0	0	0.679	0.915	0.862	0.753	0.998
Ndufa10	Uqcrc2	10116.ENSRNO	10116.ENSRNO	0	0	0	0	0.651	0.929	0	0.636	0.99
Ndufa10	Ndufaf6	10116.ENSRNO	10116.ENSRNO	0	0	0	0	0.082	0.193	0.6	0.713	0.903
Ndufa10	Uqcrb	10116.ENSRNO	10116.ENSRNO	0	0	0	0	0.513	0.808	0	0.109	0.909
Ndufa10	Ndufs5	10116.ENSRNO	10116.ENSRNO	0	0	0	0	0.5	0.816	0	0.596	0.959
Ndufa10	Ndufaf3	10116.ENSRNO	10116.ENSRNO	0	0	0	0	0.081	0	0.903	0.592	0.96
Ndufa10	rCG_38845	10116.ENSRNO	10116.ENSRNO	0	0	0	0	0.209	0.804	0.862	0.331	0.983
Ndufa10	Uqcrc1	10116.ENSRNO	10116.ENSRNO	0	0	0	0	0.654	0.929	0	0.565	0.988
Ndufa10	Uqcrfs1	10116.ENSRNO	10116.ENSRNO	0	0	0	0	0.732	0.929	0	0.491	0.989
Ndufa10	Ndufa2	10116.ENSRNO	10116.ENSRNO	0	0	0	0	0.192	0.927	0.862	0.596	0.996
Ndufa10	Ndufab1	10116.ENSRNO	10116.ENSRNO	0	0	0	0	0.5	0.929	0.862	0.605	0.997
Ndufa10	Ndufb6	10116.ENSRNO	10116.ENSRNO	0	0	0	0	0.249	0.941	0.903	0.574	0.997
Ndufa10	Ndufb7	10116.ENSRNO	10116.ENSRNO	0	0	0	0	0.601	0.941	0.903	0.485	0.998
Ndufa10	Ndufa9	10116.ENSRNO	10116.ENSRNO	0	0	0	0	0.687	0.967	0.932	0.586	0.999
Ndufa10	Ndufa8	10116.ENSRNO	10116.ENSRNO	0	0	0	0	0.676	0.941	0.903	0.579	0.999
Ndufa10	Ndufv1	10116.ENSRNO	10116.ENSRNO	0	0	0	0	0.673	0.94	0.903	0.702	0.999
Ndufa10	Ndufa11	10116.ENSRNO	10116.ENSRNO	0	0	0	0	0.709	0.939	0.932	0.618	0.999
Ndufa10	Ndufs7	10116.ENSRNO	10116.ENSRNO	0	0	0	0	0.639	0.941	0.903	0.686	0.999
Ndufa10	Ndufs2	10116.ENSRNO	10116.ENSRNO	0	0	0	0	0.704	0.941	0.932	0.676	0.999
Ndufa11	Ndufv3	10116.ENSRNO	10116.ENSRNO	0	0	0	0	0.354	0.923	0.768	0.342	0.991
Ndufa11	Ndufb4	10116.ENSRNO	10116.ENSRNO	0	0	0	0	0.561	0.783	0.879	0.634	0.995
Ndufa11	Ndufa4	10116.ENSRNO	10116.ENSRNO	0	0	0	0	0.336	0	0.768	0.421	0.903
Ndufa11	Ndufa5	10116.ENSRNO	10116.ENSRNO	0	0	0	0	0.945	0.94	0.951	0.501	0.999
Ndufa11	Ndufa12	10116.ENSRNO	10116.ENSRNO	0	0	0	0	0.9	0.93	0.827	0.726	0.999
Ndufa11	Ndufa6	10116.ENSRNO	10116.ENSRNO	0	0	0	0	0.901	0.979	0.951	0.569	0.999
Ndufa11	Ndufs3	10116.ENSRNO	10116.ENSRNO	0	0	0	0	0.872	0.979	0.903	0.491	0.999
Ndufa11	Ndufb9	10116.ENSRNO	10116.ENSRNO	0	0	0	0	0.916	0.929	0.951	0.77	0.999
Ndufa11	Ndufs4	10116.ENSRNO	10116.ENSRNO	0	0	0	0	0.925	0.938	0.932	0.427	0.999
Ndufa11	Ndufs1	10116.ENSRNO	10116.ENSRNO	0	0	0	0	0.214	0.941	0.932	0.58	0.998
Ndufa11	Ndufc2	10116.ENSRNO	10116.ENSRNO	0	0	0	0	0.805	0.929	0.951	0.441	0.999
Ndufa11	Uqcrh	10116.ENSRNO	10116.ENSRNO	0	0	0	0	0.82	0.813	0	0.457	0.98
Ndufa11	Ndufv2	10116.ENSRNO	10116.ENSRNO	0	0	0	0	0.903	0.941	0.932	0.538	0.999
Ndufa11	Ndufb8	10116.ENSRNO	10116.ENSRNO	0	0	0	0	0.962	0.939	0.951	0.666	0.999
Ndufa11	Ndufb10	10116.ENSRNO	10116.ENSRNO	0	0	0	0	0.927	0.808	0.931	0.612	0.999
Ndufa11	Uqcrc2	10116.ENSRNO	10116.ENSRNO	0	0	0	0	0.481	0.813	0	0.369	0.933
Ndufa11	Ndufa2	10116.ENSRNO	10116.ENSRNO	0	0	0	0	0.934	0.928	0.931	0.418	0.999
Ndufa11	Ndufab1	10116.ENSRNO	10116.ENSRNO	0	0	0	0	0.67	0.929	0.931	0.47	0.999
Ndufa11	Ndufv1	10116.ENSRNO	10116.ENSRNO	0	0	0	0	0.797	0.941	0.903	0.717	0.999
Ndufa11	Uqcrfs1	10116.ENSRNO	10116.ENSRNO	0	0	0	0	0.742	0.936	0	0.445	0.99
Ndufa11	Ndufaf3	10116.ENSRNO	10116.ENSRNO	0	0	0	0	0.409	0	0.903	0.759	0.985
Ndufa11	rCG_38845	10116.ENSRNO	10116.ENSRNO	0	0	0	0	0.693	0.707	0.931	0.363	0.995
Ndufa11	Uqcrb	10116.ENSRNO	10116.ENSRNO	0	0	0	0	0.679	0.543	0	0.378	0.9
Ndufa11	Ndufa9	10116.ENSRNO	10116.ENSRNO	0	0	0	0	0.762	0.939	0.932	0.583	0.999
Ndufa11	Ndufb7	10116.ENSRNO	10116.ENSRNO	0	0	0	0	0.942	0.929	0.951	0.666	0.999
Ndufa11	Ndufb6	10116.ENSRNO	10116.ENSRNO	0	0	0	0	0.933	0.939	0.903	0.515	0.999
Ndufa11	Ndufs7	10116.ENSRNO	10116.ENSRNO	0	0	0	0	0.911	0.941	0.903	0.645	0.999
Ndufa11	Ndufs5	10116.ENSRNO	10116.ENSRNO	0	0	0	0	0.819	0.817	0	0.533	0.983
Ndufa11	Uqcrc1	10116.ENSRNO	10116.ENSRNO	0	0	0	0	0.777	0.813	0	0.59	0.981
Ndufa11	Ndufa8	10116.ENSRNO	10116.ENSRNO	0	0	0	0	0.917	0.933	0.951	0.602	0.999

Ndufa11	Ndufs2	10116.ENSRNC	10116.ENSRNC	0	0	0	0	0.611	0.941	0.932	0.616	0.999
Ndufa12	Ndufv3	10116.ENSRNC	10116.ENSRNC	0	0	0	0	0.168	0.941	0.412	0.681	0.989
Ndufa12	Ndufb4	10116.ENSRNC	10116.ENSRNC	0	0	0	0	0.884	0.798	0.691	0.234	0.993
Ndufa12	Ndufa4	10116.ENSRNC	10116.ENSRNC	0	0	0	0	0.776	0.719	0.586	0.605	0.988
Ndufa12	Ndufa5	10116.ENSRNC	10116.ENSRNC	0	0	0	0	0.88	0.943	0.827	0.779	0.999
Ndufa12	Sdhb	10116.ENSRNC	10116.ENSRNC	0	0	0	0	0.545	0.859	0	0.177	0.942
Ndufa12	Uqcrc2	10116.ENSRNC	10116.ENSRNC	0	0	0	0	0.414	0.926	0	0.126	0.958
Ndufa12	Uqcrh	10116.ENSRNC	10116.ENSRNC	0	0	0	0	0.255	0.93	0	0.656	0.98
Ndufa12	Uqcrb	10116.ENSRNC	10116.ENSRNC	0	0	0	0	0.853	0.804	0	0.49	0.984
Ndufa12	Ndufs5	10116.ENSRNC	10116.ENSRNC	0	0	0	0	0.829	0.823	0	0.532	0.984
Ndufa12	Uqcrc1	10116.ENSRNC	10116.ENSRNC	0	0	0	0	0.528	0.941	0	0.476	0.984
Ndufa12	Uqcrfs1	10116.ENSRNC	10116.ENSRNC	0	0	0	0	0.746	0.941	0	0.292	0.988
Ndufa12	Ndufab1	10116.ENSRNC	10116.ENSRNC	0	0	0	0	0.718	0.928	0.754	0.327	0.996
Ndufa12	rCG_38845	10116.ENSRNC	10116.ENSRNC	0	0	0	0	0.703	0.928	0.754	0.475	0.996
Ndufa12	Ndufc2	10116.ENSRNC	10116.ENSRNC	0	0	0	0	0.744	0.925	0.827	0.528	0.998
Ndufa12	Ndufa2	10116.ENSRNC	10116.ENSRNC	0	0	0	0	0.864	0.928	0.754	0.358	0.998
Ndufa12	Ndufv1	10116.ENSRNC	10116.ENSRNC	0	0	0	0	0.593	0.943	0.827	0.753	0.998
Ndufa12	Ndufb8	10116.ENSRNC	10116.ENSRNC	0	0	0	0	0.786	0.941	0.827	0.27	0.998
Ndufa12	Ndufs2	10116.ENSRNC	10116.ENSRNC	0	0	0	0	0.406	0.943	0.827	0.698	0.998
Ndufa12	Ndufb10	10116.ENSRNC	10116.ENSRNC	0	0	0	0	0.813	0.928	0.754	0.633	0.998
Ndufa12	Ndufb7	10116.ENSRNC	10116.ENSRNC	0	0	0	0	0.764	0.943	0.827	0.343	0.998
Ndufa12	Ndufv2	10116.ENSRNC	10116.ENSRNC	0	0	0	0	0.807	0.943	0.827	0.737	0.999
Ndufa12	Ndufs1	10116.ENSRNC	10116.ENSRNC	0	0	0	0	0.213	0.982	0.827	0.726	0.999
Ndufa12	Ndufa8	10116.ENSRNC	10116.ENSRNC	0	0	0	0	0.788	0.93	0.827	0.756	0.999
Ndufa12	Ndufa9	10116.ENSRNC	10116.ENSRNC	0	0	0	0	0.689	0.982	0.827	0.607	0.999
Ndufa12	Ndufs4	10116.ENSRNC	10116.ENSRNC	0	0	0	0	0.878	0.982	0.913	0.743	0.999
Ndufa12	Ndufs3	10116.ENSRNC	10116.ENSRNC	0	0	0	0	0.82	0.982	0.827	0.525	0.999
Ndufa12	Ndufb6	10116.ENSRNC	10116.ENSRNC	0	0	0	0	0.898	0.941	0.827	0.6	0.999
Ndufa12	Ndufa6	10116.ENSRNC	10116.ENSRNC	0	0	0	0	0.935	0.982	0.827	0.66	0.999
Ndufa12	Ndufb9	10116.ENSRNC	10116.ENSRNC	0	0	0	0	0.922	0.982	0.827	0.82	0.999
Ndufa12	Ndufs7	10116.ENSRNC	10116.ENSRNC	0	0	0	0	0.68	0.982	0.827	0.679	0.999
Ndufa2	Ndufv3	10116.ENSRNC	10116.ENSRNC	0	0	0	0	0.294	0.928	0.67	0.474	0.99
Ndufa2	Ndufb4	10116.ENSRNC	10116.ENSRNC	0	0	0	0	0.852	0.662	0.849	0.53	0.996
Ndufa2	Ndufa4	10116.ENSRNC	10116.ENSRNC	0	0	0	0	0.642	0.927	0.67	0.63	0.996
Ndufa2	Ndufa5	10116.ENSRNC	10116.ENSRNC	0	0	0	0	0.845	0.928	0.931	0.572	0.999
Ndufa2	Sdhb	10116.ENSRNC	10116.ENSRNC	0	0	0	0	0.521	0.725	0	0.31	0.901
Ndufa2	Ndufa6	10116.ENSRNC	10116.ENSRNC	0	0	0	0	0.941	0.928	0.931	0.613	0.999
Ndufa2	Ndufs3	10116.ENSRNC	10116.ENSRNC	0	0	0	0	0.653	0.928	0.862	0.589	0.998
Ndufa2	Ndufb9	10116.ENSRNC	10116.ENSRNC	0	0	0	0	0.899	0.928	0.931	0.472	0.999
Ndufa2	Ndufs4	10116.ENSRNC	10116.ENSRNC	0	0	0	0	0.573	0.928	0.862	0.645	0.998
Ndufa2	Ndufs1	10116.ENSRNC	10116.ENSRNC	0	0	0	0	0.145	0.928	0.862	0.73	0.997
Ndufa2	Ndufc2	10116.ENSRNC	10116.ENSRNC	0	0	0	0	0.613	0.927	0.931	0.655	0.999
Ndufa2	Uqcrh	10116.ENSRNC	10116.ENSRNC	0	0	0	0	0.929	0.814	0.815	0.191	0.997
Ndufa2	Ndufv2	10116.ENSRNC	10116.ENSRNC	0	0	0	0	0.837	0.928	0.862	0.644	0.999
Ndufa2	Ndufb8	10116.ENSRNC	10116.ENSRNC	0	0	0	0	0.779	0.927	0.931	0.54	0.999
Ndufa2	Ndufb10	10116.ENSRNC	10116.ENSRNC	0	0	0	0	0.728	0.811	0.883	0.443	0.996
Ndufa2	Uqcrc2	10116.ENSRNC	10116.ENSRNC	0	0	0	0	0.128	0.812	0.67	0.443	0.965
Ndufa2	Ndufaf3	10116.ENSRNC	10116.ENSRNC	0	0	0	0	0.166	0	0.862	0.202	0.9
Ndufa2	Uqcrc1	10116.ENSRNC	10116.ENSRNC	0	0	0	0	0.18	0.807	0.209	0.448	0.921
Ndufa2	Usmg5	10116.ENSRNC	10116.ENSRNC	0	0	0	0	0.932	0	0	0.267	0.948
Ndufa2	Romo1	10116.ENSRNC	10116.ENSRNC	0	0	0	0	0.875	0.597	0	0.047	0.948
Ndufa2	Uqcrb	10116.ENSRNC	10116.ENSRNC	0	0	0	0	0.825	0.696	0.719	0.221	0.986
Ndufa2	Ndufs5	10116.ENSRNC	10116.ENSRNC	0	0	0	0	0.904	0.788	0	0.364	0.986
Ndufa2	Uqcrfs1	10116.ENSRNC	10116.ENSRNC	0	0	0	0	0.57	0.816	0.815	0.282	0.988
Ndufa2	Ndufab1	10116.ENSRNC	10116.ENSRNC	0	0	0	0	0.446	0.812	0.883	0.609	0.994
Ndufa2	rCG_38845	10116.ENSRNC	10116.ENSRNC	0	0	0	0	0.599	0.809	0.883	0.457	0.994
Ndufa2	Ndufa9	10116.ENSRNC	10116.ENSRNC	0	0	0	0	0.189	0.928	0.862	0.545	0.995

Ndufa2	Ndufv1	10116.ENSRNO	10116.ENSRNO	0	0	0	0	0.2	0.928	0.862	0.58	0.996
Ndufa2	Ndufs2	10116.ENSRNO	10116.ENSRNO	0	0	0	0	0.487	0.928	0.862	0.546	0.997
Ndufa2	Ndufb6	10116.ENSRNO	10116.ENSRNO	0	0	0	0	0.798	0.924	0.862	0.499	0.998
Ndufa2	Ndufa8	10116.ENSRNO	10116.ENSRNO	0	0	0	0	0.781	0.928	0.931	0.748	0.999
Ndufa2	Ndufs7	10116.ENSRNO	10116.ENSRNO	0	0	0	0	0.803	0.928	0.862	0.623	0.999
Ndufa2	Ndufb7	10116.ENSRNO	10116.ENSRNO	0	0	0	0	0.932	0.928	0.931	0.632	0.999
Ndufa4	Ndufv3	10116.ENSRNO	10116.ENSRNO	0	0	0	0	0.148	0.499	0.768	0.346	0.926
Ndufa4	Ndufb4	10116.ENSRNO	10116.ENSRNO	0	0	0	0	0.795	0.375	0.423	0.371	0.947
Ndufa4	Ndufa9	10116.ENSRNO	10116.ENSRNO	0	0	0	0	0.175	0.129	0.768	0.54	0.913
Ndufa4	Uqcrh	10116.ENSRNO	10116.ENSRNO	0	0	0	0	0.614	0.465	0	0.661	0.924
Ndufa4	Ndufs1	10116.ENSRNO	10116.ENSRNO	0	0	0	0	0.114	0.478	0.768	0.464	0.934
Ndufa4	Uqcrb	10116.ENSRNO	10116.ENSRNO	0	0	0	0	0.871	0.069	0	0.521	0.937
Ndufa4	Ndufs5	10116.ENSRNO	10116.ENSRNO	0	0	0	0	0.858	0.393	0	0.439	0.947
Ndufa4	Ndufb8	10116.ENSRNO	10116.ENSRNO	0	0	0	0	0.683	0.116	0.768	0.307	0.949
Ndufa4	Ndufs4	10116.ENSRNO	10116.ENSRNO	0	0	0	0	0.299	0.52	0.768	0.471	0.953
Ndufa4	Ndufb7	10116.ENSRNO	10116.ENSRNO	0	0	0	0	0.36	0.494	0.768	0.492	0.956
Ndufa4	Ndufv2	10116.ENSRNO	10116.ENSRNO	0	0	0	0	0.499	0.498	0.768	0.4	0.96
Ndufa4	Ndufv1	10116.ENSRNO	10116.ENSRNO	0	0	0	0	0.13	0.479	0.768	0.703	0.964
Ndufa4	Ndufs2	10116.ENSRNO	10116.ENSRNO	0	0	0	0	0.196	0.482	0.768	0.68	0.965
Ndufa4	Ndufab1	10116.ENSRNO	10116.ENSRNO	0	0	0	0	0.417	0.434	0.67	0.726	0.966
Ndufa4	Ndufa8	10116.ENSRNO	10116.ENSRNO	0	0	0	0	0.488	0.482	0.768	0.523	0.966
Ndufa4	Ndufs7	10116.ENSRNO	10116.ENSRNO	0	0	0	0	0.177	0.49	0.768	0.705	0.967
Ndufa4	Ndufb10	10116.ENSRNO	10116.ENSRNO	0	0	0	0	0.724	0.423	0.67	0.523	0.971
Ndufa4	Ndufb9	10116.ENSRNO	10116.ENSRNO	0	0	0	0	0.643	0.467	0.768	0.517	0.975
Ndufa4	Ndufc2	10116.ENSRNO	10116.ENSRNO	0	0	0	0	0.552	0.516	0.768	0.605	0.977
Ndufa4	Ndufb6	10116.ENSRNO	10116.ENSRNO	0	0	0	0	0.717	0	0.768	0.679	0.977
Ndufa4	Ndufs3	10116.ENSRNO	10116.ENSRNO	0	0	0	0	0.685	0.486	0.768	0.477	0.977
Ndufa4	Ndufa5	10116.ENSRNO	10116.ENSRNO	0	0	0	0	0.578	0.483	0.768	0.626	0.978
Ndufa4	Ndufa6	10116.ENSRNO	10116.ENSRNO	0	0	0	0	0.732	0.968	0.768	0.591	0.999
Ndufa5	Ndufv3	10116.ENSRNO	10116.ENSRNO	0	0	0	0	0.206	0.805	0.768	0.469	0.978
Ndufa5	Ndufb4	10116.ENSRNO	10116.ENSRNO	0	0	0	0	0.711	0.793	0.879	0.634	0.997
Ndufa5	Ndufaf3	10116.ENSRNO	10116.ENSRNO	0	0	0	0	0.121	0	0.903	0.215	0.927
Ndufa5	Uqcrc1	10116.ENSRNO	10116.ENSRNO	0	0	0	0	0.234	0.924	0	0.652	0.977
Ndufa5	Uqcrc2	10116.ENSRNO	10116.ENSRNO	0	0	0	0	0.573	0.929	0	0.472	0.982
Ndufa5	Ndufs5	10116.ENSRNO	10116.ENSRNO	0	0	0	0	0.833	0.852	0	0.561	0.988
Ndufa5	Uqcrb	10116.ENSRNO	10116.ENSRNO	0	0	0	0	0.818	0.807	0.67	0.571	0.994
Ndufa5	Uqcrfs1	10116.ENSRNO	10116.ENSRNO	0	0	0	0	0.676	0.93	0.783	0.521	0.997
Ndufa5	Ndufs1	10116.ENSRNO	10116.ENSRNO	0	0	0	0	0.225	0.941	0.932	0.705	0.998
Ndufa5	rCG_38845	10116.ENSRNO	10116.ENSRNO	0	0	0	0	0.602	0.926	0.931	0.433	0.998
Ndufa5	Ndufv1	10116.ENSRNO	10116.ENSRNO	0	0	0	0	0.437	0.941	0.903	0.626	0.998
Ndufa5	Ndufv2	10116.ENSRNO	10116.ENSRNO	0	0	0	0	0.946	0.941	0.932	0.653	0.999
Ndufa5	Uqcrh	10116.ENSRNO	10116.ENSRNO	0	0	0	0	0.77	0.928	0.898	0.641	0.999
Ndufa5	Ndufs4	10116.ENSRNO	10116.ENSRNO	0	0	0	0	0.881	0.939	0.932	0.546	0.999
Ndufa5	Ndufa6	10116.ENSRNO	10116.ENSRNO	0	0	0	0	0.959	0.97	0.951	0.622	0.999
Ndufa5	Ndufs7	10116.ENSRNO	10116.ENSRNO	0	0	0	0	0.735	0.97	0.903	0.756	0.999
Ndufa5	Ndufb6	10116.ENSRNO	10116.ENSRNO	0	0	0	0	0.831	0.941	0.903	0.499	0.999
Ndufa5	Ndufs2	10116.ENSRNO	10116.ENSRNO	0	0	0	0	0.587	0.941	0.932	0.655	0.999
Ndufa5	Ndufb8	10116.ENSRNO	10116.ENSRNO	0	0	0	0	0.913	0.93	0.951	0.426	0.999
Ndufa5	Ndufs3	10116.ENSRNO	10116.ENSRNO	0	0	0	0	0.809	0.99	0.903	0.764	0.999
Ndufa5	Ndufc2	10116.ENSRNO	10116.ENSRNO	0	0	0	0	0.769	0.941	0.951	0.665	0.999
Ndufa5	Ndufa9	10116.ENSRNO	10116.ENSRNO	0	0	0	0	0.486	0.965	0.932	0.632	0.999
Ndufa5	Ndufa8	10116.ENSRNO	10116.ENSRNO	0	0	0	0	0.84	0.941	0.951	0.621	0.999
Ndufa5	Ndufab1	10116.ENSRNO	10116.ENSRNO	0	0	0	0	0.724	0.929	0.931	0.583	0.999
Ndufa5	Ndufb10	10116.ENSRNO	10116.ENSRNO	0	0	0	0	0.812	0.916	0.931	0.676	0.999
Ndufa5	Ndufb9	10116.ENSRNO	10116.ENSRNO	0	0	0	0	0.943	0.942	0.951	0.778	0.999
Ndufa5	Ndufb7	10116.ENSRNO	10116.ENSRNO	0	0	0	0	0.91	0.941	0.951	0.633	0.999
Ndufa6	Ndufv3	10116.ENSRNO	10116.ENSRNO	0	0	0	0	0.235	0.943	0.768	0.455	0.993

Ndufa6	Ndufb4	10116.ENSRNC	10116.ENSRNC	0	0	0	0	0.748	0.804	0.879	0.633	0.997
Ndufa6	Sdhb	10116.ENSRNC	10116.ENSRNC	0	0	0	0	0.545	0.854	0	0.414	0.957
Ndufa6	Ndufaf3	10116.ENSRNC	10116.ENSRNC	0	0	0	0	0.155	0	0.903	0.482	0.954
Ndufa6	Uqcrc1	10116.ENSRNC	10116.ENSRNC	0	0	0	0	0.278	0.93	0.244	0.474	0.977
Ndufa6	Ndufs5	10116.ENSRNC	10116.ENSRNC	0	0	0	0	0.789	0.853	0	0.633	0.987
Ndufa6	Uqcrc2	10116.ENSRNC	10116.ENSRNC	0	0	0	0	0.196	0.929	0.783	0.433	0.992
Ndufa6	Uqcrb	10116.ENSRNC	10116.ENSRNC	0	0	0	0	0.827	0.805	0.67	0.374	0.992
Ndufa6	Uqcrfs1	10116.ENSRNC	10116.ENSRNC	0	0	0	0	0.488	0.929	0.783	0.365	0.994
Ndufa6	rCG_38845	10116.ENSRNC	10116.ENSRNC	0	0	0	0	0.711	0.926	0.931	0.264	0.998
Ndufa6	Ndufb10	10116.ENSRNC	10116.ENSRNC	0	0	0	0	0.875	0.927	0.931	0.624	0.999
Ndufa6	Ndufb9	10116.ENSRNC	10116.ENSRNC	0	0	0	0	0.958	0.988	0.951	0.784	0.999
Ndufa6	Ndufb7	10116.ENSRNC	10116.ENSRNC	0	0	0	0	0.952	0.972	0.951	0.518	0.999
Ndufa6	Ndufv1	10116.ENSRNC	10116.ENSRNC	0	0	0	0	0.415	0.99	0.903	0.619	0.999
Ndufa6	Ndufa9	10116.ENSRNC	10116.ENSRNC	0	0	0	0	0.573	0.99	0.903	0.595	0.999
Ndufa6	Ndufa8	10116.ENSRNC	10116.ENSRNC	0	0	0	0	0.846	0.972	0.951	0.673	0.999
Ndufa6	Ndufs1	10116.ENSRNC	10116.ENSRNC	0	0	0	0	0.172	0.99	0.903	0.652	0.999
Ndufa6	Ndufab1	10116.ENSRNC	10116.ENSRNC	0	0	0	0	0.718	0.929	0.931	0.682	0.999
Ndufa6	Ndufc2	10116.ENSRNC	10116.ENSRNC	0	0	0	0	0.85	0.971	0.951	0.407	0.999
Ndufa6	Ndufs7	10116.ENSRNC	10116.ENSRNC	0	0	0	0	0.802	0.972	0.903	0.684	0.999
Ndufa6	Ndufb6	10116.ENSRNC	10116.ENSRNC	0	0	0	0	0.869	0.967	0.903	0.585	0.999
Ndufa6	Ndufs2	10116.ENSRNC	10116.ENSRNC	0	0	0	0	0.287	0.972	0.903	0.608	0.999
Ndufa6	Ndufb8	10116.ENSRNC	10116.ENSRNC	0	0	0	0	0.914	0.966	0.951	0.733	0.999
Ndufa6	Ndufs3	10116.ENSRNC	10116.ENSRNC	0	0	0	0	0.837	0.99	0.903	0.548	0.999
Ndufa6	Uqcrh	10116.ENSRNC	10116.ENSRNC	0	0	0	0	0.822	0.929	0.898	0.496	0.999
Ndufa6	Ndufs4	10116.ENSRNC	10116.ENSRNC	0	0	0	0	0.759	0.966	0.903	0.741	0.999
Ndufa6	Ndufv2	10116.ENSRNC	10116.ENSRNC	0	0	0	0	0.864	0.99	0.903	0.48	0.999
Ndufa8	Ndufv3	10116.ENSRNC	10116.ENSRNC	0	0	0	0	0.201	0.899	0.768	0.257	0.984
Ndufa8	Ndufb4	10116.ENSRNC	10116.ENSRNC	0	0	0	0	0.519	0.797	0.879	0.168	0.988
Ndufa8	Sdhc	10116.ENSRNC	10116.ENSRNC	0	0	0	0	0.87	0.069	0	0.249	0.901
Ndufa8	Sdhb	10116.ENSRNC	10116.ENSRNC	0	0	0	0	0.799	0.925	0	0.492	0.991
Ndufa8	Ndufs3	10116.ENSRNC	10116.ENSRNC	0	0	0	0	0.931	0.942	0.903	0.684	0.999
Ndufa8	Ndufb9	10116.ENSRNC	10116.ENSRNC	0	0	0	0	0.933	0.94	0.951	0.759	0.999
Ndufa8	Ndufs4	10116.ENSRNC	10116.ENSRNC	0	0	0	0	0.803	0.941	0.903	0.657	0.999
Ndufa8	Ndufs1	10116.ENSRNC	10116.ENSRNC	0	0	0	0	0.556	0.942	0.903	0.666	0.999
Ndufa8	Ndufc2	10116.ENSRNC	10116.ENSRNC	0	0	0	0	0.834	0.942	0.951	0.518	0.999
Ndufa8	Uqcrh	10116.ENSRNC	10116.ENSRNC	0	0	0	0	0.695	0.93	0.898	0.355	0.998
Ndufa8	Ndufv2	10116.ENSRNC	10116.ENSRNC	0	0	0	0	0.803	0.942	0.903	0.607	0.999
Ndufa8	Ndufb8	10116.ENSRNC	10116.ENSRNC	0	0	0	0	0.916	0.938	0.951	0.262	0.999
Ndufa8	Ndufb10	10116.ENSRNC	10116.ENSRNC	0	0	0	0	0.896	0.911	0.931	0.689	0.999
Ndufa8	Uqcrc2	10116.ENSRNC	10116.ENSRNC	0	0	0	0	0.596	0.93	0.783	0.508	0.996
Ndufa8	Ndufab1	10116.ENSRNC	10116.ENSRNC	0	0	0	0	0.679	0.929	0.931	0.509	0.999
Ndufa8	Ndufv1	10116.ENSRNC	10116.ENSRNC	0	0	0	0	0.776	0.942	0.903	0.662	0.999
Ndufa8	Uqcrfs1	10116.ENSRNC	10116.ENSRNC	0	0	0	0	0.846	0.931	0.898	0.549	0.999
Ndufa8	Ndufaf3	10116.ENSRNC	10116.ENSRNC	0	0	0	0	0.228	0.801	0.903	0.127	0.985
Ndufa8	rCG_38845	10116.ENSRNC	10116.ENSRNC	0	0	0	0	0.697	0.809	0.931	0.195	0.996
Ndufa8	Uqcrb	10116.ENSRNC	10116.ENSRNC	0	0	0	0	0.669	0.734	0.815	0.446	0.989
Ndufa8	Ndufa9	10116.ENSRNC	10116.ENSRNC	0	0	0	0	0.768	0.941	0.903	0.705	0.999
Ndufa8	Ndufb7	10116.ENSRNC	10116.ENSRNC	0	0	0	0	0.905	0.942	0.951	0.637	0.999
Ndufa8	Ndufb6	10116.ENSRNC	10116.ENSRNC	0	0	0	0	0.951	0.941	0.903	0.644	0.999
Ndufa8	Ndufs7	10116.ENSRNC	10116.ENSRNC	0	0	0	0	0.89	0.942	0.903	0.695	0.999
Ndufa8	Ndufs5	10116.ENSRNC	10116.ENSRNC	0	0	0	0	0.734	0.82	0	0.612	0.979
Ndufa8	Uqcrc1	10116.ENSRNC	10116.ENSRNC	0	0	0	0	0.669	0.93	0.244	0.441	0.988
Ndufa8	Ndufs2	10116.ENSRNC	10116.ENSRNC	0	0	0	0	0.88	0.942	0.903	0.69	0.999
Ndufa9	Ndufv3	10116.ENSRNC	10116.ENSRNC	0	0	0	0	0.123	0.941	0.768	0.496	0.993
Ndufa9	Ndufb4	10116.ENSRNC	10116.ENSRNC	0	0	0	0	0.295	0.803	0.759	0.576	0.983
Ndufa9	Sdhb	10116.ENSRNC	10116.ENSRNC	0	0	0	0	0.724	0.434	0.44	0.684	0.968
Ndufa9	Ndufs3	10116.ENSRNC	10116.ENSRNC	0.095	0	0	0	0.919	0.99	0.903	0.849	0.999

Ndufa9	Ndufb9	10116.ENSRNO	10116.ENSRNO	0	0	0	0	0.7	0.941	0.903	0.599	0.999
Ndufa9	Ndufs4	10116.ENSRNO	10116.ENSRNO	0	0	0	0	0.694	0.956	0.932	0.703	0.999
Ndufa9	Ndufs1	10116.ENSRNO	10116.ENSRNO	0	0	0.201	0	0.701	0.99	0.932	0.672	0.999
Ndufa9	Ndufc2	10116.ENSRNO	10116.ENSRNO	0	0	0	0	0.486	0.93	0.903	0.533	0.998
Ndufa9	Uqcrh	10116.ENSRNO	10116.ENSRNO	0	0	0	0	0.121	0.923	0.67	0.363	0.983
Ndufa9	Ndufv2	10116.ENSRNO	10116.ENSRNO	0	0	0	0	0.711	0.988	0.932	0.721	0.999
Ndufa9	Sdha	10116.ENSRNO	10116.ENSRNO	0.042	0	0	0	0.533	0.431	0.44	0.908	0.984
Ndufa9	Ndufb8	10116.ENSRNO	10116.ENSRNO	0	0	0	0	0.664	0.988	0.903	0.714	0.999
Ndufa9	Ndufb10	10116.ENSRNO	10116.ENSRNO	0	0	0	0	0.689	0.928	0.862	0.449	0.998
Ndufa9	Uqcrc2	10116.ENSRNO	10116.ENSRNO	0	0	0	0	0.871	0.932	0	0.854	0.998
Ndufa9	Ndufab1	10116.ENSRNO	10116.ENSRNO	0.096	0	0	0	0.554	0.929	0.862	0.477	0.997
Ndufa9	Ndufv1	10116.ENSRNO	10116.ENSRNO	0	0	0	0	0.795	0.988	0.903	0.721	0.999
Ndufa9	Uqcrfs1	10116.ENSRNO	10116.ENSRNO	0.044	0	0	0	0.816	0.984	0.67	0.695	0.999
Ndufa9	Ndufaf3	10116.ENSRNO	10116.ENSRNO	0	0	0	0	0.086	0	0.903	0.221	0.925
Ndufa9	rCG_38845	10116.ENSRNO	10116.ENSRNO	0	0	0	0	0.637	0.926	0.862	0.384	0.997
Ndufa9	Ndufs5	10116.ENSRNO	10116.ENSRNO	0	0	0	0	0.258	0.853	0	0.431	0.932
Ndufa9	Uqcrc1	10116.ENSRNO	10116.ENSRNO	0	0	0	0	0.735	0.925	0	0.799	0.995
Ndufa9	Ndufs2	10116.ENSRNO	10116.ENSRNO	0.042	0	0	0	0.718	0.99	0.932	0.658	0.999
Ndufa9	Ndufb6	10116.ENSRNO	10116.ENSRNO	0	0	0	0	0.76	0.931	0.903	0.615	0.999
Ndufa9	Ndufs7	10116.ENSRNO	10116.ENSRNO	0.098	0	0	0	0.72	0.99	0.903	0.804	0.999
Ndufa9	Ndufb7	10116.ENSRNO	10116.ENSRNO	0	0	0	0	0.673	0.966	0.903	0.213	0.999
Ndufab1	Ndufv3	10116.ENSRNO	10116.ENSRNO	0	0	0	0	0.131	0.929	0.67	0.583	0.99
Ndufab1	Ndufb4	10116.ENSRNO	10116.ENSRNO	0	0	0	0	0.376	0.696	0.849	0.679	0.989
Ndufab1	Oxsm	10116.ENSRNO	10116.ENSRNO	0.128	0	0	0	0.119	0.554	0.67	0.625	0.949
Ndufab1	Ndufs3	10116.ENSRNO	10116.ENSRNO	0	0	0	0	0.679	0.929	0.862	0.648	0.998
Ndufab1	Ndufb9	10116.ENSRNO	10116.ENSRNO	0	0	0	0	0.728	0.929	0.931	0.519	0.999
Ndufab1	Ndufs4	10116.ENSRNO	10116.ENSRNO	0	0	0	0	0.707	0.929	0.862	0.545	0.998
Ndufab1	Ndufs1	10116.ENSRNO	10116.ENSRNO	0	0	0	0	0.394	0.929	0.862	0.747	0.998
Ndufab1	Ndufc2	10116.ENSRNO	10116.ENSRNO	0	0	0	0	0.692	0.929	0.931	0.657	0.999
Ndufab1	Uqcrh	10116.ENSRNO	10116.ENSRNO	0	0	0	0	0.608	0.807	0	0.542	0.962
Ndufab1	Ndufv2	10116.ENSRNO	10116.ENSRNO	0	0	0	0	0.643	0.929	0.862	0.555	0.998
Ndufab1	Ndufb8	10116.ENSRNO	10116.ENSRNO	0	0	0	0	0.692	0.929	0.931	0.479	0.999
Ndufab1	Ndufb10	10116.ENSRNO	10116.ENSRNO	0	0	0	0	0.665	0.812	0.883	0.495	0.995
Ndufab1	Uqcrc2	10116.ENSRNO	10116.ENSRNO	0.042	0	0	0	0.584	0.814	0	0.313	0.942
Ndufab1	Ndufaf3	10116.ENSRNO	10116.ENSRNO	0	0	0	0	0.101	0	0.862	0.469	0.928
Ndufab1	Uqcrc1	10116.ENSRNO	10116.ENSRNO	0.042	0	0	0	0.467	0.807	0	0.457	0.939
Ndufab1	Ndufs5	10116.ENSRNO	10116.ENSRNO	0	0	0	0	0.539	0.791	0	0.585	0.956
Ndufab1	Uqcrfs1	10116.ENSRNO	10116.ENSRNO	0	0	0	0	0.697	0.813	0.448	0.519	0.982
Ndufab1	rCG_38845	10116.ENSRNO	10116.ENSRNO	0	0	0	0	0.606	0.812	0.883	0.358	0.993
Ndufab1	Ndufv1	10116.ENSRNO	10116.ENSRNO	0	0	0	0	0.139	0.929	0.862	0.715	0.997
Ndufab1	Ndufs7	10116.ENSRNO	10116.ENSRNO	0	0	0	0	0.615	0.929	0.862	0.685	0.998
Ndufab1	Ndufb6	10116.ENSRNO	10116.ENSRNO	0	0	0	0	0.687	0.929	0.862	0.626	0.998
Ndufab1	Ndufs2	10116.ENSRNO	10116.ENSRNO	0	0	0	0	0.49	0.929	0.862	0.683	0.998
Ndufab1	Ndufb7	10116.ENSRNO	10116.ENSRNO	0	0	0	0	0.672	0.929	0.931	0.445	0.998
Ndufaf3	Ndufs3	10116.ENSRNO	10116.ENSRNO	0	0	0	0	0.514	0.924	0.903	0.57	0.998
Ndufaf3	Ndufb9	10116.ENSRNO	10116.ENSRNO	0	0	0	0	0.198	0	0.903	0.41	0.95
Ndufaf3	Ndufc2	10116.ENSRNO	10116.ENSRNO	0	0	0	0	0.124	0	0.903	0.161	0.922
Ndufaf3	Ndufb8	10116.ENSRNO	10116.ENSRNO	0	0	0	0	0.165	0	0.903	0.338	0.941
Ndufaf3	Ndufb10	10116.ENSRNO	10116.ENSRNO	0	0	0	0	0.41	0.683	0.862	0.24	0.977
Ndufaf3	Ndufv1	10116.ENSRNO	10116.ENSRNO	0	0	0	0	0.369	0	0.768	0.496	0.919
Ndufaf3	Ndufb7	10116.ENSRNO	10116.ENSRNO	0	0	0	0	0.587	0	0.903	0	0.958
Ndufaf3	Ndufb6	10116.ENSRNO	10116.ENSRNO	0	0	0	0	0.503	0	0.903	0.202	0.958
Ndufaf3	rCG_38845	10116.ENSRNO	10116.ENSRNO	0	0	0	0	0.376	0.531	0.862	0.282	0.967
Ndufaf3	Ndufs7	10116.ENSRNO	10116.ENSRNO	0	0	0	0	0.467	0.801	0.903	0.517	0.994
Ndufaf3	Ndufs2	10116.ENSRNO	10116.ENSRNO	0	0	0	0	0.187	0.924	0.903	0.566	0.997
Ndufb10	Ndufv3	10116.ENSRNO	10116.ENSRNO	0	0	0	0	0.445	0.69	0.67	0.248	0.951
Ndufb10	Ndufb4	10116.ENSRNO	10116.ENSRNO	0	0	0	0	0.694	0.641	0.849	0.246	0.985

Ndufb10	Sdhc	10116.ENSRNO	10116.ENSRNO	0	0	0	0	0.831	0	0	0.445	0.902
Ndufb10	Sdhb	10116.ENSRNO	10116.ENSRNO	0	0	0	0	0.805	0.37	0	0.458	0.927
Ndufb10	Ndufs3	10116.ENSRNO	10116.ENSRNO	0	0	0	0	0.947	0.928	0.862	0.557	0.999
Ndufb10	Ndufb9	10116.ENSRNO	10116.ENSRNO	0	0	0	0	0.895	0.853	0.931	0.71	0.999
Ndufb10	Ndufs4	10116.ENSRNO	10116.ENSRNO	0	0	0	0	0.687	0.928	0.862	0.36	0.997
Ndufb10	Ndufs1	10116.ENSRNO	10116.ENSRNO	0	0	0	0	0.258	0.924	0.862	0.476	0.995
Ndufb10	Ndufc2	10116.ENSRNO	10116.ENSRNO	0	0	0	0	0.768	0.815	0.931	0.364	0.997
Ndufb10	Uqcrh	10116.ENSRNO	10116.ENSRNO	0	0	0	0	0.634	0.813	0.729	0.444	0.988
Ndufb10	Ndufv2	10116.ENSRNO	10116.ENSRNO	0	0	0	0	0.847	0.915	0.862	0.591	0.999
Ndufb10	Ndufb8	10116.ENSRNO	10116.ENSRNO	0	0	0	0	0.843	0.817	0.931	0.286	0.998
Ndufb10	Uqcrb	10116.ENSRNO	10116.ENSRNO	0	0	0	0	0.627	0.691	0	0.279	0.909
Ndufb10	Uqcrc2	10116.ENSRNO	10116.ENSRNO	0	0	0	0	0.574	0.815	0	0.314	0.941
Ndufb10	Uqcrfs1	10116.ENSRNO	10116.ENSRNO	0	0	0	0	0.698	0.812	0	0.342	0.959
Ndufb10	Uqcrc1	10116.ENSRNO	10116.ENSRNO	0	0	0	0	0.772	0.812	0	0.36	0.97
Ndufb10	Ndufs5	10116.ENSRNO	10116.ENSRNO	0	0	0	0	0.876	0.788	0	0.472	0.985
Ndufb10	rCG_38845	10116.ENSRNO	10116.ENSRNO	0	0	0	0	0.646	0.696	0.883	0.622	0.994
Ndufb10	Ndufs2	10116.ENSRNO	10116.ENSRNO	0	0	0	0	0.275	0.915	0.862	0.607	0.996
Ndufb10	Ndufv1	10116.ENSRNO	10116.ENSRNO	0	0	0	0	0.598	0.915	0.862	0.554	0.997
Ndufb10	Ndufb7	10116.ENSRNO	10116.ENSRNO	0	0	0	0	0.933	0.918	0.931	0.66	0.999
Ndufb10	Ndufb6	10116.ENSRNO	10116.ENSRNO	0	0	0	0	0.88	0.918	0.862	0.662	0.999
Ndufb10	Ndufs7	10116.ENSRNO	10116.ENSRNO	0	0	0	0	0.9	0.922	0.862	0.408	0.999
Ndufb4	Uqcrh	10116.ENSRNO	10116.ENSRNO	0	0	0	0	0.707	0.647	0	0.298	0.921
Ndufb4	Uqcrb	10116.ENSRNO	10116.ENSRNO	0	0	0	0	0.753	0.459	0	0.499	0.927
Ndufb4	Uqcrc1	10116.ENSRNO	10116.ENSRNO	0	0	0	0	0.157	0.647	0	0.784	0.93
Ndufb4	Ndufs5	10116.ENSRNO	10116.ENSRNO	0	0	0	0	0.773	0.569	0	0.391	0.935
Ndufb4	Ndufs2	10116.ENSRNO	10116.ENSRNO	0	0	0	0	0.172	0.797	0.759	0.167	0.961
Ndufb4	rCG_38845	10116.ENSRNO	10116.ENSRNO	0	0	0	0	0.284	0.525	0.849	0.396	0.965
Ndufb4	Ndufv1	10116.ENSRNO	10116.ENSRNO	0	0	0	0	0.123	0.804	0.759	0.259	0.965
Ndufb4	Ndufs1	10116.ENSRNO	10116.ENSRNO	0	0	0	0	0.101	0.795	0.759	0.304	0.965
Ndufb4	Ndufv2	10116.ENSRNO	10116.ENSRNO	0	0	0	0	0.606	0.797	0.759	0.315	0.985
Ndufb4	Ndufs4	10116.ENSRNO	10116.ENSRNO	0	0	0	0	0.544	0.803	0.759	0.43	0.986
Ndufb4	Ndufs7	10116.ENSRNO	10116.ENSRNO	0	0	0	0	0.583	0.795	0.759	0.444	0.987
Ndufb4	Ndufb6	10116.ENSRNO	10116.ENSRNO	0	0	0	0	0.78	0.696	0.759	0.337	0.987
Ndufb4	Ndufs3	10116.ENSRNO	10116.ENSRNO	0	0	0	0	0.453	0.806	0.759	0.604	0.988
Ndufb4	Ndufc2	10116.ENSRNO	10116.ENSRNO	0	0	0	0	0.521	0.796	0.879	0.312	0.99
Ndufb4	Ndufb8	10116.ENSRNO	10116.ENSRNO	0	0	0	0	0.55	0.696	0.879	0.587	0.992
Ndufb4	Ndufb7	10116.ENSRNO	10116.ENSRNO	0	0	0	0	0.56	0.797	0.879	0.596	0.995
Ndufb4	Ndufb9	10116.ENSRNO	10116.ENSRNO	0	0	0	0	0.814	0.707	0.879	0.686	0.997
Ndufb6	Ndufv3	10116.ENSRNO	10116.ENSRNO	0	0	0	0	0.28	0.805	0.768	0.48	0.98
Ndufb6	Sdhc	10116.ENSRNO	10116.ENSRNO	0	0	0	0	0.69	0.312	0	0.579	0.902
Ndufb6	Sdhb	10116.ENSRNO	10116.ENSRNO	0	0	0	0	0.758	0.482	0	0.499	0.931
Ndufb6	Ndufs3	10116.ENSRNO	10116.ENSRNO	0	0	0	0	0.88	0.941	0.903	0.544	0.999
Ndufb6	Ndufb9	10116.ENSRNO	10116.ENSRNO	0	0	0	0	0.898	0.931	0.903	0.432	0.999
Ndufb6	Ndufs4	10116.ENSRNO	10116.ENSRNO	0	0	0	0	0.771	0.938	0.903	0.54	0.999
Ndufb6	Ndufs1	10116.ENSRNO	10116.ENSRNO	0	0	0	0	0.225	0.941	0.903	0.532	0.997
Ndufb6	Ndufc2	10116.ENSRNO	10116.ENSRNO	0	0	0	0	0.841	0.93	0.903	0.602	0.999
Ndufb6	Uqcrh	10116.ENSRNO	10116.ENSRNO	0	0	0	0	0.78	0.929	0	0.183	0.986
Ndufb6	Ndufv2	10116.ENSRNO	10116.ENSRNO	0	0	0	0	0.783	0.941	0.903	0.644	0.999
Ndufb6	Ndufb8	10116.ENSRNO	10116.ENSRNO	0	0	0	0	0.869	0.931	0.903	0.54	0.999
Ndufb6	Uqcrc2	10116.ENSRNO	10116.ENSRNO	0	0	0	0	0.404	0.929	0	0.538	0.978
Ndufb6	Ndufv1	10116.ENSRNO	10116.ENSRNO	0	0	0	0	0.39	0.94	0.903	0.758	0.999
Ndufb6	Uqcrfs1	10116.ENSRNO	10116.ENSRNO	0	0	0	0	0.684	0.929	0	0.3	0.982
Ndufb6	rCG_38845	10116.ENSRNO	10116.ENSRNO	0	0	0	0	0.675	0.809	0.862	0.456	0.994
Ndufb6	Uqcrb	10116.ENSRNO	10116.ENSRNO	0	0	0	0	0.798	0.546	0	0.168	0.917
Ndufb6	Ndufb7	10116.ENSRNO	10116.ENSRNO	0	0	0	0	0.86	0.941	0.903	0.662	0.999
Ndufb6	Uqcrc1	10116.ENSRNO	10116.ENSRNO	0	0	0	0	0.252	0.923	0	0.598	0.974
Ndufb6	Ndufs5	10116.ENSRNO	10116.ENSRNO	0	0	0	0	0.788	0.852	0	0.523	0.983

Ndufb6	Ndufs2	10116.ENSRNO	10116.ENSRNO	0	0	0	0	0.335	0.931	0.903	0.624	0.998
Ndufb6	Ndufs7	10116.ENSRNO	10116.ENSRNO	0	0	0	0	0.736	0.941	0.903	0.584	0.999
Ndufb7	Ndufv3	10116.ENSRNO	10116.ENSRNO	0	0	0	0	0.44	0.805	0.768	0.354	0.981
Ndufb7	Ndufs3	10116.ENSRNO	10116.ENSRNO	0	0	0	0	0.895	0.984	0.903	0.711	0.999
Ndufb7	Ndufb9	10116.ENSRNO	10116.ENSRNO	0	0	0	0	0.921	0.939	0.951	0.585	0.999
Ndufb7	Ndufs4	10116.ENSRNO	10116.ENSRNO	0	0	0	0	0.878	0.94	0.903	0.55	0.999
Ndufb7	Ndufs1	10116.ENSRNO	10116.ENSRNO	0	0	0	0	0.194	0.942	0.903	0.697	0.998
Ndufb7	Ndufc2	10116.ENSRNO	10116.ENSRNO	0	0	0	0	0.805	0.942	0.951	0.27	0.999
Ndufb7	Uqcrh	10116.ENSRNO	10116.ENSRNO	0	0	0	0	0.936	0.929	0.783	0.659	0.999
Ndufb7	Ndufv2	10116.ENSRNO	10116.ENSRNO	0	0	0	0	0.935	0.942	0.903	0.521	0.999
Ndufb7	Ndufb8	10116.ENSRNO	10116.ENSRNO	0	0	0	0	0.901	0.931	0.951	0.65	0.999
Ndufb7	Uqcrc2	10116.ENSRNO	10116.ENSRNO	0	0	0	0	0.498	0.933	0.783	0.118	0.992
Ndufb7	Ndufv1	10116.ENSRNO	10116.ENSRNO	0	0	0	0	0.828	0.942	0.903	0.806	0.999
Ndufb7	Uqcrfs1	10116.ENSRNO	10116.ENSRNO	0	0	0	0	0.541	0.93	0.887	0.495	0.997
Ndufb7	Romo1	10116.ENSRNO	10116.ENSRNO	0	0	0	0	0.877	0.72	0	0	0.964
Ndufb7	rCG_38845	10116.ENSRNO	10116.ENSRNO	0	0	0	0	0.746	0.809	0.931	0.416	0.997
Ndufb7	Uqcrb	10116.ENSRNO	10116.ENSRNO	0	0	0	0	0.653	0.805	0.67	0.539	0.988
Ndufb7	Ndufs5	10116.ENSRNO	10116.ENSRNO	0	0	0	0	0.818	0.852	0	0.657	0.99
Ndufb7	Uqcrc1	10116.ENSRNO	10116.ENSRNO	0	0	0	0	0.828	0.929	0.244	0.441	0.994
Ndufb7	Ndufs7	10116.ENSRNO	10116.ENSRNO	0	0	0	0	0.903	0.942	0.903	0.726	0.999
Ndufb7	Ndufs2	10116.ENSRNO	10116.ENSRNO	0	0	0	0	0.812	0.942	0.903	0.598	0.999
Ndufb8	Ndufv3	10116.ENSRNO	10116.ENSRNO	0	0	0	0	0.17	0.696	0.768	0.267	0.951
Ndufb8	Sdhc	10116.ENSRNO	10116.ENSRNO	0	0	0	0	0.839	0	0	0.447	0.907
Ndufb8	Sdhb	10116.ENSRNO	10116.ENSRNO	0	0	0	0	0.706	0	0	0.894	0.967
Ndufb8	Ndufs3	10116.ENSRNO	10116.ENSRNO	0	0	0	0	0.791	0.988	0.903	0.803	0.999
Ndufb8	Ndufb9	10116.ENSRNO	10116.ENSRNO	0	0	0	0	0.946	0.941	0.951	0.667	0.999
Ndufb8	Ndufs4	10116.ENSRNO	10116.ENSRNO	0	0	0	0	0.795	0.956	0.903	0.467	0.999
Ndufb8	Ndufs1	10116.ENSRNO	10116.ENSRNO	0	0	0	0	0.581	0.931	0.903	0.619	0.998
Ndufb8	Ndufc2	10116.ENSRNO	10116.ENSRNO	0	0	0	0	0.754	0.93	0.951	0.336	0.999
Ndufb8	Uqcrh	10116.ENSRNO	10116.ENSRNO	0	0	0	0	0.619	0.923	0.821	0.394	0.996
Ndufb8	Ndufv2	10116.ENSRNO	10116.ENSRNO	0	0	0	0	0.745	0.931	0.903	0.586	0.999
Ndufb8	Ndufs5	10116.ENSRNO	10116.ENSRNO	0	0	0	0	0.679	0.852	0	0.592	0.979
Ndufb8	Uqcrc1	10116.ENSRNO	10116.ENSRNO	0	0	0	0	0.512	0.925	0	0.624	0.985
Ndufb8	Uqcrfs1	10116.ENSRNO	10116.ENSRNO	0	0	0	0	0.787	0.929	0	0.568	0.992
Ndufb8	rCG_38845	10116.ENSRNO	10116.ENSRNO	0	0	0	0	0.693	0.804	0.931	0.332	0.996
Ndufb8	Uqcrc2	10116.ENSRNO	10116.ENSRNO	0	0	0	0	0.547	0.932	0	0.897	0.996
Ndufb8	Ndufv1	10116.ENSRNO	10116.ENSRNO	0	0	0	0	0.628	0.934	0.903	0.577	0.998
Ndufb8	Ndufs7	10116.ENSRNO	10116.ENSRNO	0	0	0	0	0.868	0.934	0.903	0.735	0.999
Ndufb8	Ndufs2	10116.ENSRNO	10116.ENSRNO	0	0	0	0	0.734	0.931	0.903	0.628	0.999
Ndufb9	Ndufv3	10116.ENSRNO	10116.ENSRNO	0	0	0	0	0.204	0.696	0.768	0.343	0.958
Ndufb9	Sdhc	10116.ENSRNO	10116.ENSRNO	0	0	0	0	0.862	0	0	0.522	0.931
Ndufb9	Sdhb	10116.ENSRNO	10116.ENSRNO	0	0	0	0	0.739	0.839	0	0.498	0.977
Ndufb9	Ndufs3	10116.ENSRNO	10116.ENSRNO	0	0	0	0	0.904	0.942	0.903	0.706	0.999
Ndufb9	Uqcrc2	10116.ENSRNO	10116.ENSRNO	0	0	0	0	0.51	0.929	0	0.451	0.979
Ndufb9	Ndufs5	10116.ENSRNO	10116.ENSRNO	0	0	0	0	0.883	0.853	0	0.468	0.99
Ndufb9	Uqcrb	10116.ENSRNO	10116.ENSRNO	0	0	0	0	0.834	0.807	0.67	0.588	0.995
Ndufb9	Uqcrc1	10116.ENSRNO	10116.ENSRNO	0	0	0	0	0.577	0.925	0	0.879	0.995
Ndufb9	Uqcrh	10116.ENSRNO	10116.ENSRNO	0	0	0	0	0.636	0.923	0.783	0.62	0.997
Ndufb9	rCG_38845	10116.ENSRNO	10116.ENSRNO	0	0	0	0	0.715	0.808	0.931	0.354	0.997
Ndufb9	Uqcrfs1	10116.ENSRNO	10116.ENSRNO	0	0	0	0	0.824	0.928	0.783	0.606	0.998
Ndufb9	Ndufv2	10116.ENSRNO	10116.ENSRNO	0	0	0	0	0.881	0.934	0.903	0.619	0.999
Ndufb9	Ndufs2	10116.ENSRNO	10116.ENSRNO	0	0	0	0	0.731	0.934	0.903	0.635	0.999
Ndufb9	Ndufs7	10116.ENSRNO	10116.ENSRNO	0	0	0	0	0.776	0.937	0.903	0.645	0.999
Ndufb9	Ndufs4	10116.ENSRNO	10116.ENSRNO	0	0	0	0	0.882	0.93	0.903	0.595	0.999
Ndufb9	Ndufs1	10116.ENSRNO	10116.ENSRNO	0	0	0	0	0.693	0.942	0.903	0.77	0.999
Ndufb9	Ndufc2	10116.ENSRNO	10116.ENSRNO	0	0	0	0	0.851	0.934	0.951	0.425	0.999
Ndufb9	Ndufv1	10116.ENSRNO	10116.ENSRNO	0	0	0	0	0.817	0.935	0.903	0.677	0.999

Ndufc2	Ndufv3	10116.ENSRNO	10116.ENSRNO	0	0	0	0	0.176	0.805	0.768	0.465	0.977
Ndufc2	Ndufs3	10116.ENSRNO	10116.ENSRNO	0	0	0	0	0.69	0.942	0.903	0.419	0.998
Ndufc2	Ndufs4	10116.ENSRNO	10116.ENSRNO	0	0	0	0	0.714	0.94	0.903	0.498	0.999
Ndufc2	Ndufs1	10116.ENSRNO	10116.ENSRNO	0	0	0	0	0.141	0.942	0.903	0.338	0.996
Ndufc2	Uqcrb	10116.ENSRNO	10116.ENSRNO	0	0	0	0	0.68	0.707	0	0.524	0.951
Ndufc2	Uqcrc1	10116.ENSRNO	10116.ENSRNO	0	0	0	0	0.226	0.882	0	0.545	0.954
Ndufc2	Ndufs5	10116.ENSRNO	10116.ENSRNO	0	0	0	0	0.665	0.821	0	0.376	0.959
Ndufc2	Uqcrc2	10116.ENSRNO	10116.ENSRNO	0	0	0	0	0.589	0.928	0	0.268	0.976
Ndufc2	Uqcrfs1	10116.ENSRNO	10116.ENSRNO	0	0	0	0	0.802	0.928	0	0.431	0.991
Ndufc2	Ndufv2	10116.ENSRNO	10116.ENSRNO	0	0	0	0	0.477	0.942	0.903	0.345	0.997
Ndufc2	Ndufv1	10116.ENSRNO	10116.ENSRNO	0	0	0	0	0.185	0.942	0.903	0.537	0.997
Ndufc2	Ndufs2	10116.ENSRNO	10116.ENSRNO	0	0	0	0	0.565	0.942	0.903	0.603	0.998
Ndufc2	Ndufs7	10116.ENSRNO	10116.ENSRNO	0	0	0	0	0.639	0.942	0.903	0.402	0.998
Ndufc2	Uqcrh	10116.ENSRNO	10116.ENSRNO	0	0	0	0	0.808	0.928	0.783	0.477	0.998
Ndufc2	rCG_38845	10116.ENSRNO	10116.ENSRNO	0	0	0	0	0.694	0.925	0.931	0.393	0.998
Ndufs1	Ndufv3	10116.ENSRNO	10116.ENSRNO	0	0	0	0	0.09	0.931	0.768	0.638	0.994
Ndufs1	Sdhc	10116.ENSRNO	10116.ENSRNO	0.043	0	0	0	0.57	0	0.708	0.608	0.946
Ndufs1	Sdhb	10116.ENSRNO	10116.ENSRNO	0	0	0	0	0.613	0.776	0.708	0.735	0.992
Ndufs1	Ndufs3	10116.ENSRNO	10116.ENSRNO	0.155	0	0.412	0	0.671	0.99	0.903	0.857	0.999
Ndufs1	Ndufs4	10116.ENSRNO	10116.ENSRNO	0	0	0	0	0.709	0.99	0.932	0.832	0.999
Ndufs1	Ndufs5	10116.ENSRNO	10116.ENSRNO	0	0	0	0	0.208	0.853	0	0.402	0.924
Ndufs1	Uqcrb	10116.ENSRNO	10116.ENSRNO	0	0	0	0	0.134	0.806	0.67	0.349	0.959
Ndufs1	Uqcrc1	10116.ENSRNO	10116.ENSRNO	0	0	0	0	0.674	0.93	0	0.697	0.992
Ndufs1	Sdha	10116.ENSRNO	10116.ENSRNO	0.042	0	0	0	0.729	0.671	0.708	0.74	0.992
Ndufs1	rCG_38845	10116.ENSRNO	10116.ENSRNO	0	0	0	0	0.328	0.926	0.862	0.461	0.995
Ndufs1	Uqcrc2	10116.ENSRNO	10116.ENSRNO	0	0	0	0	0.849	0.935	0	0.588	0.995
Ndufs1	Uqcrh	10116.ENSRNO	10116.ENSRNO	0	0	0	0	0.118	0.93	0.898	0.395	0.995
Ndufs1	Uqcrfs1	10116.ENSRNO	10116.ENSRNO	0.101	0	0	0	0.782	0.929	0.783	0.706	0.998
Ndufs1	Ndufv1	10116.ENSRNO	10116.ENSRNO	0.155	0	0.419	0	0.883	0.99	0.903	0.935	0.999
Ndufs1	Ndufs7	10116.ENSRNO	10116.ENSRNO	0.155	0	0.269	0	0.702	0.99	0.903	0.812	0.999
Ndufs1	Ndufs2	10116.ENSRNO	10116.ENSRNO	0.155	0	0.358	0	0.901	0.967	0.932	0.883	0.999
Ndufs1	Ndufv2	10116.ENSRNO	10116.ENSRNO	0.155	0	0.405	0	0.671	0.99	0.932	0.897	0.999
Ndufs2	Ndufv3	10116.ENSRNO	10116.ENSRNO	0	0	0	0	0.146	0.804	0.768	0.473	0.976
Ndufs2	Sdhc	10116.ENSRNO	10116.ENSRNO	0.072	0	0	0	0.68	0.484	0.708	0.755	0.987
Ndufs2	Sdhb	10116.ENSRNO	10116.ENSRNO	0.068	0	0	0	0.769	0.86	0.708	0.686	0.996
Ndufs2	Ndufs3	10116.ENSRNO	10116.ENSRNO	0.155	0	0.445	0	0.832	0.99	0.903	0.925	0.999
Ndufs2	Ndufs4	10116.ENSRNO	10116.ENSRNO	0	0	0	0	0.666	0.966	0.932	0.815	0.999
Ndufs2	Uqcrh	10116.ENSRNO	10116.ENSRNO	0	0	0	0	0.879	0.923	0.898	0.444	0.999
Ndufs2	Ndufv2	10116.ENSRNO	10116.ENSRNO	0.155	0	0.329	0	0.909	0.99	0.932	0.845	0.999
Ndufs2	Sdha	10116.ENSRNO	10116.ENSRNO	0.063	0	0	0	0.778	0.53	0.708	0.73	0.99
Ndufs2	Uqcrc2	10116.ENSRNO	10116.ENSRNO	0.043	0	0	0	0.659	0.93	0	0.64	0.99
Ndufs2	Ndufv1	10116.ENSRNO	10116.ENSRNO	0.155	0	0.334	0	0.874	0.99	0.903	0.922	0.999
Ndufs2	Uqcrfs1	10116.ENSRNO	10116.ENSRNO	0.044	0	0	0	0.885	0.929	0.783	0.743	0.999
Ndufs2	rCG_38845	10116.ENSRNO	10116.ENSRNO	0	0	0	0	0.497	0.926	0.862	0.417	0.996
Ndufs2	Uqcrb	10116.ENSRNO	10116.ENSRNO	0	0	0	0	0.228	0.546	0.67	0.656	0.954
Ndufs2	Ndufs7	10116.ENSRNO	10116.ENSRNO	0.155	0.887	0.446	0	0.79	0.99	0.903	0.915	0.999
Ndufs2	Ndufs5	10116.ENSRNO	10116.ENSRNO	0	0	0	0	0.235	0.853	0	0.622	0.953
Ndufs2	Uqcrc1	10116.ENSRNO	10116.ENSRNO	0.043	0	0	0	0.827	0.93	0	0.699	0.996
Ndufs3	Ndufv3	10116.ENSRNO	10116.ENSRNO	0	0	0	0	0.182	0.987	0.768	0.59	0.998
Ndufs3	Sdhc	10116.ENSRNO	10116.ENSRNO	0.041	0	0	0	0.923	0	0.708	0.526	0.988
Ndufs3	Sdhb	10116.ENSRNO	10116.ENSRNO	0.089	0	0	0	0.871	0.453	0.708	0.751	0.994
Ndufs3	Uqcrb	10116.ENSRNO	10116.ENSRNO	0	0	0	0	0.424	0.635	0.67	0.455	0.957
Ndufs3	Ndufs5	10116.ENSRNO	10116.ENSRNO	0	0	0	0	0.616	0.853	0	0.387	0.962
Ndufs3	Sdha	10116.ENSRNO	10116.ENSRNO	0	0	0	0	0.679	0.527	0.708	0.724	0.986
Ndufs3	Uqcrc2	10116.ENSRNO	10116.ENSRNO	0	0	0	0	0.625	0.937	0	0.67	0.991
Ndufs3	Uqcrc1	10116.ENSRNO	10116.ENSRNO	0	0	0	0	0.845	0.933	0	0.585	0.995
Ndufs3	Uqcrh	10116.ENSRNO	10116.ENSRNO	0	0	0	0	0.36	0.93	0.898	0.337	0.996

Ndufs3	rCG_38845	10116.ENSRNO	10116.ENSRNO	0	0	0	0	0.671	0.928	0.862	0.569	0.998
Ndufs3	Ndufv2	10116.ENSRNO	10116.ENSRNO	0.155	0	0.339	0	0.954	0.99	0.903	0.849	0.999
Ndufs3	Ndufs7	10116.ENSRNO	10116.ENSRNO	0.155	0	0.433	0	0.932	0.99	0.903	0.948	0.999
Ndufs3	Ndufs4	10116.ENSRNO	10116.ENSRNO	0	0	0	0	0.723	0.99	0.903	0.732	0.999
Ndufs3	Uqcrfs1	10116.ENSRNO	10116.ENSRNO	0.044	0	0	0	0.941	0.932	0.783	0.659	0.999
Ndufs3	Ndufv1	10116.ENSRNO	10116.ENSRNO	0.155	0	0.349	0	0.868	0.99	0.903	0.854	0.999
Ndufs4	Ndufv3	10116.ENSRNO	10116.ENSRNO	0	0	0	0	0.1	0.943	0.768	0.687	0.995
Ndufs4	Sdhb	10116.ENSRNO	10116.ENSRNO	0	0	0	0	0.589	0.852	0	0.473	0.965
Ndufs4	Uqcrc1	10116.ENSRNO	10116.ENSRNO	0	0	0	0	0.605	0.856	0	0.534	0.971
Ndufs4	Ndufs5	10116.ENSRNO	10116.ENSRNO	0	0	0	0	0.58	0.852	0	0.656	0.976
Ndufs4	Uqcrb	10116.ENSRNO	10116.ENSRNO	0	0	0	0	0.67	0.808	0.67	0.356	0.984
Ndufs4	Uqcrc2	10116.ENSRNO	10116.ENSRNO	0	0	0	0	0.675	0.929	0	0.551	0.988
Ndufs4	Uqcrfs1	10116.ENSRNO	10116.ENSRNO	0	0	0	0	0.701	0.93	0.783	0.586	0.997
Ndufs4	Uqcrh	10116.ENSRNO	10116.ENSRNO	0	0	0	0	0.425	0.936	0.898	0.493	0.997
Ndufs4	rCG_38845	10116.ENSRNO	10116.ENSRNO	0	0	0	0	0.643	0.928	0.862	0.435	0.997
Ndufs4	Ndufv1	10116.ENSRNO	10116.ENSRNO	0	0	0	0	0.585	0.99	0.903	0.865	0.999
Ndufs4	Ndufs7	10116.ENSRNO	10116.ENSRNO	0	0	0	0	0.651	0.94	0.903	0.805	0.999
Ndufs4	Ndufv2	10116.ENSRNO	10116.ENSRNO	0	0	0	0	0.786	0.966	0.932	0.801	0.999
Ndufs5	Sdhb	10116.ENSRNO	10116.ENSRNO	0	0	0	0	0.683	0.696	0	0.131	0.908
Ndufs5	Uqcrh	10116.ENSRNO	10116.ENSRNO	0	0	0	0	0.916	0.799	0	0.593	0.992
Ndufs5	Ndufv2	10116.ENSRNO	10116.ENSRNO	0	0	0	0	0.733	0.852	0	0.624	0.983
Ndufs5	Ndufv1	10116.ENSRNO	10116.ENSRNO	0	0	0	0	0.342	0.853	0	0.542	0.951
Ndufs5	Uqcrfs1	10116.ENSRNO	10116.ENSRNO	0	0	0	0	0.431	0.852	0	0.518	0.955
Ndufs5	rCG_38845	10116.ENSRNO	10116.ENSRNO	0	0	0	0	0.62	0.758	0	0.36	0.936
Ndufs5	Uqcrb	10116.ENSRNO	10116.ENSRNO	0	0	0	0	0.847	0.553	0	0.479	0.961
Ndufs5	Ndufs7	10116.ENSRNO	10116.ENSRNO	0	0	0	0	0.711	0.853	0	0.465	0.975
Ndufs5	Uqcrc1	10116.ENSRNO	10116.ENSRNO	0	0	0	0	0.154	0.853	0	0.472	0.928
Ndufs7	Ndufv3	10116.ENSRNO	10116.ENSRNO	0	0	0	0	0.359	0.863	0.768	0.491	0.988
Ndufs7	Sdhc	10116.ENSRNO	10116.ENSRNO	0.073	0	0	0	0.799	0	0.708	0.415	0.963
Ndufs7	Sdhb	10116.ENSRNO	10116.ENSRNO	0.084	0	0	0	0.836	0.925	0.708	0.685	0.998
Ndufs7	Uqcrh	10116.ENSRNO	10116.ENSRNO	0	0	0	0	0.473	0.93	0.783	0.556	0.995
Ndufs7	Ndufv2	10116.ENSRNO	10116.ENSRNO	0.155	0	0.299	0	0.733	0.942	0.903	0.857	0.999
Ndufs7	Sdha	10116.ENSRNO	10116.ENSRNO	0.063	0	0	0	0.459	0.438	0.708	0.635	0.964
Ndufs7	Uqcrc2	10116.ENSRNO	10116.ENSRNO	0.081	0	0	0	0.558	0.93	0	0.648	0.988
Ndufs7	Ndufv1	10116.ENSRNO	10116.ENSRNO	0.155	0	0.304	0	0.907	0.956	0.903	0.872	0.999
Ndufs7	Uqcrfs1	10116.ENSRNO	10116.ENSRNO	0.045	0	0	0	0.795	0.931	0.783	0.527	0.998
Ndufs7	rCG_38845	10116.ENSRNO	10116.ENSRNO	0	0	0	0	0.703	0.928	0.862	0.493	0.998
Ndufs7	Uqcrb	10116.ENSRNO	10116.ENSRNO	0	0	0	0	0.599	0.807	0.67	0.428	0.983
Ndufs7	Uqcrc1	10116.ENSRNO	10116.ENSRNO	0.081	0	0	0	0.894	0.93	0	0.598	0.996
Ndufv1	Ndufv3	10116.ENSRNO	10116.ENSRNO	0	0	0	0	0.172	0.987	0.768	0.685	0.999
Ndufv1	Sdhc	10116.ENSRNO	10116.ENSRNO	0.042	0	0	0	0.522	0	0.783	0.67	0.962
Ndufv1	Sdhb	10116.ENSRNO	10116.ENSRNO	0.044	0	0	0	0.847	0.852	0.789	0.752	0.998
Ndufv1	Uqcrh	10116.ENSRNO	10116.ENSRNO	0	0	0	0	0.132	0.93	0.909	0.517	0.996
Ndufv1	Ndufv2	10116.ENSRNO	10116.ENSRNO	0.155	0.525	0.448	0	0.785	0.99	0.903	0.965	0.999
Ndufv1	Sdha	10116.ENSRNO	10116.ENSRNO	0.153	0	0	0	0.795	0.499	0.789	0.755	0.994
Ndufv1	Uqcrc2	10116.ENSRNO	10116.ENSRNO	0	0	0	0	0.574	0.93	0.783	0.657	0.997
Ndufv1	Uqcrb	10116.ENSRNO	10116.ENSRNO	0	0	0	0	0.151	0.808	0.815	0.578	0.985
Ndufv1	rCG_38845	10116.ENSRNO	10116.ENSRNO	0	0	0	0	0.65	0.926	0.862	0.478	0.997
Ndufv1	Uqcrfs1	10116.ENSRNO	10116.ENSRNO	0.043	0	0	0	0.816	0.933	0.783	0.683	0.999
Ndufv1	Uqcrc1	10116.ENSRNO	10116.ENSRNO	0	0	0	0	0.924	0.925	0.67	0.746	0.999
Ndufv2	Ndufv3	10116.ENSRNO	10116.ENSRNO	0	0	0	0	0.17	0.987	0.768	0.715	0.999
Ndufv2	Sdhc	10116.ENSRNO	10116.ENSRNO	0.042	0	0	0	0.825	0	0.708	0.423	0.968
Ndufv2	Sdhb	10116.ENSRNO	10116.ENSRNO	0.067	0	0	0	0.745	0.551	0.708	0.625	0.986
Ndufv2	Uqcrh	10116.ENSRNO	10116.ENSRNO	0	0	0	0	0.855	0.93	0.783	0.408	0.998
Ndufv2	Sdha	10116.ENSRNO	10116.ENSRNO	0.044	0	0	0	0.369	0.464	0.708	0.658	0.961
Ndufv2	Uqcrb	10116.ENSRNO	10116.ENSRNO	0	0	0	0	0.704	0.807	0.67	0.275	0.984
Ndufv2	Uqcrc2	10116.ENSRNO	10116.ENSRNO	0	0	0	0	0.627	0.929	0	0.517	0.986

Ndufv2	Uqcrc1	10116.ENSRNC	10116.ENSRNC	0	0	0	0	0.758	0.935	0	0.588	0.992
Ndufv2	rCG_38845	10116.ENSRNC	10116.ENSRNC	0	0	0	0	0.623	0.926	0.862	0.341	0.997
Ndufv2	Uqcrfs1	10116.ENSRNC	10116.ENSRNC	0.088	0	0	0	0.885	0.929	0.783	0.479	0.999
Ndufv3	Uqcrh	10116.ENSRNC	10116.ENSRNC	0	0	0	0	0.15	0.804	0	0.571	0.922
Ndufv3	rCG_38845	10116.ENSRNC	10116.ENSRNC	0	0	0	0	0.204	0.777	0.67	0.216	0.947
Nnt	Sirt3	10116.ENSRNC	10116.ENSRNC	0.044	0	0	0	0	0.057	0.9	0.246	0.923
P4hb	Ppib	10116.ENSRNC	10116.ENSRNC	0	0	0	0	0.315	0.692	0.973	0.582	0.997
Pacsin2	Pacsin3	10116.ENSRNC	10116.ENSRNC	0	0	0	0.956	0	0.855	0.6	0.766	0.941
Pc	Pdhb	10116.ENSRNC	10116.ENSRNC	0.044	0	0	0	0.085	0	0.9	0.629	0.963
Pdhb	Pdk2	10116.ENSRNC	10116.ENSRNC	0	0	0	0	0.102	0.699	0.9	0.358	0.98
Pdhb	Uqcrc1	10116.ENSRNC	10116.ENSRNC	0	0	0	0	0.67	0.505	0	0.502	0.911
Pdhb	Uqcrc2	10116.ENSRNC	10116.ENSRNC	0	0	0	0	0.808	0.474	0	0.358	0.929
Pdhb	Sdha	10116.ENSRNC	10116.ENSRNC	0.044	0	0	0	0.482	0.453	0	0.773	0.93
Pdhb	Pdk4	10116.ENSRNC	10116.ENSRNC	0	0	0	0	0.086	0.52	0.9	0.29	0.964
Pdia3	Ppib	10116.ENSRNC	10116.ENSRNC	0	0	0	0	0.264	0.54	0.663	0.668	0.957
Pebp1	Rap1a	10116.ENSRNC	10116.ENSRNC	0	0	0	0	0	0	0.9	0.047	0.9
Pebp1	Tln1	10116.ENSRNC	10116.ENSRNC	0	0	0	0	0	0	0.9	0	0.9
Pebp1	Vcl	10116.ENSRNC	10116.ENSRNC	0	0	0	0	0.06	0	0.9	0.074	0.905
Pgm1	Pygm	10116.ENSRNC	10116.ENSRNC	0.103	0	0	0	0.592	0	0.994	0.34	0.998
Pgm1	Ugp2	10116.ENSRNC	10116.ENSRNC	0	0.241	0	0	0.583	0	0.994	0.729	0.999
Phb	Ywhab	10116.ENSRNC	10116.ENSRNC	0	0	0	0	0	0.083	0.9	0.184	0.918
Phb	Phb2	10116.ENSRNC	10116.ENSRNC	0	0	0.42	0.94	0.586	0.674	0.8	0.86	0.972
Phb	Pmpca	10116.ENSRNC	10116.ENSRNC	0.067	0	0	0	0.176	0.217	0.9	0.077	0.934
Phb2	Uqcrfs1	10116.ENSRNC	10116.ENSRNC	0	0	0	0	0.233	0.814	0	0.441	0.913
Phb2	Spg7	10116.ENSRNC	10116.ENSRNC	0	0	0	0	0.313	0.425	0.6	0.57	0.923
Phb2	Uqcrc1	10116.ENSRNC	10116.ENSRNC	0.067	0	0	0	0.625	0.762	0	0.19	0.923
Phb2	Pmpca	10116.ENSRNC	10116.ENSRNC	0.067	0	0	0	0.516	0.217	0.9	0.126	0.963
Plg	S100a10	10116.ENSRNC	10116.ENSRNC	0	0	0	0	0	0.103	0.9	0.962	0.996
Pmpca	Spg7	10116.ENSRNC	10116.ENSRNC	0.043	0	0	0	0.193	0.082	0.9	0.27	0.938
Pmpca	Uqcrc1	10116.ENSRNC	10116.ENSRNC	0	0	0.411	0.704	0.59	0.82	0	0.504	0.941
Ppib	Serpinh1	10116.ENSRNC	10116.ENSRNC	0	0	0	0	0.064	0.056	0.9	0.59	0.958
Prdx2	Prdx5	10116.ENSRNC	10116.ENSRNC	0	0	0	0	0.138	0.526	0.5	0.809	0.955
Prdx3	Prdx5	10116.ENSRNC	10116.ENSRNC	0	0	0	0	0.162	0.526	0.5	0.849	0.966
Prdx3	Txn2	10116.ENSRNC	10116.ENSRNC	0.044	0	0	0	0.169	0.518	0.9	0.872	0.994
Prdx5	Txn2	10116.ENSRNC	10116.ENSRNC	0	0	0	0	0.108	0.235	0.9	0.677	0.975
Psma5	Psmb1	10116.ENSRNC	10116.ENSRNC	0	0	0.274	0	0.92	0.986	0.924	0.856	0.999
Psma5	Psmd2	10116.ENSRNC	10116.ENSRNC	0	0	0	0	0.679	0.985	0.8	0.643	0.999
Psma5	Psmc2	10116.ENSRNC	10116.ENSRNC	0	0	0	0	0.91	0.984	0.8	0.476	0.999
Psma5	Psmd13	10116.ENSRNC	10116.ENSRNC	0	0	0	0	0.665	0.984	0.8	0.669	0.999
Psma5	Psme1	10116.ENSRNC	10116.ENSRNC	0	0	0	0	0.108	0.543	0.8	0.449	0.949
Psma5	Psmd11	10116.ENSRNC	10116.ENSRNC	0	0	0	0	0.728	0.985	0.924	0.696	0.999
Psmb1	Vcp	10116.ENSRNC	10116.ENSRNC	0	0	0	0	0.571	0.853	0	0.075	0.936
Psmb1	Psme1	10116.ENSRNC	10116.ENSRNC	0	0	0	0	0.098	0.526	0.8	0.532	0.954
Psmb1	Psmc2	10116.ENSRNC	10116.ENSRNC	0	0	0	0	0.872	0.977	0.8	0.7	0.999
Psmb1	Psmd13	10116.ENSRNC	10116.ENSRNC	0	0	0	0	0.712	0.984	0.8	0.487	0.999
Psmb1	Psmd2	10116.ENSRNC	10116.ENSRNC	0	0	0	0	0.547	0.985	0.8	0.632	0.999
Psmb1	Psmd11	10116.ENSRNC	10116.ENSRNC	0	0	0	0	0.598	0.984	0.924	0.592	0.999
Psmc2	Psmd2	10116.ENSRNC	10116.ENSRNC	0	0	0	0	0.721	0.985	0.8	0.813	0.999
Psmc2	Psme1	10116.ENSRNC	10116.ENSRNC	0	0	0	0	0.126	0.379	0.8	0.246	0.907
Psmc2	Vcp	10116.ENSRNC	10116.ENSRNC	0	0	0	0.799	0.608	0.853	0	0.514	0.945
Psmc2	Psmd11	10116.ENSRNC	10116.ENSRNC	0	0	0	0	0.657	0.985	0.8	0.714	0.999
Psmc2	Psmd13	10116.ENSRNC	10116.ENSRNC	0	0	0	0	0.833	0.984	0.8	0.78	0.999
Psmd11	Psmd2	10116.ENSRNC	10116.ENSRNC	0	0	0	0	0.8	0.985	0.8	0.765	0.999
Psmd11	Psmd13	10116.ENSRNC	10116.ENSRNC	0	0	0	0	0.721	0.985	0.8	0.836	0.999
Psmd11	Psme1	10116.ENSRNC	10116.ENSRNC	0	0	0	0	0.064	0.435	0.8	0.33	0.919
Psmd11	Vcp	10116.ENSRNC	10116.ENSRNC	0	0	0	0	0.576	0.854	0	0.348	0.956
Psmd13	Psmd2	10116.ENSRNC	10116.ENSRNC	0	0	0	0	0.597	0.984	0.8	0.85	0.999

Psmd13	Psme1	10116.ENSRNO	10116.ENSRNO	0	0	0	0	0.064	0.434	0.8	0.174	0.9
Psmd13	Vcp	10116.ENSRNO	10116.ENSRNO	0	0	0	0	0.161	0.853	0	0.265	0.901
Psmd2	Psme1	10116.ENSRNO	10116.ENSRNO	0	0	0	0	0.061	0.433	0.8	0.382	0.925
Psmd2	Vcp	10116.ENSRNO	10116.ENSRNO	0	0	0	0	0.799	0.853	0	0.476	0.983
Pygm	Ugp2	10116.ENSRNO	10116.ENSRNO	0	0	0	0	0.359	0	0.976	0.42	0.99
Rap1a	Tln1	10116.ENSRNO	10116.ENSRNO	0	0	0	0	0.049	0.507	0.58	0.558	0.901
Rap1a	Ywhaz	10116.ENSRNO	10116.ENSRNO	0	0	0	0	0	0.161	0.911	0.213	0.936
Rap1a	Rhoa	10116.ENSRNO	10116.ENSRNO	0	0	0.416	0.779	0.087	0.193	0.892	0.498	0.929
Rhoa	Vcl	10116.ENSRNO	10116.ENSRNO	0	0	0	0	0.049	0.098	0.584	0.885	0.953
Rhoa	Rock1	10116.ENSRNO	10116.ENSRNO	0	0	0	0	0	0.709	0.919	0.982	0.999
Rpl19	Rplp0	10116.ENSRNO	10116.ENSRNO	0	0	0	0	0.8	0.423	0.8	0.338	0.982
Rpl19	Rpl23	10116.ENSRNO	10116.ENSRNO	0.068	0	0	0	0.522	0.425	0.8	0.103	0.945
Rpl19	Rps5	10116.ENSRNO	10116.ENSRNO	0	0	0	0	0.878	0.538	0	0.403	0.963
Rpl19	Rpl9	10116.ENSRNO	10116.ENSRNO	0.044	0	0.281	0	0.884	0.322	0	0.508	0.968
Rpl19	Rps23	10116.ENSRNO	10116.ENSRNO	0	0	0	0	0.822	0.435	0.72	0.294	0.977
Rpl19	Rpl6	10116.ENSRNO	10116.ENSRNO	0.044	0	0	0	0.886	0.377	0.72	0.442	0.987
Rpl23	Rplp0	10116.ENSRNO	10116.ENSRNO	0.083	0	0	0	0.618	0.521	0.823	0.139	0.969
Rpl23	Rpl6	10116.ENSRNO	10116.ENSRNO	0.069	0	0	0	0.564	0.5	0.6	0.093	0.913
Rpl23	Rpl3l	10116.ENSRNO	10116.ENSRNO	0.085	0	0	0	0.542	0.516	0.65	0.2	0.932
Rpl23	Rps5	10116.ENSRNO	10116.ENSRNO	0.095	0	0	0	0.691	0.794	0	0.276	0.952
Rpl3l	Rplp0	10116.ENSRNO	10116.ENSRNO	0.083	0	0	0	0.541	0.513	0.647	0.182	0.93
Rpl3l	Rpl6	10116.ENSRNO	10116.ENSRNO	0.069	0	0	0	0.524	0.482	0.6	0.225	0.915
Rpl3l	Rps5	10116.ENSRNO	10116.ENSRNO	0.095	0	0	0	0.624	0.724	0	0.317	0.927
Rpl6	Rplp0	10116.ENSRNO	10116.ENSRNO	0	0	0	0	0.924	0.487	0.6	0.436	0.99
Rpl6	Rps23	10116.ENSRNO	10116.ENSRNO	0.06	0	0	0	0.838	0.517	0.72	0.351	0.984
Rpl6	Rps5	10116.ENSRNO	10116.ENSRNO	0.082	0	0	0	0.773	0.696	0	0.587	0.97
Rpl6	Rpl9	10116.ENSRNO	10116.ENSRNO	0.045	0	0	0	0.892	0.38	0	0.432	0.959
Rpl9	Rps23	10116.ENSRNO	10116.ENSRNO	0.07	0	0	0	0.915	0.44	0	0.32	0.965
Rpl9	Rps5	10116.ENSRNO	10116.ENSRNO	0.083	0	0	0	0.818	0.541	0	0.616	0.966
Rplp0	Rps23	10116.ENSRNO	10116.ENSRNO	0.083	0	0	0	0.745	0.527	0	0.315	0.914
Rplp0	Rps5	10116.ENSRNO	10116.ENSRNO	0.09	0	0	0	0.931	0.771	0	0.435	0.99
Rpn1	Stt3a	10116.ENSRNO	10116.ENSRNO	0	0	0	0	0.623	0.811	0.877	0.911	0.999
Rpn1	Rpn2	10116.ENSRNO	10116.ENSRNO	0	0	0	0	0.838	0.932	0.924	0.937	0.999
Rpn2	Stt3a	10116.ENSRNO	10116.ENSRNO	0	0	0	0	0.719	0.809	0.875	0.842	0.998
Rps2	Rps23	10116.ENSRNO	10116.ENSRNO	0.068	0	0	0	0.434	0.448	0.91	0.493	0.984
Rps2	Rps5	10116.ENSRNO	10116.ENSRNO	0.083	0	0	0	0.581	0.545	0.911	0.58	0.992
Rps23	Tpt1	10116.ENSRNO	10116.ENSRNO	0	0	0	0	0.825	0.508	0	0.065	0.912
Rps23	Rps5	10116.ENSRNO	10116.ENSRNO	0.095	0	0	0	0.855	0.809	0.914	0.538	0.998
Samm50	Tomm70a	10116.ENSRNO	10116.ENSRNO	0	0	0	0	0.097	0.115	0.72	0.707	0.925
Samm50	Tomm40l	10116.ENSRNO	10116.ENSRNO	0	0	0	0	0.087	0.546	0.72	0.505	0.934
Samm50	Tomm40	10116.ENSRNO	10116.ENSRNO	0	0	0	0	0.109	0.546	0.72	0.851	0.981
Samm50	Tomm22	10116.ENSRNO	10116.ENSRNO	0	0	0	0	0.081	0.801	0.72	0.791	0.987
Sdha	Sdhc	10116.ENSRNO	10116.ENSRNO	0.155	0	0	0	0.783	0.82	0.979	0.943	0.999
Sdha	Sdhb	10116.ENSRNO	10116.ENSRNO	0.155	0	0.443	0	0.88	0.971	0.98	0.992	0.999
Sdha	Uqcrh	10116.ENSRNO	10116.ENSRNO	0	0	0	0	0.21	0	0.893	0.59	0.962
Sdha	Tufm	10116.ENSRNO	10116.ENSRNO	0.082	0	0	0	0.649	0	0.67	0.431	0.931
Sdha	Uqcrb	10116.ENSRNO	10116.ENSRNO	0	0	0	0	0.175	0.288	0.802	0.519	0.936
Sdha	Uqcrc1	10116.ENSRNO	10116.ENSRNO	0	0	0	0	0.734	0.453	0.802	0.793	0.993
Sdha	Uqcrfs1	10116.ENSRNO	10116.ENSRNO	0.132	0	0	0	0.793	0.508	0.708	0.803	0.994
Sdha	Uqcrc2	10116.ENSRNO	10116.ENSRNO	0	0	0	0	0.736	0.447	0.887	0.884	0.997
Sdha	Suclg2	10116.ENSRNO	10116.ENSRNO	0.12	0	0	0	0.589	0.228	0.982	0.644	0.997
Sdhb	Sdhc	10116.ENSRNO	10116.ENSRNO	0.155	0	0	0	0.88	0.852	0.979	0.99	0.999
Sdhb	Uqcrb	10116.ENSRNO	10116.ENSRNO	0	0	0	0	0.582	0.689	0.81	0.521	0.986
Sdhb	Uqcrh	10116.ENSRNO	10116.ENSRNO	0	0	0	0	0.579	0.444	0.9	0.597	0.989
Sdhb	Uqcrc1	10116.ENSRNO	10116.ENSRNO	0.053	0	0	0	0.638	0.47	0.81	0.778	0.99
Sdhb	Suclg2	10116.ENSRNO	10116.ENSRNO	0.12	0	0	0	0.385	0.193	0.982	0.644	0.996
Sdhb	Uqcrfs1	10116.ENSRNO	10116.ENSRNO	0.102	0	0	0	0.888	0.473	0.789	0.799	0.997

Sdhb	Uqcrc2	10116.ENSRNO	10116.ENSRNO	0.053	0	0	0	0.731	0.857	0.894	0.925	0.999
Sdhc	Uqcrb	10116.ENSRNO	10116.ENSRNO	0	0	0	0	0.613	0	0.81	0.525	0.962
Sdhc	Uqcrh	10116.ENSRNO	10116.ENSRNO	0	0	0	0	0.45	0.054	0.894	0.463	0.966
Sdhc	Uqcrc1	10116.ENSRNO	10116.ENSRNO	0.055	0	0	0	0.628	0	0.81	0.648	0.973
Sdhc	Uqcrc2	10116.ENSRNO	10116.ENSRNO	0.055	0	0	0	0.745	0	0.894	0.594	0.988
Sdhc	Uqcrfs1	10116.ENSRNO	10116.ENSRNO	0.066	0	0	0	0.856	0.042	0.783	0.748	0.991
Sdhc	Suclg2	10116.ENSRNO	10116.ENSRNO	0.12	0	0	0	0.234	0	0.982	0.688	0.995
Sec22b	Vamp7	10116.ENSRNO	10116.ENSRNO	0	0	0	0.662	0.064	0.191	0.9	0.806	0.939
11-Sep	7-Sep	10116.ENSRNO	10116.ENSRNO	0	0	0	0.894	0.563	0.859	0.72	0.956	0.983
11-Sep	2-Sep	10116.ENSRNO	10116.ENSRNO	0	0	0	0.882	0.428	0.941	0.72	0.836	0.99
2-Sep	7-Sep	10116.ENSRNO	10116.ENSRNO	0	0	0	0.948	0.682	0.931	0.72	0.963	0.993
Sgca	Sgcb	10116.ENSRNO	10116.ENSRNO	0	0	0	0	0.16	0.509	0	0.848	0.932
Sgca	Sgcg	10116.ENSRNO	10116.ENSRNO	0	0	0	0	0.268	0.139	0	0.886	0.922
Sgcb	Sgcg	10116.ENSRNO	10116.ENSRNO	0	0	0	0	0.094	0.509	0	0.822	0.913
Sirt3	Sod2	10116.ENSRNO	10116.ENSRNO	0	0	0	0	0.056	0.058	0.9	0.801	0.979
Slc25a3	Uqcrfs1	10116.ENSRNO	10116.ENSRNO	0	0	0	0	0.928	0	0	0.044	0.928
Slc25a4	Vdac1	10116.ENSRNO	10116.ENSRNO	0	0	0	0	0.182	0.133	0.951	0.516	0.981
Slc25a4	Slc25a5	10116.ENSRNO	10116.ENSRNO	0	0	0	0.984	0.058	0.622	0.8	0.717	0.923
Slc25a4	Vdac2	10116.ENSRNO	10116.ENSRNO	0	0	0	0	0.183	0.133	0.931	0.508	0.972
Slc25a4	Vdac3	10116.ENSRNO	10116.ENSRNO	0	0	0	0	0.211	0.146	0.951	0.624	0.986
Slc25a5	Vdac1	10116.ENSRNO	10116.ENSRNO	0	0	0	0	0.119	0.133	0.874	0.648	0.961
Slc25a5	Vdac2	10116.ENSRNO	10116.ENSRNO	0	0	0	0	0.119	0.133	0.847	0.6	0.947
Slc25a5	Vdac3	10116.ENSRNO	10116.ENSRNO	0	0	0	0	0.204	0.133	0.874	0.536	0.954
Tagln2	Wdr1	10116.ENSRNO	10116.ENSRNO	0	0	0	0	0.125	0	0.9	0.243	0.928
Tagln2	Vcl	10116.ENSRNO	10116.ENSRNO	0	0	0	0	0.132	0	0.9	0.28	0.932
Tagln2	Tln1	10116.ENSRNO	10116.ENSRNO	0	0	0	0	0.116	0	0.9	0.301	0.932
Tln1	Vcl	10116.ENSRNO	10116.ENSRNO	0	0	0	0	0.127	0.934	0.924	0.936	0.999
Tln1	Wdr1	10116.ENSRNO	10116.ENSRNO	0	0	0	0	0.176	0	0.9	0.398	0.946
Tomm22	Tomm70a	10116.ENSRNO	10116.ENSRNO	0	0	0	0	0.098	0.809	0.9	0.983	0.999
Tomm22	Tomm40l	10116.ENSRNO	10116.ENSRNO	0	0	0	0	0.385	0.809	0.9	0.549	0.994
Tomm22	Tomm40	10116.ENSRNO	10116.ENSRNO	0	0	0	0	0.385	0.809	0.8	0.989	0.999
Tomm40	Tomm70a	10116.ENSRNO	10116.ENSRNO	0	0	0	0	0.136	0.703	0.8	0.96	0.997
Tomm40	Vdac1	10116.ENSRNO	10116.ENSRNO	0	0	0	0	0.113	0.485	0.54	0.701	0.928
Tomm40	Vdac2	10116.ENSRNO	10116.ENSRNO	0	0	0	0	0.113	0.485	0.54	0.693	0.926
Tomm40l	Tomm70a	10116.ENSRNO	10116.ENSRNO	0	0	0	0	0.136	0.703	0.9	0.584	0.987
Tomm70a	Vdac3	10116.ENSRNO	10116.ENSRNO	0	0	0	0	0.185	0.307	0.9	0.462	0.965
Tomm70a	Vdac2	10116.ENSRNO	10116.ENSRNO	0	0	0	0	0.07	0.307	0.9	0.57	0.968
Tomm70a	Vdac1	10116.ENSRNO	10116.ENSRNO	0	0	0	0	0.075	0.307	0.9	0.698	0.978
Tpm1	Tpm4	10116.ENSRNO	10116.ENSRNO	0	0	0.376	0.962	0.064	0.512	0.9	0.753	0.952
Tubb5	Ywhaz	10116.ENSRNO	10116.ENSRNO	0	0	0	0	0.119	0.984	0	0.355	0.99
Uqcrb	Uqcrh	10116.ENSRNO	10116.ENSRNO	0	0	0	0	0.709	0.821	0.961	0.777	0.999
Uqcrb	Uqcrc2	10116.ENSRNO	10116.ENSRNO	0	0	0	0	0.651	0.821	0.961	0.578	0.998
Uqcrb	Uqcrfs1	10116.ENSRNO	10116.ENSRNO	0	0	0	0	0.7	0.821	0.952	0.738	0.999
Uqcrb	Uqcrc1	10116.ENSRNO	10116.ENSRNO	0	0	0	0	0.518	0.73	0.941	0.66	0.997
Uqcrc1	Uqcrh	10116.ENSRNO	10116.ENSRNO	0	0	0	0	0.371	0.943	0.98	0.792	0.999
Uqcrc1	Uqcrc2	10116.ENSRNO	10116.ENSRNO	0	0	0.428	0.626	0.799	0.987	0.98	0.854	0.999
Uqcrc1	Uqcrfs1	10116.ENSRNO	10116.ENSRNO	0	0	0	0	0.8	0.973	0.976	0.809	0.999
Uqcrc1	rCG_38845	10116.ENSRNO	10116.ENSRNO	0	0	0	0	0.534	0.7	0	0.407	0.91
Uqcrc2	Uqcrh	10116.ENSRNO	10116.ENSRNO	0	0	0	0	0.674	0.943	0.989	0.784	0.999
Uqcrc2	Uqcrfs1	10116.ENSRNO	10116.ENSRNO	0	0	0	0	0.891	0.938	0.985	0.865	0.999
Uqcrfs1	Uqcrh	10116.ENSRNO	10116.ENSRNO	0	0	0	0	0.801	0.937	0.986	0.849	0.999
Uqcrfs1	rCG_38845	10116.ENSRNO	10116.ENSRNO	0	0	0	0	0.47	0.925	0	0.3	0.969
Vcl	Wdr1	10116.ENSRNO	10116.ENSRNO	0	0	0	0	0.117	0	0.9	0.347	0.937
Vdac1	Vdac2	10116.ENSRNO	10116.ENSRNO	0	0	0	0.978	0.103	0	0.965	0.883	0.968
Vdac1	Vdac3	10116.ENSRNO	10116.ENSRNO	0	0	0	0.97	0.119	0	0.975	0.8	0.978
Vdac2	Vdac3	10116.ENSRNO	10116.ENSRNO	0	0	0	0.974	0.169	0.084	0.965	0.912	0.972
Ywhab	Ywhae	10116.ENSRNO	10116.ENSRNO	0	0	0	0.964	0.218	0.859	0.845	0.815	0.981

Ywhab	Ywhaq	10116.ENSRNO	10116.ENSRNO	0	0	0	0.979	0.166	0.451	0.8	0.737	0.902
Ywhab	Ywhah	10116.ENSRNO	10116.ENSRNO	0	0	0	0.972	0.118	0.859	0.845	0.795	0.979
Ywhab	Ywhaz	10116.ENSRNO	10116.ENSRNO	0	0	0	0.981	0.475	0.859	0.922	0.766	0.993
Ywhae	Ywhah	10116.ENSRNO	10116.ENSRNO	0	0	0	0.961	0.095	0.855	0.845	0.761	0.978
Ywhae	Ywhaz	10116.ENSRNO	10116.ENSRNO	0	0	0	0.967	0.352	0.924	0.845	0.919	0.991
Ywhah	Ywhaz	10116.ENSRNO	10116.ENSRNO	0	0	0	0.973	0.424	0.859	0.845	0.677	0.986
Ywhaq	Ywhaz	10116.ENSRNO	10116.ENSRNO	0	0	0	0.979	0.627	0.434	0.8	0.676	0.954

Supplementary Table 8b. STRING K-means clusters						
#Clustering Method	Cluster Number	Cluster Color	Gene Count	Protein Name	Protein Identifier	Protein Description
kmeans	1	Yellow	128	Adck3	10116.ENSRNOP00000036948	Atypical kinase COQ8A, mitochondrial; Atypical kinase involved in the biosynthesis of coenzyme Q, also named ubiquinone, an essential lipid-soluble electron transporter for aerobic cellular respiration. Its substrate specificity is unclear: does not show any protein kinase activity. Probably acts as a small molecule kinase, possibly a lipid kinase that phosphorylates a prenyl lipid in the ubiquinone biosynthesis pathway, as suggested by its ability to bind coenzyme Q lipid intermediates. Shows an unusual selectivity for binding ADP over ATP
kmeans	1	Yellow	128	Aifm1	10116.ENSRNOP00000008503	Apoptosis-inducing factor 1, mitochondrial; Functions both as NADH oxidoreductase and as regulator of apoptosis. In response to apoptotic stimuli, it is released from the mitochondrion intermembrane space into the cytosol and to the nucleus, where it functions as a proapoptotic factor in a caspase-independent pathway. In contrast, functions as an antiapoptotic factor in normal mitochondria via its NADH oxidoreductase activity. The soluble form (AIFsol) found in the nucleus induces 'parthanatos' i.e. caspase-independent fragmentation of chromosomal DNA. Interacts with EIF3G,and thereby [...]
kmeans	1	Yellow	128	Apoo	10116.ENSRNOP00000065539	MICOS complex subunit; Component of the MICOS complex, a large protein complex of the mitochondrial inner membrane that plays crucial roles in the maintenance of crista junctions, inner membrane architecture, and formation of contact sites to the outer membrane
kmeans	1	Yellow	128	Atp1a1	10116.ENSRNOP00000045650	Sodium/potassium-transporting ATPase subunit alpha-1; This is the catalytic component of the active enzyme, which catalyzes the hydrolysis of ATP coupled with the exchange of sodium and potassium ions across the plasma membrane. This action creates the electrochemical gradient of sodium and potassium ions, providing the energy for active transport of various nutrients
kmeans	1	Yellow	128	Atp1a2	10116.ENSRNOP00000054947	Sodium/potassium-transporting ATPase subunit alpha-2; This is the catalytic component of the active enzyme, which catalyzes the hydrolysis of ATP coupled with the exchange of sodium and potassium ions across the plasma membrane. This action creates the electrochemical gradient of sodium and potassium ions, providing the energy for active transport of various nutrients; Belongs to the cation transport ATPase (P-type) (TC 3.A.3) family. Type IIC subfamily
kmeans	1	Yellow	128	Atp1b1	10116.ENSRNOP00000003932	Sodium/potassium-transporting ATPase subunit beta-1; This is the non-catalytic component of the active enzyme, which catalyzes the hydrolysis of ATP coupled with the exchange of Na(+) and K(+) ions across the plasma membrane. The beta subunit regulates, through assembly of alpha/beta heterodimers, the number of sodium pumps transported to the plasma membrane
kmeans	1	Yellow	128	Atp5c1	10116.ENSRNOP00000049879	ATP synthase subunit gamma, mitochondrial; Mitochondrial membrane ATP synthase (F(1)F(0) ATP synthase or Complex V) produces ATP from ADP in the presence of a proton gradient across the membrane which is generated by electron transport complexes of the respiratory chain. F-type ATPases consist of two structural domains, F(1) - containing the extramembraneous catalytic core, and F(0) - containing the membrane proton channel, linked together by a central stalk and a peripheral stalk. During catalysis, ATP synthesis in the catalytic domain of F(1) is coupled via a rotary mechanism of the [...]
kmeans	1	Yellow	128	Atp5f1	10116.ENSRNOP00000021920	ATP synthase subunit b, mitochondrial-like
kmeans	1	Yellow	128	Atp5i	10116.ENSRNOP00000000072	ATP synthase subunit e, mitochondrial; Mitochondrial membrane ATP synthase (F(1)F(0) ATP synthase or Complex V) produces ATP from ADP in the presence of a proton gradient across the membrane which is generated by electron transport complexes of the respiratory chain. F-type ATPases consist of two structural domains, F(1) - containing the extramembraneous catalytic core, and F(0) - containing the membrane proton channel, linked together by a central stalk and a peripheral stalk. During catalysis, ATP synthesis in the catalytic domain of F(1) is coupled via a rotary mechanism of the cent [...]
kmeans	1	Yellow	128	Atp5j	10116.ENSRNOP00000002116	ATP synthase-coupling factor 6, mitochondrial; Mitochondrial membrane ATP synthase (F(1)F(0) ATP synthase or Complex V) produces ATP from ADP in the presence of a proton gradient across the membrane which is generated by electron transport complexes of the respiratory chain. F-type ATPases consist of two structural domains, F(1) - containing the extramembraneous catalytic core and F(0) - containing the membrane proton channel, linked together by a central stalk and a peripheral stalk. During catalysis, ATP synthesis in the catalytic domain of F(1) is coupled via a rotary mechanism of t [...]
kmeans	1	Yellow	128	Atp5j2	10116.ENSRNOP00000029426	ATP synthase subunit f, mitochondrial; Mitochondrial membrane ATP synthase (F(1)F(0) ATP synthase or Complex V) produces ATP from ADP in the presence of a proton gradient across the membrane which is generated by electron transport complexes of the respiratory chain. F-type ATPases consist of two structural domains, F(1) - containing the extramembraneous catalytic core and F(0) - containing the membrane proton channel, linked together by a central stalk and a peripheral stalk. During catalysis, ATP synthesis in the catalytic domain of F(1) is coupled via a rotary mechanism of the centr [...]
kmeans	1	Yellow	128	Atp5l	10116.ENSRNOP00000040969	ATP synthase subunit g, mitochondrial; Mitochondrial membrane ATP synthase (F(1)F(0) ATP synthase or Complex V) produces ATP from ADP in the presence of a proton gradient across the membrane which is generated by electron transport complexes of the respiratory chain. F-type ATPases consist of two structural domains, F(1) - containing the extramembraneous catalytic core, and F(0) - containing the membrane proton channel, linked together by a central stalk and a peripheral stalk. During catalysis, ATP synthesis in the catalytic domain of F(1) is coupled via a rotary mechanism of the cent [...]

kmeans	1	Yellow	128	Atp5o	10116.ENSRNOP00000002732	ATP synthase subunit O, mitochondrial; Mitochondrial membrane ATP synthase (F(1)F(0) ATP synthase or Complex V) produces ATP from ADP in the presence of a proton gradient across the membrane which is generated by electron transport complexes of the respiratory chain. F-type ATPases consist of two structural domains, F(1) - containing the extramembraneous catalytic core and F(0) - containing the membrane proton channel, linked together by a central stalk and a peripheral stalk. During catalysis, ATP synthesis in the catalytic domain of F(1) is coupled via a rotary mechanism of the centr [...]
kmeans	1	Yellow	128	Atp5pd	10116.ENSRNOP00000004836	Mitochondrial membrane ATP synthase (F(1)F(0) ATP synthase or Complex V) produces ATP from ADP in the presence of a proton gradient across the membrane which is generated by electron transport complexes of the respiratory chain. F-type ATPases consist of two structural domains, F(1) - containing the extramembraneous catalytic core, and F(0) - containing the membrane proton channel, linked together by a central stalk and a peripheral stalk. During catalysis, ATP synthesis in the catalytic domain of F(1) is coupled via a rotary mechanism of the central stalk subunits to proton translocat [...]
kmeans	1	Yellow	128	C1qbp	10116.ENSRNOP00000031020	Complement component 1 Q subcomponent-binding protein, mitochondrial; Is believed to be a multifunctional and multicompartmental protein involved in inflammation and infection processes, ribosome biogenesis, regulation of apoptosis, transcriptional regulation and pre-mRNA splicing. At the cell surface is thought to act as an endothelial receptor for plasma proteins of the complement and kallikrein-kinin cascades. Putative receptor for C1q; specifically binds to the globular "heads" of C1q thus inhibiting C1; may perform the receptor function through a complex with C1qR/CD93. In complex [...]
kmeans	1	Yellow	128	Cacna2d1	10116.ENSRNOP00000034572	The alpha-2/delta subunit of voltage-dependent calcium channels regulates calcium current density and activation/inactivation kinetics of the calcium channel. Plays an important role in excitation- contraction coupling (By similarity)
kmeans	1	Yellow	128	Calu	10116.ENSRNOP00000008356	RCG28015, isoform CRA_b; Calumenin
kmeans	1	Yellow	128	Cap1	10116.ENSRNOP00000018711	Adenylyl cyclase-associated protein 1; Directly regulates filament dynamics and has been implicated in a number of complex developmental and morphological processes, including mRNA localization and the establishment of cell polarity; Belongs to the CAP family
kmeans	1	Yellow	128	Cars2	10116.ENSRNOP00000019993	cysteinyl-tRNA synthetase 2, mitochondrial
kmeans	1	Yellow	128	Ckap4	10116.ENSRNOP00000010601	Cytoskeleton-associated protein 4
kmeans	1	Yellow	128	Ckb	10116.ENSRNOP00000015122	Creatine kinase B-type; Reversibly catalyzes the transfer of phosphate between ATP and various phosphogens (e.g. creatine phosphate). Creatine kinase isoenzymes play a central role in energy transduction in tissues with large, fluctuating energy demands, such as skeletal muscle, heart, brain and spermatozoa
kmeans	1	Yellow	128	Ckm	10116.ENSRNOP00000022895	Creatine kinase M-type; Reversibly catalyzes the transfer of phosphate between ATP and various phosphogens (e.g. creatine phosphate). Creatine kinase isoenzymes play a central role in energy transduction in tissues with large, fluctuating energy demands, such as skeletal muscle, heart, brain and spermatozoa; Belongs to the ATP:guanido phosphotransferase family
kmeans	1	Yellow	128	Clpb	10116.ENSRNOP00000026665	May function as a regulatory ATPase and be related to secretion/protein trafficking process
kmeans	1	Yellow	128	Clybl	10116.ENSRNOP00000018918	Citramalyl-CoA lyase, mitochondrial; Mitochondrial citramalyl-CoA lyase indirectly involved in the vitamin B12 metabolism. Converts citramalyl-CoA into acetyl-CoA and pyruvate in the C5-dicarboxylate catabolism pathway. The C5-dicarboxylate catabolism pathway is required to detoxify itaconate, a vitamin B12-poisoning metabolite. Also acts as a malate synthase in vitro, converting glyoxylate and acetyl- CoA to malate. Also acts as a beta-methylmalate synthase in vitro, by mediating conversion of glyoxylate and propionyl-CoA to beta- methylmalate. Also has very weak citramalate synthase [...]
kmeans	1	Yellow	128	Col14a1	10116.ENSRNOP00000063778	Collagen, type XIV, alpha 1
kmeans	1	Yellow	128	Coq3	10116.ENSRNOP00000013384	Ubiquinone biosynthesis O-methyltransferase, mitochondrial; O-methyltransferase that catalyzes the 2 O-methylation steps in the ubiquinone biosynthetic pathway; Belongs to the class I-like SAM-binding methyltransferase superfamily. UbiG/COQ3 family
kmeans	1	Yellow	128	Coq5	10116.ENSRNOP00000001547	2-methoxy-6-polyprenyl-1,4-benzoquinol methylase, mitochondrial; Methyltransferase required for the conversion of 2- polyprenyl-6-methoxy-1,4-benzoquinol (DDMQH2) to 2-polyprenyl-3- methyl-6-methoxy-1,4-benzoquinol (DMQH2)

kmeans	1	Yellow	128	Coq6	10116.ENSRNOP00000014914	Ubiquinone biosynthesis monooxygenase COQ6, mitochondrial; FAD-dependent monooxygenase required for the C5-ring hydroxylation during ubiquinone biosynthesis. Catalyzes the hydroxylation of 3-polyprenyl-4-hydroxybenzoic acid to 3- polyprenyl-4,5-dihydroxybenzoic acid. The electrons required for the hydroxylation reaction may be funneled indirectly from NADPH via a ferredoxin/ferredoxin reductase system to COQ6; Belongs to the UbiH/COQ6 family
kmeans	1	Yellow	128	Coq9	10116.ENSRNOP00000021716	Ubiquinone biosynthesis protein COQ9, mitochondrial; Lipid-binding protein involved in the biosynthesis of coenzyme Q, also named ubiquinone, an essential lipid-soluble electron transporter for aerobic cellular respiration. Binds a phospholipid of at least 10 carbons in each acyl group. May be required to present its bound-lipid to COQ7
kmeans	1	Yellow	128	Cox4i1	10116.ENSRNOP00000024033	Cytochrome c oxidase subunit 4 isoform 1, mitochondrial; This protein is one of the nuclear-coded polypeptide chains of cytochrome c oxidase, the terminal oxidase in mitochondrial electron transport
kmeans	1	Yellow	128	Cox5a	10116.ENSRNOP00000025525	Cytochrome c oxidase subunit 5A, mitochondrial; This is the heme A-containing chain of cytochrome c oxidase, the terminal oxidase in mitochondrial electron transport
kmeans	1	Yellow	128	Cox5b	10116.ENSRNOP00000022487	Cytochrome c oxidase subunit 5B, mitochondrial; This protein is one of the nuclear-coded polypeptide chains of cytochrome c oxidase, the terminal oxidase in mitochondrial electron transport
kmeans	1	Yellow	128	Cox6b1	10116.ENSRNOP00000030997	Cytochrome c oxidase subunit VIb polypeptide 1
kmeans	1	Yellow	128	Csrp3	10116.ENSRNOP00000019310	Positive regulator of myogenesis. Acts as cofactor for myogenic bHLH transcription factors such as MYOD1, and probably MYOG and MYF6. Enhances the DNA-binding activity of the MYOD1:TCF3 isoform E47 complex and may promote formation of a functional MYOD1:TCF3 isoform E47:MEF2A complex involved in myogenesis (PubMed:7954791, PubMed:9234731). Plays a crucial and specific role in the organization of cytosolic structures in cardiomyocytes. Could play a role in mechanical stretch sensing. May be a scaffold protein that promotes the assembly of interacting proteins at Z-line structures. It is [...]
kmeans	1	Yellow	128	Cyc1	10116.ENSRNOP00000017067	Cytochrome c-1 (Predicted), isoform CRA_c; Cytochrome c-1
kmeans	1	Yellow	128	Ddx1	10116.ENSRNOP00000009100	ATP-dependent RNA helicase DDX1; Acts as an ATP-dependent RNA helicase, able to unwind both RNA-RNA and RNA-DNA duplexes. Possesses 5' single-stranded RNA overhang nuclease activity. Possesses ATPase activity on various RNA, but not DNA polynucleotides. May play a role in RNA clearance at DNA double-strand breaks (DSBs), thereby facilitating the template-guided repair of transcriptionally active regions of the genome. Together with RELA, acts as a coactivator to enhance NF-kappa-B-mediated transcriptional activation. Acts as a positive transcriptional regulator of cyclin CCND2 expressi [...]
kmeans	1	Yellow	128	Dnajc11	10116.ENSRNOP00000011960	DnaJ (Hsp40) homolog, subfamily C, member 11
kmeans	1	Yellow	128	Dnpep	10116.ENSRNOP00000026799	Aspartyl aminopeptidase, isoform CRA_c; Aspartyl aminopeptidase; Belongs to the peptidase M18 family
kmeans	1	Yellow	128	Ecsit	10116.ENSRNOP00000019115	Evolutionarily conserved signaling intermediate in Toll pathway, mitochondrial; Adapter protein of the Toll-like and IL-1 receptor signaling pathway that is involved in the activation of NF-kappa-B via MAP3K1. Promotes proteolytic activation of MAP3K1. Involved in the BMP signaling pathway. Required for normal embryonic development; Belongs to the ECSIT family
kmeans	1	Yellow	128	Eef1d	10116.ENSRNOP00000034828	Elongation factor 1-delta; Isoform 1: EF-1-beta and EF-1-delta stimulate the exchange of GDP bound to EF-1-alpha to GTP, regenerating EF-1- alpha for another round of transfer of aminoacyl-tRNAs to the ribosome
kmeans	1	Yellow	128	Eef2	10116.ENSRNOP00000041821	Elongation factor 2; Catalyzes the GTP-dependent ribosomal translocation step during translation elongation. During this step, the ribosome changes from the pre-translocational (PRE) to the post- translocational (POST) state as the newly formed A-site-bound peptidyl-tRNA and P-site-bound deacylated tRNA move to the P and E sites, respectively. Catalyzes the coordinated movement of the two tRNA molecules, the mRNA and conformational changes in the ribosome; Belongs to the TRAFAC class translation factor GTPase superfamily. Classic translation factor GTPase family. EF-G/EF-2 subfamily
kmeans	1	Yellow	128	Ehd4	10116.ENSRNOP00000010701	EH-domain containing 4; Belongs to the TRAFAC class dynamin-like GTPase superfamily. Dynamin/Fzo/YdjA family

kmeans	1	Yellow	128	Eif4a1	10116.ENSRNOP00000049419	Eukaryotic translation initiation factor 4A1; Belongs to the DEAD box helicase family
kmeans	1	Yellow	128	Erlin2	10116.ENSRNOP00000018973	Erlin-2; Component of the ERLIN1/ERLIN2 complex which mediates the endoplasmic reticulum-associated degradation (ERAD) of inositol 1,4,5-trisphosphate receptors (IP3Rs) such as ITPR1. Promotes sterol-accelerated ERAD of HMGCR probably implicating an AMFR/gp78-containing ubiquitin ligase complex. Involved in regulation of cellular cholesterol homeostasis by regulation the SREBP signaling pathway. May promote ER retention of the SCAP- SREBF complex (By similarity)
kmeans	1	Yellow	128	Fkbp11	10116.ENSRNOP00000019527	Peptidylprolyl isomerase; FK506 binding protein 11
kmeans	1	Yellow	128	Gfm1	10116.ENSRNOP00000040081	Elongation factor G, mitochondrial; Mitochondrial GTPase that catalyzes the GTP-dependent ribosomal translocation step during translation elongation. During this step, the ribosome changes from the pre-translocational (PRE) to the post-translocational (POST) state as the newly formed A- site-bound peptidyl-tRNA and P-site-bound deacylated tRNA move to the P and E sites, respectively. Catalyzes the coordinated movement of the two tRNA molecules, the mRNA and conformational changes in the ribosome. Does not mediate the disassembly of ribosomes from messenger RNA at the termination of mit [...]
kmeans	1	Yellow	128	Gnb2l1	10116.ENSRNOP00000003334	Receptor of activated protein C kinase 1; Scaffolding protein involved in the recruitment, assembly and/or regulation of a variety of signaling molecules. Interacts with a wide variety of proteins and plays a role in many cellular processes. Component of the 40S ribosomal subunit involved in translational repression. Involved in the initiation of the ribosome quality control (RQC), a pathway that takes place when a ribosome has stalled during translation, by promoting ubiquitination of a subset of 40S ribosomal subunits (By similarity). Binds to and stabilizes activated protein kinase [...]
kmeans	1	Yellow	128	Hhatl	10116.ENSRNOP00000026261	Gup1, glycerol uptake/transporter homolog (Yeast) (Predicted); Hedgehog acyltransferase-like; Belongs to the membrane-bound acyltransferase family
kmeans	1	Yellow	128	Ict1	10116.ENSRNOP00000043868	Mitochondrial ribosomal protein L58; Immature colon carcinoma transcript 1
kmeans	1	Yellow	128	Isca2	10116.ENSRNOP00000016089	Iron-sulfur cluster assembly 2
kmeans	1	Yellow	128	Macrod1	10116.ENSRNOP00000028749	Removes ADP-ribose from asparatate and glutamate residues in proteins bearing a single ADP-ribose moiety. Inactive towards proteins bearing poly-ADP-ribose. Deacetylates O-acetyl-ADP ribose, a signaling molecule generated by the deacetylation of acetylated lysine residues in histones and other proteins. Plays a role in estrogen signaling. Binds to androgen receptor (AR) and amplifies the transactivation function of AR in response to androgen. May play an important role in carcinogenesis and/or progression of hormone-dependent cancers by feed- forward mechanism that activates ESR1 trans [...]
kmeans	1	Yellow	128	Mb	10116.ENSRNOP00000006184	Myoglobin; Serves as a reserve supply of oxygen and facilitates the movement of oxygen within muscles
kmeans	1	Yellow	128	Mrpl15	10116.ENSRNOP00000011619	Mitochondrial ribosomal protein L15
kmeans	1	Yellow	128	Mrpl27	10116.ENSRNOP00000004947	Mitochondrial ribosomal protein L27
kmeans	1	Yellow	128	Mrpl38	10116.ENSRNOP00000011328	Mitochondrial ribosomal protein L38; Belongs to the phosphatidylethanolamine-binding protein family. Mitochondrion-specific ribosomal protein mL38 subfamily
kmeans	1	Yellow	128	Mrpl41	10116.ENSRNOP00000042070	39S ribosomal protein L41, mitochondrial; Component of the mitochondrial ribosome large subunit. Also involved in apoptosis and cell cycle. Enhances p53/TP53 stability, thereby contributing to p53/TP53-induced apoptosis in response to growth-inhibitory condition. Enhances p53/TP53 translocation to the mitochondria. Has the ability to arrest the cell cycle at the G1 phase, possibly by stabilizing the CDKN1A and CDKN1B (p27Kip1) proteins
kmeans	1	Yellow	128	Mrpl50	10116.ENSRNOP00000009677	Mitochondrial ribosomal protein L50

kmeans	1	Yellow	128	Mrps22	10116.ENSRNOP00000067192	Mitochondrial ribosomal protein S22
kmeans	1	Yellow	128	Mrps23	10116.ENSRNOP00000013771	Mitochondrial ribosomal protein S23
kmeans	1	Yellow	128	Mrps25	10116.ENSRNOP00000014933	Mitochondrial ribosomal protein S25; Belongs to the mitochondrion-specific ribosomal protein mS25 family
kmeans	1	Yellow	128	Mrps6	10116.ENSRNOP00000002752	Mitochondrial ribosomal protein S6
kmeans	1	Yellow	128	Mrps7	10116.ENSRNOP00000005089	Mitochondrial ribosomal protein S7; Belongs to the universal ribosomal protein uS7 family
kmeans	1	Yellow	128	Mtch2	10116.ENSRNOP00000011846	Mitochondrial carrier 2; Belongs to the mitochondrial carrier (TC 2.A.29) family
kmeans	1	Yellow	128	Murc	10116.ENSRNOP00000010561	Caveolae-associated protein 4; Modulates the morphology of formed caveolae in cardiomyocytes, but is not required for caveolar formation. Facilitates the recruitment of MAPK1/3 to caveolae within cardiomyocytes and regulates alpha-1 adrenergic receptor-induced hypertrophic responses in cardiomyocytes through MAPK1/3 activation. Contributes to proper membrane localization and stabilization of caveolin-3 (CAV3) in cardiomyocytes (By similarity). Induces RHOA activation and activates NPPA transcription and myofibrillar organization through the Rho/ROCK signaling pathway; Belongs to the CA [...]
kmeans	1	Yellow	128	Myo1c	10116.ENSRNOP00000028929	Unconventional myosin-Ic; Myosins are actin-based motor molecules with ATPase activity. Unconventional myosins serve in intracellular movements. Their highly divergent tails are presumed to bind to membranous compartments, which would be moved relative to actin filaments. Involved in glucose transporter recycling in response to insulin by regulating movement of intracellular GLUT4-containing vesicles to the plasma membrane. Component of the hair cell's (the sensory cells of the inner ear) adaptation-motor complex. Acts as a mediator of adaptation of mechanoelectrical transduction in st [...]
kmeans	1	Yellow	128	Myom2	10116.ENSRNOP00000015908	Myomesin 2
kmeans	1	Yellow	128	Ndufa10	10116.ENSRNOP00000022089	NADH dehydrogenase [ubiquinone] 1 alpha subcomplex subunit 10, mitochondrial; Accessory subunit of the mitochondrial membrane respiratory chain NADH dehydrogenase (Complex I), that is believed not to be involved in catalysis. Complex I functions in the transfer of electrons from NADH to the respiratory chain. The immediate electron acceptor for the enzyme is believed to be ubiquinone
kmeans	1	Yellow	128	Ndufa11	10116.ENSRNOP00000064246	NADH dehydrogenase [ubiquinone] 1 alpha subcomplex subunit 11; Accessory subunit of the mitochondrial membrane respiratory chain NADH dehydrogenase (Complex I), that is believed not to be involved in catalysis. Complex I functions in the transfer of electrons from NADH to the respiratory chain. The immediate electron acceptor for the enzyme is believed to be ubiquinone
kmeans	1	Yellow	128	Ndufa12	10116.ENSRNOP00000010117	NADH dehydrogenase [ubiquinone] 1 alpha subcomplex subunit 12; Accessory subunit of the mitochondrial membrane respiratory chain NADH dehydrogenase (Complex I), that is believed not to be involved in catalysis. Complex I functions in the transfer of electrons from NADH to the respiratory chain. The immediate electron acceptor for the enzyme is believed to be ubiquinone
kmeans	1	Yellow	128	Ndufa2	10116.ENSRNOP00000023811	NADH dehydrogenase [ubiquinone] 1 alpha subcomplex subunit 2; Accessory subunit of the mitochondrial membrane respiratory chain NADH dehydrogenase (Complex I), that is believed not to be involved in catalysis. Complex I functions in the transfer of electrons from NADH to the respiratory chain. The immediate electron acceptor for the enzyme is believed to be ubiquinone
kmeans	1	Yellow	128	Ndufa4	10116.ENSRNOP00000007567	NDUFA4, mitochondrial complex-associated; NADH dehydrogenase (ubiquinone) 1 alpha subcomplex, 4
kmeans	1	Yellow	128	Ndufa5	10116.ENSRNOP00000008325	NADH dehydrogenase [ubiquinone] 1 alpha subcomplex subunit 5; Accessory subunit of the mitochondrial membrane respiratory chain NADH dehydrogenase (Complex I), that is believed not to be involved in catalysis. Complex I functions in the transfer of electrons from NADH to the respiratory chain. The immediate electron acceptor for the enzyme is believed to be ubiquinone

kmeans	1	Yellow	128	Ndufa6	10116.ENSRNOP00000011484	NADH dehydrogenase [ubiquinone] 1 alpha subcomplex subunit 6; Accessory subunit of the mitochondrial membrane respiratory chain NADH dehydrogenase (Complex I), that is believed to be not involved in catalysis. Complex I functions in the transfer of electrons from NADH to the respiratory chain. The immediate electron acceptor for the enzyme is believed to be ubiquinone
kmeans	1	Yellow	128	Ndufa8	10116.ENSRNOP00000050148	Accessory subunit of the mitochondrial membrane respiratory chain NADH dehydrogenase (Complex I), that is believed not to be involved in catalysis. Complex I functions in the transfer of electrons from NADH to the respiratory chain. The immediate electron acceptor for the enzyme is believed to be ubiquinone.
kmeans	1	Yellow	128	Ndufa9	10116.ENSRNOP00000034135	NADH dehydrogenase [ubiquinone] 1 alpha subcomplex subunit 9, mitochondrial; Accessory subunit of the mitochondrial membrane respiratory chain NADH dehydrogenase (Complex I), that is believed not to be involved in catalysis. Complex I functions in the transfer of electrons from NADH to the respiratory chain. The immediate electron acceptor for the enzyme is believed to be ubiquinone
kmeans	1	Yellow	128	Ndufab1	10116.ENSRNOP00000024471	Acyl carrier protein; Carrier of the growing fatty acid chain in fatty acid biosynthesis
kmeans	1	Yellow	128	Ndufaf3	10116.ENSRNOP00000027262	NADH dehydrogenase [ubiquinone] 1 alpha subcomplex assembly factor 3; Essential factor for the assembly of mitochondrial NADH:ubiquinone oxidoreductase complex (complex I)
kmeans	1	Yellow	128	Ndufaf6	10116.ENSRNOP00000058286	NADH dehydrogenase (ubiquinone) complex I, assembly factor 6; Involved in the assembly of mitochondrial NADH:ubiquinone oxidoreductase complex (complex I) at early stages. May play a role in the biogenesis of MT-ND1 (By similarity)
kmeans	1	Yellow	128	Ndufb10	10116.ENSRNOP00000019624	NADH:ubiquinone oxidoreductase subunit B10; NADH dehydrogenase (ubiquinone) 1 beta subcomplex, 10
kmeans	1	Yellow	128	Ndufb4	10116.ENSRNOP00000003674	NADH:ubiquinone oxidoreductase subunit B4; Similar to NADH dehydrogenase (ubiquinone) 1 beta subcomplex, 4, 15kDa
kmeans	1	Yellow	128	Ndufb6	10116.ENSRNOP00000037051	NADH dehydrogenase (ubiquinone) 1 beta subcomplex, 6
kmeans	1	Yellow	128	Ndufb7	10116.ENSRNOP00000034511	NADH dehydrogenase (ubiquinone) 1 beta subcomplex, 7
kmeans	1	Yellow	128	Ndufb8	10116.ENSRNOP00000019039	NADH dehydrogenase [ubiquinone] 1 beta subcomplex subunit 8, mitochondrial; Accessory subunit of the mitochondrial membrane respiratory chain NADH dehydrogenase (Complex I), that is believed not to be involved in catalysis. Complex I functions in the transfer of electrons from NADH to the respiratory chain. The immediate electron acceptor for the enzyme is believed to be ubiquinone
kmeans	1	Yellow	128	Ndufb9	10116.ENSRNOP00000012616	NADH dehydrogenase (ubiquinone) 1 beta subcomplex, 9; Belongs to the complex I LYR family
kmeans	1	Yellow	128	Ndufc2	10116.ENSRNOP00000016509	NADH dehydrogenase [ubiquinone] 1 subunit C2; Accessory subunit of the mitochondrial membrane respiratory chain NADH dehydrogenase (Complex I), that is believed not to be involved in catalysis. Complex I functions in the transfer of electrons from NADH to the respiratory chain. The immediate electron acceptor for the enzyme is believed to be ubiquinone
kmeans	1	Yellow	128	Ndufs1	10116.ENSRNOP00000015851	NADH-ubiquinone oxidoreductase 75 kDa subunit, mitochondrial; Core subunit of the mitochondrial membrane respiratory chain NADH dehydrogenase (Complex I) that is believed to belong to the minimal assembly required for catalysis. Complex I functions in the transfer of electrons from NADH to the respiratory chain. The immediate electron acceptor for the enzyme is believed to be ubiquinone (By similarity). This is the largest subunit of complex I and it is a component of the iron-sulfur (IP) fragment of the enzyme. It may form part of the active site crevice where NADH is oxidized (By sim [...]
kmeans	1	Yellow	128	Ndufs2	10116.ENSRNOP00000055224	NADH dehydrogenase [ubiquinone] iron-sulfur protein 2, mitochondrial; Core subunit of the mitochondrial membrane respiratory chain NADH dehydrogenase (Complex I) that is believed to belong to the minimal assembly required for catalysis. Complex I functions in the transfer of electrons from NADH to the respiratory chain. The immediate electron acceptor for the enzyme is believed to be ubiquinone
kmeans	1	Yellow	128	Ndufs3	10116.ENSRNOP00000012425	NADH dehydrogenase (ubiquinone) Fe-S protein 3; Belongs to the complex I 30 kDa subunit family

kmeans	1	Yellow	128	Ndufs4	10116.ENSRNOP00000015217	NADH dehydrogenase [ubiquinone] iron-sulfur protein 4, mitochondrial; Accessory subunit of the mitochondrial membrane respiratory chain NADH dehydrogenase (Complex I), that is believed not to be involved in catalysis. Complex I functions in the transfer of electrons from NADH to the respiratory chain. The immediate electron acceptor for the enzyme is believed to be ubiquinone
kmeans	1	Yellow	128	Ndufs5	10116.ENSRNOP00000037699	rCG31129-like
kmeans	1	Yellow	128	Ndufs7	10116.ENSRNOP00000037256	NADH dehydrogenase (ubiquinone) Fe-S protein 7; Belongs to the complex I 20 kDa subunit family
kmeans	1	Yellow	128	Ndufv1	10116.ENSRNOP00000024517	NADH dehydrogenase [ubiquinone] flavoprotein 1, mitochondrial; Core subunit of the mitochondrial membrane respiratory chain NADH dehydrogenase (Complex I) that is believed to belong to the minimal assembly required for catalysis. Complex I functions in the transfer of electrons from NADH to the respiratory chain
kmeans	1	Yellow	128	Ndufv2	10116.ENSRNOP00000016965	NADH dehydrogenase [ubiquinone] flavoprotein 2, mitochondrial; Core subunit of the mitochondrial membrane respiratory chain NADH dehydrogenase (Complex I) that is believed to belong to the minimal assembly required for catalysis. Complex I functions in the transfer of electrons from NADH to the respiratory chain. The immediate electron acceptor for the enzyme is believed to be ubiquinone (By similarity)
kmeans	1	Yellow	128	Ndufv3	10116.ENSRNOP00000001564	NADH dehydrogenase (ubiquinone) flavoprotein 3
kmeans	1	Yellow	128	Nlrx1	10116.ENSRNOP00000011620	NLR family member X1; Participates in antiviral signaling; Belongs to the NLRP family
kmeans	1	Yellow	128	Ociad1	10116.ENSRNOP00000002996	OCIA domain-containing protein 1; Maintains stem cell potency (By similarity). Increases STAT3 phosphorylation and controls ERK phosphorylation (By similarity). May act as a scaffold, increasing STAT3 recruitment onto endosomes (By similarity)
kmeans	1	Yellow	128	Pitrm1	10116.ENSRNOP00000063283	Pitrilysin metallopeptidase 1
kmeans	1	Yellow	128	Ptges2	10116.ENSRNOP00000019184	Prostaglandin E synthase 2
kmeans	1	Yellow	128	RGD1304704	10116.ENSRNOP00000049155	Similar to Hypothetical protein CGI-99
kmeans	1	Yellow	128	Rmdn1	10116.ENSRNOP00000030229	Regulator of microtubule dynamics 1; Belongs to the RMDN family
kmeans	1	Yellow	128	Romo1	10116.ENSRNOP00000026856	Reactive oxygen species modulator 1-like
kmeans	1	Yellow	128	Rpl19	10116.ENSRNOP00000006359	Ribosomal protein L19
kmeans	1	Yellow	128	Rpl23	10116.ENSRNOP00000005471	Ribosomal protein L23; Belongs to the universal ribosomal protein uL14 family
kmeans	1	Yellow	128	Rpl3l	10116.ENSRNOP00000019660	Ribosomal protein L3-like; Belongs to the universal ribosomal protein uL3 family

kmeans	1	Yellow	128	Rpl6	10116.ENSRNOP00000051135	Component of the large ribosomal subunit.
kmeans	1	Yellow	128	Rpl9	10116.ENSRNOP00000032635	Belongs to the universal ribosomal protein uL6 family.
kmeans	1	Yellow	128	Rplp0	10116.ENSRNOP00000001518	60S acidic ribosomal protein P0; Ribosomal protein P0 is the functional equivalent of E.coli protein L10
kmeans	1	Yellow	128	Rps2	10116.ENSRNOP00000019508	Ribosomal protein S2; Belongs to the universal ribosomal protein uS5 family
kmeans	1	Yellow	128	Rps23	10116.ENSRNOP00000022348	40S ribosomal protein S23; Component of the ribosome, a large ribonucleoprotein complex responsible for the synthesis of proteins in the cell. The small ribosomal subunit (SSU) binds messenger RNAs (mRNAs) and translates the encoded message by selecting cognate aminoacyl- transfer RNA (tRNA) molecules. The large subunit (LSU) contains the ribosomal catalytic site termed the peptidyl transferase center (PTC), which catalyzes the formation of peptide bonds, thereby polymerizing the amino acids delivered by tRNAs into a polypeptide chain. The nascent polypeptides leave the ribosome throug [...]
kmeans	1	Yellow	128	Rps5	10116.ENSRNOP00000026528	Ribosomal protein S5; Belongs to the universal ribosomal protein uS7 family
kmeans	1	Yellow	128	Sdr39u1	10116.ENSRNOP00000063340	Short chain dehydrogenase/reductase family 39U, member 1
kmeans	1	Yellow	128	Slc25a11	10116.ENSRNOP00000005144	Catalyzes the transport of 2-oxoglutarate across the inner mitochondrial membrane in an electroneutral exchange for malate or other dicarboxylic acids, and plays an important role in several metabolic processes, including the malate-aspartate shuttle, the oxoglutarate/isocitrate shuttle, in gluconeogenesis from lactate, and in nitrogen metabolism . Maintains mitochondrial fusion and fission events, and the organization and morphology of cristae (By similarity). Involved in the regulation of apoptosis (By similarity). ECO:0000250|UniProtKB:Q9CR62, ECO:0000269|PubMed:3355813,
kmeans	1	Yellow	128	Slc25a3	10116.ENSRNOP00000011494	Transport of phosphate groups from the cytosol to the mitochondrial matrix. Phosphate is cotransported with H(+). May play a role regulation of the mitochondrial permeability transition pore (mPTP)
kmeans	1	Yellow	128	Srl	10116.ENSRNOP00000039064	Sarcalumenin; Belongs to the TRAFAC class dynamin-like GTPase superfamily. Dynamin/Fzo/YdjA family
kmeans	1	Yellow	128	Ssr4	10116.ENSRNOP00000063309	Translocon-associated protein subunit delta; TRAP proteins are part of a complex whose function is to bind calcium to the ER membrane and thereby regulate the retention of ER resident proteins
kmeans	1	Yellow	128	Tapt1	10116.ENSRNOP00000004274	Transmembrane anterior posterior transformation 1
kmeans	1	Yellow	128	Tmem143	10116.ENSRNOP00000028650	Putative uncharacterized protein RGD1305013_predicted; Transmembrane protein 143
kmeans	1	Yellow	128	Tpm5	10116.ENSRNOP00000023567	Tropomyosin alpha-3 chain; Binds to actin filaments in muscle and non-muscle cells. Plays a central role, in association with the troponin complex, in the calcium dependent regulation of vertebrate striated muscle contraction. Smooth muscle contraction is regulated by interaction with caldesmon. In non-muscle cells is implicated in stabilizing cytoskeleton actin filaments
kmeans	1	Yellow	128	Tpp1	10116.ENSRNOP00000026280	Lysosomal serine protease with tripeptidyl-peptidase I activity. May act as a non-specific lysosomal peptidase which generates tripeptides from the breakdown products produced by lysosomal proteinases. Requires substrates with an unsubstituted N-terminus.

kmeans	1	Yellow	128	Tpt1	10116.ENSRNOP00000001383	Translationally-controlled tumor protein; Involved in calcium binding and microtubule stabilization
kmeans	1	Yellow	128	Tufm	10116.ENSRNOP00000025203	Elongation factor Tu, mitochondrial; This protein promotes the GTP-dependent binding of aminoacyl-tRNA to the A-site of ribosomes during protein biosynthesis
kmeans	1	Yellow	128	Uqcrb	10116.ENSRNOP00000033706	Similar to ubiquinol-cytochrome c reductase binding protein
kmeans	1	Yellow	128	Uqcrc1	10116.ENSRNOP00000044696	Cytochrome b-c1 complex subunit 1, mitochondrial; This is a component of the ubiquinol-cytochrome c reductase complex (complex III or cytochrome b-c1 complex), which is part of the mitochondrial respiratory chain. This protein may mediate formation of the complex between cytochromes c and c1 (By similarity)
kmeans	1	Yellow	128	Uqcrc2	10116.ENSRNOP00000021514	Cytochrome b-c1 complex subunit 2, mitochondrial; This is a component of the ubiquinol-cytochrome c reductase complex (complex III or cytochrome b-c1 complex), which is part of the mitochondrial respiratory chain. The core protein 2 is required for the assembly of the complex; Belongs to the peptidase M16 family. UQCRC2/QCR2 subfamily
kmeans	1	Yellow	128	Uqcrfs1	10116.ENSRNOP00000024609	Cytochrome b-c1 complex subunit Rieske, mitochondrial; Cytochrome b-c1 complex subunit Rieske, mitochondrial: Component of the mitochondrial ubiquinol-cytochrome c reductase complex dimer (complex III dimer), which is a respiratory chain that generates an electrochemical potential coupled to ATP synthesis (By similarity). Incorporation of UQCRFS1 is the penultimate step in complex III assembly (By similarity)
kmeans	1	Yellow	128	Uqcrh	10116.ENSRNOP00000016751	Cytochrome b-c1 complex subunit 6, mitochondrial; This is a component of the ubiquinol-cytochrome c reductase complex (complex III or cytochrome b-c1 complex), which is part of the mitochondrial respiratory chain. This protein may mediate formation of the complex between cytochromes c and c1 (By similarity)
kmeans	1	Yellow	128	Usmg5	10116.ENSRNOP00000027496	Up-regulated during skeletal muscle growth protein 5; Plays a critical role in maintaining the ATP synthase population in mitochondria
kmeans	1	Yellow	128	rCG_38845	10116.ENSRNOP00000027980	NADH dehydrogenase (ubiquinone) 1 alpha subcomplex, 13
kmeans	2	Green	87	Abcb8	10116.ENSRNOP00000012222	ATP-binding cassette, subfamily B (MDR/TAP), member 8
kmeans	2	Green	87	Acaa2	10116.ENSRNOP00000060140	In the production of energy from fats, this is one of the enzymes that catalyzes the last step of the mitochondrial beta- oxidation pathway, an aerobic process breaking down fatty acids into acetyl-CoA (Probable). Using free coenzyme A/CoA, catalyzes the thiolytic cleavage of medium- to long-chain unbranched 3-oxoacyl-CoAs into acetyl-CoA and a fatty acyl-CoA shortened by two carbon atoms (Probable). Also catalyzes the condensation of two acetyl-CoA molecules into acetoacetyl-CoA and could be involved in the production of ketone bodies (Probable). Also displays hydrolase activity on va [...]
kmeans	2	Green	87	Acadl	10116.ENSRNOP00000017686	Long-chain specific acyl-CoA dehydrogenase, mitochondrial; acyl-CoA dehydrogenase, long chain
kmeans	2	Green	87	Acadm	10116.ENSRNOP00000013238	Acyl-CoA dehydrogenase specific for acyl chain lengths of 4 to 16 that catalyzes the initial step of fatty acid beta-oxidation. Utilizes the electron transfer flavoprotein (ETF) as an electron acceptor to transfer electrons to the main mitochondrial respiratory chain via ETF-ubiquinone oxidoreductase (ETF dehydrogenase).
kmeans	2	Green	87	Acads	10116.ENSRNOP00000001556	Short-chain specific acyl-CoA dehydrogenase, mitochondrial; Introduces a double bond at position 2 in saturated acyl-CoAs of short chain length, i.e. less than 6 carbon atoms; Belongs to the acyl-CoA dehydrogenase family
kmeans	2	Green	87	Acadvl	10116.ENSRNOP00000024973	Active toward esters of long-chain and very long chain fatty acids such as palmitoyl-CoA, myristoyl-CoA and stearoyl-CoA. Can accommodate substrate acyl chain lengths as long as 24 carbons, but shows little activity for substrates of less than 12 carbons

kmeans	2	Green	87	Acox1	10116.ENSRNOP00000051538	Peroxisomal acyl-coenzyme A oxidase 1; Catalyzes the desaturation of acyl-CoAs to 2-trans- enoyl-CoAs. Isoform 1 shows highest activity against medium-chain fatty acyl-CoAs and activity decreases with increasing chain length. Isoform 2 is active against a much broader range of substrates and shows activity towards very long-chain acyl-CoAs. Isoform 1 shows optimum activity with a chain length of 10 carbons while isoform 2 exhibits optimum activity with 14 carbons
kmeans	2	Green	87	Acsf3	10116.ENSRNOP00000020313	acyl-CoA synthetase family member 3
kmeans	2	Green	87	Acsl1	10116.ENSRNOP00000014235	Long-chain-fatty-acid--CoA ligase 1; Activation of long-chain fatty acids for both synthesis of cellular lipids, and degradation via beta-oxidation. Preferentially uses oleate, arachidonate, eicosapentaenoate and docosahexaenoate as substrates
kmeans	2	Green	87	Agk	10116.ENSRNOP00000015744	Acylglycerol kinase
kmeans	2	Green	87	Agl	10116.ENSRNOP00000052593	Amylo-alpha-1, 6-glucosidase, 4-alpha-glucanotransferase
kmeans	2	Green	87	Aldh6a1	10116.ENSRNOP00000015545	Plays a role in valine and pyrimidine metabolism. Binds fatty acyl-CoA
kmeans	2	Green	87	Aldh7a1	10116.ENSRNOP00000020325	Alpha-aminoadipic semialdehyde dehydrogenase; Multifunctional enzyme mediating important protective effects. Metabolizes betaine aldehyde to betaine, an important cellular osmolyte and methyl donor. Protects cells from oxidative stress by metabolizing a number of lipid peroxidation-derived aldehydes. Involved in lysine catabolism (By similarity); Belongs to the aldehyde dehydrogenase family
kmeans	2	Green	87	Anpep	10116.ENSRNOP00000020002	Broad specificity aminopeptidase which plays a role in the final digestion of peptides generated from hydrolysis of proteins by gastric and pancreatic proteases. Also involved in the processing of various peptides including peptide hormones, such as angiotensin III and IV, neuropeptides, and chemokines. May also be involved the cleavage of peptides bound to major histocompatibility complex class II molecules of antigen presenting cells. May have a role in angiogenesis and promote cholesterol crystallization. May have a role in amino acid transport by acting as binding partner of amino [...]
kmeans	2	Green	87	Anxa11	10116.ENSRNOP00000014940	Annexin A11
kmeans	2	Green	87	Anxa6	10116.ENSRNOP00000014464	May associate with CD21. May regulate the release of Ca(2+) from intracellular stores
kmeans	2	Green	87	Arf1	10116.ENSRNOP00000064960	ADP-ribosylation factor 1; GTP-binding protein that functions as an allosteric activator of the cholera toxin catalytic subunit, an ADP- ribosyltransferase. Involved in protein trafficking among different compartments. Modulates vesicle budding and uncoating within the Golgi complex. Deactivation induces the redistribution of the entire Golgi complex to the endoplasmic reticulum, suggesting a crucial role in protein trafficking. In its GTP-bound form, its triggers the association with coat proteins with the Golgi membrane. The hydrolysis of ARF1-bound GTP, which is mediated by ARFGAPs [...]
kmeans	2	Green	87	Art3	10116.ENSRNOP00000039770	ADP-ribosyltransferase 3
kmeans	2	Green	87	Atpaf2	10116.ENSRNOP00000004948	ATP synthase mitochondrial F1 complex assembly factor 2
kmeans	2	Green	87	Bdh1	10116.ENSRNOP00000002366	D-beta-hydroxybutyrate dehydrogenase, mitochondrial; 3-hydroxybutyrate dehydrogenase, type 1
kmeans	2	Green	87	Cd36	10116.ENSRNOP00000058398	Multifunctional glycoprotein that acts as receptor for a broad range of ligands. Ligands can be of proteinaceous nature like thrombospondin, fibronectin, collagen or amyloid-beta as well as of lipidic nature such as oxidized low-density lipoprotein (oxLDL), anionic phospholipids, long-chain fatty acids and bacterial diacylated lipopeptides. They are generally multivalent and can therefore engage multiple receptors simultaneously, the resulting formation of CD36 clusters initiates signal transduction and internalization of receptor- ligand complexes. The dependency on coreceptor signali [...]

kmeans	2	Green	87	Cfl2	10116.ENSRNOP00000067174	Cofilin 2, muscle; Belongs to the actin-binding proteins ADF family
kmeans	2	Green	87	Clic4	10116.ENSRNOP00000024464	Can insert into membranes and form poorly selective ion channels that may also transport chloride ions. Channel activity depends on the pH. Membrane insertion seems to be redox-regulated and may occur only under oxydizing conditions. Promotes cell-surface expression of HRH3. May play a role in angiogenesis (By similarity).
kmeans	2	Green	87	Cpt1b	10116.ENSRNOP00000013985	Carnitine O-palmitoyltransferase 1, muscle isoform; Carnitine palmitoyltransferase 1b, muscle; Belongs to the carnitine/choline acetyltransferase family
kmeans	2	Green	87	Cpt2	10116.ENSRNOP00000016954	Carnitine O-palmitoyltransferase 2, mitochondrial; Carnitine palmitoyltransferase 2; Belongs to the carnitine/choline acetyltransferase family
kmeans	2	Green	87	Crat	10116.ENSRNOP00000063089	Carnitine O-acetyltransferase; Carnitine acetylase is specific for short chain fatty acids. Carnitine acetylase seems to affect the flux through the pyruvate dehydrogenase complex. It may be involved as well in the transport of acetyl-CoA into mitochondria (By similarity)
kmeans	2	Green	87	Cryab	10116.ENSRNOP00000055901	Alpha-crystallin B chain; May contribute to the transparency and refractive index of the lens. Has chaperone-like activity, preventing aggregation of various proteins under a wide range of stress conditions; Belongs to the small heat shock protein (HSP20) family
kmeans	2	Green	87	D2hgdh	10116.ENSRNOP00000025711	Catalyzes the oxidation of D-2-hydroxyglutarate to alpha- ketoglutarate
kmeans	2	Green	87	Dbi	10116.ENSRNOP00000066874	Binds medium- and long-chain acyl-CoA esters with very high affinity and may function as an intracellular carrier of acyl-CoA esters. It is also able to displace diazepam from the benzodiazepine (BZD) recognition site located on the GABA type A receptor. It is therefore possible that this protein also acts as a neuropeptide to modulate the action of the GABA receptor
kmeans	2	Green	87	Decr1	10116.ENSRNOP00000011330	Auxiliary enzyme of beta-oxidation. It participates in the metabolism of unsaturated fatty enoyl-CoA esters having double bonds in both even- and odd-numbered positions in mitochondria. Catalyzes the NADP-dependent reduction of 2,4-dienoyl-CoA to yield trans-3-enoyl-CoA.
kmeans	2	Green	87	Dhrs4	10116.ENSRNOP00000024782	Dehydrogenase/reductase SDR family member 4; Reduces all-trans-retinal and 9-cis retinal. Can also catalyze the oxidation of all-trans-retinol with NADP as co- factor, but with much lower efficiency. Reduces alkyl phenyl ketones and alpha-dicarbonyl compounds with aromatic rings, such as pyrimidine-4-aldehyde, 3-benzoylpyridine, 4-benzoylpyridine, menadione and 4-hexanoylpyridine. Has no activity towards aliphatic aldehydes and ketones (By similarity)
kmeans	2	Green	87	ENSRNOG00000018522	10116.ENSRNOP00000025446	Enoyl CoA hydratase, short chain, 1, mitochondrial
kmeans	2	Green	87	Ech1	10116.ENSRNOP00000027537	Delta(3,5)-Delta(2,4)-dienoyl-CoA isomerase, mitochondrial; Isomerization of 3-trans,5-cis-dienoyl-CoA to 2-trans,4- trans-dienoyl-CoA; Belongs to the enoyl-CoA hydratase/isomerase family
kmeans	2	Green	87	Eci2	10116.ENSRNOP00000022022	Enoyl-CoA delta isomerase 2, mitochondrial; Able to isomerize both 3-cis and 3-trans double bonds into the 2-trans form in a range of enoyl-CoA species. Has a preference for 3-trans substrates
kmeans	2	Green	87	Eef1a2	10116.ENSRNOP00000016947	Elongation factor 1-alpha 2; This protein promotes the GTP-dependent binding of aminoacyl-tRNA to the A-site of ribosomes during protein biosynthesis
kmeans	2	Green	87	Ehhadh	10116.ENSRNOP00000002410	Peroxisomal bifunctional enzyme; enoyl-CoA, hydratase/3-hydroxyacyl CoA dehydrogenase; In the N-terminal section; belongs to the enoyl-CoA hydratase/isomerase family
kmeans	2	Green	87	Epb41l2	10116.ENSRNOP00000058049	Similar to protein 4.1G (Predicted), isoform CRA_a; Erythrocyte membrane protein band 4.1-like 2

kmeans	2	Green	87	Etfb	10116.ENSRNOP00000024083	Electron transfer flavoprotein subunit beta; Heterodimeric electron transfer flavoprotein that accepts electrons from several mitochondrial dehydrogenases, including acyl-CoA dehydrogenases, glutaryl-CoA and sarcosine dehydrogenase. It transfers the electrons to the main mitochondrial respiratory chain via ETF-ubiquinone oxidoreductase. Required for normal mitochondrial fatty acid oxidation and normal amino acid metabolism. ETFB binds an AMP molecule that probably has a purely structural role (By similarity)
kmeans	2	Green	87	Etfdh	10116.ENSRNOP00000013262	Accepts electrons from ETF and reduces ubiquinone.
kmeans	2	Green	87	Fam162a	10116.ENSRNOP00000040367	Protein FAM162A; Proposed to be involved in regulation of apoptosis; the exact mechanism may differ between cell types/tissues. May be involved in hypoxia-induced cell death of transformed cells implicating cytochrome C release and caspase activation (such as CASP9) and inducing mitochondrial permeability transition. May be involved in hypoxia-induced cell death of neuronal cells probably by promoting release of AIFM1 from mitochondria to cytoplasm and its translocation to the nucleus; Belongs to the UPF0389 family
kmeans	2	Green	87	Fbn1	10116.ENSRNOP00000052876	Fibrillin 1, isoform CRA_a; Fibrillin 1
kmeans	2	Green	87	Fermt2	10116.ENSRNOP00000012400	Fermitin family member 2
kmeans	2	Green	87	Gba	10116.ENSRNOP00000063935	Glucosylceramidase; Glucosidase, beta, acid
kmeans	2	Green	87	Gcdh	10116.ENSRNOP00000004570	glutaryl-CoA dehydrogenase
kmeans	2	Green	87	Gpx4	10116.ENSRNOP00000018691	Phospholipid hydroperoxide glutathione peroxidase, nuclear; Could play a major role in protecting mammals from the toxicity of ingested lipid hydroperoxides. Essential for embryonic development. Protects from radiation and oxidative damage. Stabilizes the condensed chromatin in sperm nuclei and is necessary male fertility; Belongs to the glutathione peroxidase family
kmeans	2	Green	87	Hadh	10116.ENSRNOP00000014658	Hydroxyacyl-coenzyme A dehydrogenase, mitochondrial; Plays an essential role in the mitochondrial beta- oxidation of short chain fatty acids. Exerts it highest activity toward 3-hydroxybutyryl-CoA; Belongs to the 3-hydroxyacyl-CoA dehydrogenase family
kmeans	2	Green	87	Hadha	10116.ENSRNOP00000038073	Trifunctional enzyme subunit alpha, mitochondrial; Bifunctional subunit; In the N-terminal section; belongs to the enoyl-CoA hydratase/isomerase family
kmeans	2	Green	87	Hibadh	10116.ENSRNOP00000011069	3-hydroxyisobutyrate dehydrogenase, mitochondrial; 3-hydroxyisobutyrate dehydrogenase
kmeans	2	Green	87	Hibch	10116.ENSRNOP00000032041	3-hydroxyisobutyryl-CoA hydrolase, mitochondrial; Hydrolyzes 3-hydroxyisobutyryl-CoA (HIBYL-CoA), a saline catabolite. Has high activity toward isobutyryl-CoA. Could be an isobutyryl-CoA dehydrogenase that functions in valine catabolism. Also hydrolyzes 3-hydroxypropanoyl-CoA (By similarity)
kmeans	2	Green	87	Hsd17b10	10116.ENSRNOP00000043608	Mitochondrial dehydrogenase that catalyzes the beta-oxidation at position 17 of androgens and estrogens and has 3-alpha- hydroxysteroid dehydrogenase activity with androsterone. Catalyzes the third step in the beta-oxidation of fatty acids. Carries out oxidative conversions of 7-alpha-OH and 7-beta-OH bile acids. Also exhibits 20- beta-OH and 21-OH dehydrogenase activities with C21 steroids. By interacting with intracellular amyloid-beta, it may contribute to the neuronal dysfunction associated with Alzheimer disease (AD). Essential for structural and functional integrity of mitochondria.
kmeans	2	Green	87	Hsd17b4	10116.ENSRNOP00000021646	Peroxisomal multifunctional enzyme type 2; Bifunctional enzyme acting on the peroxisomal beta- oxidation pathway for fatty acids. Catalyzes the formation of 3- ketoacyl-CoA intermediates from both straight-chain and 2-methyl- branched-chain fatty acids; Belongs to the short-chain dehydrogenases/reductases (SDR) family
kmeans	2	Green	87	Hsdl2	10116.ENSRNOP00000056213	Hydroxysteroid dehydrogenase-like protein 2; Has apparently no steroid dehydrogenase activity

kmeans	2	Green	87	Iars2	10116.ENSRNOP00000054206	isoleucyl-tRNA synthetase 2, mitochondrial; Belongs to the class-I aminoacyl-tRNA synthetase family
kmeans	2	Green	87	Ivd	10116.ENSRNOP00000013829	Isovaleryl-CoA dehydrogenase, mitochondrial; isovaleryl-CoA dehydrogenase
kmeans	2	Green	87	Kif5b	10116.ENSRNOP00000023860	Kinesin-1 heavy chain; Microtubule-dependent motor required for normal distribution of mitochondria and lysosomes. Can induce formation of neurite-like membrane protrusions in non-neuronal cells in a ZFYVE27-dependent manner. Regulates centrosome and nuclear positioning during mitotic entry. During the G2 phase of the cell cycle in a BICD2-dependent manner, antagonizes dynein function and drives the separation of nuclei and centrosomes; Belongs to the TRAFAC class myosin-kinesin ATPase superfamily. Kinesin family. Kinesin subfamily
kmeans	2	Green	87	L2hgdh	10116.ENSRNOP00000006473	L-2-hydroxyglutarate dehydrogenase
kmeans	2	Green	87	Lactb	10116.ENSRNOP00000024452	Lactamase, beta
kmeans	2	Green	87	Lap3	10116.ENSRNOP00000004770	Cytosol aminopeptidase; Presumably involved in the processing and regular turnover of intracellular proteins. Catalyzes the removal of unsubstituted N-terminal amino acids from various peptides (By similarity)
kmeans	2	Green	87	Lcp1	10116.ENSRNOP00000014502	Lymphocyte cytosolic protein 1
kmeans	2	Green	87	Lonp1	10116.ENSRNOP00000066618	Lon protease homolog, mitochondrial; ATP-dependent serine protease that mediates the selective degradation of misfolded, unassembled or oxidatively damaged polypeptides as well as certain short-lived regulatory proteins in the mitochondrial matrix. May also have a chaperone function in the assembly of inner membrane protein complexes. Participates in the regulation of mitochondrial gene expression and in the maintenance of the integrity of the mitochondrial genome. Binds to mitochondrial promoters and RNA in a single- stranded, site-specific, and strand-specific manner. May regulate mi [...]
kmeans	2	Green	87	Lpcat3	10116.ENSRNOP00000017090	Lysophospholipid acyltransferase 5; Seems to be the major enzyme contributing to lysophosphatidylcholine acyltransferase activity in the liver. Favors unsaturated fatty acyl-CoAs as acyl donors compared to saturated fatty acyl-CoAs. Displays lysophosphatidylserine acyltransferase (LPSAT) activity (By similarity)
kmeans	2	Green	87	Maoa	10116.ENSRNOP00000063784	Catalyzes the oxidative deamination of biogenic and xenobiotic amines and has important functions in the metabolism of neuroactive and vasoactive amines in the central nervous system and peripheral tissues. MAOA preferentially oxidizes biogenic amines such as 5-hydroxytryptamine (5-HT), norepinephrine and epinephrine
kmeans	2	Green	87	Mccc1	10116.ENSRNOP00000018942	Biotin-attachment subunit of the 3-methylcrotonyl-CoA carboxylase, an enzyme that catalyzes the conversion of 3- methylcrotonyl-CoA to 3-methylglutaconyl-CoA, a critical step for leucine and isovaleric acid catabolism
kmeans	2	Green	87	Mccc2	10116.ENSRNOP00000023900	Methylcrotonoyl-CoA carboxylase beta chain, mitochondrial; Carboxyltransferase subunit of the 3-methylcrotonyl-CoA carboxylase, an enzyme that catalyzes the conversion of 3- methylcrotonyl-CoA to 3-methylglutaconyl-CoA, a critical step for leucine and isovaleric acid catabolism
kmeans	2	Green	87	Mecr	10116.ENSRNOP00000031375	Enoyl-[acyl-carrier-protein] reductase, mitochondrial; Catalyzes the NADPH-dependent reduction of trans-2-enoyl thioesters in mitochondrial fatty acid synthesis (fatty acid synthesis type II). Fatty acid chain elongation in mitochondria uses acyl carrier protein (ACP) as an acyl group carrier, but the enzyme accepts both ACP and CoA thioesters as substrates in vitro. Has a preference for short and medium chain substrates, including trans-2-hexenoyl-CoA (C6), trans-2-decenoyl-CoA (C10), and trans- 2-hexadecenoyl-CoA (C16); Belongs to the zinc-containing alcohol dehydrogenase family. Qui [...]
kmeans	2	Green	87	Mgll	10116.ENSRNOP00000030271	Monoglyceride lipase; Converts monoacylglycerides to free fatty acids and glycerol. Hydrolyzes the endocannabinoid 2-arachidonoylglycerol, and thereby contributes to the regulation of endocannabinoid signaling, nociperception and perception of pain. Regulates the levels of fatty acids that serve as signaling molecules and promote cancer cell migration, invasion and tumor growth; Belongs to the AB hydrolase superfamily. Monoacylglycerol lipase family
kmeans	2	Green	87	Mlycd	10116.ENSRNOP00000019923	Malonyl-CoA decarboxylase, mitochondrial; Catalyzes the conversion of malonyl-CoA to acetyl-CoA. In the fatty acid biosynthesis MCD selectively removes malonyl-CoA and thus assures that methyl-malonyl-CoA is the only chain elongating substrate for fatty acid synthase and that fatty acids with multiple methyl side chains are produced. In peroxisomes it may be involved in degrading intraperoxisomal malonyl-CoA, which is generated by the peroxisomal beta-oxidation of odd chain-length dicarboxylic fatty acids. Plays a role in the metabolic balance between glucose and lipid oxidation in mus [...]

kmeans	2	Green	87	Mrps36	10116.ENSRNOP00000065298	Mitochondrial ribosomal protein S36
kmeans	2	Green	87	Nnt	10116.ENSRNOP00000029578	Nicotinamide nucleotide transhydrogenase
kmeans	2	Green	87	Oxct1	10116.ENSRNOP00000063646	Succinyl-CoA:3-ketoacid coenzyme A transferase 1, mitochondrial; Key enzyme for ketone body catabolism. Transfers the CoA moiety from succinate to acetoacetate. Formation of the enzyme-CoA intermediate proceeds via an unstable anhydride species formed between the carboxylate groups of the enzyme and substrate (By similarity)
kmeans	2	Green	87	Oxsm	10116.ENSRNOP00000008035	3-oxoacyl-[acyl-carrier-protein] synthase, mitochondrial; May play a role in the biosynthesis of lipoic acid as well as longer chain fatty acids required for optimal mitochondrial function; Belongs to the beta-ketoacyl-ACP synthases family
kmeans	2	Green	87	Pccb	10116.ENSRNOP00000021657	Propionyl-CoA carboxylase beta chain, mitochondrial; Propionyl CoA carboxylase, beta polypeptide; Belongs to the AccD/PCCB family
kmeans	2	Green	87	Pdcd6ip	10116.ENSRNOP00000012114	Programmed cell death 6-interacting protein; Multifunctional protein involved in endocytosis, multivesicular body biogenesis, membrane repair, cytokinesis, apoptosis and maintenance of tight junction integrity. Class E VPS protein involved in concentration and sorting of cargo proteins of the multivesicular body (MVB) for incorporation into intralumenal vesicles (ILVs) that are generated by invagination and scission from the limiting membrane of the endosome. Binds to the phospholipid lysobisphosphatidic acid (LBPA) which is abundant in MVBs internal membranes. The MVB pathway requires [...]
kmeans	2	Green	87	Pecr	10116.ENSRNOP00000021512	Participates in chain elongation of fatty acids. Has no 2,4- dienoyl-CoA reductase activity (By similarity)
kmeans	2	Green	87	Pls3	10116.ENSRNOP00000056912	Actin-bundling protein
kmeans	2	Green	87	Pnpt1	10116.ENSRNOP00000004919	Polyribonucleotide nucleotidyltransferase 1
kmeans	2	Green	87	Prdx6	10116.ENSRNOP00000030323	Peroxiredoxin-6; Thiol-specific peroxidase that catalyzes the reduction of hydrogen peroxide and organic hydroperoxides to water and alcohols, respectively. Can reduce H(2)O(2) and short chain organic, fatty acid, and phospholipid hydroperoxides. Also has phospholipase activity, and can therefore either reduce the oxidized sn-2 fatty acyl grup of phospholipids (peroxidase activity) or hydrolyze the sn-2 ester bond of phospholipids (phospholipase activity). These activities are dependent on binding to phospholipids at acidic pH and to oxidized phospholipds at cytosolic pH. Plays a role [...]
kmeans	2	Green	87	Pygm	10116.ENSRNOP00000028636	Phosphorylase is an important allosteric enzyme in carbohydrate metabolism. Enzymes from different sources differ in their regulatory mechanisms and in their natural substrates. However, all known phosphorylases share catalytic and structural properties
kmeans	2	Green	87	RGD1565784	10116.ENSRNOP00000040141	RGD1565784
kmeans	2	Green	87	Rab12	10116.ENSRNOP00000059602	Ras-related protein Rab-12; The small GTPases Rab are key regulators of intracellular membrane trafficking, from the formation of transport vesicles to their fusion with membranes. Rabs cycle between an inactive GDP-bound form and an active GTP-bound form that is able to recruit to membranes different set of downstream effectors directly responsible for vesicle formation, movement, tethering and fusion. That Rab may play a role in protein transport from recycling endosomes to lysosomes regulating, for instance, the degradation of the transferrin receptor. Involved in autophagy (By simi [...]
kmeans	2	Green	87	Rab2a	10116.ENSRNOP00000008522	Required for protein transport from the endoplasmic reticulum to the Golgi complex
kmeans	2	Green	87	Scamp1	10116.ENSRNOP00000014602	Secretory carrier-associated membrane protein 1; Functions in post-Golgi recycling pathways. Acts as a recycling carrier to the cell surface

kmeans	2	Green	87	Scp2	10116.ENSRNOP00000015420	Mediates in vitro the transfer of all common phospholipids, cholesterol and gangliosides between membranes. May play a role in regulating steroidogenesis
kmeans	2	Green	87	Sirt3	10116.ENSRNOP00000018861	NAD-dependent protein deacetylase; NAD-dependent protein deacetylase
kmeans	2	Green	87	Sod2	10116.ENSRNOP00000025794	Superoxide dismutase [Mn], mitochondrial; Destroys superoxide anion radicals which are normally produced within the cells and which are toxic to biological systems
kmeans	2	Green	87	Speg	10116.ENSRNOP00000026941	Striated muscle-specific serine/threonine-protein kinase; Isoform 2 may have a role in regulating the growth and differentiation of arterial smooth muscle cells; Belongs to the protein kinase superfamily. CAMK Ser/Thr protein kinase family
kmeans	2	Green	87	Sptan1	10116.ENSRNOP00000042382	Fodrin, which seems to be involved in secretion, interacts with calmodulin in a calcium-dependent manner and is thus candidate for the calcium-dependent movement of the cytoskeleton at the membrane.
kmeans	3	Purple	59	Aco2	10116.ENSRNOP00000029144	Aconitate hydratase, mitochondrial; Catalyzes the isomerization of citrate to isocitrate via cis-aconitate; Belongs to the aconitase/IPM isomerase family
kmeans	3	Purple	59	Acot2	10116.ENSRNOP00000013515	Acyl-CoA thioesterases are a group of enzymes that catalyze the hydrolysis of acyl-CoAs to the free fatty acid and coenzyme A (CoASH), providing the potential to regulate intracellular levels of acyl-CoAs, free fatty acids and CoASH (By similarity). Acyl-coenzyme A thioesterase 2/ACOT2 displays higher activity toward long chain acyl CoAs (C14-C20) . The enzyme is involved in enhancing the hepatic fatty acid oxidation in mitochondria (By similarity).
kmeans	3	Purple	59	Afg3l2	10116.ENSRNOP00000024632	AFG3-like AAA ATPase 2
kmeans	3	Purple	59	Ak2	10116.ENSRNOP00000000134	Adenylate kinase 2, mitochondrial; Catalyzes the reversible transfer of the terminal phosphate group between ATP and AMP. Plays an important role in cellular energy homeostasis and in adenine nucleotide metabolism. Adenylate kinase activity is critical for regulation of the phosphate utilization and the AMP de novo biosynthesis pathways. Plays a key role in hematopoiesis
kmeans	3	Purple	59	Aldoa	10116.ENSRNOP00000032320	Fructose-bisphosphate aldolase A; Plays a key role in glycolysis and gluconeogenesis. In addition, may also function as scaffolding protein (By similarity); Belongs to the class I fructose-bisphosphate aldolase family
kmeans	3	Purple	59	Atp5a1	10116.ENSRNOP00000022892	Mitochondrial membrane ATP synthase (F(1)F(0) ATP synthase or Complex V) produces ATP from ADP in the presence of a proton gradient across the membrane which is generated by electron transport complexes of the respiratory chain. F-type ATPases consist of two structural domains, F(1) - containing the extramembraneous catalytic core, and F(0) - containing the membrane proton channel, linked together by a central stalk and a peripheral stalk. During catalysis, ATP synthesis in the catalytic domain of F(1) is coupled via a rotary mechanism of the central stalk subunits to proton translocat [...]
kmeans	3	Purple	59	Atp5b	10116.ENSRNOP00000003965	Mitochondrial membrane ATP synthase (F(1)F(0) ATP synthase or Complex V) produces ATP from ADP in the presence of a proton gradient across the membrane which is generated by electron transport complexes of the respiratory chain. F-type ATPases consist of two structural domains, F(1) - containing the extramembraneous catalytic core, and F(0) - containing the membrane proton channel, linked together by a central stalk and a peripheral stalk. During catalysis, ATP synthesis in the catalytic domain of F(1) is coupled via a rotary mechanism of the central stalk subunits to proton translocat [...]
kmeans	3	Purple	59	Bcat2	10116.ENSRNOP00000028474	Catalyzes the first reaction in the catabolism of the essential branched chain amino acids leucine, isoleucine, and valine. May also function as a transporter of branched chain alpha-keto acids.
kmeans	3	Purple	59	Bckdha	10116.ENSRNOP00000027995	2-oxoisovalerate dehydrogenase subunit alpha, mitochondrial; The branched-chain alpha-keto dehydrogenase complex catalyzes the overall conversion of alpha-keto acids to acyl-CoA and CO(2). It contains multiple copies of three enzymatic components: branched-chain alpha-keto acid decarboxylase (E1), lipoamide acyltransferase (E2) and lipoamide dehydrogenase (E3); Belongs to the BCKDHA family
kmeans	3	Purple	59	Bckdhb	10116.ENSRNOP00000013249	2-oxoisovalerate dehydrogenase subunit beta, mitochondrial; The branched-chain alpha-keto dehydrogenase complex catalyzes the overall conversion of alpha-keto acids to acyl-CoA and CO(2). It contains multiple copies of three enzymatic components: branched-chain alpha-keto acid decarboxylase (E1), lipoamide acyltransferase (E2) and lipoamide dehydrogenase (E3)

kmeans	3	Purple	59	Cs	10116.ENSRNOP00000034921	Citrate synthase, mitochondrial; Citrate synthase; Belongs to the citrate synthase family
kmeans	3	Purple	59	Dbt	10116.ENSRNOP00000020267	Dihydrolipoamide acetyltransferase component of pyruvate dehydrogenase complex; Dihydrolipoamide branched chain transacylase E2
kmeans	3	Purple	59	Dlat	10116.ENSRNOP00000032890	Dihydrolipoamide S-acetyltransferase; The pyruvate dehydrogenase complex catalyzes the overall conversion of pyruvate to acetyl-CoA and CO(2), and thereby links the glycolytic pathway to the tricarboxylic cycle
kmeans	3	Purple	59	Dlst	10116.ENSRNOP00000007298	Dihydrolipoamide succinyltransferase (E2) component of the 2- oxoglutarate dehydrogenase complex (By similarity). The 2-oxoglutarate dehydrogenase complex catalyzes the overall conversion of 2- oxoglutarate to succinyl-CoA and CO(2) (By similarity). The 2- oxoglutarate dehydrogenase complex is mainly active in the mitochondrion. A fraction of the 2-oxoglutarate dehydrogenase complex also localizes in the nucleus and is required for lysine succinylation of histones: associates with KAT2A on chromatin and provides succinyl- CoA to histone succinyltransferase KAT2A (By similarity).
kmeans	3	Purple	59	Erp29	10116.ENSRNOP00000001822	Endoplasmic reticulum resident protein 29; Does not seem to be a disulfide isomerase. Plays an important role in the processing of secretory proteins within the endoplasmic reticulum (ER), possibly by participating in the folding of proteins in the ER
kmeans	3	Purple	59	Gapdh	10116.ENSRNOP00000040878	Glyceraldehyde-3-phosphate dehydrogenase; Has both glyceraldehyde-3-phosphate dehydrogenase and nitrosylase activities, thereby playing a role in glycolysis and nuclear functions, respectively. Glyceraldehyde-3-phosphate dehydrogenase is a key enzyme in glycolysis that catalyzes the first step of the pathway by converting D-glyceraldehyde 3- phosphate (G3P) into 3-phospho-D-glyceroyl phosphate. Modulates the organization and assembly of the cytoskeleton. Facilitates the CHP1-dependent microtubule and membrane associations through its ability to stimulate the binding of CHP1 to microtub [...]
kmeans	3	Purple	59	Gcsh	10116.ENSRNOP00000015967	Glycine cleavage system H protein, mitochondrial; The glycine cleavage system catalyzes the degradation of glycine. The H protein (GCSH) shuttles the methylamine group of glycine from the P protein (GLDC) to the T protein (GCST) (By similarity); Belongs to the GcvH family
kmeans	3	Purple	59	Glud1	10116.ENSRNOP00000013789	Glutamate dehydrogenase 1, mitochondrial; Mitochondrial glutamate dehydrogenase that converts L- glutamate into alpha-ketoglutarate. Plays a key role in glutamine anaplerosis by producing alpha-ketoglutarate, an important intermediate in the tricarboxylic acid cycle (By similarity). May be involved in learning and memory reactions by increasing the turnover of the excitatory neurotransmitter glutamate; Belongs to the Glu/Leu/Phe/Val dehydrogenases family
kmeans	3	Purple	59	Got1	10116.ENSRNOP00000022309	Aspartate aminotransferase, cytoplasmic; Biosynthesis of L-glutamate from L-aspartate or L- cysteine. Important regulator of levels of glutamate, the major excitatory neurotransmitter of the vertebrate central nervous system. Acts as a scavenger of glutamate in brain neuroprotection. The aspartate aminotransferase activity is involved in hepatic glucose synthesis during development and in adipocyte glyceroneogenesis. Using L-cysteine as substrate, regulates levels of mercaptopyruvate, an important source of hydrogen sulfide. Mercaptopyruvate is converted into H(2)S via the action of 3- [...]
kmeans	3	Purple	59	Got2	10116.ENSRNOP00000015956	Aspartate aminotransferase, mitochondrial; Catalyzes the irreversible transamination of the L- tryptophan metabolite L-kynurenine to form kynurenic acid (KA). Plays a key role in amino acid metabolism. Important for metabolite exchange between mitochondria and cytosol. Facilitates cellular uptake of long-chain free fatty acids (By similarity); Belongs to the class-I pyridoxal-phosphate-dependent aminotransferase family
kmeans	3	Purple	59	Gpi	10116.ENSRNOP00000029515	Glucose-6-phosphate isomerase; Besides it's role as a glycolytic enzyme, mammalian GPI can function as a tumor-secreted cytokine and an angiogenic factor (AMF) that stimulates endothelial cell motility. GPI is also a neurotrophic factor (Neuroleukin) for spinal and sensory neurons (By similarity)
kmeans	3	Purple	59	Idh2	10116.ENSRNOP00000019059	Isocitrate dehydrogenase [NADP], mitochondrial; Plays a role in intermediary metabolism and energy production. It may tightly associate or interact with the pyruvate dehydrogenase complex (By similarity)
kmeans	3	Purple	59	LOC500959	10116.ENSRNOP00000020647	Triosephosphate isomerase is an extremely efficient metabolic enzyme that catalyzes the interconversion between dihydroxyacetone phosphate (DHAP) and D-glyceraldehyde-3-phosphate (G3P) in glycolysis and gluconeogenesis
kmeans	3	Purple	59	Lamtor5	10116.ENSRNOP00000024473	Hepatitis B virus x interacting protein (Predicted), isoform CRA_a; Late endosomal/lysosomal adaptor, MAPK and MTOR activator 5
kmeans	3	Purple	59	Ldha	10116.ENSRNOP00000017468	Lactate dehydrogenase A

kmeans	3	Purple	59	Ldhb	10116.ENSRNOP00000017965	Lactate dehydrogenase B; Belongs to the LDH/MDH superfamily. LDH family
kmeans	3	Purple	59	Ldhd	10116.ENSRNOP00000046399	Lactate dehydrogenase D
kmeans	3	Purple	59	Lman1	10116.ENSRNOP00000035966	Mannose-specific lectin. May recognize sugar residues of glycoproteins, glycolipids, or glycosylphosphatidyl inositol anchors and may be involved in the sorting or recycling of proteins, lipids, or both. The LMAN1-MCFD2 complex forms a specific cargo receptor for the ER-to-Golgi transport of selected proteins (By similarity).
kmeans	3	Purple	59	MGC94335	10116.ENSRNOP00000021533	m-AAA protease-interacting protein 1, mitochondrial; Promotes sorting of SMDT1/EMRE in mitochondria by ensuring its maturation. Interacts with the transit peptide region of SMDT1/EMRE precursor protein in the mitochondrial matrix, leading to protect it against protein degradation by YME1L1, thereby ensuring SMDT1/EMRE maturation by the mitochondrial processing peptidase (PMPCA and PMPCB)
kmeans	3	Purple	59	Mdh1	10116.ENSRNOP00000011429	Malate dehydrogenase, cytoplasmic; Malate dehydrogenase 1, NAD (soluble)
kmeans	3	Purple	59	Mdh2	10116.ENSRNOP00000001958	Malate dehydrogenase 2, NAD (mitochondrial); Belongs to the LDH/MDH superfamily. MDH type 1 family
kmeans	3	Purple	59	Me3	10116.ENSRNOP00000023329	Malic enzyme 3, NADP(+)-dependent, mitochondrial
kmeans	3	Purple	59	Mospd1	10116.ENSRNOP00000003192	Plays a role in differentiation and/or proliferation of mesenchymal stem cells. Proposed to be involved in epithelial-to- mesenchymal transition (EMT). However, another study suggests that it is not required for EMT or stem cell self-renewal and acts during later stages of differentiation
kmeans	3	Purple	59	Mpst	10116.ENSRNOP00000000201	3-mercaptopyruvate sulfurtransferase; Transfer of a sulfur ion to cyanide or to other thiol compounds. Also has weak rhodanese activity. Detoxifies cyanide and is required for thiosulfate biosynthesis. Acts as an antioxidant. In combination with cysteine aminotransferase (CAT), contributes to the catabolism of cysteine and is an important producer of hydrogen sulfide in the brain, retina and vascular endothelial cells. Hydrogen sulfide H(2)S is an important synaptic modulator, signaling molecule, smooth muscle contractor and neuroprotectant. Its production by the 3MST/CAT pathway is re [...]
kmeans	3	Purple	59	Mrc1	10116.ENSRNOP00000024736	Mannose receptor, C type 1
kmeans	3	Purple	59	Pc	10116.ENSRNOP00000026316	Pyruvate carboxylase, mitochondrial; Pyruvate carboxylase catalyzes a 2-step reaction, involving the ATP-dependent carboxylation of the covalently attached biotin in the first step and the transfer of the carboxyl group to pyruvate in the second. Catalyzes in a tissue specific manner, the initial reactions of glucose (liver, kidney) and lipid (adipose tissue, liver, brain) synthesis from pyruvate
kmeans	3	Purple	59	Pdhb	10116.ENSRNOP00000010545	Pyruvate dehydrogenase E1 component subunit beta, mitochondrial; The pyruvate dehydrogenase complex catalyzes the overall conversion of pyruvate to acetyl-CoA and CO(2), and thereby links the glycolytic pathway to the tricarboxylic cycle
kmeans	3	Purple	59	Pdk2	10116.ENSRNOP00000005641	Kinase that plays a key role in the regulation of glucose and fatty acid metabolism and homeostasis via phosphorylation of the pyruvate dehydrogenase subunits PDHA1 and PDHA2. This inhibits pyruvate dehydrogenase activity, and thereby regulates metabolite flux through the tricarboxylic acid cycle, down-regulates aerobic respiration and inhibits the formation of acetyl-coenzyme A from pyruvate. Inhibition of pyruvate dehydrogenase decreases glucose utilization and increases fat metabolism. Mediates cellular responses to insulin. Plays an important role in maintaining normal blood glucos [...]
kmeans	3	Purple	59	Pdk4	10116.ENSRNOP00000012759	Kinase that plays a key role in regulation of glucose and fatty acid metabolism and homeostasis via phosphorylation of the pyruvate dehydrogenase subunits PDHA1 and PDHA2. This inhibits pyruvate dehydrogenase activity, and thereby regulates metabolite flux through the tricarboxylic acid cycle, down-regulates aerobic respiration and inhibits the formation of acetyl-coenzyme A from pyruvate. Inhibition of pyruvate dehydrogenase decreases glucose utilization and increases fat metabolism in response to prolonged fasting and starvation. Plays an important role in maintaining normal blood gl [...]
kmeans	3	Purple	59	Pfkm	10116.ENSRNOP00000013374	Catalyzes the phosphorylation of D-fructose 6-phosphate to fructose 1,6-bisphosphate by ATP, the first committing step of glycolysis

kmeans	3	Purple	59	Pgk1	10116.ENSRNOP00000003390	Phosphoglycerate kinase 1; In addition to its role as a glycolytic enzyme, it seems that PGK-1 acts as a polymerase alpha cofactor protein (primer recognition protein). May play a role in sperm motility
kmeans	3	Purple	59	Pgm1	10116.ENSRNOP00000013785	This enzyme participates in both the breakdown and synthesis of glucose
kmeans	3	Purple	59	Phb	10116.ENSRNOP00000066502	Prohibitin; Prohibitin inhibits DNA synthesis. It has a role in regulating proliferation. As yet it is unclear if the protein or the mRNA exhibits this effect. May play a role in regulating mitochondrial respiration activity and in aging
kmeans	3	Purple	59	Phb2	10116.ENSRNOP00000017472	Prohibitin-2; Acts as a mediator of transcriptional repression by nuclear hormone receptors via recruitment of histone deacetylases. Functions as an estrogen receptor (ER)-selective coregulator that potentiates the inhibitory activities of antiestrogens and represses the activity of estrogens. Competes with NCOA1 for modulation of ER transcriptional activity. Probably involved in regulating mitochondrial respiration activity and in aging (By similarity)
kmeans	3	Purple	59	Pmpca	10116.ENSRNOP00000037642	Substrate recognition and binding subunit of the essential mitochondrial processing protease (MPP), which cleaves the mitochondrial sequence off newly imported precursors proteins.
kmeans	3	Purple	59	Rras2	10116.ENSRNOP00000017199	Related RAS viral (r-ras) oncogene homolog 2
kmeans	3	Purple	59	Rtn4ip1	10116.ENSRNOP00000000304	Similar to NOGO-interacting mitochondrial protein (Predicted); Reticulon 4 interacting protein 1
kmeans	3	Purple	59	Sdha	10116.ENSRNOP00000018336	Succinate dehydrogenase [ubiquinone] flavoprotein subunit, mitochondrial; Flavoprotein (FP) subunit of succinate dehydrogenase (SDH) that is involved in complex II of the mitochondrial electron transport chain and is responsible for transferring electrons from succinate to ubiquinone (coenzyme Q). Can act as a tumor suppressor; Belongs to the FAD-dependent oxidoreductase 2 family. FRD/SDH subfamily
kmeans	3	Purple	59	Sdhb	10116.ENSRNOP00000010593	Succinate dehydrogenase [ubiquinone] iron-sulfur subunit, mitochondrial; Iron-sulfur protein (IP) subunit of succinate dehydrogenase (SDH) that is involved in complex II of the mitochondrial electron transport chain and is responsible for transferring electrons from succinate to ubiquinone (coenzyme Q)
kmeans	3	Purple	59	Sdhc	10116.ENSRNOP00000004228	Succinate dehydrogenase complex, subunit C, integral membrane protein
kmeans	3	Purple	59	Sgca	10116.ENSRNOP00000005381	Sarcoglycan, alpha (dystrophin-associated glycoprotein)
kmeans	3	Purple	59	Sgcb	10116.ENSRNOP00000002921	Sarcoglycan, beta (dystrophin-associated glycoprotein)
kmeans	3	Purple	59	Sgcg	10116.ENSRNOP00000019639	Sarcoglycan, gamma (dystrophin-associated glycoprotein)
kmeans	3	Purple	59	Spg7	10116.ENSRNOP00000048848	Paraplegin; ATP-dependent zinc metalloprotease. Plays a role in the formation and regulation of the mitochondrial permeability transition pore (mPTP) and its proteolytic activity is dispensable for this function; In the N-terminal section; belongs to the AAA ATPase family
kmeans	3	Purple	59	Suclg2	10116.ENSRNOP00000060990	Succinate--CoA ligase [GDP-forming] subunit beta, mitochondrial; GTP-specific succinyl-CoA synthetase functions in the citric acid cycle (TCA), coupling the hydrolysis of succinyl-CoA to the synthesis of GTP and thus represents the only step of substrate-level phosphorylation in the TCA. The beta subunit provides nucleotide specificity of the enzyme and binds the substrate succinate, while the binding sites for coenzyme A and phosphate are found in the alpha subunit
kmeans	3	Purple	59	Tecr	10116.ENSRNOP00000035416	Very-long-chain enoyl-CoA reductase; Catalyzes the last of the four reactions of the long- chain fatty acids elongation cycle. This endoplasmic reticulum-bound enzymatic process, allows the addition of 2 carbons to the chain of long- and very long-chain fatty acids/VLCFAs per cycle. This enzyme reduces the trans-2,3-enoyl-CoA fatty acid intermediate to an acyl-CoA that can be further elongated by entering a new cycle of elongation. Thereby, it participates in the production of VLCFAs of different chain lengths that are involved in multiple biological processes as precursors of membran [...]

kmeans	3	Purple	59	Ugp2	10116.ENSRNOP00000063786	UDP-glucose pyrophosphorylase 2, isoform CRA_b; UDP-glucose pyrophosphorylase 2
kmeans	3	Purple	59	Unc45b	10116.ENSRNOP00000068065	Unc-45 homolog B (C. elegans)
kmeans	3	Purple	59	Zak	10116.ENSRNOP00000002067	Sterile alpha motif and leucine zipper containing kinase AZK
kmeans	4	Red	102	Abca8a	10116.ENSRNOP00000005665	ATP-binding cassette, subfamily A (ABC1), member 8a
kmeans	4	Red	102	Abcb7	10116.ENSRNOP00000003739	Could be involved in the transport of heme from the mitochondria to the cytosol. Plays a central role in the maturation of cytosolic iron-sulfur (Fe/S) cluster-containing proteins.
kmeans	4	Red	102	Acsf2	10116.ENSRNOP00000004673	Acyl-CoA synthetase family member 2, mitochondrial; Acyl-CoA synthases catalyze the initial reaction in fatty acid metabolism, by forming a thioester with CoA. Has some preference toward medium-chain substrates. Plays a role in adipocyte differentiation (By similarity)
kmeans	4	Red	102	Actn1	10116.ENSRNOP00000061058	Alpha-actinin-1; F-actin cross-linking protein which is thought to anchor actin to a variety of intracellular structures. This is a bundling protein (By similarity)
kmeans	4	Red	102	Actn2	10116.ENSRNOP00000024098	Actinin alpha 2
kmeans	4	Red	102	Actn4	10116.ENSRNOP00000027773	Alpha-actinin-4; F-actin cross-linking protein which is thought to anchor actin to a variety of intracellular structures. This is a bundling protein. Probably involved in vesicular trafficking via its association with the CART complex. The CART complex is necessary for efficient transferrin receptor recycling but not for EGFR degradation. Involved in tight junction assembly in epithelial cells probably through interaction with MICALL2. Links MICALL2 to the actin cytoskeleton and recruits it to the tight junctions. May also function as a transcriptional coactivator, stimulating transcri [...]
kmeans	4	Red	102	Add2	10116.ENSRNOP00000021491	Membrane-cytoskeleton-associated protein that promotes the assembly of the spectrin-actin network. Binds to the erythrocyte membrane receptor SLC2A1/GLUT1 and may therefore provide a link between the spectrin cytoskeleton to the plasma membrane. Binds to calmodulin. Calmodulin binds preferentially to the beta subunit (By similarity).
kmeans	4	Red	102	Agps	10116.ENSRNOP00000002111	Catalyzes the exchange of the acyl chain in acyl- dihydroxyacetonephosphate (acyl-DHAP) for a long chain fatty alcohol, yielding the first ether linked intermediate, i.e. alkyl- dihydroxyacetonephosphate (alkyl-DHAP), in the pathway of ether lipid biosynthesis
kmeans	4	Red	102	Ak3	10116.ENSRNOP00000020744	Involved in maintaining the homeostasis of cellular nucleotides by catalyzing the interconversion of nucleoside phosphates. Has GTP:AMP phosphotransferase and ITP:AMP phosphotransferase activities
kmeans	4	Red	102	Alb	10116.ENSRNOP00000003921	Serum albumin; Serum albumin, the main protein of plasma, has a good binding capacity for water, Ca(2+), Na(+), K(+), fatty acids, hormones, bilirubin and drugs. Its main function is the regulation of the colloidal osmotic pressure of blood. Major zinc transporter in plasma, typically binds about 80% of all plasma zinc; Belongs to the ALB/AFP/VDB family
kmeans	4	Red	102	Anxa2	10116.ENSRNOP00000038428	Annexin A2; Calcium-regulated membrane-binding protein whose affinity for calcium is greatly enhanced by anionic phospholipids. It binds two calcium ions with high affinity. May be involved in heat-stress response. Inhibits PCSK9-enhanced LDLR degradation, probably reduces PCSK9 protein levels via a translational mechanism but also competes with LDLR for binding with PCSK9
kmeans	4	Red	102	Anxa5	10116.ENSRNOP00000019552	This protein is an anticoagulant protein that acts as an indirect inhibitor of the thromboplastin-specific complex, which is involved in the blood coagulation cascade

kmeans	4	Red	102	Arhgdia	10116.ENSRNOP00000051840	Rho GDP-dissociation inhibitor 1; Controls Rho proteins homeostasis. Regulates the GDP/GTP exchange reaction of the Rho proteins by inhibiting the dissociation of GDP from them, and the subsequent binding of GTP to them. Retains Rho proteins such as CDC42, RAC1 and RHOA in an inactive cytosolic pool, regulating their stability and protecting them from degradation. Actively involved in the recycling and distribution of activated Rho GTPases in the cell, mediates extraction from membranes of both inactive and activated molecules due its exceptionally high affinity for prenylated forms. T [...]
kmeans	4	Red	102	Atp6v0a1	10116.ENSRNOP00000052113	Required for assembly and activity of the vacuolar ATPase. Potential role in differential targeting and regulation of the enzyme for a specific organelle (By similarity)
kmeans	4	Red	102	Atp6v1a	10116.ENSRNOP00000002727	ATPase, H+ transporting, lysosomal V1 subunit A
kmeans	4	Red	102	Bcam	10116.ENSRNOP00000047809	Basal cell adhesion molecule; Laminin alpha-5 receptor. May mediate intracellular signaling (By similarity)
kmeans	4	Red	102	C3	10116.ENSRNOP00000066885	C3 plays a central role in the activation of the complement system. Its processing by C3 convertase is the central reaction in both classical and alternative complement pathways. After activation C3b can bind covalently, via its reactive thioester, to cell surface carbohydrates or immune aggregates. [C3-beta-c]: Acts as a chemoattractant for neutrophils in chronic inflammation
kmeans	4	Red	102	Cam	10116.ENSRNOP00000022603	Calmodulin 1; Calmodulin mediates the control of a large number of enzymes, ion channels, aquaporins and other proteins through calcium-binding. Among the enzymes to be stimulated by the calmodulin-calcium complex are a number of protein kinases and phosphatases. Together with CCP110 and centrin, is involved in a genetic pathway that regulates the centrosome cycle and progression through cytokinesis. Mediates calcium-dependent inactivation of CACNA1C. Positively regulates calcium-activated potassium channel activity of KCNN2
kmeans	4	Red	102	Camk2d	10116.ENSRNOP00000016026	Calcium/calmodulin-dependent protein kinase type II subunit delta; Calcium/calmodulin-dependent protein kinase involved in the regulation of Ca(2+) homeostatis and excitation-contraction coupling (ECC) in heart by targeting ion channels, transporters and accessory proteins involved in Ca(2+) influx into the myocyte, Ca(2+) release from the sarcoplasmic reticulum (SR), SR Ca(2+) uptake and Na(+) and K(+) channel transport. Targets also transcription factors and signaling molecules to regulate heart function. In its activated form, is involved in the pathogenesis of dilated cardiomyopath [...]
kmeans	4	Red	102	Capn2	10116.ENSRNOP00000046509	Calpain-2 catalytic subunit; Calcium-regulated non-lysosomal thiol-protease which catalyze limited proteolysis of substrates involved in cytoskeletal remodeling and signal transduction. Proteolytically cleaves MYOC at 'Arg-226'. Proteolytically cleaves CPEB3 following neuronal stimulation which abolishes CPEB3 translational repressor activity, leading to translation of CPEB3 target mRNAs; Belongs to the peptidase C2 family
kmeans	4	Red	102	Cav1	10116.ENSRNOP00000009253	May act as a scaffolding protein within caveolar membranes. Forms a stable heterooligomeric complex with CAV2 that targets to lipid rafts and drives caveolae formation. Mediates the recruitment of CAVIN proteins (CAVIN1/2/3/4) to the caveolae (By similarity). Interacts directly with G-protein alpha subunits and can functionally regulate their activity (By similarity). Involved in the costimulatory signal essential for T-cell receptor (TCR)-mediated T-cell activation. Its binding to DPP4 induces T-cell proliferation and NF-kappa-B activation in a T-cell receptor/CD3-dependent manner (By [...]
kmeans	4	Red	102	Cd34	10116.ENSRNOP00000010148	Hematopoietic progenitor cell antigen CD34-like
kmeans	4	Red	102	Cdh13	10116.ENSRNOP00000068368	Cadherin 13
kmeans	4	Red	102	Ctnnb1	10116.ENSRNOP00000026016	Catenin beta-1; Key downstream component of the canonical Wnt signaling pathway. In the absence of Wnt, forms a complex with AXIN1, AXIN2, APC, CSNK1A1 and GSK3B that promotes phosphorylation on N-terminal Ser and Thr residues and ubiquitination of CTNNB1 via BTRC and its subsequent degradation by the proteasome. In the presence of Wnt ligand, CTNNB1 is not ubiquitinated and accumulates in the nucleus, where it acts as a coactivator for transcription factors of the TCF/LEF family, leading to activate Wnt responsive genes. Involved in the regulation of cell adhesion, as component of an [...]
kmeans	4	Red	102	Ctsb	10116.ENSRNOP00000014178	Thiol protease which is believed to participate in intracellular degradation and turnover of proteins . Cleaves matrix extracellular phosphoglycoprotein MEPE (By similarity). Involved in the solubilization of cross-linked TG/thyroglobulin in the thyroid follicle lumen (By similarity). Has also been implicated in tumor invasion and metastasis (By similarity).
kmeans	4	Red	102	Ctsd	10116.ENSRNOP00000027407	Acid protease active in intracellular protein breakdown. Plays a role in APP processing following cleavage and activation by ADAM30 which leads to APP degradation

kmeans	4	Red	102	Cyb5r1	10116.ENSRNOP00000057400	NADH-cytochrome b5 reductases are involved in desaturation and elongation of fatty acids, cholesterol biosynthesis, drug metabolism, and, in erythrocyte, methemoglobin reduction.
kmeans	4	Red	102	Dag1	10116.ENSRNOP00000026327	Dystroglycan 1 (dystrophin-associated glycoprotein 1)
kmeans	4	Red	102	Dnm2	10116.ENSRNOP00000060296	Dynamin-2; Microtubule-associated force-producing protein involved in producing microtubule bundles and able to bind and hydrolyze GTP. Plays a role in the regulation of neuron morphology, axon growth and formation of neuronal growth cones. Plays an important role in vesicular trafficking processes, in particular endocytosis. Involved in cytokinesis. Regulates maturation of apoptotic cell corpse- containing phagosomes by recruiting PIK3C3 to the phagosome membrane (By similarity); Belongs to the TRAFAC class dynamin-like GTPase superfamily. Dynamin/Fzo/YdjA family
kmeans	4	Red	102	Dpysl2	10116.ENSRNOP00000012996	Dihydropyrimidinase-related protein 2; Plays a role in neuronal development and polarity, as well as in axon growth and guidance, neuronal growth cone collapse and cell migration. Necessary for signaling by class 3 semaphorins and subsequent remodeling of the cytoskeleton. May play a role in endocytosis (By similarity); Belongs to the metallo-dependent hydrolases superfamily. Hydantoinase/dihydropyrimidinase family
kmeans	4	Red	102	Ehd2	10116.ENSRNOP00000017353	EH domain-containing protein 2; ATP- and membrane-binding protein that controls membrane reorganization/tubulation upon ATP hydrolysis. Plays a role in membrane trafficking between the plasma membrane and endosomes. Important for the internalization of GLUT4. Required for fusion of myoblasts to skeletal muscle myotubes. Required for normal translocation of FER1L5 to the plasma membrane. Regulates the equilibrium between cell surface-associated and cell surface- dissociated caveolae by constraining caveolae at the cell membrane
kmeans	4	Red	102	Eno1	10116.ENSRNOP00000024106	Alpha-enolase; Multifunctional enzyme that, as well as its role in glycolysis, plays a part in various processes such as growth control, hypoxia tolerance and allergic responses. May also function in the intravascular and pericellular fibrinolytic system due to its ability to serve as a receptor and activator of plasminogen on the cell surface of several cell-types such as leukocytes and neurons. Stimulates immunoglobulin production; Belongs to the enolase family
kmeans	4	Red	102	Ep300	10116.ENSRNOP00000000206	E1A binding protein p300
kmeans	4	Red	102	Epb42	10116.ENSRNOP00000015556	Erythrocyte membrane protein band 4.2
kmeans	4	Red	102	Fam129b	10116.ENSRNOP00000021689	Niban-like protein 1; May play a role in apoptosis suppression
kmeans	4	Red	102	Fga	10116.ENSRNOP00000060007	Cleaved by the protease thrombin to yield monomers which, together with fibrinogen beta (FGB) and fibrinogen gamma (FGG), polymerize to form an insoluble fibrin matrix. Fibrin has a major function in hemostasis as one of the primary components of blood clots. In addition, functions during the early stages of wound repair to stabilize the lesion and guide cell migration during re- epithelialization. Was originally thought to be essential for platelet aggregation, based on in vitro studies using anticoagulated blood. However, subsequent studies have shown that it is not absolutely requir [...]
kmeans	4	Red	102	Flna	10116.ENSRNOP00000062690	Filamin, alpha (Predicted), isoform CRA_a; Filamin A, alpha
kmeans	4	Red	102	Flnb	10116.ENSRNOP00000061198	Filamin, beta (Predicted); Filamin B, beta
kmeans	4	Red	102	Flnc	10116.ENSRNOP00000027237	Filamin-C; Muscle-specific filamin, which plays a central role in muscle cells, probably by functioning as a large actin-cross- linking protein. May be involved in reorganizing the actin cytoskeleton in response to signaling events, and may also display structural functions at the Z lines in muscle cells. Critical for normal myogenesis and for maintaining the structural integrity of the muscle fibers
kmeans	4	Red	102	Fn1	10116.ENSRNOP00000019772	Fibronectins bind cell surfaces and various compounds including collagen, fibrin, heparin, DNA, and actin. Fibronectins are involved in cell adhesion, cell motility, opsonization, wound healing, and maintenance of cell shape. Involved in osteoblast compaction through the fibronectin fibrillogenesis cell-mediated matrix assembly process, essential for osteoblast mineralization. Participates in the regulation of type I collagen deposition by osteoblasts (By similarity)
kmeans	4	Red	102	Gdi2	10116.ENSRNOP00000024952	Rab GDP dissociation inhibitor beta; Regulates the GDP/GTP exchange reaction of most Rab proteins by inhibiting the dissociation of GDP from them, and the subsequent binding of GTP to them

kmeans	4	Red	102	Glg1	10116.ENSRNOP00000025570	Binds fibroblast growth factor and E-selectin (cell-adhesion lectin on endothelial cells mediating the binding of neutrophils).
kmeans	4	Red	102	Gna13	10116.ENSRNOP00000051938	Guanine nucleotide-binding protein subunit alpha-13; Guanine nucleotide-binding proteins (G proteins) are involved as modulators or transducers in various transmembrane signaling systems. Activates effector molecule RhoA by binding and activating RhoGEFs (ARHGEF1/p115RhoGEF, ARHGEF11/PDZ-RhoGEF and ARHGEF12/LARG) (By similarity). GNA13- dependent Rho signaling subsequently regulates transcription factor AP-1 (activating protein-1) (By similarity). Promotes tumor cell invasion and metastasis by activating RhoA/ROCK signaling pathway (By similarity). Inhibits CDH1-mediated cell adhesion [...]
kmeans	4	Red	102	Hba1	10116.ENSRNOP00000044233	Hemoglobin subunit alpha-1/2; Involved in oxygen transport from the lung to the various peripheral tissues; Belongs to the globin family
kmeans	4	Red	102	Hint2	10116.ENSRNOP00000021387	Histidine triad nucleotide binding protein 2
kmeans	4	Red	102	Hpx	10116.ENSRNOP00000024710	Hemopexin; Binds heme and transports it to the liver for breakdown and iron recovery, after which the free hemopexin returns to the circulation
kmeans	4	Red	102	Itga6	10116.ENSRNOP00000039474	Integrin, alpha 6, isoform CRA_a; Integrin, alpha 6
kmeans	4	Red	102	Itga7	10116.ENSRNOP00000058370	Integrin alpha-7; Integrin alpha-7/beta-1 is the primary laminin receptor on skeletal myoblasts and adult myofibers. During myogenic differentiation, it may induce changes in the shape and mobility of myoblasts, and facilitate their localization at laminin-rich sites of secondary fiber formation. Involved in the maintenance of the myofibers cytoarchitecture as well as for their anchorage, viability and functional integrity. Required to promote contractile phenotype acquisition in differentiated airway smooth muscle (ASM) cells (By similarity). Acts as Schwann cell receptor for laminin- [...]
kmeans	4	Red	102	Lama2	10116.ENSRNOP00000014917	Laminin, alpha 2
kmeans	4	Red	102	Lamp1	10116.ENSRNOP00000026580	Lysosome-associated membrane glycoprotein 1; Presents carbohydrate ligands to selectins. Also implicated in tumor cell metastasis
kmeans	4	Red	102	Ldb3	10116.ENSRNOP00000067940	LIM domain binding 3
kmeans	4	Red	102	Lgals1	10116.ENSRNOP00000013538	Galectin-1; Lectin that binds beta-galactoside and a wide array of complex carbohydrates. Plays a role in regulating apoptosis, cell proliferation and cell differentiation. Inhibits CD45 protein phosphatase activity and therefore the dephosphorylation of Lyn kinase. Strong inducer of T-cell apoptosis
kmeans	4	Red	102	Lrp1	10116.ENSRNOP00000034210	Low density lipoprotein receptor-related protein 1
kmeans	4	Red	102	Lrrc57	10116.ENSRNOP00000012636	Leucine-rich repeat-containing protein 57-like
kmeans	4	Red	102	Mgst3	10116.ENSRNOP00000005719	Microsomal glutathione S-transferase 3
kmeans	4	Red	102	Msn	10116.ENSRNOP00000068359	Ezrin-radixin-moesin (ERM) family protein that connects the actin cytoskeleton to the plasma membrane and thereby regulates the structure and function of specific domains of the cell cortex. Tethers actin filaments by oscillating between a resting and an activated state providing transient interactions between moesin and the actin cytoskeleton. Once phosphorylated on its C-terminal threonine, moesin is activated leading to interaction with F-actin and cytoskeletal rearrangement. These rearrangements regulate many cellular processes, including cell shape determination, membrane transpor [...]

kmeans	4	Red	102	Mvp	10116.ENSRNOP00000027360	Major vault protein; Required for normal vault structure. Vaults are multi- subunit structures that may act as scaffolds for proteins involved in signal transduction. Vaults may also play a role in nucleo- cytoplasmic transport. Down-regulates IFNG-mediated STAT1 signaling and subsequent activation of JAK. Down-regulates SRC activity and signaling through MAP kinases (By similarity)
kmeans	4	Red	102	Myh14	10116.ENSRNOP00000027132	Myosin, heavy chain 14, non-muscle; Belongs to the TRAFAC class myosin-kinesin ATPase superfamily. Myosin family
kmeans	4	Red	102	Myh7	10116.ENSRNOP00000024186	Myosins are actin-based motor molecules with ATPase activity essential for muscle contraction. Forms regular bipolar thick filaments that, together with actin thin filaments, constitute the fundamental contractile unit of skeletal and cardiac muscle.
kmeans	4	Red	102	Myl2	10116.ENSRNOP00000039707	Contractile protein that plays a role in heart development and function (By similarity). Following phosphorylation, plays a role in cross-bridge cycling kinetics and cardiac muscle contraction by increasing myosin lever arm stiffness and promoting myosin head diffusion; as a consequence of the increase in maximum contraction force and calcium sensitivity of contraction force. These events altogether slow down myosin kinetics and prolong duty cycle resulting in accumulated myosins being cooperatively recruited to actin binding sites to sustain thin filament activation as a means to fine [...]
kmeans	4	Red	102	Napa	10116.ENSRNOP00000002044	Alpha-soluble NSF attachment protein; Required for vesicular transport between the endoplasmic reticulum and the Golgi apparatus. Together with GNA12 promotes CDH5 localization to plasma membrane
kmeans	4	Red	102	Ncam1	10116.ENSRNOP00000048442	This protein is a cell adhesion molecule involved in neuron- neuron adhesion, neurite fasciculation, outgrowth of neurites, etc
kmeans	4	Red	102	Ndrg2	10116.ENSRNOP00000042949	Protein NDRG2; Contributes to the regulation of the Wnt signaling pathway. Down-regulates CTNNB1-mediated transcriptional activation of target genes, such as CCND1, and may thereby act as tumor suppressor (By similarity). May be involved in dendritic cell and neuron differentiation
kmeans	4	Red	102	Nt5e	10116.ENSRNOP00000015057	Hydrolyzes extracellular nucleotides into membrane permeable nucleosides
kmeans	4	Red	102	Nucb1	10116.ENSRNOP00000028390	Nucleobindin-1; Major calcium-binding protein of the Golgi. May have a role in calcium homeostasis
kmeans	4	Red	102	Pbxip1	10116.ENSRNOP00000028058	Pre-B-cell leukemia transcription factor-interacting protein 1; Regulator of pre-B-cell leukemia transcription factors (BPXs) function. Inhibits the binding of PBX1-HOX complex to DNA and blocks the transcriptional activity of E2A-PBX1. Tethers estrogen receptor-alpha (ESR1) to microtubules and allows them to influence estrogen receptors-alpha signaling (By similarity)
kmeans	4	Red	102	Pebp1	10116.ENSRNOP00000001500	Phosphatidylethanolamine-binding protein 1; Binds ATP, opioids and phosphatidylethanolamine. Has lower affinity for phosphatidylinositol and phosphatidylcholine. Serine protease inhibitor which inhibits thrombin, neuropsin and chymotrypsin but not trypsin, tissue type plasminogen activator and elastase (By similarity). Inhibits the kinase activity of RAF1 by inhibiting its activation and by dissociating the RAF1/MEK complex and acting as a competitive inhibitor of MEK phosphorylation (By similarity); Belongs to the phosphatidylethanolamine-binding protein family
kmeans	4	Red	102	Pecam1	10116.ENSRNOP00000044403	Platelet endothelial cell adhesion molecule; Cell adhesion molecule which is required for leukocyte transendothelial migration (TEM) under most inflammatory conditions. Tyr-660 plays a critical role in TEM and is required for efficient trafficking of PECAM1 to and from the lateral border recycling compartment (LBRC) and is also essential for the LBRC membrane to be targeted around migrating leukocytes. Heterophilic interaction with CD177 plays a role in transendothelial migration of neutrophils. Homophilic ligation of PECAM1 prevents macrophage- mediated phagocytosis of neighboring via [...]
kmeans	4	Red	102	Plg	10116.ENSRNOP00000023370	Plasminogen; Plasmin dissolves the fibrin of blood clots and acts as a proteolytic factor in a variety of other processes including embryonic development, tissue remodeling, tumor invasion, and inflammation. In ovulation, weakens the walls of the Graafian follicle. It activates the urokinase-type plasminogen activator, collagenases and several complement zymogens, such as C1 and C5. Cleavage of fibronectin and laminin leads to cell detachment and apoptosis. Also cleaves fibrin, thrombospondin and von Willebrand factor. Its role in tissue remodeling and tumor invasion may be modulated b [...]
kmeans	4	Red	102	Poldip2	10116.ENSRNOP00000013100	Polymerase (DNA-directed), delta interacting protein 2
kmeans	4	Red	102	Popdc2	10116.ENSRNOP00000003999	Popeye domain containing 2

kmeans	4	Red	102	Ppapdc 3	10116.ENS RNOP00000 013507	Inactive phospholipid phosphatase 7; Plays a role as negative regulator of myoblast differentiation, in part through effects on MTOR signaling. Has no detectable enzymatic activity (By similarity)
kmeans	4	Red	102	Ptrf	10116.ENS RNOP00000 026783	Plays an important role in caveolae formation and organization. Essential for the formation of caveolae in all tissues. Core component of the CAVIN complex which is essential for recruitment of the complex to the caveolae in presence of calveolin-1 (CAV1). Essential for normal oligomerization of CAV1. Promotes ribosomal transcriptional activity in response to metabolic challenges in the adipocytes and plays an important role in the formation of the ribosomal transcriptional loop. Dissociates transcription complexes paused by DNA-bound TTF1, thereby releasing both RNA polymerase I and p [...]
kmeans	4	Red	102	RGD13 11756	10116.ENS RNOP00000 005903	Similar to hypothetical protein FLJ20950
kmeans	4	Red	102	Rab7a	10116.ENS RNOP00000 016432	Ras-related protein Rab-7a; Key regulator in endo-lysosomal trafficking. Governs early-to-late endosomal maturation, microtubule minus-end as well as plus-end directed endosomal migration and positioning, and endosome-lysosome transport through different protein-protein interaction cascades (By similarity). Plays a central role, not only in endosomal traffic, but also in many other cellular and physiological events, such as growth-factor-mediated cell signaling, nutrient-transportor mediated nutrient uptake, neurotrophin transport in the axons of neurons and lipid metabolism (By simila [...]
kmeans	4	Red	102	Rap1a	10116.ENS RNOP00000 040409	Ras-related protein Rap-1A; Induces morphological reversion of a cell line transformed by a Ras oncogene. Counteracts the mitogenic function of Ras, at least partly because it can interact with Ras GAPs and RAF in a competitive manner. Together with ITGB1BP1, regulates KRIT1 localization to microtubules and membranes (By similarity). Plays a role in nerve growth factor (NGF)-induced neurite outgrowth. Plays a role in the regulation of embryonic blood vessel formation. Involved in the establishment of basal endothelial barrier function. May be involved in the regulation of the vascular [...]
kmeans	4	Red	102	Rhoa	10116.ENS RNOP00000 066672	Transforming protein RhoA; Regulates a signal transduction pathway linking plasma membrane receptors to the assembly of focal adhesions and actin stress fibers. Involved in a microtubule-dependent signal that is required for the myosin contractile ring formation during cell cycle cytokinesis. Plays an essential role in cleavage furrow formation. Required for the apical junction formation of keratinocyte cell-cell adhesion. May be an activator of PLCE1. Activated by ARHGEF2, which promotes the exchange of GDP for GTP. Essential for the SPATA13-mediated regulation of cell migration and a [...]
kmeans	4	Red	102	Rnh1	10116.ENS RNOP00000 022241	Ribonuclease inhibitor which inhibits RNASE1, RNASE2 and ANG. May play a role in redox homeostasis
kmeans	4	Red	102	Rnpep	10116.ENS RNOP00000 009198	Exopeptidase which selectively removes arginine and/or lysine residues from the N-terminus of several peptide substrates including Arg(0)-Leu-enkephalin, Arg(0)-Met-enkephalin and Arg(-1)-Lys(0)- somatostatin-14. Can hydrolyze leukotriene A4 (LTA-4) into leukotriene B4 (LTB-4)
kmeans	4	Red	102	Rock1	10116.ENS RNOP00000 047378	Protein kinase which is a key regulator of actin cytoskeleton and cell polarity . Involved in regulation of smooth muscle contraction, actin cytoskeleton organization, stress fiber and focal adhesion formation, neurite retraction, cell adhesion and motility via phosphorylation of DAPK3, GFAP, LIMK1, LIMK2, MYL9/MLC2, TPPP, PFN1 and PPP1R12A . Phosphorylates FHOD1 and acts synergistically with it to promote SRC-dependent non-apoptotic plasma membrane blebbing. Phosphorylates JIP3 and regulates the recruitment of JNK to JIP3 upon UVB-induced stress (By similarity). Acts as a suppressor o [...]
kmeans	4	Red	102	S100a 10	10116.ENS RNOP00000 037097	Protein S100-A10; Because S100A10 induces the dimerization of ANXA2/p36, it may function as a regulator of protein phosphorylation in that the ANXA2 monomer is the preferred target (in vitro) of tyrosine- specific kinase
kmeans	4	Red	102	Sec22b	10116.ENS RNOP00000 025327	Vesicle-trafficking protein SEC22b; SNARE involved in targeting and fusion of ER-derived transport vesicles with the Golgi complex as well as Golgi-derived retrograde transport vesicles with the ER
kmeans	4	Red	102	Snta1	10116.ENS RNOP00000 021715	Syntrophin, alpha 1
kmeans	4	Red	102	Syncrip	10116.ENS RNOP00000 048433	Heterogeneous nuclear ribonucleoprotein Q; Heterogenous nuclear ribonucleoprotein (hnRNP) implicated in mRNA processing mechanisms. Component of the CRD- mediated complex that promotes MYC mRNA stability. Is associated in vitro with pre-mRNA, splicing intermediates and mature mRNA protein complexes. Binds to apoB mRNA AU-rich sequences. Part of the APOB mRNA editosome complex and may modulate the postranscriptional C to U RNA-editing of the APOB mRNA through either by binding to A1CF (APOBEC1 complementation factor), to APOBEC1 or to RNA itself. May be involved in translationally coupl [...]
kmeans	4	Red	102	Synj2b p	10116.ENS RNOP00000 009119	Synaptojanin-2-binding protein; Regulates endocytosis of activin type 2 receptor kinases through the Ral/RALBP1-dependent pathway and may be involved in suppression of activin-induced signal transduction

kmeans	4	Red	102	Tagln2	10116.ENSRNOP00000011208	Transgelin-2; Transgelin 2; Belongs to the calponin family
kmeans	4	Red	102	Tgm2	10116.ENSRNOP00000018328	Transglutaminase 2, C polypeptide, isoform CRA_a; Transglutaminase 2
kmeans	4	Red	102	Tln1	10116.ENSRNOP00000022401	RCG55135, isoform CRA_b; Talin 1
kmeans	4	Red	102	Tpm1	10116.ENSRNOP00000024493	Tropomyosin alpha-1 chain; Binds to actin filaments in muscle and non-muscle cells. Plays a central role, in association with the troponin complex, in the calcium dependent regulation of vertebrate striated muscle contraction. Smooth muscle contraction is regulated by interaction with caldesmon. In non-muscle cells is implicated in stabilizing cytoskeleton actin filaments; Belongs to the tropomyosin family
kmeans	4	Red	102	Tpm4	10116.ENSRNOP00000021073	Tropomyosin alpha-4 chain; Binds to actin filaments in muscle and non-muscle cells. Plays a central role, in association with the troponin complex, in the calcium dependent regulation of vertebrate striated muscle contraction (By similarity). Smooth muscle contraction is regulated by interaction with caldesmon (By similarity). In non-muscle cells is implicated in stabilizing cytoskeleton actin filaments (By similarity). Binds calcium (By similarity); Belongs to the tropomyosin family
kmeans	4	Red	102	Trap1	10116.ENSRNOP00000008966	Heat shock protein 75 kDa, mitochondrial; Chaperone that expresses an ATPase activity. Involved in maintaining mitochondrial function and polarization, downstream of PINK1 and mitochondrial complex I. Is a negative regulator of mitochondrial respiration able to modulate the balance between oxidative phosphorylation and aerobic glycolysis. The impact of TRAP1 on mitochondrial respiration is probably mediated by modulation of mitochondrial SRC and inhibition of SDHA
kmeans	4	Red	102	Tubb5	10116.ENSRNOP00000001095	Tubulin beta-5 chain; Tubulin is the major constituent of microtubules. It binds two moles of GTP, one at an exchangeable site on the beta chain and one at a non-exchangeable site on the alpha chain; Belongs to the tubulin family
kmeans	4	Red	102	Vamp7	10116.ENSRNOP00000011065	Vesicle-associated membrane protein 7; Involved in the targeting and/or fusion of transport vesicles to their target membrane during transport of proteins from the early endosome to the lysosome. Required for heterotypic fusion of late endosomes with lysosomes and homotypic lysosomal fusion. Required for calcium regulated lysosomal exocytosis. Involved in the export of chylomicrons from the endoplasmic reticulum to the cis Golgi. Required for exocytosis of mediators during eosinophil and neutrophil degranulation, and target cell killing by natural killer cells. Required for focal exocy [...]
kmeans	4	Red	102	Vcl	10116.ENSRNOP00000015179	Actin filament (F-actin)-binding protein involved in cell- matrix adhesion and cell-cell adhesion. Regulates cell-surface E- cadherin expression and potentiates mechanosensing by the E-cadherin complex. May also play important roles in cell morphology and locomotion
kmeans	4	Red	102	Vps4a	10116.ENSRNOP00000046690	Vacuolar protein sorting-associated protein 4A; Involved in late steps of the endosomal multivesicular bodies (MVB) pathway. Recognizes membrane-associated ESCRT-III assemblies and catalyzes their disassembly, possibly in combination with membrane fission. Redistributes the ESCRT-III components to the cytoplasm for further rounds of MVB sorting. MVBs contain intraluminal vesicles (ILVs) that are generated by invagination and scission from the limiting membrane of the endosome and mostly are delivered to lysosomes enabling degradation of membrane proteins, such as stimulated growth fact [...]
kmeans	4	Red	102	Wdr1	10116.ENSRNOP00000024012	WD repeat-containing protein 1; Induces disassembly of actin filaments in conjunction with ADF/cofilin family proteins. Enhances cofilin-mediated actin severing. Involved in cytokinesis. Involved in chemotactic cell migration by restricting lamellipodial membrane protrusions. Involved in myocardium sarcomere organization. Required for cardiomyocyte growth and maintenance. Involved in megakaryocyte maturation and platelet shedding. Required for the establishment of planar cell polarity (PCP) during follicular epithelium development and for cell shape changes during PCP; the function see [...]
kmeans	4	Red	102	Ywhab	10116.ENSRNOP00000016981	14-3-3 protein beta/alpha; Adapter protein implicated in the regulation of a large spectrum of both general and specialized signaling pathways. Binds to a large number of partners, usually by recognition of a phosphoserine or phosphothreonine motif. Binding generally results in the modulation of the activity of the binding partner. Negative regulator of osteogenesis. Blocks the nuclear translocation of the phosphorylated form (by AKT1) of SRPK2 and antagonizes its stimulatory effect on cyclin D1 expression resulting in blockage of neuronal apoptosis elicited by SRPK2. Negative regulato [...]
kmeans	4	Red	102	Ywhae	10116.ENSRNOP00000007100	14-3-3 protein epsilon; Adapter protein implicated in the regulation of a large spectrum of both general and specialized signaling pathways. Binds to a large number of partners, usually by recognition of a phosphoserine or phosphothreonine motif. Binding generally results in the modulation of the activity of the binding partner. Positively regulates phosphorylated protein HSF1 nuclear export to the cytoplasm
kmeans	4	Red	102	Ywhah	10116.ENSRNOP00000024388	14-3-3 protein eta; Adapter protein implicated in the regulation of a large spectrum of both general and specialized signaling pathways. Binds to a large number of partners, usually by recognition of a phosphoserine or phosphothreonine motif. Binding generally results in the modulation of the activity of the binding partner. Negatively regulates the kinase activity of PDPK1 (By similarity)

kmeans	4	Red	102	Ywhaq	10116.ENSRNOP00000011501	14-3-3 protein theta; Adapter protein implicated in the regulation of a large spectrum of both general and specialized signaling pathways. Binds to a large number of partners, usually by recognition of a phosphoserine or phosphothreonine motif. Binding generally results in the modulation of the activity of the binding partner. Negatively regulates the kinase activity of PDPK1 (By similarity)
kmeans	4	Red	102	Ywhaz	10116.ENSRNOP00000030885	14-3-3 protein zeta/delta; Adapter protein implicated in the regulation of a large spectrum of both general and specialized signaling pathways. Binds to a large number of partners, usually by recognition of a phosphoserine or phosphothreonine motif. Binding generally results in the modulation of the activity of the binding partner
kmeans	4	Red	102	Zadh2	10116.ENSRNOP00000021729	Zinc binding alcohol dehydrogenase, domain containing 2
kmeans	5	Blue	77	Acot9	10116.ENSRNOP00000005033	Similar to acyl-CoA thioesterase, isoform CRA_b; acyl-CoA thioesterase 9
kmeans	5	Blue	77	Actr3	10116.ENSRNOP00000004520	Actin-related protein 3; Functions as ATP-binding component of the Arp2/3 complex which is involved in regulation of actin polymerization and together with an activating nucleation-promoting factor (NPF) mediates the formation of branched actin networks. Seems to contact the pointed end of the daughter actin filament. Plays a role in ciliogenesis (By similarity); Belongs to the actin family. ARP3 subfamily
kmeans	5	Blue	77	Aqp1	10116.ENSRNOP00000015692	Aquaporin-1; Forms a water-specific channel that provides the plasma membranes of red cells and kidney proximal tubules with high permeability to water, thereby permitting water to move in the direction of an osmotic gradient; Belongs to the MIP/aquaporin (TC 1.A.8) family
kmeans	5	Blue	77	Arl6ip5	10116.ENSRNOP00000010185	PRA1 family protein 3; Regulates intracellular concentrations of taurine and glutamate (Ref.5). Negatively modulates SLC1A1/EAAC1 glutamate transport activity by decreasing its affinity for glutamate in a PKC activity-dependent manner. May be involved in membrane traffic
kmeans	5	Blue	77	Asph	10116.ENSRNOP00000048656	Aspartate-beta-hydroxylase
kmeans	5	Blue	77	Bsg	10116.ENSRNOP00000011275	Basigin; Plays an important role in targeting the monocarboxylate transporters SLC16A1, SLC16A3 and SLC16A8 to the plasma membrane. Plays pivotal roles in spermatogenesis, embryo implantation, neural network formation and tumor progression. Stimulates adjacent fibroblasts to produce matrix metalloproteinases (MMPS). Seems to be a receptor for oligomannosidic glycans. In vitro, promotes outgrowth of astrocytic processes
kmeans	5	Blue	77	Capza1	10116.ENSRNOP00000052868	F-actin-capping protein subunit alpha-1; F-actin-capping proteins bind in a Ca(2+)-independent manner to the fast growing ends of actin filaments (barbed end) thereby blocking the exchange of subunits at these ends. Unlike other capping proteins (such as gelsolin and severin), these proteins do not sever actin filaments. May play a role in the formation of epithelial cell junctions
kmeans	5	Blue	77	Capza2	10116.ENSRNOP00000037217	F-actin-capping protein subunit alpha-2; F-actin-capping proteins bind in a Ca(2+)-independent manner to the fast growing ends of actin filaments (barbed end) thereby blocking the exchange of subunits at these ends. Unlike other capping proteins (such as gelsolin and severin), these proteins do not sever actin filaments (By similarity)
kmeans	5	Blue	77	Casq2	10116.ENSRNOP00000021846	Calsequestrin is a high-capacity, moderate affinity, calcium- binding protein and thus acts as an internal calcium store in muscle . Calcium ions are bound by clusters of acidic residues at the protein surface, especially at the interface between subunits. Can bind around 60 Ca(2+) ions. Regulates the release of lumenal Ca(2+) via the calcium release channel RYR2; this plays an important role in triggering muscle contraction. Plays a role in excitation-contraction coupling in the heart and in regulating the rate of heart beats (By similarity)
kmeans	5	Blue	77	Cav3	10116.ENSRNOP00000007601	Caveolin-3; May act as a scaffolding protein within caveolar membranes. Interacts directly with G-protein alpha subunits and can functionally regulate their activity. May also regulate voltage-gated potassium channels. Plays a role in the sarcolemma repair mechanism of both skeletal muscle and cardiomyocytes that permits rapid resealing of membranes disrupted by mechanical stress. Mediates the recruitment of CAVIN2 and CAVIN3 proteins to the caveolae
kmeans	5	Blue	77	Cct2	10116.ENSRNOP00000029234	T-complex protein 1 subunit beta; Molecular chaperone; assists the folding of proteins upon ATP hydrolysis. As part of the BBS/CCT complex may play a role in the assembly of BBSome, a complex involved in ciliogenesis regulating transports vesicles to the cilia. Known to play a role, in vitro, in the folding of actin and tubulin
kmeans	5	Blue	77	Cct3	10116.ENSRNOP00000025824	T-complex protein 1 subunit gamma; Molecular chaperone; assists the folding of proteins upon ATP hydrolysis. As part of the BBS/CCT complex may play a role in the assembly of BBSome, a complex involved in ciliogenesis regulating transports vesicles to the cilia. Known to play a role, in vitro, in the folding of actin and tubulin. Plays a role in the assembly of the von Hippel-Lindau ubiquitination complex (By similarity). Interacts with DNAAF4 (By similarity)
kmeans	5	Blue	77	Cct5	10116.ENSRNOP00000015886	T-complex protein 1 subunit epsilon; Molecular chaperone; assists the folding of proteins upon ATP hydrolysis. As part of the BBS/CCT complex may play a role in the assembly of BBSome, a complex involved in ciliogenesis regulating transports vesicles to the cilia. Known to play a role, in vitro, in the folding of actin and tubulin (By similarity)

kmeans	5	Blue	77	Cct6a	10116.ENSRNOP00000001227	Chaperonin containing Tcp1, subunit 6A (Zeta 1); Molecular chaperone; assists the folding of proteins upon ATP hydrolysis
kmeans	5	Blue	77	Cct7	10116.ENSRNOP00000021030	Chaperonin-containing TCP1 subunit 7; Molecular chaperone; assists the folding of proteins upon ATP hydrolysis
kmeans	5	Blue	77	Cct8	10116.ENSRNOP00000002169	Chaperonin subunit 8 (Theta) (Predicted), isoform CRA_a; Molecular chaperone; assists the folding of proteins upon ATP hydrolysis
kmeans	5	Blue	77	Chchd3	10116.ENSRNOP00000018001	MICOS complex subunit; Component of the MICOS complex, a large protein complex of the mitochondrial inner membrane that plays crucial roles in the maintenance of crista junctions, inner membrane architecture, and formation of contact sites to the outer membrane
kmeans	5	Blue	77	Cltc	10116.ENSRNOP00000005987	Clathrin is the major protein of the polyhedral coat of coated pits and vesicles. Two different adapter protein complexes link the clathrin lattice either to the plasma membrane or to the trans- Golgi network (By similarity). Acts as component of the TACC3/ch- TOG/clathrin complex proposed to contribute to stabilization of kinetochore fibers of the mitotic spindle by acting as inter- microtubule bridge . The TACC3/ch- TOG/clathrin complex is required for the maintenance of kinetochore fiber tension (By similarity). Plays a role in early autophagosome formation (By similarity)
kmeans	5	Blue	77	Des	10116.ENSRNOP00000026860	Muscle-specific type III intermediate filament essential for proper muscular structure and function. Plays a crucial role in maintaining the structure of sarcomeres, inter-connecting the Z-disks and forming the myofibrils, linking them not only to the sarcolemmal cytoskeleton, but also to the nucleus and mitochondria, thus providing strength for the muscle fiber during activity. In adult striated muscle they form a fibrous network connecting myofibrils to each other and to the plasma membrane from the periphery of the Z-line structures. May act as a sarcomeric microtubule-anchoring pro [...]
kmeans	5	Blue	77	Dnaja3	10116.ENSRNOP00000005479	DnaJ (Hsp40) homolog, subfamily A, member 3
kmeans	5	Blue	77	Dnajb11	10116.ENSRNOP00000002462	DnaJ homolog subfamily B member 11; Serves as a co-chaperone for HSPA5. Binds directly to both unfolded proteins that are substrates for ERAD and nascent unfolded peptide chains, but dissociates from the HSPA5-unfolded protein complex before folding is completed. May help recruiting HSPA5 and other chaperones to the substrate. Stimulates HSPA5 ATPase activity (By similarity)
kmeans	5	Blue	77	Dnajb4	10116.ENSRNOP00000017381	DnaJ (Hsp40) homolog, subfamily B, member 4
kmeans	5	Blue	77	Dnajc3	10116.ENSRNOP00000014182	DnaJ homolog subfamily C member 3; Involved in the unfolded protein response (UPR) during ER stress. Co-chaperone of HSPA8/HSC70, it stimulates its ATPase activity. May inhibit both the autophosphorylation of EIF2AK2/PKR and the ability of EIF2AK2 to catalyze phosphorylation of the EIF2A. May inhibit EIF2AK3/PERK activity (By similarity)
kmeans	5	Blue	77	ENSRNOG00000027408	10116.ENSRNOP00000035797	Peptidyl-prolyl cis-trans isomerase D; PPIases accelerate the folding of proteins. It catalyzes the cis-trans isomerization of proline imidic peptide bonds in oligopeptides. Proposed to act as a co-chaperone in HSP90 complexes such as in unligated steroid receptors heterocomplexes. Different co-chaperones seem to compete for association with HSP90 thus establishing distinct HSP90-co-chaperone-receptor complexes with the potential to exert tissue-specific receptor activity control. May have a preference for estrogen receptor complexes and is not found in glucocorticoid receptor complexe [...]
kmeans	5	Blue	77	Eepd1	10116.ENSRNOP00000009342	Endonuclease/exonuclease/phosphatase family domain containing 1
kmeans	5	Blue	77	Ehd1	10116.ENSRNOP00000051714	EH domain-containing protein 1; ATP- and membrane-binding protein that controls membrane reorganization/tubulation upon ATP hydrolysis. In vitro causes vesiculation of endocytic membranes (By similarity). Acts in early endocytic membrane fusion and membrane trafficking of recycling endosomes (By similarity). Recruited to endosomal membranes upon nerve growth factor stimulation, indirectly regulates neurite outgrowth. Plays a role in myoblast fusion (By similarity). Involved in the unidirectional retrograde dendritic transport of endocytosed BACE1 and in efficient sorting of BACE1 to ax [...]
kmeans	5	Blue	77	Fam129a	10116.ENSRNOP00000003320	Protein Niban; Regulates phosphorylation of a number of proteins involved in translation regulation including EIF2A, EIF4EBP1 and RPS6KB1. May be involved in the endoplasmic reticulum stress response (By similarity); Belongs to the Niban family
kmeans	5	Blue	77	Fam210a	10116.ENSRNOP00000022519	Protein FAM210A; Family with sequence similarity 210, member A; Belongs to the FAM210 family

kmeans	5	Blue	77	Fkbp8	10116.ENSRNOP00000027040	Peptidyl-prolyl cis-trans isomerase FKBP8; Constitutively inactive PPiase, which becomes active when bound to calmodulin and calcium. Seems to act as a chaperone for BCL2, targets it to the mitochondria and modulates its phosphorylation state. The BCL2/FKBP8/calmodulin/calcium complex probably interferes with the binding of BCL2 to its targets. The active form of FKBP8 may therefore play a role in the regulation of apoptosis (By similarity)
kmeans	5	Blue	77	Gnai2	10116.ENSRNOP00000022550	Guanine nucleotide-binding protein G(i) subunit alpha-2; Guanine nucleotide-binding proteins (G proteins) are involved as modulators or transducers in various transmembrane signaling systems. The G(i) proteins are involved in hormonal regulation of adenylate cyclase: they inhibit the cyclase in response to beta-adrenergic stimuli. May play a role in cell division
kmeans	5	Blue	77	Gnaq	10116.ENSRNOP00000019174	Guanine nucleotide-binding proteins (G proteins) are involved as modulators or transducers in various transmembrane signaling systems
kmeans	5	Blue	77	Gnas	10116.ENSRNOP00000033065	Neuroendocrine secretory protein 55; GNAS complex locus
kmeans	5	Blue	77	Gnb1	10116.ENSRNOP00000044340	Guanine nucleotide-binding protein G(I)/G(S)/G(T) subunit beta-1; Guanine nucleotide-binding proteins (G proteins) are involved as a modulator or transducer in various transmembrane signaling systems. The beta and gamma chains are required for the GTPase activity, for replacement of GDP by GTP, and for G protein- effector interaction
kmeans	5	Blue	77	Gnb2	10116.ENSRNOP00000001911	Guanine nucleotide-binding protein G(I)/G(S)/G(T) subunit beta-2; Guanine nucleotide-binding proteins (G proteins) are involved as a modulator or transducer in various transmembrane signaling systems. The beta and gamma chains are required for the GTPase activity, for replacement of GDP by GTP, and for G protein- effector interaction
kmeans	5	Blue	77	Gng12	10116.ENSRNOP00000067190	Guanine nucleotide-binding protein subunit gamma; Guanine nucleotide-binding proteins (G proteins) are involved as a modulator or transducer in various transmembrane signaling systems. The beta and gamma chains are required for the GTPase activity, for replacement of GDP by GTP, and for G protein-effector interaction
kmeans	5	Blue	77	Hsp90aa1	10116.ENSRNOP00000009556	Heat shock protein HSP 90-alpha; Molecular chaperone that promotes the maturation, structural maintenance and proper regulation of specific target proteins involved for instance in cell cycle control and signal transduction. Undergoes a functional cycle that is linked to its ATPase activity which is essential for its chaperone activity. This cycle probably induces conformational changes in the client proteins, thereby causing their activation. Interacts dynamically with various co-chaperones that modulate its substrate recognition, ATPase cycle and chaperone function. Engages with a ra [...]
kmeans	5	Blue	77	Hsp90ab1	10116.ENSRNOP00000026920	Heat shock protein HSP 90-beta; Molecular chaperone that promotes the maturation, structural maintenance and proper regulation of specific target proteins involved for instance in cell cycle control and signal transduction. Undergoes a functional cycle that is linked to its ATPase activity. This cycle probably induces conformational changes in the client proteins, thereby causing their activation. Interacts dynamically with various co-chaperones that modulate its substrate recognition, ATPase cycle and chaperone function. Engages with a range of client protein classes via its interacti [...]
kmeans	5	Blue	77	Hsp90b1	10116.ENSRNOP00000034846	Molecular chaperone that functions in the processing and transport of secreted proteins. When associated with CNPY3, required for proper folding of Toll-like receptors. Functions in endoplasmic reticulum associated degradation (ERAD). Has ATPase activity.
kmeans	5	Blue	77	Hspa12b	10116.ENSRNOP00000028856	Heat shock protein 12B
kmeans	5	Blue	77	Hspa5	10116.ENSRNOP00000025064	78 kDa glucose-regulated protein; Plays a role in facilitating the assembly of multimeric protein complexes inside the endoplasmic reticulum. Involved in the correct folding of proteins and degradation of misfolded proteins via its interaction with DNAJC10, probably to facilitate the release of DNAJC10 from its substrate (By similarity); Belongs to the heat shock protein 70 family
kmeans	5	Blue	77	Hspa8	10116.ENSRNOP00000058593	Molecular chaperone implicated in a wide variety of cellular processes, including protection of the proteome from stress, folding and transport of newly synthesized polypeptides, activation of proteolysis of misfolded proteins and the formation and dissociation of protein complexes. Plays a pivotal role in the protein quality control system, ensuring the correct folding of proteins, the re-folding of misfolded proteins and controlling the targeting of proteins for subsequent degradation. This is achieved through cycles of ATP binding, ATP hydrolysis and ADP release, mediated by co-chap [...]
kmeans	5	Blue	77	Hspa9	10116.ENSRNOP00000026696	Chaperone protein which plays an important role in mitochondrial iron-sulfur cluster (ISC) biogenesis. Interacts with and stabilizes ISC cluster assembly proteins FXN, NFU1, NFS1 and ISCU. Regulates erythropoiesis via stabilization of ISC assembly. May play a role in the control of cell proliferation and cellular aging.
kmeans	5	Blue	77	Hspd1	10116.ENSRNOP00000063666	60 kDa heat shock protein, mitochondrial; Chaperonin implicated in mitochondrial protein import and macromolecular assembly. Together with Hsp10, facilitates the correct folding of imported proteins. May also prevent misfolding and promote the refolding and proper assembly of unfolded polypeptides generated under stress conditions in the mitochondrial matrix. The functional units of these chaperonins consist of heptameric rings of the large subunit Hsp60, which function as a back-to-back double ring. In a cyclic reaction, Hsp60 ring complexes bind one unfolded substrate protein per rin [...]

kmeans	5	Blue	77	Immt	10116.ENSRNOP00000051091	MICOS complex subunit Mic60; Component of the MICOS complex, a large protein complex of the mitochondrial inner membrane that plays crucial roles in the maintenance of crista junctions, inner membrane architecture, and formation of contact sites to the outer membrane. Plays an important role in the maintenance of the MICOS complex stability and the mitochondrial cristae morphology
kmeans	5	Blue	77	Jph2	10116.ENSRNOP00000010938	Junctophilin-2; Junctophilins contribute to the formation of junctional membrane complexes (JMCs) which link the plasma membrane with the endoplasmic or sarcoplasmic reticulum in excitable cells. Provides a structural foundation for functional cross-talk between the cell surface and intracellular calcium release channels. JPH2 is necessary for proper intracellular Ca(2+) signaling in cardiac myocytes via its involvement in ryanodine receptor-mediated calcium ion release. Contributes to the construction of skeletal muscle triad junctions (By similarity)
kmeans	5	Blue	77	LOC687295	10116.ENSRNOP00000053926	Similar to translocase of inner mitochondrial membrane 50 homolog
kmeans	5	Blue	77	Lamp2	10116.ENSRNOP00000000177	Lysosome-associated membrane glycoprotein 2; Plays an important role in chaperone-mediated autophagy, a process that mediates lysosomal degradation of proteins in response to various stresses and as part of the normal turnover of proteins with a long biological half-live. Binds target proteins, such as GAPDH, and targets them for lysosomal degradation. Plays a role in lysosomal protein degradation in response to starvation. Required for the fusion of autophagosomes with lysosomes during autophagy. Cells that lack LAMP2 express normal levels of VAMP8, but fail to accumulate STX17 on aut [...]
kmeans	5	Blue	77	Lnpep	10116.ENSRNOP00000017718	Leucyl-cystinyl aminopeptidase; Release of an N-terminal amino acid, cleave before cysteine, leucine as well as other amino acids. Degrades peptide hormones such as oxytocin, vasopressin and angiotensin III, and plays a role in maintaining homeostasis during pregnancy. May be involved in the inactivation of neuronal peptides in the brain. Cleaves Met-enkephalin and dynorphin. Binds angiotensin IV and may be the angiotensin IV receptor in the brain (By similarity)
kmeans	5	Blue	77	Mfn1	10116.ENSRNOP00000060265	Mitochondrial outer membrane GTPase that mediates mitochondrial clustering and fusion . Membrane clustering requires GTPase activity. It may involve a major rearrangement of the coiled coil domains (By similarity). Mitochondria are highly dynamic organelles, and their morphology is determined by the equilibrium between mitochondrial fusion and fission events . Overexpression induces the formation of mitochondrial networks (in vitro) . Has low GTPase activity (By similarity)
kmeans	5	Blue	77	Mtx1	10116.ENSRNOP00000061267	Mtx1 protein; Metaxin 1
kmeans	5	Blue	77	Mtx2	10116.ENSRNOP00000002134	Metaxin 2
kmeans	5	Blue	77	Mul1	10116.ENSRNOP00000021833	Similar to RIKEN cDNA 0610009K11 (Predicted), isoform CRA_b; Mitochondrial E3 ubiquitin protein ligase 1
kmeans	5	Blue	77	Nos3	10116.ENSRNOP00000013058	Produces nitric oxide (NO) which is implicated in vascular smooth muscle relaxation through a cGMP-mediated signal transduction pathway. NO mediates vascular endothelial growth factor (VEGF)-induced angiogenesis in coronary vessels and promotes blood clotting through the activation of platelets
kmeans	5	Blue	77	Opa1	10116.ENSRNOP00000059078	Dynamin-like 120 kDa protein, mitochondrial; Dynamin-related GTPase that is essential for normal mitochondrial morphology by regulating the equilibrium between mitochondrial fusion and mitochondrial fission. Coexpression of isoform 1 with shorter alternative products is required for optimal activity in promoting mitochondrial fusion. Binds lipid membranes enriched in negatively charged phospholipids, such as cardiolipin, and promotes membrane tubulation. The intrinsic GTPase activity is low, and is strongly increased by interaction with lipid membranes (By similarity). Plays a role in [...]
kmeans	5	Blue	77	P4hb	10116.ENSRNOP00000051841	Protein disulfide-isomerase; This multifunctional protein catalyzes the formation, breakage and rearrangement of disulfide bonds. At the cell surface, seems to act as a reductase that cleaves disulfide bonds of proteins attached to the cell. May therefore cause structural modifications of exofacial proteins. Inside the cell, seems to form/rearrange disulfide bonds of nascent proteins. At high concentrations, functions as a chaperone that inhibits aggregation of misfolded proteins. At low concentrations, facilitates aggregation (anti-chaperone activity). May be involved with other chape [...]
kmeans	5	Blue	77	Pdia3	10116.ENSRNOP00000020478	Protein disulfide-isomerase A3; Protein disulfide isomerase family A, member 3
kmeans	5	Blue	77	Pnpla8	10116.ENSRNOP00000049214	Patatin-like phospholipase domain containing 8

kmeans	5	Blue	77	Ppib	10116.ENSRNOP00000022828	Peptidyl-prolyl cis-trans isomerase B; PPIases accelerate the folding of proteins. It catalyzes the cis-trans isomerization of proline imidic peptide bonds in oligopeptides; Belongs to the cyclophilin-type PPIase family. PPIase B subfamily
kmeans	5	Blue	77	Rab14	10116.ENSRNOP00000025649	Regulates, together with its guanine nucleotide exchange factor, DENND6A, the specific endocytic transport of ADAM10, N- cadherin/CDH2 shedding and cell-cell adhesion (By similarity). Involved in membrane trafficking between the Golgi complex and endosomes during early embryonic development. Regulates the Golgi to endosome transport of FGFR-containing vesicles during early development, a key process for developing basement membrane and epiblast and primitive endoderm lineages during early postimplantation development. May act by modulating the kinesin KIF16B-cargo association to endosomes.
kmeans	5	Blue	77	Samm50	10116.ENSRNOP00000016520	Sorting and assembly machinery component 50 homolog; Plays a crucial role in the maintenance of the structure of mitochondrial cristae and the proper assembly of the mitochondrial respiratory chain complexes. Required for the assembly of TOMM40 into the TOM complex; Belongs to the SAM50/omp85 family
kmeans	5	Blue	77	Serpinh1	10116.ENSRNOP00000022983	Binds specifically to collagen. Could be involved as a chaperone in the biosynthetic pathway of collagen
kmeans	5	Blue	77	Slc25a4	10116.ENSRNOP00000014704	Involved in mitochondrial ADP/ATP transport. Catalyzes the exchange of cytoplasmic ADP with mitochondrial ATP across the mitochondrial inner membrane
kmeans	5	Blue	77	Slc25a5	10116.ENSRNOP00000015913	ADP/ATP translocase 2; Catalyzes the exchange of cytoplasmic ADP with mitochondrial ATP across the mitochondrial inner membrane. As part of the mitotic spindle-associated MMXD complex it may play a role in chromosome segregation (By similarity)
kmeans	5	Blue	77	Slmap	10116.ENSRNOP00000060405	Sarcolemma associated protein
kmeans	5	Blue	77	Snx2	10116.ENSRNOP00000051352	Sorting nexin 2
kmeans	5	Blue	77	St13	10116.ENSRNOP00000025925	Hsc70-interacting protein; One HIP oligomer binds the ATPase domains of at least two HSC70 molecules dependent on activation of the HSC70 ATPase by HSP40. Stabilizes the ADP state of HSC70 that has a high affinity for substrate protein. Through its own chaperone activity, it may contribute to the interaction of HSC70 with various target proteins; Belongs to the FAM10 family
kmeans	5	Blue	77	Stip1	10116.ENSRNOP00000028743	Acts as a co-chaperone for HSP90AA1 (By similarity). Mediates the association of the molecular chaperones HSPA8/HSC70 and HSP90
kmeans	5	Blue	77	Tomm22	10116.ENSRNOP00000019323	Mitochondrial import receptor subunit TOM22 homolog; Central receptor component of the translocase of the outer membrane of mitochondria (TOM complex) responsible for the recognition and translocation of cytosolically synthesized mitochondrial preproteins. Together with the peripheral receptor TOM20 functions as the transit peptide receptor and facilitates the movement of preproteins into the translocation pore; Belongs to the Tom22 family
kmeans	5	Blue	77	Tomm40	10116.ENSRNOP00000025281	Channel-forming protein essential for import of protein precursors into mitochondria
kmeans	5	Blue	77	Tomm40l	10116.ENSRNOP00000004641	Mitochondrial import receptor subunit TOM40B; Potential channel-forming protein implicated in import of protein precursors into mitochondria; Belongs to the Tom40 family
kmeans	5	Blue	77	Tomm70a	10116.ENSRNOP00000002238	Mitochondrial import receptor subunit TOM70; Receptor that accelerates the import of all mitochondrial precursor proteins
kmeans	5	Blue	77	Trim72	10116.ENSRNOP00000030976	Tripartite motif-containing protein 72; Muscle-specific protein that plays a central role in cell membrane repair by nucleating the assembly of the repair machinery at injury sites. Specifically binds phosphatidylserine. Acts as a sensor of oxidation: upon membrane damage, entry of extracellular oxidative environment results in disulfide bond formation and homooligomerization at the injury site. This oligomerization acts as a nucleation site for recruitment of TRIM72-containing vesicles to the injury site, leading to membrane patch formation. Probably acts upstream of the Ca(2+)-depend [...]

kmeans	5	Blue	77	Txndc5	10116.ENSRNOP00000018310	Thioredoxin domain containing 5 (endoplasmic reticulum); Belongs to the protein disulfide isomerase family
kmeans	5	Blue	77	Vdac1	10116.ENSRNOP00000008477	Voltage-dependent anion-selective channel protein 1; Forms a channel through the mitochondrial outer membrane and also the plasma membrane. The channel at the outer mitochondrial membrane allows diffusion of small hydrophilic molecules; in the plasma membrane it is involved in cell volume regulation and apoptosis. It adopts an open conformation at low or zero membrane potential and a closed conformation at potentials above 30-40 mV. The open state has a weak anion selectivity whereas the closed state is cation-selective. May participate in the formation of the permeability transition p [...]
kmeans	5	Blue	77	Vdac2	10116.ENSRNOP00000018462	Voltage-dependent anion-selective channel protein 2; Forms a channel through the mitochondrial outer membrane that allows diffusion of small hydrophilic molecules. The channel adopts an open conformation at low or zero membrane potential and a closed conformation at potentials above 30-40 mV. The open state has a weak anion selectivity whereas the closed state is cation- selective (By similarity); Belongs to the eukaryotic mitochondrial porin family
kmeans	5	Blue	77	Vdac3	10116.ENSRNOP00000046203	Voltage-dependent anion-selective channel protein 3; Forms a channel through the mitochondrial outer membrane that allows diffusion of small hydrophilic molecules; Belongs to the eukaryotic mitochondrial porin family
kmeans	5	Blue	77	rCG_22860	10116.ENSRNOP00000038100	Mitochondrial import inner membrane translocase subunit Tim17-B-like; Essential component of the TIM23 complex, a complex that mediates the translocation of transit peptide-containing proteins across the mitochondrial inner membrane
kmeans	6	Lime Green	60	Abcc9	10116.ENSRNOP00000052402	Subunit of ATP-sensitive potassium channels (KATP). Can form cardiac and smooth muscle-type KATP channels with KCNJ11. KCNJ11 forms the channel pore while ABCC9 is required for activation and regulation
kmeans	6	Lime Green	60	Ank1	10116.ENSRNOP00000062056	Ankyrin 1, erythrocytic
kmeans	6	Lime Green	60	Apoa1bp	10116.ENSRNOP00000025986	NAD(P)H-hydrate epimerase; Catalyzes the epimerization of the S- and R-forms of NAD(P)HX, a damaged form of NAD(P)H that is a result of enzymatic or heat-dependent hydration. This is a prerequisite for the S- specific NAD(P)H-hydrate dehydratase to allow the repair of both epimers of NAD(P)HX
kmeans	6	Lime Green	60	Arpc1b	10116.ENSRNOP00000001315	Actin-related protein 2/3 complex subunit 1B; Functions as component of the Arp2/3 complex which is involved in regulation of actin polymerization and together with an activating nucleation-promoting factor (NPF) mediates the formation of branched actin networks
kmeans	6	Lime Green	60	Atp2a2	10116.ENSRNOP00000024347	This magnesium-dependent enzyme catalyzes the hydrolysis of ATP coupled with the translocation of calcium from the cytosol to the sarcoplasmic reticulum lumen. Isoform SERCA2A is involved in the regulation of the contraction/relaxation cycle. Acts as a regulator of TNFSF11-mediated Ca(2+) signaling pathways via its interaction with TMEM64 which is critical for the TNFSF11-induced CREB1 activation and mitochondrial ROS generation necessary for proper osteoclast generation. Association between TMEM64 and SERCA2 in the ER leads to cytosolic Ca (2+) spiking for activation of NFATC1 and pro [...]
kmeans	6	Lime Green	60	Bcs1l	10116.ENSRNOP00000022632	BCS1 homolog, ubiquinol-cytochrome c reductase complex chaperone; BC1 (ubiquinol-cytochrome c reductase) synthesis-like; Belongs to the AAA ATPase family
kmeans	6	Lime Green	60	Canx	10116.ENSRNOP00000040859	Calnexin; Calcium-binding protein that interacts with newly synthesized glycoproteins in the endoplasmic reticulum. It may act in assisting protein assembly and/or in the retention within the ER of unassembled protein subunits. It seems to play a major role in the quality control apparatus of the ER by the retention of incorrectly folded proteins. Associated with partial T-cell antigen receptor complexes that escape the ER of immature thymocytes, it may function as a signaling complex regulating thymocyte maturation. Additionally it may play a role in receptor- mediated endocytosis at [...]
kmeans	6	Lime Green	60	Cd59	10116.ENSRNOP00000060967	CD59 glycoprotein; Potent inhibitor of the complement membrane attack complex (MAC) action. Acts at or after the C5b-8 stage of MAC assembly
kmeans	6	Lime Green	60	Cyb5a	10116.ENSRNOP00000020446	Cytochrome b5; Cytochrome b5 is a membrane bound hemoprotein which function as an electron carrier for several membrane bound oxygenases. It is also involved in several steps of the sterol biosynthesis pathway, particularly in the C-6 double bond introduction during the C-6 desaturation
kmeans	6	Lime Green	60	Cyb5r3	10116.ENSRNOP00000012878	NADH-cytochrome b5 reductase 3; Desaturation and elongation of fatty acids, cholesterol biosynthesis, drug metabolism, and, in erythrocyte, methemoglobin reduction

kmeans	6	Lime Green	60	Dguok	10116.ENSRNOP00000062535	Deoxyguanosine kinase (Predicted), isoform CRA_a; Deoxyguanosine kinase
kmeans	6	Lime Green	60	Eif2s1	10116.ENSRNOP00000013375	Eukaryotic translation initiation factor 2 subunit 1; Functions in the early steps of protein synthesis by forming a ternary complex with GTP and initiator tRNA. This complex binds to a 40S ribosomal subunit, followed by mRNA binding to form a 43S pre-initiation complex. Junction of the 60S ribosomal subunit to form the 80S initiation complex is preceded by hydrolysis of the GTP bound to eIF-2 and release of an eIF-2- GDP binary complex. In order for eIF-2 to recycle and catalyze another round of initiation, the GDP bound to eIF-2 must exchange with GTP by way of a reaction catalyzed b [...]
kmeans	6	Lime Green	60	Epb4.1	10116.ENSRNOP00000013761	Erythrocyte membrane protein band 4.1
kmeans	6	Lime Green	60	Fahd1	10116.ENSRNOP00000019767	Acylpyruvase FAHD1, mitochondrial; Probable mitochondrial acylpyruvase which is able to hydrolyze acetylpyruvate and fumarylpyruvate in vitro. Also has oxaloacetate decarboxylase activity; Belongs to the FAH family
kmeans	6	Lime Green	60	Fitm1	10116.ENSRNOP00000025725	Fat storage-inducing transmembrane protein 1
kmeans	6	Lime Green	60	Flot2	10116.ENSRNOP00000014104	Flotillin-2; May play a role in axon growth and regeneration. May be involved in epidermal cell adhesion and epidermal structure and function; Belongs to the band 7/mec-2 family. Flotillin subfamily
kmeans	6	Lime Green	60	Ganab	10116.ENSRNOP00000036123	Glucosidase, alpha; neutral AB; Belongs to the glycosyl hydrolase 31 family
kmeans	6	Lime Green	60	Gpd1	10116.ENSRNOP00000026200	Glycerol-3-phosphate dehydrogenase 1 (soluble)
kmeans	6	Lime Green	60	Gstz1	10116.ENSRNOP00000065390	Maleylacetoacetate isomerase; Probable bifunctional enzyme showing minimal glutathione-conjugating activity with ethacrynic acid and 7- chloro-4-nitrobenz-2-oxa-1, 3-diazole and maleylacetoacetate isomerase activity. Has also low glutathione peroxidase activity with t-butyl and cumene hydroperoxides (By similarity). Is able to catalyze the glutathione dependent oxygenation of dichloroacetic acid to glyoxylic acid
kmeans	6	Lime Green	60	Hrc	10116.ENSRNOP00000028116	Histidine rich calcium binding protein
kmeans	6	Lime Green	60	LOC679794	10116.ENSRNOP00000014058	Cytochrome c, somatic; Electron carrier protein. The oxidized form of the cytochrome c heme group can accept an electron from the heme group of the cytochrome c1 subunit of cytochrome reductase. Cytochrome c then transfers this electron to the cytochrome oxidase complex, the final protein carrier in the mitochondrial electron-transport chain
kmeans	6	Lime Green	60	Letm1	10116.ENSRNOP00000022540	Mitochondrial proton/calcium exchanger protein; Mitochondrial proton/calcium antiporter that mediates proton-dependent calcium efflux from mitochondrion (By similarity). Crucial for the maintenance of mitochondrial tubular networks and for the assembly of the supercomplexes of the respiratory chain (By similarity). Required for the maintenance of the tubular shape and cristae organization (By similarity). In contrast to SLC8B1/NCLX, does not constitute the major factor for mitochondrial calcium extrusion (By similarity)
kmeans	6	Lime Green	60	Lum	10116.ENSRNOP00000006109	Lumican; Belongs to the small leucine-rich proteoglycan (SLRP) family. SLRP class II subfamily
kmeans	6	Lime Green	60	Mesdc2	10116.ENSRNOP00000052447	LRP chaperone MESD; Chaperone specifically assisting the folding of beta- propeller/EGF modules within the family of low-density lipoprotein receptors (LDLRs). Acts as a modulator of the Wnt pathway through chaperoning the coreceptors of the canonical Wnt pathway, LRP5 and LRP6, to the plasma membrane. Essential for specification of embryonic polarity and mesoderm induction. Plays an essential role in neuromuscular junction (NMJ) formation by promoting cell- surface expression of LRP4. May regulate phagocytosis of apoptotic retinal pigment epithelium (RPE) cells
kmeans	6	Lime Green	60	Mtdh	10116.ENSRNOP00000009989	Protein LYRIC; Downregulates SLC1A2/EAAT2 promoter activity when expressed ectopically. Activates the nuclear factor kappa-B (NF- kappa-B) transcription factor. Promotes anchorage-independent growth of immortalized melanocytes and astrocytes which is a key component in tumor cell expansion. Promotes lung metastasis and also has an effect on bone and brain metastasis, possibly by enhancing the seeding of tumor cells to the target organ endothelium. Induces chemoresistance (By similarity)

kmeans	6	Lime Green	60	Nap1l4	10116.ENSRNOP00000051745	Nucleosome assembly protein 1-like 4; Acts as histone chaperone in nucleosome assembly
kmeans	6	Lime Green	60	Nars2	10116.ENSRNOP00000015784	asparaginyl-tRNA synthetase 2 (mitochondrial)(putative)
kmeans	6	Lime Green	60	Pacsin2	10116.ENSRNOP00000060074	Protein kinase C and casein kinase substrate in neurons 2 protein; Lipid-binding protein that is able to promote the tubulation of the phosphatidic acid-containing membranes it preferentially binds. Plays a role in intracellular vesicle- mediated transport. Involved in the endocytosis of cell-surface receptors like the EGF receptor, contributing to its internalization in the absence of EGF stimulus. May also play a role in the formation of caveolae at the cell membrane. Recruits DNM2 to caveolae, and thereby plays a role in caveola-mediated endocytosis; Belongs to the PACSIN family
kmeans	6	Lime Green	60	Pacsin3	10116.ENSRNOP00000054003	Protein kinase C and casein kinase substrate in neurons 3
kmeans	6	Lime Green	60	Pcyox1	10116.ENSRNOP00000022532	Prenylcysteine oxidase; Involved in the degradation of prenylated proteins. Cleaves the thioether bond of prenyl-L-cysteines, such as farnesylcysteine and geranylgeranylcysteine (By similarity)
kmeans	6	Lime Green	60	Por	10116.ENSRNOP00000001961	NADPH–cytochrome P450 reductase; This enzyme is required for electron transfer from NADP to cytochrome P450 in microsomes. It can also provide electron transfer to heme oxygenase and cytochrome B5; In the N-terminal section; belongs to the flavodoxin family
kmeans	6	Lime Green	60	Prdx2	10116.ENSRNOP00000004799	Peroxiredoxin-2; Thiol-specific peroxidase that catalyzes the reduction of hydrogen peroxide and organic hydroperoxides to water and alcohols, respectively. Plays a role in cell protection against oxidative stress by detoxifying peroxides and as sensor of hydrogen peroxide-mediated signaling events. Might participate in the signaling cascades of growth factors and tumor necrosis factor-alpha by regulating the intracellular concentrations of H(2)O(2); Belongs to the peroxiredoxin family. AhpC/Prx1 subfamily
kmeans	6	Lime Green	60	Prdx3	10116.ENSRNOP00000015186	Thiol-specific peroxidase that catalyzes the reduction of hydrogen peroxide and organic hydroperoxides to water and alcohols, respectively. Plays a role in cell protection against oxidative stress by detoxifying peroxides
kmeans	6	Lime Green	60	Prdx5	10116.ENSRNOP00000028687	Peroxiredoxin-5, mitochondrial; Thiol-specific peroxidase that catalyzes the reduction of hydrogen peroxide and organic hydroperoxides to water and alcohols, respectively. Plays a role in cell protection against oxidative stress by detoxifying peroxides and as sensor of hydrogen peroxide-mediated signaling events
kmeans	6	Lime Green	60	Prkcsh	10116.ENSRNOP00000018009	Protein kinase C substrate 80K-H
kmeans	6	Lime Green	60	Psma5	10116.ENSRNOP00000026928	Component of the 20S core proteasome complex involved in the proteolytic degradation of most intracellular proteins. This complex plays numerous essential roles within the cell by associating with different regulatory particles. Associated with two 19S regulatory particles, forms the 26S proteasome and thus participates in the ATP- dependent degradation of ubiquitinated proteins. The 26S proteasome plays a key role in the maintenance of protein homeostasis by removing misfolded or damaged proteins that could impair cellular functions, and by removing proteins whose functions are no lon [...]
kmeans	6	Lime Green	60	Psmb1	10116.ENSRNOP00000002037	Component of the 20S core proteasome complex involved in the proteolytic degradation of most intracellular proteins. This complex plays numerous essential roles within the cell by associating with different regulatory particles. Associated with two 19S regulatory particles, forms the 26S proteasome and thus participates in the ATP- dependent degradation of ubiquitinated proteins. The 26S proteasome plays a key role in the maintenance of protein homeostasis by removing misfolded or damaged proteins that could impair cellular functions, and by removing proteins whose functions are no lon [...]
kmeans	6	Lime Green	60	Psmc2	10116.ENSRNOP00000016450	Component of the 26S proteasome, a multiprotein complex involved in the ATP-dependent degradation of ubiquitinated proteins. This complex plays a key role in the maintenance of protein homeostasis by removing misfolded or damaged proteins, which could impair cellular functions, and by removing proteins whose functions are no longer required. Therefore, the proteasome participates in numerous cellular processes, including cell cycle progression, apoptosis, or DNA damage repair. PSMC2 belongs to the heterohexameric ring of AAA (ATPases associated with diverse cellular activities) protein [...]
kmeans	6	Lime Green	60	Psmd11	10116.ENSRNOP00000062146	Component of the 26S proteasome, a multiprotein complex involved in the ATP-dependent degradation of ubiquitinated proteins. This complex plays a key role in the maintenance of protein homeostasis by removing misfolded or damaged proteins, which could impair cellular functions, and by removing proteins whose functions are no longer required. Therefore, the proteasome participates in numerous cellular processes, including cell cycle progression, apoptosis, or DNA damage repair. In the complex, PSMD11 is required for proteasome assembly. Plays a key role in increased proteasome activity [...]

kmeans	6	Lime Green	60	Psmd13	10116.ENSRNOP00000019642	26S proteasome non-ATPase regulatory subunit 13; Component of the 26S proteasome, a multiprotein complex involved in the ATP-dependent degradation of ubiquitinated proteins. This complex plays a key role in the maintenance of protein homeostasis by removing misfolded or damaged proteins, which could impair cellular functions, and by removing proteins whose functions are no longer required. Therefore, the proteasome participates in numerous cellular processes, including cell cycle progression, apoptosis, or DNA damage repair
kmeans	6	Lime Green	60	Psmd2	10116.ENSRNOP00000002358	26S proteasome non-ATPase regulatory subunit 2; Component of the 26S proteasome, a multiprotein complex involved in the ATP-dependent degradation of ubiquitinated proteins. This complex plays a key role in the maintenance of protein homeostasis by removing misfolded or damaged proteins, which could impair cellular functions, and by removing proteins whose functions are no longer required. Therefore, the proteasome participates in numerous cellular processes, including cell cycle progression, apoptosis, or DNA damage repair
kmeans	6	Lime Green	60	Psme1	10116.ENSRNOP00000025887	Implicated in immunoproteasome assembly and required for efficient antigen processing. The PA28 activator complex enhances the generation of class I binding peptides by altering the cleavage pattern of the proteasome
kmeans	6	Lime Green	60	Rab10	10116.ENSRNOP00000065234	The small GTPases Rab are key regulators of intracellular membrane trafficking, from the formation of transport vesicles to their fusion with membranes (By similarity). Rabs cycle between an inactive GDP-bound form and an active GTP-bound form that is able to recruit to membranes different set of downstream effectors directly responsible for vesicle formation, movement, tethering and fusion (By similarity). That Rab is mainly involved in the biosynthetic transport of proteins from the Golgi to the plasma membrane (By similarity). Regulates, for instance, SLC2A4/GLUT4 glucose transporte [...]
kmeans	6	Lime Green	60	Rpn1	10116.ENSRNOP00000066002	Subunit of the oligosaccharyl transferase (OST) complex that catalyzes the initial transfer of a defined glycan (Glc(3)Man(9)GlcNAc(2) in eukaryotes) from the lipid carrier dolichol- pyrophosphate to an asparagine residue within an Asn-X-Ser/Thr consensus motif in nascent polypeptide chains, the first step in protein N-glycosylation. N-glycosylation occurs cotranslationally and the complex associates with the Sec61 complex at the channel-forming translocon complex that mediates protein translocation across the endoplasmic reticulum (ER). All subunits are required for a maximal enzyme a [...]
kmeans	6	Lime Green	60	Rpn2	10116.ENSRNOP00000063207	Dolichyl-diphosphooligosaccharide--protein glycosyltransferase subunit 2; Essential subunit of the N-oligosaccharyl transferase (OST) complex which catalyzes the transfer of a high mannose oligosaccharide from a lipid-linked oligosaccharide donor to an asparagine residue within an Asn-X-Ser/Thr consensus motif in nascent polypeptide chains; Belongs to the SWP1 family
kmeans	6	Lime Green	60	Rrbp1	10116.ENSRNOP00000052465	Ribosome binding protein 1
kmeans	6	Lime Green	60	11-Sep	10116.ENSRNOP00000062626	Septin-11; Filament-forming cytoskeletal GTPase. May play a role in cytokinesis (Potential). May play a role in the cytoarchitecture of neurons, including dendritic arborization and dendritic spines, and in GABAergic synaptic connectivity; Belongs to the TRAFAC class TrmE-Era-EngA-EngB-Septin- like GTPase superfamily. Septin GTPase family
kmeans	6	Lime Green	60	2-Sep	10116.ENSRNOP00000024261	Septin-2; Filament-forming cytoskeletal GTPase. Forms a filamentous structure with SEPT12, SEPT6, SEPT2 and probably SEPT4 at the sperm annulus which is required for the structural integrity and motility of the sperm tail during postmeiotic differentiation (By similarity). Required for normal organization of the actin cytoskeleton. Plays a role in the biogenesis of polarized columnar-shaped epithelium by maintaining polyglutamylated microtubules, thus facilitating efficient vesicle transport, and by impeding MAP4 binding to tubulin. Required for the progression through mitosis. Forms a [...]
kmeans	6	Lime Green	60	7-Sep	10116.ENSRNOP00000008839	Filament-forming cytoskeletal GTPase. Required for normal organization of the actin cytoskeleton. Required for normal progress through mitosis. Involved in cytokinesis. Required for normal association of CENPE with the kinetochore. Plays a role in ciliogenesis and collective cell movements. Forms a filamentous structure with SEPTIN12, SEPTIN6, SEPTIN2 and probably SEPTIN4 at the sperm annulus which is required for the structural integrity and motility of the sperm tail during postmeiotic differentiation (By similarity).
kmeans	6	Lime Green	60	Slc25a13	10116.ENSRNOP00000013816	Solute carrier family 25 (aspartate/glutamate carrier), member 13; Belongs to the mitochondrial carrier (TC 2.A.29) family
kmeans	6	Lime Green	60	Snd1	10116.ENSRNOP00000041531	Staphylococcal nuclease domain-containing protein 1; Functions as a bridging factor between STAT6 and the basal transcription factor. Plays a role in PIM1 regulation of MYB activity. Plays a role in cell viability. Functions as a transcriptional coactivator for STAT5. Plays a role in cell viability (By similarity)
kmeans	6	Lime Green	60	Sntb1	10116.ENSRNOP00000006423	Syntrophin, beta 1
kmeans	6	Lime Green	60	Stt3a	10116.ENSRNOP00000040087	Serine/threonine-protein kinase Chk1; STT3A, subunit of the oligosaccharyltransferase complex (catalytic)

kmeans	6	Lime Green	60	Surf1	10116.ENSRNOP00000006855	Surfeit 1; Component of the MITRAC (mitochondrial translation regulation assembly intermediate of cytochrome c oxidase complex) complex, that regulates cytochrome c oxidase assembly
kmeans	6	Lime Green	60	Tmem186	10116.ENSRNOP00000062154	Transmembrane protein 186
kmeans	6	Lime Green	60	Tppp3	10116.ENSRNOP00000022863	Tubulin polymerization-promoting protein family member 3; Binds tubulin and has microtubule bundling activity. May play a role in cell proliferation and mitosis (By similarity)
kmeans	6	Lime Green	60	Tubb4b	10116.ENSRNOP00000013863	Tubulin is the major constituent of microtubules. It binds two moles of GTP, one at an exchangeable site on the beta chain and one at a non-exchangeable site on the alpha chain (By similarity).
kmeans	6	Lime Green	60	Txn2	10116.ENSRNOP00000060694	Thioredoxin, mitochondrial; Important for the control of mitochondrial reactive oxygen species homeostasis, apoptosis regulation and cell viability. Possesses a dithiol-reducing activity
kmeans	6	Lime Green	60	Vcp	10116.ENSRNOP00000040121	Transitional endoplasmic reticulum ATPase; Necessary for the fragmentation of Golgi stacks during mitosis and for their reassembly after mitosis. Involved in the formation of the transitional endoplasmic reticulum (tER). The transfer of membranes from the endoplasmic reticulum to the Golgi apparatus occurs via 50-70 nm transition vesicles which derive from part-rough, part-smooth transitional elements of the endoplasmic reticulum (tER). Vesicle budding from the tER is an ATP-dependent process. The ternary complex containing UFD1, VCP and NPLOC4 binds ubiquitinated proteins and is neces [...]
kmeans	6	Lime Green	60	Vim	10116.ENSRNOP00000024430	Vimentins are class-III intermediate filaments found in various non-epithelial cells, especially mesenchymal cells. Vimentin is attached to the nucleus, endoplasmic reticulum, and mitochondria, either laterally or terminally
