# Supplementary material for: Ultrastructural and proteomic profiling of mitochondria-associated endoplasmic reticulum membranes reveal aging signatures in striated muscle
Source: Cell Death Dis. 2022 Apr 2;13(4):296. doi: 10.1038/s41419-022-04746-4 (PMC8976840; doi:10.1038/s41419-022-04746-4)
Supplement: Supplementary file 11 — Supplementary Table 9. List of oligos used for qPCR assays. [file 41419_2022_4746_MOESM11_ESM.pdf]

Supplementary Table 9. Oligos for qPCR			
**Gene name**	**Protein name**	**Fwd primer sequence**	**Rev primer sequence**
IMMT	MIC60	CATGGACAATTCTGAGATTGC	TTTCTTCGCTCCTTCAACG
CHCHD3	MIC19	AGAGAAAGATCGAATGATACGG	AATTCTGAGCTCCTCTCCT
CHCHD6	MIC25	TTTAAAGTGTCCAGGGCAG	CTCCTGTTGGAACTTCTTGAG
APOO	MIC26	TCACCTTCAGAGTTTATGCAG	AGTAGAGTGAGAGCTCATCG
APOOL	MIC27	AGCCTGGTTATTTACAAAGGG	GATAAATGCCCTTGCACCA
SAMM50	SAMM50	CTCAAAGTCAACCAGGAGC	ATTCAGCTGAAGCTCAAAGTC
MTX1	MTX1	GAGCTATTCTGCTGGTCTG	AAATCTGGTATAGGTCAGCAC
MTX2	MTX2	TCAACAACATGCTGCTGAC	ACCTAGCAAGAGTGATCTCC
DNAJC11	DNAJC11	CTTATGAAGTGCTTAGCGACC	CCCATCCTTCCATCTCCAG
PHB	PHB	CAGAAGCCAATCATCTTTGAC	GACATTCTGCAAGTCTTTGC
PHB2	PHB2	TGGAGAAAGCAAAGCAGGA	CTTCTCCAAGCATCTTAGCAG
HSPA9	GRP75	TTGACATTGATGCCAATGGG	ACTGGATTACAATCTGTTGCTC
VDAC1	VDAC1	TACTAATGTGAATGATGGGACG	GAGATTGACAGCAGTCTCC
VDAC2	VDAC2	CATCAGGTACCAACTGCAC	TGACCTTTGCAGAAATAGAAGC
TOMM20	TOMM20	TTACAACAGACTCTTCCACC	AGCACTGACAATTCTCTGAC
TIMM50	TIMM50	TTACAGACAGATGATCATCGAG	TAAGGTGGCTGGTAGTAGG
OPA1	OPA1	AGGAGAACAAAGAGTTGAGG	TCAGAGATTTCTTGAGCTTCC
FIS1	FIS1	ATGACGACATCCGTAGAGG	TTCATATTCCTTGAGCCGGT
MFN1	MFN1	CAGAGCTAAACAGATACTAGACAC	GAATAAACCCTCTTCTCTGCTG
MFN2	MFN2	CCAAGTTCAGCAAGAATTGTC	TCCTGCTCCAGATTATCCC
