# Supplementary material for: Ultrastructural and proteomic profiling of mitochondria-associated endoplasmic reticulum membranes reveal aging signatures in striated muscle
Source: Cell Death Dis. 2022 Apr 2;13(4):296. doi: 10.1038/s41419-022-04746-4 (PMC8976840; doi:10.1038/s41419-022-04746-4)
Supplement: Supplementary file 12 — Uncropped Western blots [file 41419_2022_4746_MOESM12_ESM.pdf]

Fig. 3C

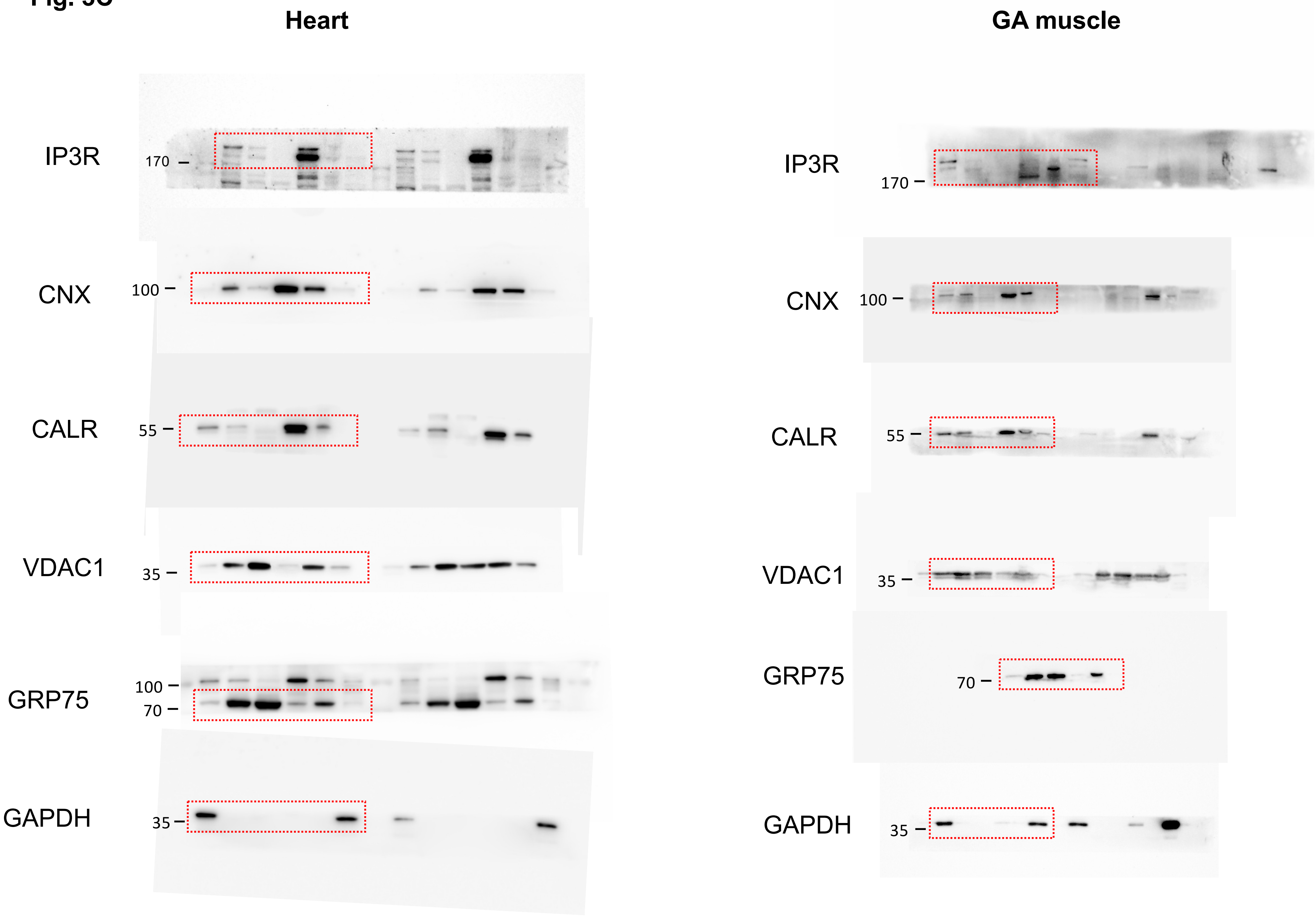

Heart
GA muscle
IP3R
170
CNX
100
CALR
55
VDAC1
35
GRP75
100
70
GAPDH
35

Fig. 6C

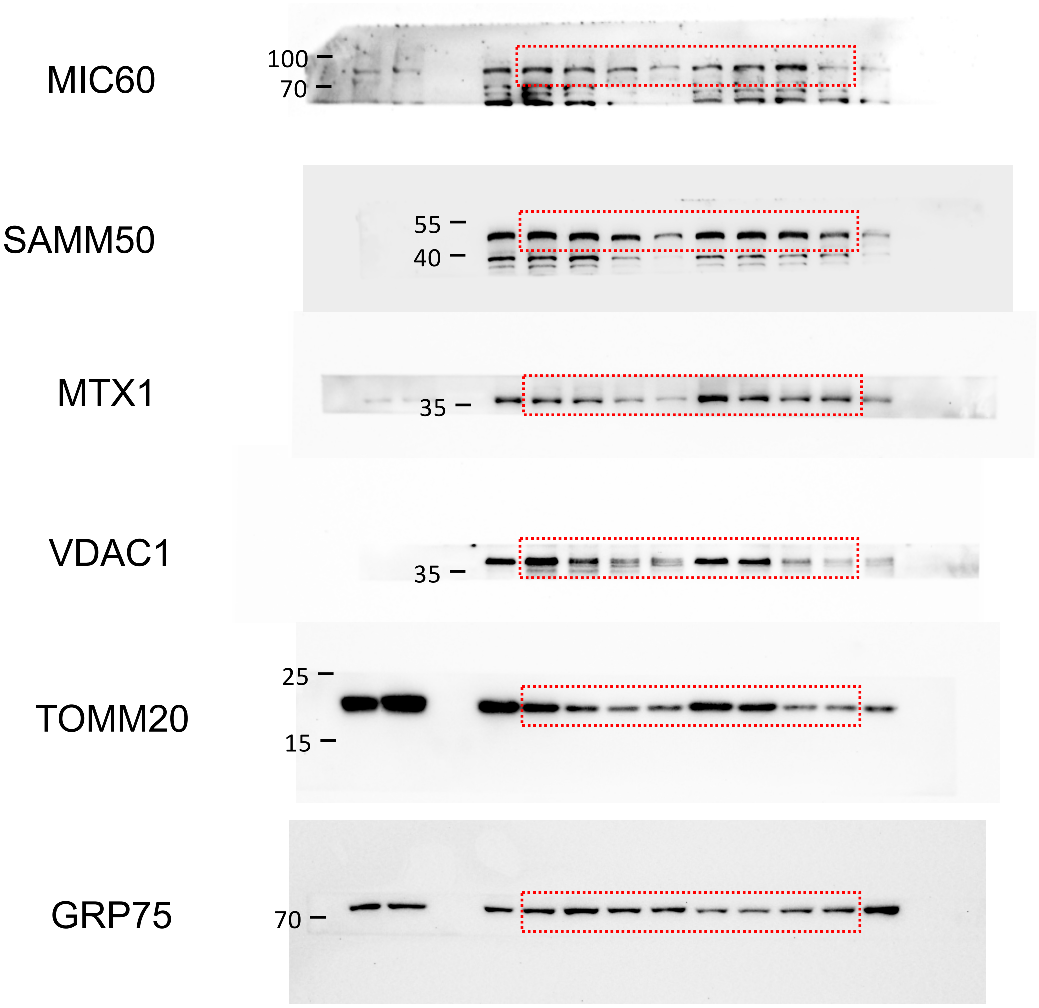

Proteins with close molecular weights were detected as back-to-back replicates.
